# Re-analysis of RNA-seq transcriptome data reveals new aspects of gene activity in Arabidopsis root hairs

**DOI:** 10.3389/fpls.2015.00421

**Published:** 2015-06-08

**Authors:** Wenfeng Li, Ping Lan

**Affiliations:** ^1^Collaborative Innovation Center of Sustainable Forestry in Southern China of Jiangsu Province, College of Biology and the Environment, Nanjing Forestry UniversityNanjing, China; ^2^State Key Laboratory of Soil and Sustainable Agriculture, Institute of Soil Science, Chinese Academy of SciencesNanjing, China

**Keywords:** root hair, novel transcript, RNA-seq, co-expression, Arabidopsis

## Abstract

Root hairs, tubular-shaped outgrowths from root epidermal cells, play important roles in the acquisition of nutrients and water, interaction with microbe, and in plant anchorage. As a specialized cell type, root hairs, especially in Arabidopsis, provide a pragmatic research system for various aspects of studies. Here, we re-analyzed the RNA-seq transcriptome profile of Arabidopsis root hair cells by Tophat software and used Cufflinks program to mine the differentially expressed genes. Results showed that *ERD14, RIN4, AT5G64401* were among the most abundant genes in the root hair cells; while *ATGSTU2*, AT5G54940, AT4G30530 were highly expressed in non-root hair tissues. In total, 5409 genes, with a fold change greater than two-fold (FDR adjusted *P* < 0.05), showed differential expression between root hair cells and non-root hair tissues. Of which, 61 were expressed only in root hair cells. One hundred and thirty-six out of 5409 genes have been reported to be “core” root epidermal genes, which could be grouped into nine clusters according to expression patterns. Gene ontology (GO) analysis of the 5409 genes showed that processes of “response to salt stress,” “ribosome biogenesis,” “protein phosphorylation,” and “response to water deprivation” were enriched. Whereas only process of “intracellular signal transduction” was enriched in the subset of 61 genes expressed only in the root hair cells. One hundred and twenty-one unannotated transcripts were identified and 14 of which were shown to be differentially expressed between root hair cells and non-root hair tissues, with transcripts XLOC_000763, XLOC_031361, and XLOC_005665 being highly expressed in the root hair cells. The comprehensive transcriptomic analysis provides new information on root hair gene activity and sets the stage for follow-up experiments to certify the biological functions of the newly identified genes and novel transcripts in root hair cell morphogenesis.

## Introduction

Root hairs provide a remarkably tractable system for various aspects of studies, such as development, cell biology, and physiology, particularly in *Arabidopsis thaliana* (Dolan et al., 1998; Ryan et al., 2001; Grebe, 2012; Grierson et al., 2014). Over the last 20 years, the mechanisms underlying root hair morphogenesis have been extensively investigated, and “how and where to build a root hair” has been getting more comprehensive (Grebe, 2012; Grierson et al., 2014). The fate of epidermal cells is determined in a position-dependent manner, cells spanning the cleft of two underlying cortical cells, namely “H position,” will form hair cells; while cells presenting over a single cortical cell, called “N position,” will stay as non-hair cells (Grebe, 2012; Grierson et al., 2014). Molecular genetics studies have shown that only 0.0625% (21 out of 33,602 genes) of Arabidopsis genes are involved in the root cell patterning formation (Grierson et al., 2014). Among them, WEREWOLF (WER), MYB23, MYC1, TRANSPARENT TESTA GLABRA(TTG), GLABRA 3 (GL3)/ENHANCER OF GLABRA 3 (EGL3), and GL2 are critical positive regulators for non-hair cell differentiation through the inhibition of *RHD6* expression (Galway et al., 1994; Di Cristina et al., 1996; Masucci and Schiefelbein, 1996; Lee and Schiefelbein, 1999; Bernhardt et al., 2003, 2005; Kang et al., 2009; Bruex et al., 2012; Pesch et al., 2013). *GL2* itself is regulated by the regulatory complex TTG-GL3/EGL3/MYC1-WER/MYB23 (Grebe, 2012; Grierson et al., 2014). Whereas CAPRICE (CPC), TRIPTYCHON (TRY), ENHANCER OF TRY AND CPC1 (ETC1) have been proven to be positive regulators determining the cell fate of root hair (Wada et al., 1997, 2002; Schellmann et al., 2002; Kirik et al., 2004; Tominaga-Wada and Wada, 2014). In addition, some upstream genes, such as *SCRAMBLED* (*SCM*), *HISTONE DEACETYLASE 18* (*HD 18*), and *JACKDAW*(*JKD*) have been identified and well-documented as critical elements in the cell patterning (Kwak et al., 2005, 2014; Xu et al., 2005; Kwak and Schiefelbein, 2007, 2008; Hassan et al., 2010; Liu et al., 2013; Kwak et al., 2014). Although being defined as a root hair, whether a cell could finally become a root hair is relied on many internal and external factors (Grierson et al., 2014). More than 45 genes including *ROOT HAIR DEFECTIVE 6* (*RHD6*), *ROOT HAIR DEFECTIVE 2* (*RHD2*), *EXPANSIN A7* (*EXPA7*), and *EXPANSIN A18* (*EXPA18*) have been proved to be involved in root hair morphogenesis by molecular genetics studies (Grierson et al., 2014). These genes coordinately regulate the processes of Rop-GTPase re-localization and subsequently mediated signaling, vesicle trafficking, cell wall reassembly, establishment of ion gradients, reorganization of cytoskeleton (actin and microtubule), and producing and homoeostasis-maintaining of reactive oxygen species (Ishida et al., 2008; Grierson et al., 2014).

During the past 10 years, with the emergence of microarray technology coupled with advanced computational methods, vast transcriptome analyses at genome-wide level have been performed in Arabidopsis either by comparing transcriptional profiles of root hair-defective mutants compared to those of wild type plants, or by direct exploration of root hair-specific transcriptional profiles from root hair protoplasts based on fluorescence-activated cell sorting (FACS) platform, with hundreds to thousands of genes being identified either involved in root hair morphogenesis or in root hair response to abiotic stresses (Jones et al., 2006; Brady et al., 2007; Dinneny et al., 2008; Gifford et al., 2008; Bruex et al., 2012; Hill et al., 2013; Kwasniewski et al., 2013; Simon et al., 2013; Becker et al., 2014; Niu et al., 2014; Tanaka et al., 2014; Wilson et al., 2015). From these omics datasets, supported by previous molecular genetics studies (Ishida et al., 2008; Grebe, 2012; Ryu et al., 2013; Grierson et al., 2014), a subset of 208 “core” epidermal genes has been identified and a gene regulatory network involved in root epidermis cell differentiation in Arabidopsis has been established (Bruex et al., 2012), which provides an advantage model to study the roles of both single and duplicate genes in a specific gene network (Simon et al., 2013). However, several technical limitations of microarrays, such as limited gene probes present in the chip, narrow dynamic range of gene expression changes, as well as incapability to distinguish homologous genes with high similarity, have failed to show the dynamicity and genome-wide range of transcriptional profiling of root hairs. Fortunately, next-generation sequencing technology has overcome such weaknesses and enabled us to explore whole transcriptomes at single-base resolution in a cost-effective manner. It has also enabled us to accurately quantify gene expression and identify unannotated transcripts and splicing isoforms via advanced computational methods (Trapnell et al., 2012).

In our previous study, the paired-end reads were separately matched to Arabidopsis genome in each biological repeat using BLAT program (Kent, 2002) and the differentially expressed genes were then identified in each replication using custom-made software RACKJ (Lan et al., 2013), which results in 1617 differentially expressed genes between root hairs (herein referred as RH) and non-root hair tissues (all root tissues except root hairs; herein referred as NRH). However, it must be noticed that although BLAT is a very effective tool for doing nucleotide alignments between mRNA and genomic DNA, it was slow and not very accurate for mapping RNA-seq reads to the genome. In addition, BLAT is not designed for the alignment of paired-end reads. RACKJ was initiated for identification of splicing isoforms. It was employed to identify differentially genes in each biological repeat via Z-sore analysis. Therefore, additional information could be revealed by re-analysis of the RNA-seq data using advanced pipeline. Tophat-Cufflinks pipeline are free, open-source software tools for gene discovery and comprehensive expression analysis of RNA-seq data (Trapnell et al., 2012). Tophat was initiated specially for RNA-seq data analysis (Trapnell et al., 2009), which enables both single-end and paired-end reads to align to huge genomes using the ultra-high-throughput short read aligner Bowtie, and then analyzes the mapping results to identify splice junctions between exons (Trapnell et al., 2009, 2012). The Cufflinks pipeline contains four programs which enables us to perform not only accurate quantification of the known gene expression but also identification and quantification of any previously unannotated transcripts with or without biological repeats (Trapnell et al., 2012). In this study, we extended previous study by re-analyzing RNA-seq data using Tophat-Cufflinks pipeline aimed to provide additional information on root hair gene activity. We revealed more than five thousands of genes that were differentially expressed between RH and NRH, with more than 4000 genes only being reported in the present study. Moreover, a subset of 14 previously unannotated transcripts was identified as to be differentially expressed between RH and NRH. The comprehensive transcriptomic analysis expands our knowledge in root hair gene activity and sets the stage for follow-up experiments on the biological functions of the newly identified genes and novel transcripts in root hair morphogenesis.

## Results

### Digital information on gene expression in root hairs and non-root hair tissues at genome-wide level

In previous study, using transgenic plants carrying *Expansin7* (*EXP7*) promoter fused to GFP as materials (Cho and Cosgrove, 2002), coupled with FACS technique, Arabidopsis root hair protoplasts were harvested and the transcriptome profiling has been explored by RNA-seq from two biological repeats. The RNA-seq data was subsequently analyzed using BLAT program (Kent, 2002) and the differentially expressed genes were further mined by custom-made software RACKJ (Lan et al., 2013). In the present study, the RNA-seq data were re-analyzed by aligning the paired-end reads to Arabidopsis Genome released in TAIR10 via Tophat program (Trapnell et al., 2009). The differentially expressed genes between RH and NRH were subsequently identified using Cufflinks pipeline (Trapnell et al., 2010, 2012). Results showed that a total of 19,743 and 19,660 genes were confidently identified (with status “OK”) in RH and NRH, respectively (Table S1 in the Supplementary Material). Of which, an overlap of 19,600 genes were expressed both in RH and NRH. In the RH, *ERD14, RIN4*, and *AT5G64401* were among the most abundant genes, with RPKM (Reads Per Kb per Million reads) value more than 1500 (Table 1). Among the 30 highest abundant genes, those encoding arabinogalactan proteins were the most enriched, and four of which were highly expressed in RH. Genes encoding glutathione S-transferases, dehydrins, thioredoxins, and proline-rich extension-like proteins were among the second enriched group in RH, with at least two members detected from each gene family (Table 1). Among the 30 highest abundant genes in NRH were arabinogalactan protein-encoding genes and genes encoding glutathione S-transferases and dehydrins (Table S2 in the Supplementary Material). A comparison of the 30 highest abundant genes in RH and NRH resulted in an overlap of 14 genes. Of which, genes encoding arabinogalactan proteins, glutathione S-transferases and dehydrins were the most enriched (Table S3 in the Supplementary Material).

**Table 1:** **List of the 30 most highly expressed transcripts in root hairs**.

**AGI**	**Annotation**	**RPKM**
AT1G76180	ERD14, Dehydrin family protein	4562.78
AT5G63270	RPM1-interacting protein 4 (RIN4) family protein	4112.99
AT5G64401	Unknown protein	3479.22
AT3G15450	Aluminum induced protein with YGL and LRDR motifs	3385.68
AT5G65207	Unknown protein	3333.24
AT1G67785	Unknown protein	3258.96
AT2G22470	AGP2, ATAGP2, arabinogalactan protein 2	3220.46
AT5G54940	Translation initiation factor SUI1 family protein	3070.01
AT5G42980	ATH3, ATTRX3, ATTRXH3, TRX3, TRXH3, thioredoxin 3	2714.9
AT1G20440	AtCOR47, COR47, RD17, cold-regulated 47	2699.7
AT2G23120	Late embryogenesis abundant protein, group 6	2371.51
AT1G17190	ATGSTU26, GSTU26, glutathione S-transferase tau 26	2344.36
AT5G40730	AGP24, ATAGP24, arabinogalactan protein 24	2027.53
AT1G20450	ERD10, LTI29, LTI45, Dehydrin family protein	2026.31
AT1G66580	RPL10C, SAG24, senescence associated gene 24	2021.75
AT2G15970	ATCOR413-PM1, cold regulated 413 plasma membrane 1	1992.6
AT5G44020	HAD superfamily, subfamily IIIB acid phosphatase	1990.44
AT3G08580	AAC1, ADP/ATP carrier 1	1968.22
AT5G20230	ATBCB, BCB, BCB, SAG14, blue-copper-binding protein	1947.38
AT5G11740	AGP15, ATAGP15, arabinogalactan protein 15	1943.79
AT1G45145	ATH5, ATTRX5, LIV1, TRX5, thioredoxin H-type 5	1928.02
AT3G54580	Proline-rich extensin-like family protein	1903.3
AT1G80920	J8, Chaperone DnaJ-domain superfamily protein	1780.59
AT2G24850	TAT, TAT3, tyrosine aminotransferase 3	1764.74
AT3G28550	Proline-rich extensin-like family protein	1748.86
AT5G64310	AGP1, ATAGP1, arabinogalactan protein 1	1739.09
AT2G29450	ATGSTU5, GSTU5, glutathione S-transferase tau 5	1729.43
AT2G40000	ATHSPRO2, HSPRO2, ortholog of sugar beet HS1 PRO-1 2	1611.9
AT3G18780	ACT2, DER1, ENL2, LSR2, actin 2	1579.69
AT5G15650	ATRGP2, RGP2, reversibly glycosylated polypeptide 2	1568.93

Gene Ontology (GO) analysis of the top 30 most abundant genes in RH and NRH revealed that stress related processes involved in “cold acclimation,” “response to water deprivation,” “toxin catabolic process,” and “aluminum ion transport” were enriched both in RH and NRH (Table S4 in the Supplementary Material). Other processes of “responsive to oxidative stress,” “response to microbial phytotoxin,” “defense response to fungus,” and “aluminum ion transport” were more enriched in RH than in NRH. In contrast, genes involved in “response to cold” and “serine–isocitratelyase pathway” were more pronounced in NRH than in RH (Table S4 in the Supplementary Material).

### Differentially expressed genes identified between root hairs and non-root hair tissues using Cufflinks pipeline

The differentially expressed genes between RH and NRH were identified using Cuffdiff algorithm in Cufflinks pipeline with following parameters: for a given gene, (1) the FDR (false discovery rate) adjusted *p*-value (that is *q*-value) must be less than 0.05; (2) a fold change between RH and NRH is greater than two-fold; (3) the RPKM value of each gene must be more than one in either of the samples. Subsequently, a total of 5409 genes were identified as differentially expressed genes between RH and NRH (Table S5 in the Supplementary Material). Of which, 2596 genes were significantly greater expressed in NRH than in RH; while abundance of the other 2813 genes was markedly higher in RH than in NRH (Table S6 in the Supplementary Material). Of the 2813 genes, a subset of 61 genes was only detected in RH (Table S7 in the Supplementary Material). Among the rest of 2752 (excluding 61 genes from 2813 genes), the most up-regulated genes were those encoding proline-rich (extensin-like) proteins, extensins, expansins such as EXP7 and EXP18, arabinogalactan proteins, xyloglucan endotransglucosylase/hydrolase, peroxidase superfamily protein, and others. Some other genes including *COBL9, COW1, LRX1, IRE, IRC1*, and others, which were reported to be required for or associated with root hair development and growth, were also found highly induced in RH.

Comparison of the 1617 differentially expressed genes identified in previous study (Lan et al., 2013) to the 5409 genes identified in the present study led to an overlap of 1259 genes (Table S8 in the Supplementary Material). Seventy-seven percent (1259 out of 1617) of the differentially expressed genes identified in previous study have been determined in the present analysis. By contrast, only 23% (1259 out of 5409) of the differentially expressed genes identified in the present study have been found in previous analysis, and 77% (4150 out of 5409) of the additional genes were only discovered in the present study using Tophat-Cufflinks pipeline. Several of these additional genes, such as *COBL9, RHS15*, and *RHS* have been reported to be associated with root hair formation; some of them were among the most up-regulated genes in RH. List of the top 100 most up-regulated genes in the present study can be found in Table 2 for detailed information.

**Table 2:** **List of the 100 most up-regulated genes in root hairs (RH) compared to non-root hair tissues (NRH)**.

**AGI**	**Annotation**	**RH (RPKM)**	**NRH (RPKM)**	**Fold-change (log2)**
AT2G24980	Proline-rich extensin-like family protein	282.55	0.145177	−10.9265
AT5G06630	proline-rich extensin-like family protein	350.094	0.263724	−10.3745
AT1G12560	ATEXP7, expansin A7	390.921	0.365196	−10.064
AT4G40090	AGP3, arabinogalactan protein 3	1002.28	1.07251	−9.86808
AT3G62680	ATPRP3, PRP3, proline-rich protein 3	419.939	0.453138	−9.85601
AT5G04960	Plant invertase/pectin methylesterase inhibitor	179.825	0.201765	−9.7997
AT3G09925	Pollen Ole e 1 allergen and extensin family protein	685.787	0.79408	−9.75426
AT4G25820	ATXTH14	758.459	0.933845	−9.66567
AT5G06640	Proline-rich extensin-like family protein	300.574	0.375472	−9.6448
AT3G54590	ATHRGP1, HRGP1, hydroxyproline-rich glycoprotein	882.994	1.10396	−9.64358
AT5G67400	RHS19, root hair specific 19	456.332	0.570824	−9.64282
AT4G13390	Proline-rich extensin-like family protein	346.903	0.436798	−9.63335
AT4G02270	RHS13, root hair specific 13	828.721	1.12794	−9.52106
AT4G00680	ADF8, actin depolymerizing factor 8	479.401	0.676228	−9.46951
AT1G62980	ATEXP18, expansin A18	208.435	0.345746	−9.23567
AT5G57530	AtXTH12	125.269	0.209592	−9.22322
AT5G35190	Proline-rich extensin-like family protein	467.479	0.808864	−9.17479
AT2G29620	Unknown protein	35.8692	0.067347	−9.05692
AT1G30870	Peroxidase superfamily protein	413.75	0.811178	−8.99453
AT1G12040	LRX1, leucine-rich repeat/extensin 1	184.771	0.367391	−8.97421
AT5G05500	Pollen Ole e 1 allergen and extensin family protein	467.413	0.954079	−8.93637
AT5G11440	CID5, IPD1, CTC-interacting domain 5	141.179	0.300056	−8.87808
AT1G48930	AtGH9C1, GH9C1, glycosyl hydrolase 9C1	212.097	0.450867	−8.87781
AT1G54970	ATPRP1, PRP1, RHS7, proline-rich protein 1	197.916	0.420925	−8.87711
AT2G41970	Protein kinase superfamily protein	232.38	0.551853	−8.71799
AT2G47540	Pollen Ole e 1 allergen and extensin family protein	213.419	0.513428	−8.69931
AT5G22410	RHS18, root hair specific 18	106.322	0.292415	−8.50621
AT2G47360	Unknown protein	62.0735	0.184463	−8.3945
AT2G30670	NAD(P)-binding Rossmann-fold superfamily protein	112.11	0.339629	−8.36674
AT3G10710	RHS12, root hair specific 12	57.5381	0.189001	−8.24998
AT2G45890	ATROPGEF4, RHS11	101.066	0.368687	−8.09869
AT5G49270	COBL9	80.4778	0.313345	−8.00469
AT5G22555	Unknown protein	196.529	0.860094	−7.83603
AT5G40860	Unknown protein	136.684	0.628081	−7.76568
AT3G54580	Proline-rich extensin-like family protein	1903.3	8.85952	−7.74706
AT4G09990	Protein of unknown function (DUF579)	189.187	0.903108	−7.7107
AT1G08090	ATNRT2.1, nitrate transporter 2:1	52.9332	0.266549	−7.63363
AT3G60330	AHA7, HA7, H(+)-ATPase 7	310.855	1.56648	−7.63257
AT2G33460	RIC1, ROP-interactive CRIB motif-containing protein 1	58.361	0.294145	−7.63233
AT4G26010	Peroxidase superfamily protein	461.842	2.38604	−7.59664
AT3G07070	Protein kinase superfamily protein	39.4694	0.207821	−7.56925
AT2G46860	AtPPa3, PPa3, pyrophosphorylase 3	111.429	0.593574	−7.55249
AT3G47040	Glycosyl hydrolase family protein	30.0456	0.168941	−7.47449
AT4G01110	Unknown protein	62.5961	0.387963	−7.33401
AT1G08990	PGSIP5, plant glycogenin-like starch initiation protein 5	62.0058	0.388489	−7.31838
AT5G51270	U-box domain-containing protein kinase family protein	31.5351	0.198535	−7.31142
AT5G58010	LRL3, LJRHL1-like 3	273.512	1.80494	−7.24351
AT3G49960	Peroxidase superfamily protein	159.373	1.06339	−7.22759
AT4G34580	COW1, SRH1	218.104	1.47609	−7.20709
AT3G51350	Eukaryotic aspartyl protease family protein	38.8901	0.274518	−7.14636
AT4G38390	RHS17, root hair specific 17	36.6331	0.260206	−7.13735
AT1G70460	RHS10, root hair specific 10	88.1011	0.641957	−7.10054
AT5G62310	IRE	64.0072	0.516494	−6.95334
AT4G29180	RHS16, root hair specific 16	55.3506	0.448309	−6.94796
AT5G17820	Peroxidase superfamily protein	1305.01	10.998	−6.89067
AT1G27740	RSL4, root hair defective 6-like 4	254.49	2.16234	−6.87888
AT1G09170	P-loop nucleoside triphosphate hydrolases	24.029	0.205787	−6.86748
AT4G30320	CAP superfamily protein	89.776	0.843779	−6.73332
AT5G49870	Mannose-binding lectin superfamily protein	31.8792	0.309214	−6.68786
AT1G51860	Leucine-rich repeat protein kinase family protein	8.1714	0.0812391	−6.65226
AT5G65160	tetratricopeptide repeat (TPR)-containing protein	74.2567	0.744421	−6.64026
AT4G02830	Unknown protein	34.9084	0.352575	−6.6295
AT5G01280	Proline-rich family protein (TAIR:AT3G09000.1)	52.2871	0.534523	−6.61206
AT5G61550	U-box domain-containing protein kinase family protein	35.9846	0.372456	−6.59417
AT4G25220	RHS15, root hair specific 15	24.1657	0.254417	−6.56962
AT2G17890	CPK16, calcium-dependent protein kinase 16	8.41945	0.0946467	−6.47503
AT2G45750	S-adenosyl-L-methionine-dependent methyltransferases	142.026	1.61829	−6.45554
AT4G25090	Riboflavin synthase-like superfamily protein	62.3366	0.714573	−6.44686
AT1G07795	Unknown protein	47.4163	0.554259	−6.41868
AT4G34380	Transducin/WD40 repeat-like superfamily protein	8.24351	0.0984524	−6.38769
AT5G25810	Tny, Integrase-type DNA-binding superfamily protein	21.4022	0.257057	−6.37953
AT2G37820	Cysteine/Histidine-rich C1 domain family protein	17.8879	0.221005	−6.33876
AT1G34760	GF14 OMICRON, GRF11, RHS5	50.7197	0.636707	−6.31577
AT4G25940	ENTH/ANTH/VHS superfamily protein	19.5987	0.246851	−6.31097
AT1G12550	D-isomer specific 2-hydroxyacid dehydrogenase	81.9584	1.03544	−6.30658
AT5G21080	Uncharacterized protein	30.2492	0.410728	−6.20257
AT5G65090	BST1, DER4, MRH3, DNAse I-like superfamily protein	41.273	0.593343	−6.12019
AT4G25110	AtMC2, MC2, metacaspase 2	25.4734	0.366441	−6.11927
AT3G18450	PLAC8 family protein	26.2681	0.378257	−6.1178
AT1G01750	ADF11, actin depolymerizing factor 11	723.49	10.7564	−6.07171
AT3G21340	Leucine-rich repeat protein kinase family protein	83.7404	1.26439	−6.04941
AT2G30660	ATP-dependent caseinolytic (Clp) protease/crotonase	24.6728	0.377473	−6.0304
AT1G10385	Vps51/Vps67 (components of vesicular transport) protein	8.71123	0.133365	−6.02942
AT4G14780	Protein kinase superfamily protein	11.7601	0.181057	−6.02132
AT3G54870	ARK1, CAE1, MRH2	48.081	0.745398	−6.01131
AT3G47050	Glycosyl hydrolase family protein	10.9459	0.183671	−5.89713
AT2G38500	2-Oxoglutarate (2OG) and Fe(II)-dependent oxygenase	91.0314	1.52791	−5.89674
AT3G07900	O-fucosyltransferase family protein	32.1397	0.546227	−5.87871
AT5G15600	SP1L4, SPIRAL1-like4	148.905	2.58613	−5.84745
AT1G53680	ATGSTU28, GSTU28, glutathione S-transferase TAU 28	304.142	5.34615	−5.8301
AT5G42785	Unknown protein	80.0565	1.42305	−5.81396
AT2G20030	RING/U-box superfamily protein	8.41905	0.150287	−5.80787
AT2G03360	Glycosyltransferase family 61 protein	6.24031	0.112152	−5.79809
AT5G12050	Unknown protein	184.136	3.34175	−5.78402
AT4G22217	Arabidopsis defensin-like protein	349.124	6.59131	−5.72703
AT4G21200	ATGA2OX8, GA2OX8, gibberellin 2-oxidase 8	11.7754	0.22266	−5.72479
AT4G08450	Disease resistance protein (TIR-NBS-LRR class) family	7.34767	0.139961	−5.71419
AT1G18420	Aluminum activated malate transporter family protein	55.8597	1.08416	−5.68716
AT1G04280	P-loop containing nucleoside triphosphate hydrolases superfamily protein	192.645	3.74287	−5.68566
AT2G17590	Cysteine/Histidine-rich C1 domain family protein	4.31448	0.0844839	−5.67437

In addition, among the 1617 differentially expressed genes identified in previous study, a subset of 635 genes was shown up-regulated in RH. Among them, 580 genes were found up-regulated in RH in present study, i.e., 91% (580 out of 635) genes up-regulated in previous study were also identified by Tophat-Cufflinks pipeline.

### Differential go analysis of differentially expressed genes

Differential GO analysis of the 5409, 4150, and 1259 genes, which were differentially expressed genes identified in this study, newly identified in this study and identified by both present and previous study, respectively, were performed. Results showed that processes of “response to salt stress,” “ribosome biogenesis,” “protein phosphorylation,” “embryo development ending in seed dormancy,” and “response to water deprivation” were most enriched (*P* ≦ 3.01E-10) in the total 5409 differentially expressed genes (Table S9 in the Supplementary Material). In the 4150 subset, processes of “protein phosphorylation,” “embryo development ending in seed dormancy,” “response to water deprivation,” “response to chitin,” “cytokinesis,” “intracellular signal transduction,” “DNA replication,” “transport,” and “microtubule-based movement” were enriched (*P* ≦ 5.21E-7). Whereas protein synthesis related processes of “ribosome biogenesis” and “translation,” and root hair related processes of root hair cell differentiation and development were underrepresented (*P* ≧ 0.55). By contrast, protein synthesis and root hair related processes as well as processes of “response to salt stress,” “response to cold,” and “response to cadmium ion” were dramatically overrepresented (*P* ≦ 2.97E-11) in the 1259 overlapping genes (Table S9 in the Supplementary Material).

GO analysis of the top 100 most induced genes in RH revealed that processes of “plant-type cell wall organization,” “root hair cell differentiation,” “trichoblast differentiation,” “response to oxidative stress,” “unidimensional cell growth,” “protein phosphorylation,” and “root hair cell tip growth” were enriched; while only the process of “intracellular signal transduction” was shown significantly (*P* < 0.01) in the subset of 61 genes expressed only in RH (Table S10 in the Supplementary Material).

### Co-expression network construction and module identification in RH

**Figure 1:**
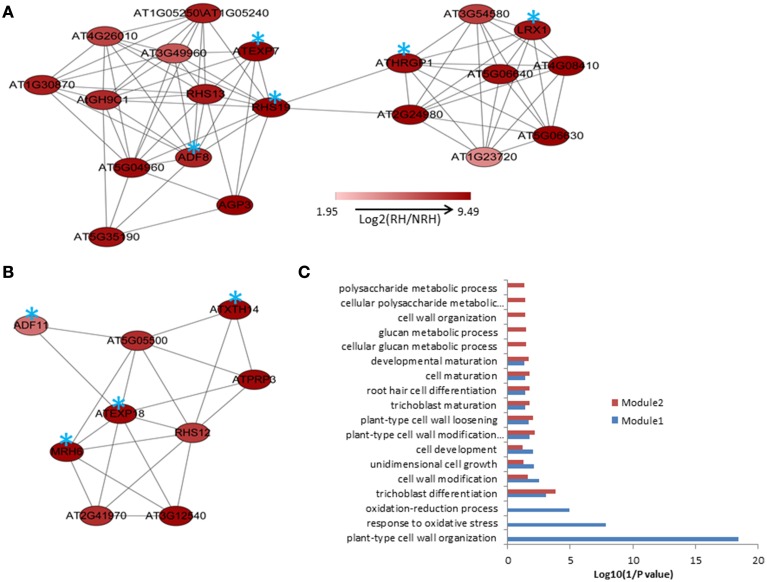
**The modules extracted from the co-expression relationships of the 635 up-regulated genes in root hairs (RH) when compared to non-root hair tissues (NRH), with pearson correlation coefficient cutoff at 0.83(A,B) and Gene Ontology (GO) enrichment analysis of the genes involved in modules (C)**. Blue stars indicate the genes requried for or associated with root hair development and growth.

MACCU program (Lin et al., 2011) was used to calculate the Pearson correlation coefficients of any two genes based on the 300 root-related arrays which were manually identified as previously described, and gene pairs with a threshold value of ≧ 0.83 were selected to build co-expression networks (Lin et al., 2011). The threshold value was selected for individual co-expression network mainly based on the GO enrichment analysis of genes involved in the network (Lin et al., 2011). Briefly, first a series of threshold values from 0.7 to 0.9 were employed to select gene pairs for co-expression networks. Then, we applied a series of GO enrichment analysis of the genes corresponding to individual co-expression network and looked for the threshold with the best enrichments of GO categories (*P* < 1E-03) among the input genes. Cytoscape (http://www.cytoscape.org) program was applied to visualize the co-expression relationships among genes and the tool of NetworkAnalyzer was employed to extract connected components (sub-network). In the present study, we mainly focused on the up-regulated genes in RH and on finding novel modules, when compared to the previous study. To this end, the co-expression analysis of the 635 up-regulated genes previously identified was performed. Results showed that a network comprising of 122 nodes from 124 genes and 367 edges (correlations between genes) was constructed. This network can be divided into one large and 12 small components (sub-networks), with the large one consisting of 93 nodes from 94 genes and 349 edges (Figure S1). Using MCODE program, two modules containing 20 and nine genes were extracted from the large component, respectively (Figures 1A,B). GO analysis showed that processes of “plant-type cell wall organization,” “response to oxidative stress,” “oxidation–reduction process,” and “trichoblast differentiation” were enriched (*P* < 0.001) in module1; while only process of “trichoblast differentiation” was enriched in module 2 (Figure 1C). To know whether this network and modules were presented in the present study, analysis of the 580 up-regulated overlapping genes showed that nearly the same co-expression network was found except that four genes were not included (nodes labeled in blue stars in Figure S1).

**Figure 2:**
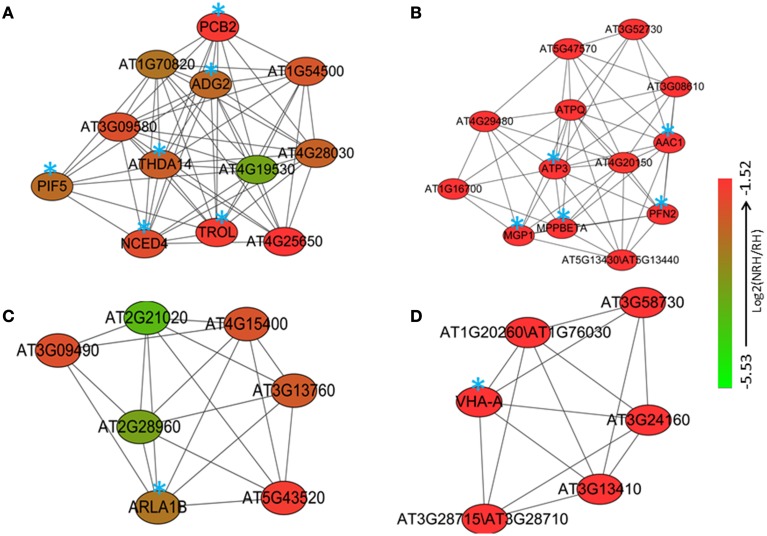
**Four modules (A–D) consisting of various numbers of nodes and edges were extracted from the co-expression network of the 2172 up-regulated genes in root hairs (RH) when compared to non-root hair tissues (NRH), with pearson correlation coefficient cutoff at 0.83**. Blue stars indicate the genes with identified biological functions.

**Figure 3:**
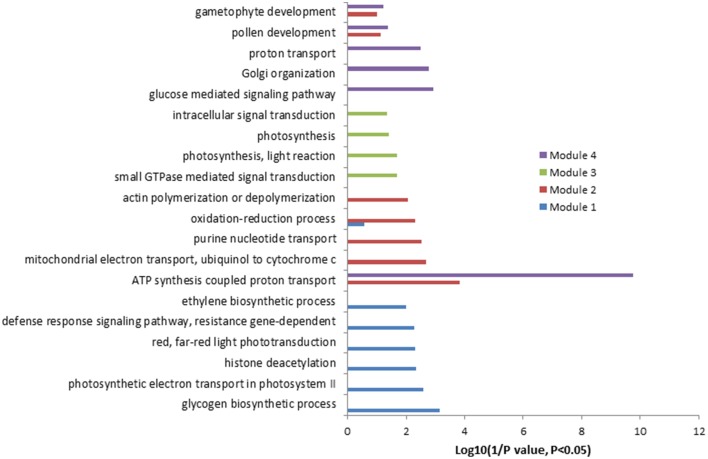
**Gene Ontology (GO) enrichment analysis of the genes involved in different modules mentioned in Figure 2**.

Co-expression analysis was then performed on the 2172 up-regulated genes identified only in the present study. A subset of 264 out of 2172 genes (12%) was co-expressed at the cutoff of 0.83. This network contained 260 nodes from 264 genes and 589 edges, which can be divided into five large (>10 nodes), eight middle (3–10 nodes), and 20 small (2 nodes) sub-networks (Figure S2). The largest sub-network contained 70 nodes from 74 genes, and the second largest sub-network contained 44 genes and the third one contained 29 genes, respectively (Figure S2). The other two large sub-networks contained 20 and 16 genes, respectively (Figure S2). GO analysis showed that processes of energy-related metabolism, such as “ATP synthesis coupled proton transport,” “photorespiration,” “response to salt stress,” “mitochondrial electron transport, ubiquinol to cytochrome c,” “response to cadmium ion,” and “proton transport” were enriched in the largest sub-network (Table S11 in the Supplementary Material). Other enriched processes were mainly related to stress responses such as cellular response to cold, cold acclimation, response to wounding, response to chitin, hyperosmotic salinity response, response to karrikin, and response to UV-B. These enriched processes were mainly distributed in the sub-networks of 3, 4, and 7 (Table S11 in the Supplementary Material). No (*P* = 1) and low (0.01 > *P* > 0.001) enriched processes were found in the sub-networks of 9, and 2, 5, 6, and 8, respectively (Table S11 in the Supplementary Material). Four functional modules were extracted from the network, which contains 12, 13, 7, and 6 nodes and various edges, respectively (Figure 2). GO analysis showed that processes of “glycogen biosynthetic process,” “photosynthetic electron transport in photosystem II,” “histone deacetylation,” “red, far-red light phototransduction,” “defense response signaling pathway, resistance gene-dependent,” and “ethylene biosynthetic process” were enriched in the module 1 (Figure 3), and processes of “ATP synthesis coupled proton transport,” “mitochondrial electron transport, ubiquinol to cytochrome c,” “purine nucleotide transport,” “oxidation–reduction process,” and “actin polymerization or depolymerization” were enriched in the module 2, respectively. In the module 3, besides the process of “ATP synthesis coupled proton transport” which was overrepresented, other processes of “glucose mediated signaling pathway,” “Golgi organization,” and “proton transport” were enriched. While signaling related processes of “small GTPase mediated signal transduction,” “photosynthesis, light reaction,” and “intracellular signal transduction” were enriched in the module 4 (Figure 3).

### Analysis of root hair regulatory element in the differentially expressed genes

Existence of Root Hair Regulatory Element (RHE) cis-element sequence “WHHDTGNNN(N)KCACGWH” (where W = A/T, H = A/T/C, D = G/T/A, K = G/T, and N = A/T/C/G) in the 5409 differentially expressed genes was investigated as previously described (Won et al., 2009). Screening within 3000 bp upstream of the start codon (Hereafter named as −3000 bp) resulted in 201 RHE hits from 194 genes, with few genes carrying two or more RHEs (Table S12 in the Supplementary Material). Among the 201 RHE hits, RHE patterns of “AAAGTGTAGAGCACGAT,” “ATCTTGGCTTTCACGTT,” and “TTCGTGAGTTTCAAATA” were relatively enriched. Subsequently, screening within introns identified 43 genes with one RHE in different intron positions (Table S13 in the Supplementary Material). Eighty nine genes were found to contain one RHE in the CDS (Encoding DNA Sequence) regions, with the sequences of “TCCATGGAAGTCACGAT,” and “TTTATGGCTGGCACGTA” being pronounced among the hits (Table S14 in the Supplementary Material). AT2G31350, encoding glyoxalase 2-5, was shown to contain two RHEs in −3000 bp region and the first intron, respectively (Table 3). Four genes AT1G18460, AT1G18470, AT2G33320, and AT3G45530 were found to harbor one RHE in −3000 bp regions and another one in the CDS regions (Table 3). Another three genes AT3G19050, AT4G03500, and AT5G27680 were shown to carry RHEs in both introns and CDS regions but not in the -3000 bp region (Table 3).

**Table 3:** **Distribution of RHE motif in the differentially expressed genes between root hairs and non-root tissues**.

**AGI**	**Annotation**	**Matching Positions**		**Hit pattern (5′–3′)**	**RH (RPKM)**	**NRH (RPKM)**	**Fold-change (log2)**
		**Start**	**End**				
**RHEs WITHIN 3000 bp UPSTREAM THE START CODON**							
AT1G18460	Alpha/beta-hydrolases superfamily protein	2170	2154	AACGTGAACACCATGGA	142.28	60.44	−1.24
AT1G18470	Transmembrane fragile-X-F-associated protein	831	815	AACGTGAAACACATGTT	104.71	48.45	−1.11
AT2G31350	GLX2-5, glyoxalase 2-5	1950	1966	ACTATGTGGATCACGTT	163.77	15.16	−3.43
AT2G33320	Calcium-dependent lipid-binding (CaLB domain) family protein	2495	2479	AACGTGAAAAACATAGA	9.96	3.39	−1.56
AT3G45530	Cysteine/Histidine-rich C1 domain family protein	344	328	AACGTGAAAACCAAAAA	3.22	0.09	−5.1
**RHEs WITHIN INTRONS**							
AT2G31350.1-1	GLX2-5, glyoxalase 2-5	161	145	TACGTGATGATCATTTT	163.77	15.16	−3.43
AT3G19050.1-19	POK2, phragmoplast orienting kinesin 2	25	41	TTCTTGTGCATCACGTA	0.47	4.6	3.29
AT4G03500.1-1	Ankyrin repeat family protein	856	840	TACGTGCTAAGCAAATT	16.3	2.45	−2.73
AT5G27680.1-8	RECQSIM, RECQ helicase SIM	72	56	TACGTGCTATTCAAATT	0.18	2.15	3.59
**RHEs WITHIN CDS REGIONS**							
AT1G18460.1	Alpha/beta-Hydrolases superfamily protein	1435	1451	AACATGTGTTTCACGTT	142.28	60.44	−1.24
AT1G18470.1	Transmembrane Fragile-X-F-associated protein	466	482	TCCATGGTGTTCACGTT	104.71	48.45	−1.11
AT2G33320.1	Calcium-dependent lipid-binding (CaLB domain) family protein	254	270	TCCGTGATGTTCACGTT	9.96	3.39	−1.56
AT3G19050.1	POK2, phragmoplastorienting kinesin 2	2555	2571	ATTTTGAGCCGCACGAA	0.47	4.6	3.29
AT3G45530.1	Cysteine/Histidine-rich C1 domain family protein	1442	1458	TCCATGGAAGTCACGAT	3.22	0.09	−5.1
AT4G03500.1	Ankyrin repeat family protein	1723	1739	TTTATGGCTGGCACGTA	16.3	2.45	−2.73
AT5G27680.1	RECQSIM, RECQ helicase SIM	1241	1257	ATTTTGGTTCTCACGAT	0.18	2.15	3.59

### Identification of conserved root epidermal genes and associated co-expression network

**Figure 4:**
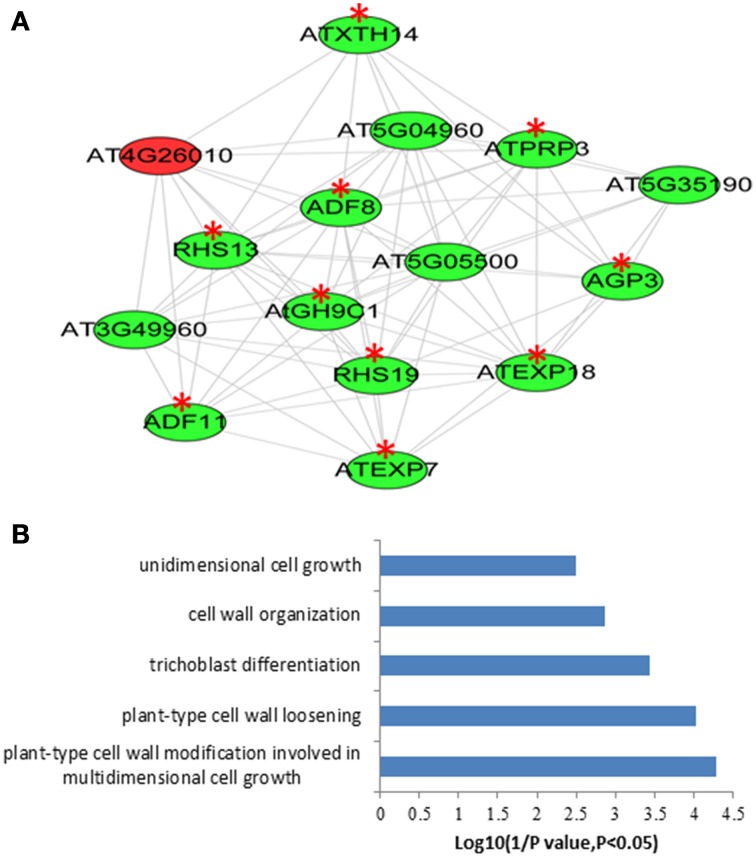
**The module extracted from the “core” root epidermal gene associated co-expression newwork of the differentially expressed genes between root hairs (RH) and non-root hair tissues (NRH), with pearson correlation coefficient cutoff at 0.83 (A) and Gene Ontology (GO) enrichment analysis of the genes involved in modules (B)**. Genes in green color indicate bait genes from “core” root epidermal gene and genes in red color indicate prey genes identified in the present study. Red stars indicate root hair-related genes.

With the attempt to identify conserved root epidermal genes, the set of 208 “core” root epidermal genes was derived from previous report (Bruex et al., 2012), and was compared with the 5409 differentially expressed genes in this study. Comparison resulted in an overlap of 136 genes (Table S15 in the Supplementary Material), which could be grouped into nine clusters according to expression patterns (Figure S3). One hundred and twenty three out of 136 genes were annotated as hair genes, but only 27 of the 123 genes carry RHEs in their promoters (Table S15 in the Supplementary Material). To obtain the conserved root epidermal gene-specific co-expression network, the 136 genes were loaded as baits with the rest of 5409 differentially expressed genes (preys) for subsequent co-expression analysis at correlation coefficient cutoff of 0.83. The final network composing of 122 nodes (genes) and 306 edges was generated after discarding edges only linked to two preys. Of the 122 genes, 50 and 72 were from baits and preys, respectively. This network can be further divided into one large and three small clusters (Figure S4). GO analysis of the bait genes involved in the network showed that processes of “trichoblast differentiation,” “plant-type cell wall organization,” and “root hair elongation” were most enriched (Figure S4), while processes of “plant-type cell wall organization” and “oxidation–reduction process” were overrepresented in the prey genes (Figure S5). One module was extracted from the network, which contains 15 nodes and 80 edges (Figure 4A). GO analysis showed that processes of “plant-type cell wall modification involved in multidimensional cell growth,” “plant-type cell wall loosening,” and “trichoblast differentiation” were most enriched in this module (Figure 4B).

### Identification of unannotated transcripts

**Figure 5:**
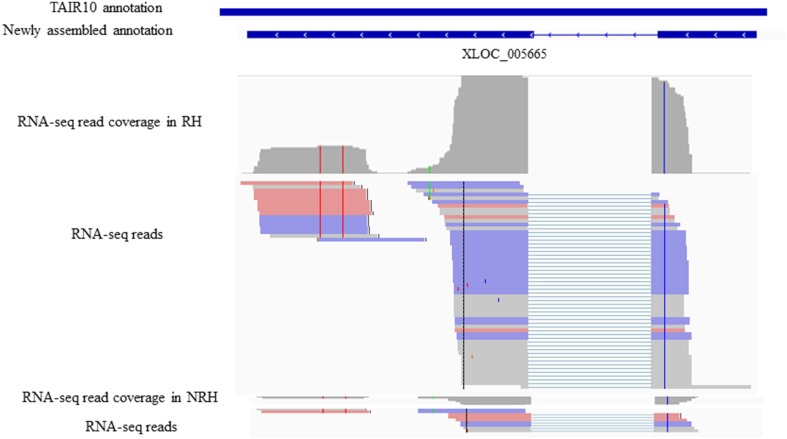
**Identifiaction of the previously unannotated transcrit XLOC_005665**. All panels are screen captures from the The Integrative Genomics Viewer (IGV) browser (http://www.broadinstitute.org/software/igv/home). Top panelsindicates gene annotations from TAIR10 and newly assemly and Bottom panels are screen captures from IGV representing individual RNA-seq read coverages and reads,where thick lines reprsents reads and thin lines represent gapped reads.

To identify previously unannotated transcripts which are differentially expressed between RH and NRH, we first assembled a new transcript on the basis of annotated transcript reference (TAIR10_GFF3_genes_gff) using Cuffmerge algorithm in the Cufflinks pipeline (Trapnell et al., 2012). Subsequently, the differentially expressed previously unannotated transcripts were analyzed using Cuffdiff program (Trapnell et al., 2012). Results showed that a total of 121 novel transcripts were identified, and 14 out of 121 unannotated transcripts were differentially expressed between RH and NRH, with transcripts XLOC_000763, XLOC_031361, and XLOC_005665 being the most expressed genes in RH (Table 4). XLOC_005665 is of particular interest, which was highly expressed in RH (Figure 5) and deduced a small peptide with 59 amino acids.

**Table 4:** **List of the 14 differentially expressed unannotated transcripts between root hairs (RH) and non-root hair tissues (NRH)**.

**Gene_id**	**Locus**	**RH (RPKM)**	**NRH (RPKM)**	**Fold-change (log2)**	***q*-values**
XLOC_000763	Chr1:5374818–5375862	23.2677	1.33563	−4.12273	0.00083917
XLOC_007957	Chr2:565024–566158	0	1.08437	inf	0.00083917
XLOC_013179	Chr3:872193–873509	0	2.1933	inf	0.00083917
XLOC_031350	Chrchloroplast:72259–88953	0.46923	5.2598	3.48665	0.00083917
XLOC_031361	Chrmitochondria:286411–363534	386.671	71.7074	−2.43091	0.00201438
XLOC_007668	Chr1:29208757–29210196	0	1.59459	inf	0.00346354
XLOC_007865	Chr1:17233557–17234225	0	0.583177	inf	0.00427705
XLOC_017836	Chr3:12558721–12560070	0	3.49622	inf	0.00427705
XLOC_008519	Chr2:4871295–4871915	0	1.02029	inf	0.0065152
XLOC_012898	Chr2:18797715–18799154	0	0.445751	inf	0.00997437
XLOC_026701	Chr5:20540459–20541185	1.4363	7.97303	2.47277	0.0321607
XLOC_005665	Chr1:12271087–12271679	13.8534	0.950421	−3.86553	0.033759
XLOC_007866	Chr1:17292409–17293002	48.231	16.1677	−1.57685	0.0422255
XLOC_026377	Chr5:17958314–17959838	5.63137	17.0462	1.59789	0.0482593

*“inf” indicates no ratio*.

## Discussion

Root hairs in Arabidopsis have been intensively studied in various respects and close to 100 genes involved in the cell fate determination and root hair formation have been identified, which provides numerous advantages for basic studies of development, cell biology, and physiology (Grierson et al., 2014).

In the last decade, high-throughput transcriptome analysis, used as alternate approaches differing from traditional molecular genetic analysis, have been adopted extensively to explore genes potentially involved in root hair morphogenesis at genome-wide in Arabidopsis (Birnbaum et al., 2003; Jones et al., 2006; Brady et al., 2007; Dinneny et al., 2008; Gifford et al., 2008; Bruex et al., 2012; Lan et al., 2013). In the current study, RNA-seq data sets were re-analyzed by Tophat-Cufflinks pipeline, and several new aspects of root hair gene expression were presented. First, RNA-seq technique facilitated obtaining the global “digital” transcriptional information on root hair genes (Table S1 in the Supplementary Material). Of the 19,743 genes detected in RH, *ERD14, RIN4, AT5G64401*, and others were among the most abundant transcripts (Table 1). *ERD14* and its homologous *ERD10* were previously isolated from a cDNA library of Arabidopsis plants hydrated for 1 h and induced by ABA treatment and dehydration (Kiyosue et al., 1994). In this study, both *ERD14* and *ERD10* were shown highly expressed in RH, and were up-regulated in RH compared to NRH (Table 1 and Table S2 in the Supplementary Material). This suggests that *ERD14* and *ERD10* might be important in root hair morphogenesis or in response to abiotic stresses. RIN4 (RPM1-interacting protein 4) is first reported to interact with *Pseudomonas syringae* type III effector or molecules, and is required for RPM1-mediated resistance in Arabidopsis (Mackey et al., 2002). Further study showed that RIN4 can interact with AHA1 and AHA2 both *in vitro* and *in vivo*, thus regulating plasma membrane (PM) H(+)-ATPases activity. PM H(+)-ATPase activation/ inactivation can regulate the opening or closure of stomata, thereby controls bacterial entry into the leaf (Liu et al., 2009). AHA2 has been reported to be a major regulator controlling the rhizosphere acidification in response to Fe deficiency (Santi and Schmidt, 2009). Taken together, it is possible that RIN4 also plays important roles in root hair morphogenesis and response to Fe deficiency by regulating (PM) H(+)-ATPases activity mediated by AHA2. The third highest expressed gene in root hairs was AT5G64401 which encodes a small peptide with unknown function (Table 1).

In previous study, a subset of 1617 genes showed differential expression between RH and NRH (Lan et al., 2013). In this study, the abundance of the 5409 genes was revealed to be changed significantly (Table S5) by Tophat-Cufflinks pipeline. Comparison of these two sets (5409 vs. 1617) resulted in an overlapped 1259 genes. We showed that additional 4150 genes were differentially expressed between RH and NRH. Genes like *COBL9* (Jones et al., 2006) and *RHS15* (Won et al., 2009; Bruex et al., 2012), which were reported to be required for or associated with root hair development and growth, were only determined in this study (Table 2). Moreover, 1/3 of cell-type patterning genes, such as *ECTOPIC ROOT HAIR2* (*ERH2/POM1*), *ECTOPIC ROOT HAIR3* (*ERH3*), *GLABRA3* (*GL3*), *ROOTHAIRLESS2* (*RHL2*), *SCRAMBLED* (*SCM/SUB*), *TRANSPARENT TESTA GLABRA2* (*TTG2*), and *WEREWOLF* (*WER*), 63% (31 out of 49) of root hair morphogenesis-related genes (Grierson et al., 2014), and 45% (five out of 11) of genes related to hormone action affecting root hair development (Grierson et al., 2014) have been identified as differentially expressed genes between RH and NRH (Table S5). This study well-complements and extends the previous study by adding new information on root hair genes' numbers and activity. Several highly up-regulated genes in RH, which were not reported previously, deserve further investigation.

Co-expression analysis, which is based on the concept that genes with coordinated expression pattern under diverse conditions are often functionally related (Eisen et al., 1998). This concept allows us to filter and select genes of unknown functions for experimental validation and functional predictions as their co-expression is related to genes of known functions (Aoki et al., 2007; Usadel et al., 2009). Not only did we identified modules from previous study (Figure 1 and Figure S1), but also revealed some new modules by the co-expression analysis of the subset of 2172 up-regulated genes in RH (Figure 2 and Figure S2). Results showed that only 12% (264 out of 2172) of the differentially expressed genes are involved in the network, and 589 relationships between genes were formed, suggesting that most of these genes are involved in diverse processes. GO analysis showed that genes associated with energy and stress related processes are enriched in the network (Table S11). This further indicates that root hair development and growth are sensitive to environmental stimuli and are energy-dependent. The conserved root epidermal genes, associated the co-expression analysis of 5409 genes, led to a network composed of 122 nodes (genes) and 306 edges (Figure S3). Unexpectedly, in the module, only one gene was from preys (Figure 4A in red color) and another 14 genes, including EXP7, EXP18, RHS12, RHS13, and RHS19, were from core root epidermal genes. (Figure 4A). Since these core genes were verified to be required for root hair development and growth, therefore it can be suggested that this prey gene plays important roles in root morphogenesis (Figure 4B). These results strongly encourage worth further investigation for those genes with unknown functions associated with the above mentioned networks.

The analysis of RHEs in the differentially expressed genes (5409) resulted in only 194 genes which carry one or two RHEs within the 3000 bp upstream of the start codon (Table S12). In an attempt to find whether such RHE localizes in other positions, we screened RHE in both introns and CDS regions. Subsets of 43 and 89 genes harboring one RHE have been hit, respectively (Tables S13, S14). Further analysis showed that only few genes carry RHE in introns and CDS, but none of them carry RHE within the three different types of positions (Table 3). Similarly, the previous study identified 154 out of 208 “core” epidermal genes in “H” position, namely root hair genes, but only 33 of them carry RHE (Bruex et al., 2012). These results suggest that regulatory elements, other than RHE, are probably involved in the transcriptional regulation of root hair gene expression.

## Conclusions

In summary, using the currently popular RNA-seq analysis programs, we here provided genome-wide “digital” information on transcriptional expression of root hair genes. We detected additional 4150 genes that are differentially expressed between RH and NRH. We also identified 14 previously unannotated transcripts, which are also differentially expressed between RH and NRH. The findings in this study well-complement and extend the previous one. Some of the highly up-regulated genes in root hairs, which were not reported in the previous study, such as *RIN4* (of known function) or *AT5G64401* (of unknown function) are worth further study. Gene clustering and the root epidermal-specific co-expression analysis revealed some potentially important genes, such as *AT5G04960, AT4G26010*, and *AT5G05500* probably function as putative novel players in root hair morphogenesis.

## Materials and methods

### Data collection and processing

Transcriptomic data sets were downloaded from a public database (NCBI: SRA045009.1) and analyzed as previously described (Trapnell et al., 2009, 2010). Microarray data of 2671 ATH1 arrays from the NASCarray database (http://affymetrix.arabidopsis.info/) were downloaded and normalized using the RMA function of the Affy package of the Bioconductor software. Three hundred root-related arrays were manually identified as previously described (Lin et al., 2011), and were used as a database for co-expression analysis.

### Mapping of RNA-seq reads and identification of differentially expressed genes

All analyses were carried out using the Tophat-Cufflinks pipeline (Trapnell et al., 2009, 2010), with the following versions: Tophat v2.0.11, Bowtie2 v2.2.2.0, and Cufflinks v2.2.1. The Arabidopsis TAIR10 genome and gene model annotation file (GFF, TAIR10_GFF3_genes_gff) downloaded from TAIR (www.arabidopsis.org) were used as reference.

To align the RNA-seq reads to the genome, we first generated a Bowtie2 index using TAIR10 genome and then run Tophat with the following options: -N 2 –read-gap-length 3 –read-edit-dist 3 –read-realign-edit-dist 0 –report-secondary-alignments –coverage-search –microexon-search –library-type fr-unstranded –b2-sensitive. The resulting aligned reads were then used to create a **RABT** (Reference Annotation Based Transcript) assembly using Cufflinks. First, Cufflinks was run in the discovery mode aimed to identify previously unannotated transcripts. Assemblies both from RH and NRH were then merged into one file using Cuffmerge, using TAIR10_GFF3_genes_gff file as the reference annotation, resulting in a **RABT** assembly, used to quantify transcript abundance. Finally, transcript abundance (RPKM) and identification of differentially expressed genes was performed using Cuffdiff with default parameters (*P* < 0.05 and FDR cutoff of 0.05%) with the options: -N –u, corresponding to upper quartile normalization and multi-read-correct. Differential transcript abundance at all genes was calculated as the logarithm base-2 of the expression ratio (RPKM_NRH_/RPKM_RH_).

### Gene ontology analysis

GO enrichment analysis using the TopGo “elim” method (Alexa et al., 2006) was based on The Gene Ontology Browsing Utility (GOBU) as previously described (Lin et al., 2006). The elim algorithm iteratively removes the genes mapped to significant terms from higher level GO terms, and thus avoids the increase of unimportant functional categories.

### Generation of co-expression networks using the MACCU toolbox

Gene co-expression networks were constructed on the basis of 300 publicly available root-related microarrays using the MACCU toolbox as previous report (Lin et al., 2011), with a Pearson correlation threshold of equal to or greater than 0.83 based on the GO enrichment analysis. The generated co-expression networks were visualized by Cytoscape (http://www.cytoscape.org), and the Cytoscape tool of NetworkAnalyzer was employed to extract connected components (sub-network).

### Module identification of co-expression networks

MCODE plugin in Cytoscape software was employed to extract functional modules as previous report (Rivera et al., 2010). First, a vertex-weighting value was calculated based on the clustering coefficient, Ci [Ci = 2 ^*^ *n*/Ki ^*^ (Ki-1)], where Ki represents the node count of the neighborhood of node i; and *n* indicates the number of edges among the Ki nodes in the neighborhood. Next, the highest weighted vertex is set as a center point, seed of the region and search node j whose weight ratio (Wj/Wseed) was >0.1. Then, it filters the predicted complexes if the minimum degree of the graph is less than the given threshold and then constructs a module by deleting the searched node from the network. The top modules with a node count >5 were selected in the co-expression networks for GO enrichment analysis.

### Conflict of interest statement

The authors declare that the research was conducted in the absence of any commercial or financial relationships that could be construed as a potential conflict of interest.

## Supplementary material

Figure S1 **Co-expression relationships of the 635 up-regulated genes in root hairs (RH) when compared to non-root hair tissues (NRH),with pearson correlation coefficient cutoff at 0.83**. Bule stars indicate the genes requried for or associated with root hair development and growth.

Figure S2 **Co-expression relationships of the 2172 up-regulated genes in root hairs (RH) when compared to non-root hair tissues (NRH),with pearson correlation coefficient cutoff at 0.83**.

Figure S3 **Hierarchical clustering analysis of changes in transcript abundance of 136 overlapping genes (Table S13 in the Supplementary Material) between 208 “core” root epidermal genes (Bruex et al., 2012) and 5409 differentially expressed genes in this study**. Transcript abundance was defined as RPKM (Reads Per Kilobase per Millionmapped reads) in the root hairs (RH) and non-root hair tissues (NRH) with two biological repeats. Color key indicates the log2 transformed intensity, gray color which not in the color key indicates that the number is missing.

Figure S4 **The “core” root epidermal gene associated co-expression newwork of the differentially expressed genes between root hairs (RH) and non-root hair tissues (NRH),with pearson correlation coefficient cutoff at 0.83**. Genes in green color indicate bait genes from “core” root epidermal gene and genes in red color indicate prey genes identified in the present study.

Figure S5 **Gene Ontology (GO) enrichment analysis of the bait and prey genes involved in the the “core” root epidermal gene associated co-expression newwork**.
