# Supplementary figures and images for: Re-analysis of RNA-seq transcriptome data reveals new aspects of gene activity in Arabidopsis root hairs
Source: Front Plant Sci. 2015 Jun 8;6:421. doi: 10.3389/fpls.2015.00421 (PMC4458573; doi:10.3389/fpls.2015.00421)

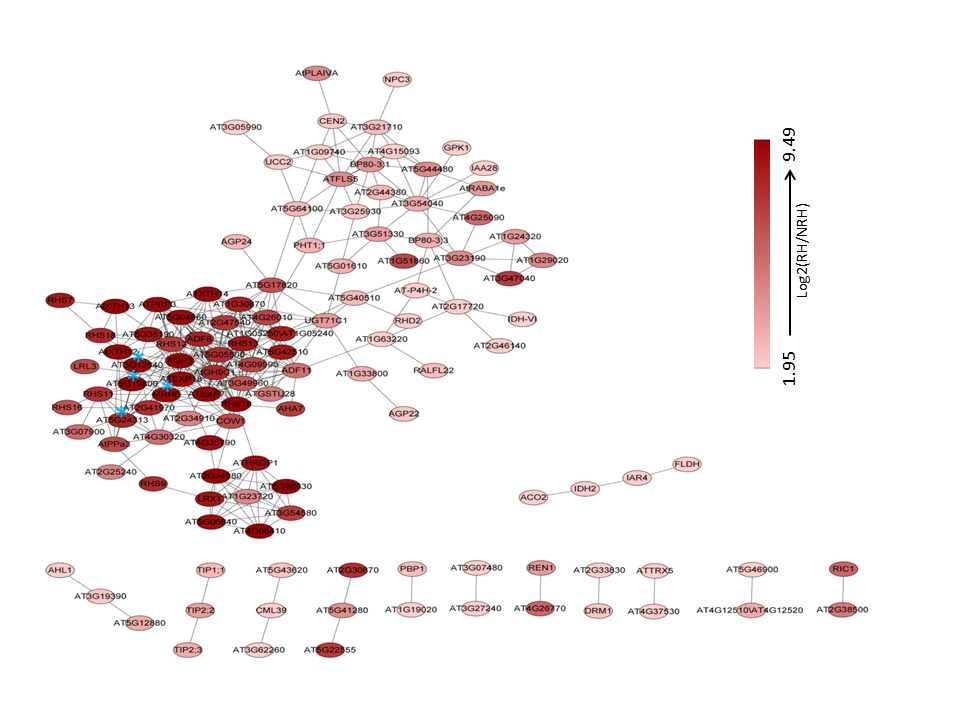

Supplement: Figure S1 — Co-expression relationships of the 635 up-regulated genes in root hairs (RH) when compared to non-root hair tissues (NRH),with pearson correlation coefficient cutoff at 0.83. Bule stars indicate the genes requried for or associated with root hair development and growth. [file Image1.TIF]

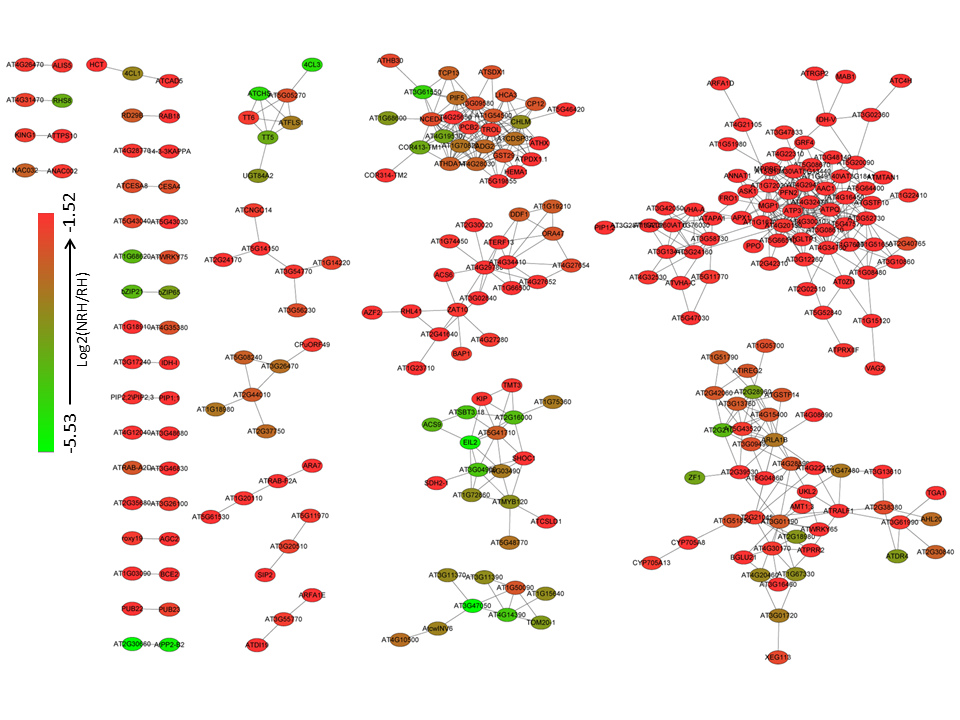

Supplement: Figure S2 — Co-expression relationships of the 2172 up-regulated genes in root hairs (RH) when compared to non-root hair tissues (NRH),with pearson correlation coefficient cutoff at 0.83. [file Image2.TIF]

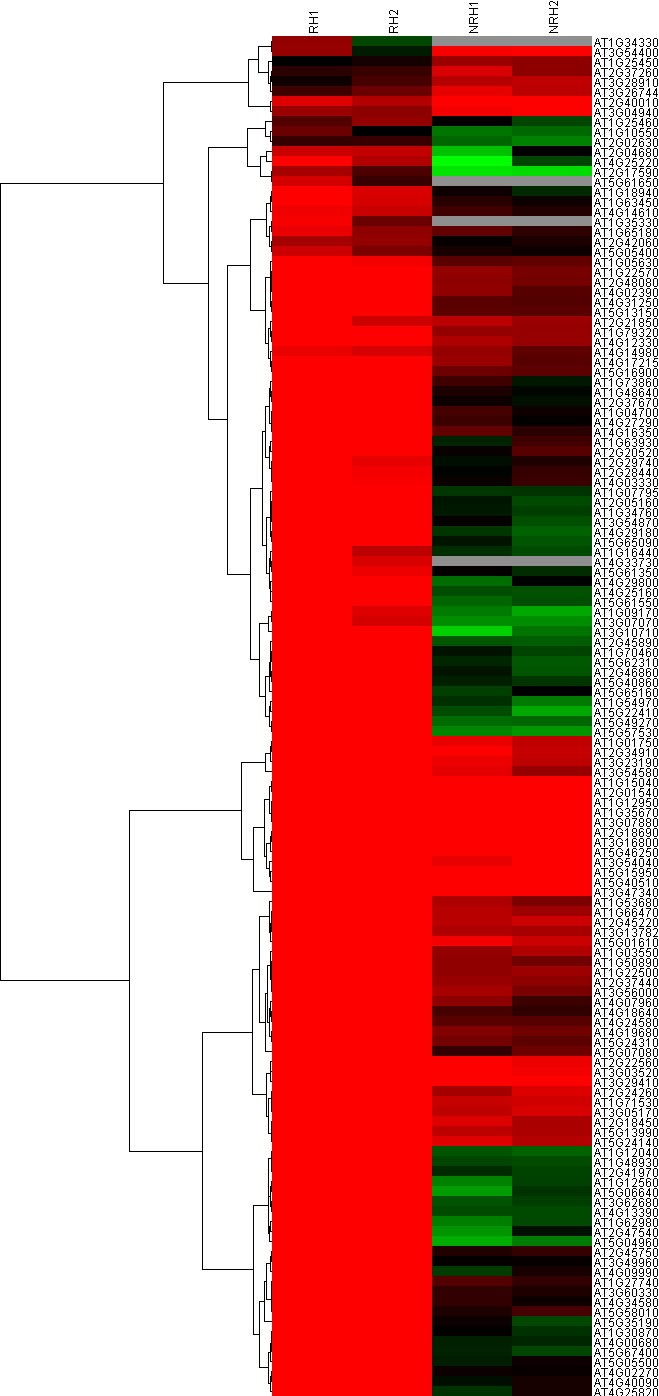

Supplement: Figure S3 — Hierarchical clustering analysis of changes in transcript abundance of 136 overlapping genes (Table S13 in the Supplementary Material) between 208 “core” root epidermal genes (Bruex et al., 2012) and 5409 differentially expressed genes in this study. Transcript abundance was defined as RPKM (Reads Per Kilobase per Millionmapped reads) in the root hairs (RH) and non-root hair tissues (NRH) with two biological repeats. Color key indicates the log2 transformed intensity, gray color which not in the color key indicates that the number is missing. [file Image3.TIF]

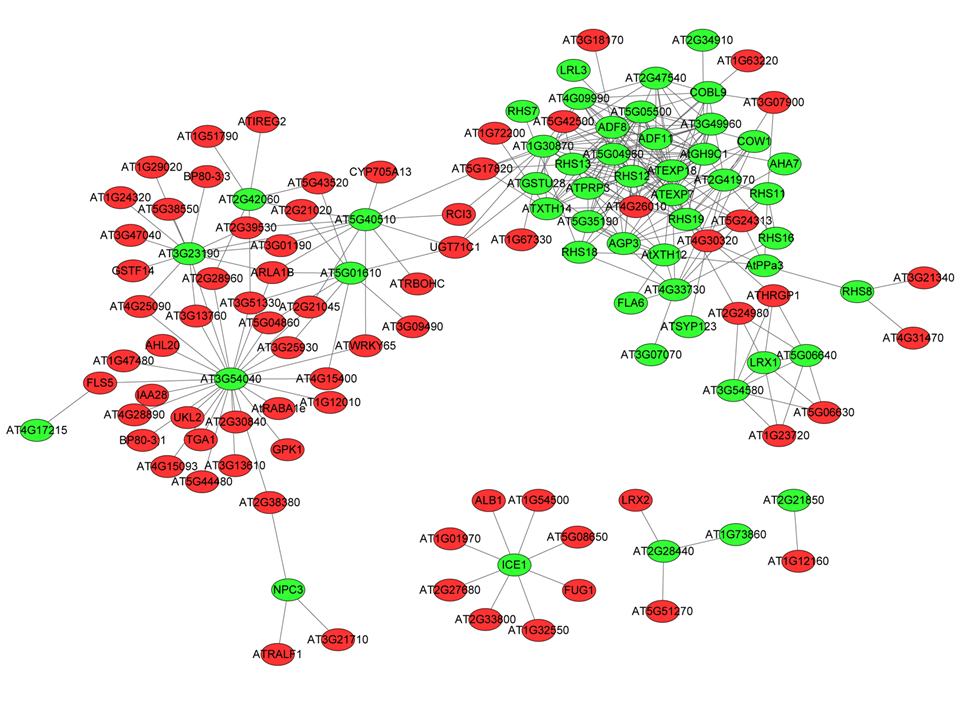

Supplement: Figure S4 — The “core” root epidermal gene associated co-expression newwork of the differentially expressed genes between root hairs (RH) and non-root hair tissues (NRH),with pearson correlation coefficient cutoff at 0.83. Genes in green color indicate bait genes from “core” root epidermal gene and genes in red color indicate prey genes identified in the present study. [file Image4.TIF]

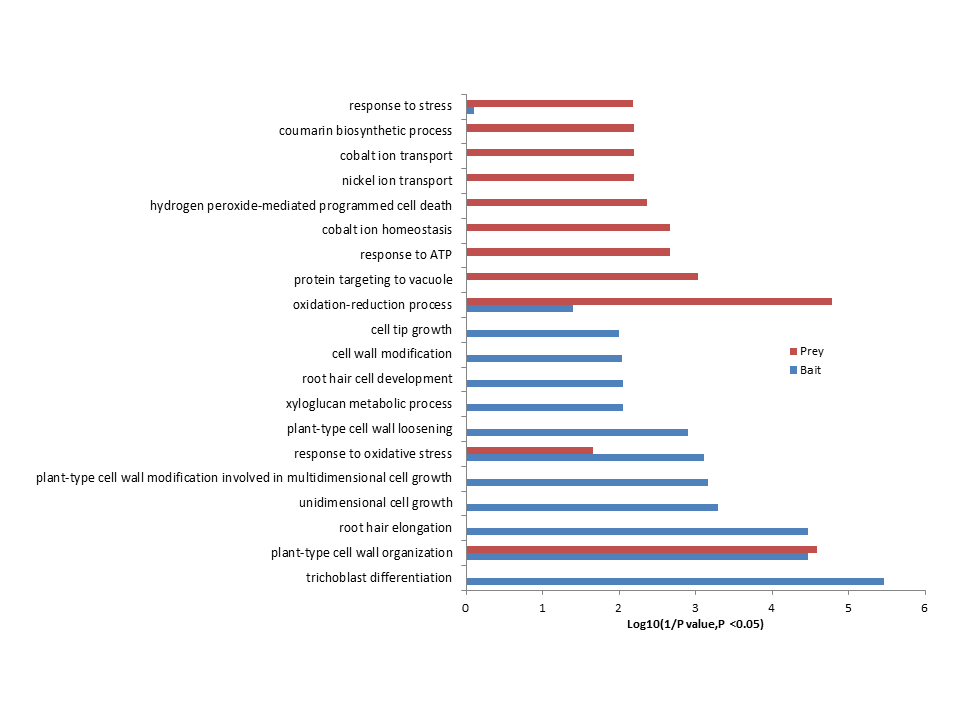

Supplement: Figure S5 — Gene Ontology (GO) enrichment analysis of the bait and prey genes involved in the the “core” root epidermal gene associated co-expression newwork. [file Image5.TIF]
