# Supplementary material for: Re-analysis of RNA-seq transcriptome data reveals new aspects of gene activity in Arabidopsis root hairs
Source: Front Plant Sci. 2015 Jun 8;6:421. doi: 10.3389/fpls.2015.00421 (PMC4458573; doi:10.3389/fpls.2015.00421)
Supplement: Supplementary file 6 [file Table1.DOC]

Table S1 The digital information of gene expression in root hairs (RH) and non-root hair tissues (NRH; RPKM, Reads Per Kilobase of exon model per Million mapped reads)

| **AGI** | **RPKM** | |
| --- | --- | --- |
|  | **RH** | **NRH** |
| AT1G76180 | 4562.78 | 1432.35 |
| AT5G63270 | 4112.99 | 6.5236 |
| AT5G64401 | 3479.22 | 123.939 |
| AT3G15450 | 3385.68 | 1127.62 |
| AT5G65207 | 3333.24 | 1232.51 |
| AT1G67785 | 3258.96 | 334.996 |
| AT2G22470 | 3220.46 | 925.556 |
| AT5G54940 | 3070.01 | 1577.99 |
| AT5G42980 | 2714.9 | 1329.65 |
| AT1G20440 | 2699.7 | 886.206 |
| AT2G23120 | 2371.51 | 560.779 |
| AT1G17190 | 2344.36 | 2175.36 |
| AT5G40730 | 2027.53 | 131.904 |
| AT1G20450 | 2026.31 | 864.185 |
| AT1G66580 | 2021.75 | 1400.11 |
| AT2G15970 | 1992.6 | 553.066 |
| AT5G44020 | 1990.44 | 355.83 |
| AT3G08580 | 1968.22 | 719.857 |
| AT5G20230 | 1947.38 | 1363.1 |
| AT5G11740 | 1943.79 | 814.374 |
| AT1G45145 | 1928.02 | 630.427 |
| AT3G54580 | 1903.3 | 8.85952 |
| AT1G80920 | 1780.59 | 739.613 |
| AT2G24850 | 1764.74 | 1355.24 |
| AT3G28550 | 1748.86 | 62.0652 |
| AT5G64310 | 1739.09 | 501.379 |
| AT2G29450 | 1729.43 | 1210.33 |
| AT2G40000 | 1611.9 | 700.54 |
| AT3G18780 | 1579.69 | 395.117 |
| AT5G15650 | 1568.93 | 581.897 |
| AT5G07440 | 1551.45 | 989.802 |
| AT3G16460 | 1522.3 | 595.036 |
| AT1G35720 | 1520.28 | 613.756 |
| AT1G63245 | 1507.52 | 93.2893 |
| AT3G23030 | 1498.11 | 742.787 |
| AT1G49240 | 1495.81 | 418.454 |
| AT4G02380 | 1491.37 | 504.116 |
| AT2G41430 | 1478.15 | 1242.35 |
| AT3G06420 | 1473.58 | 785.664 |
| AT4G30530 | 1462.25 | 1415.69 |
| AT3G47347 | 1461.62 | 2007.74 |
| AT5G05410 | 1461.19 | 490.103 |
| AT1G23720 | 1454.68 | 45.5663 |
| AT4G21850 | 1416.13 | 864.165 |
| AT3G49780 | 1411.84 | 602.512 |
| AT2G32150 | 1407.9 | 417.731 |
| AT3G13520 | 1407 | 918.673 |
| AT3G62290 | 1398.18 | 608.985 |
| AT1G54410 | 1393.94 | 327.969 |
| AT3G09940 | 1363.51 | 879.208 |
| AT1G09070 | 1357.32 | 334.265 |
| AT1G43160 | 1349.35 | 452.746 |
| AT4G36500 | 1344.94 | 467.384 |
| AT3G26520 | 1343.92 | 157.822 |
| AT2G30870 | 1336.05 | 514.049 |
| AT2G45960 | 1323.36 | 503.512 |
| AT4G39090 | 1309.12 | 413.94 |
| AT5G17820 | 1305.01 | 10.998 |
| AT2G20630 | 1277.98 | 571.82 |
| AT4G01480 | 1274.5 | 153.007 |
| AT4G19200 | 1242.91 | 266.046 |
| AT3G02230 | 1240.45 | 433.154 |
| AT1G07890 | 1219.8 | 462.538 |
| AT2G47730 | 1198.34 | 451.703 |
| AT4G11600 | 1194.3 | 797.536 |
| AT5G64400 | 1193.7 | 401.981 |
| AT4G24960 | 1190.49 | 426.809 |
| AT3G23920 | 1186.89 | 622.489 |
| AT3G48140 | 1171.8 | 457.035 |
| AT2G44790 | 1170.14 | 186.929 |
| AT3G19390 | 1148.84 | 190.392 |
| AT3G53990 | 1138.4 | 893.193 |
| AT4G11150 | 1116.71 | 351.516 |
| AT5G21940 | 1111.76 | 353.043 |
| AT2G36854 | 1102.04 | 123.183 |
| AT3G12510 | 1085.59 | 397.172 |
| AT4G36040 | 1082.74 | 378.673 |
| AT1G04410 | 1068.8 | 582.358 |
| AT1G70780 | 1068.35 | 451.218 |
| AT5G17330 | 1066.23 | 352.955 |
| AT3G21680 | 1032.79 | 343.065 |
| AT3G02470 | 1018.14 | 283.691 |
| AT1G19180 | 1016.23 | 523.243 |
| AT4G30010 | 1013.58 | 348.027 |
| AT5G47120 | 1011.32 | 385.747 |
| AT3G48340 | 1010.16 | 829.133 |
| AT4G40090 | 1002.28 | 1.07251 |
| AT5G08790 | 996.799 | 464.5 |
| AT1G01720 | 995.076 | 319.221 |
| AT5G19230 | 986.846 | 305.293 |
| AT1G74950 | 986.353 | 573.631 |
| AT3G52300 | 984.633 | 441.361 |
| AT5G02230 | 981.784 | 377.193 |
| AT1G03220 | 981.314 | 383.809 |
| AT2G36830 | 974.405 | 103.689 |
| AT1G78900 | 972.528 | 351.844 |
| AT3G50970 | 972.096 | 535.322 |
| AT3G09440 | 966.735 | 719.026 |
| AT4G22592 | 966.459 | 275.933 |
| AT1G08830 | 966.124 | 407.377 |
| AT1G14870 | 962.761 | 431.245 |
| AT5G27760 | 956.105 | 538.482 |
| AT5G39580 | 940.268 | 433.573 |
| AT2G22880 | 937.472 | 489.011 |
| AT5G49450 | 937.389 | 297.292 |
| AT4G22214 | 927.985 | 59.3972 |
| AT1G51650 | 926.751 | 376.214 |
| AT1G19910 | 922.291 | 219.083 |
| AT1G73165 | 922.191 | 34.1179 |
| AT4G15610 | 920.066 | 281.058 |
| AT4G21105 | 917.17 | 390.419 |
| AT3G16240 | 916.7 | 334.163 |
| AT5G43830 | 913.848 | 267.619 |
| AT3G13310 | 901.908 | 177.623 |
| AT3G16640 | 897.259 | 676.751 |
| AT3G54590 | 882.994 | 1.10396 |
| AT5G55180 | 875.051 | 287.572 |
| AT5G11670 | 867.564 | 240.675 |
| AT5G03240 | 860.457 | 395.35 |
| AT1G21310 | 857.72 | 377.717 |
| AT1G78040 | 852.594 | 907.939 |
| AT3G15630 | 848.819 | 164.601 |
| AT5G16010 | 846.964 | 625.311 |
| AT1G05340 | 838.556 | 228.011 |
| AT2G45170 | 836.743 | 428.868 |
| AT5G19110 | 836.29 | 420.398 |
| AT2G38240 | 833.005 | 495.576 |
| AT3G08610 | 830.467 | 376.513 |
| AT4G02270 | 828.721 | 1.12794 |
| AT5G65020 | 827.082 | 811.58 |
| AT1G68440 | 821.724 | 157.151 |
| AT3G53600 | 819.368 | 122.918 |
| AT5G62520 | 818.251 | 368.461 |
| AT5G05600 | 816.306 | 514.779 |
| AT4G32020 | 798.757 | 393.641 |
| AT2G30130 | 796.252 | 456.86 |
| AT2G33040 | 796.22 | 280.518 |
| AT3G55440 | 793.43 | 515.414 |
| AT1G78100 | 786.804 | 66.8369 |
| AT5G07460 | 777.38 | 258.102 |
| AT4G13615 | 772.4 | 107.044 |
| AT1G17860 | 772.33 | 790.088 |
| AT5G63790 | 771.058 | 366.991 |
| AT5G53300 | 767.759 | 454.618 |
| AT1G47128 | 766.855 | 220.755 |
| AT2G17660 | 761.31 | 57.4593 |
| AT1G15010 | 758.503 | 161.428 |
| AT4G25820 | 758.459 | 0.933845 |
| AT4G34720 | 758.112 | 192.907 |
| AT2G47650 | 751.056 | 92.2069 |
| AT2G46140 | 747.755 | 180.962 |
| AT4G20150 | 745.936 | 243.56 |
| AT3G05890 | 745.671 | 1183.73 |
| AT5G60680 | 740.661 | 240.743 |
| AT2G47380 | 735.512 | 273.076 |
| AT3G62420 | 735.276 | 409.708 |
| AT1G75950 | 732.56 | 294.693 |
| AT5G43060 | 731.338 | 365.089 |
| AT4G26080 | 730.496 | 241.738 |
| AT5G26340 | 730.368 | 321.327 |
| AT4G37830 | 729.118 | 198.929 |
| AT5G64100 | 727.186 | 77.0111 |
| AT1G01750 | 723.49 | 10.7564 |
| AT1G63000 | 722.014 | 302.416 |
| AT1G54100 | 714.393 | 516.791 |
| AT1G61275 | 713.21 | 60.8873 |
| AT4G35100 | 712.54 | 304.242 |
| AT4G00860 | 712.244 | 265.061 |
| AT5G65690 | 711.095 | 238.883 |
| AT1G74840 | 705.371 | 302.081 |
| AT5G01750 | 703.089 | 167.121 |
| AT5G64750 | 700.954 | 272.447 |
| AT4G02620 | 700.66 | 205.858 |
| AT2G29440 | 693.561 | 456.971 |
| AT3G15670 | 692.424 | 178.453 |
| AT4G22610 | 689.309 | 192.824 |
| AT4G29350 | 688.303 | 291.369 |
| AT3G10985 | 688.286 | 160.942 |
| AT1G04040 | 686.269 | 170.873 |
| AT3G09925 | 685.787 | 0.79408 |
| AT5G18150 | 684.619 | 60.5441 |
| AT1G76200 | 684.504 | 270.815 |
| AT5G49480 | 684.413 | 218.337 |
| AT1G01470 | 683.577 | 560.734 |
| AT2G20820 | 683.525 | 211.331 |
| AT5G06300 | 683.151 | 123.097 |
| AT2G17840 | 677.79 | 195.594 |
| AT5G13450 | 676.472 | 325.395 |
| AT4G11010 | 673.281 | 447.519 |
| AT1G78380 | 671.725 | 806.851 |
| AT5G54165 | 671.061 | 438.543 |
| AT1G72020 | 670.043 | 272.982 |
| AT5G01410 | 668.851 | 223.114 |
| AT5G42050 | 666.396 | 396.655 |
| AT3G02480 | 665.557 | 441.565 |
| AT3G30775 | 660.893 | 266.421 |
| AT3G52730 | 660.558 | 271.279 |
| AT4G04610 | 660.454 | 473.645 |
| AT5G06320 | 659.23 | 297.317 |
| AT1G15100 | 658.001 | 159.186 |
| AT3G23600 | 655.149 | 100.14 |
| AT3G49551 | 653.475 | 328.746 |
| AT1G15120 | 646.515 | 290.731 |
| AT3G60450 | 643.847 | 174.274 |
| AT4G11220 | 640.308 | 263.694 |
| AT3G06500 | 637.654 | 229.393 |
| AT2G43820 | 633.66 | 77.0631 |
| AT5G47450 | 632.373 | 55.342 |
| AT3G04120 | 631.353 | 684.897 |
| AT4G23630 | 626.743 | 421.145 |
| AT3G47340 | 622.356 | 182.487 |
| AT2G43120 | 621.829 | 250.251 |
| AT4G09000 | 619.099 | 282.817 |
| AT5G52310 | 618.896 | 194.762 |
| AT5G42300 | 614.662 | 283.277 |
| AT4G20830 | 611.451 | 271.399 |
| AT1G62570 | 611.332 | 275.651 |
| AT1G70410 | 610.017 | 263.263 |
| AT2G19760 | 609.899 | 108.766 |
| AT4G23710 | 608.332 | 202.057 |
| AT5G10980 | 606.052 | 433.049 |
| AT1G78660 | 601.741 | 312.068 |
| AT5G59820 | 601.598 | 272.254 |
| AT4G37610 | 600.848 | 226.306 |
| AT4G34050 | 599.839 | 283.075 |
| AT2G21620 | 599.182 | 313.748 |
| AT3G53420 | 598 | 164.361 |
| AT4G39080 | 597.978 | 151.661 |
| AT2G41630 | 596.381 | 388.707 |
| AT3G02780 | 595.907 | 457.266 |
| AT1G70490 | 594.299 | 210.374 |
| AT1G77120 | 594.086 | 164.008 |
| AT5G44340 | 594.043 | 364.612 |
| AT2G38750 | 591.673 | 326.887 |
| AT4G17530 | 591.336 | 262.434 |
| AT2G34910 | 587.714 | 14.9774 |
| AT3G48990 | 579.793 | 314.767 |
| AT3G16420 | 578.787 | 580.413 |
| AT3G44190 | 577.31 | 225.19 |
| AT3G45060 | 573.969 | 59.6096 |
| AT4G38920 | 572.495 | 168.82 |
| AT3G15730 | 571.459 | 237.59 |
| AT3G10860 | 570.387 | 255.834 |
| AT1G22840 | 569.71 | 293.983 |
| AT4G27652 | 566.795 | 192.382 |
| AT5G17920 | 560.349 | 494.697 |
| AT5G59613 | 560.233 | 193.887 |
| AT2G24550 | 556.833 | 266.002 |
| AT5G13220 | 550.087 | 372.275 |
| AT2G21870 | 549.933 | 245.482 |
| AT4G16450 | 549.051 | 222.091 |
| AT4G25570 | 540.611 | 106.292 |
| AT4G30170 | 540.027 | 169.19 |
| AT5G10695 | 537.896 | 203.558 |
| AT1G10170 | 537.884 | 140.222 |
| AT3G56090 | 537.297 | 112.255 |
| AT5G45280 | 536.813 | 351.142 |
| AT2G37110 | 536.592 | 257.269 |
| AT5G12140 | 530.192 | 169.894 |
| AT4G05070 | 530.071 | 174.766 |
| AT4G26710 | 528.377 | 129.273 |
| AT3G05500 | 527.923 | 272.192 |
| AT1G56660 | 519.348 | 80.1407 |
| AT5G28050 | 515.752 | 239.296 |
| AT1G20260 | 515.591 | 182.769 |
| AT1G79550 | 513.816 | 331.394 |
| AT2G41110 | 513.368 | 133.265 |
| AT3G10020 | 509.489 | 202.744 |
| AT2G45070 | 509.131 | 247.166 |
| AT1G01620 | 508.785 | 202.414 |
| AT5G13200 | 507.346 | 105.427 |
| AT2G17720 | 506.32 | 155.026 |
| AT3G24500 | 505.622 | 203.879 |
| AT5G10300 | 504.88 | 490.534 |
| AT4G40060 | 504.476 | 200.452 |
| AT4G33467 | 503.939 | 217.403 |
| AT2G38870 | 502.067 | 482.783 |
| AT4G26970 | 501.061 | 109.825 |
| AT2G27730 | 500.379 | 262.359 |
| AT3G04730 | 500.06 | 122.369 |
| AT4G12040 | 499.133 | 213.044 |
| AT2G44060 | 499.059 | 289.357 |
| AT5G14360 | 497.117 | 314.098 |
| AT5G37600 | 496.386 | 206.551 |
| AT5G39320 | 496.073 | 151.022 |
| AT1G76650 | 496.06 | 429.39 |
| AT5G59490 | 496.028 | 166.133 |
| AT3G52800 | 495.607 | 162.456 |
| AT4G16760 | 494.698 | 214.022 |
| AT2G43340 | 493.845 | 199.06 |
| AT1G26630 | 492.361 | 496.474 |
| AT1G71950 | 492.082 | 214.177 |
| AT1G11910 | 491.562 | 204.151 |
| AT1G74456 | 491.188 | 285.016 |
| AT1G76640 | 489.957 | 564.291 |
| AT4G34700 | 486.693 | 183.75 |
| AT1G67480 | 486.421 | 137.648 |
| AT5G05370 | 484.234 | 100.365 |
| AT1G79245 | 484.196 | 272.779 |
| AT3G25900 | 482.327 | 201.825 |
| AT1G20070 | 482.304 | 301.852 |
| AT5G47200 | 481.876 | 253.215 |
| AT5G48180 | 481.038 | 539.765 |
| AT3G24670 | 481.013 | 139.818 |
| AT1G17170 | 480.337 | 327.331 |
| AT4G39730 | 479.997 | 110.412 |
| AT5G12010 | 479.603 | 208.224 |
| AT4G00680 | 479.401 | 0.676228 |
| AT4G24920 | 479.172 | 311.338 |
| AT2G16660 | 478.088 | 146.703 |
| AT5G19800 | 476.424 | 0.137228 |
| AT3G55720 | 475.965 | 213.649 |
| AT5G62540 | 474.591 | 146.037 |
| AT2G01540 | 474.404 | 22.5354 |
| AT1G22300 | 472.672 | 349.137 |
| AT1G19020 | 472.598 | 155.128 |
| AT3G05490 | 471.929 | 54.1202 |
| AT5G14330 | 469.906 | 9.38705 |
| AT3G07880 | 469.668 | 58.949 |
| AT2G31570 | 469.491 | 140.861 |
| AT3G59350 | 469.079 | 140.433 |
| AT3G42050 | 468.134 | 195.586 |
| AT5G19440 | 467.899 | 385.345 |
| AT5G35190 | 467.479 | 0.808864 |
| AT5G05500 | 467.413 | 0.954079 |
| AT5G06760 | 464.108 | 128.389 |
| AT3G24160 | 464.094 | 172.006 |
| AT4G36988 | 462.778 | 146.7 |
| AT1G79340 | 462.406 | 122.438 |
| AT4G26010 | 461.842 | 2.38604 |
| AT4G29480 | 460.914 | 165.352 |
| AT5G01600 | 460.189 | 253.714 |
| AT1G53470 | 459.764 | 249.713 |
| AT2G43150 | 458.942 | 283.059 |
| AT1G64230 | 458.474 | 223.702 |
| AT1G75220 | 456.642 | 118.127 |
| AT5G47890 | 456.445 | 231.783 |
| AT3G07090 | 456.403 | 267.464 |
| AT5G67400 | 456.332 | 0.570824 |
| AT1G74020 | 455.179 | 312.702 |
| AT5G37780 | 453.683 | 253.504 |
| AT5G47030 | 453.372 | 175.961 |
| AT3G28310 | 449.724 | 54.7267 |
| AT1G12840 | 449.47 | 188.256 |
| AT5G56540 | 449.443 | 312.451 |
| AT1G03850 | 448.238 | 51.9894 |
| AT4G38810 | 447.283 | 155.03 |
| AT5G20250 | 446.084 | 106.141 |
| AT2G22170 | 444.232 | 85.2754 |
| AT2G35743 | 444.088 | 12.0399 |
| AT1G61340 | 443.474 | 283.044 |
| AT2G02990 | 443.127 | 227.149 |
| AT3G18410 | 443.05 | 194.289 |
| AT2G30490 | 442.154 | 184.208 |
| AT2G44670 | 441.449 | 56.6394 |
| AT3G27240 | 440.435 | 125.396 |
| AT4G38060 | 440.394 | 249.63 |
| AT5G01380 | 437.827 | 227.731 |
| AT2G36530 | 436.924 | 347.959 |
| AT4G24380 | 435.951 | 371.088 |
| AT3G20340 | 434.051 | 151.657 |
| AT1G64470 | 433.178 | 139.503 |
| AT5G03630 | 433.105 | 333.696 |
| AT1G30700 | 432.923 | 156.433 |
| AT1G07400 | 432.78 | 257.928 |
| AT5G13180 | 432.024 | 172.947 |
| AT1G17080 | 431.657 | 222.797 |
| AT5G42570 | 431.165 | 178.888 |
| AT5G08670 | 430.79 | 163.692 |
| AT5G64130 | 429.511 | 214.997 |
| AT1G15670 | 429.508 | 122.931 |
| AT4G15470 | 429.191 | 220.775 |
| AT3G13790 | 429.07 | 417.518 |
| AT3G04010 | 428.945 | 30.225 |
| AT1G75860 | 427.08 | 229.852 |
| AT1G62660 | 426.585 | 80.577 |
| AT3G02090 | 424.921 | 169.176 |
| AT5G45350 | 424.782 | 118.27 |
| AT1G17180 | 424.129 | 1383.85 |
| AT1G35580 | 423.926 | 144.599 |
| AT1G80380 | 422.385 | 101.617 |
| AT3G07480 | 421.963 | 112.714 |
| AT3G62680 | 419.939 | 0.453138 |
| AT5G24610 | 419.891 | 262.06 |
| AT3G61260 | 418.234 | 68.3455 |
| AT2G46390 | 417.792 | 177.261 |
| AT2G31490 | 417.256 | 236.678 |
| AT4G14880 | 416.044 | 362.347 |
| AT5G65670 | 416.016 | 189.83 |
| AT4G28300 | 415.183 | 223.461 |
| AT1G27760 | 415.005 | 296.573 |
| AT1G30870 | 413.75 | 0.811178 |
| AT1G66260 | 413.437 | 144.388 |
| AT3G59220 | 413.295 | 320.655 |
| AT5G11110 | 412.702 | 50.0113 |
| AT4G36010 | 412.286 | 229.91 |
| AT3G13410 | 412.011 | 159.687 |
| AT3G54200 | 409.925 | 218.669 |
| AT5G54810 | 408.726 | 187.42 |
| AT1G63220 | 408.652 | 100.17 |
| AT3G62400 | 408.316 | 152.137 |
| AT3G09390 | 408.014 | 252.48 |
| AT5G54170 | 407.99 | 445.499 |
| AT4G05050 | 407.234 | 176.662 |
| AT1G25400 | 406.952 | 87.8463 |
| AT3G52930 | 405.946 | 247.88 |
| AT5G50850 | 404.942 | 183.45 |
| AT1G64460 | 403.738 | 113.635 |
| AT2G29500 | 403.121 | 522.929 |
| AT2G40765 | 400.871 | 98.0433 |
| AT4G28060 | 400.379 | 187.316 |
| AT1G24180 | 398.713 | 113.525 |
| AT3G17020 | 396.817 | 126.494 |
| AT1G35160 | 396.066 | 179.483 |
| AT1G66200 | 395.813 | 225.21 |
| AT2G15960 | 395.141 | 87.6931 |
| AT5G65660 | 394.972 | 216.228 |
| AT1G08200 | 394.919 | 389.972 |
| AT4G25030 | 394.861 | 121.057 |
| AT4G02890 | 394.716 | 251.066 |
| AT2G33150 | 394.557 | 198.907 |
| AT1G53210 | 393.768 | 147.235 |
| AT2G26530 | 393.726 | 200.045 |
| AT3G01390 | 392.874 | 233.371 |
| AT1G66270 | 392.327 | 167.493 |
| AT5G65630 | 391.335 | 267.4 |
| AT1G12560 | 390.921 | 0.365196 |
| AT1G59870 | 390.551 | 253.075 |
| AT4G05010 | 389.783 | 206.137 |
| AT2G23090 | 388.963 | 201.412 |
| AT4G01850 | 388.642 | 302.311 |
| AT1G23490 | 387.257 | 226.29 |
| AT2G45820 | 386.462 | 150.942 |
| AT5G19550 | 385.198 | 131.399 |
| AT1G10140 | 383.338 | 107.714 |
| AT1G12950 | 383.267 | 47.3697 |
| AT5G01820 | 383.094 | 139.568 |
| AT5G15320 | 381.752 | 136.535 |
| AT1G17620 | 380.029 | 140.832 |
| AT1G27290 | 379.088 | 168.749 |
| AT5G16830 | 378.402 | 225.37 |
| AT1G20100 | 377.277 | 179.697 |
| AT3G58730 | 376.431 | 159.899 |
| AT5G67500 | 375.643 | 114.277 |
| AT2G02510 | 375.097 | 137.345 |
| AT2G25450 | 372.779 | 232.118 |
| AT1G65290 | 372.008 | 317.544 |
| AT4G13940 | 371.714 | 467.922 |
| AT3G17810 | 371.095 | 249.025 |
| AT3G60130 | 369.586 | 177.997 |
| AT5G43580 | 368.231 | 780.341 |
| AT2G42680 | 367.039 | 187.103 |
| AT4G24690 | 366.753 | 151.47 |
| AT5G65609 | 366.643 | 9.51047 |
| AT1G66280 | 366.552 | 441.344 |
| AT4G13395 | 366.356 | 81.3887 |
| AT3G23190 | 366.307 | 10.9146 |
| AT3G07273 | 366.138 | 140.894 |
| AT4G30190 | 365.013 | 202.301 |
| AT5G03210 | 364.618 | 152.865 |
| AT3G50900 | 363.701 | 208.659 |
| AT1G51980 | 362.84 | 138.268 |
| AT4G21980 | 362.193 | 165.322 |
| AT4G03510 | 361.587 | 102.716 |
| AT5G67590 | 361.413 | 165.16 |
| AT3G56880 | 361.289 | 166.446 |
| AT2G16900 | 361.164 | 208.121 |
| AT2G30930 | 360.634 | 30.0296 |
| AT1G32920 | 359.973 | 207.368 |
| AT1G05850 | 359.024 | 164.579 |
| AT5G39570 | 358.786 | 142.377 |
| AT1G22410 | 358.604 | 119.588 |
| AT3G09260 | 357.543 | 733.173 |
| AT2G34250 | 357.429 | 223.736 |
| AT3G56800 | 357.222 | 136.684 |
| AT3G03990 | 357.218 | 82.981 |
| AT2G47170 | 357 | 214.75 |
| AT1G23870 | 356.963 | 80.9865 |
| AT1G17290 | 356.526 | 275.931 |
| AT5G03030 | 355.549 | 106.804 |
| AT3G01290 | 354.701 | 86.6315 |
| AT3G16390 | 353.889 | 13.2526 |
| AT3G10920 | 353.627 | 155.362 |
| AT2G34930 | 352.594 | 385.763 |
| AT5G44380 | 352.121 | 218.599 |
| AT2G30362 | 351.438 | 112.469 |
| AT3G48530 | 350.623 | 106.701 |
| AT3G19240 | 350.111 | 98.3167 |
| AT5G06630 | 350.094 | 0.263724 |
| AT5G41080 | 349.839 | 116.471 |
| AT4G22217 | 349.124 | 6.59131 |
| AT4G35090 | 348.67 | 139.931 |
| AT1G28330 | 348.14 | 50.7583 |
| AT1G08930 | 347.06 | 136.441 |
| AT4G13390 | 346.903 | 0.436798 |
| AT3G52470 | 346.601 | 219.559 |
| AT4G01630 | 345.688 | 316.909 |
| AT5G65430 | 345.536 | 150.871 |
| AT1G76600 | 345.153 | 131.333 |
| AT1G64200 | 344.917 | 201.863 |
| AT5G12030 | 344.008 | 301.538 |
| AT4G30600 | 342.377 | 146.458 |
| AT1G28480 | 340.939 | 106.395 |
| AT1G23440 | 340.656 | 174.583 |
| AT1G15040 | 340.555 | 27.5151 |
| AT5G63370 | 339.112 | 164.437 |
| AT3G22830 | 338.883 | 37.2627 |
| AT2G42310 | 337.244 | 111.192 |
| AT4G21830 | 336.848 | 437.911 |
| AT4G27960 | 335.324 | 165.397 |
| AT1G09940 | 333.235 | 126.406 |
| AT1G32230 | 333.167 | 197.398 |
| AT5G38480 | 332.29 | 436.569 |
| AT1G78850 | 331.655 | 341.798 |
| AT1G66410 | 330.29 | 299.797 |
| AT5G40390 | 330.234 | 118.722 |
| AT4G05020 | 329.895 | 167.903 |
| AT1G01550 | 328.923 | 196.826 |
| AT4G01700 | 328.298 | 146.588 |
| AT5G44610 | 327.958 | 24.1866 |
| AT1G09780 | 327.559 | 339.306 |
| AT1G73920 | 327.045 | 114.536 |
| AT3G17860 | 327.036 | 255.448 |
| AT5G53560 | 326.838 | 357.051 |
| AT3G12580 | 326.198 | 247.983 |
| AT1G70290 | 326.187 | 154.226 |
| AT2G46370 | 323.281 | 302.374 |
| AT1G13440 | 322.793 | 412.895 |
| AT5G57625 | 322.516 | 0.234681 |
| AT1G53840 | 322.416 | 209.011 |
| AT1G35670 | 322.302 | 51.0252 |
| AT2G39780 | 322.217 | 93.3188 |
| AT4G03560 | 322.19 | 119.985 |
| AT5G07470 | 322.06 | 74.0045 |
| AT3G02875 | 321.924 | 173.104 |
| AT1G16840 | 321.579 | 141.243 |
| AT3G62010 | 321.403 | 132.627 |
| AT3G55970 | 321.293 | 279.319 |
| AT3G53180 | 320.947 | 218.743 |
| AT3G01280 | 320.896 | 174.063 |
| AT5G10450 | 320.273 | 172.41 |
| AT1G53540 | 319.825 | 384.312 |
| AT1G76030 | 319.447 | 125.435 |
| AT2G41410 | 317.713 | 83.4683 |
| AT1G07750 | 317.538 | 94.6886 |
| AT4G34710 | 317.253 | 168.261 |
| AT5G02500 | 317.06 | 288.754 |
| AT2G36310 | 316.913 | 111.615 |
| AT2G36220 | 316.413 | 68.0069 |
| AT3G52360 | 316.286 | 157.002 |
| AT5G08290 | 316.163 | 154.132 |
| AT5G16910 | 315.453 | 61.5323 |
| AT3G09840 | 314.761 | 219.239 |
| AT5G19760 | 314.314 | 189.167 |
| AT5G50200 | 314.129 | 245.338 |
| AT1G67350 | 314.008 | 140.799 |
| AT3G04720 | 313.931 | 213.522 |
| AT4G11820 | 312.391 | 207.497 |
| AT5G60390 | 311.456 | 534.233 |
| AT2G39900 | 311.315 | 29.5 |
| AT3G60330 | 310.855 | 1.56648 |
| AT3G54130 | 308.151 | 110.16 |
| AT3G15210 | 307.896 | 101.922 |
| AT5G12020 | 307.609 | 231.04 |
| AT5G04430 | 306.548 | 152.038 |
| AT5G03460 | 305.197 | 101.874 |
| AT4G33360 | 304.888 | 57.7994 |
| AT2G16600 | 304.791 | 285.404 |
| AT4G31340 | 304.724 | 190.66 |
| AT1G53680 | 304.142 | 5.34615 |
| AT2G06050 | 303.889 | 148.254 |
| AT3G46230 | 302.766 | 397.058 |
| AT5G10960 | 302.282 | 148.77 |
| AT5G03290 | 301.535 | 106.941 |
| AT4G22820 | 301.504 | 116.647 |
| AT4G27500 | 300.686 | 162.956 |
| AT5G06640 | 300.574 | 0.375472 |
| AT3G02360 | 299.923 | 113.415 |
| AT5G14040 | 299.615 | 200.952 |
| AT5G50460 | 299.025 | 151.591 |
| AT3G23175 | 297.022 | 21.5645 |
| AT2G20360 | 296.278 | 165.414 |
| AT1G70770 | 295.81 | 146.262 |
| AT5G11070 | 294.83 | 12.2172 |
| AT5G21990 | 294.186 | 112.192 |
| AT3G21070 | 293.348 | 88.3031 |
| AT5G62350 | 292.435 | 165.566 |
| AT5G44790 | 291.934 | 177.316 |
| AT2G46540 | 291.699 | 134.979 |
| AT1G30990 | 290.829 | 0.210897 |
| AT1G65930 | 290.648 | 294.534 |
| AT4G01070 | 290.56 | 69.3244 |
| AT2G18700 | 290.362 | 144.664 |
| AT5G04750 | 289.972 | 91.4122 |
| AT1G21980 | 289.898 | 205 |
| AT5G26260 | 289.677 | 321.661 |
| AT2G46170 | 289.426 | 90.2973 |
| AT5G63400 | 288.964 | 173.001 |
| AT2G44450 | 288.944 | 42.9701 |
| AT2G37130 | 288.775 | 234.911 |
| AT3G22850 | 288.673 | 72.8211 |
| AT1G14400 | 288.589 | 169.033 |
| AT5G02020 | 288.556 | 456.119 |
| AT5G19900 | 288.273 | 105.305 |
| AT3G15540 | 288.02 | 22.3099 |
| AT5G54490 | 287.933 | 32.7306 |
| AT4G32060 | 287.227 | 106.634 |
| AT3G54880 | 287.057 | 94.5844 |
| AT1G73260 | 286.717 | 136.376 |
| AT5G22060 | 285.99 | 142.387 |
| AT5G47570 | 285.769 | 121.953 |
| AT2G39110 | 285.64 | 55.4133 |
| AT4G40040 | 285.154 | 216.256 |
| AT4G17615 | 285.13 | 166.806 |
| AT3G14940 | 284.698 | 154.897 |
| AT4G35750 | 284.387 | 301.408 |
| AT5G11770 | 284.336 | 91.0663 |
| AT3G03640 | 284.14 | 177.748 |
| AT2G02050 | 283.544 | 147.793 |
| AT2G18210 | 283.135 | 110.168 |
| AT2G22860 | 282.89 | 264.413 |
| AT2G24980 | 282.55 | 0.145177 |
| AT5G54760 | 281.572 | 173.168 |
| AT4G10040 | 281.489 | 118.794 |
| AT2G40140 | 280.615 | 142.188 |
| AT5G17460 | 280.23 | 54.1676 |
| AT1G18210 | 279.4 | 97.969 |
| AT4G32470 | 279.211 | 132.354 |
| AT2G27860 | 278.937 | 224.626 |
| AT2G47950 | 278.231 | 74.4824 |
| AT1G50010 | 278.045 | 172.952 |
| AT1G71000 | 277.718 | 95.9805 |
| AT3G11340 | 276.9 | 132.454 |
| AT1G15400 | 276.42 | 80.8868 |
| AT5G54160 | 275.654 | 59.0608 |
| AT5G08530 | 275.606 | 157.919 |
| AT5G01800 | 275.465 | 82.1149 |
| AT5G54370 | 275.391 | 926.7 |
| AT2G39770 | 275.284 | 289.382 |
| AT2G19800 | 275.031 | 153.795 |
| AT5G18670 | 274.994 | 35.5265 |
| AT2G36320 | 274.787 | 76.6466 |
| AT4G33920 | 274.739 | 69.901 |
| AT2G25520 | 274.687 | 80.6351 |
| AT3G01190 | 273.852 | 56.5778 |
| AT2G17420 | 273.676 | 180.099 |
| AT5G58010 | 273.512 | 1.80494 |
| AT5G19120 | 273.504 | 80.5682 |
| AT5G56550 | 273.422 | 93.5739 |
| AT1G56330 | 272.732 | 216.814 |
| AT2G01450 | 272.446 | 196.793 |
| AT2G02960 | 272.174 | 42.1596 |
| AT4G23850 | 271.792 | 187.949 |
| AT3G59920 | 271.789 | 125.298 |
| AT4G23885 | 271.443 | 94.7762 |
| AT2G17450 | 270.99 | 113.16 |
| AT2G41660 | 270.944 | 77.6576 |
| AT3G55410 | 270.798 | 151.574 |
| AT4G22666 | 270.642 | 8.73029 |
| AT5G01350 | 270.581 | 222.856 |
| AT5G15970 | 269.941 | 212.915 |
| AT1G09560 | 269.882 | 263.092 |
| AT1G22985 | 269.88 | 82.3759 |
| AT3G02140 | 269.651 | 68.7033 |
| AT2G31390 | 269.533 | 396.36 |
| AT4G13180 | 269.375 | 176.803 |
| AT3G06300 | 269.199 | 57.8294 |
| AT5G11230 | 268.903 | 69.1341 |
| AT4G22220 | 268.707 | 172.167 |
| AT3G03250 | 268.528 | 102.339 |
| AT1G15270 | 268.415 | 237.138 |
| AT3G62830 | 268.258 | 80.6796 |
| AT3G03341 | 268.034 | 73.3189 |
| AT1G15385 | 267.932 | 422.266 |
| AT1G13390 | 267.851 | 85.197 |
| AT2G46330 | 267.778 | 142.706 |
| AT4G27320 | 267.712 | 74.9122 |
| AT1G08880 | 267.518 | 69.502 |
| AT4G00585 | 267.342 | 120.274 |
| AT2G46260 | 267.275 | 99.4655 |
| AT2G18160 | 267.082 | 116.389 |
| AT1G63010 | 266.377 | 124.387 |
| AT4G32530 | 266.152 | 88.1837 |
| AT4G23650 | 265.925 | 156.757 |
| AT3G44110 | 265.501 | 256.333 |
| AT2G28910 | 265.293 | 133.164 |
| AT1G69760 | 264.672 | 198.266 |
| AT2G24100 | 264.461 | 141.371 |
| AT5G47880 | 264.006 | 122.814 |
| AT3G44860 | 263.958 | 138.229 |
| AT5G07220 | 263.913 | 39.6005 |
| AT4G38510 | 263.636 | 117.476 |
| AT2G04520 | 263.464 | 163.795 |
| AT1G71697 | 263.443 | 167.059 |
| AT4G14960 | 263.122 | 194.623 |
| AT3G15356 | 262.734 | 86.5204 |
| AT5G11650 | 262.68 | 108.641 |
| AT2G27510 | 262.513 | 135.99 |
| AT5G66650 | 262.465 | 81.8255 |
| AT4G34110 | 262.381 | 198.963 |
| AT4G37710 | 261.992 | 158.392 |
| AT4G27450 | 261.962 | 69.8234 |
| AT2G27310 | 260.642 | 102.161 |
| AT2G11270 | 260.422 | 10.0694 |
| AT3G09280 | 260.201 | 45.2117 |
| AT3G26690 | 260.17 | 109.635 |
| AT2G44350 | 259.579 | 119.996 |
| AT3G54040 | 259.244 | 20.6245 |
| AT1G43890 | 259.068 | 165.362 |
| AT3G30390 | 259.037 | 89.0239 |
| AT4G34230 | 258.739 | 89.1349 |
| AT3G61430 | 258.699 | 113.041 |
| AT5G57510 | 258.131 | 61.1094 |
| AT2G42210 | 257.807 | 150.485 |
| AT1G09740 | 256.845 | 66.0785 |
| AT1G03900 | 256.122 | 76.8172 |
| AT1G78070 | 255.566 | 55.4027 |
| AT3G02880 | 255.226 | 67.3298 |
| AT5G67480 | 255.167 | 71.6529 |
| AT1G08920 | 254.934 | 116.69 |
| AT5G66510 | 254.873 | 96.5655 |
| AT2G46600 | 254.759 | 84.4076 |
| AT3G63010 | 254.594 | 142.911 |
| AT1G27740 | 254.49 | 2.16234 |
| AT3G63310 | 254.081 | 114.318 |
| AT3G17780 | 254.07 | 91.3394 |
| AT3G51550 | 253.929 | 106.244 |
| AT4G12000 | 253.892 | 78.3078 |
| AT4G30490 | 253.718 | 275.69 |
| AT4G17100 | 252.679 | 41.4201 |
| AT3G13200 | 252.642 | 187.863 |
| AT5G55290 | 252.328 | 90.4295 |
| AT3G22910 | 252.223 | 84.0932 |
| AT4G21570 | 251.908 | 298.661 |
| AT1G58270 | 251.666 | 47.6801 |
| AT3G43810 | 251.12 | 165.535 |
| AT4G32410 | 251.085 | 168.471 |
| AT1G25560 | 250.875 | 98.3487 |
| AT3G48890 | 250.768 | 144.74 |
| AT4G39980 | 250.454 | 109.341 |
| AT5G58290 | 249.238 | 160.389 |
| AT3G13930 | 248.711 | 108.991 |
| AT4G02580 | 248.636 | 142.283 |
| AT5G20080 | 248.57 | 98.5745 |
| AT4G25670 | 248.562 | 145.974 |
| AT4G27870 | 248.464 | 143.536 |
| AT5G64260 | 247.955 | 93.8201 |
| AT3G28710 | 247.945 | 84.908 |
| AT5G56980 | 246.66 | 167.521 |
| AT4G21790 | 246.026 | 109.42 |
| AT2G28400 | 246.001 | 234.168 |
| AT5G26667 | 245.813 | 137.343 |
| AT3G17240 | 245.422 | 113.617 |
| AT2G47270 | 245.218 | 122.011 |
| AT4G27090 | 244.956 | 783.115 |
| AT4G27350 | 244.81 | 25.1782 |
| AT5G42380 | 244.732 | 66.6011 |
| AT1G04250 | 244.712 | 93.5005 |
| AT5G13190 | 244.012 | 114.144 |
| AT5G56760 | 243.944 | 70.4109 |
| AT3G55770 | 243.928 | 74.1422 |
| AT1G78300 | 243.516 | 102.429 |
| AT2G20760 | 243.472 | 102.675 |
| AT5G49440 | 242.918 | 78.3335 |
| AT2G38470 | 242.839 | 167.743 |
| AT5G40510 | 242.636 | 25.6875 |
| AT3G09810 | 242.394 | 64.7445 |
| AT2G16510 | 242.249 | 69.5824 |
| AT5G61890 | 242.215 | 129.396 |
| AT4G32150 | 241.923 | 75.0958 |
| AT4G32950 | 241.781 | 9.18321 |
| AT1G06620 | 241.712 | 129.37 |
| AT3G03100 | 241.501 | 157.849 |
| AT3G52590 | 241.465 | 655.215 |
| AT1G80230 | 240.118 | 46.4778 |
| AT5G20830 | 240.01 | 105.488 |
| AT4G11570 | 239.698 | 122.784 |
| AT5G58070 | 239.412 | 129.702 |
| AT1G22930 | 239.318 | 93.3886 |
| AT1G65541 | 238.99 | 80.1863 |
| AT3G05880 | 238.971 | 472.638 |
| AT1G25275 | 238.95 | 935.678 |
| AT3G52450 | 238.403 | 100.138 |
| AT5G48810 | 237.781 | 155.069 |
| AT2G06530 | 237.631 | 122.074 |
| AT4G29780 | 237.626 | 91.4077 |
| AT1G16180 | 237.602 | 105.555 |
| AT4G32480 | 237.518 | 93.3727 |
| AT5G56630 | 237.492 | 93.6197 |
| AT1G61250 | 237.478 | 124.116 |
| AT5G47990 | 237.397 | 154.854 |
| AT3G46060 | 237.39 | 124.493 |
| AT4G35860 | 236.916 | 103.782 |
| AT5G48230 | 236.635 | 150.844 |
| AT1G77680 | 236.464 | 88.1867 |
| AT4G24340 | 236.12 | 362.547 |
| AT3G19030 | 235.993 | 30.9055 |
| AT5G54500 | 235.851 | 97.0811 |
| AT1G54050 | 235.772 | 315.976 |
| AT5G58320 | 235.739 | 65.6598 |
| AT4G30670 | 235.479 | 4.89032 |
| AT2G39420 | 235.237 | 199.002 |
| AT5G64350 | 234.547 | 183.705 |
| AT2G38760 | 234.278 | 158.622 |
| AT4G19395 | 234.183 | 18.9599 |
| AT5G18800 | 233.45 | 102.954 |
| AT1G08570 | 233.394 | 120.241 |
| AT4G33930 | 232.831 | 103.786 |
| AT3G09350 | 232.723 | 248.519 |
| AT1G22280 | 232.56 | 168.851 |
| AT5G59290 | 232.418 | 123.461 |
| AT2G41970 | 232.38 | 0.551853 |
| AT2G21160 | 231.934 | 214.261 |
| AT2G12400 | 231.808 | 49.4136 |
| AT2G39050 | 231.753 | 55.3765 |
| AT3G15640 | 231.597 | 83.4077 |
| AT3G25230 | 231.411 | 174.017 |
| AT5G56350 | 231.394 | 110.26 |
| AT3G53820 | 231.311 | 14.896 |
| AT4G15800 | 231.158 | 56.5528 |
| AT5G23820 | 230.346 | 597.935 |
| AT2G47485 | 230.02 | 121.859 |
| AT2G30500 | 229.985 | 172.703 |
| AT5G04740 | 229.554 | 110.421 |
| AT3G57870 | 229.351 | 113.572 |
| AT5G04238 | 229.223 | 30.4009 |
| AT3G03070 | 228.785 | 112.424 |
| AT2G36880 | 228.437 | 130.828 |
| AT4G05150 | 228.29 | 40.7379 |
| AT3G15950 | 227.756 | 223.484 |
| AT5G35735 | 227.712 | 209.288 |
| AT4G09570 | 227.361 | 81.021 |
| AT3G29034 | 227.012 | 138.914 |
| AT4G21990 | 226.929 | 326.367 |
| AT5G11520 | 226.478 | 129.274 |
| AT3G61560 | 226.411 | 11.9469 |
| AT1G65820 | 226.348 | 300.202 |
| AT5G03380 | 226.259 | 93.4592 |
| AT5G11970 | 225.769 | 70.8363 |
| AT3G48570 | 225.697 | 97.2641 |
| AT5G15230 | 225.647 | 830.231 |
| AT4G20260 | 225.514 | 96.1149 |
| AT2G01880 | 225.248 | 133.931 |
| AT5G52640 | 225.224 | 176.531 |
| AT1G10370 | 224.731 | 53.1103 |
| AT5G65300 | 224.482 | 154.539 |
| AT4G22310 | 224.335 | 87.2638 |
| AT1G14320 | 224.222 | 475.646 |
| AT3G56240 | 224.175 | 30.4103 |
| AT5G12340 | 223.717 | 36.0318 |
| AT1G32460 | 223.483 | 58.4616 |
| AT4G20860 | 223.47 | 46.4183 |
| AT4G23470 | 223.15 | 69.2986 |
| AT1G79310 | 223.081 | 197.515 |
| AT5G16360 | 223.009 | 124.568 |
| AT4G38800 | 222.955 | 85.7774 |
| AT3G12630 | 222.784 | 94.833 |
| AT1G51760 | 222.689 | 147.649 |
| AT4G18950 | 222.524 | 184.075 |
| AT1G52240 | 222.34 | 25.3168 |
| AT3G14280 | 222.09 | 17.6475 |
| AT5G45110 | 221.978 | 114.438 |
| AT3G12120 | 221.97 | 189.76 |
| AT1G76930 | 221.946 | 66.8919 |
| AT3G62450 | 221.769 | 33.3836 |
| AT4G34350 | 221.379 | 103.533 |
| AT1G53830 | 221.151 | 18.3097 |
| AT1G66240 | 221.148 | 179.656 |
| AT2G03680 | 221.113 | 99.607 |
| AT5G53250 | 220.897 | 22.9246 |
| AT4G13510 | 220.847 | 76.1917 |
| AT2G29420 | 220.431 | 575.141 |
| AT4G16380 | 220.292 | 128.583 |
| AT5G20150 | 219.85 | 108.818 |
| AT1G29640 | 219.314 | 93.2016 |
| AT1G80840 | 219.216 | 132.122 |
| AT5G13548 | 219.121 | 38.4399 |
| AT1G16700 | 219.072 | 88.1035 |
| AT2G05710 | 218.803 | 134.248 |
| AT2G45400 | 218.784 | 136.151 |
| AT1G28200 | 218.519 | 79.9274 |
| AT5G20290 | 218.415 | 600.703 |
| AT1G21380 | 218.263 | 83.266 |
| AT4G34580 | 218.104 | 1.47609 |
| AT5G55850 | 218.099 | 96.4481 |
| AT5G13500 | 217.879 | 36.9633 |
| AT5G51060 | 217.368 | 44.0457 |
| AT4G11393 | 216.883 | 82.9249 |
| AT5G55090 | 216.756 | 161.867 |
| AT4G24160 | 216.539 | 166.86 |
| AT1G33800 | 216.503 | 14.4211 |
| AT2G37170 | 216.435 | 72.4597 |
| AT3G10300 | 216.4 | 65.7395 |
| AT3G62260 | 216.198 | 57.4319 |
| AT2G47690 | 215.916 | 101.323 |
| AT5G02260 | 215.734 | 562.346 |
| AT5G66070 | 215.533 | 79.2505 |
| AT3G28320 | 215.373 | 87.9915 |
| AT3G03050 | 215.2 | 66.4501 |
| AT4G26690 | 215.115 | 40.482 |
| AT3G22200 | 215.053 | 101.86 |
| AT2G32240 | 213.787 | 122.795 |
| AT2G46505 | 213.752 | 89.5518 |
| AT1G53240 | 213.697 | 188.684 |
| AT4G02570 | 213.658 | 137.177 |
| AT1G01650 | 213.552 | 115.629 |
| AT3G20410 | 213.485 | 32.5019 |
| AT2G47540 | 213.419 | 0.513428 |
| AT4G01610 | 213.335 | 146.334 |
| AT3G20510 | 213.264 | 60.2618 |
| AT5G50960 | 213.222 | 110.653 |
| AT3G53620 | 213.189 | 128.196 |
| AT3G57520 | 213.073 | 82.0158 |
| AT3G22845 | 212.96 | 129.691 |
| AT1G48930 | 212.097 | 0.450867 |
| AT5G61210 | 212.044 | 127.042 |
| AT4G31290 | 211.777 | 271.39 |
| AT2G17130 | 211.379 | 37.2411 |
| AT2G41100 | 211.308 | 150.166 |
| AT3G04640 | 211.308 | 107.395 |
| AT3G51460 | 210.894 | 52.7531 |
| AT4G11360 | 210.173 | 129.724 |
| AT1G02500 | 209.882 | 143.836 |
| AT2G36380 | 209.775 | 114.576 |
| AT3G13782 | 209.37 | 6.5882 |
| AT4G19640 | 209.27 | 102.185 |
| AT5G15870 | 209.111 | 71.5251 |
| AT2G01150 | 208.721 | 108.669 |
| AT4G34450 | 208.541 | 167.797 |
| AT1G62980 | 208.435 | 0.345746 |
| AT5G58020 | 208.417 | 76.9897 |
| AT2G39570 | 208.337 | 39.6954 |
| AT4G27280 | 208.262 | 66.1948 |
| AT2G23810 | 207.95 | 157.33 |
| AT5G56170 | 207.643 | 63.5561 |
| AT4G16500 | 207.56 | 224.313 |
| AT1G13360 | 206.939 | 94.3955 |
| AT5G53460 | 206.911 | 180.459 |
| AT5G17650 | 206.846 | 125.716 |
| AT1G78080 | 206.838 | 151.802 |
| AT3G55840 | 206.042 | 64.332 |
| AT1G56280 | 205.648 | 93.3107 |
| AT5G49720 | 205.233 | 184.604 |
| AT1G77450 | 205.205 | 38.3499 |
| AT2G33220 | 204.967 | 97.6293 |
| AT1G69620 | 204.899 | 513.707 |
| AT1G53380 | 204.34 | 89.7754 |
| AT4G32030 | 204.22 | 76.3839 |
| AT3G02468 | 204.04 | 62.3007 |
| AT3G03170 | 203.964 | 59.0553 |
| AT5G25540 | 203.704 | 103.618 |
| AT5G23220 | 203.627 | 31.0288 |
| AT1G10590 | 203.603 | 146.271 |
| AT2G17440 | 203.578 | 83.2521 |
| AT1G47420 | 203.378 | 120.133 |
| AT5G47040 | 203.331 | 54.7876 |
| AT1G67430 | 202.884 | 541.451 |
| AT5G46890 | 202.875 | 70.645 |
| AT5G04410 | 202.792 | 132.759 |
| AT4G32940 | 202.72 | 120.689 |
| AT5G39950 | 202.689 | 103.976 |
| AT2G01140 | 202.672 | 98.5342 |
| AT1G30730 | 202.581 | 98.7262 |
| AT3G62770 | 202.579 | 66.6879 |
| AT2G44110 | 202.182 | 4.12752 |
| AT5G14030 | 202.11 | 126.988 |
| AT3G58680 | 201.793 | 105.212 |
| AT1G14200 | 201.43 | 159.012 |
| AT1G56070 | 201.429 | 320.325 |
| AT4G26910 | 201.259 | 59.6843 |
| AT1G14860 | 201.252 | 60.6038 |
| AT4G27410 | 201.019 | 32.0744 |
| AT3G43960 | 200.497 | 6.84545 |
| AT5G50920 | 200.439 | 162.731 |
| AT5G41700 | 200.335 | 103.878 |
| AT3G23050 | 200.176 | 214.596 |
| AT2G43018 | 199.91 | 111.824 |
| AT5G45130 | 199.808 | 90.5785 |
| AT1G10670 | 199.759 | 211.706 |
| AT2G20670 | 198.928 | 14.4747 |
| AT4G38140 | 198.819 | 76.0474 |
| AT1G20110 | 198.756 | 67.4318 |
| AT5G10430 | 198.707 | 53.4325 |
| AT4G12400 | 198.64 | 163.061 |
| AT4G24230 | 198.492 | 40.9265 |
| AT1G29400 | 198.348 | 87.6633 |
| AT1G27310 | 198.14 | 82.3122 |
| AT5G15090 | 198.111 | 167.884 |
| AT3G07390 | 198.071 | 140.519 |
| AT1G54970 | 197.916 | 0.420925 |
| AT2G25810 | 197.839 | 13.7724 |
| AT1G66400 | 197.625 | 122.733 |
| AT2G45790 | 197.612 | 162.571 |
| AT5G66050 | 197.494 | 98.0434 |
| AT2G01670 | 197.421 | 84.1543 |
| AT3G21510 | 197.317 | 96.1353 |
| AT5G08060 | 197.224 | 72.4427 |
| AT5G45410 | 197.066 | 73.4435 |
| AT2G41475 | 196.753 | 91.4025 |
| AT4G31800 | 196.736 | 153.572 |
| AT5G51440 | 196.686 | 80.3616 |
| AT5G22555 | 196.529 | 0.860094 |
| AT1G53580 | 196.427 | 63.1094 |
| AT1G23710 | 195.97 | 87.8665 |
| AT2G43510 | 195.768 | 102.6 |
| AT5G67300 | 195.702 | 53.0075 |
| AT3G47810 | 195.67 | 100.043 |
| AT3G13110 | 195.547 | 110.921 |
| AT3G05580 | 195.42 | 47.9499 |
| AT5G15950 | 195.241 | 34.0288 |
| AT5G40880 | 195.033 | 70.7431 |
| AT5G28060 | 194.707 | 597.791 |
| AT1G76990 | 194.67 | 52.2885 |
| AT5G56150 | 194.655 | 54.6342 |
| AT5G42650 | 194.635 | 99.1928 |
| AT3G51160 | 194.458 | 49.9687 |
| AT2G37750 | 194.203 | 32.2008 |
| AT4G18710 | 194.114 | 141.017 |
| AT2G33470 | 193.806 | 82.4859 |
| AT1G47260 | 193.637 | 112.789 |
| AT4G29950 | 193.441 | 97.1455 |
| AT4G34410 | 193.39 | 67.1881 |
| AT2G35736 | 193.231 | 30.2898 |
| AT1G51200 | 193.209 | 100.845 |
| AT4G29130 | 193.156 | 95.4035 |
| AT1G53920 | 192.956 | 43.0331 |
| AT5G39510 | 192.795 | 81.8063 |
| AT2G41810 | 192.726 | 273.555 |
| AT4G36880 | 192.723 | 35.1567 |
| AT1G04280 | 192.645 | 3.74287 |
| AT2G22430 | 192.545 | 114.194 |
| AT4G00430 | 192.525 | 76.9364 |
| AT3G60600 | 192.455 | 108.319 |
| AT1G76980 | 192.358 | 74.9205 |
| AT2G47140 | 192.21 | 92.5895 |
| AT1G76680 | 192.188 | 578.059 |
| AT1G17340 | 191.977 | 34.9336 |
| AT1G75750 | 191.877 | 70.459 |
| AT4G36800 | 191.714 | 114.53 |
| AT3G56190 | 191.553 | 97.8097 |
| AT3G01520 | 191.549 | 26.7047 |
| AT5G37510 | 191.545 | 96.7445 |
| AT5G60360 | 191.445 | 101.867 |
| AT3G12260 | 191.433 | 71.5732 |
| AT2G43160 | 190.965 | 75.5139 |
| AT3G49570 | 190.802 | 70.5889 |
| AT5G66760 | 190.781 | 133.639 |
| AT2G44620 | 190.649 | 123.748 |
| AT5G58730 | 190.387 | 38.1269 |
| AT1G55210 | 190.279 | 97.1365 |
| AT3G21710 | 190.137 | 19.9234 |
| AT4G32070 | 189.746 | 67.5944 |
| AT3G29575 | 189.405 | 52.182 |
| AT5G52840 | 189.27 | 89.7926 |
| AT4G09990 | 189.187 | 0.903108 |
| AT1G15690 | 188.878 | 196.863 |
| AT4G30850 | 188.51 | 103.888 |
| AT1G62300 | 188.38 | 66.6406 |
| AT5G44060 | 187.904 | 62.7145 |
| AT1G55530 | 187.9 | 82.6831 |
| AT2G45670 | 187.439 | 65.3763 |
| AT5G07350 | 187.309 | 154.498 |
| AT1G74310 | 187.077 | 251.334 |
| AT1G14010 | 186.949 | 59.1083 |
| AT5G43460 | 186.786 | 145.331 |
| AT1G56045 | 186.746 | 510.413 |
| AT5G64740 | 186.642 | 99.3864 |
| AT4G33310 | 186.622 | 7.05964 |
| AT1G30135 | 186.526 | 110.669 |
| AT1G22530 | 186.164 | 19.0348 |
| AT4G34150 | 186.066 | 117.084 |
| AT1G27000 | 185.99 | 92.4662 |
| AT1G65980 | 185.966 | 188.721 |
| AT4G34180 | 185.817 | 174.282 |
| AT5G18400 | 185.8 | 96.9797 |
| AT3G06760 | 185.496 | 102.039 |
| AT3G59050 | 185.259 | 70.166 |
| AT2G30440 | 185.245 | 128.165 |
| AT3G54620 | 185.18 | 79.2276 |
| AT1G78830 | 185.136 | 72.9902 |
| AT5G16510 | 185.052 | 59.6975 |
| AT3G07680 | 184.927 | 106.524 |
| AT1G12040 | 184.771 | 0.367391 |
| AT2G44840 | 184.521 | 67.1459 |
| AT2G45220 | 184.446 | 8.61363 |
| AT3G50910 | 184.335 | 95.6711 |
| AT4G14500 | 184.224 | 48.7814 |
| AT5G12050 | 184.136 | 3.34175 |
| AT5G16880 | 183.767 | 118.568 |
| AT4G03430 | 183.66 | 72.922 |
| AT5G03850 | 183.608 | 453 |
| AT3G51730 | 183.602 | 105.322 |
| AT4G38470 | 183.567 | 126.183 |
| AT1G64040 | 183.348 | 191.965 |
| AT5G62460 | 182.813 | 57.8837 |
| AT2G19572 | 182.798 | 82.1952 |
| AT1G32928 | 182.732 | 39.5439 |
| AT1G04820 | 182.379 | 148.938 |
| AT1G73030 | 182.181 | 80.5787 |
| AT1G27970 | 181.63 | 101.514 |
| AT1G14170 | 181.446 | 32.3096 |
| AT1G23190 | 181.41 | 101.102 |
| AT1G16890 | 181.343 | 116.241 |
| AT3G13920 | 181.072 | 235.102 |
| AT1G14450 | 180.953 | 83.9771 |
| AT5G01610 | 180.271 | 12.2245 |
| AT5G20090 | 180.122 | 67.2339 |
| AT2G37250 | 180.082 | 81.5465 |
| AT3G03270 | 179.977 | 187.253 |
| AT2G02710 | 179.907 | 55.9555 |
| AT3G06050 | 179.906 | 58.7523 |
| AT5G04960 | 179.825 | 0.201765 |
| AT3G54110 | 179.727 | 118.039 |
| AT3G44870 | 179.672 | 96.4691 |
| AT5G27520 | 179.67 | 132.136 |
| AT3G14067 | 179.394 | 38.104 |
| AT1G02780 | 179.329 | 457.563 |
| AT5G27150 | 179.064 | 38.1637 |
| AT1G21000 | 179.053 | 127.83 |
| AT1G76490 | 179.027 | 80.9479 |
| AT1G19530 | 178.746 | 138.015 |
| AT2G46030 | 178.653 | 48.6747 |
| AT5G15265 | 178.615 | 105.169 |
| AT5G57660 | 178.487 | 69.8385 |
| AT5G65110 | 178.231 | 120.947 |
| AT4G24220 | 178.206 | 65.3032 |
| AT1G29280 | 177.666 | 87.5543 |
| AT4G35320 | 177.516 | 63.4578 |
| AT3G05590 | 177.486 | 500.291 |
| AT1G31812 | 177.4 | 173.187 |
| AT1G48440 | 177.066 | 164.702 |
| AT2G43610 | 177.063 | 1205.59 |
| AT1G53310 | 177.034 | 167.213 |
| AT1G15405 | 177.029 | 16.8793 |
| AT4G29190 | 176.941 | 67.8202 |
| AT3G24180 | 176.934 | 62.0748 |
| AT5G16110 | 176.481 | 88.9422 |
| AT3G03160 | 176.314 | 59.4173 |
| AT1G79010 | 176.119 | 105.742 |
| AT4G30210 | 175.977 | 175.719 |
| AT3G14595 | 175.817 | 105.59 |
| AT1G30900 | 175.769 | 11.9986 |
| AT1G14670 | 175.661 | 141.337 |
| AT3G47833 | 175.647 | 57.2911 |
| AT5G64250 | 175.498 | 215.293 |
| AT5G58720 | 175.206 | 123.797 |
| AT5G34850 | 175.202 | 77.4234 |
| AT3G52400 | 174.986 | 37.5873 |
| AT1G19450 | 174.887 | 59.9677 |
| AT5G25890 | 174.637 | 33.5893 |
| AT1G73580 | 174.371 | 0.258723 |
| AT5G01100 | 174.355 | 24.9128 |
| AT4G27130 | 174.147 | 117.113 |
| AT4G08520 | 173.764 | 146.813 |
| AT1G61770 | 173.696 | 64.3107 |
| AT3G61580 | 173.588 | 57.601 |
| AT5G15490 | 173.378 | 99.884 |
| AT1G77940 | 173.36 | 595.726 |
| AT1G73480 | 172.807 | 109.107 |
| AT4G37390 | 172.489 | 3.74332 |
| AT2G32190 | 172.329 | 112.605 |
| AT1G77180 | 171.922 | 117.462 |
| AT3G11800 | 171.859 | 44.4368 |
| AT3G27380 | 171.825 | 65.8095 |
| AT2G36580 | 171.663 | 104.75 |
| AT3G56310 | 171.632 | 90.1474 |
| AT3G49800 | 171.605 | 93.0282 |
| AT5G27850 | 171.525 | 626.004 |
| AT1G76160 | 171.22 | 91.4726 |
| AT3G17410 | 171.128 | 40.5542 |
| AT2G04900 | 170.995 | 87.3716 |
| AT3G11780 | 170.826 | 56.9062 |
| AT3G58500 | 170.699 | 131.003 |
| AT4G22330 | 170.636 | 69.3336 |
| AT5G60790 | 170.631 | 135.41 |
| AT1G65570 | 170.571 | 113.747 |
| AT4G17170 | 170.336 | 77.9387 |
| AT2G47510 | 170.222 | 114.867 |
| AT2G45760 | 170.204 | 83.9064 |
| AT3G07340 | 170.155 | 75.8715 |
| AT1G49300 | 170.033 | 59.0985 |
| AT1G66160 | 169.823 | 27.7535 |
| AT4G34870 | 169.803 | 253.108 |
| AT3G05920 | 169.788 | 11.9305 |
| AT5G67600 | 169.351 | 171.647 |
| AT3G22890 | 168.988 | 167.591 |
| AT2G04170 | 168.974 | 21.5401 |
| AT5G63320 | 168.932 | 145.039 |
| AT1G73080 | 168.918 | 117.059 |
| AT4G27260 | 168.659 | 16.4149 |
| AT1G14830 | 168.55 | 120.434 |
| AT2G01490 | 168.482 | 139.584 |
| AT3G17390 | 168.464 | 166.254 |
| AT3G55430 | 168.431 | 89.3414 |
| AT3G15500 | 168.239 | 88.0189 |
| AT2G17390 | 168.178 | 89.6218 |
| AT5G24260 | 168.142 | 71.0576 |
| AT5G63510 | 167.996 | 55.8076 |
| AT1G01010 | 167.958 | 117.138 |
| AT3G49940 | 167.855 | 39.9379 |
| AT2G41420 | 167.793 | 232.631 |
| AT3G52060 | 167.77 | 62.5968 |
| AT2G46800 | 167.708 | 83.2501 |
| AT1G69510 | 167.703 | 100.595 |
| AT4G30960 | 167.486 | 119.037 |
| AT4G34490 | 167.472 | 59.2951 |
| AT1G49140 | 167.433 | 73.6867 |
| AT5G05010 | 167.26 | 139.591 |
| AT4G01026 | 167.219 | 87.1724 |
| AT5G56750 | 167.173 | 79.4659 |
| AT2G19830 | 167.165 | 84.3504 |
| AT4G34100 | 167.139 | 84.5154 |
| AT5G08690 | 167.043 | 74.5682 |
| AT1G55330 | 166.974 | 104.902 |
| AT3G10960 | 166.839 | 47.4165 |
| AT3G09200 | 166.759 | 365.37 |
| AT5G41992 | 166.711 | 68.5302 |
| AT5G52200 | 166.709 | 96.2985 |
| AT1G08480 | 166.663 | 74.3403 |
| AT1G09970 | 166.662 | 119.712 |
| AT3G49530 | 166.562 | 105.651 |
| AT5G48570 | 166.397 | 153.196 |
| AT1G50600 | 166.353 | 122.166 |
| AT5G35200 | 166.289 | 80.3824 |
| AT4G17230 | 166.049 | 77.2147 |
| AT1G65610 | 165.887 | 7.60339 |
| AT2G01300 | 165.857 | 143.157 |
| AT5G60660 | 165.483 | 27.2862 |
| AT1G22450 | 165.346 | 87.6754 |
| AT2G27600 | 165.339 | 84.052 |
| AT2G20880 | 165.331 | 20.7599 |
| AT2G32270 | 165.322 | 20.7386 |
| AT1G07140 | 165.168 | 98.9417 |
| AT4G37400 | 165.142 | 86.6286 |
| AT1G76240 | 165.122 | 85.795 |
| AT1G03870 | 165.099 | 54.4052 |
| AT5G13080 | 164.884 | 39.5503 |
| AT2G33700 | 164.663 | 88.582 |
| AT1G70660 | 164.031 | 49.7965 |
| AT4G38630 | 163.897 | 108.534 |
| AT3G59210 | 163.819 | 109.117 |
| AT1G09794 | 163.77 | 86.746 |
| AT2G31350 | 163.768 | 15.1616 |
| AT2G26150 | 163.723 | 150.79 |
| AT5G17850 | 163.494 | 81.3051 |
| AT3G60245 | 163.292 | 395.318 |
| AT5G04170 | 163.288 | 55.2346 |
| AT3G28715 | 163.176 | 67.3854 |
| AT4G19230 | 163.144 | 38.5162 |
| AT1G72510 | 163.085 | 126.202 |
| AT1G32640 | 162.918 | 111.796 |
| AT4G22460 | 162.841 | 23.3611 |
| AT5G19860 | 162.762 | 71.951 |
| AT5G16470 | 162.648 | 83.8027 |
| AT1G10630 | 162.625 | 80.8213 |
| AT1G54320 | 162.565 | 57.8693 |
| AT1G20050 | 162.558 | 110.913 |
| AT1G04770 | 162.343 | 88.2119 |
| AT1G02360 | 162.167 | 70.8857 |
| AT4G28770 | 162.082 | 53.7877 |
| AT2G45980 | 161.908 | 69.7028 |
| AT1G53180 | 161.89 | 131.729 |
| AT5G04120 | 161.856 | 117.808 |
| AT1G66470 | 161.46 | 6.58115 |
| AT1G08650 | 161.444 | 64.0705 |
| AT4G37640 | 161.434 | 37.1259 |
| AT4G16520 | 161.317 | 105.979 |
| AT1G23750 | 161.203 | 24.3002 |
| AT1G30690 | 161.157 | 48.9575 |
| AT1G26920 | 161.084 | 160.138 |
| AT2G29460 | 160.781 | 180.004 |
| AT1G77000 | 160.574 | 69.242 |
| AT5G12880 | 160.477 | 10.1072 |
| AT3G46640 | 160.418 | 54.5747 |
| AT2G35680 | 160.344 | 71.5481 |
| AT3G20770 | 160.341 | 132.127 |
| AT3G09500 | 160.176 | 397.975 |
| AT3G52990 | 159.909 | 117.321 |
| AT4G36648 | 159.858 | 108.21 |
| AT5G53050 | 159.641 | 89.0136 |
| AT1G76070 | 159.589 | 33.0842 |
| AT3G49960 | 159.373 | 1.06339 |
| AT1G01730 | 159.28 | 17.9865 |
| AT1G25550 | 159.219 | 59.0713 |
| AT1G63840 | 159.136 | 56.144 |
| AT3G17510 | 159.127 | 101.33 |
| AT3G48100 | 159.127 | 45.2639 |
| AT3G54680 | 159.124 | 46.0036 |
| AT5G45510 | 159.03 | 65.1305 |
| AT1G70700 | 158.898 | 136.027 |
| AT1G47710 | 158.772 | 110.367 |
| AT2G02180 | 158.719 | 99.9517 |
| AT2G18690 | 158.694 | 36.0347 |
| AT3G20300 | 158.605 | 48.7911 |
| AT1G15430 | 158.202 | 89.5264 |
| AT5G40810 | 158.197 | 87.9228 |
| AT3G57530 | 158 | 90.8877 |
| AT4G29160 | 157.983 | 89.8516 |
| AT3G51910 | 157.402 | 103.492 |
| AT3G59280 | 156.975 | 81.548 |
| AT2G27030 | 156.76 | 125.352 |
| AT5G18650 | 156.373 | 96.385 |
| AT4G03520 | 156.213 | 52.1189 |
| AT1G14290 | 156.165 | 86.4917 |
| AT1G01800 | 156.135 | 159.671 |
| AT5G61820 | 156.067 | 280.485 |
| AT4G30280 | 155.654 | 88.4442 |
| AT2G20420 | 155.545 | 111.058 |
| AT3G19580 | 155.351 | 65.2845 |
| AT3G28180 | 155.16 | 87.064 |
| AT3G06700 | 155.026 | 319.977 |
| AT5G57830 | 154.744 | 55.1579 |
| AT3G49845 | 154.697 | 47.7893 |
| AT1G15210 | 154.625 | 106.378 |
| AT4G39780 | 154.394 | 66.145 |
| AT4G35790 | 154.161 | 55.5105 |
| AT1G06410 | 153.871 | 77.9868 |
| AT4G18100 | 153.826 | 535.466 |
| AT5G22920 | 153.729 | 13.2964 |
| AT2G35120 | 153.668 | 171.306 |
| AT1G05810 | 153.349 | 12.397 |
| AT3G21700 | 153.294 | 35.7869 |
| AT5G35680 | 153.188 | 116.948 |
| AT4G22640 | 153.154 | 88.7999 |
| AT5G65210 | 153.007 | 68.1556 |
| AT3G20670 | 152.832 | 91.7405 |
| AT3G51920 | 152.785 | 34.0138 |
| AT3G18820 | 152.473 | 94.3434 |
| AT3G11130 | 152.408 | 91.6651 |
| AT5G04760 | 152.387 | 65.0378 |
| AT1G06570 | 152.351 | 56.6254 |
| AT3G27090 | 152.312 | 114.031 |
| AT2G41840 | 152.097 | 339.905 |
| AT5G59910 | 152.032 | 169.787 |
| AT5G58375 | 151.981 | 61.2825 |
| AT5G15200 | 151.746 | 448.284 |
| AT5G65470 | 151.635 | 100.747 |
| AT4G19860 | 151.628 | 49.5037 |
| AT5G16550 | 151.309 | 56.8968 |
| AT3G27890 | 151.262 | 43.0954 |
| AT2G30110 | 151.211 | 84.9481 |
| AT5G10860 | 150.986 | 183.387 |
| AT5G13490 | 150.766 | 82.5304 |
| AT4G18070 | 150.668 | 44.1548 |
| AT3G22950 | 150.392 | 72.0816 |
| AT2G23790 | 150.339 | 81.8565 |
| AT3G20390 | 150.322 | 155.244 |
| AT5G13330 | 150.29 | 38.518 |
| AT3G55610 | 150.128 | 80.6039 |
| AT3G47730 | 150.124 | 86.6342 |
| AT5G08300 | 150.012 | 78.3418 |
| AT1G15110 | 149.973 | 66.874 |
| AT1G19360 | 149.934 | 47.574 |
| AT4G17900 | 149.916 | 76.1717 |
| AT2G19730 | 149.85 | 428.481 |
| AT3G53480 | 149.703 | 147.172 |
| AT5G59880 | 149.43 | 127.746 |
| AT5G08040 | 149.38 | 144.647 |
| AT4G37620 | 149.344 | 31.0778 |
| AT3G25600 | 149.32 | 33.1992 |
| AT4G13110 | 149.155 | 86.3455 |
| AT2G27080 | 149.092 | 28.6707 |
| AT3G19760 | 149.057 | 112.579 |
| AT4G12720 | 148.906 | 106.02 |
| AT5G15600 | 148.905 | 2.58613 |
| AT3G52200 | 148.89 | 67.1345 |
| AT5G42020 | 148.821 | 103.915 |
| AT4G39675 | 148.814 | 17.1982 |
| AT5G04340 | 148.762 | 60.9907 |
| AT3G49870 | 148.721 | 83.7885 |
| AT5G43780 | 148.717 | 26.1097 |
| AT4G21450 | 148.601 | 98.5868 |
| AT5G46250 | 148.519 | 61.0539 |
| AT4G30290 | 148.307 | 89.7811 |
| AT2G25460 | 148.263 | 68.7616 |
| AT1G16030 | 148.121 | 226.915 |
| AT2G41740 | 148 | 67.3669 |
| AT2G47550 | 147.805 | 76.6359 |
| AT5G22000 | 147.551 | 85.3561 |
| AT3G03150 | 147.501 | 82.0241 |
| AT5G05080 | 147.45 | 90.5731 |
| AT1G30630 | 147.438 | 141.911 |
| AT1G27330 | 147.383 | 92.7231 |
| AT2G47000 | 147.216 | 82.8026 |
| AT1G62740 | 147.182 | 80.5596 |
| AT5G48540 | 146.921 | 177.382 |
| AT3G19010 | 146.836 | 111.407 |
| AT5G63990 | 146.675 | 54.9581 |
| AT3G18950 | 146.545 | 51.2526 |
| AT1G07600 | 146.376 | 47.0929 |
| AT3G13870 | 146.355 | 92.2689 |
| AT1G79660 | 146.249 | 81.5598 |
| AT3G56930 | 146.087 | 13.7746 |
| AT5G56670 | 146.002 | 370.272 |
| AT1G68410 | 145.953 | 46.9068 |
| AT5G15960 | 145.895 | 69.0192 |
| AT3G01540 | 145.772 | 87.3071 |
| AT5G45630 | 145.646 | 39.0753 |
| AT1G21750 | 145.605 | 150.78 |
| AT3G47960 | 145.322 | 55.5909 |
| AT5G63450 | 145.002 | 44.7236 |
| AT5G61780 | 144.926 | 101.272 |
| AT4G17500 | 144.904 | 76.3233 |
| AT1G70590 | 144.528 | 44.3325 |
| AT2G33830 | 144.465 | 23.3838 |
| AT2G43460 | 144.367 | 470.249 |
| AT2G14890 | 144.361 | 190.765 |
| AT4G06746 | 144.094 | 21.8161 |
| AT3G52220 | 144.089 | 78.5093 |
| AT2G20230 | 143.986 | 34.7292 |
| AT4G24820 | 143.923 | 157.867 |
| AT1G53910 | 143.82 | 90.647 |
| AT3G24170 | 143.745 | 79.3281 |
| AT3G18060 | 143.205 | 25.5061 |
| AT4G31780 | 142.992 | 140.8 |
| AT5G48655 | 142.685 | 124.478 |
| AT2G38360 | 142.622 | 57.3274 |
| AT5G56340 | 142.462 | 51.493 |
| AT5G47770 | 142.296 | 146.512 |
| AT1G18460 | 142.281 | 60.435 |
| AT5G17380 | 142.213 | 82.8987 |
| AT1G12360 | 142.14 | 65.539 |
| AT3G62250 | 142.098 | 322.287 |
| AT2G45750 | 142.026 | 1.61829 |
| AT3G27300 | 141.963 | 25.9497 |
| AT4G09150 | 141.343 | 89.7737 |
| AT5G41810 | 141.275 | 162.594 |
| AT4G18700 | 141.222 | 82.2122 |
| AT2G40620 | 141.222 | 56.8848 |
| AT5G11440 | 141.179 | 0.300056 |
| AT1G56220 | 141.148 | 36.1249 |
| AT3G09740 | 141.097 | 73.5206 |
| AT3G51540 | 140.796 | 4.18419 |
| AT3G53110 | 140.675 | 94.7891 |
| AT5G57110 | 140.426 | 86.556 |
| AT1G45688 | 140.17 | 56.5209 |
| AT5G40370 | 139.96 | 123.398 |
| AT3G07780 | 139.491 | 83.3784 |
| AT3G62100 | 139.472 | 9.59808 |
| AT4G30260 | 139.373 | 50.6832 |
| AT3G20060 | 139.218 | 81.454 |
| AT2G37120 | 139.199 | 77.6719 |
| AT2G25610 | 139.097 | 60.0439 |
| AT1G79530 | 139.06 | 102.599 |
| AT4G31985 | 139.057 | 308.019 |
| AT1G12740 | 139.051 | 244.571 |
| AT3G03280 | 139.049 | 46.7287 |
| AT5G02380 | 138.976 | 135.336 |
| AT1G65840 | 138.871 | 67.1144 |
| AT3G53670 | 138.867 | 66.3955 |
| AT4G22212 | 138.844 | 62.8338 |
| AT1G64520 | 138.79 | 124.497 |
| AT5G10625 | 138.725 | 70.768 |
| AT4G15130 | 138.654 | 80.5198 |
| AT1G04400 | 138.599 | 75.6472 |
| AT1G12310 | 138.584 | 50.9035 |
| AT3G17110 | 138.42 | 73.5625 |
| AT1G09140 | 138.305 | 80.1804 |
| AT1G52760 | 138.289 | 48.888 |
| AT4G31750 | 138.088 | 96.4789 |
| AT4G01000 | 138.087 | 81.3963 |
| AT5G16050 | 137.843 | 167.476 |
| AT2G17500 | 137.839 | 84.4176 |
| AT5G11680 | 137.805 | 78.0673 |
| AT1G19380 | 137.654 | 84.0223 |
| AT4G01895 | 137.609 | 70.0451 |
| AT3G13650 | 137.574 | 20.828 |
| AT4G35985 | 137.36 | 27.7708 |
| AT4G14410 | 137.35 | 55.1423 |
| AT5G20190 | 137.08 | 183.549 |
| AT3G05990 | 136.921 | 27.9731 |
| AT3G29360 | 136.779 | 80.7306 |
| AT3G05530 | 136.684 | 123.044 |
| AT5G40860 | 136.684 | 0.628081 |
| AT5G47230 | 136.589 | 24.3925 |
| AT1G29330 | 136.324 | 100.246 |
| AT5G56950 | 136.201 | 39.182 |
| AT5G24620 | 136.189 | 58.7006 |
| AT1G56700 | 135.873 | 20.6475 |
| AT3G47080 | 135.781 | 65.5732 |
| AT5G08080 | 135.671 | 110.008 |
| AT1G03080 | 135.648 | 92.736 |
| AT4G35830 | 135.578 | 160.523 |
| AT5G61530 | 135.558 | 51.2875 |
| AT2G43590 | 135.519 | 175.763 |
| AT2G47710 | 135.497 | 68.2699 |
| AT4G00720 | 135.23 | 58.7615 |
| AT5G13930 | 134.966 | 4.77061 |
| AT5G36880 | 134.904 | 126.203 |
| AT5G45580 | 134.741 | 14.3338 |
| AT1G66500 | 134.628 | 47.8711 |
| AT3G44326 | 134.453 | 85.1163 |
| AT5G60920 | 134.441 | 84.4595 |
| AT5G38710 | 134.102 | 31.5828 |
| AT1G18150 | 134.08 | 30.2339 |
| AT3G52880 | 134.066 | 111.666 |
| AT5G42740 | 133.84 | 49.8466 |
| AT4G35260 | 133.746 | 52.3263 |
| AT3G05840 | 133.74 | 85.4732 |
| AT1G43170 | 133.498 | 425.174 |
| AT3G17090 | 133.495 | 58.9309 |
| AT4G00300 | 133.469 | 50.9446 |
| AT1G51420 | 133.461 | 62.6726 |
| AT3G13435 | 133.389 | 168.376 |
| AT1G09270 | 133.346 | 52.5573 |
| AT3G57330 | 132.976 | 37.6509 |
| AT2G46270 | 132.972 | 67.461 |
| AT1G17890 | 132.868 | 100.908 |
| AT5G10550 | 132.829 | 86.5036 |
| AT3G57090 | 132.829 | 53.0448 |
| AT1G64090 | 132.817 | 97.4524 |
| AT1G08800 | 132.777 | 82.5966 |
| AT3G51840 | 132.709 | 58.198 |
| AT1G32400 | 132.682 | 61.9542 |
| AT1G69640 | 132.665 | 58.9873 |
| AT1G01240 | 132.524 | 50.8107 |
| AT5G26740 | 132.408 | 27.153 |
| AT5G49810 | 132.392 | 77.6301 |
| AT2G37040 | 132.303 | 34.8381 |
| AT1G11890 | 132.287 | 69.7836 |
| AT3G17440 | 132.224 | 34.2436 |
| AT1G65220 | 132.136 | 78.6421 |
| AT5G13750 | 132.038 | 140.357 |
| AT5G55070 | 131.941 | 74.6244 |
| AT1G51060 | 131.902 | 89.6222 |
| AT4G15093 | 131.876 | 25.5678 |
| AT5G65140 | 131.562 | 39.1553 |
| AT3G61440 | 131.445 | 157.741 |
| AT3G08530 | 131.329 | 115.434 |
| AT4G37870 | 131.217 | 301.599 |
| AT3G02080 | 131.104 | 287.526 |
| AT2G32380 | 130.904 | 31.276 |
| AT5G62920 | 130.832 | 26.2483 |
| AT4G00570 | 130.685 | 91.4472 |
| AT2G46500 | 130.676 | 75.3009 |
| AT1G01140 | 130.675 | 29.1015 |
| AT1G69810 | 130.618 | 68.7878 |
| AT1G52750 | 130.458 | 14.2276 |
| AT1G15350 | 130.385 | 41.801 |
| AT3G54300 | 130.374 | 58.9712 |
| AT3G11330 | 130.341 | 43.7582 |
| AT2G23070 | 130.054 | 82.7384 |
| AT1G27450 | 129.87 | 125.586 |
| AT1G74000 | 129.751 | 106.903 |
| AT5G25940 | 129.719 | 47.0969 |
| AT5G27700 | 129.32 | 285.645 |
| AT5G35630 | 129.302 | 79.2832 |
| AT5G13420 | 129.288 | 111.681 |
| AT3G19660 | 129.249 | 161.332 |
| AT1G73540 | 129.176 | 80.0599 |
| AT5G49520 | 129.07 | 102.118 |
| AT5G43620 | 128.958 | 18.8136 |
| AT4G00080 | 128.759 | 122.862 |
| AT2G34350 | 128.657 | 42.0444 |
| AT5G17190 | 128.571 | 45.4935 |
| AT4G39670 | 128.564 | 39.7069 |
| AT5G49460 | 128.551 | 124.512 |
| AT5G13070 | 128.529 | 59.8338 |
| AT2G45140 | 128.442 | 97.9863 |
| AT5G40780 | 128.422 | 147.638 |
| AT2G18020 | 128.176 | 249.519 |
| AT4G02080 | 128.174 | 90.6201 |
| AT1G11260 | 128.169 | 133.474 |
| AT2G41250 | 128.12 | 80.9562 |
| AT2G35610 | 128.058 | 33.415 |
| AT2G04410 | 127.962 | 40.9436 |
| AT5G51040 | 127.65 | 85.3096 |
| AT2G30050 | 127.581 | 53.6666 |
| AT4G20780 | 127.502 | 46.141 |
| AT1G32790 | 127.427 | 61.4576 |
| AT5G21090 | 127.346 | 126.07 |
| AT1G13245 | 127.33 | 31.9512 |
| AT1G19394 | 127.329 | 3.47113 |
| AT1G58290 | 127.17 | 54.5355 |
| AT1G04850 | 127.03 | 87.5716 |
| AT3G23180 | 126.77 | 54.6428 |
| AT5G13030 | 126.601 | 61.7215 |
| AT1G04440 | 126.516 | 66.8476 |
| AT1G11650 | 126.5 | 79.8376 |
| AT5G52240 | 126.488 | 116.16 |
| AT5G37770 | 126.377 | 79.3815 |
| AT3G48520 | 126.147 | 35.7016 |
| AT4G09030 | 126.076 | 98.8259 |
| AT1G02000 | 126.03 | 119.667 |
| AT5G47620 | 125.943 | 53.0924 |
| AT5G16840 | 125.841 | 98.7227 |
| AT5G43520 | 125.817 | 38.662 |
| AT1G18740 | 125.755 | 49.6226 |
| AT3G46430 | 125.674 | 59.4686 |
| AT2G38400 | 125.6 | 83.1846 |
| AT1G80440 | 125.421 | 15.0842 |
| AT4G24990 | 125.374 | 79.9002 |
| AT1G01230 | 125.366 | 72.446 |
| AT3G62120 | 125.332 | 113.753 |
| AT2G18193 | 125.312 | 156.007 |
| AT5G57530 | 125.269 | 0.209592 |
| AT5G44065 | 125.232 | 2.05658 |
| AT4G12590 | 125.19 | 108.171 |
| AT3G15020 | 125.129 | 24.775 |
| AT1G69530 | 125.073 | 39.0356 |
| AT2G21045 | 125.05 | 38.1773 |
| AT2G30040 | 125.014 | 22.104 |
| AT3G13430 | 124.937 | 101.391 |
| AT4G12090 | 124.764 | 30.0976 |
| AT3G18740 | 124.524 | 274.163 |
| AT3G54363 | 124.475 | 11.6312 |
| AT3G57410 | 124.342 | 36.5097 |
| AT1G68300 | 124.142 | 43.2172 |
| AT1G47380 | 124.109 | 68.4854 |
| AT3G61190 | 124.091 | 37.2426 |
| AT1G60940 | 123.974 | 74.7029 |
| AT5G56710 | 123.972 | 438.197 |
| AT1G79590 | 123.737 | 47.5749 |
| AT4G19880 | 123.647 | 192.615 |
| AT4G26290 | 123.471 | 4.69016 |
| AT2G47470 | 123.376 | 164.606 |
| AT3G25780 | 123.349 | 198.54 |
| AT5G32440 | 123.319 | 47.5331 |
| AT1G65720 | 123.283 | 107.516 |
| AT3G11320 | 123.131 | 37.5714 |
| AT3G43430 | 123.129 | 25.2947 |
| AT3G08710 | 122.989 | 70.3581 |
| AT5G21274 | 122.757 | 119.244 |
| AT4G36820 | 122.752 | 34.5936 |
| AT3G13320 | 122.732 | 82.2991 |
| AT3G15580 | 122.631 | 77.6704 |
| AT1G60730 | 122.535 | 78.0474 |
| AT5G61228 | 122.494 | 44.2993 |
| AT1G14960 | 122.489 | 8.48455 |
| AT1G14740 | 122.409 | 77.7118 |
| AT3G59540 | 122.329 | 437.689 |
| AT4G23100 | 122.311 | 193.692 |
| AT1G10150 | 122.213 | 53.7906 |
| AT4G28260 | 122.192 | 87.9532 |
| AT4G37010 | 122.131 | 19.1228 |
| AT1G79270 | 122.041 | 18.9737 |
| AT4G15910 | 122.019 | 155.306 |
| AT1G74380 | 121.985 | 54.8819 |
| AT5G18520 | 121.863 | 69.8313 |
| AT1G50640 | 121.806 | 52.18 |
| AT1G27730 | 121.752 | 39.6096 |
| AT2G44310 | 121.684 | 61.1378 |
| AT5G13210 | 121.552 | 34.0587 |
| AT4G12790 | 121.541 | 124.925 |
| AT2G43330 | 121.522 | 35.8465 |
| AT5G37260 | 121.514 | 51.2159 |
| AT3G12480 | 121.436 | 58.5501 |
| AT3G11260 | 121.338 | 60.3963 |
| AT1G30230 | 121.142 | 242.629 |
| AT1G27320 | 121.108 | 69.0781 |
| AT2G19790 | 121.087 | 73.0925 |
| AT5G62390 | 121.04 | 181.27 |
| AT1G10200 | 120.914 | 17.3714 |
| AT4G12650 | 120.875 | 113.334 |
| AT4G35310 | 120.79 | 85.5642 |
| AT1G12090 | 120.708 | 150.706 |
| AT1G30130 | 120.671 | 48.2212 |
| AT4G11260 | 120.658 | 85.0319 |
| AT1G20090 | 120.63 | 31.0274 |
| AT1G06400 | 120.474 | 53.3898 |
| AT5G19070 | 120.401 | 45.1077 |
| AT3G27880 | 120.374 | 170.138 |
| AT2G27720 | 120.372 | 248.44 |
| AT5G05140 | 120.357 | 71.0003 |
| AT4G04840 | 120.257 | 40.4225 |
| AT3G07890 | 119.607 | 44.1154 |
| AT3G51330 | 119.334 | 7.01409 |
| AT3G16800 | 119.3 | 44.0168 |
| AT2G41160 | 119.294 | 64.5898 |
| AT2G26290 | 119.218 | 15.8924 |
| AT1G20970 | 119.209 | 72.4688 |
| AT4G12560 | 119.164 | 90.6437 |
| AT3G16690 | 119.137 | 18.8178 |
| AT1G31130 | 119.031 | 100.965 |
| AT5G63620 | 118.809 | 68.2131 |
| AT5G03080 | 118.749 | 62.7404 |
| AT3G58710 | 118.658 | 34.1444 |
| AT5G51070 | 118.652 | 82.0493 |
| AT1G80460 | 118.637 | 75.2698 |
| AT1G22470 | 118.513 | 34.0065 |
| AT3G49910 | 118.394 | 291.858 |
| AT2G17380 | 118.347 | 74.0224 |
| AT1G60140 | 118.214 | 52.8562 |
| AT3G12740 | 117.997 | 95.9634 |
| AT2G37470 | 117.96 | 162.825 |
| AT5G57560 | 117.776 | 150.476 |
| AT4G01870 | 117.679 | 124.183 |
| AT1G64142 | 117.636 | 78.7714 |
| AT5G19520 | 117.547 | 191.823 |
| AT5G53310 | 117.381 | 78.5098 |
| AT5G12450 | 117.312 | 0.566203 |
| AT1G75630 | 117.246 | 59.8659 |
| AT2G28840 | 117.161 | 81.2378 |
| AT1G50290 | 117.154 | 26.6541 |
| AT3G50260 | 117.099 | 46.2149 |
| AT1G77710 | 117.09 | 89.8361 |
| AT5G05170 | 117.058 | 102.753 |
| AT5G49280 | 117.025 | 67.2605 |
| AT3G51500 | 117.02 | 70.062 |
| AT3G50830 | 116.875 | 25.7024 |
| AT1G19400 | 116.859 | 64.723 |
| AT4G00460 | 116.672 | 7.66071 |
| AT5G67620 | 116.588 | 8.13089 |
| AT5G04250 | 116.575 | 88.9713 |
| AT1G02140 | 116.564 | 109.15 |
| AT2G34480 | 116.552 | 400.313 |
| AT3G01650 | 116.495 | 41.2241 |
| AT5G39590 | 116.486 | 54.1456 |
| AT5G24590 | 116.455 | 59.9115 |
| AT5G13710 | 116.217 | 97.6203 |
| AT1G69490 | 116.082 | 116.696 |
| AT2G21130 | 116.016 | 196.629 |
| AT5G20900 | 115.854 | 64.3572 |
| AT3G01720 | 115.849 | 15.0466 |
| AT1G51070 | 115.714 | 37.3772 |
| AT3G21180 | 115.613 | 3.74594 |
| AT3G59970 | 115.606 | 104.833 |
| AT4G30810 | 115.603 | 73.8428 |
| AT5G15190 | 115.585 | 32.6688 |
| AT5G46410 | 115.391 | 57.4541 |
| AT1G30360 | 115.329 | 23.805 |
| AT3G53020 | 115.27 | 391.953 |
| AT1G68580 | 115.215 | 50.8815 |
| AT3G17420 | 115.141 | 28.969 |
| AT5G45490 | 115.1 | 27.2926 |
| AT5G20500 | 115.094 | 137.228 |
| AT1G03090 | 115.017 | 40.3469 |
| AT1G26550 | 115.016 | 93.1416 |
| AT3G14230 | 114.842 | 59.2535 |
| AT1G04480 | 114.812 | 203.838 |
| AT5G64370 | 114.793 | 40.408 |
| AT2G40340 | 114.79 | 71.086 |
| AT3G11410 | 114.762 | 38.6339 |
| AT3G57550 | 114.726 | 80.672 |
| AT3G08930 | 114.664 | 47.4671 |
| AT5G20650 | 114.657 | 33.1603 |
| AT5G45550 | 114.632 | 74.0268 |
| AT1G69600 | 114.574 | 64.1721 |
| AT1G27300 | 114.566 | 108.932 |
| AT5G19510 | 114.548 | 207.974 |
| AT1G20840 | 114.533 | 28.0419 |
| AT4G15000 | 114.484 | 395.472 |
| AT1G18720 | 114.421 | 73.0624 |
| AT3G25250 | 114.308 | 52.1866 |
| AT4G23730 | 114.307 | 31.1309 |
| AT3G04840 | 114.226 | 446.351 |
| AT5G09810 | 114.14 | 197.984 |
| AT1G16850 | 114.135 | 105.806 |
| AT5G13990 | 114.103 | 7.30302 |
| AT3G57450 | 114.079 | 52.5734 |
| AT2G34470 | 114.03 | 59.0848 |
| AT5G28630 | 113.9 | 31.8575 |
| AT5G18110 | 113.796 | 76.6114 |
| AT3G60300 | 113.674 | 49.1471 |
| AT1G16210 | 113.656 | 66.0451 |
| AT3G44010 | 113.593 | 221.121 |
| AT5G47740 | 113.395 | 39.317 |
| AT1G78570 | 113.24 | 148.111 |
| AT2G31090 | 113.235 | 82.8558 |
| AT2G35930 | 113.232 | 32.1137 |
| AT4G04800 | 113.15 | 42.5369 |
| AT3G19130 | 113.066 | 72.3859 |
| AT1G63460 | 112.971 | 63.5869 |
| AT2G03720 | 112.811 | 0.0211319 |
| AT5G58740 | 112.794 | 61.3209 |
| AT3G53610 | 112.779 | 65.6764 |
| AT1G26850 | 112.68 | 94.83 |
| AT5G03040 | 112.653 | 74.2693 |
| AT4G33430 | 112.45 | 71.0119 |
| AT1G51510 | 112.293 | 77.4921 |
| AT1G10470 | 112.292 | 46.7094 |
| AT1G61890 | 112.258 | 102.258 |
| AT3G07560 | 112.209 | 78.3394 |
| AT2G29430 | 112.114 | 39.1246 |
| AT2G30670 | 112.11 | 0.339629 |
| AT2G27830 | 112.097 | 55.1746 |
| AT5G64430 | 112.044 | 37.22 |
| AT1G55920 | 112.04 | 167.787 |
| AT2G42490 | 111.936 | 31.1836 |
| AT2G20990 | 111.93 | 103.745 |
| AT4G30440 | 111.756 | 41.545 |
| AT3G07568 | 111.724 | 49.503 |
| AT4G18430 | 111.568 | 4.4472 |
| AT1G61560 | 111.56 | 19.044 |
| AT2G27820 | 111.43 | 23.5146 |
| AT2G46860 | 111.429 | 0.593574 |
| AT5G47930 | 111.366 | 365.982 |
| AT1G52690 | 111.235 | 302.545 |
| AT1G67310 | 111.217 | 54.1888 |
| AT4G25230 | 111.175 | 60.8486 |
| AT1G70600 | 110.991 | 307.179 |
| AT4G04910 | 110.93 | 68.4002 |
| AT5G18380 | 110.898 | 214.74 |
| AT1G26270 | 110.887 | 73.838 |
| AT5G11090 | 110.826 | 27.648 |
| AT3G28740 | 110.768 | 192.273 |
| AT1G71780 | 110.731 | 29.9951 |
| AT5G43940 | 110.665 | 108.69 |
| AT5G45500 | 110.658 | 15.6216 |
| AT1G79160 | 110.65 | 31.3521 |
| AT3G53430 | 110.571 | 211.962 |
| AT4G30780 | 110.568 | 71.8368 |
| AT1G03590 | 110.554 | 69.3303 |
| AT2G18450 | 110.514 | 9.11401 |
| AT1G29150 | 110.512 | 92.1168 |
| AT4G20890 | 110.493 | 110.735 |
| AT3G44330 | 110.487 | 83.1502 |
| AT2G18960 | 110.458 | 120.675 |
| AT5G60950 | 110.4 | 111.256 |
| AT5G15350 | 110.398 | 98.6467 |
| AT2G41800 | 110.379 | 273.359 |
| AT3G25520 | 110.351 | 450.782 |
| AT5G44070 | 110.321 | 117.325 |
| AT1G62850 | 110.321 | 14.2257 |
| AT2G22760 | 110.257 | 47.5466 |
| AT4G25900 | 110.202 | 117.659 |
| AT2G38740 | 110.145 | 35.7121 |
| AT4G12420 | 110.048 | 87.6236 |
| AT1G21900 | 110.015 | 53.9116 |
| AT3G11900 | 109.997 | 26.1879 |
| AT1G80040 | 109.975 | 85.1081 |
| AT4G21660 | 109.964 | 84.2908 |
| AT1G36732 | 109.944 | 82.1627 |
| AT5G02450 | 109.926 | 348.769 |
| AT4G02480 | 109.897 | 89.1852 |
| AT1G10940 | 109.869 | 53.0696 |
| AT3G29180 | 109.86 | 64.7265 |
| AT4G19680 | 109.85 | 3.84865 |
| AT3G49550 | 109.765 | 82.9727 |
| AT2G37478 | 109.731 | 64.752 |
| AT3G49790 | 109.677 | 70.7676 |
| AT1G14220 | 109.629 | 30.3451 |
| AT5G54870 | 109.523 | 89.0878 |
| AT1G07030 | 109.426 | 34.1605 |
| AT1G69260 | 109.405 | 45.8526 |
| AT3G03520 | 109.385 | 16.6552 |
| AT5G49890 | 109.347 | 24.0093 |
| AT5G14240 | 109.318 | 64.5788 |
| AT2G17290 | 109.288 | 69.1727 |
| AT5G57900 | 109.213 | 64.5471 |
| AT1G09920 | 109.208 | 65.4135 |
| AT1G34370 | 109.135 | 85.6114 |
| AT3G29035 | 109.127 | 30.6844 |
| AT1G15930 | 109.082 | 240.561 |
| AT5G66280 | 109.064 | 27.3395 |
| AT3G15430 | 109.051 | 40.1306 |
| AT1G19770 | 109.031 | 68.9864 |
| AT4G01100 | 109.001 | 104.654 |
| AT5G66400 | 108.849 | 38.0731 |
| AT4G35480 | 108.772 | 40.5335 |
| AT4G33865 | 108.764 | 313.296 |
| AT5G38650 | 108.563 | 90.7758 |
| AT5G54080 | 108.499 | 65.1643 |
| AT5G24140 | 108.444 | 9.65024 |
| AT3G52560 | 108.435 | 68.9553 |
| AT1G72370 | 108.382 | 317.522 |
| AT2G24765 | 108.217 | 69.6968 |
| AT5G54680 | 108.215 | 56.2449 |
| AT4G24620 | 108.188 | 58.2648 |
| AT1G79990 | 108.15 | 122.137 |
| AT1G07350 | 108.125 | 50.8134 |
| AT1G13060 | 108.115 | 109.314 |
| AT1G74030 | 108.072 | 64.2074 |
| AT1G04750 | 107.974 | 91.7128 |
| AT5G59950 | 107.911 | 60.0687 |
| AT3G11700 | 107.844 | 37.5015 |
| AT3G60550 | 107.833 | 14.0374 |
| AT1G51160 | 107.826 | 76.1884 |
| AT3G61070 | 107.811 | 60.9125 |
| AT2G40080 | 107.772 | 56.3609 |
| AT3G12600 | 107.766 | 67.1606 |
| AT4G02370 | 107.765 | 38.4737 |
| AT1G11400 | 107.741 | 50.0477 |
| AT2G30250 | 107.69 | 47.772 |
| AT3G22600 | 107.674 | 85.5993 |
| AT5G67520 | 107.667 | 14.9742 |
| AT5G46630 | 107.647 | 78.13 |
| AT5G48657 | 107.606 | 16.8773 |
| AT4G12080 | 107.577 | 32.389 |
| AT3G57280 | 107.573 | 61.4337 |
| AT3G09820 | 107.476 | 189.064 |
| AT2G35980 | 107.34 | 37.8361 |
| AT1G13950 | 107.303 | 12.4879 |
| AT1G59860 | 107.292 | 101.285 |
| AT5G40650 | 107.139 | 60.6266 |
| AT1G76690 | 107.101 | 98.3691 |
| AT3G28940 | 107.087 | 105.654 |
| AT5G07080 | 106.986 | 2.56867 |
| AT1G55680 | 106.977 | 61.2214 |
| AT3G53890 | 106.962 | 274.759 |
| AT1G14980 | 106.824 | 148.057 |
| AT1G63940 | 106.815 | 62.1853 |
| AT1G72150 | 106.601 | 48.4544 |
| AT3G24100 | 106.541 | 29.181 |
| AT3G10800 | 106.522 | 58.3709 |
| AT4G17490 | 106.475 | 105.438 |
| AT5G22410 | 106.322 | 0.292415 |
| AT2G43290 | 106.298 | 77.7134 |
| AT4G13930 | 106.245 | 113.185 |
| AT1G72500 | 106.123 | 67.5116 |
| AT3G02800 | 106.093 | 88.0476 |
| AT1G08340 | 106.051 | 18.6353 |
| AT4G13440 | 106.024 | 3.03328 |
| AT2G18280 | 106.004 | 68.9908 |
| AT5G10780 | 105.974 | 80.649 |
| AT5G14730 | 105.952 | 58.6944 |
| AT3G04400 | 105.944 | 347.941 |
| AT3G04240 | 105.859 | 71.5815 |
| AT4G38900 | 105.813 | 38.6376 |
| AT5G53588 | 105.767 | 88.7414 |
| AT2G24180 | 105.709 | 54.9324 |
| AT3G54020 | 105.698 | 29.158 |
| AT3G51450 | 105.622 | 95.291 |
| AT3G11730 | 105.535 | 68.5797 |
| AT5G61790 | 105.509 | 97.2112 |
| AT5G11500 | 105.468 | 63.3898 |
| AT4G25390 | 105.451 | 65.4348 |
| AT4G24520 | 105.433 | 66.9553 |
| AT2G29530 | 105.375 | 127.423 |
| AT5G02270 | 105.327 | 53.4881 |
| AT5G62280 | 105.288 | 3.58599 |
| AT3G01640 | 105.269 | 51.9187 |
| AT3G06780 | 105.24 | 38.3617 |
| AT2G47610 | 105.215 | 366.362 |
| AT5G19590 | 105.21 | 49.8041 |
| AT1G13260 | 105.171 | 33.873 |
| AT3G11940 | 105.121 | 306.907 |
| AT1G54030 | 105.033 | 93.298 |
| AT5G06800 | 104.978 | 4.74244 |
| AT5G62300 | 104.965 | 267.169 |
| AT1G04630 | 104.963 | 55.3674 |
| AT1G13880 | 104.947 | 61.4481 |
| AT4G35450 | 104.937 | 79.9482 |
| AT1G54830 | 104.9 | 61.1927 |
| AT1G22190 | 104.875 | 84.0283 |
| AT3G12050 | 104.714 | 91.3597 |
| AT1G18470 | 104.712 | 48.4471 |
| AT1G04270 | 104.621 | 307.284 |
| AT1G55840 | 104.54 | 136.636 |
| AT1G16670 | 104.533 | 41.418 |
| AT3G12490 | 104.466 | 167.8 |
| AT4G35110 | 104.436 | 92.6731 |
| AT4G25130 | 104.405 | 47.6701 |
| AT4G14615 | 104.359 | 64.3946 |
| AT4G08850 | 104.317 | 57.8279 |
| AT1G13420 | 104.237 | 56.4079 |
| AT5G54540 | 104.232 | 51.3161 |
| AT1G13590 | 104.211 | 212.79 |
| AT5G35700 | 104.208 | 22.0692 |
| AT1G32210 | 104.16 | 117.52 |
| AT1G07610 | 104.157 | 28.0747 |
| AT3G29250 | 104.045 | 20.6767 |
| AT1G23290 | 104.011 | 279.255 |
| AT5G21170 | 104.002 | 18.1796 |
| AT1G68000 | 103.924 | 60.081 |
| AT3G12400 | 103.913 | 44.8979 |
| AT1G67920 | 103.836 | 60.0242 |
| AT5G59550 | 103.794 | 32.5617 |
| AT1G29970 | 103.763 | 45.4359 |
| AT3G19970 | 103.689 | 46.3211 |
| AT5G42890 | 103.67 | 89.5492 |
| AT3G56340 | 103.597 | 233.459 |
| AT1G36980 | 103.52 | 45.8485 |
| AT3G24830 | 103.392 | 350.856 |
| AT3G62790 | 103.362 | 79.0221 |
| AT4G15160 | 103.353 | 430.357 |
| AT2G26670 | 103.31 | 95.1228 |
| AT5G67250 | 103.304 | 66.745 |
| AT1G26670 | 103.273 | 52.4596 |
| AT5G62500 | 103.249 | 59.705 |
| AT1G75388 | 103.249 | 53.1455 |
| AT1G29025 | 103.229 | 90.9869 |
| AT1G05100 | 103.106 | 62.5911 |
| AT5G19025 | 103.039 | 21.3631 |
| AT2G03120 | 102.995 | 71.0398 |
| AT2G03730 | 102.95 | 30.3333 |
| AT5G42580 | 102.872 | 229.455 |
| AT5G61500 | 102.855 | 68.9785 |
| AT2G30550 | 102.566 | 55.7598 |
| AT4G38410 | 102.548 | 219.627 |
| AT5G54300 | 102.494 | 36.2608 |
| AT1G77330 | 102.362 | 226.015 |
| AT5G13810 | 102.324 | 38.9278 |
| AT5G42790 | 102.265 | 82.3458 |
| AT2G38700 | 102.149 | 112.057 |
| AT3G15810 | 102.074 | 120.442 |
| AT1G79075 | 102.06 | 123.288 |
| AT3G17770 | 102.042 | 63.7984 |
| AT5G24310 | 101.958 | 3.18457 |
| AT5G55940 | 101.93 | 71.7568 |
| AT3G62870 | 101.923 | 340.959 |
| AT2G18110 | 101.902 | 111.325 |
| AT5G54510 | 101.655 | 14.5543 |
| AT4G19110 | 101.509 | 69.3419 |
| AT2G02390 | 101.463 | 85.891 |
| AT3G06860 | 101.426 | 64.9006 |
| AT3G02910 | 101.403 | 39.2968 |
| AT5G63130 | 101.391 | 27.9117 |
| AT5G64140 | 101.388 | 286.412 |
| AT5G02170 | 101.152 | 45.575 |
| AT5G18860 | 101.121 | 27.2386 |
| AT2G45890 | 101.066 | 0.368687 |
| AT1G74340 | 101.027 | 79.0139 |
| AT4G23010 | 101.013 | 65.623 |
| AT5G52060 | 101.007 | 44.8949 |
| AT3G54100 | 100.861 | 49.773 |
| AT4G23050 | 100.797 | 75.2925 |
| AT4G37790 | 100.781 | 20.199 |
| AT2G01250 | 100.764 | 384.206 |
| AT1G52300 | 100.59 | 225.16 |
| AT4G31320 | 100.545 | 149.476 |
| AT4G28240 | 100.491 | 56.9618 |
| AT4G34000 | 100.457 | 44.5271 |
| AT3G12540 | 100.301 | 0.090463 |
| AT3G02520 | 100.295 | 117.207 |
| AT4G14990 | 100.286 | 49.2291 |
| AT4G39200 | 100.183 | 238.594 |
| AT5G25265 | 100.183 | 34.9042 |
| AT2G03690 | 100.179 | 43.6369 |
| AT4G36960 | 100.141 | 60.7024 |
| AT1G64750 | 100.048 | 154.004 |
| AT5G17770 | 99.9405 | 90.9525 |
| AT1G52740 | 99.9386 | 107.454 |
| AT1G57680 | 99.9128 | 37.2119 |
| AT1G72770 | 99.8724 | 36.9211 |
| AT5G19000 | 99.7462 | 42.9959 |
| AT2G30410 | 99.6529 | 41.9471 |
| AT2G41010 | 99.5977 | 42.8458 |
| AT2G18040 | 99.5891 | 116.787 |
| AT5G19400 | 99.5581 | 48.3784 |
| AT4G02520 | 99.5182 | 38.632 |
| AT1G12760 | 99.4697 | 55.1525 |
| AT4G32870 | 99.4631 | 48.3356 |
| AT3G04090 | 99.3784 | 65.1762 |
| AT5G17060 | 99.3639 | 39.8485 |
| AT1G07590 | 99.3453 | 104.225 |
| AT5G20885 | 99.3007 | 18.4252 |
| AT2G35510 | 99.2482 | 78.8332 |
| AT4G38640 | 99.0455 | 52.5628 |
| AT1G28380 | 98.861 | 79.406 |
| AT1G03370 | 98.8063 | 44.3384 |
| AT1G74450 | 98.7871 | 32.1647 |
| AT1G17550 | 98.7538 | 70.9754 |
| AT2G47700 | 98.7352 | 29.0208 |
| AT4G18730 | 98.6908 | 238.892 |
| AT5G46860 | 98.6521 | 43.7071 |
| AT1G21790 | 98.629 | 50.198 |
| AT1G77770 | 98.5733 | 50.1275 |
| AT5G40930 | 98.5513 | 61.9352 |
| AT3G07790 | 98.487 | 53.3919 |
| AT4G22010 | 98.4865 | 70.5117 |
| AT3G05570 | 98.4789 | 40.7456 |
| AT2G17560 | 98.3963 | 198.112 |
| AT5G09390 | 98.3937 | 63.9359 |
| AT1G19310 | 98.3126 | 122.803 |
| AT1G70790 | 98.2008 | 60.4646 |
| AT5G38700 | 98.1841 | 26.2368 |
| AT5G59140 | 98.1601 | 64.5573 |
| AT4G31470 | 98.0658 | 27.4756 |
| AT1G79230 | 98.0262 | 46.9804 |
| AT3G58750 | 97.984 | 58.0657 |
| AT1G67330 | 97.9835 | 10.0397 |
| AT5G02960 | 97.9541 | 447.113 |
| AT5G38470 | 97.9539 | 82.991 |
| AT3G11200 | 97.8476 | 69.9505 |
| AT3G14720 | 97.8352 | 69.5121 |
| AT5G60530 | 97.8294 | 309.051 |
| AT1G13210 | 97.8112 | 55.8033 |
| AT1G11670 | 97.7685 | 78.4023 |
| AT5G54310 | 97.7109 | 47.4574 |
| AT5G08680 | 97.6736 | 43.3602 |
| AT5G24890 | 97.6031 | 53.2265 |
| AT2G39530 | 97.5468 | 34.0835 |
| AT3G61640 | 97.54 | 90.0733 |
| AT3G58040 | 97.3995 | 48.6086 |
| AT1G08940 | 97.368 | 38.0252 |
| AT4G33120 | 97.3602 | 56.5154 |
| AT2G21520 | 97.2493 | 67.3533 |
| AT1G62790 | 97.2476 | 57.4401 |
| AT4G19210 | 97.1689 | 91.1939 |
| AT5G60800 | 97.0321 | 13.6142 |
| AT3G46010 | 96.9475 | 57.6002 |
| AT5G27930 | 96.7972 | 15.0631 |
| AT5G67190 | 96.7601 | 25.8656 |
| AT1G17060 | 96.6858 | 104.483 |
| AT5G51570 | 96.5853 | 35.2336 |
| AT1G01360 | 96.5763 | 57.2617 |
| AT5G05440 | 96.5699 | 27.9988 |
| AT1G07360 | 96.5123 | 46.6408 |
| AT2G29750 | 96.4608 | 4.05632 |
| AT3G11120 | 96.4318 | 392.828 |
| AT4G15120 | 96.4306 | 43.4572 |
| AT2G24500 | 96.2565 | 102.739 |
| AT1G16858 | 96.0083 | 55.8139 |
| AT2G36160 | 95.9392 | 317.692 |
| AT5G55400 | 95.8395 | 31.0082 |
| AT3G52850 | 95.7934 | 122.913 |
| AT4G31450 | 95.7907 | 12.5648 |
| AT1G69410 | 95.7717 | 97.9096 |
| AT3G59940 | 95.7151 | 36.8476 |
| AT5G35180 | 95.7102 | 26.293 |
| AT5G36290 | 95.7061 | 91.0854 |
| AT5G42930 | 95.6437 | 35.6642 |
| AT4G19030 | 95.6057 | 139.122 |
| AT5G36230 | 95.6053 | 119.665 |
| AT1G37130 | 95.4617 | 89.8568 |
| AT3G60750 | 95.4569 | 82.5238 |
| AT5G42000 | 95.4137 | 59.1965 |
| AT2G02800 | 95.2993 | 63.2671 |
| AT4G39890 | 95.2884 | 27.5567 |
| AT4G36690 | 95.2413 | 70.159 |
| AT3G51130 | 95.2327 | 48.1163 |
| AT1G12110 | 95.1444 | 20.3747 |
| AT1G09640 | 95.1423 | 117.996 |
| AT5G03540 | 95.0941 | 38.6426 |
| AT2G16720 | 95.0576 | 23.2494 |
| AT3G59760 | 95.0248 | 73.1293 |
| AT3G11580 | 94.9186 | 40.8246 |
| AT5G11790 | 94.9003 | 32.2797 |
| AT4G37120 | 94.8426 | 74.6438 |
| AT3G62980 | 94.83 | 75.1054 |
| AT2G34600 | 94.8236 | 58.2897 |
| AT4G30240 | 94.8102 | 47.3666 |
| AT1G63500 | 94.7283 | 54.4449 |
| AT4G30160 | 94.7152 | 26.0059 |
| AT2G29960 | 94.6899 | 106.835 |
| AT2G38410 | 94.686 | 35.4149 |
| AT1G07150 | 94.6702 | 46.8284 |
| AT2G20562 | 94.6584 | 44.9998 |
| AT5G08160 | 94.4509 | 48.5771 |
| AT4G31990 | 94.4378 | 75.7083 |
| AT4G33090 | 94.3097 | 67.7462 |
| AT3G56490 | 94.2974 | 73.3822 |
| AT3G48930 | 94.2605 | 282.545 |
| AT5G43850 | 94.2377 | 46.8603 |
| AT1G78280 | 94.1896 | 46.3048 |
| AT1G14000 | 94.1617 | 105.404 |
| AT3G61890 | 94.0996 | 100.061 |
| AT5G57655 | 94.0655 | 59.9235 |
| AT5G63680 | 94.0386 | 108.626 |
| AT1G75380 | 94.0376 | 28.071 |
| AT4G31700 | 93.9881 | 285.444 |
| AT1G08190 | 93.7789 | 31.9353 |
| AT1G60780 | 93.7483 | 55.7992 |
| AT1G21660 | 93.6772 | 34.708 |
| AT2G36895 | 93.6666 | 34.3901 |
| AT5G19250 | 93.4861 | 21.5756 |
| AT5G11960 | 93.4594 | 40.3331 |
| AT5G44110 | 93.4266 | 68.6418 |
| AT1G25490 | 93.3796 | 77.0225 |
| AT3G08650 | 93.353 | 28.3398 |
| AT3G25040 | 93.2794 | 58.2393 |
| AT4G15802 | 93.2694 | 110.167 |
| AT5G15640 | 93.207 | 39.3618 |
| AT2G15830 | 93.1343 | 233.621 |
| AT5G21020 | 93.082 | 62.0359 |
| AT3G10815 | 92.9718 | 70.6735 |
| AT3G25910 | 92.9463 | 74.4104 |
| AT1G48600 | 92.9363 | 51.886 |
| AT4G26470 | 92.9078 | 29.7429 |
| AT3G48680 | 92.8943 | 34.4114 |
| AT1G02400 | 92.8745 | 13.5141 |
| AT1G78600 | 92.8573 | 28.608 |
| AT5G04830 | 92.7697 | 89.5831 |
| AT5G40690 | 92.644 | 30.8061 |
| AT3G22380 | 92.5974 | 50.5469 |
| AT2G23310 | 92.594 | 48.2938 |
| AT3G53730 | 92.559 | 97.1652 |
| AT4G40030 | 92.4626 | 66.836 |
| AT4G31300 | 92.4492 | 73.8495 |
| AT1G70330 | 92.4346 | 25.5205 |
| AT5G24290 | 92.394 | 30.3502 |
| AT4G39030 | 92.2074 | 36.3607 |
| AT1G05205 | 92.1847 | 35.2143 |
| AT2G21660 | 92.1486 | 314.974 |
| AT4G10810 | 92.0803 | 33.7553 |
| AT5G10410 | 91.9798 | 6.41601 |
| AT5G63195 | 91.9621 | 44.8024 |
| AT3G56200 | 91.9618 | 74.3758 |
| AT2G46510 | 91.9514 | 90.6194 |
| AT1G01100 | 91.919 | 165.426 |
| AT5G52020 | 91.8093 | 10.1282 |
| AT4G14560 | 91.7057 | 75.008 |
| AT1G78050 | 91.6902 | 41.0381 |
| AT1G15340 | 91.6614 | 59.473 |
| AT1G48830 | 91.6377 | 106.127 |
| AT5G04040 | 91.6138 | 51.6901 |
| AT1G15130 | 91.5767 | 34.8092 |
| AT4G14420 | 91.4806 | 97.6899 |
| AT2G30740 | 91.3616 | 46.266 |
| AT1G53750 | 91.2869 | 97.9938 |
| AT3G11020 | 91.2854 | 28.8944 |
| AT5G21280 | 91.1919 | 19.7542 |
| AT4G25380 | 91.1715 | 56.1042 |
| AT2G27580 | 91.168 | 43.5613 |
| AT2G25210 | 91.0872 | 374.855 |
| AT2G38500 | 91.0314 | 1.52791 |
| AT5G52560 | 90.9236 | 54.8585 |
| AT3G04630 | 90.8727 | 14.4064 |
| AT5G42510 | 90.8371 | 0.370902 |
| AT1G62440 | 90.8058 | 17.1865 |
| AT5G03520 | 90.7762 | 52.3817 |
| AT1G31340 | 90.652 | 57.604 |
| AT3G43230 | 90.6426 | 52.696 |
| AT5G64270 | 90.5728 | 47.5624 |
| AT1G56450 | 90.5519 | 137.202 |
| AT1G44090 | 90.5283 | 12.5487 |
| AT3G15370 | 90.4573 | 60.7785 |
| AT3G54960 | 90.3296 | 52.4375 |
| AT3G22370 | 90.2186 | 59.0533 |
| AT3G52120 | 90.1797 | 48.1562 |
| AT4G08230 | 90.1689 | 78.0728 |
| AT3G08730 | 90.1387 | 87.1442 |
| AT4G27020 | 90.1242 | 46.3619 |
| AT5G58380 | 90.1232 | 38.2209 |
| AT3G05170 | 90.1163 | 9.58352 |
| AT5G37480 | 90.0775 | 41.4186 |
| AT2G27500 | 90.0572 | 52.1074 |
| AT4G39220 | 89.9968 | 60.104 |
| AT1G02890 | 89.9526 | 60.2902 |
| AT1G22360 | 89.927 | 104.612 |
| AT2G46240 | 89.8832 | 110.483 |
| AT4G17940 | 89.8776 | 214.145 |
| AT3G46620 | 89.8614 | 49.6166 |
| AT5G64300 | 89.8592 | 57.9953 |
| AT4G30320 | 89.776 | 0.843779 |
| AT5G09440 | 89.7397 | 4.259 |
| AT1G22780 | 89.6342 | 316.587 |
| AT4G33150 | 89.4918 | 86.081 |
| AT5G57870 | 89.4788 | 56.892 |
| AT3G62720 | 89.3846 | 35.0464 |
| AT3G11820 | 89.3844 | 55.6 |
| AT1G16560 | 89.3749 | 38.5719 |
| AT2G25250 | 89.3514 | 48.9822 |
| AT3G54140 | 89.3348 | 120.7 |
| AT2G31810 | 89.3123 | 47.6745 |
| AT4G36640 | 89.3035 | 107.169 |
| AT3G13670 | 89.2792 | 54.1267 |
| AT3G16910 | 89.2772 | 23.8106 |
| AT2G38790 | 89.2405 | 5.07359 |
| AT3G54840 | 89.2247 | 64.2252 |
| AT1G23260 | 89.1927 | 54.9332 |
| AT5G13430 | 89.1908 | 35.06 |
| AT3G20370 | 89.0895 | 69.1095 |
| AT3G23090 | 89.0892 | 17.3789 |
| AT5G35460 | 89.058 | 21.2174 |
| AT1G51710 | 88.9278 | 81.2703 |
| AT5G65930 | 88.8466 | 14.3017 |
| AT4G10140 | 88.7803 | 40.7406 |
| AT2G20960 | 88.4976 | 38.6291 |
| AT4G30650 | 88.4964 | 102.378 |
| AT2G21600 | 88.4545 | 63.3446 |
| AT5G13440 | 88.4102 | 40.4281 |
| AT1G69450 | 88.4043 | 86.5024 |
| AT5G23840 | 88.3959 | 137.178 |
| AT5G54640 | 88.3601 | 75.5752 |
| AT2G18440 | 88.3522 | 97.3304 |
| AT2G46255 | 88.3381 | 11.315 |
| AT3G21630 | 88.2421 | 38.4498 |
| AT1G61740 | 88.217 | 27.1569 |
| AT1G24050 | 88.1986 | 80.4212 |
| AT1G70460 | 88.1011 | 0.641957 |
| AT1G20460 | 88.0849 | 39.5722 |
| AT4G32551 | 88.0707 | 71.709 |
| AT3G29075 | 88.0526 | 44.27 |
| AT2G23450 | 87.9509 | 27.5119 |
| AT5G45750 | 87.9464 | 54.954 |
| AT1G24170 | 87.9336 | 28.2139 |
| AT4G36750 | 87.8109 | 31.7811 |
| AT4G20380 | 87.7684 | 45.2759 |
| AT1G19200 | 87.7559 | 15.4307 |
| AT1G05960 | 87.7408 | 49.7252 |
| AT4G08170 | 87.7254 | 63.041 |
| AT5G26030 | 87.702 | 48.5767 |
| AT4G38790 | 87.6774 | 44.563 |
| AT3G18290 | 87.6759 | 42.1226 |
| AT1G53050 | 87.6062 | 54.5236 |
| AT2G27020 | 87.4972 | 107.552 |
| AT4G21560 | 87.4757 | 53.1243 |
| AT3G14560 | 87.4144 | 40.957 |
| AT5G52580 | 87.3573 | 55.155 |
| AT5G07090 | 87.3458 | 278.028 |
| AT5G20165 | 87.2986 | 64.6371 |
| AT3G16440 | 87.2407 | 245.075 |
| AT3G23390 | 87.2029 | 331.019 |
| AT5G17280 | 87.1239 | 30.7571 |
| AT4G28540 | 87.041 | 41.6859 |
| AT2G25430 | 87.0358 | 40.883 |
| AT4G07960 | 87.0289 | 3.41639 |
| AT2G15620 | 87.0021 | 39.4568 |
| AT3G29410 | 86.9658 | 23.6998 |
| AT4G33910 | 86.8344 | 79.0136 |
| AT4G31130 | 86.8175 | 50.4096 |
| AT4G14320 | 86.7677 | 255.661 |
| AT3G13845 | 86.756 | 41.274 |
| AT1G15580 | 86.7121 | 10.1769 |
| AT3G10410 | 86.654 | 64.3661 |
| AT5G48560 | 86.6315 | 67.746 |
| AT1G75780 | 86.6214 | 108.063 |
| AT5G09890 | 86.554 | 55.7231 |
| AT4G29040 | 86.4986 | 99.7801 |
| AT1G61790 | 86.4725 | 88.1075 |
| AT5G10360 | 86.437 | 226.543 |
| AT5G51260 | 86.4245 | 50.2569 |
| AT5G65580 | 86.3905 | 31.7821 |
| AT1G27530 | 86.3831 | 40.6978 |
| AT2G44610 | 86.3726 | 60.877 |
| AT1G17280 | 86.3153 | 56.4968 |
| AT2G04780 | 86.2902 | 46.708 |
| AT1G69050 | 86.2487 | 43.6494 |
| AT3G26020 | 86.2224 | 44.2297 |
| AT1G07985 | 86.1179 | 124.273 |
| AT1G65960 | 86.1003 | 52.0348 |
| AT4G20840 | 86.0855 | 18.5794 |
| AT2G14750 | 86.0834 | 29.3909 |
| AT4G19160 | 86.0259 | 40.6071 |
| AT1G52910 | 86.0247 | 78.2964 |
| AT4G24400 | 86.0133 | 37.3208 |
| AT5G64510 | 86.0094 | 14.7539 |
| AT1G19000 | 86.0069 | 70.8197 |
| AT2G46490 | 85.9957 | 52.3502 |
| AT4G18640 | 85.9666 | 1.95314 |
| AT1G09330 | 85.9114 | 50.174 |
| AT4G03320 | 85.9046 | 46.1206 |
| AT3G51660 | 85.9043 | 76.0523 |
| AT1G09210 | 85.849 | 165.04 |
| AT1G28280 | 85.7963 | 35.8002 |
| AT3G07270 | 85.7799 | 57.0373 |
| AT3G63380 | 85.7103 | 36.6285 |
| AT3G12320 | 85.6195 | 66.0238 |
| AT1G75840 | 85.6157 | 13.6596 |
| AT5G43320 | 85.6062 | 35.9573 |
| AT5G62020 | 85.586 | 27.5297 |
| AT5G51400 | 85.5675 | 62.2244 |
| AT5G46230 | 85.5275 | 24.2494 |
| AT5G04870 | 85.4747 | 50.9259 |
| AT5G42080 | 85.4163 | 72.8736 |
| AT2G27530 | 85.4112 | 398.682 |
| AT5G52650 | 85.3214 | 179.917 |
| AT5G51830 | 85.311 | 143.105 |
| AT3G04670 | 85.311 | 67.215 |
| AT2G31880 | 85.2947 | 56.1649 |
| AT3G50060 | 85.2814 | 28.4441 |
| AT5G01830 | 85.2762 | 13.036 |
| AT5G11950 | 85.2588 | 22.6206 |
| AT3G25882 | 85.1208 | 20.4362 |
| AT5G18270 | 85.1067 | 28.603 |
| AT2G38800 | 85.0766 | 28.4919 |
| AT5G37070 | 85.0324 | 68.0846 |
| AT3G20310 | 84.9658 | 21.787 |
| AT3G62810 | 84.8685 | 41.1591 |
| AT2G43640 | 84.8327 | 66.5215 |
| AT2G39518 | 84.8301 | 14.1797 |
| AT5G09900 | 84.8127 | 102.83 |
| AT5G47180 | 84.7183 | 39.8722 |
| AT5G04930 | 84.7063 | 42.5876 |
| AT1G69740 | 84.7052 | 42.1524 |
| AT5G42590 | 84.6663 | 97.7507 |
| AT1G16240 | 84.6625 | 53.0705 |
| AT1G69295 | 84.6253 | 40.026 |
| AT2G21880 | 84.6027 | 3.98521 |
| AT3G15070 | 84.5994 | 38.5197 |
| AT5G20570 | 84.5602 | 52.3204 |
| AT3G60820 | 84.4293 | 93.6913 |
| AT1G75400 | 84.4087 | 51.4295 |
| AT2G42500 | 84.3982 | 74.2168 |
| AT3G07565 | 84.3965 | 51.2366 |
| AT5G19050 | 84.3927 | 40.6916 |
| AT5G54730 | 84.3504 | 41.039 |
| AT4G18280 | 84.3423 | 182.133 |
| AT4G18170 | 84.3395 | 45.4145 |
| AT3G25220 | 84.339 | 114.979 |
| AT1G11660 | 84.1365 | 24.9657 |
| AT3G19910 | 84.0853 | 58.9538 |
| AT1G53500 | 84.0048 | 57.5814 |
| AT5G23540 | 83.9808 | 80.2444 |
| AT1G21010 | 83.9291 | 27.1343 |
| AT3G63460 | 83.9004 | 56.4612 |
| AT3G07350 | 83.8947 | 29.6186 |
| AT1G71530 | 83.882 | 9.47798 |
| AT3G13500 | 83.8114 | 1.71324 |
| AT1G72880 | 83.742 | 21.7963 |
| AT3G21340 | 83.7404 | 1.26439 |
| AT2G44490 | 83.5631 | 56.5199 |
| AT4G18140 | 83.5229 | 38.5121 |
| AT3G11930 | 83.4995 | 55.1481 |
| AT1G15860 | 83.4432 | 64.9738 |
| AT3G60260 | 83.4407 | 31.885 |
| AT4G18160 | 83.4012 | 44.2846 |
| AT1G02170 | 83.3753 | 31.0515 |
| AT5G35620 | 83.3657 | 95.861 |
| AT5G57290 | 83.3011 | 104.256 |
| AT4G21160 | 83.2264 | 28.8137 |
| AT5G66580 | 83.2222 | 13.6914 |
| AT4G20870 | 83.2207 | 45.6627 |
| AT1G57720 | 83.2165 | 188.35 |
| AT1G67070 | 83.0979 | 26.3534 |
| AT2G34770 | 83.0511 | 61.2234 |
| AT2G39460 | 83.0108 | 408.604 |
| AT1G44830 | 82.9964 | 69.7289 |
| AT5G24810 | 82.9763 | 34.8598 |
| AT1G61100 | 82.9566 | 37.8302 |
| AT5G23830 | 82.9299 | 128.804 |
| AT3G11510 | 82.9163 | 291.149 |
| AT1G01120 | 82.8902 | 10.6765 |
| AT1G58200 | 82.8888 | 40.0591 |
| AT1G54270 | 82.8775 | 67.8055 |
| AT4G11380 | 82.8032 | 51.297 |
| AT2G43320 | 82.8001 | 44.1787 |
| AT3G19950 | 82.7952 | 33.9288 |
| AT1G16170 | 82.7914 | 16.9436 |
| AT1G20310 | 82.778 | 17.9261 |
| AT2G20370 | 82.7673 | 56.9698 |
| AT1G32170 | 82.7574 | 17.8588 |
| AT1G76790 | 82.7342 | 134.532 |
| AT2G40095 | 82.705 | 98.5746 |
| AT2G26660 | 82.6735 | 52.6921 |
| AT5G42090 | 82.5743 | 51.7587 |
| AT5G63600 | 82.4453 | 2.92904 |
| AT2G33310 | 82.2792 | 8.13975 |
| AT4G18380 | 82.2139 | 44.0537 |
| AT1G56340 | 82.1498 | 106.134 |
| AT5G05780 | 82.1105 | 77.7532 |
| AT2G22500 | 82.0692 | 37.3739 |
| AT1G50460 | 82.0532 | 50.787 |
| AT3G24503 | 82.0023 | 74.2024 |
| AT2G01570 | 81.9937 | 34.1963 |
| AT2G47600 | 81.9693 | 46.4931 |
| AT1G12550 | 81.9584 | 1.03544 |
| AT3G05420 | 81.9277 | 38.8215 |
| AT2G32260 | 81.8792 | 30.137 |
| AT3G17210 | 81.8648 | 70.3528 |
| AT5G57123 | 81.8321 | 48.5171 |
| AT3G14205 | 81.8254 | 54.6984 |
| AT1G18070 | 81.8219 | 78.8101 |
| AT4G30560 | 81.8161 | 1.17742 |
| AT4G17340 | 81.8083 | 4.46908 |
| AT1G50670 | 81.7715 | 47.8486 |
| AT5G58060 | 81.7199 | 47.9754 |
| AT1G05620 | 81.7066 | 67.61 |
| AT4G09320 | 81.6957 | 257.591 |
| AT2G31610 | 81.5254 | 233.666 |
| AT5G54860 | 81.4927 | 45.1399 |
| AT4G16720 | 81.481 | 235.805 |
| AT1G29850 | 81.4693 | 70.5447 |
| AT4G37850 | 81.4674 | 101.114 |
| AT5G24530 | 81.4499 | 14.5107 |
| AT2G18750 | 81.3487 | 94.9388 |
| AT1G08360 | 81.3253 | 355.83 |
| AT1G57990 | 81.2862 | 30.8932 |
| AT1G29340 | 81.2147 | 68.4543 |
| AT5G46430 | 81.081 | 243.717 |
| AT2G25980 | 81.0169 | 162.963 |
| AT5G14120 | 80.9475 | 13.2065 |
| AT1G58030 | 80.8838 | 55.4483 |
| AT3G16350 | 80.7608 | 22.9207 |
| AT2G19160 | 80.7333 | 21.3898 |
| AT1G72140 | 80.5298 | 150.868 |
| AT3G18200 | 80.5179 | 14.3929 |
| AT4G33940 | 80.4888 | 65.6689 |
| AT5G49270 | 80.4778 | 0.313345 |
| AT3G02700 | 80.4128 | 34.9104 |
| AT5G65683 | 80.3407 | 73.5758 |
| AT5G64905 | 80.2458 | 52.6369 |
| AT1G09430 | 80.2126 | 41.0339 |
| AT3G04920 | 80.1522 | 320.615 |
| AT3G24070 | 80.1151 | 28.6435 |
| AT4G27470 | 80.1146 | 25.338 |
| AT2G45160 | 80.1101 | 25.9677 |
| AT5G46900 | 80.0738 | 23.358 |
| AT5G42785 | 80.0565 | 1.42305 |
| AT4G09800 | 80.0455 | 299.964 |
| AT1G30640 | 80.037 | 15.3104 |
| AT4G38680 | 79.9954 | 63.7513 |
| AT1G72700 | 79.8422 | 29.9921 |
| AT4G22930 | 79.7268 | 67.1004 |
| AT5G41520 | 79.7031 | 187.057 |
| AT2G41220 | 79.6966 | 42.6377 |
| AT5G37740 | 79.6733 | 60.4655 |
| AT5G27840 | 79.6706 | 42.6966 |
| AT3G04880 | 79.6404 | 14.5778 |
| AT5G59960 | 79.6069 | 28.0324 |
| AT5G01720 | 79.5281 | 17.6356 |
| AT1G11310 | 79.4894 | 71.9832 |
| AT5G27470 | 79.4629 | 87.2344 |
| AT4G39910 | 79.4582 | 52.6451 |
| AT3G13720 | 79.445 | 21.8349 |
| AT2G28740 | 79.4158 | 157.298 |
| AT1G77370 | 79.3915 | 47.6446 |
| AT1G70750 | 79.3693 | 53.273 |
| AT4G39260 | 79.2664 | 69.8016 |
| AT4G13020 | 79.254 | 49.9412 |
| AT5G18680 | 79.2469 | 12.9936 |
| AT4G36780 | 79.147 | 121.126 |
| AT3G56150 | 79.1086 | 83.7773 |
| AT1G79750 | 79.0957 | 62.1302 |
| AT4G15990 | 79.088 | 15.1879 |
| AT2G44410 | 79.0765 | 32.9902 |
| AT5G26280 | 79.0741 | 70.6991 |
| AT4G32130 | 79.039 | 62.3486 |
| AT4G32440 | 79.028 | 57.8626 |
| AT3G29170 | 79.0126 | 60.4506 |
| AT1G68840 | 78.961 | 48.2435 |
| AT2G37190 | 78.9364 | 253.414 |
| AT2G19450 | 78.936 | 26.608 |
| AT1G11840 | 78.9091 | 98.3458 |
| AT4G24440 | 78.8775 | 52.16 |
| AT4G33530 | 78.8427 | 37.8523 |
| AT4G13270 | 78.8421 | 44.6272 |
| AT2G40800 | 78.7952 | 32.1192 |
| AT2G39960 | 78.7531 | 87.2459 |
| AT1G07770 | 78.7206 | 220.625 |
| AT2G34560 | 78.6851 | 109.967 |
| AT2G45010 | 78.4777 | 34.5411 |
| AT3G19820 | 78.4693 | 186.005 |
| AT1G79450 | 78.4252 | 25.443 |
| AT3G28210 | 78.3903 | 65.9772 |
| AT3G02555 | 78.3567 | 41.3341 |
| AT4G24330 | 78.3316 | 60.5213 |
| AT2G31800 | 78.2638 | 38.7769 |
| AT3G02790 | 78.2546 | 95.4882 |
| AT3G04060 | 78.1568 | 18.409 |
| AT5G23670 | 78.1418 | 43.5423 |
| AT2G42880 | 78.1379 | 64.5344 |
| AT3G14590 | 78.1298 | 36.2022 |
| AT2G46340 | 78.1265 | 36.8071 |
| AT5G01040 | 78.0965 | 51.9353 |
| AT2G29490 | 78.0709 | 113.623 |
| AT3G05200 | 78.0553 | 38.506 |
| AT5G65280 | 78.007 | 29.861 |
| AT2G18980 | 77.9982 | 7.26961 |
| AT4G21840 | 77.986 | 72.108 |
| AT4G18930 | 77.9801 | 44.6053 |
| AT4G25070 | 77.9769 | 5.12659 |
| AT4G38250 | 77.9488 | 22.6118 |
| AT1G16000 | 77.8835 | 40.0594 |
| AT2G47320 | 77.8693 | 82.7293 |
| AT5G57340 | 77.8256 | 45.0083 |
| AT3G59360 | 77.7428 | 34.848 |
| AT1G12810 | 77.6718 | 23.5191 |
| AT2G32210 | 77.5577 | 66.6624 |
| AT5G06600 | 77.5472 | 51.8254 |
| AT5G62865 | 77.5281 | 15.0966 |
| AT1G02816 | 77.5073 | 32.1333 |
| AT1G10650 | 77.4289 | 34.163 |
| AT4G02940 | 77.3472 | 64.8766 |
| AT1G78680 | 77.3343 | 23.7285 |
| AT3G48170 | 77.301 | 63.063 |
| AT4G26770 | 77.2703 | 0.783864 |
| AT1G31910 | 77.2446 | 61.058 |
| AT1G64530 | 77.2329 | 12.3854 |
| AT1G23480 | 77.2241 | 17.4475 |
| AT5G24270 | 77.209 | 29.9928 |
| AT2G28430 | 77.1847 | 27.268 |
| AT5G64500 | 77.1243 | 46.6263 |
| AT2G01100 | 77.0031 | 51.9349 |
| AT3G27190 | 76.997 | 27.6417 |
| AT5G58700 | 76.9284 | 63.4143 |
| AT5G25350 | 76.9017 | 46.6216 |
| AT2G16060 | 76.7655 | 166.921 |
| AT4G33640 | 76.7475 | 37.0833 |
| AT5G42870 | 76.6876 | 35.8462 |
| AT1G26580 | 76.6863 | 55.9913 |
| AT1G80780 | 76.589 | 44.4657 |
| AT1G36370 | 76.5353 | 76.2676 |
| AT4G13010 | 76.4882 | 32.8095 |
| AT4G26320 | 76.4772 | 111.381 |
| AT1G79380 | 76.4026 | 52.6039 |
| AT1G10030 | 76.3961 | 69.4534 |
| AT3G13360 | 76.3172 | 43.5742 |
| AT4G00100 | 76.2889 | 214.325 |
| AT5G23450 | 76.2715 | 50.4906 |
| AT2G34500 | 76.233 | 3.75831 |
| AT5G58950 | 76.2189 | 53.8657 |
| AT1G28250 | 76.0887 | 57.7645 |
| AT2G17360 | 76.0841 | 265.569 |
| AT3G14350 | 76.0462 | 16.6905 |
| AT3G43300 | 76.0275 | 53.4215 |
| AT5G55960 | 75.9385 | 10.8671 |
| AT5G61960 | 75.896 | 46.3947 |
| AT5G46750 | 75.8925 | 68.5055 |
| AT4G03110 | 75.7694 | 21.3893 |
| AT1G78610 | 75.7308 | 58.9284 |
| AT5G25770 | 75.721 | 31.3532 |
| AT1G54000 | 75.7055 | 72.7131 |
| AT5G53650 | 75.6275 | 52.8249 |
| AT3G05545 | 75.4836 | 36.6007 |
| AT3G52740 | 75.4654 | 12.0904 |
| AT4G24740 | 75.4543 | 41.9862 |
| AT3G16510 | 75.4123 | 16.69 |
| AT1G11480 | 75.4091 | 45.0041 |
| AT2G22780 | 75.3377 | 69.1803 |
| AT3G60690 | 75.3346 | 27.1516 |
| AT1G06890 | 75.2094 | 82.5955 |
| AT1G27350 | 75.1673 | 83.1794 |
| AT4G11860 | 75.0775 | 31.9417 |
| AT3G12700 | 75.0427 | 9.33747 |
| AT5G26751 | 74.9727 | 54.6549 |
| AT4G20460 | 74.9464 | 8.57923 |
| AT3G17880 | 74.9397 | 75.3852 |
| AT5G53330 | 74.9355 | 46.0667 |
| AT1G13960 | 74.8601 | 46.7845 |
| AT2G20330 | 74.8511 | 34.9562 |
| AT4G19410 | 74.7794 | 120.665 |
| AT5G44480 | 74.7474 | 2.64666 |
| AT1G18080 | 74.7469 | 186.183 |
| AT1G67560 | 74.7293 | 60.6218 |
| AT3G55050 | 74.7094 | 43.2178 |
| AT4G35000 | 74.6761 | 38.0986 |
| AT4G28610 | 74.6471 | 44.3633 |
| AT1G28290 | 74.6466 | 581.627 |
| AT1G29690 | 74.6177 | 33.786 |
| AT3G22110 | 74.6025 | 107.279 |
| AT4G38930 | 74.5368 | 31.734 |
| AT1G64060 | 74.5143 | 46.7624 |
| AT1G44770 | 74.5119 | 30.3499 |
| AT2G36460 | 74.4942 | 63.2624 |
| AT2G37940 | 74.4619 | 45.4926 |
| AT2G35880 | 74.4461 | 46.5086 |
| AT2G19180 | 74.4342 | 41.9586 |
| AT5G65990 | 74.4234 | 14.3419 |
| AT3G50000 | 74.356 | 61.3433 |
| AT2G39270 | 74.2887 | 53.2704 |
| AT5G53400 | 74.2616 | 78.2458 |
| AT2G03470 | 74.2598 | 70.6042 |
| AT5G65160 | 74.2567 | 0.744421 |
| AT5G25930 | 74.2534 | 42.5661 |
| AT2G41640 | 74.2369 | 36.01 |
| AT4G16695 | 74.235 | 42.7503 |
| AT1G26830 | 74.2023 | 44.2205 |
| AT1G49600 | 74.1275 | 59.3104 |
| AT2G47060 | 74.1196 | 81.9564 |
| AT1G72730 | 74.0333 | 40.5123 |
| AT5G45428 | 74.0037 | 51.7447 |
| AT3G25597 | 74.0012 | 149.426 |
| AT1G18540 | 73.999 | 366.877 |
| AT1G05500 | 73.9745 | 59.498 |
| AT1G54710 | 73.9124 | 33.5335 |
| AT1G67230 | 73.8894 | 40.652 |
| AT5G25100 | 73.8611 | 69.5796 |
| AT1G73250 | 73.8429 | 32.5125 |
| AT4G15410 | 73.8014 | 31.6752 |
| AT2G29980 | 73.7183 | 54.1529 |
| AT5G61900 | 73.6955 | 50.7231 |
| AT2G44500 | 73.6579 | 43.899 |
| AT1G48330 | 73.6267 | 134.987 |
| AT3G54030 | 73.6248 | 44.6429 |
| AT3G07570 | 73.5843 | 13.8761 |
| AT4G12070 | 73.5546 | 19.9464 |
| AT5G02420 | 73.5097 | 17.3867 |
| AT3G14260 | 73.5029 | 37.4352 |
| AT1G15230 | 73.497 | 39.7699 |
| AT5G26600 | 73.489 | 34.1652 |
| AT1G59580 | 73.4368 | 37.1292 |
| AT4G36950 | 73.4335 | 83.624 |
| AT4G29390 | 73.4066 | 170.429 |
| AT5G46780 | 73.4034 | 21.0306 |
| AT2G47960 | 73.3726 | 37.0399 |
| AT3G55620 | 73.3376 | 111.318 |
| AT3G13610 | 73.298 | 31.0472 |
| AT2G30230 | 73.2051 | 24.444 |
| AT5G20990 | 73.17 | 27.6679 |
| AT3G02840 | 73.0916 | 30.8079 |
| AT5G22360 | 72.9364 | 41.8904 |
| AT2G30970 | 72.895 | 47.3755 |
| AT2G21390 | 72.8725 | 61.1568 |
| AT1G76900 | 72.8638 | 24.5432 |
| AT5G38900 | 72.8471 | 0.77725 |
| AT2G42270 | 72.8414 | 25.7423 |
| AT1G09200 | 72.8383 | 84.2755 |
| AT1G52280 | 72.8166 | 69.5268 |
| AT4G24020 | 72.7891 | 34.8201 |
| AT1G72690 | 72.785 | 36.8245 |
| AT1G72790 | 72.7564 | 13.9791 |
| AT5G22950 | 72.7047 | 41.0799 |
| AT5G18630 | 72.6801 | 37.4097 |
| AT5G54145 | 72.6663 | 59.9213 |
| AT2G44525 | 72.6661 | 35.9009 |
| AT2G18090 | 72.6228 | 61.7251 |
| AT5G44050 | 72.4441 | 20.3635 |
| AT1G10700 | 72.4164 | 90.6878 |
| AT5G11710 | 72.3847 | 44.0558 |
| AT5G03160 | 72.3611 | 55.4491 |
| AT1G68945 | 72.3286 | 33.4531 |
| AT2G38290 | 72.3125 | 17.5239 |
| AT4G28850 | 72.3025 | 0.052452 |
| AT3G19290 | 72.2665 | 27.2031 |
| AT3G19450 | 72.2626 | 37.3258 |
| AT3G48360 | 72.2546 | 25.3099 |
| AT3G52748 | 72.2441 | 38.8346 |
| AT5G46020 | 72.243 | 107.819 |
| AT4G00305 | 72.2014 | 38.2767 |
| AT1G02090 | 72.1775 | 55.7252 |
| AT1G47740 | 72.1497 | 25.7954 |
| AT1G01780 | 72.1454 | 4.27052 |
| AT1G17745 | 72.1124 | 44.8837 |
| AT1G57560 | 72.101 | 2.33254 |
| AT4G17390 | 72.0515 | 151.029 |
| AT5G24400 | 72.0347 | 81.3105 |
| AT2G46680 | 72.0262 | 47.4078 |
| AT1G18860 | 71.9443 | 11.7393 |
| AT1G22520 | 71.8631 | 43.631 |
| AT1G21720 | 71.8394 | 86.5869 |
| AT2G23780 | 71.819 | 32.401 |
| AT5G27920 | 71.768 | 23.8018 |
| AT3G22230 | 71.7602 | 173.074 |
| AT1G17730 | 71.7267 | 42.563 |
| AT3G02740 | 71.6389 | 66.2542 |
| AT4G23400 | 71.5946 | 23.2746 |
| AT2G02870 | 71.5707 | 37.5789 |
| AT2G40110 | 71.5415 | 15.8843 |
| AT4G25680 | 71.489 | 47.6313 |
| AT2G44100 | 71.4818 | 65.6227 |
| AT3G55470 | 71.4818 | 42.0218 |
| AT1G34047 | 71.4472 | 98.1876 |
| AT5G16760 | 71.4448 | 43.9677 |
| AT1G64390 | 71.3597 | 149.024 |
| AT5G58560 | 71.3247 | 22.7148 |
| AT1G30580 | 71.29 | 158.499 |
| AT5G51460 | 71.2541 | 27.1365 |
| AT1G56580 | 71.2521 | 66.7714 |
| AT4G13430 | 71.2215 | 84.1018 |
| AT2G31020 | 71.1677 | 14.1562 |
| AT2G35635 | 71.1646 | 63.4605 |
| AT5G06140 | 71.1594 | 51.1895 |
| AT5G59160 | 71.1482 | 26.6621 |
| AT5G42390 | 71.0713 | 39.398 |
| AT3G66654 | 71.0508 | 45.6273 |
| AT1G36050 | 70.9726 | 39.2793 |
| AT4G24580 | 70.9671 | 2.63631 |
| AT5G62530 | 70.9509 | 71.1186 |
| AT1G56550 | 70.9225 | 4.9953 |
| AT3G12620 | 70.9108 | 32.903 |
| AT4G09720 | 70.9056 | 48.312 |
| AT2G44730 | 70.8934 | 69.8223 |
| AT5G24870 | 70.8318 | 18.0283 |
| AT2G25260 | 70.7576 | 11.7957 |
| AT1G04120 | 70.6652 | 43.8389 |
| AT5G20160 | 70.624 | 155.856 |
| AT5G27770 | 70.6174 | 294.347 |
| AT1G17147 | 70.5991 | 34.4232 |
| AT5G63570 | 70.572 | 31.3799 |
| AT4G02050 | 70.5523 | 31.1933 |
| AT3G12290 | 70.5382 | 64.1285 |
| AT4G30100 | 70.5207 | 48.0476 |
| AT3G10770 | 70.4999 | 42.5588 |
| AT1G79970 | 70.4907 | 23.1748 |
| AT5G66690 | 70.4113 | 34.0864 |
| AT2G26140 | 70.3786 | 58.1325 |
| AT4G38220 | 70.357 | 32.0089 |
| AT1G78420 | 70.3338 | 59.2844 |
| AT5G25280 | 70.3278 | 36.0634 |
| AT2G39200 | 70.3006 | 61.5693 |
| AT4G17486 | 70.2837 | 34.3099 |
| AT5G03340 | 70.2711 | 79.7628 |
| AT1G62370 | 70.2079 | 17.4973 |
| AT3G26910 | 70.1943 | 37.5318 |
| AT3G22290 | 70.1849 | 65.6513 |
| AT2G39390 | 70.1616 | 236.577 |
| AT1G63440 | 70.1084 | 45.6809 |
| AT1G73860 | 70.0785 | 1.40914 |
| AT5G10830 | 70.0782 | 26.0664 |
| AT3G13460 | 70.0379 | 89.1616 |
| AT4G29330 | 69.9904 | 61.8646 |
| AT3G63080 | 69.9564 | 23.021 |
| AT1G05320 | 69.93 | 4.65597 |
| AT3G17800 | 69.8345 | 76.2723 |
| AT1G74230 | 69.8316 | 41.703 |
| AT3G23820 | 69.8001 | 63.4818 |
| AT5G60460 | 69.7808 | 14.4345 |
| AT4G21580 | 69.7772 | 68.3162 |
| AT5G65390 | 69.7704 | 26.1388 |
| AT2G29470 | 69.7378 | 168.027 |
| AT1G76140 | 69.6534 | 52.0341 |
| AT4G15940 | 69.6264 | 30.0887 |
| AT4G34670 | 69.6244 | 348.524 |
| AT3G11170 | 69.5908 | 82.1884 |
| AT2G27350 | 69.5898 | 40.7967 |
| AT3G25800 | 69.5893 | 39.442 |
| AT5G13160 | 69.5825 | 62.7718 |
| AT2G01600 | 69.5799 | 52.2373 |
| AT5G41280 | 69.5715 | 3.55533 |
| AT2G24200 | 69.5465 | 96.6567 |
| AT1G06840 | 69.5418 | 13.9755 |
| AT3G52090 | 69.4799 | 61.6313 |
| AT5G04590 | 69.4622 | 142.031 |
| AT4G28085 | 69.4596 | 21.5945 |
| AT1G03860 | 69.4224 | 61.3915 |
| AT1G79390 | 69.414 | 40.3214 |
| AT1G03550 | 69.3496 | 6.09104 |
| AT3G53740 | 69.3473 | 261.563 |
| AT3G47160 | 69.3431 | 41.2683 |
| AT3G24550 | 69.3398 | 39.2465 |
| AT2G36900 | 69.3321 | 43.2905 |
| AT2G28190 | 69.2558 | 65.0636 |
| AT2G20860 | 69.2135 | 38.8235 |
| AT2G03820 | 69.1575 | 50.6237 |
| AT3G02750 | 69.1263 | 38.8008 |
| AT1G43710 | 69.0729 | 58.4397 |
| AT1G02840 | 69.0308 | 83.9954 |
| AT5G46030 | 68.996 | 80.8653 |
| AT5G01010 | 68.9182 | 37.2616 |
| AT4G33780 | 68.9051 | 37.5849 |
| AT3G02570 | 68.7884 | 33.4355 |
| AT5G49910 | 68.7243 | 47.0223 |
| AT4G39660 | 68.6979 | 81.8605 |
| AT2G34690 | 68.6308 | 47.016 |
| AT3G48070 | 68.6277 | 47.9145 |
| AT5G60410 | 68.6125 | 49.2211 |
| AT4G28480 | 68.5705 | 38.0872 |
| AT2G22121 | 68.5414 | 16.8882 |
| AT5G24240 | 68.5324 | 31.6761 |
| AT5G08240 | 68.5301 | 13.01 |
| AT2G17300 | 68.4975 | 32.8976 |
| AT1G26640 | 68.4817 | 20.9517 |
| AT3G10915 | 68.465 | 40.1711 |
| AT5G24170 | 68.4319 | 6.62262 |
| AT2G25255 | 68.3439 | 1.30013 |
| AT5G23575 | 68.3347 | 90.4201 |
| AT2G32080 | 68.2973 | 40.4391 |
| AT1G80240 | 68.2769 | 27.916 |
| AT5G35530 | 68.2488 | 174.725 |
| AT5G01710 | 68.248 | 39.2915 |
| AT5G66170 | 68.2404 | 81.5684 |
| AT3G09770 | 68.2205 | 52.6439 |
| AT5G19100 | 68.1949 | 178.547 |
| AT2G44080 | 68.172 | 137.009 |
| AT5G20060 | 68.1508 | 32.8874 |
| AT5G54780 | 68.1195 | 24.939 |
| AT2G25110 | 68.1175 | 56.631 |
| AT3G48000 | 68.1123 | 178.105 |
| AT5G66730 | 68.0712 | 54.214 |
| AT5G59150 | 68.0582 | 14.7476 |
| AT4G26940 | 67.9875 | 64.2363 |
| AT5G49080 | 67.9871 | 0.0529656 |
| AT3G22530 | 67.8971 | 35.3263 |
| AT1G50590 | 67.8621 | 25.3163 |
| AT3G10380 | 67.8332 | 39.5179 |
| AT4G32760 | 67.8295 | 42.4772 |
| AT3G07330 | 67.8097 | 28.3219 |
| AT5G67560 | 67.7974 | 36.5313 |
| AT1G74790 | 67.7972 | 25.4186 |
| AT5G20920 | 67.7892 | 90.2327 |
| AT4G24550 | 67.7571 | 47.125 |
| AT5G46420 | 67.7567 | 29.0512 |
| AT5G49900 | 67.753 | 8.07035 |
| AT2G22795 | 67.7529 | 54.4134 |
| AT5G10030 | 67.738 | 37.0487 |
| AT1G23149 | 67.7014 | 49.384 |
| AT2G44160 | 67.6687 | 70.9882 |
| AT3G05220 | 67.6208 | 52.1425 |
| AT5G66250 | 67.552 | 27.6104 |
| AT4G01370 | 67.5353 | 29.5181 |
| AT4G11280 | 67.5176 | 27.0464 |
| AT2G32510 | 67.5074 | 9.13729 |
| AT3G10820 | 67.4511 | 44.7678 |
| AT1G26690 | 67.4155 | 33.4275 |
| AT1G66680 | 67.3766 | 36.7801 |
| AT5G61170 | 67.3293 | 198.384 |
| AT3G01820 | 67.3201 | 51.9609 |
| AT4G24310 | 67.2739 | 12.4374 |
| AT4G31420 | 67.2055 | 40.7941 |
| AT2G25900 | 67.1578 | 55.0177 |
| AT3G27430 | 67.15 | 59.6235 |
| AT3G04620 | 67.1018 | 42.0001 |
| AT4G26570 | 67.0954 | 45.2985 |
| AT4G28290 | 67.0803 | 105.194 |
| AT1G60420 | 67.0665 | 25.233 |
| AT5G35590 | 67.0373 | 92.4978 |
| AT5G12370 | 67.0316 | 35.0649 |
| AT2G21170 | 67.027 | 67.7533 |
| AT4G01360 | 66.9772 | 16.7422 |
| AT5G27670 | 66.9639 | 101.516 |
| AT3G04540 | 66.9511 | 0.922073 |
| AT1G47200 | 66.8961 | 45.3606 |
| AT4G24030 | 66.8524 | 6.9489 |
| AT1G60190 | 66.8287 | 21.5536 |
| AT3G46440 | 66.7118 | 68.7715 |
| AT1G20780 | 66.7021 | 41.8446 |
| AT5G48010 | 66.6788 | 86.6215 |
| AT2G01470 | 66.6417 | 60.2169 |
| AT4G01320 | 66.6364 | 85.9173 |
| AT3G46830 | 66.6165 | 27.5503 |
| AT2G43350 | 66.571 | 35.8871 |
| AT5G47640 | 66.5702 | 37.7793 |
| AT1G75270 | 66.5525 | 68.1443 |
| AT5G14310 | 66.5391 | 15.7283 |
| AT3G60980 | 66.5243 | 11.9634 |
| AT1G10950 | 66.4997 | 81.5319 |
| AT2G16500 | 66.479 | 57.2466 |
| AT1G48900 | 66.3836 | 49.6933 |
| AT4G02500 | 66.383 | 60.6727 |
| AT4G27270 | 66.3732 | 14.355 |
| AT2G25910 | 66.3663 | 35.1007 |
| AT5G13780 | 66.2414 | 56.0336 |
| AT1G78895 | 66.2371 | 45.9134 |
| AT3G11530 | 66.1961 | 32.2209 |
| AT5G16120 | 66.195 | 29.8132 |
| AT1G54290 | 66.1774 | 41.1588 |
| AT3G56500 | 66.1724 | 0.575744 |
| AT2G20580 | 66.1712 | 58.1646 |
| AT5G12470 | 66.1444 | 65.3961 |
| AT3G08720 | 66.1055 | 62.733 |
| AT1G09932 | 66.0425 | 28.3703 |
| AT2G05940 | 66.0352 | 18.9922 |
| AT4G32040 | 66.033 | 31.0963 |
| AT1G07790 | 66.0168 | 86.594 |
| AT5G14800 | 66.0094 | 81.819 |
| AT1G75170 | 65.9623 | 28.4406 |
| AT1G32130 | 65.9588 | 52.5893 |
| AT3G14840 | 65.9554 | 59 |
| AT3G47300 | 65.9069 | 35.4131 |
| AT4G38932 | 65.8912 | 36.0746 |
| AT5G12190 | 65.8231 | 30.7995 |
| AT1G21760 | 65.7834 | 34.5626 |
| AT3G53870 | 65.7694 | 216.63 |
| AT3G09630 | 65.7655 | 195.693 |
| AT1G33980 | 65.7148 | 44.3852 |
| AT5G12120 | 65.7132 | 40.0145 |
| AT5G01460 | 65.6713 | 31.7398 |
| AT4G12340 | 65.6374 | 47.0021 |
| AT2G02860 | 65.6281 | 41.8791 |
| AT1G67250 | 65.6128 | 68.3896 |
| AT5G48930 | 65.5914 | 29.7136 |
| AT2G46400 | 65.5559 | 92.1559 |
| AT5G67320 | 65.5406 | 30.5166 |
| AT4G00810 | 65.4927 | 127.218 |
| AT2G01690 | 65.4389 | 33.5541 |
| AT3G10720 | 65.4133 | 215.818 |
| AT2G47970 | 65.4127 | 27.6397 |
| AT2G34180 | 65.395 | 3.33008 |
| AT1G01910 | 65.3894 | 59.0237 |
| AT5G55050 | 65.3686 | 6.53833 |
| AT1G06780 | 65.343 | 54.1848 |
| AT1G06700 | 65.3337 | 61.8176 |
| AT3G04070 | 65.2786 | 4.64433 |
| AT3G06190 | 65.2615 | 43.5161 |
| AT5G59420 | 65.2097 | 58.5768 |
| AT4G14430 | 65.1954 | 16.0831 |
| AT5G24980 | 65.1522 | 24.6959 |
| AT2G43535 | 65.0661 | 100.611 |
| AT4G30550 | 65.054 | 65.6398 |
| AT1G53850 | 64.9781 | 88.7008 |
| AT1G72160 | 64.9676 | 25.294 |
| AT1G06290 | 64.9551 | 33.0393 |
| AT1G33590 | 64.9448 | 174.174 |
| AT1G48790 | 64.8856 | 28.7935 |
| AT5G60710 | 64.8766 | 39.8325 |
| AT2G35730 | 64.8507 | 3.94136 |
| AT2G32520 | 64.85 | 45.8433 |
| AT3G46460 | 64.8416 | 60.0553 |
| AT4G22758 | 64.7652 | 4.80327 |
| AT4G39680 | 64.7565 | 58.7489 |
| AT3G25585 | 64.7497 | 55.5978 |
| AT1G07000 | 64.7368 | 13.2685 |
| AT5G07450 | 64.7111 | 2.49592 |
| AT1G44170 | 64.6943 | 22.3794 |
| AT1G29310 | 64.6312 | 50.7738 |
| AT4G14800 | 64.6285 | 49.5677 |
| AT1G68670 | 64.6195 | 31.4639 |
| AT5G10480 | 64.6187 | 52.3515 |
| AT5G39730 | 64.5917 | 63.3791 |
| AT5G12310 | 64.5785 | 36.4457 |
| AT3G02560 | 64.5742 | 247.416 |
| AT5G02350 | 64.5379 | 15.4004 |
| AT3G62600 | 64.5369 | 56.5665 |
| AT1G45000 | 64.5232 | 77.6054 |
| AT5G08535 | 64.4844 | 28.3386 |
| AT1G17880 | 64.444 | 182.702 |
| AT3G60770 | 64.4118 | 250.469 |
| AT4G10840 | 64.3685 | 55.6198 |
| AT3G02070 | 64.3665 | 25.7361 |
| AT4G21920 | 64.337 | 42.8225 |
| AT2G16365 | 64.3346 | 31.3194 |
| AT5G47430 | 64.3144 | 36.9067 |
| AT3G56050 | 64.302 | 57.8062 |
| AT3G53230 | 64.2928 | 91.2758 |
| AT4G37070 | 64.281 | 2.6652 |
| AT5G48530 | 64.245 | 68.1422 |
| AT3G50360 | 64.2231 | 94.0337 |
| AT2G24260 | 64.1733 | 8.81101 |
| AT3G10260 | 64.1554 | 47.5212 |
| AT5G63880 | 64.1395 | 46.0404 |
| AT1G03230 | 64.119 | 137.252 |
| AT3G59090 | 64.0939 | 27.903 |
| AT1G64190 | 64.0777 | 43.3442 |
| AT3G47550 | 64.0455 | 51.333 |
| AT1G21080 | 64.0345 | 44.5808 |
| AT5G15180 | 64.0184 | 6.26012 |
| AT5G62310 | 64.0072 | 0.516494 |
| AT1G64650 | 63.9999 | 66.7027 |
| AT1G45976 | 63.9206 | 39.5389 |
| AT3G14200 | 63.917 | 62.3419 |
| AT5G67420 | 63.8819 | 28.6128 |
| AT2G33120 | 63.8742 | 34.4288 |
| AT2G42520 | 63.8609 | 26.015 |
| AT3G24800 | 63.8442 | 36.8712 |
| AT4G37180 | 63.8401 | 48.5811 |
| AT1G06760 | 63.8301 | 83.9334 |
| AT3G10500 | 63.7798 | 39.8976 |
| AT1G68820 | 63.7703 | 22.159 |
| AT5G22350 | 63.7546 | 27.18 |
| AT5G58030 | 63.7451 | 53.6408 |
| AT3G49590 | 63.7078 | 28.2052 |
| AT5G54750 | 63.6863 | 65.8814 |
| AT3G07320 | 63.6626 | 67.7514 |
| AT2G37550 | 63.6591 | 54.6488 |
| AT1G59590 | 63.6587 | 28.5954 |
| AT5G63030 | 63.5991 | 67.3905 |
| AT1G17500 | 63.4985 | 28.0971 |
| AT3G10930 | 63.4911 | 32.562 |
| AT3G45310 | 63.4832 | 83.5344 |
| AT5G63260 | 63.4627 | 28.706 |
| AT5G01490 | 63.4543 | 24.1935 |
| AT5G04920 | 63.3766 | 28.7155 |
| AT3G53580 | 63.3581 | 36.062 |
| AT4G31080 | 63.3132 | 28.0074 |
| AT5G11700 | 63.3087 | 36.8367 |
| AT1G73230 | 63.2581 | 173.205 |
| AT5G26990 | 63.2206 | 27.6831 |
| AT3G45640 | 63.1789 | 132.297 |
| AT2G19680 | 63.1189 | 75.732 |
| AT5G07370 | 63.108 | 37.9264 |
| AT3G61790 | 63.1015 | 39.0816 |
| AT2G39950 | 63.0984 | 22.0124 |
| AT3G17000 | 63.0919 | 66.3461 |
| AT1G21930 | 63.0837 | 21.3822 |
| AT3G44100 | 63.0466 | 68.538 |
| AT5G42990 | 63.0328 | 54.048 |
| AT1G58180 | 63.0272 | 38.9062 |
| AT5G37370 | 63.0143 | 49.2951 |
| AT4G30390 | 63.005 | 30.5506 |
| AT1G53560 | 63.0002 | 24.436 |
| AT3G14430 | 62.9747 | 38.1703 |
| AT1G61820 | 62.9659 | 46.7825 |
| AT2G20560 | 62.9584 | 47.4535 |
| AT5G49940 | 62.9316 | 48.623 |
| AT4G10380 | 62.9293 | 37.8018 |
| AT3G14185 | 62.9255 | 2.86014 |
| AT1G06210 | 62.9194 | 60.2738 |
| AT1G02610 | 62.8885 | 12.4142 |
| AT5G39660 | 62.8389 | 28.4673 |
| AT1G74590 | 62.8311 | 25.4314 |
| AT5G42970 | 62.8294 | 44.8102 |
| AT3G53120 | 62.7953 | 47.836 |
| AT1G10050 | 62.7757 | 22.2312 |
| AT5G10650 | 62.7646 | 22.6341 |
| AT3G07100 | 62.7544 | 42.5721 |
| AT2G21190 | 62.7374 | 37.5873 |
| AT2G20080 | 62.7169 | 19.6498 |
| AT3G08970 | 62.6818 | 63.3374 |
| AT3G06350 | 62.6618 | 35.1732 |
| AT2G46080 | 62.6498 | 41.1532 |
| AT2G39720 | 62.6372 | 25.9228 |
| AT3G10250 | 62.627 | 38.6246 |
| AT4G33666 | 62.615 | 13.3773 |
| AT1G55310 | 62.6006 | 26.561 |
| AT4G01110 | 62.5961 | 0.387963 |
| AT5G56190 | 62.4989 | 34.9447 |
| AT1G34750 | 62.4903 | 13.0327 |
| AT5G43050 | 62.4764 | 24.7659 |
| AT5G50430 | 62.4515 | 36.7641 |
| AT3G05155 | 62.3995 | 2.00907 |
| AT1G71010 | 62.3941 | 33.0619 |
| AT4G32600 | 62.3853 | 26.9047 |
| AT5G17350 | 62.355 | 18.116 |
| AT5G63905 | 62.3535 | 39.5439 |
| AT4G25090 | 62.3366 | 0.714573 |
| AT2G44970 | 62.308 | 39.1915 |
| AT3G06850 | 62.2713 | 27.7867 |
| AT1G78870 | 62.2494 | 52.0134 |
| AT3G27960 | 62.2265 | 28.8217 |
| AT1G07135 | 62.2142 | 36.9224 |
| AT1G48030 | 62.2089 | 37.1371 |
| AT1G13990 | 62.1929 | 89.9003 |
| AT5G67530 | 62.1712 | 25.0897 |
| AT5G44100 | 62.1649 | 38.0112 |
| AT1G78210 | 62.151 | 106.539 |
| AT5G46170 | 62.1307 | 51.4059 |
| AT3G42790 | 62.1098 | 74.9636 |
| AT2G24840 | 62.0978 | 2.70141 |
| AT2G47360 | 62.0735 | 0.184463 |
| AT1G20575 | 62.0617 | 43.0883 |
| AT1G08990 | 62.0058 | 0.388489 |
| AT5G18900 | 62.0034 | 30.5596 |
| AT1G09590 | 62.0026 | 166.559 |
| AT2G02970 | 61.9371 | 21.1999 |
| AT1G13195 | 61.9236 | 53.6936 |
| AT1G05350 | 61.9147 | 44.8743 |
| AT3G12360 | 61.9038 | 42.4141 |
| AT1G63770 | 61.8655 | 53.4327 |
| AT2G25735 | 61.8509 | 19.7673 |
| AT5G63220 | 61.844 | 21.5837 |
| AT5G43430 | 61.7393 | 49.29 |
| AT1G22440 | 61.6693 | 76.6366 |
| AT1G56200 | 61.6483 | 65.8317 |
| AT3G57020 | 61.6307 | 45.0035 |
| AT4G22780 | 61.6287 | 16.1688 |
| AT4G29070 | 61.6264 | 53.9793 |
| AT5G64170 | 61.6213 | 32.1913 |
| AT5G01760 | 61.6202 | 16.7934 |
| AT5G59480 | 61.541 | 74.9938 |
| AT5G19240 | 61.5293 | 9.59602 |
| AT2G42790 | 61.5177 | 37.5558 |
| AT1G11960 | 61.4936 | 35.835 |
| AT1G72710 | 61.4909 | 61.4485 |
| AT4G35770 | 61.4599 | 26.0504 |
| AT4G35550 | 61.4362 | 17.9595 |
| AT5G11850 | 61.4303 | 27.5569 |
| AT3G48450 | 61.3894 | 66.5976 |
| AT1G69460 | 61.3667 | 54.8905 |
| AT4G12120 | 61.3372 | 25.5521 |
| AT5G59570 | 61.3321 | 28.4982 |
| AT2G39705 | 61.2742 | 48.6426 |
| AT2G24610 | 61.2646 | 18.3104 |
| AT4G25790 | 61.2641 | 0.0589981 |
| AT4G35800 | 61.2423 | 52.0724 |
| AT2G35190 | 61.2385 | 29.2884 |
| AT1G70160 | 61.2197 | 26.0033 |
| AT1G74940 | 61.2174 | 7.83036 |
| AT1G67060 | 61.1948 | 35.8751 |
| AT2G22475 | 61.1754 | 31.6896 |
| AT4G20300 | 61.1596 | 20.2828 |
| AT2G21120 | 61.1568 | 50.1683 |
| AT3G56950 | 61.1554 | 21.3058 |
| AT5G14000 | 61.1485 | 22.9818 |
| AT3G49560 | 61.077 | 35.5164 |
| AT1G71520 | 61.0628 | 47.483 |
| AT3G15660 | 61.0051 | 67.7885 |
| AT1G04780 | 60.9844 | 46.0087 |
| AT1G80500 | 60.9451 | 60.4997 |
| AT2G20840 | 60.939 | 30.8446 |
| AT2G30020 | 60.9359 | 27.1335 |
| AT5G09980 | 60.9127 | 115.551 |
| AT3G01910 | 60.8446 | 38.9323 |
| AT2G24040 | 60.841 | 32.6338 |
| AT4G20410 | 60.8366 | 27.4976 |
| AT1G75560 | 60.8225 | 38.5018 |
| AT1G76920 | 60.7336 | 22.9938 |
| AT1G80670 | 60.7148 | 36.0959 |
| AT5G48160 | 60.7128 | 49.5864 |
| AT2G22425 | 60.6907 | 51.3678 |
| AT1G53190 | 60.6396 | 36.1843 |
| AT1G03610 | 60.6313 | 48.8204 |
| AT5G16660 | 60.6116 | 42.9874 |
| AT5G39340 | 60.5632 | 40.2325 |
| AT3G61110 | 60.5084 | 209.111 |
| AT3G05560 | 60.4581 | 231.229 |
| AT2G38020 | 60.4358 | 23.4902 |
| AT3G21720 | 60.4263 | 54.1884 |
| AT5G02040 | 60.4105 | 25.2234 |
| AT5G47730 | 60.3999 | 34.7567 |
| AT5G19770 | 60.3932 | 55.3846 |
| AT5G53350 | 60.3918 | 35.4588 |
| AT1G52070 | 60.3826 | 556.082 |
| AT5G57710 | 60.3405 | 18.3719 |
| AT2G46740 | 60.3351 | 60.9619 |
| AT5G26010 | 60.2823 | 8.08689 |
| AT1G72175 | 60.2102 | 38.6012 |
| AT5G65720 | 60.2051 | 49.2493 |
| AT3G48760 | 60.1919 | 35.8631 |
| AT1G31300 | 60.1795 | 27.905 |
| AT2G43970 | 60.1719 | 67.072 |
| AT3G09560 | 60.1678 | 42.051 |
| AT3G09300 | 60.1614 | 45.3818 |
| AT2G31200 | 60.1548 | 49.2373 |
| AT2G19270 | 60.1153 | 53.1002 |
| AT2G27690 | 60.1014 | 21.9249 |
| AT4G21110 | 60.0966 | 64.3185 |
| AT3G07360 | 60.0916 | 16.6531 |
| AT5G40340 | 60.048 | 42.083 |
| AT2G36950 | 60.0338 | 97.4463 |
| AT1G47830 | 60.0335 | 44.0654 |
| AT5G54110 | 60.0159 | 39.7966 |
| AT5G56870 | 59.9873 | 7.7497 |
| AT5G07300 | 59.978 | 49.0387 |
| AT2G45330 | 59.914 | 16.066 |
| AT3G05000 | 59.8836 | 36.0491 |
| AT3G23605 | 59.8812 | 30.4342 |
| AT5G07070 | 59.874 | 22.1559 |
| AT3G48180 | 59.8132 | 23.9092 |
| AT4G32920 | 59.7635 | 17.4505 |
| AT2G42350 | 59.7442 | 8.36996 |
| AT3G46000 | 59.722 | 40.0412 |
| AT4G13860 | 59.7131 | 3.76483 |
| AT1G15370 | 59.7113 | 35.7586 |
| AT5G15740 | 59.7072 | 13.121 |
| AT4G24275 | 59.6963 | 123.722 |
| AT4G30470 | 59.6223 | 70.1205 |
| AT1G78920 | 59.6166 | 44.0303 |
| AT1G69960 | 59.6064 | 37.5223 |
| AT4G37520 | 59.5944 | 66.321 |
| AT2G17830 | 59.5139 | 3.51293 |
| AT1G34190 | 59.5092 | 33.9665 |
| AT5G46910 | 59.5022 | 36.1921 |
| AT5G38830 | 59.4999 | 44.4705 |
| AT5G35980 | 59.4934 | 30.3318 |
| AT1G62180 | 59.4609 | 87.2746 |
| AT5G11340 | 59.4514 | 36.512 |
| AT5G60170 | 59.4383 | 37.3939 |
| AT5G43560 | 59.4363 | 36.3741 |
| AT5G63300 | 59.4206 | 24.2889 |
| AT4G16660 | 59.3665 | 58.3233 |
| AT4G34640 | 59.3587 | 61.2314 |
| AT1G53320 | 59.3527 | 42.4398 |
| AT5G42092 | 59.3294 | 42.2108 |
| AT3G06060 | 59.2922 | 23.6819 |
| AT5G17390 | 59.2781 | 1.62984 |
| AT2G26080 | 59.2672 | 67.403 |
| AT5G40850 | 59.25 | 36.3722 |
| AT1G74270 | 59.2237 | 79.9174 |
| AT3G12300 | 59.2169 | 48.7505 |
| AT2G36910 | 59.2061 | 63.0414 |
| AT4G17890 | 59.2026 | 47.8203 |
| AT4G29220 | 59.1846 | 10.4152 |
| AT5G05100 | 59.1553 | 32.9632 |
| AT5G58430 | 59.1532 | 45.5056 |
| AT5G24030 | 59.1096 | 8.40522 |
| AT5G23740 | 59.0756 | 255.234 |
| AT2G34340 | 59.0629 | 74.8365 |
| AT5G49830 | 59.0439 | 37.0145 |
| AT1G02660 | 58.9969 | 8.77953 |
| AT1G69240 | 58.9809 | 0.124002 |
| AT3G57340 | 58.9193 | 32.6878 |
| AT1G30620 | 58.8998 | 58.8591 |
| AT3G13050 | 58.894 | 35.3018 |
| AT4G12230 | 58.8927 | 37.1081 |
| AT1G33230 | 58.8811 | 43.0001 |
| AT5G65290 | 58.8752 | 26.5078 |
| AT3G12710 | 58.8684 | 57.6013 |
| AT2G20900 | 58.8638 | 56.9453 |
| AT1G50630 | 58.8096 | 55.5702 |
| AT4G01410 | 58.7911 | 89.066 |
| AT3G62920 | 58.7642 | 47.8453 |
| AT2G33630 | 58.7472 | 32.8411 |
| AT5G20050 | 58.7207 | 27.1784 |
| AT1G10290 | 58.7087 | 41.0987 |
| AT5G63910 | 58.6875 | 26.5321 |
| AT5G15470 | 58.674 | 46.8733 |
| AT5G59690 | 58.6095 | 125.61 |
| AT1G61780 | 58.5568 | 55.415 |
| AT5G53000 | 58.5493 | 62.426 |
| AT1G69030 | 58.5307 | 58.6739 |
| AT2G22720 | 58.5197 | 36.8685 |
| AT5G64240 | 58.5095 | 33.7748 |
| AT5G55610 | 58.5058 | 33.6002 |
| AT2G26430 | 58.4438 | 39.9265 |
| AT3G42150 | 58.3746 | 35.7232 |
| AT2G33460 | 58.361 | 0.294145 |
| AT1G20010 | 58.3411 | 95.5414 |
| AT4G26650 | 58.2405 | 26.0839 |
| AT1G79830 | 58.2022 | 47.3089 |
| AT2G30060 | 58.1942 | 77.0447 |
| AT3G54360 | 58.187 | 33.2349 |
| AT2G30860 | 58.1687 | 166.785 |
| AT4G08460 | 58.1384 | 43.8923 |
| AT3G01130 | 58.1268 | 54.7926 |
| AT5G20020 | 58.1219 | 73.16 |
| AT3G47670 | 58.0724 | 19.0232 |
| AT4G14640 | 58.0532 | 14.1588 |
| AT5G02220 | 58.0286 | 24.2306 |
| AT2G23340 | 58.0188 | 9.1049 |
| AT5G27430 | 57.9377 | 37.014 |
| AT5G04860 | 57.9319 | 20.8617 |
| AT1G74920 | 57.9118 | 91.6317 |
| AT3G07230 | 57.9002 | 131.663 |
| AT1G78240 | 57.8825 | 94.8876 |
| AT1G43670 | 57.7474 | 35.3298 |
| AT5G43900 | 57.7317 | 31.0274 |
| AT3G27260 | 57.7315 | 37.2997 |
| AT4G00355 | 57.7306 | 21.6882 |
| AT5G46417 | 57.6788 | 13.8209 |
| AT5G07270 | 57.6701 | 30.5378 |
| AT3G28340 | 57.667 | 27.0611 |
| AT2G22560 | 57.6544 | 17.5945 |
| AT3G15200 | 57.6436 | 20.5358 |
| AT2G32060 | 57.6381 | 238.001 |
| AT3G57040 | 57.6293 | 14.7806 |
| AT1G74930 | 57.6123 | 12.6532 |
| AT4G36730 | 57.5724 | 35.0388 |
| AT4G33905 | 57.5654 | 25.0099 |
| AT1G30200 | 57.5473 | 34.6546 |
| AT3G10710 | 57.5381 | 0.189001 |
| AT1G80120 | 57.5308 | 57.5249 |
| AT1G42990 | 57.5241 | 21.8062 |
| AT1G18270 | 57.5176 | 19.0897 |
| AT1G50430 | 57.5063 | 80.8958 |
| AT4G15150 | 57.3962 | 22.229 |
| AT5G08570 | 57.3142 | 57.5927 |
| AT4G02640 | 57.2894 | 31.4658 |
| AT5G13850 | 57.2826 | 92.2712 |
| AT2G26210 | 57.2813 | 25.4561 |
| AT1G17720 | 57.2792 | 47.0917 |
| AT5G51390 | 57.279 | 9.92978 |
| AT4G27860 | 57.2694 | 83.6697 |
| AT3G02300 | 57.2587 | 41.3411 |
| AT3G20920 | 57.2371 | 38.7141 |
| AT5G40890 | 57.2303 | 97.3285 |
| AT3G16050 | 57.2197 | 48.2603 |
| AT5G59300 | 57.1868 | 44.3914 |
| AT2G43790 | 57.1377 | 72.4644 |
| AT3G61800 | 57.132 | 26.8988 |
| AT5G25880 | 57.118 | 2.17689 |
| AT1G05270 | 57.1052 | 33.3369 |
| AT3G10640 | 57.0866 | 45.1667 |
| AT1G23170 | 57.0863 | 25.5955 |
| AT5G11560 | 57.0853 | 54.1657 |
| AT3G56000 | 57.0746 | 5.07221 |
| AT3G57010 | 57.0361 | 32.3734 |
| AT2G15128 | 56.9671 | 4.84228 |
| AT4G17870 | 56.9645 | 29.5183 |
| AT2G46225 | 56.9448 | 56.5237 |
| AT1G12640 | 56.9024 | 21.3311 |
| AT5G42940 | 56.8982 | 37.4828 |
| AT4G22360 | 56.8904 | 36.2115 |
| AT5G20820 | 56.7986 | 7.52724 |
| AT3G63150 | 56.7568 | 23.4936 |
| AT5G39740 | 56.7214 | 325.196 |
| AT4G13340 | 56.7171 | 12.9646 |
| AT3G07650 | 56.6933 | 33.9356 |
| AT2G43500 | 56.6839 | 34.9864 |
| AT1G04140 | 56.6768 | 31.6527 |
| AT1G13320 | 56.6593 | 33.9982 |
| AT5G67350 | 56.6379 | 15.5192 |
| AT1G14685 | 56.6268 | 34.8533 |
| AT3G25655 | 56.6149 | 19.5074 |
| AT5G19450 | 56.5468 | 75.342 |
| AT1G64440 | 56.4451 | 38.1425 |
| AT5G13240 | 56.4304 | 44.0678 |
| AT5G08200 | 56.3996 | 28.6356 |
| AT1G10500 | 56.3849 | 59.6738 |
| AT4G01950 | 56.3639 | 26.5343 |
| AT5G64660 | 56.3152 | 20.6285 |
| AT3G45980 | 56.2935 | 94.8555 |
| AT3G07870 | 56.2917 | 9.87512 |
| AT5G65870 | 56.2767 | 44.4317 |
| AT1G80110 | 56.2628 | 51.2156 |
| AT4G20330 | 56.2504 | 45.4909 |
| AT5G45360 | 56.2068 | 33.7013 |
| AT5G47700 | 56.2019 | 122.598 |
| AT3G09735 | 56.1609 | 38.6066 |
| AT3G24350 | 56.1476 | 46.5069 |
| AT2G44770 | 56.1395 | 27.1458 |
| AT2G34070 | 56.1148 | 34.2962 |
| AT5G04770 | 56.1107 | 19.7316 |
| AT3G12350 | 56.038 | 33.7236 |
| AT4G36900 | 56.0284 | 20.8732 |
| AT1G28490 | 56.0121 | 40.4007 |
| AT1G26110 | 55.9661 | 53.0659 |
| AT4G09100 | 55.9544 | 0.302551 |
| AT2G26240 | 55.9543 | 32.9576 |
| AT3G04780 | 55.911 | 67.1009 |
| AT5G16150 | 55.908 | 52.4538 |
| AT1G18420 | 55.8597 | 1.08416 |
| AT3G03610 | 55.7913 | 48.2593 |
| AT5G16380 | 55.7806 | 16.4159 |
| AT4G39820 | 55.7166 | 26.298 |
| AT1G75540 | 55.6897 | 39.3248 |
| AT3G01472 | 55.6672 | 35.3049 |
| AT1G07040 | 55.6657 | 16.4369 |
| AT1G53400 | 55.6608 | 38.1263 |
| AT2G23770 | 55.6336 | 13.0234 |
| AT4G37260 | 55.6028 | 26.7604 |
| AT2G47390 | 55.5878 | 30.6969 |
| AT5G16130 | 55.5382 | 255.076 |
| AT5G23750 | 55.5246 | 10.7293 |
| AT5G40670 | 55.5146 | 43.5107 |
| AT3G14370 | 55.5131 | 8.93205 |
| AT5G44290 | 55.5098 | 27.9563 |
| AT3G29350 | 55.5083 | 62.047 |
| AT5G04160 | 55.5043 | 21.5547 |
| AT4G12730 | 55.5024 | 26.7495 |
| AT3G12920 | 55.4476 | 84.0191 |
| AT1G06200 | 55.4442 | 58.2901 |
| AT3G56020 | 55.3891 | 229.233 |
| AT4G29180 | 55.3506 | 0.448309 |
| AT5G12200 | 55.3213 | 25.5991 |
| AT2G21270 | 55.3184 | 54.1005 |
| AT5G40760 | 55.31 | 56.3387 |
| AT1G47278 | 55.299 | 55.6489 |
| AT5G61390 | 55.2953 | 24.1082 |
| AT5G10840 | 55.2699 | 57.5813 |
| AT5G47390 | 55.2604 | 17.4632 |
| AT5G49540 | 55.24 | 87.2434 |
| AT2G47410 | 55.2061 | 28.3729 |
| AT3G01150 | 55.1817 | 37.3114 |
| AT5G64410 | 55.1657 | 45.9269 |
| AT5G65910 | 55.12 | 49.4377 |
| AT5G27450 | 55.1185 | 35.5185 |
| AT2G36890 | 55.0995 | 18.9423 |
| AT1G64195 | 55.055 | 19.8464 |
| AT5G12230 | 55.0311 | 49.3932 |
| AT3G48560 | 55.0149 | 59.886 |
| AT3G12502 | 55.0065 | 5.80044 |
| AT1G12610 | 55.0001 | 11.2748 |
| AT4G16150 | 54.9934 | 37.7188 |
| AT3G51770 | 54.9882 | 19.6388 |
| AT5G26660 | 54.9783 | 5.93874 |
| AT3G49010 | 54.9471 | 191.61 |
| AT4G33985 | 54.9466 | 20.2031 |
| AT3G50500 | 54.9278 | 29.6841 |
| AT4G20850 | 54.9093 | 77.5846 |
| AT1G70810 | 54.8635 | 7.8275 |
| AT2G19740 | 54.7917 | 192.638 |
| AT3G51370 | 54.7843 | 49.4859 |
| AT1G60010 | 54.7641 | 13.2725 |
| AT1G58520 | 54.7524 | 20.0112 |
| AT5G17640 | 54.7388 | 30.6982 |
| AT5G47240 | 54.7061 | 14.9332 |
| AT4G31170 | 54.704 | 35.6901 |
| AT5G57990 | 54.7002 | 53.1608 |
| AT2G05260 | 54.638 | 17.3988 |
| AT3G56760 | 54.6139 | 14.6612 |
| AT1G78310 | 54.5883 | 11.0456 |
| AT1G72680 | 54.5816 | 89.7053 |
| AT1G64660 | 54.5661 | 23.5609 |
| AT5G09590 | 54.5609 | 53.0694 |
| AT5G14540 | 54.5585 | 23.9687 |
| AT4G01900 | 54.5408 | 23.7929 |
| AT4G28640 | 54.5393 | 10.6777 |
| AT1G52050 | 54.5289 | 498.896 |
| AT3G61180 | 54.5134 | 15.8121 |
| AT2G26480 | 54.487 | 8.38316 |
| AT3G11770 | 54.485 | 36.6015 |
| AT1G48850 | 54.4471 | 43.3537 |
| AT2G25670 | 54.4301 | 70.765 |
| AT2G20010 | 54.4102 | 19.893 |
| AT2G17520 | 54.4026 | 27.1067 |
| AT5G01810 | 54.3986 | 27.2358 |
| AT2G41880 | 54.3491 | 37.4166 |
| AT3G25930 | 54.343 | 7.70837 |
| AT2G33370 | 54.3103 | 173.074 |
| AT3G17100 | 54.2598 | 48.1301 |
| AT3G53500 | 54.2435 | 51.4831 |
| AT5G65480 | 54.1482 | 27.0586 |
| AT3G12500 | 54.134 | 5.00602 |
| AT5G01450 | 54.1153 | 17.7375 |
| AT3G48880 | 54.1083 | 24.7575 |
| AT4G19045 | 54.0514 | 14.5491 |
| AT2G27000 | 54.0366 | 20.1544 |
| AT1G09130 | 54.0017 | 32.3467 |
| AT3G27820 | 53.9594 | 30.3944 |
| AT1G77440 | 53.9421 | 50.6553 |
| AT2G02760 | 53.934 | 51.4121 |
| AT1G76090 | 53.9295 | 35.9896 |
| AT1G06002 | 53.924 | 21.2371 |
| AT5G42250 | 53.901 | 19.7122 |
| AT3G57750 | 53.9008 | 41.4493 |
| AT4G33980 | 53.8921 | 22.3509 |
| AT5G42560 | 53.8559 | 31.531 |
| AT1G04300 | 53.85 | 32.0016 |
| AT4G29735 | 53.8491 | 73.2122 |
| AT5G58110 | 53.8482 | 104.431 |
| AT3G07160 | 53.8428 | 54.4017 |
| AT5G42950 | 53.8347 | 41.9125 |
| AT1G62320 | 53.8165 | 2.38029 |
| AT5G20910 | 53.8047 | 24.1218 |
| AT1G17380 | 53.7997 | 56.0741 |
| AT2G38840 | 53.7518 | 25.9669 |
| AT5G64880 | 53.7357 | 27.2391 |
| AT4G02450 | 53.7197 | 119.4 |
| AT1G04200 | 53.7191 | 30.9957 |
| AT5G04840 | 53.7091 | 21.4757 |
| AT4G25690 | 53.7049 | 46.7637 |
| AT2G31680 | 53.6824 | 22.3527 |
| AT4G26750 | 53.6248 | 22.4383 |
| AT5G54960 | 53.561 | 29.8293 |
| AT1G07820 | 53.5509 | 117.344 |
| AT3G59850 | 53.4824 | 123.944 |
| AT5G58420 | 53.4745 | 228.385 |
| AT4G39540 | 53.4598 | 17.5885 |
| AT1G64850 | 53.4425 | 34.9272 |
| AT3G62650 | 53.4357 | 111.545 |
| AT4G32285 | 53.398 | 49.6094 |
| AT5G25760 | 53.3978 | 41.9902 |
| AT5G55100 | 53.3693 | 32.5778 |
| AT5G40590 | 53.3652 | 37.8681 |
| AT2G37410 | 53.3265 | 61.7542 |
| AT2G01820 | 53.323 | 25.5956 |
| AT1G78000 | 53.2565 | 73.6277 |
| AT3G07810 | 53.2542 | 28.2271 |
| AT5G10260 | 53.2358 | 27.5124 |
| AT3G57400 | 53.2336 | 19.5987 |
| AT5G63160 | 53.2257 | 16.2543 |
| AT5G03730 | 53.2246 | 35.7185 |
| AT1G68370 | 53.1981 | 35.8298 |
| AT1G29760 | 53.1879 | 24.4435 |
| AT5G16370 | 53.1633 | 13.9336 |
| AT2G14835 | 53.1623 | 31.5465 |
| AT3G09000 | 53.1497 | 10.8236 |
| AT2G40890 | 53.1148 | 17.3933 |
| AT5G47220 | 53.0766 | 24.4988 |
| AT2G38230 | 53.0601 | 22.4962 |
| AT5G06370 | 53.0583 | 38.2866 |
| AT4G39690 | 53.016 | 35.2699 |
| AT3G02990 | 52.9976 | 56.0258 |
| AT4G35360 | 52.9816 | 41.5184 |
| AT5G01270 | 52.9658 | 30.0099 |
| AT1G08090 | 52.9332 | 0.266549 |
| AT3G14770 | 52.9303 | 23.2679 |
| AT2G25240 | 52.9154 | 1.59392 |
| AT3G14075 | 52.9087 | 30.596 |
| AT4G14160 | 52.8522 | 34.7316 |
| AT2G37480 | 52.8477 | 26.3624 |
| AT4G17790 | 52.822 | 57.4108 |
| AT3G19420 | 52.81 | 65.8772 |
| AT2G34940 | 52.7697 | 4.5124 |
| AT5G13970 | 52.7245 | 45.3283 |
| AT5G13150 | 52.697 | 2.7557 |
| AT5G63940 | 52.6358 | 41.0747 |
| AT1G29020 | 52.5806 | 1.50385 |
| AT3G05050 | 52.5673 | 21.3469 |
| AT1G77130 | 52.4954 | 23.6296 |
| AT5G59840 | 52.4583 | 38.3788 |
| AT2G02040 | 52.4565 | 32.3419 |
| AT2G24170 | 52.4348 | 24.573 |
| AT5G24650 | 52.4081 | 45.9591 |
| AT1G76390 | 52.3731 | 42.8134 |
| AT4G01120 | 52.3565 | 9.89213 |
| AT4G24390 | 52.3408 | 23.7111 |
| AT5G40540 | 52.3322 | 33.5235 |
| AT4G08180 | 52.3103 | 19.4261 |
| AT4G17895 | 52.3061 | 38.7296 |
| AT1G79720 | 52.3006 | 42.3779 |
| AT5G01280 | 52.2871 | 0.534523 |
| AT5G03345 | 52.2671 | 45.0722 |
| AT4G37530 | 52.2388 | 15.299 |
| AT5G66780 | 52.2296 | 61.4477 |
| AT5G15930 | 52.2198 | 27.7153 |
| AT1G17360 | 52.1901 | 51.3739 |
| AT1G30400 | 52.1799 | 66.8059 |
| AT3G01590 | 52.1719 | 41.3952 |
| AT4G32140 | 52.1707 | 44.2649 |
| AT2G40730 | 52.1473 | 30.5046 |
| AT1G72800 | 52.1081 | 7.61144 |
| AT5G47830 | 52.1028 | 42.6065 |
| AT5G59720 | 52.0794 | 56.9608 |
| AT1G75230 | 52.0697 | 19.7808 |
| AT1G19440 | 52.0617 | 13.9648 |
| AT3G15518 | 52.0407 | 8.70883 |
| AT3G02340 | 52.0351 | 23.3717 |
| AT3G50930 | 52.0137 | 45.8326 |
| AT1G67360 | 51.9789 | 63.7355 |
| AT4G37910 | 51.9552 | 75.0989 |
| AT1G01300 | 51.9485 | 26.0193 |
| AT1G12710 | 51.9472 | 9.70666 |
| AT3G07470 | 51.8664 | 16.3284 |
| AT1G62886 | 51.8663 | 6.46637 |
| AT3G16760 | 51.8636 | 21.2461 |
| AT5G42420 | 51.8589 | 45.6561 |
| AT2G03760 | 51.8474 | 39.4518 |
| AT1G76700 | 51.8222 | 31.4098 |
| AT2G18730 | 51.7958 | 24.3484 |
| AT3G15260 | 51.758 | 32.8953 |
| AT1G28190 | 51.7511 | 69.0144 |
| AT5G66930 | 51.751 | 26.5946 |
| AT5G23630 | 51.7381 | 31.5223 |
| AT1G75600 | 51.7313 | 40.7535 |
| AT1G20630 | 51.7123 | 27.6782 |
| AT3G62580 | 51.6964 | 42.5304 |
| AT3G09270 | 51.6758 | 113.559 |
| AT1G18910 | 51.6658 | 20.1109 |
| AT4G14300 | 51.6213 | 43.8105 |
| AT3G15980 | 51.62 | 44.0059 |
| AT1G48920 | 51.5798 | 165.15 |
| AT3G56360 | 51.5737 | 24.3843 |
| AT3G15410 | 51.4963 | 38.4051 |
| AT1G21910 | 51.4891 | 35.5985 |
| AT5G52660 | 51.4391 | 29.1127 |
| AT1G23030 | 51.4383 | 9.41957 |
| AT1G80360 | 51.4259 | 22.6069 |
| AT5G22070 | 51.4224 | 19.6846 |
| AT1G19230 | 51.3866 | 1.65536 |
| AT2G32730 | 51.3525 | 67.5227 |
| AT3G01220 | 51.3422 | 55.6649 |
| AT1G09690 | 51.315 | 180.012 |
| AT4G35630 | 51.2711 | 50.8439 |
| AT5G25640 | 51.2661 | 8.17973 |
| AT2G13560 | 51.2424 | 63.4254 |
| AT5G52980 | 51.2032 | 32.9594 |
| AT4G30060 | 51.1928 | 19.5875 |
| AT2G40205 | 51.184 | 136.443 |
| AT5G58710 | 51.1823 | 88.1446 |
| AT1G59610 | 51.1761 | 38.8185 |
| AT4G22080 | 51.1689 | 0.0619022 |
| AT1G21680 | 51.121 | 26.4708 |
| AT1G61150 | 51.0837 | 33.6785 |
| AT2G24103 | 51.0591 | 1.38261 |
| AT1G56600 | 51.0532 | 51.6896 |
| AT3G58030 | 50.9898 | 36.4194 |
| AT1G07670 | 50.9827 | 44.805 |
| AT3G03860 | 50.9791 | 40.317 |
| AT1G30510 | 50.9755 | 45.1785 |
| AT1G14780 | 50.9473 | 21.6532 |
| AT5G52530 | 50.9324 | 30.4578 |
| AT4G26400 | 50.9217 | 33.1926 |
| AT4G40050 | 50.9067 | 43.6179 |
| AT2G37270 | 50.8714 | 162.403 |
| AT1G65270 | 50.847 | 46.6125 |
| AT2G01680 | 50.8468 | 18.0227 |
| AT1G50660 | 50.7916 | 43.1674 |
| AT4G38600 | 50.7406 | 41.0747 |
| AT5G20520 | 50.7315 | 31.9542 |
| AT1G34760 | 50.7197 | 0.636707 |
| AT4G39160 | 50.7053 | 11.7527 |
| AT3G12570 | 50.6668 | 37.583 |
| AT5G18310 | 50.6607 | 79.885 |
| AT5G41560 | 50.5804 | 35.7784 |
| AT5G02870 | 50.577 | 175.168 |
| AT5G03300 | 50.5698 | 106.678 |
| AT5G67490 | 50.5577 | 28.4398 |
| AT2G43090 | 50.5519 | 73.0397 |
| AT5G41950 | 50.5508 | 45.5647 |
| AT1G04160 | 50.5496 | 12.575 |
| AT3G13275 | 50.544 | 22.0179 |
| AT1G68490 | 50.5368 | 38.7298 |
| AT5G66210 | 50.5362 | 76.3214 |
| AT4G16190 | 50.5087 | 70.578 |
| AT3G24120 | 50.4577 | 36.9122 |
| AT3G16270 | 50.4154 | 29.3517 |
| AT5G52210 | 50.4098 | 35.5607 |
| AT3G05670 | 50.3754 | 37.393 |
| AT5G49760 | 50.3669 | 10.7752 |
| AT3G49390 | 50.3511 | 26.3215 |
| AT3G16857 | 50.3459 | 31.378 |
| AT5G41670 | 50.3431 | 46.8182 |
| AT1G13350 | 50.3157 | 23.8079 |
| AT1G53760 | 50.3082 | 38.4675 |
| AT1G52720 | 50.27 | 38.2954 |
| AT1G76010 | 50.2505 | 49.5867 |
| AT2G06025 | 50.2503 | 30.807 |
| AT1G47480 | 50.2463 | 6.95046 |
| AT3G01120 | 50.2272 | 59.939 |
| AT5G06560 | 50.2131 | 28.0541 |
| AT2G17705 | 50.1857 | 27.5245 |
| AT1G59820 | 50.1853 | 26.2071 |
| AT4G39390 | 50.18 | 28.7221 |
| AT4G39840 | 50.1749 | 72.3456 |
| AT4G27000 | 50.1687 | 59.2152 |
| AT2G29550 | 50.1578 | 105.504 |
| AT1G77500 | 50.1367 | 16.7365 |
| AT4G12250 | 50.134 | 23.4041 |
| AT2G17850 | 50.133 | 27.7166 |
| AT5G57610 | 50.1205 | 23.0448 |
| AT1G74453 | 50.1187 | 11.9421 |
| AT1G75590 | 50.1066 | 21.0077 |
| AT3G05165 | 50.1055 | 29.2531 |
| AT4G21810 | 50.0967 | 47.5949 |
| AT4G35080 | 50.096 | 25.6666 |
| AT4G23040 | 50.0832 | 25.1834 |
| AT1G61690 | 50.0715 | 27.1069 |
| AT3G01090 | 50.0675 | 23.3842 |
| AT2G36620 | 50.0633 | 152.889 |
| AT4G27654 | 50.0616 | 12.866 |
| AT5G63290 | 50.0258 | 23.1257 |
| AT5G55896.1 | 50.0176 | 29.1849 |
| AT3G52420 | 50.0093 | 21.2155 |
| AT1G34030 | 49.9913 | 175.238 |
| AT4G15545 | 49.9784 | 10.4892 |
| AT5G45010 | 49.9729 | 50.1796 |
| AT2G44360 | 49.954 | 33.6982 |
| AT2G30990 | 49.937 | 49.4284 |
| AT1G04700 | 49.9354 | 1.67292 |
| AT4G29850 | 49.8956 | 21.9222 |
| AT4G30460 | 49.893 | 2.00971 |
| AT5G57070 | 49.8687 | 3.78625 |
| AT5G54800 | 49.8499 | 26.5666 |
| AT2G29700 | 49.8381 | 23.4563 |
| AT2G25490 | 49.8309 | 35.5949 |
| AT2G37555 | 49.8079 | 29.6895 |
| AT2G47490 | 49.8046 | 31.1201 |
| AT3G50950 | 49.7832 | 20.4758 |
| AT1G14890 | 49.776 | 34.9398 |
| AT1G02120 | 49.7745 | 37.5711 |
| AT4G25200 | 49.7737 | 48.611 |
| AT2G03340 | 49.7524 | 18.1149 |
| AT5G63970 | 49.7303 | 56.5885 |
| AT1G30450 | 49.7299 | 25.8357 |
| AT1G30270 | 49.7212 | 24.7296 |
| AT4G16210 | 49.6943 | 26.7917 |
| AT3G14050 | 49.6886 | 76.5686 |
| AT5G06865 | 49.6787 | 144.851 |
| AT4G35500 | 49.6345 | 17.482 |
| AT1G03140 | 49.6139 | 38.5175 |
| AT4G39640 | 49.6067 | 20.0516 |
| AT2G30260 | 49.5847 | 62.4158 |
| AT5G63640 | 49.58 | 43.2615 |
| AT5G66880 | 49.5793 | 53.8818 |
| AT5G09880 | 49.5783 | 43.04 |
| AT4G29440 | 49.5507 | 24.4934 |
| AT4G17640 | 49.5281 | 34.4493 |
| AT2G37440 | 49.5012 | 5.07101 |
| AT3G25730 | 49.4997 | 58.151 |
| AT2G02010 | 49.4969 | 26.7116 |
| AT5G56140 | 49.4801 | 31.1134 |
| AT5G57887 | 49.4774 | 18.6155 |
| AT1G78150 | 49.4532 | 108.304 |
| AT2G20520 | 49.4497 | 1.87766 |
| AT4G17270 | 49.4408 | 40.5667 |
| AT4G02880 | 49.4304 | 28.2508 |
| AT1G41830 | 49.4095 | 108.554 |
| AT2G46520 | 49.3935 | 45.2028 |
| AT2G43060 | 49.3807 | 78.2651 |
| AT5G23340 | 49.3634 | 29.8539 |
| AT1G52660 | 49.3632 | 0.159861 |
| AT5G35940 | 49.3543 | 144.916 |
| AT1G75370 | 49.3119 | 38.6077 |
| AT1G14570 | 49.2992 | 32.2978 |
| AT3G48940 | 49.2343 | 1.64026 |
| AT5G07920 | 49.2252 | 17.1494 |
| AT5G57490 | 49.2043 | 30.7947 |
| AT1G25682 | 49.196 | 37.0212 |
| AT5G51880 | 49.1936 | 35.5616 |
| AT4G14350 | 49.189 | 31.7376 |
| AT1G26880 | 49.1634 | 149.396 |
| AT1G09630 | 49.1448 | 59.5604 |
| AT5G08130 | 49.1364 | 57.5612 |
| AT2G37340 | 49.1269 | 35.7129 |
| AT5G11920 | 49.1181 | 5.8747 |
| AT5G13820 | 49.0942 | 35.2989 |
| AT2G14878 | 49.0804 | 61.2916 |
| AT4G33240 | 49.0644 | 27.6788 |
| AT3G11030 | 49.0593 | 18.8426 |
| AT1G48160 | 49.0436 | 40.4138 |
| AT3G29160 | 49.009 | 29.086 |
| AT3G45010 | 49.003 | 131.181 |
| AT1G14370 | 48.9812 | 54.669 |
| AT1G01725 | 48.9771 | 17.93 |
| AT3G15440 | 48.957 | 10.3117 |
| AT2G40970 | 48.9457 | 17.7576 |
| AT1G27910 | 48.9338 | 14.3056 |
| AT3G13330 | 48.9121 | 48.2699 |
| AT3G57230 | 48.8883 | 13.727 |
| AT5G40770 | 48.873 | 60.1007 |
| AT5G58040 | 48.8647 | 34.6359 |
| AT2G32850 | 48.8521 | 21.5533 |
| AT1G34510 | 48.8487 | 0.01921 |
| AT2G43070 | 48.8138 | 29.1701 |
| AT2G32235 | 48.8014 | 22.9967 |
| AT4G19003 | 48.7775 | 27.5054 |
| AT1G07080 | 48.7681 | 28.1214 |
| AT5G65030 | 48.7309 | 25.3283 |
| AT4G32650 | 48.6848 | 26.4212 |
| AT2G21410 | 48.671 | 32.9637 |
| AT1G51140 | 48.6661 | 16.5139 |
| AT4G13520 | 48.659 | 84.1445 |
| AT5G19780 | 48.6432 | 63.1775 |
| AT2G39350 | 48.6393 | 119.995 |
| AT3G59990 | 48.6386 | 55.2762 |
| AT3G04500 | 48.619 | 39.1316 |
| AT3G02420 | 48.5787 | 41.6056 |
| AT5G46760 | 48.5786 | 31.1864 |
| AT2G26230 | 48.5737 | 32.4147 |
| AT2G32020 | 48.5725 | 28.9024 |
| AT3G54950 | 48.5347 | 5.48234 |
| AT4G32850 | 48.5162 | 27.2281 |
| AT3G61130 | 48.4958 | 41.371 |
| AT3G18430 | 48.4826 | 44.8058 |
| AT2G46900 | 48.4588 | 26.0386 |
| AT5G20010 | 48.4566 | 38.0737 |
| AT1G49310 | 48.4549 | 2.48173 |
| AT1G11020 | 48.4115 | 20.5031 |
| AT1G20823 | 48.4016 | 33.5665 |
| AT2G44520 | 48.3944 | 26.1682 |
| AT5G63490 | 48.3892 | 27.9003 |
| AT3G26670 | 48.3805 | 32.4272 |
| AT4G31720 | 48.379 | 36.3369 |
| AT1G57765 | 48.3628 | 41.2555 |
| AT3G01040 | 48.3259 | 18.9942 |
| AT3G12030 | 48.3214 | 20.0925 |
| AT5G61380 | 48.3185 | 33.6554 |
| AT3G09980 | 48.3164 | 61.7958 |
| AT5G20350 | 48.3074 | 27.0793 |
| AT2G01970 | 48.2831 | 42.4679 |
| AT5G12040 | 48.2394 | 32.1329 |
| AT2G04350 | 48.2383 | 26.0969 |
| AT1G09850 | 48.2332 | 30.9473 |
| AT1G06550 | 48.2293 | 24.8496 |
| AT5G64070 | 48.2265 | 31.5422 |
| AT5G55110 | 48.2223 | 186.738 |
| AT5G03740 | 48.2036 | 55.662 |
| AT5G09680 | 48.1986 | 42.1972 |
| AT2G40270 | 48.1885 | 48.5668 |
| AT2G32700 | 48.1739 | 36.3172 |
| AT2G37975 | 48.1492 | 28.9931 |
| AT3G07940 | 48.1482 | 4.57929 |
| AT1G32860 | 48.1418 | 59.1149 |
| AT5G09920 | 48.137 | 54.6244 |
| AT3G54990 | 48.1095 | 8.4719 |
| AT1G71860 | 48.1021 | 46.8507 |
| AT1G28240 | 48.1016 | 19.7432 |
| AT5G49665 | 48.0897 | 35.9738 |
| AT3G54870 | 48.081 | 0.745398 |
| AT1G14330 | 48.0693 | 31.3587 |
| AT3G28450 | 48.0473 | 26.0856 |
| AT1G65430 | 48.0461 | 22.1443 |
| AT1G76970 | 47.9952 | 32.542 |
| AT5G60620 | 47.9934 | 39.8264 |
| AT1G49950 | 47.9928 | 26.1033 |
| AT2G47260 | 47.9611 | 49.7187 |
| AT1G17420 | 47.9487 | 54.8989 |
| AT1G60070 | 47.9055 | 33.0908 |
| AT3G09710 | 47.9049 | 5.68657 |
| AT1G24440 | 47.8712 | 20.4145 |
| AT5G35320 | 47.8588 | 50.293 |
| AT1G15880 | 47.7997 | 42.7547 |
| AT1G61360 | 47.7828 | 17.5205 |
| AT3G05320 | 47.7753 | 20.9784 |
| AT2G45710 | 47.7653 | 63.5307 |
| AT5G02770 | 47.7476 | 61.8117 |
| AT1G03350 | 47.7394 | 35.2451 |
| AT3G16630 | 47.7143 | 32.0114 |
| AT3G08760 | 47.714 | 33.5858 |
| AT1G18260 | 47.6999 | 27.8588 |
| AT5G35160 | 47.6881 | 38.0929 |
| AT1G20140 | 47.6784 | 22.6031 |
| AT3G53150 | 47.678 | 6.00347 |
| AT3G17820 | 47.6736 | 149.648 |
| AT3G14290 | 47.6404 | 78.9743 |
| AT3G53520 | 47.6369 | 42.222 |
| AT4G26410 | 47.6039 | 32.0679 |
| AT2G15695 | 47.5502 | 25.2477 |
| AT1G50890 | 47.4969 | 4.22426 |
| AT1G75180 | 47.4792 | 45.0705 |
| AT3G45970 | 47.4784 | 24.3032 |
| AT2G23320 | 47.4538 | 38.7321 |
| AT5G62930 | 47.4475 | 34.1172 |
| AT3G63070 | 47.4339 | 28.2852 |
| AT2G02570 | 47.4291 | 48.0789 |
| AT1G07795 | 47.4163 | 0.554259 |
| AT2G24330 | 47.4154 | 8.73636 |
| AT1G26910 | 47.3595 | 49.5286 |
| AT1G08510 | 47.3342 | 36.6493 |
| AT5G48175 | 47.3048 | 55.3936 |
| AT1G03740 | 47.2938 | 37.3576 |
| AT3G62360 | 47.2906 | 53.6614 |
| AT1G55450 | 47.2898 | 26.5934 |
| AT3G53403 | 47.2839 | 1.21982 |
| AT1G20770 | 47.272 | 30.8283 |
| AT1G02900 | 47.2155 | 18.3495 |
| AT5G22120 | 47.2143 | 29.559 |
| AT3G17940 | 47.2139 | 37.3435 |
| AT1G12970 | 47.1973 | 23.2776 |
| AT3G55270 | 47.1958 | 39.8499 |
| AT2G26380 | 47.1538 | 40.4391 |
| AT5G04460 | 47.1495 | 21.6808 |
| AT5G19820 | 47.1484 | 34.8608 |
| AT3G23690 | 47.1281 | 46.8074 |
| AT3G13910 | 47.1269 | 15.3329 |
| AT1G30650 | 47.0529 | 9.182 |
| AT3G44340 | 47.0516 | 47.0096 |
| AT5G55970 | 47.0115 | 9.74342 |
| AT4G01400 | 47.0015 | 41.3434 |
| AT5G07310 | 46.9955 | 28.9907 |
| AT5G53160 | 46.9672 | 45.8881 |
| AT1G23140 | 46.8932 | 11.2883 |
| AT3G55740 | 46.8865 | 44.7725 |
| AT5G23155 | 46.8837 | 2.85512 |
| AT5G06390 | 46.8757 | 16.7675 |
| AT2G44430 | 46.8532 | 24.299 |
| AT1G20980 | 46.8522 | 42.1627 |
| AT2G15570 | 46.8267 | 37.5515 |
| AT1G16010 | 46.809 | 35.0537 |
| AT1G79440 | 46.7667 | 84.4819 |
| AT2G18876 | 46.7595 | 9.31223 |
| AT2G44120 | 46.757 | 175.861 |
| AT5G28830 | 46.7518 | 38.9864 |
| AT5G03700 | 46.741 | 43.0848 |
| AT1G22830 | 46.7237 | 7.80635 |
| AT5G64230 | 46.7093 | 26.7139 |
| AT5G63780 | 46.6983 | 12.9448 |
| AT3G54640 | 46.6621 | 48.3347 |
| AT5G38940 | 46.6574 | 6.59934 |
| AT5G57740 | 46.6273 | 7.55527 |
| AT2G32980 | 46.5964 | 28.6794 |
| AT5G05850 | 46.5866 | 20.971 |
| AT3G26440 | 46.5842 | 31.4739 |
| AT4G37990 | 46.579 | 10.4301 |
| AT2G26570 | 46.5513 | 8.36438 |
| AT5G43650 | 46.5183 | 59.19 |
| AT1G80830 | 46.4711 | 70.84 |
| AT1G53590 | 46.4609 | 22.1611 |
| AT3G09570 | 46.4071 | 25.0681 |
| AT2G40940 | 46.3922 | 71.2027 |
| AT3G16110 | 46.3323 | 37.3157 |
| AT1G76185 | 46.3039 | 16.7009 |
| AT5G52510 | 46.2976 | 24.1146 |
| AT5G53140 | 46.2911 | 57.2365 |
| AT1G26960 | 46.2893 | 22.3584 |
| AT1G21630 | 46.2746 | 29.0647 |
| AT3G57070 | 46.2669 | 24.4292 |
| AT1G27200 | 46.2442 | 16.8087 |
| AT1G59910 | 46.2268 | 23.9564 |
| AT3G11420 | 46.2253 | 20.3288 |
| AT1G75800 | 46.2034 | 48.8282 |
| AT4G35890 | 46.2014 | 30.8901 |
| AT1G44350 | 46.1976 | 49.0472 |
| AT5G28540 | 46.1912 | 50.2323 |
| AT3G54540 | 46.1862 | 45.1338 |
| AT4G19150 | 46.1761 | 37.2143 |
| AT2G34630 | 46.1679 | 35.0002 |
| AT1G28510 | 46.1376 | 32.591 |
| AT1G54170 | 46.1346 | 38.465 |
| AT2G46780 | 46.134 | 25.9313 |
| AT3G11810 | 46.1199 | 26.6847 |
| AT5G42010 | 46.0646 | 20.6466 |
| AT1G48140 | 46.0562 | 27.8927 |
| AT2G43410 | 45.9981 | 21.6278 |
| AT5G07960 | 45.9969 | 31.7964 |
| AT5G38560 | 45.9813 | 31.8734 |
| AT1G11380 | 45.9811 | 9.28869 |
| AT4G32390 | 45.9676 | 49.6026 |
| AT1G11390 | 45.9659 | 26.4867 |
| AT3G04810 | 45.9456 | 30.3615 |
| AT3G60200 | 45.9345 | 16.5708 |
| AT1G07310 | 45.9341 | 18.8343 |
| AT4G11660 | 45.9252 | 33.1242 |
| AT1G72450 | 45.9146 | 22.3687 |
| AT3G12560 | 45.9051 | 29.0969 |
| AT5G14790 | 45.886 | 29.7587 |
| AT3G57480 | 45.8663 | 16.8966 |
| AT5G13310 | 45.8398 | 24.6342 |
| AT5G08560 | 45.8261 | 35.1855 |
| AT2G24490 | 45.8088 | 63.8718 |
| AT3G51890 | 45.8057 | 26.7154 |
| AT5G53530 | 45.8027 | 26.428 |
| AT1G66800 | 45.8008 | 44.2652 |
| AT3G14600 | 45.7158 | 197.444 |
| AT3G61980 | 45.6948 | 33.1519 |
| AT1G09950 | 45.6906 | 18.5278 |
| AT5G10820 | 45.6676 | 43.6351 |
| AT2G47130 | 45.6521 | 14.8097 |
| AT4G29670 | 45.6165 | 22.325 |
| AT4G19120 | 45.6121 | 61.5536 |
| AT2G16860 | 45.599 | 46.0225 |
| AT5G43330 | 45.5838 | 10.4805 |
| AT4G28140 | 45.5681 | 22.3982 |
| AT3G19200 | 45.5475 | 107.578 |
| AT2G32410 | 45.5466 | 35.4088 |
| AT5G42290 | 45.5262 | 10.4498 |
| AT1G71820 | 45.4973 | 26.5853 |
| AT3G10740 | 45.4211 | 22.8521 |
| AT4G19140 | 45.4088 | 37.5784 |
| AT5G40580 | 45.3926 | 63.1592 |
| AT3G14362 | 45.3817 | 17.0997 |
| AT4G22620 | 45.3796 | 3.64854 |
| AT1G16470 | 45.3742 | 73.6429 |
| AT5G38530 | 45.3491 | 60.6362 |
| AT3G26400 | 45.3434 | 24.9578 |
| AT5G55060 | 45.3203 | 15.8217 |
| AT5G52550 | 45.3083 | 35.3125 |
| AT1G16370 | 45.3011 | 34.6304 |
| AT3G01513 | 45.2728 | 33.1943 |
| AT5G43100 | 45.2704 | 24.7361 |
| AT2G17880 | 45.2704 | 8.16076 |
| AT5G16730 | 45.2606 | 41.8381 |
| AT2G27050 | 45.2604 | 35.7128 |
| AT1G79210 | 45.2426 | 57.5829 |
| AT3G23870 | 45.1662 | 30.6023 |
| AT1G50370 | 45.1578 | 43.8927 |
| AT5G59970 | 45.1446 | 65.0684 |
| AT2G39650 | 45.1427 | 26.8338 |
| AT5G59850 | 45.1323 | 226.554 |
| AT4G36130 | 45.1305 | 130.075 |
| AT5G05670 | 45.0749 | 43.356 |
| AT2G39800 | 45.0652 | 81.7544 |
| AT2G25310 | 45.0377 | 42.29 |
| AT5G50900 | 45.0341 | 46.9735 |
| AT2G28570 | 45.033 | 36.9341 |
| AT3G50210 | 44.9671 | 27.2388 |
| AT1G11100 | 44.9633 | 34.7064 |
| AT5G19330 | 44.9489 | 32.838 |
| AT1G02270 | 44.9394 | 38.4264 |
| AT5G61450 | 44.9083 | 30.9206 |
| AT5G39610 | 44.8862 | 36.6615 |
| AT1G30000 | 44.8783 | 29.2817 |
| AT2G40880 | 44.87 | 19.3769 |
| AT2G24050 | 44.8467 | 25.478 |
| AT1G76260 | 44.8419 | 27.9397 |
| AT1G75680 | 44.8235 | 63.0826 |
| AT1G76670 | 44.8078 | 79.8273 |
| AT5G12480 | 44.7348 | 59.5064 |
| AT5G62360 | 44.7045 | 37.5227 |
| AT4G18040 | 44.6788 | 52.5937 |
| AT2G22670 | 44.6549 | 73.8767 |
| AT1G78260 | 44.6488 | 4.73431 |
| AT3G50800 | 44.6428 | 21.8559 |
| AT3G07720 | 44.6267 | 46.4987 |
| AT1G10900 | 44.6163 | 14.609 |
| AT1G70170 | 44.6145 | 0.961701 |
| AT1G52890 | 44.593 | 50.4731 |
| AT3G07370 | 44.5776 | 42.0724 |
| AT1G30070 | 44.5176 | 78.7834 |
| AT3G53160 | 44.4914 | 28.6121 |
| AT4G03260 | 44.4745 | 32.0575 |
| AT5G16650 | 44.4742 | 47.5468 |
| AT1G04830 | 44.4685 | 23.0422 |
| AT3G04080 | 44.454 | 15.9001 |
| AT5G53120 | 44.4477 | 31.0365 |
| AT2G16710 | 44.4398 | 25.8174 |
| AT3G56230 | 44.4348 | 10.7348 |
| AT1G07960 | 44.4189 | 43.4111 |
| AT5G02100 | 44.405 | 19.616 |
| AT2G01650 | 44.3746 | 21.9838 |
| AT1G21920 | 44.3217 | 28.6226 |
| AT1G55300 | 44.3204 | 21.9681 |
| AT1G54950 | 44.3117 | 0.720504 |
| AT2G30620 | 44.2774 | 43.4682 |
| AT1G18480 | 44.2622 | 34.9191 |
| AT5G41800 | 44.2582 | 24.4991 |
| AT5G54430 | 44.2371 | 73.1042 |
| AT2G20930 | 44.2357 | 29.9933 |
| AT5G24105 | 44.2348 | 4.92272 |
| AT5G55160 | 44.2298 | 66.9284 |
| AT2G27710 | 44.2225 | 104.141 |
| AT4G34370 | 44.2154 | 25.7686 |
| AT1G07870 | 44.214 | 24.2665 |
| AT2G45920 | 44.2043 | 21.9447 |
| AT5G59210 | 44.2015 | 31.803 |
| AT4G39150 | 44.1967 | 47.3519 |
| AT1G77840 | 44.1737 | 46.4756 |
| AT1G64105 | 44.1368 | 17.1847 |
| AT5G01960 | 44.1262 | 33.5676 |
| AT3G05830 | 44.1042 | 46.8225 |
| AT1G51690 | 44.1019 | 36.5376 |
| AT2G43490 | 44.0976 | 16.3244 |
| AT1G22500 | 44.0959 | 5.34257 |
| AT1G48110 | 44.0896 | 29.0051 |
| AT5G44820 | 44.0843 | 8.63482 |
| AT2G17790 | 44.064 | 27.0459 |
| AT5G11280 | 44.0333 | 27.7506 |
| AT2G18840 | 44.031 | 17.5447 |
| AT2G27390 | 44.0217 | 13.7384 |
| AT1G74210 | 43.9993 | 23.8155 |
| AT1G30755 | 43.9409 | 25.9072 |
| AT5G66920 | 43.9372 | 93.5 |
| AT3G05230 | 43.9255 | 28.4296 |
| AT4G09830 | 43.9228 | 24.8214 |
| AT1G66480 | 43.898 | 34.8408 |
| AT1G63090 | 43.8885 | 30.2461 |
| AT5G15260 | 43.8841 | 25.353 |
| AT4G25520 | 43.8684 | 40.7558 |
| AT1G74010 | 43.8429 | 62.8859 |
| AT3G51260 | 43.8365 | 61.513 |
| AT3G54770 | 43.7494 | 14.2642 |
| AT3G14850 | 43.7417 | 2.83187 |
| AT1G70300 | 43.7267 | 27.6522 |
| AT3G05970 | 43.6852 | 30.6266 |
| AT4G14030 | 43.6777 | 62.6978 |
| AT3G19260 | 43.6767 | 21.5266 |
| AT4G24110 | 43.6277 | 36.5733 |
| AT1G21360 | 43.6204 | 1.34333 |
| AT1G22510 | 43.6078 | 56.584 |
| AT5G57330 | 43.5796 | 62.9825 |
| AT4G33080 | 43.5762 | 31.2287 |
| AT3G15300 | 43.5715 | 13.9914 |
| AT4G02350 | 43.5701 | 24.2339 |
| AT4G27680 | 43.5697 | 35.5508 |
| AT4G02840 | 43.5676 | 96.2675 |
| AT4G29010 | 43.5645 | 43.7086 |
| AT2G45300 | 43.5642 | 30.2344 |
| AT1G05300 | 43.5397 | 15.7696 |
| AT1G61370 | 43.5158 | 23.3222 |
| AT1G30880 | 43.5069 | 99.1328 |
| AT2G37970 | 43.4777 | 15.7302 |
| AT2G27490 | 43.4666 | 40.1636 |
| AT4G00090 | 43.4519 | 29.1114 |
| AT1G51740 | 43.4489 | 27.195 |
| AT5G04200 | 43.4436 | 45.6551 |
| AT2G17190 | 43.4042 | 21.6785 |
| AT2G32120 | 43.3938 | 41.73 |
| AT2G04630 | 43.3868 | 34.8368 |
| AT3G24740 | 43.3791 | 22.1651 |
| AT1G63930 | 43.3653 | 1.36232 |
| AT4G13660 | 43.3578 | 18.6721 |
| AT2G36680 | 43.349 | 22.9949 |
| AT3G18040 | 43.3478 | 26.1574 |
| AT2G26370 | 43.3457 | 35.9863 |
| AT5G54840 | 43.3296 | 24.6308 |
| AT2G22290 | 43.3276 | 0.218487 |
| AT3G12140 | 43.3203 | 39.1695 |
| AT5G61970 | 43.2949 | 45.6583 |
| AT1G70730 | 43.289 | 35.3136 |
| AT5G46870 | 43.2856 | 9.2109 |
| AT3G03720 | 43.2814 | 15.8769 |
| AT3G43800 | 43.2803 | 52.5532 |
| AT1G05575 | 43.2398 | 36.5168 |
| AT1G19025 | 43.1803 | 15.0099 |
| AT3G10370 | 43.1059 | 22.0794 |
| AT5G57020 | 43.1055 | 36.0371 |
| AT1G59940 | 43.077 | 22.5914 |
| AT5G18190 | 43.0511 | 29.2163 |
| AT3G14990 | 43.0321 | 199.57 |
| AT4G40085 | 43.012 | 18.1947 |
| AT5G03190 | 42.9977 | 15.3871 |
| AT5G22400 | 42.9908 | 7.55046 |
| AT3G57290 | 42.9714 | 92.552 |
| AT3G23280 | 42.9641 | 31.1871 |
| AT5G56030 | 42.9603 | 43.2956 |
| AT5G62070 | 42.9538 | 23.3462 |
| AT3G26730 | 42.9382 | 33.9382 |
| AT5G55310 | 42.9173 | 34.9539 |
| AT2G30395 | 42.9151 | 5.56788 |
| AT5G65270 | 42.9089 | 24.7932 |
| AT1G48640 | 42.8959 | 1.15054 |
| AT5G11260 | 42.8895 | 20.0587 |
| AT5G47540 | 42.8815 | 31.3473 |
| AT5G67390 | 42.8241 | 26.7899 |
| AT5G04480 | 42.8097 | 36.5872 |
| AT3G47370 | 42.7813 | 135.434 |
| AT1G49520 | 42.7778 | 43.7672 |
| AT1G04960 | 42.7539 | 32.653 |
| AT5G41100 | 42.7489 | 26.5541 |
| AT5G10730 | 42.736 | 25.5164 |
| AT3G16080 | 42.7314 | 185.198 |
| AT4G17650 | 42.7225 | 31.885 |
| AT1G09090 | 42.6703 | 25.2086 |
| AT4G17830 | 42.6549 | 36.2328 |
| AT3G57760 | 42.6451 | 35.8908 |
| AT1G69252 | 42.6097 | 26.2861 |
| AT5G65360 | 42.5754 | 99.4859 |
| AT5G44090 | 42.5516 | 45.6808 |
| AT2G33390 | 42.5378 | 23.9492 |
| AT1G29470 | 42.5349 | 126.805 |
| AT5G13100 | 42.5091 | 31.8842 |
| AT4G24190 | 42.4991 | 121.498 |
| AT2G26510 | 42.4387 | 41.5695 |
| AT3G05010 | 42.4326 | 29.8447 |
| AT5G62770 | 42.4198 | 13.3982 |
| AT1G02310 | 42.4173 | 9.40736 |
| AT5G07290 | 42.406 | 28.9223 |
| AT3G61050 | 42.3838 | 26.8879 |
| AT5G20490 | 42.3812 | 19.6762 |
| AT3G49100 | 42.377 | 30.2005 |
| AT4G26230 | 42.376 | 154.126 |
| AT1G18300 | 42.3746 | 11.423 |
| AT2G47800 | 42.3536 | 72.4754 |
| AT5G22570 | 42.3492 | 3.49925 |
| AT1G80350 | 42.3392 | 18.7238 |
| AT1G58360 | 42.3092 | 8.09489 |
| AT4G10790 | 42.2731 | 25.6436 |
| AT1G03910 | 42.2653 | 42.0586 |
| AT5G48412 | 42.2613 | 22.2676 |
| AT1G50480 | 42.2608 | 40.4144 |
| AT2G44200 | 42.2594 | 26.7284 |
| AT5G17020 | 42.2558 | 56.9182 |
| AT5G42220 | 42.2552 | 28.3474 |
| AT3G14100 | 42.2503 | 39.4573 |
| AT4G29905 | 42.2351 | 1.56744 |
| AT1G27400 | 42.2189 | 205.821 |
| AT1G12000 | 42.2082 | 109.913 |
| AT5G24880 | 42.2037 | 0.149918 |
| AT1G09020 | 42.1751 | 20.9438 |
| AT1G20960 | 42.1401 | 42.3839 |
| AT1G33700 | 42.1354 | 1.14091 |
| AT1G18160 | 42.1107 | 16.363 |
| AT1G06923 | 42.1088 | 0.0830508 |
| AT5G22450 | 42.1073 | 31.9179 |
| AT1G58190 | 42.1046 | 13.05 |
| AT1G29220 | 42.1036 | 30.5008 |
| AT2G35800 | 42.0854 | 13.0801 |
| AT5G60640 | 42.085 | 83.2408 |
| AT5G27280 | 42.0485 | 25.533 |
| AT5G05220 | 42.0364 | 47.2942 |
| AT2G24360 | 42.0296 | 34.3071 |
| AT1G02305 | 42.0016 | 30.6706 |
| AT3G11830 | 41.9922 | 170.551 |
| AT4G19112 | 41.9808 | 3.73473 |
| AT2G05630 | 41.9784 | 15.9814 |
| AT2G20530 | 41.9723 | 72.5905 |
| AT1G23900 | 41.9721 | 21.0574 |
| AT5G62575 | 41.9338 | 26.8499 |
| AT2G40290 | 41.9138 | 51.1513 |
| AT2G30140 | 41.9081 | 22.6912 |
| AT2G27660 | 41.893 | 4.62137 |
| AT5G62090 | 41.8912 | 20.4247 |
| AT3G57785 | 41.8584 | 15.7982 |
| AT5G57540 | 41.8202 | 0.0626455 |
| AT4G04860 | 41.8171 | 24.6894 |
| AT5G13010 | 41.8134 | 33.5964 |
| AT3G55980 | 41.8108 | 45.1071 |
| AT3G10660 | 41.8031 | 14.7242 |
| AT1G58110 | 41.7993 | 15.1132 |
| AT5G35370 | 41.7803 | 9.43711 |
| AT1G10740 | 41.7726 | 22.5379 |
| AT3G46930 | 41.7588 | 20.9489 |
| AT3G45600 | 41.749 | 65.9762 |
| AT3G10420 | 41.7345 | 17.1255 |
| AT1G78340 | 41.7122 | 167.626 |
| AT2G29020 | 41.6946 | 53.9471 |
| AT4G36760 | 41.6854 | 50.6174 |
| AT3G05280 | 41.6817 | 29.1512 |
| AT3G57890 | 41.6665 | 32.661 |
| AT1G17130 | 41.6644 | 45.0889 |
| AT5G20620 | 41.6561 | 27.8456 |
| AT5G59890 | 41.6541 | 39.6242 |
| AT5G55700 | 41.6505 | 20.8635 |
| AT1G25280 | 41.6457 | 40.1207 |
| AT3G63060 | 41.6347 | 12.7883 |
| AT1G04170 | 41.5962 | 61.2383 |
| AT2G43240 | 41.5728 | 29.442 |
| AT1G56010 | 41.566 | 3.30191 |
| AT5G64810 | 41.5514 | 33.6482 |
| AT3G07460 | 41.546 | 17.0871 |
| AT1G74510 | 41.5422 | 30.932 |
| AT1G52200 | 41.5399 | 18.4876 |
| AT1G74830 | 41.5366 | 1.84494 |
| AT2G45290 | 41.5335 | 47.0149 |
| AT3G21865 | 41.5318 | 43.5557 |
| AT3G56170 | 41.5275 | 48.1243 |
| AT1G28120 | 41.5239 | 42.7442 |
| AT2G34520 | 41.5155 | 39.5797 |
| AT3G55380 | 41.5021 | 36.7208 |
| AT1G10682 | 41.4989 | 70.1073 |
| AT5G58220 | 41.4933 | 21.1402 |
| AT5G17560 | 41.478 | 53.9552 |
| AT1G07410 | 41.47 | 23.3947 |
| AT3G27210 | 41.4602 | 10.9693 |
| AT2G30100 | 41.4521 | 20.7321 |
| AT3G19640 | 41.402 | 30.8071 |
| AT3G19860 | 41.3867 | 33.4804 |
| AT4G25620 | 41.3688 | 19.1818 |
| AT2G02160 | 41.3685 | 48.3875 |
| AT1G71080 | 41.3303 | 38.5116 |
| AT2G07360 | 41.3278 | 30.3123 |
| AT1G20696 | 41.3243 | 77.5559 |
| AT3G20630 | 41.2976 | 37.6961 |
| AT3G23170 | 41.2768 | 12.7546 |
| AT5G54100 | 41.2735 | 19.6672 |
| AT5G65090 | 41.273 | 0.593343 |
| AT4G29210 | 41.2656 | 6.87892 |
| AT4G13350 | 41.2618 | 23.6049 |
| AT4G30200 | 41.2592 | 49.53 |
| AT1G71730 | 41.2558 | 32.234 |
| AT1G08315 | 41.2541 | 10.2643 |
| AT5G01640 | 41.2527 | 6.98327 |
| AT4G00560 | 41.2468 | 27.2707 |
| AT3G46080 | 41.2372 | 8.51227 |
| AT3G49310 | 41.1637 | 28.9713 |
| AT1G12680 | 41.1512 | 24.134 |
| AT5G35730 | 41.1158 | 37.3353 |
| AT3G43190 | 41.1093 | 35.7096 |
| AT4G21150 | 41.0922 | 72.7586 |
| AT3G19980 | 41.0783 | 23.9427 |
| AT5G39670 | 41.0601 | 33.9447 |
| AT3G07760 | 41.0342 | 28.6381 |
| AT3G15880 | 41.001 | 28.6004 |
| AT5G13020 | 40.991 | 34.1635 |
| AT1G17455 | 40.97 | 24.2651 |
| AT1G72750 | 40.9379 | 25.0936 |
| AT1G30820 | 40.9354 | 35.3662 |
| AT3G08550 | 40.9223 | 40.405 |
| AT1G32050 | 40.9205 | 69.3735 |
| AT5G57910 | 40.9084 | 52.2196 |
| AT1G02810 | 40.8978 | 24.8571 |
| AT5G04540 | 40.8951 | 26.0181 |
| AT4G10610 | 40.8891 | 47.8376 |
| AT2G37840 | 40.8739 | 19.7747 |
| AT1G15750 | 40.8682 | 49.9791 |
| AT1G72170 | 40.8509 | 43.9018 |
| AT3G05710 | 40.8439 | 21.9045 |
| AT4G35490 | 40.8309 | 31.5078 |
| AT1G52360 | 40.8288 | 43.4864 |
| AT3G20800 | 40.827 | 33.7062 |
| AT3G07110 | 40.8247 | 128.53 |
| AT3G22260 | 40.8103 | 27.9035 |
| AT1G73340 | 40.8101 | 13.7892 |
| AT5G08170 | 40.8038 | 15.2623 |
| AT2G36300 | 40.7937 | 17.8175 |
| AT3G53370 | 40.7762 | 7.93863 |
| AT5G56290 | 40.7434 | 28.5304 |
| AT3G13445 | 40.7325 | 42.9214 |
| AT1G70320 | 40.7109 | 30.1698 |
| AT2G37670 | 40.673 | 0.994725 |
| AT5G59220 | 40.6677 | 11.3602 |
| AT3G14010 | 40.6644 | 37.1843 |
| AT3G57630 | 40.6597 | 17.859 |
| AT5G47210 | 40.6458 | 153.534 |
| AT3G51380 | 40.6409 | 1.97698 |
| AT1G04080 | 40.6204 | 48.5046 |
| AT1G15380 | 40.618 | 41.6324 |
| AT1G13930 | 40.6054 | 3.54977 |
| AT2G17200 | 40.5945 | 26.0533 |
| AT1G66900 | 40.5715 | 15.4221 |
| AT5G10540 | 40.5681 | 54.5476 |
| AT4G02195 | 40.5493 | 24.2583 |
| AT4G24800 | 40.5217 | 43.008 |
| AT5G61510 | 40.5095 | 38.1959 |
| AT5G18490 | 40.5079 | 20.1387 |
| AT2G01180 | 40.5073 | 18.6739 |
| AT1G20760 | 40.4992 | 24.959 |
| AT2G04400 | 40.4776 | 50.7306 |
| AT3G13772 | 40.4774 | 37.9423 |
| AT2G28370 | 40.477 | 30.3028 |
| AT2G47530 | 40.4546 | 0.0462881 |
| AT5G15890 | 40.4476 | 1.13924 |
| AT4G38940 | 40.418 | 18.3447 |
| AT2G33710 | 40.3703 | 25.085 |
| AT1G09575 | 40.3699 | 9.36591 |
| AT2G42570 | 40.3608 | 116.921 |
| AT2G26300 | 40.3548 | 74.2688 |
| AT3G04830 | 40.3292 | 48.742 |
| AT2G42780 | 40.3275 | 33.1412 |
| AT5G13550 | 40.309 | 39.1022 |
| AT2G32900 | 40.3001 | 25.9996 |
| AT3G47990 | 40.2884 | 21.1079 |
| AT3G19930 | 40.2763 | 45.6698 |
| AT2G24692 | 40.2445 | 6.40035 |
| AT3G14395 | 40.2402 | 14.5332 |
| AT5G15120 | 40.2202 | 21.9005 |
| AT4G16110 | 40.1997 | 12.4712 |
| AT5G37790 | 40.1903 | 18.6451 |
| AT1G53625 | 40.1863 | 33.1815 |
| AT2G02220 | 40.1799 | 32.1072 |
| AT4G08390 | 40.1754 | 38.6946 |
| AT1G24320 | 40.1752 | 2.04551 |
| AT3G24480 | 40.1733 | 31.262 |
| AT4G34131 | 40.1631 | 19.198 |
| AT1G30720 | 40.1599 | 35.6395 |
| AT3G62240 | 40.1402 | 23.2532 |
| AT2G39130 | 40.1377 | 26.7822 |
| AT4G18130 | 40.1283 | 9.00403 |
| AT5G67290 | 40.1255 | 9.92739 |
| AT1G11580 | 40.1105 | 104.645 |
| AT2G40420 | 40.1045 | 7.70715 |
| AT5G50380 | 40.0892 | 33.0482 |
| AT1G12470 | 40.089 | 19.1493 |
| AT1G26300 | 40.0675 | 23.4926 |
| AT1G68140 | 40.0412 | 39.3965 |
| AT2G21470 | 40.036 | 46.9985 |
| AT4G01960 | 40.0337 | 21.4435 |
| AT5G04470 | 40.0035 | 5.50779 |
| AT4G32690 | 39.9906 | 31.1916 |
| AT3G53930 | 39.9884 | 22.6392 |
| AT3G55280 | 39.9824 | 224.466 |
| AT1G59725 | 39.9778 | 0.0515449 |
| AT5G03285 | 39.9573 | 46.8309 |
| AT5G66040 | 39.9519 | 5.92621 |
| AT5G66850 | 39.9218 | 25.3901 |
| AT1G43690 | 39.9205 | 26.9491 |
| AT4G38360 | 39.9037 | 18.442 |
| AT5G18830 | 39.8696 | 19.1483 |
| AT1G13020 | 39.8577 | 27.5077 |
| AT3G15710 | 39.8563 | 60.1729 |
| AT1G64280 | 39.8419 | 19.5798 |
| AT4G22230 | 39.8335 | 63.7543 |
| AT5G18230 | 39.8122 | 37.6799 |
| AT2G28390 | 39.7777 | 21.5154 |
| AT3G06670 | 39.7762 | 29.6778 |
| AT1G12080 | 39.772 | 26.9199 |
| AT3G25070 | 39.7373 | 63.846 |
| AT1G09770 | 39.7322 | 59.1649 |
| AT3G50550 | 39.691 | 47.6722 |
| AT4G01030 | 39.6794 | 22.5586 |
| AT4G31250 | 39.6763 | 2.62096 |
| AT4G29410 | 39.6759 | 167.062 |
| AT4G39270 | 39.671 | 21.1939 |
| AT1G54210 | 39.6564 | 35.1469 |
| AT4G29380 | 39.6527 | 23.0942 |
| AT1G11210 | 39.6188 | 11.9545 |
| AT1G69930 | 39.5638 | 62.7419 |
| AT3G61820 | 39.5569 | 36.9379 |
| AT1G14510 | 39.5524 | 48.9744 |
| AT1G01820 | 39.5154 | 51.8233 |
| AT2G46790 | 39.5027 | 17.1016 |
| AT3G07070 | 39.4694 | 0.207821 |
| AT2G42030 | 39.4646 | 32.3785 |
| AT2G15980 | 39.4528 | 2.4858 |
| AT4G39360 | 39.434 | 10.9713 |
| AT1G04810 | 39.4276 | 38.1629 |
| AT5G13910 | 39.4181 | 11.2636 |
| AT1G71696 | 39.4051 | 20.5875 |
| AT5G57090 | 39.3992 | 91.7715 |
| AT3G49350 | 39.3889 | 16.0442 |
| AT4G28910 | 39.3756 | 45.3248 |
| AT5G19690 | 39.3507 | 54.6399 |
| AT1G24560 | 39.3385 | 21.596 |
| AT3G08030 | 39.3277 | 92.5896 |
| AT5G19010 | 39.3267 | 44.9408 |
| AT3G12980 | 39.2893 | 24.2273 |
| AT4G32240 | 39.2745 | 29.0801 |
| AT1G09645 | 39.251 | 62.1752 |
| AT1G14530 | 39.2488 | 16.7323 |
| AT3G60280 | 39.2416 | 0.0956863 |
| AT2G18510 | 39.2356 | 32.6189 |
| AT3G45040 | 39.2041 | 36.4248 |
| AT3G51040 | 39.1966 | 22.3764 |
| AT4G35040 | 39.192 | 19.3166 |
| AT5G66120 | 39.1897 | 32.2981 |
| AT2G18630 | 39.1629 | 35.9716 |
| AT2G45440 | 39.1457 | 25.1567 |
| AT1G10090 | 39.1357 | 8.32378 |
| AT4G36210 | 39.1349 | 31.6413 |
| AT5G54470 | 39.1342 | 13.9481 |
| AT5G57300 | 39.1225 | 62.5466 |
| AT4G27290 | 39.0958 | 1.50421 |
| AT5G45430 | 39.091 | 10.0133 |
| AT2G26990 | 39.0789 | 39.5913 |
| AT4G07390 | 39.0741 | 29.7097 |
| AT5G20180 | 39.0664 | 51.0163 |
| AT5G57035 | 39.0488 | 24.1565 |
| AT2G36170 | 39.0439 | 107.766 |
| AT4G24570 | 39.0275 | 20.2036 |
| AT2G16950 | 39.0209 | 22.8699 |
| AT2G17550 | 39.0094 | 16.9374 |
| AT3G14180 | 39.0055 | 23.5541 |
| AT1G08370 | 38.9948 | 34.808 |
| AT1G78890 | 38.9918 | 27.3963 |
| AT4G05330 | 38.9895 | 5.29924 |
| AT3G55940 | 38.9852 | 14.3651 |
| AT2G26400 | 38.9796 | 2.29614 |
| AT1G67580 | 38.9488 | 29.5785 |
| AT2G43810 | 38.9414 | 27.5606 |
| AT2G20140 | 38.9366 | 40.7013 |
| AT4G24290 | 38.9357 | 45.6356 |
| AT5G06990 | 38.9156 | 2.06317 |
| AT2G19710 | 38.9134 | 21.4556 |
| AT1G69980 | 38.892 | 28.4473 |
| AT3G07490 | 38.8904 | 1.13937 |
| AT3G51350 | 38.8901 | 0.274518 |
| AT3G14400 | 38.889 | 27.2442 |
| AT4G15420 | 38.8869 | 32.1251 |
| AT2G27260 | 38.8844 | 34.5422 |
| AT5G46350 | 38.8387 | 8.41764 |
| AT4G23700 | 38.8386 | 21.8921 |
| AT3G59500 | 38.8126 | 40.7501 |
| AT1G11680 | 38.8084 | 78.4509 |
| AT2G22970 | 38.8072 | 12.5773 |
| AT1G18500 | 38.806 | 47.7627 |
| AT3G10650 | 38.7968 | 26.7306 |
| AT2G06255 | 38.7752 | 16.2876 |
| AT1G05170 | 38.7739 | 40.936 |
| AT2G20320 | 38.757 | 30.7893 |
| AT1G62390 | 38.7425 | 36.2164 |
| AT5G27720 | 38.7274 | 41.4101 |
| AT1G67190 | 38.7243 | 18.4393 |
| AT5G13110 | 38.7197 | 27.011 |
| AT3G23810 | 38.717 | 7.60556 |
| AT1G03330 | 38.6829 | 62.4967 |
| AT1G19700 | 38.6821 | 10.7547 |
| AT1G12520 | 38.6666 | 24.7264 |
| AT3G26100 | 38.6651 | 12.1369 |
| AT1G64220 | 38.6637 | 45.3013 |
| AT3G60340 | 38.6429 | 27.9127 |
| AT1G29300 | 38.6333 | 18.4414 |
| AT3G12587 | 38.6241 | 51.033 |
| AT2G44710 | 38.6147 | 24.6892 |
| AT3G45090 | 38.604 | 12.5221 |
| AT1G09815 | 38.5911 | 25.4809 |
| AT5G04420 | 38.5633 | 33.429 |
| AT1G11185 | 38.5567 | 64.8629 |
| AT4G23270 | 38.5526 | 29.1808 |
| AT4G36090 | 38.5512 | 22.134 |
| AT5G22080 | 38.54 | 32.1282 |
| AT4G26060 | 38.5366 | 39.6841 |
| AT1G21450 | 38.528 | 24.1106 |
| AT5G10010 | 38.5113 | 42.6965 |
| AT5G08280 | 38.5084 | 26.7929 |
| AT4G00550 | 38.4664 | 19.641 |
| AT4G16490 | 38.4605 | 27.0777 |
| AT1G47290 | 38.4377 | 22.8783 |
| AT1G33250 | 38.4278 | 19.1628 |
| AT2G03510 | 38.377 | 30.9836 |
| AT2G33840 | 38.3594 | 33.4791 |
| AT2G16790 | 38.3578 | 17.5207 |
| AT2G31360 | 38.3529 | 59.0063 |
| AT5G62560 | 38.3476 | 10.1543 |
| AT2G25180 | 38.3216 | 29.1663 |
| AT3G09830 | 38.3123 | 42.2489 |
| AT3G01830 | 38.3092 | 37.0012 |
| AT1G09730 | 38.3085 | 33.1086 |
| AT2G43750 | 38.2837 | 42.8935 |
| AT1G55190 | 38.2822 | 12.3188 |
| AT1G51680 | 38.278 | 4.34022 |
| AT1G22810 | 38.2491 | 11.6484 |
| AT3G03380 | 38.2477 | 30.6881 |
| AT3G07195 | 38.2359 | 2.76776 |
| AT1G58280 | 38.2336 | 18.5336 |
| AT5G58940 | 38.1678 | 4.1385 |
| AT3G60410 | 38.1594 | 18.4453 |
| AT2G30340 | 38.1545 | 103.73 |
| AT5G54390 | 38.1391 | 98.3844 |
| AT3G54480 | 38.1299 | 26.142 |
| AT2G06045.1 | 38.1133 | 17.7567 |
| AT1G06040 | 38.1087 | 38.3639 |
| AT4G39800 | 38.1057 | 57.5074 |
| AT3G26500 | 38.0725 | 1.7151 |
| AT1G69340 | 38.0476 | 22.3666 |
| AT5G63190 | 38.0454 | 20.9075 |
| AT3G10550 | 38.0118 | 18.1849 |
| AT4G02600 | 38.0084 | 33.4622 |
| AT3G19340 | 38.0073 | 17.8716 |
| AT1G10865 | 37.9942 | 21.3344 |
| AT4G31860 | 37.977 | 29.8231 |
| AT5G63470 | 37.9743 | 14.4741 |
| AT5G62000 | 37.9681 | 22.0325 |
| AT5G43570 | 37.9458 | 37.9329 |
| AT5G65380 | 37.9458 | 19.0946 |
| AT2G33510 | 37.9458 | 10.5024 |
| AT4G22240 | 37.9441 | 38.0036 |
| AT4G39050 | 37.9438 | 44.7584 |
| AT5G27860 | 37.9407 | 34.8811 |
| AT1G62040 | 37.9378 | 26.108 |
| AT2G22480 | 37.9319 | 23.8333 |
| AT1G47970 | 37.9272 | 68.7403 |
| AT5G23590 | 37.9182 | 28.8415 |
| AT5G25360 | 37.8827 | 18.0211 |
| AT4G02390 | 37.8809 | 3.78638 |
| AT4G30410 | 37.8712 | 26.0518 |
| AT1G76380 | 37.8679 | 45.6409 |
| AT4G36515 | 37.8598 | 11.6054 |
| AT1G48300 | 37.8383 | 24.0212 |
| AT5G09310 | 37.8249 | 17.7768 |
| AT5G51120 | 37.8231 | 59.862 |
| AT4G19190 | 37.821 | 28.2703 |
| AT2G29560 | 37.8172 | 24.2923 |
| AT1G18890 | 37.7627 | 26.2536 |
| AT1G20670 | 37.7512 | 24.7149 |
| AT4G33440 | 37.7438 | 23.2025 |
| AT5G66680 | 37.7395 | 70.2562 |
| AT2G46620 | 37.7178 | 9.56485 |
| AT1G76892 | 37.7102 | 8.78509 |
| AT1G26750 | 37.7062 | 34.9237 |
| AT5G47050 | 37.7051 | 44.608 |
| AT3G25610 | 37.7009 | 35.1111 |
| AT3G10760 | 37.684 | 15.6015 |
| AT3G02200 | 37.6766 | 70.3117 |
| AT2G42590 | 37.6701 | 64.9326 |
| AT5G24165 | 37.6647 | 7.48493 |
| AT3G05800 | 37.6626 | 1.29728 |
| AT3G44200 | 37.6365 | 19.6707 |
| AT5G16210 | 37.6245 | 41.1366 |
| AT3G53490 | 37.5854 | 9.12581 |
| AT4G12880 | 37.5702 | 49.9603 |
| AT1G47530 | 37.5516 | 20.9945 |
| AT2G01720 | 37.5479 | 52.1535 |
| AT2G15270 | 37.5464 | 36.3255 |
| AT3G03000 | 37.5296 | 2.62963 |
| AT2G43080 | 37.5236 | 26.9996 |
| AT5G60340 | 37.5117 | 41.3444 |
| AT3G48115 | 37.4917 | 17.4035 |
| AT1G50060 | 37.49 | 251.173 |
| AT3G16870 | 37.4864 | 24.8519 |
| AT1G61226 | 37.4807 | 2.70781 |
| AT1G26665 | 37.4694 | 24.7976 |
| AT3G25840 | 37.468 | 23.5231 |
| AT1G14360 | 37.4651 | 39.2936 |
| AT1G56080 | 37.4646 | 18.6111 |
| AT5G20680 | 37.4288 | 17.1269 |
| AT2G30360 | 37.4269 | 14.9429 |
| AT2G21580 | 37.415 | 145.525 |
| AT4G22670 | 37.3892 | 64.455 |
| AT1G55320 | 37.389 | 11.7766 |
| AT1G03905 | 37.3828 | 24.2455 |
| AT5G60548 | 37.3777 | 18.4759 |
| AT5G27420 | 37.3679 | 7.35327 |
| AT1G26940 | 37.3398 | 22.2035 |
| AT4G32450 | 37.3333 | 20.9244 |
| AT2G02310 | 37.3252 | 13.3183 |
| AT3G58510 | 37.321 | 26.5941 |
| AT1G76590 | 37.3167 | 49.2929 |
| AT5G55190 | 37.3098 | 94.3228 |
| AT1G79690 | 37.3077 | 25.3529 |
| AT4G36020 | 37.2933 | 26.5923 |
| AT5G49570 | 37.2927 | 17.6391 |
| AT1G04490 | 37.2751 | 17.5972 |
| AT2G23350 | 37.2697 | 49.3844 |
| AT3G12965 | 37.2624 | 117.817 |
| AT4G33250 | 37.2538 | 105.804 |
| AT3G24730 | 37.2491 | 21.0858 |
| AT3G58610 | 37.2474 | 132.293 |
| AT1G01453 | 37.2356 | 0.0750574 |
| AT1G79000 | 37.2191 | 26.7868 |
| AT1G63580 | 37.164 | 5.35363 |
| AT3G51580 | 37.1611 | 18.124 |
| AT1G49032 | 37.1449 | 9.9723 |
| AT1G19580 | 37.1375 | 28.5212 |
| AT4G12520 | 37.1342 | 3.83656 |
| AT4G33540 | 37.1155 | 66.3713 |
| AT5G54585 | 37.1068 | 10.7015 |
| AT5G63380 | 37.0972 | 18.9386 |
| AT5G60570 | 37.0933 | 23.2913 |
| AT1G21390 | 37.068 | 29.5235 |
| AT3G59910 | 37.0661 | 19.9268 |
| AT1G77750 | 37.0558 | 34.3464 |
| AT3G06310 | 37.041 | 26.9496 |
| AT2G39970 | 37.0381 | 23.7847 |
| AT3G21230 | 37.0357 | 6.6544 |
| AT5G48380 | 37.025 | 26.656 |
| AT3G10360 | 36.9904 | 22.7113 |
| AT3G13750 | 36.9881 | 24.8663 |
| AT3G59300 | 36.9744 | 22.045 |
| AT2G34585 | 36.9671 | 9.98873 |
| AT3G55960 | 36.9557 | 24.7397 |
| AT2G14120 | 36.9429 | 32.5833 |
| AT4G34390 | 36.9167 | 23.4139 |
| AT1G32700 | 36.8889 | 106.25 |
| AT3G12610 | 36.8703 | 68.4507 |
| AT4G08690 | 36.8414 | 15.4037 |
| AT1G71410 | 36.8195 | 20.5104 |
| AT4G21960 | 36.811 | 102.031 |
| AT4G11560 | 36.8084 | 32.4159 |
| AT2G05840 | 36.7974 | 36.0415 |
| AT5G02880 | 36.7951 | 28.4922 |
| AT2G22660 | 36.792 | 56.794 |
| AT3G14080 | 36.7867 | 41.3188 |
| AT4G27080 | 36.7685 | 35.6303 |
| AT4G13850 | 36.7638 | 147.221 |
| AT1G76210 | 36.7597 | 2.41801 |
| AT4G38740 | 36.7551 | 95.9059 |
| AT1G27600 | 36.7261 | 28.5252 |
| AT5G23900 | 36.691 | 99.1495 |
| AT2G02470 | 36.6822 | 33.5422 |
| AT3G54860 | 36.6508 | 25.2491 |
| AT3G47850 | 36.6464 | 24.4523 |
| AT3G60440 | 36.6373 | 17.7367 |
| AT3G61415 | 36.6331 | 22.593 |
| AT4G38390 | 36.6331 | 0.260206 |
| AT3G23250 | 36.6327 | 22.527 |
| AT4G32160 | 36.6286 | 28.4117 |
| AT3G47780 | 36.6021 | 23.0594 |
| AT3G62660 | 36.5758 | 14.7213 |
| AT2G37150 | 36.5583 | 17.603 |
| AT1G49050 | 36.5509 | 24.6976 |
| AT4G36630 | 36.5201 | 17.2482 |
| AT2G35790 | 36.4798 | 40.594 |
| AT1G03290 | 36.4482 | 21.4076 |
| AT5G44240 | 36.4479 | 26.4046 |
| AT1G04980 | 36.4419 | 32.3039 |
| AT5G06830 | 36.4217 | 20.1912 |
| AT3G27530 | 36.4209 | 20.2775 |
| AT2G31260 | 36.399 | 19.9514 |
| AT5G18500 | 36.3981 | 31.0428 |
| AT4G29810 | 36.3917 | 41.235 |
| AT2G01480 | 36.3783 | 25.8572 |
| AT5G12350 | 36.3725 | 18.0228 |
| AT4G32930 | 36.3475 | 51.8011 |
| AT3G23990 | 36.3393 | 104.925 |
| AT1G62975 | 36.3364 | 6.26109 |
| AT2G41473 | 36.29 | 0.158519 |
| AT3G50590 | 36.2829 | 28.4827 |
| AT1G10410 | 36.2631 | 26.0463 |
| AT5G62910 | 36.2551 | 28.1998 |
| AT1G54460 | 36.2442 | 20.1387 |
| AT1G60750 | 36.2223 | 10.3391 |
| AT1G77220 | 36.2158 | 25.6925 |
| AT1G27030 | 36.2116 | 3.07547 |
| AT1G80450 | 36.2114 | 10.1338 |
| AT1G22640 | 36.1842 | 28.3032 |
| AT1G49040 | 36.1702 | 19.6774 |
| AT1G27770 | 36.1487 | 56.5637 |
| AT3G61060 | 36.1383 | 2.74545 |
| AT4G32590 | 36.1303 | 21.3992 |
| AT1G15030 | 36.1241 | 48.0048 |
| AT1G76880 | 36.1227 | 27.6657 |
| AT1G17530 | 36.1147 | 23.6536 |
| AT5G06570 | 36.0666 | 7.58522 |
| AT2G26920 | 36.0523 | 19.8855 |
| AT4G01290 | 36.052 | 22.0461 |
| AT1G55850 | 36.0404 | 50.5131 |
| AT2G17870 | 36.0255 | 19.8305 |
| AT4G12005 | 36.0092 | 17.3551 |
| AT3G51240 | 35.9907 | 11.1429 |
| AT5G61550 | 35.9846 | 0.372456 |
| AT5G52040 | 35.9794 | 30.1931 |
| AT2G16460 | 35.9775 | 19.6143 |
| AT2G18770 | 35.9663 | 19.0968 |
| AT5G18280 | 35.9659 | 24.7231 |
| AT5G11900 | 35.9511 | 37.4602 |
| AT2G27100 | 35.944 | 37.9262 |
| AT3G55070 | 35.939 | 20.3301 |
| AT5G14720 | 35.931 | 27.8951 |
| AT5G41685 | 35.9168 | 50.8074 |
| AT4G34280 | 35.9089 | 27.85 |
| AT3G29200 | 35.9021 | 16.518 |
| AT1G20510 | 35.9019 | 59.2417 |
| AT4G05000 | 35.8916 | 22.6202 |
| AT2G22010 | 35.8908 | 33.6089 |
| AT4G10710 | 35.8746 | 35.7661 |
| AT2G29620 | 35.8692 | 0.067347 |
| AT3G01780 | 35.8578 | 26.063 |
| AT5G66200 | 35.8486 | 14.3294 |
| AT4G36520 | 35.8293 | 12.0626 |
| AT1G27100 | 35.8099 | 26.6559 |
| AT3G60860 | 35.77 | 21.6027 |
| AT1G63170 | 35.7314 | 22.9636 |
| AT2G24570 | 35.7225 | 26.1527 |
| AT4G24370 | 35.6995 | 24.243 |
| AT2G17710 | 35.6985 | 10.1854 |
| AT4G29840 | 35.6922 | 60.4845 |
| AT3G60540 | 35.6917 | 2.10156 |
| AT4G11800 | 35.6905 | 35.7883 |
| AT3G56680 | 35.6868 | 37.2547 |
| AT3G51520 | 35.6729 | 33.5186 |
| AT4G37590 | 35.6596 | 44.3248 |
| AT2G39805 | 35.6408 | 18.5556 |
| AT2G30950 | 35.6391 | 38.8354 |
| AT2G46000 | 35.6332 | 27.2474 |
| AT2G41960 | 35.5742 | 24.9257 |
| AT2G32970 | 35.55 | 26.2026 |
| AT1G54150 | 35.5391 | 22.9205 |
| AT5G63610 | 35.5169 | 19.2654 |
| AT3G03780 | 35.5167 | 23.645 |
| AT1G34220 | 35.5076 | 18.1036 |
| AT4G23640 | 35.4945 | 13.1821 |
| AT1G20693 | 35.4935 | 45.6593 |
| AT2G37060 | 35.484 | 40.7729 |
| AT1G14540 | 35.4839 | 6.23932 |
| AT5G28150 | 35.4704 | 13.2912 |
| AT5G56010 | 35.46 | 32.0357 |
| AT1G66070 | 35.434 | 31.1076 |
| AT5G16680 | 35.4273 | 23.1215 |
| AT5G62620 | 35.4252 | 17.2544 |
| AT5G41680 | 35.4244 | 2.6138 |
| AT4G02100 | 35.4233 | 18.0438 |
| AT5G18580 | 35.4144 | 40.115 |
| AT5G45390 | 35.4119 | 51.4157 |
| AT5G15130 | 35.4063 | 11.3726 |
| AT1G77170 | 35.4056 | 20.5233 |
| AT1G19396 | 35.4028 | 5.63352 |
| AT2G02230 | 35.3758 | 23.3629 |
| AT3G52460 | 35.3533 | 5.83853 |
| AT4G35940 | 35.3153 | 26.3401 |
| AT1G30890 | 35.3129 | 23.5763 |
| AT5G43880 | 35.3069 | 28.9834 |
| AT2G19860 | 35.3011 | 21.0831 |
| AT2G35630 | 35.299 | 29.5552 |
| AT4G34200 | 35.2852 | 95.095 |
| AT1G72416 | 35.2788 | 44.9999 |
| AT4G14342 | 35.2548 | 53.9666 |
| AT3G07950 | 35.2493 | 37.3338 |
| AT2G26600 | 35.2209 | 15.7922 |
| AT5G16270 | 35.2056 | 38.5847 |
| AT3G27310 | 35.1976 | 24.6112 |
| AT4G25360 | 35.1897 | 48.3956 |
| AT5G24470 | 35.1893 | 12.9871 |
| AT1G69800 | 35.1853 | 10.1535 |
| AT3G20250 | 35.1822 | 20.9868 |
| AT3G22160 | 35.1822 | 17.7126 |
| AT3G18830 | 35.1791 | 14.9441 |
| AT5G62640 | 35.1623 | 24.1874 |
| AT5G48150 | 35.1545 | 13.2461 |
| AT1G71940 | 35.153 | 22.7802 |
| AT1G22750 | 35.1327 | 9.76815 |
| AT1G07990 | 35.0787 | 28.343 |
| AT5G45775 | 35.0495 | 181.876 |
| AT4G38730 | 35.0408 | 15.0826 |
| AT2G36390 | 35.0344 | 29.6732 |
| AT3G25400 | 35.0327 | 27.6503 |
| AT3G01340 | 35.0301 | 27.9736 |
| AT4G20110 | 35.0237 | 1.52904 |
| AT2G23960 | 35.0038 | 7.96444 |
| AT5G65950 | 34.9819 | 30.3534 |
| AT5G58160 | 34.973 | 7.83548 |
| AT3G22630 | 34.9511 | 25.9089 |
| AT1G04985 | 34.9408 | 25.7537 |
| AT3G52230 | 34.9112 | 35.337 |
| AT3G02250 | 34.9089 | 18.3836 |
| AT4G02830 | 34.9084 | 0.352575 |
| AT4G16350 | 34.8977 | 2.29663 |
| AT5G04020 | 34.8892 | 11.6601 |
| AT5G58470 | 34.8833 | 26.6914 |
| AT1G52540 | 34.8787 | 28.9644 |
| AT3G56860 | 34.871 | 22.0239 |
| AT3G12110 | 34.8619 | 34.5476 |
| AT5G26749 | 34.8426 | 17.6786 |
| AT4G29960 | 34.8257 | 23.9667 |
| AT4G38950 | 34.8146 | 15.0918 |
| AT5G54670 | 34.8012 | 36.5103 |
| AT2G38880 | 34.7544 | 31.3141 |
| AT5G65640 | 34.7423 | 77.2895 |
| AT4G22750 | 34.7209 | 23.6117 |
| AT3G03600 | 34.7201 | 30.8935 |
| AT4G18610 | 34.7173 | 4.49472 |
| AT4G20320 | 34.7157 | 44.5152 |
| AT4G25170 | 34.7135 | 10.4834 |
| AT4G30660 | 34.6942 | 68.7236 |
| AT2G39580 | 34.6743 | 19.9447 |
| AT3G07170 | 34.6345 | 44.7769 |
| AT2G29995 | 34.6276 | 73.8017 |
| AT5G38895 | 34.6013 | 17.4256 |
| AT1G56300 | 34.5954 | 12.3162 |
| AT3G14270 | 34.5761 | 14.7774 |
| AT3G06380 | 34.5724 | 20.7955 |
| AT3G51990 | 34.5659 | 11.1008 |
| AT3G53340 | 34.5632 | 30.9527 |
| AT3G52640 | 34.5621 | 23.6435 |
| AT2G17990 | 34.557 | 31.9125 |
| AT2G36930 | 34.5513 | 57.2522 |
| AT3G25770 | 34.5256 | 49.6525 |
| AT2G04880 | 34.5125 | 20.5909 |
| AT5G03230 | 34.5076 | 25.2655 |
| AT1G10890 | 34.5055 | 23.1476 |
| AT4G31460 | 34.4931 | 30.1597 |
| AT1G70710 | 34.4885 | 29.2766 |
| AT5G27640 | 34.4825 | 55.424 |
| AT5G51620 | 34.4555 | 30.3171 |
| AT3G18165 | 34.4488 | 45.2942 |
| AT1G49340 | 34.4437 | 13.2183 |
| AT2G34730 | 34.4365 | 28.1767 |
| AT5G41940 | 34.4286 | 16.4382 |
| AT4G21540 | 34.426 | 21.9581 |
| AT5G06230 | 34.4147 | 3.75889 |
| AT1G50740 | 34.4047 | 34.3485 |
| AT2G40060 | 34.4037 | 59.2011 |
| AT2G38010 | 34.3858 | 33.5019 |
| AT5G43350 | 34.3803 | 3.62091 |
| AT2G34160 | 34.3799 | 31.7841 |
| AT1G04690 | 34.3674 | 67.8625 |
| AT5G18850 | 34.3581 | 15.7631 |
| AT3G54085 | 34.3508 | 21.9332 |
| AT1G16810 | 34.3489 | 57.1455 |
| AT4G05160 | 34.3442 | 25.6752 |
| AT4G11420 | 34.3409 | 66.4666 |
| AT1G67730 | 34.338 | 68.172 |
| AT2G45910 | 34.3303 | 16.5747 |
| AT3G52190 | 34.3239 | 30.893 |
| AT2G48100 | 34.2876 | 30.8776 |
| AT5G24320 | 34.2827 | 19.9051 |
| AT2G15990.1 | 34.2799 | 0.978216 |
| AT1G79360 | 34.2739 | 13.6342 |
| AT2G26650 | 34.2601 | 30.4482 |
| AT2G01850 | 34.2499 | 28.8227 |
| AT5G23610 | 34.2485 | 27.398 |
| AT2G38580 | 34.2377 | 16.3011 |
| AT5G53480 | 34.2354 | 34.4059 |
| AT1G79870 | 34.2182 | 9.53903 |
| AT5G42960 | 34.2126 | 31.9262 |
| AT5G55550 | 34.1876 | 7.0897 |
| AT5G49680 | 34.1874 | 11.3609 |
| AT4G35785 | 34.1823 | 31.4248 |
| AT1G03950 | 34.155 | 7.38174 |
| AT3G04530 | 34.1546 | 7.5911 |
| AT3G16060 | 34.1427 | 24.1795 |
| AT1G48745 | 34.1412 | 1.02216 |
| AT1G12920 | 34.1411 | 66.9114 |
| AT5G28840 | 34.1286 | 73.8176 |
| AT2G36850 | 34.1273 | 34.8746 |
| AT1G55160 | 34.126 | 21.5096 |
| AT5G05730 | 34.1044 | 89.5548 |
| AT4G24415 | 34.1025 | 14.2649 |
| AT1G13190 | 34.0898 | 36.5939 |
| AT2G30600 | 34.0741 | 17.2045 |
| AT3G11400 | 34.0635 | 49.3913 |
| AT3G24050 | 34.0472 | 19.2305 |
| AT3G61760 | 34.0428 | 8.31952 |
| AT1G03730 | 34.02 | 15.5377 |
| AT5G04990 | 34.0131 | 18.2454 |
| AT1G69040 | 34.007 | 54.4659 |
| AT1G54530 | 34.0035 | 3.32935 |
| AT1G65540 | 33.9713 | 19.3486 |
| AT1G10160.1 | 33.967 | 23.3582 |
| AT1G32870 | 33.9668 | 27.4189 |
| AT5G62890 | 33.9651 | 58.1995 |
| AT2G47330 | 33.959 | 22.8148 |
| AT5G55040 | 33.9588 | 21.8285 |
| AT4G25650 | 33.9499 | 15.3832 |
| AT1G14210 | 33.9472 | 39.8269 |
| AT5G52050 | 33.9467 | 30.7076 |
| AT5G02610 | 33.9452 | 98.6777 |
| AT5G06930 | 33.9366 | 9.23135 |
| AT1G60470 | 33.93 | 22.1337 |
| AT3G22750 | 33.9134 | 13.6671 |
| AT5G49650 | 33.8958 | 23.5246 |
| AT2G43890 | 33.8874 | 64.8783 |
| AT1G10720 | 33.8595 | 19.255 |
| AT1G33050 | 33.8393 | 22.2424 |
| AT2G31710 | 33.8387 | 22.1079 |
| AT5G34930 | 33.8319 | 10.0518 |
| AT5G66810 | 33.8275 | 16.6926 |
| AT4G30790 | 33.8221 | 27.5381 |
| AT4G13530 | 33.8061 | 20.6904 |
| AT5G46210 | 33.7921 | 43.9258 |
| AT4G33565 | 33.7707 | 30.9461 |
| AT1G27650 | 33.7662 | 29.0272 |
| AT5G48000 | 33.7625 | 39.7395 |
| AT1G79920 | 33.7603 | 26.1758 |
| AT1G10350 | 33.7586 | 8.30636 |
| AT5G03560 | 33.7492 | 20.7009 |
| AT2G39660 | 33.7334 | 19.3931 |
| AT1G71140 | 33.6964 | 63.4591 |
| AT1G53030 | 33.6956 | 23.5521 |
| AT1G11090 | 33.6791 | 15.8409 |
| AT3G49370 | 33.6561 | 7.78148 |
| AT5G58800 | 33.652 | 9.79985 |
| AT4G26210 | 33.6435 | 34.9135 |
| AT3G15740 | 33.6194 | 10.2635 |
| AT2G38250 | 33.6165 | 31.1558 |
| AT3G23000 | 33.604 | 32.1197 |
| AT2G41730 | 33.5927 | 7.18733 |
| AT3G27100 | 33.5851 | 12.078 |
| AT1G08770 | 33.5794 | 8.72335 |
| AT4G13040 | 33.5776 | 17.8852 |
| AT1G12240 | 33.5628 | 32.4136 |
| AT4G30097 | 33.5626 | 4.37782 |
| AT1G21780 | 33.5603 | 18.9861 |
| AT1G48240 | 33.5463 | 7.72199 |
| AT2G03220 | 33.5285 | 15.8898 |
| AT1G06870 | 33.525 | 30.0015 |
| AT5G24710 | 33.516 | 22.0937 |
| AT3G54190 | 33.5057 | 39.2193 |
| AT1G52330 | 33.4813 | 9.44995 |
| AT3G09850 | 33.4612 | 31.1887 |
| AT5G11490 | 33.4254 | 23.015 |
| AT3G16830 | 33.4179 | 21.7621 |
| AT4G24840 | 33.4093 | 29.4637 |
| AT5G17760 | 33.4031 | 31.3763 |
| AT3G13235 | 33.4023 | 37.9715 |
| AT3G09470 | 33.3834 | 22.8398 |
| AT2G39760 | 33.377 | 27.9925 |
| AT1G07020 | 33.3639 | 24.3322 |
| AT3G27770 | 33.3596 | 55.9685 |
| AT5G43970 | 33.3526 | 41.4708 |
| AT2G01830 | 33.3522 | 19.6166 |
| AT1G74640 | 33.3475 | 8.66905 |
| AT1G22570 | 33.3471 | 4.37621 |
| AT5G66030 | 33.3051 | 34.0577 |
| AT1G32410 | 33.2743 | 25.1371 |
| AT4G26160 | 33.2706 | 14.9998 |
| AT2G05590 | 33.2286 | 24.1077 |
| AT5G24430 | 33.2116 | 30.4107 |
| AT2G39990 | 33.2085 | 61.5304 |
| AT1G55590 | 33.1979 | 16.8427 |
| AT2G35710 | 33.1764 | 11.5141 |
| AT1G15530 | 33.1619 | 12.5379 |
| AT1G75850 | 33.1453 | 14.456 |
| AT4G03505 | 33.1346 | 5.77085 |
| AT1G76150 | 33.134 | 27.9773 |
| AT2G21180 | 33.113 | 31.6835 |
| AT5G66390 | 33.0889 | 31.5404 |
| AT5G02800 | 33.077 | 14.9237 |
| AT3G27060 | 33.0716 | 17.9947 |
| AT1G23040 | 33.0531 | 22.5001 |
| AT1G27630 | 33.05 | 20.8608 |
| AT5G60350 | 33.0364 | 18.5914 |
| AT1G02330 | 33.0302 | 32.4941 |
| AT2G15240 | 33.025 | 23.5144 |
| AT5G19930 | 33.0243 | 18.5162 |
| AT3G06150 | 33.0038 | 23.9612 |
| AT4G14220 | 32.9822 | 20.8145 |
| AT5G51510 | 32.9796 | 26.4386 |
| AT4G23760 | 32.9734 | 26.7969 |
| AT1G54130 | 32.9599 | 22.7943 |
| AT5G67310 | 32.9499 | 3.81115 |
| AT4G16143 | 32.9454 | 44.3146 |
| AT1G04000 | 32.9345 | 14.3145 |
| AT4G35950 | 32.9335 | 13.2209 |
| AT1G73670 | 32.9148 | 24.1182 |
| AT1G76860 | 32.9038 | 30.8326 |
| AT1G02930 | 32.8692 | 92.9471 |
| AT2G26890 | 32.8545 | 12.4019 |
| AT5G25820 | 32.8516 | 8.34222 |
| AT5G63890 | 32.8451 | 40.1263 |
| AT2G19480 | 32.8448 | 64.9509 |
| AT5G11000 | 32.8341 | 13.1742 |
| AT5G02290 | 32.8291 | 38.8301 |
| AT1G79260 | 32.8183 | 18.2896 |
| AT2G48080 | 32.817 | 4.20925 |
| AT3G11910 | 32.8112 | 45.4198 |
| AT2G34590 | 32.7971 | 55.8938 |
| AT4G00660 | 32.796 | 21.1442 |
| AT5G43400 | 32.7917 | 8.81557 |
| AT1G08820 | 32.7545 | 55.2796 |
| AT4G03240 | 32.7482 | 30.9912 |
| AT5G38630 | 32.7482 | 22.3924 |
| AT3G55750 | 32.7479 | 163.081 |
| AT4G35380 | 32.7441 | 7.57528 |
| AT5G51050 | 32.743 | 24.2918 |
| AT4G34265 | 32.7392 | 58.3609 |
| AT1G09795 | 32.7358 | 51.6857 |
| AT2G22690 | 32.7337 | 19.6029 |
| AT4G03330 | 32.7223 | 1.51902 |
| AT1G27340 | 32.7185 | 24.8935 |
| AT1G26820 | 32.7167 | 46.8144 |
| AT5G07740 | 32.7096 | 18.1389 |
| AT5G38220 | 32.684 | 16.7337 |
| AT4G29520 | 32.6743 | 35.1201 |
| AT1G56190 | 32.6405 | 17.1351 |
| AT1G51470 | 32.6281 | 152.762 |
| AT4G39990 | 32.6095 | 27.6728 |
| AT3G27110 | 32.6068 | 22.9563 |
| AT3G06400 | 32.6042 | 42.1274 |
| AT4G27650 | 32.5916 | 30.6287 |
| AT4G01040 | 32.5847 | 17.1303 |
| AT4G12020 | 32.5696 | 13.9837 |
| AT3G17250 | 32.5566 | 55.9091 |
| AT1G03150 | 32.5554 | 27.0875 |
| AT4G36032 | 32.5494 | 6.21787 |
| AT5G40450 | 32.547 | 31.3347 |
| AT3G23660 | 32.5428 | 22.4488 |
| AT1G25540 | 32.52 | 16.1851 |
| AT3G28900 | 32.5153 | 121.802 |
| AT1G54580 | 32.5004 | 109.248 |
| AT5G12290 | 32.4903 | 17.3015 |
| AT4G26630 | 32.4831 | 58.2647 |
| AT5G07820 | 32.4623 | 10.6263 |
| AT5G55860 | 32.461 | 22.5837 |
| AT2G15560 | 32.4454 | 19.6401 |
| AT1G74500 | 32.4425 | 109.861 |
| AT3G58460 | 32.4413 | 25.2539 |
| AT5G06970 | 32.4395 | 36.5348 |
| AT5G51980 | 32.4266 | 40.6911 |
| AT1G55520 | 32.4129 | 24.2018 |
| AT4G40010 | 32.4091 | 1.28034 |
| AT5G16440 | 32.4068 | 63.1593 |
| AT3G49601 | 32.3933 | 30.6314 |
| AT2G36835 | 32.3868 | 21.3156 |
| AT2G41500 | 32.3783 | 32.8677 |
| AT2G25510 | 32.3447 | 4.78721 |
| AT4G28940 | 32.3272 | 100.127 |
| AT2G17980 | 32.3257 | 24.6098 |
| AT5G22355 | 32.3133 | 17.8129 |
| AT1G30810 | 32.3089 | 22.3071 |
| AT2G45430 | 32.2989 | 21.3612 |
| AT5G03670 | 32.2972 | 6.40875 |
| AT2G04430 | 32.2966 | 5.58723 |
| AT5G64220 | 32.2849 | 36.734 |
| AT5G15330 | 32.2728 | 10.7847 |
| AT4G15390 | 32.2645 | 22.7305 |
| AT4G08620 | 32.2429 | 0.056465 |
| AT3G17900 | 32.2408 | 21.112 |
| AT3G59020 | 32.239 | 39.9724 |
| AT5G03455 | 32.2376 | 12.4495 |
| AT5G08640 | 32.2254 | 4.1713 |
| AT4G23420 | 32.2195 | 31.4682 |
| AT5G63350 | 32.2146 | 33.481 |
| AT5G04800 | 32.2033 | 74.6684 |
| AT5G17910 | 32.2005 | 14.0885 |
| AT2G43210 | 32.1739 | 22.2036 |
| AT5G49710 | 32.1737 | 16.3196 |
| AT2G21150 | 32.1636 | 28.4047 |
| AT1G58320 | 32.1595 | 0.951443 |
| AT4G16330 | 32.1442 | 17.1831 |
| AT2G16920 | 32.144 | 9.88216 |
| AT3G07900 | 32.1397 | 0.546227 |
| AT1G54630 | 32.1228 | 101.714 |
| AT3G53260 | 32.1076 | 16.1184 |
| AT1G54080 | 32.0919 | 41.4161 |
| AT5G43420 | 32.0838 | 27.691 |
| AT4G26850 | 32.0837 | 5.78333 |
| AT4G02230 | 32.0766 | 86.6097 |
| AT5G57630 | 32.0369 | 25.2619 |
| AT5G39970 | 32.0089 | 9.49371 |
| AT5G42860 | 31.9927 | 16.6181 |
| AT4G35570 | 31.985 | 112.637 |
| AT5G20840 | 31.9599 | 19.3314 |
| AT4G26100 | 31.9589 | 15.5999 |
| AT5G23380 | 31.9407 | 17.0476 |
| AT5G04090 | 31.9182 | 29.7956 |
| AT1G55020 | 31.9155 | 15.4606 |
| AT3G46510 | 31.8894 | 23.104 |
| AT5G43910 | 31.8878 | 14.5061 |
| AT4G29930 | 31.8805 | 49.4848 |
| AT4G21710 | 31.8797 | 41.5297 |
| AT5G49870 | 31.8792 | 0.309214 |
| AT3G06580 | 31.8582 | 38.6073 |
| AT2G41790 | 31.8164 | 39.6616 |
| AT5G64620 | 31.8113 | 8.6449 |
| AT5G67380 | 31.8074 | 29.4072 |
| AT5G19790 | 31.8033 | 0.776449 |
| AT2G28690 | 31.7945 | 21.4449 |
| AT5G06980 | 31.7942 | 20.7104 |
| AT3G59950 | 31.7157 | 21.7027 |
| AT2G28760 | 31.7042 | 39.8104 |
| AT3G49680 | 31.701 | 32.9085 |
| AT1G10840 | 31.6854 | 73.0347 |
| AT5G60370 | 31.6761 | 14.6699 |
| AT2G23110 | 31.6746 | 46.1922 |
| AT3G59770 | 31.6688 | 21.7931 |
| AT3G46560 | 31.655 | 61.666 |
| AT5G66100 | 31.6502 | 15.4488 |
| AT2G27480 | 31.6343 | 6.86141 |
| AT5G14420 | 31.6332 | 35.6555 |
| AT3G13440 | 31.6251 | 28.9225 |
| AT4G02590 | 31.6206 | 36.8017 |
| AT1G07420 | 31.6197 | 33.2442 |
| AT3G07020 | 31.6132 | 34.9455 |
| AT1G71380 | 31.5975 | 45.2791 |
| AT3G47520 | 31.5937 | 66.5602 |
| AT5G63460 | 31.5911 | 27.5667 |
| AT3G41762 | 31.5833 | 4.81253 |
| AT3G51250 | 31.5825 | 10.9138 |
| AT2G01190 | 31.5797 | 12.3094 |
| AT4G06676 | 31.5605 | 11.6444 |
| AT1G24620 | 31.5412 | 0.102315 |
| AT5G51270 | 31.5351 | 0.198535 |
| AT1G73730 | 31.5223 | 26.6478 |
| AT3G61710 | 31.5186 | 21.6839 |
| AT3G20460 | 31.5123 | 5.47693 |
| AT2G17530 | 31.5117 | 18.9627 |
| AT5G20280 | 31.5106 | 33.6194 |
| AT4G39520 | 31.5015 | 42.9853 |
| AT1G21370 | 31.4973 | 20.7363 |
| AT1G07728 | 31.477 | 15.4102 |
| AT1G79610 | 31.46 | 26.29 |
| AT3G62860 | 31.437 | 14.4283 |
| AT4G10250 | 31.4193 | 50.7501 |
| AT4G34040 | 31.4164 | 20.8796 |
| AT5G07110 | 31.41 | 11.6706 |
| AT5G18610 | 31.4023 | 23.236 |
| AT5G14260 | 31.4004 | 15.9876 |
| AT5G06260 | 31.3987 | 18.1075 |
| AT5G15660 | 31.3791 | 17.3286 |
| AT1G79090 | 31.3642 | 23.4568 |
| AT4G34460 | 31.3622 | 26.5516 |
| AT5G40210 | 31.3616 | 8.469 |
| AT3G01950 | 31.3408 | 27.1199 |
| AT1G01350 | 31.3354 | 31.6592 |
| AT1G56145 | 31.3292 | 25.7673 |
| AT3G01900 | 31.3288 | 15.5803 |
| AT3G27020 | 31.3183 | 14.19 |
| AT3G20290 | 31.3173 | 28.9395 |
| AT4G12545 | 31.3141 | 38.9361 |
| AT5G65925 | 31.3071 | 34.8139 |
| AT4G37720 | 31.3046 | 9.51102 |
| AT1G04970 | 31.3 | 23.5406 |
| AT1G05890 | 31.2975 | 26.2771 |
| AT1G17490 | 31.297 | 37.3618 |
| AT4G04955 | 31.281 | 23.3456 |
| AT1G66570 | 31.2731 | 1.0048 |
| AT4G23460 | 31.2624 | 24.0393 |
| AT2G25690 | 31.262 | 24.0596 |
| AT2G40830 | 31.2619 | 25.9791 |
| AT5G54650 | 31.2613 | 20.68 |
| AT3G17890 | 31.2559 | 12.4177 |
| AT1G49590 | 31.2453 | 24.4633 |
| AT1G76850 | 31.2383 | 26.6892 |
| AT1G47510 | 31.2342 | 141.935 |
| AT3G26000 | 31.2335 | 19.5948 |
| AT3G24200 | 31.2332 | 20.6142 |
| AT4G24590 | 31.2137 | 20.3201 |
| AT1G02880 | 31.1952 | 43.1101 |
| AT3G54210 | 31.1945 | 17.72 |
| AT2G35860 | 31.1922 | 20.2253 |
| AT5G63310 | 31.1655 | 23.9483 |
| AT3G18215 | 31.1561 | 13.6425 |
| AT5G46710 | 31.1446 | 37.189 |
| AT3G18140 | 31.1202 | 19.4808 |
| AT3G22240 | 31.1049 | 17.5711 |
| AT4G36945 | 31.0997 | 13.342 |
| AT1G10390 | 31.0994 | 22.722 |
| AT4G18120 | 31.0908 | 17.5985 |
| AT5G56240 | 31.0683 | 17.8095 |
| AT5G24110 | 31.0568 | 11.0955 |
| AT5G64440 | 31.0341 | 25.2346 |
| AT1G63800 | 31.0328 | 9.52441 |
| AT1G77760 | 31.0166 | 28.3308 |
| AT1G29071 | 31.0144 | 14.9103 |
| AT2G41780 | 31.0083 | 12.5665 |
| AT4G29900 | 30.9981 | 34.4499 |
| AT2G02480 | 30.9978 | 9.47418 |
| AT3G63260 | 30.9931 | 27.834 |
| AT4G02030 | 30.9882 | 21.9066 |
| AT3G59900 | 30.9834 | 48.4431 |
| AT3G14830 | 30.9752 | 24.742 |
| AT1G08680 | 30.9657 | 23.2407 |
| AT2G44140 | 30.9551 | 23.1496 |
| AT3G55450 | 30.9423 | 25.9967 |
| AT1G19110 | 30.9171 | 27.681 |
| AT3G54810 | 30.913 | 35.0828 |
| AT5G66240 | 30.8888 | 27.8525 |
| AT2G45540 | 30.8885 | 18.7524 |
| AT2G25964 | 30.8861 | 20.0469 |
| AT5G48020 | 30.8813 | 34.6775 |
| AT4G35510 | 30.8553 | 21.8439 |
| AT2G42230 | 30.8374 | 17.3474 |
| AT2G31400 | 30.8282 | 29.7934 |
| AT3G29240 | 30.8266 | 3.53292 |
| AT5G53180 | 30.8128 | 22.5672 |
| AT4G37330 | 30.7963 | 25.7196 |
| AT1G74370 | 30.794 | 19.1212 |
| AT5G37540 | 30.7711 | 15.9285 |
| AT3G02260 | 30.7649 | 20.9941 |
| AT5G09250 | 30.7642 | 37.3248 |
| AT5G33355 | 30.7596 | 37.8044 |
| AT1G61620 | 30.7506 | 36.5276 |
| AT1G15420 | 30.7454 | 51.1435 |
| AT5G45100 | 30.7255 | 25.2146 |
| AT5G22630 | 30.7117 | 26.1126 |
| AT4G10770 | 30.6851 | 2.25224 |
| AT4G19040 | 30.6645 | 11.9508 |
| AT4G31180 | 30.6517 | 51.1886 |
| AT5G62580 | 30.639 | 32.1501 |
| AT5G26080 | 30.619 | 0.0592904 |
| AT1G58080 | 30.6157 | 36.4141 |
| AT1G54490 | 30.6042 | 18.3483 |
| AT5G61440 | 30.6006 | 26.561 |
| AT1G79940 | 30.5981 | 30.461 |
| AT5G58620 | 30.5813 | 10.2916 |
| AT1G70000 | 30.5723 | 18.2681 |
| AT1G33780 | 30.5603 | 21.5342 |
| AT3G55920 | 30.5603 | 16.2316 |
| AT2G36060 | 30.5583 | 28.7767 |
| AT2G44370 | 30.5466 | 15.6589 |
| AT4G19490 | 30.5452 | 26.1483 |
| AT1G24350 | 30.5393 | 14.319 |
| AT2G03890 | 30.532 | 19.6595 |
| AT2G22900 | 30.5198 | 48.1996 |
| AT4G35060 | 30.5191 | 3.80277 |
| AT5G05820 | 30.5152 | 23.784 |
| AT5G61260 | 30.5146 | 1.31106 |
| AT3G49720 | 30.5044 | 64.8422 |
| AT2G38000 | 30.4984 | 27.2947 |
| AT4G27600 | 30.4851 | 16.8938 |
| AT4G00755 | 30.476 | 19.652 |
| AT3G02040 | 30.4672 | 51.1225 |
| AT2G23820 | 30.4614 | 28.1548 |
| AT4G15920 | 30.4564 | 14.2251 |
| AT1G73530 | 30.4514 | 17.882 |
| AT3G11590 | 30.4502 | 15.352 |
| AT5G06360 | 30.4496 | 110.168 |
| AT5G61350 | 30.4309 | 0.832928 |
| AT5G11860 | 30.4243 | 24.0797 |
| AT5G19875 | 30.4115 | 40.6697 |
| AT5G63050 | 30.4023 | 26.0752 |
| AT1G43190 | 30.3976 | 28.2779 |
| AT1G15640 | 30.39 | 3.20276 |
| AT1G28540 | 30.3647 | 20.7331 |
| AT1G06530 | 30.3539 | 28.2012 |
| AT2G42140 | 30.3481 | 0.241443 |
| AT5G58590 | 30.3457 | 51.2071 |
| AT5G17310 | 30.3434 | 34.9422 |
| AT2G19460 | 30.3411 | 40.9529 |
| AT1G72130 | 30.3404 | 12.4373 |
| AT4G19006 | 30.3202 | 42.3231 |
| AT5G05570 | 30.3129 | 13.9541 |
| AT2G43040 | 30.2961 | 23.7179 |
| AT1G17050 | 30.2948 | 32.7239 |
| AT4G34660 | 30.2856 | 24.9776 |
| AT1G65510 | 30.285 | 13.7869 |
| AT1G68310 | 30.284 | 44.2533 |
| AT1G60490 | 30.281 | 21.4084 |
| AT5G59870 | 30.2748 | 100.396 |
| AT5G61760 | 30.2743 | 15.6835 |
| AT1G65580 | 30.2668 | 18.3327 |
| AT4G25500 | 30.252 | 58.3452 |
| AT5G45840 | 30.2517 | 0.839383 |
| AT5G21080 | 30.2492 | 0.410728 |
| AT3G30380 | 30.2479 | 27.6259 |
| AT5G10350 | 30.2446 | 38.0153 |
| AT4G23060 | 30.2422 | 21.0401 |
| AT5G63150 | 30.2357 | 52.2233 |
| AT2G06850 | 30.2261 | 48.8542 |
| AT5G47060 | 30.1968 | 31.4285 |
| AT2G33130 | 30.1929 | 2.57585 |
| AT2G04650 | 30.1746 | 14.6036 |
| AT5G42880 | 30.1737 | 12.7177 |
| AT1G66760 | 30.1724 | 35.1258 |
| AT1G01540 | 30.1699 | 26.0511 |
| AT1G02100 | 30.1615 | 28.9876 |
| AT1G62840 | 30.1257 | 1.58387 |
| AT3G46600 | 30.1205 | 30.5301 |
| AT5G09410 | 30.1143 | 38.3271 |
| AT3G15190 | 30.102 | 22.833 |
| AT3G07274 | 30.0896 | 33.8211 |
| AT1G09250 | 30.0765 | 26.7436 |
| AT3G48780 | 30.0631 | 15.8094 |
| AT1G67940 | 30.0499 | 10.8892 |
| AT3G47040 | 30.0456 | 0.168941 |
| AT2G31305 | 30.0429 | 27.82 |
| AT4G25370 | 30.0428 | 32.0863 |
| AT2G26190 | 30.0398 | 30.0068 |
| AT4G08240 | 30.0231 | 15.8256 |
| AT3G15700 | 30.0211 | 0.0358098 |
| AT3G12040 | 30.0115 | 2.9131 |
| AT3G15115 | 30.0093 | 9.96011 |
| AT5G47480 | 29.9989 | 14.7482 |
| AT3G23540 | 29.9912 | 17.271 |
| AT1G27420 | 29.9869 | 3.50773 |
| AT2G22090 | 29.9766 | 19.2266 |
| AT4G27950 | 29.9746 | 27.3063 |
| AT3G09480 | 29.9743 | 39.7124 |
| AT4G18230 | 29.964 | 24.5962 |
| AT1G12770 | 29.9586 | 18.6499 |
| AT3G44280 | 29.9577 | 16.887 |
| AT4G00170 | 29.9521 | 33.9479 |
| AT1G75510 | 29.9517 | 26.0772 |
| AT5G03905 | 29.9393 | 32.4851 |
| AT1G31730 | 29.9328 | 24.1754 |
| AT1G51035 | 29.9289 | 8.36649 |
| AT1G54520 | 29.9058 | 22.3154 |
| AT3G59660 | 29.9052 | 19.4623 |
| AT3G06610 | 29.9011 | 44.5006 |
| AT2G27389 | 29.8847 | 13.8867 |
| AT1G48410 | 29.884 | 36.8615 |
| AT2G28550 | 29.8828 | 8.61754 |
| AT5G09711 | 29.8806 | 2.73796 |
| AT5G48385 | 29.874 | 56.0204 |
| AT1G03457 | 29.8703 | 12.152 |
| AT4G02930 | 29.867 | 68.7868 |
| AT2G37330 | 29.8486 | 3.61676 |
| AT3G01360 | 29.8108 | 15.2617 |
| AT4G38130 | 29.8022 | 81.5549 |
| AT4G16444 | 29.7954 | 12.4395 |
| AT4G28710 | 29.7934 | 9.22395 |
| AT5G45710 | 29.784 | 17.2734 |
| AT1G65660 | 29.7831 | 33.6515 |
| AT2G25160 | 29.749 | 19.6635 |
| AT3G04710 | 29.7435 | 25.7735 |
| AT5G18470 | 29.7412 | 52.7713 |
| AT2G21510 | 29.7385 | 8.32959 |
| AT1G72430 | 29.7269 | 3.62723 |
| AT1G49530 | 29.7262 | 10.1621 |
| AT2G17787 | 29.7248 | 23.9755 |
| AT1G05260 | 29.7138 | 199.823 |
| AT5G14170 | 29.7128 | 36.8309 |
| AT2G25150 | 29.7066 | 8.08563 |
| AT5G57015 | 29.6982 | 34.4809 |
| AT2G10950 | 29.6931 | 17.4282 |
| AT5G56210 | 29.6905 | 11.8781 |
| AT5G05700 | 29.6836 | 14.0743 |
| AT5G45190 | 29.6471 | 27.3626 |
| AT1G77740 | 29.6454 | 17.6783 |
| AT4G17800 | 29.637 | 14.9818 |
| AT3G56275 | 29.6053 | 8.88538 |
| AT1G79910 | 29.603 | 5.17834 |
| AT1G30470 | 29.594 | 25.6984 |
| AT1G32360 | 29.5855 | 26.0535 |
| AT5G60750 | 29.5846 | 19.9363 |
| AT5G20720 | 29.5745 | 98.6472 |
| AT3G02290 | 29.5718 | 16.8612 |
| AT4G10920 | 29.5691 | 25.4094 |
| AT5G09650 | 29.5653 | 57.6 |
| AT5G19480 | 29.5504 | 29.5116 |
| AT2G42010 | 29.5479 | 14.6447 |
| AT3G06810 | 29.5416 | 22.4513 |
| AT4G25550 | 29.5301 | 37.5058 |
| AT4G35200 | 29.5242 | 1.8513 |
| AT2G30530 | 29.5023 | 24.4383 |
| AT1G60170 | 29.4953 | 39.3781 |
| AT4G36790 | 29.4844 | 21.8199 |
| AT5G12210 | 29.4681 | 28.5368 |
| AT2G22910 | 29.4485 | 11.4144 |
| AT1G79320 | 29.4424 | 5.22689 |
| AT1G18180 | 29.4322 | 8.94334 |
| AT2G01350 | 29.4204 | 26.2478 |
| AT3G17205 | 29.4081 | 23.2468 |
| AT2G38860 | 29.3995 | 15.4564 |
| AT3G23400 | 29.391 | 21.6338 |
| AT5G25070 | 29.3885 | 21.7387 |
| AT3G06170 | 29.3765 | 18.7642 |
| AT4G31580 | 29.3709 | 33.9967 |
| AT3G08780 | 29.3667 | 11.425 |
| AT5G64813 | 29.3521 | 26.1789 |
| AT2G19880 | 29.3496 | 27.8779 |
| AT4G30220 | 29.3357 | 96.8378 |
| AT5G23520 | 29.3343 | 22.2181 |
| AT1G69360 | 29.325 | 24.0579 |
| AT5G64120 | 29.2919 | 12.122 |
| AT5G01980 | 29.2821 | 12.5752 |
| AT3G52290 | 29.2816 | 13.2268 |
| AT2G25620 | 29.2705 | 17.3341 |
| AT3G23800 | 29.2415 | 4.6188 |
| AT1G61210 | 29.2216 | 13.073 |
| AT5G53440 | 29.2102 | 22.4654 |
| AT4G34310 | 29.2024 | 16.548 |
| AT5G24360 | 29.198 | 16.7197 |
| AT1G80950 | 29.1809 | 24.0718 |
| AT5G53370 | 29.1557 | 2.06983 |
| AT1G15470 | 29.1532 | 12.7097 |
| AT4G26000 | 29.1297 | 23.5333 |
| AT2G05160 | 29.1287 | 0.614505 |
| AT5G08190 | 29.1195 | 25.4147 |
| AT1G22770 | 29.117 | 17.8176 |
| AT1G19130 | 29.115 | 27.6384 |
| AT5G48580 | 29.0988 | 44.7575 |
| AT1G03060 | 29.0926 | 16.8684 |
| AT3G15395 | 29.0852 | 21.6189 |
| AT1G34300 | 29.0705 | 24.3486 |
| AT2G40380 | 29.0617 | 34.0205 |
| AT5G47960 | 29.0616 | 14.731 |
| AT5G50230 | 29.0586 | 18.9321 |
| AT1G32530 | 29.0389 | 21.5116 |
| AT4G14716 | 29.0369 | 7.0156 |
| AT4G01660 | 29.0317 | 18.8879 |
| AT5G05660 | 29.0253 | 21.6491 |
| AT1G16510 | 29.0176 | 3.83719 |
| AT5G12840 | 29.0131 | 16.2159 |
| AT2G34650 | 28.9916 | 12.4472 |
| AT5G64610 | 28.9837 | 30.4757 |
| AT1G17210 | 28.9733 | 37.518 |
| AT2G19950 | 28.9721 | 19.8977 |
| AT1G07980 | 28.9688 | 34.1966 |
| AT2G16430 | 28.9498 | 10.9355 |
| AT3G24530 | 28.9465 | 21.2917 |
| AT2G29480 | 28.9387 | 39.6526 |
| AT3G52250 | 28.9337 | 18.7925 |
| AT1G32750 | 28.9324 | 25.6455 |
| AT4G05060 | 28.9304 | 16.0679 |
| AT5G05360 | 28.9245 | 19.4471 |
| AT4G02200 | 28.9205 | 21.6752 |
| AT3G01980 | 28.8917 | 9.09412 |
| AT4G17730 | 28.8847 | 46.1734 |
| AT5G47520 | 28.8821 | 16.1703 |
| AT5G64930 | 28.8591 | 15.0193 |
| AT4G18580 | 28.8492 | 21.8981 |
| AT1G05065 | 28.8429 | 0.829409 |
| AT2G26540 | 28.8412 | 19.4159 |
| AT3G05700 | 28.8342 | 12.189 |
| AT2G44090 | 28.834 | 13.8273 |
| AT5G49400 | 28.8292 | 30.5002 |
| AT3G61390 | 28.8102 | 2.77069 |
| AT1G17310 | 28.7973 | 17.0474 |
| AT3G48380 | 28.7969 | 24.8699 |
| AT5G11810 | 28.793 | 26.0762 |
| AT4G29800 | 28.7895 | 0.638612 |
| AT5G66410 | 28.7595 | 20.5226 |
| AT2G03240 | 28.7528 | 30.2116 |
| AT1G32260 | 28.7455 | 7.35315 |
| AT5G16780 | 28.7434 | 22.825 |
| AT4G25640 | 28.7402 | 14.6846 |
| AT2G47115 | 28.7087 | 89.9077 |
| AT4G08410 | 28.7043 | 0.0285677 |
| AT1G22620 | 28.7029 | 12.9851 |
| AT4G00150 | 28.659 | 13.7302 |
| AT5G66420 | 28.652 | 25.3757 |
| AT1G80210 | 28.6466 | 17.4004 |
| AT2G19060 | 28.6291 | 1.04724 |
| AT5G53540 | 28.6088 | 31.4981 |
| AT3G53970 | 28.5973 | 21.6561 |
| AT1G65040 | 28.5955 | 20.7278 |
| AT1G72630 | 28.5791 | 25.179 |
| AT2G34830 | 28.5706 | 4.66111 |
| AT5G03440 | 28.564 | 18.9789 |
| AT1G53165 | 28.5493 | 18.8253 |
| AT1G77140 | 28.5463 | 16.3568 |
| AT5G53970 | 28.5294 | 14.3756 |
| AT5G67340 | 28.5287 | 26.1761 |
| AT1G69270 | 28.5261 | 16.0134 |
| AT2G34300 | 28.5249 | 102.378 |
| AT3G09950 | 28.5247 | 52.5198 |
| AT2G22080 | 28.52 | 30.3405 |
| AT1G71090 | 28.5045 | 10.5134 |
| AT1G53570 | 28.5013 | 18.1001 |
| AT2G01260 | 28.4979 | 12.2997 |
| AT5G51160 | 28.485 | 4.77672 |
| AT3G52240 | 28.4797 | 28.9487 |
| AT3G55600 | 28.4715 | 22.5695 |
| AT5G23720 | 28.4651 | 19.6859 |
| AT5G65620 | 28.4589 | 27.3967 |
| AT5G64180 | 28.4565 | 13.768 |
| AT2G29080 | 28.4553 | 27.032 |
| AT5G51430 | 28.4545 | 27.3346 |
| AT4G05100 | 28.4483 | 14.4606 |
| AT3G19990 | 28.4457 | 13.9931 |
| AT5G49700 | 28.4385 | 16.3055 |
| AT1G61795 | 28.435 | 1.01884 |
| AT4G37880 | 28.4298 | 27.596 |
| AT2G36960 | 28.4207 | 22.0292 |
| AT5G58670 | 28.4206 | 9.47433 |
| AT2G14910 | 28.4184 | 27.3307 |
| AT1G43910 | 28.4085 | 24.8593 |
| AT1G16740 | 28.4055 | 45.5405 |
| AT3G15290 | 28.3966 | 17.746 |
| AT4G16580 | 28.3624 | 16.6866 |
| AT4G12432 | 28.3497 | 20.7137 |
| AT3G26980 | 28.3486 | 41.1903 |
| AT3G55950 | 28.3315 | 12.9513 |
| AT3G13470 | 28.3076 | 41.3001 |
| AT4G33880 | 28.3019 | 0.0166714 |
| AT2G21290 | 28.2964 | 33.3007 |
| AT3G20050 | 28.2907 | 83.6201 |
| AT5G58750 | 28.2834 | 11.1555 |
| AT4G14370 | 28.2606 | 8.26189 |
| AT4G33380 | 28.2591 | 27.4706 |
| AT1G43130 | 28.2507 | 47.37 |
| AT4G18425 | 28.2426 | 7.51435 |
| AT3G56110 | 28.2153 | 16.2187 |
| AT1G76940 | 28.2076 | 23.21 |
| AT4G29270 | 28.2043 | 32.0837 |
| AT5G64920 | 28.1787 | 10.9882 |
| AT1G55535 | 28.1715 | 18.3008 |
| AT1G28050 | 28.1709 | 19.0961 |
| AT3G27160 | 28.17 | 24.2454 |
| AT3G52710 | 28.1665 | 11.7538 |
| AT5G18420 | 28.1468 | 28.6223 |
| AT3G48350 | 28.1464 | 15.6845 |
| AT1G53300 | 28.1391 | 44.6432 |
| AT1G01630 | 28.1378 | 20.6787 |
| AT1G66880 | 28.1356 | 10.2333 |
| AT1G54920 | 28.1344 | 28.6635 |
| AT3G12150 | 28.1302 | 12.5421 |
| AT5G54440 | 28.1249 | 23.0759 |
| AT4G37460 | 28.1233 | 26.9524 |
| AT1G11410 | 28.1055 | 9.1435 |
| AT3G11230 | 28.1045 | 20.9011 |
| AT2G23140 | 28.0964 | 12.4949 |
| AT3G09320 | 28.0958 | 18.0822 |
| AT4G02920 | 28.0926 | 24.5826 |
| AT5G51180 | 28.0923 | 20.1343 |
| AT2G28440 | 28.0882 | 1.40113 |
| AT4G14455 | 28.081 | 29.3726 |
| AT2G35050 | 28.0796 | 20.8527 |
| AT4G01540 | 28.0794 | 21.5276 |
| AT5G20400 | 28.0722 | 39.0132 |
| AT1G01050 | 28.055 | 56.1177 |
| AT3G47836 | 28.0436 | 27.3997 |
| AT3G12640 | 28.0304 | 17.7627 |
| AT3G07600 | 28.0227 | 1.01218 |
| AT1G20225 | 27.9937 | 13.5312 |
| AT3G59290 | 27.9887 | 15.8471 |
| AT5G05520 | 27.9866 | 27.5069 |
| AT4G17720 | 27.9788 | 85.0244 |
| AT1G05785 | 27.9766 | 24.1422 |
| AT1G71020 | 27.9674 | 14.3597 |
| AT5G55480 | 27.9435 | 41.9528 |
| AT1G18980 | 27.905 | 3.97892 |
| AT5G19855 | 27.8964 | 10.158 |
| AT1G17070 | 27.8942 | 14.3718 |
| AT1G77350 | 27.8883 | 43.4156 |
| AT1G05360 | 27.8853 | 21.4826 |
| AT4G10320 | 27.8849 | 46.1737 |
| AT4G14950 | 27.8737 | 25.0208 |
| AT3G02770 | 27.8704 | 36.2716 |
| AT3G32980 | 27.8678 | 46.3738 |
| AT4G29260 | 27.8669 | 9.45206 |
| AT1G61140 | 27.8543 | 24.0706 |
| AT5G42850 | 27.8466 | 25.4172 |
| AT5G51410 | 27.8455 | 23.2995 |
| AT3G52580 | 27.8444 | 95.3416 |
| AT5G13800 | 27.8424 | 31.9602 |
| AT5G16060 | 27.8354 | 25.379 |
| AT2G32070 | 27.8313 | 56.2527 |
| AT5G26780 | 27.8281 | 42.5999 |
| AT5G08590 | 27.8075 | 89.0262 |
| AT2G40510 | 27.8053 | 48.916 |
| AT4G24026 | 27.805 | 3.31551 |
| AT5G05380 | 27.8001 | 5.04465 |
| AT5G53340 | 27.7871 | 32.368 |
| AT1G66600 | 27.7772 | 10.0215 |
| AT4G10080 | 27.7729 | 18.8456 |
| AT1G67980 | 27.7677 | 19.2248 |
| AT3G29000 | 27.7469 | 13.7714 |
| AT3G18170 | 27.7438 | 1.61814 |
| AT5G02280 | 27.7427 | 18.7451 |
| AT4G12600 | 27.7011 | 171.627 |
| AT4G28440 | 27.6988 | 37.9141 |
| AT1G79710 | 27.6721 | 28.2065 |
| AT1G05210 | 27.6676 | 16.1557 |
| AT3G01170 | 27.6592 | 15.6462 |
| AT1G17330 | 27.6454 | 30.5611 |
| AT1G50260 | 27.6396 | 20.2751 |
| AT5G19990 | 27.6372 | 31.786 |
| AT5G09260 | 27.6353 | 17.1743 |
| AT4G25260 | 27.6335 | 110.761 |
| AT3G51880 | 27.6247 | 40.9737 |
| AT1G21580 | 27.624 | 15.0132 |
| AT4G15230 | 27.6221 | 17.0448 |
| AT4G38550 | 27.6152 | 10.8383 |
| AT3G19830 | 27.5903 | 17.7959 |
| AT5G51520 | 27.5604 | 197.649 |
| AT5G08500 | 27.5416 | 25.7115 |
| AT5G45620 | 27.5384 | 56.3849 |
| AT2G04240 | 27.5224 | 26.0745 |
| AT4G16144 | 27.5072 | 13.6519 |
| AT4G21800 | 27.5006 | 23.4625 |
| AT3G16785 | 27.4984 | 14.1835 |
| AT5G01220 | 27.4903 | 26.7558 |
| AT2G27450 | 27.4863 | 29.6448 |
| AT1G62020 | 27.4638 | 28.8769 |
| AT4G17510 | 27.4415 | 33.9091 |
| AT5G62570 | 27.432 | 18.5978 |
| AT1G27700 | 27.4269 | 20.1866 |
| AT1G32370 | 27.4209 | 10.6283 |
| AT1G31120 | 27.419 | 6.69974 |
| AT5G10240 | 27.4071 | 28.8373 |
| AT5G62260 | 27.3837 | 22.9955 |
| AT5G63930 | 27.3711 | 19.5801 |
| AT4G24680 | 27.3684 | 19.7247 |
| AT3G19615 | 27.3647 | 10.609 |
| AT4G29490 | 27.3589 | 19.5188 |
| AT5G51840 | 27.3548 | 22.3343 |
| AT5G60270 | 27.3543 | 21.532 |
| AT4G32660 | 27.3457 | 19.8985 |
| AT5G40200 | 27.3385 | 43.8606 |
| AT4G27585 | 27.3359 | 29.3195 |
| AT5G01340 | 27.3344 | 22.414 |
| AT5G47860 | 27.3187 | 18.3674 |
| AT2G21840 | 27.2894 | 7.1375 |
| AT5G03660 | 27.2885 | 46.6203 |
| AT3G61270 | 27.2833 | 4.41893 |
| AT5G06660 | 27.283 | 27.3756 |
| AT1G22610 | 27.2675 | 19.9682 |
| AT4G39400 | 27.2666 | 45.9494 |
| AT1G71980 | 27.2413 | 19.5553 |
| AT3G57880 | 27.2312 | 33.8983 |
| AT4G21320 | 27.2297 | 23.3014 |
| AT5G19080 | 27.2269 | 12.2706 |
| AT4G17260 | 27.2179 | 24.8677 |
| AT2G32910 | 27.2023 | 17.3701 |
| AT2G21240 | 27.1956 | 22.3794 |
| AT1G51350 | 27.1802 | 13.093 |
| AT4G17410 | 27.1676 | 15.2344 |
| AT3G20500 | 27.1433 | 43.7873 |
| AT4G26840 | 27.1369 | 47.5395 |
| AT1G31780 | 27.1253 | 23.1869 |
| AT5G51780 | 27.1065 | 4.97286 |
| AT3G03740 | 27.1032 | 27.0354 |
| AT3G60250 | 27.0996 | 37.6108 |
| AT4G27880 | 27.0944 | 21.1764 |
| AT4G12770 | 27.0879 | 14.5791 |
| AT1G71190 | 27.082 | 17.5473 |
| AT5G23430 | 27.0625 | 22.9793 |
| AT1G07480 | 27.0619 | 22.7169 |
| AT5G61560 | 27.0559 | 10.7775 |
| AT3G22540 | 27.0551 | 3.79169 |
| AT1G14550 | 27.0548 | 2.90707 |
| AT5G07320 | 27.0463 | 31.3729 |
| AT5G26760 | 27.0418 | 22.2095 |
| AT3G26700 | 27.0415 | 13.5497 |
| AT1G13980 | 27.0389 | 14.3231 |
| AT2G01340 | 27.0325 | 48.1687 |
| AT2G41490 | 27.0325 | 12.105 |
| AT3G03880 | 27.0241 | 30.6759 |
| AT1G23460 | 27.0181 | 16.8675 |
| AT5G16630 | 27.0144 | 19.8453 |
| AT5G67260 | 27.0101 | 30.3015 |
| AT4G35905 | 27.004 | 31.8774 |
| AT2G26800 | 26.9773 | 15.4931 |
| AT5G58530 | 26.9739 | 13.1225 |
| AT5G58350 | 26.97 | 5.48419 |
| AT1G03070 | 26.9696 | 83.7187 |
| AT1G12750 | 26.9651 | 14.0425 |
| AT2G07340 | 26.9568 | 36.5987 |
| AT2G22680 | 26.9372 | 22.8739 |
| AT5G51940 | 26.9274 | 41.0667 |
| AT3G03340 | 26.9168 | 26.0268 |
| AT2G47210 | 26.9164 | 18.8923 |
| AT4G01710 | 26.9114 | 15.2168 |
| AT1G29260 | 26.9102 | 19.6054 |
| AT2G47275 | 26.9055 | 12.3543 |
| AT5G20890 | 26.9035 | 110.703 |
| AT4G02720 | 26.8933 | 26.2961 |
| AT4G18030 | 26.8751 | 119.187 |
| AT5G08660 | 26.8726 | 32.8846 |
| AT4G32640 | 26.8338 | 18.5439 |
| AT5G56360 | 26.814 | 47.0305 |
| AT1G12530 | 26.8138 | 11.6549 |
| AT4G27750 | 26.8078 | 19.4612 |
| AT3G05760 | 26.7984 | 27.5361 |
| AT1G50570 | 26.798 | 23.0996 |
| AT1G06060 | 26.7842 | 22.2326 |
| AT2G38480 | 26.7815 | 6.40336 |
| AT4G12450 | 26.7767 | 6.04734 |
| AT5G42900 | 26.7548 | 21.0968 |
| AT1G51360 | 26.7404 | 7.52311 |
| AT3G45030 | 26.6977 | 113.166 |
| AT2G20940 | 26.676 | 14.6165 |
| AT4G08330 | 26.6693 | 8.1364 |
| AT5G67080 | 26.6646 | 43.2546 |
| AT2G22790 | 26.6624 | 5.7819 |
| AT1G51590 | 26.6402 | 28.0882 |
| AT5G16280 | 26.64 | 17.0738 |
| AT4G19700 | 26.599 | 29.9405 |
| AT2G38310 | 26.5848 | 15.6888 |
| AT5G65100 | 26.583 | 0.65571 |
| AT3G42170 | 26.58 | 34.6515 |
| AT3G04420 | 26.5673 | 20.6071 |
| AT1G77810 | 26.5665 | 14.6469 |
| AT5G57820 | 26.5539 | 9.75705 |
| AT2G39360 | 26.5529 | 3.54205 |
| AT4G28860 | 26.5513 | 13.8867 |
| AT1G54060 | 26.5457 | 20.988 |
| AT1G27210 | 26.5418 | 27.9807 |
| AT1G75660 | 26.5306 | 31.6676 |
| AT1G80410 | 26.5102 | 41.2895 |
| AT3G24840 | 26.5066 | 20.1475 |
| AT1G72200 | 26.4992 | 1.38354 |
| AT1G23960 | 26.4974 | 23.3275 |
| AT2G32930 | 26.4974 | 19.5139 |
| AT4G25080 | 26.4964 | 2.73635 |
| AT5G36940 | 26.4842 | 4.69556 |
| AT1G50500 | 26.4816 | 24.2667 |
| AT3G61420 | 26.4764 | 27.0348 |
| AT3G26510 | 26.4751 | 5.85085 |
| AT2G20450 | 26.4747 | 116.905 |
| AT5G44250 | 26.474 | 21.3434 |
| AT3G01100 | 26.4689 | 27.7823 |
| AT1G03250 | 26.453 | 22.1637 |
| AT5G11720 | 26.4302 | 12.6157 |
| AT5G67330 | 26.4283 | 20.5348 |
| AT5G47910 | 26.4246 | 14.9759 |
| AT3G11100 | 26.4193 | 17.3655 |
| AT1G69900 | 26.4183 | 3.13205 |
| AT1G77510 | 26.4056 | 41.4461 |
| AT2G15480 | 26.4021 | 27.4812 |
| AT3G05090 | 26.4015 | 20.5934 |
| AT2G04550 | 26.3999 | 22.5662 |
| AT5G19340 | 26.3961 | 71.0811 |
| AT1G02380 | 26.3932 | 1.94938 |
| AT3G25760 | 26.3893 | 10.7304 |
| AT4G30890 | 26.3801 | 33.7029 |
| AT1G72900 | 26.361 | 4.57568 |
| AT5G63840 | 26.3586 | 34.2317 |
| AT1G76760 | 26.358 | 15.7414 |
| AT3G60080 | 26.3528 | 12.6752 |
| AT2G32400 | 26.3471 | 21.3807 |
| AT1G19220 | 26.3406 | 8.67235 |
| AT5G03550 | 26.3369 | 5.0834 |
| AT3G59470 | 26.3336 | 18.1005 |
| AT2G33730 | 26.3273 | 29.0407 |
| AT5G39040 | 26.3208 | 58.3968 |
| AT1G34260 | 26.3208 | 16.132 |
| AT5G35690 | 26.3191 | 28.3201 |
| AT2G34660 | 26.3113 | 33.4353 |
| AT5G66530 | 26.2908 | 34.2445 |
| AT3G27390 | 26.285 | 17.7006 |
| AT3G50670 | 26.2697 | 38.2047 |
| AT3G18450 | 26.2681 | 0.378257 |
| AT1G80910 | 26.2649 | 12.449 |
| AT1G09570 | 26.2465 | 32.5547 |
| AT3G01050 | 26.2441 | 33.1253 |
| AT2G07180 | 26.2408 | 23.9319 |
| AT5G06120 | 26.2222 | 15.3325 |
| AT1G54940 | 26.2163 | 0.0381531 |
| AT2G26260 | 26.2137 | 9.2883 |
| AT4G21192 | 26.1987 | 18.8347 |
| AT5G15910 | 26.1939 | 20.7099 |
| AT3G05940 | 26.1882 | 16.8892 |
| AT5G43010 | 26.1833 | 34.431 |
| AT4G35410 | 26.1733 | 18.2 |
| AT1G77300 | 26.1654 | 14.5608 |
| AT5G13260 | 26.164 | 19.0319 |
| AT1G09540 | 26.1597 | 2.41019 |
| AT4G16100 | 26.1483 | 9.46691 |
| AT2G05920 | 26.148 | 28.1199 |
| AT2G07050 | 26.138 | 42.0025 |
| AT2G18915 | 26.1349 | 12.7992 |
| AT5G10740 | 26.1331 | 25.5966 |
| AT1G07660 | 26.0946 | 22.0172 |
| AT2G42400 | 26.0686 | 19.7024 |
| AT3G22440 | 26.063 | 54.9103 |
| AT1G20490 | 26.0584 | 17.6193 |
| AT5G06700 | 26.0526 | 19.1956 |
| AT3G56440 | 26.0424 | 8.91905 |
| AT4G35350 | 26.0374 | 15.2982 |
| AT1G21700 | 26.0352 | 21.3107 |
| AT3G10330 | 26.0241 | 21.7898 |
| AT1G06800 | 26.0207 | 10.4076 |
| AT3G12650 | 26.0081 | 16.6975 |
| AT5G13890 | 25.9954 | 13.0626 |
| AT1G12580 | 25.9933 | 27.8757 |
| AT3G13600 | 25.9897 | 13.4342 |
| AT1G15920 | 25.9836 | 14.4648 |
| AT4G08320 | 25.9781 | 19.3066 |
| AT2G35690 | 25.9739 | 13.4364 |
| AT1G72280 | 25.9607 | 11.5536 |
| AT4G21440 | 25.9516 | 5.37407 |
| AT3G19870 | 25.9462 | 12.4481 |
| AT1G80800 | 25.9456 | 1.43393 |
| AT5G22440 | 25.9392 | 164.293 |
| AT1G17410 | 25.9377 | 13.6846 |
| AT5G11140 | 25.9287 | 10.6952 |
| AT3G19150 | 25.9147 | 28.544 |
| AT5G24735 | 25.9099 | 12.7669 |
| AT1G77920 | 25.8745 | 13.9955 |
| AT2G19310 | 25.873 | 37.1746 |
| AT4G28350 | 25.872 | 11.8471 |
| AT5G09430 | 25.8681 | 1.99078 |
| AT5G18940 | 25.862 | 14.4868 |
| AT1G30110 | 25.8513 | 21.2766 |
| AT4G12610 | 25.8474 | 23.4113 |
| AT2G30120 | 25.8158 | 16.4259 |
| AT2G26830 | 25.7988 | 15.9204 |
| AT3G22250 | 25.7965 | 3.87234 |
| AT5G42110 | 25.7918 | 22.8743 |
| AT2G45060 | 25.782 | 17.4424 |
| AT1G06515 | 25.7817 | 36.9605 |
| AT2G14210 | 25.7774 | 3.01874 |
| AT4G19670 | 25.7772 | 32.0605 |
| AT4G36440 | 25.774 | 14.4602 |
| AT5G01050 | 25.7688 | 2.79978 |
| AT4G32180 | 25.7679 | 18.1523 |
| AT3G14475 | 25.7593 | 6.00441 |
| AT1G15710 | 25.7587 | 9.70868 |
| AT5G46620 | 25.7503 | 32.8188 |
| AT1G29120 | 25.7469 | 8.2233 |
| AT4G24805 | 25.7345 | 13.4158 |
| AT1G17110 | 25.6962 | 30.9981 |
| AT1G68690 | 25.6891 | 34.5536 |
| AT4G02130 | 25.6791 | 15.0528 |
| AT3G46190 | 25.6757 | 0.100786 |
| AT3G56790 | 25.6723 | 9.45787 |
| AT1G52600 | 25.6704 | 51.6541 |
| AT4G33140 | 25.6493 | 20.9522 |
| AT5G59930 | 25.6457 | 11.8733 |
| AT1G62990 | 25.6446 | 8.04854 |
| AT1G77080 | 25.641 | 34.3335 |
| AT1G11820 | 25.613 | 12.0497 |
| AT2G28520 | 25.6008 | 20.9681 |
| AT2G23080 | 25.5945 | 23.5215 |
| AT4G31550 | 25.5943 | 14.4491 |
| AT1G34418 | 25.5908 | 42.4524 |
| AT1G05720 | 25.5883 | 24.4122 |
| AT2G04305 | 25.5755 | 22.9101 |
| AT4G16830 | 25.575 | 28.178 |
| AT5G66010 | 25.5727 | 13.8167 |
| AT4G32190 | 25.5657 | 15.0651 |
| AT1G62820 | 25.5554 | 12.4258 |
| AT2G15910 | 25.5415 | 24.5676 |
| AT5G19020 | 25.5289 | 7.52262 |
| AT1G10430 | 25.5276 | 17.134 |
| AT5G57150 | 25.5244 | 21.5001 |
| AT3G33530 | 25.4956 | 11.8956 |
| AT1G24735 | 25.4884 | 2.03224 |
| AT1G23780 | 25.483 | 20.7461 |
| AT5G16800 | 25.4792 | 20.9141 |
| AT4G25110 | 25.4734 | 0.366441 |
| AT5G07360 | 25.468 | 23.7751 |
| AT1G05710 | 25.4665 | 8.15425 |
| AT2G17280 | 25.46 | 140.086 |
| AT5G47010 | 25.4551 | 21.702 |
| AT3G26950 | 25.4522 | 15.9021 |
| AT1G12990 | 25.4406 | 18.3916 |
| AT2G04160 | 25.4359 | 97.0762 |
| AT4G01050 | 25.4304 | 7.85427 |
| AT3G51790 | 25.4184 | 11.0929 |
| AT4G27480 | 25.4177 | 2.28265 |
| AT5G47310 | 25.413 | 27.4285 |
| AT1G65730 | 25.4127 | 64.5294 |
| AT2G46410 | 25.4033 | 26.9847 |
| AT4G16070 | 25.4008 | 10.0289 |
| AT4G24280 | 25.387 | 45.6733 |
| AT2G33590 | 25.3782 | 25.718 |
| AT2G37980 | 25.3766 | 16.9495 |
| AT2G31670 | 25.3614 | 23.3962 |
| AT2G13650 | 25.3606 | 22.7885 |
| AT2G14100 | 25.3328 | 7.90979 |
| AT3G21750 | 25.327 | 2.88012 |
| AT5G15450 | 25.3239 | 72.1246 |
| AT3G58570 | 25.3129 | 14.7804 |
| AT5G67580 | 25.3122 | 15.9422 |
| AT3G03570 | 25.3102 | 15.0923 |
| AT5G12400 | 25.3084 | 19.5823 |
| AT4G35600 | 25.2979 | 12.5462 |
| AT3G51100 | 25.2887 | 18.9828 |
| AT3G18560 | 25.2787 | 2.44045 |
| AT2G01930 | 25.2778 | 12.5959 |
| AT4G21910 | 25.274 | 20.5025 |
| AT2G25070 | 25.2734 | 30.958 |
| AT5G03430 | 25.2711 | 19.2119 |
| AT3G04930 | 25.2707 | 14.0159 |
| AT4G37900 | 25.2673 | 13.0407 |
| AT3G23750 | 25.2665 | 20.1845 |
| AT2G30105 | 25.259 | 26.1284 |
| AT4G32610 | 25.2524 | 47.6971 |
| AT3G59080 | 25.2509 | 19.6013 |
| AT4G27780 | 25.2476 | 5.79329 |
| AT3G01840 | 25.2385 | 15.4036 |
| AT5G44320 | 25.2295 | 35.2087 |
| AT2G38730 | 25.2248 | 24.3636 |
| AT5G03280 | 25.2242 | 26.1078 |
| AT4G36430 | 25.223 | 20.517 |
| AT5G62880 | 25.2184 | 69.8448 |
| AT5G07100 | 25.2142 | 29.8683 |
| AT2G37520 | 25.2116 | 26.5881 |
| AT1G33240 | 25.2048 | 26.2338 |
| AT5G19980 | 25.2028 | 15.258 |
| AT5G47690 | 25.1927 | 40.617 |
| AT2G37180 | 25.1921 | 9.43978 |
| AT5G38790 | 25.1907 | 6.55392 |
| AT5G48110 | 25.1906 | 40.8693 |
| AT5G19560 | 25.1796 | 0.103618 |
| AT5G19180 | 25.1743 | 20.8867 |
| AT3G57650 | 25.1662 | 33.4736 |
| AT3G61600 | 25.1478 | 20.3119 |
| AT1G64480 | 25.1419 | 2.17402 |
| AT4G29790 | 25.128 | 11.9841 |
| AT5G46040 | 25.1274 | 2.08116 |
| AT4G36720 | 25.1209 | 20.7353 |
| AT5G51700 | 25.1075 | 24.2737 |
| AT1G24267 | 25.0901 | 31.321 |
| AT1G04430 | 25.0784 | 89.0115 |
| AT2G23170 | 25.0764 | 2.52091 |
| AT1G25390 | 25.072 | 9.54529 |
| AT4G03820 | 25.0608 | 12.5139 |
| AT3G20040 | 25.0599 | 11.2058 |
| AT5G41471 | 25.0594 | 11.9421 |
| AT5G03552 | 25.0572 | 26.6709 |
| AT1G47310 | 25.0536 | 17.5067 |
| AT1G50320 | 25.0482 | 9.77487 |
| AT1G58440 | 25.0476 | 24.0107 |
| AT5G28770 | 25.0421 | 10.8921 |
| AT2G32250 | 25.0391 | 20.5074 |
| AT1G53170 | 25.0326 | 19.6134 |
| AT2G37540 | 25.0303 | 17.4072 |
| AT5G08630 | 25.0099 | 18.3158 |
| AT1G29390 | 25.0068 | 10.1543 |
| AT1G03970 | 25.0052 | 15.8584 |
| AT4G00330 | 25.0038 | 7.19712 |
| AT3G47380 | 24.9944 | 82.5882 |
| AT3G11500 | 24.992 | 58.6925 |
| AT5G13560 | 24.9868 | 34.5444 |
| AT3G08910 | 24.9836 | 16.4212 |
| AT3G58600 | 24.983 | 26.1377 |
| AT1G15140 | 24.9766 | 16.7203 |
| AT2G01060 | 24.9656 | 15.7301 |
| AT5G15610 | 24.9609 | 37.0956 |
| AT5G52570 | 24.9589 | 11.0859 |
| AT5G44585 | 24.955 | 68.1733 |
| AT3G21160 | 24.9501 | 22.3225 |
| AT4G27310 | 24.9488 | 19.8182 |
| AT1G52155 | 24.9453 | 2.95257 |
| AT2G33585 | 24.9419 | 18.7822 |
| AT4G04020 | 24.9372 | 12.4126 |
| AT5G49690 | 24.9225 | 18.2385 |
| AT3G08510 | 24.9022 | 23.2801 |
| AT1G52060 | 24.8965 | 336.642 |
| AT1G10580 | 24.8936 | 28.679 |
| AT3G60140 | 24.8912 | 34.3073 |
| AT5G19280 | 24.8898 | 13.4242 |
| AT2G35270 | 24.8569 | 13.1668 |
| AT3G13580 | 24.8403 | 70.5245 |
| AT4G05590 | 24.8354 | 15.3022 |
| AT1G15020 | 24.8126 | 12.4157 |
| AT4G10730 | 24.799 | 24.173 |
| AT1G68450 | 24.7955 | 19.8039 |
| AT1G51600 | 24.7947 | 19.3122 |
| AT3G08850 | 24.786 | 16.4564 |
| AT3G23900 | 24.768 | 20.6141 |
| AT5G32450 | 24.7641 | 33.724 |
| AT5G43600 | 24.7606 | 15.4314 |
| AT3G07300 | 24.759 | 22.8754 |
| AT3G09370 | 24.7583 | 15.0864 |
| AT3G63360 | 24.7553 | 1.16833 |
| AT2G01220 | 24.7518 | 17.2817 |
| AT3G51630 | 24.7474 | 13.9195 |
| AT3G51610 | 24.7461 | 30.9594 |
| AT1G02080 | 24.7413 | 26.749 |
| AT1G80180 | 24.7397 | 6.35601 |
| AT5G59732 | 24.7383 | 20.3534 |
| AT1G17145 | 24.738 | 21.4625 |
| AT1G61040 | 24.7351 | 18.3049 |
| AT5G17990 | 24.7249 | 31.4498 |
| AT1G32490 | 24.7181 | 27.4392 |
| AT4G23550 | 24.711 | 1.67022 |
| AT3G57990 | 24.7005 | 13.4536 |
| AT2G30720 | 24.6938 | 43.0221 |
| AT1G80820 | 24.6743 | 10.1207 |
| AT2G30660 | 24.6728 | 0.377473 |
| AT2G43980 | 24.6612 | 14.1478 |
| AT4G17060 | 24.6496 | 22.4376 |
| AT3G22740 | 24.6335 | 271.882 |
| AT4G25225 | 24.6264 | 14.0503 |
| AT1G22800 | 24.6227 | 17.0805 |
| AT5G10630 | 24.6111 | 17.6509 |
| AT5G35360 | 24.6107 | 114.512 |
| AT1G18390 | 24.6012 | 12.1733 |
| AT5G61240 | 24.5965 | 38.0948 |
| AT5G09330 | 24.5948 | 34.7128 |
| AT1G13300 | 24.5871 | 3.04804 |
| AT5G66060 | 24.5859 | 26.4208 |
| AT5G63500 | 24.5846 | 17.4027 |
| AT2G38960 | 24.5756 | 16.2914 |
| AT2G38670 | 24.5674 | 32.9017 |
| AT1G63830 | 24.5577 | 40.3746 |
| AT4G16260 | 24.5554 | 62.5874 |
| AT5G35560 | 24.549 | 11.7214 |
| AT1G55525 | 24.544 | 30.8468 |
| AT2G36100 | 24.5398 | 22.8939 |
| AT3G12130 | 24.5363 | 37.5819 |
| AT4G37440 | 24.5247 | 21.4966 |
| AT3G13100 | 24.521 | 5.04793 |
| AT5G18480 | 24.5195 | 34.9127 |
| AT1G05630 | 24.5091 | 2.82692 |
| AT3G03180 | 24.5064 | 12.7236 |
| AT1G08420 | 24.4999 | 15.8116 |
| AT3G59380 | 24.4987 | 20.9199 |
| AT2G46940 | 24.4941 | 3.83807 |
| AT1G64560 | 24.4909 | 6.34022 |
| AT2G02250 | 24.4822 | 0.648693 |
| AT5G64360 | 24.4792 | 13.1364 |
| AT5G14105 | 24.4745 | 19.4289 |
| AT2G32390 | 24.4592 | 8.96156 |
| AT1G73010 | 24.4524 | 27.6388 |
| AT1G12230 | 24.4493 | 27.6295 |
| AT3G61140 | 24.443 | 23.8792 |
| AT5G61840 | 24.4425 | 23.5675 |
| AT5G60160 | 24.4421 | 29.5702 |
| AT5G57400 | 24.4393 | 44.0364 |
| AT4G11230 | 24.4326 | 1.37182 |
| AT2G40316 | 24.431 | 11.9836 |
| AT3G15850 | 24.4275 | 18.443 |
| AT5G16900 | 24.4234 | 3.05334 |
| AT1G04210 | 24.4178 | 27.7897 |
| AT5G47435 | 24.4091 | 19.2489 |
| AT4G03020 | 24.4071 | 24.7123 |
| AT5G57460 | 24.398 | 17.439 |
| AT3G22210 | 24.3973 | 19.5615 |
| AT3G53560 | 24.3833 | 16.5272 |
| AT3G15060 | 24.368 | 16.3422 |
| AT5G46180 | 24.3667 | 40.6199 |
| AT3G60800 | 24.3559 | 30.7193 |
| AT3G52055 | 24.3545 | 1.08786 |
| AT5G37720 | 24.3462 | 26.7135 |
| AT1G11905 | 24.339 | 21.5384 |
| AT2G24640 | 24.3224 | 24.6365 |
| AT3G08590 | 24.3008 | 39.4643 |
| AT5G04550 | 24.2993 | 30.1788 |
| AT3G46030 | 24.2875 | 33.6407 |
| AT1G56090 | 24.287 | 15.6114 |
| AT5G07870 | 24.2822 | 41.528 |
| AT1G52560 | 24.2722 | 36.3375 |
| AT3G10810 | 24.2648 | 22.1041 |
| AT2G02730 | 24.2644 | 19.3876 |
| AT5G42820 | 24.2562 | 33.5945 |
| AT5G50720 | 24.2488 | 9.60179 |
| AT1G23450 | 24.2469 | 19.496 |
| AT5G42190 | 24.2386 | 34.5566 |
| AT4G39350 | 24.2274 | 24.7928 |
| AT1G01430 | 24.2192 | 27.247 |
| AT1G49720 | 24.2106 | 22.7035 |
| AT2G05170 | 24.2014 | 11.2467 |
| AT5G14250 | 24.1992 | 30.7376 |
| AT4G15400 | 24.1973 | 5.41439 |
| AT1G30540 | 24.1972 | 11.262 |
| AT5G51380 | 24.1913 | 9.36353 |
| AT4G01140 | 24.1913 | 2.78544 |
| AT1G61570 | 24.1897 | 52.4204 |
| AT2G47070 | 24.1895 | 50.3249 |
| AT5G56260 | 24.1869 | 12.1985 |
| AT2G45380 | 24.1841 | 12.9361 |
| AT4G39850 | 24.1836 | 29.1228 |
| AT2G21540 | 24.1827 | 11.1742 |
| AT4G25220 | 24.1657 | 0.254417 |
| AT1G31817 | 24.1331 | 29.1387 |
| AT2G39550 | 24.1238 | 20.5497 |
| AT5G60980 | 24.1225 | 43.3048 |
| AT5G26940 | 24.1131 | 27.2294 |
| AT5G24460 | 24.1055 | 7.27495 |
| AT1G09580 | 24.0984 | 17.7622 |
| AT1G29195 | 24.0946 | 11.8062 |
| AT4G18890 | 24.0945 | 31.78 |
| AT1G19870 | 24.0832 | 53.5322 |
| AT4G30310 | 24.0797 | 14.672 |
| AT4G39070 | 24.0744 | 2.40985 |
| AT3G03920 | 24.0662 | 81.8627 |
| AT4G28050 | 24.059 | 17.1244 |
| AT3G63180 | 24.0564 | 18.5165 |
| AT5G16300 | 24.0559 | 20.9395 |
| AT3G21610 | 24.0546 | 19.4994 |
| AT3G16480 | 24.0485 | 24.4151 |
| AT2G26590 | 24.037 | 35.0503 |
| AT5G23040 | 24.0306 | 32.3098 |
| AT1G09170 | 24.029 | 0.205787 |
| AT5G45900 | 24.0242 | 14.13 |
| AT2G44230 | 24.0215 | 2.58786 |
| AT4G08555 | 24.0212 | 54.46 |
| AT5G15420 | 24.0171 | 6.23667 |
| AT4G39700 | 24.017 | 31.5995 |
| AT5G19200 | 24.013 | 8.06916 |
| AT5G25450 | 24.0129 | 17.0186 |
| AT5G48610 | 24.0055 | 26.2 |
| AT5G32470 | 23.9933 | 18.8245 |
| AT4G22530 | 23.9903 | 13.3126 |
| AT5G47980 | 23.9769 | 4.01052 |
| AT3G58110 | 23.974 | 25.2825 |
| AT4G25210 | 23.9508 | 38.506 |
| AT2G21630 | 23.9347 | 17.7535 |
| AT5G10720 | 23.9295 | 40.112 |
| AT1G13900 | 23.9254 | 11.5184 |
| AT5G58410 | 23.9252 | 19.0866 |
| AT1G47640 | 23.9231 | 22.3798 |
| AT2G32920 | 23.9205 | 50.8347 |
| AT3G02580 | 23.9039 | 17.6487 |
| AT4G02075 | 23.8967 | 6.96019 |
| AT5G29000 | 23.8862 | 16.4105 |
| AT2G38040 | 23.8833 | 64.6057 |
| AT3G55850 | 23.8754 | 20.188 |
| AT4G29310 | 23.8666 | 5.72436 |
| AT5G03555 | 23.8641 | 7.08281 |
| AT2G41830 | 23.8635 | 11.9241 |
| AT4G34260 | 23.8625 | 37.7582 |
| AT2G43430 | 23.8591 | 16.9619 |
| AT5G37475 | 23.8568 | 31.2997 |
| AT5G11390 | 23.8541 | 19.7566 |
| AT1G60440 | 23.8468 | 15.2407 |
| AT5G48970 | 23.837 | 15.9608 |
| AT5G40000 | 23.8363 | 27.1523 |
| AT1G48950 | 23.8296 | 13.65 |
| AT2G16280 | 23.8115 | 27.1131 |
| AT3G50960 | 23.8093 | 24.244 |
| AT1G28370 | 23.8071 | 4.35025 |
| AT1G32330 | 23.7812 | 13.7842 |
| AT5G46340 | 23.7706 | 9.895 |
| AT3G60850 | 23.7644 | 13.8681 |
| AT5G51740 | 23.7638 | 14.8714 |
| AT4G10480 | 23.7579 | 100.414 |
| AT1G80300 | 23.7549 | 8.4935 |
| AT4G11110 | 23.7504 | 20.1972 |
| AT1G04295 | 23.7186 | 4.8312 |
| AT1G70440 | 23.7183 | 7.54799 |
| AT1G67300 | 23.7133 | 17.373 |
| AT2G31945 | 23.7005 | 43.9073 |
| AT4G25660 | 23.6996 | 11.0486 |
| AT3G23980 | 23.6939 | 17.7315 |
| AT5G67510 | 23.6934 | 33.4367 |
| AT5G10750 | 23.6928 | 7.36058 |
| AT4G00840 | 23.6878 | 14.0721 |
| AT1G07530 | 23.6657 | 26.9868 |
| AT2G11240.1 | 23.6653 | 20.5652 |
| AT1G11000 | 23.6523 | 65.938 |
| AT3G15940 | 23.6516 | 17.9322 |
| AT1G68100 | 23.6514 | 19.2078 |
| AT4G02740 | 23.6417 | 12.7437 |
| AT1G25230 | 23.6341 | 44.4169 |
| AT4G21470 | 23.6242 | 21.7075 |
| AT2G36650 | 23.6239 | 28.0644 |
| AT1G60710 | 23.6146 | 16.0455 |
| AT3G46040 | 23.6042 | 99.0316 |
| AT3G11397 | 23.5966 | 17.9059 |
| AT3G52760 | 23.5866 | 7.83734 |
| AT5G51450 | 23.5765 | 10.8369 |
| AT4G27657 | 23.5732 | 5.72081 |
| AT1G13450 | 23.5731 | 20.1889 |
| AT3G06650 | 23.5636 | 56.869 |
| AT1G06230 | 23.5584 | 27.7701 |
| AT3G03790 | 23.5547 | 16.7485 |
| AT4G17140 | 23.534 | 17.0031 |
| AT5G61150 | 23.5233 | 24.941 |
| AT2G36810 | 23.5163 | 10.1918 |
| AT1G22020 | 23.5159 | 15.1164 |
| AT4G30140 | 23.5062 | 3.15126 |
| AT3G46450 | 23.4921 | 16.0962 |
| AT3G13060 | 23.486 | 22.2128 |
| AT4G04770 | 23.4809 | 23.7395 |
| AT1G22070 | 23.4794 | 12.7874 |
| AT1G07510 | 23.4784 | 13.2968 |
| AT2G18390 | 23.4758 | 30.2058 |
| AT3G61570 | 23.475 | 17.0503 |
| AT1G08050 | 23.4732 | 12.3097 |
| AT3G04680 | 23.4717 | 21.8864 |
| AT1G20200 | 23.4654 | 29.81 |
| AT4G13360 | 23.4596 | 18.6869 |
| AT1G09980 | 23.4457 | 15.4038 |
| AT4G25740 | 23.4433 | 67.5948 |
| AT3G09760 | 23.443 | 12.8864 |
| AT3G60286 | 23.439 | 2.98299 |
| AT1G09100 | 23.4342 | 25.2386 |
| AT3G15352 | 23.4302 | 29.2117 |
| AT1G06090 | 23.4209 | 62.0475 |
| AT1G18570 | 23.4093 | 13.7717 |
| AT2G23980 | 23.3958 | 21.0087 |
| AT1G30300 | 23.3958 | 15.0621 |
| AT2G27950 | 23.3893 | 20.017 |
| AT4G12330 | 23.3883 | 6.06555 |
| AT3G60030 | 23.3791 | 23.9151 |
| AT2G39400 | 23.3521 | 42.5163 |
| AT5G60770 | 23.3093 | 1.76064 |
| AT3G50520 | 23.2998 | 22.5711 |
| AT5G20660 | 23.2785 | 22.1531 |
| AT1G74320 | 23.2778 | 27.996 |
| AT5G13760 | 23.2715 | 12.6245 |
| AT2G23380 | 23.2672 | 22.676 |
| AT1G15950 | 23.25 | 12.8413 |
| AT1G52730 | 23.2437 | 26.622 |
| AT2G29740 | 23.2408 | 1.13071 |
| AT2G26710 | 23.236 | 10.6747 |
| AT1G55460 | 23.2315 | 19.2436 |
| AT2G23940 | 23.2264 | 22.0525 |
| AT5G19350 | 23.2182 | 32.6845 |
| AT3G11710 | 23.2087 | 55.0875 |
| AT5G12250 | 23.2084 | 65.4639 |
| AT1G50360 | 23.2053 | 11.073 |
| AT3G63500 | 23.1791 | 38.309 |
| AT4G14520 | 23.1685 | 6.06817 |
| AT3G52960 | 23.1593 | 31.505 |
| AT3G24190 | 23.1566 | 10.4285 |
| AT1G01090 | 23.1515 | 55.2546 |
| AT3G45630 | 23.1482 | 18.0718 |
| AT2G25280 | 23.146 | 18.2113 |
| AT5G58640 | 23.1434 | 24.7384 |
| AT4G02120 | 23.1412 | 8.06052 |
| AT4G30840 | 23.1223 | 15.1169 |
| AT4G17570 | 23.1125 | 7.05021 |
| AT1G62380 | 23.1062 | 24.8028 |
| AT2G44280 | 23.0948 | 26.248 |
| AT4G08280 | 23.0943 | 13.9002 |
| AT2G38270 | 23.0942 | 22.5534 |
| AT4G38710 | 23.0891 | 16.0448 |
| AT3G18520 | 23.0887 | 25.5194 |
| AT1G70340 | 23.086 | 9.86115 |
| AT5G42150 | 23.085 | 24.7755 |
| AT1G21170 | 23.0661 | 11.3089 |
| AT1G56590 | 23.0628 | 15.2138 |
| AT1G65650 | 23.052 | 25.4585 |
| AT5G18120 | 23.0486 | 27.4121 |
| AT2G26355 | 23.0447 | 16.8873 |
| AT3G58720 | 23.0394 | 7.81559 |
| AT5G22650 | 23.0289 | 100.062 |
| AT3G61930 | 23.0083 | 89.9316 |
| AT2G38340 | 22.9997 | 25.8595 |
| AT3G49490 | 22.9996 | 14.433 |
| AT4G20480 | 22.9976 | 22.6841 |
| AT3G54690 | 22.9871 | 18.3145 |
| AT1G50200 | 22.9828 | 37.9842 |
| AT4G12550 | 22.9767 | 12.1688 |
| AT1G58100 | 22.9726 | 11.6493 |
| AT1G31750 | 22.9725 | 0.195684 |
| AT3G19460 | 22.9698 | 19.0276 |
| AT3G51830 | 22.9655 | 18.2967 |
| AT3G51310 | 22.9621 | 22.5322 |
| AT1G28350 | 22.9537 | 20.8709 |
| AT4G11740 | 22.9403 | 13.8492 |
| AT4G16845 | 22.93 | 25.4421 |
| AT1G42960 | 22.9244 | 84.7081 |
| AT4G08500 | 22.9122 | 15.3415 |
| AT5G25060 | 22.9094 | 19.1419 |
| AT3G05020 | 22.9066 | 121.44 |
| AT5G05750 | 22.8946 | 12.0574 |
| AT5G11640 | 22.892 | 29.1792 |
| AT4G37280 | 22.8908 | 25.0266 |
| AT2G38640 | 22.8906 | 1.70323 |
| AT3G49210 | 22.8859 | 10.4896 |
| AT1G19397 | 22.88 | 5.46617 |
| AT5G07250 | 22.8688 | 8.54836 |
| AT4G34740 | 22.8674 | 8.68136 |
| AT5G14470 | 22.8497 | 7.14908 |
| AT1G26340 | 22.8306 | 21.4017 |
| AT5G17550 | 22.8299 | 24.7447 |
| AT5G17610 | 22.8236 | 26.4654 |
| AT4G01720 | 22.8129 | 4.51053 |
| AT1G24510 | 22.8093 | 122.667 |
| AT3G50110 | 22.809 | 9.82651 |
| AT1G25570 | 22.796 | 16.0339 |
| AT3G19100 | 22.7923 | 50.9387 |
| AT5G49510 | 22.7683 | 62.3262 |
| AT3G12800 | 22.7671 | 32.6748 |
| AT5G52470 | 22.7644 | 64.4985 |
| AT5G41290 | 22.7611 | 2.18897 |
| AT5G04820 | 22.7576 | 3.98028 |
| AT5G42830 | 22.7574 | 3.04995 |
| AT1G71220 | 22.7509 | 40.054 |
| AT1G17520 | 22.7467 | 19.5339 |
| AT1G69680 | 22.7437 | 16.2737 |
| AT1G19650 | 22.7383 | 8.68943 |
| AT4G33070 | 22.7335 | 14.8599 |
| AT2G28080 | 22.7174 | 6.91992 |
| AT5G58690 | 22.7073 | 15.3822 |
| AT5G25520 | 22.6992 | 15.047 |
| AT2G25970 | 22.6985 | 21.2948 |
| AT1G61860 | 22.6968 | 1.88542 |
| AT1G74740 | 22.694 | 5.80925 |
| AT5G53450 | 22.6906 | 17.2258 |
| AT2G21610 | 22.6871 | 62.5818 |
| AT5G50870 | 22.6861 | 41.5889 |
| AT5G19040 | 22.6776 | 2.63966 |
| AT1G72125 | 22.674 | 2.32216 |
| AT3G03890 | 22.6698 | 11.3589 |
| AT3G62560 | 22.6607 | 31.6287 |
| AT5G61670 | 22.6481 | 20.6917 |
| AT4G29690 | 22.6346 | 77.9902 |
| AT5G55710 | 22.6316 | 9.49691 |
| AT5G58005 | 22.6126 | 27.7184 |
| AT4G17620 | 22.6059 | 20.865 |
| AT2G22770 | 22.6026 | 78.5063 |
| AT3G26810 | 22.5996 | 15.4555 |
| AT5G07010 | 22.5978 | 54.1621 |
| AT1G05520 | 22.587 | 26.4797 |
| AT5G35580 | 22.5848 | 3.17853 |
| AT1G53730 | 22.5846 | 13.5753 |
| AT1G23880 | 22.573 | 8.40646 |
| AT4G37240 | 22.5727 | 12.8252 |
| AT3G20560 | 22.5684 | 9.12075 |
| AT3G18350 | 22.5673 | 13.1407 |
| AT2G02130 | 22.5552 | 99.5676 |
| AT1G01180 | 22.5398 | 2.25418 |
| AT5G25110 | 22.5314 | 13.1865 |
| AT5G52990 | 22.5286 | 9.62474 |
| AT2G18790 | 22.5237 | 17.6909 |
| AT4G28880 | 22.518 | 16.5331 |
| AT4G31740 | 22.5139 | 11.1048 |
| AT1G78700 | 22.5094 | 40.4663 |
| AT3G26590 | 22.5086 | 12.8526 |
| AT2G20890 | 22.4957 | 20.5252 |
| AT4G35783 | 22.4939 | 4.06662 |
| AT2G33990 | 22.4906 | 16.2877 |
| AT4G39955 | 22.4869 | 30.4038 |
| AT5G11980 | 22.4769 | 22.5841 |
| AT2G38710 | 22.4686 | 23.4666 |
| AT3G18440 | 22.4604 | 18.9487 |
| AT5G16870 | 22.4535 | 12.9803 |
| AT2G20280 | 22.453 | 28.7962 |
| AT4G10110 | 22.4459 | 17.3842 |
| AT2G20740 | 22.4448 | 14.8788 |
| AT5G65310 | 22.4398 | 5.8317 |
| AT4G37020 | 22.4312 | 15.3629 |
| AT3G05210 | 22.4295 | 9.62639 |
| AT1G65440 | 22.422 | 23.9235 |
| AT5G18640 | 22.4163 | 15.7792 |
| AT3G06070 | 22.4143 | 11.0234 |
| AT1G25260 | 22.3998 | 87.1278 |
| AT3G22480 | 22.3947 | 38.6023 |
| AT5G16260 | 22.3856 | 20.5745 |
| AT5G57580 | 22.3843 | 39.1903 |
| AT3G58050 | 22.3773 | 12.7541 |
| AT5G11040 | 22.377 | 21.8638 |
| AT4G15020 | 22.3715 | 22.0576 |
| AT3G52950 | 22.3644 | 10.9992 |
| AT4G17215 | 22.3471 | 4.07523 |
| AT1G55080 | 22.3456 | 23.0955 |
| AT5G21040 | 22.3416 | 16.8539 |
| AT3G01970 | 22.3337 | 22.4456 |
| AT3G13970 | 22.3329 | 6.48896 |
| AT5G14640 | 22.3288 | 74.1812 |
| AT3G18620 | 22.3215 | 14.4971 |
| AT3G43440 | 22.3113 | 14.4584 |
| AT3G06040 | 22.3094 | 43.6069 |
| AT4G02730 | 22.2985 | 14.9097 |
| AT5G27610 | 22.2971 | 15.6852 |
| AT1G02410 | 22.2894 | 24.3671 |
| AT2G43760 | 22.2834 | 23.1367 |
| AT1G25220 | 22.2763 | 19.0416 |
| AT5G56680 | 22.2654 | 40.4374 |
| AT3G16400 | 22.2624 | 19.0323 |
| AT1G67800 | 22.2364 | 12.3941 |
| AT1G09080 | 22.2283 | 11.1115 |
| AT5G37930 | 22.226 | 11.8689 |
| AT1G54450 | 22.2222 | 6.01481 |
| AT1G07210 | 22.2207 | 43.1083 |
| AT3G07910 | 22.2178 | 37.0418 |
| AT1G12120 | 22.2102 | 16.9946 |
| AT2G43870 | 22.2064 | 58.1351 |
| AT2G47900 | 22.202 | 30.9317 |
| AT4G40045 | 22.1989 | 14.5971 |
| AT1G15280 | 22.1906 | 22.309 |
| AT3G53000 | 22.1853 | 14.4667 |
| AT4G30270 | 22.165 | 1.31669 |
| AT2G33340 | 22.1636 | 57.5146 |
| AT5G47420 | 22.1593 | 16.0743 |
| AT1G28390 | 22.1529 | 16.4007 |
| AT1G17940 | 22.1481 | 13.9296 |
| AT4G35980 | 22.1456 | 28.8063 |
| AT3G13432 | 22.1435 | 5.96833 |
| AT1G72240 | 22.142 | 9.2729 |
| AT2G32600 | 22.1356 | 20.5128 |
| AT3G08690 | 22.1332 | 53.0109 |
| AT1G56140 | 22.1297 | 20.6524 |
| AT1G49030 | 22.1289 | 4.57406 |
| AT1G06640 | 22.1031 | 15.244 |
| AT1G73880 | 22.0899 | 14.784 |
| AT5G45660 | 22.0879 | 18.6466 |
| AT5G10990 | 22.083 | 5.24685 |
| AT5G22030 | 22.0825 | 17.9972 |
| AT1G04990 | 22.0816 | 33.2299 |
| AT1G29060 | 22.0767 | 11.6834 |
| AT1G27090 | 22.0722 | 34.5686 |
| AT5G25560 | 22.0682 | 20.4549 |
| AT5G64900 | 22.0656 | 20.5971 |
| AT3G53390 | 22.0611 | 16.6803 |
| AT4G36195 | 22.0482 | 23.2576 |
| AT1G16440 | 22.044 | 0.524572 |
| AT5G38200 | 22.0414 | 26.8815 |
| AT3G02160 | 22.041 | 17.38 |
| AT4G34135 | 22.0281 | 16.0302 |
| AT1G02750 | 22.0277 | 12.0996 |
| AT5G08450 | 22.0147 | 23.4702 |
| AT3G20660 | 22.0123 | 17.8319 |
| AT5G47140 | 22.0108 | 7.02159 |
| AT2G17120 | 22.0046 | 33.2596 |
| AT5G67540 | 22.003 | 11.2936 |
| AT2G24590 | 21.9986 | 36.2907 |
| AT3G56460 | 21.9937 | 36.6952 |
| AT5G09850 | 21.9873 | 21.4388 |
| AT5G14180 | 21.987 | 46.1886 |
| AT1G32340 | 21.9843 | 10.6433 |
| AT2G18740 | 21.9792 | 95.1869 |
| AT5G67610 | 21.9763 | 11.9265 |
| AT3G02190 | 21.9699 | 35.5096 |
| AT1G52260 | 21.963 | 16.1574 |
| AT5G61590 | 21.9605 | 9.43372 |
| AT2G32580 | 21.958 | 32.0682 |
| AT1G29230 | 21.9501 | 0.435261 |
| AT5G64960 | 21.9462 | 24.5082 |
| AT4G14270 | 21.9455 | 29.5953 |
| AT3G60680 | 21.9444 | 12.2149 |
| AT1G15240 | 21.942 | 21.7972 |
| AT3G09085 | 21.9269 | 12.3487 |
| AT4G25770 | 21.926 | 21.0548 |
| AT4G32680 | 21.9159 | 14.8572 |
| AT4G22910 | 21.9113 | 7.7863 |
| AT1G31870 | 21.8968 | 17.7502 |
| AT2G29400 | 21.8851 | 47.4557 |
| AT1G65020 | 21.8804 | 20.0994 |
| AT4G18940 | 21.8767 | 3.56177 |
| AT1G19120 | 21.8692 | 34.0842 |
| AT5G40460 | 21.8648 | 13.3978 |
| AT1G52420 | 21.859 | 26.7081 |
| AT4G38230 | 21.8516 | 13.6569 |
| AT1G59650 | 21.8416 | 11.9315 |
| AT5G64160 | 21.8305 | 31.6372 |
| AT5G63110 | 21.8268 | 18.6042 |
| AT3G16230 | 21.8256 | 18.4008 |
| AT3G27570 | 21.8237 | 30.5339 |
| AT1G18620 | 21.8158 | 6.46175 |
| AT3G61860 | 21.8075 | 22.8201 |
| AT3G02460 | 21.8039 | 7.78703 |
| AT3G13700 | 21.7998 | 3.82168 |
| AT2G45200 | 21.7875 | 37.7192 |
| AT1G49410 | 21.7843 | 60.946 |
| AT5G35080 | 21.7744 | 19.8552 |
| AT3G59830 | 21.766 | 0.914138 |
| AT3G04650 | 21.7629 | 13.9212 |
| AT5G18910 | 21.7623 | 0.0212668 |
| AT1G19850 | 21.7545 | 61.4566 |
| AT1G50560 | 21.7498 | 16.6883 |
| AT5G24760 | 21.7493 | 16.2244 |
| AT1G62800 | 21.7478 | 7.77989 |
| AT5G43130 | 21.7393 | 21.1246 |
| AT1G22200 | 21.7338 | 29.8424 |
| AT1G10560 | 21.7319 | 23.7292 |
| AT2G21300 | 21.7042 | 24.5132 |
| AT1G48630 | 21.6994 | 106.3 |
| AT3G03940 | 21.6967 | 25.4936 |
| AT4G17420 | 21.6847 | 19.1026 |
| AT5G55640 | 21.6832 | 13.6528 |
| AT1G19600 | 21.6799 | 33.427 |
| AT1G65845 | 21.6604 | 22.2942 |
| AT1G66340 | 21.6592 | 14.3357 |
| AT5G07910 | 21.6574 | 9.29112 |
| AT5G65960 | 21.6542 | 28.3102 |
| AT2G17570 | 21.6201 | 15.6026 |
| AT1G68795 | 21.618 | 27.6791 |
| AT1G20640 | 21.6159 | 13.3602 |
| AT5G24690 | 21.6098 | 28.8923 |
| AT1G28410 | 21.6041 | 16.496 |
| AT3G01400 | 21.6041 | 9.39501 |
| AT3G11950 | 21.5951 | 9.10515 |
| AT3G54850 | 21.5944 | 12.1739 |
| AT1G70570 | 21.5853 | 26.2993 |
| AT3G55150 | 21.5778 | 1.76753 |
| AT3G55520 | 21.56 | 34.0377 |
| AT3G55260 | 21.5374 | 8.18777 |
| AT2G22125 | 21.5305 | 26.7229 |
| AT3G01930 | 21.525 | 16.744 |
| AT5G60120 | 21.5244 | 7.9194 |
| AT5G08180 | 21.5234 | 80.6209 |
| AT5G51130 | 21.5206 | 15.6391 |
| AT5G15820 | 21.5046 | 8.85723 |
| AT1G07940 | 21.4965 | 33.4269 |
| AT5G48870 | 21.4875 | 70.4242 |
| AT2G39410 | 21.486 | 6.84624 |
| AT1G28060 | 21.4792 | 22.404 |
| AT1G24360 | 21.4784 | 69.3303 |
| AT2G29670 | 21.4732 | 30.2579 |
| AT5G62950 | 21.4435 | 24.4267 |
| AT5G11730 | 21.4386 | 16.0502 |
| AT2G15490 | 21.433 | 95.7568 |
| AT5G09225 | 21.4152 | 31.2249 |
| AT3G48195 | 21.4124 | 12.5535 |
| AT4G14550 | 21.4089 | 12.1282 |
| AT3G52030 | 21.4057 | 14.9396 |
| AT5G25810 | 21.4022 | 0.257057 |
| AT1G79730 | 21.3874 | 23.334 |
| AT5G24640 | 21.3747 | 6.09081 |
| AT1G55290 | 21.3654 | 0.487674 |
| AT2G28460 | 21.3637 | 1.21562 |
| AT1G66150 | 21.3323 | 27.6659 |
| AT4G26542 | 21.3301 | 32.077 |
| AT1G21590 | 21.323 | 8.723 |
| AT3G02170 | 21.316 | 35.2273 |
| AT4G17330 | 21.3097 | 33.6419 |
| AT2G36640 | 21.3094 | 13.6112 |
| AT4G33580 | 21.3068 | 37.1697 |
| AT4G22630 | 21.3064 | 0.180932 |
| AT3G52870 | 21.305 | 53.0686 |
| AT1G06010 | 21.3035 | 25.5052 |
| AT1G79600 | 21.2973 | 15.9803 |
| AT1G04340 | 21.2913 | 33.24 |
| AT3G17710 | 21.2893 | 7.45126 |
| AT3G54380 | 21.2816 | 15.3003 |
| AT3G10730 | 21.2733 | 15.1526 |
| AT2G42620 | 21.2676 | 12.8627 |
| AT1G47056 | 21.2673 | 12.5454 |
| AT4G36480 | 21.2617 | 20.4377 |
| AT5G48250 | 21.2601 | 15.0194 |
| AT2G37678 | 21.2549 | 24.9566 |
| AT1G08720 | 21.2433 | 18.7396 |
| AT5G46190 | 21.2425 | 23.265 |
| AT5G44720 | 21.2245 | 26.2962 |
| AT2G39260 | 21.2231 | 19.8481 |
| AT5G15150 | 21.2158 | 30.6366 |
| AT5G54280 | 21.2052 | 11.3944 |
| AT2G42160 | 21.2016 | 15.627 |
| AT4G05420 | 21.2005 | 31.2574 |
| AT4G34800 | 21.1972 | 4.32398 |
| AT5G40740 | 21.1762 | 19.1085 |
| AT4G03550 | 21.1753 | 23.587 |
| AT1G23020 | 21.1704 | 3.19104 |
| AT2G28305 | 21.16 | 9.41417 |
| AT5G51630 | 21.1548 | 16.9448 |
| AT4G28600 | 21.1514 | 19.6519 |
| AT3G54250 | 21.1442 | 38.0742 |
| AT5G24520 | 21.1422 | 17.4426 |
| AT1G61850 | 21.1289 | 10.8478 |
| AT4G09520 | 21.1288 | 12.7728 |
| AT3G14090 | 21.1268 | 15.827 |
| AT2G46020 | 21.1254 | 21.918 |
| AT2G02370 | 21.1184 | 16.9466 |
| AT4G38580 | 21.1178 | 29.939 |
| AT1G71130 | 21.1066 | 20.4665 |
| AT2G22300 | 21.1044 | 44.7642 |
| AT1G74910 | 21.101 | 44.2098 |
| AT1G02130 | 21.0994 | 41.0792 |
| AT5G65880 | 21.0731 | 22.8331 |
| AT4G14110 | 21.07 | 31.6882 |
| AT1G12440 | 21.0659 | 19.2654 |
| AT5G43760 | 21.0644 | 12.0492 |
| AT4G26880 | 21.056 | 56.4067 |
| AT5G14390 | 21.0367 | 13.0056 |
| AT5G23535 | 21.036 | 31.827 |
| AT5G63135 | 21.0342 | 16.6307 |
| AT1G20330 | 21.0123 | 39.2816 |
| AT1G19140 | 21.005 | 23.7427 |
| AT1G60900 | 20.9973 | 18.2672 |
| AT4G38260 | 20.9947 | 8.20076 |
| AT3G63200 | 20.9886 | 6.57083 |
| AT3G12760 | 20.9869 | 41.4369 |
| AT1G68680 | 20.9657 | 22.0314 |
| AT4G05530 | 20.9632 | 22.3392 |
| AT3G63220 | 20.9563 | 8.88342 |
| AT2G43630 | 20.9548 | 26.7415 |
| AT1G21830 | 20.9527 | 30.4527 |
| AT3G15040 | 20.9503 | 12.6247 |
| AT5G13460 | 20.9498 | 21.7411 |
| AT1G27140 | 20.9489 | 0.825657 |
| AT1G06000 | 20.9466 | 7.42428 |
| AT5G50000 | 20.9409 | 45.5459 |
| AT2G33490 | 20.9408 | 11.1612 |
| AT5G06450 | 20.9367 | 16.4994 |
| AT3G28690 | 20.9357 | 18.602 |
| AT5G26770 | 20.9177 | 21.9796 |
| AT1G22910 | 20.9162 | 20.7852 |
| AT4G09650 | 20.9106 | 11.917 |
| AT3G63000 | 20.9021 | 16.3131 |
| AT1G69890 | 20.8987 | 15.568 |
| AT2G47830 | 20.8916 | 6.32791 |
| AT1G12450 | 20.89 | 9.56417 |
| AT2G15280 | 20.8862 | 16.1136 |
| AT1G73200 | 20.8853 | 17.4747 |
| AT3G47610 | 20.8828 | 16.0587 |
| AT3G54650 | 20.8772 | 26.6455 |
| AT4G26455 | 20.8759 | 12.3106 |
| AT5G24850 | 20.8502 | 12.225 |
| AT3G11720 | 20.8497 | 9.2311 |
| AT2G41310 | 20.8408 | 14.5984 |
| AT4G33650 | 20.8379 | 14.8994 |
| AT1G54390 | 20.8357 | 19.5257 |
| AT2G10410.1 | 20.8221 | 24.6839 |
| AT2G41900 | 20.8195 | 18.6841 |
| AT2G38560 | 20.8055 | 25.9836 |
| AT5G02370 | 20.7936 | 13.5621 |
| AT3G16560 | 20.7902 | 6.03176 |
| AT5G13640 | 20.784 | 15.2689 |
| AT3G55460 | 20.7645 | 28.9615 |
| AT4G28550 | 20.7639 | 7.32025 |
| AT5G20260 | 20.7634 | 3.38258 |
| AT1G05380 | 20.7586 | 15.7233 |
| AT3G23830 | 20.7582 | 116.867 |
| AT4G32400 | 20.7572 | 20.9765 |
| AT1G68060 | 20.7526 | 37.6811 |
| AT3G47930 | 20.7478 | 16.5401 |
| AT1G77540 | 20.7399 | 18.4332 |
| AT2G17800 | 20.7245 | 22.5947 |
| AT3G11690 | 20.7242 | 8.07341 |
| AT5G02530 | 20.7183 | 33.0948 |
| AT3G51800 | 20.7088 | 106.78 |
| AT4G34630 | 20.7053 | 21.9772 |
| AT1G12140 | 20.6828 | 6.48309 |
| AT2G11890 | 20.6818 | 30.8632 |
| AT2G47180 | 20.677 | 29.1168 |
| AT5G09400 | 20.6712 | 12.9147 |
| AT4G12570 | 20.6638 | 14.7801 |
| AT4G08350 | 20.6619 | 24.8054 |
| AT2G04135 | 20.6508 | 8.28613 |
| AT5G49550 | 20.6485 | 16.197 |
| AT1G76810 | 20.6467 | 32.5555 |
| AT3G61200 | 20.6401 | 11.4893 |
| AT5G20610 | 20.6131 | 14.343 |
| AT2G44290 | 20.5867 | 2.2888 |
| AT5G19220 | 20.5738 | 3.47471 |
| AT4G29680 | 20.5701 | 17.7542 |
| AT1G73430 | 20.5672 | 26.4603 |
| AT3G06240 | 20.5647 | 14.3303 |
| AT5G43260 | 20.5613 | 11.0763 |
| AT1G05180 | 20.5544 | 21.4097 |
| AT3G51980 | 20.545 | 16.6828 |
| AT5G20960 | 20.5435 | 17.2123 |
| AT3G53710 | 20.5364 | 17.4949 |
| AT3G18860 | 20.5323 | 27.2363 |
| AT1G79280 | 20.5265 | 24.6126 |
| AT2G18250 | 20.5231 | 18.9213 |
| AT5G05987 | 20.4983 | 17.3475 |
| AT3G59930 | 20.4959 | 18.1899 |
| AT5G43230 | 20.4952 | 0.029917 |
| AT4G26480 | 20.494 | 14.5598 |
| AT4G24200 | 20.4926 | 16.2422 |
| AT1G63450 | 20.4915 | 1.37791 |
| AT3G25140 | 20.4846 | 33.3783 |
| AT4G27100 | 20.4804 | 9.52623 |
| AT5G11430 | 20.4762 | 15.4293 |
| AT2G41980 | 20.4745 | 10.433 |
| AT3G26780 | 20.4434 | 21.6273 |
| AT1G33090 | 20.4393 | 0.862959 |
| AT3G06720 | 20.428 | 31.099 |
| AT5G47840 | 20.4171 | 19.1378 |
| AT5G48335 | 20.4166 | 19.791 |
| AT1G27435 | 20.4118 | 26.1127 |
| AT5G13950 | 20.4091 | 17.9958 |
| AT3G13300 | 20.4019 | 22.6908 |
| AT1G64050 | 20.3972 | 28.388 |
| AT1G65010 | 20.3937 | 22.2185 |
| AT3G06180 | 20.3904 | 15.0102 |
| AT3G14870 | 20.379 | 37.6894 |
| AT1G74860 | 20.3785 | 10.4439 |
| AT5G26220 | 20.3759 | 43.9264 |
| AT4G08770 | 20.3746 | 39.0682 |
| AT1G30850 | 20.3706 | 0.0324601 |
| AT1G08890 | 20.3586 | 31.6395 |
| AT1G49670 | 20.3571 | 31.1634 |
| AT2G20750 | 20.3547 | 7.06312 |
| AT1G12410 | 20.3536 | 37.1541 |
| AT5G60300 | 20.3501 | 25.4433 |
| AT3G15470 | 20.3472 | 17.2284 |
| AT3G49430 | 20.3469 | 21.1863 |
| AT2G28890 | 20.3374 | 16.6719 |
| AT3G45620 | 20.3318 | 16.9985 |
| AT1G59865 | 20.3316 | 9.43126 |
| AT4G22150 | 20.3183 | 20.1091 |
| AT4G38540 | 20.3158 | 27.8148 |
| AT2G20920 | 20.3151 | 14.2447 |
| AT5G10130 | 20.3089 | 159.269 |
| AT4G15475 | 20.3074 | 8.17589 |
| AT3G07800 | 20.3034 | 8.92409 |
| AT5G10070 | 20.3033 | 23.1474 |
| AT5G56610 | 20.3032 | 4.68714 |
| AT3G16850 | 20.2977 | 17.859 |
| AT1G20580 | 20.2933 | 59.4659 |
| AT1G03660 | 20.2929 | 0.419252 |
| AT5G50210 | 20.2909 | 25.2811 |
| AT4G35580 | 20.284 | 19.3623 |
| AT3G20790 | 20.2688 | 29.9027 |
| AT4G09630 | 20.2575 | 16.6298 |
| AT4G02440 | 20.2568 | 11.7487 |
| AT4G02980 | 20.2506 | 16.4409 |
| AT3G50640 | 20.2406 | 20.4611 |
| AT3G22980 | 20.2302 | 14.0161 |
| AT3G05980 | 20.2248 | 13.6931 |
| AT1G62600 | 20.218 | 6.20364 |
| AT1G73940 | 20.214 | 49.5882 |
| AT4G22740 | 20.2128 | 21.222 |
| AT2G31140 | 20.1889 | 21.8326 |
| AT5G40190 | 20.1814 | 22.8119 |
| AT5G65050 | 20.1771 | 19.8035 |
| AT1G08970 | 20.1761 | 14.5344 |
| AT3G19770 | 20.1728 | 13.1246 |
| AT5G51290 | 20.1573 | 8.44172 |
| AT1G55500 | 20.1234 | 38.2935 |
| AT4G39880 | 20.1212 | 31.3852 |
| AT5G26830 | 20.1077 | 33.3632 |
| AT3G14930 | 20.0999 | 14.2249 |
| AT4G38480 | 20.0972 | 12.1028 |
| AT5G60820 | 20.0957 | 15.5473 |
| AT2G46070 | 20.0943 | 9.52709 |
| AT2G17680 | 20.0939 | 0.0702565 |
| AT5G23680 | 20.0909 | 21.0186 |
| AT5G64390 | 20.0874 | 11.7463 |
| AT1G71790 | 20.0848 | 18.0671 |
| AT4G31875 | 20.0661 | 5.86968 |
| AT5G10490 | 20.0599 | 15.45 |
| AT3G10850 | 20.0445 | 23.7707 |
| AT1G11930 | 20.0445 | 17.5493 |
| AT2G21950 | 20.0346 | 13.4343 |
| AT5G49920 | 20.0276 | 5.16642 |
| AT4G27740 | 20.0257 | 6.12532 |
| AT3G09920 | 20.0086 | 42.2203 |
| AT5G08770 | 20.0073 | 6.24761 |
| AT2G23620 | 19.9987 | 1.30218 |
| AT5G57860 | 19.9829 | 41.8434 |
| AT3G06370 | 19.9797 | 0.952007 |
| AT4G17750 | 19.9722 | 17.2434 |
| AT1G26945 | 19.9635 | 10.3243 |
| AT3G14410 | 19.9629 | 13.8793 |
| AT2G16700 | 19.9607 | 6.29187 |
| AT3G16940 | 19.9525 | 19.1645 |
| AT1G56630 | 19.9494 | 10.6809 |
| AT3G22320 | 19.9468 | 41.8806 |
| AT1G74360 | 19.9304 | 10.3032 |
| AT3G25540 | 19.9282 | 16.8451 |
| AT1G01930 | 19.9272 | 13.1597 |
| AT2G14170 | 19.9262 | 19.1118 |
| AT1G10810 | 19.9255 | 19.0126 |
| AT2G36290 | 19.9241 | 12.822 |
| AT3G61410 | 19.9168 | 5.70126 |
| AT1G76060 | 19.908 | 12.0308 |
| AT2G37620 | 19.9069 | 22.2351 |
| AT2G26280 | 19.906 | 28.6927 |
| AT1G33520 | 19.9013 | 16.5559 |
| AT4G08400 | 19.887 | 0.0242886 |
| AT1G04530 | 19.8815 | 25.4455 |
| AT2G46830 | 19.881 | 22.6681 |
| AT2G37640 | 19.8782 | 48.824 |
| AT5G17290 | 19.8771 | 13.6056 |
| AT5G45480 | 19.875 | 5.44875 |
| AT5G20000 | 19.8731 | 15.6604 |
| AT5G60860 | 19.8686 | 9.10242 |
| AT5G35110 | 19.8631 | 1.38303 |
| AT1G19880 | 19.8622 | 33.6742 |
| AT1G67930 | 19.862 | 18.1731 |
| AT2G20780 | 19.8619 | 10.9669 |
| AT3G26445 | 19.8581 | 0.957237 |
| AT5G55300 | 19.8566 | 26.5478 |
| AT2G02810 | 19.8392 | 13.0036 |
| AT4G02405 | 19.8227 | 7.12615 |
| AT4G29870 | 19.8208 | 24.4692 |
| AT3G53830 | 19.8172 | 9.03811 |
| AT3G21240 | 19.8165 | 9.06757 |
| AT3G48390 | 19.8156 | 10.011 |
| AT1G33990 | 19.8126 | 30.7561 |
| AT2G19080 | 19.8025 | 31.6618 |
| AT5G14950 | 19.8022 | 9.42214 |
| AT1G54120 | 19.7781 | 25.1305 |
| AT1G65260 | 19.7687 | 17.7234 |
| AT3G13690 | 19.7687 | 6.49005 |
| AT4G33890 | 19.7616 | 17.6127 |
| AT5G58650 | 19.7586 | 2.3792 |
| AT1G07860 | 19.7403 | 4.95516 |
| AT1G54370 | 19.7391 | 13.1924 |
| AT2G41060 | 19.7368 | 14.3554 |
| AT1G63530 | 19.735 | 3.35862 |
| AT2G14110 | 19.7321 | 13.1031 |
| AT3G54366 | 19.7271 | 9.317 |
| AT2G23930 | 19.7257 | 86.8061 |
| AT3G13965 | 19.7231 | 3.50236 |
| AT3G60220 | 19.7158 | 18.804 |
| AT5G20730 | 19.7131 | 10.6435 |
| AT3G58120 | 19.7119 | 23.6342 |
| AT1G06900 | 19.7105 | 31.304 |
| AT1G58235 | 19.7101 | 15.7488 |
| AT1G47600 | 19.7017 | 108.261 |
| AT3G25120 | 19.6993 | 16.4334 |
| AT1G18830 | 19.6928 | 10.5695 |
| AT5G02810 | 19.6841 | 24.0944 |
| AT5G06160 | 19.6737 | 28.4335 |
| AT4G25160 | 19.6735 | 0.418077 |
| AT5G13570 | 19.6715 | 25.4398 |
| AT1G09000 | 19.6614 | 3.0553 |
| AT5G14150 | 19.6594 | 7.49199 |
| AT4G27840 | 19.655 | 8.66998 |
| AT4G38090 | 19.652 | 15.5846 |
| AT3G60240 | 19.6504 | 26.372 |
| AT1G17470 | 19.6453 | 31.2484 |
| AT5G35430 | 19.6382 | 20.3975 |
| AT1G10130 | 19.6376 | 18.1321 |
| AT1G64980 | 19.6373 | 54.7721 |
| AT1G19270 | 19.6364 | 12.0796 |
| AT3G62330 | 19.6154 | 24.1642 |
| AT5G18410 | 19.615 | 18.3472 |
| AT3G17650 | 19.6092 | 9.66766 |
| AT3G04740 | 19.6035 | 13.1155 |
| AT4G25940 | 19.5987 | 0.246851 |
| AT3G02350 | 19.5958 | 36.6218 |
| AT1G11860 | 19.5886 | 57.3918 |
| AT1G36380 | 19.5826 | 13.9043 |
| AT4G13235 | 19.5765 | 0.951116 |
| AT1G68160 | 19.5734 | 13.3069 |
| AT1G05790 | 19.569 | 10.5641 |
| AT1G17750 | 19.5475 | 24.5246 |
| AT3G62840 | 19.5392 | 90.2324 |
| AT4G22745 | 19.5287 | 10.3907 |
| AT3G28200 | 19.5234 | 35.4308 |
| AT3G02550 | 19.5221 | 30.1583 |
| AT3G18480 | 19.5037 | 22.7771 |
| AT5G47320 | 19.5029 | 37.9426 |
| AT1G64572 | 19.5016 | 10.0375 |
| AT3G45240 | 19.4981 | 11.1023 |
| AT5G28740 | 19.4919 | 25.5943 |
| AT1G45160 | 19.4899 | 18.3127 |
| AT1G75960 | 19.483 | 3.57696 |
| AT1G13560 | 19.4787 | 48.2947 |
| AT4G30830 | 19.4482 | 10.8579 |
| AT5G12430 | 19.4435 | 9.08701 |
| AT3G19553 | 19.4375 | 11.7661 |
| AT3G45190 | 19.421 | 21.7128 |
| AT1G10020 | 19.4171 | 2.30678 |
| AT2G17760 | 19.416 | 26.3534 |
| AT4G21600 | 19.4085 | 20.0667 |
| AT5G01215 | 19.4062 | 1.17744 |
| AT4G12060 | 19.3993 | 16.8704 |
| AT3G45300 | 19.393 | 24.5696 |
| AT1G01640 | 19.3803 | 8.61394 |
| AT2G47460 | 19.3744 | 26.5378 |
| AT1G75810 | 19.3714 | 5.63276 |
| AT5G03330 | 19.3709 | 43.5725 |
| AT5G49210 | 19.3695 | 31.4256 |
| AT4G14385 | 19.3292 | 19.9857 |
| AT2G28200 | 19.3203 | 3.78208 |
| AT1G03475 | 19.3182 | 22.2506 |
| AT1G23310 | 19.3159 | 18.3175 |
| AT1G71440 | 19.312 | 19.7692 |
| AT3G06540 | 19.3018 | 18.3777 |
| AT1G15200 | 19.2988 | 26.0445 |
| AT4G10930 | 19.2964 | 15.5248 |
| AT1G29800 | 19.2963 | 10.4202 |
| AT3G14172 | 19.2958 | 15.5686 |
| AT5G64870 | 19.2939 | 17.9787 |
| AT3G10670 | 19.2865 | 21.5238 |
| AT5G54900 | 19.274 | 24.9939 |
| AT2G04390 | 19.271 | 55.3886 |
| AT3G19680 | 19.2705 | 11.7929 |
| AT5G62190 | 19.2704 | 83.3518 |
| AT5G62050 | 19.2632 | 24.7276 |
| AT1G23800 | 19.2496 | 32.7054 |
| AT4G12050 | 19.2419 | 11.177 |
| AT3G19190 | 19.2341 | 10.0011 |
| AT3G58170 | 19.2332 | 26.0574 |
| AT1G29965 | 19.2236 | 32.0613 |
| AT1G71270 | 19.2106 | 16.8839 |
| AT5G09960 | 19.2076 | 17.6702 |
| AT4G30996 | 19.206 | 29.5876 |
| AT2G22120 | 19.2009 | 16.7301 |
| AT2G27900 | 19.185 | 16.1436 |
| AT2G31560 | 19.1827 | 11.5135 |
| AT1G21730 | 19.1823 | 11.4959 |
| AT2G14095 | 19.181 | 8.97443 |
| AT3G16530 | 19.1794 | 7.11834 |
| AT4G22070 | 19.1717 | 1.20887 |
| AT2G27200 | 19.1713 | 14.4454 |
| AT4G33210 | 19.1432 | 22.2791 |
| AT1G08780 | 19.142 | 53.5117 |
| AT3G26030 | 19.1419 | 21.4227 |
| AT2G21850 | 19.14 | 6.79004 |
| AT1G70140 | 19.1307 | 13.8177 |
| AT4G01810 | 19.127 | 18.74 |
| AT3G02720 | 19.1086 | 43.8207 |
| AT4G38200 | 19.0989 | 12.3936 |
| AT1G79570 | 19.0967 | 21.0796 |
| AT5G24230 | 19.0721 | 1.58035 |
| AT5G17510 | 19.0648 | 14.9067 |
| AT1G71360 | 19.0522 | 10.5326 |
| AT4G00830 | 19.0505 | 34.6233 |
| AT5G25270 | 19.0476 | 12.9816 |
| AT1G70420 | 19.0445 | 18.56 |
| AT1G48420 | 19.0413 | 22.1495 |
| AT1G51800 | 19.0238 | 4.8626 |
| AT1G80020 | 19.0225 | 18.0146 |
| AT1G71865 | 19.015 | 13.2609 |
| AT3G22170 | 19.0104 | 23.4397 |
| AT3G13672 | 19.0051 | 11.7266 |
| AT4G25810 | 18.9859 | 10.1089 |
| AT1G21670 | 18.9844 | 11.5039 |
| AT5G15730 | 18.9829 | 10.5198 |
| AT4G01575 | 18.9756 | 10.2024 |
| AT2G35530 | 18.9632 | 14.2564 |
| AT2G17350 | 18.9465 | 20.6797 |
| AT1G72340 | 18.9453 | 13.5663 |
| AT1G77250 | 18.9421 | 22.1071 |
| AT1G01660 | 18.9362 | 0.0977191 |
| AT4G14230 | 18.9351 | 8.75896 |
| AT3G10390 | 18.9211 | 19.4999 |
| AT1G62730 | 18.9182 | 14.5101 |
| AT5G08410 | 18.9107 | 12.4382 |
| AT4G15780 | 18.909 | 44.3074 |
| AT2G30800 | 18.9059 | 16.25 |
| AT5G65810 | 18.8769 | 40.4609 |
| AT1G20850 | 18.8715 | 9.83439 |
| AT1G75000 | 18.8688 | 10.5277 |
| AT1G05010 | 18.8498 | 92.2947 |
| AT4G00895 | 18.848 | 13.9552 |
| AT3G09032 | 18.8447 | 11.4993 |
| AT2G42750 | 18.8349 | 10.6268 |
| AT5G27380 | 18.8307 | 30.7146 |
| AT5G08120 | 18.8306 | 24.1893 |
| AT1G80930 | 18.8242 | 36.0923 |
| AT1G75394 | 18.8241 | 0.114842 |
| AT4G37220 | 18.8171 | 1.47407 |
| AT5G21010 | 18.8114 | 17.7741 |
| AT1G25380 | 18.806 | 16.2457 |
| AT2G28160 | 18.8058 | 4.52206 |
| AT1G80160 | 18.8051 | 8.71544 |
| AT2G14900 | 18.8007 | 41.1592 |
| AT1G17580 | 18.7978 | 23.6176 |
| AT2G15320 | 18.7968 | 10.064 |
| AT2G44380 | 18.7913 | 2.79727 |
| AT5G04240 | 18.7893 | 15.4431 |
| AT1G67410 | 18.7843 | 10.027 |
| AT1G23860 | 18.7833 | 13.2287 |
| AT4G34890 | 18.7828 | 15.4237 |
| AT3G53410 | 18.7754 | 6.45322 |
| AT1G51620 | 18.7737 | 4.50864 |
| AT4G37432 | 18.7606 | 6.24577 |
| AT4G34810 | 18.7507 | 13.3976 |
| AT1G55620 | 18.7476 | 14.1481 |
| AT3G61990 | 18.745 | 6.11433 |
| AT2G42700 | 18.7347 | 15.2182 |
| AT3G55480 | 18.7338 | 13.1768 |
| AT2G34790 | 18.7249 | 11.7763 |
| AT5G61980 | 18.7205 | 7.15797 |
| AT2G39340 | 18.7108 | 13.5578 |
| AT1G33360 | 18.7054 | 29.022 |
| AT5G46700 | 18.7018 | 81.9476 |
| AT5G06750 | 18.6987 | 25.4525 |
| AT2G42670 | 18.6877 | 10.7612 |
| AT1G20920 | 18.6853 | 14.1006 |
| AT2G30880 | 18.6815 | 19.3577 |
| AT4G02510 | 18.6793 | 20.0241 |
| AT4G19180 | 18.6676 | 11.8979 |
| AT1G78230 | 18.663 | 1.39465 |
| AT2G04100 | 18.6607 | 8.16424 |
| AT4G13890 | 18.6517 | 5.72393 |
| AT1G35220 | 18.6492 | 10.278 |
| AT1G21610 | 18.6339 | 13.2534 |
| AT1G28340 | 18.6182 | 13.2699 |
| AT5G24500 | 18.613 | 14.2811 |
| AT1G69220 | 18.6094 | 19.1474 |
| AT1G69370 | 18.6048 | 12.8458 |
| AT1G43860 | 18.6037 | 17.0674 |
| AT1G07470 | 18.5935 | 16.1622 |
| AT5G09830 | 18.5881 | 37.2375 |
| AT3G18690 | 18.5835 | 10.375 |
| AT1G30825 | 18.5712 | 9.37742 |
| AT2G18350 | 18.5596 | 8.47505 |
| AT1G03380 | 18.5489 | 10.6813 |
| AT1G55690 | 18.544 | 22.9135 |
| AT2G33410 | 18.5434 | 27.4133 |
| AT4G01940 | 18.5433 | 12.7874 |
| AT4G01250 | 18.543 | 37.5269 |
| AT3G05290 | 18.5403 | 24.9389 |
| AT1G55730 | 18.5387 | 14.3485 |
| AT1G19730 | 18.5387 | 9.74453 |
| AT3G56060 | 18.538 | 30.1888 |
| AT1G79930 | 18.5379 | 19.2897 |
| AT5G59510 | 18.5372 | 33.0807 |
| AT3G12012 | 18.5322 | 18.6075 |
| AT5G37750 | 18.5309 | 5.15544 |
| AT3G06620 | 18.5263 | 9.20771 |
| AT4G25840 | 18.5261 | 21.5882 |
| AT5G45472 | 18.5247 | 5.19783 |
| AT4G28980 | 18.522 | 14.4594 |
| AT2G31510 | 18.5194 | 16.3073 |
| AT5G63980 | 18.513 | 45.0875 |
| AT1G61280 | 18.5008 | 12.3775 |
| AT3G24820 | 18.4989 | 15.8898 |
| AT1G15700 | 18.4975 | 11.0341 |
| AT5G15020 | 18.489 | 17.0718 |
| AT4G30980 | 18.4855 | 3.01509 |
| AT3G14470 | 18.4803 | 2.26929 |
| AT4G33510 | 18.4763 | 10.8035 |
| AT4G24730 | 18.4733 | 10.928 |
| AT2G27090 | 18.4668 | 19.8083 |
| AT2G47580 | 18.4639 | 26.9333 |
| AT5G10930 | 18.4612 | 6.07835 |
| AT2G44660 | 18.4605 | 7.70433 |
| AT3G21220 | 18.457 | 18.787 |
| AT1G67680 | 18.45 | 16.2022 |
| AT5G01500 | 18.4493 | 14.5694 |
| AT3G03960 | 18.4491 | 86.9818 |
| AT5G57050 | 18.4448 | 7.68494 |
| AT2G39100 | 18.4401 | 15.0736 |
| AT3G22990 | 18.4358 | 18.9701 |
| AT2G47760 | 18.4349 | 14.5089 |
| AT2G39450 | 18.4339 | 38.6164 |
| AT2G46150 | 18.4302 | 11.5428 |
| AT2G45640 | 18.4281 | 50.9979 |
| AT4G10100 | 18.4205 | 10.5389 |
| AT1G29040 | 18.4173 | 15.0233 |
| AT4G17040 | 18.4077 | 35.5912 |
| AT2G44065 | 18.4071 | 23.2437 |
| AT2G14720 | 18.4047 | 33.8432 |
| AT3G01730 | 18.3959 | 0.130037 |
| AT1G63050 | 18.3913 | 24.1502 |
| AT1G25520 | 18.384 | 14.2534 |
| AT3G16720 | 18.3792 | 11.9069 |
| AT3G21200 | 18.3681 | 10.2496 |
| AT5G39380 | 18.3654 | 32.6055 |
| AT1G25420 | 18.3548 | 20.7071 |
| AT2G26390 | 18.3546 | 14.992 |
| AT4G22000 | 18.3545 | 48.5731 |
| AT4G07990 | 18.3512 | 10.3316 |
| AT5G06839 | 18.3423 | 1.49624 |
| AT2G45315 | 18.3278 | 12.1284 |
| AT3G18230 | 18.3248 | 21.9519 |
| AT2G16770 | 18.3224 | 5.24778 |
| AT3G28600 | 18.3181 | 31.5942 |
| AT3G49860 | 18.318 | 2.61222 |
| AT4G26700 | 18.3111 | 32.6885 |
| AT2G30270 | 18.306 | 18.1315 |
| AT5G51990 | 18.3051 | 5.7372 |
| AT5G24020 | 18.2896 | 12.4808 |
| AT5G07730 | 18.2837 | 11.8251 |
| AT2G05220 | 18.2797 | 37.6379 |
| AT4G01897 | 18.2761 | 25.0616 |
| AT3G18190 | 18.2622 | 80.6411 |
| AT3G24440 | 18.2469 | 13.5464 |
| AT3G01810 | 18.2464 | 8.47447 |
| AT5G43190 | 18.2434 | 12.814 |
| AT3G08990 | 18.236 | 29.2109 |
| AT2G47890 | 18.2343 | 8.42199 |
| AT4G33300 | 18.2279 | 23.2541 |
| AT3G09020 | 18.227 | 1.26062 |
| AT2G45500 | 18.2228 | 17.1626 |
| AT1G19350 | 18.2176 | 21.5063 |
| AT5G47090 | 18.2173 | 22.8496 |
| AT4G01550 | 18.2169 | 7.86108 |
| AT3G50790 | 18.2137 | 11.3871 |
| AT5G15400 | 18.2114 | 20.7894 |
| AT4G34240 | 18.209 | 14.9909 |
| AT1G53390 | 18.197 | 10.316 |
| AT5G36925 | 18.1895 | 14.8412 |
| AT5G02840 | 18.189 | 21.5131 |
| AT1G68340 | 18.1884 | 12.1246 |
| AT3G52100 | 18.1832 | 14.3255 |
| AT1G57540 | 18.1803 | 15.5477 |
| AT1G17440 | 18.1694 | 20.0836 |
| AT5G10790 | 18.1494 | 13.331 |
| AT4G10390 | 18.135 | 12.3977 |
| AT5G38100 | 18.1307 | 9.17249 |
| AT1G75020 | 18.1306 | 11.9962 |
| AT5G04600 | 18.1238 | 68.5986 |
| AT3G22300 | 18.1233 | 32.0013 |
| AT2G46040 | 18.1174 | 11.1908 |
| AT2G32220 | 18.1117 | 12.8249 |
| AT4G04960 | 18.096 | 11.7766 |
| AT1G01440 | 18.0843 | 6.3126 |
| AT3G10090 | 18.0779 | 92.1006 |
| AT3G14790 | 18.0767 | 27.0484 |
| AT1G24150 | 18.0755 | 4.14474 |
| AT1G53060 | 18.0751 | 15.9125 |
| AT5G28020 | 18.0745 | 29.7011 |
| AT3G11450 | 18.0743 | 25.5381 |
| AT3G13224 | 18.0732 | 19.5533 |
| AT1G80900 | 18.0583 | 8.03253 |
| AT1G79350 | 18.0543 | 22.0904 |
| AT1G79670 | 18.0543 | 12.0253 |
| AT2G19340 | 18.0337 | 10.8031 |
| AT1G76580 | 18.0333 | 14.2112 |
| AT3G11570 | 18.0289 | 15.8485 |
| AT5G23130 | 18.0269 | 13.7815 |
| AT5G05210 | 18.0145 | 32.316 |
| AT4G36980 | 18.0135 | 18.7017 |
| AT1G74780 | 18.0087 | 3.96505 |
| AT2G11891 | 17.9928 | 31.2947 |
| AT5G55200 | 17.9909 | 15.455 |
| AT2G25737 | 17.9903 | 8.69095 |
| AT4G10370 | 17.9892 | 3.26055 |
| AT3G62370 | 17.9785 | 14.8681 |
| AT3G52105 | 17.9784 | 8.95503 |
| AT4G25280 | 17.9659 | 27.6479 |
| AT1G17120 | 17.9561 | 11.2058 |
| AT1G12150 | 17.9559 | 0.861049 |
| AT1G27500 | 17.9537 | 6.81706 |
| AT4G17350 | 17.948 | 9.5063 |
| AT1G15520 | 17.9469 | 56.2606 |
| AT5G17300 | 17.9431 | 13.4924 |
| AT5G46295 | 17.9428 | 4.68336 |
| AT5G45370 | 17.9421 | 5.02035 |
| AT4G32560 | 17.9344 | 11.5023 |
| AT1G65890 | 17.9297 | 7.43053 |
| AT3G50690 | 17.92 | 21.0033 |
| AT2G35330 | 17.9137 | 16.2619 |
| AT2G30980 | 17.9086 | 34.3767 |
| AT1G02860 | 17.9035 | 22.4365 |
| AT2G04690 | 17.8986 | 30.5929 |
| AT5G23150 | 17.897 | 11.5991 |
| AT1G51540 | 17.893 | 19.2827 |
| AT1G70310 | 17.8905 | 36.2303 |
| AT1G72520 | 17.8889 | 13.1889 |
| AT2G37820 | 17.8879 | 0.221005 |
| AT1G16020 | 17.8717 | 12.1618 |
| AT4G13730 | 17.8622 | 17.3778 |
| AT2G27285 | 17.8577 | 19.5427 |
| AT4G31880 | 17.8477 | 25.7129 |
| AT4G25410 | 17.8439 | 8.07179 |
| AT5G30490 | 17.8432 | 13.0001 |
| AT1G48840 | 17.8422 | 13.1371 |
| AT3G07660 | 17.8355 | 19.148 |
| AT5G03610 | 17.8353 | 42.5939 |
| AT2G45685 | 17.8296 | 10.7814 |
| AT2G40090 | 17.8288 | 20.0126 |
| AT1G70090 | 17.8181 | 18.8474 |
| AT5G62720 | 17.815 | 1.04459 |
| AT3G52820 | 17.8125 | 16.935 |
| AT1G49860 | 17.8026 | 3.87911 |
| AT1G01490 | 17.8005 | 26.0208 |
| AT3G13340 | 17.8002 | 31.6822 |
| AT1G13180 | 17.7824 | 16.0229 |
| AT3G20970 | 17.7786 | 16.2018 |
| AT5G25460 | 17.7753 | 54.5325 |
| AT5G20070 | 17.7732 | 25.533 |
| AT1G69250 | 17.773 | 38.1886 |
| AT5G60190 | 17.7715 | 12.504 |
| AT3G51750 | 17.766 | 25.4097 |
| AT3G61740 | 17.7617 | 15.4272 |
| AT1G34350 | 17.7545 | 28.1211 |
| AT1G14020 | 17.7505 | 13.3361 |
| AT3G49580 | 17.7457 | 9.99056 |
| AT4G14465 | 17.7409 | 2.88216 |
| AT3G55000 | 17.7193 | 18.1082 |
| AT4G05120 | 17.7142 | 34.7209 |
| AT5G48760 | 17.7102 | 54.3328 |
| AT1G07160 | 17.7078 | 9.30231 |
| AT3G51010 | 17.6979 | 31.1305 |
| AT2G36360 | 17.696 | 11.0972 |
| AT3G11670 | 17.6876 | 13.5894 |
| AT3G15610 | 17.6847 | 15.3182 |
| AT1G16920 | 17.6727 | 65.4491 |
| AT4G35230 | 17.6643 | 23.2892 |
| AT3G48090 | 17.662 | 12.3278 |
| AT1G70740 | 17.6617 | 14.8628 |
| AT4G27120 | 17.6555 | 18.0995 |
| AT5G14710 | 17.6554 | 19.2723 |
| AT1G47750 | 17.6554 | 16.3927 |
| AT1G16300 | 17.6024 | 21.2378 |
| AT5G57480 | 17.601 | 15.2521 |
| AT2G15860 | 17.5959 | 30.1063 |
| AT3G58490 | 17.5952 | 14.3569 |
| AT5G02160 | 17.5861 | 9.5804 |
| AT5G56880 | 17.5834 | 6.63038 |
| AT1G11760 | 17.5806 | 15.554 |
| AT3G58580 | 17.5719 | 17.3387 |
| AT5G26710 | 17.5633 | 36.0555 |
| AT1G29050 | 17.5531 | 8.93632 |
| AT4G01650 | 17.5458 | 23.1349 |
| AT5G09620 | 17.5331 | 12.6598 |
| AT2G16760 | 17.5319 | 1.50405 |
| AT4G25970 | 17.5283 | 23.6655 |
| AT3G53570 | 17.5222 | 9.85638 |
| AT4G00710 | 17.5075 | 43.1594 |
| AT1G47840 | 17.5068 | 15.2562 |
| AT1G50090 | 17.5034 | 3.8121 |
| AT1G60200 | 17.4851 | 23.5333 |
| AT4G09460 | 17.4781 | 19.7752 |
| AT2G45180 | 17.4734 | 23.1071 |
| AT2G31440 | 17.4693 | 11.5408 |
| AT4G34290 | 17.4655 | 17.2169 |
| AT4G17670 | 17.4655 | 4.53365 |
| AT4G26870 | 17.4615 | 28.9098 |
| AT1G64561 | 17.4582 | 4.14273 |
| AT5G38880 | 17.4533 | 22.455 |
| AT5G39360 | 17.4533 | 16.3284 |
| AT4G01150 | 17.4518 | 11.2019 |
| AT3G02150 | 17.4453 | 3.18378 |
| AT5G22850 | 17.4371 | 14.0716 |
| AT2G25760 | 17.4356 | 15.945 |
| AT5G59710 | 17.4321 | 18.7958 |
| AT1G08500 | 17.4309 | 18.0649 |
| AT1G08110 | 17.4157 | 27.0761 |
| AT2G21940 | 17.4152 | 11.5785 |
| AT4G34340 | 17.4096 | 17.3557 |
| AT5G08565 | 17.406 | 10.1761 |
| AT1G41880 | 17.4038 | 114.852 |
| AT1G47570 | 17.4023 | 6.64635 |
| AT4G38240 | 17.3946 | 17.5872 |
| AT1G67030 | 17.3946 | 6.43359 |
| AT1G15800 | 17.3906 | 24.8095 |
| AT4G02560 | 17.3875 | 14.445 |
| AT4G21410 | 17.3776 | 11.5101 |
| AT3G48690 | 17.3774 | 20.1352 |
| AT5G41340 | 17.3754 | 27.7095 |
| AT3G06250 | 17.3754 | 15.9307 |
| AT1G61730 | 17.374 | 30.4768 |
| AT1G31930 | 17.3725 | 24.6453 |
| AT2G31410 | 17.3715 | 49.4705 |
| AT1G07130 | 17.3692 | 8.10913 |
| AT1G59520 | 17.367 | 14.3108 |
| AT5G17070 | 17.3656 | 20.5108 |
| AT1G05940 | 17.352 | 12.3744 |
| AT3G50370 | 17.3507 | 18.7442 |
| AT3G05820 | 17.3463 | 3.40996 |
| AT4G17520 | 17.3443 | 72.9227 |
| AT3G25870 | 17.3409 | 8.45197 |
| AT1G28320 | 17.3321 | 12.1506 |
| AT2G20790 | 17.3273 | 10.9694 |
| AT5G19320 | 17.3217 | 23.9924 |
| AT4G18010 | 17.3211 | 3.66368 |
| AT3G09375 | 17.32 | 4.43946 |
| AT2G44010 | 17.3172 | 3.52548 |
| AT4G31410 | 17.3149 | 15.6598 |
| AT5G60670 | 17.3108 | 80.9614 |
| AT2G03150 | 17.3038 | 20.7829 |
| AT1G76560 | 17.2998 | 15.4258 |
| AT4G37510 | 17.2968 | 9.62655 |
| AT1G30970 | 17.2936 | 19.0837 |
| AT3G02540 | 17.2754 | 33.4799 |
| AT1G19190 | 17.2723 | 1.46088 |
| AT4G24470 | 17.2692 | 15.334 |
| AT2G44530 | 17.2681 | 19.512 |
| AT1G69390 | 17.2579 | 15.6488 |
| AT4G10960 | 17.2554 | 20.6649 |
| AT2G47520 | 17.249 | 4.73635 |
| AT3G53880 | 17.2409 | 12.0853 |
| AT5G23140 | 17.2364 | 17.3077 |
| AT5G43150 | 17.2338 | 1.34832 |
| AT4G38215 | 17.2332 | 8.74338 |
| AT1G68920 | 17.2288 | 37.0572 |
| AT2G39210 | 17.2275 | 20.0055 |
| AT5G67220 | 17.2274 | 35.9909 |
| AT2G41650 | 17.2273 | 70.1913 |
| AT5G58540 | 17.2241 | 15.2443 |
| AT2G01170 | 17.2223 | 15.6665 |
| AT5G62180 | 17.2119 | 9.85314 |
| AT1G60670 | 17.2116 | 12.8174 |
| AT1G67530 | 17.2063 | 15.0199 |
| AT4G27400 | 17.2036 | 117.743 |
| AT1G02520 | 17.2021 | 12.6441 |
| AT5G62610 | 17.1947 | 40.1723 |
| AT5G44860 | 17.1882 | 9.86803 |
| AT2G17030 | 17.1867 | 11.9536 |
| AT5G18130 | 17.1829 | 7.1484 |
| AT2G25140 | 17.182 | 27.0521 |
| AT5G36250 | 17.1784 | 40.5044 |
| AT2G35900 | 17.1784 | 21.5036 |
| AT5G51970 | 17.1761 | 12.2748 |
| AT2G24240 | 17.1747 | 6.79136 |
| AT5G49930 | 17.1746 | 21.5012 |
| AT5G19430 | 17.1736 | 17.619 |
| AT3G47940 | 17.1735 | 15.6489 |
| AT4G35560 | 17.1691 | 17.3749 |
| AT5G66140 | 17.1665 | 18.4863 |
| AT2G26980 | 17.1537 | 14.954 |
| AT1G11160 | 17.1499 | 1.1623 |
| AT5G65800 | 17.145 | 1.25001 |
| AT2G06005 | 17.1368 | 7.91426 |
| AT3G09910 | 17.1275 | 25.3504 |
| AT2G38420 | 17.1257 | 10.7533 |
| AT1G73965 | 17.125 | 23.8465 |
| AT1G09930 | 17.1248 | 6.61336 |
| AT2G16930 | 17.1242 | 20.1045 |
| AT3G59710 | 17.1201 | 6.08803 |
| AT4G21720 | 17.1195 | 9.24852 |
| AT5G17630 | 17.1127 | 23.6618 |
| AT3G51970 | 17.1107 | 3.47699 |
| AT3G54270 | 17.1092 | 9.53026 |
| AT5G06480 | 17.1077 | 17.9864 |
| AT2G19350 | 17.1021 | 11.7963 |
| AT5G02310 | 17.1019 | 14.2998 |
| AT2G35890 | 17.1009 | 1.53235 |
| AT5G14700 | 17.1007 | 20.3104 |
| AT5G16590 | 17.0951 | 10.3098 |
| AT5G06910 | 17.0926 | 7.71924 |
| AT1G30475 | 17.0898 | 14.2179 |
| AT1G17350 | 17.0884 | 20.8966 |
| AT2G28320 | 17.0824 | 11.7171 |
| AT2G31130 | 17.0814 | 14.7502 |
| AT1G08460 | 17.0783 | 13.63 |
| AT1G55810 | 17.0766 | 15.8266 |
| AT2G27960 | 17.0743 | 15.5887 |
| AT1G59750 | 17.0682 | 17.7346 |
| AT1G54690 | 17.0617 | 56.9813 |
| AT4G36890 | 17.0591 | 13.8293 |
| AT3G15160 | 17.0566 | 9.52141 |
| AT1G06630 | 17.0531 | 15.0497 |
| AT1G53040 | 17.048 | 13.3313 |
| AT1G31020 | 17.0447 | 18.4554 |
| AT4G34430 | 17.0385 | 17.8487 |
| AT3G27610 | 17.0357 | 11.0298 |
| AT5G62740 | 17.0278 | 25.1414 |
| AT1G12060 | 17.0255 | 6.18201 |
| AT1G61670 | 17.0251 | 12.9635 |
| AT1G01260 | 17.0216 | 18.7334 |
| AT2G22850 | 17.0066 | 14.1635 |
| AT4G28840 | 16.9961 | 10.9012 |
| AT1G47240 | 16.9938 | 15.33 |
| AT5G09978 | 16.9892 | 6.61049 |
| AT4G20450 | 16.9875 | 0.0110145 |
| AT4G17840 | 16.987 | 30.7768 |
| AT3G20000 | 16.985 | 60.3176 |
| AT5G58100 | 16.9818 | 25.3495 |
| AT2G37035 | 16.9783 | 12.5895 |
| AT1G05650 | 16.961 | 6.93673 |
| AT1G26440 | 16.9595 | 7.21237 |
| AT4G34750 | 16.9547 | 2.74517 |
| AT1G17090 | 16.947 | 8.51979 |
| AT3G10030 | 16.946 | 15.7731 |
| AT2G28210 | 16.943 | 6.55258 |
| AT1G79790 | 16.9418 | 6.53474 |
| AT5G53570 | 16.9398 | 32.8598 |
| AT5G07890 | 16.939 | 15.3363 |
| AT1G25350 | 16.9355 | 28.4017 |
| AT5G62270 | 16.9329 | 27.798 |
| AT2G18400 | 16.9273 | 45.0538 |
| AT1G32200 | 16.9238 | 15.2936 |
| AT3G56070 | 16.9181 | 59.0457 |
| AT2G38820 | 16.9021 | 22.844 |
| AT1G74770 | 16.8997 | 5.16404 |
| AT5G08350 | 16.8933 | 17.602 |
| AT2G21500 | 16.8886 | 13.6303 |
| AT2G27440 | 16.888 | 0.182014 |
| AT1G25370 | 16.8872 | 4.97684 |
| AT4G34500 | 16.8852 | 12.4493 |
| AT3G56400 | 16.8807 | 1.14721 |
| AT5G55670 | 16.8762 | 17.284 |
| AT3G62310 | 16.8655 | 26.0725 |
| AT2G48160 | 16.8636 | 16.4829 |
| AT4G21215 | 16.8602 | 1.05 |
| AT5G54850 | 16.8586 | 10.461 |
| AT1G55820 | 16.8575 | 14.1916 |
| AT4G14290 | 16.8559 | 14.3348 |
| AT5G64030 | 16.853 | 36.0858 |
| AT5G42030 | 16.8509 | 9.80309 |
| AT3G05150 | 16.833 | 11.5976 |
| AT5G03070 | 16.8305 | 16.9687 |
| AT4G34210 | 16.8281 | 24.8309 |
| AT4G03610 | 16.8203 | 7.86133 |
| AT2G43945 | 16.8141 | 12.3444 |
| AT1G34630 | 16.8065 | 10.8262 |
| AT5G37850 | 16.8025 | 24.0092 |
| AT2G35020 | 16.8018 | 9.75669 |
| AT1G63390 | 16.8016 | 0.736269 |
| AT4G31480 | 16.7953 | 19.8613 |
| AT5G19140 | 16.7927 | 5.77249 |
| AT2G20410 | 16.7923 | 14.0623 |
| AT5G05950 | 16.7867 | 21.2781 |
| AT1G65310 | 16.7779 | 9.66595 |
| AT3G17920 | 16.7755 | 16.7434 |
| AT2G36630 | 16.7727 | 11.6246 |
| AT3G26890 | 16.762 | 11.609 |
| AT5G44080 | 16.7586 | 14.2232 |
| AT1G05120 | 16.7576 | 15.8355 |
| AT4G23910 | 16.7538 | 23.944 |
| AT3G11660 | 16.7524 | 26.7339 |
| AT1G32090 | 16.7517 | 28.9781 |
| AT5G50320 | 16.7325 | 18.8468 |
| AT3G08890 | 16.7317 | 15.6429 |
| AT4G40070 | 16.7292 | 3.12417 |
| AT3G52430 | 16.7211 | 11.4031 |
| AT3G58640 | 16.7203 | 22.6828 |
| AT1G30120 | 16.7172 | 37.9992 |
| AT1G63290 | 16.716 | 14.8538 |
| AT5G54130 | 16.7033 | 2.32207 |
| AT5G24670 | 16.7022 | 14.3764 |
| AT3G03320 | 16.7016 | 40.817 |
| AT3G07010 | 16.6974 | 11.635 |
| AT2G44050 | 16.6925 | 32.0868 |
| AT5G58270 | 16.6891 | 21.2397 |
| AT3G54150 | 16.6883 | 26.1931 |
| AT5G10270 | 16.685 | 12.6497 |
| AT4G16420 | 16.6834 | 20.913 |
| AT2G15880 | 16.6763 | 9.73956 |
| AT5G11420 | 16.6661 | 28.4257 |
| AT5G04850 | 16.665 | 13.8471 |
| AT3G10980 | 16.6643 | 13.6756 |
| AT3G60590 | 16.6614 | 9.83825 |
| AT1G73510 | 16.6562 | 3.76284 |
| AT3G60480 | 16.6384 | 8.82808 |
| AT1G24460 | 16.6285 | 18.6697 |
| AT4G26095 | 16.6273 | 7.19804 |
| AT1G14230 | 16.6268 | 13.1181 |
| AT4G16680 | 16.6191 | 5.4107 |
| AT2G24420 | 16.6152 | 13.7857 |
| AT3G04910 | 16.6149 | 15.0435 |
| AT5G53500 | 16.6055 | 46.3983 |
| AT1G48770 | 16.6013 | 17.3979 |
| AT1G75990 | 16.5936 | 19.2535 |
| AT3G51050 | 16.5933 | 18.5873 |
| AT4G22850 | 16.5927 | 12.2681 |
| AT5G23395 | 16.5863 | 19.5292 |
| AT5G47100 | 16.5849 | 18.0236 |
| AT4G13780 | 16.5829 | 35.5525 |
| AT5G63000 | 16.575 | 11.4811 |
| AT3G20720 | 16.5719 | 10.321 |
| AT2G43570 | 16.5687 | 8.0326 |
| AT2G31580 | 16.5666 | 10.7543 |
| AT3G15351 | 16.5662 | 19.8157 |
| AT1G62480 | 16.5631 | 101.061 |
| AT1G55090 | 16.5617 | 15.9286 |
| AT5G56080 | 16.554 | 8.13705 |
| AT1G20430 | 16.5523 | 23.0261 |
| AT5G05110 | 16.5449 | 25.8782 |
| AT1G74400 | 16.5434 | 6.40993 |
| AT1G04910 | 16.5265 | 18.8002 |
| AT1G09060 | 16.5259 | 20.9648 |
| AT5G41910 | 16.5023 | 12.2338 |
| AT3G03560 | 16.4982 | 13.8474 |
| AT1G03106 | 16.4945 | 0.865869 |
| AT1G30040 | 16.4893 | 10.9229 |
| AT1G04445 | 16.4878 | 2.98234 |
| AT2G43950 | 16.4854 | 26.0792 |
| AT3G06490 | 16.4844 | 9.13992 |
| AT1G74100 | 16.4828 | 33.924 |
| AT4G22300 | 16.4806 | 19.0386 |
| AT2G28000 | 16.4675 | 52.5604 |
| AT4G33100 | 16.4647 | 16.9618 |
| AT5G42825 | 16.4615 | 10.926 |
| AT1G50410 | 16.4586 | 13.9207 |
| AT4G14147 | 16.4549 | 12.6009 |
| AT1G14590 | 16.4481 | 14.0471 |
| AT3G44310 | 16.4418 | 5.64391 |
| AT4G26890 | 16.4378 | 6.02283 |
| AT3G11630 | 16.426 | 52.0349 |
| AT3G54440 | 16.4234 | 17.5453 |
| AT1G15060 | 16.403 | 12.67 |
| AT4G23920 | 16.4026 | 54.9604 |
| AT2G42580 | 16.4009 | 16.9419 |
| AT4G31920 | 16.3955 | 31.5569 |
| AT1G77890 | 16.3941 | 14.2393 |
| AT2G20390 | 16.383 | 17.7662 |
| AT4G34120 | 16.3824 | 11.6183 |
| AT1G12780 | 16.3777 | 80.5161 |
| AT1G06850 | 16.3686 | 10.8293 |
| AT5G01290 | 16.3643 | 15.2246 |
| AT2G29050 | 16.3578 | 20.9768 |
| AT3G04610 | 16.3525 | 26.6995 |
| AT2G19750 | 16.3515 | 79.4874 |
| AT1G80070 | 16.3458 | 38.9212 |
| AT3G27470 | 16.3202 | 3.94036 |
| AT3G16090 | 16.3193 | 12.2125 |
| AT4G17080 | 16.3115 | 10.1194 |
| AT3G09100 | 16.3099 | 22.4402 |
| AT1G13460 | 16.3058 | 10.2637 |
| AT1G75410 | 16.2989 | 2.76465 |
| AT4G03500 | 16.2964 | 2.4529 |
| AT5G27540 | 16.2953 | 28.0342 |
| AT4G27070 | 16.2902 | 14.7946 |
| AT2G45240 | 16.2888 | 23.549 |
| AT5G22280 | 16.2855 | 39.724 |
| AT2G43540 | 16.2788 | 8.22105 |
| AT1G79510 | 16.2776 | 9.37657 |
| AT4G14280 | 16.2735 | 3.89118 |
| AT4G17085 | 16.2728 | 21.1352 |
| AT1G43700 | 16.2725 | 14.7714 |
| AT4G38552 | 16.2693 | 8.82102 |
| AT1G71100 | 16.2622 | 13.653 |
| AT3G57062 | 16.257 | 8.96985 |
| AT4G26300 | 16.2507 | 34.7351 |
| AT2G38440 | 16.2401 | 11.8217 |
| AT4G29420 | 16.2395 | 11.3871 |
| AT4G30760 | 16.2171 | 16.0012 |
| AT1G26970 | 16.2133 | 1.67576 |
| AT3G21810 | 16.2121 | 11.677 |
| AT5G23600 | 16.2113 | 8.67141 |
| AT5G01970 | 16.2043 | 10.6784 |
| AT3G53780 | 16.2026 | 17.1214 |
| AT1G20550 | 16.1881 | 8.37029 |
| AT1G73840 | 16.187 | 13.7663 |
| AT5G59450 | 16.179 | 13.946 |
| AT2G28360 | 16.1777 | 9.90587 |
| AT2G25950 | 16.1765 | 18.4694 |
| AT1G27752 | 16.1756 | 13.5959 |
| AT1G67890 | 16.1733 | 10.2634 |
| AT3G52040 | 16.1698 | 44.8975 |
| AT2G39020 | 16.1658 | 34.0419 |
| AT3G07960 | 16.1538 | 4.87844 |
| AT2G25870 | 16.1479 | 27.37 |
| AT1G35430 | 16.1451 | 10.2832 |
| AT5G52300 | 16.1364 | 3.30409 |
| AT3G09880 | 16.1302 | 14.332 |
| AT3G52660 | 16.1262 | 18.2724 |
| AT1G03260 | 16.1244 | 11.5525 |
| AT5G13590 | 16.1215 | 13.7374 |
| AT2G42390 | 16.116 | 11.8859 |
| AT5G54590 | 16.1115 | 7.64786 |
| AT5G12240 | 16.1079 | 17.9307 |
| AT1G17370 | 16.1034 | 25.8496 |
| AT5G41770 | 16.1028 | 25.1206 |
| AT3G10540 | 16.0953 | 15.1121 |
| AT5G59940 | 16.0925 | 7.4783 |
| AT5G45000 | 16.0892 | 2.80833 |
| AT2G36480 | 16.0859 | 13.4903 |
| AT3G61690 | 16.0855 | 9.83863 |
| AT5G12390 | 16.0851 | 7.00902 |
| AT3G57790 | 16.0836 | 5.20771 |
| AT3G15010 | 16.0803 | 16.7334 |
| AT1G75080 | 16.0774 | 17.9514 |
| AT5G06780 | 16.0772 | 24.6532 |
| AT1G07240 | 16.0597 | 9.96151 |
| AT4G04700 | 16.0592 | 2.47984 |
| AT5G53850 | 16.0527 | 24.4902 |
| AT2G14820 | 16.0507 | 15.635 |
| AT1G53710 | 16.0492 | 12.4327 |
| AT4G10500 | 16.0491 | 2.50061 |
| AT2G46550 | 16.0422 | 18.8114 |
| AT5G46060 | 16.0353 | 2.83077 |
| AT5G07770 | 16.0326 | 1.31705 |
| AT1G56560 | 16.0222 | 15.8935 |
| AT5G15530 | 16.0184 | 30.194 |
| AT4G05390 | 16.01 | 22.1867 |
| AT3G03220 | 16.006 | 14.1715 |
| AT4G28470 | 16.0046 | 25.9321 |
| AT3G62270 | 16.0046 | 14.38 |
| AT4G10360 | 16.0014 | 10.6628 |
| AT3G22620 | 15.9958 | 29.0724 |
| AT1G53645 | 15.9915 | 19.7554 |
| AT5G13700 | 15.9883 | 11.3 |
| AT5G22140 | 15.9854 | 76.6152 |
| AT1G26470 | 15.9807 | 28.9955 |
| AT1G01990 | 15.9777 | 16.7214 |
| AT4G32960 | 15.9756 | 9.15664 |
| AT1G71110 | 15.971 | 36.1973 |
| AT1G18940 | 15.9634 | 0.904401 |
| AT1G24100 | 15.9606 | 23.0449 |
| AT1G45249 | 15.9588 | 8.3764 |
| AT1G70720 | 15.9509 | 0.345402 |
| AT5G51300 | 15.9482 | 20.6209 |
| AT3G19630 | 15.9394 | 18.2304 |
| AT5G64090 | 15.9375 | 14.0883 |
| AT1G48760 | 15.9306 | 14.5732 |
| AT1G35780 | 15.9296 | 72.8949 |
| AT4G02860 | 15.9292 | 16.9912 |
| AT1G02160 | 15.9245 | 24.2369 |
| AT3G55030 | 15.9228 | 22.8249 |
| AT3G46820 | 15.921 | 12.5323 |
| AT1G36160 | 15.9147 | 16.6315 |
| AT2G17080 | 15.9122 | 5.98287 |
| AT1G70530 | 15.9083 | 23.3947 |
| AT1G49760 | 15.9053 | 60.4242 |
| AT5G23030 | 15.9045 | 0.177511 |
| AT5G42520 | 15.8931 | 17.8662 |
| AT1G15780 | 15.8891 | 10.3354 |
| AT4G33945 | 15.8727 | 17.7359 |
| AT3G48330 | 15.8726 | 25.1508 |
| AT3G59320 | 15.8717 | 8.05161 |
| AT2G26560 | 15.8694 | 18.5054 |
| AT5G20170 | 15.8626 | 17.188 |
| AT1G64890 | 15.8609 | 3.18315 |
| AT1G23820 | 15.8605 | 58.325 |
| AT1G48090 | 15.8603 | 13.0785 |
| AT4G38890 | 15.8492 | 15.5776 |
| AT5G06740 | 15.8401 | 6.25601 |
| AT3G43520 | 15.8328 | 24.303 |
| AT3G56720 | 15.8321 | 14.2381 |
| AT3G24420 | 15.8116 | 3.09828 |
| AT5G01160 | 15.8097 | 10.812 |
| AT1G24340 | 15.804 | 9.4511 |
| AT5G49220 | 15.802 | 27.4739 |
| AT4G12010 | 15.7912 | 6.16154 |
| AT2G47980 | 15.7853 | 21.8852 |
| AT3G25530 | 15.7847 | 39.4516 |
| AT2G32295 | 15.7838 | 8.02424 |
| AT1G30190 | 15.7828 | 2.27015 |
| AT4G35780 | 15.7822 | 9.94794 |
| AT1G30480 | 15.7784 | 13.2278 |
| AT3G52140 | 15.7628 | 34.686 |
| AT1G53430 | 15.7619 | 14.7186 |
| AT3G07550 | 15.7527 | 10.883 |
| AT1G64330 | 15.7463 | 24.8786 |
| AT5G55120 | 15.7297 | 31.4932 |
| AT3G28850 | 15.7242 | 4.30781 |
| AT5G56020 | 15.7236 | 9.5269 |
| AT5G25250 | 15.721 | 2.85528 |
| AT5G25080 | 15.712 | 19.3049 |
| AT2G39170 | 15.708 | 21.4444 |
| AT3G21295 | 15.6964 | 8.69533 |
| AT3G03110 | 15.6935 | 20.3848 |
| AT2G32040 | 15.6906 | 16.3012 |
| AT3G23560 | 15.6825 | 11.0922 |
| AT2G40820 | 15.6802 | 11.7753 |
| AT2G45950 | 15.678 | 13.9119 |
| AT1G16190 | 15.669 | 38.2864 |
| AT5G24450 | 15.6517 | 11.6125 |
| AT5G58900 | 15.6422 | 11.2819 |
| AT3G48870 | 15.6384 | 39.7538 |
| AT2G32720 | 15.6307 | 26.2299 |
| AT3G06410 | 15.6284 | 17.4963 |
| AT4G25720 | 15.6283 | 12.1005 |
| AT2G26550 | 15.6268 | 10.8148 |
| AT2G18760 | 15.6256 | 6.84883 |
| AT4G38500 | 15.6187 | 15.5341 |
| AT3G47060 | 15.6094 | 13.4289 |
| AT1G63720 | 15.6075 | 10.9781 |
| AT5G11170 | 15.6053 | 35.4058 |
| AT1G07810 | 15.5994 | 15.5367 |
| AT1G15490 | 15.5963 | 3.97284 |
| AT1G72870 | 15.5963 | 1.63679 |
| AT2G03480 | 15.591 | 6.3875 |
| AT5G10290 | 15.5892 | 19.1598 |
| AT3G16780 | 15.5842 | 63.7194 |
| AT3G19220 | 15.5839 | 8.53964 |
| AT2G22640 | 15.5818 | 16.6071 |
| AT1G66740 | 15.5763 | 15.8812 |
| AT4G26200 | 15.5717 | 4.27107 |
| AT2G17410 | 15.5676 | 27.8535 |
| AT3G10220 | 15.5516 | 30.9605 |
| AT3G10210 | 15.5516 | 15.1872 |
| AT3G26340 | 15.5496 | 16.9403 |
| AT5G17590 | 15.5451 | 0.230873 |
| AT3G61960 | 15.5421 | 22.7157 |
| AT1G01950 | 15.5359 | 11.4571 |
| AT5G08139 | 15.5337 | 20.156 |
| AT3G51620 | 15.5286 | 12.4817 |
| AT3G59800 | 15.5276 | 22.4667 |
| AT3G62900 | 15.5228 | 13.6609 |
| AT4G39100 | 15.5204 | 10.5853 |
| AT1G16270 | 15.5202 | 27.3032 |
| AT3G58700 | 15.5191 | 58.609 |
| AT1G33610 | 15.5179 | 13.1371 |
| AT3G09490 | 15.5116 | 3.70568 |
| AT2G42850 | 15.5032 | 28.2243 |
| AT1G78172 | 15.503 | 4.41614 |
| AT1G34550 | 15.5005 | 18.1756 |
| AT1G74410 | 15.4909 | 11.0487 |
| AT5G19960 | 15.4857 | 13.392 |
| AT5G26360 | 15.4786 | 91.2002 |
| AT4G01090 | 15.4774 | 17.4089 |
| AT5G10950 | 15.4705 | 23.0134 |
| AT1G19050 | 15.4698 | 8.98948 |
| AT4G22550 | 15.4669 | 9.68958 |
| AT3G12390 | 15.4539 | 67.5073 |
| AT5G58200 | 15.4495 | 13.3099 |
| AT5G62810 | 15.4481 | 13.4339 |
| AT1G05330 | 15.4411 | 2.30497 |
| AT3G57120 | 15.4335 | 7.19612 |
| AT1G12910 | 15.4328 | 18.7104 |
| AT5G06280 | 15.42 | 12.9516 |
| AT2G33255 | 15.4199 | 7.28157 |
| AT3G08960 | 15.4197 | 14.864 |
| AT5G39050 | 15.4103 | 48.4594 |
| AT4G38620 | 15.3817 | 0.906201 |
| AT2G17240 | 15.3814 | 21.0125 |
| AT1G01710 | 15.3808 | 11.3727 |
| AT4G38350 | 15.3788 | 17.0538 |
| AT1G03130 | 15.3742 | 8.79679 |
| AT4G19960 | 15.3728 | 2.69787 |
| AT2G38120 | 15.3719 | 66.222 |
| AT2G20724 | 15.3694 | 8.15172 |
| AT1G11190 | 15.365 | 16.5141 |
| AT5G03690 | 15.3554 | 11.9811 |
| AT4G03190 | 15.3526 | 102.702 |
| AT1G06130 | 15.3516 | 30.8722 |
| AT1G14610 | 15.3512 | 33.9394 |
| AT5G20950 | 15.3429 | 62.6194 |
| AT4G12780 | 15.3423 | 9.84138 |
| AT5G18620 | 15.3327 | 24.7548 |
| AT5G43280 | 15.3326 | 11.9867 |
| AT4G26110 | 15.3288 | 52.1288 |
| AT5G36260 | 15.322 | 2.60715 |
| AT3G50050 | 15.3175 | 11.0734 |
| AT5G61410 | 15.3137 | 11.5229 |
| AT2G30470 | 15.3118 | 11.2282 |
| AT3G44320 | 15.3103 | 45.3185 |
| AT2G42810 | 15.3093 | 27.3169 |
| AT1G59830 | 15.3085 | 14.4211 |
| AT1G04260 | 15.3019 | 8.77359 |
| AT1G29890 | 15.2951 | 19.0644 |
| AT2G38130 | 15.2929 | 32.6061 |
| AT2G27210 | 15.2912 | 13.0629 |
| AT4G33010 | 15.2873 | 17.2094 |
| AT3G27220 | 15.2829 | 12.2864 |
| AT1G26930 | 15.2801 | 38.3111 |
| AT1G12420 | 15.2801 | 9.41653 |
| AT3G01790 | 15.2795 | 32.4299 |
| AT4G27040 | 15.2692 | 10.5519 |
| AT5G24410 | 15.2605 | 1.59477 |
| AT4G20440 | 15.2341 | 27.2463 |
| AT1G73390 | 15.2337 | 15.1918 |
| AT1G19835 | 15.2329 | 22.0668 |
| AT4G37700 | 15.2324 | 13.0597 |
| AT1G07520 | 15.2306 | 15.2983 |
| AT4G13830 | 15.2285 | 9.84302 |
| AT1G16320 | 15.2227 | 14.2665 |
| AT3G48020 | 15.2227 | 1.43213 |
| AT2G24790 | 15.2223 | 27.6203 |
| AT1G32310 | 15.2137 | 14.3408 |
| AT4G13980 | 15.2133 | 11.2772 |
| AT2G34380 | 15.205 | 10.8477 |
| AT2G02090 | 15.2039 | 14.3678 |
| AT3G54230 | 15.2039 | 13.9503 |
| AT5G41210 | 15.2029 | 20.6242 |
| AT5G45030 | 15.1841 | 14.7294 |
| AT1G17980 | 15.1764 | 11.0475 |
| AT3G50920 | 15.1736 | 14.7493 |
| AT1G22330 | 15.1725 | 2.13332 |
| AT5G02320 | 15.172 | 12.565 |
| AT5G37380 | 15.1709 | 10.7993 |
| AT1G18640 | 15.1695 | 24.109 |
| AT4G35220 | 15.1676 | 15.2994 |
| AT3G07000 | 15.1634 | 1.97575 |
| AT1G24600 | 15.1612 | 6.57666 |
| AT4G16440 | 15.1608 | 12.1993 |
| AT5G19570 | 15.1526 | 12.3361 |
| AT1G35190 | 15.1504 | 12.1007 |
| AT1G50920 | 15.1414 | 54.6685 |
| AT2G34840 | 15.1402 | 8.19526 |
| AT2G19620 | 15.1397 | 13.6194 |
| AT4G14710 | 15.1304 | 15.2572 |
| AT1G44750 | 15.1298 | 15.3526 |
| AT2G44420 | 15.1242 | 7.52377 |
| AT3G18295 | 15.1176 | 7.26648 |
| AT3G24090 | 15.1167 | 24.4524 |
| AT4G22120 | 15.1157 | 7.79295 |
| AT5G19380 | 15.0947 | 20.8594 |
| AT1G05870 | 15.0909 | 17.7633 |
| AT2G43400 | 15.0854 | 9.72174 |
| AT1G17160 | 15.0816 | 10.1048 |
| AT4G38270 | 15.0799 | 11.9228 |
| AT2G39725 | 15.0781 | 20.3644 |
| AT4G24560 | 15.0771 | 15.0854 |
| AT2G43880 | 15.0492 | 58.7877 |
| AT5G65200 | 15.0479 | 4.9046 |
| AT3G06320 | 15.0446 | 38.1459 |
| AT2G31370 | 15.0395 | 20.6316 |
| AT1G73930 | 15.0256 | 9.27962 |
| AT1G51850 | 15.024 | 3.40618 |
| AT5G55500 | 15.017 | 7.72786 |
| AT2G24860 | 15.0113 | 8.32475 |
| AT1G05860 | 15.0053 | 13.0916 |
| AT3G19430 | 15.0042 | 89.8936 |
| AT2G48020 | 15.0033 | 27.2 |
| AT3G05936 | 15.0029 | 3.86097 |
| AT2G19570 | 15.0016 | 8.64706 |
| AT1G51730 | 14.9947 | 27.9297 |
| AT5G10700 | 14.9942 | 7.4461 |
| AT5G43960 | 14.981 | 33.4275 |
| AT1G53340 | 14.9653 | 1.20473 |
| AT1G28070 | 14.9627 | 14.105 |
| AT2G39080 | 14.957 | 7.77238 |
| AT5G42965 | 14.9418 | 14.7825 |
| AT5G52400 | 14.9366 | 10.7383 |
| AT3G05040 | 14.9342 | 11.4732 |
| AT5G57190 | 14.9324 | 2.55125 |
| AT1G20950 | 14.9309 | 64.265 |
| AT1G70060 | 14.9221 | 18.437 |
| AT1G35620 | 14.9139 | 22.9325 |
| AT2G41600 | 14.9117 | 16.6334 |
| AT3G13225 | 14.9038 | 17.5285 |
| AT5G03510 | 14.9028 | 10.5556 |
| AT5G56160 | 14.8964 | 0.969393 |
| AT5G67450 | 14.8959 | 1.13073 |
| AT5G51280 | 14.8953 | 21.355 |
| AT2G40935 | 14.8946 | 13.9523 |
| AT3G10630 | 14.8862 | 9.90405 |
| AT4G19600 | 14.8843 | 13.5206 |
| AT1G62200 | 14.8716 | 10.1675 |
| AT1G72330 | 14.8701 | 10.2729 |
| AT2G17370 | 14.869 | 14.2107 |
| AT3G28480 | 14.8656 | 15.6064 |
| AT2G44820 | 14.8655 | 28.4021 |
| AT5G42060 | 14.8593 | 13.6461 |
| AT4G25350 | 14.8408 | 18.4799 |
| AT5G13860 | 14.8396 | 9.79015 |
| AT3G30841 | 14.8371 | 6.55701 |
| AT3G52370 | 14.8346 | 4.30956 |
| AT5G16540 | 14.8324 | 2.04106 |
| AT1G54740 | 14.8321 | 1.03078 |
| AT4G03030 | 14.8249 | 9.87313 |
| AT3G05520 | 14.8245 | 12.4253 |
| AT5G41600 | 14.823 | 34.5528 |
| AT3G20870 | 14.8196 | 14.1802 |
| AT1G59970 | 14.8158 | 6.37032 |
| AT2G26000 | 14.8099 | 11.824 |
| AT5G16070 | 14.7988 | 48.4299 |
| AT2G31141 | 14.7929 | 3.19149 |
| AT3G55005 | 14.7925 | 12.7404 |
| AT1G31860 | 14.7889 | 38.8499 |
| AT1G71900 | 14.7877 | 22.0367 |
| AT2G38610 | 14.7856 | 20.606 |
| AT1G32150 | 14.7837 | 11.6734 |
| AT2G29730 | 14.7789 | 2.48924 |
| AT2G34355 | 14.7767 | 8.59318 |
| AT5G14930 | 14.7737 | 21.7563 |
| AT4G27610 | 14.7674 | 6.19864 |
| AT1G65985 | 14.7657 | 3.37933 |
| AT1G30320 | 14.7633 | 18.6838 |
| AT5G18525 | 14.7602 | 9.63404 |
| AT4G25440 | 14.7554 | 9.6226 |
| AT5G46840 | 14.7538 | 36.5836 |
| AT3G50850 | 14.7501 | 3.5271 |
| AT3G05510 | 14.7449 | 10.8581 |
| AT2G27360 | 14.7443 | 1.32258 |
| AT1G42480 | 14.7442 | 21.1366 |
| AT5G24930 | 14.742 | 10.8312 |
| AT3G26935 | 14.7381 | 17.6592 |
| AT5G13050 | 14.7373 | 7.64998 |
| AT4G30480 | 14.7312 | 13.0994 |
| AT3G10525 | 14.7277 | 7.41571 |
| AT1G22540 | 14.7275 | 2.15645 |
| AT2G41530 | 14.7272 | 29.2154 |
| AT3G01850 | 14.7237 | 1.72598 |
| AT5G62600 | 14.7206 | 12.3632 |
| AT4G09750 | 14.7203 | 8.88288 |
| AT5G26800 | 14.7174 | 23.1924 |
| AT2G14045 | 14.7152 | 20.9212 |
| AT3G14190 | 14.7115 | 29.1842 |
| AT5G58190 | 14.7058 | 15.781 |
| AT4G35140 | 14.7051 | 16.6664 |
| AT5G12080 | 14.703 | 11.9356 |
| AT2G21230 | 14.702 | 13.5089 |
| AT4G26240 | 14.6959 | 13.9064 |
| AT1G79330 | 14.6955 | 5.70211 |
| AT5G01732 | 14.6943 | 3.36436 |
| AT5G05930 | 14.6932 | 11.7663 |
| AT5G57360 | 14.6887 | 10.8064 |
| AT1G03040 | 14.687 | 19.9253 |
| AT4G15900 | 14.6778 | 38.7042 |
| AT1G52380 | 14.6663 | 26.4855 |
| AT2G33845 | 14.6635 | 5.78552 |
| AT2G22250 | 14.6613 | 16.7555 |
| AT2G37160 | 14.6598 | 10.5716 |
| AT1G64720 | 14.6527 | 22.6437 |
| AT1G72740 | 14.6523 | 14.7183 |
| AT4G17010 | 14.6518 | 11.7538 |
| AT3G24570 | 14.6451 | 25.852 |
| AT1G20220 | 14.6404 | 42.5307 |
| AT5G60700 | 14.6395 | 59.6668 |
| AT2G03870 | 14.6384 | 49.5225 |
| AT5G20110 | 14.6289 | 24.0313 |
| AT1G08910 | 14.6262 | 17.4204 |
| AT1G50930 | 14.6227 | 0.257329 |
| AT1G54140 | 14.619 | 26.2732 |
| AT2G15220 | 14.617 | 9.0625 |
| AT2G33790 | 14.6109 | 133.751 |
| AT5G01700 | 14.6044 | 3.84432 |
| AT1G14810 | 14.5878 | 43.5799 |
| AT1G65690 | 14.5858 | 12.1234 |
| AT1G49980 | 14.5839 | 8.90906 |
| AT4G26830 | 14.5795 | 0.0973208 |
| AT2G17700 | 14.5747 | 19.5029 |
| AT5G46290 | 14.5691 | 68.4326 |
| AT1G49710 | 14.5626 | 13.1426 |
| AT1G50940 | 14.5586 | 16.7444 |
| AT5G38005 | 14.5558 | 6.9017 |
| AT5G05000 | 14.5519 | 25.7956 |
| AT1G19860 | 14.5477 | 10.7337 |
| AT1G70350 | 14.546 | 21.6292 |
| AT3G04605 | 14.5372 | 10.5102 |
| AT3G26720 | 14.5305 | 15.5892 |
| AT5G65495 | 14.5256 | 10.0148 |
| AT3G26360 | 14.5243 | 24.2058 |
| AT1G14130 | 14.5012 | 16.8928 |
| AT3G43220 | 14.4938 | 10.1288 |
| AT3G60350 | 14.4725 | 14.3138 |
| AT1G66860 | 14.4694 | 1.85397 |
| AT5G36220 | 14.4661 | 6.46147 |
| AT3G19520 | 14.464 | 15.4026 |
| AT1G27430 | 14.4639 | 11.077 |
| AT5G10510 | 14.4633 | 66.8074 |
| AT4G26450 | 14.4586 | 13.3833 |
| AT4G10330 | 14.452 | 11.9806 |
| AT2G17845 | 14.4517 | 2.33067 |
| AT4G26550 | 14.4461 | 13.0548 |
| AT1G79860 | 14.4339 | 0.0338565 |
| AT1G13580 | 14.4333 | 16.2412 |
| AT1G28960 | 14.4264 | 22.8439 |
| AT2G39630 | 14.4178 | 23.2563 |
| AT5G56280 | 14.4117 | 23.0001 |
| AT1G26800 | 14.4048 | 11.7045 |
| AT1G29250 | 14.3987 | 69.714 |
| AT2G42690 | 14.3932 | 3.40426 |
| AT1G75330 | 14.3925 | 37.5142 |
| AT2G46700 | 14.389 | 9.92718 |
| AT5G21900 | 14.3879 | 16.9504 |
| AT4G27940 | 14.3867 | 7.93386 |
| AT1G08620 | 14.3852 | 13.6757 |
| AT4G05071 | 14.384 | 2.18891 |
| AT3G11850 | 14.3726 | 5.18043 |
| AT2G34040 | 14.3723 | 27.0709 |
| AT3G46280 | 14.367 | 15.909 |
| AT5G57120 | 14.3608 | 38.1706 |
| AT5G06110 | 14.3608 | 31.3954 |
| AT1G58470 | 14.3509 | 14.7722 |
| AT1G80610 | 14.3485 | 11.5903 |
| AT5G46560 | 14.3465 | 15.8595 |
| AT5G51640 | 14.3462 | 16.5984 |
| AT3G45770 | 14.3461 | 10.6187 |
| AT4G15760 | 14.3385 | 24.0585 |
| AT1G77800 | 14.3358 | 13.5101 |
| AT1G04070 | 14.3341 | 8.4485 |
| AT5G01990 | 14.3254 | 25.0602 |
| AT2G25190 | 14.324 | 18.6029 |
| AT4G14600 | 14.3201 | 10.7642 |
| AT4G31770 | 14.317 | 18.0202 |
| AT3G58620 | 14.3136 | 7.55179 |
| AT3G02900 | 14.3087 | 28.9232 |
| AT1G52342 | 14.306 | 2.91217 |
| AT4G33060 | 14.2956 | 22.5126 |
| AT4G37270 | 14.2954 | 5.34573 |
| AT4G20390 | 14.2946 | 5.07943 |
| AT5G13280 | 14.2933 | 26.162 |
| AT1G59960 | 14.2843 | 27.5748 |
| AT2G35810 | 14.2737 | 9.54238 |
| AT5G58550 | 14.2702 | 14.7026 |
| AT5G08520 | 14.2671 | 14.7866 |
| AT5G11600 | 14.266 | 11.8134 |
| AT5G66310 | 14.2658 | 14.4204 |
| AT2G24290 | 14.2642 | 18.8508 |
| AT5G15880 | 14.2641 | 19.8334 |
| AT2G28710 | 14.2587 | 1.53816 |
| AT3G05070 | 14.2507 | 26.7897 |
| AT3G16470 | 14.2405 | 2.32649 |
| AT2G36485 | 14.2386 | 10.7808 |
| AT5G13740 | 14.2365 | 25.2442 |
| AT5G12170 | 14.2251 | 4.90272 |
| AT5G49470 | 14.2233 | 9.20827 |
| AT1G48200 | 14.2196 | 10.2045 |
| AT1G79650 | 14.2154 | 31.9788 |
| AT5G35570 | 14.1996 | 14.649 |
| AT5G14050 | 14.1947 | 28.1014 |
| AT5G57890 | 14.1908 | 13.1576 |
| AT1G07920 | 14.178 | 22.3472 |
| AT3G55830 | 14.1752 | 14.2938 |
| AT4G16141 | 14.175 | 14.4249 |
| AT1G74660 | 14.1727 | 7.24873 |
| AT2G25730 | 14.1614 | 7.42837 |
| AT1G77640 | 14.1612 | 9.28617 |
| AT2G25930 | 14.1588 | 16.5026 |
| AT4G15030 | 14.1526 | 17.1711 |
| AT5G55260 | 14.152 | 7.01863 |
| AT2G38090 | 14.1479 | 2.40107 |
| AT4G38830 | 14.1427 | 0.913051 |
| AT5G40230 | 14.14 | 8.18578 |
| AT1G07930 | 14.1374 | 24.5101 |
| AT4G25250 | 14.1303 | 33.0668 |
| AT2G30520 | 14.1246 | 19.8299 |
| AT1G55365 | 14.1245 | 1.65217 |
| AT1G71310 | 14.1208 | 15.1243 |
| AT2G30710 | 14.1184 | 14.5387 |
| AT1G74250 | 14.1057 | 15.5888 |
| AT1G54220 | 14.1007 | 15.2537 |
| AT5G41612 | 14.0983 | 13.214 |
| AT5G12850 | 14.0944 | 16.4825 |
| AT3G11960 | 14.0819 | 17.7558 |
| AT2G42660 | 14.0796 | 6.36932 |
| AT1G76950 | 14.0779 | 8.87054 |
| AT2G28960 | 14.0629 | 1.1159 |
| AT3G51140 | 14.0628 | 8.91725 |
| AT4G26860 | 14.0618 | 10.0014 |
| AT1G01680 | 14.0603 | 2.63551 |
| AT3G43250 | 14.0589 | 45.6781 |
| AT5G11760 | 14.0549 | 17.3438 |
| AT2G20130 | 14.0484 | 11.4797 |
| AT5G51710 | 14.0455 | 14.4421 |
| AT5G49950 | 14.0343 | 17.4115 |
| AT3G18130 | 14.0314 | 83.6378 |
| AT1G29290 | 14.0299 | 39.0917 |
| AT1G06470 | 14.0296 | 15.0467 |
| AT1G11240 | 14.0194 | 31.5912 |
| AT3G17790 | 14.0022 | 11.6221 |
| AT1G31540 | 13.9931 | 2.92638 |
| AT5G45420 | 13.9914 | 24.3901 |
| AT5G60280 | 13.9867 | 10.7384 |
| AT2G44980 | 13.9783 | 9.16441 |
| AT5G58870 | 13.9742 | 12.6744 |
| AT4G25630 | 13.9495 | 103.693 |
| AT3G57740 | 13.9428 | 9.09567 |
| AT4G35930 | 13.9377 | 18.1857 |
| AT3G24315 | 13.937 | 14.5455 |
| AT3G07400 | 13.9286 | 13.0292 |
| AT1G78560 | 13.9284 | 32.4079 |
| AT5G23580 | 13.9274 | 17.1741 |
| AT1G53035 | 13.9203 | 9.31439 |
| AT4G00800 | 13.9175 | 12.9021 |
| AT2G46910 | 13.9012 | 6.27408 |
| AT5G03530 | 13.8972 | 32.0075 |
| AT3G05910 | 13.8893 | 25.3034 |
| AT5G09870 | 13.8824 | 18.077 |
| AT2G47350 | 13.8818 | 13.2609 |
| AT1G07110 | 13.8766 | 14.4201 |
| AT1G11175 | 13.8755 | 6.91654 |
| AT5G66000 | 13.8724 | 3.66417 |
| AT1G68760 | 13.8704 | 4.32462 |
| AT5G04280 | 13.8645 | 20.2231 |
| AT2G25800 | 13.8643 | 13.634 |
| AT4G28652 | 13.8604 | 10.6854 |
| AT1G27150 | 13.86 | 18.5985 |
| AT5G48960 | 13.851 | 14.0182 |
| AT5G14020 | 13.8509 | 76.169 |
| AT2G17480 | 13.8496 | 26.652 |
| AT1G16540 | 13.8431 | 15.2333 |
| AT5G63800 | 13.8423 | 37.391 |
| AT5G52870 | 13.8423 | 13.741 |
| AT1G07473 | 13.842 | 4.41395 |
| AT5G03320 | 13.8361 | 8.41698 |
| AT3G03020 | 13.8296 | 17.4117 |
| AT4G22260 | 13.8262 | 16.6921 |
| AT2G48010 | 13.8136 | 12.6228 |
| AT4G36120 | 13.811 | 2.36961 |
| AT2G34140 | 13.8056 | 23.6382 |
| AT5G38840 | 13.8043 | 16.5255 |
| AT5G27490 | 13.8008 | 12.0482 |
| AT1G65280 | 13.7987 | 18.9222 |
| AT5G26240 | 13.7914 | 10.7401 |
| AT1G15500 | 13.788 | 30.2841 |
| AT1G35516 | 13.7817 | 14.9641 |
| AT5G02740 | 13.7813 | 21.8181 |
| AT5G47080 | 13.7802 | 21.7949 |
| AT5G57000 | 13.7773 | 6.52341 |
| AT4G14860 | 13.7763 | 1.30665 |
| AT2G37290 | 13.7737 | 2.26582 |
| AT4G00630 | 13.767 | 14.0065 |
| AT4G11370 | 13.7528 | 9.60438 |
| AT3G09090 | 13.733 | 21.6567 |
| AT1G18290 | 13.7301 | 2.53331 |
| AT1G26120 | 13.7206 | 11.757 |
| AT1G27510 | 13.7193 | 16.082 |
| AT1G08010 | 13.716 | 23.6356 |
| AT4G38080 | 13.7156 | 6.35553 |
| AT4G16180 | 13.7154 | 17.629 |
| AT3G49690 | 13.715 | 18.2123 |
| AT1G17690 | 13.7106 | 14.7403 |
| AT2G01330 | 13.7087 | 9.73231 |
| AT3G47980 | 13.7067 | 4.94974 |
| AT3G05810 | 13.7052 | 25.016 |
| AT1G22180 | 13.702 | 12.2123 |
| AT4G14750 | 13.6953 | 5.89403 |
| AT5G10870 | 13.6952 | 9.7029 |
| AT5G14480 | 13.6914 | 6.03747 |
| AT4G21534 | 13.6904 | 5.38207 |
| AT3G18035 | 13.6779 | 23.8181 |
| AT1G06070 | 13.6697 | 19.3271 |
| AT3G52500 | 13.6631 | 23.9173 |
| AT3G13800 | 13.6619 | 13.9697 |
| AT2G39500 | 13.655 | 21.9249 |
| AT1G01740 | 13.6531 | 2.05532 |
| AT1G16610 | 13.6302 | 22.4732 |
| AT5G58920 | 13.6236 | 22.6822 |
| AT2G31585 | 13.6199 | 9.48704 |
| AT5G64200 | 13.6191 | 24.2695 |
| AT3G50860 | 13.6153 | 8.26706 |
| AT5G05190 | 13.6129 | 7.57129 |
| AT5G57410 | 13.6071 | 20.2081 |
| AT4G32720 | 13.5969 | 62.0473 |
| AT3G16330 | 13.5959 | 12.9193 |
| AT1G67960 | 13.5924 | 11.7876 |
| AT5G66820 | 13.5814 | 5.01974 |
| AT2G44940 | 13.5784 | 11.6212 |
| AT4G29660 | 13.572 | 10.5675 |
| AT4G09580 | 13.567 | 18.0154 |
| AT3G07150 | 13.5663 | 8.32752 |
| AT5G41360 | 13.5659 | 11.5876 |
| AT5G58575 | 13.5601 | 10.9349 |
| AT3G09030 | 13.5575 | 8.47189 |
| AT5G18780 | 13.5575 | 4.58249 |
| AT1G03520 | 13.5521 | 8.98912 |
| AT2G46280 | 13.5518 | 44.3914 |
| AT2G45860 | 13.5456 | 43.1658 |
| AT4G30110 | 13.5443 | 6.75426 |
| AT5G58240 | 13.5379 | 22.9083 |
| AT5G57620 | 13.5367 | 27.1447 |
| AT4G05053 | 13.5349 | 5.67019 |
| AT1G55250 | 13.5344 | 17.6778 |
| AT1G15740 | 13.5343 | 12.6633 |
| AT5G51150 | 13.5323 | 13.9362 |
| AT4G26050 | 13.5315 | 2.60432 |
| AT1G60430 | 13.5203 | 8.86509 |
| AT3G62700 | 13.5191 | 20.5892 |
| AT1G76960 | 13.5143 | 3.41315 |
| AT5G66675 | 13.5032 | 21.7172 |
| AT5G52830 | 13.5005 | 9.48028 |
| AT1G74960 | 13.4977 | 34.5203 |
| AT4G14368 | 13.4937 | 2.97325 |
| AT5G46070 | 13.4912 | 20.0489 |
| AT2G03350 | 13.488 | 28.7432 |
| AT3G44735 | 13.4868 | 11.3848 |
| AT2G04865 | 13.4863 | 11.9187 |
| AT4G11240 | 13.4854 | 20.9455 |
| AT1G64990 | 13.4851 | 15.6061 |
| AT1G30750 | 13.482 | 12.4391 |
| AT4G19660 | 13.4816 | 13.1455 |
| AT4G25870 | 13.4805 | 11.4903 |
| AT1G72390 | 13.4785 | 11.8049 |
| AT1G64570 | 13.4784 | 9.20011 |
| AT1G67550 | 13.4768 | 12.9857 |
| AT4G21860 | 13.4765 | 16.9739 |
| AT5G15440 | 13.4725 | 13.2602 |
| AT2G35910 | 13.4724 | 10.4503 |
| AT2G29140 | 13.4654 | 10.2987 |
| AT3G29635 | 13.4612 | 1.97546 |
| AT1G72070 | 13.4609 | 11.088 |
| AT3G16990 | 13.4606 | 5.2431 |
| AT3G56040 | 13.4523 | 14.9913 |
| AT1G73790 | 13.4515 | 13.1943 |
| AT5G23090 | 13.4511 | 12.2251 |
| AT5G45340 | 13.4426 | 28.7659 |
| AT1G08700 | 13.4351 | 14.5195 |
| AT4G10750 | 13.4331 | 10.5392 |
| AT1G71800 | 13.4288 | 14.4919 |
| AT2G01410 | 13.4282 | 5.49585 |
| AT5G47660 | 13.4237 | 13.9367 |
| AT1G31440 | 13.4227 | 17.9201 |
| AT1G72470 | 13.4206 | 14.4 |
| AT5G13530 | 13.4203 | 14.4782 |
| AT1G23390 | 13.4122 | 5.41725 |
| AT4G11270 | 13.4075 | 11.9024 |
| AT3G47640 | 13.4019 | 28.4253 |
| AT3G12680 | 13.4009 | 15.7401 |
| AT5G66260 | 13.393 | 0.633899 |
| AT5G17400 | 13.3882 | 16.0389 |
| AT1G11950 | 13.384 | 8.28614 |
| AT5G19680 | 13.3811 | 20.2968 |
| AT1G32810 | 13.3785 | 15.1895 |
| AT4G37760 | 13.3689 | 10.5756 |
| AT1G33280 | 13.3652 | 88.2689 |
| AT3G17120 | 13.3649 | 25.6181 |
| AT1G18630 | 13.3625 | 15.5809 |
| AT4G03200 | 13.3593 | 14.6657 |
| AT2G01090 | 13.3498 | 12.3877 |
| AT1G24610 | 13.3426 | 13.2551 |
| AT5G07330 | 13.3425 | 30.2682 |
| AT5G56220 | 13.3409 | 10.6749 |
| AT3G29270 | 13.3396 | 10.9864 |
| AT3G55880 | 13.3384 | 15.6123 |
| AT4G40080 | 13.3302 | 8.27767 |
| AT2G18917 | 13.3254 | 9.15852 |
| AT2G42430 | 13.3239 | 2.96005 |
| AT2G20490 | 13.3169 | 47.7876 |
| AT1G73980 | 13.3078 | 12.1021 |
| AT4G36110 | 13.3057 | 4.01619 |
| AT3G61460 | 13.298 | 18.049 |
| AT3G53365 | 13.2811 | 5.97782 |
| AT5G07150 | 13.2811 | 0.705037 |
| AT5G25510 | 13.278 | 14.0722 |
| AT2G40600 | 13.2779 | 17.6486 |
| AT3G48750 | 13.2776 | 29.7572 |
| AT4G10340 | 13.2597 | 7.2613 |
| AT1G02560 | 13.2576 | 37.5611 |
| AT4G11790 | 13.2555 | 19.3303 |
| AT5G05340 | 13.2543 | 2.78239 |
| AT1G03210 | 13.2532 | 23.7475 |
| AT5G54200 | 13.2521 | 11.1791 |
| AT1G22110 | 13.2423 | 2.75462 |
| AT1G47250 | 13.2402 | 20.2976 |
| AT5G62130 | 13.2294 | 6.02587 |
| AT5G61880 | 13.2275 | 24.6186 |
| AT2G45620 | 13.2267 | 17.694 |
| AT5G11240 | 13.2217 | 14.2443 |
| AT4G19185 | 13.2215 | 11.5616 |
| AT3G46290 | 13.2212 | 10.0473 |
| AT5G62290 | 13.2187 | 13.7621 |
| AT1G80860 | 13.2186 | 18.3431 |
| AT5G10940 | 13.2147 | 13.4327 |
| AT3G03440 | 13.2139 | 8.81916 |
| AT3G56750 | 13.2111 | 14.9837 |
| AT2G03620 | 13.2081 | 11.2454 |
| AT4G01350 | 13.2072 | 1.56638 |
| AT4G00900 | 13.2061 | 7.81768 |
| AT2G33835 | 13.2058 | 8.57477 |
| AT1G48605 | 13.2025 | 12.7242 |
| AT1G49450 | 13.1922 | 1.0766 |
| AT4G33690 | 13.1887 | 17.1453 |
| AT3G15360 | 13.1831 | 9.49107 |
| AT1G61290 | 13.1794 | 3.3257 |
| AT3G60360 | 13.1716 | 25.1429 |
| AT4G40042 | 13.1709 | 7.84348 |
| AT1G74060 | 13.1704 | 49.8944 |
| AT3G44750 | 13.162 | 72.838 |
| AT3G07280 | 13.1614 | 13.5431 |
| AT3G18710 | 13.1608 | 6.77724 |
| AT2G17220 | 13.1594 | 26.7181 |
| AT4G19870 | 13.1502 | 8.06574 |
| AT2G15390 | 13.1441 | 1.30192 |
| AT2G21550 | 13.1435 | 11.1728 |
| AT4G36400 | 13.1337 | 7.23641 |
| AT3G04350 | 13.1273 | 12.555 |
| AT4G15880 | 13.1246 | 17.3063 |
| AT5G55660 | 13.122 | 36.1259 |
| AT3G13550 | 13.118 | 11.1595 |
| AT4G31500 | 13.1178 | 18.3047 |
| AT3G06470 | 13.1107 | 3.91154 |
| AT2G41440 | 13.1091 | 9.08729 |
| AT2G42890 | 13.1079 | 23.8932 |
| AT3G46220 | 13.1057 | 12.605 |
| AT1G80000 | 13.1002 | 6.74567 |
| AT1G03980 | 13.0798 | 8.01593 |
| AT1G67570 | 13.0727 | 4.64875 |
| AT3G60520 | 13.0695 | 28.5984 |
| AT1G09520 | 13.0635 | 13.2419 |
| AT4G28400 | 13.0582 | 23.2483 |
| AT4G21120 | 13.0569 | 14.6425 |
| AT4G16780 | 13.0536 | 9.15422 |
| AT1G48450 | 13.0534 | 13.9525 |
| AT4G00240 | 13.0529 | 4.07637 |
| AT4G27540 | 13.0467 | 6.66701 |
| AT2G17340 | 13.0464 | 12.1415 |
| AT1G64550 | 13.0446 | 30.6326 |
| AT1G67810 | 13.0372 | 10.1887 |
| AT1G44960 | 13.0349 | 12.1819 |
| AT2G38320 | 13.0348 | 1.89896 |
| AT5G47780 | 13.0323 | 26.8972 |
| AT1G51720 | 13.0273 | 16.4454 |
| AT5G14680 | 13.0182 | 16.638 |
| AT2G43940 | 13.0163 | 20.3151 |
| AT3G27080 | 13.0154 | 19.9235 |
| AT1G72550 | 13.0073 | 32.0278 |
| AT1G05805 | 13.0071 | 20.752 |
| AT5G55770 | 13.0036 | 0.0413948 |
| AT2G29900 | 13.0006 | 6.84417 |
| AT3G22060 | 12.9982 | 42.3939 |
| AT5G54380 | 12.9962 | 16.0342 |
| AT2G18990 | 12.9935 | 17.2213 |
| AT2G15530 | 12.9922 | 10.4192 |
| AT1G72860 | 12.9882 | 1.33751 |
| AT1G50030 | 12.9873 | 10.2233 |
| AT3G14960 | 12.9784 | 7.51201 |
| AT3G12977 | 12.9739 | 5.76451 |
| AT3G55120 | 12.97 | 0.896791 |
| AT3G60640 | 12.9669 | 17.3422 |
| AT2G35000 | 12.9662 | 6.21068 |
| AT5G05860 | 12.966 | 4.96653 |
| AT5G13340 | 12.9644 | 11.0713 |
| AT4G04210 | 12.954 | 17.6711 |
| AT3G15770 | 12.9517 | 15.4155 |
| AT2G29070 | 12.9498 | 11.0105 |
| AT3G60930.2 | 12.9498 | 4.89775 |
| AT4G32300 | 12.9491 | 3.74348 |
| AT2G21780 | 12.9486 | 8.12587 |
| AT3G59340 | 12.9469 | 1.55281 |
| AT1G56060 | 12.9375 | 7.37638 |
| AT3G17430 | 12.9301 | 14.2004 |
| AT2G36210 | 12.9229 | 2.74903 |
| AT2G44870 | 12.9191 | 15.3508 |
| AT4G40065 | 12.9175 | 5.14436 |
| AT1G27390 | 12.916 | 38.762 |
| AT1G54990 | 12.9143 | 22.0593 |
| AT1G22220 | 12.9136 | 1.37217 |
| AT1G78060 | 12.9001 | 3.86713 |
| AT3G07220 | 12.8996 | 7.63928 |
| AT4G08220 | 12.8805 | 12.6655 |
| AT5G63120 | 12.8796 | 14.7523 |
| AT2G26310 | 12.8761 | 10.2615 |
| AT3G53630 | 12.8702 | 13.4885 |
| AT2G42330 | 12.8676 | 11.7228 |
| AT3G21430 | 12.864 | 12.2084 |
| AT2G34810 | 12.8607 | 0.647047 |
| AT1G73740 | 12.8598 | 8.27632 |
| AT2G43780 | 12.8545 | 22.9777 |
| AT5G42470 | 12.8523 | 14.7165 |
| AT2G36305 | 12.8483 | 9.74403 |
| AT1G30090 | 12.8422 | 7.38015 |
| AT3G05120 | 12.8384 | 15.2782 |
| AT2G02700 | 12.837 | 1.27248 |
| AT3G13882 | 12.8366 | 38.8936 |
| AT3G04470 | 12.8324 | 18.4579 |
| AT5G58510 | 12.8203 | 9.34381 |
| AT5G22040 | 12.8157 | 11.522 |
| AT1G19660 | 12.8064 | 2.78656 |
| AT1G03100 | 12.8034 | 3.14071 |
| AT1G63490 | 12.8022 | 13.9771 |
| AT2G45360 | 12.7995 | 3.75517 |
| AT1G59660 | 12.7969 | 9.56429 |
| AT1G51390 | 12.7936 | 13.7471 |
| AT1G04100 | 12.7933 | 6.23621 |
| AT1G07170 | 12.7903 | 32.2747 |
| AT3G62190 | 12.7888 | 7.89652 |
| AT4G15180 | 12.7883 | 12.4525 |
| AT3G19740 | 12.7814 | 19.0062 |
| AT3G49760 | 12.7805 | 5.78175 |
| AT5G49560 | 12.7799 | 3.98493 |
| AT5G55000 | 12.7741 | 8.77918 |
| AT3G57420 | 12.7683 | 8.15683 |
| AT1G77610 | 12.7652 | 40.9093 |
| AT1G47870 | 12.7616 | 15.295 |
| AT3G50080 | 12.7596 | 16.3393 |
| AT5G06190 | 12.7515 | 16.8587 |
| AT3G28430 | 12.7507 | 7.34323 |
| AT1G76065 | 12.7494 | 5.87515 |
| AT1G78620 | 12.7297 | 9.69386 |
| AT5G05460 | 12.7284 | 19.7877 |
| AT1G23060 | 12.7268 | 47.5709 |
| AT4G02150 | 12.7264 | 28.8285 |
| AT3G19840 | 12.7226 | 15.152 |
| AT4G16430 | 12.7173 | 16.0605 |
| AT3G57300 | 12.7143 | 15.4455 |
| AT2G25850 | 12.7106 | 11.0493 |
| AT2G40300 | 12.7057 | 15.6131 |
| AT1G02920 | 12.7012 | 74.2917 |
| AT5G56270 | 12.6972 | 12.2685 |
| AT1G16825 | 12.6849 | 10.8628 |
| AT3G27050 | 12.6809 | 8.93969 |
| AT2G15555 | 12.679 | 2.88111 |
| AT4G19220 | 12.6786 | 2.56004 |
| AT5G14970 | 12.678 | 11.8723 |
| AT1G05780 | 12.6721 | 9.58664 |
| AT2G48060 | 12.6713 | 7.851 |
| AT1G67140 | 12.6711 | 13.238 |
| AT2G47420 | 12.6656 | 13.0565 |
| AT3G29100 | 12.6644 | 9.00931 |
| AT1G55340 | 12.664 | 23.5239 |
| AT3G17300 | 12.6625 | 18.6253 |
| AT5G44800 | 12.6618 | 15.1249 |
| AT1G22730 | 12.6588 | 10.6614 |
| AT1G03030 | 12.6587 | 14.8799 |
| AT4G30750 | 12.6542 | 15.8191 |
| AT2G33210 | 12.6536 | 57.3312 |
| AT2G47770 | 12.6522 | 7.12061 |
| AT5G03370 | 12.6506 | 8.21392 |
| AT1G58025 | 12.6484 | 18.1986 |
| AT1G31350 | 12.6475 | 8.3051 |
| AT5G55910 | 12.6378 | 19.0954 |
| AT3G63120 | 12.6367 | 9.97128 |
| AT1G54115 | 12.6304 | 11.2421 |
| AT1G55170 | 12.6295 | 12.4905 |
| AT3G21215 | 12.6289 | 22.1727 |
| AT5G35210 | 12.628 | 13.3628 |
| AT2G15890 | 12.6248 | 15.7068 |
| AT3G21175 | 12.6221 | 12.3351 |
| AT2G46735 | 12.6173 | 3.85361 |
| AT1G44820 | 12.6169 | 8.5329 |
| AT5G65220 | 12.6122 | 17.607 |
| AT3G18380 | 12.5977 | 15.1101 |
| AT4G08810 | 12.597 | 15.2472 |
| AT3G09860 | 12.594 | 24.9035 |
| AT3G17910 | 12.5854 | 12.5079 |
| AT1G28685 | 12.5852 | 5.85708 |
| AT1G70950 | 12.5804 | 12.3091 |
| AT1G02570 | 12.5783 | 0.173528 |
| AT5G11800 | 12.5766 | 14.8773 |
| AT1G19170 | 12.5726 | 10.9785 |
| AT5G37130 | 12.5699 | 15.5151 |
| AT3G47800 | 12.5688 | 36.4577 |
| AT2G44680 | 12.5649 | 14.5428 |
| AT3G07690 | 12.5612 | 11.9322 |
| AT5G17440 | 12.5565 | 14.9369 |
| AT1G69010 | 12.5529 | 45.8924 |
| AT3G03970 | 12.5519 | 11.0608 |
| AT3G20330 | 12.5482 | 16.5193 |
| AT5G67210 | 12.545 | 3.08767 |
| AT1G59900 | 12.5432 | 28.5305 |
| AT1G16970 | 12.5421 | 7.79636 |
| AT4G02540 | 12.5413 | 8.46922 |
| AT4G26610 | 12.5309 | 17.6472 |
| AT5G63770 | 12.522 | 8.43224 |
| AT5G08780 | 12.5207 | 3.40009 |
| AT4G14965 | 12.5178 | 18.8081 |
| AT4G31430 | 12.5172 | 18.2276 |
| AT1G29357 | 12.5154 | 19.164 |
| AT4G30510 | 12.5128 | 9.20014 |
| AT5G45590 | 12.5114 | 16.6903 |
| AT2G23460 | 12.5108 | 19.1299 |
| AT4G26510 | 12.5085 | 15.3984 |
| AT5G17490 | 12.5023 | 2.31459 |
| AT2G42760 | 12.5014 | 5.45311 |
| AT3G09690 | 12.4959 | 24.3191 |
| AT5G06820 | 12.4835 | 1.25235 |
| AT4G18060 | 12.4805 | 23.105 |
| AT3G01420 | 12.4787 | 0.361786 |
| AT1G62422 | 12.4774 | 4.67216 |
| AT1G69840 | 12.4707 | 34.3177 |
| AT2G35010 | 12.4702 | 15.9983 |
| AT2G47400 | 12.4594 | 2.74819 |
| AT2G15900 | 12.4588 | 6.74064 |
| AT1G27900 | 12.4578 | 8.97687 |
| AT3G28950 | 12.4561 | 10.6891 |
| AT4G14340 | 12.4552 | 21.2361 |
| AT3G29970 | 12.454 | 16.1909 |
| AT3G02220 | 12.4539 | 31.4667 |
| AT5G01850 | 12.4522 | 4.30297 |
| AT1G73960 | 12.4287 | 13.5197 |
| AT2G35658 | 12.4222 | 7.56005 |
| AT1G78140 | 12.4206 | 8.82825 |
| AT1G15970 | 12.418 | 6.02011 |
| AT4G34215 | 12.4162 | 9.17042 |
| AT3G14920 | 12.4159 | 3.63744 |
| AT3G26560 | 12.4132 | 14.1054 |
| AT3G02180 | 12.4114 | 19.3733 |
| AT1G62290 | 12.4113 | 5.63716 |
| AT1G04180 | 12.406 | 24.7611 |
| AT4G05460 | 12.4043 | 11.4681 |
| AT3G46740 | 12.402 | 15.9274 |
| AT5G33320 | 12.3978 | 55.9428 |
| AT3G54826 | 12.3949 | 14.3183 |
| AT4G14630 | 12.3881 | 13.9196 |
| AT1G05280 | 12.3842 | 58.7869 |
| AT5G38850 | 12.3823 | 2.78548 |
| AT4G08960 | 12.3818 | 12.9027 |
| AT1G69060 | 12.3773 | 10.1805 |
| AT4G00450 | 12.3719 | 11.5255 |
| AT5G20360 | 12.3703 | 11.6703 |
| AT2G31345 | 12.3679 | 3.50687 |
| AT1G14850 | 12.3655 | 17.1205 |
| AT1G50120 | 12.3647 | 10.0964 |
| AT3G56940 | 12.3618 | 0.678618 |
| AT1G06390 | 12.3613 | 33.3425 |
| AT2G20142 | 12.3526 | 6.15924 |
| AT5G54830 | 12.3525 | 9.00025 |
| AT1G03280 | 12.3517 | 13.857 |
| AT3G16220 | 12.3511 | 11.5511 |
| AT3G03330 | 12.3476 | 11.9166 |
| AT5G16320 | 12.3469 | 8.54891 |
| AT3G47000 | 12.3437 | 13.0268 |
| AT3G27700 | 12.3401 | 11.8105 |
| AT1G67050 | 12.3242 | 3.47135 |
| AT3G49820 | 12.3158 | 2.82297 |
| AT1G80490 | 12.3147 | 20.0603 |
| AT1G05840 | 12.3135 | 15.38 |
| AT3G04480 | 12.3134 | 14.2821 |
| AT4G28025 | 12.3114 | 12.3778 |
| AT5G19460 | 12.311 | 11.5246 |
| AT5G56930 | 12.3097 | 11.9053 |
| AT4G25890 | 12.3057 | 27.3245 |
| AT5G48430 | 12.3038 | 9.01637 |
| AT4G32620 | 12.3021 | 16.3698 |
| AT1G45233 | 12.3011 | 15.5477 |
| AT1G48040 | 12.3002 | 8.19951 |
| AT4G25400 | 12.298 | 23.1304 |
| AT1G13570 | 12.2969 | 20.2652 |
| AT4G22190 | 12.2954 | 13.835 |
| AT5G18550 | 12.2943 | 13.7615 |
| AT5G44130 | 12.2916 | 0.797854 |
| AT5G20300 | 12.2801 | 10.6389 |
| AT5G39250 | 12.2786 | 4.94749 |
| AT3G15358 | 12.2757 | 6.31842 |
| AT1G11655 | 12.2745 | 0.133663 |
| AT3G45650 | 12.2711 | 12.1479 |
| AT5G14520 | 12.2698 | 43.334 |
| AT3G24860 | 12.2685 | 11.4252 |
| AT4G03120 | 12.268 | 23.9041 |
| AT1G29990 | 12.2651 | 37.4931 |
| AT3G08880 | 12.263 | 20.5873 |
| AT4G13590 | 12.2533 | 13.3614 |
| AT1G43620 | 12.2509 | 7.55867 |
| AT1G10690 | 12.239 | 24.3146 |
| AT4G27180 | 12.2352 | 11.1707 |
| AT1G04790 | 12.2288 | 12.9114 |
| AT4G17190 | 12.2276 | 50.5493 |
| AT3G48240 | 12.222 | 5.84715 |
| AT1G13280 | 12.2216 | 29.8827 |
| AT1G71710 | 12.2201 | 10.1347 |
| AT3G59520 | 12.2167 | 9.11483 |
| AT1G60995 | 12.2161 | 11.1791 |
| AT4G16146 | 12.2138 | 11.7333 |
| AT4G30500 | 12.2136 | 17.3979 |
| AT3G16190 | 12.2092 | 18.3957 |
| AT2G16405 | 12.2088 | 10.3462 |
| AT2G39000 | 12.2055 | 8.62003 |
| AT2G18030 | 12.2048 | 10.1499 |
| AT3G21290 | 12.1976 | 12.2832 |
| AT1G35660 | 12.1923 | 6.8939 |
| AT1G13870 | 12.1909 | 12.7615 |
| AT5G41750 | 12.1785 | 4.87351 |
| AT1G63980 | 12.1689 | 50.2259 |
| AT3G17600 | 12.1628 | 0.409842 |
| AT3G01510 | 12.1608 | 16.6964 |
| AT1G21525 | 12.1577 | 3.63113 |
| AT4G14900 | 12.1546 | 19.1214 |
| AT2G33440 | 12.1538 | 14.0377 |
| AT3G54740 | 12.1417 | 37.9831 |
| AT2G45700 | 12.1308 | 13.4445 |
| AT2G43770 | 12.1278 | 26.1223 |
| AT1G71400 | 12.1269 | 22.8107 |
| AT4G00740 | 12.1252 | 31.0699 |
| AT2G38450 | 12.1242 | 15.8399 |
| AT3G12020 | 12.1229 | 14.5484 |
| AT2G32130 | 12.1212 | 4.09047 |
| AT3G54470 | 12.1166 | 55.2769 |
| AT2G24545 | 12.1118 | 3.14438 |
| AT4G00440 | 12.1084 | 3.41702 |
| AT3G26040 | 12.1016 | 4.33962 |
| AT3G03290 | 12.098 | 1.09724 |
| AT4G15010 | 12.0928 | 9.28721 |
| AT3G06790 | 12.0894 | 16.5408 |
| AT1G11545 | 12.0766 | 10.6702 |
| AT4G11330 | 12.0765 | 11.9321 |
| AT1G31885 | 12.0701 | 0.130859 |
| AT1G66910 | 12.069 | 1.28736 |
| AT5G02490 | 12.0688 | 5.69627 |
| AT5G41180 | 12.0668 | 16.5553 |
| AT2G32140 | 12.0663 | 5.14406 |
| AT2G25720 | 12.0579 | 9.16631 |
| AT4G22270 | 12.054 | 8.04915 |
| AT5G41010 | 12.0511 | 96.2012 |
| AT5G02502 | 12.0467 | 26.9785 |
| AT4G36920 | 12.0447 | 13.9295 |
| AT4G27852 | 12.0398 | 2.59794 |
| AT2G38490 | 12.0333 | 0.92151 |
| AT1G10960 | 12.0329 | 3.50512 |
| AT1G09800 | 12.0316 | 8.43622 |
| AT5G37055 | 12.0305 | 10.3964 |
| AT3G07080 | 12.0289 | 4.29919 |
| AT3G09800 | 12.0207 | 22.9922 |
| AT5G07120 | 12.0187 | 14.3389 |
| AT4G24100 | 12.0179 | 15.9372 |
| AT1G22740 | 12.0154 | 14.6231 |
| AT2G31865 | 12.014 | 8.82693 |
| AT1G04290 | 12.0104 | 3.16395 |
| AT1G43850 | 12.0095 | 12.7173 |
| AT4G14980 | 12.0079 | 4.01286 |
| AT2G14255 | 12.0036 | 11.5641 |
| AT4G16360 | 12.0024 | 18.1717 |
| AT3G45070 | 11.9938 | 2.64868 |
| AT5G38690 | 11.9922 | 17.4726 |
| AT4G27230 | 11.9919 | 25.8115 |
| AT3G45740 | 11.9861 | 8.16005 |
| AT1G48620 | 11.9858 | 21.6635 |
| AT4G35850 | 11.9855 | 34.3779 |
| AT3G04320 | 11.984 | 118.005 |
| AT3G22220 | 11.9803 | 15.718 |
| AT3G48590 | 11.9801 | 3.28945 |
| AT1G48230 | 11.9693 | 23.7958 |
| AT2G41620 | 11.9643 | 14.9644 |
| AT3G15480 | 11.9612 | 52.8028 |
| AT4G19510 | 11.9608 | 7.06247 |
| AT2G46180 | 11.9581 | 13.3818 |
| AT1G05200 | 11.9453 | 25.7538 |
| AT3G54920 | 11.9435 | 23.0963 |
| AT1G52565 | 11.9372 | 26.8225 |
| AT5G46080 | 11.9369 | 6.52879 |
| AT3G03900 | 11.9359 | 10.7531 |
| AT3G05850.1 | 11.9341 | 8.46733 |
| AT3G21250 | 11.9331 | 21.5916 |
| AT1G22882 | 11.9275 | 9.90815 |
| AT2G41760 | 11.9261 | 7.00466 |
| AT1G07630 | 11.9229 | 11.4946 |
| AT1G09760 | 11.9221 | 39.7292 |
| AT1G74690 | 11.9168 | 42.0815 |
| AT1G04860 | 11.9135 | 12.8748 |
| AT2G46100 | 11.9105 | 7.51451 |
| AT5G19030 | 11.9066 | 9.28384 |
| AT3G16280 | 11.906 | 6.85895 |
| AT2G46930 | 11.9034 | 25.532 |
| AT5G50440 | 11.9016 | 7.92884 |
| AT1G66670 | 11.9012 | 23.5304 |
| AT2G46470 | 11.8965 | 17.4738 |
| AT5G64730 | 11.8915 | 14.9691 |
| AT3G25580 | 11.8882 | 15.4452 |
| AT3G20520 | 11.8833 | 2.10967 |
| AT5G51220 | 11.882 | 22.4424 |
| AT4G30080 | 11.8778 | 32.2976 |
| AT5G49980 | 11.8742 | 17.6525 |
| AT5G61220 | 11.8721 | 30.2218 |
| AT5G27030 | 11.8718 | 21.3541 |
| AT1G18950 | 11.8694 | 14.3713 |
| AT1G56110 | 11.861 | 86.2635 |
| AT5G09740 | 11.8606 | 11.4479 |
| AT3G51950 | 11.8595 | 10.7658 |
| AT5G52410 | 11.8535 | 13.688 |
| AT4G08980 | 11.8534 | 22.818 |
| AT4G16840 | 11.8441 | 7.59022 |
| AT3G54170 | 11.8414 | 20.7128 |
| AT2G41190 | 11.8403 | 4.56825 |
| AT4G25560 | 11.8331 | 3.54028 |
| AT1G44100 | 11.8274 | 4.63074 |
| AT5G17620 | 11.8236 | 16.2219 |
| AT4G00525 | 11.8205 | 7.07306 |
| AT4G34270 | 11.816 | 20.4185 |
| AT1G78800 | 11.8074 | 10.3559 |
| AT5G18360 | 11.8011 | 7.15552 |
| AT5G60540 | 11.7995 | 22.7244 |
| AT4G33625 | 11.7982 | 14.1599 |
| AT1G05680 | 11.7951 | 106.525 |
| AT5G14780 | 11.7934 | 8.71461 |
| AT4G15330 | 11.7934 | 6.51914 |
| AT1G25580 | 11.7929 | 13.9725 |
| AT4G01750 | 11.7881 | 20.0509 |
| AT4G14610 | 11.7875 | 1.74873 |
| AT4G14680 | 11.781 | 36.5862 |
| AT4G21200 | 11.7754 | 0.22266 |
| AT3G20860 | 11.7719 | 2.99603 |
| AT2G17020 | 11.7682 | 16.085 |
| AT5G05610 | 11.7658 | 30.2184 |
| AT5G17370 | 11.7646 | 10.5735 |
| AT5G24070 | 11.763 | 64.6283 |
| AT4G14780 | 11.7601 | 0.181057 |
| AT5G51890 | 11.751 | 2.68105 |
| AT4G19191 | 11.7491 | 7.87737 |
| AT4G19550 | 11.7445 | 10.0399 |
| AT2G44180 | 11.7417 | 36.6173 |
| AT4G09760 | 11.7407 | 1.28028 |
| AT5G02240 | 11.738 | 21.3465 |
| AT4G32330 | 11.7314 | 39.6759 |
| AT3G26640 | 11.7309 | 10.414 |
| AT5G02090 | 11.7274 | 8.3523 |
| AT1G53510 | 11.7267 | 11.9918 |
| AT3G24660 | 11.7206 | 8.13957 |
| AT1G01960 | 11.7191 | 30.0203 |
| AT1G62430 | 11.7167 | 18.2195 |
| AT2G40490 | 11.7159 | 8.55802 |
| AT2G20810 | 11.7051 | 19.8103 |
| AT2G39700 | 11.7038 | 9.91237 |
| AT5G24600 | 11.7031 | 13.5375 |
| AT4G23330 | 11.6961 | 7.10975 |
| AT4G11290 | 11.6934 | 44.2774 |
| AT1G08320 | 11.6862 | 0.675343 |
| AT5G62440 | 11.681 | 39.5867 |
| AT2G26770 | 11.6795 | 13.0812 |
| AT2G37990 | 11.6723 | 47.5459 |
| AT2G31955 | 11.672 | 30.6907 |
| AT5G65650 | 11.668 | 12.7401 |
| AT1G21160 | 11.662 | 13.4648 |
| AT3G59600 | 11.656 | 30.6416 |
| AT1G61070 | 11.6557 | 11.5186 |
| AT5G24350 | 11.6557 | 9.5808 |
| AT5G52280 | 11.651 | 12.8698 |
| AT5G20550 | 11.649 | 15.2678 |
| AT5G05270 | 11.6427 | 2.74408 |
| AT4G30350 | 11.6384 | 7.78012 |
| AT3G49650 | 11.6355 | 2.72315 |
| AT4G34620 | 11.6305 | 16.0571 |
| AT4G01690 | 11.6275 | 10.7521 |
| AT4G36360 | 11.6213 | 40.2905 |
| AT1G51370 | 11.619 | 3.40115 |
| AT5G55630 | 11.6181 | 5.80194 |
| AT1G52340 | 11.6098 | 19.943 |
| AT4G26810 | 11.6018 | 9.84106 |
| AT2G35490 | 11.5981 | 11.3952 |
| AT5G25440 | 11.5967 | 18.0118 |
| AT3G62410 | 11.5926 | 13.2583 |
| AT1G26160 | 11.5902 | 11.2303 |
| AT2G16940 | 11.5851 | 22.324 |
| AT5G53150 | 11.5848 | 11.902 |
| AT1G24706 | 11.5771 | 13.395 |
| AT1G67970 | 11.574 | 10.2442 |
| AT4G21610 | 11.5624 | 8.97429 |
| AT5G41260 | 11.5614 | 14.7864 |
| AT3G55730 | 11.5613 | 10.4092 |
| AT1G55000 | 11.5574 | 9.22197 |
| AT5G40630 | 11.5468 | 10.1861 |
| AT1G20120 | 11.5468 | 3.20837 |
| AT1G20190 | 11.545 | 57.9073 |
| AT3G09240 | 11.5432 | 0.51995 |
| AT1G67280 | 11.5384 | 17.915 |
| AT1G17680 | 11.5383 | 9.97837 |
| AT5G23710 | 11.5365 | 12.2435 |
| AT1G49160 | 11.5342 | 5.27092 |
| AT4G34030 | 11.5317 | 13.4876 |
| AT4G31020 | 11.5297 | 1.41059 |
| AT3G13530 | 11.5274 | 10.4787 |
| AT3G03090 | 11.5271 | 7.91258 |
| AT3G48460 | 11.5256 | 38.3683 |
| AT1G09160 | 11.5242 | 17.2466 |
| AT1G01790 | 11.5239 | 6.13171 |
| AT5G56660 | 11.5216 | 5.80045 |
| AT4G18450 | 11.5197 | 24.2095 |
| AT4G27690 | 11.5191 | 20.5022 |
| AT2G29590 | 11.5177 | 6.19069 |
| AT2G21060 | 11.5172 | 45.3419 |
| AT4G01037 | 11.5117 | 7.11228 |
| AT1G48970 | 11.509 | 10.1354 |
| AT3G06290 | 11.507 | 10.1517 |
| AT2G20060 | 11.5039 | 44.7854 |
| AT5G64760 | 11.5001 | 18.8968 |
| AT3G16430 | 11.4996 | 34.1336 |
| AT4G27745 | 11.4946 | 5.86434 |
| AT1G77420 | 11.4944 | 11.8583 |
| AT3G63520 | 11.493 | 5.38195 |
| AT5G28040 | 11.4926 | 9.19678 |
| AT3G11773 | 11.4881 | 1.9608 |
| AT2G23985 | 11.4863 | 3.68226 |
| AT2G43620 | 11.4845 | 14.3745 |
| AT2G34357 | 11.4817 | 15.1396 |
| AT3G51440 | 11.4817 | 7.45937 |
| AT1G64380 | 11.4781 | 0.554446 |
| AT4G37210 | 11.4747 | 20.5678 |
| AT1G19210 | 11.4724 | 2.3099 |
| AT5G23080 | 11.4721 | 15.6511 |
| AT5G15070 | 11.4711 | 16.5888 |
| AT3G13990 | 11.4702 | 14.5866 |
| AT2G41520 | 11.4693 | 11.4912 |
| AT5G16390 | 11.4652 | 33.2857 |
| AT1G20900 | 11.4622 | 18.9172 |
| AT3G23080 | 11.4611 | 9.27682 |
| AT5G15220 | 11.456 | 20.9883 |
| AT1G14240 | 11.4482 | 22.1674 |
| AT2G20830 | 11.4412 | 9.01724 |
| AT4G31790 | 11.4402 | 19.4559 |
| AT4G15110 | 11.4401 | 15.9946 |
| AT1G55805 | 11.438 | 13.0982 |
| AT3G05390 | 11.4318 | 28.2789 |
| AT2G02780 | 11.4312 | 21.0001 |
| AT3G27000 | 11.4299 | 12.5385 |
| AT1G62045 | 11.4263 | 3.30152 |
| AT3G20830 | 11.4152 | 2.05503 |
| AT4G23430 | 11.4139 | 22.4361 |
| AT2G19540 | 11.405 | 23.8375 |
| AT1G17100 | 11.4006 | 7.85824 |
| AT1G69310 | 11.3989 | 5.12364 |
| AT1G78670 | 11.3962 | 7.97271 |
| AT2G32760 | 11.392 | 10.8148 |
| AT5G15680 | 11.3914 | 9.40839 |
| AT3G03980 | 11.3821 | 5.25118 |
| AT5G18540 | 11.3794 | 10.1885 |
| AT2G02620 | 11.374 | 0.682936 |
| AT4G25315 | 11.3656 | 17.9048 |
| AT1G13110 | 11.3656 | 2.25497 |
| AT1G24300 | 11.3637 | 8.82187 |
| AT3G47120 | 11.3622 | 9.07008 |
| AT3G19400 | 11.3589 | 9.11656 |
| AT3G54660 | 11.3546 | 17.2933 |
| AT1G14310 | 11.3525 | 7.1659 |
| AT5G58440 | 11.3448 | 22.1512 |
| AT5G03470 | 11.3439 | 20.4245 |
| AT1G56440 | 11.3399 | 12.6954 |
| AT3G58630 | 11.3319 | 6.51943 |
| AT2G06520 | 11.3292 | 5.91814 |
| AT2G39980 | 11.3216 | 8.1978 |
| AT4G28220 | 11.3198 | 18.1924 |
| AT2G32950 | 11.3181 | 7.38728 |
| AT1G17285 | 11.3118 | 75.8551 |
| AT2G22740 | 11.3095 | 12.8749 |
| AT5G51230 | 11.309 | 17.8577 |
| AT5G50810 | 11.3084 | 60.7798 |
| AT1G62305 | 11.3033 | 9.52048 |
| AT3G56030 | 11.2918 | 3.8901 |
| AT3G14910 | 11.2904 | 8.33927 |
| AT1G12775 | 11.2893 | 3.94567 |
| AT5G06770 | 11.2873 | 5.32768 |
| AT1G14560 | 11.2813 | 7.37954 |
| AT5G50760 | 11.2801 | 0.249566 |
| AT2G13370 | 11.2797 | 10.1676 |
| AT2G27190 | 11.2766 | 28.9862 |
| AT4G03180 | 11.2756 | 22.2547 |
| AT5G22110 | 11.2754 | 10.0567 |
| AT2G40430 | 11.2692 | 41.6321 |
| AT3G44510 | 11.2669 | 1.11307 |
| AT5G09760 | 11.2659 | 15.2425 |
| AT2G45520 | 11.2639 | 26.093 |
| AT5G46050 | 11.2631 | 3.73381 |
| AT5G12860 | 11.2578 | 26.165 |
| AT5G09770 | 11.2546 | 29.3881 |
| AT5G05310 | 11.2544 | 5.80129 |
| AT1G16790 | 11.2514 | 8.20425 |
| AT3G18250 | 11.2414 | 2.99608 |
| AT5G44030 | 11.2382 | 2.64624 |
| AT5G66950 | 11.2368 | 11.5289 |
| AT1G68150 | 11.2352 | 23.9172 |
| AT1G68585 | 11.2326 | 3.35535 |
| AT5G41300 | 11.2169 | 1.7369 |
| AT1G32500 | 11.2163 | 10.8385 |
| AT1G09840 | 11.215 | 10.4008 |
| AT3G61680 | 11.2089 | 10.5873 |
| AT1G33600 | 11.2081 | 7.50182 |
| AT4G14746 | 11.1907 | 22.8404 |
| AT4G10430 | 11.1899 | 14.1241 |
| AT2G20500 | 11.1816 | 9.43091 |
| AT5G24090 | 11.1754 | 4.79526 |
| AT1G08450 | 11.1737 | 20.084 |
| AT4G06599 | 11.1695 | 11.4652 |
| AT5G09350 | 11.1648 | 10.149 |
| AT3G18310 | 11.1609 | 8.24799 |
| AT1G31070 | 11.1584 | 20.2995 |
| AT5G14760 | 11.158 | 1.50731 |
| AT3G09890 | 11.1539 | 10.5743 |
| AT3G02530 | 11.1522 | 47.7101 |
| AT3G55360 | 11.1495 | 24.6463 |
| AT1G69780 | 11.149 | 7.24191 |
| AT1G45180 | 11.1446 | 9.39208 |
| AT4G14530 | 11.133 | 2.99793 |
| AT3G02760 | 11.1298 | 24.3994 |
| AT5G10710 | 11.1232 | 15.1551 |
| AT1G18800 | 11.1168 | 41.8453 |
| AT1G49970 | 11.1125 | 15.4507 |
| AT2G37430 | 11.1101 | 8.18978 |
| AT1G18450 | 11.1091 | 28.5708 |
| AT5G01430 | 11.1075 | 10.4547 |
| AT4G25692 | 11.1071 | 6.845 |
| AT2G30400 | 11.1031 | 14.938 |
| AT1G54217 | 11.1002 | 3.93887 |
| AT4G17905 | 11.0973 | 2.20958 |
| AT2G47250 | 11.0968 | 25.6869 |
| AT1G50440 | 11.0936 | 10.9604 |
| AT5G60060 | 11.0925 | 1.69081 |
| AT1G60960 | 11.0916 | 5.91641 |
| AT3G16040 | 11.0913 | 1.65716 |
| AT4G39170 | 11.0901 | 10.7358 |
| AT1G48380 | 11.0889 | 7.95362 |
| AT2G42080 | 11.085 | 8.34819 |
| AT1G48550 | 11.0839 | 6.90382 |
| AT4G20880 | 11.082 | 6.41682 |
| AT5G14270 | 11.076 | 16.9785 |
| AT5G17840 | 11.0732 | 17.8028 |
| AT2G32810 | 11.07 | 9.17687 |
| AT1G27520 | 11.062 | 14.1451 |
| AT5G65780 | 11.0585 | 14.4616 |
| AT3G59810 | 11.0548 | 37.0124 |
| AT4G26965 | 11.0525 | 13.0151 |
| AT2G47160 | 11.0516 | 9.13898 |
| AT4G11670 | 11.0464 | 8.13462 |
| AT3G03500 | 11.0456 | 108.026 |
| AT2G32320 | 11.0331 | 11.063 |
| AT2G29390 | 11.0316 | 29.8157 |
| AT5G53470 | 11.0309 | 13.9419 |
| AT3G50530 | 11.03 | 23.162 |
| AT4G38420 | 11.0293 | 4.69104 |
| AT1G80010 | 11.0233 | 5.1537 |
| AT3G02950 | 11.0208 | 15.651 |
| AT1G70620 | 11.0176 | 14.0086 |
| AT2G34750 | 11.011 | 33.6104 |
| AT5G61910 | 11.0097 | 8.80545 |
| AT1G14260 | 11.0077 | 7.6386 |
| AT5G54930 | 11.0069 | 12.1276 |
| AT5G52920 | 11.0057 | 65.8539 |
| AT5G37670 | 11.0017 | 26.9752 |
| AT1G08210 | 11.0014 | 20.0787 |
| AT4G36940 | 10.9937 | 11.688 |
| AT1G80510 | 10.9851 | 9.96513 |
| AT1G52130 | 10.9839 | 0.117064 |
| AT3G22960 | 10.9819 | 42.5505 |
| AT1G55510 | 10.9818 | 4.88325 |
| AT5G23510 | 10.9811 | 16.7659 |
| AT2G34970 | 10.977 | 12.7171 |
| AT5G27210 | 10.9763 | 12.3013 |
| AT1G53290 | 10.9751 | 12.7279 |
| AT3G20320 | 10.9697 | 14.3231 |
| AT5G26610 | 10.9695 | 12.8615 |
| AT1G12350 | 10.9676 | 14.4343 |
| AT3G61898 | 10.9612 | 9.37508 |
| AT3G58560 | 10.9586 | 13.5092 |
| AT3G06340 | 10.9579 | 10.8671 |
| AT5G61010 | 10.9526 | 16.1892 |
| AT5G10770 | 10.952 | 1.24741 |
| AT1G50710 | 10.9476 | 11.5415 |
| AT3G47050 | 10.9459 | 0.183671 |
| AT2G27760 | 10.9421 | 10.3963 |
| AT1G16916 | 10.9418 | 14.1495 |
| AT5G04440 | 10.9414 | 9.17827 |
| AT5G52882 | 10.9377 | 30.242 |
| AT4G37410 | 10.9341 | 33.907 |
| AT1G45150 | 10.9339 | 6.26017 |
| AT4G14305 | 10.9317 | 8.79506 |
| AT4G17070 | 10.9188 | 6.27452 |
| AT1G48860 | 10.9186 | 20.6612 |
| AT3G59310 | 10.9184 | 8.91958 |
| AT3G13860 | 10.9142 | 29.3709 |
| AT2G17070 | 10.9019 | 2.45931 |
| AT5G48240 | 10.9016 | 40.4157 |
| AT4G35540 | 10.893 | 10.6552 |
| AT1G50730 | 10.89 | 9.81908 |
| AT4G02650 | 10.8851 | 2.52248 |
| AT2G40770 | 10.8834 | 9.34731 |
| AT2G02560 | 10.8756 | 24.9803 |
| AT5G28646 | 10.8735 | 10.4243 |
| AT4G33020 | 10.8718 | 0.54834 |
| AT3G60210 | 10.8672 | 25.1536 |
| AT1G13970 | 10.8565 | 11.3441 |
| AT5G41110 | 10.8544 | 3.54203 |
| AT5G24800 | 10.8521 | 15.6981 |
| AT3G23490 | 10.8444 | 17.3098 |
| AT2G18670 | 10.8424 | 5.97758 |
| AT4G32250 | 10.8407 | 17.0597 |
| AT1G14710 | 10.8381 | 25.9543 |
| AT4G36970 | 10.835 | 5.56318 |
| AT1G73090 | 10.8318 | 6.13539 |
| AT1G24190 | 10.8211 | 12.8355 |
| AT2G35240 | 10.815 | 28.7742 |
| AT5G65770 | 10.8124 | 19.7479 |
| AT3G47820 | 10.8078 | 1.83904 |
| AT1G80550 | 10.8034 | 12.4694 |
| AT4G04720 | 10.8007 | 7.20556 |
| AT5G13090 | 10.7996 | 7.38835 |
| AT5G53550 | 10.7917 | 20.8516 |
| AT2G36080 | 10.7815 | 22.8273 |
| AT3G17715 | 10.7798 | 5.11964 |
| AT2G28620 | 10.7789 | 6.87676 |
| AT3G05937 | 10.7763 | 15.5745 |
| AT3G57080 | 10.7755 | 8.43616 |
| AT5G57815 | 10.7717 | 13.2356 |
| AT5G19485 | 10.766 | 15.3696 |
| AT5G22220 | 10.7628 | 9.10713 |
| AT1G50420 | 10.7626 | 6.86383 |
| AT3G06550 | 10.7411 | 35.3317 |
| AT1G27130 | 10.7401 | 13.0886 |
| AT4G27720 | 10.7365 | 35.4994 |
| AT5G04720 | 10.7326 | 21.2715 |
| AT2G15230 | 10.7233 | 13.491 |
| AT5G23290 | 10.7156 | 24.4897 |
| AT1G50140 | 10.7131 | 14.0426 |
| AT5G28220 | 10.7123 | 8.45979 |
| AT3G15000 | 10.7037 | 43.9662 |
| AT4G30940 | 10.7011 | 9.12704 |
| AT1G76550 | 10.7005 | 34.5947 |
| AT3G09180 | 10.6999 | 10.7829 |
| AT2G26350 | 10.6988 | 8.12227 |
| AT5G54855 | 10.698 | 13.0468 |
| AT3G11600 | 10.6881 | 2.5913 |
| AT2G18240 | 10.688 | 8.18269 |
| AT1G25480 | 10.6865 | 10.0841 |
| AT2G38280 | 10.6851 | 22.4704 |
| AT3G60630 | 10.684 | 4.55524 |
| AT1G70518 | 10.6746 | 5.48615 |
| AT1G65590 | 10.6735 | 11.4756 |
| AT3G46320 | 10.6729 | 24.9473 |
| AT1G07230 | 10.6718 | 12.3983 |
| AT1G08490 | 10.6648 | 20.5727 |
| AT2G20000 | 10.6594 | 13.2893 |
| AT2G16640 | 10.6495 | 15.6451 |
| AT2G23290 | 10.6476 | 7.01527 |
| AT2G34900 | 10.6475 | 17.1632 |
| AT1G08040 | 10.6393 | 11.2649 |
| AT1G13640 | 10.6362 | 23.9571 |
| AT3G04900 | 10.6306 | 0.518696 |
| AT2G31820 | 10.6275 | 17.9436 |
| AT5G65575 | 10.6273 | 10.6131 |
| AT5G65490 | 10.6263 | 21.7507 |
| AT1G80560 | 10.6229 | 29.1191 |
| AT1G32940 | 10.6206 | 5.04965 |
| AT3G63470 | 10.6189 | 11.2119 |
| AT1G29680 | 10.6132 | 2.99796 |
| AT1G50620 | 10.6123 | 12.2193 |
| AT3G04560 | 10.612 | 15.5467 |
| AT5G11880 | 10.6083 | 34.7592 |
| AT5G52030 | 10.6033 | 2.23232 |
| AT5G09510 | 10.6007 | 48.6064 |
| AT4G37090 | 10.6002 | 23.4233 |
| AT3G06940.1 | 10.5999 | 9.84663 |
| AT1G51770 | 10.5971 | 3.19391 |
| AT1G20030 | 10.5941 | 2.76791 |
| AT2G39840 | 10.5907 | 15.86 |
| AT1G02990 | 10.5905 | 10.9162 |
| AT2G46610 | 10.5903 | 5.03827 |
| AT2G40711 | 10.5887 | 3.79057 |
| AT3G46200 | 10.5793 | 9.06361 |
| AT4G37370 | 10.5756 | 3.63136 |
| AT3G59200 | 10.5718 | 9.56163 |
| AT1G57620 | 10.5683 | 23.796 |
| AT5G47750 | 10.5599 | 10.0556 |
| AT3G20910 | 10.5544 | 10.9163 |
| AT1G68620 | 10.5542 | 0.621648 |
| AT1G80750 | 10.5489 | 57.4875 |
| AT4G11160 | 10.5331 | 10.0475 |
| AT1G26900 | 10.5307 | 8.18618 |
| AT5G08185 | 10.5304 | 5.9855 |
| AT3G15750 | 10.5282 | 3.14541 |
| AT1G23540 | 10.5267 | 0.0640614 |
| AT3G07610 | 10.5266 | 6.99887 |
| AT1G69790 | 10.5225 | 1.20867 |
| AT5G22250 | 10.521 | 2.16107 |
| AT4G34555 | 10.5184 | 47.0744 |
| AT1G12850 | 10.5179 | 15.0536 |
| AT5G42460 | 10.5154 | 1.40553 |
| AT3G15355 | 10.5084 | 14.8523 |
| AT5G36160 | 10.5029 | 7.50819 |
| AT2G19560 | 10.4951 | 17.7438 |
| AT2G18170 | 10.495 | 17.8406 |
| AT4G23530 | 10.4926 | 2.36527 |
| AT5G63440 | 10.4889 | 19.879 |
| AT1G53770 | 10.4861 | 8.50571 |
| AT2G17975 | 10.4857 | 8.59919 |
| AT4G14390 | 10.4832 | 0.480931 |
| AT4G12710 | 10.4797 | 10.3115 |
| AT1G80600 | 10.4767 | 17.2273 |
| AT5G21105 | 10.4762 | 6.96529 |
| AT3G63340 | 10.4741 | 8.91827 |
| AT3G01300 | 10.4696 | 8.42903 |
| AT1G51630 | 10.4643 | 19.399 |
| AT4G07950 | 10.4619 | 7.54152 |
| AT1G61120 | 10.4585 | 12.4653 |
| AT1G04930 | 10.4556 | 4.78614 |
| AT1G72360 | 10.4537 | 21.6309 |
| AT2G39940 | 10.452 | 14.0685 |
| AT1G15125 | 10.4515 | 5.56598 |
| AT5G18070 | 10.4489 | 10.5323 |
| AT1G80570 | 10.4467 | 3.80956 |
| AT1G06050 | 10.4456 | 5.84132 |
| AT1G02110 | 10.4437 | 10.0412 |
| AT1G73500 | 10.4424 | 25.6603 |
| AT3G12590 | 10.4399 | 11.3724 |
| AT5G21950 | 10.4381 | 0.552717 |
| AT3G10490 | 10.4354 | 19.0131 |
| AT2G31270 | 10.4349 | 7.89038 |
| AT3G58810 | 10.4308 | 1.2688 |
| AT2G35650 | 10.4302 | 6.81304 |
| AT2G20495 | 10.4263 | 7.5998 |
| AT3G03310 | 10.4247 | 40.2835 |
| AT4G04780 | 10.424 | 14.913 |
| AT5G50020 | 10.4233 | 8.35871 |
| AT2G36330 | 10.4228 | 6.73845 |
| AT5G47370 | 10.422 | 16.3184 |
| AT2G44860 | 10.4207 | 53.7097 |
| AT5G47250 | 10.4161 | 1.71412 |
| AT4G29700 | 10.4146 | 6.1734 |
| AT2G35360 | 10.4116 | 8.17622 |
| AT1G71170 | 10.4097 | 5.34091 |
| AT5G64050 | 10.4082 | 13.882 |
| AT1G04510 | 10.4081 | 28.6156 |
| AT5G44370 | 10.4069 | 9.51488 |
| AT1G13340 | 10.4022 | 4.14741 |
| AT5G43930 | 10.402 | 9.95179 |
| AT5G48620 | 10.4003 | 4.81739 |
| AT4G00670 | 10.4003 | 2.96852 |
| AT5G11530 | 10.3983 | 9.02438 |
| AT3G15790 | 10.3953 | 19.8902 |
| AT3G57560 | 10.3951 | 9.17797 |
| AT1G71070 | 10.395 | 20.1337 |
| AT5G01520 | 10.3938 | 4.57791 |
| AT3G04570 | 10.3917 | 11.7561 |
| AT1G74520 | 10.3895 | 18.8671 |
| AT4G17300 | 10.3893 | 13.8342 |
| AT1G27750 | 10.388 | 8.44491 |
| AT3G55400 | 10.3874 | 23.23 |
| AT5G61810 | 10.3874 | 5.24974 |
| AT2G41280 | 10.3855 | 52.4019 |
| AT4G26540 | 10.3754 | 15.9482 |
| AT1G76040 | 10.375 | 6.05527 |
| AT1G44835 | 10.3743 | 16.059 |
| AT3G45100 | 10.3733 | 12.2882 |
| AT3G02130 | 10.3731 | 10.2974 |
| AT5G60960 | 10.3729 | 30.1731 |
| AT1G64400 | 10.3705 | 0.887508 |
| AT3G22330 | 10.3687 | 14.8832 |
| AT1G23090 | 10.368 | 1.46506 |
| AT5G04270 | 10.3677 | 16.7802 |
| AT1G13000 | 10.3654 | 24.9249 |
| AT3G22425 | 10.3644 | 15.3249 |
| AT5G55600 | 10.3614 | 13.1326 |
| AT5G41730 | 10.3606 | 0.0664949 |
| AT1G22550 | 10.3585 | 3.09013 |
| AT4G22380 | 10.3582 | 20.7142 |
| AT1G05830 | 10.3541 | 9.09263 |
| AT4G39940 | 10.35 | 19.9179 |
| AT4G25433 | 10.3498 | 0.508522 |
| AT2G45530 | 10.3483 | 6.83394 |
| AT2G15760 | 10.3456 | 3.2626 |
| AT5G15170 | 10.3452 | 3.40474 |
| AT5G44180 | 10.3449 | 11.6062 |
| AT3G07770 | 10.3436 | 31.302 |
| AT1G28580 | 10.3405 | 9.97487 |
| AT4G29000 | 10.335 | 6.62718 |
| AT5G22875 | 10.3345 | 13.14 |
| AT1G29880 | 10.3317 | 30.4127 |
| AT1G08350 | 10.3296 | 8.61925 |
| AT2G33580 | 10.3286 | 15.7348 |
| AT4G28030 | 10.3257 | 1.91132 |
| AT3G19895 | 10.3255 | 7.21518 |
| AT2G32200 | 10.3195 | 5.97834 |
| AT1G76230 | 10.3168 | 0.897649 |
| AT3G07640 | 10.3112 | 18.9582 |
| AT4G11350 | 10.3105 | 10.5321 |
| AT2G25530 | 10.3091 | 2.74497 |
| AT1G11800 | 10.307 | 3.50259 |
| AT1G80420 | 10.3008 | 10.1793 |
| AT2G01980 | 10.2969 | 7.20606 |
| AT4G02400 | 10.2899 | 17.0139 |
| AT4G16566 | 10.2895 | 7.92385 |
| AT3G03490 | 10.2892 | 11.0977 |
| AT1G03365 | 10.289 | 9.29123 |
| AT5G05760 | 10.2871 | 6.43894 |
| AT1G21200 | 10.2804 | 9.2588 |
| AT3G50980 | 10.2775 | 6.07256 |
| AT3G50840 | 10.2772 | 6.18482 |
| AT3G47480 | 10.2739 | 10.5128 |
| AT2G46230 | 10.2722 | 44.4095 |
| AT3G47590 | 10.2667 | 15.3014 |
| AT1G48405 | 10.2639 | 7.31473 |
| AT1G32950 | 10.2627 | 0.788284 |
| AT3G25150 | 10.2524 | 22.6336 |
| AT3G62529 | 10.2519 | 0.895338 |
| AT5G08100 | 10.2518 | 20.3359 |
| AT5G03110 | 10.2515 | 9.85476 |
| AT4G17550 | 10.2507 | 2.62519 |
| AT1G56612 | 10.2483 | 7.4728 |
| AT4G23570 | 10.2474 | 10.2008 |
| AT4G11850 | 10.2462 | 11.03 |
| AT3G01310 | 10.2451 | 10.0433 |
| AT5G03760 | 10.2407 | 17.8286 |
| AT2G37280 | 10.2405 | 3.82392 |
| AT1G33270 | 10.2391 | 14.8193 |
| AT3G08630 | 10.2384 | 17.7086 |
| AT3G56410 | 10.2364 | 4.53247 |
| AT2G05755 | 10.2358 | 5.19398 |
| AT1G12050 | 10.2345 | 11.8812 |
| AT5G64470 | 10.2323 | 9.44416 |
| AT4G14130 | 10.2277 | 9.07254 |
| AT5G65687 | 10.2272 | 8.89671 |
| AT3G24490 | 10.222 | 21.3169 |
| AT5G35732 | 10.2188 | 3.23787 |
| AT4G02170 | 10.2176 | 2.09727 |
| AT1G08710 | 10.2159 | 12.2095 |
| AT3G26618 | 10.2112 | 15.0323 |
| AT1G05820 | 10.2105 | 6.06925 |
| AT5G15080 | 10.2092 | 13.8829 |
| AT5G26210 | 10.2014 | 25.3787 |
| AT2G47630 | 10.1963 | 4.03781 |
| AT1G20470 | 10.1938 | 4.21748 |
| AT3G61850 | 10.1928 | 24.6525 |
| AT3G59640 | 10.1917 | 12.6802 |
| AT2G32000 | 10.1866 | 11.8743 |
| AT3G24210 | 10.1845 | 11.0933 |
| AT3G17450 | 10.182 | 8.71348 |
| AT2G32710 | 10.179 | 23.9062 |
| AT4G11680 | 10.1763 | 5.11809 |
| AT1G70450 | 10.1736 | 0.132146 |
| AT2G20770 | 10.167 | 6.8696 |
| AT1G16250 | 10.1667 | 10.0376 |
| AT3G15180 | 10.1649 | 15.9794 |
| AT1G59710 | 10.1648 | 10.0752 |
| AT3G48660 | 10.1641 | 39.3126 |
| AT3G50130 | 10.1628 | 0.237054 |
| AT4G03000 | 10.1611 | 7.02814 |
| AT4G14920 | 10.1598 | 11.4668 |
| AT3G51470 | 10.1581 | 1.88337 |
| AT2G33740 | 10.1576 | 18.6263 |
| AT4G27430 | 10.1531 | 17.1956 |
| AT2G13975.2 | 10.1529 | 19.1257 |
| AT3G62140 | 10.1528 | 12.1333 |
| AT1G77240 | 10.1506 | 4.72782 |
| AT4G30710 | 10.146 | 5.46123 |
| AT4G03230 | 10.1406 | 8.38956 |
| AT1G13220 | 10.1347 | 18.8367 |
| AT1G07570 | 10.1322 | 5.50978 |
| AT5G11630 | 10.1312 | 14.5874 |
| AT5G01330 | 10.1311 | 6.37725 |
| AT1G70470 | 10.1311 | 0.258996 |
| AT2G17265 | 10.1303 | 12.3975 |
| AT1G31810 | 10.1208 | 8.21255 |
| AT1G22400 | 10.1149 | 50.6732 |
| AT2G30330 | 10.1142 | 10.2366 |
| AT4G04470 | 10.1124 | 24.3691 |
| AT2G40590 | 10.1072 | 34.2001 |
| AT2G37370 | 10.1016 | 0.0492906 |
| AT2G03640 | 10.1015 | 21.9306 |
| AT1G28680 | 10.1015 | 6.59368 |
| AT2G24600 | 10.09 | 27.9264 |
| AT5G41330 | 10.0864 | 6.17643 |
| AT5G17710 | 10.0779 | 25.3262 |
| AT4G21740 | 10.0778 | 5.41017 |
| AT1G02720 | 10.0763 | 16.6832 |
| AT5G39550 | 10.0739 | 7.54171 |
| AT4G31310 | 10.0722 | 16.2843 |
| AT3G15920 | 10.071 | 10.4972 |
| AT4G38980 | 10.0706 | 12.2427 |
| AT3G24010 | 10.0698 | 8.70064 |
| AT4G03080 | 10.0683 | 11.2062 |
| AT3G15620 | 10.0675 | 3.61663 |
| AT5G11840 | 10.0667 | 11.9837 |
| AT3G29130 | 10.0638 | 13.4211 |
| AT1G43800 | 10.0587 | 4.738 |
| AT4G23810 | 10.0572 | 3.86118 |
| AT3G28670 | 10.0559 | 11.3559 |
| AT3G54460 | 10.0543 | 6.76582 |
| AT3G03060 | 10.0523 | 10.0737 |
| AT5G17530 | 10.0507 | 18.4991 |
| AT2G46915 | 10.0488 | 13.4782 |
| AT4G00231 | 10.0463 | 10.237 |
| AT5G01200 | 10.0362 | 0.566426 |
| AT3G49880 | 10.0355 | 8.32939 |
| AT1G21520 | 10.0353 | 14.354 |
| AT3G05270 | 10.0341 | 15.8151 |
| AT2G15430 | 10.0327 | 16.2985 |
| AT1G76270 | 10.0242 | 11.5714 |
| AT4G04695 | 10.0239 | 1.89791 |
| AT5G35410 | 10.021 | 5.24722 |
| AT3G22430 | 10.0178 | 6.78525 |
| AT5G25050 | 10.0151 | 8.2515 |
| AT2G01710 | 10.0136 | 9.67207 |
| AT2G41770 | 10.0115 | 9.876 |
| AT2G20585 | 10.0109 | 21.2755 |
| AT1G27930 | 10.0094 | 4.67102 |
| AT1G79020 | 10.0087 | 10.5257 |
| AT5G07610 | 10.0066 | 11.3599 |
| AT4G26140 | 10.0043 | 20.0722 |
| AT1G24330 | 10.0019 | 3.71994 |
| AT5G13120 | 9.99649 | 27.8233 |
| AT5G24340 | 9.99605 | 6.75241 |
| AT1G32520 | 9.99501 | 5.90787 |
| AT2G40780 | 9.9864 | 12.2553 |
| AT4G19420 | 9.98606 | 5.35268 |
| AT3G20160 | 9.98425 | 41.3285 |
| AT3G45930 | 9.9842 | 18.1064 |
| AT5G04235.1 | 9.9816 | 4.12157 |
| AT1G73990 | 9.9808 | 11.7876 |
| AT1G11540 | 9.97971 | 3.56174 |
| AT3G04590 | 9.97211 | 8.32942 |
| AT5G43180 | 9.97131 | 1.90042 |
| AT2G31010 | 9.97085 | 12.591 |
| AT5G14440 | 9.96977 | 20.7113 |
| AT1G27385 | 9.96807 | 11.9253 |
| AT2G33320 | 9.96399 | 3.38615 |
| AT1G34320 | 9.96347 | 17.9746 |
| AT5G47455 | 9.95938 | 14.1708 |
| AT1G55150 | 9.95225 | 13.603 |
| AT4G18910 | 9.94938 | 2.19134 |
| AT4G33030 | 9.94308 | 15.6475 |
| AT1G78590 | 9.94074 | 8.9464 |
| AT2G37600 | 9.93532 | 36.6261 |
| AT2G01270 | 9.93223 | 18.4584 |
| AT4G21180 | 9.93128 | 16.0524 |
| AT3G19670 | 9.92807 | 11.8513 |
| AT2G25080 | 9.92566 | 22.2162 |
| AT2G13665 | 9.92241 | 9.17317 |
| AT3G01710 | 9.91863 | 2.29465 |
| AT5G60600 | 9.91766 | 13.9086 |
| AT1G07950 | 9.91735 | 13.2302 |
| AT2G30615 | 9.91532 | 3.3009 |
| AT1G18610 | 9.91503 | 9.37945 |
| AT5G65750 | 9.91097 | 20.8111 |
| AT2G47640 | 9.90824 | 46.0255 |
| AT3G06035 | 9.90805 | 35.7227 |
| AT3G47740 | 9.9067 | 0.014007 |
| AT3G20890 | 9.88678 | 10.3106 |
| AT4G24480 | 9.88548 | 10.786 |
| AT1G28440 | 9.88307 | 10.7722 |
| AT5G64280 | 9.87986 | 6.09366 |
| AT4G39240 | 9.87984 | 6.89164 |
| AT4G33630 | 9.87869 | 11.457 |
| AT1G61750 | 9.87134 | 0.680327 |
| AT3G62590 | 9.86562 | 4.16819 |
| AT3G09970 | 9.86459 | 8.27353 |
| AT1G31170 | 9.86393 | 4.96183 |
| AT1G11940 | 9.86139 | 6.20882 |
| AT3G18760 | 9.8601 | 24.2917 |
| AT1G10280 | 9.85991 | 5.04962 |
| AT5G42140 | 9.85965 | 5.51337 |
| AT4G13620 | 9.85908 | 1.91991 |
| AT5G58090 | 9.85905 | 16.7088 |
| AT2G38950 | 9.85654 | 12.193 |
| AT3G54560 | 9.85564 | 31.5296 |
| AT5G04520 | 9.84957 | 7.74377 |
| AT4G25700 | 9.83877 | 6.01221 |
| AT1G51660 | 9.83668 | 10.2547 |
| AT4G13420 | 9.83467 | 1.68897 |
| AT5G57100 | 9.83396 | 14.6316 |
| AT4G20140 | 9.83353 | 10.1726 |
| AT3G49640 | 9.83042 | 10.7374 |
| AT1G02340 | 9.82746 | 0.889122 |
| AT3G18990 | 9.82633 | 21.0014 |
| AT1G51450 | 9.82478 | 7.75772 |
| AT5G53830 | 9.82475 | 0.442394 |
| AT1G23850 | 9.82346 | 15.1718 |
| AT3G44720 | 9.82197 | 5.4273 |
| AT5G16520 | 9.82193 | 9.91388 |
| AT3G51850 | 9.8182 | 22.0439 |
| AT5G27600 | 9.81699 | 11.8071 |
| AT1G19570 | 9.81458 | 4.99778 |
| AT4G18880 | 9.81433 | 21.7092 |
| AT2G28671 | 9.81384 | 10.7243 |
| AT5G19390 | 9.80967 | 20.3357 |
| AT3G29390 | 9.80555 | 5.71177 |
| AT1G22270 | 9.80137 | 21.6634 |
| AT3G20170 | 9.79889 | 11.8377 |
| AT1G68910 | 9.79618 | 9.33695 |
| AT1G22920 | 9.78766 | 16.0862 |
| AT3G02240 | 9.78551 | 3.80123 |
| AT3G63400 | 9.78206 | 19.6467 |
| AT4G12460 | 9.77522 | 10.1407 |
| AT5G04970 | 9.77448 | 13.0894 |
| AT3G55020 | 9.77354 | 34.9414 |
| AT1G07070 | 9.77295 | 52.9787 |
| AT4G02330 | 9.76951 | 9.39851 |
| AT3G56820 | 9.76499 | 9.27246 |
| AT4G34412 | 9.76466 | 18.5362 |
| AT5G17050 | 9.75961 | 11.1858 |
| AT4G21680 | 9.73911 | 7.78702 |
| AT1G13780 | 9.73083 | 10.2601 |
| AT4G31160 | 9.71896 | 14.1334 |
| AT3G25500 | 9.71655 | 17.3669 |
| AT5G66870 | 9.7157 | 19.7733 |
| AT3G54120 | 9.71522 | 6.08506 |
| AT2G34410 | 9.71506 | 11.5697 |
| AT5G08335 | 9.71151 | 7.31616 |
| AT1G12390 | 9.71007 | 9.75703 |
| AT3G03870 | 9.70798 | 7.99983 |
| AT2G35100 | 9.70646 | 6.01426 |
| AT5G56180 | 9.70585 | 6.17151 |
| AT3G59700 | 9.70302 | 7.17581 |
| AT3G17609 | 9.69465 | 5.15646 |
| AT2G41890 | 9.67838 | 10.1583 |
| AT3G57150 | 9.67676 | 56.082 |
| AT4G07410 | 9.67675 | 25.847 |
| AT2G45695 | 9.67534 | 22.0484 |
| AT5G59410 | 9.67282 | 10.841 |
| AT1G76080 | 9.66811 | 1.48445 |
| AT2G25940 | 9.66698 | 1.71502 |
| AT5G03020 | 9.66453 | 1.08704 |
| AT1G10870 | 9.66379 | 14.7844 |
| AT4G28380 | 9.65946 | 5.83306 |
| AT5G36270 | 9.65698 | 0.029944 |
| AT4G35070 | 9.65326 | 19.4836 |
| AT1G45050 | 9.65176 | 14.5588 |
| AT1G19370 | 9.64786 | 8.41035 |
| AT5G43070 | 9.64738 | 5.10517 |
| AT1G75440 | 9.64677 | 6.60558 |
| AT5G08550 | 9.64452 | 9.44655 |
| AT3G06820 | 9.64345 | 8.55656 |
| AT2G42910 | 9.63691 | 27.9103 |
| AT1G07830 | 9.63372 | 22.3322 |
| AT2G19050 | 9.63236 | 0.278971 |
| AT3G61240 | 9.63024 | 30.4491 |
| AT2G33110 | 9.63022 | 4.86668 |
| AT1G54360 | 9.61927 | 13.7228 |
| AT5G45290 | 9.61691 | 7.87934 |
| AT5G06810 | 9.61365 | 2.85003 |
| AT1G19610 | 9.61063 | 3.71992 |
| AT4G31570 | 9.6024 | 10.1206 |
| AT5G04320 | 9.60134 | 13.4704 |
| AT5G26720 | 9.60011 | 4.23644 |
| AT1G73680 | 9.59924 | 0.835131 |
| AT3G56840 | 9.5991 | 4.26905 |
| AT3G01760 | 9.59626 | 0.465696 |
| AT3G16210 | 9.59477 | 0.569167 |
| AT2G20650 | 9.59256 | 4.9402 |
| AT1G10155 | 9.59256 | 2.70245 |
| AT5G59050 | 9.59187 | 12.103 |
| AT1G03930 | 9.58994 | 7.06662 |
| AT3G10610 | 9.58928 | 44.7769 |
| AT4G31490 | 9.58556 | 12.2548 |
| AT2G18860 | 9.58341 | 8.28934 |
| AT2G20270 | 9.56952 | 11.1027 |
| AT3G13230 | 9.56661 | 29.6628 |
| AT4G28510 | 9.56059 | 25.1294 |
| AT2G20480 | 9.55797 | 7.2938 |
| AT1G58340 | 9.55703 | 7.12345 |
| AT4G04614 | 9.55596 | 11.8383 |
| AT1G17140 | 9.55051 | 6.55308 |
| AT3G17340 | 9.55003 | 6.47965 |
| AT2G35210 | 9.54962 | 4.20775 |
| AT5G42810 | 9.54544 | 11.9765 |
| AT3G63050 | 9.53778 | 0.737608 |
| AT3G51530 | 9.53704 | 6.99447 |
| AT5G15570 | 9.53615 | 10.7326 |
| AT4G15480 | 9.53167 | 1.96802 |
| AT1G01510 | 9.53006 | 18.3366 |
| AT2G37585 | 9.5224 | 11.1237 |
| AT2G47440 | 9.51999 | 10.3174 |
| AT5G45330 | 9.5175 | 14.1343 |
| AT3G22570 | 9.51663 | 0.252635 |
| AT5G58680 | 9.51357 | 5.96315 |
| AT3G57360 | 9.5124 | 4.6224 |
| AT5G61580 | 9.51147 | 10.6446 |
| AT5G01510 | 9.5096 | 6.98836 |
| AT4G23895 | 9.50119 | 14.2169 |
| AT3G28920 | 9.49959 | 1.83695 |
| AT4G18800 | 9.49861 | 4.5855 |
| AT3G24110 | 9.49646 | 8.17365 |
| AT1G71330 | 9.4964 | 7.37618 |
| AT2G46320 | 9.49605 | 6.69767 |
| AT4G18630 | 9.49518 | 1.4296 |
| AT3G55170 | 9.4949 | 33.583 |
| AT5G55990 | 9.49484 | 11.3678 |
| AT2G13970 | 9.49474 | 12.2546 |
| AT2G45320 | 9.49434 | 7.56196 |
| AT5G43380 | 9.4936 | 19.3488 |
| AT3G17160 | 9.49223 | 43.7647 |
| AT4G36420 | 9.49062 | 28.3357 |
| AT3G27884 | 9.49056 | 0.973835 |
| AT1G05430 | 9.48911 | 16.2056 |
| AT1G24120 | 9.48857 | 17.5878 |
| AT1G66510 | 9.48834 | 14.405 |
| AT3G05060 | 9.48303 | 59.3268 |
| AT2G36270 | 9.48011 | 15.3879 |
| AT2G45150 | 9.4793 | 5.23392 |
| AT2G03230 | 9.47221 | 6.31292 |
| AT1G78270 | 9.47015 | 7.44177 |
| AT1G74050 | 9.46952 | 43.1989 |
| AT5G27650 | 9.46946 | 7.82868 |
| AT5G64650 | 9.46646 | 12.3253 |
| AT1G61030 | 9.46629 | 12.0869 |
| AT5G67030 | 9.45346 | 10.659 |
| AT5G64290 | 9.45182 | 17.2472 |
| AT5G56940 | 9.45055 | 24.5176 |
| AT1G36990 | 9.4505 | 8.88351 |
| AT3G11840 | 9.44953 | 3.64883 |
| AT1G34150 | 9.44845 | 8.21732 |
| AT3G23610 | 9.44708 | 3.67133 |
| AT5G53930 | 9.44688 | 14.4218 |
| AT3G25790 | 9.44608 | 0.793658 |
| AT1G54890 | 9.44591 | 27.1481 |
| AT1G04010 | 9.44127 | 7.64122 |
| AT4G31000 | 9.43857 | 19.141 |
| AT3G25470 | 9.43475 | 14.3664 |
| AT2G28800 | 9.43461 | 13.934 |
| AT3G04600 | 9.43377 | 24.5365 |
| AT1G55830 | 9.43234 | 9.49884 |
| AT5G17810 | 9.42876 | 1.25121 |
| AT1G28090 | 9.42536 | 8.43898 |
| AT4G02610 | 9.42487 | 14.77 |
| AT3G23325 | 9.42449 | 27.0245 |
| AT3G53170 | 9.42272 | 7.05552 |
| AT4G03440 | 9.42261 | 1.44462 |
| AT1G75350 | 9.42196 | 17.6638 |
| AT1G31480 | 9.4219 | 6.60923 |
| AT3G01202 | 9.42139 | 9.60332 |
| AT2G29990 | 9.41787 | 14.8749 |
| AT4G11920 | 9.41614 | 5.17169 |
| AT2G40010 | 9.40867 | 24.6959 |
| AT5G59180 | 9.40458 | 18.0105 |
| AT5G20540 | 9.40444 | 2.69888 |
| AT2G18710 | 9.40262 | 11.9049 |
| AT5G63280 | 9.40107 | 13.3629 |
| AT3G59870 | 9.40103 | 6.88202 |
| AT2G24320 | 9.39367 | 1.13674 |
| AT5G21070 | 9.3766 | 9.23901 |
| AT3G23150 | 9.37639 | 9.71685 |
| AT1G09150 | 9.37107 | 23.567 |
| AT4G14365 | 9.36975 | 3.57403 |
| AT1G09870 | 9.36895 | 19.5087 |
| AT5G57700 | 9.36866 | 15.1557 |
| AT4G18372 | 9.36672 | 12.219 |
| AT2G46580 | 9.36563 | 7.97162 |
| AT1G66080 | 9.36421 | 20.6958 |
| AT1G69670 | 9.36352 | 8.59151 |
| AT1G59850 | 9.36163 | 0.500439 |
| AT5G05230 | 9.36101 | 8.13308 |
| AT5G38380 | 9.35955 | 14.14 |
| AT1G63110 | 9.35623 | 14.0206 |
| AT2G36130 | 9.35365 | 28.807 |
| AT5G67160 | 9.35251 | 2.52002 |
| AT3G11210 | 9.34809 | 12.739 |
| AT5G60310 | 9.34708 | 8.35013 |
| AT5G16220 | 9.34382 | 8.62887 |
| AT1G80200 | 9.34368 | 4.00884 |
| AT5G61140 | 9.34163 | 10.5452 |
| AT5G65240 | 9.33837 | 5.8033 |
| AT5G43630 | 9.3375 | 8.09292 |
| AT3G63420 | 9.33277 | 14.4485 |
| AT2G20610 | 9.32746 | 17.1197 |
| AT3G29810 | 9.32744 | 8.51037 |
| AT3G23940 | 9.32529 | 44.7999 |
| AT5G63480 | 9.32171 | 7.68107 |
| AT3G12830 | 9.31857 | 3.62177 |
| AT2G32170 | 9.3169 | 8.35595 |
| AT3G02630 | 9.31177 | 22.4357 |
| AT2G24400 | 9.31114 | 6.66087 |
| AT1G61470 | 9.30761 | 7.95499 |
| AT3G10572 | 9.30584 | 12.0976 |
| AT4G16400 | 9.30397 | 1.50363 |
| AT5G63860 | 9.29926 | 16.8991 |
| AT5G62200 | 9.29731 | 15.7806 |
| AT5G39890 | 9.29683 | 6.3101 |
| AT2G01640 | 9.29446 | 19.0146 |
| AT1G01340 | 9.29395 | 6.2676 |
| AT3G46690 | 9.29009 | 1.89046 |
| AT1G04680 | 9.28823 | 20.0141 |
| AT5G57230 | 9.28741 | 10.2802 |
| AT2G24830 | 9.28456 | 10.4985 |
| AT2G21640 | 9.28362 | 7.47055 |
| AT2G16800 | 9.28315 | 10.881 |
| AT4G37450 | 9.28119 | 7.2044 |
| AT3G08670 | 9.27907 | 16.3517 |
| AT3G60110 | 9.27338 | 5.99867 |
| AT3G49810 | 9.2733 | 16.2066 |
| AT1G31175 | 9.27318 | 6.86587 |
| AT2G01460 | 9.2706 | 7.16144 |
| AT3G13380 | 9.26606 | 1.83324 |
| AT5G11150 | 9.26575 | 14.8176 |
| AT3G27325 | 9.26528 | 12.9114 |
| AT2G01735 | 9.25586 | 12.6872 |
| AT2G14850 | 9.25572 | 9.30041 |
| AT1G16430 | 9.25429 | 14.1776 |
| AT1G63600 | 9.25114 | 0.0475462 |
| AT2G45990 | 9.25091 | 17.3052 |
| AT4G17740 | 9.24535 | 5.78654 |
| AT3G09960 | 9.24363 | 2.20792 |
| AT1G56423 | 9.24202 | 16.4963 |
| AT5G02910 | 9.2412 | 9.39982 |
| AT5G03545 | 9.24082 | 10.3625 |
| AT4G14360 | 9.23933 | 29.011 |
| AT2G15790 | 9.23803 | 17.941 |
| AT4G32730 | 9.23602 | 14.2195 |
| AT3G46590 | 9.22922 | 5.94886 |
| AT2G46560 | 9.22788 | 8.39721 |
| AT2G31890 | 9.2235 | 10.2222 |
| AT1G06240 | 9.22323 | 8.24696 |
| AT1G80530 | 9.21897 | 23.4189 |
| AT5G59380 | 9.21893 | 8.64546 |
| AT5G41350 | 9.21781 | 8.83493 |
| AT5G23890 | 9.21448 | 12.8367 |
| AT5G40660 | 9.21116 | 17.3816 |
| AT3G10700 | 9.20817 | 8.38341 |
| AT1G11060 | 9.20545 | 10.9276 |
| AT1G32120 | 9.20298 | 5.6743 |
| AT3G47700 | 9.20214 | 7.10938 |
| AT1G72840 | 9.19783 | 6.21405 |
| AT5G49360 | 9.19674 | 3.22218 |
| AT5G25150 | 9.19452 | 12.6207 |
| AT5G66090 | 9.19148 | 10.0041 |
| AT2G24300 | 9.19131 | 17.7721 |
| AT3G26430 | 9.18518 | 9.0325 |
| AT1G33415 | 9.18487 | 5.85762 |
| AT1G28420 | 9.18396 | 11.3991 |
| AT3G15820 | 9.18297 | 7.13677 |
| AT4G17460 | 9.17486 | 14.7318 |
| AT5G43030 | 9.17098 | 3.60298 |
| AT5G19060 | 9.16852 | 5.32832 |
| AT4G15080 | 9.16519 | 17.2866 |
| AT1G17760 | 9.15896 | 15.256 |
| AT4G39580 | 9.15706 | 6.79851 |
| AT3G50630 | 9.15539 | 10.1298 |
| AT2G29210 | 9.15464 | 16.6649 |
| AT3G17611 | 9.14471 | 6.75447 |
| AT4G24490 | 9.14091 | 10.3138 |
| AT2G40950 | 9.14043 | 15.7744 |
| AT1G16930 | 9.13863 | 4.97408 |
| AT2G27010 | 9.13833 | 8.56095 |
| AT3G15460 | 9.13405 | 19.7654 |
| AT3G22942 | 9.13288 | 5.25291 |
| AT3G57390 | 9.13231 | 10.7834 |
| AT3G62880 | 9.1297 | 8.56347 |
| AT3G27280 | 9.12905 | 22.1336 |
| AT4G37470 | 9.12597 | 2.04398 |
| AT4G25610 | 9.12247 | 7.91033 |
| AT1G31850 | 9.1168 | 33.6086 |
| AT4G11830 | 9.11376 | 5.08305 |
| AT1G61950 | 9.11182 | 0.00939534 |
| AT4G17220 | 9.10986 | 11.4494 |
| AT4G25310 | 9.10739 | 125.286 |
| AT2G30200 | 9.10679 | 32.9064 |
| AT5G47490 | 9.1058 | 6.43921 |
| AT4G39140 | 9.09788 | 12.9361 |
| AT3G22270 | 9.09186 | 16.9136 |
| AT4G12760 | 9.09107 | 8.15725 |
| AT4G25340 | 9.09031 | 46.45 |
| AT4G34400 | 9.0899 | 5.55922 |
| AT1G33140 | 9.08839 | 27.2992 |
| AT5G22300 | 9.08484 | 3.17038 |
| AT5G21920 | 9.08429 | 7.08038 |
| AT4G11090 | 9.08134 | 7.11321 |
| AT1G13520 | 9.07737 | 3.47035 |
| AT1G15415 | 9.07667 | 5.96762 |
| AT5G57440 | 9.07541 | 17.566 |
| AT3G42950 | 9.0735 | 10.0421 |
| AT3G02860 | 9.07318 | 15.6997 |
| AT5G48640 | 9.07124 | 11.3272 |
| AT4G09680 | 9.06973 | 9.34382 |
| AT1G16710 | 9.06956 | 9.68207 |
| AT5G06100 | 9.06642 | 8.93914 |
| AT4G16600 | 9.0648 | 0.345115 |
| AT5G59230 | 9.06408 | 4.59334 |
| AT2G33100 | 9.06158 | 3.85812 |
| AT3G09230 | 9.05132 | 4.55187 |
| AT1G16900 | 9.05069 | 17.7624 |
| AT5G59430 | 9.04991 | 16.4013 |
| AT2G04030 | 9.04988 | 31.9473 |
| AT4G39570 | 9.04526 | 10.4438 |
| AT1G67750 | 9.0389 | 8.27311 |
| AT5G53800 | 9.03678 | 22.2704 |
| AT5G64890 | 9.03668 | 2.17201 |
| AT5G39990 | 9.0316 | 11.3716 |
| AT5G41315 | 9.02493 | 1.34671 |
| AT3G53810 | 9.02445 | 5.61724 |
| AT1G78790 | 9.0241 | 9.90506 |
| AT4G00700 | 9.02293 | 1.34296 |
| AT2G40810 | 9.01829 | 7.37719 |
| AT5G40270 | 9.01667 | 2.23347 |
| AT5G52250 | 9.01629 | 2.53009 |
| AT1G65180 | 9.01323 | 2.29993 |
| AT1G26620 | 9.00462 | 11.5824 |
| AT3G46090 | 9.00391 | 1.86306 |
| AT4G15415 | 9.00298 | 10.8123 |
| AT4G39870 | 9.00279 | 6.29933 |
| AT4G36390 | 8.99454 | 10.4081 |
| AT5G49770 | 8.99355 | 0.0417508 |
| AT4G24210 | 8.98788 | 8.50022 |
| AT3G03140 | 8.9851 | 9.66198 |
| AT3G20550 | 8.97531 | 14.6306 |
| AT3G13740 | 8.97515 | 9.86109 |
| AT5G67470 | 8.97504 | 5.92655 |
| AT5G49945 | 8.97357 | 15.3449 |
| AT2G39290 | 8.97253 | 9.56061 |
| AT3G59820 | 8.96535 | 24.5297 |
| AT2G32960 | 8.96415 | 3.34035 |
| AT2G27840 | 8.96018 | 43.0833 |
| AT4G39860 | 8.95933 | 23.378 |
| AT2G04680 | 8.95639 | 0.551561 |
| AT4G26990 | 8.95385 | 4.28721 |
| AT5G24040 | 8.94661 | 1.32902 |
| AT1G11790 | 8.94411 | 15.5257 |
| AT4G18260 | 8.94384 | 7.74354 |
| AT5G36180 | 8.94245 | 4.91788 |
| AT1G31814 | 8.94042 | 5.96275 |
| AT5G27460 | 8.94041 | 8.5571 |
| AT1G48210 | 8.93277 | 4.46372 |
| AT2G14680 | 8.93231 | 6.15812 |
| AT3G51390 | 8.93187 | 10.3091 |
| AT4G39420 | 8.91415 | 5.61616 |
| AT4G16480 | 8.91291 | 3.60611 |
| AT4G23450 | 8.90805 | 7.35709 |
| AT2G25170 | 8.90759 | 21.473 |
| AT2G41945 | 8.90385 | 13.0667 |
| AT3G49850 | 8.90277 | 7.59604 |
| AT2G04540 | 8.90083 | 8.44304 |
| AT1G61590 | 8.8991 | 12.0989 |
| AT2G45000 | 8.89572 | 14.1334 |
| AT1G27720 | 8.89567 | 3.71194 |
| AT1G24280 | 8.8955 | 8.97 |
| AT2G22630 | 8.89465 | 6.29519 |
| AT1G21400 | 8.89396 | 15.6617 |
| AT4G26130 | 8.89271 | 3.16552 |
| AT5G04910 | 8.88663 | 14.2628 |
| AT1G01020 | 8.88583 | 11.2414 |
| AT3G25660 | 8.88285 | 9.67078 |
| AT2G43140 | 8.8826 | 11.7205 |
| AT5G62220 | 8.8814 | 8.26085 |
| AT1G67325 | 8.88034 | 10.7084 |
| AT2G01910 | 8.87988 | 12.0273 |
| AT5G64480 | 8.87605 | 4.85899 |
| AT5G51451 | 8.87595 | 3.10392 |
| AT4G19060 | 8.87564 | 5.30934 |
| AT1G18100 | 8.87312 | 10.4166 |
| AT4G29530 | 8.86897 | 8.71597 |
| AT2G40860 | 8.86577 | 4.81791 |
| AT1G61640 | 8.86528 | 9.38158 |
| AT3G06010 | 8.86527 | 14.3208 |
| AT2G35170 | 8.86524 | 7.11361 |
| AT3G59130 | 8.86294 | 1.93403 |
| AT5G06490 | 8.86261 | 0.382623 |
| AT4G37820 | 8.85017 | 16.0111 |
| AT5G63650 | 8.84481 | 95.3899 |
| AT2G35840 | 8.8402 | 27.249 |
| AT3G63300 | 8.83785 | 4.92113 |
| AT5G48900 | 8.83755 | 68.2504 |
| AT3G21690 | 8.83283 | 5.51873 |
| AT2G40650 | 8.83177 | 21.3534 |
| AT3G51780 | 8.82505 | 7.3279 |
| AT1G76170 | 8.8185 | 4.95382 |
| AT1G31470 | 8.81599 | 3.89412 |
| AT5G47820 | 8.80633 | 18.4405 |
| AT5G48630 | 8.80503 | 7.36312 |
| AT1G05220 | 8.80349 | 4.61758 |
| AT5G15860 | 8.79618 | 6.63847 |
| AT4G32460 | 8.79605 | 10.9685 |
| AT1G06770 | 8.79459 | 9.80457 |
| AT1G72890 | 8.79457 | 0.64264 |
| AT2G39480 | 8.78576 | 7.38896 |
| AT5G16505.2 | 8.78493 | 5.89971 |
| AT5G19290 | 8.78114 | 10.6228 |
| AT5G47870 | 8.77914 | 9.98473 |
| AT1G48050 | 8.77586 | 10.736 |
| AT3G13120 | 8.77482 | 11.3388 |
| AT4G38520 | 8.77348 | 15.0175 |
| AT1G27595 | 8.77287 | 14.3964 |
| AT1G58060 | 8.76706 | 8.5339 |
| AT1G18335 | 8.76502 | 5.66914 |
| AT5G49580 | 8.76466 | 9.94242 |
| AT1G68290 | 8.75735 | 11.2387 |
| AT3G11880 | 8.75622 | 14.2213 |
| AT1G28130 | 8.75409 | 10.0475 |
| AT4G20980 | 8.74119 | 28.3496 |
| AT5G42100 | 8.72502 | 12.7679 |
| AT5G19260 | 8.72232 | 4.16332 |
| AT1G27120 | 8.719 | 12.5795 |
| AT5G51690 | 8.71851 | 6.11822 |
| AT2G25920 | 8.71765 | 7.1694 |
| AT2G44600 | 8.71181 | 2.40821 |
| AT1G10385 | 8.71123 | 0.133365 |
| AT2G01890 | 8.7089 | 9.2462 |
| AT3G51120 | 8.70839 | 5.16842 |
| AT4G01560 | 8.706 | 32.1453 |
| AT2G05990 | 8.70482 | 58.5864 |
| AT1G48370 | 8.70465 | 12.0871 |
| AT5G22370 | 8.70458 | 18.9472 |
| AT3G53100 | 8.7029 | 0.485619 |
| AT3G20960 | 8.6995 | 5.28015 |
| AT1G13480 | 8.69447 | 17.7962 |
| AT1G54340 | 8.69327 | 11.9553 |
| AT1G10480 | 8.69083 | 17.1231 |
| AT1G06110 | 8.69054 | 8.31065 |
| AT1G73805 | 8.69024 | 2.25135 |
| AT5G16560 | 8.68939 | 10.3742 |
| AT3G52890 | 8.68851 | 20.176 |
| AT2G40930 | 8.68806 | 16.5281 |
| AT2G37220 | 8.6867 | 21.6958 |
| AT3G13080 | 8.68618 | 19.6325 |
| AT1G30757 | 8.68346 | 2.55832 |
| AT4G31980 | 8.67979 | 3.2177 |
| AT1G17870 | 8.67952 | 7.23183 |
| AT3G58865 | 8.67852 | 7.28321 |
| AT5G02050 | 8.67697 | 45.218 |
| AT3G15690 | 8.67276 | 16.6298 |
| AT5G48440 | 8.66989 | 11.7899 |
| AT3G23300 | 8.66649 | 26.1796 |
| AT1G76630 | 8.66004 | 10.7941 |
| AT5G47680 | 8.65879 | 25.2352 |
| AT1G19840 | 8.65766 | 3.91148 |
| AT1G27850 | 8.65257 | 11.9649 |
| AT5G06130 | 8.64952 | 9.97271 |
| AT2G03390 | 8.64483 | 11.051 |
| AT1G72300 | 8.64289 | 1.7199 |
| AT5G59030 | 8.63904 | 33.1047 |
| AT4G32840 | 8.63374 | 8.59013 |
| AT2G27170 | 8.62961 | 18.693 |
| AT3G11550 | 8.62783 | 8.56492 |
| AT2G26780 | 8.62672 | 12.4862 |
| AT3G03305 | 8.62446 | 10.1984 |
| AT3G21640 | 8.62432 | 18.5709 |
| AT1G05160 | 8.62252 | 7.62066 |
| AT5G58450 | 8.61773 | 12.3297 |
| AT5G58340 | 8.61662 | 7.10586 |
| AT2G23370 | 8.61201 | 11.5201 |
| AT2G30460 | 8.61167 | 11.5797 |
| AT2G41140 | 8.61148 | 11.5279 |
| AT5G44670 | 8.60921 | 3.96075 |
| AT3G04460 | 8.60884 | 10.3862 |
| AT2G11910 | 8.60622 | 47.7739 |
| AT3G29400 | 8.60002 | 7.01264 |
| AT5G08230 | 8.59952 | 8.44849 |
| AT3G27540 | 8.59467 | 2.89814 |
| AT4G00520 | 8.59314 | 9.84841 |
| AT4G23890 | 8.59271 | 5.53111 |
| AT2G38650 | 8.59218 | 26.3704 |
| AT1G32100 | 8.59197 | 3.87416 |
| AT4G26720 | 8.58835 | 11.4581 |
| AT5G41710.1 | 8.58781 | 1.65892 |
| AT4G31100 | 8.58235 | 19.3153 |
| AT1G08130 | 8.57985 | 14.0713 |
| AT4G06634 | 8.57881 | 9.80523 |
| AT1G03790 | 8.57628 | 21.2066 |
| AT2G26420 | 8.57448 | 0.625113 |
| AT2G46950 | 8.57363 | 10.7988 |
| AT4G10450 | 8.56964 | 79.7185 |
| AT5G65180 | 8.56397 | 12.1358 |
| AT3G17465 | 8.557 | 28.8782 |
| AT3G03420 | 8.55188 | 17.5752 |
| AT4G18440 | 8.53801 | 32.0003 |
| AT1G11720 | 8.5355 | 13.0964 |
| AT5G20270 | 8.53128 | 7.29757 |
| AT4G36860 | 8.52882 | 10.4365 |
| AT4G23190 | 8.52483 | 5.34277 |
| AT3G02320 | 8.51719 | 11.5252 |
| AT1G07300 | 8.51421 | 5.69911 |
| AT5G38600 | 8.51378 | 15.7428 |
| AT2G43850 | 8.51246 | 2.90163 |
| AT1G62880 | 8.51104 | 10.4316 |
| AT5G16890 | 8.50861 | 9.68577 |
| AT1G26460 | 8.50254 | 19.8468 |
| AT3G18790 | 8.50243 | 20.0739 |
| AT3G28720 | 8.5023 | 15.4885 |
| AT5G55140 | 8.49616 | 22.2375 |
| AT3G07580 | 8.4959 | 9.2179 |
| AT4G18460 | 8.49167 | 2.73443 |
| AT1G70100 | 8.48743 | 6.01794 |
| AT5G48370 | 8.48404 | 9.50817 |
| AT2G03980 | 8.484 | 1.60043 |
| AT1G12270 | 8.48363 | 30.4323 |
| AT1G73350 | 8.48312 | 15.1434 |
| AT2G40360 | 8.47455 | 43.8702 |
| AT3G50780 | 8.47343 | 7.46158 |
| AT5G16600 | 8.47145 | 2.84493 |
| AT2G42280 | 8.46965 | 16.3617 |
| AT5G65890 | 8.46833 | 15.1421 |
| AT1G69920 | 8.46634 | 16.1405 |
| AT5G39600 | 8.46214 | 20.4745 |
| AT1G68590 | 8.45991 | 1.99325 |
| AT2G14247 | 8.45741 | 2.0331 |
| AT1G33420 | 8.45676 | 5.45643 |
| AT5G67140 | 8.4528 | 21.1481 |
| AT4G34840 | 8.45125 | 8.22455 |
| AT1G14620 | 8.44918 | 16.3445 |
| AT1G04870 | 8.44696 | 34.9522 |
| AT4G34300 | 8.4424 | 4.11238 |
| AT5G57950 | 8.43909 | 10.8787 |
| AT4G39370 | 8.43653 | 4.96902 |
| AT5G53045 | 8.43613 | 16.641 |
| AT3G19170 | 8.43174 | 16.4712 |
| AT5G17360 | 8.43174 | 4.22951 |
| AT4G12410 | 8.42949 | 1.33892 |
| AT1G22040 | 8.42907 | 8.13338 |
| AT2G37650 | 8.42329 | 6.26978 |
| AT1G01830 | 8.42218 | 12.7218 |
| AT2G13960 | 8.42146 | 9.2502 |
| AT2G17890 | 8.41945 | 0.0946467 |
| AT2G20030 | 8.41905 | 0.150287 |
| AT5G45440 | 8.41739 | 3.17163 |
| AT2G09990 | 8.41383 | 22.0101 |
| AT5G19151 | 8.41329 | 6.40963 |
| AT4G30580 | 8.413 | 13.5172 |
| AT3G23620 | 8.41279 | 44.4323 |
| AT3G26090 | 8.41073 | 21.7367 |
| AT3G58130 | 8.41025 | 6.91437 |
| AT3G07700 | 8.4096 | 7.87057 |
| AT3G28860 | 8.40245 | 32.3013 |
| AT1G03990 | 8.3978 | 3.51865 |
| AT4G21670 | 8.39674 | 8.92401 |
| AT2G02360 | 8.39403 | 6.3741 |
| AT5G08210 | 8.38768 | 13.3298 |
| AT2G28060 | 8.3872 | 10.8703 |
| AT5G42920 | 8.38268 | 12.3894 |
| AT3G56990 | 8.37981 | 14.8939 |
| AT3G07410 | 8.37957 | 8.22551 |
| AT3G22800 | 8.3775 | 6.37852 |
| AT2G22890 | 8.3744 | 3.97937 |
| AT4G04320 | 8.36808 | 14.0874 |
| AT2G16485 | 8.36498 | 8.8589 |
| AT1G32930 | 8.36478 | 4.65535 |
| AT2G13790 | 8.36392 | 5.21208 |
| AT5G46150 | 8.36174 | 11.5095 |
| AT3G13620 | 8.36132 | 9.28345 |
| AT1G45130 | 8.36116 | 29.2758 |
| AT3G02600 | 8.36026 | 13.2015 |
| AT3G07310 | 8.35278 | 11.0656 |
| AT1G70190 | 8.34864 | 23.0182 |
| AT2G15000 | 8.34809 | 21.3417 |
| AT5G66160 | 8.34767 | 7.75151 |
| AT3G29760 | 8.34592 | 5.31476 |
| AT2G20620 | 8.3447 | 0.609416 |
| AT1G30440 | 8.34059 | 32.8416 |
| AT3G12370 | 8.33972 | 11.8417 |
| AT3G43590 | 8.33708 | 14.3543 |
| AT1G44160 | 8.33491 | 0.407212 |
| AT4G01830 | 8.33081 | 0.0196989 |
| AT5G05840 | 8.3297 | 0.34828 |
| AT2G01620 | 8.3259 | 3.93293 |
| AT3G26420 | 8.32169 | 14.2343 |
| AT1G21190 | 8.31893 | 19.0546 |
| AT2G41540 | 8.31677 | 5.98958 |
| AT5G22940 | 8.3148 | 2.8421 |
| AT5G16290 | 8.31293 | 19.5026 |
| AT3G45830 | 8.31074 | 11.6512 |
| AT5G01400 | 8.31058 | 6.32 |
| AT5G44150 | 8.30802 | 8.9181 |
| AT5G59830 | 8.30685 | 8.19233 |
| AT5G50840 | 8.30558 | 21.4076 |
| AT2G11280 | 8.30551 | 1.53259 |
| AT5G66860 | 8.3009 | 29.0717 |
| AT2G39740 | 8.29988 | 4.35125 |
| AT1G48610 | 8.29878 | 19.1092 |
| AT3G01460 | 8.29455 | 11.548 |
| AT4G03140 | 8.29405 | 4.08394 |
| AT3G43980 | 8.29401 | 47.6252 |
| AT4G37660 | 8.29342 | 13.446 |
| AT3G11760 | 8.28869 | 11.8168 |
| AT1G29160 | 8.28762 | 9.54906 |
| AT5G04560 | 8.28653 | 9.90489 |
| AT5G15580 | 8.28517 | 17.3069 |
| AT4G30640 | 8.28116 | 0.69283 |
| AT3G28730 | 8.28064 | 22.3999 |
| AT3G58970 | 8.28008 | 8.18507 |
| AT5G64020 | 8.27455 | 15.3376 |
| AT2G36430 | 8.27017 | 0.0516817 |
| AT3G46630 | 8.26667 | 5.25508 |
| AT4G28270 | 8.26507 | 5.62338 |
| AT3G26370 | 8.25857 | 17.1628 |
| AT5G02430 | 8.25663 | 3.49507 |
| AT1G76660 | 8.25516 | 23.1574 |
| AT5G15540 | 8.2543 | 8.08555 |
| AT1G72210 | 8.25256 | 6.92938 |
| AT4G27850 | 8.24892 | 0.310588 |
| AT4G34380 | 8.24351 | 0.0984524 |
| AT3G21400 | 8.2412 | 14.8271 |
| AT4G32670 | 8.23407 | 5.96941 |
| AT1G61580 | 8.2267 | 35.1682 |
| AT1G25240 | 8.22127 | 0.0439023 |
| AT4G13130 | 8.21983 | 1.78453 |
| AT4G23820 | 8.21713 | 16.0943 |
| AT5G63830 | 8.21111 | 8.99447 |
| AT1G65295 | 8.2105 | 16.379 |
| AT3G24020 | 8.20943 | 12.245 |
| AT3G58010 | 8.20832 | 4.49401 |
| AT2G46710 | 8.20356 | 8.44049 |
| AT1G74300 | 8.20215 | 1.10113 |
| AT1G80310 | 8.19945 | 3.05368 |
| AT3G13061 | 8.1973 | 11.1812 |
| AT4G28630 | 8.19267 | 1.79713 |
| AT2G40400 | 8.19192 | 7.40073 |
| AT2G21380 | 8.19172 | 8.57184 |
| AT4G36710 | 8.19008 | 4.61061 |
| AT1G71340 | 8.18734 | 6.97022 |
| AT1G11200 | 8.18571 | 15.2145 |
| AT4G39950 | 8.18523 | 65.1306 |
| AT3G15530 | 8.18497 | 9.53949 |
| AT5G33251 | 8.17816 | 11.2106 |
| AT1G49500 | 8.178 | 1.77846 |
| AT2G16980 | 8.17464 | 2.89167 |
| AT1G14205 | 8.17462 | 9.30905 |
| AT5G65205 | 8.17423 | 3.26267 |
| AT1G31830 | 8.17264 | 8.59889 |
| AT2G23890 | 8.17167 | 9.85067 |
| AT1G51860 | 8.1714 | 0.0812391 |
| AT5G61340 | 8.16768 | 8.75274 |
| AT5G53760 | 8.16588 | 30.7844 |
| AT2G29970 | 8.16452 | 5.06697 |
| AT4G32915 | 8.16149 | 11.7745 |
| AT1G55120 | 8.1603 | 5.5043 |
| AT2G48110 | 8.15972 | 4.97911 |
| AT5G47445 | 8.15971 | 4.04579 |
| AT2G38770 | 8.15917 | 23.5788 |
| AT1G01160 | 8.15586 | 14.1399 |
| AT5G63870 | 8.15488 | 21.9456 |
| AT4G14510 | 8.15276 | 3.53311 |
| AT3G49600 | 8.15265 | 7.70942 |
| AT3G24750 | 8.15078 | 9.43744 |
| AT4G21865 | 8.14916 | 39.6767 |
| AT3G62800 | 8.14214 | 7.73486 |
| AT5G23210 | 8.13603 | 52.3781 |
| AT5G61640 | 8.13167 | 0.187898 |
| AT5G63080 | 8.13155 | 13.5504 |
| AT1G23230 | 8.12854 | 7.4246 |
| AT5G59790 | 8.12778 | 20.8394 |
| AT5G54240 | 8.12545 | 0.604209 |
| AT3G51000 | 8.12066 | 15.0543 |
| AT3G52860 | 8.11999 | 7.12567 |
| AT3G44880 | 8.11743 | 3.51128 |
| AT3G08640 | 8.1125 | 14.0413 |
| AT3G45210 | 8.10952 | 6.56689 |
| AT4G21780 | 8.10851 | 0.894419 |
| AT4G32320 | 8.1083 | 3.93593 |
| AT3G13090 | 8.10747 | 4.34995 |
| AT1G24764 | 8.09671 | 22.9579 |
| AT3G58660 | 8.09071 | 38.206 |
| AT1G70230 | 8.08509 | 2.17674 |
| AT5G38610 | 8.08063 | 4.51227 |
| AT3G56320 | 8.07994 | 9.35728 |
| AT4G27910 | 8.07726 | 11.779 |
| AT2G43680 | 8.07449 | 15.2597 |
| AT4G26980 | 8.07068 | 6.27945 |
| AT3G56910 | 8.06988 | 11.5291 |
| AT3G57910 | 8.06353 | 7.28912 |
| AT5G11350 | 8.06175 | 8.38379 |
| AT2G39180 | 8.05943 | 9.66482 |
| AT5G66055 | 8.05931 | 9.55766 |
| AT5G35738 | 8.05676 | 1.1391 |
| AT1G11870 | 8.05602 | 5.94265 |
| AT1G18190 | 8.05346 | 11.9612 |
| AT1G13530 | 8.04761 | 6.96472 |
| AT3G62990 | 8.04326 | 0.935722 |
| AT1G14650 | 8.04152 | 13.411 |
| AT3G16150 | 8.03938 | 15.0998 |
| AT3G03210 | 8.03848 | 8.03025 |
| AT3G17750 | 8.03782 | 10.4391 |
| AT1G52980 | 8.0364 | 24.1479 |
| AT1G01940 | 8.03429 | 17.8717 |
| AT4G13250 | 8.03369 | 12.6481 |
| AT4G13630 | 8.0289 | 19.3972 |
| AT3G56130 | 8.02752 | 35.1884 |
| AT2G31060 | 8.02585 | 24.726 |
| AT1G21640 | 8.02356 | 9.91679 |
| AT1G22050 | 8.02246 | 4.3324 |
| AT2G25740 | 8.02041 | 10.4889 |
| AT5G55530 | 8.01935 | 9.3977 |
| AT1G67170 | 8.01838 | 10.0756 |
| AT3G02830 | 8.0104 | 6.06948 |
| AT2G40190 | 8.01014 | 8.36102 |
| AT1G53633 | 8.00909 | 6.76294 |
| AT5G03910 | 8.00756 | 11.2825 |
| AT5G02470 | 7.99998 | 9.09023 |
| AT2G20625 | 7.99965 | 1.65959 |
| AT5G65530 | 7.99931 | 16.0368 |
| AT1G71840 | 7.99577 | 18.2771 |
| AT3G56740 | 7.99386 | 8.67298 |
| AT3G16310 | 7.98926 | 18.4717 |
| AT5G56900 | 7.98856 | 20.5398 |
| AT1G54500 | 7.98526 | 1.73585 |
| AT5G66720 | 7.98397 | 17.2278 |
| AT4G33700 | 7.98317 | 15.716 |
| AT2G18100 | 7.98301 | 6.11163 |
| AT1G05660 | 7.98248 | 18.2354 |
| AT4G24940 | 7.98072 | 23.1305 |
| AT3G19490 | 7.97967 | 14.162 |
| AT1G49730 | 7.97967 | 8.23347 |
| AT3G60020 | 7.96983 | 5.06764 |
| AT3G61160 | 7.96973 | 5.69362 |
| AT5G22010 | 7.96454 | 9.43152 |
| AT5G44710 | 7.95663 | 28.1612 |
| AT5G39500 | 7.9531 | 9.28002 |
| AT3G08020 | 7.95041 | 12.0159 |
| AT3G47790 | 7.94812 | 5.59308 |
| AT2G41705 | 7.94807 | 10.3797 |
| AT3G28190 | 7.94609 | 28.0135 |
| AT5G07900 | 7.94578 | 2.35226 |
| AT2G47190 | 7.94444 | 13.9651 |
| AT1G19990 | 7.94195 | 14.3578 |
| AT2G38170 | 7.9415 | 6.23551 |
| AT5G49880 | 7.93339 | 11.8409 |
| AT3G54760 | 7.93217 | 22.7242 |
| AT3G03010 | 7.93201 | 13.9306 |
| AT1G68660 | 7.93147 | 14.05 |
| AT3G01200 | 7.92988 | 7.96522 |
| AT4G32120 | 7.92921 | 11.7729 |
| AT3G08950 | 7.92728 | 11.6333 |
| AT4G13170 | 7.9238 | 30.3495 |
| AT4G03390 | 7.92078 | 15.0965 |
| AT5G58003 | 7.92048 | 13.7556 |
| AT4G28570 | 7.91941 | 9.10367 |
| AT5G15270 | 7.90994 | 10.1115 |
| AT5G65685 | 7.90911 | 7.42055 |
| AT2G24150 | 7.90585 | 5.30181 |
| AT3G58540 | 7.90463 | 8.02755 |
| AT3G47400 | 7.90405 | 16.4037 |
| AT5G27100 | 7.90356 | 0.735548 |
| AT3G57050 | 7.90179 | 24.1943 |
| AT2G20690 | 7.89177 | 9.60746 |
| AT2G44900 | 7.89051 | 6.38105 |
| AT3G53400 | 7.88863 | 18.6829 |
| AT2G25570 | 7.88564 | 8.02814 |
| AT3G53540 | 7.88195 | 7.29938 |
| AT5G11910 | 7.88149 | 6.09073 |
| AT4G33350 | 7.88056 | 16.0524 |
| AT4G26680 | 7.88051 | 1.25092 |
| AT1G63660 | 7.87793 | 20.6472 |
| AT2G18465 | 7.87744 | 6.36627 |
| AT4G37430 | 7.87656 | 4.2277 |
| AT5G65760 | 7.87525 | 17.0766 |
| AT1G08660 | 7.87452 | 19.7251 |
| AT5G08420 | 7.87278 | 31.9127 |
| AT3G58760 | 7.87087 | 13.7701 |
| AT3G14310 | 7.87028 | 13.5479 |
| AT3G62390 | 7.86914 | 7.72869 |
| AT4G34070 | 7.86797 | 8.00322 |
| AT2G40180 | 7.86752 | 7.13834 |
| AT3G13350 | 7.8659 | 14.211 |
| AT5G07322 | 7.86303 | 2.5956 |
| AT1G16520 | 7.85879 | 13.3248 |
| AT2G35040 | 7.85793 | 34.1793 |
| AT1G48170 | 7.85763 | 8.09916 |
| AT4G30900 | 7.85642 | 6.48083 |
| AT5G66080 | 7.85417 | 7.13934 |
| AT3G22520 | 7.85286 | 11.9841 |
| AT3G20490 | 7.85136 | 9.68386 |
| AT3G13480 | 7.8509 | 5.36987 |
| AT2G14740 | 7.8508 | 23.1174 |
| AT5G56000 | 7.84914 | 12.5682 |
| AT1G65032 | 7.84876 | 11.2335 |
| AT5G52430 | 7.84806 | 17.8666 |
| AT3G62530 | 7.84634 | 33.1564 |
| AT3G49120 | 7.84592 | 2.10598 |
| AT1G17665 | 7.84296 | 12.1417 |
| AT3G58020 | 7.83864 | 14.3712 |
| AT4G22233 | 7.83694 | 1.37574 |
| AT2G16380 | 7.83655 | 2.74814 |
| AT5G02150 | 7.83597 | 12.8739 |
| AT1G12820 | 7.83204 | 7.56417 |
| AT5G51960 | 7.82924 | 19.6302 |
| AT5G59580 | 7.82851 | 9.20361 |
| AT1G22860 | 7.82677 | 12.0323 |
| AT4G19990 | 7.82374 | 12.3554 |
| AT5G46360 | 7.82314 | 0.209611 |
| AT1G29810 | 7.81958 | 13.497 |
| AT3G18100 | 7.81943 | 5.83543 |
| AT3G04870 | 7.81867 | 9.23323 |
| AT5G01030 | 7.81742 | 4.49479 |
| AT4G30950 | 7.81625 | 10.3106 |
| AT4G31670 | 7.81432 | 10.1265 |
| AT1G53720 | 7.81429 | 10.9195 |
| AT4G23840 | 7.81125 | 13.7201 |
| AT1G60860 | 7.81077 | 9.15263 |
| AT1G60890 | 7.81038 | 3.77621 |
| AT5G38860 | 7.81031 | 4.29692 |
| AT5G49800 | 7.81006 | 14.7091 |
| AT5G24352 | 7.80988 | 8.4062 |
| AT4G23590 | 7.80659 | 75.7888 |
| AT2G33610 | 7.79551 | 15.5844 |
| AT4G16670 | 7.79042 | 6.27634 |
| AT2G47990 | 7.79019 | 10.7639 |
| AT4G33680 | 7.78889 | 30.6789 |
| AT2G40280 | 7.78579 | 18.5148 |
| AT5G25752 | 7.78401 | 8.63037 |
| AT4G24630 | 7.78272 | 6.33779 |
| AT2G03810 | 7.78186 | 17.8606 |
| AT1G07200 | 7.7812 | 6.09259 |
| AT2G06510 | 7.77833 | 10.4299 |
| AT5G20200 | 7.77656 | 11.8464 |
| AT2G48030 | 7.77222 | 6.18825 |
| AT3G03120 | 7.77169 | 7.41519 |
| AT1G19980 | 7.77081 | 11.8263 |
| AT4G11120 | 7.76913 | 20.1203 |
| AT4G13160 | 7.76671 | 18.2419 |
| AT5G61360 | 7.76214 | 2.02832 |
| AT1G09620 | 7.76209 | 35.7316 |
| AT4G15955 | 7.75859 | 6.50323 |
| AT1G49400 | 7.75468 | 20.0895 |
| AT2G03800 | 7.75147 | 6.65005 |
| AT5G37710 | 7.74266 | 11.1306 |
| AT5G50370 | 7.74263 | 21.4611 |
| AT3G54500 | 7.74183 | 18.5501 |
| AT2G48000 | 7.74063 | 7.53517 |
| AT4G09560 | 7.74051 | 5.76513 |
| AT1G05150 | 7.73828 | 10.7392 |
| AT3G55530 | 7.73204 | 7.65702 |
| AT2G34655 | 7.72599 | 0.363566 |
| AT3G59480 | 7.72429 | 70.3164 |
| AT5G58787 | 7.72367 | 10.8026 |
| AT1G06430 | 7.72055 | 5.42013 |
| AT1G80650 | 7.71795 | 2.83346 |
| AT1G34780 | 7.71684 | 10.5482 |
| AT5G24314 | 7.71561 | 3.50863 |
| AT2G33290 | 7.71558 | 2.8643 |
| AT5G06265 | 7.71256 | 15.6951 |
| AT4G24170 | 7.70902 | 4.72723 |
| AT1G11280 | 7.69621 | 14.7452 |
| AT1G67792 | 7.69503 | 6.78658 |
| AT2G26460 | 7.69299 | 7.90856 |
| AT1G64740 | 7.69126 | 5.96742 |
| AT3G56891 | 7.69099 | 4.15678 |
| AT1G20560 | 7.68785 | 6.65471 |
| AT1G31500 | 7.68754 | 10.4941 |
| AT5G20700 | 7.68687 | 7.79794 |
| AT3G01500 | 7.68422 | 8.23005 |
| AT3G48800 | 7.68198 | 7.33902 |
| AT5G42660 | 7.68173 | 13.8718 |
| AT1G19690 | 7.68016 | 6.29379 |
| AT5G26860 | 7.67917 | 15.8096 |
| AT3G07980 | 7.67807 | 3.85375 |
| AT4G27520 | 7.67752 | 7.28082 |
| AT1G63430 | 7.67524 | 10.8382 |
| AT1G80590 | 7.67315 | 7.20122 |
| AT5G07380 | 7.67228 | 9.65507 |
| AT1G14910 | 7.67036 | 19.5996 |
| AT4G09770 | 7.66564 | 0.574639 |
| AT3G59110 | 7.65649 | 11.9543 |
| AT4G32010 | 7.65284 | 12.3941 |
| AT5G22790 | 7.6523 | 11.0054 |
| AT3G25740 | 7.64964 | 14.9052 |
| AT5G15710 | 7.64959 | 6.21222 |
| AT1G21065 | 7.64663 | 14.5183 |
| AT2G31790 | 7.646 | 1.73103 |
| AT1G70560 | 7.64519 | 20.6513 |
| AT4G33960 | 7.64379 | 2.79233 |
| AT2G16870 | 7.64277 | 2.23463 |
| AT2G14260 | 7.63928 | 13.2021 |
| AT2G47820 | 7.63873 | 11.204 |
| AT1G49820 | 7.6376 | 15.0015 |
| AT5G60200 | 7.62839 | 15.8982 |
| AT1G63180 | 7.6266 | 23.5249 |
| AT5G03640 | 7.6264 | 0.00705422 |
| AT3G16570 | 7.62188 | 4.49514 |
| AT3G50340 | 7.62093 | 15.3968 |
| AT4G39560 | 7.61923 | 4.67989 |
| AT2G37680 | 7.61754 | 10.0662 |
| AT1G62960 | 7.61373 | 6.61128 |
| AT1G35460 | 7.61342 | 6.06074 |
| AT1G74330 | 7.61119 | 6.17928 |
| AT5G41150 | 7.61057 | 6.1733 |
| AT1G08540 | 7.60852 | 5.57454 |
| AT1G06540 | 7.60718 | 5.27833 |
| AT1G76510 | 7.60674 | 13.8757 |
| AT5G57060 | 7.60021 | 11.5064 |
| AT1G08845 | 7.59871 | 8.74267 |
| AT1G19490 | 7.59102 | 9.77961 |
| AT3G10620 | 7.58774 | 15.9222 |
| AT2G34680 | 7.58735 | 13.3255 |
| AT2G47940 | 7.58579 | 10.4476 |
| AT3G23640 | 7.58375 | 10.3526 |
| AT3G59980 | 7.57708 | 7.96284 |
| AT2G37570 | 7.57583 | 10.1956 |
| AT2G10606 | 7.57451 | 1.72517 |
| AT3G21650 | 7.57014 | 7.66586 |
| AT5G59540 | 7.57002 | 1.55911 |
| AT3G49220 | 7.56981 | 8.69861 |
| AT1G12500 | 7.56715 | 10.832 |
| AT5G65540 | 7.56255 | 10.2772 |
| AT4G16410 | 7.56239 | 7.32285 |
| AT3G06210 | 7.5614 | 10.4179 |
| AT4G16280 | 7.5586 | 6.20518 |
| AT5G02480 | 7.55769 | 12.5558 |
| AT3G45660 | 7.55682 | 6.34769 |
| AT2G03070 | 7.55574 | 10.0388 |
| AT5G16040 | 7.55341 | 10.283 |
| AT5G17250 | 7.54869 | 4.09979 |
| AT1G64385 | 7.5473 | 10.5171 |
| AT5G07842 | 7.54505 | 7.09176 |
| AT2G04940 | 7.54384 | 6.0955 |
| AT4G33050 | 7.54189 | 1.26527 |
| AT5G65920 | 7.54145 | 17.9905 |
| AT3G54000 | 7.54122 | 7.66987 |
| AT5G42600 | 7.54056 | 6.17477 |
| AT5G65040 | 7.53919 | 7.24901 |
| AT3G60620 | 7.53834 | 5.71513 |
| AT1G04230 | 7.53602 | 12.3667 |
| AT5G54920 | 7.5351 | 6.45373 |
| AT5G58490 | 7.53389 | 13.3814 |
| AT5G40170 | 7.53384 | 2.32045 |
| AT2G34020 | 7.5331 | 55.5858 |
| AT5G06960 | 7.5327 | 7.22134 |
| AT1G69170 | 7.53171 | 9.8885 |
| AT1G27840 | 7.52959 | 9.48539 |
| AT4G21745 | 7.52484 | 0.204376 |
| AT2G47930 | 7.52467 | 10.3753 |
| AT1G02870 | 7.52352 | 32.4797 |
| AT1G50020 | 7.52221 | 6.99136 |
| AT5G64680 | 7.52167 | 20.1638 |
| AT1G21550 | 7.51991 | 3.31921 |
| AT4G22140 | 7.51841 | 16.3248 |
| AT5G17010 | 7.5183 | 5.11816 |
| AT1G07730 | 7.51799 | 1.84677 |
| AT1G75310 | 7.51565 | 6.2183 |
| AT2G39090 | 7.51021 | 15.2588 |
| AT3G49060 | 7.5098 | 6.14866 |
| AT1G67370 | 7.50852 | 7.19925 |
| AT3G60180 | 7.50737 | 6.2629 |
| AT2G35720 | 7.50368 | 8.19297 |
| AT2G32160 | 7.50187 | 0.794936 |
| AT3G29780 | 7.50129 | 1.63102 |
| AT3G26990 | 7.49947 | 6.66395 |
| AT1G74160 | 7.49943 | 6.94709 |
| AT3G01560 | 7.49799 | 6.68983 |
| AT3G14440 | 7.49749 | 2.23388 |
| AT4G23690 | 7.49509 | 12.7568 |
| AT2G25320 | 7.49125 | 4.89149 |
| AT1G64880 | 7.48854 | 35.0248 |
| AT1G78990 | 7.4828 | 4.44644 |
| AT5G12410 | 7.47852 | 29.3834 |
| AT3G22590 | 7.47831 | 13.2062 |
| AT5G10190 | 7.47478 | 13.7909 |
| AT4G31510 | 7.47419 | 24.5276 |
| AT4G24830 | 7.47327 | 24.6018 |
| AT2G41120 | 7.47315 | 1.59486 |
| AT3G61300 | 7.47312 | 0.0478457 |
| AT5G19630 | 7.47266 | 4.75092 |
| AT5G24120 | 7.47194 | 5.44571 |
| AT5G47670 | 7.47185 | 2.56227 |
| AT4G22600 | 7.47183 | 0.331342 |
| AT4G03490 | 7.47103 | 0.985009 |
| AT5G15520 | 7.46798 | 29.7931 |
| AT1G05640 | 7.46793 | 2.01089 |
| AT1G80890 | 7.46725 | 17.3292 |
| AT5G01650 | 7.4669 | 11.3704 |
| AT5G47020 | 7.46258 | 10.6138 |
| AT1G09350 | 7.45717 | 2.97078 |
| AT5G06580 | 7.45387 | 5.38211 |
| AT2G03450 | 7.45306 | 4.65491 |
| AT1G60770 | 7.45239 | 18.3669 |
| AT1G71720 | 7.44554 | 7.74481 |
| AT4G30340 | 7.43915 | 16.5353 |
| AT4G27030 | 7.43382 | 2.92956 |
| AT3G30580 | 7.42804 | 8.20209 |
| AT5G11380 | 7.42522 | 8.96294 |
| AT5G52880 | 7.42468 | 2.90661 |
| AT4G26640 | 7.41848 | 12.8385 |
| AT2G33450 | 7.41842 | 14.326 |
| AT3G48050 | 7.41689 | 5.38968 |
| AT3G21560 | 7.41631 | 0.706689 |
| AT1G01560 | 7.40602 | 3.08209 |
| AT3G01800 | 7.39939 | 10.9478 |
| AT2G03270 | 7.39901 | 9.98856 |
| AT1G55860 | 7.39662 | 7.24389 |
| AT1G62610 | 7.39584 | 2.2348 |
| AT1G53670 | 7.39552 | 12.5133 |
| AT3G26600 | 7.39311 | 8.61494 |
| AT3G18280 | 7.3924 | 5.39979 |
| AT4G38210 | 7.3922 | 4.50369 |
| AT3G20850 | 7.38969 | 0.195471 |
| AT1G08760 | 7.3868 | 11.8546 |
| AT3G09405 | 7.38534 | 3.58827 |
| AT1G48780 | 7.3844 | 7.41522 |
| AT1G52670 | 7.37847 | 26.4032 |
| AT1G16870 | 7.37629 | 20.8393 |
| AT3G59490 | 7.3735 | 13.0764 |
| AT3G61550 | 7.37236 | 0.295112 |
| AT5G41790 | 7.37013 | 15.5794 |
| AT5G58300 | 7.3679 | 7.35979 |
| AT5G22020 | 7.36727 | 4.65689 |
| AT1G79200 | 7.36316 | 10.6781 |
| AT1G15250 | 7.36141 | 9.25807 |
| AT5G16950 | 7.35174 | 12.4419 |
| AT4G08035 | 7.34994 | 13.0753 |
| AT5G06090 | 7.34847 | 0.4861 |
| AT4G08450 | 7.34767 | 0.139961 |
| AT5G26920 | 7.34471 | 9.11153 |
| AT4G29830 | 7.34069 | 12.5701 |
| AT1G08750 | 7.34059 | 15.546 |
| AT4G04620 | 7.3384 | 6.75012 |
| AT1G74750 | 7.33795 | 6.75754 |
| AT4G31530 | 7.33713 | 6.23015 |
| AT1G49510 | 7.33591 | 7.84888 |
| AT3G62940 | 7.33259 | 19.9188 |
| AT5G45560 | 7.32815 | 6.33955 |
| AT5G18180 | 7.32783 | 4.61265 |
| AT4G20190 | 7.32657 | 0.0423595 |
| AT5G65460 | 7.32507 | 11.4066 |
| AT1G73180 | 7.32256 | 16.7016 |
| AT1G02980 | 7.31974 | 4.39184 |
| AT5G17780 | 7.31657 | 3.12753 |
| AT2G45510 | 7.31616 | 8.56567 |
| AT1G52370 | 7.31392 | 10.2421 |
| AT3G19920 | 7.31043 | 1.21619 |
| AT4G32272 | 7.30951 | 8.29877 |
| AT2G04500 | 7.30778 | 1.49049 |
| AT1G54070 | 7.30669 | 0.744897 |
| AT4G11630 | 7.29593 | 14.4508 |
| AT3G20020 | 7.29208 | 12.9616 |
| AT1G01220 | 7.28841 | 7.94713 |
| AT2G41700 | 7.28535 | 7.81338 |
| AT3G07740 | 7.28532 | 10.1718 |
| AT5G07860 | 7.27803 | 14.1754 |
| AT1G32730 | 7.27284 | 13.7611 |
| AT3G11070 | 7.27169 | 17.8803 |
| AT2G27505 | 7.27167 | 1.31178 |
| AT5G44650 | 7.26934 | 2.91119 |
| AT4G36680 | 7.26891 | 12.6416 |
| AT5G10560 | 7.2678 | 17.9274 |
| AT4G22320 | 7.26691 | 9.42896 |
| AT2G44650 | 7.26665 | 10.2132 |
| AT5G57040 | 7.26338 | 3.92512 |
| AT4G38545 | 7.26311 | 11.7188 |
| AT1G60810 | 7.26192 | 11.0792 |
| AT2G36410 | 7.26053 | 13.7937 |
| AT1G03920 | 7.26049 | 2.24393 |
| AT4G23660 | 7.26016 | 7.81345 |
| AT5G61020 | 7.24788 | 39.4456 |
| AT1G76400 | 7.24773 | 22.9888 |
| AT5G11425 | 7.24681 | 4.6874 |
| AT4G13720 | 7.24503 | 13.8715 |
| AT1G73100 | 7.23967 | 5.64389 |
| AT3G23590 | 7.23776 | 10.9298 |
| AT2G25650 | 7.23762 | 9.77485 |
| AT4G36052 | 7.23535 | 5.89311 |
| AT3G55230 | 7.23472 | 9.5631 |
| AT5G45116 | 7.23456 | 0.873391 |
| AT5G24100 | 7.23416 | 2.32379 |
| AT2G33600 | 7.23379 | 4.79378 |
| AT3G50350 | 7.23274 | 8.66042 |
| AT4G23070 | 7.2282 | 0.511619 |
| AT5G39785 | 7.22313 | 4.88454 |
| AT3G13450 | 7.22274 | 6.06035 |
| AT1G55152 | 7.22175 | 3.80336 |
| AT3G51510 | 7.21864 | 5.02053 |
| AT1G18600 | 7.21511 | 5.49534 |
| AT4G15530 | 7.2151 | 37.4071 |
| AT5G28626.1 | 7.21426 | 3.81875 |
| AT4G37000 | 7.21312 | 2.52623 |
| AT5G64670 | 7.21182 | 23.1142 |
| AT4G18590 | 7.20667 | 20.1144 |
| AT5G14500 | 7.20534 | 11.7338 |
| AT3G12280 | 7.20454 | 13.169 |
| AT1G53780 | 7.2032 | 8.74249 |
| AT5G20600 | 7.20273 | 16.8809 |
| AT2G37025 | 7.18913 | 5.87863 |
| AT4G36810 | 7.18791 | 7.24184 |
| AT5G35740 | 7.18714 | 18.8862 |
| AT2G26870 | 7.18614 | 14.6314 |
| AT1G56170 | 7.18404 | 12.7286 |
| AT3G10520 | 7.18231 | 9.12649 |
| AT1G16570 | 7.18132 | 10.7074 |
| AT2G44850 | 7.18086 | 1.88688 |
| AT4G23440 | 7.17984 | 7.12432 |
| AT1G29418 | 7.17852 | 25.6431 |
| AT2G19940 | 7.17704 | 14.8003 |
| AT1G75710 | 7.17571 | 21.2198 |
| AT4G26310 | 7.17522 | 13.2918 |
| AT3G43240 | 7.17513 | 13.7325 |
| AT5G05790 | 7.17415 | 10.8063 |
| AT5G47070 | 7.1732 | 7.77783 |
| AT3G56080 | 7.1716 | 0.031445 |
| AT2G38050 | 7.16438 | 4.28665 |
| AT4G23740 | 7.15936 | 11.9171 |
| AT5G57200 | 7.15807 | 0.0236834 |
| AT4G31115 | 7.15542 | 17.2575 |
| AT3G09010 | 7.15196 | 2.13839 |
| AT1G21340 | 7.15111 | 60.2249 |
| AT1G19680 | 7.15002 | 7.64564 |
| AT3G07990 | 7.14969 | 19.371 |
| AT1G06980 | 7.14962 | 5.83589 |
| AT5G13980 | 7.14824 | 12.3219 |
| AT2G14960 | 7.14755 | 9.97341 |
| AT4G10030 | 7.14686 | 7.09183 |
| AT5G19910 | 7.1428 | 8.68572 |
| AT5G08430 | 7.1402 | 7.43187 |
| AT5G15550 | 7.14015 | 26.7712 |
| AT2G27460 | 7.13824 | 13.5557 |
| AT5G23250 | 7.13727 | 17.9859 |
| AT4G02290 | 7.13386 | 30.7548 |
| AT2G37195 | 7.12873 | 6.67018 |
| AT4G12690 | 7.12832 | 1.2062 |
| AT2G32560 | 7.12737 | 17.6348 |
| AT4G35870 | 7.12561 | 4.76108 |
| AT5G16170 | 7.12509 | 11.2864 |
| AT5G63660 | 7.12469 | 88.4502 |
| AT1G58230 | 7.12142 | 6.85082 |
| AT4G22340 | 7.12135 | 18.553 |
| AT3G48150 | 7.12089 | 11.4573 |
| AT2G04700 | 7.1202 | 16.4425 |
| AT3G51360 | 7.11907 | 0.0474443 |
| AT1G80850 | 7.11874 | 2.98137 |
| AT3G52570 | 7.11705 | 8.5364 |
| AT2G42240 | 7.11681 | 12.165 |
| AT4G14570 | 7.11652 | 12.0984 |
| AT3G46960 | 7.11205 | 10.4081 |
| AT1G14080 | 7.10867 | 14.151 |
| AT5G15460 | 7.1083 | 5.93158 |
| AT5G51580 | 7.10749 | 2.93904 |
| AT3G18600 | 7.10633 | 36.7029 |
| AT5G64850 | 7.10592 | 4.00659 |
| AT3G25719 | 7.10214 | 679.041 |
| AT1G09995 | 7.1003 | 7.94966 |
| AT5G07710 | 7.09763 | 4.97014 |
| AT1G54010 | 7.09741 | 88.6631 |
| AT5G02560 | 7.09739 | 2.8611 |
| AT2G41380 | 7.0924 | 5.8951 |
| AT3G19210 | 7.08858 | 5.88584 |
| AT2G40610 | 7.08773 | 7.00744 |
| AT4G02550 | 7.08739 | 8.49347 |
| AT5G01060 | 7.08496 | 0.0105532 |
| AT3G12970 | 7.08247 | 4.55578 |
| AT5G03900 | 7.08118 | 12.1214 |
| AT4G16563 | 7.0778 | 1.08938 |
| AT5G16450 | 7.07647 | 9.76507 |
| AT3G44260 | 7.07385 | 5.49537 |
| AT1G67950 | 7.07381 | 12.8381 |
| AT1G03180 | 7.07152 | 7.50649 |
| AT3G45230 | 7.06551 | 33.0954 |
| AT1G27110 | 7.06431 | 8.13845 |
| AT4G17950 | 7.0637 | 16.3469 |
| AT2G35110 | 7.06248 | 11.2814 |
| AT3G27170 | 7.05942 | 2.11832 |
| AT4G25710 | 7.05866 | 4.45415 |
| AT2G22370 | 7.05823 | 6.89148 |
| AT5G48720 | 7.05618 | 16.614 |
| AT2G30160 | 7.04948 | 11.9763 |
| AT5G41610 | 7.04922 | 5.73084 |
| AT5G16940 | 7.04823 | 5.34058 |
| AT3G22660 | 7.04476 | 39.3913 |
| AT5G08110 | 7.04295 | 8.99894 |
| AT4G37483 | 7.04209 | 15.8015 |
| AT5G63340 | 7.04089 | 4.5179 |
| AT5G40470 | 7.04045 | 3.22599 |
| AT1G68050 | 7.03761 | 4.49194 |
| AT3G45430 | 7.03618 | 11.3515 |
| AT5G46090 | 7.0332 | 2.92357 |
| AT2G44760 | 7.02696 | 5.82324 |
| AT1G15810 | 7.0204 | 19.8383 |
| AT5G46770 | 7.0148 | 1.74851 |
| AT3G06770 | 7.01275 | 4.83176 |
| AT5G47950 | 7.0119 | 13.0801 |
| AT1G53785 | 7.00905 | 7.09448 |
| AT5G48330 | 7.00809 | 4.34659 |
| AT2G39430 | 7.00502 | 3.72046 |
| AT4G26220 | 7.00296 | 3.04174 |
| AT2G45720 | 6.99841 | 6.84621 |
| AT3G54670 | 6.99787 | 15.8735 |
| AT5G11270 | 6.99416 | 6.41599 |
| AT5G48680 | 6.99153 | 15.1732 |
| AT5G21326 | 6.98845 | 12.0692 |
| AT4G22770 | 6.98543 | 2.71641 |
| AT1G29070 | 6.98529 | 8.30427 |
| AT1G70180 | 6.98458 | 11.4566 |
| AT4G14905 | 6.98368 | 15.3431 |
| AT1G13120 | 6.98256 | 10.8503 |
| AT3G56850 | 6.97971 | 14.4905 |
| AT3G50280 | 6.97949 | 2.48473 |
| AT3G59650 | 6.97916 | 25.9214 |
| AT4G02210 | 6.97762 | 11.1184 |
| AT5G46850 | 6.97125 | 7.38174 |
| AT3G51110 | 6.96467 | 8.02837 |
| AT3G07050 | 6.96071 | 48.4259 |
| AT4G25470 | 6.95951 | 1.86786 |
| AT4G39920 | 6.9578 | 2.90841 |
| AT2G34720 | 6.95692 | 13.5385 |
| AT1G79820 | 6.95355 | 17.2304 |
| AT2G04378 | 6.95253 | 18.9582 |
| AT1G48635 | 6.95112 | 11.5239 |
| AT3G13490 | 6.95102 | 12.5952 |
| AT1G05894 | 6.95004 | 5.8453 |
| AT1G50490 | 6.94782 | 36.4717 |
| AT5G41580 | 6.94773 | 10.7792 |
| AT1G49620 | 6.94554 | 7.46096 |
| AT1G02960 | 6.94523 | 7.85949 |
| AT3G14070 | 6.94484 | 2.80479 |
| AT3G13570 | 6.94106 | 19.7262 |
| AT5G64970 | 6.94002 | 6.63834 |
| AT3G06483 | 6.93999 | 16.8443 |
| AT4G03460 | 6.9382 | 2.437 |
| AT3G13710 | 6.93742 | 3.0725 |
| AT1G19250 | 6.93717 | 0.577603 |
| AT1G70800 | 6.93491 | 6.13122 |
| AT3G62630 | 6.93403 | 6.7267 |
| AT5G02940 | 6.92921 | 6.77501 |
| AT2G36400 | 6.92783 | 13.3531 |
| AT3G09900 | 6.92563 | 25.1379 |
| AT4G01890 | 6.92313 | 4.7771 |
| AT3G47630 | 6.92269 | 8.94293 |
| AT3G06590 | 6.92159 | 4.38137 |
| AT3G55140 | 6.92011 | 16.6811 |
| AT5G34853.1 | 6.91902 | 8.04766 |
| AT4G04710 | 6.9186 | 0.0212235 |
| AT4G27670 | 6.91786 | 18.5853 |
| AT4G38960 | 6.91756 | 6.02802 |
| AT1G63270 | 6.91578 | 9.46318 |
| AT1G16350 | 6.91552 | 16.5271 |
| AT4G02340 | 6.91414 | 18.8245 |
| AT5G14140 | 6.91282 | 13.6446 |
| AT1G80810 | 6.91111 | 7.62239 |
| AT1G12830 | 6.90818 | 16.9719 |
| AT5G59730 | 6.90394 | 6.17665 |
| AT3G05680 | 6.89438 | 10.0873 |
| AT1G09300 | 6.89206 | 5.68042 |
| AT5G43680 | 6.89056 | 5.99729 |
| AT5G58130 | 6.88969 | 13.424 |
| AT5G07340 | 6.88872 | 21.5 |
| AT5G49840 | 6.88858 | 8.43086 |
| AT1G74560 | 6.88408 | 51.6981 |
| AT4G32050 | 6.88308 | 6.04917 |
| AT3G59150 | 6.88257 | 7.78699 |
| AT2G21250 | 6.87316 | 26.4885 |
| AT2G19690 | 6.87315 | 6.8695 |
| AT3G60310 | 6.87096 | 5.27439 |
| AT5G63200 | 6.87091 | 10.2264 |
| AT4G19080 | 6.86966 | 3.79871 |
| AT1G33330 | 6.86905 | 4.59461 |
| AT1G10230 | 6.86709 | 5.54362 |
| AT1G33120 | 6.86193 | 32.2172 |
| AT1G68070 | 6.85959 | 13.0765 |
| AT4G15550 | 6.85892 | 24.4531 |
| AT1G52930 | 6.85407 | 45.3142 |
| AT3G56140 | 6.85286 | 3.88122 |
| AT4G13310 | 6.84932 | 0.169303 |
| AT5G06950 | 6.84785 | 6.79054 |
| AT5G09290 | 6.84672 | 1.37236 |
| AT5G47940 | 6.84627 | 5.28264 |
| AT3G20940 | 6.84581 | 30.9444 |
| AT1G18900 | 6.84503 | 7.85382 |
| AT5G54140 | 6.84481 | 6.2821 |
| AT4G25180 | 6.84456 | 7.36456 |
| AT5G18200 | 6.84154 | 4.72106 |
| AT4G15953 | 6.83435 | 3.14822 |
| AT4G36791 | 6.83389 | 56.6575 |
| AT5G09445 | 6.83068 | 6.50119 |
| AT3G54750 | 6.83067 | 18.0858 |
| AT4G00290 | 6.82948 | 4.07734 |
| AT3G54280 | 6.8293 | 10.1075 |
| AT5G07620 | 6.82821 | 10.149 |
| AT4G36770 | 6.82741 | 3.26631 |
| AT3G15350 | 6.82661 | 7.5525 |
| AT1G57700 | 6.82657 | 6.88072 |
| AT5G46790 | 6.82014 | 5.91232 |
| AT1G78780 | 6.81706 | 4.66218 |
| AT4G18400 | 6.81664 | 6.42302 |
| AT5G21050 | 6.81651 | 1.37136 |
| AT4G31810 | 6.81555 | 24.4537 |
| AT5G61600 | 6.8146 | 5.3361 |
| AT1G27980 | 6.81409 | 9.05931 |
| AT1G03200 | 6.81337 | 2.59831 |
| AT5G52070 | 6.81161 | 11.4925 |
| AT2G36740 | 6.80351 | 19.27 |
| AT2G45730 | 6.79727 | 16.4934 |
| AT3G58670 | 6.79707 | 15.3456 |
| AT4G30370 | 6.79582 | 3.7296 |
| AT2G20310 | 6.79551 | 7.5037 |
| AT3G01430 | 6.79385 | 1.764 |
| AT4G28360 | 6.79295 | 27.0217 |
| AT3G58800 | 6.79099 | 7.78347 |
| AT3G57800 | 6.78936 | 18.8184 |
| AT2G39690 | 6.78931 | 0.00991281 |
| AT3G57000 | 6.78771 | 28.7758 |
| AT2G02410 | 6.78504 | 1.94344 |
| AT5G62630 | 6.78242 | 2.50346 |
| AT5G65260 | 6.78043 | 9.74492 |
| AT3G06510 | 6.77534 | 6.98933 |
| AT3G27460 | 6.77474 | 9.5744 |
| AT5G09690 | 6.77141 | 4.54401 |
| AT5G15750 | 6.77087 | 36.6954 |
| AT5G55510 | 6.76816 | 8.98321 |
| AT1G77660 | 6.76596 | 8.0427 |
| AT1G56680 | 6.7644 | 43.2239 |
| AT3G57170 | 6.76141 | 9.72695 |
| AT5G10160 | 6.75571 | 31.2506 |
| AT1G15440 | 6.75535 | 13.8882 |
| AT1G56540 | 6.75452 | 1.44745 |
| AT5G66985 | 6.75327 | 2.55891 |
| AT2G05910 | 6.75198 | 1.05939 |
| AT5G02550 | 6.75097 | 21.0352 |
| AT4G15520 | 6.7507 | 10.5793 |
| AT1G30455 | 6.74576 | 1.8058 |
| AT5G65440 | 6.74499 | 14.214 |
| AT5G67170 | 6.74384 | 9.78397 |
| AT5G05400 | 6.73759 | 1.22751 |
| AT3G08940 | 6.73511 | 2.76652 |
| AT3G25545 | 6.7329 | 9.70696 |
| AT2G35520 | 6.73146 | 17.2284 |
| AT2G43130 | 6.73063 | 11.2615 |
| AT3G06990 | 6.72998 | 0.811217 |
| AT5G67070 | 6.72567 | 24.1212 |
| AT1G57660 | 6.72496 | 54.9453 |
| AT5G41020 | 6.72407 | 18.4102 |
| AT4G15840 | 6.72327 | 8.35924 |
| AT1G75130 | 6.72154 | 8.05758 |
| AT4G04925 | 6.71874 | 3.11704 |
| AT3G11240 | 6.71757 | 5.57226 |
| AT5G37590 | 6.71674 | 8.96862 |
| AT1G51950 | 6.71643 | 9.25376 |
| AT2G02955 | 6.71312 | 2.0716 |
| AT5G11820 | 6.71241 | 1.65294 |
| AT5G38110 | 6.70716 | 17.1931 |
| AT4G09340 | 6.70636 | 9.53727 |
| AT2G18640 | 6.698 | 0.0288604 |
| AT1G75490 | 6.69755 | 7.0354 |
| AT1G22140 | 6.69742 | 8.92213 |
| AT3G03750 | 6.69517 | 5.01238 |
| AT1G49480 | 6.69453 | 19.8486 |
| AT4G39280 | 6.69381 | 21.1227 |
| AT1G72830 | 6.69314 | 7.56817 |
| AT4G35335 | 6.69301 | 20.2726 |
| AT5G12940 | 6.68961 | 19.4961 |
| AT3G20590 | 6.68929 | 3.65022 |
| AT3G19650 | 6.68097 | 13.0431 |
| AT5G20045 | 6.68029 | 47.5378 |
| AT4G35470 | 6.67958 | 9.70119 |
| AT4G18550 | 6.67936 | 2.43019 |
| AT5G23860 | 6.67904 | 39.7529 |
| AT2G44740 | 6.67845 | 22.9347 |
| AT4G20280 | 6.67734 | 14.5368 |
| AT1G23550 | 6.67282 | 1.89675 |
| AT4G31930 | 6.66797 | 8.11237 |
| AT3G22190 | 6.66705 | 9.14597 |
| AT5G27120 | 6.66396 | 48.9089 |
| AT2G02680 | 6.66328 | 0.956055 |
| AT5G21060 | 6.66095 | 12.3886 |
| AT1G63690 | 6.66075 | 22.1752 |
| AT5G47970 | 6.65784 | 7.71818 |
| AT3G22840 | 6.65427 | 4.70006 |
| AT3G20650 | 6.65185 | 13.452 |
| AT3G07180 | 6.65089 | 19.3719 |
| AT3G16770 | 6.6431 | 7.46702 |
| AT3G52390 | 6.64277 | 13.5201 |
| AT2G44270 | 6.63921 | 15.415 |
| AT5G27927 | 6.63672 | 0.237799 |
| AT4G34140 | 6.63565 | 7.26609 |
| AT3G09250 | 6.63252 | 4.19998 |
| AT5G54600 | 6.63221 | 10.7723 |
| AT5G25170 | 6.6304 | 13.0823 |
| AT3G02890 | 6.62773 | 16.771 |
| AT5G13390 | 6.62751 | 12.3636 |
| AT3G10130 | 6.62735 | 6.5473 |
| AT4G02715 | 6.62545 | 28.6751 |
| AT3G55900 | 6.62419 | 0.856611 |
| AT4G11300 | 6.62397 | 1.12289 |
| AT5G05710 | 6.62271 | 3.47109 |
| AT5G11200 | 6.6205 | 20.4477 |
| AT3G11490 | 6.61927 | 7.21827 |
| AT2G34050 | 6.61885 | 11.5946 |
| AT1G70900 | 6.61685 | 10.9186 |
| AT5G66052 | 6.61602 | 4.67703 |
| AT2G38152 | 6.61408 | 0.0206828 |
| AT3G24093 | 6.6084 | 2.44773 |
| AT2G30700 | 6.60628 | 9.1435 |
| AT3G23530 | 6.60469 | 4.55566 |
| AT3G13070 | 6.60092 | 15.3751 |
| AT4G34990 | 6.59972 | 5.04909 |
| AT3G66658 | 6.59909 | 14.2419 |
| AT5G07810 | 6.59539 | 5.02365 |
| AT1G29770 | 6.59497 | 3.34254 |
| AT5G66540 | 6.59445 | 20.4849 |
| AT5G22310 | 6.59357 | 3.99215 |
| AT1G70520 | 6.59079 | 3.21588 |
| AT5G01840 | 6.58961 | 1.08844 |
| AT3G50660 | 6.58274 | 3.90454 |
| AT4G16510 | 6.58207 | 8.65746 |
| AT5G11510 | 6.58138 | 7.05686 |
| AT1G55265 | 6.5778 | 10.1284 |
| AT1G14040 | 6.57759 | 8.33081 |
| AT5G17930 | 6.57217 | 13.2756 |
| AT1G01770 | 6.56889 | 2.98304 |
| AT1G76360 | 6.56714 | 2.82015 |
| AT2G42360 | 6.56444 | 2.27987 |
| AT4G33620 | 6.56339 | 6.62427 |
| AT5G57520 | 6.56333 | 0.0785694 |
| AT3G12100 | 6.56182 | 7.97925 |
| AT2G33435 | 6.56139 | 7.07712 |
| AT5G14690 | 6.56093 | 6.34209 |
| AT3G01920 | 6.55908 | 5.20238 |
| AT3G14740 | 6.55806 | 5.95229 |
| AT2G40850 | 6.55781 | 2.88558 |
| AT1G80790 | 6.55661 | 9.7469 |
| AT1G05570 | 6.55656 | 4.55459 |
| AT5G14430 | 6.55618 | 20.552 |
| AT4G01590 | 6.55614 | 17.6147 |
| AT5G04260 | 6.55569 | 8.09924 |
| AT3G11945 | 6.55401 | 14.4127 |
| AT5G60760 | 6.55334 | 7.36202 |
| AT2G35230 | 6.55228 | 6.36827 |
| AT1G69587 | 6.55182 | 3.11604 |
| AT4G04450 | 6.54948 | 2.03994 |
| AT2G04360 | 6.54207 | 7.05113 |
| AT2G19210 | 6.53921 | 0.00562177 |
| AT1G02390 | 6.53419 | 16.5073 |
| AT1G12650 | 6.53307 | 23.0751 |
| AT1G50750 | 6.53135 | 2.96311 |
| AT5G66180 | 6.52295 | 11.1646 |
| AT5G62430 | 6.52183 | 6.38139 |
| AT4G04460 | 6.52072 | 33.043 |
| AT5G41970 | 6.52024 | 25.1861 |
| AT4G17310 | 6.518 | 8.09186 |
| AT1G69830 | 6.51576 | 17.7192 |
| AT2G32300 | 6.51166 | 8.61053 |
| AT5G09450 | 6.50919 | 11.3925 |
| AT4G09620 | 6.50851 | 9.70213 |
| AT5G19950 | 6.50673 | 7.61218 |
| AT1G34020 | 6.50661 | 11.9187 |
| AT2G40750 | 6.50533 | 2.69219 |
| AT5G48520 | 6.50485 | 8.81894 |
| AT2G01505 | 6.50213 | 6.47491 |
| AT4G36650 | 6.50117 | 10.2415 |
| AT4G08950 | 6.49839 | 6.62349 |
| AT4G22305 | 6.4983 | 5.50328 |
| AT1G36060 | 6.49627 | 37.8333 |
| AT1G55180 | 6.49536 | 3.88256 |
| AT3G50845 | 6.49132 | 6.05613 |
| AT5G66290 | 6.48845 | 10.9075 |
| AT4G15260 | 6.48784 | 10.9618 |
| AT4G02360 | 6.48004 | 1.95291 |
| AT2G26200 | 6.48001 | 12.2231 |
| AT4G35910 | 6.47287 | 10.4793 |
| AT1G53460 | 6.4725 | 10.9606 |
| AT1G21651 | 6.46427 | 2.95012 |
| AT5G52820 | 6.46384 | 26.7773 |
| AT4G22540 | 6.45631 | 14.138 |
| AT5G62150 | 6.45428 | 8.34794 |
| AT1G67105 | 6.45057 | 0.377261 |
| AT1G20270 | 6.44944 | 14.8983 |
| AT1G73720 | 6.44926 | 15.4112 |
| AT3G56370 | 6.44612 | 12.6733 |
| AT1G51610 | 6.4459 | 5.41939 |
| AT4G22580 | 6.43919 | 2.89959 |
| AT4G30150 | 6.4386 | 6.55623 |
| AT3G24040 | 6.43732 | 11.1613 |
| AT1G55240 | 6.43615 | 27.7914 |
| AT4G13050 | 6.43423 | 20.5107 |
| AT3G52480 | 6.43136 | 3.29378 |
| AT4G04930 | 6.43093 | 0.424232 |
| AT2G06990 | 6.43054 | 15.9969 |
| AT3G51270 | 6.42909 | 25.829 |
| AT1G44800 | 6.42503 | 2.48449 |
| AT3G07140 | 6.42471 | 9.01292 |
| AT1G70640 | 6.42372 | 1.57584 |
| AT1G30860 | 6.42225 | 0.883213 |
| AT1G54730 | 6.41857 | 9.89929 |
| AT3G46760 | 6.41775 | 0.0411991 |
| AT4G31040 | 6.41289 | 10.6564 |
| AT1G11475 | 6.40857 | 38.4659 |
| AT4G27760 | 6.40566 | 12.5566 |
| AT4G34920 | 6.40281 | 1.65538 |
| AT5G22780 | 6.4028 | 5.65317 |
| AT5G04670 | 6.40239 | 7.82543 |
| AT5G18960 | 6.40139 | 9.56457 |
| AT3G15390 | 6.40004 | 15.9438 |
| AT2G21830 | 6.39639 | 1.96239 |
| AT1G67470 | 6.39511 | 1.37422 |
| AT1G42440 | 6.39196 | 15.6354 |
| AT3G60530 | 6.3891 | 20.7078 |
| AT3G44850 | 6.38778 | 3.71713 |
| AT4G00070 | 6.38288 | 2.13335 |
| AT3G05630 | 6.38131 | 8.47896 |
| AT4G22840 | 6.37893 | 4.2303 |
| AT1G30460 | 6.37405 | 8.64007 |
| AT1G15260 | 6.37164 | 2.58569 |
| AT5G66380 | 6.3709 | 6.4848 |
| AT1G74800 | 6.37012 | 10.0457 |
| AT1G10320 | 6.36985 | 8.86218 |
| AT4G00590 | 6.36488 | 5.08594 |
| AT2G04190 | 6.36444 | 0.0560017 |
| AT1G68020 | 6.36273 | 12.7054 |
| AT1G76300 | 6.36221 | 23.8579 |
| AT5G18260 | 6.36133 | 6.51966 |
| AT5G04710 | 6.36077 | 9.84683 |
| AT1G48145 | 6.35839 | 0.558326 |
| AT1G61380 | 6.35608 | 12.2535 |
| AT3G12345 | 6.35578 | 5.33944 |
| AT3G27070 | 6.35289 | 0.554656 |
| AT2G30000 | 6.35067 | 17.1306 |
| AT1G16025 | 6.34913 | 0.977968 |
| AT5G63550 | 6.3476 | 13.8526 |
| AT2G29410 | 6.34742 | 2.31671 |
| AT5G26300 | 6.34717 | 5.10201 |
| AT1G06730 | 6.34591 | 10.4186 |
| AT5G03990 | 6.34574 | 9.88561 |
| AT1G76710 | 6.34087 | 11.6913 |
| AT5G18790 | 6.33593 | 18.6734 |
| AT4G23870 | 6.33461 | 0.952756 |
| AT3G63240 | 6.32874 | 5.02919 |
| AT5G63670 | 6.32718 | 14.3755 |
| AT1G78810 | 6.32595 | 4.90161 |
| AT4G25150 | 6.3256 | 1.97989 |
| AT3G50410 | 6.32493 | 3.2893 |
| AT1G11430 | 6.32139 | 12.7103 |
| AT5G61990 | 6.31743 | 6.2356 |
| AT3G16920 | 6.31503 | 2.41109 |
| AT4G21460 | 6.31498 | 8.99008 |
| AT4G33740 | 6.31376 | 6.40332 |
| AT5G35330 | 6.31301 | 7.05301 |
| AT2G27920 | 6.31225 | 1.92692 |
| AT2G36990 | 6.31034 | 3.62144 |
| AT3G51860 | 6.31005 | 0.730419 |
| AT3G11040 | 6.30615 | 4.19319 |
| AT3G12380 | 6.30593 | 9.02422 |
| AT4G12510 | 6.30432 | 0.330729 |
| AT2G23420 | 6.30284 | 12.5648 |
| AT2G35780 | 6.30264 | 16.3834 |
| AT5G50175 | 6.30201 | 41.0337 |
| AT1G47270 | 6.30154 | 4.33473 |
| AT5G56650 | 6.30147 | 4.70379 |
| AT3G13062 | 6.30127 | 7.89832 |
| AT1G78120 | 6.29958 | 2.34687 |
| AT2G38810 | 6.29887 | 33.7035 |
| AT1G64640 | 6.29542 | 10.8008 |
| AT1G22310 | 6.29221 | 14.0126 |
| AT3G11280 | 6.28956 | 2.47729 |
| AT3G19790 | 6.28916 | 13.5564 |
| AT1G18660 | 6.28728 | 9.06938 |
| AT4G31240 | 6.28486 | 2.68233 |
| AT5G11030 | 6.28472 | 10.3675 |
| AT4G37940 | 6.2809 | 10.7295 |
| AT3G60380 | 6.27811 | 11.8888 |
| AT1G80030 | 6.27686 | 6.48367 |
| AT5G50350 | 6.27616 | 10.1602 |
| AT3G18270 | 6.27158 | 6.35995 |
| AT2G34920 | 6.27147 | 1.54983 |
| AT3G25990 | 6.26975 | 3.19823 |
| AT5G09270 | 6.26951 | 18.5964 |
| AT4G32630 | 6.26738 | 5.37113 |
| AT5G35970 | 6.26631 | 7.17274 |
| AT2G17600 | 6.26607 | 0.102578 |
| AT3G25290 | 6.26604 | 12.4288 |
| AT4G28760 | 6.26568 | 15.6287 |
| AT2G46590 | 6.26346 | 7.78156 |
| AT1G03687 | 6.26334 | 6.86848 |
| AT5G01950 | 6.26277 | 9.45407 |
| AT1G17510 | 6.26171 | 9.38087 |
| AT2G30390 | 6.25887 | 2.83771 |
| AT4G37480 | 6.25676 | 9.58957 |
| AT1G07476 | 6.25459 | 3.20235 |
| AT5G10060 | 6.25258 | 14.5871 |
| AT1G02280 | 6.25122 | 10.5258 |
| AT3G26680 | 6.25064 | 6.62847 |
| AT2G24020 | 6.24986 | 12.4347 |
| AT1G27190 | 6.24905 | 11.3884 |
| AT5G59440 | 6.24457 | 9.32743 |
| AT1G53700 | 6.24151 | 5.4114 |
| AT2G34390 | 6.24126 | 3.98229 |
| AT2G03360 | 6.24031 | 0.112152 |
| AT1G23120 | 6.23979 | 3.4768 |
| AT2G38680 | 6.2262 | 11.2491 |
| AT1G21100 | 6.22541 | 1.21403 |
| AT5G49640 | 6.21544 | 19.3124 |
| AT3G17590 | 6.21493 | 19.3568 |
| AT5G27200 | 6.21394 | 5.0321 |
| AT4G00060 | 6.21352 | 6.55806 |
| AT4G01680 | 6.21071 | 2.28051 |
| AT1G55325 | 6.20551 | 5.63646 |
| AT3G11540 | 6.20423 | 13.5684 |
| AT2G41070 | 6.20262 | 13.2231 |
| AT4G02090 | 6.20044 | 3.50078 |
| AT2G46220 | 6.19924 | 3.92578 |
| AT5G52420 | 6.19539 | 3.98491 |
| AT1G27470 | 6.19412 | 14.8108 |
| AT2G43420 | 6.19401 | 7.83877 |
| AT2G25220 | 6.18899 | 6.32276 |
| AT5G43710 | 6.18886 | 9.70351 |
| AT5G24300 | 6.18859 | 9.26414 |
| AT1G07650 | 6.18471 | 5.33055 |
| AT3G27350 | 6.18452 | 11.6714 |
| AT5G15581 | 6.18389 | 15.6343 |
| AT5G62760 | 6.17963 | 5.94501 |
| AT1G75190 | 6.17897 | 5.7374 |
| AT2G19390 | 6.17851 | 4.47497 |
| AT5G57980 | 6.17837 | 7.42861 |
| AT1G11750 | 6.17701 | 18.698 |
| AT3G04490 | 6.17558 | 8.06668 |
| AT4G04920 | 6.17411 | 8.65776 |
| AT3G19510 | 6.17354 | 6.70822 |
| AT5G52670 | 6.17343 | 0.811487 |
| AT1G45015 | 6.16754 | 2.04147 |
| AT1G48270 | 6.16718 | 6.57701 |
| AT3G15970 | 6.16641 | 11.6047 |
| AT4G31820 | 6.16432 | 18.3662 |
| AT2G22800 | 6.16267 | 0.0906436 |
| AT3G16740 | 6.16073 | 8.0567 |
| AT5G40010 | 6.15818 | 0.624946 |
| AT3G02515 | 6.15723 | 2.73987 |
| AT3G02710 | 6.15577 | 14.5075 |
| AT1G49170 | 6.15508 | 5.04834 |
| AT1G63590 | 6.15313 | 0.0449508 |
| AT2G46750 | 6.14732 | 0.458944 |
| AT1G13510 | 6.14524 | 0.750605 |
| AT3G03950 | 6.14331 | 17.8056 |
| AT4G15770 | 6.14284 | 26.0458 |
| AT3G09360 | 6.13772 | 9.2337 |
| AT3G01070 | 6.13623 | 24.4643 |
| AT3G49080 | 6.13118 | 25.4858 |
| AT4G04870 | 6.12972 | 9.33995 |
| AT4G18360 | 6.12924 | 12.6187 |
| AT5G65170 | 6.12471 | 14.5843 |
| AT5G57140 | 6.12466 | 4.16149 |
| AT1G10360 | 6.12411 | 1.45348 |
| AT2G20550 | 6.12369 | 3.34677 |
| AT2G28940 | 6.12234 | 6.49618 |
| AT3G28970 | 6.1216 | 9.27914 |
| AT3G18370 | 6.12156 | 10.4086 |
| AT5G43040 | 6.12025 | 1.62447 |
| AT4G27580 | 6.1132 | 4.13979 |
| AT5G38300 | 6.11225 | 3.12868 |
| AT5G09860 | 6.11155 | 10.1274 |
| AT3G04230 | 6.10819 | 4.89197 |
| AT5G10460 | 6.10732 | 14.7296 |
| AT1G71350 | 6.10615 | 13.9523 |
| AT5G05470 | 6.10543 | 12.4958 |
| AT5G01530 | 6.10406 | 2.01554 |
| AT5G21160 | 6.10208 | 15.7342 |
| AT1G48320 | 6.10186 | 2.49154 |
| AT3G26470 | 6.09926 | 0.945453 |
| AT1G26660 | 6.09705 | 16.1617 |
| AT1G13160 | 6.09614 | 20.0816 |
| AT3G09670 | 6.09578 | 7.01432 |
| AT2G39040 | 6.09021 | 16.8776 |
| AT5G64840 | 6.08896 | 13.894 |
| AT2G45830 | 6.08808 | 4.352 |
| AT5G53680 | 6.08483 | 2.29262 |
| AT1G73875 | 6.08408 | 7.43086 |
| AT4G31380 | 6.08319 | 0.683178 |
| AT5G54970 | 6.08011 | 28.3772 |
| AT2G17033 | 6.07903 | 5.29199 |
| AT5G63950 | 6.07417 | 11.2631 |
| AT1G29900 | 6.07142 | 21.3133 |
| AT2G28720 | 6.06795 | 15.5287 |
| AT1G09010 | 6.06587 | 13.9785 |
| AT5G16230 | 6.06413 | 24.1573 |
| AT5G41590 | 6.06318 | 7.47089 |
| AT3G19720 | 6.05958 | 11.1658 |
| AT1G23410 | 6.05838 | 44.899 |
| AT5G06290 | 6.05799 | 8.10562 |
| AT4G20400 | 6.05784 | 15.8242 |
| AT5G48880 | 6.05543 | 0.779137 |
| AT1G08592 | 6.05049 | 8.43554 |
| AT4G22720 | 6.0408 | 10.6603 |
| AT1G75335 | 6.04011 | 7.43473 |
| AT1G73240 | 6.03896 | 7.67252 |
| AT3G08600 | 6.03633 | 5.4362 |
| AT2G02080 | 6.03437 | 1.75353 |
| AT4G29430 | 6.03301 | 19.4229 |
| AT1G54090 | 6.03248 | 9.51978 |
| AT1G23100 | 6.03204 | 26.0618 |
| AT1G25500 | 6.03181 | 5.16439 |
| AT1G08580 | 6.02861 | 31.5528 |
| AT2G36670 | 6.02659 | 8.16839 |
| AT2G01070 | 6.02547 | 11.7083 |
| AT5G57370 | 6.02244 | 8.47371 |
| AT1G43900 | 6.01987 | 8.44141 |
| AT2G01290 | 6.01887 | 8.14072 |
| AT1G32560 | 6.01758 | 17.9405 |
| AT1G61550 | 6.01644 | 1.29779 |
| AT5G42500 | 6.0099 | 1.35197 |
| AT2G41820 | 6.00771 | 5.10189 |
| AT2G20950 | 6.00665 | 1.06215 |
| AT4G19750 | 6.00647 | 0.0153971 |
| AT2G26900 | 6.00257 | 14.2722 |
| AT3G11270 | 6.0016 | 10.6022 |
| AT2G05520 | 6.00098 | 8.5114 |
| AT1G64590 | 6.00034 | 1.2144 |
| AT1G56290 | 5.99651 | 6.79188 |
| AT2G03430 | 5.99566 | 11.1237 |
| AT2G06210 | 5.99546 | 13.817 |
| AT1G33490 | 5.99496 | 13.4635 |
| AT5G22770 | 5.99163 | 4.06874 |
| AT3G17470 | 5.99059 | 4.55544 |
| AT4G29510 | 5.9904 | 18.6334 |
| AT1G32580 | 5.99031 | 25.9919 |
| AT1G33810 | 5.98972 | 15.4725 |
| AT4G36840 | 5.98835 | 3.36852 |
| AT1G08060 | 5.98794 | 7.09119 |
| AT1G09176 | 5.98612 | 0.323628 |
| AT1G19920 | 5.98343 | 33.7967 |
| AT4G39300 | 5.98052 | 24.3042 |
| AT2G19810 | 5.97672 | 8.12534 |
| AT3G08680 | 5.96838 | 25.2811 |
| AT1G21140 | 5.96755 | 2.02818 |
| AT3G04330 | 5.96559 | 53.8403 |
| AT4G28230 | 5.96506 | 4.90914 |
| AT2G19470 | 5.96176 | 17.0669 |
| AT2G31210 | 5.95861 | 1.31186 |
| AT1G13170 | 5.95699 | 18.5221 |
| AT1G04420 | 5.95418 | 3.77768 |
| AT2G45880 | 5.9513 | 8.29817 |
| AT1G80520 | 5.94589 | 11.7973 |
| AT1G26740 | 5.94432 | 18.3118 |
| AT2G46495 | 5.94194 | 2.28594 |
| AT4G32750 | 5.93917 | 4.9865 |
| AT2G46690 | 5.93719 | 0.202591 |
| AT4G15090 | 5.93621 | 9.12639 |
| AT2G32280 | 5.93155 | 6.30854 |
| AT1G51402 | 5.92766 | 1.55717 |
| AT1G27440 | 5.92571 | 5.38668 |
| AT1G32070 | 5.9249 | 6.78745 |
| AT5G07040 | 5.92463 | 11.1809 |
| AT2G22490 | 5.92442 | 12.0739 |
| AT1G02220 | 5.91975 | 1.35926 |
| AT5G13300 | 5.91927 | 13.1269 |
| AT2G35795 | 5.91848 | 7.14382 |
| AT3G09330 | 5.91769 | 0.6641 |
| AT5G51340 | 5.9158 | 10.3172 |
| AT4G13030 | 5.91373 | 3.60009 |
| AT4G36140 | 5.91223 | 7.39842 |
| AT5G53360 | 5.91119 | 3.28674 |
| AT5G53280 | 5.90487 | 8.55287 |
| AT2G30920 | 5.90132 | 6.91704 |
| AT5G05980 | 5.90056 | 17.7619 |
| AT5G10810 | 5.90038 | 15.47 |
| AT5G44200 | 5.89899 | 23.3287 |
| AT2G31750 | 5.89734 | 3.79904 |
| AT3G08980 | 5.89705 | 9.65045 |
| AT4G05018 | 5.8949 | 1.68969 |
| AT1G13750 | 5.89288 | 7.00536 |
| AT4G35920 | 5.89122 | 9.25571 |
| AT2G04110 | 5.89033 | 4.83407 |
| AT1G80480 | 5.88941 | 9.05501 |
| AT1G55915 | 5.8866 | 5.13892 |
| AT5G45113 | 5.88293 | 0.820934 |
| AT3G63350 | 5.88179 | 14.5733 |
| AT1G64930 | 5.88106 | 0.273518 |
| AT3G14225 | 5.87977 | 2.55417 |
| AT4G13370 | 5.87965 | 4.59816 |
| AT3G57610 | 5.86917 | 25.6269 |
| AT1G02680 | 5.86885 | 9.17573 |
| AT2G27775 | 5.86692 | 17.2547 |
| AT3G05905 | 5.86013 | 14.5747 |
| AT5G01390 | 5.86003 | 9.39015 |
| AT2G38530 | 5.8554 | 4.86543 |
| AT1G15390 | 5.85003 | 7.05605 |
| AT1G60790 | 5.84953 | 13.231 |
| AT1G68650 | 5.84765 | 12.1385 |
| AT5G40260 | 5.84408 | 0.464072 |
| AT5G56500 | 5.84215 | 29.8172 |
| AT5G10050 | 5.84102 | 6.17033 |
| AT5G34940 | 5.83983 | 4.93039 |
| AT1G09320 | 5.83726 | 7.21825 |
| AT1G42470 | 5.83702 | 8.9787 |
| AT1G19010 | 5.83611 | 4.23726 |
| AT1G62810 | 5.83331 | 11.3414 |
| AT4G10170 | 5.83167 | 5.33171 |
| AT4G10940 | 5.82908 | 6.45142 |
| AT1G16905 | 5.82876 | 111.883 |
| AT1G26360 | 5.82446 | 0.627281 |
| AT1G10830 | 5.8236 | 7.71328 |
| AT4G03250 | 5.8236 | 4.50035 |
| AT5G61540 | 5.82192 | 2.88324 |
| AT3G12670 | 5.82069 | 24.6266 |
| AT5G19750 | 5.81557 | 19.422 |
| AT5G16480 | 5.81322 | 9.08104 |
| AT5G14200 | 5.81261 | 14.5749 |
| AT5G06210 | 5.80697 | 11.415 |
| AT2G42150 | 5.80678 | 2.52238 |
| AT1G79500 | 5.80516 | 21.9765 |
| AT1G59890 | 5.79914 | 14.7392 |
| AT1G54420 | 5.79672 | 0.410942 |
| AT3G01740 | 5.79613 | 24.2543 |
| AT2G04340 | 5.79572 | 13.3888 |
| AT2G43710 | 5.79564 | 19.4274 |
| AT1G79960 | 5.79332 | 5.96503 |
| AT1G13940 | 5.79251 | 7.56232 |
| AT3G23310 | 5.79162 | 12.3673 |
| AT1G24095 | 5.78461 | 7.43683 |
| AT5G64460 | 5.78377 | 14.058 |
| AT1G08960 | 5.78312 | 11.7426 |
| AT2G26690 | 5.78299 | 5.74339 |
| AT2G26030 | 5.78271 | 4.79317 |
| AT3G63390 | 5.78119 | 4.00427 |
| AT4G36380 | 5.77683 | 2.06879 |
| AT5G65158 | 5.77498 | 0.169588 |
| AT1G08600 | 5.7739 | 11.2358 |
| AT5G26040 | 5.77234 | 3.61386 |
| AT2G35480 | 5.76895 | 4.1427 |
| AT2G35390 | 5.76856 | 8.86603 |
| AT1G11630 | 5.76561 | 6.85026 |
| AT1G18840 | 5.76536 | 24.4687 |
| AT5G64150 | 5.76426 | 1.86044 |
| AT5G20810 | 5.76356 | 8.53011 |
| AT1G57870 | 5.76152 | 7.47616 |
| AT1G35210 | 5.76013 | 5.09745 |
| AT5G65060 | 5.7583 | 7.2958 |
| AT1G55270 | 5.75698 | 10.8318 |
| AT4G26190 | 5.75576 | 14.8204 |
| AT2G35410 | 5.75085 | 6.40217 |
| AT2G22570 | 5.75055 | 8.57159 |
| AT3G19250 | 5.74596 | 1.50749 |
| AT5G44910 | 5.74594 | 2.39732 |
| AT4G23900 | 5.74255 | 2.84295 |
| AT5G02180 | 5.73906 | 10.1461 |
| AT2G34170 | 5.73758 | 10.0862 |
| AT4G04740 | 5.73344 | 3.24582 |
| AT1G19480 | 5.73237 | 3.43729 |
| AT1G11360 | 5.73231 | 11.9251 |
| AT2G20440 | 5.73114 | 4.3877 |
| AT2G42060 | 5.72991 | 1.22976 |
| AT4G09500 | 5.72847 | 0.215031 |
| AT3G10120 | 5.7269 | 1.56557 |
| AT5G03415 | 5.7257 | 7.89336 |
| AT5G49000 | 5.72446 | 5.93036 |
| AT1G03420.1 | 5.72198 | 7.8595 |
| AT2G20240 | 5.7163 | 5.42032 |
| AT3G44590 | 5.71557 | 20.4024 |
| AT1G65000 | 5.7127 | 6.25291 |
| AT4G00955 | 5.71203 | 1.02502 |
| AT5G27820 | 5.7114 | 26.2771 |
| AT1G17820 | 5.71052 | 3.79489 |
| AT2G40540 | 5.71036 | 7.5466 |
| AT4G19350 | 5.70793 | 18.8241 |
| AT3G16180 | 5.70065 | 18.5303 |
| AT3G18930 | 5.6968 | 4.69021 |
| AT2G22830 | 5.69585 | 4.02697 |
| AT2G22240 | 5.69508 | 4.08692 |
| AT5G52540 | 5.69421 | 5.61564 |
| AT2G16570 | 5.69336 | 10.6617 |
| AT5G59460 | 5.69262 | 12.7316 |
| AT5G01250 | 5.6873 | 0.227479 |
| AT3G17680 | 5.68718 | 10.6913 |
| AT2G25090 | 5.68718 | 0.0348873 |
| AT3G51940 | 5.68662 | 5.53974 |
| AT1G72852 | 5.68307 | 3.62193 |
| AT5G41140 | 5.68093 | 6.67084 |
| AT1G55750 | 5.67994 | 6.88085 |
| AT3G14220 | 5.67725 | 11.1322 |
| AT4G28150 | 5.67665 | 0.922664 |
| AT5G08470 | 5.67573 | 7.74131 |
| AT1G56460 | 5.67509 | 11.2373 |
| AT2G23690 | 5.67344 | 2.88428 |
| AT3G01480 | 5.67133 | 5.25379 |
| AT3G57930 | 5.67103 | 40.0047 |
| AT2G26640 | 5.67001 | 6.24985 |
| AT1G08300 | 5.66911 | 4.25003 |
| AT3G44620 | 5.66116 | 5.0748 |
| AT3G19900 | 5.65991 | 8.95587 |
| AT4G16130 | 5.6597 | 7.41594 |
| AT1G02230 | 5.65829 | 3.39468 |
| AT1G01200 | 5.6553 | 2.6517 |
| AT5G53060 | 5.65448 | 9.59644 |
| AT5G53130 | 5.65141 | 11.2734 |
| AT5G24630 | 5.64937 | 10.0377 |
| AT4G10350 | 5.64203 | 35.5799 |
| AT5G25210 | 5.64121 | 6.4881 |
| AT3G56210 | 5.64053 | 8.57559 |
| AT5G56230 | 5.63694 | 1.28601 |
| AT3G06680 | 5.63581 | 20.7758 |
| AT3G60910 | 5.63472 | 7.84728 |
| AT1G29395 | 5.63284 | 0.402593 |
| AT5G53620 | 5.6328 | 18.4521 |
| AT3G29010 | 5.63011 | 3.0806 |
| AT5G40550 | 5.62974 | 7.50047 |
| AT4G33660 | 5.62891 | 8.02076 |
| AT4G20170 | 5.61991 | 11.3187 |
| AT5G10850 | 5.6182 | 2.37869 |
| AT2G33400 | 5.61804 | 7.00059 |
| AT4G37320 | 5.61526 | 6.00603 |
| AT1G11592 | 5.61489 | 2.73391 |
| AT5G37310 | 5.61213 | 7.7399 |
| AT3G54010 | 5.61024 | 12.7598 |
| AT4G05440 | 5.60941 | 9.0937 |
| AT3G13780 | 5.60525 | 9.12977 |
| AT2G15730 | 5.60347 | 15.0951 |
| AT5G53090 | 5.60156 | 13.8399 |
| AT5G59610 | 5.60001 | 6.33811 |
| AT1G14990 | 5.59878 | 9.68285 |
| AT4G21437 | 5.59868 | 0.827062 |
| AT1G35470 | 5.5985 | 7.49887 |
| AT5G64420 | 5.59849 | 15.3409 |
| AT5G41860 | 5.59707 | 2.28214 |
| AT4G12670 | 5.59655 | 7.78989 |
| AT4G38490 | 5.59399 | 6.65763 |
| AT5G56420 | 5.59176 | 6.03869 |
| AT3G50290 | 5.59056 | 1.85491 |
| AT3G20010 | 5.58072 | 5.74796 |
| AT1G20480 | 5.57906 | 2.85574 |
| AT1G67880 | 5.57215 | 3.96469 |
| AT2G15370 | 5.57187 | 0.438409 |
| AT3G03550 | 5.57111 | 2.53086 |
| AT4G38840 | 5.57057 | 0.0709888 |
| AT5G55170 | 5.57049 | 1.88622 |
| AT4G12130 | 5.56841 | 7.23902 |
| AT1G67340 | 5.56801 | 9.65157 |
| AT1G71240 | 5.56736 | 4.48456 |
| AT3G18940 | 5.56705 | 24.2044 |
| AT5G27710 | 5.56483 | 3.70918 |
| AT4G34480 | 5.56393 | 9.73337 |
| AT1G73020 | 5.5639 | 7.12914 |
| AT5G59470 | 5.56376 | 5.76313 |
| AT3G02065 | 5.56048 | 7.35256 |
| AT5G23110 | 5.56048 | 3.47432 |
| AT5G40530 | 5.56029 | 17.9816 |
| AT5G04510 | 5.55641 | 16.1133 |
| AT4G14490 | 5.55617 | 6.6695 |
| AT5G65940 | 5.55615 | 12.4488 |
| AT3G06480 | 5.556 | 7.04298 |
| AT5G64330 | 5.55175 | 14.8822 |
| AT1G75160 | 5.55033 | 0.0108437 |
| AT1G22960 | 5.54841 | 6.20911 |
| AT3G27290 | 5.54584 | 3.02974 |
| AT1G55900 | 5.54038 | 16.6537 |
| AT4G19830 | 5.53975 | 2.89475 |
| AT3G55640 | 5.53945 | 9.56559 |
| AT4G00490 | 5.539 | 17.9456 |
| AT3G16860 | 5.53122 | 2.31431 |
| AT5G36890 | 5.52943 | 20.3107 |
| AT4G20340 | 5.5289 | 9.75553 |
| AT3G15090 | 5.52808 | 8.6201 |
| AT5G08390 | 5.52801 | 11.4516 |
| AT3G48860 | 5.52209 | 13.4759 |
| AT3G27930 | 5.52117 | 5.11179 |
| AT3G27416 | 5.5208 | 11.5135 |
| AT2G31990 | 5.51793 | 3.61907 |
| AT5G14340 | 5.51768 | 1.87282 |
| AT4G20730.1 | 5.51128 | 0.397336 |
| AT1G74490 | 5.5097 | 22.7952 |
| AT1G33290 | 5.50635 | 27.6906 |
| AT5G44260 | 5.50629 | 0.696468 |
| AT1G77670 | 5.50576 | 5.70177 |
| AT1G32760 | 5.50546 | 11.7163 |
| AT3G04970 | 5.50386 | 4.99133 |
| AT5G06470 | 5.50304 | 2.45098 |
| AT1G73650 | 5.50293 | 6.60216 |
| AT2G39520 | 5.50239 | 0.960597 |
| AT5G09230 | 5.50096 | 6.24071 |
| AT2G38060 | 5.49993 | 0.696335 |
| AT5G52370 | 5.49772 | 15.4993 |
| AT4G02430 | 5.49554 | 8.17001 |
| AT2G11290 | 5.49121 | 0.413844 |
| AT4G08930 | 5.49111 | 0.548858 |
| AT4G26260 | 5.49002 | 0.213983 |
| AT2G39010 | 5.48926 | 6.51602 |
| AT5G02620 | 5.48844 | 4.79652 |
| AT1G76410 | 5.48829 | 2.19648 |
| AT4G38100 | 5.48796 | 11.7755 |
| AT1G31420 | 5.47304 | 11.4884 |
| AT3G18210 | 5.472 | 10.8114 |
| AT2G03060 | 5.47125 | 6.15419 |
| AT5G07970 | 5.47083 | 8.11022 |
| AT5G24740 | 5.46852 | 3.96178 |
| AT1G55350 | 5.46657 | 10.8811 |
| AT5G15940 | 5.46488 | 29.2617 |
| AT1G52500 | 5.46146 | 5.33072 |
| AT2G20190 | 5.45915 | 15.2765 |
| AT3G15357 | 5.45866 | 9.89445 |
| AT1G43675 | 5.45833 | 9.44723 |
| AT1G10660 | 5.45803 | 7.19219 |
| AT4G38040 | 5.45789 | 17.9405 |
| AT5G22290 | 5.45464 | 7.02564 |
| AT1G58220 | 5.45358 | 5.78437 |
| AT1G22870 | 5.45316 | 6.35704 |
| AT5G27740 | 5.45257 | 13.6949 |
| AT4G33460 | 5.4523 | 5.75605 |
| AT5G19370 | 5.45173 | 13.239 |
| AT1G10570 | 5.45026 | 7.39598 |
| AT2G28510 | 5.44756 | 3.99171 |
| AT3G09310 | 5.44633 | 6.0855 |
| AT5G28290 | 5.4451 | 12.0523 |
| AT4G00420 | 5.44497 | 9.09453 |
| AT5G12130 | 5.43932 | 9.41943 |
| AT5G39410 | 5.4391 | 10.7315 |
| AT3G17850 | 5.43735 | 17.8292 |
| AT1G77930 | 5.43724 | 6.36162 |
| AT3G16100 | 5.43651 | 7.59421 |
| AT1G16150 | 5.42747 | 7.84611 |
| AT5G10690 | 5.42311 | 6.13695 |
| AT1G20880 | 5.42242 | 4.3391 |
| AT4G38570 | 5.42241 | 10.7293 |
| AT3G53750 | 5.42199 | 18.5682 |
| AT4G21390 | 5.41924 | 9.31957 |
| AT2G38660 | 5.41801 | 9.02489 |
| AT5G61190 | 5.4146 | 6.65273 |
| AT1G64790 | 5.41192 | 18.3146 |
| AT2G40330 | 5.41132 | 1.16989 |
| AT1G44608 | 5.41124 | 1.82879 |
| AT1G79740 | 5.41041 | 7.40765 |
| AT3G02370 | 5.41003 | 3.62734 |
| AT4G21090 | 5.40959 | 10.7668 |
| AT5G41400 | 5.40955 | 3.77485 |
| AT3G24080 | 5.40766 | 20.794 |
| AT5G57970 | 5.40462 | 3.35262 |
| AT1G61760 | 5.40258 | 0.25305 |
| AT1G02690 | 5.40101 | 16.9841 |
| AT2G23910 | 5.40046 | 1.56798 |
| AT1G55960 | 5.3965 | 7.28012 |
| AT5G20850 | 5.39619 | 9.56479 |
| AT1G51430 | 5.38891 | 4.39941 |
| AT1G04390 | 5.38719 | 5.77176 |
| AT5G10920 | 5.38532 | 18.7865 |
| AT2G27150 | 5.3846 | 8.01711 |
| AT3G17660 | 5.3823 | 2.27218 |
| AT1G73066 | 5.38214 | 2.68645 |
| AT4G25270 | 5.38194 | 8.21473 |
| AT4G00026 | 5.37983 | 12.6129 |
| AT5G42270 | 5.37935 | 9.52484 |
| AT3G01435 | 5.37933 | 5.83507 |
| AT1G77480 | 5.37886 | 12.7914 |
| AT2G40410 | 5.37873 | 12.5749 |
| AT5G05450 | 5.37698 | 12.2892 |
| AT3G17185 | 5.37654 | 2.28378 |
| AT3G15220 | 5.37402 | 8.20933 |
| AT2G32765 | 5.374 | 2.94596 |
| AT4G04760 | 5.37359 | 0.722476 |
| AT1G06960 | 5.37355 | 12.0503 |
| AT4G11390 | 5.36556 | 0.271666 |
| AT1G63740 | 5.36432 | 5.81725 |
| AT3G21350 | 5.36416 | 13.208 |
| AT5G53170 | 5.3627 | 12.5722 |
| AT5G03200 | 5.36233 | 5.47766 |
| AT1G69850 | 5.35793 | 20.7669 |
| AT1G68825 | 5.35079 | 3.29486 |
| AT3G16320 | 5.35074 | 4.35073 |
| AT3G53460 | 5.3491 | 17.2191 |
| AT5G27970 | 5.34704 | 10.5898 |
| AT2G21195 | 5.34361 | 9.71917 |
| AT4G04970 | 5.342 | 5.57863 |
| AT4G06744 | 5.34157 | 0.714639 |
| AT1G16230 | 5.34016 | 0.254393 |
| AT1G34180 | 5.34013 | 3.50914 |
| AT5G53290 | 5.33884 | 9.76717 |
| AT5G25590 | 5.33806 | 7.10343 |
| AT3G04890 | 5.33793 | 1.91836 |
| AT5G46470 | 5.33779 | 4.80884 |
| AT3G49700 | 5.33736 | 0.315157 |
| AT1G67900 | 5.33605 | 4.26059 |
| AT1G77280 | 5.33581 | 3.05384 |
| AT1G09157 | 5.3353 | 2.49911 |
| AT1G05910 | 5.33521 | 11.2236 |
| AT3G51680 | 5.33094 | 8.4507 |
| AT5G13480 | 5.32815 | 9.81279 |
| AT3G10480 | 5.32764 | 9.12707 |
| AT4G16765 | 5.3261 | 8.66506 |
| AT3G54350 | 5.32352 | 12.3575 |
| AT3G54420 | 5.32299 | 1.41238 |
| AT4G21100 | 5.32208 | 11.996 |
| AT5G66840 | 5.32105 | 6.69212 |
| AT5G23390 | 5.31875 | 6.48029 |
| AT5G64010 | 5.31867 | 3.87849 |
| AT1G56180 | 5.31678 | 9.032 |
| AT3G22900 | 5.31653 | 2.4433 |
| AT1G16420 | 5.31635 | 0.252483 |
| AT1G78290 | 5.3163 | 3.47796 |
| AT5G52120 | 5.31586 | 1.06545 |
| AT2G11520 | 5.31535 | 6.84562 |
| AT4G00110 | 5.31454 | 6.90974 |
| AT5G47760 | 5.31386 | 9.64523 |
| AT5G57850 | 5.31378 | 7.28396 |
| AT2G43470 | 5.31159 | 26.0554 |
| AT4G02725 | 5.3113 | 7.11919 |
| AT1G47565.1 | 5.31086 | 2.48128 |
| AT4G15500 | 5.306 | 1.42474 |
| AT1G34120 | 5.30546 | 5.35945 |
| AT1G18700 | 5.30333 | 13.1291 |
| AT2G02350 | 5.30203 | 3.72911 |
| AT1G34130 | 5.30153 | 18.0146 |
| AT4G08040 | 5.301 | 1.02531 |
| AT5G07630 | 5.29891 | 7.67422 |
| AT5G38550 | 5.29845 | 1.66839 |
| AT4G31150 | 5.29733 | 4.47504 |
| AT5G23880 | 5.28995 | 11.6248 |
| AT5G62700 | 5.28788 | 27.859 |
| AT1G01920 | 5.28697 | 9.65232 |
| AT2G47560 | 5.28696 | 1.04121 |
| AT5G05800 | 5.28516 | 11.1907 |
| AT3G06080 | 5.28383 | 5.60425 |
| AT3G07630 | 5.28132 | 8.43214 |
| AT2G34330 | 5.27881 | 0.257361 |
| AT2G41150 | 5.2783 | 11.151 |
| AT5G08730 | 5.27758 | 1.9497 |
| AT4G15563 | 5.27627 | 2.56909 |
| AT3G06440 | 5.27503 | 10.5898 |
| AT1G79410 | 5.27431 | 3.41643 |
| AT3G62080 | 5.27348 | 4.78479 |
| AT5G53815 | 5.27216 | 0.490348 |
| AT1G03600 | 5.2712 | 3.91349 |
| AT4G39720 | 5.26915 | 6.87582 |
| AT5G46570 | 5.26722 | 8.82638 |
| AT3G18640 | 5.2666 | 9.81401 |
| AT1G44790 | 5.26411 | 12.8448 |
| AT5G43770 | 5.26234 | 0.758655 |
| AT5G03310 | 5.25987 | 1.08408 |
| AT3G22275 | 5.25907 | 37.5873 |
| AT5G01470 | 5.25864 | 5.41567 |
| AT1G80290 | 5.25466 | 3.59728 |
| AT4G28370 | 5.25349 | 4.61915 |
| AT1G09860 | 5.25067 | 1.20818 |
| AT1G70130 | 5.24911 | 17.2984 |
| AT1G51700 | 5.24458 | 2.27691 |
| AT2G35750 | 5.24146 | 4.9995 |
| AT5G09800 | 5.2413 | 5.0505 |
| AT5G28750 | 5.2368 | 6.58134 |
| AT1G54160 | 5.23355 | 2.94591 |
| AT1G30420 | 5.23141 | 3.31796 |
| AT5G55130 | 5.22706 | 11.5477 |
| AT1G11055 | 5.22509 | 61.0575 |
| AT3G04750 | 5.22341 | 5.67067 |
| AT4G31590 | 5.22143 | 10.2722 |
| AT2G41350 | 5.22133 | 9.10117 |
| AT2G42070 | 5.22065 | 8.85825 |
| AT1G25375 | 5.21989 | 9.73995 |
| AT4G38880 | 5.21862 | 0.458217 |
| AT3G56590 | 5.21763 | 11.8925 |
| AT1G20300 | 5.21508 | 6.70127 |
| AT3G61350 | 5.21378 | 5.21813 |
| AT5G60210 | 5.2137 | 14.2015 |
| AT3G14800 | 5.21288 | 7.67366 |
| AT1G73280 | 5.21056 | 1.48513 |
| AT3G43700 | 5.20797 | 6.00535 |
| AT5G50300 | 5.20716 | 5.3816 |
| AT5G07780 | 5.2055 | 0.144088 |
| AT5G03490 | 5.20438 | 3.69125 |
| AT1G31970 | 5.20258 | 36.2868 |
| AT4G20000 | 5.20093 | 9.34755 |
| AT1G68552 | 5.19998 | 1.55195 |
| AT1G72440 | 5.1982 | 10.9248 |
| AT2G30590 | 5.19815 | 16.0556 |
| AT5G53990 | 5.19645 | 3.4842 |
| AT1G17830 | 5.19218 | 13.1824 |
| AT2G36240 | 5.19022 | 7.92481 |
| AT1G58350 | 5.18934 | 4.30045 |
| AT3G16340 | 5.18765 | 7.1897 |
| AT4G09180 | 5.18619 | 5.54252 |
| AT5G61130 | 5.18558 | 24.6176 |
| AT2G35320 | 5.18318 | 14.1892 |
| AT5G65350 | 5.18092 | 18.8021 |
| AT5G27950 | 5.1788 | 3.99091 |
| AT2G46920 | 5.17719 | 8.44166 |
| AT3G12180 | 5.1771 | 9.71483 |
| AT4G27380 | 5.17705 | 14.8867 |
| AT4G30630 | 5.17657 | 4.93161 |
| AT3G51410 | 5.1744 | 1.29844 |
| AT4G32770 | 5.17269 | 1.16746 |
| AT2G41000 | 5.17211 | 1.53224 |
| AT3G44900 | 5.17185 | 0.942873 |
| AT1G53990 | 5.16993 | 0.303195 |
| AT2G23100 | 5.16973 | 1.36594 |
| AT5G55280 | 5.1691 | 11.0206 |
| AT5G40280 | 5.16907 | 9.73636 |
| AT4G22570 | 5.16783 | 3.5249 |
| AT3G06750 | 5.16746 | 2.51516 |
| AT1G54610 | 5.16715 | 19.4604 |
| AT4G29285 | 5.1652 | 8.47351 |
| AT3G09410 | 5.16329 | 7.87674 |
| AT3G28030 | 5.16318 | 2.60966 |
| AT2G39890 | 5.16253 | 3.21138 |
| AT1G64610 | 5.16136 | 2.24031 |
| AT2G28900 | 5.16021 | 13.123 |
| AT4G14240 | 5.15964 | 5.15105 |
| AT1G80620 | 5.15832 | 20.2858 |
| AT1G72410 | 5.15662 | 14.2347 |
| AT1G30845 | 5.15398 | 6.86336 |
| AT5G61030 | 5.15351 | 26.5737 |
| AT1G66725 | 5.15305 | 0.589336 |
| AT1G74390 | 5.15145 | 3.49334 |
| AT5G10200 | 5.15009 | 11.0547 |
| AT1G66090 | 5.14808 | 12.9441 |
| AT2G27370 | 5.14792 | 5.27918 |
| AT3G16410 | 5.14576 | 33.9805 |
| AT5G14850 | 5.14523 | 7.47754 |
| AT1G17300 | 5.14164 | 16.8863 |
| AT2G32905 | 5.14119 | 2.30103 |
| AT2G17930 | 5.1407 | 7.41253 |
| AT3G63130 | 5.14033 | 17.9963 |
| AT4G31140 | 5.13932 | 7.72113 |
| AT3G59765 | 5.13725 | 12.3403 |
| AT1G60650 | 5.1363 | 8.35599 |
| AT4G33110 | 5.13369 | 11.741 |
| AT5G03890 | 5.13147 | 8.07 |
| AT3G05030 | 5.13053 | 15.5769 |
| AT1G23465 | 5.12247 | 3.89965 |
| AT5G61310 | 5.12006 | 11.169 |
| AT2G28540 | 5.11949 | 9.0139 |
| AT1G14660 | 5.11901 | 1.91194 |
| AT1G36310 | 5.11782 | 16.7218 |
| AT1G06190 | 5.1166 | 13.9402 |
| AT2G41020 | 5.11454 | 6.49627 |
| AT5G49430 | 5.11413 | 6.31348 |
| AT4G29940 | 5.11358 | 10.5969 |
| AT2G25010 | 5.11343 | 5.57134 |
| AT4G16530 | 5.11281 | 3.16249 |
| AT4G29120 | 5.11128 | 9.63233 |
| AT4G03115 | 5.10899 | 1.66066 |
| AT1G73750 | 5.10693 | 2.97345 |
| AT3G04940 | 5.10637 | 15.5167 |
| AT5G67570 | 5.10518 | 5.78504 |
| AT2G17430 | 5.10469 | 4.40072 |
| AT1G07250 | 5.10396 | 3.63507 |
| AT5G64343 | 5.1013 | 13.3538 |
| AT1G76520 | 5.10099 | 44.9443 |
| AT1G60220 | 5.09829 | 7.34162 |
| AT5G57080 | 5.09716 | 1.20582 |
| AT1G28560 | 5.09597 | 4.68363 |
| AT2G03440 | 5.0951 | 3.86746 |
| AT3G21620 | 5.09422 | 3.25403 |
| AT4G00820 | 5.09225 | 16.2115 |
| AT4G33470 | 5.09225 | 0.989774 |
| AT4G24500 | 5.0909 | 5.16602 |
| AT5G23050 | 5.0908 | 5.34714 |
| AT3G52940 | 5.08839 | 17.3477 |
| AT1G35180 | 5.08664 | 0.0979331 |
| AT1G78882 | 5.08499 | 18.6846 |
| AT4G01280 | 5.08245 | 7.89601 |
| AT3G05870 | 5.08131 | 11.4448 |
| AT1G76965 | 5.08016 | 2.00137 |
| AT5G45220 | 5.07928 | 0.338321 |
| AT3G27580 | 5.07907 | 11.7982 |
| AT1G62830 | 5.07814 | 4.35896 |
| AT4G30820 | 5.07808 | 7.45486 |
| AT1G33040 | 5.07711 | 21.6882 |
| AT3G08505 | 5.0765 | 5.38643 |
| AT5G58760 | 5.07635 | 6.2457 |
| AT4G04692 | 5.07536 | 3.95413 |
| AT5G50970 | 5.07508 | 7.18149 |
| AT5G53430 | 5.07486 | 5.20674 |
| AT2G16780 | 5.07471 | 11.5826 |
| AT3G15890 | 5.07405 | 2.45597 |
| AT5G35100 | 5.07369 | 3.00973 |
| AT5G23360 | 5.07353 | 4.22546 |
| AT1G56420 | 5.07202 | 6.29238 |
| AT5G12150 | 5.07111 | 11.1216 |
| AT5G19150 | 5.06735 | 13.1756 |
| AT1G09280 | 5.06728 | 11.7545 |
| AT5G18170 | 5.06556 | 14.8227 |
| AT5G40330 | 5.06447 | 8.60188 |
| AT5G66190 | 5.06325 | 1.95167 |
| AT5G51370 | 5.06283 | 4.0284 |
| AT5G07590 | 5.06234 | 7.60448 |
| AT1G64490 | 5.06203 | 14.3611 |
| AT4G10760 | 5.0548 | 10.6138 |
| AT5G45260 | 5.05348 | 4.545 |
| AT5G16750 | 5.05346 | 18.1749 |
| AT1G23560 | 5.0527 | 0.914674 |
| AT2G42740 | 5.05268 | 25.1544 |
| AT5G43170 | 5.05019 | 1.353 |
| AT5G37550 | 5.04858 | 1.93648 |
| AT5G14280 | 5.04549 | 3.78827 |
| AT1G28760 | 5.04317 | 6.84544 |
| AT2G27270 | 5.04239 | 3.30952 |
| AT2G46890 | 5.04055 | 13.3112 |
| AT4G00230 | 5.03744 | 2.17247 |
| AT2G37920 | 5.03451 | 4.90656 |
| AT3G61810 | 5.03417 | 1.96109 |
| AT5G26880 | 5.03343 | 11.3579 |
| AT4G33200 | 5.03336 | 8.84677 |
| AT5G66830 | 5.03265 | 3.38916 |
| AT5G13520 | 5.02999 | 14.651 |
| AT4G18110 | 5.02989 | 0.334098 |
| AT5G14220 | 5.02774 | 7.0038 |
| AT3G53030 | 5.02618 | 8.94268 |
| AT3G15240 | 5.02612 | 17.0739 |
| AT2G17540 | 5.02347 | 8.46937 |
| AT4G15570 | 5.02327 | 8.56734 |
| AT1G73655 | 5.02258 | 7.91998 |
| AT2G31940 | 5.02049 | 56.4294 |
| AT5G48545 | 5.02027 | 7.48413 |
| AT2G38025 | 5.01849 | 8.70769 |
| AT1G03750 | 5.01632 | 7.84847 |
| AT5G24680 | 5.01564 | 5.55057 |
| AT1G75340 | 5.01418 | 12.9336 |
| AT5G24840 | 5.01417 | 11.614 |
| AT4G15790 | 5.01185 | 23.6744 |
| AT5G59400 | 5.00857 | 6.314 |
| AT5G22330 | 5.00766 | 18.5012 |
| AT1G33680 | 5.00765 | 15.1076 |
| AT2G17510 | 5.00732 | 11.7211 |
| AT1G80770 | 5.00671 | 9.44664 |
| AT5G55125 | 5.00493 | 18.3384 |
| AT1G73150 | 5.00382 | 6.7613 |
| AT2G02400 | 5.0035 | 2.20773 |
| AT3G62570 | 5.00192 | 7.41937 |
| AT1G74080 | 4.99956 | 0.306451 |
| AT1G80630 | 4.99906 | 6.9591 |
| AT4G25730 | 4.99895 | 25.616 |
| AT5G62690 | 4.9964 | 26.8251 |
| AT4G39900 | 4.9952 | 16.035 |
| AT3G07510 | 4.99451 | 8.052 |
| AT4G02220 | 4.99284 | 10.3695 |
| AT5G63250 | 4.99211 | 0.164797 |
| AT1G05562 | 4.99144 | 51.0242 |
| AT1G49580 | 4.99125 | 13.3824 |
| AT2G30320 | 4.98996 | 12.0195 |
| AT5G64552 | 4.98945 | 1.69732 |
| AT2G26060 | 4.98741 | 20.7038 |
| AT5G41000 | 4.98552 | 5.62194 |
| AT4G24050 | 4.98533 | 9.01217 |
| AT3G59410 | 4.98415 | 4.46195 |
| AT1G65520 | 4.98341 | 2.15152 |
| AT5G15340 | 4.98064 | 1.30315 |
| AT2G19590 | 4.97635 | 10.1913 |
| AT3G25700 | 4.97545 | 5.57906 |
| AT1G72610 | 4.97419 | 6.52891 |
| AT1G73380 | 4.96896 | 9.50034 |
| AT5G08320 | 4.96802 | 11.396 |
| AT1G70400 | 4.96755 | 2.76811 |
| AT3G27340 | 4.96467 | 6.31933 |
| AT5G12000 | 4.96377 | 0.659195 |
| AT5G44750 | 4.96125 | 2.91441 |
| AT2G42900 | 4.96029 | 3.64728 |
| AT1G68720 | 4.95486 | 8.7125 |
| AT2G18220 | 4.95423 | 19.9203 |
| AT4G00752 | 4.95406 | 11.1605 |
| AT3G27740 | 4.95313 | 26.6987 |
| AT4G24420 | 4.95266 | 1.20334 |
| AT5G09610 | 4.95062 | 0.648558 |
| AT1G04940 | 4.94942 | 8.85693 |
| AT1G23220 | 4.94932 | 13.6815 |
| AT1G53280 | 4.94662 | 9.30406 |
| AT2G30580 | 4.94208 | 5.6267 |
| AT2G37830 | 4.94195 | 0.107167 |
| AT2G02300 | 4.93553 | 0.157395 |
| AT1G76220 | 4.93342 | 0.0477533 |
| AT1G28600 | 4.93177 | 0.342918 |
| AT1G10510 | 4.93107 | 10.1627 |
| AT1G10310 | 4.92452 | 6.70194 |
| AT4G08250 | 4.92292 | 1.61171 |
| AT3G01610 | 4.92213 | 9.64671 |
| AT1G28520 | 4.92165 | 12.1728 |
| AT1G50170 | 4.92003 | 7.9174 |
| AT5G06940 | 4.91989 | 2.50974 |
| AT4G33950 | 4.91331 | 3.4419 |
| AT3G45638 | 4.913 | 10.2008 |
| AT5G07390 | 4.91222 | 0.293337 |
| AT1G63120 | 4.91022 | 5.65646 |
| AT1G17450 | 4.90735 | 5.28676 |
| AT1G21690 | 4.90712 | 18.4002 |
| AT2G03140 | 4.90551 | 5.64621 |
| AT4G30610 | 4.90473 | 9.69292 |
| AT2G40070 | 4.90435 | 8.86063 |
| AT1G29170 | 4.90299 | 3.79126 |
| AT4G15930 | 4.90271 | 20.9179 |
| AT4G31440 | 4.90053 | 5.3849 |
| AT2G40660 | 4.89582 | 21.1996 |
| AT2G02148 | 4.89441 | 6.96849 |
| AT5G45600 | 4.89243 | 13.6897 |
| AT1G52080 | 4.88981 | 2.94976 |
| AT1G11050 | 4.88848 | 10.5039 |
| AT3G14130 | 4.88702 | 3.97388 |
| AT4G30993 | 4.88512 | 4.74146 |
| AT2G36070 | 4.88508 | 16.6216 |
| AT1G73270 | 4.8846 | 3.47649 |
| AT3G52280 | 4.8845 | 7.97092 |
| AT4G19540 | 4.88255 | 4.64351 |
| AT5G13650 | 4.88188 | 10.0526 |
| AT2G31040 | 4.88012 | 6.22768 |
| AT4G30935 | 4.87517 | 10.2425 |
| AT1G63900 | 4.87476 | 7.28916 |
| AT2G36885 | 4.87453 | 11.0849 |
| AT4G03635 | 4.87392 | 4.62158 |
| AT1G74700 | 4.87252 | 4.11129 |
| AT5G26290 | 4.87246 | 6.31666 |
| AT5G28610 | 4.86956 | 2.06908 |
| AT5G64600 | 4.86643 | 4.94384 |
| AT1G13310 | 4.86606 | 2.96965 |
| AT1G70480 | 4.86344 | 8.13809 |
| AT1G43790 | 4.86295 | 1.29207 |
| AT4G02040 | 4.86288 | 3.29776 |
| AT1G24160 | 4.86267 | 23.5945 |
| AT5G49200 | 4.86264 | 4.3962 |
| AT1G53620 | 4.86249 | 0.446371 |
| AT4G28485 | 4.86244 | 1.93601 |
| AT5G09300 | 4.86096 | 10.8213 |
| AT1G15190 | 4.86092 | 2.87358 |
| AT5G19130 | 4.86073 | 6.82152 |
| AT1G70990 | 4.8602 | 2.88448 |
| AT1G06590 | 4.85777 | 10.1345 |
| AT5G22760 | 4.85216 | 5.77601 |
| AT4G04972 | 4.85112 | 1.54722 |
| AT1G51913 | 4.84949 | 0.272056 |
| AT1G03760 | 4.84747 | 8.37215 |
| AT5G04080 | 4.84744 | 6.85132 |
| AT5G57250 | 4.84693 | 5.47675 |
| AT2G41835 | 4.84635 | 5.93343 |
| AT5G50330 | 4.84476 | 14.0014 |
| AT3G61540 | 4.84299 | 9.93776 |
| AT5G44730 | 4.84107 | 9.83174 |
| AT1G19485 | 4.84001 | 7.38141 |
| AT5G03180 | 4.83739 | 5.47212 |
| AT5G41270 | 4.83735 | 6.48737 |
| AT1G10450 | 4.83714 | 12.5623 |
| AT4G22130 | 4.83697 | 24.13 |
| AT2G26360 | 4.83277 | 9.23489 |
| AT3G54930 | 4.82926 | 1.65563 |
| AT2G29065 | 4.82656 | 4.62528 |
| AT4G30360 | 4.82258 | 10.941 |
| AT1G21070 | 4.82176 | 8.21457 |
| AT3G11560 | 4.81953 | 7.51181 |
| AT1G76405 | 4.81788 | 8.56036 |
| AT1G30050 | 4.81614 | 0.250021 |
| AT4G21902 | 4.81454 | 21.0445 |
| AT2G06925 | 4.81437 | 3.24209 |
| AT5G49530 | 4.81302 | 10.0307 |
| AT5G44000 | 4.81138 | 4.1194 |
| AT5G41370 | 4.81009 | 7.19628 |
| AT4G33410 | 4.80891 | 11.7235 |
| AT4G05450 | 4.80809 | 8.97435 |
| AT1G08590 | 4.80753 | 4.95959 |
| AT2G19330 | 4.80613 | 3.47072 |
| AT3G43670 | 4.8044 | 8.12049 |
| AT2G27980 | 4.80302 | 7.94104 |
| AT5G10470 | 4.8027 | 5.80412 |
| AT1G20540 | 4.79952 | 9.07081 |
| AT4G16650 | 4.79944 | 15.4362 |
| AT5G48340 | 4.79938 | 5.43996 |
| AT2G40840 | 4.79701 | 20.7178 |
| AT4G31390 | 4.79515 | 4.05984 |
| AT2G23430 | 4.79502 | 4.81121 |
| AT5G27990 | 4.79419 | 17.2961 |
| AT1G11180 | 4.79279 | 3.25213 |
| AT5G16200 | 4.79173 | 4.15529 |
| AT5G59770 | 4.79116 | 5.78932 |
| AT1G70500 | 4.78983 | 1.84474 |
| AT1G29790 | 4.78664 | 8.20363 |
| AT3G59390 | 4.7866 | 5.8038 |
| AT4G31110 | 4.78527 | 11.0222 |
| AT1G50250 | 4.78423 | 5.15684 |
| AT1G21710 | 4.78354 | 9.07587 |
| AT5G08415 | 4.78351 | 10.9766 |
| AT3G45730 | 4.78274 | 6.30623 |
| AT5G04110 | 4.78162 | 5.40366 |
| AT5G22270 | 4.78124 | 4.11549 |
| AT4G19010 | 4.77769 | 12.5302 |
| AT1G70690 | 4.77603 | 3.44954 |
| AT1G74280 | 4.77527 | 3.16523 |
| AT5G02400 | 4.77503 | 0.816618 |
| AT3G12250 | 4.7746 | 6.5764 |
| AT3G10986 | 4.77177 | 1.78946 |
| AT4G16310 | 4.77127 | 6.34524 |
| AT2G30640 | 4.76964 | 3.69337 |
| AT1G80400 | 4.76899 | 8.26501 |
| AT5G39980 | 4.76138 | 1.64409 |
| AT4G06598 | 4.76135 | 2.40923 |
| AT1G14060 | 4.759 | 21.5558 |
| AT4G12530 | 4.75683 | 0.385889 |
| AT1G62710 | 4.75644 | 1.60403 |
| AT1G05900 | 4.7553 | 5.51007 |
| AT3G01320 | 4.75503 | 7.51504 |
| AT1G31360 | 4.75332 | 6.82721 |
| AT1G08550 | 4.75315 | 2.42467 |
| AT1G68330 | 4.75121 | 2.46013 |
| AT3G50880 | 4.74955 | 8.44823 |
| AT2G36120 | 4.74951 | 1.95579 |
| AT4G02005 | 4.74884 | 7.3066 |
| AT5G02970 | 4.74716 | 7.16198 |
| AT4G16850 | 4.74715 | 5.14561 |
| AT1G79460 | 4.74674 | 4.80676 |
| AT2G14290 | 4.74471 | 12.409 |
| AT3G14750 | 4.74136 | 3.72542 |
| AT4G38460 | 4.74111 | 4.01493 |
| AT5G19210 | 4.73996 | 4.21822 |
| AT2G19385 | 4.7379 | 19.1766 |
| AT5G08440 | 4.73745 | 7.5278 |
| AT3G01990 | 4.73717 | 1.29036 |
| AT2G47020 | 4.73498 | 4.42144 |
| AT1G19520 | 4.73458 | 19.0714 |
| AT3G06330 | 4.73368 | 2.02291 |
| AT5G35995 | 4.73199 | 5.07212 |
| AT5G19310 | 4.7309 | 7.48202 |
| AT1G48260 | 4.73053 | 1.93674 |
| AT5G49970 | 4.72589 | 9.92542 |
| AT1G05030 | 4.72554 | 6.09684 |
| AT2G38140 | 4.71864 | 16.891 |
| AT2G47620 | 4.71851 | 9.23426 |
| AT1G73330 | 4.71834 | 0.416565 |
| AT2G18620 | 4.71811 | 1.25625 |
| AT3G48030 | 4.71768 | 2.62689 |
| AT2G01130 | 4.71671 | 7.27264 |
| AT5G10020 | 4.71584 | 9.46595 |
| AT3G59530 | 4.71583 | 4.56123 |
| AT4G25100 | 4.71557 | 6.36237 |
| AT5G62900 | 4.71425 | 13.8292 |
| AT2G22360 | 4.70878 | 7.27386 |
| AT1G53790 | 4.70666 | 1.36268 |
| AT2G20120 | 4.70586 | 7.10553 |
| AT3G07250 | 4.70548 | 4.39819 |
| AT4G35460 | 4.70444 | 6.22678 |
| AT5G37830 | 4.70437 | 8.50897 |
| AT3G13226 | 4.70434 | 9.62045 |
| AT2G45680 | 4.70382 | 2.48906 |
| AT3G48120 | 4.70269 | 4.85997 |
| AT5G49820 | 4.70262 | 12.5193 |
| AT3G24300 | 4.70156 | 1.53068 |
| AT3G23570 | 4.70019 | 7.26861 |
| AT5G37500 | 4.69971 | 7.09046 |
| AT1G31660 | 4.69402 | 21.6654 |
| AT3G20780 | 4.69385 | 7.12105 |
| AT5G17420 | 4.69207 | 2.01486 |
| AT2G39435 | 4.68996 | 3.37114 |
| AT3G49360 | 4.68973 | 0.866203 |
| AT5G23280 | 4.68931 | 4.31411 |
| AT2G42870 | 4.68788 | 18.5274 |
| AT1G66700 | 4.68774 | 21.9632 |
| AT5G52010 | 4.68591 | 2.6743 |
| AT3G62130 | 4.68432 | 8.96152 |
| AT5G61830 | 4.68419 | 9.96678 |
| AT1G73170 | 4.68307 | 3.53853 |
| AT1G05055 | 4.68037 | 8.03831 |
| AT2G41200 | 4.67975 | 2.04136 |
| AT2G48070 | 4.67466 | 3.27328 |
| AT4G34960 | 4.6733 | 14.6382 |
| AT3G05250 | 4.67321 | 5.92316 |
| AT3G14240 | 4.67206 | 4.95442 |
| AT3G04580 | 4.66801 | 8.25862 |
| AT5G10400 | 4.66434 | 19.3878 |
| AT2G05790 | 4.66268 | 16.8098 |
| AT1G44910 | 4.66124 | 11.1528 |
| AT1G15410 | 4.66069 | 8.9032 |
| AT1G09230 | 4.65886 | 8.92155 |
| AT1G13730 | 4.65547 | 23.5099 |
| AT4G02760 | 4.65361 | 4.55531 |
| AT2G02580 | 4.6498 | 0.0413129 |
| AT5G58230 | 4.64851 | 19.1653 |
| AT1G69070 | 4.64825 | 15.588 |
| AT1G03540 | 4.64765 | 2.52179 |
| AT2G13100 | 4.64461 | 5.67037 |
| AT1G03530 | 4.64375 | 6.29893 |
| AT5G63595 | 4.64365 | 0.903461 |
| AT1G27590 | 4.64228 | 4.68848 |
| AT5G48590 | 4.63852 | 6.32985 |
| AT1G16040 | 4.63527 | 17.0212 |
| AT5G35840 | 4.63496 | 6.59328 |
| AT2G05995 | 4.63449 | 1.22653 |
| AT3G14610 | 4.63384 | 10.5593 |
| AT1G10120 | 4.6312 | 12.9788 |
| AT5G05090 | 4.6311 | 1.38431 |
| AT2G44578 | 4.62882 | 2.93439 |
| AT4G16250 | 4.62559 | 8.92836 |
| AT2G32840 | 4.62432 | 7.84898 |
| AT1G26170 | 4.62309 | 8.2139 |
| AT2G39120 | 4.62184 | 3.88596 |
| AT1G77590 | 4.62 | 14.7636 |
| AT5G42070 | 4.61951 | 2.83901 |
| AT3G25860 | 4.61896 | 36.0991 |
| AT5G27410 | 4.61818 | 5.2047 |
| AT3G09070 | 4.61645 | 5.27981 |
| AT5G62340 | 4.61395 | 16.8905 |
| AT5G66560 | 4.61322 | 3.75387 |
| AT4G00040 | 4.61295 | 1.07253 |
| AT4G34588 | 4.61129 | 4.26439 |
| AT3G20280 | 4.60886 | 4.48047 |
| AT1G44760 | 4.608 | 28.2736 |
| AT3G26580 | 4.60326 | 10.2034 |
| AT3G59750 | 4.59744 | 2.61453 |
| AT1G19300 | 4.59646 | 8.37476 |
| AT1G35340 | 4.59594 | 3.6642 |
| AT3G17330 | 4.59519 | 14.5271 |
| AT1G55280 | 4.59473 | 6.29148 |
| AT1G06510 | 4.59319 | 4.69669 |
| AT1G32690 | 4.58777 | 5.56406 |
| AT5G42320 | 4.58733 | 14.8739 |
| AT2G32090 | 4.58661 | 4.76594 |
| AT5G01730 | 4.58482 | 0.944651 |
| AT4G01200 | 4.58294 | 2.52422 |
| AT4G08580 | 4.58239 | 6.58976 |
| AT2G05812 | 4.58231 | 5.13982 |
| AT4G09510 | 4.58171 | 3.25582 |
| AT4G35300 | 4.5765 | 9.31241 |
| AT2G40760 | 4.57519 | 7.58656 |
| AT1G48520 | 4.57516 | 8.7404 |
| AT4G15630 | 4.57473 | 2.38966 |
| AT1G64563 | 4.57445 | 1.79414 |
| AT1G34430 | 4.57425 | 22.4358 |
| AT4G10070 | 4.57214 | 4.88146 |
| AT4G37690 | 4.57194 | 3.27019 |
| AT3G50760 | 4.57141 | 7.40222 |
| AT4G22520 | 4.57046 | 1.39379 |
| AT3G46385 | 4.56957 | 2.59953 |
| AT4G18465 | 4.56564 | 13.2931 |
| AT5G19420 | 4.56389 | 7.3928 |
| AT5G65520 | 4.56317 | 3.1103 |
| AT3G07040 | 4.56268 | 1.77353 |
| AT4G27340 | 4.56256 | 2.16973 |
| AT1G73210 | 4.56198 | 8.49964 |
| AT3G12990 | 4.56121 | 6.15646 |
| AT5G35910 | 4.55823 | 12.2607 |
| AT4G10970 | 4.55677 | 10.2965 |
| AT1G74420 | 4.55381 | 2.06044 |
| AT2G17650 | 4.55052 | 0.959102 |
| AT1G48490 | 4.55013 | 4.61993 |
| AT1G61900 | 4.54538 | 24.6589 |
[truncated: 274,508 more chars]
