# Supplementary material for: Re-analysis of RNA-seq transcriptome data reveals new aspects of gene activity in Arabidopsis root hairs
Source: Front Plant Sci. 2015 Jun 8;6:421. doi: 10.3389/fpls.2015.00421 (PMC4458573; doi:10.3389/fpls.2015.00421)
Supplement: Supplementary file 7 [file Table2.DOC]

**Table S2** List of the 30 most highly expressed transcripts in non-root hair tissues.

| AGI | Annotation | RPKM |
| --- | --- | --- |
| AT1G17190 | ATGSTU26, GSTU26, glutathione S-transferase tau 26 | 2175.36 |
| AT3G47347 | snoRNA | 2007.74 |
| AT5G54940 | Translation initiation factor SUI1 family protein | 1577.99 |
| AT1G76180 | ERD14, Dehydrin family protein | 1432.35 |
| AT4G30530 | Class I glutamine amidotransferase-like superfamily protein | 1415.69 |
| AT1G66580 | RPL10C, SAG24, senescence associated gene 24 | 1400.11 |
| AT1G17180 | ATGSTU25, GSTU25, glutathione S-transferase TAU 25 | 1383.85 |
| AT5G20230 | ATBCB, BCB, BCB, SAG14, blue-copper-binding protein | 1363.1 |
| AT2G24850 | TAT, TAT3, tyrosine aminotransferase 3 | 1355.24 |
| AT5G42980 | ATH3, ATTRX3, ATTRXH3, TRX3, TRXH3, thioredoxin 3 | 1329.65 |
| AT2G41430 | CID1, ERD15, LSR1, dehydration-induced protein (ERD15) | 1242.35 |
| AT5G65207 | unknown protein | 1232.51 |
| AT2G29450 | ATGSTU5, GSTU5, glutathione S-transferase tau 5 | 1210.33 |
| AT2G43610 | Chitinase family protein | 1205.59 |
| AT3G05890 | RCI2B, Low temperature and salt responsive protein family | 1183.73 |
| AT3G15450 | Aluminium induced protein with YGL and LRDR motifs | 1127.62 |
| AT5G07440 | GDH2, glutamate dehydrogenase 2 | 989.80 |
| AT1G25275 | unknown protein | 935.68 |
| AT5G54370 | Late embryogenesis abundant (LEA) protein-related | 926.7 |
| AT2G22470 | AGP2, ATAGP2, arabinogalactan protein 2 | 925.56 |
| AT3G13520 | AGP12, ATAGP12, arabinogalactan protein 12 | 918.67 |
| AT1G78040 | Pollen Ole e 1 allergen and extensin family protein | 907.94 |
| AT3G53990 | Adenine nucleotide alpha hydrolases-like protein | 893.19 |
| AT1G20440 | AtCOR47, COR47, RD17, cold-regulated 47 | 886.21 |
| AT3G09940 | ATMDAR3, monodehydroascorbate reductase | 879.21 |
| AT1G20450 | ERD10, LTI29, LTI45, Dehydrin family protein | 864.19 |
| AT4G21850 | ATMSRB9, MSRB9, methionine sulfoxide reductase B9 | 864.17 |
| AT5G15230 | GASA4, GAST1 protein homolog 4 | 830.23 |
| AT3G48340 | Cysteine proteinases superfamily protein | 829.13 |
| AT5G11740 | AGP15, ATAGP15, arabinogalactan protein 15 | 814.37 |
