# Supplementary material for: Re-analysis of RNA-seq transcriptome data reveals new aspects of gene activity in Arabidopsis root hairs
Source: Front Plant Sci. 2015 Jun 8;6:421. doi: 10.3389/fpls.2015.00421 (PMC4458573; doi:10.3389/fpls.2015.00421)
Supplement: Supplementary file 8 [file Table3.DOC]

**Table S3** List of the 14 most highly expressed transcripts overlapping between root hairs (RH) and non-root hair tissues (NRH)

| AGI | Annotation | RH(RPKM) | NRH(RPKM) | Fold_change(log2) | p_value | | q_value |
| --- | --- | --- | --- | --- | --- | --- | --- |
| AT1G17190 | ATGSTU26 | 2344.36 | 2175.36 | -0.10794 | 0.86295 | 0.91122 | |
| AT1G20440 | AtCOR47 | 2699.7 | 886.206 | -1.60709 | 0.00145 | 0.0094058 | |
| AT1G20450 | ERD10 | 2026.31 | 864.185 | -1.22944 | 0.01365 | 0.0494285 | |
| AT1G66580 | RPL10C | 2021.75 | 1400.11 | -0.530068 | 0.31225 | 0.460412 | |
| AT1G76180 | ERD14 | 4562.78 | 1432.35 | -1.67153 | 0.00085 | 0.0063046 | |
| AT2G22470 | ATAGP2 | 3220.46 | 925.556 | -1.79887 | 0.0017 | 0.0106071 | |
| AT2G24850 | tyrosine aminotransferase 3 | 1764.74 | 1355.24 | -0.380899 | 0.4533 | 0.599596 | |
| AT2G29450 | ATGSTU5 | 1729.43 | 1210.33 | -0.514897 | 0.31515 | 0.463343 | |
| AT3G15450 | Aluminium induced protein with YGL and LRDR motifs | 3385.68 | 1127.62 | -1.58617 | 0.0033 | 0.0173075 | |
| AT5G11740 | ATAGP15, | 1943.79 | 814.374 | -1.25511 | 0.00575 | 0.0263656 | |
| AT5G20230 | ATBCB,blue-copper-binding protein | 1947.38 | 1363.1 | -0.514641 | 0.3174 | 0.465442 | |
| AT5G42980 | ATTRX3, thioredoxin 3 | 2714.9 | 1329.65 | -1.02985 | 0.05015 | 0.126971 | |
| AT5G54940 | Translation initiation factor SUI1 family protein | 3070.01 | 1577.99 | -0.960158 | 0.0554 | 0.136528 | |
| AT5G65207 | unknown protein | 3333.24 | 1232.51 | -1.43533 | 0.0023 | 0.0132526 | |
