# Supplementary material for: Re-analysis of RNA-seq transcriptome data reveals new aspects of gene activity in Arabidopsis root hairs
Source: Front Plant Sci. 2015 Jun 8;6:421. doi: 10.3389/fpls.2015.00421 (PMC4458573; doi:10.3389/fpls.2015.00421)
Supplement: Supplementary file 9 [file Table4.DOC]

**Table S4** Gene Ontology enrichment was assessed using GOBU (Lin et al., 2006) in the 30 highest abundant genes from root hairs(RH) and non-root hair tissues (NRH) with *elim* method (P<0.01). In the term type column, P, F and C indicate biological process, functional process and subcellular localization, respectively.

| **GOID** | **GO Name** | **Term Type** | **Gene count(reference)** | **Gene count(RH 30)** | **Gene count(NRH 30 )** | **P value (elim, RH 30)** | **P value (elim, NRH 30)** |
| --- | --- | --- | --- | --- | --- | --- | --- |
| GO:0009631 | cold acclimation | P | 21 | 3 | 2 | 8.45E-07 | 1.60E-04 |
| GO:0006979 | response to oxidative stress | P | 278 | 5 | 3 | 4.50E-06 | 0.0019278 |
| GO:0010188 | response to microbial phytotoxin | P | 6 | 2 | 1 | 1.15E-05 | 0.0053453 |
| GO:0009414 | response to water deprivation | P | 218 | 4 | 5 | 4.14E-05 | 1.37E-06 |
| GO:0050832 | defense response to fungus | P | 132 | 3 | 2 | 2.23E-04 | 0.0061993 |
| GO:0009737 | response to abscisic acid stimulus | P | 359 | 4 | 3 | 2.82E-04 | 0.0039646 |
| GO:0009407 | toxin catabolic process | P | 46 | 2 | 3 | 7.78E-04 | 9.50E-06 |
| GO:0009635 | response to herbicide | P | 1 | 1 | 1 | 8.93E-04 | 8.93E-04 |
| GO:0015690 | aluminum ion transport | P | 1 | 1 | 1 | 8.93E-04 | 8.93E-04 |
| GO:0010035 | response to inorganic substance | P | 536 | 4 | 2 | 0.0012625 | 0.0824367 |
| GO:0009664 | plant-type cell wall organization | P | 79 | 2 | 0 | 0.0022748 | 1 |
| GO:0009642 | response to light intensity | P | 80 | 2 | 2 | 0.00233184 | 0.0023318 |
| GO:0015865 | purine nucleotide transport | P | 7 | 1 | 0 | 0.00623347 | 1 |
| GO:0009611 | response to wounding | P | 153 | 2 | 2 | 0.00824129 | 0.0082413 |
| GO:0009409 | response to cold | P | 279 | 5 | 6 | 0.022178 | 7.95E-05 |
| GO:0019496 | serine-isocitrate lyase pathway | P | 1 | 0 | 1 | 1 | 8.93E-04 |
| GO:0043903 | regulation of symbiosis, encompassing mutualism through parasitism | P | 2 | 0 | 1 | 1 | 0.0017848 |
| GO:0019683 | glyceraldehyde-3-phosphate catabolic process | P | 3 | 0 | 1 | 1 | 0.0026761 |
| GO:0019564 | aerobic glycerol catabolic process | P | 3 | 0 | 1 | 1 | 0.0026761 |
| GO:0019656 | glucose catabolic process to D-lactate and ethanol | P | 4 | 0 | 1 | 1 | 0.0035666 |
| GO:0019650 | glucose catabolic process to butanediol | P | 5 | 0 | 1 | 1 | 0.0044563 |
| GO:0019658 | glucose catabolic process to lactate and acetate | P | 6 | 0 | 1 | 1 | 0.0053453 |
| GO:0006970 | response to osmotic stress | P | 449 | 2 | 3 | 0.06061804 | 0.0073619 |
| GO:0009610 | response to symbiotic fungus | P | 9 | 0 | 1 | 1 | 0.0080076 |
| GO:0006094 | gluconeogenesis | P | 9 | 0 | 1 | 1 | 0.0080076 |
| GO:0009753 | response to jasmonic acid stimulus | P | 163 | 1 | 2 | 0.13579417 | 0.0093061 |
| GO:0016671 | oxidoreductase activity, acting on a sulfur group of donors, disulfide as acceptor | F | 27 | 2 | 2 | 2.67E-04 | 2.67E-04 |
| GO:0005199 | structural constituent of cell wall | F | 33 | 2 | 0 | 4.00E-04 | 1 |
| GO:0004364 | glutathione transferase activity | F | 48 | 2 | 3 | 8.47E-04 | 1.08E-05 |
| GO:0004838 | L-tyrosine:2-oxoglutarate aminotransferase activity | F | 3 | 1 | 1 | 0.0026761 | 0.0026761 |
| GO:0008466 | glycogenin glucosyltransferase activity | F | 4 | 1 | 0 | 0.0035666 | 1 |
| GO:0005471 | ATP:ADP antiporter activity | F | 6 | 1 | 0 | 0.00534528 | 1 |
| GO:0043295 | glutathione binding | F | 10 | 1 | 1 | 0.00889344 | 0.0088934 |
| GO:0034722 | gamma-glutamyl-peptidase activity | F | 1 | 0 | 1 | 1 | 8.93E-04 |
| GO:0004352 | glutamate dehydrogenase activity | F | 1 | 0 | 1 | 1 | 8.93E-04 |
| GO:0004353 | glutamate dehydrogenase [NAD(P)+] activity | F | 2 | 0 | 1 | 1 | 0.0017848 |
| GO:0016656 | monodehydroascorbate reductase (NADH) activity | F | 4 | 0 | 1 | 1 | 0.0035666 |
| GO:0008061 | chitin binding | F | 8 | 0 | 1 | 1 | 0.0071209 |
| GO:0031225 | anchored to membrane | C | 247 | 5 | 4 | 2.53E-06 | 6.72E-05 |
| GO:0005773 | vacuole | C | 833 | 6 | 4 | 8.14E-05 | 0.0061543 |
| GO:0005886 | plasma membrane | C | 1821 | 8 | 5 | 1.47E-04 | 0.0213616 |
| GO:0005829 | cytosol | C | 792 | 4 | 4 | 0.00515621 | 0.0051562 |
