# Supplementary material for: Re-analysis of RNA-seq transcriptome data reveals new aspects of gene activity in Arabidopsis root hairs
Source: Front Plant Sci. 2015 Jun 8;6:421. doi: 10.3389/fpls.2015.00421 (PMC4458573; doi:10.3389/fpls.2015.00421)
Supplement: Supplementary file 10 [file Table5.DOC]

**Table S5** Differentially expressed transcripts between root hairs (RH) and non-root hair tissues (NRH).

| AGI | Annotation | RH(RPKM) | NRH(RPKM) | Fold_change(log2) | p_value | q_value |
| --- | --- | --- | --- | --- | --- | --- |
| AT2G44340 | VQ motif-containing protein | 13.3286 | 0 | #NAME? | 0.00005 | 0.00070028 |
| AT3G58000 | VQ motif-containing protein | 10.8119 | 0 | #NAME? | 0.00005 | 0.00070028 |
| AT5G24313 | unknown protein | 46.1877 | 0 | #NAME? | 0.00005 | 0.00070028 |
| AT4G06534 | unknown protein | 10.6746 | 0 | #NAME? | 0.00005 | 0.00070028 |
| AT5G23903 | unknown protein | 1.07389 | 0 | #NAME? | 0.0006 | 0.00487984 |
| AT5G41761 | unknown protein | 1.37593 | 0 | #NAME? | 0.00005 | 0.00070028 |
| AT4G10860 | unknown protein | 1.83738 | 0 | #NAME? | 0.00005 | 0.00070028 |
| AT5G54790 | unknown protein | 1.91974 | 0 | #NAME? | 0.00005 | 0.00070028 |
| AT3G20557 | unknown protein | 5.65851 | 0 | #NAME? | 0.00005 | 0.00070028 |
| AT5G48700 | Ubiquitin-like superfamily protein | 1.54823 | 0 | #NAME? | 0.0011 | 0.00759303 |
| AT4G06536 | SPla/RYanodine receptor (SPRY) domain-containing protein | 21.5337 | 0 | #NAME? | 0.00005 | 0.00070028 |
| AT1G69230 | SP1L2, SPIRAL1-like2 | 1.27038 | 0 | #NAME? | 0.00005 | 0.00070028 |
| AT3G61900 | SAUR-like auxin-responsive protein family | 1.06586 | 0 | #NAME? | 0.00835 | 0.0345732 |
| AT1G35330 | RING/U-box superfamily protein | 9.22559 | 0 | #NAME? | 0.00005 | 0.00070028 |
| AT4G04900 | RIC10, ROP-interactive CRIB motif-containing protein 10 | 12.4933 | 0 | #NAME? | 0.00005 | 0.00070028 |
| AT1G51880 | RHS6, root hair specific 6 | 6.35543 | 0 | #NAME? | 0.00005 | 0.00070028 |
| AT1G05990 | RHS1, EF hand calcium-binding protein family | 11.0517 | 0 | #NAME? | 0.00005 | 0.00070028 |
| AT3G04735 | RALFL21, RALF-like 21 | 1.53836 | 0 | #NAME? | 0.0136 | 0.0492835 |
| AT2G29010 | pseudogene, receptor protein kinase | 1.4021 | 0 | #NAME? | 0.00005 | 0.00070028 |
| AT1G34330 | pseudogene, putative peroxidase | 2.80905 | 0 | #NAME? | 0.00005 | 0.00070028 |
| AT5G22390 | Protein of unknown function (DUF3049) | 1.14436 | 0 | #NAME? | 0.00005 | 0.00070028 |
| AT4G25930 | Protein of unknown function (DUF295) | 1.30174 | 0 | #NAME? | 0.00005 | 0.00070028 |
| AT1G26250 | Proline-rich extensin-like family protein | 6.18218 | 0 | #NAME? | 0.00005 | 0.00070028 |
| AT1G33870 | P-loop containing nucleoside triphosphate hydrolases superfamily protein | 1.18212 | 0 | #NAME? | 0.00005 | 0.00070028 |
| AT4G34930 | PLC-like phosphodiesterases superfamily protein | 3.67788 | 0 | #NAME? | 0.00005 | 0.00070028 |
| AT5G22560 | Plant protein of unknown function (DUF247) | 3.89387 | 0 | #NAME? | 0.00005 | 0.00070028 |
| AT2G23240 | Plant EC metallothionein-like protein, family 15 | 1.13736 | 0 | #NAME? | 0.0082 | 0.0341376 |
| AT3G18460 | PLAC8 family protein | 1.75622 | 0 | #NAME? | 0.00015 | 0.00170235 |
| AT3G18470 | PLAC8 family protein | 1.63194 | 0 | #NAME? | 0.00005 | 0.00070028 |
| AT1G11920 | Pectin lyase-like superfamily protein | 1.31645 | 0 | #NAME? | 0.00005 | 0.00070028 |
| AT1G60050 | Nodulin MtN21 /EamA-like transporter family protein | 17.708 | 0 | #NAME? | 0.00005 | 0.00070028 |
| AT1G34520 | MBOAT (membrane bound O-acyl transferase) family protein | 2.21347 | 0 | #NAME? | 0.00005 | 0.00070028 |
| AT1G51810 | Leucine-rich repeat protein kinase family protein | 7.33672 | 0 | #NAME? | 0.00005 | 0.00070028 |
| AT2G29000 | Leucine-rich repeat protein kinase family protein | 3.02968 | 0 | #NAME? | 0.00005 | 0.00070028 |
| AT3G46340 | Leucine-rich repeat protein kinase family protein | 2.43475 | 0 | #NAME? | 0.00005 | 0.00070028 |
| AT3G46370 | Leucine-rich repeat protein kinase family protein | 1.21859 | 0 | #NAME? | 0.00005 | 0.00070028 |
| AT5G21130 | Late embryogenesis abundant (LEA) hydroxyproline-rich glycoprotein family | 4.04504 | 0 | #NAME? | 0.00005 | 0.00070028 |
| AT2G26410 | Iqd4, IQ-domain 4 | 9.48085 | 0 | #NAME? | 0.00005 | 0.00070028 |
| AT1G61080 | Hydroxyproline-rich glycoprotein family protein | 2.79338 | 0 | #NAME? | 0.00005 | 0.00070028 |
| AT4G19800 | Glycosyl hydrolase family protein with chitinase insertion domain | 1.70628 | 0 | #NAME? | 0.00005 | 0.00070028 |
| AT4G19760 | Glycosyl hydrolase family protein with chitinase insertion domain | 1.65757 | 0 | #NAME? | 0.00005 | 0.00070028 |
| AT4G19770 | Glycosyl hydrolase family protein with chitinase insertion domain | 1.20959 | 0 | #NAME? | 0.00005 | 0.00070028 |
| AT1G24485 | function unknown | 10.4234 | 0 | #NAME? | 0.00005 | 0.00070028 |
| AT5G05420 | FKBP-like peptidyl-prolyl cis-trans isomerase family protein | 1.99553 | 0 | #NAME? | 0.0011 | 0.00759303 |
| AT3G50710 | F-box/RNI-like/FBD-like domains-containing protein | 10.9567 | 0 | #NAME? | 0.00005 | 0.00070028 |
| AT4G25190 | Family of unknown function (DUF566) | 21.5618 | 0 | #NAME? | 0.00005 | 0.00070028 |
| AT5G54050 | Cysteine/Histidine-rich C1 domain family protein | 3.26743 | 0 | #NAME? | 0.00005 | 0.00070028 |
| AT2G43220 | Cysteine/Histidine-rich C1 domain family protein | 1.36488 | 0 | #NAME? | 0.00005 | 0.00070028 |
| AT1G34540 | CYP94D1, cytochrome P450, family 94, subfamily D, polypeptide 1 | 25.9102 | 0 | #NAME? | 0.00005 | 0.00070028 |
| AT5G61650 | CYCP4, CYCP4;2, CYCLIN P4;2 | 6.11587 | 0 | #NAME? | 0.00005 | 0.00070028 |
| AT4G33730 | CAP (Cysteine-rich secretory proteins, Antigen 5, and Pathogenesis-related 1 protein) superfamily protein | 29.2434 | 0 | #NAME? | 0.00005 | 0.00070028 |
| AT5G56200 | C2H2 type zinc finger transcription factor family | 1.6753 | 0 | #NAME? | 0.00005 | 0.00070028 |
| AT4G12360 | Bifunctional inhibitor/lipid-transfer protein/seed storage 2S albumin superfamily protein | 2.02718 | 0 | #NAME? | 0.00005 | 0.00070028 |
| AT5G43175 | basic helix-loop-helix (bHLH) DNA-binding superfamily protein | 14.6086 | 0 | #NAME? | 0.00005 | 0.00070028 |
| AT2G14760 | basic helix-loop-helix (bHLH) DNA-binding superfamily protein | 1.99999 | 0 | #NAME? | 0.00005 | 0.00070028 |
| AT2G37740 | ATZFP10, ZFP10, zinc-finger protein 10 | 1.88115 | 0 | #NAME? | 0.00005 | 0.00070028 |
| AT4G38850 | ATSAUR15, SAUR-AC1, SAUR15, SAUR_AC1, SAUR-like auxin-responsive protein family | 1.45983 | 0 | #NAME? | 0.0003 | 0.00292382 |
| AT5G58360 | ATOFP3, OFP3, ovate family protein 3 | 2.8683 | 0 | #NAME? | 0.00005 | 0.00070028 |
| AT3G25650 | ASK15, SK15, SKP1-like 15 | 1.26335 | 0 | #NAME? | 0.0026 | 0.0145003 |
| AT1G08100 | ACH2, ATNRT2.2, NRT2.2, NRT2;2AT, nitrate transporter 2.2 | 4.91682 | 0 | #NAME? | 0.00005 | 0.00070028 |
| AT3G46480 | 2-oxoglutarate (2OG) and Fe(II)-dependent oxygenase superfamily protein | 1.00277 | 0 | #NAME? | 0.0001 | 0.00123775 |
| AT3G48346 | unknown protein | 0 | 119.382 | inf | 0.0013 | 0.00867426 |
| AT2G05270 | unknown protein | 0 | 1.21048 | inf | 0.00675 | 0.0295876 |
| AT4G18335 | unknown protein | 0 | 1.17798 | inf | 0.00095 | 0.00685384 |
| AT1G47395 | unknown protein | 0 | 1.73289 | inf | 0.00125 | 0.00840292 |
| AT5G45740 | Ubiquitin domain-containing protein | 0 | 4.61344 | inf | 0.0001 | 0.00123775 |
| AT1G13145 | pseudogene of unknown protein | 0 | 1.27083 | inf | 0.0003 | 0.00292382 |
| AT5G43250 | NF-YC13, nuclear factor Y, subunit C13 | 0 | 1.4447 | inf | 0.00135 | 0.0089118 |
| AT1G52680 | late embryogenesis abundant protein-related / LEA protein-related | 0 | 1.6161 | inf | 0.00005 | 0.00070028 |
| AT1G78520 | Carbohydrate-binding X8 domain superfamily protein | 0 | 6.50444 | inf | 0.00005 | 0.00070028 |
| AT1G73550 | Bifunctional inhibitor/lipid-transfer protein/seed storage 2S albumin superfamily protein | 0 | 1.00346 | inf | 0.002 | 0.0119879 |
| AT2G24980 | Proline-rich extensin-like family protein | 282.55 | 0.145177 | -10.9265 | 0.00615 | 0.0276932 |
| AT5G06630 | proline-rich extensin-like family protein | 350.094 | 0.263724 | -10.3745 | 0.0003 | 0.00292382 |
| AT1G12560 | ATEXP7, ATEXPA7, ATHEXP ALPHA 1.26, EXP7, EXPA7, expansin A7 | 390.921 | 0.365196 | -10.064 | 0.00025 | 0.00253767 |
| AT4G40090 | AGP3, arabinogalactan protein 3 | 1002.28 | 1.07251 | -9.86808 | 0.00005 | 0.00070028 |
| AT3G62680 | ATPRP3, PRP3, proline-rich protein 3 | 419.939 | 0.453138 | -9.85601 | 0.0002 | 0.00214445 |
| AT5G04960 | Plant invertase/pectin methylesterase inhibitor superfamily | 179.825 | 0.201765 | -9.7997 | 0.0004 | 0.00359419 |
| AT3G09925 | Pollen Ole e 1 allergen and extensin family protein | 685.787 | 0.79408 | -9.75426 | 0.00575 | 0.0263656 |
| AT4G25820 | ATXTH14, XTH14, XTR9, xyloglucan endotransglucosylase/hydrolase 14 | 758.459 | 0.933845 | -9.66567 | 0.00005 | 0.00070028 |
| AT5G06640 | Proline-rich extensin-like family protein | 300.574 | 0.375472 | -9.6448 | 0.00005 | 0.00070028 |
| AT3G54590 | ATHRGP1, HRGP1, hydroxyproline-rich glycoprotein | 882.994 | 1.10396 | -9.64358 | 0.00005 | 0.00070028 |
| AT5G67400 | RHS19, root hair specific 19 | 456.332 | 0.570824 | -9.64282 | 0.00005 | 0.00070028 |
| AT4G13390 | Proline-rich extensin-like family protein | 346.903 | 0.436798 | -9.63335 | 0.00005 | 0.00070028 |
| AT4G02270 | RHS13, root hair specific 13 | 828.721 | 1.12794 | -9.52106 | 0.00005 | 0.00070028 |
| AT4G00680 | ADF8, actin depolymerizing factor 8 | 479.401 | 0.676228 | -9.46951 | 0.00065 | 0.00516972 |
| AT1G62980 | ATEXP18, ATEXPA18, ATHEXP ALPHA 1.25, EXP18, EXPA18, expansin A18 | 208.435 | 0.345746 | -9.23567 | 0.0009 | 0.0065867 |
| AT5G57530 | AtXTH12, XTH12, xyloglucan endotransglucosylase/hydrolase 12 | 125.269 | 0.209592 | -9.22322 | 0.0041 | 0.0203755 |
| AT5G35190 | proline-rich extensin-like family protein | 467.479 | 0.808864 | -9.17479 | 0.00005 | 0.00070028 |
| AT2G29620 | unknown protein | 35.8692 | 0.067347 | -9.05692 | 0.00635 | 0.0283169 |
| AT1G30870 | Peroxidase superfamily protein | 413.75 | 0.811178 | -8.99453 | 0.00005 | 0.00070028 |
| AT1G12040 | LRX1, leucine-rich repeat/extensin 1 | 184.771 | 0.367391 | -8.97421 | 0.00005 | 0.00070028 |
| AT5G05500 | Pollen Ole e 1 allergen and extensin family protein | 467.413 | 0.954079 | -8.93637 | 0.0001 | 0.00123775 |
| AT5G11440 | CID5, IPD1, CTC-interacting domain 5 | 141.179 | 0.300056 | -8.87808 | 0.0024 | 0.0136815 |
| AT1G48930 | AtGH9C1, GH9C1, glycosyl hydrolase 9C1 | 212.097 | 0.450867 | -8.87781 | 0.00005 | 0.00070028 |
| AT1G54970 | ATPRP1, PRP1, RHS7, proline-rich protein 1 | 197.916 | 0.420925 | -8.87711 | 0.0003 | 0.00292382 |
| AT2G41970 | Protein kinase superfamily protein | 232.38 | 0.551853 | -8.71799 | 0.00005 | 0.00070028 |
| AT2G47540 | Pollen Ole e 1 allergen and extensin family protein | 213.419 | 0.513428 | -8.69931 | 0.00465 | 0.0223951 |
| AT5G22410 | RHS18, root hair specific 18 | 106.322 | 0.292415 | -8.50621 | 0.0003 | 0.00292382 |
| AT2G47360 | unknown protein | 62.0735 | 0.184463 | -8.3945 | 0.00825 | 0.0342522 |
| AT2G30670 | NAD(P)-binding Rossmann-fold superfamily protein | 112.11 | 0.339629 | -8.36674 | 0.0035 | 0.0180741 |
| AT3G10710 | RHS12, root hair specific 12 | 57.5381 | 0.189001 | -8.24998 | 0.00035 | 0.00327416 |
| AT2G45890 | ATROPGEF4, RHS11, ROPGEF4, RHO guanyl-nucleotide exchange factor 4 | 101.066 | 0.368687 | -8.09869 | 0.00005 | 0.00070028 |
| AT5G49270 | COBL9, DER9, MRH4, SHV2, COBRA-like extracellular glycosyl-phosphatidyl inositol-anchored protein family | 80.4778 | 0.313345 | -8.00469 | 0.00005 | 0.00070028 |
| AT5G22555 | unknown protein | 196.529 | 0.860094 | -7.83603 | 0.00745 | 0.0318523 |
| AT5G40860 | unknown protein | 136.684 | 0.628081 | -7.76568 | 0.0003 | 0.00292382 |
| AT3G54580 | Proline-rich extensin-like family protein | 1903.3 | 8.85952 | -7.74706 | 0.00005 | 0.00070028 |
| AT4G09990 | Protein of unknown function (DUF579) | 189.187 | 0.903108 | -7.7107 | 0.00005 | 0.00070028 |
| AT1G08090 | ACH1, ATNRT2.1, ATNRT2:1, LIN1, NRT2, NRT2.1, NRT2:1, NRT2;1AT, nitrate transporter 2:1 | 52.9332 | 0.266549 | -7.63363 | 0.0001 | 0.00123775 |
| AT3G60330 | AHA7, HA7, H(+)-ATPase 7 | 310.855 | 1.56648 | -7.63257 | 0.00005 | 0.00070028 |
| AT2G33460 | RIC1, ROP-interactive CRIB motif-containing protein 1 | 58.361 | 0.294145 | -7.63233 | 0.0037 | 0.0188077 |
| AT4G26010 | Peroxidase superfamily protein | 461.842 | 2.38604 | -7.59664 | 0.00005 | 0.00070028 |
| AT3G07070 | Protein kinase superfamily protein | 39.4694 | 0.207821 | -7.56925 | 0.0004 | 0.00359419 |
| AT2G46860 | AtPPa3, PPa3, pyrophosphorylase 3 | 111.429 | 0.593574 | -7.55249 | 0.00015 | 0.00170235 |
| AT3G47040 | Glycosyl hydrolase family protein | 30.0456 | 0.168941 | -7.47449 | 0.00055 | 0.00457655 |
| AT4G01110 | unknown protein | 62.5961 | 0.387963 | -7.33401 | 0.0001 | 0.00123775 |
| AT1G08990 | PGSIP5, plant glycogenin-like starch initiation protein 5 | 62.0058 | 0.388489 | -7.31838 | 0.00005 | 0.00070028 |
| AT5G51270 | U-box domain-containing protein kinase family protein | 31.5351 | 0.198535 | -7.31142 | 0.0001 | 0.00123775 |
| AT5G58010 | LRL3, LJRHL1-like 3 | 273.512 | 1.80494 | -7.24351 | 0.00005 | 0.00070028 |
| AT3G49960 | Peroxidase superfamily protein | 159.373 | 1.06339 | -7.22759 | 0.00005 | 0.00070028 |
| AT4G34580 | COW1, SRH1, Sec14p-like phosphatidylinositol transfer family protein | 218.104 | 1.47609 | -7.20709 | 0.00005 | 0.00070028 |
| AT3G51350 | Eukaryotic aspartyl protease family protein | 38.8901 | 0.274518 | -7.14636 | 0.00005 | 0.00070028 |
| AT4G38390 | RHS17, root hair specific 17 | 36.6331 | 0.260206 | -7.13735 | 0.0001 | 0.00123775 |
| AT1G70460 | RHS10, root hair specific 10 | 88.1011 | 0.641957 | -7.10054 | 0.00005 | 0.00070028 |
| AT5G62310 | IRE, AGC (cAMP-dependent, cGMP-dependent and protein kinase C) kinase family protein | 64.0072 | 0.516494 | -6.95334 | 0.00005 | 0.00070028 |
| AT4G29180 | RHS16, root hair specific 16 | 55.3506 | 0.448309 | -6.94796 | 0.00005 | 0.00070028 |
| AT5G17820 | Peroxidase superfamily protein | 1305.01 | 10.998 | -6.89067 | 0.00005 | 0.00070028 |
| AT1G27740 | RSL4, root hair defective 6-like 4 | 254.49 | 2.16234 | -6.87888 | 0.00005 | 0.00070028 |
| AT1G09170 | P-loop nucleoside triphosphate hydrolases superfamily protein with CH (Calponin Homology) domain | 24.029 | 0.205787 | -6.86748 | 0.00005 | 0.00070028 |
| AT4G30320 | CAP (Cysteine-rich secretory proteins, Antigen 5, and Pathogenesis-related 1 protein) superfamily protein | 89.776 | 0.843779 | -6.73332 | 0.00465 | 0.0223951 |
| AT5G49870 | Mannose-binding lectin superfamily protein | 31.8792 | 0.309214 | -6.68786 | 0.00005 | 0.00070028 |
| AT1G51860 | Leucine-rich repeat protein kinase family protein | 8.1714 | 0.0812391 | -6.65226 | 0.0093 | 0.0372979 |
| AT5G65160 | tetratricopeptide repeat (TPR)-containing protein | 74.2567 | 0.744421 | -6.64026 | 0.00005 | 0.00070028 |
| AT4G02830 | unknown protein | 34.9084 | 0.352575 | -6.6295 | 0.00155 | 0.009918 |
| AT5G01280 | BEST Arabidopsis thaliana protein match is: proline-rich family protein (TAIR:AT3G09000.1) | 52.2871 | 0.534523 | -6.61206 | 0.00005 | 0.00070028 |
| AT5G61550 | U-box domain-containing protein kinase family protein | 35.9846 | 0.372456 | -6.59417 | 0.00005 | 0.00070028 |
| AT4G25220 | RHS15, root hair specific 15 | 24.1657 | 0.254417 | -6.56962 | 0.0007 | 0.00547072 |
| AT2G17890 | CPK16, calcium-dependent protein kinase 16 | 8.41945 | 0.0946467 | -6.47503 | 0.00895 | 0.0364179 |
| AT2G45750 | S-adenosyl-L-methionine-dependent methyltransferases superfamily protein | 142.026 | 1.61829 | -6.45554 | 0.00005 | 0.00070028 |
| AT4G25090 | Riboflavin synthase-like superfamily protein | 62.3366 | 0.714573 | -6.44686 | 0.00005 | 0.00070028 |
| AT1G07795 | unknown protein | 47.4163 | 0.554259 | -6.41868 | 0.0016 | 0.0101494 |
| AT4G34380 | Transducin/WD40 repeat-like superfamily protein | 8.24351 | 0.0984524 | -6.38769 | 0.00915 | 0.0369056 |
| AT5G25810 | tny, Integrase-type DNA-binding superfamily protein | 21.4022 | 0.257057 | -6.37953 | 0.00775 | 0.0327601 |
| AT2G37820 | Cysteine/Histidine-rich C1 domain family protein | 17.8879 | 0.221005 | -6.33876 | 0.01175 | 0.0443994 |
| AT1G34760 | GF14 OMICRON, GRF11, RHS5, general regulatory factor 11 | 50.7197 | 0.636707 | -6.31577 | 0.00005 | 0.00070028 |
| AT4G25940 | ENTH/ANTH/VHS superfamily protein | 19.5987 | 0.246851 | -6.31097 | 0.00005 | 0.00070028 |
| AT1G12550 | D-isomer specific 2-hydroxyacid dehydrogenase family protein | 81.9584 | 1.03544 | -6.30658 | 0.00005 | 0.00070028 |
| AT5G21080 | Uncharacterized protein | 30.2492 | 0.410728 | -6.20257 | 0.00005 | 0.00070028 |
| AT5G65090 | BST1, DER4, MRH3, DNAse I-like superfamily protein | 41.273 | 0.593343 | -6.12019 | 0.00005 | 0.00070028 |
| AT4G25110 | AtMC2, MC2, metacaspase 2 | 25.4734 | 0.366441 | -6.11927 | 0.00005 | 0.00070028 |
| AT3G18450 | PLAC8 family protein | 26.2681 | 0.378257 | -6.1178 | 0.0037 | 0.0188077 |
| AT1G01750 | ADF11, actin depolymerizing factor 11 | 723.49 | 10.7564 | -6.07171 | 0.00005 | 0.00070028 |
| AT3G21340 | Leucine-rich repeat protein kinase family protein | 83.7404 | 1.26439 | -6.04941 | 0.00005 | 0.00070028 |
| AT2G30660 | ATP-dependent caseinolytic (Clp) protease/crotonase family protein | 24.6728 | 0.377473 | -6.0304 | 0.00015 | 0.00170235 |
| AT1G10385 | Vps51/Vps67 family (components of vesicular transport) protein | 8.71123 | 0.133365 | -6.02942 | 0.0008 | 0.00603091 |
| AT4G14780 | Protein kinase superfamily protein | 11.7601 | 0.181057 | -6.02132 | 0.002 | 0.0119879 |
| AT3G54870 | ARK1, CAE1, MRH2, Armadillo/beta-catenin repeat family protein / kinesin motor family protein | 48.081 | 0.745398 | -6.01131 | 0.00005 | 0.00070028 |
| AT3G47050 | Glycosyl hydrolase family protein | 10.9459 | 0.183671 | -5.89713 | 0.00005 | 0.00070028 |
| AT2G38500 | 2-oxoglutarate (2OG) and Fe(II)-dependent oxygenase superfamily protein | 91.0314 | 1.52791 | -5.89674 | 0.00005 | 0.00070028 |
| AT3G07900 | O-fucosyltransferase family protein | 32.1397 | 0.546227 | -5.87871 | 0.00005 | 0.00070028 |
| AT5G15600 | SP1L4, SPIRAL1-like4 | 148.905 | 2.58613 | -5.84745 | 0.00005 | 0.00070028 |
| AT1G53680 | ATGSTU28, GSTU28, glutathione S-transferase TAU 28 | 304.142 | 5.34615 | -5.8301 | 0.00005 | 0.00070028 |
| AT5G42785 | unknown protein | 80.0565 | 1.42305 | -5.81396 | 0.00005 | 0.00070028 |
| AT2G20030 | RING/U-box superfamily protein | 8.41905 | 0.150287 | -5.80787 | 0.00895 | 0.0364179 |
| AT2G03360 | Glycosyltransferase family 61 protein | 6.24031 | 0.112152 | -5.79809 | 0.0102 | 0.0400002 |
| AT5G12050 | unknown protein | 184.136 | 3.34175 | -5.78402 | 0.00005 | 0.00070028 |
| AT4G22217 | Arabidopsis defensin-like protein | 349.124 | 6.59131 | -5.72703 | 0.00005 | 0.00070028 |
| AT4G21200 | ATGA2OX8, GA2OX8, gibberellin 2-oxidase 8 | 11.7754 | 0.22266 | -5.72479 | 0.0034 | 0.01771 |
| AT4G08450 | Disease resistance protein (TIR-NBS-LRR class) family | 7.34767 | 0.139961 | -5.71419 | 0.00005 | 0.00070028 |
| AT1G18420 | Aluminium activated malate transporter family protein | 55.8597 | 1.08416 | -5.68716 | 0.00005 | 0.00070028 |
| AT1G04280 | P-loop containing nucleoside triphosphate hydrolases superfamily protein | 192.645 | 3.74287 | -5.68566 | 0.00005 | 0.00070028 |
| AT2G17590 | Cysteine/Histidine-rich C1 domain family protein | 4.31448 | 0.0844839 | -5.67437 | 0.00825 | 0.0342522 |
| AT1G29230 | ATCIPK18, ATWL1, CIPK18, SnRK3.20, WL1, CBL-interacting protein kinase 18 | 21.9501 | 0.435261 | -5.6562 | 0.00005 | 0.00070028 |
| AT5G14330 | unknown protein | 469.906 | 9.38705 | -5.64556 | 0.00005 | 0.00070028 |
| AT1G73860 | P-loop containing nucleoside triphosphate hydrolases superfamily protein | 70.0785 | 1.40914 | -5.63609 | 0.00005 | 0.00070028 |
| AT4G30670 | Putative membrane lipoprotein | 235.479 | 4.89032 | -5.58953 | 0.00005 | 0.00070028 |
| AT2G05160 | CCCH-type zinc fingerfamily protein with RNA-binding domain | 29.1287 | 0.614505 | -5.56687 | 0.00005 | 0.00070028 |
| AT4G25160 | U-box domain-containing protein kinase family protein | 19.6735 | 0.418077 | -5.55634 | 0.00005 | 0.00070028 |
| AT1G70170 | MMP, matrix metalloproteinase | 44.6145 | 0.961701 | -5.53578 | 0.00005 | 0.00070028 |
| AT1G70720 | Plant invertase/pectin methylesterase inhibitor superfamily protein | 15.9509 | 0.345402 | -5.52921 | 0.00225 | 0.0130442 |
| AT4G37390 | AUR3, BRU6, GH3-2, GH3.2, YDK1, Auxin-responsive GH3 family protein | 172.489 | 3.74332 | -5.52604 | 0.00005 | 0.00070028 |
| AT4G29800 | PLA IVD, PLP8, PATATIN-like protein 8 | 28.7895 | 0.638612 | -5.49446 | 0.00005 | 0.00070028 |
| AT4G18640 | MRH1, Leucine-rich repeat protein kinase family protein | 85.9666 | 1.95314 | -5.45991 | 0.00005 | 0.00070028 |
| AT1G55290 | 2-oxoglutarate (2OG) and Fe(II)-dependent oxygenase superfamily protein | 21.3654 | 0.487674 | -5.45322 | 0.00005 | 0.00070028 |
| AT3G50130 | Plant protein of unknown function (DUF247) | 10.1628 | 0.237054 | -5.42194 | 0.00015 | 0.00170235 |
| AT1G16440 | RSH3, root hair specific 3 | 22.044 | 0.524572 | -5.3931 | 0.00005 | 0.00070028 |
| AT5G07080 | HXXXD-type acyl-transferase family protein | 106.986 | 2.56867 | -5.38025 | 0.00005 | 0.00070028 |
| AT2G37670 | Transducin/WD40 repeat-like superfamily protein | 40.673 | 0.994725 | -5.35363 | 0.00005 | 0.00070028 |
| AT3G51570 | Disease resistance protein (TIR-NBS-LRR class) family | 1.95741 | 0.0481138 | -5.34635 | 0.0132 | 0.0483114 |
| AT5G65100 | Ethylene insensitive 3 family protein | 26.583 | 0.65571 | -5.3413 | 0.00005 | 0.00070028 |
| AT4G13310 | CYP71A20, cytochrome P450, family 71, subfamily A, polypeptide 20 | 6.84932 | 0.169303 | -5.33828 | 0.0013 | 0.00867426 |
| AT2G34910 | BEST Arabidopsis thaliana protein match is: root hair specific 4 (TAIR:AT1G30850.1) | 587.714 | 14.9774 | -5.29425 | 0.00005 | 0.00070028 |
| AT5G21120 | EIL2, ETHYLENE-INSENSITIVE3-like 2 | 4.37142 | 0.115572 | -5.24125 | 0.0041 | 0.0203755 |
| AT2G02250 | AtPP2-B2, PP2-B2, phloem protein 2-B2 | 24.4822 | 0.648693 | -5.23805 | 0.0001 | 0.00123775 |
| AT5G46360 | ATKCO3, KCO3, Ca2+ activated outward rectifying K+ channel 3 | 7.82314 | 0.209611 | -5.22196 | 0.0109 | 0.0419234 |
| AT1G48640 | Transmembrane amino acid transporter family protein | 42.8959 | 1.15054 | -5.22046 | 0.00005 | 0.00070028 |
| AT4G21745 | PAK-box/P21-Rho-binding family protein | 7.52484 | 0.204376 | -5.20236 | 0.00635 | 0.0283169 |
| AT5G61350 | Protein kinase superfamily protein | 30.4309 | 0.832928 | -5.1912 | 0.00005 | 0.00070028 |
| AT5G17390 | Adenine nucleotide alpha hydrolases-like superfamily protein | 59.2781 | 1.62984 | -5.1847 | 0.00005 | 0.00070028 |
| AT5G07780 | Actin-binding FH2 (formin homology 2) family protein | 5.2055 | 0.144088 | -5.17501 | 0.00175 | 0.0108609 |
| AT5G45840 | Leucine-rich repeat protein kinase family protein | 30.2517 | 0.839383 | -5.17155 | 0.00005 | 0.00070028 |
| AT5G23270 | ATSTP11, STP11, sugar transporter 11 | 3.49755 | 0.0984643 | -5.1506 | 0.0086 | 0.0353203 |
| AT2G15990.1 | transposable element gene | 34.2799 | 0.978216 | -5.13107 | 0.00895 | 0.0364179 |
| AT1G29020 | Calcium-binding EF-hand family protein | 52.5806 | 1.50385 | -5.1278 | 0.00005 | 0.00070028 |
| AT4G13440 | Calcium-binding EF-hand family protein | 106.024 | 3.03328 | -5.12736 | 0.00005 | 0.00070028 |
| AT2G19050 | GDSL-like Lipase/Acylhydrolase superfamily protein | 9.63236 | 0.278971 | -5.1097 | 0.00575 | 0.0263656 |
| AT3G01420 | ALPHA-DOX1, DIOX1, DOX1, PADOX-1, Peroxidase superfamily protein | 12.4787 | 0.361786 | -5.10818 | 0.00005 | 0.00070028 |
| AT3G45530 | Cysteine/Histidine-rich C1 domain family protein | 3.21827 | 0.0937341 | -5.10157 | 0.01285 | 0.0472926 |
| AT4G16920 | Disease resistance protein (TIR-NBS-LRR class) family | 2.87594 | 0.0840651 | -5.09638 | 0.00045 | 0.003919 |
| AT3G07490 | AGD11, ARF-GAP domain 11 | 38.8904 | 1.13937 | -5.0931 | 0.00085 | 0.00630464 |
| AT1G58320 | PLAC8 family protein | 32.1595 | 0.951443 | -5.07898 | 0.00225 | 0.0130442 |
| AT3G51540 | unknown protein | 140.796 | 4.18419 | -5.07251 | 0.00005 | 0.00070028 |
| AT3G23190 | HR-like lesion-inducing protein-related | 366.307 | 10.9146 | -5.06871 | 0.00005 | 0.00070028 |
| AT1G48745 | unknown protein | 34.1412 | 1.02216 | -5.06182 | 0.00015 | 0.00170235 |
| AT2G25240 | Serine protease inhibitor (SERPIN) family protein | 52.9154 | 1.59392 | -5.05303 | 0.00005 | 0.00070028 |
| AT1G21360 | GLTP2, glycolipid transfer protein 2 | 43.6204 | 1.34333 | -5.02112 | 0.00005 | 0.00070028 |
| AT5G24310 | ABIL3, ABL interactor-like protein 3 | 101.958 | 3.18457 | -5.00073 | 0.00005 | 0.00070028 |
| AT1G23720 | Proline-rich extensin-like family protein | 1454.68 | 45.5663 | -4.99659 | 0.00005 | 0.00070028 |
| AT1G79250 | AGC1.7, AGC kinase 1.7 | 3.7035 | 0.116275 | -4.99327 | 0.00185 | 0.0113253 |
| AT1G63930 | ROH1, from the Czech 'roh' meaning 'corner' | 43.3653 | 1.36232 | -4.99241 | 0.00005 | 0.00070028 |
| AT3G13782 | NAP1;4, NFA04, NFA4, nucleosome assembly protein1;4 | 209.37 | 6.5882 | -4.99003 | 0.00005 | 0.00070028 |
| AT2G02300 | AtPP2-B5, PP2-B5, phloem protein 2-B5 | 4.93553 | 0.157395 | -4.97075 | 0.00635 | 0.0283169 |
| AT1G66570 | ATSUC7, SUC7, sucrose-proton symporter 7 | 31.2731 | 1.0048 | -4.95994 | 0.00005 | 0.00070028 |
| AT3G05155 | Major facilitator superfamily protein | 62.3995 | 2.00907 | -4.95693 | 0.00005 | 0.00070028 |
| AT1G19230 | Riboflavin synthase-like superfamily protein | 51.3866 | 1.65536 | -4.95617 | 0.00005 | 0.00070028 |
| AT4G22666 | Bifunctional inhibitor/lipid-transfer protein/seed storage 2S albumin superfamily protein | 270.642 | 8.73029 | -4.95421 | 0.00005 | 0.00070028 |
| AT1G57560 | AtMYB50, MYB50, myb domain protein 50 | 72.101 | 2.33254 | -4.95005 | 0.00005 | 0.00070028 |
| AT3G21180 | ACA9, ATACA9, autoinhibited Ca(2+)-ATPase 9 | 115.613 | 3.74594 | -4.94783 | 0.00005 | 0.00070028 |
| AT3G48940 | Remorin family protein | 49.2343 | 1.64026 | -4.90767 | 0.00005 | 0.00070028 |
| AT1G04700 | PB1 domain-containing protein tyrosine kinase | 49.9354 | 1.67292 | -4.89962 | 0.00005 | 0.00070028 |
| AT1G05020 | ENTH/ANTH/VHS superfamily protein | 4.18853 | 0.140718 | -4.89556 | 0.00075 | 0.00575475 |
| AT3G17600 | IAA31, indole-3-acetic acid inducible 31 | 12.1628 | 0.409842 | -4.89126 | 0.0016 | 0.0101494 |
| AT1G65060 | 4CL3, 4-coumarate:CoA ligase 3 | 4.04638 | 0.136674 | -4.88782 | 0.0037 | 0.0188077 |
| AT5G62280 | Protein of unknown function (DUF1442) | 105.288 | 3.58599 | -4.87583 | 0.00005 | 0.00070028 |
| AT3G43960 | Cysteine proteinases superfamily protein | 200.497 | 6.84545 | -4.87229 | 0.00005 | 0.00070028 |
| AT3G05800 | AIF1, AtBS1(activation-tagged BRI1 suppressor 1)-interacting factor 1 | 37.6626 | 1.29728 | -4.85957 | 0.00005 | 0.00070028 |
| AT4G19680 | ATIRT2, IRT2, iron regulated transporter 2 | 109.85 | 3.84865 | -4.83504 | 0.00005 | 0.00070028 |
| AT5G13930 | ATCHS, CHS, TT4, Chalcone and stilbene synthase family protein | 134.966 | 4.77061 | -4.82228 | 0.00005 | 0.00070028 |
| AT5G44480 | DUR, NAD(P)-binding Rossmann-fold superfamily protein | 74.7474 | 2.64666 | -4.81978 | 0.00005 | 0.00070028 |
| AT3G28550 | Proline-rich extensin-like family protein | 1748.86 | 62.0652 | -4.81649 | 0.0008 | 0.00603091 |
| AT5G63600 | ATFLS5, FLS5, flavonol synthase 5 | 82.4453 | 2.92904 | -4.81494 | 0.00005 | 0.00070028 |
| AT1G75620 | glyoxal oxidase-related protein | 2.79705 | 0.100012 | -4.80566 | 0.00635 | 0.0283169 |
| AT1G61795 | PAK-box/P21-Rho-binding family protein | 28.435 | 1.01884 | -4.80267 | 0.00005 | 0.00070028 |
| AT2G19060 | SGNH hydrolase-type esterase superfamily protein | 28.6291 | 1.04724 | -4.77283 | 0.00005 | 0.00070028 |
| AT1G73165 | CLE1, CLAVATA3/ESR-RELATED 1 | 922.191 | 34.1179 | -4.75647 | 0.00005 | 0.00070028 |
| AT4G29905 | unknown protein | 42.2351 | 1.56744 | -4.75196 | 0.00005 | 0.00070028 |
| AT4G24580 | REN1, Rho GTPase activation protein (RhoGAP) with PH domain | 70.9671 | 2.63631 | -4.75056 | 0.00005 | 0.00070028 |
| AT3G16390 | NSP3, nitrile specifier protein 3 | 353.889 | 13.2526 | -4.73895 | 0.00005 | 0.00070028 |
| AT4G09500 | UDP-Glycosyltransferase superfamily protein | 5.72847 | 0.215031 | -4.73553 | 0.00035 | 0.00327416 |
| AT2G20520 | FLA6, FASCICLIN-like arabinogalactan 6 | 49.4497 | 1.87766 | -4.71895 | 0.00005 | 0.00070028 |
| AT4G32950 | Protein phosphatase 2C family protein | 241.781 | 9.18321 | -4.71856 | 0.00005 | 0.00070028 |
| AT4G16600 | Nucleotide-diphospho-sugar transferases superfamily protein | 9.0648 | 0.345115 | -4.71513 | 0.0005 | 0.00424432 |
| AT5G25880 | ATNADP-ME3, NADP-ME3, NADP-malic enzyme 3 | 57.118 | 2.17689 | -4.7136 | 0.00005 | 0.00070028 |
| AT4G27290 | S-locus lectin protein kinase family protein | 39.0958 | 1.50421 | -4.69993 | 0.00005 | 0.00070028 |
| AT5G07450 | CYCP4;3, cyclin p4;3 | 64.7111 | 2.49592 | -4.69637 | 0.00005 | 0.00070028 |
| AT2G11270 | citrate synthase-related | 260.422 | 10.0694 | -4.6928 | 0.0024 | 0.0136815 |
| AT4G26260 | MIOX4, myo-inositol oxygenase 4 | 5.49002 | 0.213983 | -4.68125 | 0.0046 | 0.0222191 |
| AT4G07960 | ATCSLC12, CSLC12, CSLC12, Cellulose-synthase-like C12 | 87.0289 | 3.41639 | -4.67095 | 0.00005 | 0.00070028 |
| AT1G27140 | ATGSTU14, GST13, GSTU14, glutathione S-transferase tau 14 | 20.9489 | 0.825657 | -4.66519 | 0.00045 | 0.003919 |
| AT4G40010 | SNRK2-7, SNRK2.7, SRK2F, SNF1-related protein kinase 2.7 | 32.4091 | 1.28034 | -4.6618 | 0.00005 | 0.00070028 |
| AT4G18430 | AtRABA1e, RABA1e, RAB GTPase homolog A1E | 111.568 | 4.4472 | -4.64888 | 0.00005 | 0.00070028 |
| AT5G01250 | alpha 1,4-glycosyltransferase family protein | 5.6873 | 0.227479 | -4.64394 | 0.0006 | 0.00487984 |
| AT3G61550 | RING/U-box superfamily protein | 7.37236 | 0.295112 | -4.64279 | 0.0061 | 0.0274993 |
| AT4G30460 | glycine-rich protein | 49.893 | 2.00971 | -4.63378 | 0.00005 | 0.00070028 |
| AT1G66470 | RHD6, ROOT HAIR DEFECTIVE6 | 161.46 | 6.58115 | -4.61669 | 0.00005 | 0.00070028 |
| AT5G11070 | unknown protein | 294.83 | 12.2172 | -4.59289 | 0.00005 | 0.00070028 |
| AT4G37070 | AtPLAIVA, PLA IVA, PLP1, Acyl transferase/acyl hydrolase/lysophospholipase superfamily protein | 64.281 | 2.6652 | -4.59208 | 0.00005 | 0.00070028 |
| AT5G05840 | Protein of unknown function (DUF620) | 8.3297 | 0.34828 | -4.57995 | 0.0004 | 0.00359419 |
| AT3G59830 | Integrin-linked protein kinase family | 21.766 | 0.914138 | -4.57352 | 0.00005 | 0.00070028 |
| AT2G29750 | UGT71C1, UDP-glucosyl transferase 71C1 | 96.4608 | 4.05632 | -4.5717 | 0.00005 | 0.00070028 |
| AT1G33090 | MATE efflux family protein | 20.4393 | 0.862959 | -4.56591 | 0.00005 | 0.00070028 |
| AT5G61260 | Plant calmodulin-binding protein-related | 30.5146 | 1.31106 | -4.54069 | 0.00005 | 0.00070028 |
| AT2G24840 | AGL61, DIA, AGAMOUS-like 61 | 62.0978 | 2.70141 | -4.52276 | 0.00005 | 0.00070028 |
| AT4G20110 | BP80-3;1, VSR3;1, VSR7, VACUOLAR SORTING RECEPTOR 7 | 35.0237 | 1.52904 | -4.51763 | 0.00005 | 0.00070028 |
| AT1G44020 | Cysteine/Histidine-rich C1 domain family protein | 1.82474 | 0.0802579 | -4.5069 | 0.0046 | 0.0222191 |
| AT1G62320 | ERD (early-responsive to dehydration stress) family protein | 53.8165 | 2.38029 | -4.49884 | 0.00005 | 0.00070028 |
| AT4G22600 | unknown protein | 7.47183 | 0.331342 | -4.49507 | 0.0038 | 0.0192125 |
| AT1G74830 | Protein of unknown function, DUF593 | 41.5366 | 1.84494 | -4.49274 | 0.00005 | 0.00070028 |
| AT4G10010 | Protein kinase superfamily protein | 2.0297 | 0.090408 | -4.48867 | 0.0041 | 0.0203755 |
| AT5G53830 | VQ motif-containing protein | 9.82475 | 0.442394 | -4.47302 | 0.0009 | 0.0065867 |
| AT3G09240 | Protein kinase protein with tetratricopeptide repeat domain | 11.5432 | 0.51995 | -4.47252 | 0.00005 | 0.00070028 |
| AT3G26500 | PIRL2, plant intracellular ras group-related LRR 2 | 38.0725 | 1.7151 | -4.47239 | 0.00005 | 0.00070028 |
| AT5G06800 | myb-like HTH transcriptional regulator family protein | 104.978 | 4.74244 | -4.46832 | 0.00005 | 0.00070028 |
| AT1G65610 | ATGH9A2, KOR2, Six-hairpin glycosidases superfamily protein | 165.887 | 7.60339 | -4.44741 | 0.00005 | 0.00070028 |
| AT4G14390 | Ankyrin repeat family protein | 10.4832 | 0.480931 | -4.44611 | 0.00005 | 0.00070028 |
| AT4G03330 | ATSYP123, SYP123, syntaxin of plants 123 | 32.7223 | 1.51902 | -4.42906 | 0.0027 | 0.0149318 |
| AT1G64930 | CYP89A7, cytochrome P450, family 87, subfamily A, polypeptide 7 | 5.88106 | 0.273518 | -4.42637 | 0.00035 | 0.00327416 |
| AT2G45220 | Plant invertase/pectin methylesterase inhibitor superfamily | 184.446 | 8.61363 | -4.42043 | 0.00005 | 0.00070028 |
| AT2G34655 | unknown protein | 7.72599 | 0.363566 | -4.40943 | 0.00175 | 0.0108609 |
| AT2G21880 | ATRAB7A, ATRABG2, RAB7A, RAB GTPase homolog 7A | 84.6027 | 3.98521 | -4.40798 | 0.00005 | 0.00070028 |
| AT5G09440 | EXL4, EXORDIUM like 4 | 89.7397 | 4.259 | -4.39716 | 0.00005 | 0.00070028 |
| AT1G16420 | ATMC8, MC8, metacaspase 8 | 5.31635 | 0.252483 | -4.39618 | 0.0009 | 0.0065867 |
| AT2G01540 | Calcium-dependent lipid-binding (CaLB domain) family protein | 474.404 | 22.5354 | -4.39585 | 0.00005 | 0.00070028 |
| AT3G06370 | ATNHX4, NHX4, sodium hydrogen exchanger 4 | 19.9797 | 0.952007 | -4.39142 | 0.00005 | 0.00070028 |
| AT5G48320 | Cysteine/Histidine-rich C1 domain family protein | 1.90417 | 0.0914807 | -4.37955 | 0.0012 | 0.00815259 |
| AT1G64380 | Integrase-type DNA-binding superfamily protein | 11.4781 | 0.554446 | -4.3717 | 0.0001 | 0.00123775 |
| AT3G01760 | Transmembrane amino acid transporter family protein | 9.59626 | 0.465696 | -4.36501 | 0.00005 | 0.00070028 |
| AT2G29740 | UGT71C2, UDP-glucosyl transferase 71C2 | 23.2408 | 1.13071 | -4.36136 | 0.00005 | 0.00070028 |
| AT3G04900 | Heavy metal transport/detoxification superfamily protein | 10.6306 | 0.518696 | -4.35719 | 0.0026 | 0.0145003 |
| AT4G25433 | peptidoglycan-binding LysM domain-containing protein | 10.3498 | 0.508522 | -4.34715 | 0.0025 | 0.0140894 |
| AT2G34500 | CYP710A1, cytochrome P450, family 710, subfamily A, polypeptide 1 | 76.233 | 3.75831 | -4.34226 | 0.00005 | 0.00070028 |
| AT4G26330 | ATSBT3.18, UNE17, Subtilisin-like serine endopeptidase family protein | 2.04589 | 0.101208 | -4.33734 | 0.00215 | 0.0126571 |
| AT1G45140 | transposable element gene | 3.4818 | 0.173154 | -4.3297 | 0.00005 | 0.00070028 |
| AT2G28440 | proline-rich family protein | 28.0882 | 1.40113 | -4.3253 | 0.00005 | 0.00070028 |
| AT2G34810 | FAD-binding Berberine family protein | 12.8607 | 0.647047 | -4.31296 | 0.00005 | 0.00070028 |
| AT4G33020 | ATZIP9, ZIP9, ZIP metal ion transporter family | 10.8718 | 0.54834 | -4.30938 | 0.0004 | 0.00359419 |
| AT4G11390 | Cysteine/Histidine-rich C1 domain family protein | 5.36556 | 0.271666 | -4.30382 | 0.0003 | 0.00292382 |
| AT1G24320 | Six-hairpin glycosidases superfamily protein | 40.1752 | 2.04551 | -4.29578 | 0.00145 | 0.00940576 |
| AT2G34180 | ATWL2, CIPK13, SnRK3.7, WL2, CBL-interacting protein kinase 13 | 65.395 | 3.33008 | -4.29555 | 0.00275 | 0.0151029 |
| AT5G41280 | Receptor-like protein kinase-related family protein | 69.5715 | 3.55533 | -4.29044 | 0.00005 | 0.00070028 |
| AT1G49310 | unknown protein | 48.4549 | 2.48173 | -4.28723 | 0.0004 | 0.00359419 |
| AT4G19690 | ATIRT1, IRT1, iron-regulated transporter 1 | 3.32618 | 0.171025 | -4.28159 | 0.0099 | 0.0391493 |
| AT1G30050 | unknown protein | 4.81614 | 0.250021 | -4.26775 | 0.00075 | 0.00575475 |
| AT1G72200 | RING/U-box superfamily protein | 26.4992 | 1.38354 | -4.25951 | 0.00005 | 0.00070028 |
| AT5G13150 | ATEXO70C1, EXO70C1, exocyst subunit exo70 family protein C1 | 52.697 | 2.7557 | -4.25723 | 0.00005 | 0.00070028 |
| AT1G03106 | unknown protein | 16.4945 | 0.865869 | -4.25169 | 0.00025 | 0.00253767 |
| AT3G61560 | Reticulon family protein | 226.411 | 11.9469 | -4.24424 | 0.00005 | 0.00070028 |
| AT5G21950 | alpha/beta-Hydrolases superfamily protein | 10.4381 | 0.552717 | -4.23917 | 0.00035 | 0.00327416 |
| AT5G06990 | Protein of unknown function, DUF617 | 38.9156 | 2.06317 | -4.23742 | 0.00005 | 0.00070028 |
| AT5G07150 | Leucine-rich repeat protein kinase family protein | 13.2811 | 0.705037 | -4.23553 | 0.00005 | 0.00070028 |
| AT1G59850 | ARM repeat superfamily protein | 9.36163 | 0.500439 | -4.22549 | 0.0053 | 0.0248135 |
| AT4G17340 | DELTA-TIP2, TIP2;2, tonoplast intrinsic protein 2;2 | 81.8083 | 4.46908 | -4.1942 | 0.00005 | 0.00070028 |
| AT2G16000.1 | transposable element gene | 1.50571 | 0.082748 | -4.18558 | 0.0006 | 0.00487984 |
| AT1G55390 | Cysteine/Histidine-rich C1 domain family protein | 1.2962 | 0.0723664 | -4.16283 | 0.00915 | 0.0369056 |
| AT4G11230 | Riboflavin synthase-like superfamily protein | 24.4326 | 1.37182 | -4.15465 | 0.00005 | 0.00070028 |
| AT5G01200 | Duplicated homeodomain-like superfamily protein | 10.0362 | 0.566426 | -4.14718 | 0.00045 | 0.003919 |
| AT1G18940 | Nodulin-like / Major Facilitator Superfamily protein | 15.9634 | 0.904401 | -4.14167 | 0.00005 | 0.00070028 |
| AT2G38790 | unknown protein | 89.2405 | 5.07359 | -4.13662 | 0.00005 | 0.00070028 |
| AT2G28460 | Cysteine/Histidine-rich C1 domain family protein | 21.3637 | 1.21562 | -4.1354 | 0.00005 | 0.00070028 |
| AT1G08320 | bZIP21, TGA9, bZIP transcription factor family protein | 11.6862 | 0.675343 | -4.11304 | 0.00005 | 0.00070028 |
| AT2G21020 | pseudogene, major intrinsic protein (MIP) family, contains Pfam profile: MIP PF00230; blastp match of 61% identity and 1.8e-40 P-value to PIR|S01444|S01444 nodulin-26 precursor - soybean | 2.48189 | 0.143677 | -4.11053 | 0.0044 | 0.0214942 |
| AT3G18170 | Glycosyltransferase family 61 protein | 27.7438 | 1.61814 | -4.09976 | 0.00005 | 0.00070028 |
| AT1G67105 | other RNA | 6.45057 | 0.377261 | -4.09579 | 0.0025 | 0.0140894 |
| AT5G62720 | Integral membrane HPP family protein | 17.815 | 1.04459 | -4.09208 | 0.00005 | 0.00070028 |
| AT1G53990 | GLIP3, GDSL-motif lipase 3 | 5.16993 | 0.303195 | -4.09183 | 0.00165 | 0.0103801 |
| AT3G51330 | Eukaryotic aspartyl protease family protein | 119.334 | 7.01409 | -4.0886 | 0.00005 | 0.00070028 |
| AT3G60540 | Preprotein translocase Sec, Sec61-beta subunit protein | 35.6917 | 2.10156 | -4.08606 | 0.0004 | 0.00359419 |
| AT1G68620 | alpha/beta-Hydrolases superfamily protein | 10.5542 | 0.621648 | -4.08557 | 0.00005 | 0.00070028 |
| AT2G26400 | ARD, ARD3, ATARD3, acireductone dioxygenase 3 | 38.9796 | 2.29614 | -4.08543 | 0.00005 | 0.00070028 |
| AT4G38620 | ATMYB4, MYB4, myb domain protein 4 | 15.3817 | 0.906201 | -4.08524 | 0.00005 | 0.00070028 |
| AT2G17830 | F-box and associated interaction domains-containing protein | 59.5139 | 3.51293 | -4.08248 | 0.00005 | 0.00070028 |
| AT3G49700 | ACS9, AtACS9, ETO3, 1-aminocyclopropane-1-carboxylate synthase 9 | 5.33736 | 0.315157 | -4.08199 | 0.0009 | 0.0065867 |
| AT1G01780 | GATA type zinc finger transcription factor family protein | 72.1454 | 4.27052 | -4.07842 | 0.00005 | 0.00070028 |
| AT3G16210 | F-box family protein | 9.59477 | 0.569167 | -4.07532 | 0.00045 | 0.003919 |
| AT5G04390 | C2H2-type zinc finger family protein | 2.804 | 0.166354 | -4.07516 | 0.0079 | 0.0332028 |
| AT4G30270 | MERI-5, MERI5B, SEN4, XTH24, xyloglucan endotransglucosylase/hydrolase 24 | 22.165 | 1.31669 | -4.07329 | 0.00005 | 0.00070028 |
| AT1G58037 | Cysteine/Histidine-rich C1 domain family protein | 2.63675 | 0.157259 | -4.06754 | 0.00165 | 0.0103801 |
| AT2G02620 | Cysteine/Histidine-rich C1 domain family protein | 11.374 | 0.682936 | -4.05785 | 0.00005 | 0.00070028 |
| AT5G01215 | other RNA | 19.4062 | 1.17744 | -4.0428 | 0.00005 | 0.00070028 |
| AT2G35730 | Heavy metal transport/detoxification superfamily protein | 64.8507 | 3.94136 | -4.04036 | 0.00005 | 0.00070028 |
| AT2G28110 | FRA8, IRX7, Exostosin family protein | 4.15433 | 0.253887 | -4.03236 | 0.00105 | 0.00735817 |
| AT1G74080 | ATMYB122, MYB122, myb domain protein 122 | 4.99956 | 0.306451 | -4.02807 | 0.0011 | 0.00759303 |
| AT1G61840 | Cysteine/Histidine-rich C1 domain family protein | 4.18771 | 0.256998 | -4.02633 | 0.00065 | 0.00516972 |
| AT2G04680 | Cysteine/Histidine-rich C1 domain family protein | 8.95639 | 0.551561 | -4.02133 | 0.0001 | 0.00123775 |
| AT5G44900 | Toll-Interleukin-Resistance (TIR) domain family protein | 4.17558 | 0.257429 | -4.01973 | 0.00945 | 0.0377542 |
| AT1G63245 | CLE14, CLAVATA3/ESR-RELATED 14 | 1507.52 | 93.2893 | -4.01432 | 0.00005 | 0.00070028 |
| AT4G21215 | unknown protein | 16.8602 | 1.05 | -4.00517 | 0.00005 | 0.00070028 |
| AT4G35200 | Arabidopsis protein of unknown function (DUF241) | 29.5242 | 1.8513 | -3.99529 | 0.00005 | 0.00070028 |
| AT5G12880 | proline-rich family protein | 160.477 | 10.1072 | -3.98891 | 0.00005 | 0.00070028 |
| AT2G15980 | Tetratricopeptide repeat (TPR)-like superfamily protein | 39.4528 | 2.4858 | -3.98834 | 0.00005 | 0.00070028 |
| AT4G13860 | RNA-binding (RRM/RBD/RNP motifs) family protein | 59.7131 | 3.76483 | -3.98739 | 0.00565 | 0.0260215 |
| AT4G22070 | ATWRKY31, WRKY31, WRKY DNA-binding protein 31 | 19.1717 | 1.20887 | -3.98725 | 0.00005 | 0.00070028 |
| AT5G13990 | ATEXO70C2, EXO70C2, exocyst subunit exo70 family protein C2 | 114.103 | 7.30302 | -3.9657 | 0.00005 | 0.00070028 |
| AT4G22214 | Defensin-like (DEFL) family protein | 927.985 | 59.3972 | -3.96563 | 0.00005 | 0.00070028 |
| AT2G34960 | CAT5, cationic amino acid transporter 5 | 4.33775 | 0.278032 | -3.96362 | 0.0009 | 0.0065867 |
| AT3G53820 | C2H2 and C2HC zinc fingers superfamily protein | 231.311 | 14.896 | -3.95684 | 0.00005 | 0.00070028 |
| AT4G38830 | CRK26, cysteine-rich RLK (RECEPTOR-like protein kinase) 26 | 14.1427 | 0.913051 | -3.95322 | 0.00005 | 0.00070028 |
| AT3G14850 | TBL41, TRICHOME BIREFRINGENCE-LIKE 41 | 43.7417 | 2.83187 | -3.94918 | 0.00005 | 0.00070028 |
| AT5G44130 | FLA13, FASCICLIN-like arabinogalactan protein 13 precursor | 12.2916 | 0.797854 | -3.94541 | 0.00005 | 0.00070028 |
| AT5G40730 | AGP24, ATAGP24, arabinogalactan protein 24 | 2027.53 | 131.904 | -3.94216 | 0.00005 | 0.00070028 |
| AT5G56160 | Sec14p-like phosphatidylinositol transfer family protein | 14.8964 | 0.969393 | -3.94174 | 0.00005 | 0.00070028 |
| AT2G23620 | ATMES1, MES1, methyl esterase 1 | 19.9987 | 1.30218 | -3.9409 | 0.00005 | 0.00070028 |
| AT3G27510 | Cysteine/Histidine-rich C1 domain family protein | 2.11736 | 0.138215 | -3.93728 | 0.00475 | 0.0228046 |
| AT4G00460 | ATROPGEF3, ROPGEF3, RHO guanyl-nucleotide exchange factor 3 | 116.672 | 7.66071 | -3.92884 | 0.00005 | 0.00070028 |
| AT4G25070 | unknown protein | 77.9769 | 5.12659 | -3.92697 | 0.00005 | 0.00070028 |
| AT4G35660 | Arabidopsis protein of unknown function (DUF241) | 3.58664 | 0.23592 | -3.92626 | 0.00845 | 0.0348633 |
| AT1G76210 | Arabidopsis protein of unknown function (DUF241) | 36.7597 | 2.41801 | -3.92623 | 0.00005 | 0.00070028 |
| AT4G16350 | CBL6, SCABP2, calcineurin B-like protein 6 | 34.8977 | 2.29663 | -3.92554 | 0.00005 | 0.00070028 |
| AT1G18410 | P-loop containing nucleoside triphosphate hydrolases superfamily protein | 2.52524 | 0.166804 | -3.9202 | 0.0001 | 0.00123775 |
| AT4G31250 | Leucine-rich repeat protein kinase family protein | 39.6763 | 2.62096 | -3.92011 | 0.00005 | 0.00070028 |
| AT5G06090 | ATGPAT7, GPAT7, glycerol-3-phosphate acyltransferase 7 | 7.34847 | 0.4861 | -3.91812 | 0.00015 | 0.00170235 |
| AT1G05320 | function unknown | 69.93 | 4.65597 | -3.90876 | 0.00005 | 0.00070028 |
| AT5G45220 | Disease resistance protein (TIR-NBS-LRR class) family | 5.07928 | 0.338321 | -3.90816 | 0.00105 | 0.00735817 |
| AT1G33800 | Protein of unknown function (DUF579) | 216.503 | 14.4211 | -3.90813 | 0.00005 | 0.00070028 |
| AT1G63450 | RHS8, root hair specific 8 | 20.4915 | 1.37791 | -3.89447 | 0.00005 | 0.00070028 |
| AT2G31860 | pseudogene, poly (ADP-ribose) glycohydrolase, putative, contains Pfam domain, PF05028: poly (ADP-ribose) glycohydrolase (PARG); blastp match of 59% identity and 1.4e-19 P-value to EGAD|134505|143482 114E2.c protein {Drosophila melanogaster} | 2.91927 | 0.19662 | -3.89212 | 0.00025 | 0.00253767 |
| AT4G23550 | ATWRKY29, WRKY29, WRKY family transcription factor | 24.711 | 1.67022 | -3.88704 | 0.00005 | 0.00070028 |
| AT1G11160 | Transducin/WD40 repeat-like superfamily protein | 17.1499 | 1.1623 | -3.88315 | 0.00005 | 0.00070028 |
| AT5G01610 | Protein of unknown function, DUF538 | 180.271 | 12.2245 | -3.88232 | 0.00005 | 0.00070028 |
| AT3G56400 | ATWRKY70, WRKY70, WRKY DNA-binding protein 70 | 16.8807 | 1.14721 | -3.87918 | 0.00005 | 0.00070028 |
| AT1G34040 | Pyridoxal phosphate (PLP)-dependent transferases superfamily protein | 2.69736 | 0.183857 | -3.87489 | 0.00295 | 0.0159014 |
| AT1G30900 | BP80-3;3, VSR3;3, VSR6, VACUOLAR SORTING RECEPTOR 6 | 175.769 | 11.9986 | -3.87274 | 0.00005 | 0.00070028 |
| AT3G62100 | IAA30, indole-3-acetic acid inducible 30 | 139.472 | 9.59808 | -3.86109 | 0.00005 | 0.00070028 |
| AT1G61750 | Receptor-like protein kinase-related family protein | 9.87134 | 0.680327 | -3.85895 | 0.00005 | 0.00070028 |
| AT3G55120 | A11, CFI, TT5, Chalcone-flavanone isomerase family protein | 12.97 | 0.896791 | -3.85426 | 0.00025 | 0.00253767 |
| AT3G09020 | alpha 1,4-glycosyltransferase family protein | 18.227 | 1.26062 | -3.85387 | 0.00005 | 0.00070028 |
| AT1G14960 | Polyketide cyclase/dehydrase and lipid transport superfamily protein | 122.489 | 8.48455 | -3.85167 | 0.00005 | 0.00070028 |
| AT1G54740 | Protein of unknown function (DUF3049) | 14.8321 | 1.03078 | -3.84692 | 0.00005 | 0.00070028 |
| AT1G28600 | GDSL-like Lipase/Acylhydrolase superfamily protein | 4.93177 | 0.342918 | -3.84617 | 0.00065 | 0.00516972 |
| AT2G25810 | TIP4;1, tonoplast intrinsic protein 4;1 | 197.839 | 13.7724 | -3.84447 | 0.00005 | 0.00070028 |
| AT5G67620 | unknown protein | 116.588 | 8.13089 | -3.84186 | 0.00005 | 0.00070028 |
| AT5G10410 | ENTH/ANTH/VHS superfamily protein | 91.9798 | 6.41601 | -3.84157 | 0.00005 | 0.00070028 |
| AT3G03000 | EF hand calcium-binding protein family | 37.5296 | 2.62963 | -3.8351 | 0.00005 | 0.00070028 |
| AT1G64210 | Leucine-rich repeat protein kinase family protein | 2.32369 | 0.16328 | -3.831 | 0.00155 | 0.009918 |
| AT3G05920 | Heavy metal transport/detoxification superfamily protein | 169.788 | 11.9305 | -3.831 | 0.00005 | 0.00070028 |
| AT1G56550 | RXGT1, RhamnoGalacturonan speci&#64257;c Xylosyltransferase 1 | 70.9225 | 4.9953 | -3.8276 | 0.00005 | 0.00070028 |
| AT3G04010 | O-Glycosyl hydrolases family 17 protein | 428.945 | 30.225 | -3.82698 | 0.00005 | 0.00070028 |
| AT4G23070 | ATRBL7, RBL7, RHOMBOID-like protein 7 | 7.2282 | 0.511619 | -3.82049 | 0.00115 | 0.00787231 |
| AT5G53370 | ATPMEPCRF, PMEPCRF, pectin methylesterase PCR fragment F | 29.1557 | 2.06983 | -3.8162 | 0.00005 | 0.00070028 |
| AT3G04070 | anac047, NAC047, NAC domain containing protein 47 | 65.2786 | 4.64433 | -3.81307 | 0.00005 | 0.00070028 |
| AT1G29395 | COR413-TM1, COR413IM1, COR414-TM1, COLD REGULATED 314 INNER MEMBRANE 1 | 5.63284 | 0.402593 | -3.80647 | 0.0029 | 0.0157089 |
| AT4G20730.1 | transposable element gene | 5.51128 | 0.397336 | -3.79396 | 0.00005 | 0.00070028 |
| AT3G07195 | RPM1-interacting protein 4 (RIN4) family protein | 38.2359 | 2.76776 | -3.78814 | 0.00005 | 0.00070028 |
| AT3G23175 | HR-like lesion-inducing protein-related | 297.022 | 21.5645 | -3.78384 | 0.00005 | 0.00070028 |
| AT2G20670 | Protein of unknown function (DUF506) | 198.928 | 14.4747 | -3.78064 | 0.00005 | 0.00070028 |
| AT2G26420 | PIP5K3, 1-phosphatidylinositol-4-phosphate 5-kinase 3 | 8.57448 | 0.625113 | -3.77786 | 0.00005 | 0.00070028 |
| AT5G65800 | ACS5, ATACS5, CIN5, ETO2, ACC synthase 5 | 17.145 | 1.25001 | -3.77778 | 0.00005 | 0.00070028 |
| AT2G20620 | Protein of unknown function (DUF626) | 8.3447 | 0.609416 | -3.77536 | 0.001 | 0.00711095 |
| AT1G72890 | Disease resistance protein (TIR-NBS class) | 8.79457 | 0.64264 | -3.77453 | 0.00005 | 0.00070028 |
| AT4G10770 | ATOPT7, OPT7, oligopeptide transporter 7 | 30.6851 | 2.25224 | -3.7681 | 0.00005 | 0.00070028 |
| AT5G44610 | MAP18, PCAP2, microtubule-associated protein 18 | 327.958 | 24.1866 | -3.76123 | 0.00005 | 0.00070028 |
| AT5G41680 | Protein kinase superfamily protein | 35.4244 | 2.6138 | -3.76052 | 0.00485 | 0.0231612 |
| AT1G02380 | unknown protein | 26.3932 | 1.94938 | -3.75908 | 0.00005 | 0.00070028 |
| AT4G22758 | unknown protein | 64.7652 | 4.80327 | -3.75313 | 0.00005 | 0.00070028 |
| AT5G54240 | Protein of unknown function (DUF1223) | 8.12545 | 0.604209 | -3.74933 | 0.00085 | 0.00630464 |
| AT2G38640 | Protein of unknown function (DUF567) | 22.8906 | 1.70323 | -3.7484 | 0.00005 | 0.00070028 |
| AT2G46750 | D-arabinono-1,4-lactone oxidase family protein | 6.14732 | 0.458944 | -3.74357 | 0.00005 | 0.00070028 |
| AT1G78230 | Outer arm dynein light chain 1 protein | 18.663 | 1.39465 | -3.7422 | 0.00005 | 0.00070028 |
| AT1G61230 | Mannose-binding lectin superfamily protein | 2.43767 | 0.182278 | -3.74129 | 0.00335 | 0.0175049 |
| AT4G09770 | TRAF-like family protein | 7.66564 | 0.574639 | -3.73768 | 0.0009 | 0.0065867 |
| AT2G11290 | transposable element gene | 5.49121 | 0.413844 | -3.72997 | 0.0001 | 0.00123775 |
| AT2G17660 | RPM1-interacting protein 4 (RIN4) family protein | 761.31 | 57.4593 | -3.72787 | 0.00005 | 0.00070028 |
| AT5G60770 | ATNRT2.4, NRT2.4, nitrate transporter 2.4 | 23.3093 | 1.76064 | -3.72673 | 0.00005 | 0.00070028 |
| AT5G67450 | AZF1, ZF1, zinc-finger protein 1 | 14.8959 | 1.13073 | -3.71959 | 0.00005 | 0.00070028 |
| AT5G57070 | hydroxyproline-rich glycoprotein family protein | 49.8687 | 3.78625 | -3.71929 | 0.00005 | 0.00070028 |
| AT4G19530 | disease resistance protein (TIR-NBS-LRR class) family | 2.24198 | 0.170742 | -3.71488 | 0.00015 | 0.00170235 |
| AT2G38490 | CIPK22, SnRK3.19, CBL-interacting protein kinase 22 | 12.0333 | 0.92151 | -3.70689 | 0.00005 | 0.00070028 |
| AT1G32950 | Subtilase family protein | 10.2627 | 0.788284 | -3.70256 | 0.00005 | 0.00070028 |
| AT5G09430 | alpha/beta-Hydrolases superfamily protein | 25.8681 | 1.99078 | -3.69977 | 0.00005 | 0.00070028 |
| AT3G10470 | C2H2-type zinc finger family protein | 2.1944 | 0.16977 | -3.69217 | 0.01325 | 0.0484049 |
| AT3G15540 | IAA19, MSG2, indole-3-acetic acid inducible 19 | 288.02 | 22.3099 | -3.69041 | 0.00005 | 0.00070028 |
| AT5G43150 | unknown protein | 17.2338 | 1.34832 | -3.67601 | 0.00135 | 0.0089118 |
| AT4G37220 | Cold acclimation protein WCOR413 family | 18.8171 | 1.47407 | -3.67417 | 0.00005 | 0.00070028 |
| AT2G15370 | ATFUT5, FUT5, fucosyltransferase 5 | 5.57187 | 0.438409 | -3.66781 | 0.00025 | 0.00253767 |
| AT5G55950 | Nucleotide/sugar transporter family protein | 4.15352 | 0.328867 | -3.65876 | 0.0011 | 0.00759303 |
| AT2G28960 | Leucine-rich repeat protein kinase family protein | 14.0629 | 1.1159 | -3.65562 | 0.00005 | 0.00070028 |
| AT5G40260 | Nodulin MtN3 family protein | 5.84408 | 0.464072 | -3.65456 | 0.00085 | 0.00630464 |
| AT1G56010 | anac021, ANAC022, NAC1, NAC domain containing protein 1 | 41.566 | 3.30191 | -3.65403 | 0.00005 | 0.00070028 |
| AT3G11385 | Cysteine/Histidine-rich C1 domain family protein | 4.47628 | 0.355941 | -3.65259 | 0.00005 | 0.00070028 |
| AT3G54040 | PAR1 protein | 259.244 | 20.6245 | -3.65188 | 0.00005 | 0.00070028 |
| AT4G22620 | SAUR-like auxin-responsive protein family | 45.3796 | 3.64854 | -3.63665 | 0.0003 | 0.00292382 |
| AT1G53340 | Cysteine/Histidine-rich C1 domain family protein | 14.9653 | 1.20473 | -3.63484 | 0.00005 | 0.00070028 |
| AT1G15040 | Class I glutamine amidotransferase-like superfamily protein | 340.555 | 27.5151 | -3.62959 | 0.0001 | 0.00123775 |
| AT1G05810 | ARA, ARA-1, ATRAB11D, ATRABA5E, RABA5E, RAB GTPase homolog A5E | 153.349 | 12.397 | -3.62875 | 0.00005 | 0.00070028 |
| AT5G06839 | bZIP65, TGA10, bZIP transcription factor family protein | 18.3423 | 1.49624 | -3.61577 | 0.00005 | 0.00070028 |
| AT1G49450 | Transducin/WD40 repeat-like superfamily protein | 13.1922 | 1.0766 | -3.61514 | 0.00005 | 0.00070028 |
| AT3G55150 | ATEXO70H1, EXO70H1, exocyst subunit exo70 family protein H1 | 21.5778 | 1.76753 | -3.60974 | 0.00005 | 0.00070028 |
| AT5G07770 | Actin-binding FH2 protein | 16.0326 | 1.31705 | -3.60562 | 0.00005 | 0.00070028 |
| AT2G18450 | SDH1-2, succinate dehydrogenase 1-2 | 110.514 | 9.11401 | -3.6 | 0.00005 | 0.00070028 |
| AT5G22570 | ATWRKY38, WRKY38, WRKY DNA-binding protein 38 | 42.3492 | 3.49925 | -3.59722 | 0.00005 | 0.00070028 |
| AT1G53830 | ATPME2, PME2, pectin methylesterase 2 | 221.151 | 18.3097 | -3.59435 | 0.00005 | 0.00070028 |
| AT5G46040 | Major facilitator superfamily protein | 25.1274 | 2.08116 | -3.5938 | 0.00005 | 0.00070028 |
| AT1G61860 | Protein kinase superfamily protein | 22.6968 | 1.88542 | -3.58953 | 0.00005 | 0.00070028 |
| AT1G19250 | FMO1, flavin-dependent monooxygenase 1 | 6.93717 | 0.577603 | -3.5862 | 0.00005 | 0.00070028 |
| AT2G30930 | unknown protein | 360.634 | 30.0296 | -3.58608 | 0.00005 | 0.00070028 |
| AT4G30640 | RNI-like superfamily protein | 8.28116 | 0.69283 | -3.57926 | 0.00145 | 0.00940576 |
| AT3G25790 | myb-like transcription factor family protein | 9.44608 | 0.793658 | -3.57313 | 0.0001 | 0.00123775 |
| AT1G19190 | alpha/beta-Hydrolases superfamily protein | 17.2723 | 1.46088 | -3.56356 | 0.00005 | 0.00070028 |
| AT1G27030 | unknown protein | 36.2116 | 3.07547 | -3.55758 | 0.00005 | 0.00070028 |
| AT1G78100 | F-box family protein | 786.804 | 66.8369 | -3.55729 | 0.00005 | 0.00070028 |
| AT1G61275 | U12, U12; snRNA | 713.21 | 60.8873 | -3.55011 | 0.00315 | 0.0166977 |
| AT2G34940 | BP80-3;2, VSR3;2, VSR5, VACUOLAR SORTING RECEPTOR 5 | 52.7697 | 4.5124 | -3.54774 | 0.00005 | 0.00070028 |
| AT1G64400 | LACS3, AMP-dependent synthetase and ligase family protein | 10.3705 | 0.887508 | -3.54658 | 0.00005 | 0.00070028 |
| AT2G16760 | Calcium-dependent phosphotriesterase superfamily protein | 17.5319 | 1.50405 | -3.54306 | 0.00005 | 0.00070028 |
| AT1G64480 | CBL8, calcineurin B-like protein 8 | 25.1419 | 2.17402 | -3.53166 | 0.00005 | 0.00070028 |
| AT5G22920 | CHY-type/CTCHY-type/RING-type Zinc finger protein | 153.729 | 13.2964 | -3.53128 | 0.00005 | 0.00070028 |
| AT1G73680 | ALPHA DOX2, alpha dioxygenase | 9.59924 | 0.835131 | -3.52285 | 0.00005 | 0.00070028 |
| AT1G76230 | unknown protein | 10.3168 | 0.897649 | -3.52269 | 0.00145 | 0.00940576 |
| AT3G27070 | TOM20-1, translocase outer membrane 20-1 | 6.35289 | 0.554656 | -3.51775 | 0.0028 | 0.0152969 |
| AT1G13930 | Involved in response to salt stress. Knockout mutants are hypersensitive to salt stress. | 40.6054 | 3.54977 | -3.51588 | 0.00005 | 0.00070028 |
| AT5G47450 | ATTIP2;3, DELTA-TIP3, TIP2;3, tonoplast intrinsic protein 2;3 | 632.373 | 55.342 | -3.51433 | 0.00005 | 0.00070028 |
| AT4G38880 | ASE3, ATASE3, GLN phosphoribosyl pyrophosphate amidotransferase 3 | 5.21862 | 0.458217 | -3.50957 | 0.00085 | 0.00630464 |
| AT1G03550 | Secretory carrier membrane protein (SCAMP) family protein | 69.3496 | 6.09104 | -3.50913 | 0.00005 | 0.00070028 |
| AT1G73330 | ATDR4, DR4, drought-repressed 4 | 4.71834 | 0.416565 | -3.50166 | 0.00785 | 0.0330699 |
| AT5G18150 | Methyltransferase-related protein | 684.619 | 60.5441 | -3.49924 | 0.00005 | 0.00070028 |
| AT3G56000 | ATCSLA14, CSLA14, cellulose synthase like A14 | 57.0746 | 5.07221 | -3.49216 | 0.00005 | 0.00070028 |
| AT1G50890 | ARM repeat superfamily protein | 47.4969 | 4.22426 | -3.49106 | 0.00005 | 0.00070028 |
| AT5G24140 | SQP2, squalene monooxygenase 2 | 108.444 | 9.65024 | -3.49024 | 0.00005 | 0.00070028 |
| AT2G35890 | CPK25, calcium-dependent protein kinase 25 | 17.1009 | 1.53235 | -3.48025 | 0.00005 | 0.00070028 |
| AT2G27360 | GDSL-like Lipase/Acylhydrolase superfamily protein | 14.7443 | 1.32258 | -3.47874 | 0.00005 | 0.00070028 |
| AT4G27480 | Core-2/I-branching beta-1,6-N-acetylglucosaminyltransferase family protein | 25.4177 | 2.28265 | -3.47705 | 0.00005 | 0.00070028 |
| AT1G60110 | Mannose-binding lectin superfamily protein | 1.22031 | 0.109971 | -3.47205 | 0.0071 | 0.0306604 |
| AT1G68600 | Aluminium activated malate transporter family protein | 1.70899 | 0.154394 | -3.46846 | 0.00375 | 0.0189985 |
| AT1G02340 | FBI1, HFR1, REP1, RSF1, basic helix-loop-helix (bHLH) DNA-binding superfamily protein | 9.82746 | 0.889122 | -3.46637 | 0.00005 | 0.00070028 |
| AT3G03290 | Adenine nucleotide alpha hydrolases-like superfamily protein | 12.098 | 1.09724 | -3.46281 | 0.00015 | 0.00170235 |
| AT4G11470 | CRK31, cysteine-rich RLK (RECEPTOR-like protein kinase) 31 | 3.33979 | 0.307411 | -3.44152 | 0.001 | 0.00711095 |
| AT1G09540 | ATMYB61, MYB61, myb domain protein 61 | 26.1597 | 2.41019 | -3.44012 | 0.00005 | 0.00070028 |
| AT3G12500 | ATHCHIB, B-CHI, CHI-B, HCHIB, PR-3, PR3, basic chitinase | 54.134 | 5.00602 | -3.4348 | 0.00005 | 0.00070028 |
| AT2G31350 | GLX2-5, glyoxalase 2-5 | 163.768 | 15.1616 | -3.43316 | 0.00005 | 0.00070028 |
| AT5G37490 | ARM repeat superfamily protein | 1.96624 | 0.182152 | -3.43222 | 0.00345 | 0.0179093 |
| AT5G27100 | ATGLR2.1, GLR2.1, glutamate receptor 2.1 | 7.90356 | 0.735548 | -3.42561 | 0.00005 | 0.00070028 |
| AT2G18980 | Peroxidase superfamily protein | 77.9982 | 7.26961 | -3.42349 | 0.00005 | 0.00070028 |
| AT3G54363 | unknown protein | 124.475 | 11.6312 | -3.41978 | 0.00135 | 0.0089118 |
| AT5G27350 | SFP1, Major facilitator superfamily protein | 3.59609 | 0.336705 | -3.41687 | 0.00015 | 0.00170235 |
| AT1G04470 | Protein of unknown function (DUF810) | 1.46973 | 0.137931 | -3.41354 | 0.00255 | 0.014306 |
| AT3G48020 | unknown protein | 15.2227 | 1.43213 | -3.40999 | 0.00105 | 0.00735817 |
| AT3G56930 | DHHC-type zinc finger family protein | 146.087 | 13.7746 | -3.40674 | 0.00005 | 0.00070028 |
| AT2G39900 | GATA type zinc finger transcription factor family protein | 311.315 | 29.5 | -3.39959 | 0.00005 | 0.00070028 |
| AT4G14860 | atofp11, OFP11, ovate family protein 11 | 13.7763 | 1.30665 | -3.39824 | 0.0011 | 0.00759303 |
| AT3G07940 | Calcium-dependent ARF-type GTPase activating protein family | 48.1482 | 4.57929 | -3.39429 | 0.00005 | 0.00070028 |
| AT3G21560 | UGT84A2, UDP-Glycosyltransferase superfamily protein | 7.41631 | 0.706689 | -3.39155 | 0.0001 | 0.00123775 |
| AT1G15405 | other RNA | 177.029 | 16.8793 | -3.39066 | 0.00015 | 0.00170235 |
| AT3G61390 | RING/U-box superfamily protein | 28.8102 | 2.77069 | -3.37826 | 0.00005 | 0.00070028 |
| AT5G41290 | Receptor-like protein kinase-related family protein | 22.7611 | 2.18897 | -3.37825 | 0.00005 | 0.00070028 |
| AT3G18560 | unknown protein | 25.2787 | 2.44045 | -3.3727 | 0.00005 | 0.00070028 |
| AT5G24170 | Got1/Sft2-like vescicle transport protein family | 68.4319 | 6.62262 | -3.3692 | 0.00005 | 0.00070028 |
| AT5G48290 | Heavy metal transport/detoxification superfamily protein | 3.77746 | 0.366212 | -3.36667 | 0.0052 | 0.0244668 |
| AT3G12040 | DNA-3-methyladenine glycosylase (MAG) | 30.0115 | 2.9131 | -3.36489 | 0.00005 | 0.00070028 |
| AT4G27260 | GH3.5, WES1, Auxin-responsive GH3 family protein | 168.659 | 16.4149 | -3.36103 | 0.00005 | 0.00070028 |
| AT2G46300 | Late embryogenesis abundant (LEA) hydroxyproline-rich glycoprotein family | 2.80355 | 0.273016 | -3.3602 | 0.0042 | 0.0207631 |
| AT5G15180 | Peroxidase superfamily protein | 64.0184 | 6.26012 | -3.35423 | 0.00005 | 0.00070028 |
| AT1G54530 | Calcium-binding EF hand family protein | 34.0035 | 3.32935 | -3.35237 | 0.00225 | 0.0130442 |
| AT3G44510 | alpha/beta-Hydrolases superfamily protein | 11.2669 | 1.11307 | -3.33947 | 0.0001 | 0.00123775 |
| AT2G33310 | IAA13, auxin-induced protein 13 | 82.2792 | 8.13975 | -3.33747 | 0.00005 | 0.00070028 |
| AT2G15390 | atfut4, FUT4, fucosyltransferase 4 | 13.1441 | 1.30192 | -3.3357 | 0.00005 | 0.00070028 |
| AT3G09520 | ATEXO70H4, EXO70H4, exocyst subunit exo70 family protein H4 | 3.12196 | 0.309394 | -3.33494 | 0.0013 | 0.00867426 |
| AT2G02700 | Cysteine/Histidine-rich C1 domain family protein | 12.837 | 1.27248 | -3.33459 | 0.00005 | 0.00070028 |
| AT5G38340 | Disease resistance protein (TIR-NBS-LRR class) family | 2.67217 | 0.265224 | -3.33273 | 0.0009 | 0.0065867 |
| AT5G55020 | ATMYB120, MYB120, myb domain protein 120 | 2.53083 | 0.252435 | -3.32562 | 0.00195 | 0.0117451 |
| AT4G02390 | APP, ATPARP1, PARP1, PP, poly(ADP-ribose) polymerase | 37.8809 | 3.78638 | -3.32258 | 0.00005 | 0.00070028 |
| AT1G01180 | S-adenosyl-L-methionine-dependent methyltransferases superfamily protein | 22.5398 | 2.25418 | -3.3218 | 0.00005 | 0.00070028 |
| AT5G55050 | GDSL-like Lipase/Acylhydrolase superfamily protein | 65.3686 | 6.53833 | -3.3216 | 0.00005 | 0.00070028 |
| AT4G39070 | B-box zinc finger family protein | 24.0744 | 2.40985 | -3.32048 | 0.00005 | 0.00070028 |
| AT2G22905 | Expressed protein | 4.44945 | 0.446063 | -3.31831 | 0.0048 | 0.0229834 |
| AT5G06820 | SRF2, STRUBBELIG-receptor family 2 | 12.4835 | 1.25235 | -3.31731 | 0.00005 | 0.00070028 |
| AT2G23170 | GH3.3, Auxin-responsive GH3 family protein | 25.0764 | 2.52091 | -3.31431 | 0.00005 | 0.00070028 |
| AT1G65850 | Disease resistance protein (TIR-NBS-LRR class) family | 1.6799 | 0.169224 | -3.31137 | 0.00155 | 0.009918 |
| AT5G40010 | AATP1, AAA-ATPase 1 | 6.15818 | 0.624946 | -3.3007 | 0.00025 | 0.00253767 |
| AT1G22530 | PATL2, PATELLIN 2 | 186.164 | 19.0348 | -3.28986 | 0.00005 | 0.00070028 |
| AT1G72125 | Major facilitator superfamily protein | 22.674 | 2.32216 | -3.2875 | 0.00005 | 0.00070028 |
| AT2G37440 | DNAse I-like superfamily protein | 49.5012 | 5.07101 | -3.28712 | 0.00005 | 0.00070028 |
| AT1G67330 | Protein of unknown function (DUF579) | 97.9835 | 10.0397 | -3.28682 | 0.00005 | 0.00070028 |
| AT3G27884 | other RNA | 9.49056 | 0.973835 | -3.28474 | 0.0023 | 0.0132526 |
| AT1G67785 | unknown protein | 3258.96 | 334.996 | -3.2822 | 0.00005 | 0.00070028 |
| AT4G27350 | Protein of unknown function (DUF1223) | 244.81 | 25.1782 | -3.28142 | 0.00005 | 0.00070028 |
| AT1G72860 | Disease resistance protein (TIR-NBS-LRR class) family | 12.9882 | 1.33751 | -3.27958 | 0.00005 | 0.00070028 |
| AT4G25080 | CHLM, magnesium-protoporphyrin IX methyltransferase | 26.4964 | 2.73635 | -3.27547 | 0.00005 | 0.00070028 |
| AT4G12520 | Bifunctional inhibitor/lipid-transfer protein/seed storage 2S albumin superfamily protein | 37.1342 | 3.83656 | -3.27486 | 0.00025 | 0.00253767 |
| AT1G26970 | Protein kinase superfamily protein | 16.2133 | 1.67576 | -3.27429 | 0.00005 | 0.00070028 |
| AT5G53250 | AGP22, ATAGP22, arabinogalactan protein 22 | 220.897 | 22.9246 | -3.2684 | 0.00005 | 0.00070028 |
| AT3G45060 | ATNRT2.6, NRT2.6, high affinity nitrate transporter 2.6 | 573.969 | 59.6096 | -3.26736 | 0.00005 | 0.00070028 |
| AT2G22460 | Protein of unknown function, DUF617 | 2.03965 | 0.211913 | -3.26678 | 0.0106 | 0.0411532 |
| AT3G11390 | Cysteine/Histidine-rich C1 domain family protein | 3.76711 | 0.391929 | -3.26479 | 0.00005 | 0.00070028 |
| AT1G13100 | CYP71B29, cytochrome P450, family 71, subfamily B, polypeptide 29 | 1.91746 | 0.199706 | -3.26324 | 0.00435 | 0.0212815 |
| AT5G24410 | PGL4, 6-phosphogluconolactonase 4 | 15.2605 | 1.59477 | -3.25839 | 0.00005 | 0.00070028 |
| AT3G21710 | unknown protein | 190.137 | 19.9234 | -3.2545 | 0.00005 | 0.00070028 |
| AT1G72870 | Disease resistance protein (TIR-NBS class) | 15.5963 | 1.63679 | -3.25227 | 0.00005 | 0.00070028 |
| AT5G43350 | ATPT1, PHT1;1, phosphate transporter 1;1 | 34.3803 | 3.62091 | -3.24716 | 0.00005 | 0.00070028 |
| AT1G15640 | unknown protein | 30.39 | 3.20276 | -3.24621 | 0.00005 | 0.00070028 |
| AT3G12502 | other RNA | 55.0065 | 5.80044 | -3.24537 | 0.0109 | 0.0419234 |
| AT5G40510 | Sucrase/ferredoxin-like family protein | 242.636 | 25.6875 | -3.23965 | 0.00005 | 0.00070028 |
| AT5G64100 | Peroxidase superfamily protein | 727.186 | 77.0111 | -3.23919 | 0.00005 | 0.00070028 |
| AT2G32160 | S-adenosyl-L-methionine-dependent methyltransferases superfamily protein | 7.50187 | 0.794936 | -3.23834 | 0.00015 | 0.00170235 |
| AT1G78260 | RNA-binding (RRM/RBD/RNP motifs) family protein | 44.6488 | 4.73431 | -3.2374 | 0.00005 | 0.00070028 |
| AT1G22220 | F-box family protein | 12.9136 | 1.37217 | -3.23437 | 0.00005 | 0.00070028 |
| AT3G05170 | Phosphoglycerate mutase family protein | 90.1163 | 9.58352 | -3.23316 | 0.00005 | 0.00070028 |
| AT5G45580 | Homeodomain-like superfamily protein | 134.741 | 14.3338 | -3.2327 | 0.00005 | 0.00070028 |
| AT2G36830 | GAMMA-TIP, GAMMA-TIP1, TIP1;1, gamma tonoplast intrinsic protein | 974.405 | 103.689 | -3.23226 | 0.00005 | 0.00070028 |
| AT1G66910 | Protein kinase superfamily protein | 12.069 | 1.28736 | -3.22882 | 0.00005 | 0.00070028 |
| AT2G03370 | Glycosyltransferase family 61 protein | 2.03019 | 0.218015 | -3.21912 | 0.005 | 0.0237003 |
| AT1G14550 | Peroxidase superfamily protein | 27.0548 | 2.90707 | -3.21825 | 0.00005 | 0.00070028 |
| AT1G26360 | ATMES13, MES13, methyl esterase 13 | 5.82446 | 0.627281 | -3.21494 | 0.00015 | 0.00170235 |
| AT2G44230 | Plant protein of unknown function (DUF946) | 24.0215 | 2.58786 | -3.2145 | 0.00005 | 0.00070028 |
| AT2G28710 | C2H2-type zinc finger family protein | 14.2587 | 1.53816 | -3.21257 | 0.00015 | 0.00170235 |
| AT5G26660 | ATMYB86, MYB86, myb domain protein 86 | 54.9783 | 5.93874 | -3.21063 | 0.00005 | 0.00070028 |
| AT5G58940 | CRCK1, calmodulin-binding receptor-like cytoplasmic kinase 1 | 38.1678 | 4.1385 | -3.20517 | 0.00005 | 0.00070028 |
| AT5G01050 | Laccase/Diphenol oxidase family protein | 25.7688 | 2.79978 | -3.20224 | 0.00005 | 0.00070028 |
| AT3G11370 | Cysteine/Histidine-rich C1 domain family protein | 1.49717 | 0.163125 | -3.19818 | 0.0043 | 0.0211046 |
| AT1G52750 | alpha/beta-Hydrolases superfamily protein | 130.458 | 14.2276 | -3.19682 | 0.0001 | 0.00123775 |
| AT5G06230 | TBL9, TRICHOME BIREFRINGENCE-LIKE 9 | 34.4147 | 3.75889 | -3.19464 | 0.0058 | 0.0265396 |
| AT3G22830 | AT-HSFA6B, HSFA6B, heat shock transcription factor A6B | 338.883 | 37.2627 | -3.18498 | 0.00005 | 0.00070028 |
| AT5G65500 | U-box domain-containing protein kinase family protein | 1.41618 | 0.155872 | -3.18357 | 0.003 | 0.0161227 |
| AT4G21780 | unknown protein | 8.10851 | 0.894419 | -3.18041 | 0.0042 | 0.0207631 |
| AT2G27660 | Cysteine/Histidine-rich C1 domain family protein | 41.893 | 4.62137 | -3.18032 | 0.00005 | 0.00070028 |
| AT5G52020 | Integrase-type DNA-binding superfamily protein | 91.8093 | 10.1282 | -3.18026 | 0.00005 | 0.00070028 |
| AT5G04310 | Pectin lyase-like superfamily protein | 4.23116 | 0.470243 | -3.16958 | 0.0001 | 0.00123775 |
| AT2G44290 | Bifunctional inhibitor/lipid-transfer protein/seed storage 2S albumin superfamily protein | 20.5867 | 2.2888 | -3.16905 | 0.00005 | 0.00070028 |
| AT5G24105 | AGP41, arabinogalactan protein 41 | 44.2348 | 4.92272 | -3.16766 | 0.00005 | 0.00070028 |
| AT4G37950 | Rhamnogalacturonate lyase family protein | 1.35937 | 0.152123 | -3.15964 | 0.0048 | 0.0229834 |
| AT3G09330 | Transmembrane amino acid transporter family protein | 5.91769 | 0.6641 | -3.15556 | 0.0011 | 0.00759303 |
| AT4G31380 | FLP1, FPF1-like protein 1 | 6.08319 | 0.683178 | -3.15449 | 0.00285 | 0.0154761 |
| AT4G21250 | Sulfite exporter TauE/SafE family protein | 2.56826 | 0.28897 | -3.1518 | 0.0021 | 0.0124516 |
| AT1G01730 | unknown protein | 159.28 | 17.9865 | -3.14657 | 0.00005 | 0.00070028 |
| AT3G54950 | PLA IIIA, PLP7, patatin-like protein 6 | 48.5347 | 5.48234 | -3.14615 | 0.00005 | 0.00070028 |
| AT1G71530 | Protein kinase superfamily protein | 83.882 | 9.47798 | -3.14571 | 0.00005 | 0.00070028 |
| AT1G51680 | 4CL.1, 4CL1, AT4CL1, 4-coumarate:CoA ligase 1 | 38.278 | 4.34022 | -3.14068 | 0.00005 | 0.00070028 |
| AT5G54490 | PBP1, pinoid-binding protein 1 | 287.933 | 32.7306 | -3.13702 | 0.00005 | 0.00070028 |
| AT3G21750 | UGT71B1, UDP-glucosyl transferase 71B1 | 25.327 | 2.88012 | -3.13647 | 0.00005 | 0.00070028 |
| AT1G52240 | ATROPGEF11, PIRF1, ROPGEF11, RHO guanyl-nucleotide exchange factor 11 | 222.34 | 25.3168 | -3.1346 | 0.00005 | 0.00070028 |
| AT5G10770 | Eukaryotic aspartyl protease family protein | 10.952 | 1.24741 | -3.13418 | 0.0002 | 0.00214445 |
| AT1G66725 | MIR163, MIR163; miRNA | 5.15305 | 0.589336 | -3.12826 | 0.0078 | 0.0329152 |
| AT4G00940 | Dof-type zinc finger DNA-binding family protein | 3.70093 | 0.423566 | -3.12723 | 0.00255 | 0.014306 |
| AT4G20460 | NAD(P)-binding Rossmann-fold superfamily protein | 74.9464 | 8.57923 | -3.12694 | 0.00005 | 0.00070028 |
| AT3G29240 | Protein of unknown function (DUF179) | 30.8266 | 3.53292 | -3.12524 | 0.00005 | 0.00070028 |
| AT3G05490 | RALFL22, ralf-like 22 | 471.929 | 54.1202 | -3.12433 | 0.00005 | 0.00070028 |
| AT1G69790 | Protein kinase superfamily protein | 10.5225 | 1.20867 | -3.12198 | 0.00005 | 0.00070028 |
| AT4G01140 | Protein of unknown function (DUF1191) | 24.1913 | 2.78544 | -3.11851 | 0.00005 | 0.00070028 |
| AT1G05630 | 5PTASE13, AT5PTASE13, Endonuclease/exonuclease/phosphatase family protein | 24.5091 | 2.82692 | -3.11601 | 0.00005 | 0.00070028 |
| AT4G39675 | unknown protein | 148.814 | 17.1982 | -3.11318 | 0.00005 | 0.00070028 |
| AT3G51860 | ATCAX3, ATHCX1, CAX1-LIKE, CAX3, cation exchanger 3 | 6.31005 | 0.730419 | -3.11085 | 0.00015 | 0.00170235 |
| AT1G03850 | Glutaredoxin family protein | 448.238 | 51.9894 | -3.10797 | 0.00005 | 0.00070028 |
| AT2G02690 | Cysteine/Histidine-rich C1 domain family protein | 2.57251 | 0.298491 | -3.10742 | 0.0024 | 0.0136815 |
| AT3G62990 | unknown protein | 8.04326 | 0.935722 | -3.10363 | 0.00495 | 0.0235479 |
| AT1G13950 | ATELF5A-1, EIF-5A, EIF5A, ELF5A-1, eukaryotic elongation factor 5A-1 | 107.303 | 12.4879 | -3.10308 | 0.00005 | 0.00070028 |
| AT5G19040 | ATIPT5, IPT5, isopentenyltransferase 5 | 22.6776 | 2.63966 | -3.10284 | 0.00005 | 0.00070028 |
| AT1G55365 | unknown protein | 14.1245 | 1.65217 | -3.09577 | 0.0116 | 0.0439836 |
| AT1G27420 | Galactose oxidase/kelch repeat superfamily protein | 29.9869 | 3.50773 | -3.09572 | 0.00005 | 0.00070028 |
| AT2G14210 | AGL44, ANR1, AGAMOUS-like 44 | 25.7774 | 3.01874 | -3.09409 | 0.00005 | 0.00070028 |
| AT3G01850 | Aldolase-type TIM barrel family protein | 14.7237 | 1.72598 | -3.09265 | 0.00005 | 0.00070028 |
| AT5G55780 | Cysteine/Histidine-rich C1 domain family protein | 1.85406 | 0.217527 | -3.09142 | 0.0026 | 0.0145003 |
| AT1G15580 | ATAUX2-27, AUX2-27, IAA5, indole-3-acetic acid inducible 5 | 86.7121 | 10.1769 | -3.09094 | 0.00005 | 0.00070028 |
| AT3G26520 | GAMMA-TIP2, SITIP, TIP1;2, TIP2, tonoplast intrinsic protein 2 | 1343.92 | 157.822 | -3.09008 | 0.00005 | 0.00070028 |
| AT1G52155 | unknown protein | 24.9453 | 2.95257 | -3.07872 | 0.00005 | 0.00070028 |
| AT1G69900 | Actin cross-linking protein | 26.4183 | 3.13205 | -3.07636 | 0.00005 | 0.00070028 |
| AT4G01350 | Cysteine/Histidine-rich C1 domain family protein | 13.2072 | 1.56638 | -3.07582 | 0.00005 | 0.00070028 |
| AT3G09710 | IQD1, IQ-domain 1 | 47.9049 | 5.68657 | -3.07454 | 0.00005 | 0.00070028 |
| AT1G10020 | Protein of unknown function (DUF1005) | 19.4171 | 2.30678 | -3.07337 | 0.00005 | 0.00070028 |
| AT5G49900 | Beta-glucosidase, GBA2 type family protein | 67.753 | 8.07035 | -3.06958 | 0.00005 | 0.00070028 |
| AT5G11920 | AtcwINV6, cwINV6, 6-&1-fructan exohydrolase | 49.1181 | 5.8747 | -3.06367 | 0.00005 | 0.00070028 |
| AT5G01550 | LECRKA4.2, lectin receptor kinase a4.1 | 1.39997 | 0.167486 | -3.06329 | 0.0054 | 0.0251864 |
| AT1G18773 | function unknown | 2.89381 | 0.347053 | -3.05974 | 0.01 | 0.0393953 |
| AT3G59340 | Eukaryotic protein of unknown function (DUF914) | 12.9469 | 1.55281 | -3.05965 | 0.00005 | 0.00070028 |
| AT4G01480 | AtPPa5, PPa5, pyrophosphorylase 5 | 1274.5 | 153.007 | -3.05827 | 0.00005 | 0.00070028 |
| AT1G80440 | Galactose oxidase/kelch repeat superfamily protein | 125.421 | 15.0842 | -3.05566 | 0.00005 | 0.00070028 |
| AT3G06990 | Cysteine/Histidine-rich C1 domain family protein | 6.72998 | 0.811217 | -3.05244 | 0.0003 | 0.00292382 |
| AT5G45116 | transposable element gene | 7.23456 | 0.873391 | -3.05021 | 0.00005 | 0.00070028 |
| AT5G45180 | Flavin-binding monooxygenase family protein | 1.30539 | 0.157622 | -3.04994 | 0.01055 | 0.0410315 |
| AT2G24320 | alpha/beta-Hydrolases superfamily protein | 9.39367 | 1.13674 | -3.04678 | 0.00095 | 0.00685384 |
| AT1G22500 | RING/U-box superfamily protein | 44.0959 | 5.34257 | -3.04504 | 0.01105 | 0.0423687 |
| AT5G11110 | ATSPS2F, KNS2, SPS1, SPS2F, sucrose phosphate synthase 2F | 412.702 | 50.0113 | -3.04478 | 0.00005 | 0.00070028 |
| AT2G43820 | ATSAGT1, GT, SAGT1, SGT1, UGT74F2, UDP-glucosyltransferase 74F2 | 633.66 | 77.0631 | -3.0396 | 0.00005 | 0.00070028 |
| AT3G58810 | ATMTP3, ATMTPA2, MTP3, MTPA2, metal tolerance protein A2 | 10.4308 | 1.2688 | -3.03931 | 0.00005 | 0.00070028 |
| AT3G28310 | Protein of unknown function (DUF677) | 449.724 | 54.7267 | -3.03872 | 0.00015 | 0.00170235 |
| AT1G72430 | SAUR-like auxin-responsive protein family | 29.7269 | 3.62723 | -3.03483 | 0.00005 | 0.00070028 |
| AT1G13510 | Protein of unknown function (DUF1262) | 6.14524 | 0.750605 | -3.03334 | 0.00035 | 0.00327416 |
| AT4G31020 | alpha/beta-Hydrolases superfamily protein | 11.5297 | 1.41059 | -3.03098 | 0.0084 | 0.0346931 |
| AT2G47650 | UXS4, UDP-xylose synthase 4 | 751.056 | 92.2069 | -3.02597 | 0.00005 | 0.00070028 |
| AT3G14470 | NB-ARC domain-containing disease resistance protein | 18.4803 | 2.26929 | -3.02568 | 0.00005 | 0.00070028 |
| AT1G12950 | RSH2, root hair specific 2 | 383.267 | 47.3697 | -3.01631 | 0.00005 | 0.00070028 |
| AT1G13300 | HRS1, myb-like transcription factor family protein | 24.5871 | 3.04804 | -3.01195 | 0.00005 | 0.00070028 |
| AT3G12700 | Eukaryotic aspartyl protease family protein | 75.0427 | 9.33747 | -3.00661 | 0.00005 | 0.00070028 |
| AT4G35060 | Heavy metal transport/detoxification superfamily protein | 30.5191 | 3.80277 | -3.00459 | 0.00015 | 0.00170235 |
| AT5G16900 | Leucine-rich repeat protein kinase family protein | 24.4234 | 3.05334 | -2.9998 | 0.00005 | 0.00070028 |
| AT2G32270 | ZIP3, zinc transporter 3 precursor | 165.322 | 20.7386 | -2.99489 | 0.00005 | 0.00070028 |
| AT3G07880 | SCN1, Immunoglobulin E-set superfamily protein | 469.668 | 58.949 | -2.9941 | 0.00005 | 0.00070028 |
| AT2G20880 | Integrase-type DNA-binding superfamily protein | 165.331 | 20.7599 | -2.99348 | 0.00005 | 0.00070028 |
| AT4G04410 | transposable element gene | 1.00098 | 0.125942 | -2.99058 | 0.00355 | 0.0182751 |
| AT3G53150 | UGT73D1, UDP-glucosyl transferase 73D1 | 47.678 | 6.00347 | -2.98945 | 0.00005 | 0.00070028 |
| AT5G44260 | Zinc finger C-x8-C-x5-C-x3-H type family protein | 5.50629 | 0.696468 | -2.98295 | 0.00035 | 0.00327416 |
| AT2G38060 | PHT4;2, phosphate transporter 4;2 | 5.49993 | 0.696335 | -2.98156 | 0.0002 | 0.00214445 |
| AT2G04170 | TRAF-like family protein | 168.974 | 21.5401 | -2.9717 | 0.00005 | 0.00070028 |
| AT1G74940 | Protein of unknown function (DUF581) | 61.2174 | 7.83036 | -2.96679 | 0.00005 | 0.00070028 |
| AT1G66860 | Class I glutamine amidotransferase-like superfamily protein | 14.4694 | 1.85397 | -2.96431 | 0.00005 | 0.00070028 |
| AT3G45710 | Major facilitator superfamily protein | 3.20043 | 0.410486 | -2.96286 | 0.00165 | 0.0103801 |
| AT2G48080 | oxidoreductase, 2OG-Fe(II) oxygenase family protein | 32.817 | 4.20925 | -2.96281 | 0.00005 | 0.00070028 |
| AT2G44670 | Protein of unknown function (DUF581) | 441.449 | 56.6394 | -2.96237 | 0.00005 | 0.00070028 |
| AT1G01120 | KCS1, 3-ketoacyl-CoA synthase 1 | 82.8902 | 10.6765 | -2.95676 | 0.00005 | 0.00070028 |
| AT1G62850 | Class I peptide chain release factor | 110.321 | 14.2257 | -2.95513 | 0.00005 | 0.00070028 |
| AT5G56870 | BGAL4, beta-galactosidase 4 | 59.9873 | 7.7497 | -2.95244 | 0.00005 | 0.00070028 |
| AT5G18670 | BAM9, BMY3, beta-amylase 3 | 274.994 | 35.5265 | -2.95243 | 0.00005 | 0.00070028 |
| AT3G27270 | TRAM, LAG1 and CLN8 (TLC) lipid-sensing domain containing protein | 3.32571 | 0.430144 | -2.95077 | 0.00685 | 0.0299134 |
| AT5G08640 | ATFLS1, FLS, FLS1, flavonol synthase 1 | 32.2254 | 4.1713 | -2.94963 | 0.00005 | 0.00070028 |
| AT4G18610 | LSH9, Protein of unknown function (DUF640) | 34.7173 | 4.49472 | -2.94935 | 0.00005 | 0.00070028 |
| AT2G30395 | ATOFP17, OFP17, ovate family protein 17 | 42.9151 | 5.56788 | -2.94629 | 0.00005 | 0.00070028 |
| AT3G01720 | unknown protein | 115.849 | 15.0466 | -2.94474 | 0.00005 | 0.00070028 |
| AT3G60550 | CYCP3;2, cyclin p3;2 | 107.833 | 14.0374 | -2.94145 | 0.00005 | 0.00070028 |
| AT3G07000 | Cysteine/Histidine-rich C1 domain family protein | 15.1634 | 1.97575 | -2.94012 | 0.00005 | 0.00070028 |
| AT3G19030 | unknown protein | 235.993 | 30.9055 | -2.93281 | 0.00005 | 0.00070028 |
| AT5G09610 | APUM21, PUM21, pumilio 21 | 4.95062 | 0.648558 | -2.9323 | 0.00185 | 0.0113253 |
| AT4G31450 | RING/U-box superfamily protein | 95.7907 | 12.5648 | -2.9305 | 0.00005 | 0.00070028 |
| AT1G22570 | Major facilitator superfamily protein | 33.3471 | 4.37621 | -2.92981 | 0.00005 | 0.00070028 |
| AT4G03490 | Ankyrin repeat family protein | 7.47103 | 0.985009 | -2.9231 | 0.00005 | 0.00070028 |
| AT1G16510 | SAUR-like auxin-responsive protein family | 29.0176 | 3.83719 | -2.9188 | 0.00005 | 0.00070028 |
| AT5G54020 | Cysteine/Histidine-rich C1 domain family protein | 2.89791 | 0.383735 | -2.91683 | 0.00055 | 0.00457655 |
| AT5G20820 | SAUR-like auxin-responsive protein family | 56.7986 | 7.52724 | -2.91566 | 0.00005 | 0.00070028 |
| AT2G33280 | Major facilitator superfamily protein | 1.6743 | 0.222025 | -2.91476 | 0.00925 | 0.0371953 |
| AT5G04238 | unknown protein | 229.223 | 30.4009 | -2.91457 | 0.0127 | 0.0469062 |
| AT5G17810 | WOX12, WUSCHEL related homeobox 12 | 9.42876 | 1.25121 | -2.91374 | 0.0004 | 0.00359419 |
| AT5G12000 | Protein kinase protein with adenine nucleotide alpha hydrolases-like domain | 4.96377 | 0.659195 | -2.91266 | 0.00005 | 0.00070028 |
| AT2G26290 | ARSK1, root-specific kinase 1 | 119.218 | 15.8924 | -2.90719 | 0.00005 | 0.00070028 |
| AT2G39360 | Protein kinase superfamily protein | 26.5529 | 3.54205 | -2.90622 | 0.00005 | 0.00070028 |
| AT2G19130 | S-locus lectin protein kinase family protein | 2.53099 | 0.338059 | -2.90436 | 0.0006 | 0.00487984 |
| AT5G42460 | F-box and associated interaction domains-containing protein | 10.5154 | 1.40553 | -2.90332 | 0.00005 | 0.00070028 |
| AT1G75360 | unknown protein | 2.17262 | 0.290653 | -2.90206 | 0.00575 | 0.0263656 |
| AT4G06744 | Leucine-rich repeat (LRR) family protein | 5.34157 | 0.714639 | -2.90198 | 0.0007 | 0.00547072 |
| AT5G42830 | HXXXD-type acyl-transferase family protein | 22.7574 | 3.04995 | -2.89948 | 0.00005 | 0.00070028 |
| AT4G30140 | CDEF1, GDSL-like Lipase/Acylhydrolase superfamily protein | 23.5062 | 3.15126 | -2.89904 | 0.00005 | 0.00070028 |
| AT5G26010 | Protein phosphatase 2C family protein | 60.2823 | 8.08689 | -2.89808 | 0.00005 | 0.00070028 |
| AT4G04760 | Major facilitator superfamily protein | 5.37359 | 0.722476 | -2.89487 | 0.0006 | 0.00487984 |
| AT5G14760 | AO, L-aspartate oxidase | 11.158 | 1.50731 | -2.88802 | 0.00005 | 0.00070028 |
| AT3G62850 | zinc finger protein-related | 1.97074 | 0.266734 | -2.88527 | 0.00745 | 0.0318523 |
| AT2G32510 | MAPKKK17, mitogen-activated protein kinase kinase kinase 17 | 67.5074 | 9.13729 | -2.88521 | 0.00005 | 0.00070028 |
| AT3G56240 | CCH, copper chaperone | 224.175 | 30.4103 | -2.882 | 0.00005 | 0.00070028 |
| AT1G04110 | SDD1, Subtilase family protein | 2.07955 | 0.28225 | -2.88123 | 0.00105 | 0.00735817 |
| AT4G05330 | AGD13, ARF-GAP domain 13 | 38.9895 | 5.29924 | -2.87923 | 0.00005 | 0.00070028 |
| AT5G42840 | Cysteine/Histidine-rich C1 domain family protein | 4.03071 | 0.552239 | -2.86767 | 0.0005 | 0.00424432 |
| AT2G04130.1 | transposable element gene | 4.2392 | 0.581482 | -2.86599 | 0.00035 | 0.00327416 |
| AT1G10550 | XET, XTH33, xyloglucan:xyloglucosyl transferase 33 | 2.14146 | 0.293809 | -2.86565 | 0.00915 | 0.0369056 |
| AT2G24260 | LRL1, LJRHL1-like 1 | 64.1733 | 8.81101 | -2.86459 | 0.00005 | 0.00070028 |
| AT1G30860 | RING/U-box superfamily protein | 6.42225 | 0.883213 | -2.86225 | 0.00005 | 0.00070028 |
| AT5G16540 | ZFN3, zinc finger nuclease 3 | 14.8324 | 2.04106 | -2.86137 | 0.00005 | 0.00070028 |
| AT5G04470 | SIM, cyclin-dependent protein kinase inhibitors | 40.0035 | 5.50779 | -2.86058 | 0.00005 | 0.00070028 |
| AT1G44130 | Eukaryotic aspartyl protease family protein | 4.01786 | 0.554995 | -2.85588 | 0.00465 | 0.0223951 |
| AT1G47480 | alpha/beta-Hydrolases superfamily protein | 50.2463 | 6.95046 | -2.85384 | 0.00005 | 0.00070028 |
| AT3G25240 | Protein of unknown function (DUF506) | 3.97439 | 0.550249 | -2.85258 | 0.00485 | 0.0231612 |
| AT4G13615 | Uncharacterised protein family SERF | 772.4 | 107.044 | -2.85115 | 0.00005 | 0.00070028 |
| AT1G44090 | ATGA20OX5, GA20OX5, gibberellin 20-oxidase 5 | 90.5283 | 12.5487 | -2.85083 | 0.00005 | 0.00070028 |
| AT4G33330 | GUX2, PGSIP3, plant glycogenin-like starch initiation protein 3 | 1.16072 | 0.161244 | -2.8477 | 0.00755 | 0.0321272 |
| AT5G54130 | Calcium-binding endonuclease/exonuclease/phosphatase family | 16.7033 | 2.32207 | -2.84665 | 0.00005 | 0.00070028 |
| AT1G07430 | HAI2, highly ABA-induced PP2C gene 2 | 2.35077 | 0.326942 | -2.84603 | 0.00305 | 0.0163293 |
| AT5G67520 | APK4, adenosine-5'-phosphosulfate (APS) kinase 4 | 107.667 | 14.9742 | -2.84602 | 0.00005 | 0.00070028 |
| AT3G01520 | Adenine nucleotide alpha hydrolases-like superfamily protein | 191.549 | 26.7047 | -2.84254 | 0.00005 | 0.00070028 |
| AT5G45113 | mitochondrial transcription termination factor-related / mTERF-related | 5.88293 | 0.820934 | -2.8412 | 0.00065 | 0.00516972 |
| AT2G42350 | RING/U-box superfamily protein | 59.7442 | 8.36996 | -2.83551 | 0.00005 | 0.00070028 |
| AT3G22540 | Protein of unknown function (DUF1677) | 27.0551 | 3.79169 | -2.83499 | 0.00015 | 0.00170235 |
| AT5G60800 | Heavy metal transport/detoxification superfamily protein | 97.0321 | 13.6142 | -2.83335 | 0.00005 | 0.00070028 |
| AT3G49520 | F-box and associated interaction domains-containing protein | 3.02165 | 0.424061 | -2.83299 | 0.0045 | 0.0218802 |
| AT1G22330 | RNA-binding (RRM/RBD/RNP motifs) family protein | 15.1725 | 2.13332 | -2.83029 | 0.00015 | 0.00170235 |
| AT5G35580 | Protein kinase superfamily protein | 22.5848 | 3.17853 | -2.82892 | 0.00005 | 0.00070028 |
| AT5G45500 | RNI-like superfamily protein | 110.658 | 15.6216 | -2.82449 | 0.00005 | 0.00070028 |
| AT1G23090 | AST91, SULTR3;3, sulfate transporter 91 | 10.368 | 1.46506 | -2.8231 | 0.00005 | 0.00070028 |
| AT5G38940 | RmlC-like cupins superfamily protein | 46.6574 | 6.59934 | -2.82171 | 0.00005 | 0.00070028 |
| AT3G25930 | Adenine nucleotide alpha hydrolases-like superfamily protein | 54.343 | 7.70837 | -2.8176 | 0.00005 | 0.00070028 |
| AT5G24030 | SLAH3, SLAC1 homologue 3 | 59.1096 | 8.40522 | -2.81404 | 0.00005 | 0.00070028 |
| AT1G18980 | RmlC-like cupins superfamily protein | 27.905 | 3.97892 | -2.81008 | 0.0001 | 0.00123775 |
| AT3G49860 | ARLA1B, ATARLA1B, ADP-ribosylation factor-like A1B | 18.318 | 2.61222 | -2.80992 | 0.0006 | 0.00487984 |
| AT1G70810 | Calcium-dependent lipid-binding (CaLB domain) family protein | 54.8635 | 7.8275 | -2.80922 | 0.00005 | 0.00070028 |
| AT5G01100 | O-fucosyltransferase family protein | 174.355 | 24.9128 | -2.80707 | 0.00005 | 0.00070028 |
| AT5G39760 | AtHB23, HB23, homeobox protein 23 | 3.36572 | 0.481387 | -2.80565 | 0.0007 | 0.00547072 |
| AT5G55960 | unknown protein | 75.9385 | 10.8671 | -2.80487 | 0.00005 | 0.00070028 |
| AT5G54510 | DFL1, GH3.6, Auxin-responsive GH3 family protein | 101.655 | 14.5543 | -2.80417 | 0.00005 | 0.00070028 |
| AT2G35940 | BLH1, EDA29, BEL1-like homeodomain 1 | 3.12393 | 0.448125 | -2.80139 | 0.00105 | 0.00735817 |
| AT4G22460 | Bifunctional inhibitor/lipid-transfer protein/seed storage 2S albumin superfamily protein | 162.841 | 23.3611 | -2.80128 | 0.00005 | 0.00070028 |
| AT2G02680 | Cysteine/Histidine-rich C1 domain family protein | 6.66328 | 0.956055 | -2.80107 | 0.00005 | 0.00070028 |
| AT1G10200 | WLIM1, GATA type zinc finger transcription factor family protein | 120.914 | 17.3714 | -2.79919 | 0.00005 | 0.00070028 |
| AT1G30190 | unknown protein | 15.7828 | 2.27015 | -2.79749 | 0.00005 | 0.00070028 |
| AT1G63580 | Receptor-like protein kinase-related family protein | 37.164 | 5.35363 | -2.79532 | 0.00005 | 0.00070028 |
| AT1G02400 | ATGA2OX4, ATGA2OX6, DTA1, GA2OX6, gibberellin 2-oxidase 6 | 92.8745 | 13.5141 | -2.78082 | 0.00005 | 0.00070028 |
| AT4G22980 | function unknown | 1.68263 | 0.245126 | -2.77912 | 0.0044 | 0.0214942 |
| AT2G38320 | TBL34, TRICHOME BIREFRINGENCE-LIKE 34 | 13.0348 | 1.89896 | -2.77909 | 0.0002 | 0.00214445 |
| AT1G28330 | DRM1, DYL1, dormancy-associated protein-like 1 | 348.14 | 50.7583 | -2.77795 | 0.00005 | 0.00070028 |
| AT1G70820 | phosphoglucomutase, putative / glucose phosphomutase, putative | 1.24047 | 0.180959 | -2.77715 | 0.0068 | 0.029741 |
| AT5G43620 | Pre-mRNA cleavage complex II | 128.958 | 18.8136 | -2.77706 | 0.00005 | 0.00070028 |
| AT1G72800 | RNA-binding (RRM/RBD/RNP motifs) family protein | 52.1081 | 7.61144 | -2.77527 | 0.00005 | 0.00070028 |
| AT1G22540 | Major facilitator superfamily protein | 14.7275 | 2.15645 | -2.77178 | 0.00005 | 0.00070028 |
| AT3G29635 | HXXXD-type acyl-transferase family protein | 13.4612 | 1.97546 | -2.76854 | 0.0002 | 0.00214445 |
| AT5G48770 | Disease resistance protein (TIR-NBS-LRR class) family | 2.49246 | 0.366208 | -2.76684 | 0.0064 | 0.0284694 |
| AT2G25510 | unknown protein | 32.3447 | 4.78721 | -2.75627 | 0.0008 | 0.00603091 |
| AT5G66040 | STR16, sulfurtransferase protein 16 | 39.9519 | 5.92621 | -2.75308 | 0.0001 | 0.00123775 |
| AT4G14610 | pseudogene, disease resistance protein (CC-NBS-LRR class), putative, domain signature CC-NBS-LRR exists, suggestive of a disease resistance protein.; blastp match of 45% identity and 2.2e-162 P-value to GP|24461866|gb|AAN62353.1|AF506028_20|AF506028 NBS-LRR type disease resistance protein {Poncirus trifoliata} | 11.7875 | 1.74873 | -2.75288 | 0.00005 | 0.00070028 |
| AT5G24040 | Protein of unknown function (DUF295) | 8.94661 | 1.32902 | -2.75098 | 0.0004 | 0.00359419 |
| AT2G44450 | BGLU15, beta glucosidase 15 | 288.944 | 42.9701 | -2.74938 | 0.00005 | 0.00070028 |
| AT1G02660 | alpha/beta-Hydrolases superfamily protein | 58.9969 | 8.77953 | -2.74842 | 0.00005 | 0.00070028 |
| AT4G00700 | C2 calcium/lipid-binding plant phosphoribosyltransferase family protein | 9.02293 | 1.34296 | -2.74818 | 0.00005 | 0.00070028 |
| AT2G44380 | Cysteine/Histidine-rich C1 domain family protein | 18.7913 | 2.79727 | -2.74797 | 0.00015 | 0.00170235 |
| AT5G41315 | GL3, GL3, MYC6.2, basic helix-loop-helix (bHLH) DNA-binding superfamily protein | 9.02493 | 1.34671 | -2.74448 | 0.0001 | 0.00123775 |
| AT1G05330 | unknown protein | 15.4411 | 2.30497 | -2.74396 | 0.0068 | 0.029741 |
| AT1G66170 | MMD1, RING/FYVE/PHD zinc finger superfamily protein | 1.89059 | 0.283249 | -2.7387 | 0.0009 | 0.0065867 |
| AT3G53600 | C2H2-type zinc finger family protein | 819.368 | 122.918 | -2.73682 | 0.00005 | 0.00070028 |
| AT5G07220 | ATBAG3, BAG3, BCL-2-associated athanogene 3 | 263.913 | 39.6005 | -2.73647 | 0.00005 | 0.00070028 |
| AT3G22250 | UDP-Glycosyltransferase superfamily protein | 25.7965 | 3.87234 | -2.7359 | 0.00005 | 0.00070028 |
| AT2G02630 | Cysteine/Histidine-rich C1 domain family protein | 1.84969 | 0.277782 | -2.73525 | 0.001 | 0.00711095 |
| AT4G23870 | unknown protein | 6.33461 | 0.952756 | -2.73308 | 0.0016 | 0.0101494 |
| AT4G03500 | Ankyrin repeat family protein | 16.2964 | 2.4529 | -2.732 | 0.00005 | 0.00070028 |
| AT3G62450 | unknown protein | 221.769 | 33.3836 | -2.73185 | 0.0062 | 0.0278676 |
| AT1G01740 | Protein kinase protein with tetratricopeptide repeat domain | 13.6531 | 2.05532 | -2.7318 | 0.00005 | 0.00070028 |
| AT4G18630 | Protein of unknown function (DUF688) | 9.49518 | 1.4296 | -2.73158 | 0.00025 | 0.00253767 |
| AT1G23020 | ATFRO3, FRO3, ferric reduction oxidase 3 | 21.1704 | 3.19104 | -2.72995 | 0.00005 | 0.00070028 |
| AT1G23750 | Nucleic acid-binding, OB-fold-like protein | 161.203 | 24.3002 | -2.72984 | 0.00005 | 0.00070028 |
| AT1G48670 | auxin-responsive GH3 family protein | 1.29426 | 0.195272 | -2.72857 | 0.012 | 0.0450518 |
| AT3G13650 | Disease resistance-responsive (dirigent-like protein) family protein | 137.574 | 20.828 | -2.72361 | 0.00005 | 0.00070028 |
| AT4G06746 | DEAR5, RAP2.9, related to AP2 9 | 144.094 | 21.8161 | -2.72354 | 0.00005 | 0.00070028 |
| AT4G01820 | MDR3, PGP3, P-glycoprotein 3 | 3.15113 | 0.477195 | -2.72322 | 0.00005 | 0.00070028 |
| AT5G62100 | ATBAG2, BAG2, BCL-2-associated athanogene 2 | 2.66142 | 0.4036 | -2.7212 | 0.00835 | 0.0345732 |
| AT1G56700 | Peptidase C15, pyroglutamyl peptidase I-like | 135.873 | 20.6475 | -2.71822 | 0.00005 | 0.00070028 |
| AT1G18310 | glycosyl hydrolase family 81 protein | 1.07641 | 0.163659 | -2.71746 | 0.0084 | 0.0346931 |
| AT2G21430 | Papain family cysteine protease | 4.37994 | 0.666511 | -2.71621 | 0.00065 | 0.00516972 |
| AT3G20410 | CPK9, calmodulin-domain protein kinase 9 | 213.485 | 32.5019 | -2.71554 | 0.00005 | 0.00070028 |
| AT3G03520 | NPC3, non-specific phospholipase C3 | 109.385 | 16.6552 | -2.71536 | 0.00005 | 0.00070028 |
| AT3G41762 | unknown protein | 31.5833 | 4.81253 | -2.7143 | 0.0022 | 0.0128446 |
| AT5G23220 | NIC3, nicotinamidase 3 | 203.627 | 31.0288 | -2.71425 | 0.00005 | 0.00070028 |
| AT5G60060 | Protein of unknown function (DUF295) | 11.0925 | 1.69081 | -2.71379 | 0.00045 | 0.003919 |
| AT5G45520 | Leucine-rich repeat (LRR) family protein | 1.26185 | 0.19259 | -2.71193 | 0.0014 | 0.00917155 |
| AT3G23600 | alpha/beta-Hydrolases superfamily protein | 655.149 | 100.14 | -2.70981 | 0.00005 | 0.00070028 |
| AT5G01830 | ARM repeat superfamily protein | 85.2762 | 13.036 | -2.70964 | 0.00005 | 0.00070028 |
| AT4G03440 | Ankyrin repeat family protein | 9.42261 | 1.44462 | -2.70544 | 0.00005 | 0.00070028 |
| AT1G76080 | ATCDSP32, CDSP32, chloroplastic drought-induced stress protein of 32 kD | 9.66811 | 1.48445 | -2.7033 | 0.00015 | 0.00170235 |
| AT2G26480 | UGT76D1, UDP-glucosyl transferase 76D1 | 54.487 | 8.38316 | -2.70035 | 0.00005 | 0.00070028 |
| AT4G16563 | Eukaryotic aspartyl protease family protein | 7.0778 | 1.08938 | -2.69979 | 0.00035 | 0.00327416 |
| AT1G56660 | unknown protein | 519.348 | 80.1407 | -2.6961 | 0.00005 | 0.00070028 |
| AT4G04700 | CPK27, calcium-dependent protein kinase 27 | 16.0592 | 2.47984 | -2.69508 | 0.00015 | 0.00170235 |
| AT5G41300 | Receptor-like protein kinase-related family protein | 11.2169 | 1.7369 | -2.69109 | 0.0007 | 0.00547072 |
| AT2G02960 | RING/FYVE/PHD zinc finger superfamily protein | 272.174 | 42.1596 | -2.6906 | 0.00005 | 0.00070028 |
| AT3G26470 | Powdery mildew resistance protein, RPW8 domain | 6.09926 | 0.945453 | -2.68956 | 0.00155 | 0.009918 |
| AT1G79270 | ECT8, evolutionarily conserved C-terminal region 8 | 122.041 | 18.9737 | -2.6853 | 0.00005 | 0.00070028 |
| AT5G27930 | Protein phosphatase 2C family protein | 96.7972 | 15.0631 | -2.68395 | 0.00005 | 0.00070028 |
| AT5G52900 | unknown protein | 3.78403 | 0.589383 | -2.68265 | 0.0041 | 0.0203755 |
| AT4G10500 | 2-oxoglutarate (2OG) and Fe(II)-dependent oxygenase superfamily protein | 16.0491 | 2.50061 | -2.68214 | 0.00005 | 0.00070028 |
| AT5G19240 | Glycoprotein membrane precursor GPI-anchored | 61.5293 | 9.59602 | -2.68076 | 0.00005 | 0.00070028 |
| AT4G37010 | CEN2, centrin 2 | 122.131 | 19.1228 | -2.67505 | 0.00005 | 0.00070028 |
| AT2G46940 | unknown protein | 24.4941 | 3.83807 | -2.67398 | 0.0001 | 0.00123775 |
| AT5G48657 | defense protein-related | 107.606 | 16.8773 | -2.67261 | 0.00005 | 0.00070028 |
| AT2G05910 | Protein of unknown function (DUF567) | 6.75198 | 1.05939 | -2.67208 | 0.00165 | 0.0103801 |
| AT2G23340 | DEAR3, DREB and EAR motif protein 3 | 58.0188 | 9.1049 | -2.67181 | 0.00005 | 0.00070028 |
| AT3G16690 | Nodulin MtN3 family protein | 119.137 | 18.8178 | -2.66245 | 0.00005 | 0.00070028 |
| AT3G23800 | SBP3, selenium-binding protein 3 | 29.2415 | 4.6188 | -2.66243 | 0.00005 | 0.00070028 |
| AT1G35670 | ATCDPK2, ATCPK11, CDPK2, CPK11, calcium-dependent protein kinase 2 | 322.302 | 51.0252 | -2.65913 | 0.00005 | 0.00070028 |
| AT3G04630 | WDL1, WVD2-like 1 | 90.8727 | 14.4064 | -2.65713 | 0.00005 | 0.00070028 |
| AT4G12410 | SAUR-like auxin-responsive protein family | 8.42949 | 1.33892 | -2.65437 | 0.00565 | 0.0260215 |
| AT1G75840 | ARAC5, ATGP3, ATROP4, ROP4, RAC-like GTP binding protein 5 | 85.6157 | 13.6596 | -2.64796 | 0.00065 | 0.00516972 |
| AT4G27410 | ANAC072, RD26, NAC (No Apical Meristem) domain transcriptional regulator superfamily protein | 201.019 | 32.0744 | -2.64784 | 0.00005 | 0.00070028 |
| AT2G44790 | UCC2, uclacyanin 2 | 1170.14 | 186.929 | -2.64612 | 0.00005 | 0.00070028 |
| AT3G52740 | unknown protein | 75.4654 | 12.0904 | -2.64196 | 0.00005 | 0.00070028 |
| AT1G64530 | Plant regulator RWP-RK family protein | 77.2329 | 12.3854 | -2.64057 | 0.00005 | 0.00070028 |
| AT3G14370 | WAG2, Protein kinase superfamily protein | 55.5131 | 8.93205 | -2.63577 | 0.00005 | 0.00070028 |
| AT5G65930 | KCBP, PKCBP, ZWI, kinesin-like calmodulin-binding protein (ZWICHEL) | 88.8466 | 14.3017 | -2.63513 | 0.00005 | 0.00070028 |
| AT5G12340 | unknown protein | 223.717 | 36.0318 | -2.63433 | 0.00005 | 0.00070028 |
| AT2G17845 | NAD(P)-binding Rossmann-fold superfamily protein | 14.4517 | 2.33067 | -2.63242 | 0.00015 | 0.00170235 |
| AT2G01430 | ATHB-17, ATHB17, HB17, homeobox-leucine zipper protein 17 | 4.00225 | 0.64577 | -2.63172 | 0.0062 | 0.0278676 |
| AT2G33830 | Dormancy/auxin associated family protein | 144.465 | 23.3838 | -2.62714 | 0.00005 | 0.00070028 |
| AT4G34750 | SAUR-like auxin-responsive protein family | 16.9547 | 2.74517 | -2.62672 | 0.00035 | 0.00327416 |
| AT3G61270 | Arabidopsis thaliana protein of unknown function (DUF821) | 27.2833 | 4.41893 | -2.62625 | 0.00005 | 0.00070028 |
| AT5G57740 | XBAT32, XB3 ortholog 2 in Arabidopsis thaliana | 46.6273 | 7.55527 | -2.62562 | 0.00005 | 0.00070028 |
| AT1G60130 | Mannose-binding lectin superfamily protein | 4.26065 | 0.690922 | -2.62448 | 0.00145 | 0.00940576 |
| AT4G14465 | AHL20, AT-hook motif nuclear-localized protein 20 | 17.7409 | 2.88216 | -2.62186 | 0.00005 | 0.00070028 |
| AT4G28150 | Protein of unknown function (DUF789) | 5.67665 | 0.922664 | -2.62116 | 0.00135 | 0.0089118 |
| AT3G59060 | PIF5, PIL6, phytochrome interacting factor 3-like 6 | 1.19621 | 0.19472 | -2.619 | 0.00365 | 0.0186349 |
| AT4G18940 | RNA ligase/cyclic nucleotide phosphodiesterase family protein | 21.8767 | 3.56177 | -2.61872 | 0.0007 | 0.00547072 |
| AT5G20260 | Exostosin family protein | 20.7634 | 3.38258 | -2.61785 | 0.0001 | 0.00123775 |
| AT4G30980 | LRL2, LJRHL1-like 2 | 18.4855 | 3.01509 | -2.61612 | 0.00005 | 0.00070028 |
| AT2G34830 | AtWRKY35, MEE24, WRKY35, WRKY DNA-binding protein 35 | 28.5706 | 4.66111 | -2.61579 | 0.00005 | 0.00070028 |
| AT5G14120 | Major facilitator superfamily protein | 80.9475 | 13.2065 | -2.61573 | 0.00005 | 0.00070028 |
| AT1G18860 | ATWRKY61, WRKY61, WRKY DNA-binding protein 61 | 71.9443 | 11.7393 | -2.61553 | 0.00005 | 0.00070028 |
| AT3G62780 | Calcium-dependent lipid-binding (CaLB domain) family protein | 4.07743 | 0.665696 | -2.61473 | 0.006 | 0.027135 |
| AT4G19950 | unknown protein | 2.22853 | 0.364077 | -2.61378 | 0.0075 | 0.0319763 |
| AT3G16470 | JR1, Mannose-binding lectin superfamily protein | 14.2405 | 2.32649 | -2.61377 | 0.00005 | 0.00070028 |
| AT3G61260 | Remorin family protein | 418.234 | 68.3455 | -2.61339 | 0.00005 | 0.00070028 |
| AT1G66160 | ATCMPG1, CMPG1, CYS, MET, PRO, and GLY protein 1 | 169.823 | 27.7535 | -2.61329 | 0.00005 | 0.00070028 |
| AT4G17100 | CONTAINS InterPro DOMAIN/s: Endoribonuclease XendoU (InterPro:IPR018998) | 252.679 | 41.4201 | -2.60891 | 0.00005 | 0.00070028 |
| AT5G18680 | AtTLP11, TLP11, tubby like protein 11 | 79.2469 | 12.9936 | -2.60855 | 0.00005 | 0.00070028 |
| AT2G37290 | Ypt/Rab-GAP domain of gyp1p superfamily protein | 13.7737 | 2.26582 | -2.60381 | 0.00005 | 0.00070028 |
| AT5G66580 | unknown protein | 83.2222 | 13.6914 | -2.6037 | 0.00005 | 0.00070028 |
| AT5G47250 | LRR and NB-ARC domains-containing disease resistance protein | 10.4161 | 1.71412 | -2.60328 | 0.00005 | 0.00070028 |
| AT5G60660 | PIP2;4, PIP2F, plasma membrane intrinsic protein 2;4 | 165.483 | 27.2862 | -2.60044 | 0.00005 | 0.00070028 |
| AT5G44770 | Cysteine/Histidine-rich C1 domain family protein | 3.52784 | 0.58218 | -2.59925 | 0.00155 | 0.009918 |
| AT3G52460 | hydroxyproline-rich glycoprotein family protein | 35.3533 | 5.83853 | -2.59817 | 0.00005 | 0.00070028 |
| AT5G01840 | ATOFP1, OFP1, ovate family protein 1 | 6.58961 | 1.08844 | -2.59794 | 0.0011 | 0.00759303 |
| AT3G19390 | Granulin repeat cysteine protease family protein | 1148.84 | 190.392 | -2.59313 | 0.00005 | 0.00070028 |
| AT2G37750 | unknown protein | 194.203 | 32.2008 | -2.5924 | 0.00005 | 0.00070028 |
| AT3G19920 | unknown protein | 7.31043 | 1.21619 | -2.58759 | 0.0008 | 0.00603091 |
| AT4G14580 | CIPK4, SnRK3.3, CBL-interacting protein kinase 4 | 1.64706 | 0.274195 | -2.58662 | 0.01295 | 0.0475634 |
| AT4G29210 | GGT3, GGT4, gamma-glutamyl transpeptidase 4 | 41.2656 | 6.87892 | -2.58468 | 0.0006 | 0.00487984 |
| AT2G25260 | unknown protein | 70.7576 | 11.7957 | -2.58463 | 0.00005 | 0.00070028 |
| AT1G22830 | Tetratricopeptide repeat (TPR)-like superfamily protein | 46.7237 | 7.80635 | -2.58144 | 0.00005 | 0.00070028 |
| AT5G41830 | RNI-like superfamily protein | 3.40181 | 0.568359 | -2.58143 | 0.00225 | 0.0130442 |
| AT2G39518 | Uncharacterised protein family (UPF0497) | 84.8301 | 14.1797 | -2.58075 | 0.00005 | 0.00070028 |
| AT5G47980 | HXXXD-type acyl-transferase family protein | 23.9769 | 4.01052 | -2.57979 | 0.00005 | 0.00070028 |
| AT3G15518 | unknown protein | 52.0407 | 8.70883 | -2.57909 | 0.0011 | 0.00759303 |
| AT5G51160 | Ankyrin repeat family protein | 28.485 | 4.77672 | -2.57611 | 0.00005 | 0.00070028 |
| AT4G33050 | EDA39, calmodulin-binding family protein | 7.54189 | 1.26527 | -2.57548 | 0.00035 | 0.00327416 |
| AT2G29730 | UGT71D1, UDP-glucosyl transferase 71D1 | 14.7789 | 2.48924 | -2.56976 | 0.00005 | 0.00070028 |
| AT5G19220 | ADG2, APL1, ADP glucose pyrophosphorylase large subunit 1 | 20.5738 | 3.47471 | -2.56584 | 0.0046 | 0.0222191 |
| AT4G12690 | Plant protein of unknown function (DUF868) | 7.12832 | 1.2062 | -2.56309 | 0.00045 | 0.003919 |
| AT5G01640 | PRA1.B5, prenylated RAB acceptor 1.B5 | 41.2527 | 6.98327 | -2.56251 | 0.0001 | 0.00123775 |
| AT4G29550 | Protein of unknown function (DUF626) | 4.15822 | 0.704024 | -2.56227 | 0.00135 | 0.0089118 |
| AT4G11300 | Protein of unknown function (DUF793) | 6.62397 | 1.12289 | -2.56049 | 0.00115 | 0.00787231 |
| AT1G75410 | BLH3, BEL1-like homeodomain 3 | 16.2989 | 2.76465 | -2.55961 | 0.00005 | 0.00070028 |
| AT5G13500 | unknown protein | 217.879 | 36.9633 | -2.55936 | 0.00005 | 0.00070028 |
| AT2G38090 | Duplicated homeodomain-like superfamily protein | 14.1479 | 2.40107 | -2.55884 | 0.00005 | 0.00070028 |
| AT5G36260 | Eukaryotic aspartyl protease family protein | 15.322 | 2.60715 | -2.55506 | 0.00005 | 0.00070028 |
| AT3G47820 | PUB39, PLANT U-BOX 39 | 10.8078 | 1.83904 | -2.55505 | 0.00005 | 0.00070028 |
| AT1G63530 | BEST Arabidopsis thaliana protein match is: hydroxyproline-rich glycoprotein family protein (TAIR:AT1G63540.1) | 19.735 | 3.35862 | -2.55481 | 0.00005 | 0.00070028 |
| AT5G13320 | GDG1, GH3.12, PBS3, WIN3, Auxin-responsive GH3 family protein | 1.20584 | 0.205598 | -2.55214 | 0.00575 | 0.0263656 |
| AT1G63540 | hydroxyproline-rich glycoprotein family protein | 4.22751 | 0.720945 | -2.55185 | 0.0011 | 0.00759303 |
| AT3G11773 | Thioredoxin superfamily protein | 11.4881 | 1.9608 | -2.55063 | 0.00835 | 0.0345732 |
| AT1G61560 | ATMLO6, MLO6, Seven transmembrane MLO family protein | 111.56 | 19.044 | -2.55041 | 0.00005 | 0.00070028 |
| AT5G62470 | ATMYB96, MYB96, MYBCOV1, myb domain protein 96 | 2.05094 | 0.3503 | -2.54962 | 0.0024 | 0.0136815 |
| AT5G57190 | PSD2, phosphatidylserine decarboxylase 2 | 14.9324 | 2.55125 | -2.54917 | 0.00005 | 0.00070028 |
| AT5G02400 | PLL2, pol-like 2 | 4.77503 | 0.816618 | -2.54778 | 0.0005 | 0.00424432 |
| AT5G64510 | unknown protein | 86.0094 | 14.7539 | -2.54339 | 0.00005 | 0.00070028 |
| AT4G36120 | Plant protein of unknown function (DUF869) | 13.811 | 2.36961 | -2.5431 | 0.00005 | 0.00070028 |
| AT4G13420 | ATHAK5, HAK5, high affinity K+ transporter 5 | 9.83467 | 1.68897 | -2.54173 | 0.00015 | 0.00170235 |
| AT1G62975 | basic helix-loop-helix (bHLH) DNA-binding superfamily protein | 36.3364 | 6.26109 | -2.53693 | 0.00005 | 0.00070028 |
| AT2G04430 | atnudt5, NUDT5, nudix hydrolase homolog 5 | 32.2966 | 5.58723 | -2.53118 | 0.00005 | 0.00070028 |
| AT5G38030 | MATE efflux family protein | 1.72729 | 0.299381 | -2.52846 | 0.00925 | 0.0371953 |
| AT1G72900 | Toll-Interleukin-Resistance (TIR) domain-containing protein | 26.361 | 4.57568 | -2.52635 | 0.00005 | 0.00070028 |
| AT3G09280 | unknown protein | 260.201 | 45.2117 | -2.52486 | 0.00005 | 0.00070028 |
| AT3G20460 | Major facilitator superfamily protein | 31.5123 | 5.47693 | -2.52447 | 0.00005 | 0.00070028 |
| AT5G15950 | Adenosylmethionine decarboxylase family protein | 195.241 | 34.0288 | -2.52043 | 0.00005 | 0.00070028 |
| AT2G37000 | TCP family transcription factor | 3.17257 | 0.553102 | -2.52003 | 0.0075 | 0.0319763 |
| AT5G45000 | Disease resistance protein (TIR-NBS-LRR class) family | 16.0892 | 2.80833 | -2.51831 | 0.0001 | 0.00123775 |
| AT5G21170 | AKINBETA1, 5'-AMP-activated protein kinase beta-2 subunit protein | 104.002 | 18.1796 | -2.51621 | 0.00005 | 0.00070028 |
| AT5G04820 | ATOFP13, OFP13, ovate family protein 13 | 22.7576 | 3.98028 | -2.5154 | 0.00005 | 0.00070028 |
| AT1G79910 | Regulator of Vps4 activity in the MVB pathway protein | 29.603 | 5.17834 | -2.51518 | 0.0002 | 0.00214445 |
| AT5G46520 | Disease resistance protein (TIR-NBS-LRR class) family | 1.16053 | 0.203355 | -2.51272 | 0.0066 | 0.0290976 |
| AT3G13700 | RNA-binding (RRM/RBD/RNP motifs) family protein | 21.7998 | 3.82168 | -2.51204 | 0.0018 | 0.0110912 |
| AT5G13548 | Pseudogene of AT3G12600; ATNUDT16 (Arabidopsis thaliana Nudix hydrolase homolog 16); hydrolase | 219.121 | 38.4399 | -2.51105 | 0.00355 | 0.0182751 |
| AT3G07870 | F-box and associated interaction domains-containing protein | 56.2917 | 9.87512 | -2.51105 | 0.00005 | 0.00070028 |
| AT4G19960 | ATKUP9, HAK9, KT9, KUP9, K+ uptake permease 9 | 15.3728 | 2.69787 | -2.51048 | 0.00005 | 0.00070028 |
| AT5G43780 | APS4, Pseudouridine synthase/archaeosine transglycosylase-like family protein | 148.717 | 26.1097 | -2.50991 | 0.00005 | 0.00070028 |
| AT5G22400 | Rho GTPase activating protein with PAK-box/P21-Rho-binding domain | 42.9908 | 7.55046 | -2.50939 | 0.00005 | 0.00070028 |
| AT1G08340 | Rho GTPase activating protein with PAK-box/P21-Rho-binding domain | 106.051 | 18.6353 | -2.50865 | 0.0001 | 0.00123775 |
| AT1G14540 | Peroxidase superfamily protein | 35.4839 | 6.23932 | -2.50771 | 0.00005 | 0.00070028 |
| AT1G19200 | Protein of unknown function (DUF581) | 87.7559 | 15.4307 | -2.50769 | 0.00005 | 0.00070028 |
| AT4G29220 | PFK1, phosphofructokinase 1 | 59.1846 | 10.4152 | -2.50654 | 0.00005 | 0.00070028 |
| AT3G54990 | SMZ, Integrase-type DNA-binding superfamily protein | 48.1095 | 8.4719 | -2.50556 | 0.00005 | 0.00070028 |
| AT2G17130 | IDH-II, IDH2, isocitrate dehydrogenase subunit 2 | 211.379 | 37.2411 | -2.50486 | 0.0001 | 0.00123775 |
| AT5G02630 | Lung seven transmembrane receptor family protein | 2.62401 | 0.462396 | -2.50458 | 0.0028 | 0.0152969 |
| AT5G46060 | Protein of unknown function, DUF599 | 16.0353 | 2.83077 | -2.50198 | 0.00015 | 0.00170235 |
| AT2G30040 | MAPKKK14, mitogen-activated protein kinase kinase kinase 14 | 125.014 | 22.104 | -2.49971 | 0.00005 | 0.00070028 |
| AT2G20950 | Arabidopsis phospholipase-like protein (PEARLI 4) family | 6.00665 | 1.06215 | -2.49957 | 0.00035 | 0.00327416 |
| AT5G36940 | CAT3, cationic amino acid transporter 3 | 26.4842 | 4.69556 | -2.49576 | 0.00005 | 0.00070028 |
| AT1G79320 | AtMC6, MC6, metacaspase 6 | 29.4424 | 5.22689 | -2.49387 | 0.00005 | 0.00070028 |
| AT3G20520 | SVL3, SHV3-like 3 | 11.8833 | 2.10967 | -2.49385 | 0.0001 | 0.00123775 |
| AT3G13965 | pseudogene, hypothetical protein | 19.7231 | 3.50236 | -2.49349 | 0.00055 | 0.00457655 |
| AT1G14170 | RNA-binding KH domain-containing protein | 181.446 | 32.3096 | -2.4895 | 0.00005 | 0.00070028 |
| AT3G18060 | transducin family protein / WD-40 repeat family protein | 143.205 | 25.5061 | -2.48917 | 0.00005 | 0.00070028 |
| AT5G24530 | DMR6, 2-oxoglutarate (2OG) and Fe(II)-dependent oxygenase superfamily protein | 81.4499 | 14.5107 | -2.4888 | 0.00005 | 0.00070028 |
| AT2G19760 | PFN1, PRF1, profilin 1 | 609.899 | 108.766 | -2.48734 | 0.00005 | 0.00070028 |
| AT4G05150 | Octicosapeptide/Phox/Bem1p family protein | 228.29 | 40.7379 | -2.48642 | 0.00005 | 0.00070028 |
| AT5G47230 | ATERF-5, ATERF5, ERF5, ethylene responsive element binding factor 5 | 136.589 | 24.3925 | -2.48533 | 0.00005 | 0.00070028 |
| AT3G18200 | nodulin MtN21 /EamA-like transporter family protein | 80.5179 | 14.3929 | -2.48395 | 0.00005 | 0.00070028 |
| AT5G44020 | HAD superfamily, subfamily IIIB acid phosphatase | 1990.44 | 355.83 | -2.48383 | 0.0001 | 0.00123775 |
| AT2G45210 | SAUR-like auxin-responsive protein family | 3.50308 | 0.626337 | -2.48361 | 0.00195 | 0.0117451 |
| AT4G00955 | function unknown | 5.71203 | 1.02502 | -2.47836 | 0.00275 | 0.0151029 |
| AT3G21230 | 4CL5, 4-coumarate:CoA ligase 5 | 37.0357 | 6.6544 | -2.47654 | 0.00005 | 0.00070028 |
| AT2G26570 | Plant protein of unknown function (DUF827) | 46.5513 | 8.36438 | -2.47649 | 0.00005 | 0.00070028 |
| AT3G60980 | Tetratricopeptide repeat (TPR)-like superfamily protein | 66.5243 | 11.9634 | -2.47525 | 0.00005 | 0.00070028 |
| AT3G20830 | AGC (cAMP-dependent, cGMP-dependent and protein kinase C) kinase family protein | 11.4152 | 2.05503 | -2.47373 | 0.0012 | 0.00815259 |
| AT5G06300 | Putative lysine decarboxylase family protein | 683.151 | 123.097 | -2.47241 | 0.00005 | 0.00070028 |
| AT4G26850 | VTC2, mannose-1-phosphate guanylyltransferase (GDP)s;GDP-galactose:mannose-1-phosphate guanylyltransferases;GDP-galactose:glucose-1-phosphate guanylyltransferases;GDP-galactose:myoinositol-1-phosphate guanylyltransferases;glucose-1-phosphate guanylyltransferase | 32.0837 | 5.78333 | -2.47187 | 0.00005 | 0.00070028 |
| AT2G17880 | Chaperone DnaJ-domain superfamily protein | 45.2704 | 8.16076 | -2.47179 | 0.00005 | 0.00070028 |
| AT2G27505 | FBD-like domain family protein | 7.27167 | 1.31178 | -2.47076 | 0.00225 | 0.0130442 |
| AT4G35783 | DVL17, RTFL6, ROTUNDIFOLIA like 6 | 22.4939 | 4.06662 | -2.46763 | 0.0063 | 0.0281701 |
| AT1G04445 | C2H2-like zinc finger protein | 16.4878 | 2.98234 | -2.46688 | 0.0036 | 0.0184414 |
| AT1G23560 | Domain of unknown function (DUF220) | 5.0527 | 0.914674 | -2.46573 | 0.00795 | 0.0333705 |
| AT4G10370 | Cysteine/Histidine-rich C1 domain family protein | 17.9892 | 3.26055 | -2.46395 | 0.00005 | 0.00070028 |
| AT5G05380 | PRA1.B3, prenylated RAB acceptor 1.B3 | 27.8001 | 5.04465 | -2.46226 | 0.00015 | 0.00170235 |
| AT5G25250 | SPFH/Band 7/PHB domain-containing membrane-associated protein family | 15.721 | 2.85528 | -2.46099 | 0.00025 | 0.00253767 |
| AT1G17340 | Phosphoinositide phosphatase family protein | 191.977 | 34.9336 | -2.45825 | 0.00005 | 0.00070028 |
| AT5G05400 | LRR and NB-ARC domains-containing disease resistance protein | 6.73759 | 1.22751 | -2.45649 | 0.00005 | 0.00070028 |
| AT3G44900 | ATCHX4, CHX4, cation/H+ exchanger 4 | 5.17185 | 0.942873 | -2.45555 | 0.0003 | 0.00292382 |
| AT4G17215 | Pollen Ole e 1 allergen and extensin family protein | 22.3471 | 4.07523 | -2.45513 | 0.0004 | 0.00359419 |
| AT4G36880 | CP1, cysteine proteinase1 | 192.723 | 35.1567 | -2.45466 | 0.00005 | 0.00070028 |
| AT3G02150 | PTF1, TCP13, TFPD, plastid transcription factor 1 | 17.4453 | 3.18378 | -2.45403 | 0.0001 | 0.00123775 |
| AT1G28370 | ATERF11, ERF11, ERF domain protein 11 | 23.8071 | 4.35025 | -2.45222 | 0.00005 | 0.00070028 |
| AT3G27300 | G6PD5, glucose-6-phosphate dehydrogenase 5 | 141.963 | 25.9497 | -2.45173 | 0.00005 | 0.00070028 |
| AT3G04880 | DRT102, DNA-damage-repair/toleration protein (DRT102) | 79.6404 | 14.5778 | -2.44973 | 0.0024 | 0.0136815 |
| AT1G23030 | ARM repeat superfamily protein | 51.4383 | 9.41957 | -2.44911 | 0.00005 | 0.00070028 |
| AT5G51780 | basic helix-loop-helix (bHLH) DNA-binding superfamily protein | 27.1065 | 4.97286 | -2.44649 | 0.0002 | 0.00214445 |
| AT1G75960 | AMP-dependent synthetase and ligase family protein | 19.483 | 3.57696 | -2.44541 | 0.0001 | 0.00123775 |
| AT5G02640 | unknown protein | 3.99626 | 0.734672 | -2.44348 | 0.00855 | 0.0351514 |
| AT2G24330 | Protein of unknown function (DUF2296) | 47.4154 | 8.73636 | -2.44025 | 0.00005 | 0.00070028 |
| AT1G18290 | unknown protein | 13.7301 | 2.53331 | -2.43824 | 0.0004 | 0.00359419 |
| AT2G11280 | pseudogene, hypothetical protein | 8.30551 | 1.53259 | -2.43809 | 0.001 | 0.00711095 |
| AT4G02410 | Concanavalin A-like lectin protein kinase family protein | 1.97336 | 0.36432 | -2.43738 | 0.00225 | 0.0130442 |
| AT3G49360 | PGL2, 6-phosphogluconolactonase 2 | 4.68973 | 0.866203 | -2.43673 | 0.00165 | 0.0103801 |
| AT4G24310 | Protein of unknown function (DUF679) | 67.2739 | 12.4374 | -2.43536 | 0.00005 | 0.00070028 |
| AT4G28030 | Acyl-CoA N-acyltransferases (NAT) superfamily protein | 10.3257 | 1.91132 | -2.4336 | 0.00095 | 0.00685384 |
| AT5G17490 | RGL3, RGA-like protein 3 | 12.5023 | 2.31459 | -2.43337 | 0.0002 | 0.00214445 |
| AT3G51470 | Protein phosphatase 2C family protein | 10.1581 | 1.88337 | -2.43124 | 0.0003 | 0.00292382 |
| AT5G20885 | RING/U-box superfamily protein | 99.3007 | 18.4252 | -2.43012 | 0.00005 | 0.00070028 |
| AT5G15630 | COBL4, IRX6, COBRA-like extracellular glycosyl-phosphatidyl inositol-anchored protein family | 2.94826 | 0.547178 | -2.42978 | 0.0028 | 0.0152969 |
| AT4G40070 | RING/U-box superfamily protein | 16.7292 | 3.12417 | -2.42083 | 0.00015 | 0.00170235 |
| AT1G12710 | AtPP2-A12, PP2-A12, phloem protein 2-A12 | 51.9472 | 9.70666 | -2.42 | 0.00005 | 0.00070028 |
| AT1G77450 | anac032, NAC032, NAC domain containing protein 32 | 205.205 | 38.3499 | -2.41977 | 0.00005 | 0.00070028 |
| AT2G30840 | 2-oxoglutarate (2OG) and Fe(II)-dependent oxygenase superfamily protein | 2.45811 | 0.460706 | -2.41564 | 0.012 | 0.0450518 |
| AT1G01680 | ATPUB54, PUB54, plant U-box 54 | 14.0603 | 2.63551 | -2.41547 | 0.00025 | 0.00253767 |
| AT4G26690 | GPDL2, MRH5, SHV3, PLC-like phosphodiesterase family protein | 215.115 | 40.482 | -2.40976 | 0.00005 | 0.00070028 |
| AT2G26440 | Plant invertase/pectin methylesterase inhibitor superfamily | 4.43266 | 0.834946 | -2.40842 | 0.00165 | 0.0103801 |
| AT3G07570 | Cytochrome b561/ferric reductase transmembrane with DOMON related domain | 73.5843 | 13.8761 | -2.4068 | 0.00005 | 0.00070028 |
| AT2G03980 | GDSL-like Lipase/Acylhydrolase superfamily protein | 8.484 | 1.60043 | -2.40629 | 0.0004 | 0.00359419 |
| AT2G38080 | ATLMCO4, IRX12, LAC4, LMCO4, Laccase/Diphenol oxidase family protein | 3.72969 | 0.703765 | -2.40589 | 0.00175 | 0.0108609 |
| AT1G62660 | Glycosyl hydrolases family 32 protein | 426.585 | 80.577 | -2.40439 | 0.00005 | 0.00070028 |
| AT4G01120 | ATBZIP54, GBF2, G-box binding factor 2 | 52.3565 | 9.89213 | -2.40402 | 0.00005 | 0.00070028 |
| AT1G62440 | LRX2, leucine-rich repeat/extensin 2 | 90.8058 | 17.1865 | -2.40151 | 0.00005 | 0.00070028 |
| AT4G04695 | CPK31, calcium-dependent protein kinase 31 | 10.0239 | 1.89791 | -2.40096 | 0.00045 | 0.003919 |
| AT1G58270 | ZW9, TRAF-like family protein | 251.666 | 47.6801 | -2.40005 | 0.00005 | 0.00070028 |
| AT4G33360 | FLDH, NAD(P)-binding Rossmann-fold superfamily protein | 304.888 | 57.7994 | -2.39915 | 0.00005 | 0.00070028 |
| AT5G08240 | unknown protein | 68.5301 | 13.01 | -2.39712 | 0.00005 | 0.00070028 |
| AT1G15680 | F-box family protein | 1.77711 | 0.337676 | -2.39582 | 0.01255 | 0.0465083 |
| AT2G39570 | ACT domain-containing protein | 208.337 | 39.6954 | -2.39188 | 0.00005 | 0.00070028 |
| AT5G43180 | Protein of unknown function, DUF599 | 9.97131 | 1.90042 | -2.39146 | 0.00085 | 0.00630464 |
| AT3G46400 | Leucine-rich repeat protein kinase family protein | 3.5756 | 0.681735 | -2.3909 | 0.0017 | 0.0106071 |
| AT3G47540 | Chitinase family protein | 3.33021 | 0.634983 | -2.39082 | 0.01195 | 0.0449578 |
| AT4G36032 | Potential natural antisense gene, locus overlaps with AT4G36030 | 32.5494 | 6.21787 | -2.38814 | 0.00005 | 0.00070028 |
| AT1G25460 | NAD(P)-binding Rossmann-fold superfamily protein | 3.82986 | 0.731831 | -2.38771 | 0.0116 | 0.0439836 |
| AT1G68440 | unknown protein | 821.724 | 157.151 | -2.3865 | 0.00005 | 0.00070028 |
| AT1G30640 | Protein kinase family protein | 80.037 | 15.3104 | -2.38616 | 0.00005 | 0.00070028 |
| AT1G58360 | AAP1, NAT2, amino acid permease 1 | 42.3092 | 8.09489 | -2.38589 | 0.00005 | 0.00070028 |
| AT2G22170 | Lipase/lipooxygenase, PLAT/LH2 family protein | 444.232 | 85.2754 | -2.38111 | 0.00005 | 0.00070028 |
| AT4G15990 | unknown protein | 79.088 | 15.1879 | -2.38053 | 0.0005 | 0.00424432 |
| AT1G72790 | hydroxyproline-rich glycoprotein family protein | 72.7564 | 13.9791 | -2.3798 | 0.00005 | 0.00070028 |
| AT2G40420 | Transmembrane amino acid transporter family protein | 40.1045 | 7.70715 | -2.37949 | 0.00005 | 0.00070028 |
| AT2G27080 | Late embryogenesis abundant (LEA) hydroxyproline-rich glycoprotein family | 149.092 | 28.6707 | -2.37855 | 0.00045 | 0.003919 |
| AT5G25890 | IAA28, IAR2, indole-3-acetic acid inducible 28 | 174.637 | 33.5893 | -2.37829 | 0.00005 | 0.00070028 |
| AT4G26050 | PIRL8, plant intracellular ras group-related LRR 8 | 13.5315 | 2.60432 | -2.37735 | 0.00015 | 0.00170235 |
| AT5G65990 | Transmembrane amino acid transporter family protein | 74.4234 | 14.3419 | -2.37552 | 0.00005 | 0.00070028 |
| AT5G41710.1 | transposable element gene | 8.58781 | 1.65892 | -2.37205 | 0.00005 | 0.00070028 |
| AT5G23750 | Remorin family protein | 55.5246 | 10.7293 | -2.37156 | 0.00005 | 0.00070028 |
| AT5G17460 | unknown protein | 280.23 | 54.1676 | -2.37111 | 0.00005 | 0.00070028 |
| AT3G28920 | AtHB34, HB34, homeobox protein 34 | 9.49959 | 1.83695 | -2.37055 | 0.00225 | 0.0130442 |
| AT4G08040 | ACS11, 1-aminocyclopropane-1-carboxylate synthase 11 | 5.301 | 1.02531 | -2.37021 | 0.0023 | 0.0132526 |
| AT1G80230 | Rubredoxin-like superfamily protein | 240.118 | 46.4778 | -2.36913 | 0.00005 | 0.00070028 |
| AT4G15093 | catalytic LigB subunit of aromatic ring-opening dioxygenase family | 131.876 | 25.5678 | -2.36678 | 0.00005 | 0.00070028 |
| AT3G15630 | unknown protein | 848.819 | 164.601 | -2.36648 | 0.00005 | 0.00070028 |
| AT2G39110 | Protein kinase superfamily protein | 285.64 | 55.4133 | -2.3659 | 0.00005 | 0.00070028 |
| AT4G33470 | ATHDA14, hda14, histone deacetylase 14 | 5.09225 | 0.989774 | -2.36313 | 0.00095 | 0.00685384 |
| AT5G63595 | ATFLS4, FLS4, flavonol synthase 4 | 4.64365 | 0.903461 | -2.36172 | 0.00315 | 0.0166977 |
| AT3G53370 | S1FA-like DNA-binding protein | 40.7762 | 7.93863 | -2.36077 | 0.00025 | 0.00253767 |
| AT5G62865 | unknown protein | 77.5281 | 15.0966 | -2.3605 | 0.0001 | 0.00123775 |
| AT4G13620 | Integrase-type DNA-binding superfamily protein | 9.85908 | 1.91991 | -2.36041 | 0.00045 | 0.003919 |
| AT1G21100 | O-methyltransferase family protein | 6.22541 | 1.21403 | -2.35836 | 0.00155 | 0.009918 |
| AT5G16910 | ATCSLD2, CSLD2, cellulose-synthase like D2 | 315.453 | 61.5323 | -2.35801 | 0.00005 | 0.00070028 |
| AT3G23090 | TPX2 (targeting protein for Xklp2) protein family | 89.0892 | 17.3789 | -2.35792 | 0.00005 | 0.00070028 |
| AT1G30650 | AR411, ATWRKY14, WRKY14, WRKY DNA-binding protein 14 | 47.0529 | 9.182 | -2.3574 | 0.00005 | 0.00070028 |
| AT1G30370 | alpha/beta-Hydrolases superfamily protein | 2.57269 | 0.503002 | -2.35464 | 0.0041 | 0.0203755 |
| AT2G28200 | C2H2-type zinc finger family protein | 19.3203 | 3.78208 | -2.35286 | 0.00005 | 0.00070028 |
| AT4G28640 | IAA11, indole-3-acetic acid inducible 11 | 54.5393 | 10.6777 | -2.3527 | 0.00005 | 0.00070028 |
| AT5G44820 | Nucleotide-diphospho-sugar transferase family protein | 44.0843 | 8.63482 | -2.35203 | 0.00005 | 0.00070028 |
| AT3G24420 | alpha/beta-Hydrolases superfamily protein | 15.8116 | 3.09828 | -2.35144 | 0.0003 | 0.00292382 |
| AT3G23810 | ATSAHH2, SAHH2, S-adenosyl-l-homocysteine (SAH) hydrolase 2 | 38.717 | 7.60556 | -2.34784 | 0.00005 | 0.00070028 |
| AT3G05820 | At-A/N-InvH, INVH, invertase H | 17.3463 | 3.40996 | -2.3468 | 0.0001 | 0.00123775 |
| AT4G25570 | ACYB-2, Cytochrome b561/ferric reductase transmembrane protein family | 540.611 | 106.292 | -2.34655 | 0.00005 | 0.00070028 |
| AT5G27420 | ATL31, CNI1, carbon/nitrogen insensitive 1 | 37.3679 | 7.35327 | -2.34534 | 0.00005 | 0.00070028 |
| AT3G13310 | Chaperone DnaJ-domain superfamily protein | 901.908 | 177.623 | -2.34416 | 0.00005 | 0.00070028 |
| AT4G40020 | Myosin heavy chain-related protein | 2.03292 | 0.400854 | -2.34241 | 0.0036 | 0.0184414 |
| AT1G02610 | RING/FYVE/PHD zinc finger superfamily protein | 62.8885 | 12.4142 | -2.34081 | 0.00005 | 0.00070028 |
| AT1G51790 | Leucine-rich repeat protein kinase family protein | 4.46373 | 0.881674 | -2.33993 | 0.00045 | 0.003919 |
| AT4G01720 | AtWRKY47, WRKY47, WRKY family transcription factor | 22.8129 | 4.51053 | -2.33848 | 0.00005 | 0.00070028 |
| AT3G13380 | BRL3, BRI1-like 3 | 9.26606 | 1.83324 | -2.33756 | 0.00005 | 0.00070028 |
| AT3G15020 | mMDH2, Lactate/malate dehydrogenase family protein | 125.129 | 24.775 | -2.33646 | 0.00005 | 0.00070028 |
| AT1G13110 | CYP71B7, cytochrome P450, family 71 subfamily B, polypeptide 7 | 11.3656 | 2.25497 | -2.33349 | 0.00015 | 0.00170235 |
| AT5G03670 | unknown protein | 32.2972 | 6.40875 | -2.33329 | 0.00005 | 0.00070028 |
| AT5G24165 | unknown protein | 37.6647 | 7.48493 | -2.33115 | 0.00055 | 0.00457655 |
| AT3G29250 | NAD(P)-binding Rossmann-fold superfamily protein | 104.045 | 20.6767 | -2.33113 | 0.00005 | 0.00070028 |
| AT3G14680 | CYP72A14, cytochrome P450, family 72, subfamily A, polypeptide 14 | 2.79947 | 0.556603 | -2.33044 | 0.0031 | 0.0165078 |
| AT2G31020 | ORP1A, OSBP(oxysterol binding protein)-related protein 1A | 71.1677 | 14.1562 | -2.32979 | 0.00005 | 0.00070028 |
| AT1G72300 | Leucine-rich receptor-like protein kinase family protein | 8.64289 | 1.7199 | -2.32919 | 0.00005 | 0.00070028 |
| AT4G17905 | ATL4H, RING/U-box superfamily protein | 11.0973 | 2.20958 | -2.32837 | 0.0006 | 0.00487984 |
| AT3G13810 | AtIDD11, IDD11, indeterminate(ID)-domain 11 | 1.23961 | 0.24684 | -2.32824 | 0.0087 | 0.0356202 |
| AT2G18876 | Afadin/alpha-actinin-binding protein | 46.7595 | 9.31223 | -2.32806 | 0.00005 | 0.00070028 |
| AT5G58730 | pfkB-like carbohydrate kinase family protein | 190.387 | 38.1269 | -2.32005 | 0.00005 | 0.00070028 |
| AT5G60780 | ATNRT2.3, NRT2.3, nitrate transporter 2.3 | 2.98772 | 0.598393 | -2.31988 | 0.0034 | 0.01771 |
| AT4G37790 | HAT22, Homeobox-leucine zipper protein family | 100.781 | 20.199 | -2.31886 | 0.00005 | 0.00070028 |
| AT5G52120 | AtPP2-A14, PP2-A14, phloem protein 2-A14 | 5.31586 | 1.06545 | -2.31883 | 0.0018 | 0.0110912 |
| AT5G62920 | ARR6, response regulator 6 | 130.832 | 26.2483 | -2.31742 | 0.00005 | 0.00070028 |
| AT1G64890 | Major facilitator superfamily protein | 15.8609 | 3.18315 | -2.31695 | 0.00005 | 0.00070028 |
| AT1G06840 | Leucine-rich repeat protein kinase family protein | 69.5418 | 13.9755 | -2.31498 | 0.00005 | 0.00070028 |
| AT1G35730 | APUM9, PUM9, pumilio 9 | 1.99754 | 0.401597 | -2.31441 | 0.0043 | 0.0211046 |
| AT5G21050 | LOCATED IN: chloroplast; EXPRESSED IN: 14 plant structures; EXPRESSED DURING: 8 growth stages; CONTAINS InterPro DOMAIN/s: Hyccin (InterPro:IPR018619); BEST Arabidopsis thaliana protein match is: unknown protein (TAIR:AT5G64090.1); Has 206 Blast hits to 206 proteins in 60 species: Archae - 0; Bacteria - 0; Metazoa - 145; Fungi - 0; Plants - 50; Viruses - 0; Other Eukaryotes - 11 (source: NCBI BLink). | 6.81651 | 1.37136 | -2.31343 | 0.0035 | 0.0180741 |
| AT5G59920 | ULI3, Cysteine/Histidine-rich C1 domain family protein | 1.0775 | 0.216832 | -2.31304 | 0.01015 | 0.0398672 |
| AT1G19210 | Integrase-type DNA-binding superfamily protein | 11.4724 | 2.3099 | -2.31226 | 0.00355 | 0.0182751 |
| AT4G19220 | Tetratricopeptide repeat (TPR)-like superfamily protein | 12.6786 | 2.56004 | -2.30815 | 0.0001 | 0.00123775 |
| AT1G11380 | PLAC8 family protein | 45.9811 | 9.28869 | -2.30749 | 0.00005 | 0.00070028 |
| AT4G35985 | Senescence/dehydration-associated protein-related | 137.36 | 27.7708 | -2.30632 | 0.00005 | 0.00070028 |
| AT1G78310 | VQ motif-containing protein | 54.5883 | 11.0456 | -2.30512 | 0.00005 | 0.00070028 |
| AT1G64590 | NAD(P)-binding Rossmann-fold superfamily protein | 6.00034 | 1.2144 | -2.3048 | 0.00065 | 0.00516972 |
| AT5G51060 | ATRBOHC, RBOHC, RHD2, NADPH/respiratory burst oxidase protein D | 217.368 | 44.0457 | -2.30307 | 0.00005 | 0.00070028 |
| AT4G22760 | Tetratricopeptide repeat (TPR)-like superfamily protein | 2.83829 | 0.575261 | -2.30273 | 0.004 | 0.0200243 |
| AT5G58350 | WNK4, ZIK2, with no lysine (K) kinase 4 | 26.97 | 5.48419 | -2.29801 | 0.0001 | 0.00123775 |
| AT3G46690 | UDP-Glycosyltransferase superfamily protein | 9.29009 | 1.89046 | -2.29696 | 0.0002 | 0.00214445 |
| AT1G52342 | unknown protein | 14.306 | 2.91217 | -2.29645 | 0.00275 | 0.0151029 |
| AT2G44010 | unknown protein | 17.3172 | 3.52548 | -2.29632 | 0.00055 | 0.00457655 |
| AT3G09000 | proline-rich family protein | 53.1497 | 10.8236 | -2.29589 | 0.00005 | 0.00070028 |
| AT2G04500 | Cysteine/Histidine-rich C1 domain family protein | 7.30778 | 1.49049 | -2.29365 | 0.00085 | 0.00630464 |
| AT3G05990 | Leucine-rich repeat (LRR) family protein | 136.921 | 27.9731 | -2.29123 | 0.00005 | 0.00070028 |
| AT1G16170 | unknown protein | 82.7914 | 16.9436 | -2.28874 | 0.00005 | 0.00070028 |
| AT5G52300 | LTI65, RD29B, CAP160 protein | 16.1364 | 3.30409 | -2.28799 | 0.00005 | 0.00070028 |
| AT1G07000 | ATEXO70B2, EXO70B2, exocyst subunit exo70 family protein B2 | 64.7368 | 13.2685 | -2.28658 | 0.00005 | 0.00070028 |
| AT1G12610 | DDF1, Integrase-type DNA-binding superfamily protein | 55.0001 | 11.2748 | -2.28634 | 0.00005 | 0.00070028 |
| AT5G26740 | Protein of unknown function (DUF300) | 132.408 | 27.153 | -2.28581 | 0.00005 | 0.00070028 |
| AT4G02170 | unknown protein | 10.2176 | 2.09727 | -2.28447 | 0.01125 | 0.0429112 |
| AT5G22250 | Polynucleotidyl transferase, ribonuclease H-like superfamily protein | 10.521 | 2.16107 | -2.28346 | 0.00105 | 0.00735817 |
| AT3G43430 | RING/U-box superfamily protein | 123.129 | 25.2947 | -2.28326 | 0.00005 | 0.00070028 |
| AT3G13100 | ATMRP7, MRP7, MRP7, multidrug resistance-associated protein 7 | 24.521 | 5.04793 | -2.28026 | 0.00005 | 0.00070028 |
| AT5G59540 | 2-oxoglutarate (2OG) and Fe(II)-dependent oxygenase superfamily protein | 7.57002 | 1.55911 | -2.27958 | 0.00055 | 0.00457655 |
| AT1G30360 | ERD4, Early-responsive to dehydration stress protein (ERD4) | 115.329 | 23.805 | -2.27643 | 0.00005 | 0.00070028 |
| AT3G46080 | C2H2-type zinc finger family protein | 41.2372 | 8.51227 | -2.27633 | 0.00115 | 0.00787231 |
| AT3G01190 | Peroxidase superfamily protein | 273.852 | 56.5778 | -2.27509 | 0.00005 | 0.00070028 |
| AT1G49030 | PLAC8 family protein | 22.1289 | 4.57406 | -2.27439 | 0.00015 | 0.00170235 |
| AT5G60460 | Preprotein translocase Sec, Sec61-beta subunit protein | 69.7808 | 14.4345 | -2.2733 | 0.00025 | 0.00253767 |
| AT3G46090 | ZAT7, C2H2 and C2HC zinc fingers superfamily protein | 9.00391 | 1.86306 | -2.27288 | 0.0038 | 0.0192125 |
| AT4G21440 | ATM4, ATMYB102, MYB102, MYB102, MYB-like 102 | 25.9516 | 5.37407 | -2.27174 | 0.00015 | 0.00170235 |
| AT5G55970 | RING/U-box superfamily protein | 47.0115 | 9.74342 | -2.27051 | 0.00005 | 0.00070028 |
| AT5G05370 | Cytochrome b-c1 complex, subunit 8 protein | 484.234 | 100.365 | -2.27045 | 0.00005 | 0.00070028 |
| AT1G76070 | unknown protein | 159.589 | 33.0842 | -2.27015 | 0.00005 | 0.00070028 |
| AT5G19025 | Ribosomal protein L34e superfamily protein | 103.039 | 21.3631 | -2.27 | 0.00005 | 0.00070028 |
| AT3G13760 | Cysteine/Histidine-rich C1 domain family protein | 1.67958 | 0.348245 | -2.26993 | 0.00455 | 0.0220583 |
| AT5G55550 | RNA-binding (RRM/RBD/RNP motifs) family protein | 34.1876 | 7.0897 | -2.26968 | 0.00005 | 0.00070028 |
| AT4G20860 | FAD-binding Berberine family protein | 223.47 | 46.4183 | -2.26732 | 0.0004 | 0.00359419 |
| AT5G13200 | GRAM domain family protein | 507.346 | 105.427 | -2.26673 | 0.00005 | 0.00070028 |
| AT1G22110 | structural constituent of ribosome | 13.2423 | 2.75462 | -2.26522 | 0.0003 | 0.00292382 |
| AT4G37620 | transposable element gene | 149.344 | 31.0778 | -2.26468 | 0.00005 | 0.00070028 |
| AT1G34750 | Protein phosphatase 2C family protein | 62.4903 | 13.0327 | -2.2615 | 0.00005 | 0.00070028 |
| AT3G56090 | ATFER3, FER3, ferritin 3 | 537.297 | 112.255 | -2.25894 | 0.00005 | 0.00070028 |
| AT1G31540 | Disease resistance protein (TIR-NBS-LRR class) family | 13.9931 | 2.92638 | -2.25753 | 0.00005 | 0.00070028 |
| AT5G05340 | Peroxidase superfamily protein | 13.2543 | 2.78239 | -2.25207 | 0.00025 | 0.00253767 |
| AT5G06570 | alpha/beta-Hydrolases superfamily protein | 36.0666 | 7.58522 | -2.2494 | 0.00005 | 0.00070028 |
| AT5G52030 | TraB family protein | 10.6033 | 2.23232 | -2.24789 | 0.0002 | 0.00214445 |
| AT3G15440 | BEST Arabidopsis thaliana protein match is: RING/U-box superfamily protein (TAIR:AT3G15740.1) | 48.957 | 10.3117 | -2.24724 | 0.00195 | 0.0117451 |
| AT2G17650 | AMP-dependent synthetase and ligase family protein | 4.55052 | 0.959102 | -2.24628 | 0.00065 | 0.00516972 |
| AT1G53330 | Pentatricopeptide repeat (PPR) superfamily protein | 3.22577 | 0.679999 | -2.24604 | 0.0045 | 0.0218802 |
| AT5G65600 | Concanavalin A-like lectin protein kinase family protein | 1.56894 | 0.330751 | -2.24597 | 0.01165 | 0.0441141 |
| AT2G27820 | ADT3, PD1, prephenate dehydratase 1 | 111.43 | 23.5146 | -2.2445 | 0.00005 | 0.00070028 |
| AT5G37840 | BEST Arabidopsis thaliana protein match is: plastid movement impaired 2 (TAIR:AT1G66480.1) | 4.52025 | 0.956007 | -2.24131 | 0.00475 | 0.0228046 |
| AT4G18010 | 5PTASE2, AT5PTASE2, IP5PII, myo-inositol polyphosphate 5-phosphatase 2 | 17.3211 | 3.66368 | -2.24116 | 0.00005 | 0.00070028 |
| AT4G37340 | CYP81D3, cytochrome P450, family 81, subfamily D, polypeptide 3 | 3.40692 | 0.721171 | -2.24005 | 0.00325 | 0.0171315 |
| AT5G35700 | FIM2, fimbrin-like protein 2 | 104.208 | 22.0692 | -2.23936 | 0.00005 | 0.00070028 |
| AT3G14067 | Subtilase family protein | 179.394 | 38.104 | -2.23512 | 0.0001 | 0.00123775 |
| AT3G27810 | ATMYB21, ATMYB3, MYB21, myb domain protein 21 | 2.57032 | 0.546322 | -2.23412 | 0.0131 | 0.0480164 |
| AT1G10090 | Early-responsive to dehydration stress protein (ERD4) | 39.1357 | 8.32378 | -2.23317 | 0.00005 | 0.00070028 |
| AT2G36210 | SAUR-like auxin-responsive protein family | 12.9229 | 2.74903 | -2.23293 | 0.00215 | 0.0126571 |
| AT5G46870 | RNA-binding (RRM/RBD/RNP motifs) family protein | 43.2856 | 9.2109 | -2.23247 | 0.00025 | 0.00253767 |
| AT1G15010 | unknown protein | 758.503 | 161.428 | -2.23226 | 0.00005 | 0.00070028 |
| AT5G59170 | Proline-rich extensin-like family protein | 2.61203 | 0.555923 | -2.23221 | 0.0063 | 0.0281701 |
| AT3G27650 | LBD25, LOB domain-containing protein 25 | 4.11443 | 0.87623 | -2.23131 | 0.01255 | 0.0465083 |
| AT5G27150 | AT-NHX1, ATNHX, ATNHX1, NHX1, Na+/H+ exchanger 1 | 179.064 | 38.1637 | -2.2302 | 0.00005 | 0.00070028 |
| AT2G12400 | unknown protein | 231.808 | 49.4136 | -2.22995 | 0.00005 | 0.00070028 |
| AT4G33666 | unknown protein | 62.615 | 13.3773 | -2.22672 | 0.01095 | 0.0420749 |
| AT5G49760 | Leucine-rich repeat protein kinase family protein | 50.3669 | 10.7752 | -2.22477 | 0.00005 | 0.00070028 |
| AT2G41730 | unknown protein | 33.5927 | 7.18733 | -2.22462 | 0.0005 | 0.00424432 |
| AT4G19200 | proline-rich family protein | 1242.91 | 266.046 | -2.22397 | 0.00005 | 0.00070028 |
| AT1G12110 | ATNRT1, B-1, CHL1, CHL1-1, NRT1, NRT1.1, nitrate transporter 1.1 | 95.1444 | 20.3747 | -2.22334 | 0.00005 | 0.00070028 |
| AT5G62210 | Embryo-specific protein 3, (ATS3) | 4.0361 | 0.864379 | -2.22323 | 0.0054 | 0.0251864 |
| AT5G54160 | ATOMT1, OMT1, O-methyltransferase 1 | 275.654 | 59.0608 | -2.22259 | 0.00005 | 0.00070028 |
| AT1G56540 | Disease resistance protein (TIR-NBS-LRR class) family | 6.75452 | 1.44745 | -2.22234 | 0.00025 | 0.00253767 |
| AT2G42060 | Cysteine/Histidine-rich C1 domain family protein | 5.72991 | 1.22976 | -2.22013 | 0.00435 | 0.0212815 |
| AT3G52400 | ATSYP122, SYP122, syntaxin of plants 122 | 174.986 | 37.5873 | -2.21892 | 0.00005 | 0.00070028 |
| AT3G06300 | AT-P4H-2, P4H isoform 2 | 269.199 | 57.8294 | -2.2188 | 0.00005 | 0.00070028 |
| AT1G67470 | Protein kinase superfamily protein | 6.39511 | 1.37422 | -2.21836 | 0.0033 | 0.0173075 |
| AT2G36220 | unknown protein | 316.413 | 68.0069 | -2.21805 | 0.00005 | 0.00070028 |
| AT1G61550 | S-locus lectin protein kinase family protein | 6.01644 | 1.29779 | -2.21286 | 0.00025 | 0.00253767 |
| AT1G32170 | XTH30, XTR4, xyloglucan endotransglucosylase/hydrolase 30 | 82.7574 | 17.8588 | -2.21226 | 0.00005 | 0.00070028 |
| AT4G20840 | FAD-binding Berberine family protein | 86.0855 | 18.5794 | -2.21206 | 0.00005 | 0.00070028 |
| AT5G49850 | Mannose-binding lectin superfamily protein | 2.38981 | 0.515843 | -2.21189 | 0.00375 | 0.0189985 |
| AT1G25400 | unknown protein | 406.952 | 87.8463 | -2.21181 | 0.00005 | 0.00070028 |
| AT1G03950 | VPS2.3, vacuolar protein sorting-associated protein 2.3 | 34.155 | 7.38174 | -2.21006 | 0.00005 | 0.00070028 |
| AT2G40330 | PYL6, RCAR9, PYR1-like 6 | 5.41132 | 1.16989 | -2.20961 | 0.00335 | 0.0175049 |
| AT4G27270 | Quinone reductase family protein | 66.3732 | 14.355 | -2.20905 | 0.00005 | 0.00070028 |
| AT1G32928 | unknown protein | 182.732 | 39.5439 | -2.2082 | 0.00005 | 0.00070028 |
| AT5G03570 | ATIREG2, FPN2, IREG2, iron regulated 2 | 3.00962 | 0.651364 | -2.20804 | 0.0036 | 0.0184414 |
| AT1G20310 | unknown protein | 82.778 | 17.9261 | -2.20718 | 0.00005 | 0.00070028 |
| AT5G21280 | hydroxyproline-rich glycoprotein family protein | 91.1919 | 19.7542 | -2.20675 | 0.00005 | 0.00070028 |
| AT5G59150 | ATRAB-A2D, ATRABA2D, RABA2D, RAB GTPase homolog A2D | 68.0582 | 14.7476 | -2.20629 | 0.00005 | 0.00070028 |
| AT5G46350 | ATWRKY8, WRKY8, WRKY DNA-binding protein 8 | 38.8387 | 8.41764 | -2.20601 | 0.00005 | 0.00070028 |
| AT1G78070 | Transducin/WD40 repeat-like superfamily protein | 255.566 | 55.4027 | -2.20567 | 0.00005 | 0.00070028 |
| AT2G22790 | unknown protein | 26.6624 | 5.7819 | -2.20519 | 0.00015 | 0.00170235 |
| AT4G13130 | Cysteine/Histidine-rich C1 domain family protein | 8.21983 | 1.78453 | -2.20357 | 0.00015 | 0.00170235 |
| AT1G54500 | Rubredoxin-like superfamily protein | 7.98526 | 1.73585 | -2.2017 | 0.0037 | 0.0188077 |
| AT1G49500 | unknown protein | 8.178 | 1.77846 | -2.20112 | 0.0009 | 0.0065867 |
| AT1G19660 | Wound-responsive family protein | 12.8064 | 2.78656 | -2.20031 | 0.00035 | 0.00327416 |
| AT1G50090 | D-aminoacid aminotransferase-like PLP-dependent enzymes superfamily protein | 17.5034 | 3.8121 | -2.19898 | 0.00035 | 0.00327416 |
| AT1G49860 | ATGSTF14, GSTF14, glutathione S-transferase (class phi) 14 | 17.8026 | 3.87911 | -2.19829 | 0.00085 | 0.00630464 |
| AT3G59130 | Cysteine/Histidine-rich C1 domain family protein | 8.86294 | 1.93403 | -2.19618 | 0.0011 | 0.00759303 |
| AT4G26970 | ACO2, aconitase 2 | 501.061 | 109.825 | -2.18977 | 0.00015 | 0.00170235 |
| AT4G28630 | ATATM1, ATM1, ABC transporter of the mitochondrion 1 | 8.19267 | 1.79713 | -2.18864 | 0.0002 | 0.00214445 |
| AT3G14350 | SRF7, STRUBBELIG-receptor family 7 | 76.0462 | 16.6905 | -2.18784 | 0.00005 | 0.00070028 |
| AT5G49890 | ATCLC-C, CLC-C, chloride channel C | 109.347 | 24.0093 | -2.18724 | 0.00005 | 0.00070028 |
| AT1G74930 | ORA47, Integrase-type DNA-binding superfamily protein | 57.6123 | 12.6532 | -2.18687 | 0.00005 | 0.00070028 |
| AT5G15740 | O-fucosyltransferase family protein | 59.7072 | 13.121 | -2.18603 | 0.00005 | 0.00070028 |
| AT3G50830 | ATCOR413-PM2, COR413-PM2, cold-regulated 413-plasma membrane 2 | 116.875 | 25.7024 | -2.18499 | 0.00005 | 0.00070028 |
| AT2G31210 | basic helix-loop-helix (bHLH) DNA-binding superfamily protein | 5.95861 | 1.31186 | -2.18336 | 0.00135 | 0.0089118 |
| AT1G74780 | Nodulin-like / Major Facilitator Superfamily protein | 18.0087 | 3.96505 | -2.18328 | 0.0001 | 0.00123775 |
| AT4G18910 | ATNLM2, NIP1;2, NLM2, NOD26-like intrinsic protein 1;2 | 9.94938 | 2.19134 | -2.18279 | 0.00125 | 0.00840292 |
| AT4G14368 | Regulator of chromosome condensation (RCC1) family protein | 13.4937 | 2.97325 | -2.18217 | 0.0001 | 0.00123775 |
| AT2G47400 | CP12, CP12-1, CP12 domain-containing protein 1 | 12.4594 | 2.74819 | -2.18069 | 0.01095 | 0.0420749 |
| AT3G45070 | P-loop containing nucleoside triphosphate hydrolases superfamily protein | 11.9938 | 2.64868 | -2.17895 | 0.00135 | 0.0089118 |
| AT3G26510 | Octicosapeptide/Phox/Bem1p family protein | 26.4751 | 5.85085 | -2.17792 | 0.0004 | 0.00359419 |
| AT3G16510 | Calcium-dependent lipid-binding (CaLB domain) family protein | 75.4123 | 16.69 | -2.17581 | 0.00005 | 0.00070028 |
| AT5G01720 | RNI-like superfamily protein | 79.5281 | 17.6356 | -2.17298 | 0.00005 | 0.00070028 |
| AT1G02310 | MAN1, Glycosyl hydrolase superfamily protein | 42.4173 | 9.40736 | -2.17279 | 0.0104 | 0.0405756 |
| AT2G15960 | unknown protein | 395.141 | 87.6931 | -2.17183 | 0.0001 | 0.00123775 |
| AT2G40110 | Yippee family putative zinc-binding protein | 71.5415 | 15.8843 | -2.17118 | 0.00005 | 0.00070028 |
| AT4G13395 | DVL10, RTFL12, ROTUNDIFOLIA like 12 | 366.356 | 81.3887 | -2.17035 | 0.00005 | 0.00070028 |
| AT2G42430 | ASL18, LBD16, lateral organ boundaries-domain 16 | 13.3239 | 2.96005 | -2.17032 | 0.00015 | 0.00170235 |
| AT3G04530 | ATPPCK2, PEPCK2, PPCK2, phosphoenolpyruvate carboxylase kinase 2 | 34.1546 | 7.5911 | -2.1697 | 0.0001 | 0.00123775 |
| AT3G25600 | Calcium-binding EF-hand family protein | 149.32 | 33.1992 | -2.16919 | 0.00005 | 0.00070028 |
| AT4G28890 | RING/U-box superfamily protein | 4.41765 | 0.982761 | -2.16837 | 0.00135 | 0.0089118 |
| AT3G51920 | ATCML9, CAM9, CML9, calmodulin 9 | 152.785 | 34.0138 | -2.16731 | 0.00005 | 0.00070028 |
| AT1G01140 | CIPK9, PKS6, SnRK3.12, CBL-interacting protein kinase 9 | 130.675 | 29.1015 | -2.16681 | 0.00005 | 0.00070028 |
| AT1G53920 | GLIP5, GDSL-motif lipase 5 | 192.956 | 43.0331 | -2.16475 | 0.00005 | 0.00070028 |
| AT4G15400 | HXXXD-type acyl-transferase family protein | 24.1973 | 5.41439 | -2.15998 | 0.00005 | 0.00070028 |
| AT4G37990 | ATCAD8, CAD-B2, ELI3, ELI3-2, elicitor-activated gene 3-2 | 46.579 | 10.4301 | -2.15893 | 0.00005 | 0.00070028 |
| AT4G37470 | alpha/beta-Hydrolases superfamily protein | 9.12597 | 2.04398 | -2.1586 | 0.00095 | 0.00685384 |
| AT5G58160 | actin binding | 34.973 | 7.83548 | -2.15815 | 0.00005 | 0.00070028 |
| AT4G18130 | PHYE, phytochrome E | 40.1283 | 9.00403 | -2.15598 | 0.00225 | 0.0130442 |
| AT5G42500 | Disease resistance-responsive (dirigent-like protein) family protein | 6.0099 | 1.35197 | -2.15228 | 0.0059 | 0.0268421 |
| AT5G38850 | Disease resistance protein (TIR-NBS-LRR class) | 12.3823 | 2.78548 | -2.15228 | 0.00005 | 0.00070028 |
| AT5G22540 | Plant protein of unknown function (DUF247) | 2.63577 | 0.593463 | -2.15099 | 0.0121 | 0.0453155 |
| AT3G19000 | 2-oxoglutarate (2OG) and Fe(II)-dependent oxygenase superfamily protein | 3.47281 | 0.782038 | -2.15079 | 0.0091 | 0.0368164 |
| AT1G18150 | ATMPK8, Protein kinase superfamily protein | 134.08 | 30.2339 | -2.14885 | 0.00005 | 0.00070028 |
| AT2G17070 | Arabidopsis protein of unknown function (DUF241) | 10.9019 | 2.45931 | -2.14826 | 0.00105 | 0.00735817 |
| AT3G09540 | Pectin lyase-like superfamily protein | 3.65896 | 0.825736 | -2.14768 | 0.0055 | 0.0254967 |
| AT4G32770 | ATSDX1, VTE1, tocopherol cyclase, chloroplast / vitamin E deficient 1 (VTE1) / sucrose export defective 1 (SXD1) | 5.17269 | 1.16746 | -2.14754 | 0.00125 | 0.00840292 |
| AT4G12450 | unknown protein | 26.7767 | 6.04734 | -2.14661 | 0.00025 | 0.00253767 |
| AT1G73510 | unknown protein | 16.6562 | 3.76284 | -2.14617 | 0.00165 | 0.0103801 |
| AT1G23480 | ATCSLA03, ATCSLA3, CSLA03, CSLA03, CSLA3, cellulose synthase-like A3 | 77.2241 | 17.4475 | -2.14603 | 0.00005 | 0.00070028 |
| AT1G51850 | Leucine-rich repeat protein kinase family protein | 15.024 | 3.40618 | -2.14104 | 0.00035 | 0.00327416 |
| AT1G23870 | ATTPS9, TPS9, TPS9, trehalose-phosphatase/synthase 9 | 356.963 | 80.9865 | -2.14002 | 0.00005 | 0.00070028 |
| AT4G03480 | Ankyrin repeat family protein | 1.44797 | 0.328597 | -2.13964 | 0.008 | 0.0335306 |
| AT2G18690 | unknown protein | 158.694 | 36.0347 | -2.13879 | 0.00005 | 0.00070028 |
| AT2G15555 | other RNA | 12.679 | 2.88111 | -2.13775 | 0.00135 | 0.0089118 |
| AT1G50290 | unknown protein | 117.154 | 26.6541 | -2.13598 | 0.00005 | 0.00070028 |
| AT2G23960 | Class I glutamine amidotransferase-like superfamily protein | 35.0038 | 7.96444 | -2.13587 | 0.0005 | 0.00424432 |
| AT5G51890 | Peroxidase superfamily protein | 11.751 | 2.68105 | -2.13192 | 0.0011 | 0.00759303 |
| AT4G13340 | Leucine-rich repeat (LRR) family protein | 56.7171 | 12.9646 | -2.1292 | 0.00005 | 0.00070028 |
| AT5G03360 | DC1 domain-containing protein | 4.11148 | 0.940874 | -2.12758 | 0.00115 | 0.00787231 |
| AT1G65985 | Plant protein of unknown function (DUF247) | 14.7657 | 3.37933 | -2.12744 | 0.0005 | 0.00424432 |
| AT4G01920 | Cysteine/Histidine-rich C1 domain family protein | 3.67449 | 0.841037 | -2.1273 | 0.00105 | 0.00735817 |
| AT5G01732 | other RNA | 14.6943 | 3.36436 | -2.12686 | 0.0001 | 0.00123775 |
| AT3G49820 | unknown protein | 12.3158 | 2.82297 | -2.12522 | 0.00815 | 0.0339652 |
| AT1G24150 | ATFH4, FH4, formin homologue 4 | 18.0755 | 4.14474 | -2.12468 | 0.00005 | 0.00070028 |
| AT4G27780 | ACBP2, acyl-CoA binding protein 2 | 25.2476 | 5.79329 | -2.12369 | 0.00015 | 0.00170235 |
| AT5G42290 | transcription activator-related | 45.5262 | 10.4498 | -2.12323 | 0.00125 | 0.00840292 |
| AT1G02220 | ANAC003, NAC003, NAC domain containing protein 3 | 5.91975 | 1.35926 | -2.12271 | 0.0017 | 0.0106071 |
| AT4G26120 | Ankyrin repeat family protein / BTB/POZ domain-containing protein | 2.73458 | 0.627948 | -2.1226 | 0.0037 | 0.0188077 |
| AT5G07470 | ATMSRA3, PMSR3, peptidemethionine sulfoxide reductase 3 | 322.06 | 74.0045 | -2.12164 | 0.00005 | 0.00070028 |
| AT5G43330 | Lactate/malate dehydrogenase family protein | 45.5838 | 10.4805 | -2.12081 | 0.00005 | 0.00070028 |
| AT4G37640 | ACA2, calcium ATPase 2 | 161.434 | 37.1259 | -2.12045 | 0.00005 | 0.00070028 |
| AT4G39730 | Lipase/lipooxygenase, PLAT/LH2 family protein | 479.997 | 110.412 | -2.12013 | 0.00005 | 0.00070028 |
| AT1G48240 | ATNPSN12, NPSN12, novel plant snare 12 | 33.5463 | 7.72199 | -2.11911 | 0.00005 | 0.00070028 |
| AT5G19250 | Glycoprotein membrane precursor GPI-anchored | 93.4861 | 21.5756 | -2.11535 | 0.00005 | 0.00070028 |
| AT5G56610 | Phosphotyrosine protein phosphatases superfamily protein | 20.3032 | 4.68714 | -2.11493 | 0.0006 | 0.00487984 |
| AT3G49370 | Calcium-dependent protein kinase (CDPK) family protein | 33.6561 | 7.78148 | -2.11275 | 0.00005 | 0.00070028 |
| AT3G01710 | TPX2 (targeting protein for Xklp2) protein family | 9.91863 | 2.29465 | -2.11187 | 0.00075 | 0.00575475 |
| AT4G35380 | SEC7-like guanine nucleotide exchange family protein | 32.7441 | 7.57528 | -2.11186 | 0.00005 | 0.00070028 |
| AT3G24630 | unknown protein | 3.71161 | 0.859925 | -2.10976 | 0.0016 | 0.0101494 |
| AT4G02650 | ENTH/ANTH/VHS superfamily protein | 10.8851 | 2.52248 | -2.10944 | 0.0007 | 0.00547072 |
| AT4G39160 | Homeodomain-like superfamily protein | 50.7053 | 11.7527 | -2.10914 | 0.00005 | 0.00070028 |
| AT1G09575 | Protein of unknown function (DUF607) | 40.3699 | 9.36591 | -2.10779 | 0.00005 | 0.00070028 |
| AT1G61520 | LHCA3, photosystem I light harvesting complex gene 3 | 1.85903 | 0.431724 | -2.10637 | 0.01375 | 0.0496726 |
| AT3G03990 | alpha/beta-Hydrolases superfamily protein | 357.218 | 82.981 | -2.10595 | 0.00005 | 0.00070028 |
| AT4G00040 | Chalcone and stilbene synthase family protein | 4.61295 | 1.07253 | -2.10467 | 0.0056 | 0.0258574 |
| AT1G76892 | other RNA | 37.7102 | 8.78509 | -2.10183 | 0.00005 | 0.00070028 |
| AT3G14460 | LRR and NB-ARC domains-containing disease resistance protein | 1.97703 | 0.460729 | -2.10134 | 0.00235 | 0.0134663 |
| AT3G21700 | ATSGP2, SGP2, Ras-related small GTP-binding family protein | 153.294 | 35.7869 | -2.0988 | 0.00005 | 0.00070028 |
| AT3G10985 | ATWI-12, SAG20, WI12, senescence associated gene 20 | 688.286 | 160.942 | -2.09646 | 0.00005 | 0.00070028 |
| AT3G49650 | P-loop containing nucleoside triphosphate hydrolases superfamily protein | 11.6355 | 2.72315 | -2.09519 | 0.00005 | 0.00070028 |
| AT2G23770 | protein kinase family protein / peptidoglycan-binding LysM domain-containing protein | 55.6336 | 13.0234 | -2.09485 | 0.00005 | 0.00070028 |
| AT1G54410 | dehydrin family protein | 1393.94 | 327.969 | -2.08754 | 0.00005 | 0.00070028 |
| AT5G44030 | CESA4, IRX5, NWS2, cellulose synthase A4 | 11.2382 | 2.64624 | -2.0864 | 0.0009 | 0.0065867 |
| AT5G38710 | Methylenetetrahydrofolate reductase family protein | 134.102 | 31.5828 | -2.08612 | 0.00005 | 0.00070028 |
| AT3G04060 | anac046, NAC046, NAC domain containing protein 46 | 78.1568 | 18.409 | -2.08596 | 0.00005 | 0.00070028 |
| AT5G05270 | Chalcone-flavanone isomerase family protein | 11.6427 | 2.74408 | -2.08503 | 0.0054 | 0.0251864 |
| AT4G19230 | CYP707A1, cytochrome P450, family 707, subfamily A, polypeptide 1 | 163.144 | 38.5162 | -2.08261 | 0.00005 | 0.00070028 |
| AT1G10370 | ATGSTU17, ERD9, GST30, GST30B, Glutathione S-transferase family protein | 224.731 | 53.1103 | -2.08113 | 0.00005 | 0.00070028 |
| AT5G14310 | AtCXE16, CXE16, carboxyesterase 16 | 66.5391 | 15.7283 | -2.08084 | 0.00005 | 0.00070028 |
| AT3G09580 | FAD/NAD(P)-binding oxidoreductase family protein | 3.2806 | 0.775579 | -2.08061 | 0.00545 | 0.025354 |
| AT2G23120 | Late embryogenesis abundant protein, group 6 | 2371.51 | 560.779 | -2.0803 | 0.00005 | 0.00070028 |
| AT5G02420 | unknown protein | 73.5097 | 17.3867 | -2.07995 | 0.00005 | 0.00070028 |
| AT5G57510 | unknown protein | 258.131 | 61.1094 | -2.07864 | 0.00005 | 0.00070028 |
| AT3G17410 | Protein kinase superfamily protein | 171.128 | 40.5542 | -2.07715 | 0.00005 | 0.00070028 |
| AT5G45490 | P-loop containing nucleoside triphosphate hydrolases superfamily protein | 115.1 | 27.2926 | -2.07631 | 0.00045 | 0.003919 |
| AT1G10360 | ATGSTU18, GST29, GSTU18, glutathione S-transferase TAU 18 | 6.12411 | 1.45348 | -2.07499 | 0.00575 | 0.0263656 |
| AT4G02180 | DC1 domain-containing protein | 1.30317 | 0.309295 | -2.07497 | 0.0075 | 0.0319763 |
| AT1G19910 | ATVHA-C2, AVA-2PE, AVA-P2, ATPase, F0/V0 complex, subunit C protein | 922.291 | 219.083 | -2.07375 | 0.00005 | 0.00070028 |
| AT5G10990 | SAUR-like auxin-responsive protein family | 22.083 | 5.24685 | -2.07342 | 0.00225 | 0.0130442 |
| AT5G01480 | Cysteine/Histidine-rich C1 domain family protein | 2.89672 | 0.688419 | -2.07306 | 0.0067 | 0.0294336 |
| AT5G01750 | Protein of unknown function (DUF567) | 703.089 | 167.121 | -2.07282 | 0.00005 | 0.00070028 |
| AT3G49940 | LBD38, LOB domain-containing protein 38 | 167.855 | 39.9379 | -2.07138 | 0.0001 | 0.00123775 |
| AT5G20250 | DIN10, Raffinose synthase family protein | 446.084 | 106.141 | -2.07133 | 0.0001 | 0.00123775 |
| AT3G11900 | ANT1, aromatic and neutral transporter 1 | 109.997 | 26.1879 | -2.0705 | 0.00005 | 0.00070028 |
| AT5G35460 | unknown protein | 89.058 | 21.2174 | -2.0695 | 0.00005 | 0.00070028 |
| AT4G01070 | GT72B1, UGT72B1, UDP-Glycosyltransferase superfamily protein | 290.56 | 69.3244 | -2.0674 | 0.00005 | 0.00070028 |
| AT5G02350 | Cysteine/Histidine-rich C1 domain family protein | 64.5379 | 15.4004 | -2.06717 | 0.00005 | 0.00070028 |
| AT3G09960 | Calcineurin-like metallo-phosphoesterase superfamily protein | 9.24363 | 2.20792 | -2.06577 | 0.001 | 0.00711095 |
| AT3G09490 | Tetratricopeptide repeat (TPR)-like superfamily protein | 15.5116 | 3.70568 | -2.06554 | 0.00035 | 0.00327416 |
| AT1G19397 | unknown protein | 22.88 | 5.46617 | -2.06548 | 0.00305 | 0.0163293 |
| AT2G32380 | Transmembrane protein 97, predicted | 130.904 | 31.276 | -2.06539 | 0.00005 | 0.00070028 |
| AT3G51180 | Zinc finger C-x8-C-x5-C-x3-H type family protein | 3.51032 | 0.838695 | -2.06538 | 0.00205 | 0.012221 |
| AT2G39050 | hydroxyproline-rich glycoprotein family protein | 231.753 | 55.3765 | -2.06524 | 0.00005 | 0.00070028 |
| AT4G14280 | ARM repeat superfamily protein | 16.2735 | 3.89118 | -2.06424 | 0.00015 | 0.00170235 |
| AT4G29310 | Protein of unknown function (DUF1005) | 23.8666 | 5.72436 | -2.0598 | 0.0002 | 0.00214445 |
| AT5G13080 | ATWRKY75, WRKY75, WRKY DNA-binding protein 75 | 164.884 | 39.5503 | -2.05969 | 0.00005 | 0.00070028 |
| AT3G25882 | NIMIN-2, NIM1-interacting 2 | 85.1208 | 20.4362 | -2.05838 | 0.00015 | 0.00170235 |
| AT1G51620 | Protein kinase superfamily protein | 18.7737 | 4.50864 | -2.05795 | 0.00015 | 0.00170235 |
| AT5G64890 | PROPEP2, elicitor peptide 2 precursor | 9.03668 | 2.17201 | -2.05676 | 0.0068 | 0.029741 |
| AT1G05700 | Leucine-rich repeat transmembrane protein kinase protein | 3.93789 | 0.946686 | -2.05647 | 0.00245 | 0.0139065 |
| AT2G28160 | ATBHLH029, ATBHLH29, ATFIT1, BHLH029, FIT1, FRU, FER-like regulator of iron uptake | 18.8058 | 4.52206 | -2.05612 | 0.00055 | 0.00457655 |
| AT1G80380 | P-loop containing nucleoside triphosphate hydrolases superfamily protein | 422.385 | 101.617 | -2.05542 | 0.00005 | 0.00070028 |
| AT1G23140 | Calcium-dependent lipid-binding (CaLB domain) family protein | 46.8932 | 11.2883 | -2.05455 | 0.00055 | 0.00457655 |
| AT2G20230 | Tetraspanin family protein | 143.986 | 34.7292 | -2.05171 | 0.00005 | 0.00070028 |
| AT4G12090 | Cornichon family protein | 124.764 | 30.0976 | -2.05148 | 0.00095 | 0.00685384 |
| AT4G18780 | ATCESA8, CESA8, IRX1, LEW2, cellulose synthase family protein | 3.73202 | 0.900624 | -2.05096 | 0.00085 | 0.00630464 |
| AT3G27470 | Protein of unknown function (DUF707) | 16.3202 | 3.94036 | -2.05026 | 0.0001 | 0.00123775 |
| AT3G56230 | BTB/POZ domain-containing protein | 44.4348 | 10.7348 | -2.0494 | 0.00005 | 0.00070028 |
| AT4G14716 | ARD1, ATARD1, acireductone dioxygenase 1 | 29.0369 | 7.0156 | -2.04925 | 0.0006 | 0.00487984 |
| AT1G15100 | RHA2A, RING-H2 finger A2A | 658.001 | 159.186 | -2.04738 | 0.00005 | 0.00070028 |
| AT2G46140 | Late embryogenesis abundant protein | 747.755 | 180.962 | -2.04687 | 0.00005 | 0.00070028 |
| AT2G38290 | AMT2, AMT2;1, ATAMT2, ammonium transporter 2 | 72.3125 | 17.5239 | -2.04492 | 0.00005 | 0.00070028 |
| AT1G60010 | unknown protein | 54.7641 | 13.2725 | -2.0448 | 0.00005 | 0.00070028 |
| AT3G11600 | unknown protein | 10.6881 | 2.5913 | -2.04426 | 0.0023 | 0.0132526 |
| AT4G27657 | unknown protein | 23.5732 | 5.72081 | -2.04286 | 0.0023 | 0.0132526 |
| AT3G53490 | unknown protein | 37.5854 | 9.12581 | -2.04215 | 0.0003 | 0.00292382 |
| AT4G15070 | Cysteine/Histidine-rich C1 domain family protein | 3.90052 | 0.950654 | -2.03667 | 0.0022 | 0.0128446 |
| AT4G14640 | CAM8, calmodulin 8 | 58.0532 | 14.1588 | -2.03568 | 0.0004 | 0.00359419 |
| AT3G01290 | SPFH/Band 7/PHB domain-containing membrane-associated protein family | 354.701 | 86.6315 | -2.03364 | 0.00005 | 0.00070028 |
| AT1G31120 | KUP10, K+ uptake permease 10 | 27.419 | 6.69974 | -2.033 | 0.00005 | 0.00070028 |
| AT3G61760 | ADL1B, DL1B, DYNAMIN-like 1B | 34.0428 | 8.31952 | -2.03278 | 0.00005 | 0.00070028 |
| AT3G02620 | Plant stearoyl-acyl-carrier-protein desaturase family protein | 3.65249 | 0.892621 | -2.03276 | 0.0074 | 0.0317207 |
| AT2G40765 | unknown protein | 400.871 | 98.0433 | -2.03165 | 0.00825 | 0.0342522 |
| AT2G16720 | ATMYB7, ATY49, MYB7, myb domain protein 7 | 95.0576 | 23.2494 | -2.03161 | 0.00005 | 0.00070028 |
| AT4G15800 | RALFL33, ralf-like 33 | 231.158 | 56.5528 | -2.03121 | 0.00005 | 0.00070028 |
| AT4G26710 | ATPase, V0 complex, subunit E | 528.377 | 129.273 | -2.03115 | 0.00005 | 0.00070028 |
| AT3G04730 | IAA16, indoleacetic acid-induced protein 16 | 500.06 | 122.369 | -2.03086 | 0.00005 | 0.00070028 |
| AT1G20840 | TMT1, tonoplast monosaccharide transporter1 | 114.533 | 28.0419 | -2.0301 | 0.00005 | 0.00070028 |
| AT1G20390 | transposable element gene | 3.7622 | 0.92127 | -2.02988 | 0.00005 | 0.00070028 |
| AT1G14520 | MIOX1, myo-inositol oxygenase 1 | 3.21246 | 0.78678 | -2.02965 | 0.00925 | 0.0371953 |
| AT1G63220 | Calcium-dependent lipid-binding (CaLB domain) family protein | 408.652 | 100.17 | -2.02843 | 0.00005 | 0.00070028 |
| AT1G70640 | octicosapeptide/Phox/Bem1p (PB1) domain-containing protein | 6.42372 | 1.57584 | -2.02729 | 0.00935 | 0.037453 |
| AT3G05580 | Calcineurin-like metallo-phosphoesterase superfamily protein | 195.42 | 47.9499 | -2.02698 | 0.00005 | 0.00070028 |
| AT4G13000 | AGC (cAMP-dependent, cGMP-dependent and protein kinase C) kinase family protein | 2.27344 | 0.557861 | -2.0269 | 0.0102 | 0.0400002 |
| AT1G10350 | DNAJ heat shock family protein | 33.7586 | 8.30636 | -2.02297 | 0.0001 | 0.00123775 |
| AT1G09070 | (AT)SRC2, SRC2, soybean gene regulated by cold-2 | 1357.32 | 334.265 | -2.0217 | 0.0001 | 0.00123775 |
| AT4G14430 | ATECI2, ECHIB, ECI2, IBR10, PEC12, indole-3-butyric acid response 10 | 65.1954 | 16.0831 | -2.01923 | 0.00005 | 0.00070028 |
| AT5G67290 | FAD-dependent oxidoreductase family protein | 40.1255 | 9.92739 | -2.01503 | 0.00005 | 0.00070028 |
| AT3G57640 | Protein kinase superfamily protein | 2.67527 | 0.662157 | -2.01444 | 0.0106 | 0.0411532 |
| AT5G40270 | HD domain-containing metal-dependent phosphohydrolase family protein | 9.01667 | 2.23347 | -2.01331 | 0.0005 | 0.00424432 |
| AT2G38380 | Peroxidase superfamily protein | 2.87893 | 0.714075 | -2.01138 | 0.01065 | 0.0412906 |
| AT1G04160 | ATXIB, XI-8, XI-B, XIB, myosin XI B | 50.5496 | 12.575 | -2.00714 | 0.00005 | 0.00070028 |
| AT1G04040 | HAD superfamily, subfamily IIIB acid phosphatase | 686.269 | 170.873 | -2.00585 | 0.00025 | 0.00253767 |
| AT1G62370 | RING/U-box superfamily protein | 70.2079 | 17.4973 | -2.0045 | 0.00035 | 0.00327416 |
| AT4G19170 | CCD4, NCED4, nine-cis-epoxycarotenoid dioxygenase 4 | 1.15204 | 0.28724 | -2.00386 | 0.01085 | 0.0417717 |
| AT5G11090 | serine-rich protein-related | 110.826 | 27.648 | -2.00305 | 0.00005 | 0.00070028 |
| AT1G75820 | ATCLV1, CLV1, FAS3, FLO5, Leucine-rich receptor-like protein kinase family protein | 1.58151 | 0.394542 | -2.00305 | 0.00235 | 0.0134663 |
| AT4G01360 | unknown protein | 66.9772 | 16.7422 | -2.00018 | 0.0001 | 0.00123775 |
| AT3G18780 | ACT2, DER1, ENL2, LSR2, actin 2 | 1579.69 | 395.117 | -1.99929 | 0.0001 | 0.00123775 |
| AT3G51460 | RHD4, Phosphoinositide phosphatase family protein | 210.894 | 52.7531 | -1.99919 | 0.00005 | 0.00070028 |
| AT5G02790 | GSTL3, Glutathione S-transferase family protein | 3.75013 | 0.938682 | -1.99823 | 0.0111 | 0.0425192 |
| AT5G66280 | GMD1, GDP-D-mannose 4,6-dehydratase 1 | 109.064 | 27.3395 | -1.99612 | 0.00005 | 0.00070028 |
| AT1G13245 | DVL4, RTFL17, ROTUNDIFOLIA like 17 | 127.33 | 31.9512 | -1.99463 | 0.00005 | 0.00070028 |
| AT3G53950 | glyoxal oxidase-related protein | 3.74011 | 0.939628 | -1.99292 | 0.00775 | 0.0327601 |
| AT3G17420 | GPK1, glyoxysomal protein kinase 1 | 115.141 | 28.969 | -1.99082 | 0.00005 | 0.00070028 |
| AT5G15240 | Transmembrane amino acid transporter family protein | 1.79825 | 0.453392 | -1.98776 | 0.0092 | 0.0370545 |
| AT3G22850 | Aluminium induced protein with YGL and LRDR motifs | 288.673 | 72.8211 | -1.98701 | 0.00005 | 0.00070028 |
| AT1G61290 | ATSYP124, SYP124, syntaxin of plants 124 | 13.1794 | 3.3257 | -1.98655 | 0.0008 | 0.00603091 |
| AT2G46620 | P-loop containing nucleoside triphosphate hydrolases superfamily protein | 37.7178 | 9.56485 | -1.97943 | 0.00005 | 0.00070028 |
| AT4G39080 | VHA-A3, vacuolar proton ATPase A3 | 597.978 | 151.661 | -1.97924 | 0.0002 | 0.00214445 |
| AT5G25820 | Exostosin family protein | 32.8516 | 8.34222 | -1.97746 | 0.0001 | 0.00123775 |
| AT4G33920 | Protein phosphatase 2C family protein | 274.739 | 69.901 | -1.97468 | 0.00005 | 0.00070028 |
| AT4G34720 | ATVHA-C1, AVA-P1, VHA-C1, ATPase, F0/V0 complex, subunit C protein | 758.112 | 192.907 | -1.97451 | 0.00005 | 0.00070028 |
| AT3G20860 | ATNEK5, NEK5, NIMA-related kinase 5 | 11.7719 | 2.99603 | -1.97422 | 0.0008 | 0.00603091 |
| AT5G24870 | RING/U-box superfamily protein | 70.8318 | 18.0283 | -1.97413 | 0.00005 | 0.00070028 |
| AT1G15490 | alpha/beta-Hydrolases superfamily protein | 15.5963 | 3.97284 | -1.97296 | 0.00035 | 0.00327416 |
| AT3G02140 | AFP4, TMAC2, AFP2 (ABI five-binding protein 2) family protein | 269.651 | 68.7033 | -1.97264 | 0.00005 | 0.00070028 |
| AT1G65180 | Cysteine/Histidine-rich C1 domain family protein | 9.01323 | 2.29993 | -1.97045 | 0.0006 | 0.00487984 |
| AT1G51800 | Leucine-rich repeat protein kinase family protein | 19.0238 | 4.8626 | -1.968 | 0.00005 | 0.00070028 |
| AT1G32260 | unknown protein | 28.7455 | 7.35315 | -1.9669 | 0.00025 | 0.00253767 |
| AT1G56220 | Dormancy/auxin associated family protein | 141.148 | 36.1249 | -1.96614 | 0.00005 | 0.00070028 |
| AT1G74740 | ATCPK30, CDPK1A, CPK30, calcium-dependent protein kinase 30 | 22.694 | 5.80925 | -1.96589 | 0.00005 | 0.00070028 |
| AT4G17550 | Major facilitator superfamily protein | 10.2507 | 2.62519 | -1.96523 | 0.00295 | 0.0159014 |
| AT5G13330 | Rap2.6L, related to AP2 6l | 150.29 | 38.518 | -1.96415 | 0.00005 | 0.00070028 |
| AT3G09375 | pseudogene, putative eukaryotic translation initiation factor 4A-3 (eIF-4A-3/eIF4A-3), strong similarity to SP|P41380|IF43_NICPL Eukaryotic initiation factor 4A-3 (eIF-4A-3) (eIF4A-3) {Nicotiana plumbaginifolia}; blastp match of 84% identity and 4.1e-163 P-value to GP|13957631|gb|AAK50586.1|AC084404_11|AC084404 putative translation initiation factor {Oryza sativa} | 17.32 | 4.43946 | -1.96398 | 0.0004 | 0.00359419 |
| AT3G20310 | ATERF-7, ATERF7, ERF7, ethylene response factor 7 | 84.9658 | 21.787 | -1.96342 | 0.00005 | 0.00070028 |
| AT3G57040 | ARR9, ATRR4, response regulator 9 | 57.6293 | 14.7806 | -1.9631 | 0.00005 | 0.00070028 |
| AT1G80180 | unknown protein | 24.7397 | 6.35601 | -1.96064 | 0.00175 | 0.0108609 |
| AT3G51160 | GMD2, MUR1, MUR_1, NAD(P)-binding Rossmann-fold superfamily protein | 194.458 | 49.9687 | -1.96036 | 0.00005 | 0.00070028 |
| AT4G27654 | unknown protein | 50.0616 | 12.866 | -1.96014 | 0.0021 | 0.0124516 |
| AT5G11230 | Nucleotide-sugar transporter family protein | 268.903 | 69.1341 | -1.95962 | 0.0001 | 0.00123775 |
| AT1G20090 | ARAC4, ATRAC4, ATROP2, ROP2, RHO-related protein from plants 2 | 120.63 | 31.0274 | -1.95897 | 0.00005 | 0.00070028 |
| AT1G09740 | Adenine nucleotide alpha hydrolases-like superfamily protein | 256.845 | 66.0785 | -1.95865 | 0.00005 | 0.00070028 |
| AT3G05936 | unknown protein | 15.0029 | 3.86097 | -1.95821 | 0.00305 | 0.0163293 |
| AT3G15670 | Late embryogenesis abundant protein (LEA) family protein | 692.424 | 178.453 | -1.95611 | 0.00005 | 0.00070028 |
| AT4G34920 | PLC-like phosphodiesterases superfamily protein | 6.40281 | 1.65538 | -1.95154 | 0.0034 | 0.01771 |
| AT3G11800 | unknown protein | 171.859 | 44.4368 | -1.9514 | 0.00005 | 0.00070028 |
| AT1G75220 | Major facilitator superfamily protein | 456.642 | 118.127 | -1.95072 | 0.00005 | 0.00070028 |
| AT3G17440 | ATNPSN13, NPSN13, novel plant snare 13 | 132.224 | 34.2436 | -1.94908 | 0.00005 | 0.00070028 |
| AT1G73805 | Calmodulin binding protein-like | 8.69024 | 2.25135 | -1.9486 | 0.00095 | 0.00685384 |
| AT4G12330 | CYP706A7, cytochrome P450, family 706, subfamily A, polypeptide 7 | 23.3883 | 6.06555 | -1.94708 | 0.0001 | 0.00123775 |
| AT4G17670 | Protein of unknown function (DUF581) | 17.4655 | 4.53365 | -1.94577 | 0.00075 | 0.00575475 |
| AT2G41110 | ATCAL5, CAM2, calmodulin 2 | 513.368 | 133.265 | -1.94569 | 0.00005 | 0.00070028 |
| AT2G24545 | other RNA | 12.1118 | 3.14438 | -1.94556 | 0.00105 | 0.00735817 |
| AT3G01430 | BEST Arabidopsis thaliana protein match is: NHL domain-containing protein (TAIR:AT5G14890.1) | 6.79385 | 1.764 | -1.94538 | 0.00965 | 0.0383598 |
| AT5G15420 | unknown protein | 24.0171 | 6.23667 | -1.94522 | 0.0018 | 0.0110912 |
| AT1G08770 | PRA1.E, prenylated RAB acceptor 1.E | 33.5794 | 8.72335 | -1.94462 | 0.00025 | 0.00253767 |
| AT1G08880 | G-H2AX, GAMMA-H2AX, H2AXA, HTA5, Histone superfamily protein | 267.518 | 69.502 | -1.94451 | 0.0001 | 0.00123775 |
| AT5G65310 | ATHB-5, ATHB5, HB5, homeobox protein 5 | 22.4398 | 5.8317 | -1.94407 | 0.00025 | 0.00253767 |
| AT1G74640 | alpha/beta-Hydrolases superfamily protein | 33.3475 | 8.66905 | -1.94363 | 0.00015 | 0.00170235 |
| AT3G45095 | transposable element gene | 3.31498 | 0.86187 | -1.94346 | 0.0009 | 0.0065867 |
| AT5G38790 | unknown protein | 25.1907 | 6.55392 | -1.94246 | 0.01205 | 0.0451624 |
| AT1G72880 | Survival protein SurE-like phosphatase/nucleotidase | 83.742 | 21.7963 | -1.94187 | 0.00005 | 0.00070028 |
| AT5G60020 | ATLAC17, LAC17, laccase 17 | 3.08018 | 0.801737 | -1.94181 | 0.00755 | 0.0321272 |
| AT1G64820 | MATE efflux family protein | 2.49566 | 0.650383 | -1.94006 | 0.0113 | 0.0430687 |
| AT5G24910 | CYP714A1, cytochrome P450, family 714, subfamily A, polypeptide 1 | 3.46155 | 0.902335 | -1.93968 | 0.00355 | 0.0182751 |
| AT1G10170 | ATNFXL1, NFXL1, NF-X-like 1 | 537.884 | 140.222 | -1.93958 | 0.0002 | 0.00214445 |
| AT5G15210 | ATHB30, HB30, ZFHD3, homeobox protein 30 | 3.2252 | 0.841463 | -1.93842 | 0.0085 | 0.0349894 |
| AT2G35610 | XEG113, xyloglucanase 113 | 128.058 | 33.415 | -1.93822 | 0.00005 | 0.00070028 |
| AT1G20030 | Pathogenesis-related thaumatin superfamily protein | 10.5941 | 2.76791 | -1.9364 | 0.00215 | 0.0126571 |
| AT2G21840 | Cysteine/Histidine-rich C1 domain family protein | 27.2894 | 7.1375 | -1.93485 | 0.00015 | 0.00170235 |
| AT1G32460 | unknown protein | 223.483 | 58.4616 | -1.93461 | 0.00005 | 0.00070028 |
| AT5G15340 | Pentatricopeptide repeat (PPR) superfamily protein | 4.98064 | 1.30315 | -1.93432 | 0.00355 | 0.0182751 |
| AT4G14520 | DNA-directed RNA polymerase II-related | 23.1685 | 6.06817 | -1.93283 | 0.0011 | 0.00759303 |
| AT5G60320 | Concanavalin A-like lectin protein kinase family protein | 3.99412 | 1.04658 | -1.9322 | 0.0033 | 0.0173075 |
| AT5G16370 | AAE5, acyl activating enzyme 5 | 53.1633 | 13.9336 | -1.93186 | 0.00005 | 0.00070028 |
| AT3G19250 | Protein of unknown function (DUF677) | 5.74596 | 1.50749 | -1.9304 | 0.0035 | 0.0180741 |
| AT4G22780 | ACR7, ACT domain repeat 7 | 61.6287 | 16.1688 | -1.93039 | 0.00005 | 0.00070028 |
| AT2G41410 | Calcium-binding EF-hand family protein | 317.713 | 83.4683 | -1.92842 | 0.00005 | 0.00070028 |
| AT5G01700 | Protein phosphatase 2C family protein | 14.6044 | 3.84432 | -1.9256 | 0.00095 | 0.00685384 |
| AT2G37040 | ATPAL1, PAL1, PHE ammonia lyase 1 | 132.303 | 34.8381 | -1.92511 | 0.00005 | 0.00070028 |
| AT3G02880 | Leucine-rich repeat protein kinase family protein | 255.226 | 67.3298 | -1.92246 | 0.00005 | 0.00070028 |
| AT2G23100 | Cysteine/Histidine-rich C1 domain family protein | 5.16973 | 1.36594 | -1.9202 | 0.00135 | 0.0089118 |
| AT3G27210 | unknown protein | 41.4602 | 10.9693 | -1.91826 | 0.0003 | 0.00292382 |
| AT1G79340 | AtMC4, MC4, metacaspase 4 | 462.406 | 122.438 | -1.91711 | 0.00065 | 0.00516972 |
| AT4G14500 | Polyketide cyclase/dehydrase and lipid transport superfamily protein | 184.224 | 48.7814 | -1.91706 | 0.00005 | 0.00070028 |
| AT5G62560 | RING/U-box superfamily protein with ARM repeat domain | 38.3476 | 10.1543 | -1.91704 | 0.0001 | 0.00123775 |
| AT2G19160 | Core-2/I-branching beta-1,6-N-acetylglucosaminyltransferase family protein | 80.7333 | 21.3898 | -1.91624 | 0.0001 | 0.00123775 |
| AT3G10010 | DML2, demeter-like 2 | 1.23067 | 0.326241 | -1.91543 | 0.0026 | 0.0145003 |
| AT5G11950 | Putative lysine decarboxylase family protein | 85.2588 | 22.6206 | -1.91421 | 0.00005 | 0.00070028 |
| AT3G54420 | ATCHITIV, ATEP3, CHIV, EP3, homolog of carrot EP3-3 chitinase | 5.32299 | 1.41238 | -1.91411 | 0.0081 | 0.0338066 |
| AT5G43040 | Cysteine/Histidine-rich C1 domain family protein | 6.12025 | 1.62447 | -1.91362 | 0.00385 | 0.0194256 |
| AT3G62260 | Protein phosphatase 2C family protein | 216.198 | 57.4319 | -1.91243 | 0.00005 | 0.00070028 |
| AT4G18425 | Protein of unknown function (DUF679) | 28.2426 | 7.51435 | -1.91015 | 0.00165 | 0.0103801 |
| AT2G18620 | Terpenoid synthases superfamily protein | 4.71811 | 1.25625 | -1.90908 | 0.00775 | 0.0327601 |
| AT2G25530 | AFG1-like ATPase family protein | 10.3091 | 2.74497 | -1.90905 | 0.00025 | 0.00253767 |
| AT4G27450 | Aluminium induced protein with YGL and LRDR motifs | 261.962 | 69.8234 | -1.90757 | 0.00005 | 0.00070028 |
| AT3G16910 | AAE7, ACN1, acyl-activating enzyme 7 | 89.2772 | 23.8106 | -1.90669 | 0.00005 | 0.00070028 |
| AT3G09810 | IDH-VI, isocitrate dehydrogenase VI | 242.394 | 64.7445 | -1.90453 | 0.00005 | 0.00070028 |
| AT3G07480 | 2Fe-2S ferredoxin-like superfamily protein | 421.963 | 112.714 | -1.90445 | 0.00005 | 0.00070028 |
| AT5G38700 | unknown protein | 98.1841 | 26.2368 | -1.9039 | 0.00005 | 0.00070028 |
| AT5G67190 | DEAR2, DREB and EAR motif protein 2 | 96.7601 | 25.8656 | -1.90338 | 0.0002 | 0.00214445 |
| AT1G30455 | transcription regulators;translation initiation factors;zinc ion binding;transcription activators | 6.74576 | 1.8058 | -1.90135 | 0.0109 | 0.0419234 |
| AT2G47950 | unknown protein | 278.231 | 74.4824 | -1.90131 | 0.00005 | 0.00070028 |
| AT2G45330 | emb1067, RNA 2'-phosphotransferase, Tpt1 / KptA family | 59.914 | 16.066 | -1.89888 | 0.00035 | 0.00327416 |
| AT1G19440 | KCS4, 3-ketoacyl-CoA synthase 4 | 52.0617 | 13.9648 | -1.89843 | 0.00005 | 0.00070028 |
| AT5G45630 | Protein of unknown function, DUF584 | 145.646 | 39.0753 | -1.89814 | 0.00035 | 0.00327416 |
| AT4G25470 | ATCBF2, CBF2, DREB1C, FTQ4, C-repeat/DRE binding factor 2 | 6.95951 | 1.86786 | -1.8976 | 0.0103 | 0.0403045 |
| AT3G49120 | ATPCB, ATPERX34, PERX34, PRX34, PRXCB, peroxidase CB | 7.84592 | 2.10598 | -1.89745 | 0.0033 | 0.0173075 |
| AT3G56760 | Protein kinase superfamily protein | 54.6139 | 14.6612 | -1.89727 | 0.00005 | 0.00070028 |
| AT1G49032 | unknown protein | 37.1449 | 9.9723 | -1.89717 | 0.00065 | 0.00516972 |
| AT4G15500 | UGT84A4, UDP-Glycosyltransferase superfamily protein | 5.306 | 1.42474 | -1.89692 | 0.00285 | 0.0154761 |
| AT1G76990 | ACR3, ACT domain repeat 3 | 194.67 | 52.2885 | -1.89646 | 0.00005 | 0.00070028 |
| AT5G43400 | Uncharacterised conserved protein UCP015417, vWA | 32.7917 | 8.81557 | -1.8952 | 0.0001 | 0.00123775 |
| AT5G10430 | AGP4, ATAGP4, arabinogalactan protein 4 | 198.707 | 53.4325 | -1.89485 | 0.0001 | 0.00123775 |
| AT4G19045 | Mob1/phocein family protein | 54.0514 | 14.5491 | -1.8934 | 0.00015 | 0.00170235 |
| AT1G70230 | TBL27, TRICHOME BIREFRINGENCE-LIKE 27 | 8.08509 | 2.17674 | -1.8931 | 0.00135 | 0.0089118 |
| AT1G05640 | Ankyrin repeat family protein | 7.46793 | 2.01089 | -1.89287 | 0.00145 | 0.00940576 |
| AT5G18860 | inosine-uridine preferring nucleoside hydrolase family protein | 101.121 | 27.2386 | -1.89236 | 0.00005 | 0.00070028 |
| AT5G67160 | EPS1, HXXXD-type acyl-transferase family protein | 9.35251 | 2.52002 | -1.89192 | 0.00125 | 0.00840292 |
| AT5G47040 | LON2, lon protease 2 | 203.331 | 54.7876 | -1.89191 | 0.00005 | 0.00070028 |
| AT1G07610 | MT1C, metallothionein 1C | 104.157 | 28.0747 | -1.89142 | 0.00075 | 0.00575475 |
| AT1G18300 | atnudt4, NUDT4, nudix hydrolase homolog 4 | 42.3746 | 11.423 | -1.89126 | 0.00015 | 0.00170235 |
| AT5G40210 | nodulin MtN21 /EamA-like transporter family protein | 31.3616 | 8.469 | -1.88874 | 0.0003 | 0.00292382 |
| AT2G34585 | unknown protein | 36.9671 | 9.98873 | -1.88787 | 0.00065 | 0.00516972 |
| AT3G61280 | Arabidopsis thaliana protein of unknown function (DUF821) | 1.91361 | 0.51783 | -1.88574 | 0.0122 | 0.0455694 |
| AT1G54450 | Calcium-binding EF-hand family protein | 22.2222 | 6.01481 | -1.88541 | 0.00045 | 0.003919 |
| AT3G60450 | Phosphoglycerate mutase family protein | 643.847 | 174.274 | -1.88536 | 0.00005 | 0.00070028 |
| AT5G67300 | ATMYB44, ATMYBR1, MYB44, MYBR1, myb domain protein r1 | 195.702 | 53.0075 | -1.88439 | 0.00005 | 0.00070028 |
| AT1G71780 | unknown protein | 110.731 | 29.9951 | -1.88427 | 0.00005 | 0.00070028 |
| AT4G12070 | unknown protein | 73.5546 | 19.9464 | -1.88269 | 0.00005 | 0.00070028 |
| AT5G64620 | ATC/VIF2, C/VIF2, cell wall / vacuolar inhibitor of fructosidase 2 | 31.8113 | 8.6449 | -1.87962 | 0.00125 | 0.00840292 |
| AT1G05340 | unknown protein | 838.556 | 228.011 | -1.8788 | 0.00005 | 0.00070028 |
| AT5G06930 | LOCATED IN: chloroplast; EXPRESSED IN: 15 plant structures; EXPRESSED DURING: 7 growth stages; BEST Arabidopsis thaliana protein match is: nucleolar protein gar2-related (TAIR:AT2G42320.2); Has 3369 Blast hits to 1526 proteins in 313 species: Archae - 2; Bacteria - 910; Metazoa - 754; Fungi - 336; Plants - 137; Viruses - 11; Other Eukaryotes - 1219 (source: NCBI BLink). | 33.9366 | 9.23135 | -1.87823 | 0.00005 | 0.00070028 |
| AT5G42380 | CML37, CML39, calmodulin like 37 | 244.732 | 66.6011 | -1.87758 | 0.00005 | 0.00070028 |
| AT2G25150 | HXXXD-type acyl-transferase family protein | 29.7066 | 8.08563 | -1.87735 | 0.00005 | 0.00070028 |
| AT4G23730 | Galactose mutarotase-like superfamily protein | 114.307 | 31.1309 | -1.87649 | 0.0001 | 0.00123775 |
| AT3G01990 | ACR6, ACT domain repeat 6 | 4.73717 | 1.29036 | -1.87626 | 0.00415 | 0.020593 |
| AT2G46030 | UBC6, ubiquitin-conjugating enzyme 6 | 178.653 | 48.6747 | -1.87591 | 0.00005 | 0.00070028 |
| AT3G29410 | Terpenoid cyclases/Protein prenyltransferases superfamily protein | 86.9658 | 23.6998 | -1.87557 | 0.00005 | 0.00070028 |
| AT5G01760 | ENTH/VHS/GAT family protein | 61.6202 | 16.7934 | -1.87551 | 0.00005 | 0.00070028 |
| AT4G37830 | cytochrome c oxidase-related | 729.118 | 198.929 | -1.8739 | 0.0001 | 0.00123775 |
| AT5G47240 | atnudt8, NUDT8, nudix hydrolase homolog 8 | 54.7061 | 14.9332 | -1.87318 | 0.0001 | 0.00123775 |
| AT3G10120 | unknown protein | 5.7269 | 1.56557 | -1.87107 | 0.0091 | 0.0368164 |
| AT3G03341 | unknown protein | 268.034 | 73.3189 | -1.87016 | 0.0002 | 0.00214445 |
| AT3G24100 | Uncharacterised protein family SERF | 106.541 | 29.181 | -1.86831 | 0.0036 | 0.0184414 |
| AT3G28850 | Glutaredoxin family protein | 15.7242 | 4.30781 | -1.86796 | 0.00045 | 0.003919 |
| AT5G67350 | unknown protein | 56.6379 | 15.5192 | -1.86771 | 0.00005 | 0.00070028 |
| AT4G26200 | ACS7, ATACS7, 1-amino-cyclopropane-1-carboxylate synthase 7 | 15.5717 | 4.27107 | -1.86626 | 0.00035 | 0.00327416 |
| AT4G30160 | ATVLN4, VLN4, villin 4 | 94.7152 | 26.0059 | -1.86475 | 0.00005 | 0.00070028 |
| AT3G48590 | ATHAP5A, HAP5A, NF-YC1, nuclear factor Y, subunit C1 | 11.9801 | 3.28945 | -1.86471 | 0.0036 | 0.0184414 |
| AT2G47520 | HRE2, Integrase-type DNA-binding superfamily protein | 17.249 | 4.73635 | -1.86466 | 0.00805 | 0.0336689 |
| AT5G35180 | Protein of unknown function (DUF1336) | 95.7102 | 26.293 | -1.86399 | 0.0001 | 0.00123775 |
| AT3G13720 | PRA1.F3, PRA8, PRA1 (Prenylated rab acceptor) family protein | 79.445 | 21.8349 | -1.86332 | 0.00005 | 0.00070028 |
| AT3G53420 | PIP2, PIP2;1, PIP2A, plasma membrane intrinsic protein 2A | 598 | 164.361 | -1.86328 | 0.00005 | 0.00070028 |
| AT5G63130 | Octicosapeptide/Phox/Bem1p family protein | 101.391 | 27.9117 | -1.86099 | 0.00005 | 0.00070028 |
| AT3G29575 | AFP3, ABI five binding protein 3 | 189.405 | 52.182 | -1.85985 | 0.0002 | 0.00214445 |
| AT3G54020 | AtIPCS1, Arabidopsis Inositol phosphorylceramide synthase 1 | 105.698 | 29.158 | -1.85799 | 0.0001 | 0.00123775 |
| AT1G77120 | ADH, ADH1, ATADH, ATADH1, alcohol dehydrogenase 1 | 594.086 | 164.008 | -1.85691 | 0.00035 | 0.00327416 |
| AT1G70330 | ENT1, ENT1,AT, equilibrative nucleotide transporter 1 | 92.4346 | 25.5205 | -1.85678 | 0.00065 | 0.00516972 |
| AT2G44600 | unknown protein | 8.71181 | 2.40821 | -1.85501 | 0.00105 | 0.00735817 |
| AT5G06760 | LEA4-5, Late Embryogenesis Abundant 4-5 | 464.108 | 128.389 | -1.85394 | 0.00005 | 0.00070028 |
| AT2G33510 | CONTAINS InterPro DOMAIN/s: WW/Rsp5/WWP (InterPro:IPR001202) | 37.9458 | 10.5024 | -1.85322 | 0.00045 | 0.003919 |
| AT1G14220 | Ribonuclease T2 family protein | 109.629 | 30.3451 | -1.8531 | 0.0002 | 0.00214445 |
| AT3G07360 | ATPUB9, PUB9, plant U-box 9 | 60.0916 | 16.6531 | -1.85137 | 0.00005 | 0.00070028 |
| AT5G11670 | ATNADP-ME2, NADP-ME2, NADP-malic enzyme 2 | 867.564 | 240.675 | -1.84989 | 0.00105 | 0.00735817 |
| AT2G15970 | ATCOR413-PM1, cold regulated 413 plasma membrane 1 | 1992.6 | 553.066 | -1.84913 | 0.00065 | 0.00516972 |
| AT1G20120 | GDSL-like Lipase/Acylhydrolase superfamily protein | 11.5468 | 3.20837 | -1.84759 | 0.0017 | 0.0106071 |
| AT1G19700 | BEL10, BLH10, BEL1-like homeodomain 10 | 38.6821 | 10.7547 | -1.84669 | 0.00025 | 0.00253767 |
| AT1G22750 | unknown protein | 35.1327 | 9.76815 | -1.84666 | 0.00075 | 0.00575475 |
| AT5G37750 | Chaperone DnaJ-domain superfamily protein | 18.5309 | 5.15544 | -1.84576 | 0.00325 | 0.0171315 |
| AT4G39360 | unknown protein | 39.434 | 10.9713 | -1.84571 | 0.0005 | 0.00424432 |
| AT5G45350 | proline-rich family protein | 424.782 | 118.27 | -1.84464 | 0.00005 | 0.00070028 |
| AT5G58320 | Kinase interacting (KIP1-like) family protein | 235.739 | 65.6598 | -1.84411 | 0.00015 | 0.00170235 |
| AT2G42490 | Copper amine oxidase family protein | 111.936 | 31.1836 | -1.84382 | 0.00005 | 0.00070028 |
| AT3G02470 | SAMDC, S-adenosylmethionine decarboxylase | 1018.14 | 283.691 | -1.84354 | 0.0113 | 0.0430687 |
| AT1G79870 | D-isomer specific 2-hydroxyacid dehydrogenase family protein | 34.2182 | 9.53903 | -1.84285 | 0.0002 | 0.00214445 |
| AT2G36320 | A20/AN1-like zinc finger family protein | 274.787 | 76.6466 | -1.84202 | 0.00005 | 0.00070028 |
| AT5G59220 | HAI1, highly ABA-induced PP2C gene 1 | 40.6677 | 11.3602 | -1.83989 | 0.0005 | 0.00424432 |
| AT5G28630 | glycine-rich protein | 113.9 | 31.8575 | -1.83807 | 0.00025 | 0.00253767 |
| AT4G22610 | Bifunctional inhibitor/lipid-transfer protein/seed storage 2S albumin superfamily protein | 689.309 | 192.824 | -1.83787 | 0.00005 | 0.00070028 |
| AT1G49240 | ACT8, actin 8 | 1495.81 | 418.454 | -1.83779 | 0.0002 | 0.00214445 |
| AT5G45370 | nodulin MtN21 /EamA-like transporter family protein | 17.9421 | 5.02035 | -1.83749 | 0.0007 | 0.00547072 |
| AT4G27320 | ATPHOS34, PHOS34, Adenine nucleotide alpha hydrolases-like superfamily protein | 267.712 | 74.9122 | -1.83741 | 0.0001 | 0.00123775 |
| AT1G80450 | VQ motif-containing protein | 36.2114 | 10.1338 | -1.83727 | 0.0004 | 0.00359419 |
| AT2G21510 | DNAJ heat shock N-terminal domain-containing protein | 29.7385 | 8.32959 | -1.83601 | 0.0004 | 0.00359419 |
| AT4G31470 | CAP (Cysteine-rich secretory proteins, Antigen 5, and Pathogenesis-related 1 protein) superfamily protein | 98.0658 | 27.4756 | -1.8356 | 0.00035 | 0.00327416 |
| AT5G13210 | Uncharacterised conserved protein UCP015417, vWA | 121.552 | 34.0587 | -1.83548 | 0.00005 | 0.00070028 |
| AT5G45472 | Potential natural antisense gene, locus overlaps with AT5G45470 | 18.5247 | 5.19783 | -1.83347 | 0.00115 | 0.00787231 |
| AT5G52250 | Transducin/WD40 repeat-like superfamily protein | 9.01629 | 2.53009 | -1.83334 | 0.00135 | 0.0089118 |
| AT5G56150 | UBC30, ubiquitin-conjugating enzyme 30 | 194.655 | 54.6342 | -1.83304 | 0.00005 | 0.00070028 |
| AT3G57230 | AGL16, AGAMOUS-like 16 | 48.8883 | 13.727 | -1.83247 | 0.00025 | 0.00253767 |
| AT5G67480 | ATBT4, BT4, BTB and TAZ domain protein 4 | 255.167 | 71.6529 | -1.83235 | 0.00005 | 0.00070028 |
| AT3G19240 | Vacuolar import/degradation, Vid27-related protein | 350.111 | 98.3167 | -1.8323 | 0.0001 | 0.00123775 |
| AT1G10140 | Uncharacterised conserved protein UCP031279 | 383.338 | 107.714 | -1.83141 | 0.00005 | 0.00070028 |
| AT5G44050 | MATE efflux family protein | 72.4441 | 20.3635 | -1.83088 | 0.00005 | 0.00070028 |
| AT3G29035 | ANAC059, ATNAC3, NAC3, NAC domain containing protein 3 | 109.127 | 30.6844 | -1.83043 | 0.00005 | 0.00070028 |
| AT1G51360 | ATDABB1, DABB1, dimeric A/B barrel domainS-protein 1 | 26.7404 | 7.52311 | -1.82962 | 0.00195 | 0.0117451 |
| AT1G64460 | Protein kinase superfamily protein | 403.738 | 113.635 | -1.82901 | 0.00005 | 0.00070028 |
| AT1G67050 | unknown protein | 12.3242 | 3.47135 | -1.82792 | 0.0019 | 0.0115387 |
| AT4G36820 | Protein of unknown function (DUF607) | 122.752 | 34.5936 | -1.82717 | 0.0001 | 0.00123775 |
| AT4G00440 | Protein of unknown function (DUF3741) | 12.1084 | 3.41702 | -1.82519 | 0.0003 | 0.00292382 |
| AT4G03110 | AtRBP-DR1, RBP-DR1, RNA-binding protein-defense related 1 | 75.7694 | 21.3893 | -1.82472 | 0.00005 | 0.00070028 |
| AT1G29680 | Protein of unknown function (DUF1264) | 10.6132 | 2.99796 | -1.82381 | 0.0065 | 0.0287784 |
| AT3G20510 | Transmembrane proteins 14C | 213.264 | 60.2618 | -1.82332 | 0.00015 | 0.00170235 |
| AT5G15190 | unknown protein | 115.585 | 32.6688 | -1.82297 | 0.0002 | 0.00214445 |
| AT1G67480 | Galactose oxidase/kelch repeat superfamily protein | 486.421 | 137.648 | -1.82123 | 0.00015 | 0.00170235 |
| AT3G48520 | CYP94B3, cytochrome P450, family 94, subfamily B, polypeptide 3 | 126.147 | 35.7016 | -1.82104 | 0.00005 | 0.00070028 |
| AT3G57330 | ACA11, autoinhibited Ca2+-ATPase 11 | 132.976 | 37.6509 | -1.82041 | 0.00005 | 0.00070028 |
| AT1G79160 | unknown protein | 110.65 | 31.3521 | -1.81937 | 0.00015 | 0.00170235 |
| AT5G46230 | Protein of unknown function, DUF538 | 85.5275 | 24.2494 | -1.81844 | 0.00065 | 0.00516972 |
| AT2G31345 | unknown protein | 12.3679 | 3.50687 | -1.81835 | 0.0119 | 0.0448549 |
| AT2G35930 | PUB23, plant U-box 23 | 113.232 | 32.1137 | -1.81802 | 0.00005 | 0.00070028 |
| AT3G16350 | Homeodomain-like superfamily protein | 80.7608 | 22.9207 | -1.817 | 0.00005 | 0.00070028 |
| AT4G03510 | ATRMA1, RMA1, RING membrane-anchor 1 | 361.587 | 102.716 | -1.81569 | 0.00005 | 0.00070028 |
| AT3G10960 | ATAZG1, AZG1, AZA-guanine resistant1 | 166.839 | 47.4165 | -1.81499 | 0.00005 | 0.00070028 |
| AT1G23550 | SRO2, similar to RCD one 2 | 6.67282 | 1.89675 | -1.81477 | 0.011 | 0.0422342 |
| AT3G48100 | ARR5, ATRR2, IBC6, RR5, response regulator 5 | 159.127 | 45.2639 | -1.81375 | 0.00005 | 0.00070028 |
| AT3G27240 | Cytochrome C1 family | 440.435 | 125.396 | -1.81243 | 0.00005 | 0.00070028 |
| AT1G24180 | IAR4, Thiamin diphosphate-binding fold (THDP-binding) superfamily protein | 398.713 | 113.525 | -1.81234 | 0.00015 | 0.00170235 |
| AT3G27890 | NQR, NADPH:quinone oxidoreductase | 151.262 | 43.0954 | -1.81144 | 0.00005 | 0.00070028 |
| AT5G24640 | unknown protein | 21.3747 | 6.09081 | -1.8112 | 0.01255 | 0.0465083 |
| AT2G17710 | unknown protein | 35.6985 | 10.1854 | -1.80936 | 0.0005 | 0.00424432 |
| AT1G60750 | NAD(P)-linked oxidoreductase superfamily protein | 36.2223 | 10.3391 | -1.80877 | 0.0006 | 0.00487984 |
| AT4G22592 | CPuORF27, conserved peptide upstream open reading frame 27 | 966.459 | 275.933 | -1.80839 | 0.00025 | 0.00253767 |
| AT5G13910 | LEP, Integrase-type DNA-binding superfamily protein | 39.4181 | 11.2636 | -1.8072 | 0.00215 | 0.0126571 |
| AT1G15670 | Galactose oxidase/kelch repeat superfamily protein | 429.508 | 122.931 | -1.80484 | 0.00005 | 0.00070028 |
| AT3G61410 | BEST Arabidopsis thaliana protein match is: U-box domain-containing protein kinase family protein (TAIR:AT2G45910.1) | 19.9168 | 5.70126 | -1.80463 | 0.00065 | 0.00516972 |
| AT2G16770 | bZIP23, Basic-leucine zipper (bZIP) transcription factor family protein | 18.3224 | 5.24778 | -1.80383 | 0.00085 | 0.00630464 |
| AT2G02410 | unknown protein | 6.78504 | 1.94344 | -1.80374 | 0.00745 | 0.0318523 |
| AT5G46780 | VQ motif-containing protein | 73.4034 | 21.0306 | -1.80336 | 0.00015 | 0.00170235 |
| AT2G41660 | MIZ1, Protein of unknown function, DUF617 | 270.944 | 77.6576 | -1.8028 | 0.00005 | 0.00070028 |
| AT1G22470 | unknown protein | 118.513 | 34.0065 | -1.80117 | 0.0003 | 0.00292382 |
| AT5G20540 | ATBRXL4, BRX-LIKE4, BRXL4, BREVIS RADIX-like 4 | 9.40444 | 2.69888 | -1.80098 | 0.0011 | 0.00759303 |
| AT2G16510 | ATPase, F0/V0 complex, subunit C protein | 242.249 | 69.5824 | -1.79969 | 0.00005 | 0.00070028 |
| AT1G68940 | Armadillo/beta-catenin-like repeat family protein | 2.78771 | 0.801094 | -1.79904 | 0.0055 | 0.0254967 |
| AT2G22470 | AGP2, ATAGP2, arabinogalactan protein 2 | 3220.46 | 925.556 | -1.79887 | 0.0017 | 0.0106071 |
| AT2G05940 | Protein kinase superfamily protein | 66.0352 | 18.9922 | -1.79783 | 0.0002 | 0.00214445 |
| AT5G56950 | NAP1;3, NFA03, NFA3, nucleosome assembly protein 1;3 | 136.201 | 39.182 | -1.79748 | 0.0001 | 0.00123775 |
| AT3G58710 | ATWRKY69, WRKY69, WRKY DNA-binding protein 69 | 118.658 | 34.1444 | -1.79709 | 0.00025 | 0.00253767 |
| AT4G00330 | CRCK2, calmodulin-binding receptor-like cytoplasmic kinase 2 | 25.0038 | 7.19712 | -1.79665 | 0.00045 | 0.003919 |
| AT1G47128 | RD21, RD21A, Granulin repeat cysteine protease family protein | 766.855 | 220.755 | -1.79651 | 0.00055 | 0.00457655 |
| AT5G64310 | AGP1, ATAGP1, arabinogalactan protein 1 | 1739.09 | 501.379 | -1.79436 | 0.00015 | 0.00170235 |
| AT2G28550 | RAP2.7, TOE1, related to AP2.7 | 29.8828 | 8.61754 | -1.79397 | 0.00065 | 0.00516972 |
| AT5G54585 | unknown protein | 37.1068 | 10.7015 | -1.79388 | 0.0013 | 0.00867426 |
| AT1G69800 | Cystathionine beta-synthase (CBS) protein | 35.1853 | 10.1535 | -1.79299 | 0.0003 | 0.00292382 |
| AT2G17840 | ERD7, Senescence/dehydration-associated protein-related | 677.79 | 195.594 | -1.79298 | 0.0005 | 0.00424432 |
| AT5G56760 | ATSERAT1;1, SAT-52, SAT5, SERAT1;1, serine acetyltransferase 1;1 | 243.944 | 70.4109 | -1.79268 | 0.00005 | 0.00070028 |
| AT4G32300 | SD2-5, S-domain-2 5 | 12.9491 | 3.74348 | -1.79041 | 0.0002 | 0.00214445 |
| AT3G54680 | proteophosphoglycan-related | 159.124 | 46.0036 | -1.79033 | 0.00005 | 0.00070028 |
| AT4G39890 | AtRABH1c, RABH1c, RAB GTPase homolog H1C | 95.2884 | 27.5567 | -1.7899 | 0.00015 | 0.00170235 |
| AT5G20650 | COPT5, copper transporter 5 | 114.657 | 33.1603 | -1.78979 | 0.00015 | 0.00170235 |
| AT1G53790 | F-box and associated interaction domains-containing protein | 4.70666 | 1.36268 | -1.78825 | 0.0059 | 0.0268421 |
| AT3G03170 | unknown protein | 203.964 | 59.0553 | -1.78817 | 0.0001 | 0.00123775 |
| AT2G39780 | RNS2, ribonuclease 2 | 322.217 | 93.3188 | -1.78779 | 0.00005 | 0.00070028 |
| AT5G05440 | PYL5, RCAR8, Polyketide cyclase/dehydrase and lipid transport superfamily protein | 96.5699 | 27.9988 | -1.78621 | 0.0001 | 0.00123775 |
| AT3G61180 | RING/U-box superfamily protein | 54.5134 | 15.8121 | -1.78558 | 0.00005 | 0.00070028 |
| AT4G38250 | Transmembrane amino acid transporter family protein | 77.9488 | 22.6118 | -1.78545 | 0.0037 | 0.0188077 |
| AT3G16560 | Protein phosphatase 2C family protein | 20.7902 | 6.03176 | -1.78525 | 0.0004 | 0.00359419 |
| AT2G23910 | NAD(P)-binding Rossmann-fold superfamily protein | 5.40046 | 1.56798 | -1.78418 | 0.00935 | 0.037453 |
| AT3G52370 | FLA15, FASCICLIN-like arabinogalactan protein 15 precursor | 14.8346 | 4.30956 | -1.78335 | 0.00095 | 0.00685384 |
| AT5G17350 | unknown protein | 62.355 | 18.116 | -1.78324 | 0.00325 | 0.0171315 |
| AT3G13970 | APG12, APG12B, Ubiquitin-like superfamily protein | 22.3329 | 6.48896 | -1.78311 | 0.0057 | 0.0262091 |
| AT2G02080 | AtIDD4, IDD4, indeterminate(ID)-domain 4 | 6.03437 | 1.75353 | -1.78294 | 0.0025 | 0.0140894 |
| AT3G24670 | Pectin lyase-like superfamily protein | 481.013 | 139.818 | -1.78252 | 0.0008 | 0.00603091 |
| AT1G75810 | unknown protein | 19.3714 | 5.63276 | -1.78201 | 0.00875 | 0.0357584 |
| AT5G58800 | Quinone reductase family protein | 33.652 | 9.79985 | -1.77986 | 0.0041 | 0.0203755 |
| AT4G02075 | PIT1, RING/FYVE/PHD zinc finger superfamily protein | 23.8967 | 6.96019 | -1.77961 | 0.0023 | 0.0132526 |
| AT5G46900 | Bifunctional inhibitor/lipid-transfer protein/seed storage 2S albumin superfamily protein | 80.0738 | 23.358 | -1.77741 | 0.001 | 0.00711095 |
| AT4G32920 | glycine-rich protein | 59.7635 | 17.4505 | -1.776 | 0.00005 | 0.00070028 |
| AT4G35550 | ATWOX13, HB-4, WOX13, WUSCHEL related homeobox 13 | 61.4362 | 17.9595 | -1.77434 | 0.0027 | 0.0149318 |
| AT4G14370 | Disease resistance protein (TIR-NBS-LRR class) family | 28.2606 | 8.26189 | -1.77425 | 0.00005 | 0.00070028 |
| AT1G27910 | ATPUB45, PUB45, plant U-box 45 | 48.9338 | 14.3056 | -1.77425 | 0.00025 | 0.00253767 |
| AT2G16870 | Disease resistance protein (TIR-NBS-LRR class) family | 7.64277 | 2.23463 | -1.77406 | 0.00105 | 0.00735817 |
| AT4G31875 | unknown protein | 20.0661 | 5.86968 | -1.77341 | 0.0056 | 0.0258574 |
| AT1G15400 | unknown protein | 276.42 | 80.8868 | -1.77288 | 0.00035 | 0.00327416 |
| AT1G51370 | F-box/RNI-like/FBD-like domains-containing protein | 11.619 | 3.40115 | -1.77239 | 0.00225 | 0.0130442 |
| AT5G43830 | Aluminium induced protein with YGL and LRDR motifs | 913.848 | 267.619 | -1.77178 | 0.0001 | 0.00123775 |
| AT4G37530 | Peroxidase superfamily protein | 52.2388 | 15.299 | -1.77169 | 0.0005 | 0.00424432 |
| AT3G14920 | Peptide-N4-(N-acetyl-beta-glucosaminyl)asparagine amidase A protein | 12.4159 | 3.63744 | -1.7712 | 0.00105 | 0.00735817 |
| AT4G18070 | unknown protein | 150.668 | 44.1548 | -1.77073 | 0.00015 | 0.00170235 |
| AT3G47340 | ASN1, AT-ASN1, DIN6, glutamine-dependent asparagine synthase 1 | 622.356 | 182.487 | -1.76994 | 0.001 | 0.00711095 |
| AT2G25520 | Drug/metabolite transporter superfamily protein | 274.687 | 80.6351 | -1.76831 | 0.00015 | 0.00170235 |
| AT3G57410 | ATVLN3, VLN3, villin 3 | 124.342 | 36.5097 | -1.76796 | 0.00005 | 0.00070028 |
| AT4G02620 | vacuolar ATPase subunit F family protein | 700.66 | 205.858 | -1.76706 | 0.00015 | 0.00170235 |
| AT2G47700 | RFI2, RING/U-box superfamily protein | 98.7352 | 29.0208 | -1.76648 | 0.0093 | 0.0372979 |
| AT5G16380 | Protein of unknown function, DUF538 | 55.7806 | 16.4159 | -1.76466 | 0.0008 | 0.00603091 |
| AT5G19120 | Eukaryotic aspartyl protease family protein | 273.504 | 80.5682 | -1.76328 | 0.00025 | 0.00253767 |
| AT2G03730 | ACR5, ACT domain repeat 5 | 102.95 | 30.3333 | -1.76297 | 0.0001 | 0.00123775 |
| AT5G19020 | MEF18, mitochondrial editing factor 18 | 25.5289 | 7.52262 | -1.76282 | 0.0001 | 0.00123775 |
| AT1G25370 | Protein of unknown function (DUF1639) | 16.8872 | 4.97684 | -1.76263 | 0.0019 | 0.0115387 |
| AT4G38920 | ATVHA-C3, AVA-P3, VHA-C3, vacuolar-type H(+)-ATPase C3 | 572.495 | 168.82 | -1.76177 | 0.00005 | 0.00070028 |
| AT2G43330 | ATINT1, INT1, inositol transporter 1 | 121.522 | 35.8465 | -1.76131 | 0.00005 | 0.00070028 |
| AT1G07040 | unknown protein | 55.6657 | 16.4369 | -1.75985 | 0.0001 | 0.00123775 |
| AT1G18620 | unknown protein | 21.8158 | 6.46175 | -1.75538 | 0.00015 | 0.00170235 |
| AT2G41000 | Chaperone DnaJ-domain superfamily protein | 5.17211 | 1.53224 | -1.75511 | 0.00895 | 0.0364179 |
| AT5G06810 | Mitochondrial transcription termination factor family protein | 9.61365 | 2.85003 | -1.75411 | 0.00035 | 0.00327416 |
| AT4G26910 | Dihydrolipoamide succinyltransferase | 201.259 | 59.6843 | -1.75363 | 0.0006 | 0.00487984 |
| AT5G39970 | catalytics | 32.0089 | 9.49371 | -1.75343 | 0.00045 | 0.003919 |
| AT2G32150 | Haloacid dehalogenase-like hydrolase (HAD) superfamily protein | 1407.9 | 417.731 | -1.7529 | 0.00085 | 0.00630464 |
| AT1G11660 | heat shock protein 70 (Hsp 70) family protein | 84.1365 | 24.9657 | -1.75279 | 0.00015 | 0.00170235 |
| AT5G03555 | permease, cytosine/purines, uracil, thiamine, allantoin family protein | 23.8641 | 7.08281 | -1.75245 | 0.0005 | 0.00424432 |
| AT5G34930 | arogenate dehydrogenase | 33.8319 | 10.0518 | -1.75093 | 0.00015 | 0.00170235 |
| AT5G65140 | Haloacid dehalogenase-like hydrolase (HAD) superfamily protein | 131.562 | 39.1553 | -1.74847 | 0.00015 | 0.00170235 |
| AT3G14440 | ATNCED3, NCED3, SIS7, STO1, nine-cis-epoxycarotenoid dioxygenase 3 | 7.49749 | 2.23388 | -1.74686 | 0.0018 | 0.0110912 |
| AT5G01800 | saposin B domain-containing protein | 275.465 | 82.1149 | -1.74616 | 0.0002 | 0.00214445 |
| AT1G07750 | RmlC-like cupins superfamily protein | 317.538 | 94.6886 | -1.74567 | 0.0001 | 0.00123775 |
| AT1G22550 | Major facilitator superfamily protein | 10.3585 | 3.09013 | -1.74508 | 0.00085 | 0.00630464 |
| AT3G06470 | GNS1/SUR4 membrane protein family | 13.1107 | 3.91154 | -1.74494 | 0.0029 | 0.0157089 |
| AT1G75380 | ATBBD1, BBD1, bifunctional nuclease in basal defense response 1 | 94.0376 | 28.071 | -1.74416 | 0.0001 | 0.00123775 |
| AT3G15750 | Essential protein Yae1, N-terminal | 10.5282 | 3.14541 | -1.74294 | 0.0064 | 0.0284694 |
| AT2G24610 | ATCNGC14, CNGC14, cyclic nucleotide-gated channel 14 | 61.2646 | 18.3104 | -1.74239 | 0.0003 | 0.00292382 |
| AT5G05090 | Homeodomain-like superfamily protein | 4.6311 | 1.38431 | -1.74218 | 0.0087 | 0.0356202 |
| AT3G09010 | Protein kinase superfamily protein | 7.15196 | 2.13839 | -1.74181 | 0.0026 | 0.0145003 |
| AT4G25560 | AtMYB18, MYB18, myb domain protein 18 | 11.8331 | 3.54028 | -1.7409 | 0.0077 | 0.0326253 |
| AT3G29770 | ATMES11, MES11, methyl esterase 11 | 3.83842 | 1.14905 | -1.74007 | 0.0084 | 0.0346931 |
| AT3G59350 | Protein kinase superfamily protein | 469.079 | 140.433 | -1.73995 | 0.0004 | 0.00359419 |
| AT1G03900 | ATNAP4, NAP4, non-intrinsic ABC protein 4 | 256.122 | 76.8172 | -1.73733 | 0.00015 | 0.00170235 |
| AT2G31570 | ATGPX2, GPX2, glutathione peroxidase 2 | 469.491 | 140.861 | -1.73683 | 0.0002 | 0.00214445 |
| AT3G27170 | ATCLC-B, CLC-B, chloride channel B | 7.05942 | 2.11832 | -1.73663 | 0.00645 | 0.0286275 |
| AT3G61190 | BAP1, BON association protein 1 | 124.091 | 37.2426 | -1.73637 | 0.0001 | 0.00123775 |
| AT3G56275 | pseudogene of unknown protein | 29.6053 | 8.88538 | -1.73635 | 0.00295 | 0.0159014 |
| AT5G03030 | Chaperone DnaJ-domain superfamily protein | 355.549 | 106.804 | -1.73508 | 0.00015 | 0.00170235 |
| AT3G62830 | ATUXS2, AUD1, UXS2, NAD(P)-binding Rossmann-fold superfamily protein | 268.258 | 80.6796 | -1.73334 | 0.00055 | 0.00457655 |
| AT3G21070 | ATNADK-1, NADK1, NAD kinase 1 | 293.348 | 88.3031 | -1.73207 | 0.0002 | 0.00214445 |
| AT4G12080 | AHL1, ATAHL1, AT-hook motif nuclear-localized protein 1 | 107.577 | 32.389 | -1.7318 | 0.00005 | 0.00070028 |
| AT1G14860 | atnudt18, NUDT18, nudix hydrolase homolog 18 | 201.252 | 60.6038 | -1.73153 | 0.00005 | 0.00070028 |
| AT1G76930 | ATEXT1, ATEXT4, EXT1, EXT4, ORG5, extensin 4 | 221.946 | 66.8919 | -1.73031 | 0.0003 | 0.00292382 |
| AT1G51770 | Core-2/I-branching beta-1,6-N-acetylglucosaminyltransferase family protein | 10.5971 | 3.19391 | -1.73027 | 0.0039 | 0.0196279 |
| AT1G11210 | Protein of unknown function (DUF761) | 39.6188 | 11.9545 | -1.72863 | 0.00035 | 0.00327416 |
| AT5G24460 | unknown protein | 24.1055 | 7.27495 | -1.72835 | 0.00465 | 0.0223951 |
| AT4G36110 | SAUR-like auxin-responsive protein family | 13.3057 | 4.01619 | -1.72814 | 0.0022 | 0.0128446 |
| AT4G25170 | Uncharacterised conserved protein (UCP012943) | 34.7135 | 10.4834 | -1.72739 | 0.00405 | 0.0202184 |
| AT3G07960 | Phosphatidylinositol-4-phosphate 5-kinase family protein | 16.1538 | 4.87844 | -1.72738 | 0.00045 | 0.003919 |
| AT1G12810 | proline-rich family protein | 77.6718 | 23.5191 | -1.72356 | 0.0077 | 0.0326253 |
| AT2G47830 | Cation efflux family protein | 20.8916 | 6.32791 | -1.72312 | 0.0006 | 0.00487984 |
| AT3G08650 | ZIP metal ion transporter family | 93.353 | 28.3398 | -1.71987 | 0.00025 | 0.00253767 |
| AT1G70660 | MMZ2, UEV1B, MMS ZWEI homologue 2 | 164.031 | 49.7965 | -1.71986 | 0.00025 | 0.00253767 |
| AT3G10300 | Calcium-binding EF-hand family protein | 216.4 | 65.7395 | -1.71887 | 0.0001 | 0.00123775 |
| AT1G30690 | Sec14p-like phosphatidylinositol transfer family protein | 161.157 | 48.9575 | -1.71886 | 0.00005 | 0.00070028 |
| AT3G55770 | GATA type zinc finger transcription factor family protein | 243.928 | 74.1422 | -1.71809 | 0.00005 | 0.00070028 |
| AT5G67500 | ATVDAC2, VDAC2, voltage dependent anion channel 2 | 375.643 | 114.277 | -1.71683 | 0.00015 | 0.00170235 |
| AT3G48530 | KING1, SNF1-related protein kinase regulatory subunit gamma 1 | 350.623 | 106.701 | -1.71635 | 0.0001 | 0.00123775 |
| AT5G39320 | UDP-glucose 6-dehydrogenase family protein | 496.073 | 151.022 | -1.71579 | 0.00215 | 0.0126571 |
| AT5G57710 | Double Clp-N motif-containing P-loop nucleoside triphosphate hydrolases superfamily protein | 60.3405 | 18.3719 | -1.71563 | 0.0002 | 0.00214445 |
| AT2G28080 | UDP-Glycosyltransferase superfamily protein | 22.7174 | 6.91992 | -1.71497 | 0.0005 | 0.00424432 |
| AT4G17570 | GATA26, GATA transcription factor 26 | 23.1125 | 7.05021 | -1.71294 | 0.0007 | 0.00547072 |
| AT3G11320 | Nucleotide-sugar transporter family protein | 123.131 | 37.5714 | -1.71248 | 0.0001 | 0.00123775 |
| AT2G22560 | Kinase interacting (KIP1-like) family protein | 57.6544 | 17.5945 | -1.71231 | 0.0001 | 0.00123775 |
| AT1G19940 | AtGH9B5, GH9B5, glycosyl hydrolase 9B5 | 3.81656 | 1.16492 | -1.71204 | 0.0107 | 0.0414196 |
| AT1G22985 | Integrase-type DNA-binding superfamily protein | 269.88 | 82.3759 | -1.71203 | 0.00015 | 0.00170235 |
| AT3G15740 | RING/U-box superfamily protein | 33.6194 | 10.2635 | -1.71177 | 0.00165 | 0.0103801 |
| AT2G21045 | Rhodanese/Cell cycle control phosphatase superfamily protein | 125.05 | 38.1773 | -1.71172 | 0.00045 | 0.003919 |
| AT5G63160 | BT1, BTB and TAZ domain protein 1 | 53.2257 | 16.2543 | -1.7113 | 0.00045 | 0.003919 |
| AT2G46735 | unknown protein | 12.6173 | 3.85361 | -1.71113 | 0.0073 | 0.0313803 |
| AT4G15610 | Uncharacterised protein family (UPF0497) | 920.066 | 281.058 | -1.71087 | 0.0001 | 0.00123775 |
| AT1G74770 | zinc ion binding | 16.8997 | 5.16404 | -1.71042 | 0.00035 | 0.00327416 |
| AT2G02480 | STI, AAA-type ATPase family protein | 30.9978 | 9.47418 | -1.71009 | 0.00025 | 0.00253767 |
| AT4G27740 | Yippee family putative zinc-binding protein | 20.0257 | 6.12532 | -1.70899 | 0.00805 | 0.0336689 |
| AT1G26640 | Amino acid kinase family protein | 68.4817 | 20.9517 | -1.70865 | 0.0002 | 0.00214445 |
| AT5G56170 | LLG1, LORELEI-LIKE-GPI-ANCHORED PROTEIN 1 | 207.643 | 63.5561 | -1.708 | 0.0001 | 0.00123775 |
| AT2G17720 | 2-oxoglutarate (2OG) and Fe(II)-dependent oxygenase superfamily protein | 506.32 | 155.026 | -1.70754 | 0.0005 | 0.00424432 |
| AT4G36515 | unknown protein | 37.8598 | 11.6054 | -1.70587 | 0.0071 | 0.0306604 |
| AT4G25030 | unknown protein | 394.861 | 121.057 | -1.70565 | 0.0001 | 0.00123775 |
| AT1G70590 | F-box family protein | 144.528 | 44.3325 | -1.70491 | 0.0001 | 0.00123775 |
| AT2G21830 | Cysteine/Histidine-rich C1 domain family protein | 6.39639 | 1.96239 | -1.70465 | 0.0039 | 0.0196279 |
| AT1G78680 | ATGGH2, GGH2, gamma-glutamyl hydrolase 2 | 77.3343 | 23.7285 | -1.70449 | 0.00045 | 0.003919 |
| AT1G62600 | Flavin-binding monooxygenase family protein | 20.218 | 6.20364 | -1.70445 | 0.0019 | 0.0115387 |
| AT2G16660 | Major facilitator superfamily protein | 478.088 | 146.703 | -1.70438 | 0.0004 | 0.00359419 |
| AT4G13890 | EDA36, EDA37, SHM5, Pyridoxal phosphate (PLP)-dependent transferases superfamily protein | 18.6517 | 5.72393 | -1.70423 | 0.0024 | 0.0136815 |
| AT1G63800 | UBC5, ubiquitin-conjugating enzyme 5 | 31.0328 | 9.52441 | -1.70409 | 0.0016 | 0.0101494 |
| AT3G63060 | EDL3, EID1-like 3 | 41.6347 | 12.7883 | -1.70296 | 0.0011 | 0.00759303 |
| AT5G43520 | Cysteine/Histidine-rich C1 domain family protein | 125.817 | 38.662 | -1.70234 | 0.00055 | 0.00457655 |
| AT2G16920 | PFU2, UBC23, ubiquitin-conjugating enzyme 23 | 32.144 | 9.88216 | -1.70165 | 0.00005 | 0.00070028 |
| AT3G20300 | Protein of unknown function (DUF3537) | 158.605 | 48.7911 | -1.70075 | 0.00005 | 0.00070028 |
| AT5G62540 | UBC3, ubiquitin-conjugating enzyme 3 | 474.591 | 146.037 | -1.70035 | 0.0004 | 0.00359419 |
| AT1G61740 | Sulfite exporter TauE/SafE family protein | 88.217 | 27.1569 | -1.69974 | 0.00015 | 0.00170235 |
| AT5G40170 | AtRLP54, RLP54, receptor like protein 54 | 7.53384 | 2.32045 | -1.69898 | 0.00225 | 0.0130442 |
| AT1G78600 | DBB3, LZF1, STH3, light-regulated zinc finger protein 1 | 92.8573 | 28.608 | -1.6986 | 0.0002 | 0.00214445 |
| AT4G12000 | SNARE associated Golgi protein family | 253.892 | 78.3078 | -1.69699 | 0.0001 | 0.00123775 |
| AT5G63450 | CYP94B1, cytochrome P450, family 94, subfamily B, polypeptide 1 | 145.002 | 44.7236 | -1.69696 | 0.00005 | 0.00070028 |
| AT2G02955 | MEE12, maternal effect embryo arrest 12 | 6.71312 | 2.0716 | -1.69624 | 0.00175 | 0.0108609 |
| AT3G03050 | ATCSLD3, CSLD3, KJK, cellulose synthase-like D3 | 215.2 | 66.4501 | -1.69533 | 0.0023 | 0.0132526 |
| AT4G39670 | Glycolipid transfer protein (GLTP) family protein | 128.564 | 39.7069 | -1.69503 | 0.0001 | 0.00123775 |
| AT4G01050 | TROL, thylakoid rhodanese-like | 25.4304 | 7.85427 | -1.69501 | 0.00035 | 0.00327416 |
| AT3G49845 | unknown protein | 154.697 | 47.7893 | -1.69469 | 0.0003 | 0.00292382 |
| AT3G23170 | unknown protein | 41.2768 | 12.7546 | -1.69432 | 0.0007 | 0.00547072 |
| AT1G03920 | Protein kinase family protein | 7.26049 | 2.24393 | -1.69404 | 0.0031 | 0.0165078 |
| AT2G20820 | unknown protein | 683.525 | 211.331 | -1.69349 | 0.0001 | 0.00123775 |
| AT5G19230 | Glycoprotein membrane precursor GPI-anchored | 986.846 | 305.293 | -1.69263 | 0.0003 | 0.00292382 |
| AT4G28710 | ATXIH, XIH, Myosin family protein with Dil domain | 29.7934 | 9.22395 | -1.69154 | 0.00035 | 0.00327416 |
| AT3G51240 | F3'H, F3H, TT6, flavanone 3-hydroxylase | 35.9907 | 11.1429 | -1.69151 | 0.00285 | 0.0154761 |
| AT1G58190 | AtRLP9, RLP9, receptor like protein 9 | 42.1046 | 13.05 | -1.68993 | 0.0001 | 0.00123775 |
| AT4G16110 | ARR2, RR2, response regulator 2 | 40.1997 | 12.4712 | -1.68859 | 0.0001 | 0.00123775 |
| AT4G32150 | ATVAMP711, VAMP711, vesicle-associated membrane protein 711 | 241.923 | 75.0958 | -1.68774 | 0.00015 | 0.00170235 |
| AT4G23470 | PLAC8 family protein | 223.15 | 69.2986 | -1.68711 | 0.00005 | 0.00070028 |
| AT5G10750 | Protein of unknown function (DUF1336) | 23.6928 | 7.36058 | -1.68656 | 0.0035 | 0.0180741 |
| AT2G35800 | mitochondrial substrate carrier family protein | 42.0854 | 13.0801 | -1.68594 | 0.00005 | 0.00070028 |
| AT4G28085 | unknown protein | 69.4596 | 21.5945 | -1.68551 | 0.00265 | 0.0147335 |
| AT2G02710 | PLP, PLPA, PLPB, PLPC, PAS/LOV protein B | 179.907 | 55.9555 | -1.6849 | 0.0002 | 0.00214445 |
| AT5G24010 | Protein kinase superfamily protein | 1.75838 | 0.547495 | -1.68333 | 0.0113 | 0.0430687 |
| AT4G04450 | AtWRKY42, WRKY42, WRKY family transcription factor | 6.54948 | 2.03994 | -1.68285 | 0.0102 | 0.0400002 |
| AT5G66650 | Protein of unknown function (DUF607) | 262.465 | 81.8255 | -1.6815 | 0.00005 | 0.00070028 |
| AT1G68760 | ATNUDT1, ATNUDX1, NUDX1, NUDX1, nudix hydrolase 1 | 13.8704 | 4.32462 | -1.68137 | 0.01 | 0.0393953 |
| AT5G49560 | Putative methyltransferase family protein | 12.7799 | 3.98493 | -1.68125 | 0.00675 | 0.0295876 |
| AT2G46170 | Reticulon family protein | 289.426 | 90.2973 | -1.68044 | 0.0003 | 0.00292382 |
| AT1G28480 | GRX480, roxy19, Thioredoxin superfamily protein | 340.939 | 106.395 | -1.68009 | 0.00005 | 0.00070028 |
| AT1G69530 | AT-EXP1, ATEXP1, ATEXPA1, ATHEXP ALPHA 1.2, EXP1, EXPA1, expansin A1 | 125.073 | 39.0356 | -1.67991 | 0.00015 | 0.00170235 |
| AT1G07030 | Mitochondrial substrate carrier family protein | 109.426 | 34.1605 | -1.67956 | 0.0002 | 0.00214445 |
| AT3G55840 | Hs1pro-1 protein | 206.042 | 64.332 | -1.67933 | 0.0003 | 0.00292382 |
| AT2G14100 | CYP705A13, cytochrome P450, family 705, subfamily A, polypeptide 13 | 25.3328 | 7.90979 | -1.67929 | 0.00075 | 0.00575475 |
| AT5G08770 | unknown protein | 20.0073 | 6.24761 | -1.67915 | 0.0045 | 0.0218802 |
| AT4G00240 | PLDBETA2, phospholipase D beta 2 | 13.0529 | 4.07637 | -1.67901 | 0.00095 | 0.00685384 |
| AT2G23450 | Protein kinase superfamily protein | 87.9509 | 27.5119 | -1.67664 | 0.0001 | 0.00123775 |
| AT5G14470 | GHMP kinase family protein | 22.8497 | 7.14908 | -1.67634 | 0.00115 | 0.00787231 |
| AT3G63200 | PLA IIIB, PLP9, PATATIN-like protein 9 | 20.9886 | 6.57083 | -1.67546 | 0.00225 | 0.0130442 |
| AT4G30170 | Peroxidase family protein | 540.027 | 169.19 | -1.67439 | 0.00055 | 0.00457655 |
| AT2G20080 | unknown protein | 62.7169 | 19.6498 | -1.67434 | 0.001 | 0.00711095 |
| AT5G51990 | CBF4, DREB1D, C-repeat-binding factor 4 | 18.3051 | 5.7372 | -1.67383 | 0.00525 | 0.0246552 |
| AT1G12140 | FMO GS-OX5, flavin-monooxygenase glucosinolate S-oxygenase 5 | 20.6828 | 6.48309 | -1.67368 | 0.0124 | 0.046125 |
| AT5G59550 | zinc finger (C3HC4-type RING finger) family protein | 103.794 | 32.5617 | -1.67247 | 0.0002 | 0.00214445 |
| AT5G11970 | Protein of unknown function (DUF3511) | 225.769 | 70.8363 | -1.67228 | 0.00035 | 0.00327416 |
| AT1G62990 | IXR11, KNAT7, KNOTTED-like homeobox of Arabidopsis thaliana 7 | 25.6446 | 8.04854 | -1.67186 | 0.0007 | 0.00547072 |
| AT3G26100 | Regulator of chromosome condensation (RCC1) family protein | 38.6651 | 12.1369 | -1.67163 | 0.0003 | 0.00292382 |
| AT1G76180 | ERD14, Dehydrin family protein | 4562.78 | 1432.35 | -1.67153 | 0.00085 | 0.00630464 |
| AT5G52310 | COR78, RD29A, | 618.896 | 194.762 | -1.66798 | 0.00305 | 0.0163293 |
| AT3G01980 | NAD(P)-binding Rossmann-fold superfamily protein | 28.8917 | 9.09412 | -1.66765 | 0.00135 | 0.0089118 |
| AT4G11150 | emb2448, TUF, TUFF, VHA-E1, vacuolar ATP synthase subunit E1 | 1116.71 | 351.516 | -1.6676 | 0.0003 | 0.00292382 |
| AT3G07470 | Protein of unknown function, DUF538 | 51.8664 | 16.3284 | -1.66741 | 0.001 | 0.00711095 |
| AT5G38550 | Mannose-binding lectin superfamily protein | 5.29845 | 1.66839 | -1.66711 | 0.00445 | 0.0217117 |
| AT1G55320 | AAE18, acyl-activating enzyme 18 | 37.389 | 11.7766 | -1.66669 | 0.0002 | 0.00214445 |
| AT2G16700 | ADF5, ATADF5, actin depolymerizing factor 5 | 19.9607 | 6.29187 | -1.66561 | 0.00415 | 0.020593 |
| AT2G15760 | Protein of unknown function (DUF1645) | 10.3456 | 3.2626 | -1.66492 | 0.0084 | 0.0346931 |
| AT5G62770 | Protein of unknown function (DUF1645) | 42.4198 | 13.3982 | -1.6627 | 0.0008 | 0.00603091 |
| AT5G40720 | Domain of unknown function (DUF23) | 3.65133 | 1.15367 | -1.6622 | 0.0099 | 0.0391493 |
| AT5G47390 | myb-like transcription factor family protein | 55.2604 | 17.4632 | -1.66193 | 0.00025 | 0.00253767 |
| AT4G39090 | RD19, RD19A, Papain family cysteine protease | 1309.12 | 413.94 | -1.6611 | 0.00215 | 0.0126571 |
| AT4G27470 | ATRMA3, RMA3, RING membrane-anchor 3 | 80.1146 | 25.338 | -1.66077 | 0.00005 | 0.00070028 |
| AT3G11020 | DREB2, DREB2B, DRE/CRT-binding protein 2B | 91.2854 | 28.8944 | -1.65959 | 0.0003 | 0.00292382 |
| AT5G62460 | RING/FYVE/PHD zinc finger superfamily protein | 182.813 | 57.8837 | -1.65914 | 0.0002 | 0.00214445 |
| AT4G36988 | CPuORF49, conserved peptide upstream open reading frame 49 | 462.778 | 146.7 | -1.65745 | 0.00065 | 0.00516972 |
| AT4G15290 | ATCSLB05, ATCSLB5, CSLB05, Cellulose synthase family protein | 3.61547 | 1.14629 | -1.65721 | 0.01045 | 0.0407225 |
| AT1G67070 | DIN9, PMI2, Mannose-6-phosphate isomerase, type I | 83.0979 | 26.3534 | -1.65682 | 0.00035 | 0.00327416 |
| AT5G49450 | AtbZIP1, bZIP1, basic leucine-zipper 1 | 937.389 | 297.292 | -1.65677 | 0.00075 | 0.00575475 |
| AT1G19360 | Nucleotide-diphospho-sugar transferase family protein | 149.934 | 47.574 | -1.65608 | 0.00075 | 0.00575475 |
| AT5G21940 | unknown protein | 1111.76 | 353.043 | -1.65493 | 0.00085 | 0.00630464 |
| AT4G27280 | Calcium-binding EF-hand family protein | 208.262 | 66.1948 | -1.65361 | 0.00045 | 0.003919 |
| AT1G13390 | unknown protein | 267.851 | 85.197 | -1.65256 | 0.00075 | 0.00575475 |
| AT1G70440 | SRO3, similar to RCD one 3 | 23.7183 | 7.54799 | -1.65183 | 0.0022 | 0.0128446 |
| AT2G05260 | alpha/beta-Hydrolases superfamily protein | 54.638 | 17.3988 | -1.65092 | 0.00015 | 0.00170235 |
| AT5G58560 | Phosphatidate cytidylyltransferase family protein | 71.3247 | 22.7148 | -1.65077 | 0.00025 | 0.00253767 |
| AT2G39410 | alpha/beta-Hydrolases superfamily protein | 21.486 | 6.84624 | -1.65001 | 0.0028 | 0.0152969 |
| AT3G17020 | Adenine nucleotide alpha hydrolases-like superfamily protein | 396.817 | 126.494 | -1.6494 | 0.00015 | 0.00170235 |
| AT5G47140 | GATA27, GATA transcription factor 27 | 22.0108 | 7.02159 | -1.64834 | 0.00085 | 0.00630464 |
| AT5G49480 | ATCP1, CP1, Ca2+-binding protein 1 | 684.413 | 218.337 | -1.64831 | 0.0002 | 0.00214445 |
| AT1G29120 | Hydrolase-like protein family | 25.7469 | 8.2233 | -1.64661 | 0.0006 | 0.00487984 |
| AT2G25735 | unknown protein | 61.8509 | 19.7673 | -1.64568 | 0.00155 | 0.009918 |
| AT2G30362 | other RNA | 351.438 | 112.469 | -1.64375 | 0.0007 | 0.00547072 |
| AT4G26470 | Calcium-binding EF-hand family protein | 92.9078 | 29.7429 | -1.64325 | 0.009 | 0.0365462 |
| AT1G05710 | basic helix-loop-helix (bHLH) DNA-binding superfamily protein | 25.4665 | 8.15425 | -1.64298 | 0.0033 | 0.0173075 |
| AT5G11770 | NADH-ubiquinone oxidoreductase 20 kDa subunit, mitochondrial | 284.336 | 91.0663 | -1.64261 | 0.00025 | 0.00253767 |
| AT5G12140 | ATCYS1, CYS1, cystatin-1 | 530.192 | 169.894 | -1.64188 | 0.0001 | 0.00123775 |
| AT1G15350 | unknown protein | 130.385 | 41.801 | -1.64117 | 0.0002 | 0.00214445 |
| AT4G10560 | MEE53, Cysteine/Histidine-rich C1 domain family protein | 2.06922 | 0.663673 | -1.64055 | 0.0128 | 0.0471875 |
| AT1G01720 | ANAC002, | 995.076 | 319.221 | -1.64025 | 0.0004 | 0.00359419 |
| AT1G24170 | GATL8, LGT9, Nucleotide-diphospho-sugar transferases superfamily protein | 87.9336 | 28.2139 | -1.64001 | 0.00025 | 0.00253767 |
| AT3G15300 | VQ motif-containing protein | 43.5715 | 13.9914 | -1.63884 | 0.00075 | 0.00575475 |
| AT3G51990 | Protein kinase superfamily protein | 34.5659 | 11.1008 | -1.63869 | 0.0003 | 0.00292382 |
| AT5G15130 | ATWRKY72, WRKY72, WRKY DNA-binding protein 72 | 35.4063 | 11.3726 | -1.63844 | 0.00065 | 0.00516972 |
| AT5G24100 | Leucine-rich repeat protein kinase family protein | 7.23416 | 2.32379 | -1.63835 | 0.0029 | 0.0157089 |
| AT1G53580 | ETHE1, GLX2-3, GLY3, glyoxalase II 3 | 196.427 | 63.1094 | -1.63807 | 0.0002 | 0.00214445 |
| AT1G68410 | Protein phosphatase 2C family protein | 145.953 | 46.9068 | -1.63764 | 0.00015 | 0.00170235 |
| AT5G62020 | AT-HSFB2A, HSFB2A, heat shock transcription factor B2A | 85.586 | 27.5297 | -1.63639 | 0.00025 | 0.00253767 |
| AT1G55190 | PRA1.F2, PRA7, PRA1 (Prenylated rab acceptor) family protein | 38.2822 | 12.3188 | -1.63581 | 0.0033 | 0.0173075 |
| AT5G18660 | PCB2, NAD(P)-binding Rossmann-fold superfamily protein | 3.52208 | 1.13344 | -1.63573 | 0.01325 | 0.0484049 |
| AT1G64470 | Ubiquitin-like superfamily protein | 433.178 | 139.503 | -1.63466 | 0.00015 | 0.00170235 |
| AT1G13260 | EDF4, RAV1, related to ABI3/VP1 1 | 105.171 | 33.873 | -1.63453 | 0.0001 | 0.00123775 |
| AT5G49440 | unknown protein | 242.918 | 78.3335 | -1.63277 | 0.00015 | 0.00170235 |
| AT1G60190 | ARM repeat superfamily protein | 66.8287 | 21.5536 | -1.63254 | 0.0002 | 0.00214445 |
| AT5G16510 | Alpha-1,4-glucan-protein synthase family protein | 185.052 | 59.6975 | -1.63219 | 0.00025 | 0.00253767 |
| AT1G51070 | bHLH115, basic helix-loop-helix (bHLH) DNA-binding superfamily protein | 115.714 | 37.3772 | -1.63034 | 0.00035 | 0.00327416 |
| AT1G21010 | unknown protein | 83.9291 | 27.1343 | -1.62905 | 0.00135 | 0.0089118 |
| AT5G55400 | Actin binding Calponin homology (CH) domain-containing protein | 95.8395 | 31.0082 | -1.62797 | 0.0004 | 0.00359419 |
| AT3G57790 | Pectin lyase-like superfamily protein | 16.0836 | 5.20771 | -1.62687 | 0.001 | 0.00711095 |
| AT2G22970 | SCPL11, serine carboxypeptidase-like 11 | 38.8072 | 12.5773 | -1.62551 | 0.0008 | 0.00603091 |
| AT2G45160 | ATHAM1, HAM1, GRAS family transcription factor | 80.1101 | 25.9677 | -1.62526 | 0.00005 | 0.00070028 |
| AT2G38740 | Haloacid dehalogenase-like hydrolase (HAD) superfamily protein | 110.145 | 35.7121 | -1.62492 | 0.0005 | 0.00424432 |
| AT3G45090 | P-loop containing nucleoside triphosphate hydrolases superfamily protein | 38.604 | 12.5221 | -1.62427 | 0.0003 | 0.00292382 |
| AT2G47130 | NAD(P)-binding Rossmann-fold superfamily protein | 45.6521 | 14.8097 | -1.62414 | 0.0014 | 0.00917155 |
| AT1G79450 | ALIS5, ALA-interacting subunit 5 | 78.4252 | 25.443 | -1.62405 | 0.0004 | 0.00359419 |
| AT5G60680 | Protein of unknown function, DUF584 | 740.661 | 240.743 | -1.62132 | 0.0002 | 0.00214445 |
| AT4G03115 | Mitochondrial substrate carrier family protein | 5.10899 | 1.66066 | -1.62128 | 0.0042 | 0.0207631 |
| AT3G48400 | Cysteine/Histidine-rich C1 domain family protein | 3.11462 | 1.01241 | -1.62127 | 0.01245 | 0.0462329 |
| AT4G23400 | PIP1;5, PIP1D, plasma membrane intrinsic protein 1;5 | 71.5946 | 23.2746 | -1.62109 | 0.0012 | 0.00815259 |
| AT2G28120 | Major facilitator superfamily protein | 4.19448 | 1.36367 | -1.621 | 0.0107 | 0.0414196 |
| AT1G27730 | STZ, ZAT10, salt tolerance zinc finger | 121.752 | 39.6096 | -1.62003 | 0.0003 | 0.00292382 |
| AT1G11410 | S-locus lectin protein kinase family protein | 28.1055 | 9.1435 | -1.62003 | 0.00025 | 0.00253767 |
| AT3G13910 | Protein of unknown function (DUF3511) | 47.1269 | 15.3329 | -1.61992 | 0.00615 | 0.0276932 |
| AT3G24300 | AMT1;3, ATAMT1;3, ammonium transporter 1;3 | 4.70156 | 1.53068 | -1.61896 | 0.0122 | 0.0455694 |
| AT4G16680 | P-loop containing nucleoside triphosphate hydrolases superfamily protein | 16.6191 | 5.4107 | -1.61895 | 0.0041 | 0.0203755 |
| AT1G74450 | Protein of unknown function (DUF793) | 98.7871 | 32.1647 | -1.61885 | 0.0003 | 0.00292382 |
| AT5G65200 | ATPUB38, PUB38, plant U-box 38 | 15.0479 | 4.9046 | -1.61735 | 0.0025 | 0.0140894 |
| AT3G51490 | TMT3, tonoplast monosaccharide transporter3 | 2.70395 | 0.881323 | -1.61732 | 0.01375 | 0.0496726 |
| AT3G54770 | RNA-binding (RRM/RBD/RNP motifs) family protein | 43.7494 | 14.2642 | -1.61686 | 0.00045 | 0.003919 |
| AT3G47833 | unknown protein | 175.647 | 57.2911 | -1.6163 | 0.0004 | 0.00359419 |
| AT3G61990 | S-adenosyl-L-methionine-dependent methyltransferases superfamily protein | 18.745 | 6.11433 | -1.61624 | 0.00285 | 0.0154761 |
| AT5G41110 | unknown protein | 10.8544 | 3.54203 | -1.61563 | 0.00105 | 0.00735817 |
| AT4G19860 | alpha/beta-Hydrolases superfamily protein | 151.628 | 49.5037 | -1.61493 | 0.0002 | 0.00214445 |
| AT4G20150 | unknown protein | 745.936 | 243.56 | -1.61477 | 0.0009 | 0.0065867 |
| AT3G06050 | ATPRXIIF, PRXIIF, peroxiredoxin IIF | 179.906 | 58.7523 | -1.61452 | 0.0001 | 0.00123775 |
| AT2G34350 | Nodulin-like / Major Facilitator Superfamily protein | 128.657 | 42.0444 | -1.61354 | 0.00025 | 0.00253767 |
| AT1G45145 | ATH5, ATTRX5, LIV1, TRX5, thioredoxin H-type 5 | 1928.02 | 630.427 | -1.61272 | 0.0011 | 0.00759303 |
| AT5G07820 | Plant calmodulin-binding protein-related | 32.4623 | 10.6263 | -1.61112 | 0.00065 | 0.00516972 |
| AT4G08250 | GRAS family transcription factor | 4.92292 | 1.61171 | -1.61093 | 0.012 | 0.0450518 |
| AT1G10900 | Phosphatidylinositol-4-phosphate 5-kinase family protein | 44.6163 | 14.609 | -1.61072 | 0.0003 | 0.00292382 |
| AT2G40890 | CYP98A3, cytochrome P450, family 98, subfamily A, polypeptide 3 | 53.1148 | 17.3933 | -1.61059 | 0.0014 | 0.00917155 |
| AT3G47670 | Plant invertase/pectin methylesterase inhibitor superfamily protein | 58.0724 | 19.0232 | -1.61009 | 0.00035 | 0.00327416 |
| AT5G01450 | RING/U-box superfamily protein | 54.1153 | 17.7375 | -1.60924 | 0.0002 | 0.00214445 |
| AT3G52800 | A20/AN1-like zinc finger family protein | 495.607 | 162.456 | -1.60914 | 0.0003 | 0.00292382 |
| AT1G19020 | unknown protein | 472.598 | 155.128 | -1.60715 | 0.0001 | 0.00123775 |
| AT1G20440 | AtCOR47, COR47, RD17, cold-regulated 47 | 2699.7 | 886.206 | -1.60709 | 0.00145 | 0.00940576 |
| AT3G13690 | Protein kinase protein with adenine nucleotide alpha hydrolases-like domain | 19.7687 | 6.49005 | -1.60691 | 0.00025 | 0.00253767 |
| AT5G24290 | Vacuolar iron transporter (VIT) family protein | 92.394 | 30.3502 | -1.60609 | 0.00075 | 0.00575475 |
| AT5G55180 | O-Glycosyl hydrolases family 17 protein | 875.051 | 287.572 | -1.60544 | 0.0008 | 0.00603091 |
| AT1G79970 | unknown protein | 70.4907 | 23.1748 | -1.60487 | 0.0003 | 0.00292382 |
| AT4G39540 | ATSK2, SK2, shikimate kinase 2 | 53.4598 | 17.5885 | -1.60382 | 0.00035 | 0.00327416 |
| AT3G63080 | ATGPX5, GPX5, MEE42, glutathione peroxidase 5 | 69.9564 | 23.021 | -1.60351 | 0.001 | 0.00711095 |
| AT5G15170 | TDP1, tyrosyl-DNA phosphodiesterase-related | 10.3452 | 3.40474 | -1.60335 | 0.00315 | 0.0166977 |
| AT3G62770 | AtATG18a, Transducin/WD40 repeat-like superfamily protein | 202.579 | 66.6879 | -1.60299 | 0.0002 | 0.00214445 |
| AT1G19220 | ARF11, ARF19, IAA22, auxin response factor 19 | 26.3406 | 8.67235 | -1.60279 | 0.0003 | 0.00292382 |
| AT5G10930 | CIPK5, SnRK3.24, CBL-interacting protein kinase 5 | 18.4612 | 6.07835 | -1.60274 | 0.001 | 0.00711095 |
| AT3G15356 | Legume lectin family protein | 262.734 | 86.5204 | -1.60249 | 0.0002 | 0.00214445 |
| AT3G54880 | unknown protein | 287.057 | 94.5844 | -1.60166 | 0.00045 | 0.003919 |
| AT1G03870 | FLA9, FASCICLIN-like arabinoogalactan 9 | 165.099 | 54.4052 | -1.60152 | 0.0006 | 0.00487984 |
| AT4G05070 | Wound-responsive family protein | 530.071 | 174.766 | -1.60076 | 0.00045 | 0.003919 |
| AT2G42310 | unknown protein | 337.244 | 111.192 | -1.60074 | 0.0001 | 0.00123775 |
| AT4G26080 | ABI1, AtABI1, Protein phosphatase 2C family protein | 730.496 | 241.738 | -1.59543 | 0.00235 | 0.0134663 |
| AT3G15210 | ATERF-4, ATERF4, ERF4, RAP2.5, ethylene responsive element binding factor 4 | 307.896 | 101.922 | -1.59498 | 0.00035 | 0.00327416 |
| AT2G46600 | Calcium-binding EF-hand family protein | 254.759 | 84.4076 | -1.59369 | 0.0006 | 0.00487984 |
| AT4G32530 | ATPase, F0/V0 complex, subunit C protein | 266.152 | 88.1837 | -1.59367 | 0.00015 | 0.00170235 |
| AT5G46050 | ATPTR3, PTR3, peptide transporter 3 | 11.2631 | 3.73381 | -1.59288 | 0.00485 | 0.0231612 |
| AT4G20300 | Protein of unknown function (DUF1639) | 61.1596 | 20.2828 | -1.59232 | 0.0003 | 0.00292382 |
| AT5G27920 | F-box family protein | 71.768 | 23.8018 | -1.59227 | 0.00015 | 0.00170235 |
| AT3G61580 | Fatty acid/sphingolipid desaturase | 173.588 | 57.601 | -1.5915 | 0.0004 | 0.00359419 |
| AT4G28770 | Tetraspanin family protein | 162.082 | 53.7877 | -1.59138 | 0.0059 | 0.0268421 |
| AT1G18270 | ketose-bisphosphate aldolase class-II family protein | 57.5176 | 19.0897 | -1.59121 | 0.00025 | 0.00253767 |
| AT3G15115 | unknown protein | 30.0093 | 9.96011 | -1.59118 | 0.00125 | 0.00840292 |
| AT5G07460 | ATMSRA2, PMSR2, peptidemethionine sulfoxide reductase 2 | 777.38 | 258.102 | -1.59068 | 0.00095 | 0.00685384 |
| AT4G23710 | VAG2, VATG2, VHA-G2, vacuolar ATP synthase subunit G2 | 608.332 | 202.057 | -1.5901 | 0.00025 | 0.00253767 |
| AT3G21680 | unknown protein | 1032.79 | 343.065 | -1.58999 | 0.0005 | 0.00424432 |
| AT5G64430 | Octicosapeptide/Phox/Bem1p family protein | 112.044 | 37.22 | -1.58991 | 0.0001 | 0.00123775 |
| AT5G63510 | GAMMA CAL1, gamma carbonic anhydrase like 1 | 167.996 | 55.8076 | -1.58989 | 0.00015 | 0.00170235 |
| AT3G52760 | Integral membrane Yip1 family protein | 23.5866 | 7.83734 | -1.58953 | 0.00345 | 0.0179093 |
| AT5G49680 | KIP, Golgi-body localisation protein domain ;RNA pol II promoter Fmp27 protein domain | 34.1874 | 11.3609 | -1.58939 | 0.00035 | 0.00327416 |
| AT5G40690 | CONTAINS InterPro DOMAIN/s: EF-Hand 1, calcium-binding site (InterPro:IPR018247) | 92.644 | 30.8061 | -1.58848 | 0.00105 | 0.00735817 |
| AT5G41080 | PLC-like phosphodiesterases superfamily protein | 349.839 | 116.471 | -1.58673 | 0.0005 | 0.00424432 |
| AT3G15450 | Aluminium induced protein with YGL and LRDR motifs | 3385.68 | 1127.62 | -1.58617 | 0.0033 | 0.0173075 |
| AT3G11780 | MD-2-related lipid recognition domain-containing protein / ML domain-containing protein | 170.826 | 56.9062 | -1.58587 | 0.0005 | 0.00424432 |
| AT5G58670 | ATPLC, ATPLC1, PLC1, PLC1, phospholipase C1 | 28.4206 | 9.47433 | -1.58484 | 0.0006 | 0.00487984 |
| AT3G22910 | ATPase E1-E2 type family protein / haloacid dehalogenase-like hydrolase family protein | 252.223 | 84.0932 | -1.58464 | 0.0004 | 0.00359419 |
| AT1G22410 | Class-II DAHP synthetase family protein | 358.604 | 119.588 | -1.58431 | 0.0006 | 0.00487984 |
| AT3G50060 | MYB77, myb domain protein 77 | 85.2814 | 28.4441 | -1.5841 | 0.0013 | 0.00867426 |
| AT5G01410 | ATPDX1, ATPDX1.3, PDX1, PDX1.3, RSR4, Aldolase-type TIM barrel family protein | 668.851 | 223.114 | -1.5839 | 0.0005 | 0.00424432 |
| AT4G03520 | ATHM2, Thioredoxin superfamily protein | 156.213 | 52.1189 | -1.58364 | 0.0002 | 0.00214445 |
| AT5G44060 | unknown protein | 187.904 | 62.7145 | -1.58313 | 0.00025 | 0.00253767 |
| AT5G03460 | unknown protein | 305.197 | 101.874 | -1.58296 | 0.0002 | 0.00214445 |
| AT1G77500 | Protein of unknown function (DUF630 and DUF632) | 50.1367 | 16.7365 | -1.58287 | 0.00025 | 0.00253767 |
| AT2G30230 | unknown protein | 73.2051 | 24.444 | -1.58246 | 0.0006 | 0.00487984 |
| AT5G15330 | ATSPX4, SPX4, SPX domain gene 4 | 32.2728 | 10.7847 | -1.58134 | 0.0013 | 0.00867426 |
| AT4G14980 | Cysteine/Histidine-rich C1 domain family protein | 12.0079 | 4.01286 | -1.58128 | 0.00315 | 0.0166977 |
| AT5G04020 | calmodulin binding | 34.8892 | 11.6601 | -1.5812 | 0.0004 | 0.00359419 |
| AT2G37170 | PIP2;2, PIP2B, plasma membrane intrinsic protein 2 | 216.435 | 72.4597 | -1.57868 | 0.0001 | 0.00123775 |
| AT2G38800 | Plant calmodulin-binding protein-related | 85.0766 | 28.4919 | -1.57821 | 0.0002 | 0.00214445 |
| AT5G59490 | Haloacid dehalogenase-like hydrolase (HAD) superfamily protein | 496.028 | 166.133 | -1.57809 | 0.00025 | 0.00253767 |
| AT1G43160 | RAP2.6, related to AP2 6 | 1349.35 | 452.746 | -1.57549 | 0.00125 | 0.00840292 |
| AT3G11330 | PIRL9, plant intracellular ras group-related LRR 9 | 130.341 | 43.7582 | -1.57466 | 0.00025 | 0.00253767 |
| AT5G16600 | AtMYB43, MYB43, myb domain protein 43 | 8.47145 | 2.84493 | -1.57421 | 0.0133 | 0.048507 |
| AT5G65690 | PCK2, PEPCK, phosphoenolpyruvate carboxykinase 2 | 711.095 | 238.883 | -1.57374 | 0.0023 | 0.0132526 |
| AT5G19200 | NAD(P)-binding Rossmann-fold superfamily protein | 24.013 | 8.06916 | -1.57333 | 0.00285 | 0.0154761 |
| AT3G03280 | unknown protein | 139.049 | 46.7287 | -1.57321 | 0.00065 | 0.00516972 |
| AT5G18270 | ANAC087, Arabidopsis NAC domain containing protein 87 | 85.1067 | 28.603 | -1.57311 | 0.00045 | 0.003919 |
| AT4G04840 | ATMSRB6, MSRB6, methionine sulfoxide reductase B6 | 120.257 | 40.4225 | -1.57289 | 0.0004 | 0.00359419 |
| AT5G58620 | zinc finger (CCCH-type) family protein | 30.5813 | 10.2916 | -1.57118 | 0.0005 | 0.00424432 |
| AT3G11410 | AHG3, ATPP2CA, PP2CA, protein phosphatase 2CA | 114.762 | 38.6339 | -1.57071 | 0.00035 | 0.00327416 |
| AT4G36520 | Chaperone DnaJ-domain superfamily protein | 35.8293 | 12.0626 | -1.5706 | 0.0002 | 0.00214445 |
| AT5G64400 | CONTAINS InterPro DOMAIN/s: CHCH (InterPro:IPR010625) | 1193.7 | 401.981 | -1.57023 | 0.0004 | 0.00359419 |
| AT1G76900 | AtTLP1, TLP1, tubby like protein 1 | 72.8638 | 24.5432 | -1.56988 | 0.00025 | 0.00253767 |
| AT3G03160 | function unknown | 176.314 | 59.4173 | -1.56919 | 0.0006 | 0.00487984 |
| AT2G19450 | ABX45, AS11, ATDGAT, DGAT1, RDS1, TAG1, membrane bound O-acyl transferase (MBOAT) family protein | 78.936 | 26.608 | -1.56882 | 0.00035 | 0.00327416 |
| AT3G27540 | beta-1,4-N-acetylglucosaminyltransferase family protein | 8.59467 | 2.89814 | -1.56831 | 0.0022 | 0.0128446 |
| AT1G73340 | Cytochrome P450 superfamily protein | 40.8101 | 13.7892 | -1.56539 | 0.0004 | 0.00359419 |
| AT4G02380 | AtLEA5, SAG21, senescence-associated gene 21 | 1491.37 | 504.116 | -1.5648 | 0.0007 | 0.00547072 |
| AT5G04170 | Calcium-binding EF-hand family protein | 163.288 | 55.2346 | -1.56378 | 0.0005 | 0.00424432 |
| AT1G21930 | unknown protein | 63.0837 | 21.3822 | -1.56086 | 0.00295 | 0.0159014 |
| AT4G29260 | HAD superfamily, subfamily IIIB acid phosphatase | 27.8669 | 9.45206 | -1.55985 | 0.0017 | 0.0106071 |
| AT1G20110 | RING/FYVE/PHD zinc finger superfamily protein | 198.756 | 67.4318 | -1.5595 | 0.0005 | 0.00424432 |
| AT1G51140 | basic helix-loop-helix (bHLH) DNA-binding superfamily protein | 48.6661 | 16.5139 | -1.55924 | 0.00035 | 0.00327416 |
| AT4G27652 | unknown protein | 566.795 | 192.382 | -1.55885 | 0.00025 | 0.00253767 |
| AT1G11800 | endonuclease/exonuclease/phosphatase family protein | 10.307 | 3.50259 | -1.55712 | 0.0035 | 0.0180741 |
| AT2G33320 | Calcium-dependent lipid-binding (CaLB domain) family protein | 9.96399 | 3.38615 | -1.55708 | 0.0012 | 0.00815259 |
| AT5G11790 | NDL2, N-MYC downregulated-like 2 | 94.9003 | 32.2797 | -1.55579 | 0.0005 | 0.00424432 |
| AT5G64552 | CPuORF22, conserved peptide upstream open reading frame 22 | 4.98945 | 1.69732 | -1.55562 | 0.00675 | 0.0295876 |
| AT3G46640 | LUX, PCL1, Homeodomain-like superfamily protein | 160.418 | 54.5747 | -1.55553 | 0.0004 | 0.00359419 |
| AT1G08190 | ATVAM2, ATVPS41, VAM2, VPS41, ZIP2, vacuolar protein sorting 41 | 93.7789 | 31.9353 | -1.55411 | 0.0008 | 0.00603091 |
| AT2G43850 | Integrin-linked protein kinase family | 8.51246 | 2.90163 | -1.55271 | 0.00905 | 0.0366741 |
| AT1G35580 | CINV1, cytosolic invertase 1 | 423.926 | 144.599 | -1.55176 | 0.0006 | 0.00487984 |
| AT5G19550 | AAT2, ASP2, aspartate aminotransferase 2 | 385.198 | 131.399 | -1.55164 | 0.0004 | 0.00359419 |
| AT2G14750 | AKN1, APK, APK1, ATAKN1, APS kinase | 86.0834 | 29.3909 | -1.55036 | 0.0008 | 0.00603091 |
| AT5G22940 | F8H, FRA8 homolog | 8.3148 | 2.8421 | -1.54872 | 0.0036 | 0.0184414 |
| AT5G15870 | glycosyl hydrolase family 81 protein | 209.111 | 71.5251 | -1.54775 | 0.0004 | 0.00359419 |
| AT5G56550 | ATOXS3, OXS3, oxidative stress 3 | 273.422 | 93.5739 | -1.54695 | 0.00065 | 0.00516972 |
| AT2G02970 | GDA1/CD39 nucleoside phosphatase family protein | 61.9371 | 21.1999 | -1.54674 | 0.0005 | 0.00424432 |
| AT3G28710 | ATPase, V0/A0 complex, subunit C/D | 247.945 | 84.908 | -1.54605 | 0.0008 | 0.00603091 |
| AT3G56440 | ATATG18D, ATG18D, homolog of yeast autophagy 18 (ATG18) D | 26.0424 | 8.91905 | -1.5459 | 0.00065 | 0.00516972 |
| AT3G54930 | Protein phosphatase 2A regulatory B subunit family protein | 4.82926 | 1.65563 | -1.54442 | 0.00715 | 0.0308561 |
| AT1G19450 | Major facilitator superfamily protein | 174.887 | 59.9677 | -1.54417 | 0.00055 | 0.00457655 |
| AT3G26240 | Cysteine/Histidine-rich C1 domain family protein | 3.43686 | 1.17964 | -1.54275 | 0.00755 | 0.0321272 |
| AT3G44310 | ATNIT1, NIT1, NITI, nitrilase 1 | 16.4418 | 5.64391 | -1.5426 | 0.0054 | 0.0251864 |
| AT4G30010 | unknown protein | 1013.58 | 348.027 | -1.54219 | 0.0006 | 0.00487984 |
| AT4G37370 | CYP81D8, cytochrome P450, family 81, subfamily D, polypeptide 8 | 10.5756 | 3.63136 | -1.54216 | 0.0024 | 0.0136815 |
| AT3G30390 | Transmembrane amino acid transporter family protein | 259.037 | 89.0239 | -1.5409 | 0.00045 | 0.003919 |
| AT3G53410 | RING/U-box superfamily protein | 18.7754 | 6.45322 | -1.54075 | 0.00345 | 0.0179093 |
| AT2G36890 | ATMYB38, BIT1, MYB38, RAX2, Duplicated homeodomain-like superfamily protein | 55.0995 | 18.9423 | -1.54043 | 0.0004 | 0.00359419 |
| AT4G34230 | ATCAD5, CAD-5, CAD5, cinnamyl alcohol dehydrogenase 5 | 258.739 | 89.1349 | -1.53744 | 0.00035 | 0.00327416 |
| AT3G25655 | IDL1, inflorescence deficient in abscission (IDA)-like 1 | 56.6149 | 19.5074 | -1.53716 | 0.00575 | 0.0263656 |
| AT5G12170 | CLT3, CRT (chloroquine-resistance transporter)-like transporter 3 | 14.2251 | 4.90272 | -1.53679 | 0.0043 | 0.0211046 |
| AT4G13510 | AMT1;1, ATAMT1, ATAMT1;1, ammonium transporter 1;1 | 220.847 | 76.1917 | -1.53534 | 0.00065 | 0.00516972 |
| AT5G01850 | Protein kinase superfamily protein | 12.4522 | 4.30297 | -1.533 | 0.004 | 0.0200243 |
| AT3G51250 | Senescence/dehydration-associated protein-related | 31.5825 | 10.9138 | -1.53297 | 0.00055 | 0.00457655 |
| AT1G71000 | Chaperone DnaJ-domain superfamily protein | 277.718 | 95.9805 | -1.53281 | 0.0026 | 0.0145003 |
| AT1G44170 | ALDH3H1, ALDH4, aldehyde dehydrogenase 3H1 | 64.6943 | 22.3794 | -1.53147 | 0.0012 | 0.00815259 |
| AT5G59613 | unknown protein | 560.233 | 193.887 | -1.53081 | 0.0003 | 0.00292382 |
| AT4G21160 | AGD12, ZAC, Calcium-dependent ARF-type GTPase activating protein family | 83.2264 | 28.8137 | -1.53029 | 0.00105 | 0.00735817 |
| AT4G38810 | Calcium-binding EF-hand family protein | 447.283 | 155.03 | -1.52865 | 0.00065 | 0.00516972 |
| AT5G47740 | Adenine nucleotide alpha hydrolases-like superfamily protein | 113.395 | 39.317 | -1.52813 | 0.00515 | 0.024295 |
| AT2G20750 | ATEXPB1, ATHEXP BETA 1.5, EXPB1, expansin B1 | 20.3547 | 7.06312 | -1.52699 | 0.0069 | 0.0300655 |
| AT2G35710 | Nucleotide-diphospho-sugar transferases superfamily protein | 33.1764 | 11.5141 | -1.52675 | 0.00205 | 0.012221 |
| AT4G34410 | RRTF1, redox responsive transcription factor 1 | 193.39 | 67.1881 | -1.52524 | 0.00095 | 0.00685384 |
| AT1G68820 | Transmembrane Fragile-X-F-associated protein | 63.7703 | 22.159 | -1.52499 | 0.0004 | 0.00359419 |
| AT4G36500 | unknown protein | 1344.94 | 467.384 | -1.52486 | 0.00135 | 0.0089118 |
| AT1G49300 | ATRAB7, ATRABG3E, RABG3E, RAB GTPase homolog G3E | 170.033 | 59.0985 | -1.52462 | 0.0003 | 0.00292382 |
| AT1G19025 | DNA repair metallo-beta-lactamase family protein | 43.1803 | 15.0099 | -1.52446 | 0.0014 | 0.00917155 |
| AT3G11700 | FLA18, FASCICLIN-like arabinogalactan protein 18 precursor | 107.844 | 37.5015 | -1.52393 | 0.00045 | 0.003919 |
| AT1G68300 | Adenine nucleotide alpha hydrolases-like superfamily protein | 124.142 | 43.2172 | -1.52231 | 0.0045 | 0.0218802 |
| AT5G46890 | Bifunctional inhibitor/lipid-transfer protein/seed storage 2S albumin superfamily protein | 202.875 | 70.645 | -1.52193 | 0.00155 | 0.009918 |
| AT4G02120 | CTP synthase family protein | 23.1412 | 8.06052 | -1.52152 | 0.00095 | 0.00685384 |
| AT3G56950 | SIP2, SIP2;1, small and basic intrinsic protein 2;1 | 61.1554 | 21.3058 | -1.52124 | 0.0037 | 0.0188077 |
| AT5G07920 | ATDGK1, DGK1, diacylglycerol kinase1 | 49.2252 | 17.1494 | -1.52123 | 0.0004 | 0.00359419 |
| AT5G25265 | unknown protein | 100.183 | 34.9042 | -1.52117 | 0.00035 | 0.00327416 |
| AT2G45670 | calcineurin B subunit-related | 187.439 | 65.3763 | -1.51958 | 0.0011 | 0.00759303 |
| AT2G39950 | unknown protein | 63.0984 | 22.0124 | -1.51929 | 0.00065 | 0.00516972 |
| AT2G29430 | Family of unknown function (DUF572) | 112.114 | 39.1246 | -1.51882 | 0.00255 | 0.014306 |
| AT5G22300 | AtNIT4, NIT4, nitrilase 4 | 9.08484 | 3.17038 | -1.5188 | 0.0108 | 0.041652 |
| AT1G01440 | Protein of unknown function (DUF3133) | 18.0843 | 6.3126 | -1.51843 | 0.00165 | 0.0103801 |
| AT5G55060 | unknown protein | 45.3203 | 15.8217 | -1.51825 | 0.0008 | 0.00603091 |
| AT4G23885 | unknown protein | 271.443 | 94.7762 | -1.51805 | 0.0008 | 0.00603091 |
| AT1G66260 | RNA-binding (RRM/RBD/RNP motifs) family protein | 413.437 | 144.388 | -1.51772 | 0.0008 | 0.00603091 |
| AT3G20340 | Expression of the gene is downregulated in the presence of paraquat, an inducer of photoxidative stress. | 434.051 | 151.657 | -1.51705 | 0.001 | 0.00711095 |
| AT2G39530 | Uncharacterised protein family (UPF0497) | 97.5468 | 34.0835 | -1.51702 | 0.00205 | 0.012221 |
| AT4G36040 | Chaperone DnaJ-domain superfamily protein | 1082.74 | 378.673 | -1.51567 | 0.00205 | 0.012221 |
| AT3G18950 | Transducin/WD40 repeat-like superfamily protein | 146.545 | 51.2526 | -1.51565 | 0.0006 | 0.00487984 |
| AT5G66400 | ATDI8, RAB18, Dehydrin family protein | 108.849 | 38.0731 | -1.51548 | 0.00145 | 0.00940576 |
| AT3G17710 | F-box and associated interaction domains-containing protein | 21.2893 | 7.45126 | -1.51457 | 0.0019 | 0.0115387 |
| AT1G73920 | alpha/beta-Hydrolases superfamily protein | 327.045 | 114.536 | -1.51369 | 0.00085 | 0.00630464 |
| AT3G48360 | ATBT2, BT2, BTB and TAZ domain protein 2 | 72.2546 | 25.3099 | -1.51339 | 0.00145 | 0.00940576 |
| AT5G49360 | ATBXL1, BXL1, beta-xylosidase 1 | 9.19674 | 3.22218 | -1.51308 | 0.0048 | 0.0229834 |
| AT1G18210 | Calcium-binding EF-hand family protein | 279.4 | 97.969 | -1.51194 | 0.00125 | 0.00840292 |
| AT2G16380 | Sec14p-like phosphatidylinositol transfer family protein | 7.83655 | 2.74814 | -1.51176 | 0.00695 | 0.0302036 |
| AT1G03090 | MCCA, methylcrotonyl-CoA carboxylase alpha chain, mitochondrial / 3-methylcrotonyl-CoA carboxylase 1 (MCCA) | 115.017 | 40.3469 | -1.51132 | 0.00135 | 0.0089118 |
| AT3G24180 | Beta-glucosidase, GBA2 type family protein | 176.934 | 62.0748 | -1.51113 | 0.0005 | 0.00424432 |
| AT4G03460 | Ankyrin repeat family protein | 6.9382 | 2.437 | -1.50945 | 0.00735 | 0.0315405 |
| AT5G04770 | ATCAT6, CAT6, cationic amino acid transporter 6 | 56.1107 | 19.7316 | -1.50777 | 0.0016 | 0.0101494 |
| AT5G64370 | BETA-UP, PYD3, beta-ureidopropionase | 114.793 | 40.408 | -1.50632 | 0.0005 | 0.00424432 |
| AT5G59960 | unknown protein | 79.6069 | 28.0324 | -1.5058 | 0.00025 | 0.00253767 |
| AT2G36310 | URH1, uridine-ribohydrolase 1 | 316.913 | 111.615 | -1.50555 | 0.00045 | 0.003919 |
| AT4G35500 | Protein kinase superfamily protein | 49.6345 | 17.482 | -1.50547 | 0.00085 | 0.00630464 |
| AT3G10660 | ATCPK2, CPK2, calmodulin-domain protein kinase cdpk isoform 2 | 41.8031 | 14.7242 | -1.50542 | 0.0007 | 0.00547072 |
| AT2G33040 | ATP3, gamma subunit of Mt ATP synthase | 796.22 | 280.518 | -1.50507 | 0.00255 | 0.014306 |
| AT2G35980 | ATNHL10, NHL10, YLS9, Late embryogenesis abundant (LEA) hydroxyproline-rich glycoprotein family | 107.34 | 37.8361 | -1.50436 | 0.0009 | 0.0065867 |
| AT4G28550 | Ypt/Rab-GAP domain of gyp1p superfamily protein | 20.7639 | 7.32025 | -1.50411 | 0.0028 | 0.0152969 |
| AT1G63840 | RING/U-box superfamily protein | 159.136 | 56.144 | -1.50306 | 0.00065 | 0.00516972 |
| AT5G17280 | CONTAINS InterPro DOMAIN/s: Oxidoreductase-like, N-terminal (InterPro:IPR019180) | 87.1239 | 30.7571 | -1.50215 | 0.0018 | 0.0110912 |
| AT3G07350 | Protein of unknown function (DUF506) | 83.8947 | 29.6186 | -1.50207 | 0.0009 | 0.0065867 |
| AT2G28430 | unknown protein | 77.1847 | 27.268 | -1.50111 | 0.0042 | 0.0207631 |
| AT2G42270 | U5 small nuclear ribonucleoprotein helicase | 72.8414 | 25.7423 | -1.50062 | 0.001 | 0.00711095 |
| AT1G52760 | LysoPL2, lysophospholipase 2 | 138.289 | 48.888 | -1.50013 | 0.0006 | 0.00487984 |
| AT2G16980 | Major facilitator superfamily protein | 8.17464 | 2.89167 | -1.49925 | 0.0111 | 0.0425192 |
| AT1G62300 | ATWRKY6, WRKY6, WRKY family transcription factor | 188.38 | 66.6406 | -1.49917 | 0.0006 | 0.00487984 |
| AT5G54300 | Protein of unknown function (DUF761) | 102.494 | 36.2608 | -1.49906 | 0.00075 | 0.00575475 |
| AT5G17190 | function unknown | 128.571 | 45.4935 | -1.49883 | 0.001 | 0.00711095 |
| AT3G01650 | RGLG1, RING domain ligase1 | 116.495 | 41.2241 | -1.49871 | 0.00075 | 0.00575475 |
| AT1G70490 | ARFA1D, ATARFA1D, Ras-related small GTP-binding family protein | 594.299 | 210.374 | -1.49823 | 0.00125 | 0.00840292 |
| AT4G34490 | ATCAP1, CAP 1, CAP1, cyclase associated protein 1 | 167.472 | 59.2951 | -1.49793 | 0.0014 | 0.00917155 |
| AT1G10050 | glycosyl hydrolase family 10 protein / carbohydrate-binding domain-containing protein | 62.7757 | 22.2312 | -1.49762 | 0.0004 | 0.00359419 |
| AT2G26260 | 3BETAHSD/D2, AT3BETAHSD/D2, 3beta-hydroxysteroid-dehydrogenase/decarboxylase isoform 2 | 26.2137 | 9.2883 | -1.49683 | 0.0016 | 0.0101494 |
| AT2G01680 | Ankyrin repeat family protein | 50.8468 | 18.0227 | -1.49634 | 0.0008 | 0.00603091 |
| AT1G20260 | ATPase, V1 complex, subunit B protein | 515.591 | 182.769 | -1.49621 | 0.0016 | 0.0101494 |
| AT5G03290 | IDH-V, isocitrate dehydrogenase V | 301.535 | 106.941 | -1.49552 | 0.0009 | 0.0065867 |
| AT2G21850 | Cysteine/Histidine-rich C1 domain family protein | 19.14 | 6.79004 | -1.4951 | 0.00215 | 0.0126571 |
| AT4G22910 | CCS52A1, FZR2, FIZZY-related 2 | 21.9113 | 7.7863 | -1.49266 | 0.00435 | 0.0212815 |
| AT1G66500 | Pre-mRNA cleavage complex II | 134.628 | 47.8711 | -1.49175 | 0.00125 | 0.00840292 |
| AT3G59710 | NAD(P)-binding Rossmann-fold superfamily protein | 17.1201 | 6.08803 | -1.49165 | 0.00435 | 0.0212815 |
| AT1G67570 | Protein of unknown function (DUF3537) | 13.0727 | 4.64875 | -1.49164 | 0.004 | 0.0200243 |
| AT4G26130 | unknown protein | 8.89271 | 3.16552 | -1.49018 | 0.0108 | 0.041652 |
| AT1G54320 | LEM3 (ligand-effect modulator 3) family protein / CDC50 family protein | 162.565 | 57.8693 | -1.49014 | 0.0009 | 0.0065867 |
| AT3G50280 | HXXXD-type acyl-transferase family protein | 6.97949 | 2.48473 | -1.49003 | 0.00915 | 0.0369056 |
| AT1G56300 | Chaperone DnaJ-domain superfamily protein | 34.5954 | 12.3162 | -1.49002 | 0.0035 | 0.0180741 |
| AT4G32070 | Octicosapeptide/Phox/Bem1p (PB1) domain-containing protein / tetratricopeptide repeat (TPR)-containing protein | 189.746 | 67.5944 | -1.48909 | 0.00035 | 0.00327416 |
| AT3G15200 | Tetratricopeptide repeat (TPR)-like superfamily protein | 57.6436 | 20.5358 | -1.48902 | 0.0049 | 0.0233549 |
| AT4G09570 | ATCPK4, CPK4, calcium-dependent protein kinase 4 | 227.361 | 81.021 | -1.48862 | 0.0005 | 0.00424432 |
| AT5G54470 | B-box type zinc finger family protein | 39.1342 | 13.9481 | -1.48837 | 0.00235 | 0.0134663 |
| AT5G57830 | Protein of unknown function, DUF593 | 154.744 | 55.1579 | -1.48825 | 0.00105 | 0.00735817 |
| AT2G02310 | AtPP2-B6, PP2-B6, phloem protein 2-B6 | 37.3252 | 13.3183 | -1.48674 | 0.003 | 0.0161227 |
| AT4G02370 | Protein of unknown function, DUF538 | 107.765 | 38.4737 | -1.48595 | 0.00085 | 0.00630464 |
| AT3G02460 | Ypt/Rab-GAP domain of gyp1p superfamily protein | 21.8039 | 7.78703 | -1.48544 | 0.00235 | 0.0134663 |
| AT3G51770 | ATEOL1, ETO1, tetratricopeptide repeat (TPR)-containing protein | 54.9882 | 19.6388 | -1.48541 | 0.00075 | 0.00575475 |
| AT5G24110 | ATWRKY30, WRKY30, WRKY DNA-binding protein 30 | 31.0568 | 11.0955 | -1.48494 | 0.0053 | 0.0248135 |
| AT4G35320 | unknown protein | 177.516 | 63.4578 | -1.48407 | 0.00035 | 0.00327416 |
| AT3G54130 | Josephin family protein | 308.151 | 110.16 | -1.48404 | 0.0004 | 0.00359419 |
| AT1G47740 | PPPDE putative thiol peptidase family protein | 72.1497 | 25.7954 | -1.48388 | 0.0042 | 0.0207631 |
| AT3G24070 | Zinc knuckle (CCHC-type) family protein | 80.1151 | 28.6435 | -1.48387 | 0.00075 | 0.00575475 |
| AT1G80300 | ATNTT1, NTT1, nucleotide transporter 1 | 23.7549 | 8.4935 | -1.48379 | 0.0017 | 0.0106071 |
| AT5G15320 | unknown protein | 381.752 | 136.535 | -1.48336 | 0.0006 | 0.00487984 |
| AT3G04080 | APY1, ATAPY1, apyrase 1 | 44.454 | 15.9001 | -1.48328 | 0.0014 | 0.00917155 |
| AT5G06390 | FLA17, FASCICLIN-like arabinogalactan protein 17 precursor | 46.8757 | 16.7675 | -1.48317 | 0.00085 | 0.00630464 |
| AT5G03190 | CPUORF47, conserved peptide upstream open reading frame 47 | 42.9977 | 15.3871 | -1.48254 | 0.0015 | 0.00967937 |
| AT4G36380 | ROT3, Cytochrome P450 superfamily protein | 5.77683 | 2.06879 | -1.48149 | 0.00415 | 0.020593 |
| AT5G55290 | ATPase, V0 complex, subunit E | 252.328 | 90.4295 | -1.48043 | 0.0013 | 0.00867426 |
| AT4G24960 | ATHVA22D, HVA22D, HVA22 homologue D | 1190.49 | 426.809 | -1.4799 | 0.00145 | 0.00940576 |
| AT3G26040 | HXXXD-type acyl-transferase family protein | 12.1016 | 4.33962 | -1.47955 | 0.00375 | 0.0189985 |
| AT4G29480 | Mitochondrial ATP synthase subunit G protein | 460.914 | 165.352 | -1.47896 | 0.00085 | 0.00630464 |
| AT3G27190 | UKL2, uridine kinase-like 2 | 76.997 | 27.6417 | -1.47795 | 0.0007 | 0.00547072 |
| AT3G15620 | UVR3, DNA photolyase family protein | 10.0675 | 3.61663 | -1.47699 | 0.00595 | 0.0269704 |
| AT3G17780 | function unknown | 254.07 | 91.3394 | -1.47592 | 0.00055 | 0.00457655 |
| AT5G40390 | SIP1, Raffinose synthase family protein | 330.234 | 118.722 | -1.4759 | 0.00225 | 0.0130442 |
| AT3G27100 | function unknown | 33.5851 | 12.078 | -1.47544 | 0.0058 | 0.0265396 |
| AT3G06500 | Plant neutral invertase family protein | 637.654 | 229.393 | -1.47495 | 0.007 | 0.0303609 |
| AT4G35790 | ATPLDDELTA, PLDDELTA, phospholipase D delta | 154.161 | 55.5105 | -1.47361 | 0.00155 | 0.009918 |
| AT5G04860 | unknown protein | 57.9319 | 20.8617 | -1.4735 | 0.00085 | 0.00630464 |
| AT3G15640 | Rubredoxin-like superfamily protein | 231.597 | 83.4077 | -1.47337 | 0.00095 | 0.00685384 |
| AT3G60690 | SAUR-like auxin-responsive protein family | 75.3346 | 27.1516 | -1.47228 | 0.00195 | 0.0117451 |
| AT3G11850 | Protein of unknown function, DUF593 | 14.3726 | 5.18043 | -1.47218 | 0.00435 | 0.0212815 |
| AT5G10650 | RING/U-box superfamily protein | 62.7646 | 22.6341 | -1.47145 | 0.0004 | 0.00359419 |
| AT1G76185 | unknown protein | 46.3039 | 16.7009 | -1.47121 | 0.0027 | 0.0149318 |
| AT3G60200 | unknown protein | 45.9345 | 16.5708 | -1.47094 | 0.00145 | 0.00940576 |
| AT1G66600 | ABO3, ATWRKY63, WRKY63, ABA overly sensitive mutant 3 | 27.7772 | 10.0215 | -1.4708 | 0.00785 | 0.0330699 |
| AT3G47980 | Integral membrane HPP family protein | 13.7067 | 4.94974 | -1.46945 | 0.00955 | 0.0380846 |
| AT3G14395 | unknown protein | 40.2402 | 14.5332 | -1.46929 | 0.0058 | 0.0265396 |
| AT1G30700 | FAD-binding Berberine family protein | 432.923 | 156.433 | -1.46856 | 0.00155 | 0.009918 |
| AT5G56340 | ATCRT1, RING/U-box superfamily protein | 142.462 | 51.493 | -1.46813 | 0.00055 | 0.00457655 |
| AT1G58110 | Basic-leucine zipper (bZIP) transcription factor family protein | 41.7993 | 15.1132 | -1.46766 | 0.0007 | 0.00547072 |
| AT5G61228 | CPuORF15, conserved peptide upstream open reading frame 15 | 122.494 | 44.2993 | -1.46736 | 0.0005 | 0.00424432 |
| AT1G78900 | VHA-A, vacuolar ATP synthase subunit A | 972.528 | 351.844 | -1.4668 | 0.012 | 0.0450518 |
| AT4G20780 | CML42, calmodulin like 42 | 127.502 | 46.141 | -1.46639 | 0.0012 | 0.00815259 |
| AT4G36750 | Quinone reductase family protein | 87.8109 | 31.7811 | -1.46623 | 0.00075 | 0.00575475 |
| AT4G16100 | Protein of unknown function (DUF789) | 26.1483 | 9.46691 | -1.46575 | 0.0028 | 0.0152969 |
| AT1G05300 | ZIP5, zinc transporter 5 precursor | 43.5397 | 15.7696 | -1.46519 | 0.00185 | 0.0113253 |
| AT2G40970 | MYBC1, Homeodomain-like superfamily protein | 48.9457 | 17.7576 | -1.46274 | 0.00125 | 0.00840292 |
| AT1G27200 | Domain of unknown function (DUF23) | 46.2442 | 16.8087 | -1.46006 | 0.0008 | 0.00603091 |
| AT4G30260 | Integral membrane Yip1 family protein | 139.373 | 50.6832 | -1.45937 | 0.00065 | 0.00516972 |
| AT1G66880 | Protein kinase superfamily protein | 28.1356 | 10.2333 | -1.45913 | 0.00075 | 0.00575475 |
| AT2G44840 | ATERF13, EREBP, ERF13, ethylene-responsive element binding factor 13 | 184.521 | 67.1459 | -1.45841 | 0.0007 | 0.00547072 |
| AT2G03340 | WRKY3, WRKY DNA-binding protein 3 | 49.7524 | 18.1149 | -1.45759 | 0.0013 | 0.00867426 |
| AT5G19855 | Chaperonin-like RbcX protein | 27.8964 | 10.158 | -1.45746 | 0.0032 | 0.0169175 |
| AT5G01820 | ATCIPK14, ATSR1, CIPK14, SnRK3.15, SR1, serine/threonine protein kinase 1 | 383.094 | 139.568 | -1.45673 | 0.0015 | 0.00967937 |
| AT5G52290 | SHOC1, shortage in chiasmata 1 | 1.7618 | 0.641892 | -1.45664 | 0.0103 | 0.0403045 |
| AT3G06780 | glycine-rich protein | 105.24 | 38.3617 | -1.45594 | 0.0032 | 0.0169175 |
| AT3G16240 | AQP1,delta tonoplast integral protein | 916.7 | 334.163 | -1.4559 | 0.0017 | 0.0106071 |
| AT1G80570 | RNI-like superfamily protein | 10.4467 | 3.80956 | -1.45535 | 0.0053 | 0.0248135 |
| AT5G51570 | SPFH/Band 7/PHB domain-containing membrane-associated protein family | 96.5853 | 35.2336 | -1.45485 | 0.00065 | 0.00516972 |
| AT2G27690 | CYP94C1, cytochrome P450, family 94, subfamily C, polypeptide 1 | 60.1014 | 21.9249 | -1.45483 | 0.0007 | 0.00547072 |
| AT4G38900 | Basic-leucine zipper (bZIP) transcription factor family protein | 105.813 | 38.6376 | -1.45344 | 0.00105 | 0.00735817 |
| AT5G19900 | PRLI-interacting factor, putative | 288.273 | 105.305 | -1.45286 | 0.0009 | 0.0065867 |
| AT1G58520 | RXW8, lipases;hydrolases, acting on ester bonds | 54.7524 | 20.0112 | -1.45211 | 0.001 | 0.00711095 |
| AT2G20010 | Protein of unknown function (DUF810) | 54.4102 | 19.893 | -1.45161 | 0.00075 | 0.00575475 |
| AT5G42250 | Zinc-binding alcohol dehydrogenase family protein | 53.901 | 19.7122 | -1.45122 | 0.00175 | 0.0108609 |
| AT3G08580 | AAC1, ADP/ATP carrier 1 | 1968.22 | 719.857 | -1.45111 | 0.0037 | 0.0188077 |
| AT1G28200 | FIP1, FH interacting protein 1 | 218.519 | 79.9274 | -1.45099 | 0.00075 | 0.00575475 |
| AT3G12510 | MADS-box family protein | 1085.59 | 397.172 | -1.45064 | 0.0007 | 0.00547072 |
| AT5G54780 | Ypt/Rab-GAP domain of gyp1p superfamily protein | 68.1195 | 24.939 | -1.44967 | 0.0009 | 0.0065867 |
| AT2G02510 | NADH dehydrogenase (ubiquinone)s | 375.097 | 137.345 | -1.44946 | 0.00075 | 0.00575475 |
| AT5G64660 | ATCMPG2, CMPG2, CYS, MET, PRO, and GLY protein 2 | 56.3152 | 20.6285 | -1.44889 | 0.00105 | 0.00735817 |
| AT2G32390 | ATGLR3.5, GLR3.5, GLR6, glutamate receptor 3.5 | 24.4592 | 8.96156 | -1.44856 | 0.0014 | 0.00917155 |
| AT4G26890 | MAPKKK16, mitogen-activated protein kinase kinase kinase 16 | 16.4378 | 6.02283 | -1.44851 | 0.0036 | 0.0184414 |
| AT1G27170 | transmembrane receptors;ATP binding | 2.6846 | 0.983701 | -1.44841 | 0.008 | 0.0335306 |
| AT4G10810 | unknown protein | 92.0803 | 33.7553 | -1.44778 | 0.0036 | 0.0184414 |
| AT1G61360 | S-locus lectin protein kinase family protein | 47.7828 | 17.5205 | -1.44745 | 0.00065 | 0.00516972 |
| AT3G03720 | CAT4, cationic amino acid transporter 4 | 43.2814 | 15.8769 | -1.44682 | 0.0017 | 0.0106071 |
| AT2G36895 | unknown protein | 93.6666 | 34.3901 | -1.44554 | 0.002 | 0.0119879 |
| AT1G75750 | GASA1, GAST1 protein homolog 1 | 191.877 | 70.459 | -1.44532 | 0.0015 | 0.00967937 |
| AT5G08060 | unknown protein | 197.224 | 72.4427 | -1.44493 | 0.0007 | 0.00547072 |
| AT1G12310 | Calcium-binding EF-hand family protein | 138.584 | 50.9035 | -1.44493 | 0.00115 | 0.00787231 |
| AT4G33985 | Protein of unknown function (DUF1685) | 54.9466 | 20.2031 | -1.44346 | 0.00715 | 0.0308561 |
| AT5G66070 | RING/U-box superfamily protein | 215.533 | 79.2505 | -1.44342 | 0.00105 | 0.00735817 |
| AT5G60120 | TOE2, target of early activation tagged (EAT) 2 | 21.5244 | 7.9194 | -1.44251 | 0.0036 | 0.0184414 |
| AT3G15430 | Regulator of chromosome condensation (RCC1) family protein | 109.051 | 40.1306 | -1.44222 | 0.0005 | 0.00424432 |
| AT2G32260 | ATCCT1, CCT1, phosphorylcholine cytidylyltransferase | 81.8792 | 30.137 | -1.44196 | 0.0009 | 0.0065867 |
| AT3G57400 | unknown protein | 53.2336 | 19.5987 | -1.44158 | 0.0012 | 0.00815259 |
| AT3G57480 | zinc finger (C2H2 type, AN1-like) family protein | 45.8663 | 16.8966 | -1.4407 | 0.00135 | 0.0089118 |
| AT3G56790 | RNA splicing factor-related | 25.6723 | 9.45787 | -1.44063 | 0.0101 | 0.039726 |
| AT3G55940 | Phosphoinositide-specific phospholipase C family protein | 38.9852 | 14.3651 | -1.44036 | 0.0013 | 0.00867426 |
| AT1G71090 | Auxin efflux carrier family protein | 28.5045 | 10.5134 | -1.43896 | 0.00175 | 0.0108609 |
| AT3G07890 | Ypt/Rab-GAP domain of gyp1p superfamily protein | 119.607 | 44.1154 | -1.43895 | 0.00075 | 0.00575475 |
| AT4G06676 | CONTAINS InterPro DOMAIN/s: Etoposide-induced 2.4 (InterPro:IPR009890) | 31.5605 | 11.6444 | -1.43849 | 0.00145 | 0.00940576 |
| AT3G16800 | Protein phosphatase 2C family protein | 119.3 | 44.0168 | -1.43847 | 0.0009 | 0.0065867 |
| AT5G24470 | APRR5, PRR5, pseudo-response regulator 5 | 35.1893 | 12.9871 | -1.43806 | 0.00155 | 0.009918 |
| AT5G62630 | HIPL2, hipl2 protein precursor | 6.78242 | 2.50346 | -1.43787 | 0.00795 | 0.0333705 |
| AT5G58020 | unknown protein | 208.417 | 76.9897 | -1.43674 | 0.00345 | 0.0179093 |
| AT5G08730 | ARI16, ATARI16, IBR domain-containing protein | 5.27758 | 1.9497 | -1.43663 | 0.01195 | 0.0449578 |
| AT5G66820 | unknown protein | 13.5814 | 5.01974 | -1.43595 | 0.00555 | 0.0256744 |
| AT1G72770 | HAB1, homology to ABI1 | 99.8724 | 36.9211 | -1.43564 | 0.00115 | 0.00787231 |
| AT5G65207 | unknown protein | 3333.24 | 1232.51 | -1.43533 | 0.0023 | 0.0132526 |
| AT3G49570 | LSU3, response to low sulfur 3 | 190.802 | 70.5889 | -1.43457 | 0.0025 | 0.0140894 |
| AT5G07070 | CIPK2, SnRK3.2, CBL-interacting protein kinase 2 | 59.874 | 22.1559 | -1.43424 | 0.0013 | 0.00867426 |
| AT2G43490 | Ypt/Rab-GAP domain of gyp1p superfamily protein | 44.0976 | 16.3244 | -1.43367 | 0.00205 | 0.012221 |
| AT1G61770 | Chaperone DnaJ-domain superfamily protein | 173.696 | 64.3107 | -1.43344 | 0.0009 | 0.0065867 |
| AT3G48680 | GAMMA CAL2, gamma carbonic anhydrase-like 2 | 92.8943 | 34.4114 | -1.4327 | 0.0017 | 0.0106071 |
| AT1G21660 | Chaperone DnaJ-domain superfamily protein | 93.6772 | 34.708 | -1.43243 | 0.00075 | 0.00575475 |
| AT1G17620 | Late embryogenesis abundant (LEA) hydroxyproline-rich glycoprotein family | 380.029 | 140.832 | -1.43213 | 0.00095 | 0.00685384 |
| AT3G24160 | PMP, putative type 1 membrane protein | 464.094 | 172.006 | -1.43196 | 0.0025 | 0.0140894 |
| AT4G31980 | unknown protein | 8.67979 | 3.2177 | -1.43163 | 0.00515 | 0.024295 |
| AT5G15650 | ATRGP2, RGP2, reversibly glycosylated polypeptide 2 | 1568.93 | 581.897 | -1.43094 | 0.00395 | 0.0198341 |
| AT1G25550 | myb-like transcription factor family protein | 159.219 | 59.0713 | -1.43048 | 0.0011 | 0.00759303 |
| AT3G16530 | Legume lectin family protein | 19.1794 | 7.11834 | -1.42994 | 0.0055 | 0.0254967 |
| AT2G33290 | ATSUVH2, SDG3, SUVH2, SU(VAR)3-9 homolog 2 | 7.71558 | 2.8643 | -1.4296 | 0.0063 | 0.0281701 |
| AT4G32060 | calcium-binding EF hand family protein | 287.227 | 106.634 | -1.42952 | 0.0017 | 0.0106071 |
| AT4G08180 | ORP1C, OSBP(oxysterol binding protein)-related protein 1C | 52.3103 | 19.4261 | -1.4291 | 0.0008 | 0.00603091 |
| AT4G23640 | ATKT3, KUP4, TRH1, Potassium transporter family protein | 35.4945 | 13.1821 | -1.42901 | 0.0011 | 0.00759303 |
| AT1G06570 | HPD, PDS1, phytoene desaturation 1 | 152.351 | 56.6254 | -1.42788 | 0.00065 | 0.00516972 |
| AT4G30440 | GAE1, UDP-D-glucuronate 4-epimerase 1 | 111.756 | 41.545 | -1.4276 | 0.0008 | 0.00603091 |
| AT1G24330 | ARM repeat superfamily protein | 10.0019 | 3.71994 | -1.42693 | 0.003 | 0.0161227 |
| AT2G46260 | BTB/POZ/Kelch-associated protein | 267.275 | 99.4655 | -1.42606 | 0.0008 | 0.00603091 |
| AT4G00860 | AT0ZI1, ATOZI1, Protein of unknown function (DUF1138) | 712.244 | 265.061 | -1.42605 | 0.0012 | 0.00815259 |
| AT4G03560 | ATCCH1, ATTPC1, FOU2, TPC1, TPC1, two-pore channel 1 | 322.19 | 119.985 | -1.42506 | 0.00185 | 0.0113253 |
| AT1G23880 | NHL domain-containing protein | 22.573 | 8.40646 | -1.42503 | 0.00165 | 0.0103801 |
| AT1G80310 | sulfate transmembrane transporters | 8.19945 | 3.05368 | -1.42498 | 0.0115 | 0.0436881 |
| AT1G02170 | AMC1, ATMC1, ATMCPB1, LOL3, MCP1B, metacaspase 1 | 83.3753 | 31.0515 | -1.42496 | 0.0016 | 0.0101494 |
| AT5G42740 | Sugar isomerase (SIS) family protein | 133.84 | 49.8466 | -1.42494 | 0.00055 | 0.00457655 |
| AT1G57680 | function unknown | 99.9128 | 37.2119 | -1.4249 | 0.00085 | 0.00630464 |
| AT4G36900 | DEAR4, RAP2.10, related to AP2 10 | 56.0284 | 20.8732 | -1.42451 | 0.0025 | 0.0140894 |
| AT3G62400 | unknown protein | 408.316 | 152.137 | -1.42431 | 0.00105 | 0.00735817 |
| AT2G32960 | Phosphotyrosine protein phosphatases superfamily protein | 8.96415 | 3.34035 | -1.42417 | 0.0116 | 0.0439836 |
| AT4G35480 | RHA3B, RING-H2 finger A3B | 108.772 | 40.5335 | -1.42412 | 0.00115 | 0.00787231 |
| AT5G45410 | unknown protein | 197.066 | 73.4435 | -1.42397 | 0.001 | 0.00711095 |
| AT5G42930 | alpha/beta-Hydrolases superfamily protein | 95.6437 | 35.6642 | -1.42319 | 0.00165 | 0.0103801 |
| AT1G77680 | Ribonuclease II/R family protein | 236.464 | 88.1867 | -1.42299 | 0.00255 | 0.014306 |
| AT2G27000 | CYP705A8, cytochrome P450, family 705, subfamily A, polypeptide 8 | 54.0366 | 20.1544 | -1.42284 | 0.00145 | 0.00940576 |
| AT1G50590 | RmlC-like cupins superfamily protein | 67.8621 | 25.3163 | -1.42254 | 0.00165 | 0.0103801 |
| AT3G52060 | Core-2/I-branching beta-1,6-N-acetylglucosaminyltransferase family protein | 167.77 | 62.5968 | -1.42233 | 0.0007 | 0.00547072 |
| AT5G20090 | Uncharacterised protein family (UPF0041) | 180.122 | 67.2339 | -1.42172 | 0.0011 | 0.00759303 |
| AT2G37280 | ATPDR5, PDR5, pleiotropic drug resistance 5 | 10.2405 | 3.82392 | -1.42116 | 0.00465 | 0.0223951 |
| AT1G14660 | ATNHX8, NHX8, NHX8, Na+/H+ exchanger 8 | 5.11901 | 1.91194 | -1.42083 | 0.0134 | 0.0487461 |
| AT5G07250 | ATRBL3, RBL3, RHOMBOID-like protein 3 | 22.8688 | 8.54836 | -1.41966 | 0.0063 | 0.0281701 |
| AT3G12260 | LYR family of Fe/S cluster biogenesis protein | 191.433 | 71.5732 | -1.41935 | 0.00315 | 0.0166977 |
| AT1G53210 | sodium/calcium exchanger family protein / calcium-binding EF hand family protein | 393.768 | 147.235 | -1.41923 | 0.0017 | 0.0106071 |
| AT4G32030 | unknown protein | 204.22 | 76.3839 | -1.41879 | 0.0007 | 0.00547072 |
| AT2G38410 | ENTH/VHS/GAT family protein | 94.686 | 35.4149 | -1.41879 | 0.001 | 0.00711095 |
| AT5G08170 | ATAIH, EMB1873, porphyromonas-type peptidyl-arginine deiminase family protein | 40.8038 | 15.2623 | -1.41873 | 0.0017 | 0.0106071 |
| AT1G62422 | unknown protein | 12.4774 | 4.67216 | -1.41716 | 0.0137 | 0.0495371 |
| AT5G65390 | AGP7, arabinogalactan protein 7 | 69.7704 | 26.1388 | -1.41642 | 0.0054 | 0.0251864 |
| AT5G19070 | SNARE associated Golgi protein family | 120.401 | 45.1077 | -1.4164 | 0.00095 | 0.00685384 |
| AT5G63990 | Inositol monophosphatase family protein | 146.675 | 54.9581 | -1.41622 | 0.00135 | 0.0089118 |
| AT5G28150 | Plant protein of unknown function (DUF868) | 35.4704 | 13.2912 | -1.41614 | 0.00155 | 0.009918 |
| AT2G37180 | PIP2;3, PIP2C, RD28, Aquaporin-like superfamily protein | 25.1921 | 9.43978 | -1.41614 | 0.00325 | 0.0171315 |
| AT5G59160 | PPO, TOPP2, type one serine/threonine protein phosphatase 2 | 71.1482 | 26.6621 | -1.41604 | 0.00215 | 0.0126571 |
| AT1G12640 | MBOAT (membrane bound O-acyl transferase) family protein | 56.9024 | 21.3311 | -1.41553 | 0.00195 | 0.0117451 |
| AT1G74790 | catalytics | 67.7972 | 25.4186 | -1.41534 | 0.00095 | 0.00685384 |
| AT1G72700 | ATPase E1-E2 type family protein / haloacid dehalogenase-like hydrolase family protein | 79.8422 | 29.9921 | -1.41257 | 0.00075 | 0.00575475 |
| AT4G00355 | unknown protein | 57.7306 | 21.6882 | -1.41243 | 0.0021 | 0.0124516 |
| AT5G14000 | anac084, NAC084, NAC domain containing protein 84 | 61.1485 | 22.9818 | -1.41183 | 0.00255 | 0.014306 |
| AT4G04800 | ATMSRB3, MSRB3, methionine sulfoxide reductase B3 | 113.15 | 42.5369 | -1.41145 | 0.00195 | 0.0117451 |
| AT5G16550 | unknown protein | 151.309 | 56.8968 | -1.41108 | 0.00265 | 0.0147335 |
| AT1G60420 | DC1 domain-containing protein | 67.0665 | 25.233 | -1.41028 | 0.00255 | 0.014306 |
| AT5G57887 | unknown protein | 49.4774 | 18.6155 | -1.41026 | 0.0119 | 0.0448549 |
| AT3G19290 | ABF4, AREB2, ABRE binding factor 4 | 72.2665 | 27.2031 | -1.40956 | 0.00185 | 0.0113253 |
| AT4G37610 | BT5, BTB and TAZ domain protein 5 | 600.848 | 226.306 | -1.40873 | 0.00235 | 0.0134663 |
| AT5G48150 | PAT1, GRAS family transcription factor | 35.1545 | 13.2461 | -1.40814 | 0.0019 | 0.0115387 |
| AT1G15710 | prephenate dehydrogenase family protein | 25.7587 | 9.70868 | -1.40772 | 0.00575 | 0.0263656 |
| AT3G57785 | unknown protein | 41.8584 | 15.7982 | -1.40575 | 0.0071 | 0.0306604 |
| AT2G26890 | GRV2, KAM2, DNAJ heat shock N-terminal domain-containing protein | 32.8545 | 12.4019 | -1.40553 | 0.0009 | 0.0065867 |
| AT4G34700 | LYR family of Fe/S cluster biogenesis protein | 486.693 | 183.75 | -1.40527 | 0.0016 | 0.0101494 |
| AT2G16430 | ATPAP10, PAP10, purple acid phosphatase 10 | 28.9498 | 10.9355 | -1.40453 | 0.0031 | 0.0165078 |
| AT1G15530 | Concanavalin A-like lectin protein kinase family protein | 33.1619 | 12.5379 | -1.40323 | 0.0016 | 0.0101494 |
| AT5G20990 | B73, CHL6, CNX, CNX1, SIR4, molybdopterin biosynthesis CNX1 protein / molybdenum cofactor biosynthesis enzyme CNX1 (CNX1) | 73.17 | 27.6679 | -1.40304 | 0.0008 | 0.00603091 |
| AT3G02360 | 6-phosphogluconate dehydrogenase family protein | 299.923 | 113.415 | -1.40299 | 0.00075 | 0.00575475 |
| AT5G61530 | small G protein family protein / RhoGAP family protein | 135.558 | 51.2875 | -1.40223 | 0.0008 | 0.00603091 |
| AT5G64260 | EXL2, EXORDIUM like 2 | 247.955 | 93.8201 | -1.40211 | 0.0016 | 0.0101494 |
| AT5G10695 | unknown protein | 537.896 | 203.558 | -1.40189 | 0.0019 | 0.0115387 |
| AT1G76920 | F-box family protein | 60.7336 | 22.9938 | -1.40125 | 0.0019 | 0.0115387 |
| AT3G59050 | ATPAO3, PAO3, polyamine oxidase 3 | 185.259 | 70.166 | -1.4007 | 0.0009 | 0.0065867 |
| AT5G66510 | GAMMA CA3, gamma carbonic anhydrase 3 | 254.873 | 96.5655 | -1.4002 | 0.00075 | 0.00575475 |
| AT1G42990 | ATBZIP60, BZIP60, BZIP60, basic region/leucine zipper motif 60 | 57.5241 | 21.8062 | -1.39943 | 0.00305 | 0.0163293 |
| AT1G07890 | APX1, ATAPX01, ATAPX1, CS1, MEE6, ascorbate peroxidase 1 | 1219.8 | 462.538 | -1.39899 | 0.0053 | 0.0248135 |
| AT1G09940 | HEMA2, Glutamyl-tRNA reductase family protein | 333.235 | 126.406 | -1.39847 | 0.00215 | 0.0126571 |
| AT4G34740 | ASE2, ATASE2, ATPURF2, CIA1, GLN phosphoribosyl pyrophosphate amidotransferase 2 | 22.8674 | 8.68136 | -1.3973 | 0.00235 | 0.0134663 |
| AT1G27500 | Tetratricopeptide repeat (TPR)-like superfamily protein | 17.9537 | 6.81706 | -1.39706 | 0.00225 | 0.0130442 |
| AT1G75230 | DNA glycosylase superfamily protein | 52.0697 | 19.7808 | -1.39635 | 0.0021 | 0.0124516 |
| AT5G08670 | ATP synthase alpha/beta family protein | 430.79 | 163.692 | -1.396 | 0.0053 | 0.0248135 |
| AT1G57990 | ATPUP18, PUP18, purine permease 18 | 81.2862 | 30.8932 | -1.39572 | 0.00105 | 0.00735817 |
| AT1G15130 | Endosomal targeting BRO1-like domain-containing protein | 91.5767 | 34.8092 | -1.39551 | 0.00115 | 0.00787231 |
| AT1G66900 | alpha/beta-Hydrolases superfamily protein | 40.5715 | 15.4221 | -1.39547 | 0.0027 | 0.0149318 |
| AT3G55260 | ATHEX2, HEXO1, beta-hexosaminidase 1 | 21.5374 | 8.18777 | -1.3953 | 0.0029 | 0.0157089 |
| AT5G13810 | Glutaredoxin family protein | 102.324 | 38.9278 | -1.39427 | 0.0007 | 0.00547072 |
| AT2G45960 | ATHH2, PIP1;2, PIP1B, TMP-A, plasma membrane intrinsic protein 1B | 1323.36 | 503.512 | -1.39411 | 0.0038 | 0.0192125 |
| AT1G76600 | unknown protein | 345.153 | 131.333 | -1.394 | 0.00135 | 0.0089118 |
| AT2G05630 | ATG8D, Ubiquitin-like superfamily protein | 41.9784 | 15.9814 | -1.39326 | 0.0091 | 0.0368164 |
| AT1G25390 | Protein kinase superfamily protein | 25.072 | 9.54529 | -1.39321 | 0.0027 | 0.0149318 |
| AT5G51460 | ATTPPA, Haloacid dehalogenase-like hydrolase (HAD) superfamily protein | 71.2541 | 27.1365 | -1.39274 | 0.00135 | 0.0089118 |
| AT1G51980 | Insulinase (Peptidase family M16) protein | 362.84 | 138.268 | -1.39187 | 0.00265 | 0.0147335 |
| AT5G14150 | Protein of unknown function, DUF642 | 19.6594 | 7.49199 | -1.3918 | 0.00795 | 0.0333705 |
| AT3G03250 | AtUGP1, UGP, UGP1, UDP-GLUCOSE PYROPHOSPHORYLASE 1 | 268.528 | 102.339 | -1.39171 | 0.00755 | 0.0321272 |
| AT5G63470 | NF-YC4, nuclear factor Y, subunit C4 | 37.9743 | 14.4741 | -1.39155 | 0.00325 | 0.0171315 |
| AT5G01490 | ATCAX4, CAX4, cation exchanger 4 | 63.4543 | 24.1935 | -1.3911 | 0.0014 | 0.00917155 |
| AT5G21990 | Tetratricopeptide repeat (TPR)-like superfamily protein | 294.186 | 112.192 | -1.39076 | 0.00125 | 0.00840292 |
| AT5G47120 | ATBI-1, ATBI1, BI-1, BI1, BAX inhibitor 1 | 1011.32 | 385.747 | -1.39051 | 0.00305 | 0.0163293 |
| AT1G21380 | Target of Myb protein 1 | 218.263 | 83.266 | -1.39027 | 0.0045 | 0.0218802 |
| AT4G00300 | fringe-related protein | 133.469 | 50.9446 | -1.38951 | 0.0014 | 0.00917155 |
| AT1G47570 | RING/U-box superfamily protein | 17.4023 | 6.64635 | -1.38864 | 0.0048 | 0.0229834 |
| AT1G04250 | AXR3, IAA17, AUX/IAA transcriptional regulator family protein | 244.712 | 93.5005 | -1.38804 | 0.00145 | 0.00940576 |
| AT3G60260 | ELMO/CED-12 family protein | 83.4407 | 31.885 | -1.38787 | 0.00185 | 0.0113253 |
| AT1G19650 | Sec14p-like phosphatidylinositol transfer family protein | 22.7383 | 8.68943 | -1.38779 | 0.0037 | 0.0188077 |
| AT1G13520 | Protein of unknown function (DUF1262) | 9.07737 | 3.47035 | -1.38719 | 0.0129 | 0.0474149 |
| AT4G38140 | RING/U-box superfamily protein | 198.819 | 76.0474 | -1.38649 | 0.0062 | 0.0278676 |
| AT3G47960 | Major facilitator superfamily protein | 145.322 | 55.5909 | -1.38633 | 0.00145 | 0.00940576 |
| AT4G30060 | Core-2/I-branching beta-1,6-N-acetylglucosaminyltransferase family protein | 51.1928 | 19.5875 | -1.38601 | 0.00225 | 0.0130442 |
| AT3G56800 | acam-3, CAM3, calmodulin 3 | 357.222 | 136.684 | -1.38598 | 0.00195 | 0.0117451 |
| AT5G65280 | GCL1, GCR2-like 1 | 78.007 | 29.861 | -1.38534 | 0.00125 | 0.00840292 |
| AT3G27380 | SDH2-1, succinate dehydrogenase 2-1 | 171.825 | 65.8095 | -1.38457 | 0.0018 | 0.0110912 |
| AT5G05860 | UGT76C2, UDP-glucosyl transferase 76C2 | 12.966 | 4.96653 | -1.38442 | 0.0081 | 0.0338066 |
| AT4G29190 | Zinc finger C-x8-C-x5-C-x3-H type family protein | 176.941 | 67.8202 | -1.38348 | 0.0016 | 0.0101494 |
| AT1G01240 | unknown protein | 132.524 | 50.8107 | -1.38305 | 0.00155 | 0.009918 |
| AT1G49340 | ATPI4K ALPHA, Phosphatidylinositol 3- and 4-kinase family protein | 34.4437 | 13.2183 | -1.38171 | 0.00105 | 0.00735817 |
| AT3G11030 | TBL32, TRICHOME BIREFRINGENCE-LIKE 32 | 49.0593 | 18.8426 | -1.38053 | 0.0031 | 0.0165078 |
| AT5G02230 | Haloacid dehalogenase-like hydrolase (HAD) superfamily protein | 981.784 | 377.193 | -1.3801 | 0.00615 | 0.0276932 |
| AT1G79590 | ATSYP52, SYP52, syntaxin of plants 52 | 123.737 | 47.5749 | -1.379 | 0.0014 | 0.00917155 |
| AT4G29780 | unknown protein | 237.626 | 91.4077 | -1.3783 | 0.0015 | 0.00967937 |
| AT4G38800 | ATMTAN1, ATMTN1, MTAN1, MTN1, methylthioadenosine nucleosidase 1 | 222.955 | 85.7774 | -1.37808 | 0.0093 | 0.0372979 |
| AT2G30870 | ATGSTF10, ATGSTF4, ERD13, GSTF10, glutathione S-transferase PHI 10 | 1336.05 | 514.049 | -1.378 | 0.0051 | 0.0241339 |
| AT3G57520 | AtSIP2, SIP2, seed imbibition 2 | 213.073 | 82.0158 | -1.37738 | 0.0026 | 0.0145003 |
| AT3G59940 | Galactose oxidase/kelch repeat superfamily protein | 95.7151 | 36.8476 | -1.37717 | 0.01085 | 0.0417717 |
| AT5G32440 | Ubiquitin system component Cue protein | 123.319 | 47.5331 | -1.37539 | 0.0024 | 0.0136815 |
| AT2G41190 | Transmembrane amino acid transporter family protein | 11.8403 | 4.56825 | -1.37399 | 0.00335 | 0.0175049 |
| AT3G11840 | PUB24, plant U-box 24 | 9.44953 | 3.64883 | -1.37281 | 0.0109 | 0.0419234 |
| AT5G03455 | ACR2, ARATH;CDC25, CDC25, Rhodanese/Cell cycle control phosphatase superfamily protein | 32.2376 | 12.4495 | -1.37266 | 0.0093 | 0.0372979 |
| AT1G09930 | ATOPT2, OPT2, oligopeptide transporter 2 | 17.1248 | 6.61336 | -1.37263 | 0.00455 | 0.0220583 |
| AT4G22820 | A20/AN1-like zinc finger family protein | 301.504 | 116.647 | -1.37003 | 0.00095 | 0.00685384 |
| AT1G74400 | Tetratricopeptide repeat (TPR)-like superfamily protein | 16.5434 | 6.40993 | -1.36788 | 0.00575 | 0.0263656 |
| AT3G02910 | AIG2-like (avirulence induced gene) family protein | 101.403 | 39.2968 | -1.36762 | 0.0026 | 0.0145003 |
| AT3G13410 | unknown protein | 412.011 | 159.687 | -1.36744 | 0.00145 | 0.00940576 |
| AT2G22910 | NAGS1, N-acetyl-l-glutamate synthase 1 | 29.4485 | 11.4144 | -1.36734 | 0.002 | 0.0119879 |
| AT1G53560 | Ribosomal protein L18ae family | 63.0002 | 24.436 | -1.36635 | 0.00705 | 0.0305176 |
| AT1G79330 | AMC6, ATMC5, ATMCP2B, MC5, metacaspase 5 | 14.6955 | 5.70211 | -1.36581 | 0.0087 | 0.0356202 |
| AT5G47030 | ATPase, F1 complex, delta/epsilon subunit | 453.372 | 175.961 | -1.36544 | 0.00305 | 0.0163293 |
| AT4G02520 | ATGSTF2, ATPM24, ATPM24.1, GST2, GSTF2, glutathione S-transferase PHI 2 | 99.5182 | 38.632 | -1.36516 | 0.0059 | 0.0268421 |
| AT5G04160 | Nucleotide-sugar transporter family protein | 55.5043 | 21.5547 | -1.3646 | 0.00375 | 0.0189985 |
| AT5G24270 | ATSOS3, CBL4, SOS3, Calcium-binding EF-hand family protein | 77.209 | 29.9928 | -1.36415 | 0.0021 | 0.0124516 |
| AT1G18160 | Protein kinase superfamily protein | 42.1107 | 16.363 | -1.36375 | 0.0012 | 0.00815259 |
| AT1G02900 | ATRALF1, RALF1, RALFL1, rapid alkalinization factor 1 | 47.2155 | 18.3495 | -1.36352 | 0.00775 | 0.0327601 |
| AT5G64750 | ABR1, Integrase-type DNA-binding superfamily protein | 700.954 | 272.447 | -1.36335 | 0.00945 | 0.0377542 |
| AT3G07040 | RPM1, RPS3, NB-ARC domain-containing disease resistance protein | 4.56268 | 1.77353 | -1.36326 | 0.00695 | 0.0302036 |
| AT4G22310 | Uncharacterised protein family (UPF0041) | 224.335 | 87.2638 | -1.3622 | 0.00305 | 0.0163293 |
| AT3G08780 | unknown protein | 29.3667 | 11.425 | -1.36199 | 0.006 | 0.027135 |
| AT2G18210 | unknown protein | 283.135 | 110.168 | -1.36179 | 0.00215 | 0.0126571 |
| AT1G18910 | zinc ion binding;zinc ion binding | 51.6658 | 20.1109 | -1.36123 | 0.0043 | 0.0211046 |
| AT1G72160 | Sec14p-like phosphatidylinositol transfer family protein | 64.9676 | 25.294 | -1.36093 | 0.0036 | 0.0184414 |
| AT1G76980 | BEST Arabidopsis thaliana protein match is: embryo defective 2170 (TAIR:AT1G21390.1) | 192.358 | 74.9205 | -1.36036 | 0.0015 | 0.00967937 |
| AT3G11690 | unknown protein | 20.7242 | 8.07341 | -1.36006 | 0.0052 | 0.0244668 |
| AT4G19040 | EDR2, ENHANCED DISEASE RESISTANCE 2 | 30.6645 | 11.9508 | -1.35946 | 0.0026 | 0.0145003 |
| AT2G01190 | Octicosapeptide/Phox/Bem1p family protein | 31.5797 | 12.3094 | -1.35924 | 0.00185 | 0.0113253 |
| AT5G64920 | CIP8, COP1-interacting protein 8 | 28.1787 | 10.9882 | -1.35865 | 0.00285 | 0.0154761 |
| AT3G48140 | B12D protein | 1171.8 | 457.035 | -1.35834 | 0.00145 | 0.00940576 |
| AT3G44190 | FAD/NAD(P)-binding oxidoreductase family protein | 577.31 | 225.19 | -1.3582 | 0.004 | 0.0200243 |
| AT4G12010 | Disease resistance protein (TIR-NBS-LRR class) family | 15.7912 | 6.16154 | -1.35775 | 0.0019 | 0.0115387 |
| AT1G22930 | T-complex protein 11 | 239.318 | 93.3886 | -1.35761 | 0.0034 | 0.01771 |
| AT1G50320 | ATHX, ATX, THX, thioredoxin X | 25.0482 | 9.77487 | -1.35756 | 0.0085 | 0.0349894 |
| AT1G08940 | Phosphoglycerate mutase family protein | 97.368 | 38.0252 | -1.35649 | 0.00375 | 0.0189985 |
| AT2G45820 | Remorin family protein | 386.462 | 150.942 | -1.35633 | 0.0018 | 0.0110912 |
| AT3G16460 | Mannose-binding lectin superfamily protein | 1522.3 | 595.036 | -1.35521 | 0.00965 | 0.0383598 |
| AT1G03220 | Eukaryotic aspartyl protease family protein | 981.314 | 383.809 | -1.35433 | 0.00695 | 0.0302036 |
| AT4G35260 | IDH-I, IDH1, isocitrate dehydrogenase 1 | 133.746 | 52.3263 | -1.35389 | 0.00195 | 0.0117451 |
| AT5G57660 | ATCOL5, COL5, CONSTANS-like 5 | 178.487 | 69.8385 | -1.35372 | 0.00205 | 0.012221 |
| AT1G44100 | AAP5, amino acid permease 5 | 11.8274 | 4.63074 | -1.35282 | 0.00755 | 0.0321272 |
| AT2G27310 | F-box family protein | 260.642 | 102.161 | -1.35123 | 0.00195 | 0.0117451 |
| AT1G25560 | EDF1, TEM1, AP2/B3 transcription factor family protein | 250.875 | 98.3487 | -1.35099 | 0.0018 | 0.0110912 |
| AT3G62720 | ATXT1, XT1, XXT1, xylosyltransferase 1 | 89.3846 | 35.0464 | -1.35076 | 0.0017 | 0.0106071 |
| AT1G64563 | other RNA | 4.57445 | 1.79414 | -1.35031 | 0.00745 | 0.0318523 |
| AT1G76030 | ATPase, V1 complex, subunit B protein | 319.447 | 125.435 | -1.34863 | 0.0025 | 0.0140894 |
| AT5G43030 | Cysteine/Histidine-rich C1 domain family protein | 9.17098 | 3.60298 | -1.34788 | 0.00815 | 0.0339652 |
| AT3G01040 | GAUT13, galacturonosyltransferase 13 | 48.3259 | 18.9942 | -1.34724 | 0.002 | 0.0119879 |
| AT5G13430 | Ubiquinol-cytochrome C reductase iron-sulfur subunit | 89.1908 | 35.06 | -1.34707 | 0.0024 | 0.0136815 |
| AT4G32480 | Protein of unknown function (DUF506) | 237.518 | 93.3727 | -1.34697 | 0.001 | 0.00711095 |
| AT4G21534 | Diacylglycerol kinase family protein | 13.6904 | 5.38207 | -1.34693 | 0.01275 | 0.0470557 |
| AT1G08930 | ERD6, Major facilitator superfamily protein | 347.06 | 136.441 | -1.34691 | 0.002 | 0.0119879 |
| AT1G09270 | IMPA-4, importin alpha isoform 4 | 133.346 | 52.5573 | -1.34321 | 0.0059 | 0.0268421 |
| AT5G56630 | PFK7, phosphofructokinase 7 | 237.492 | 93.6197 | -1.34299 | 0.00225 | 0.0130442 |
| AT1G78830 | Curculin-like (mannose-binding) lectin family protein | 185.136 | 72.9902 | -1.34281 | 0.0009 | 0.0065867 |
| AT4G39030 | EDS5, SID1, MATE efflux family protein | 92.2074 | 36.3607 | -1.3425 | 0.0019 | 0.0115387 |
| AT5G58750 | NAD(P)-binding Rossmann-fold superfamily protein | 28.2834 | 11.1555 | -1.34221 | 0.0037 | 0.0188077 |
| AT1G18740 | Protein of unknown function (DUF793) | 125.755 | 49.6226 | -1.34154 | 0.00215 | 0.0126571 |
| AT3G50260 | ATERF#011, CEJ1, DEAR1, cooperatively regulated by ethylene and jasmonate 1 | 117.099 | 46.2149 | -1.3413 | 0.00275 | 0.0151029 |
| AT4G16070 | Mono-/di-acylglycerol lipase, N-terminal;Lipase, class 3 | 25.4008 | 10.0289 | -1.34072 | 0.0022 | 0.0128446 |
| AT5G06740 | Concanavalin A-like lectin protein kinase family protein | 15.8401 | 6.25601 | -1.34027 | 0.00595 | 0.0269704 |
| AT2G24240 | BTB/POZ domain with WD40/YVTN repeat-like protein | 17.1747 | 6.79136 | -1.33851 | 0.00805 | 0.0336689 |
| AT2G43160 | ENTH/VHS family protein | 190.965 | 75.5139 | -1.33849 | 0.00545 | 0.025354 |
| AT1G76200 | unknown protein | 684.504 | 270.815 | -1.33775 | 0.0019 | 0.0115387 |
| AT1G16670 | Protein kinase superfamily protein | 104.533 | 41.418 | -1.33563 | 0.00125 | 0.00840292 |
| AT5G43050 | NPQ6, Protein of unknown function (DUF565) | 62.4764 | 24.7659 | -1.33495 | 0.00645 | 0.0286275 |
| AT5G20080 | FAD/NAD(P)-binding oxidoreductase | 248.57 | 98.5745 | -1.33436 | 0.00185 | 0.0113253 |
| AT5G39570 | function unknown | 358.786 | 142.377 | -1.33341 | 0.0046 | 0.0222191 |
| AT1G08650 | ATPPCK1, PPCK1, phosphoenolpyruvate carboxylase kinase 1 | 161.444 | 64.0705 | -1.3333 | 0.0023 | 0.0132526 |
| AT4G03430 | EMB2770, STA1, pre-mRNA splicing factor-related | 183.66 | 72.922 | -1.33261 | 0.00385 | 0.0194256 |
| AT3G17890 | unknown protein | 31.2559 | 12.4177 | -1.33173 | 0.0112 | 0.0427699 |
| AT4G40060 | ATHB-16, ATHB16, HB16, homeobox protein 16 | 504.476 | 200.452 | -1.33153 | 0.00295 | 0.0159014 |
| AT1G79360 | 2-Oct, ATOCT2, OCT2, organic cation/carnitine transporter 2 | 34.2739 | 13.6342 | -1.32988 | 0.00605 | 0.0273236 |
| AT1G01620 | PIP1;3, PIP1C, TMP-B, plasma membrane intrinsic protein 1C | 508.785 | 202.414 | -1.32975 | 0.00545 | 0.025354 |
| AT3G10020 | unknown protein | 509.489 | 202.744 | -1.32939 | 0.0059 | 0.0268421 |
| AT3G02090 | MPPBETA, Insulinase (Peptidase family M16) protein | 424.921 | 169.176 | -1.32867 | 0.009 | 0.0365462 |
| AT4G40065 | other RNA | 12.9175 | 5.14436 | -1.32826 | 0.01105 | 0.0423687 |
| AT5G61560 | U-box domain-containing protein kinase family protein | 27.0559 | 10.7775 | -1.32792 | 0.00345 | 0.0179093 |
| AT2G18840 | Integral membrane Yip1 family protein | 44.031 | 17.5447 | -1.32748 | 0.0065 | 0.0287784 |
| AT3G57090 | BIGYIN, FIS1A, Tetratricopeptide repeat (TPR)-like superfamily protein | 132.829 | 53.0448 | -1.32429 | 0.00295 | 0.0159014 |
| AT3G06060 | NAD(P)-binding Rossmann-fold superfamily protein | 59.2922 | 23.6819 | -1.32406 | 0.00365 | 0.0186349 |
| AT1G30130 | unknown protein | 120.671 | 48.2212 | -1.32333 | 0.00205 | 0.012221 |
| AT4G00430 | PIP1;4, PIP1E, TMP-C, plasma membrane intrinsic protein 1;4 | 192.525 | 76.9364 | -1.32331 | 0.00245 | 0.0139065 |
| AT5G66200 | ARO2, armadillo repeat only 2 | 35.8486 | 14.3294 | -1.32294 | 0.0075 | 0.0319763 |
| AT3G02070 | Cysteine proteinases superfamily protein | 64.3665 | 25.7361 | -1.32252 | 0.00235 | 0.0134663 |
| AT5G04840 | bZIP protein | 53.7091 | 21.4757 | -1.32246 | 0.00285 | 0.0154761 |
| AT5G56210 | WIP2, WPP domain interacting protein 2 | 29.6905 | 11.8781 | -1.32169 | 0.00365 | 0.0186349 |
| AT5G41750 | Disease resistance protein (TIR-NBS-LRR class) family | 12.1785 | 4.87351 | -1.3213 | 0.00675 | 0.0295876 |
| AT5G13180 | ANAC083, NAC083, VNI2, NAC domain containing protein 83 | 432.024 | 172.947 | -1.32078 | 0.0022 | 0.0128446 |
| AT4G11280 | ACS6, ATACS6, 1-aminocyclopropane-1-carboxylic acid (acc) synthase 6 | 67.5176 | 27.0464 | -1.31982 | 0.00205 | 0.012221 |
| AT5G17060 | ARFB1B, ATARFB1B, ADP-ribosylation factor B1B | 99.3639 | 39.8485 | -1.3182 | 0.00175 | 0.0108609 |
| AT5G11000 | Plant protein of unknown function (DUF868) | 32.8341 | 13.1742 | -1.31748 | 0.00335 | 0.0175049 |
| AT4G35090 | CAT2, catalase 2 | 348.67 | 139.931 | -1.31714 | 0.0046 | 0.0222191 |
| AT4G35950 | ARAC6, ATRAC6, ATROP5, RAC2, RAC6, ROP5, RAC-like 6 | 32.9335 | 13.2209 | -1.31673 | 0.00895 | 0.0364179 |
| AT4G14410 | bHLH104, basic helix-loop-helix (bHLH) DNA-binding superfamily protein | 137.35 | 55.1423 | -1.31663 | 0.00275 | 0.0151029 |
| AT2G38360 | PRA1.B4, prenylated RAB acceptor 1.B4 | 142.622 | 57.3274 | -1.3149 | 0.0036 | 0.0184414 |
| AT1G16700 | Alpha-helical ferredoxin | 219.072 | 88.1035 | -1.31413 | 0.0025 | 0.0140894 |
| AT1G75950 | ASK1, ATSKP1, SKP1, SKP1A, UIP1, S phase kinase-associated protein 1 | 732.56 | 294.693 | -1.31374 | 0.00195 | 0.0117451 |
| AT2G43120 | RmlC-like cupins superfamily protein | 621.829 | 250.251 | -1.31314 | 0.0046 | 0.0222191 |
| AT3G62660 | GATL7, galacturonosyltransferase-like 7 | 36.5758 | 14.7213 | -1.31299 | 0.00385 | 0.0194256 |
| AT2G40620 | Basic-leucine zipper (bZIP) transcription factor family protein | 141.222 | 56.8848 | -1.31186 | 0.00455 | 0.0220583 |
| AT3G22750 | Protein kinase superfamily protein | 33.9134 | 13.6671 | -1.31115 | 0.00275 | 0.0151029 |
| AT2G43340 | Protein of unknown function (DUF1685) | 493.845 | 199.06 | -1.31085 | 0.00595 | 0.0269704 |
| AT3G30775 | AT-POX, Methylenetetrahydrofolate reductase family protein | 660.893 | 266.421 | -1.31071 | 0.0064 | 0.0284694 |
| AT5G58375 | Methyltransferase-related protein | 151.981 | 61.2825 | -1.31034 | 0.0025 | 0.0140894 |
| AT1G45688 | unknown protein | 140.17 | 56.5209 | -1.31032 | 0.0022 | 0.0128446 |
| AT5G67530 | ATPUB49, PUB49, plant U-box 49 | 62.1712 | 25.0897 | -1.30915 | 0.00825 | 0.0342522 |
| AT1G35720 | ANNAT1, ATOXY5, OXY5, annexin 1 | 1520.28 | 613.756 | -1.3086 | 0.0137 | 0.0495371 |
| AT3G14070 | ATCCX3, CAX9, CCX3, cation exchanger 9 | 6.94484 | 2.80479 | -1.30805 | 0.01325 | 0.0484049 |
| AT1G23390 | Kelch repeat-containing F-box family protein | 13.4122 | 5.41725 | -1.30791 | 0.0122 | 0.0455694 |
| AT3G20560 | ATPDI12, ATPDIL5-3, PDI12, PDIL5-3, PDI-like 5-3 | 22.5684 | 9.12075 | -1.30708 | 0.0049 | 0.0233549 |
| AT4G39640 | GGT1, gamma-glutamyl transpeptidase 1 | 49.6067 | 20.0516 | -1.30682 | 0.00365 | 0.0186349 |
| AT4G16450 | unknown protein | 549.051 | 222.091 | -1.30579 | 0.00175 | 0.0108609 |
| AT4G16480 | ATINT4, INT4, inositol transporter 4 | 8.91291 | 3.60611 | -1.30545 | 0.01205 | 0.0451624 |
| AT1G74590 | ATGSTU10, GSTU10, glutathione S-transferase TAU 10 | 62.8311 | 25.4314 | -1.30487 | 0.00515 | 0.024295 |
| AT2G41780 | unknown protein | 31.0083 | 12.5665 | -1.30307 | 0.0125 | 0.0463577 |
| AT1G09950 | RAS1, RESPONSE TO ABA AND SALT 1 | 45.6906 | 18.5278 | -1.30221 | 0.0043 | 0.0211046 |
| AT1G51650 | ATP synthase epsilon chain, mitochondrial | 926.751 | 376.214 | -1.30063 | 0.00305 | 0.0163293 |
| AT1G29390 | COR314-TM2, COR413IM2, cold regulated 314 thylakoid membrane 2 | 25.0068 | 10.1543 | -1.30023 | 0.01165 | 0.0441141 |
| AT3G05050 | Protein kinase superfamily protein | 52.5673 | 21.3469 | -1.30014 | 0.0021 | 0.0124516 |
| AT4G22330 | ATCES1, Alkaline phytoceramidase (aPHC) | 170.636 | 69.3336 | -1.2993 | 0.00165 | 0.0103801 |
| AT5G03540 | ATEXO70A1, EXO70A1, exocyst subunit exo70 family protein A1 | 95.0941 | 38.6426 | -1.29916 | 0.002 | 0.0119879 |
| AT1G03457 | RNA-binding (RRM/RBD/RNP motifs) family protein | 29.8703 | 12.152 | -1.29752 | 0.00495 | 0.0235479 |
| AT1G44770 | unknown protein | 74.5119 | 30.3499 | -1.29578 | 0.0035 | 0.0180741 |
| AT3G49350 | Ypt/Rab-GAP domain of gyp1p superfamily protein | 39.3889 | 16.0442 | -1.29574 | 0.0031 | 0.0165078 |
| AT2G42520 | P-loop containing nucleoside triphosphate hydrolases superfamily protein | 63.8609 | 26.015 | -1.29559 | 0.0028 | 0.0152969 |
| AT1G72020 | unknown protein | 670.043 | 272.982 | -1.29545 | 0.0028 | 0.0152969 |
| AT2G40800 | unknown protein | 78.7952 | 32.1192 | -1.29467 | 0.0024 | 0.0136815 |
| AT3G28320 | Protein of unknown function (DUF677) | 215.373 | 87.9915 | -1.2914 | 0.0064 | 0.0284694 |
| AT5G51440 | HSP20-like chaperones superfamily protein | 196.686 | 80.3616 | -1.29132 | 0.0135 | 0.0489749 |
| AT5G66250 | kinectin-related | 67.552 | 27.6104 | -1.29079 | 0.0052 | 0.0244668 |
| AT1G80240 | Protein of unknown function, DUF642 | 68.2769 | 27.916 | -1.29031 | 0.0034 | 0.01771 |
| AT2G17440 | PIRL5, plant intracellular ras group-related LRR 5 | 203.578 | 83.2521 | -1.29003 | 0.0055 | 0.0254967 |
| AT1G21590 | Protein kinase protein with adenine nucleotide alpha hydrolases-like domain | 21.323 | 8.723 | -1.28952 | 0.00595 | 0.0269704 |
| AT5G45510 | Leucine-rich repeat (LRR) family protein | 159.03 | 65.1305 | -1.28789 | 0.0075 | 0.0319763 |
| AT3G16760 | Tetratricopeptide repeat (TPR)-like superfamily protein | 51.8636 | 21.2461 | -1.28753 | 0.00275 | 0.0151029 |
| AT2G03480 | QUL2, QUASIMODO2 LIKE 2 | 15.591 | 6.3875 | -1.28739 | 0.00645 | 0.0286275 |
| AT3G19950 | RING/U-box superfamily protein | 82.7952 | 33.9288 | -1.28703 | 0.0028 | 0.0152969 |
| AT1G72130 | Major facilitator superfamily protein | 30.3404 | 12.4373 | -1.28657 | 0.0049 | 0.0233549 |
| AT5G04340 | C2H2, CZF2, ZAT6, zinc finger of Arabidopsis thaliana 6 | 148.762 | 60.9907 | -1.28634 | 0.00425 | 0.0209475 |
| AT1G07310 | Calcium-dependent lipid-binding (CaLB domain) family protein | 45.9341 | 18.8343 | -1.2862 | 0.00455 | 0.0220583 |
| AT1G80820 | ATCCR2, CCR2, cinnamoyl coa reductase | 24.6743 | 10.1207 | -1.2857 | 0.01015 | 0.0398672 |
| AT3G10420 | P-loop containing nucleoside triphosphate hydrolases superfamily protein | 41.7345 | 17.1255 | -1.28509 | 0.0023 | 0.0132526 |
| AT1G28240 | Protein of unknown function (DUF616) | 48.1016 | 19.7432 | -1.28473 | 0.0017 | 0.0106071 |
| AT3G52730 | ubiquinol-cytochrome C reductase UQCRX/QCR9-like family protein | 660.558 | 271.279 | -1.28391 | 0.00275 | 0.0151029 |
| AT5G41992 | CPuORF58, conserved peptide upstream open reading frame 58 | 166.711 | 68.5302 | -1.28254 | 0.00405 | 0.0202184 |
| AT5G46250 | RNA-binding protein | 148.519 | 61.0539 | -1.28249 | 0.0018 | 0.0110912 |
| AT3G07460 | Protein of unknown function, DUF538 | 41.546 | 17.0871 | -1.28181 | 0.0059 | 0.0268421 |
| AT3G50950 | ZAR1, HOPZ-ACTIVATED RESISTANCE 1 | 49.7832 | 20.4758 | -1.28174 | 0.00815 | 0.0339652 |
| AT5G54500 | FQR1, flavodoxin-like quinone reductase 1 | 235.851 | 97.0811 | -1.28061 | 0.0022 | 0.0128446 |
| AT4G11220 | BTI2, RTNLB2, VIRB2-interacting protein 2 | 640.308 | 263.694 | -1.2799 | 0.0045 | 0.0218802 |
| AT5G15820 | RING/U-box superfamily protein | 21.5046 | 8.85723 | -1.27972 | 0.00885 | 0.0361074 |
| AT3G62010 | unknown protein | 321.403 | 132.627 | -1.27701 | 0.01375 | 0.0496726 |
| AT5G04235.1 | transposable element gene | 9.9816 | 4.12157 | -1.27608 | 0.00475 | 0.0228046 |
| AT3G28715 | ATPase, V0/A0 complex, subunit C/D | 163.176 | 67.3854 | -1.27593 | 0.0047 | 0.0226084 |
| AT4G24690 | ubiquitin-associated (UBA)/TS-N domain-containing protein / octicosapeptide/Phox/Bemp1 (PB1) domain-containing protein | 366.753 | 151.47 | -1.27578 | 0.0057 | 0.0262091 |
| AT5G03380 | Heavy metal transport/detoxification superfamily protein | 226.259 | 93.4592 | -1.27557 | 0.0021 | 0.0124516 |
| AT3G46830 | ATRAB-A2C, ATRAB11A, ATRABA2C, RAB-A2C, RABA2c, RAB GTPase homolog A2C | 66.6165 | 27.5503 | -1.27381 | 0.0071 | 0.0306604 |
| AT5G11650 | alpha/beta-Hydrolases superfamily protein | 262.68 | 108.641 | -1.27373 | 0.00425 | 0.0209475 |
| AT3G05570 | unknown protein | 98.4789 | 40.7456 | -1.27317 | 0.0083 | 0.0344309 |
| AT5G64120 | Peroxidase superfamily protein | 29.2919 | 12.122 | -1.27287 | 0.0089 | 0.0362815 |
| AT2G39720 | RHC2A, RING-H2 finger C2A | 62.6372 | 25.9228 | -1.2728 | 0.00285 | 0.0154761 |
| AT3G63150 | ATCBG, MIRO2, MIRO-related GTP-ase 2 | 56.7568 | 23.4936 | -1.27252 | 0.0033 | 0.0173075 |
| AT3G08930 | LMBR1-like membrane protein | 114.664 | 47.4671 | -1.27241 | 0.00215 | 0.0126571 |
| AT3G10760 | Homeodomain-like superfamily protein | 37.684 | 15.6015 | -1.27227 | 0.0061 | 0.0274993 |
| AT5G25770 | alpha/beta-Hydrolases superfamily protein | 75.721 | 31.3532 | -1.27208 | 0.0113 | 0.0430687 |
| AT1G65890 | AAE12, acyl activating enzyme 12 | 17.9297 | 7.43053 | -1.27082 | 0.00595 | 0.0269704 |
| AT1G02816 | Protein of unknown function, DUF538 | 77.5073 | 32.1333 | -1.27026 | 0.00345 | 0.0179093 |
| AT4G33980 | BEST Arabidopsis thaliana protein match is: cold regulated gene 27 (TAIR:AT5G42900.2) | 53.8921 | 22.3509 | -1.26974 | 0.0092 | 0.0370545 |
| AT5G42570 | B-cell receptor-associated 31-like | 431.165 | 178.888 | -1.26918 | 0.0027 | 0.0149318 |
| AT1G11910 | APA1, ATAPA1, aspartic proteinase A1 | 491.562 | 204.151 | -1.26774 | 0.00735 | 0.0315405 |
| AT1G80920 | J8, Chaperone DnaJ-domain superfamily protein | 1780.59 | 739.613 | -1.26751 | 0.01245 | 0.0462329 |
| AT3G12030 | Protein of unknown function DUF106, transmembrane | 48.3214 | 20.0925 | -1.266 | 0.0098 | 0.0388625 |
| AT1G10470 | ARR4, ATRR1, IBC7, MEE7, response regulator 4 | 112.292 | 46.7094 | -1.26547 | 0.00395 | 0.0198341 |
| AT5G18130 | unknown protein | 17.1829 | 7.1484 | -1.26528 | 0.0119 | 0.0448549 |
| AT5G37600 | ATGLN1;1, ATGSR1, GLN1;1, GSR 1, glutamine synthase clone R1 | 496.386 | 206.551 | -1.26497 | 0.00745 | 0.0318523 |
| AT5G46340 | O-acetyltransferase family protein | 23.7706 | 9.895 | -1.2644 | 0.0093 | 0.0372979 |
| AT2G31680 | AtRABA5d, RABA5d, RAB GTPase homolog A5D | 53.6824 | 22.3527 | -1.264 | 0.0053 | 0.0248135 |
| AT4G12120 | ATSEC1B, SEC1B, Sec1/munc18-like (SM) proteins superfamily | 61.3372 | 25.5521 | -1.26332 | 0.0031 | 0.0165078 |
| AT2G30490 | ATC4H, C4H, CYP73A5, REF3, cinnamate-4-hydroxylase | 442.154 | 184.208 | -1.26321 | 0.00605 | 0.0273236 |
| AT5G57050 | ABI2, AtABI2, Protein phosphatase 2C family protein | 18.4448 | 7.68494 | -1.26311 | 0.00865 | 0.0354741 |
| AT2G01570 | RGA, RGA1, GRAS family transcription factor family protein | 81.9937 | 34.1963 | -1.26168 | 0.0046 | 0.0222191 |
| AT2G44410 | RING/U-box superfamily protein | 79.0765 | 32.9902 | -1.26121 | 0.00245 | 0.0139065 |
| AT1G28280 | VQ motif-containing protein | 85.7963 | 35.8002 | -1.26095 | 0.0048 | 0.0229834 |
| AT3G52710 | unknown protein | 28.1665 | 11.7538 | -1.26086 | 0.00915 | 0.0369056 |
| AT4G16444 | function unknown | 29.7954 | 12.4395 | -1.26017 | 0.00895 | 0.0364179 |
| AT5G02040 | PRA1.A1, prenylated RAB acceptor 1.A1 | 60.4105 | 25.2234 | -1.26004 | 0.006 | 0.027135 |
| AT2G17450 | RHA3A, RING-H2 finger A3A | 270.99 | 113.16 | -1.25988 | 0.00275 | 0.0151029 |
| AT3G07330 | ATCSLC06, ATCSLC6, CSLC06, CSLC6, Cellulose-synthase-like C6 | 67.8097 | 28.3219 | -1.25958 | 0.0066 | 0.0290976 |
| AT3G42050 | vacuolar ATP synthase subunit H family protein | 468.134 | 195.586 | -1.25912 | 0.0064 | 0.0284694 |
| AT4G08690 | Sec14p-like phosphatidylinositol transfer family protein | 36.8414 | 15.4037 | -1.25805 | 0.00765 | 0.0324899 |
| AT3G51550 | FER, Malectin/receptor-like protein kinase family protein | 253.929 | 106.244 | -1.25704 | 0.0077 | 0.0326253 |
| AT4G26750 | hydroxyproline-rich glycoprotein family protein | 53.6248 | 22.4383 | -1.25694 | 0.00595 | 0.0269704 |
| AT3G25900 | ATHMT-1, HMT-1, Homocysteine S-methyltransferase family protein | 482.327 | 201.825 | -1.25691 | 0.0043 | 0.0211046 |
| AT5G51290 | Diacylglycerol kinase family protein | 20.1573 | 8.44172 | -1.2557 | 0.00605 | 0.0273236 |
| AT1G12840 | ATVHA-C, DET3, vacuolar ATP synthase subunit C (VATC) / V-ATPase C subunit / vacuolar proton pump C subunit (DET3) | 449.47 | 188.256 | -1.25553 | 0.00585 | 0.0267005 |
| AT1G63000 | NRS/ER, UER1, nucleotide-rhamnose synthase/epimerase-reductase | 722.014 | 302.416 | -1.25549 | 0.00805 | 0.0336689 |
| AT2G46505 | SDH4, succinate dehydrogenase subunit 4 | 213.752 | 89.5518 | -1.25514 | 0.00315 | 0.0166977 |
| AT5G11740 | AGP15, ATAGP15, arabinogalactan protein 15 | 1943.79 | 814.374 | -1.25511 | 0.00575 | 0.0263656 |
| AT5G03210 | unknown protein | 364.618 | 152.865 | -1.25413 | 0.00915 | 0.0369056 |
| AT1G75590 | SAUR-like auxin-responsive protein family | 50.1066 | 21.0077 | -1.25408 | 0.00975 | 0.0387185 |
| AT5G43320 | ckl8, casein kinase I-like 8 | 85.6062 | 35.9573 | -1.25143 | 0.0031 | 0.0165078 |
| AT3G52450 | PUB22, plant U-box 22 | 238.403 | 100.138 | -1.25141 | 0.00285 | 0.0154761 |
| AT5G24810 | ABC1 family protein | 82.9763 | 34.8598 | -1.25114 | 0.00515 | 0.024295 |
| AT3G19580 | AZF2, ZF2, zinc-finger protein 2 | 155.351 | 65.2845 | -1.25072 | 0.0042 | 0.0207631 |
| AT1G78300 | 14-3-3OMEGA, GF14 OMEGA, GRF2, general regulatory factor 2 | 243.516 | 102.429 | -1.24939 | 0.00635 | 0.0283169 |
| AT2G30050 | transducin family protein / WD-40 repeat family protein | 127.581 | 53.6666 | -1.24932 | 0.0035 | 0.0180741 |
| AT2G30410 | KIS, TFCA, tubulin folding cofactor A (KIESEL) | 99.6529 | 41.9471 | -1.24834 | 0.00665 | 0.0292594 |
| AT3G26690 | ATNUDT13, ATNUDX13, NUDX13, nudix hydrolase homolog 13 | 260.17 | 109.635 | -1.24674 | 0.005 | 0.0237003 |
| AT5G37260 | CIR1, RVE2, Homeodomain-like superfamily protein | 121.514 | 51.2159 | -1.24646 | 0.009 | 0.0365462 |
| AT5G42880 | Plant protein of unknown function (DUF827) | 30.1737 | 12.7177 | -1.24645 | 0.00465 | 0.0223951 |
| AT3G02840 | ARM repeat superfamily protein | 73.0916 | 30.8079 | -1.2464 | 0.0046 | 0.0222191 |
| AT5G47620 | RNA-binding (RRM/RBD/RNP motifs) family protein | 125.943 | 53.0924 | -1.24619 | 0.00275 | 0.0151029 |
| AT3G11950 | TRAF-like superfamily protein | 21.5951 | 9.10515 | -1.24595 | 0.0091 | 0.0368164 |
| AT1G08830 | CSD1, copper/zinc superoxide dismutase 1 | 966.124 | 407.377 | -1.24584 | 0.0081 | 0.0338066 |
| AT2G20760 | Clathrin light chain protein | 243.472 | 102.675 | -1.24567 | 0.00625 | 0.0280351 |
| AT4G10040 | CYTC-2, cytochrome c-2 | 281.489 | 118.794 | -1.24462 | 0.0034 | 0.01771 |
| AT5G15640 | Mitochondrial substrate carrier family protein | 93.207 | 39.3618 | -1.24364 | 0.00355 | 0.0182751 |
| AT1G70780 | unknown protein | 1068.35 | 451.218 | -1.24349 | 0.0086 | 0.0353203 |
| AT2G47970 | Nuclear pore localisation protein NPL4 | 65.4127 | 27.6397 | -1.24283 | 0.0048 | 0.0229834 |
| AT5G24260 | prolyl oligopeptidase family protein | 168.142 | 71.0576 | -1.24262 | 0.0035 | 0.0180741 |
| AT3G05700 | Drought-responsive family protein | 28.8342 | 12.189 | -1.2422 | 0.01215 | 0.0454426 |
| AT4G29350 | PFN2, PRF2, PRO2, profilin 2 | 688.303 | 291.369 | -1.2402 | 0.0036 | 0.0184414 |
| AT1G11020 | RING/FYVE/PHD zinc finger superfamily protein | 48.4115 | 20.5031 | -1.23951 | 0.0043 | 0.0211046 |
| AT3G13610 | 2-oxoglutarate (2OG) and Fe(II)-dependent oxygenase superfamily protein | 73.298 | 31.0472 | -1.23931 | 0.0128 | 0.0471875 |
| AT4G35770 | ATSEN1, DIN1, SEN1, SEN1, Rhodanese/Cell cycle control phosphatase superfamily protein | 61.4599 | 26.0504 | -1.23834 | 0.0114 | 0.0433581 |
| AT3G63220 | Galactose oxidase/kelch repeat superfamily protein | 20.9563 | 8.88342 | -1.2382 | 0.0135 | 0.0489749 |
| AT2G38230 | ATPDX1.1, PDX1.1, pyridoxine biosynthesis 1.1 | 53.0601 | 22.4962 | -1.23795 | 0.00805 | 0.0336689 |
| AT5G58380 | CIPK10, PKS2, SIP1, SNRK3.8, SOS3-interacting protein 1 | 90.1232 | 38.2209 | -1.23754 | 0.00315 | 0.0166977 |
| AT1G55310 | ATSCL33, SCL33, SR33, SC35-like splicing factor 33 | 62.6006 | 26.561 | -1.23687 | 0.0129 | 0.0474149 |
| AT5G39510 | ATVTI11, ATVTI1A, SGR4, VTI11, VTI1A, ZIG, ZIG1, Vesicle transport v-SNARE family protein | 192.795 | 81.8063 | -1.23678 | 0.00245 | 0.0139065 |
| AT1G70160 | unknown protein | 61.2197 | 26.0033 | -1.2353 | 0.00475 | 0.0228046 |
| AT1G18460 | alpha/beta-Hydrolases superfamily protein | 142.281 | 60.435 | -1.23529 | 0.00495 | 0.0235479 |
| AT3G58730 | vacuolar ATP synthase subunit D (VATD) / V-ATPase D subunit / vacuolar proton pump D subunit (VATPD) | 376.431 | 159.899 | -1.23522 | 0.00425 | 0.0209475 |
| AT3G18830 | ATPLT5, ATPMT5, PMT5, polyol/monosaccharide transporter 5 | 35.1791 | 14.9441 | -1.23514 | 0.00625 | 0.0280351 |
| AT1G29640 | Protein of unknown function, DUF584 | 219.314 | 93.2016 | -1.23457 | 0.00515 | 0.024295 |
| AT1G14780 | MAC/Perforin domain-containing protein | 50.9473 | 21.6532 | -1.23442 | 0.00555 | 0.0256744 |
| AT4G38420 | sks9, SKU5 similar 9 | 11.0293 | 4.69104 | -1.23336 | 0.0127 | 0.0469062 |
| AT4G11860 | Protein of unknown function (DUF544) | 75.0775 | 31.9417 | -1.23294 | 0.00435 | 0.0212815 |
| AT2G33470 | ATGLTP1, GLTP1, glycolipid transfer protein 1 | 193.806 | 82.4859 | -1.23239 | 0.0039 | 0.0196279 |
| AT3G12630 | A20/AN1-like zinc finger family protein | 222.784 | 94.833 | -1.23218 | 0.0029 | 0.0157089 |
| AT4G21105 | cytochrome-c oxidases;electron carriers | 917.17 | 390.419 | -1.23217 | 0.0031 | 0.0165078 |
| AT2G33100 | ATCSLD1, CSLD1, CSLD1, cellulose synthase-like D1 | 9.06158 | 3.85812 | -1.23186 | 0.01245 | 0.0462329 |
| AT4G20260 | ATPCAP1, PCAP1, plasma-membrane associated cation-binding protein 1 | 225.514 | 96.1149 | -1.23038 | 0.00915 | 0.0369056 |
| AT2G01670 | atnudt17, NUDT17, nudix hydrolase homolog 17 | 197.421 | 84.1543 | -1.23017 | 0.00305 | 0.0163293 |
| AT1G14530 | THH1, Protein of unknown function (DUF1084) | 39.2488 | 16.7323 | -1.23001 | 0.0069 | 0.0300655 |
| AT5G22350 | ELM1, Protein of unknown function (DUF1022) | 63.7546 | 27.18 | -1.22998 | 0.0042 | 0.0207631 |
| AT1G24440 | RING/U-box superfamily protein | 47.8712 | 20.4145 | -1.22956 | 0.0077 | 0.0326253 |
| AT1G20450 | ERD10, LTI29, LTI45, Dehydrin family protein | 2026.31 | 864.185 | -1.22944 | 0.01365 | 0.0494285 |
| AT5G47570 | unknown protein | 285.769 | 121.953 | -1.22853 | 0.0037 | 0.0188077 |
| AT3G49780 | ATPSK3 (FORMER SYMBOL), ATPSK4, PSK4, phytosulfokine 4 precursor | 1411.84 | 602.512 | -1.22851 | 0.00685 | 0.0299134 |
| AT4G12040 | A20/AN1-like zinc finger family protein | 499.133 | 213.044 | -1.22827 | 0.00405 | 0.0202184 |
| AT1G66270 | BGLU21, Glycosyl hydrolase superfamily protein | 392.327 | 167.493 | -1.22796 | 0.0106 | 0.0411532 |
| AT4G35100 | PIP2;7, PIP3, PIP3A, SIMIP, plasma membrane intrinsic protein 3 | 712.54 | 304.242 | -1.22775 | 0.01035 | 0.0404522 |
| AT5G52990 | SNARE-like superfamily protein | 22.5286 | 9.62474 | -1.22694 | 0.0108 | 0.041652 |
| AT3G63380 | ATPase E1-E2 type family protein / haloacid dehalogenase-like hydrolase family protein | 85.7103 | 36.6285 | -1.2265 | 0.0052 | 0.0244668 |
| AT4G30600 | signal recognition particle receptor alpha subunit family protein | 342.377 | 146.458 | -1.2251 | 0.00705 | 0.0305176 |
| AT3G54620 | ATBZIP25, BZIP25, BZO2H4, basic leucine zipper 25 | 185.18 | 79.2276 | -1.22485 | 0.0034 | 0.01771 |
| AT1G74840 | Homeodomain-like superfamily protein | 705.371 | 302.081 | -1.22345 | 0.00665 | 0.0292594 |
| AT1G50640 | ATERF3, ERF3, ethylene responsive element binding factor 3 | 121.806 | 52.18 | -1.22301 | 0.00425 | 0.0209475 |
| AT4G39780 | Integrase-type DNA-binding superfamily protein | 154.394 | 66.145 | -1.22291 | 0.00585 | 0.0267005 |
| AT5G46420 | 16S rRNA processing protein RimM family | 67.7567 | 29.0512 | -1.22177 | 0.0029 | 0.0157089 |
| AT1G58290 | HEMA1, Glutamyl-tRNA reductase family protein | 127.17 | 54.5355 | -1.2215 | 0.0041 | 0.0203755 |
| AT4G13010 | Oxidoreductase, zinc-binding dehydrogenase family protein | 76.4882 | 32.8095 | -1.22113 | 0.00635 | 0.0283169 |
| AT3G05210 | ERCC1, UVR7, nucleotide repair protein, putative | 22.4295 | 9.62639 | -1.22033 | 0.008 | 0.0335306 |
| AT4G15410 | PUX5, serine/threonine protein phosphatase 2A 55 kDa regulatory subunit B prime gamma | 73.8014 | 31.6752 | -1.22029 | 0.0051 | 0.0241339 |
| AT2G34650 | ABR, PID, Protein kinase superfamily protein | 28.9916 | 12.4472 | -1.21981 | 0.00825 | 0.0342522 |
| AT4G12020 | ATWRKY19, MAPKKK11, MEKK4, WRKY19, protein kinase family protein | 32.5696 | 13.9837 | -1.21978 | 0.00655 | 0.0289545 |
| AT5G01980 | RING/U-box superfamily protein | 29.2821 | 12.5752 | -1.21944 | 0.00865 | 0.0354741 |
| AT1G09932 | Phosphoglycerate mutase family protein | 66.0425 | 28.3703 | -1.21901 | 0.00625 | 0.0280351 |
| AT3G11580 | AP2/B3-like transcriptional factor family protein | 94.9186 | 40.8246 | -1.21725 | 0.0072 | 0.0310516 |
| AT2G41010 | ATCAMBP25, CAMBP25, calmodulin (CAM)-binding protein of 25 kDa | 99.5977 | 42.8458 | -1.21696 | 0.0069 | 0.0300655 |
| AT4G38730 | Protein of unknown function (DUF803) | 35.0408 | 15.0826 | -1.21615 | 0.01015 | 0.0398672 |
| AT2G45980 | unknown protein | 161.908 | 69.7028 | -1.21589 | 0.00525 | 0.0246552 |
| AT4G13660 | ATPRR2, PRR2, pinoresinol reductase 2 | 43.3578 | 18.6721 | -1.21541 | 0.0092 | 0.0370545 |
| AT3G50110 | ATPEN3, PEN3, PTEN 3 | 22.809 | 9.82651 | -1.21485 | 0.0125 | 0.0463577 |
| AT3G48570 | secE/sec61-gamma protein transport protein | 225.697 | 97.2641 | -1.21441 | 0.0052 | 0.0244668 |
| AT5G24620 | Pathogenesis-related thaumatin superfamily protein | 136.189 | 58.7006 | -1.21416 | 0.0082 | 0.0341377 |
| AT3G23180 | HR-like lesion-inducing protein-related | 126.77 | 54.6428 | -1.21411 | 0.00645 | 0.0286275 |
| AT1G77000 | ATSKP2;2, SKP2B, RNI-like superfamily protein | 160.574 | 69.242 | -1.21352 | 0.0053 | 0.0248135 |
| AT2G22760 | basic helix-loop-helix (bHLH) DNA-binding superfamily protein | 110.257 | 47.5466 | -1.21346 | 0.00675 | 0.0295876 |
| AT4G32600 | RING/U-box superfamily protein | 62.3853 | 26.9047 | -1.21335 | 0.00535 | 0.025018 |
| AT4G33467 | unknown protein | 503.939 | 217.403 | -1.21287 | 0.00685 | 0.0299134 |
| AT4G01550 | anac069, NAC069, NAC domain containing protein 69 | 18.2169 | 7.86108 | -1.21248 | 0.01335 | 0.0486446 |
| AT5G11960 | Protein of unknown function (DUF803) | 93.4594 | 40.3331 | -1.21238 | 0.0042 | 0.0207631 |
| AT1G70410 | ATBCA4, BCA4, CA4, beta carbonic anhydrase 4 | 610.017 | 263.263 | -1.21235 | 0.0106 | 0.0411532 |
| AT2G01260 | Protein of unknown function (DUF789) | 28.4979 | 12.2997 | -1.21224 | 0.0075 | 0.0319763 |
| AT2G25610 | ATPase, F0/V0 complex, subunit C protein | 139.097 | 60.0439 | -1.212 | 0.00435 | 0.0212815 |
| AT3G12400 | ATELC, ELC, Ubiquitin-conjugating enzyme/RWD-like protein | 103.913 | 44.8979 | -1.21066 | 0.0049 | 0.0233549 |
| AT4G15940 | Fumarylacetoacetate (FAA) hydrolase family | 69.6264 | 30.0887 | -1.21041 | 0.00555 | 0.0256744 |
| AT3G60300 | RWD domain-containing protein | 113.674 | 49.1471 | -1.20972 | 0.00455 | 0.0220583 |
| AT4G16760 | ACX1, ATACX1, acyl-CoA oxidase 1 | 494.698 | 214.022 | -1.20879 | 0.01075 | 0.0415725 |
| AT2G46790 | APRR9, PRR9, TL1, pseudo-response regulator 9 | 39.5027 | 17.1016 | -1.20782 | 0.00625 | 0.0280351 |
| AT2G36810 | ARM repeat superfamily protein | 23.5163 | 10.1918 | -1.20625 | 0.00465 | 0.0223951 |
| AT4G38950 | ATP binding microtubule motor family protein | 34.8146 | 15.0918 | -1.20593 | 0.00485 | 0.0231612 |
| AT4G05050 | UBQ11, ubiquitin 11 | 407.234 | 176.662 | -1.20486 | 0.0138 | 0.0497896 |
| AT4G24400 | ATCIPK8, CIPK8, PKS11, SnRK3.13, CBL-interacting protein kinase 8 | 86.0133 | 37.3208 | -1.20458 | 0.0056 | 0.0258574 |
| AT3G02700 | NC domain-containing protein-related | 80.4128 | 34.9104 | -1.20377 | 0.0064 | 0.0284694 |
| AT5G12010 | unknown protein | 479.603 | 208.224 | -1.2037 | 0.00745 | 0.0318523 |
| AT2G17550 | unknown protein | 39.0094 | 16.9374 | -1.20361 | 0.00765 | 0.0324899 |
| AT4G33905 | Peroxisomal membrane 22 kDa (Mpv17/PMP22) family protein | 57.5654 | 25.0099 | -1.2027 | 0.00565 | 0.0260215 |
| AT4G00720 | ASKTHETA, ATSK32, SK32, shaggy-like protein kinase 32 | 135.23 | 58.7615 | -1.20247 | 0.00595 | 0.0269704 |
| AT3G01400 | ARM repeat superfamily protein | 21.6041 | 9.39501 | -1.20134 | 0.01265 | 0.0467826 |
| AT1G71950 | Proteinase inhibitor, propeptide | 492.082 | 214.177 | -1.2001 | 0.01175 | 0.0443994 |
| AT3G59090 | CONTAINS InterPro DOMAIN/s: Protein of unknown function DUF1084 (InterPro:IPR009457) | 64.0939 | 27.903 | -1.19977 | 0.00775 | 0.0327601 |
| AT3G62290 | ARFA1E, ATARFA1E, ADP-ribosylation factor A1E | 1398.18 | 608.985 | -1.19907 | 0.00705 | 0.0305176 |
| AT3G13275 | unknown protein | 50.544 | 22.0179 | -1.19886 | 0.01265 | 0.0467826 |
| AT3G21630 | CERK1, LYSM RLK1, chitin elicitor receptor kinase 1 | 88.2421 | 38.4498 | -1.19849 | 0.0052 | 0.0244668 |
| AT2G18160 | ATBZIP2, bZIP2, GBF5, basic leucine-zipper 2 | 267.082 | 116.389 | -1.19833 | 0.0066 | 0.0290976 |
| AT1G15470 | Transducin/WD40 repeat-like superfamily protein | 29.1532 | 12.7097 | -1.19773 | 0.00965 | 0.0383598 |
| AT5G61390 | Polynucleotidyl transferase, ribonuclease H-like superfamily protein | 55.2953 | 24.1082 | -1.19763 | 0.0062 | 0.0278676 |
| AT4G01900 | GLB1, PII, GLNB1 homolog | 54.5408 | 23.7929 | -1.19681 | 0.0091 | 0.0368164 |
| AT3G51790 | ATG1, TG1, transmembrane protein G1P-related 1 | 25.4184 | 11.0929 | -1.19624 | 0.01345 | 0.0488831 |
| AT2G20960 | pEARLI4, Arabidopsis phospholipase-like protein (PEARLI 4) family | 88.4976 | 38.6291 | -1.19595 | 0.0061 | 0.0274993 |
| AT4G39980 | DHS1, 3-deoxy-D-arabino-heptulosonate 7-phosphate synthase 1 | 250.454 | 109.341 | -1.19571 | 0.00745 | 0.0318523 |
| AT5G65430 | 14-3-3KAPPA, GF14 KAPPA, GRF8, general regulatory factor 8 | 345.536 | 150.871 | -1.19552 | 0.0059 | 0.0268421 |
| AT2G36300 | Integral membrane Yip1 family protein | 40.7937 | 17.8175 | -1.19505 | 0.00835 | 0.0345732 |
| AT3G61430 | ATPIP1, PIP1, PIP1;1, PIP1A, plasma membrane intrinsic protein 1A | 258.699 | 113.041 | -1.19443 | 0.0063 | 0.0281701 |
| AT4G01370 | ATMPK4, MPK4, MAP kinase 4 | 67.5353 | 29.5181 | -1.19404 | 0.00425 | 0.0209475 |
| AT1G02360 | Chitinase family protein | 162.167 | 70.8857 | -1.19392 | 0.00515 | 0.024295 |
| AT5G17910 | unknown protein | 32.2005 | 14.0885 | -1.19256 | 0.005 | 0.0237003 |
| AT5G26990 | Drought-responsive family protein | 63.2206 | 27.6831 | -1.19139 | 0.00695 | 0.0302036 |
| AT3G13930 | Dihydrolipoamide acetyltransferase, long form protein | 248.711 | 108.991 | -1.19026 | 0.0085 | 0.0349894 |
| AT2G18760 | CHR8, chromatin remodeling 8 | 15.6256 | 6.84883 | -1.18998 | 0.00685 | 0.0299134 |
| AT3G18410 | Complex I subunit NDUFS6 | 443.05 | 194.289 | -1.18926 | 0.01065 | 0.0412906 |
| AT3G51840 | ACX4, ATG6, ATSCX, acyl-CoA oxidase 4 | 132.709 | 58.198 | -1.18922 | 0.00575 | 0.0263656 |
| AT3G05320 | O-fucosyltransferase family protein | 47.7753 | 20.9784 | -1.18736 | 0.0077 | 0.0326253 |
| AT1G16840 | unknown protein | 321.579 | 141.243 | -1.18699 | 0.0078 | 0.0329152 |
| AT3G57630 | exostosin family protein | 40.6597 | 17.859 | -1.18695 | 0.0105 | 0.0408771 |
| AT5G14540 | Protein of unknown function (DUF1421) | 54.5585 | 23.9687 | -1.18665 | 0.0064 | 0.0284694 |
| AT3G10920 | ATMSD1, MEE33, MSD1, manganese superoxide dismutase 1 | 353.627 | 155.362 | -1.18659 | 0.0059 | 0.0268421 |
| AT1G53380 | Plant protein of unknown function (DUF641) | 204.34 | 89.7754 | -1.18658 | 0.005 | 0.0237003 |
| AT5G08535 | D111/G-patch domain-containing protein | 64.4844 | 28.3386 | -1.18618 | 0.01175 | 0.0443994 |
| AT3G14770 | Nodulin MtN3 family protein | 52.9303 | 23.2679 | -1.18575 | 0.00825 | 0.0342522 |
| AT1G80360 | Pyridoxal phosphate (PLP)-dependent transferases superfamily protein | 51.4259 | 22.6069 | -1.18573 | 0.00695 | 0.0302036 |
| AT3G11420 | Protein of unknown function (DUF604) | 46.2253 | 20.3288 | -1.18516 | 0.0083 | 0.0344309 |
| AT1G55530 | RING/U-box superfamily protein | 187.9 | 82.6831 | -1.1843 | 0.0074 | 0.0317207 |
| AT1G49140 | Complex I subunit NDUFS6 | 167.433 | 73.6867 | -1.18411 | 0.00655 | 0.0289545 |
| AT1G10150 | Carbohydrate-binding protein | 122.213 | 53.7906 | -1.18398 | 0.0065 | 0.0287784 |
| AT2G45010 | PLAC8 family protein | 78.4777 | 34.5411 | -1.18397 | 0.009 | 0.0365462 |
| AT1G73250 | ATFX, GER1, GDP-4-keto-6-deoxymannose-3,5-epimerase-4-reductase 1 | 73.8429 | 32.5125 | -1.18346 | 0.009 | 0.0365462 |
| AT5G18800 | Cox19-like CHCH family protein | 233.45 | 102.954 | -1.18111 | 0.0068 | 0.029741 |
| AT2G32850 | Protein kinase superfamily protein | 48.8521 | 21.5533 | -1.18051 | 0.00795 | 0.0333705 |
| AT1G10650 | SBP (S-ribonuclease binding protein) family protein | 77.4289 | 34.163 | -1.18044 | 0.00445 | 0.0217117 |
| AT3G17090 | Protein phosphatase 2C family protein | 133.495 | 58.9309 | -1.17969 | 0.0056 | 0.0258574 |
| AT1G68580 | agenet domain-containing protein / bromo-adjacent homology (BAH) domain-containing protein | 115.215 | 50.8815 | -1.17912 | 0.00565 | 0.0260215 |
| AT5G02100 | ORP3A, UNE18, Oxysterol-binding family protein | 44.405 | 19.616 | -1.17869 | 0.00755 | 0.0321272 |
| AT5G34850 | ATPAP26, PAP26, purple acid phosphatase 26 | 175.202 | 77.4234 | -1.17818 | 0.0066 | 0.0290976 |
| AT1G29400 | AML5, ML5, MEI2-like protein 5 | 198.348 | 87.6633 | -1.17799 | 0.00825 | 0.0342522 |
| AT5G19860 | Protein of unknown function, DUF538 | 162.762 | 71.951 | -1.17768 | 0.0049 | 0.0233549 |
| AT5G55850 | NOI, RPM1-interacting protein 4 (RIN4) family protein | 218.099 | 96.4481 | -1.17716 | 0.007 | 0.0303609 |
| AT1G80350 | AAA1, ATKTN1, BOT1, ERH3, FRA2, FRC2, FTR, KTN1, LUE1, P-loop containing nucleoside triphosphate hydrolases superfamily protein | 42.3392 | 18.7238 | -1.17713 | 0.0088 | 0.035933 |
| AT1G73030 | CHMP1A, VPS46.2, SNF7 family protein | 182.181 | 80.5787 | -1.1769 | 0.00535 | 0.025018 |
| AT4G31080 | Protein of unknown function (DUF2296) | 63.3132 | 28.0074 | -1.1767 | 0.00605 | 0.0273236 |
| AT1G17500 | ATPase E1-E2 type family protein / haloacid dehalogenase-like hydrolase family protein | 63.4985 | 28.0971 | -1.1763 | 0.0107 | 0.0414196 |
| AT3G49590 | Autophagy-related protein 13 | 63.7078 | 28.2052 | -1.17551 | 0.0072 | 0.0310516 |
| AT3G11720 | Polyketide cyclase/dehydrase and lipid transport superfamily protein | 20.8497 | 9.2311 | -1.17545 | 0.013 | 0.0477116 |
| AT3G56410 | Protein of unknown function (DUF3133) | 10.2364 | 4.53247 | -1.17534 | 0.00945 | 0.0377542 |
| AT1G36980 | unknown protein | 103.52 | 45.8485 | -1.17496 | 0.011 | 0.0422342 |
| AT5G46860 | ATSYP22, ATVAM3, SGR3, SYP22, VAM3, Syntaxin/t-SNARE family protein | 98.6521 | 43.7071 | -1.17448 | 0.008 | 0.0335306 |
| AT4G34000 | ABF3, DPBF5, abscisic acid responsive elements-binding factor 3 | 100.457 | 44.5271 | -1.17382 | 0.0079 | 0.0332028 |
| AT2G30250 | ATWRKY25, WRKY25, WRKY DNA-binding protein 25 | 107.69 | 47.772 | -1.17265 | 0.006 | 0.027135 |
| AT1G48790 | AMSH1, associated molecule with the SH3 domain of STAM 1 | 64.8856 | 28.7935 | -1.17216 | 0.00405 | 0.0202184 |
| AT4G20830 | FAD-binding Berberine family protein | 611.451 | 271.399 | -1.17182 | 0.0135 | 0.0489749 |
| AT5G08680 | ATP synthase alpha/beta family protein | 97.6736 | 43.3602 | -1.1716 | 0.0064 | 0.0284694 |
| AT1G16180 | Serinc-domain containing serine and sphingolipid biosynthesis protein | 237.602 | 105.555 | -1.17055 | 0.00855 | 0.0351514 |
| AT2G26210 | Ankyrin repeat family protein | 57.2813 | 25.4561 | -1.17005 | 0.0112 | 0.0427699 |
| AT5G52060 | ATBAG1, BAG1, BCL-2-associated athanogene 1 | 101.007 | 44.8949 | -1.16983 | 0.00595 | 0.0269704 |
| AT1G69640 | SBH1, sphingoid base hydroxylase 1 | 132.665 | 58.9873 | -1.16931 | 0.0062 | 0.0278676 |
| AT5G63570 | GSA1, glutamate-1-semialdehyde-2,1-aminomutase | 70.572 | 31.3799 | -1.16925 | 0.00715 | 0.0308561 |
| AT2G23140 | RING/U-box superfamily protein with ARM repeat domain | 28.0964 | 12.4949 | -1.16904 | 0.00865 | 0.0354741 |
| AT4G21790 | ATTOM1, TOM1, tobamovirus multiplication 1 | 246.026 | 109.42 | -1.16894 | 0.0055 | 0.0254967 |
| AT1G52200 | PLAC8 family protein | 41.5399 | 18.4876 | -1.16794 | 0.0124 | 0.046125 |
| AT1G27290 | unknown protein | 379.088 | 168.749 | -1.16766 | 0.00635 | 0.0283169 |
| AT2G30020 | Protein phosphatase 2C family protein | 60.9359 | 27.1335 | -1.16722 | 0.00835 | 0.0345732 |
| AT5G65210 | TGA1, bZIP transcription factor family protein | 153.007 | 68.1556 | -1.1667 | 0.0053 | 0.0248135 |
| AT4G33930 | Cupredoxin superfamily protein | 232.831 | 103.786 | -1.16568 | 0.0081 | 0.0338066 |
| AT3G12150 | unknown protein | 28.1302 | 12.5421 | -1.16534 | 0.01325 | 0.0484049 |
| AT3G07340 | basic helix-loop-helix (bHLH) DNA-binding superfamily protein | 170.155 | 75.8715 | -1.16522 | 0.0054 | 0.0251864 |
| AT1G15110 | phosphatidyl serine synthase family protein | 149.973 | 66.874 | -1.16518 | 0.00525 | 0.0246552 |
| AT1G08480 | unknown protein | 166.663 | 74.3403 | -1.16472 | 0.0102 | 0.0400002 |
| AT2G35680 | Phosphotyrosine protein phosphatases superfamily protein | 160.344 | 71.5481 | -1.16418 | 0.0064 | 0.0284694 |
| AT3G06850 | BCE2, DIN3, LTA1, 2-oxoacid dehydrogenases acyltransferase family protein | 62.2713 | 27.7867 | -1.16417 | 0.00915 | 0.0369056 |
| AT2G21870 | MGP1, copper ion binding;cobalt ion binding;zinc ion binding | 549.933 | 245.482 | -1.16364 | 0.00875 | 0.0357584 |
| AT4G01700 | Chitinase family protein | 328.298 | 146.588 | -1.16324 | 0.0124 | 0.046125 |
| AT3G19970 | alpha/beta-Hydrolases superfamily protein | 103.689 | 46.3211 | -1.16252 | 0.0061 | 0.0274993 |
| AT1G60140 | ATTPS10, TPS10, TPS10, trehalose phosphate synthase | 118.214 | 52.8562 | -1.16126 | 0.006 | 0.027135 |
| AT3G20040 | ATHXK4, HKL2, Hexokinase | 25.0599 | 11.2058 | -1.16113 | 0.01265 | 0.0467826 |
| AT1G61210 | Transducin/WD40 repeat-like superfamily protein | 29.2216 | 13.073 | -1.16045 | 0.0061 | 0.0274993 |
| AT4G26650 | RNA-binding (RRM/RBD/RNP motifs) family protein | 58.2405 | 26.0839 | -1.15886 | 0.01235 | 0.0460168 |
| AT5G67420 | ASL39, LBD37, LOB domain-containing protein 37 | 63.8819 | 28.6128 | -1.15874 | 0.0077 | 0.0326253 |
| AT1G14870 | PCR2, PLANT CADMIUM RESISTANCE 2 | 962.761 | 431.245 | -1.15867 | 0.0108 | 0.041652 |
| AT5G42010 | Transducin/WD40 repeat-like superfamily protein | 46.0646 | 20.6466 | -1.15776 | 0.0078 | 0.0329152 |
| AT3G59360 | ATUTR6, UTR6, UDP-galactose transporter 6 | 77.7428 | 34.848 | -1.15764 | 0.00985 | 0.0390139 |
| AT3G52300 | ATPQ, ATP synthase D chain, mitochondrial | 984.633 | 441.361 | -1.15763 | 0.01245 | 0.0462329 |
| AT1G23170 | Protein of unknown function DUF2359, transmembrane | 57.0863 | 25.5955 | -1.15725 | 0.0108 | 0.041652 |
| AT1G23710 | Protein of unknown function (DUF1645) | 195.97 | 87.8665 | -1.15725 | 0.0084 | 0.0346931 |
| AT2G26600 | Glycosyl hydrolase superfamily protein | 35.2209 | 15.7922 | -1.15722 | 0.012 | 0.0450518 |
| AT3G10860 | Cytochrome b-c1 complex, subunit 8 protein | 570.387 | 255.834 | -1.15674 | 0.00675 | 0.0295876 |
| AT5G11850 | Protein kinase superfamily protein | 61.4303 | 27.5569 | -1.15654 | 0.00775 | 0.0327601 |
| AT1G03370 | C2 calcium/lipid-binding and GRAM domain containing protein | 98.8063 | 44.3384 | -1.15605 | 0.0096 | 0.0382301 |
| AT3G55720 | Protein of unknown function (DUF620) | 475.965 | 213.649 | -1.15561 | 0.01215 | 0.0454426 |
| AT3G02340 | RING/U-box superfamily protein | 52.0351 | 23.3717 | -1.15472 | 0.01 | 0.0393953 |
| AT1G59590 | ZCF37, ZCF37 | 63.6587 | 28.5954 | -1.15457 | 0.0084 | 0.0346931 |
| AT2G19572 | Potential natural antisense gene, locus overlaps with AT2G19570 | 182.798 | 82.1952 | -1.15312 | 0.0124 | 0.046125 |
| AT1G15120 | Ubiquinol-cytochrome C reductase hinge protein | 646.515 | 290.731 | -1.153 | 0.00675 | 0.0295876 |
| AT4G00585 | unknown protein | 267.342 | 120.274 | -1.15235 | 0.0085 | 0.0349894 |
| AT1G74380 | XXT5, xyloglucan xylosyltransferase 5 | 121.985 | 54.8819 | -1.1523 | 0.00725 | 0.03124 |
| AT3G63310 | BIL4, Bax inhibitor-1 family protein | 254.081 | 114.318 | -1.15223 | 0.0103 | 0.0403045 |
| AT1G77130 | GUX3, PGSIP2, plant glycogenin-like starch initiation protein 2 | 52.4954 | 23.6296 | -1.1516 | 0.0082 | 0.0341377 |
| AT5G05850 | PIRL1, plant intracellular ras group-related LRR 1 | 46.5866 | 20.971 | -1.15152 | 0.01005 | 0.0395765 |
| AT5G65290 | LMBR1-like membrane protein | 58.8752 | 26.5078 | -1.15124 | 0.0059 | 0.0268421 |
| AT5G16120 | alpha/beta-Hydrolases superfamily protein | 66.195 | 29.8132 | -1.15077 | 0.0115 | 0.0436881 |
| AT5G02170 | Transmembrane amino acid transporter family protein | 101.152 | 45.575 | -1.15021 | 0.0079 | 0.0332028 |
| AT3G52200 | LTA3, Dihydrolipoamide acetyltransferase, long form protein | 148.89 | 67.1345 | -1.14913 | 0.00555 | 0.0256744 |
| AT5G06320 | NHL3, NDR1/HIN1-like 3 | 659.23 | 297.317 | -1.14878 | 0.0112 | 0.0427699 |
| AT2G23780 | RING/U-box superfamily protein | 71.819 | 32.401 | -1.14833 | 0.01045 | 0.0407225 |
| AT5G02800 | Protein kinase superfamily protein | 33.077 | 14.9237 | -1.14823 | 0.0077 | 0.0326253 |
| AT4G20410 | GAMMA-SNAP, GSNAP, gamma-soluble NSF attachment protein | 60.8366 | 27.4976 | -1.14564 | 0.00895 | 0.0364179 |
| AT5G63910 | FCLY, farnesylcysteine lyase | 58.6875 | 26.5321 | -1.14532 | 0.0069 | 0.0300655 |
| AT3G54300 | ATVAMP727, VAMP727, VAMP727, vesicle-associated membrane protein 727 | 130.374 | 58.9712 | -1.14458 | 0.00785 | 0.0330699 |
| AT5G63260 | Zinc finger C-x8-C-x5-C-x3-H type family protein | 63.4627 | 28.706 | -1.14456 | 0.0063 | 0.0281701 |
| AT1G22620 | ATSAC1, Phosphoinositide phosphatase family protein | 28.7029 | 12.9851 | -1.14434 | 0.0069 | 0.0300655 |
| AT5G59820 | RHL41, ZAT12, C2H2-type zinc finger family protein | 601.598 | 272.254 | -1.14385 | 0.0066 | 0.0290976 |
| AT4G22212 | Arabidopsis defensin-like protein | 138.844 | 62.8338 | -1.14385 | 0.00895 | 0.0364179 |
| AT1G29690 | CAD1, MAC/Perforin domain-containing protein | 74.6177 | 33.786 | -1.14309 | 0.0079 | 0.0332028 |
| AT2G37250 | ADK, ATPADK1, adenosine kinase | 180.082 | 81.5465 | -1.14296 | 0.0104 | 0.0405756 |
| AT5G48930 | HCT, hydroxycinnamoyl-CoA shikimate/quinate hydroxycinnamoyl transferase | 65.5914 | 29.7136 | -1.14238 | 0.01025 | 0.0401565 |
| AT5G39660 | CDF2, cycling DOF factor 2 | 62.8389 | 28.4673 | -1.14235 | 0.0099 | 0.0391493 |
| AT5G50850 | MAB1, Transketolase family protein | 404.942 | 183.45 | -1.14233 | 0.009 | 0.0365462 |
| AT3G27020 | YSL6, YELLOW STRIPE like 6 | 31.3183 | 14.19 | -1.14213 | 0.00825 | 0.0342522 |
| AT5G04920 | EAP30/Vps36 family protein | 63.3766 | 28.7155 | -1.14212 | 0.00595 | 0.0269704 |
| AT4G25650 | ACD1-LIKE, PTC52, TIC55-IV, ACD1-like | 33.9499 | 15.3832 | -1.14205 | 0.012 | 0.0450518 |
| AT1G35160 | 14-3-3PHI, GF14 PHI, GRF4, GF14 protein phi chain | 396.066 | 179.483 | -1.14189 | 0.01055 | 0.0410315 |
| AT5G45130 | ATRAB-F2A, ATRAB5A, ATRABF2A, RAB-F2A, RAB5A, RABF2A, RHA1, RAB homolog 1 | 199.808 | 90.5785 | -1.14138 | 0.0077 | 0.0326253 |
| AT3G08610 | unknown protein | 830.467 | 376.513 | -1.14122 | 0.0066 | 0.0290976 |
| AT2G15620 | ATHNIR, NIR, NIR1, nitrite reductase 1 | 87.0021 | 39.4568 | -1.14078 | 0.0129 | 0.0474149 |
| AT1G54710 | ATATG18H, ATG18H, homolog of yeast autophagy 18 (ATG18) H | 73.9124 | 33.5335 | -1.14021 | 0.00645 | 0.0286275 |
| AT1G56280 | ATDI19, DI19, drought-induced 19 | 205.648 | 93.3107 | -1.14007 | 0.00915 | 0.0369056 |
| AT4G38940 | Galactose oxidase/kelch repeat superfamily protein | 40.418 | 18.3447 | -1.13963 | 0.0094 | 0.0376304 |
| AT1G72150 | PATL1, PATELLIN 1 | 106.601 | 48.4544 | -1.13753 | 0.01225 | 0.045713 |
| AT4G38220 | Peptidase M20/M25/M40 family protein | 70.357 | 32.0089 | -1.13622 | 0.01175 | 0.0443994 |
| AT2G41740 | ATVLN2, VLN2, villin 2 | 148 | 67.3669 | -1.13549 | 0.0112 | 0.0427699 |
| AT2G22500 | ATPUMP5, DIC1, UCP5, uncoupling protein 5 | 82.0692 | 37.3739 | -1.13481 | 0.0073 | 0.0313803 |
| AT1G61100 | disease resistance protein (TIR class), putative | 82.9566 | 37.8302 | -1.13282 | 0.00865 | 0.0354741 |
| AT3G25250 | AGC2, AGC2-1, AtOXI1, OXI1, AGC (cAMP-dependent, cGMP-dependent and protein kinase C) kinase family protein | 114.308 | 52.1866 | -1.13117 | 0.00935 | 0.037453 |
| AT4G25130 | PMSR4, peptide met sulfoxide reductase 4 | 104.405 | 47.6701 | -1.13103 | 0.00965 | 0.0383598 |
| AT5G67590 | FRO1, NADH-ubiquinone oxidoreductase-related | 361.413 | 165.16 | -1.12979 | 0.0072 | 0.0310516 |
| AT3G55950 | ATCRR3, CCR3, CRINKLY4 related 3 | 28.3315 | 12.9513 | -1.12931 | 0.00955 | 0.0380846 |
| AT5G13440 | Ubiquinol-cytochrome C reductase iron-sulfur subunit | 88.4102 | 40.4281 | -1.12885 | 0.0095 | 0.0379387 |
| AT4G17170 | AT-RAB2, ATRAB-B1B, ATRAB2A, ATRABB1C, RAB-B1B, RAB2A, RABB1C, RAB GTPase homolog B1C | 170.336 | 77.9387 | -1.12797 | 0.01045 | 0.0407225 |
| AT5G20680 | TBL16, TRICHOME BIREFRINGENCE-LIKE 16 | 37.4288 | 17.1269 | -1.12788 | 0.00985 | 0.0390139 |
| AT1G08920 | ESL1, ERD (early response to dehydration) six-like 1 | 254.934 | 116.69 | -1.12745 | 0.01 | 0.0393953 |
| AT4G28350 | Concanavalin A-like lectin protein kinase family protein | 25.872 | 11.8471 | -1.12686 | 0.00945 | 0.0377542 |
| AT1G05850 | ATCTL1, CTL1, ELP, ELP1, ERH2, HOT2, POM1, Chitinase family protein | 359.024 | 164.579 | -1.1253 | 0.01285 | 0.0472926 |
| AT5G54810 | ATTSB1, TRP2, TRPB, TSB1, tryptophan synthase beta-subunit 1 | 408.726 | 187.42 | -1.12486 | 0.00905 | 0.0366741 |
| AT4G10140 | unknown protein | 88.7803 | 40.7406 | -1.12377 | 0.01215 | 0.0454426 |
| AT1G29760 | Putative adipose-regulatory protein (Seipin) | 53.1879 | 24.4435 | -1.12165 | 0.01 | 0.0393953 |
| AT5G51450 | RIN3, RPM1 interacting protein 3 | 23.5765 | 10.8369 | -1.12139 | 0.0137 | 0.0495371 |
| AT5G57610 | Protein kinase superfamily protein with octicosapeptide/Phox/Bem1p domain | 50.1205 | 23.0448 | -1.12096 | 0.0091 | 0.0368164 |
| AT5G37480 | unknown protein | 90.0775 | 41.4186 | -1.12089 | 0.0093 | 0.0372979 |
| AT5G04460 | RING/U-box superfamily protein | 47.1495 | 21.6808 | -1.12082 | 0.00955 | 0.0380846 |
| AT5G05570 | transducin family protein / WD-40 repeat family protein | 30.3129 | 13.9541 | -1.11925 | 0.00985 | 0.0390139 |
| AT3G56880 | VQ motif-containing protein | 361.289 | 166.446 | -1.1181 | 0.0073 | 0.0313803 |
| AT3G57450 | unknown protein | 114.079 | 52.5734 | -1.11763 | 0.0107 | 0.0414196 |
| AT5G42300 | UBL5, ubiquitin-like protein 5 | 614.662 | 283.277 | -1.11758 | 0.01105 | 0.0423687 |
| AT1G65430 | ARI8, ATARI8, IBR domain-containing protein | 48.0461 | 22.1443 | -1.11748 | 0.0102 | 0.0400002 |
| AT2G01180 | ATLPP1, ATPAP1, LPP1, PAP1, phosphatidic acid phosphatase 1 | 40.5073 | 18.6739 | -1.11716 | 0.01185 | 0.0447431 |
| AT3G59920 | ATGDI2, GDI2, RAB GDP dissociation inhibitor 2 | 271.789 | 125.298 | -1.11712 | 0.0098 | 0.0388625 |
| AT1G12360 | KEU, Sec1/munc18-like (SM) proteins superfamily | 142.14 | 65.539 | -1.11688 | 0.0116 | 0.0439836 |
| AT4G18140 | SSP4b, SCP1-like small phosphatase 4b | 83.5229 | 38.5121 | -1.11686 | 0.01075 | 0.0415725 |
| AT4G14230 | CBS domain-containing protein with a domain of unknown function (DUF21) | 18.9351 | 8.75896 | -1.11223 | 0.0138 | 0.0497896 |
| AT1G18470 | Transmembrane Fragile-X-F-associated protein | 104.712 | 48.4471 | -1.11194 | 0.00745 | 0.0318523 |
| AT2G46540 | unknown protein | 291.699 | 134.979 | -1.11174 | 0.0133 | 0.048507 |
| AT5G12200 | PYD2, pyrimidine 2 | 55.3213 | 25.5991 | -1.11174 | 0.00905 | 0.0366741 |
| AT3G17240 | mtLPD2, lipoamide dehydrogenase 2 | 245.422 | 113.617 | -1.11109 | 0.00845 | 0.0348633 |
| AT3G27960 | Tetratricopeptide repeat (TPR)-like superfamily protein | 62.2265 | 28.8217 | -1.11037 | 0.0082 | 0.0341377 |
| AT3G14590 | NTMC2T6.2, NTMC2TYPE6.2, Calcium-dependent lipid-binding (CaLB domain) family protein | 78.1298 | 36.2022 | -1.1098 | 0.01135 | 0.0432177 |
| AT2G25460 | CONTAINS InterPro DOMAIN/s: C2 calcium-dependent membrane targeting (InterPro:IPR000008) | 148.263 | 68.7616 | -1.10849 | 0.0081 | 0.0338066 |
| AT5G37790 | Protein kinase superfamily protein | 40.1903 | 18.6451 | -1.10805 | 0.01325 | 0.0484049 |
| AT5G28050 | Cytidine/deoxycytidylate deaminase family protein | 515.752 | 239.296 | -1.10788 | 0.0112 | 0.0427699 |
| AT3G12620 | Protein phosphatase 2C family protein | 70.9108 | 32.903 | -1.10778 | 0.009 | 0.0365462 |
| AT1G14450 | NADH dehydrogenase (ubiquinone)s | 180.953 | 83.9771 | -1.10755 | 0.01105 | 0.0423687 |
| AT5G20490 | ATXIK, XI-17, XIK, Myosin family protein with Dil domain | 42.3812 | 19.6762 | -1.10698 | 0.0106 | 0.0411532 |
| AT3G01810 | function unknown | 18.2464 | 8.47447 | -1.10642 | 0.01225 | 0.045713 |
| AT1G11400 | PYM, partner of Y14-MAGO | 107.741 | 50.0477 | -1.10619 | 0.0114 | 0.0433581 |
| AT2G41475 | Embryo-specific protein 3, (ATS3) | 196.753 | 91.4025 | -1.10608 | 0.01165 | 0.0441141 |
| AT5G59570 | Homeodomain-like superfamily protein | 61.3321 | 28.4982 | -1.10577 | 0.0102 | 0.0400002 |
| AT2G05170 | ATVPS11, VPS11, vacuolar protein sorting 11 | 24.2014 | 11.2467 | -1.10559 | 0.01135 | 0.0432177 |
| AT5G26600 | Pyridoxal phosphate (PLP)-dependent transferases superfamily protein | 73.489 | 34.1652 | -1.105 | 0.0099 | 0.0391493 |
| AT4G17230 | SCL13, SCARECROW-like 13 | 166.049 | 77.2147 | -1.10466 | 0.0103 | 0.0403045 |
| AT5G47880 | ERF1-1, eukaryotic release factor 1-1 | 264.006 | 122.814 | -1.10409 | 0.01325 | 0.0484049 |
| AT5G13070 | MSF1-like family protein | 128.529 | 59.8338 | -1.10307 | 0.01015 | 0.0398672 |
| AT5G67320 | HOS15, WD-40 repeat family protein | 65.5406 | 30.5166 | -1.1028 | 0.0093 | 0.0372979 |
| AT4G12250 | GAE5, UDP-D-glucuronate 4-epimerase 5 | 50.134 | 23.4041 | -1.09903 | 0.013 | 0.0477116 |
| AT1G32400 | TOM2A, tobamovirus multiplication 2A | 132.682 | 61.9542 | -1.0987 | 0.0093 | 0.0372979 |
| AT2G20330 | Transducin/WD40 repeat-like superfamily protein | 74.8511 | 34.9562 | -1.09848 | 0.01085 | 0.0417717 |
| AT3G01090 | AKIN10, KIN10, KIN10, SNRK1.1, SNF1 kinase homolog 10 | 50.0675 | 23.3842 | -1.09834 | 0.00905 | 0.0366741 |
| AT5G12430 | Heat shock protein DnaJ with tetratricopeptide repeat | 19.4435 | 9.08701 | -1.09741 | 0.0138 | 0.0497896 |
| AT5G42870 | ATPAH2, PAH2, phosphatidic acid phosphohydrolase 2 | 76.6876 | 35.8462 | -1.09717 | 0.0071 | 0.0306604 |
| AT5G13190 | CONTAINS InterPro DOMAIN/s: LPS-induced tumor necrosis factor alpha factor (InterPro:IPR006629) | 244.012 | 114.144 | -1.0961 | 0.00795 | 0.0333705 |
| AT4G28060 | Cytochrome c oxidase, subunit Vib family protein | 400.379 | 187.316 | -1.09589 | 0.01265 | 0.0467826 |
| AT3G14560 | unknown protein | 87.4144 | 40.957 | -1.09376 | 0.01245 | 0.0462329 |
| AT2G24170 | Endomembrane protein 70 protein family | 52.4348 | 24.573 | -1.09345 | 0.01035 | 0.0404522 |
| AT1G51420 | ATSPP1, SPP1, sucrose-phosphatase 1 | 133.461 | 62.6726 | -1.09051 | 0.0114 | 0.0433581 |
| AT2G25430 | epsin N-terminal homology (ENTH) domain-containing protein / clathrin assembly protein-related | 87.0358 | 40.883 | -1.09011 | 0.01085 | 0.0417717 |
| AT2G18730 | ATDGK3, DGK3, diacylglycerol kinase 3 | 51.7958 | 24.3484 | -1.089 | 0.0099 | 0.0391493 |
| AT1G11820 | O-Glycosyl hydrolases family 17 protein | 25.613 | 12.0497 | -1.08788 | 0.01335 | 0.0486446 |
| AT5G47180 | Plant VAMP (vesicle-associated membrane protein) family protein | 84.7183 | 39.8722 | -1.08729 | 0.0119 | 0.0448549 |
| AT3G61800 | CONTAINS InterPro DOMAIN/s: Protein of unknown function DUF2043 (InterPro:IPR018610), ENTH/VHS (InterPro:IPR008942) | 57.132 | 26.8988 | -1.08676 | 0.0074 | 0.0317207 |
| AT4G32040 | KNAT5, KNOTTED1-like homeobox gene 5 | 66.033 | 31.0963 | -1.08644 | 0.0106 | 0.0411532 |
| AT2G46340 | SPA1, SPA (suppressor of phyA-105) protein family | 78.1265 | 36.8071 | -1.08583 | 0.0079 | 0.0332028 |
| AT4G39820 | Tetratricopeptide repeat (TPR)-like superfamily protein | 55.7166 | 26.298 | -1.08315 | 0.0107 | 0.0414196 |
| AT4G36630 | EMB2754, Vacuolar sorting protein 39 | 36.5201 | 17.2482 | -1.08224 | 0.01115 | 0.0426612 |
| AT1G13350 | Protein kinase superfamily protein | 50.3157 | 23.8079 | -1.07957 | 0.0117 | 0.0442695 |
| AT5G19590 | Protein of unknown function, DUF538 | 105.21 | 49.8041 | -1.07893 | 0.0133 | 0.048507 |
| AT3G22200 | GABA-T, HER1, POP2, Pyridoxal phosphate (PLP)-dependent transferases superfamily protein | 215.053 | 101.86 | -1.0781 | 0.01195 | 0.0449578 |
| AT3G05420 | ACBP4, acyl-CoA binding protein 4 | 81.9277 | 38.8215 | -1.0775 | 0.0135 | 0.0489749 |
| AT2G03220 | ATFT1, ATFUT1, FT1, MUR2, fucosyltransferase 1 | 33.5285 | 15.8898 | -1.07729 | 0.0125 | 0.0463577 |
| AT4G32470 | Cytochrome bd ubiquinol oxidase, 14kDa subunit | 279.211 | 132.354 | -1.07695 | 0.01055 | 0.0410315 |
| AT5G52840 | NADH-ubiquinone oxidoreductase-related | 189.27 | 89.7926 | -1.07578 | 0.0125 | 0.0463577 |
| AT5G56750 | NDL1, N-MYC downregulated-like 1 | 167.173 | 79.4659 | -1.07293 | 0.01385 | 0.0499336 |
| AT1G73260 | ATKTI1, KTI1, kunitz trypsin inhibitor 1 | 286.717 | 136.376 | -1.07203 | 0.0126 | 0.0466761 |
| AT1G20100 | unknown protein | 377.277 | 179.697 | -1.07006 | 0.01135 | 0.0432177 |
| AT2G33220 | GRIM-19 protein | 204.967 | 97.6293 | -1.07001 | 0.01195 | 0.0449578 |
| AT4G29790 | unknown protein | 25.128 | 11.9841 | -1.06817 | 0.01245 | 0.0462329 |
| AT1G53590 | NTMC2T6.1, NTMC2TYPE6.1, Calcium-dependent lipid-binding (CaLB domain) family protein | 46.4609 | 22.1611 | -1.06799 | 0.01225 | 0.045713 |
| AT1G50360 | ATVIIIA, VIIIA, P-loop containing nucleoside triphosphate hydrolases superfamily protein | 23.2053 | 11.073 | -1.06741 | 0.0112 | 0.0427699 |
| AT1G12470 | zinc ion binding | 40.089 | 19.1493 | -1.06591 | 0.0135 | 0.0489749 |
| AT2G35190 | ATNPSN11, NPSN11, NSPN11, novel plant snare 11 | 61.2385 | 29.2884 | -1.06411 | 0.0137 | 0.0495371 |
| AT4G24020 | NLP7, NIN like protein 7 | 72.7891 | 34.8201 | -1.0638 | 0.01265 | 0.0467826 |
| AT3G10550 | Myotubularin-like phosphatases II superfamily | 38.0118 | 18.1849 | -1.0637 | 0.0135 | 0.0489749 |
| AT4G28540 | CKL6, PAPK1, casein kinase I-like 6 | 87.041 | 41.6859 | -1.06214 | 0.0133 | 0.048507 |
| AT1G79230 | ATMST1, ATRDH1, MST1, ST1, STR1, mercaptopyruvate sulfurtransferase 1 | 98.0262 | 46.9804 | -1.06111 | 0.0112 | 0.0427699 |
| AT3G19870 | unknown protein | 25.9462 | 12.4481 | -1.0596 | 0.01235 | 0.0460168 |
| AT2G01820 | Leucine-rich repeat protein kinase family protein | 53.323 | 25.5956 | -1.05886 | 0.01215 | 0.0454426 |
| AT4G33530 | KUP5, K+ uptake permease 5 | 78.8427 | 37.8523 | -1.0586 | 0.01295 | 0.0475634 |
| AT3G18290 | BTS, EMB2454, zinc finger protein-related | 87.6759 | 42.1226 | -1.05759 | 0.0133 | 0.048507 |
| AT5G04740 | ACT domain-containing protein | 229.554 | 110.421 | -1.05582 | 0.00995 | 0.0392764 |
| AT2G47140 | NAD(P)-binding Rossmann-fold superfamily protein | 192.21 | 92.5895 | -1.05377 | 0.0092 | 0.0370545 |
| AT5G19050 | alpha/beta-Hydrolases superfamily protein | 84.3927 | 40.6916 | -1.05239 | 0.01355 | 0.0491383 |
| AT2G45910 | U-box domain-containing protein kinase family protein | 34.3303 | 16.5747 | -1.0505 | 0.0126 | 0.0466761 |
| AT3G47080 | Tetratricopeptide repeat (TPR)-like superfamily protein | 135.781 | 65.5732 | -1.0501 | 0.0131 | 0.0480164 |
| AT1G10940 | ASK1, SNRK2-4, SNRK2.4, SRK2A, Protein kinase superfamily protein | 109.869 | 53.0696 | -1.04982 | 0.0094 | 0.0376304 |
| AT2G38840 | Guanylate-binding family protein | 53.7518 | 25.9669 | -1.04964 | 0.01295 | 0.0475634 |
| AT3G05545 | RING/U-box superfamily protein | 75.4836 | 36.6007 | -1.04429 | 0.01175 | 0.0443994 |
| AT2G41640 | Glycosyltransferase family 61 protein | 74.2369 | 36.01 | -1.04374 | 0.01305 | 0.0478685 |
| AT5G54730 | ATATG18F, ATG18F, G18F, homolog of yeast autophagy 18 (ATG18) F | 84.3504 | 41.039 | -1.0394 | 0.01195 | 0.0449578 |
| AT2G16365 | F-box family protein | 64.3346 | 31.3194 | -1.03854 | 0.0133 | 0.048507 |
| AT3G21510 | AHP1, histidine-containing phosphotransmitter 1 | 197.317 | 96.1353 | -1.03738 | 0.01265 | 0.0467826 |
| AT1G67310 | Calmodulin-binding transcription activator protein with CG-1 and Ankyrin domains | 111.217 | 54.1888 | -1.03731 | 0.0138 | 0.0497896 |
| AT1G64230 | UBC28, ubiquitin-conjugating enzyme 28 | 458.474 | 223.702 | -1.03526 | 0.013 | 0.0477116 |
| AT4G19640 | ARA-7, ARA7, ATRAB-F2B, ATRAB5B, ATRABF2B, RAB-F2B, RABF2B, Ras-related small GTP-binding family protein | 209.27 | 102.185 | -1.03418 | 0.0131 | 0.0480164 |
| AT1G29280 | ATWRKY65, WRKY65, WRKY DNA-binding protein 65 | 177.666 | 87.5543 | -1.02092 | 0.0138 | 0.0497896 |
| AT2G27720 | 60S acidic ribosomal protein family | 120.372 | 248.44 | 1.0454 | 0.0134 | 0.0487461 |
| AT5G47820 | FRA1, P-loop containing nucleoside triphosphate hydrolases superfamily protein | 8.80633 | 18.4405 | 1.06627 | 0.0136 | 0.0492835 |
| AT4G17390 | Ribosomal protein L23/L15e family protein | 72.0515 | 151.029 | 1.06773 | 0.013 | 0.0477116 |
| AT5G65750 | 2-oxoglutarate dehydrogenase, E1 component | 9.91097 | 20.8111 | 1.07025 | 0.0136 | 0.0492835 |
| AT2G29550 | TUB7, tubulin beta-7 chain | 50.1578 | 105.504 | 1.07276 | 0.0127 | 0.0469062 |
| AT3G22440 | FRIGIDA-like protein | 26.063 | 54.9103 | 1.07507 | 0.0135 | 0.0489749 |
| AT5G52650 | RNA binding Plectin/S10 domain-containing protein | 85.3214 | 179.917 | 1.07636 | 0.0134 | 0.0487461 |
| AT3G09920 | PIP5K9, phosphatidyl inositol monophosphate 5 kinase | 20.0086 | 42.2203 | 1.07731 | 0.0111 | 0.0425192 |
| AT5G41790 | CIP1, COP1-interactive protein 1 | 7.37013 | 15.5794 | 1.07988 | 0.0129 | 0.0474149 |
| AT5G66920 | sks17, SKU5 similar 17 | 43.9372 | 93.5 | 1.08953 | 0.0119 | 0.0448549 |
| AT4G26300 | emb1027, Arginyl-tRNA synthetase, class Ic | 16.2507 | 34.7351 | 1.09589 | 0.0123 | 0.0458737 |
| AT1G03230 | Eukaryotic aspartyl protease family protein | 64.119 | 137.252 | 1.098 | 0.00955 | 0.0380846 |
| AT5G64030 | S-adenosyl-L-methionine-dependent methyltransferases superfamily protein | 16.853 | 36.0858 | 1.09843 | 0.00845 | 0.0348633 |
| AT4G13780 | methionine--tRNA ligase, putative / methionyl-tRNA synthetase, putative / MetRS, putative | 16.5829 | 35.5525 | 1.10025 | 0.0096 | 0.0382301 |
| AT1G69250 | Nuclear transport factor 2 (NTF2) family protein with RNA binding (RRM-RBD-RNP motifs) domain | 17.773 | 38.1886 | 1.10345 | 0.01245 | 0.0462329 |
| AT1G68920 | basic helix-loop-helix (bHLH) DNA-binding superfamily protein | 17.2288 | 37.0572 | 1.10493 | 0.01375 | 0.0496726 |
| AT3G57290 | ATEIF3E-1, ATINT6, EIF3E, INT-6, INT6, TIF3E1, eukaryotic translation initiation factor 3E | 42.9714 | 92.552 | 1.10689 | 0.00925 | 0.0371953 |
| AT4G18280 | glycine-rich cell wall protein-related | 84.3423 | 182.133 | 1.11067 | 0.0117 | 0.0442695 |
| AT5G28840 | GME, GDP-D-mannose 3',5'-epimerase | 34.1286 | 73.8176 | 1.11298 | 0.00775 | 0.0327601 |
| AT4G36080 | phosphotransferases, alcohol group as acceptor;binding;inositol or phosphatidylinositol kinases | 1.90639 | 4.12419 | 1.11327 | 0.0138 | 0.0497896 |
| AT2G27170 | SMC3, TTN7, Structural maintenance of chromosomes (SMC) family protein | 8.62961 | 18.693 | 1.11513 | 0.0108 | 0.041652 |
| AT2G29400 | PP1-AT, TOPP1, type one protein phosphatase 1 | 21.8851 | 47.4557 | 1.11664 | 0.01045 | 0.0407225 |
| AT2G16060 | AHB1, ARATH GLB1, ATGLB1, GLB1, HB1, NSHB1, hemoglobin 1 | 76.7655 | 166.921 | 1.12064 | 0.0067 | 0.0294336 |
| AT5G47700 | 60S acidic ribosomal protein family | 56.2019 | 122.598 | 1.12524 | 0.01345 | 0.0488831 |
| AT5G06110 | DnaJ domain ;Myb-like DNA-binding domain | 14.3608 | 31.3954 | 1.12842 | 0.0121 | 0.0453155 |
| AT3G11540 | SPY, Tetratricopeptide repeat (TPR)-like superfamily protein | 6.20423 | 13.5684 | 1.12893 | 0.01335 | 0.0486446 |
| AT1G78150 | unknown protein | 49.4532 | 108.304 | 1.13094 | 0.00915 | 0.0369056 |
| AT3G09200 | Ribosomal protein L10 family protein | 166.759 | 365.37 | 1.13159 | 0.00725 | 0.03124 |
| AT3G02760 | Class II aaRS and biotin synthetases superfamily protein | 11.1298 | 24.3994 | 1.13242 | 0.00995 | 0.0392764 |
| AT3G02080 | Ribosomal protein S19e family protein | 131.104 | 287.526 | 1.13298 | 0.0078 | 0.0329152 |
| AT1G15500 | ATNTT2, TLC ATP/ADP transporter | 13.788 | 30.2841 | 1.13515 | 0.00925 | 0.0371953 |
| AT5G51660 | ATCPSF160, CPSF160, cleavage and polyadenylation specificity factor 160 | 3.97922 | 8.74211 | 1.1355 | 0.01355 | 0.0491383 |
| AT1G41830 | SKS6, SKS6, SKU5-similar 6 | 49.4095 | 108.554 | 1.13554 | 0.01285 | 0.0472926 |
| AT3G09270 | ATGSTU8, GSTU8, glutathione S-transferase TAU 8 | 51.6758 | 113.559 | 1.13589 | 0.0129 | 0.0474149 |
| AT3G52140 | tetratricopeptide repeat (TPR)-containing protein | 15.7628 | 34.686 | 1.13783 | 0.00855 | 0.0351514 |
| AT3G18740 | Ribosomal protein L7Ae/L30e/S12e/Gadd45 family protein | 124.524 | 274.163 | 1.13861 | 0.01075 | 0.0415725 |
| AT1G15930 | Ribosomal protein L7Ae/L30e/S12e/Gadd45 family protein | 109.082 | 240.561 | 1.14098 | 0.00665 | 0.0292594 |
| AT5G20160 | Ribosomal protein L7Ae/L30e/S12e/Gadd45 family protein | 70.624 | 155.856 | 1.14198 | 0.0098 | 0.0388625 |
| AT3G25150 | Nuclear transport factor 2 (NTF2) family protein with RNA binding (RRM-RBD-RNP motifs) domain | 10.2524 | 22.6336 | 1.1425 | 0.01195 | 0.0449578 |
| AT1G77330 | 2-oxoglutarate (2OG) and Fe(II)-dependent oxygenase superfamily protein | 102.362 | 226.015 | 1.14273 | 0.0079 | 0.0332028 |
| AT5G27700 | Ribosomal protein S21e | 129.32 | 285.645 | 1.14327 | 0.01 | 0.0393953 |
| AT4G02840 | Small nuclear ribonucleoprotein family protein | 43.5676 | 96.2675 | 1.14379 | 0.01285 | 0.0472926 |
| AT1G14610 | TWN2, VALRS, valyl-tRNA synthetase / valine--tRNA ligase (VALRS) | 15.3512 | 33.9394 | 1.14461 | 0.0069 | 0.0300655 |
| AT4G18880 | AT-HSFA4A, HSF A4A, heat shock transcription factor A4A | 9.81433 | 21.7092 | 1.14534 | 0.0137 | 0.0495371 |
| AT5G13300 | AGD3, SFC, VAN3, ARF GTPase-activating protein | 5.91927 | 13.1269 | 1.14903 | 0.01085 | 0.0417717 |
| AT5G39040 | ALS1, ATTAP2, TAP2, transporter associated with antigen processing protein 2 | 26.3208 | 58.3968 | 1.14969 | 0.0093 | 0.0372979 |
| AT1G19870 | iqd32, IQ-domain 32 | 24.0832 | 53.5322 | 1.15238 | 0.00635 | 0.0283169 |
| AT1G30580 | GTP binding | 71.29 | 158.499 | 1.15271 | 0.007 | 0.0303609 |
| AT5G65640 | bHLH093, beta HLH protein 93 | 34.7423 | 77.2895 | 1.15358 | 0.01035 | 0.0404522 |
| AT5G42580 | CYP705A12, cytochrome P450, family 705, subfamily A, polypeptide 12 | 102.872 | 229.455 | 1.15736 | 0.00935 | 0.037453 |
| AT5G43960 | Nuclear transport factor 2 (NTF2) family protein with RNA binding (RRM-RBD-RNP motifs) domain | 14.981 | 33.4275 | 1.1579 | 0.0092 | 0.0370545 |
| AT2G41840 | Ribosomal protein S5 family protein | 152.097 | 339.905 | 1.16014 | 0.0052 | 0.0244668 |
| AT3G19100 | Protein kinase superfamily protein | 22.7923 | 50.9387 | 1.16021 | 0.0046 | 0.0222191 |
| AT1G52300 | Zinc-binding ribosomal protein family protein | 100.59 | 225.16 | 1.16247 | 0.00655 | 0.0289545 |
| AT1G06670 | NIH, nuclear DEIH-boxhelicase | 3.97485 | 8.89949 | 1.16282 | 0.01035 | 0.0404522 |
| AT3G48750 | CDC2, CDC2A, CDC2AAT, CDK2, CDKA1, CDKA;1, cell division control 2 | 13.2776 | 29.7572 | 1.16424 | 0.0108 | 0.041652 |
| AT3G51850 | CPK13, calcium-dependent protein kinase 13 | 9.8182 | 22.0439 | 1.16685 | 0.0104 | 0.0405756 |
| AT5G03330 | Cysteine proteinases superfamily protein | 19.3709 | 43.5725 | 1.16953 | 0.0096 | 0.0382301 |
| AT1G79650 | RAD23, RAD23B, Rad23 UV excision repair protein family | 14.2154 | 31.9788 | 1.16966 | 0.01 | 0.0393953 |
| AT1G13640 | Phosphatidylinositol 3- and 4-kinase family protein | 10.6362 | 23.9571 | 1.17147 | 0.00695 | 0.0302036 |
| AT3G56340 | Ribosomal protein S26e family protein | 103.597 | 233.459 | 1.17218 | 0.00595 | 0.0269704 |
| AT4G21100 | DDB1B, damaged DNA binding protein 1B | 5.32208 | 11.996 | 1.1725 | 0.01055 | 0.0410315 |
| AT3G13080 | ATMRP3, MRP3, MRP3, multidrug resistance-associated protein 3 | 8.68618 | 19.6325 | 1.17645 | 0.0087 | 0.0356202 |
| AT1G57720 | Translation elongation factor EF1B, gamma chain | 83.2165 | 188.35 | 1.17847 | 0.0071 | 0.0306604 |
| AT4G02150 | ATIMPALPHA3, IMPA-3, MOS6, ARM repeat superfamily protein | 12.7264 | 28.8285 | 1.17967 | 0.00835 | 0.0345732 |
| AT1G71110 | unknown protein | 15.971 | 36.1973 | 1.18043 | 0.0105 | 0.0408771 |
| AT3G62250 | UBQ5, ubiquitin 5 | 142.098 | 322.287 | 1.18146 | 0.00565 | 0.0260215 |
| AT3G54670 | ATSMC1, SMC1, TTN8, Structural maintenance of chromosomes (SMC) family protein | 6.99787 | 15.8735 | 1.18163 | 0.0081 | 0.0338066 |
| AT5G60930 | P-loop containing nucleoside triphosphate hydrolases superfamily protein | 3.98926 | 9.0627 | 1.18382 | 0.01275 | 0.0470557 |
| AT1G30120 | PDH-E1 BETA, pyruvate dehydrogenase E1 beta | 16.7172 | 37.9992 | 1.18464 | 0.0103 | 0.0403045 |
| AT3G54010 | DEI1, PAS1, FKBP-type peptidyl-prolyl cis-trans isomerase family protein | 5.61024 | 12.7598 | 1.18547 | 0.01235 | 0.0460168 |
| AT1G59900 | AT-E1 ALPHA, E1 ALPHA, pyruvate dehydrogenase complex E1 alpha subunit | 12.5432 | 28.5305 | 1.1856 | 0.00815 | 0.0339652 |
| AT1G30880 | unknown protein | 43.5069 | 99.1328 | 1.18812 | 0.0104 | 0.0405756 |
| AT5G64840 | ATGCN5, GCN5, general control non-repressible 5 | 6.08896 | 13.894 | 1.1902 | 0.0137 | 0.0495371 |
| AT5G05450 | P-loop containing nucleoside triphosphate hydrolases superfamily protein | 5.37698 | 12.2892 | 1.19252 | 0.01195 | 0.0449578 |
| AT1G71840 | transducin family protein / WD-40 repeat family protein | 7.99577 | 18.2771 | 1.19273 | 0.0137 | 0.0495371 |
| AT4G13970 | zinc ion binding | 3.46135 | 7.93332 | 1.19659 | 0.0104 | 0.0405756 |
| AT3G02720 | Class I glutamine amidotransferase-like superfamily protein | 19.1086 | 43.8207 | 1.19739 | 0.00645 | 0.0286275 |
| AT2G02560 | ATCAND1, CAND1, ETA2, HVE, TIP120, cullin-associated and neddylation dissociated | 10.8756 | 24.9803 | 1.19969 | 0.00535 | 0.025018 |
| AT4G37870 | PCK1, PEPCK, phosphoenolpyruvate carboxykinase 1 | 131.217 | 301.599 | 1.20067 | 0.006 | 0.027135 |
| AT4G02930 | GTP binding Elongation factor Tu family protein | 29.867 | 68.7868 | 1.20358 | 0.0049 | 0.0233549 |
| AT5G03530 | ATRAB, ATRAB ALPHA, ATRAB18B, ATRABC2A, RABC2A, RAB GTPase homolog C2A | 13.8972 | 32.0075 | 1.20361 | 0.01275 | 0.0470557 |
| AT2G06210 | ELF8, VIP6, binding | 5.99546 | 13.817 | 1.20451 | 0.0054 | 0.0251864 |
| AT1G10840 | TIF3H1, translation initiation factor 3 subunit H1 | 31.6854 | 73.0347 | 1.20476 | 0.00525 | 0.0246552 |
| AT3G16310 | mitotic phosphoprotein N' end (MPPN) family protein | 7.98926 | 18.4717 | 1.20919 | 0.0108 | 0.041652 |
| AT4G17950 | AT hook motif DNA-binding family protein | 7.0637 | 16.3469 | 1.21052 | 0.01315 | 0.0481551 |
| AT2G47250 | RNA helicase family protein | 11.0968 | 25.6869 | 1.21089 | 0.00595 | 0.0269704 |
| AT3G59850 | Pectin lyase-like superfamily protein | 53.4824 | 123.944 | 1.21255 | 0.0045 | 0.0218802 |
| AT3G54350 | emb1967, Forkhead-associated (FHA) domain-containing protein | 5.32352 | 12.3575 | 1.21493 | 0.01285 | 0.0472926 |
| AT4G29390 | Ribosomal protein S30 family protein | 73.4066 | 170.429 | 1.21519 | 0.01105 | 0.0423687 |
| AT3G52890 | KIPK, KCBP-interacting protein kinase | 8.68851 | 20.176 | 1.21546 | 0.00695 | 0.0302036 |
| AT5G12860 | DiT1, dicarboxylate transporter 1 | 11.2578 | 26.165 | 1.21672 | 0.00945 | 0.0377542 |
| AT1G78560 | Sodium Bile acid symporter family | 13.9284 | 32.4079 | 1.21831 | 0.008 | 0.0335306 |
| AT3G13070 | CBS domain-containing protein / transporter associated domain-containing protein | 6.60092 | 15.3751 | 1.21986 | 0.0073 | 0.0313803 |
| AT5G57090 | AGR, AGR1, ATPIN2, EIR1, PIN2, WAV6, Auxin efflux carrier family protein | 39.3992 | 91.7715 | 1.21988 | 0.0059 | 0.0268421 |
| AT5G41600 | BTI3, RTNLB4, VIRB2-interacting protein 3 | 14.823 | 34.5528 | 1.22097 | 0.0119 | 0.0448549 |
| AT5G10060 | ENTH/VHS family protein | 6.25258 | 14.5871 | 1.22217 | 0.01215 | 0.0454426 |
| AT1G26460 | Tetratricopeptide repeat (TPR)-like superfamily protein | 8.50254 | 19.8468 | 1.22294 | 0.00725 | 0.03124 |
| AT4G18600 | ATSCAR-LIKE, SCARL, WAVE5, SCAR family protein | 1.41643 | 3.30719 | 1.22334 | 0.0123 | 0.0458737 |
| AT5G62610 | basic helix-loop-helix (bHLH) DNA-binding superfamily protein | 17.1947 | 40.1723 | 1.22424 | 0.0073 | 0.0313803 |
| AT5G65360 | Histone superfamily protein | 42.5754 | 99.4859 | 1.22447 | 0.0074 | 0.0317207 |
| AT4G15780 | ATVAMP724, VAMP724, vesicle-associated membrane protein 724 | 18.909 | 44.3074 | 1.22847 | 0.00995 | 0.0392764 |
| AT5G53170 | FTSH11, FTSH protease 11 | 5.3627 | 12.5722 | 1.22921 | 0.00765 | 0.0324899 |
| AT5G16290 | VAT1, VALINE-TOLERANT 1 | 8.31293 | 19.5026 | 1.23024 | 0.01245 | 0.0462329 |
| AT5G41520 | RNA binding Plectin/S10 domain-containing protein | 79.7031 | 187.057 | 1.23077 | 0.00365 | 0.0186349 |
| AT1G64550 | ATGCN3, GCN3, general control non-repressible 3 | 13.0446 | 30.6326 | 1.23162 | 0.00465 | 0.0223951 |
| AT2G32710 | ACK2, ICK7, KRP4, KRP4, Cyclin-dependent kinase inhibitor family protein | 10.179 | 23.9062 | 1.23179 | 0.0104 | 0.0405756 |
| AT3G08030 | Protein of unknown function, DUF642 | 39.3277 | 92.5896 | 1.2353 | 0.0032 | 0.0169175 |
| AT4G02110 | transcription coactivators | 2.47651 | 5.83066 | 1.23535 | 0.00915 | 0.0369056 |
| AT2G27710 | 60S acidic ribosomal protein family | 44.2225 | 104.141 | 1.23568 | 0.01155 | 0.0438444 |
| AT3G02710 | ARM repeat superfamily protein | 6.15577 | 14.5075 | 1.23679 | 0.01085 | 0.0417717 |
| AT5G36250 | Protein phosphatase 2C family protein | 17.1784 | 40.5044 | 1.23748 | 0.0049 | 0.0233549 |
| AT3G48110 | EDD, EDD1, glycine-tRNA ligases | 2.72133 | 6.41968 | 1.23819 | 0.0098 | 0.0388625 |
| AT3G18790 | function unknown | 8.50243 | 20.0739 | 1.23937 | 0.0107 | 0.0414196 |
| AT3G53400 | BEST Arabidopsis thaliana protein match is: conserved peptide upstream open reading frame 47 (TAIR:AT5G03190.1) | 7.88863 | 18.6829 | 1.24387 | 0.00515 | 0.024295 |
| AT3G47890 | Ubiquitin carboxyl-terminal hydrolase-related protein | 1.83546 | 4.3482 | 1.24427 | 0.0121 | 0.0453155 |
| AT3G19820 | CBB1, DIM, DIM1, DWF1, EVE1, cell elongation protein / DWARF1 / DIMINUTO (DIM) | 78.4693 | 186.005 | 1.24514 | 0.0066 | 0.0290976 |
| AT3G11710 | ATKRS-1, lysyl-tRNA synthetase 1 | 23.2087 | 55.0875 | 1.24706 | 0.0036 | 0.0184414 |
| AT2G26900 | Sodium Bile acid symporter family | 6.00257 | 14.2722 | 1.24955 | 0.01365 | 0.0494285 |
| AT2G40280 | S-adenosyl-L-methionine-dependent methyltransferases superfamily protein | 7.78579 | 18.5148 | 1.24976 | 0.0101 | 0.039726 |
| AT1G80070 | EMB14, EMB177, EMB33, SUS2, Pre-mRNA-processing-splicing factor | 16.3458 | 38.9212 | 1.25164 | 0.00405 | 0.0202184 |
| AT4G39200 | Ribosomal protein S25 family protein | 100.183 | 238.594 | 1.25192 | 0.0039 | 0.0196279 |
| AT4G17940 | Tetratricopeptide repeat (TPR)-like superfamily protein | 89.8776 | 214.145 | 1.25255 | 0.0045 | 0.0218802 |
| AT5G65920 | ARM repeat superfamily protein | 7.54145 | 17.9905 | 1.25432 | 0.00595 | 0.0269704 |
| AT1G01090 | PDH-E1 ALPHA, pyruvate dehydrogenase E1 alpha | 23.1515 | 55.2546 | 1.25499 | 0.004 | 0.0200243 |
| AT4G01860 | Transducin family protein / WD-40 repeat family protein | 4.46681 | 10.6649 | 1.25556 | 0.0055 | 0.0254967 |
| AT3G18610 | ATNUC-L2, NUC-L2, PARLL1, nucleolin like 2 | 3.72097 | 8.88564 | 1.25579 | 0.011 | 0.0422342 |
| AT5G03610 | GDSL-like Lipase/Acylhydrolase superfamily protein | 17.8353 | 42.5939 | 1.25591 | 0.0043 | 0.0211046 |
| AT1G73720 | SMU1, transducin family protein / WD-40 repeat family protein | 6.44926 | 15.4112 | 1.25677 | 0.0085 | 0.0349894 |
| AT1G16350 | Aldolase-type TIM barrel family protein | 6.91552 | 16.5271 | 1.25692 | 0.00995 | 0.0392764 |
| AT1G27470 | transducin family protein / WD-40 repeat family protein | 6.19412 | 14.8108 | 1.25768 | 0.0085 | 0.0349894 |
| AT1G44910 | ATPRP40A, PRP40A, pre-mRNA-processing protein 40A | 4.66124 | 11.1528 | 1.25862 | 0.00705 | 0.0305176 |
| AT1G14710 | hydroxyproline-rich glycoprotein family protein | 10.8381 | 25.9543 | 1.25986 | 0.00555 | 0.0256744 |
| AT3G08690 | ATUBC11, UBC11, ubiquitin-conjugating enzyme 11 | 22.1332 | 53.0109 | 1.26008 | 0.0129 | 0.0474149 |
| AT3G54500 | BEST Arabidopsis thaliana protein match is: dentin sialophosphoprotein-related (TAIR:AT5G64170.1) | 7.74183 | 18.5501 | 1.26068 | 0.00945 | 0.0377542 |
| AT5G07010 | ATST2A, ST2A, sulfotransferase 2A | 22.5978 | 54.1621 | 1.2611 | 0.00435 | 0.0212815 |
| AT1G13000 | Protein of unknown function (DUF707) | 10.3654 | 24.9249 | 1.26581 | 0.0124 | 0.046125 |
| AT3G02630 | Plant stearoyl-acyl-carrier-protein desaturase family protein | 9.31177 | 22.4357 | 1.26867 | 0.00745 | 0.0318523 |
| AT2G29470 | ATGSTU3, GST21, GSTU3, glutathione S-transferase tau 3 | 69.7378 | 168.027 | 1.26868 | 0.0068 | 0.029741 |
| AT2G25170 | CHD3, CHR6, GYM, PKL, SSL2, chromatin remodeling factor CHD3 (PICKLE) | 8.90759 | 21.473 | 1.26942 | 0.0026 | 0.0145003 |
| AT5G04060 | S-adenosyl-L-methionine-dependent methyltransferases superfamily protein | 4.21122 | 10.1553 | 1.26992 | 0.0108 | 0.041652 |
| AT3G22230 | Ribosomal L27e protein family | 71.7602 | 173.074 | 1.27013 | 0.0101 | 0.039726 |
| AT3G06650 | ACLB-1, ATP-citrate lyase B-1 | 23.5636 | 56.869 | 1.27108 | 0.00735 | 0.0315405 |
| AT4G13630 | Protein of unknown function, DUF593 | 8.0289 | 19.3972 | 1.27258 | 0.006 | 0.027135 |
| AT2G40650 | PRP38 family protein | 8.83177 | 21.3534 | 1.27369 | 0.00805 | 0.0336689 |
| AT3G61850 | DAG1, Dof-type zinc finger DNA-binding family protein | 10.1928 | 24.6525 | 1.27419 | 0.01165 | 0.0441141 |
| AT5G49555 | FAD/NAD(P)-binding oxidoreductase family protein | 3.23748 | 7.83632 | 1.27531 | 0.01205 | 0.0451624 |
| AT4G18730 | RPL16B, ribosomal protein L16B | 98.6908 | 238.892 | 1.27537 | 0.00205 | 0.012221 |
| AT3G60245 | Zinc-binding ribosomal protein family protein | 163.292 | 395.318 | 1.27556 | 0.00385 | 0.0194256 |
| AT3G27280 | ATPHB4, PHB4, prohibitin 4 | 9.12905 | 22.1336 | 1.2777 | 0.0121 | 0.0453155 |
| AT2G19600 | ATKEA4, KEA4, KEA4, K+ efflux antiporter 4 | 4.36098 | 10.5746 | 1.27788 | 0.00755 | 0.0321272 |
| AT3G06483 | ATPDHK, PDK, pyruvate dehydrogenase kinase | 6.93999 | 16.8443 | 1.27925 | 0.0074 | 0.0317207 |
| AT4G09980 | EMB1691, Methyltransferase MT-A70 family protein | 3.25044 | 7.90636 | 1.28238 | 0.00995 | 0.0392764 |
| AT5G63980 | ALX8, ATSAL1, FRY1, HOS2, RON1, SAL1, Inositol monophosphatase family protein | 18.513 | 45.0875 | 1.28419 | 0.0048 | 0.0229834 |
| AT5G22280 | unknown protein | 16.2855 | 39.724 | 1.28642 | 0.00585 | 0.0267005 |
| AT3G48860 | unknown protein | 5.52209 | 13.4759 | 1.2871 | 0.0081 | 0.0338066 |
| AT1G16190 | RAD23A, Rad23 UV excision repair protein family | 15.669 | 38.2864 | 1.28892 | 0.00505 | 0.0239202 |
| AT1G13280 | AOC4, allene oxide cyclase 4 | 12.2216 | 29.8827 | 1.28988 | 0.0083 | 0.0344309 |
| AT1G42440 | function unknown | 6.39196 | 15.6354 | 1.29049 | 0.0075 | 0.0319763 |
| AT1G58250 | SAB, Golgi-body localisation protein domain ;RNA pol II promoter Fmp27 protein domain | 2.90655 | 7.12304 | 1.29318 | 0.00365 | 0.0186349 |
| AT1G73940 | unknown protein | 20.214 | 49.5882 | 1.29464 | 0.0085 | 0.0349894 |
| AT2G37640 | ATEXP3, ATEXPA3, ATHEXP ALPHA 1.9, EXP3, Barwin-like endoglucanases superfamily protein | 19.8782 | 48.824 | 1.2964 | 0.007 | 0.0303609 |
| AT1G73500 | ATMKK9, MKK9, MAP kinase kinase 9 | 10.4424 | 25.6603 | 1.29708 | 0.00815 | 0.0339652 |
| AT3G11070 | Outer membrane OMP85 family protein | 7.27169 | 17.8803 | 1.29801 | 0.00915 | 0.0369056 |
| AT1G26370 | RNA helicase family protein | 3.15225 | 7.76127 | 1.29991 | 0.0099 | 0.0391493 |
| AT1G72550 | tRNA synthetase beta subunit family protein | 13.0073 | 32.0278 | 1.3 | 0.00335 | 0.0175049 |
| AT5G53800 | unknown protein | 9.03678 | 22.2704 | 1.30125 | 0.00675 | 0.0295876 |
| AT1G60770 | Tetratricopeptide repeat (TPR)-like superfamily protein | 7.45239 | 18.3669 | 1.30133 | 0.0079 | 0.0332028 |
| AT3G49910 | Translation protein SH3-like family protein | 118.394 | 291.858 | 1.30167 | 0.0059 | 0.0268421 |
| AT4G00710 | BSK3, BR-signaling kinase 3 | 17.5075 | 43.1594 | 1.30171 | 0.00315 | 0.0166977 |
| AT5G10560 | Glycosyl hydrolase family protein | 7.2678 | 17.9274 | 1.30257 | 0.0043 | 0.0211046 |
| AT5G03850 | Nucleic acid-binding, OB-fold-like protein | 183.608 | 453 | 1.30288 | 0.00285 | 0.0154761 |
| AT5G53090 | NAD(P)-binding Rossmann-fold superfamily protein | 5.60156 | 13.8399 | 1.30493 | 0.0086 | 0.0353203 |
| AT1G03790 | SOM, Zinc finger C-x8-C-x5-C-x3-H type family protein | 8.57628 | 21.2066 | 1.30609 | 0.01265 | 0.0467826 |
| AT1G28520 | ATVOZ1, VOZ1, vascular plant one zinc finger protein | 4.92165 | 12.1728 | 1.30645 | 0.00985 | 0.0390139 |
| AT2G32560 | F-box family protein | 7.12737 | 17.6348 | 1.30698 | 0.00875 | 0.0357584 |
| AT1G18700 | DNAJ heat shock N-terminal domain-containing protein | 5.30333 | 13.1291 | 1.3078 | 0.0065 | 0.0287784 |
| AT2G41800 | Protein of unknown function, DUF642 | 110.379 | 273.359 | 1.30833 | 0.0032 | 0.0169175 |
| AT1G13560 | AAPT1, ATAAPT1, aminoalcoholphosphotransferase 1 | 19.4787 | 48.2947 | 1.30997 | 0.00315 | 0.0166977 |
| AT5G46840 | RNA-binding (RRM/RBD/RNP motifs) family protein | 14.7538 | 36.5836 | 1.31011 | 0.0032 | 0.0169175 |
| AT3G09500 | Ribosomal L29 family protein | 160.176 | 397.975 | 1.31302 | 0.00185 | 0.0113253 |
| AT2G44950 | HUB1, RDO4, histone mono-ubiquitination 1 | 4.4216 | 10.9928 | 1.31392 | 0.00405 | 0.0202184 |
| AT2G06990 | HEN2, RNA helicase, ATP-dependent, SK12/DOB1 protein | 6.43054 | 15.9969 | 1.31478 | 0.00195 | 0.0117451 |
| AT1G18080 | ATARCA, RACK1A, RACK1A_AT, Transducin/WD40 repeat-like superfamily protein | 74.7469 | 186.183 | 1.31663 | 0.0023 | 0.0132526 |
| AT3G52870 | IQ calmodulin-binding motif family protein | 21.305 | 53.0686 | 1.31667 | 0.0029 | 0.0157089 |
| AT2G37220 | RNA-binding (RRM/RBD/RNP motifs) family protein | 8.6867 | 21.6958 | 1.32054 | 0.0065 | 0.0287784 |
| AT2G23150 | ATNRAMP3, NRAMP3, natural resistance-associated macrophage protein 3 | 3.36748 | 8.41475 | 1.32125 | 0.00915 | 0.0369056 |
| AT5G49030 | OVA2, tRNA synthetase class I (I, L, M and V) family protein | 3.9946 | 9.98362 | 1.32151 | 0.0054 | 0.0251864 |
| AT3G25530 | ATGHBDH, GHBDH, GLYR1, GR1, glyoxylate reductase 1 | 15.7847 | 39.4516 | 1.32156 | 0.00625 | 0.0280351 |
| AT3G07210 | unknown protein | 3.5513 | 8.88942 | 1.32374 | 0.01175 | 0.0443994 |
| AT1G08660 | MGP2, MALE GAMETOPHYTE DEFECTIVE 2 | 7.87452 | 19.7251 | 1.32477 | 0.0075 | 0.0319763 |
| AT1G69620 | RPL34, ribosomal protein L34 | 204.899 | 513.707 | 1.32603 | 0.0018 | 0.0110912 |
| AT1G26930 | Galactose oxidase/kelch repeat superfamily protein | 15.2801 | 38.3111 | 1.32611 | 0.0061 | 0.0274993 |
| AT2G15830 | unknown protein | 93.1343 | 233.621 | 1.32679 | 0.01245 | 0.0462329 |
| AT5G27740 | EMB161, EMB251, EMB2775, RFC3, ATPase family associated with various cellular activities (AAA) | 5.45257 | 13.6949 | 1.32862 | 0.0119 | 0.0448549 |
| AT5G17710 | EMB1241, Co-chaperone GrpE family protein | 10.0779 | 25.3262 | 1.32943 | 0.00985 | 0.0390139 |
| AT1G66090 | Disease resistance protein (TIR-NBS class) | 5.14808 | 12.9441 | 1.33019 | 0.01115 | 0.0426612 |
| AT1G09150 | pseudouridine synthase and archaeosine transglycosylase (PUA) domain-containing protein | 9.37107 | 23.567 | 1.33048 | 0.012 | 0.0450518 |
| AT5G23250 | Succinyl-CoA ligase, alpha subunit | 7.13727 | 17.9859 | 1.33342 | 0.007 | 0.0303609 |
| AT1G07170 | PHF5-like protein | 12.7903 | 32.2747 | 1.33536 | 0.01385 | 0.0499336 |
| AT4G38440 | LOCATED IN: chloroplast; EXPRESSED IN: 21 plant structures; EXPRESSED DURING: 12 growth stages; CONTAINS InterPro DOMAIN/s: RNA polymerase II-associated protein 1, C-terminal (InterPro:IPR013929), RNA polymerase II-associated protein 1, N-terminal (InterPro:IPR013930); Has 276 Blast hits to 220 proteins in 102 species: Archae - 0; Bacteria - 2; Metazoa - 151; Fungi - 65; Plants - 41; Viruses - 0; Other Eukaryotes - 17 (source: NCBI BLink). | 1.79418 | 4.53186 | 1.33678 | 0.0101 | 0.039726 |
| AT1G02390 | ATGPAT2, GPAT2, glycerol-3-phosphate acyltransferase 2 | 6.53419 | 16.5073 | 1.33702 | 0.00515 | 0.024295 |
| AT3G02220 | unknown protein | 12.4539 | 31.4667 | 1.33723 | 0.01045 | 0.0407225 |
| AT5G55190 | ATRAN3, RAN3, RAN GTPase 3 | 37.3098 | 94.3228 | 1.33805 | 0.0023 | 0.0132526 |
| AT1G53200 | unknown protein | 2.51049 | 6.34914 | 1.33859 | 0.01225 | 0.045713 |
| AT3G02890 | RING/FYVE/PHD zinc finger superfamily protein | 6.62773 | 16.771 | 1.33938 | 0.00195 | 0.0117451 |
| AT5G57270 | Core-2/I-branching beta-1,6-N-acetylglucosaminyltransferase family protein | 3.87593 | 9.82385 | 1.34175 | 0.0116 | 0.0439836 |
| AT3G58790 | GAUT15, galacturonosyltransferase 15 | 4.42029 | 11.2118 | 1.3428 | 0.0076 | 0.0322984 |
| AT1G17830 | Protein of unknown function (DUF789) | 5.19218 | 13.1824 | 1.3442 | 0.0091 | 0.0368164 |
| AT1G65730 | YSL7, YELLOW STRIPE like 7 | 25.4127 | 64.5294 | 1.34441 | 0.0042 | 0.0207631 |
| AT1G80530 | Major facilitator superfamily protein | 9.21897 | 23.4189 | 1.345 | 0.00285 | 0.0154761 |
| AT1G59890 | SNL5, SIN3-like 5 | 5.79914 | 14.7392 | 1.34575 | 0.00335 | 0.0175049 |
| AT3G48870 | ATCLPC, ATHSP93-III, HSP93-III, Clp ATPase | 15.6384 | 39.7538 | 1.346 | 0.0016 | 0.0101494 |
| AT5G62300 | Ribosomal protein S10p/S20e family protein | 104.965 | 267.169 | 1.34784 | 0.00385 | 0.0194256 |
| AT1G09830 | Glycinamide ribonucleotide (GAR) synthetase | 3.55439 | 9.05588 | 1.34925 | 0.0092 | 0.0370545 |
| AT4G16260 | Glycosyl hydrolase superfamily protein | 24.5554 | 62.5874 | 1.34983 | 0.00645 | 0.0286275 |
| AT1G02780 | emb2386, Ribosomal protein L19e family protein | 179.329 | 457.563 | 1.35136 | 0.0025 | 0.0140894 |
| AT3G28345 | ABC transporter family protein | 1.96787 | 5.02577 | 1.35271 | 0.0108 | 0.041652 |
| AT1G14910 | ENTH/ANTH/VHS superfamily protein | 7.67036 | 19.5996 | 1.35346 | 0.00345 | 0.0179093 |
| AT1G74960 | ATKAS2, FAB1, KAS2, fatty acid biosynthesis 1 | 13.4977 | 34.5203 | 1.35473 | 0.0031 | 0.0165078 |
| AT5G35530 | Ribosomal protein S3 family protein | 68.2488 | 174.725 | 1.35621 | 0.00215 | 0.0126571 |
| AT1G01960 | EDA10, SEC7-like guanine nucleotide exchange family protein | 11.7191 | 30.0203 | 1.35708 | 0.01075 | 0.0415725 |
| AT4G00740 | S-adenosyl-L-methionine-dependent methyltransferases superfamily protein | 12.1252 | 31.0699 | 1.35751 | 0.00525 | 0.0246552 |
| AT5G38110 | ASF1B, SGA01, SGA1, anti- silencing function 1b | 6.70716 | 17.1931 | 1.35805 | 0.0077 | 0.0326253 |
| AT5G67360 | ARA12, Subtilase family protein | 3.76403 | 9.64881 | 1.35807 | 0.00595 | 0.0269704 |
| AT5G59790 | Domain of unknown function (DUF966) | 8.12778 | 20.8394 | 1.35838 | 0.0058 | 0.0265396 |
| AT5G05610 | AL1, alfin-like 1 | 11.7658 | 30.2184 | 1.36082 | 0.0058 | 0.0265396 |
| AT3G53890 | Ribosomal protein S21e | 106.962 | 274.759 | 1.36107 | 0.00235 | 0.0134663 |
| AT2G27190 | ATPAP1, ATPAP12, PAP1, PAP12, purple acid phosphatase 12 | 11.2766 | 28.9862 | 1.36203 | 0.00305 | 0.0163293 |
| AT5G56900 | CwfJ-like family protein / zinc finger (CCCH-type) family protein | 7.98856 | 20.5398 | 1.36241 | 0.00425 | 0.0209475 |
| AT1G18450 | ARP4, ATARP4, actin-related protein 4 | 11.1091 | 28.5708 | 1.3628 | 0.0035 | 0.0180741 |
| AT4G26190 | Haloacid dehalogenase-like hydrolase (HAD) superfamily protein | 5.75576 | 14.8204 | 1.36451 | 0.0025 | 0.0140894 |
| AT5G50840 | CONTAINS InterPro DOMAIN/s: Taxilin (InterPro:IPR019132) | 8.30558 | 21.4076 | 1.36597 | 0.0063 | 0.0281701 |
| AT5G21160 | LA RNA-binding protein | 6.10208 | 15.7342 | 1.36653 | 0.00235 | 0.0134663 |
| AT1G75340 | Zinc finger C-x8-C-x5-C-x3-H type family protein | 5.01418 | 12.9336 | 1.36704 | 0.0108 | 0.041652 |
| AT5G54390 | AHL, ATAHL, HL, HAL2-like | 38.1391 | 98.3844 | 1.36716 | 0.0009 | 0.0065867 |
| AT1G60800 | NIK3, NSP-interacting kinase 3 | 2.39918 | 6.19037 | 1.36748 | 0.012 | 0.0450518 |
| AT3G26090 | ATRGS1, RGS1, G-protein coupled receptors;GTPase activators | 8.41073 | 21.7367 | 1.36983 | 0.00345 | 0.0179093 |
| AT4G11120 | translation elongation factor Ts (EF-Ts), putative | 7.76913 | 20.1203 | 1.37282 | 0.0048 | 0.0229834 |
| AT1G49400 | emb1129, Nucleic acid-binding, OB-fold-like protein | 7.75468 | 20.0895 | 1.3733 | 0.00965 | 0.0383598 |
| AT3G58530 | RNI-like superfamily protein | 4.46677 | 11.5725 | 1.37339 | 0.008 | 0.0335306 |
| AT2G33340 | MAC3B, MOS4-associated complex 3B | 22.1636 | 57.5146 | 1.37573 | 0.00195 | 0.0117451 |
| AT5G23820 | MD-2-related lipid recognition domain-containing protein | 230.346 | 597.935 | 1.37619 | 0.0018 | 0.0110912 |
| AT1G63700 | EMB71, MAPKKK4, YDA, Protein kinase superfamily protein | 4.53888 | 11.7837 | 1.37639 | 0.0023 | 0.0132526 |
| AT1G10450 | SNL6, SIN3-like 6 | 4.83714 | 12.5623 | 1.37688 | 0.00415 | 0.020593 |
| AT2G35780 | scpl26, serine carboxypeptidase-like 26 | 6.30264 | 16.3834 | 1.3782 | 0.0086 | 0.0353203 |
| AT3G04600 | Nucleotidylyl transferase superfamily protein | 9.43377 | 24.5365 | 1.37902 | 0.0035 | 0.0180741 |
| AT2G39190 | ATATH8, Protein kinase superfamily protein | 2.15437 | 5.60582 | 1.37966 | 0.00865 | 0.0354741 |
| AT1G12000 | Phosphofructokinase family protein | 42.2082 | 109.913 | 1.38077 | 0.002 | 0.0119879 |
| AT4G22340 | CDS2, cytidinediphosphate diacylglycerol synthase 2 | 7.12135 | 18.553 | 1.38143 | 0.0045 | 0.0218802 |
| AT1G75330 | OTC, ornithine carbamoyltransferase | 14.3925 | 37.5142 | 1.38212 | 0.0044 | 0.0214942 |
| AT5G02260 | ATEXP9, ATEXPA9, ATHEXP ALPHA 1.10, EXP9, EXPA9, expansin A9 | 215.734 | 562.346 | 1.3822 | 0.0028 | 0.0152969 |
| AT1G11580 | ATPMEPCRA, PMEPCRA, methylesterase PCR A | 40.1105 | 104.645 | 1.38345 | 0.0012 | 0.00815259 |
| AT2G29420 | ATGSTU7, GST25, GSTU7, glutathione S-transferase tau 7 | 220.431 | 575.141 | 1.38359 | 0.0011 | 0.00759303 |
| AT4G38740 | ROC1, rotamase CYP 1 | 36.7551 | 95.9059 | 1.38368 | 0.00175 | 0.0108609 |
| AT4G39860 | unknown protein | 8.95933 | 23.378 | 1.38369 | 0.00515 | 0.024295 |
| AT5G09770 | Ribosomal protein L17 family protein | 11.2546 | 29.3881 | 1.38471 | 0.0063 | 0.0281701 |
| AT4G20400 | JMJ14, PKDM7B, JUMONJI 14 | 6.05784 | 15.8242 | 1.38526 | 0.00235 | 0.0134663 |
| AT3G48000 | ALDH2, ALDH2A, ALDH2B4, aldehyde dehydrogenase 2B4 | 68.1123 | 178.105 | 1.38674 | 0.0015 | 0.00967937 |
| AT4G20360 | ATRAB8D, ATRABE1B, RABE1b, RAB GTPase homolog E1B | 2.84507 | 7.44287 | 1.38739 | 0.0137 | 0.0495371 |
| AT5G55140 | ribosomal protein L30 family protein | 8.49616 | 22.2375 | 1.38811 | 0.01305 | 0.0478685 |
| AT2G43870 | Pectin lyase-like superfamily protein | 22.2064 | 58.1351 | 1.38844 | 0.00275 | 0.0151029 |
| AT5G19100 | Eukaryotic aspartyl protease family protein | 68.1949 | 178.547 | 1.38857 | 0.0014 | 0.00917155 |
| AT1G14390 | Leucine-rich repeat protein kinase family protein | 1.70924 | 4.47641 | 1.38899 | 0.0134 | 0.0487461 |
| AT1G63660 | GMP synthase (glutamine-hydrolyzing), putative / glutamine amidotransferase, putative | 7.87793 | 20.6472 | 1.39006 | 0.00625 | 0.0280351 |
| AT5G10360 | EMB3010, RPS6B, Ribosomal protein S6e | 86.437 | 226.543 | 1.39007 | 0.00085 | 0.00630464 |
| AT5G40250 | RING/U-box superfamily protein | 3.38277 | 8.86618 | 1.3901 | 0.00975 | 0.0387185 |
| AT4G19010 | AMP-dependent synthetase and ligase family protein | 4.77769 | 12.5302 | 1.39103 | 0.00455 | 0.0220583 |
| AT4G03090 | sequence-specific DNA binding;sequence-specific DNA binding transcription factors | 2.36958 | 6.21609 | 1.39138 | 0.00745 | 0.0318523 |
| AT2G40010 | Ribosomal protein L10 family protein | 9.40867 | 24.6959 | 1.39221 | 0.005 | 0.0237003 |
| AT5G05730 | AMT1, ASA1, JDL1, TRP5, WEI2, anthranilate synthase alpha subunit 1 | 34.1044 | 89.5548 | 1.39281 | 0.00135 | 0.0089118 |
| AT1G31860 | AT-IE, HISN2, histidine biosynthesis bifunctional protein (HISIE) | 14.7889 | 38.8499 | 1.3934 | 0.00425 | 0.0209475 |
| AT4G28510 | ATPHB1, PHB1, prohibitin 1 | 9.56059 | 25.1294 | 1.39421 | 0.0066 | 0.0290976 |
| AT3G59600 | NRPB8B, NRPD8B, NRPE8B, RNA polymerase Rpb8 | 11.656 | 30.6416 | 1.39441 | 0.0081 | 0.0338066 |
| AT2G31955 | CNX2, cofactor of nitrate reductase and xanthine dehydrogenase 2 | 11.672 | 30.6907 | 1.39475 | 0.0025 | 0.0140894 |
| AT1G57600 | MBOAT (membrane bound O-acyl transferase) family protein | 2.87173 | 7.5613 | 1.39671 | 0.01145 | 0.0435148 |
| AT4G15900 | PRL1, pleiotropic regulatory locus 1 | 14.6778 | 38.7042 | 1.39885 | 0.00165 | 0.0103801 |
| AT3G12810 | chr13, PIE1, SRCAP, SNF2 domain-containing protein / helicase domain-containing protein | 2.19684 | 5.79364 | 1.39904 | 0.00255 | 0.014306 |
| AT2G46890 | Protein of unknown function (DUF1295) | 5.04055 | 13.3112 | 1.40099 | 0.0107 | 0.0414196 |
| AT4G22000 | unknown protein | 18.3545 | 48.5731 | 1.40403 | 0.00305 | 0.0163293 |
| AT2G37050 | Leucine-rich repeat protein kinase family protein | 4.33708 | 11.4796 | 1.40428 | 0.002 | 0.0119879 |
| AT3G07060 | emb1974, NHL domain-containing protein | 2.14943 | 5.69109 | 1.40475 | 0.00995 | 0.0392764 |
| AT3G54750 | unknown protein | 6.83067 | 18.0858 | 1.40476 | 0.00495 | 0.0235479 |
| AT1G06090 | Fatty acid desaturase family protein | 23.4209 | 62.0475 | 1.40558 | 0.00325 | 0.0171315 |
| AT3G15120 | P-loop containing nucleoside triphosphate hydrolases superfamily protein | 2.5475 | 6.76937 | 1.40994 | 0.002 | 0.0119879 |
| AT5G57120 | function unknown | 14.3608 | 38.1706 | 1.41033 | 0.0022 | 0.0128446 |
| AT1G70370 | PG2, polygalacturonase 2 | 2.52032 | 6.7052 | 1.41168 | 0.00975 | 0.0387185 |
| AT2G35240 | plastid developmental protein DAG, putative | 10.815 | 28.7742 | 1.41174 | 0.01065 | 0.0412906 |
| AT5G49820 | EMB1879, RUS6, Protein of unknown function, DUF647 | 4.70262 | 12.5193 | 1.41261 | 0.0033 | 0.0173075 |
| AT5G50400 | ATPAP27, PAP27, purple acid phosphatase 27 | 1.77958 | 4.7424 | 1.41408 | 0.0124 | 0.046125 |
| AT3G10970 | Haloacid dehalogenase-like hydrolase (HAD) superfamily protein | 2.89601 | 7.72142 | 1.4148 | 0.0101 | 0.039726 |
| AT1G26190 | Phosphoribulokinase / Uridine kinase family | 1.96298 | 5.23418 | 1.41492 | 0.00785 | 0.0330699 |
| AT3G43600 | AAO2, AO3, AOgamma, atAO-2, AtAO3, aldehyde oxidase 2 | 4.47627 | 11.9409 | 1.41554 | 0.0035 | 0.0180741 |
| AT1G67430 | Ribosomal protein L22p/L17e family protein | 202.884 | 541.451 | 1.41618 | 0.0014 | 0.00917155 |
| AT3G56580 | RING/U-box superfamily protein | 4.25148 | 11.3512 | 1.41681 | 0.0085 | 0.0349894 |
| AT4G07410 | Transducin family protein / WD-40 repeat family protein | 9.67675 | 25.847 | 1.4174 | 0.0023 | 0.0132526 |
| AT1G35510 | O-fucosyltransferase family protein | 3.68814 | 9.85906 | 1.41856 | 0.00275 | 0.0151029 |
| AT5G59090 | ATSBT4.12, SBT4.12, subtilase 4.12 | 3.47996 | 9.3035 | 1.4187 | 0.00355 | 0.0182751 |
| AT3G45010 | scpl48, serine carboxypeptidase-like 48 | 49.003 | 131.181 | 1.42061 | 0.00365 | 0.0186349 |
| AT5G04130 | GYRB2, DNA GYRASE B2 | 3.82739 | 10.2499 | 1.42118 | 0.00395 | 0.0198341 |
| AT4G26880 | Stigma-specific Stig1 family protein | 21.056 | 56.4067 | 1.42163 | 0.01145 | 0.0435148 |
| AT5G04290 | KTF1, SPT5L, kow domain-containing transcription factor 1 | 0.858994 | 2.30269 | 1.4226 | 0.01 | 0.0393953 |
| AT5G64680 | unknown protein | 7.52167 | 20.1638 | 1.42264 | 0.01065 | 0.0412906 |
| AT1G49580 | Calcium-dependent protein kinase (CDPK) family protein | 4.99125 | 13.3824 | 1.42287 | 0.004 | 0.0200243 |
| AT1G33590 | Leucine-rich repeat (LRR) family protein | 64.9448 | 174.174 | 1.42324 | 0.0011 | 0.00759303 |
| AT4G20070 | AAH, ATAAH, allantoate amidohydrolase | 4.15922 | 11.1587 | 1.42379 | 0.0076 | 0.0322984 |
| AT4G09140 | ATMLH1, MLH1, MUTL-homologue 1 | 2.64716 | 7.10714 | 1.42482 | 0.00525 | 0.0246552 |
| AT1G23290 | RPL27A, RPL27AB, Ribosomal protein L18e/L15 superfamily protein | 104.011 | 279.255 | 1.42484 | 0.00195 | 0.0117451 |
| AT1G66750 | AT;CDKD;2, CAK4, CAK4AT, CDKD1;2, CDKD;2, CDK-activating kinase 4 | 3.68289 | 9.89117 | 1.4253 | 0.0134 | 0.0487461 |
| AT3G53350 | RIP4, ROP interactive partner 4 | 4.19273 | 11.2608 | 1.42534 | 0.00985 | 0.0390139 |
| AT1G09590 | Translation protein SH3-like family protein | 62.0026 | 166.559 | 1.42564 | 0.00315 | 0.0166977 |
| AT5G35910 | Polynucleotidyl transferase, ribonuclease H fold protein with HRDC domain | 4.55823 | 12.2607 | 1.4275 | 0.00375 | 0.0189985 |
| AT3G13860 | HSP60-3A, heat shock protein 60-3A | 10.9142 | 29.3709 | 1.42818 | 0.00245 | 0.0139065 |
| AT5G63870 | ATPP7, PP7, serine/threonine phosphatase 7 | 8.15488 | 21.9456 | 1.4282 | 0.0056 | 0.0258574 |
| AT5G19340 | unknown protein | 26.3961 | 71.0811 | 1.42914 | 0.00295 | 0.0159014 |
| AT2G15730 | P-loop containing nucleoside triphosphate hydrolases superfamily protein | 5.60347 | 15.0951 | 1.42969 | 0.00565 | 0.0260215 |
| AT4G34200 | EDA9, D-3-phosphoglycerate dehydrogenase | 35.2852 | 95.095 | 1.43031 | 0.00125 | 0.00840292 |
| AT5G45160 | Root hair defective 3 GTP-binding protein (RHD3) | 2.29141 | 6.1777 | 1.43083 | 0.0068 | 0.029741 |
| AT4G04885 | PCFS4, PCF11P-similar protein 4 | 2.47612 | 6.68271 | 1.43235 | 0.0069 | 0.0300655 |
| AT4G02230 | Ribosomal protein L19e family protein | 32.0766 | 86.6097 | 1.43301 | 0.0018 | 0.0110912 |
| AT1G70560 | SAV3, TAA1, WEI8, tryptophan aminotransferase of Arabidopsis 1 | 7.64519 | 20.6513 | 1.43361 | 0.0134 | 0.0487461 |
| AT5G63800 | BGAL6, MUM2, Glycosyl hydrolase family 35 protein | 13.8423 | 37.391 | 1.43361 | 0.0015 | 0.00967937 |
| AT5G23900 | Ribosomal protein L13e family protein | 36.691 | 99.1495 | 1.43418 | 0.0026 | 0.0145003 |
| AT2G29390 | ATSMO2, SMO2-2, sterol 4-alpha-methyl-oxidase 2-2 | 11.0316 | 29.8157 | 1.43443 | 0.0042 | 0.0207631 |
| AT2G38040 | CAC3, acetyl Co-enzyme a carboxylase carboxyltransferase alpha subunit | 23.8833 | 64.6057 | 1.43566 | 0.0015 | 0.00967937 |
| AT3G07990 | SCPL27, serine carboxypeptidase-like 27 | 7.14969 | 19.371 | 1.43795 | 0.00905 | 0.0366741 |
| AT3G52590 | EMB2167, ERD16, HAP4, UBQ1, ubiquitin extension protein 1 | 241.465 | 655.215 | 1.44015 | 0.0013 | 0.00867426 |
| AT3G62940 | Cysteine proteinases superfamily protein | 7.33259 | 19.9188 | 1.44174 | 0.00595 | 0.0269704 |
| AT2G30340 | LBD13, LOB domain-containing protein 13 | 38.1545 | 103.73 | 1.4429 | 0.00205 | 0.012221 |
| AT4G30080 | ARF16, auxin response factor 16 | 11.8778 | 32.2976 | 1.44316 | 0.0013 | 0.00867426 |
| AT1G69830 | AMY3, ATAMY3, alpha-amylase-like 3 | 6.51576 | 17.7192 | 1.44331 | 0.00155 | 0.009918 |
| AT1G52690 | Late embryogenesis abundant protein (LEA) family protein | 111.235 | 302.545 | 1.44354 | 0.00045 | 0.003919 |
| AT1G72560 | PSD, ARM repeat superfamily protein | 2.16189 | 5.88494 | 1.44474 | 0.00485 | 0.0231612 |
| AT4G02340 | alpha/beta-Hydrolases superfamily protein | 6.91414 | 18.8245 | 1.44499 | 0.0068 | 0.029741 |
| AT5G40300 | Uncharacterised protein family (UPF0497) | 4.05612 | 11.0453 | 1.44525 | 0.0114 | 0.0433581 |
| AT5G60210 | RIP5, ROP interactive partner 5 | 5.2137 | 14.2015 | 1.44566 | 0.004 | 0.0200243 |
| AT1G06190 | Rho termination factor | 5.1166 | 13.9402 | 1.44599 | 0.00655 | 0.0289545 |
| AT4G21430 | B160, Zinc finger, RING-type;Transcription factor jumonji/aspartyl beta-hydroxylase | 3.01835 | 8.22906 | 1.44697 | 0.0041 | 0.0203755 |
| AT1G56045 | Ribosomal protein L41 family | 186.746 | 510.413 | 1.45058 | 0.0127 | 0.0469062 |
| AT3G59820 | LETM1-like protein | 8.96535 | 24.5297 | 1.4521 | 0.00115 | 0.00787231 |
| AT4G38130 | ATHD1, ATHDA19, HD1, HDA1, HDA19, RPD3A, histone deacetylase 1 | 29.8022 | 81.5549 | 1.45235 | 0.0007 | 0.00547072 |
| AT5G49510 | PFD3, prefoldin 3 | 22.7683 | 62.3262 | 1.45281 | 0.00175 | 0.0108609 |
| AT2G35320 | ATEYA, EYA, EYES ABSENT homolog | 5.18318 | 14.1892 | 1.45288 | 0.00475 | 0.0228046 |
| AT5G41020 | myb family transcription factor | 6.72407 | 18.4102 | 1.4531 | 0.005 | 0.0237003 |
| AT1G73230 | Nascent polypeptide-associated complex NAC | 63.2581 | 173.205 | 1.45316 | 0.00105 | 0.00735817 |
| AT5G64420 | DNA polymerase V family | 5.59849 | 15.3409 | 1.45427 | 0.00165 | 0.0103801 |
| AT1G80560 | ATIMD2, IMD2, isopropylmalate dehydrogenase 2 | 10.6229 | 29.1191 | 1.45478 | 0.001 | 0.00711095 |
| AT1G04510 | MAC3A, MOS4-associated complex 3A | 10.4081 | 28.6156 | 1.45909 | 0.001 | 0.00711095 |
| AT5G20290 | Ribosomal protein S8e family protein | 218.415 | 600.703 | 1.45958 | 0.00125 | 0.00840292 |
| AT2G16370 | THY-1, thymidylate synthase 1 | 2.39089 | 6.57809 | 1.46012 | 0.01085 | 0.0417717 |
| AT1G69840 | SPFH/Band 7/PHB domain-containing membrane-associated protein family | 12.4707 | 34.3177 | 1.46041 | 0.00375 | 0.0189985 |
| AT5G55660 | DEK domain-containing chromatin associated protein | 13.122 | 36.1259 | 1.46105 | 0.00135 | 0.0089118 |
| AT3G51430 | SSL5, YLS2, Calcium-dependent phosphotriesterase superfamily protein | 2.69605 | 7.42332 | 1.46122 | 0.0098 | 0.0388625 |
| AT3G06030 | ANP3, MAPKKK12, NP3, NPK1-related protein kinase 3 | 4.08958 | 11.2691 | 1.46234 | 0.0031 | 0.0165078 |
| AT5G18820 | EMB3007, TCP-1/cpn60 chaperonin family protein | 1.46828 | 4.04785 | 1.46303 | 0.0105 | 0.0408771 |
| AT1G70190 | Ribosomal protein L7/L12, oligomerisation;Ribosomal protein L7/L12, C-terminal/adaptor protein ClpS-like | 8.34864 | 23.0182 | 1.46316 | 0.00775 | 0.0327601 |
| AT1G72990 | BGAL17, beta-galactosidase 17 | 1.72366 | 4.75415 | 1.46371 | 0.00995 | 0.0392764 |
| AT2G21610 | ATPE11, PE11, pectinesterase 11 | 22.6871 | 62.5818 | 1.46387 | 0.0019 | 0.0115387 |
| AT2G36170 | Ubiquitin supergroup;Ribosomal protein L40e | 39.0439 | 107.766 | 1.46474 | 0.00105 | 0.00735817 |
| AT2G18940 | Tetratricopeptide repeat (TPR)-like superfamily protein | 1.28634 | 3.55223 | 1.46546 | 0.00945 | 0.0377542 |
| AT3G46970 | ATPHS2, PHS2, alpha-glucan phosphorylase 2 | 3.60006 | 9.94669 | 1.4662 | 0.0029 | 0.0157089 |
| AT2G19490 | recA DNA recombination family protein | 1.85556 | 5.12735 | 1.46636 | 0.01055 | 0.0410315 |
| AT5G52882 | P-loop containing nucleoside triphosphate hydrolases superfamily protein | 10.9377 | 30.242 | 1.46725 | 0.0008 | 0.00603091 |
| AT1G18670 | IBS1, Protein kinase superfamily protein | 1.82317 | 5.04147 | 1.46739 | 0.0106 | 0.0411532 |
| AT2G45640 | ATSAP18, SAP18, SIN3 associated polypeptide P18 | 18.4281 | 50.9979 | 1.46853 | 0.0023 | 0.0132526 |
| AT1G70600 | Ribosomal protein L18e/L15 superfamily protein | 110.991 | 307.179 | 1.46863 | 0.00125 | 0.00840292 |
| AT2G24600 | Ankyrin repeat family protein | 10.09 | 27.9264 | 1.46871 | 0.004 | 0.0200243 |
| AT5G62880 | ARAC10, ATRAC10, ATROP11, RAC10, RAC-like 10 | 25.2184 | 69.8448 | 1.46967 | 0.00125 | 0.00840292 |
| AT2G39040 | Peroxidase superfamily protein | 6.09021 | 16.8776 | 1.47054 | 0.00965 | 0.0383598 |
| AT4G21960 | PRXR1, Peroxidase superfamily protein | 36.811 | 102.031 | 1.47079 | 0.00045 | 0.003919 |
| AT3G57800 | basic helix-loop-helix (bHLH) DNA-binding superfamily protein | 6.78936 | 18.8184 | 1.47079 | 0.0026 | 0.0145003 |
| AT5G50370 | Adenylate kinase family protein | 7.74263 | 21.4611 | 1.47083 | 0.0056 | 0.0258574 |
| AT1G73950 | Transmembrane Fragile-X-F-associated protein | 1.64787 | 4.57238 | 1.47234 | 0.00955 | 0.0380846 |
| AT4G16570 | ATPRMT7, PRMT7, protein arginine methyltransferase 7 | 1.21162 | 3.36523 | 1.47377 | 0.0119 | 0.0448549 |
| AT5G13120 | ATCYP20-2, CYP20-2, cyclophilin 20-2 | 9.99649 | 27.8233 | 1.4768 | 0.00245 | 0.0139065 |
| AT1G11000 | ATMLO4, MLO4, Seven transmembrane MLO family protein | 23.6523 | 65.938 | 1.47913 | 0.00045 | 0.003919 |
| AT1G64980 | Nucleotide-diphospho-sugar transferases superfamily protein | 19.6373 | 54.7721 | 1.47984 | 0.00145 | 0.00940576 |
| AT2G19640 | ASHR2, SDG39, ASH1-related protein 2 | 2.88973 | 8.06065 | 1.47996 | 0.0075 | 0.0319763 |
| AT5G53500 | Transducin/WD40 repeat-like superfamily protein | 16.6055 | 46.3983 | 1.48241 | 0.0004 | 0.00359419 |
| AT1G08780 | AIP3, PFD4, ABI3-interacting protein 3 | 19.142 | 53.5117 | 1.48311 | 0.0045 | 0.0218802 |
| AT1G49410 | TOM6, translocase of the outer mitochondrial membrane 6 | 21.7843 | 60.946 | 1.48425 | 0.0057 | 0.0262091 |
| AT5G45600 | GAS41, TAF14B, YEATS family protein | 4.89243 | 13.6897 | 1.48447 | 0.0063 | 0.0281701 |
| AT2G20190 | ATCLASP, CLASP, CLIP-associated protein | 5.45915 | 15.2765 | 1.48457 | 0.00085 | 0.00630464 |
| AT1G10120 | basic helix-loop-helix (bHLH) DNA-binding superfamily protein | 4.6312 | 12.9788 | 1.48669 | 0.0071 | 0.0306604 |
| AT1G07770 | RPS15A, ribosomal protein S15A | 78.7206 | 220.625 | 1.48679 | 0.00095 | 0.00685384 |
| AT1G48510 | Surfeit locus 1 cytochrome c oxidase biogenesis protein | 4.16121 | 11.6687 | 1.48757 | 0.0081 | 0.0338066 |
| AT1G76660 | function unknown | 8.25516 | 23.1574 | 1.48811 | 0.0025 | 0.0140894 |
| AT1G76280 | Tetratricopeptide repeat (TPR)-like superfamily protein | 2.02529 | 5.68891 | 1.49002 | 0.00705 | 0.0305176 |
| AT3G16440 | ATMLP-300B, MEE36, MLP-300B, myrosinase-binding protein-like protein-300B | 87.2407 | 245.075 | 1.49015 | 0.0004 | 0.00359419 |
| AT4G00100 | ATRPS13A, PFL2, RPS13, RPS13A, ribosomal protein S13A | 76.2889 | 214.325 | 1.49025 | 0.00045 | 0.003919 |
| AT2G36250 | ATFTSZ2-1, FTSZ2-1, Tubulin/FtsZ family protein | 3.3905 | 9.53095 | 1.49112 | 0.00505 | 0.0239202 |
| AT2G18330 | AAA-type ATPase family protein | 3.06277 | 8.61148 | 1.49143 | 0.0053 | 0.0248135 |
| AT4G14210 | PDE226, PDS, PDS3, phytoene desaturase 3 | 1.46069 | 4.10786 | 1.49173 | 0.01025 | 0.0401565 |
| AT4G12700 | unknown protein | 2.10878 | 5.93146 | 1.49198 | 0.01 | 0.0393953 |
| AT4G25880 | APUM6, PUM6, pumilio 6 | 3.63668 | 10.2301 | 1.49212 | 0.00225 | 0.0130442 |
| AT1G03110 | Transducin/WD40 repeat-like superfamily protein | 4.22809 | 11.9111 | 1.49423 | 0.00475 | 0.0228046 |
| AT1G77470 | RFC3, RFC5, replication factor C subunit 3 | 3.43008 | 9.66546 | 1.4946 | 0.0085 | 0.0349894 |
| AT3G17690 | ATCNGC19, CNGC19, cyclic nucleotide gated channel 19 | 4.3348 | 12.2159 | 1.49472 | 0.00255 | 0.014306 |
| AT3G05590 | RPL18, ribosomal protein L18 | 177.486 | 500.291 | 1.49506 | 0.0005 | 0.00424432 |
| AT5G52370 | unknown protein | 5.49772 | 15.4993 | 1.4953 | 0.0096 | 0.0382301 |
| AT5G12250 | TUB6, beta-6 tubulin | 23.2084 | 65.4639 | 1.49605 | 0.0006 | 0.00487984 |
| AT5G24280 | GMI1, gamma-irradiation and mitomycin c induced 1 | 1.19237 | 3.36636 | 1.49736 | 0.00435 | 0.0212815 |
| AT5G64140 | RPS28, ribosomal protein S28 | 101.388 | 286.412 | 1.49821 | 0.0013 | 0.00867426 |
| AT1G19850 | ARF5, IAA24, MP, Transcriptional factor B3 family protein / auxin-responsive factor AUX/IAA-related | 21.7545 | 61.4566 | 1.49825 | 0.00055 | 0.00457655 |
| AT1G16870 | mitochondrial 28S ribosomal protein S29-related | 7.37629 | 20.8393 | 1.49834 | 0.00255 | 0.014306 |
| AT1G15810 | S15/NS1, RNA-binding protein | 7.0204 | 19.8383 | 1.49867 | 0.0039 | 0.0196279 |
| AT1G02930 | ATGST1, ATGSTF3, ATGSTF6, ERD11, GST1, GSTF6, glutathione S-transferase 6 | 32.8692 | 92.9471 | 1.49967 | 0.001 | 0.00711095 |
| AT2G35920 | RNA helicase family protein | 3.15812 | 8.93108 | 1.49977 | 0.00255 | 0.014306 |
| AT4G26620 | Sucrase/ferredoxin-like family protein | 3.72266 | 10.5286 | 1.49991 | 0.0037 | 0.0188077 |
| AT4G20910 | CRM2, HEN1, double-stranded RNA binding protein-related / DsRBD protein-related | 1.0639 | 3.01087 | 1.50082 | 0.0084 | 0.0346931 |
| AT5G05560 | EMB2771, E3 ubiquitin ligase, putative | 1.05975 | 3.00061 | 1.50153 | 0.00395 | 0.0198341 |
| AT2G36740 | ATSWC2, SWC2, sequence-specific DNA binding transcription factors;DNA binding;DNA binding | 6.80351 | 19.27 | 1.50201 | 0.0019 | 0.0115387 |
| AT2G42600 | ATPPC2, PPC2, phosphoenolpyruvate carboxylase 2 | 0.81601 | 2.31172 | 1.50231 | 0.01035 | 0.0404522 |
| AT4G15560 | CLA, CLA1, DEF, DXPS2, DXS, Deoxyxylulose-5-phosphate synthase | 4.27651 | 12.1156 | 1.50236 | 0.00215 | 0.0126571 |
| AT1G02560 | CLPP5, NCLPP1, NCLPP5, nuclear encoded CLP protease 5 | 13.2576 | 37.5611 | 1.50242 | 0.003 | 0.0161227 |
| AT5G52470 | ATFBR1, ATFIB1, FBR1, FIB1, SKIP7, fibrillarin 1 | 22.7644 | 64.4985 | 1.50249 | 0.0022 | 0.0128446 |
| AT3G25680 | function unknown | 1.58313 | 4.48637 | 1.50277 | 0.0091 | 0.0368164 |
| AT1G17880 | ATBTF3, BTF3, basic transcription factor 3 | 64.444 | 182.702 | 1.50338 | 0.0011 | 0.00759303 |
| AT1G24764 | ATMAP70-2, MAP70-2, microtubule-associated proteins 70-2 | 8.09671 | 22.9579 | 1.50358 | 0.0098 | 0.0388625 |
| AT3G13580 | Ribosomal protein L30/L7 family protein | 24.8403 | 70.5245 | 1.50544 | 0.0019 | 0.0115387 |
| AT4G01210 | glycosyl transferase family 1 protein | 3.50091 | 9.93979 | 1.50548 | 0.00205 | 0.012221 |
| AT1G30330 | ARF6, auxin response factor 6 | 3.66311 | 10.4021 | 1.50574 | 0.0033 | 0.0173075 |
| AT4G33250 | ATTIF3K1, EIF3K, TIF3K1, eukaryotic translation initiation factor 3K | 37.2538 | 105.804 | 1.50594 | 0.00065 | 0.00516972 |
| AT5G57930 | APO2, emb1629, Arabidopsis thaliana protein of unknown function (DUF794) | 1.86229 | 5.28965 | 1.50609 | 0.00805 | 0.0336689 |
| AT3G13570 | SCL30A, SC35-like splicing factor 30A | 6.94106 | 19.7262 | 1.50688 | 0.0038 | 0.0192125 |
| AT2G25710 | HCS1, holocarboxylase synthase 1 | 3.85211 | 10.9594 | 1.50844 | 0.0078 | 0.0329152 |
| AT2G39300 | unknown protein | 2.42585 | 6.90182 | 1.50849 | 0.0052 | 0.0244668 |
| AT2G31410 | unknown protein | 17.3715 | 49.4705 | 1.50985 | 0.0036 | 0.0184414 |
| AT5G15450 | APG6, CLPB-P, CLPB3, casein lytic proteinase B3 | 25.3239 | 72.1246 | 1.50999 | 0.00755 | 0.0321272 |
| AT4G33760 | tRNA synthetase class II (D, K and N) family protein | 2.67459 | 7.62842 | 1.51207 | 0.00355 | 0.0182751 |
| AT5G55490 | ATGEX1, GEX1, gamete expressed protein 1 | 1.6451 | 4.69338 | 1.51245 | 0.0128 | 0.0471875 |
| AT5G40520 | unknown protein | 1.61552 | 4.60986 | 1.51273 | 0.008 | 0.0335306 |
| AT1G63640 | P-loop nucleoside triphosphate hydrolases superfamily protein with CH (Calponin Homology) domain | 2.74259 | 7.83338 | 1.51409 | 0.00255 | 0.014306 |
| AT3G12200 | AtNek7, Nek7, NIMA-related kinase 7 | 4.52114 | 12.9236 | 1.51525 | 0.0025 | 0.0140894 |
| AT4G24190 | AtHsp90-7, AtHsp90.7, HSP90.7, SHD, Chaperone protein htpG family protein | 42.4991 | 121.498 | 1.51543 | 0.00075 | 0.00575475 |
| AT2G19730 | Ribosomal L28e protein family | 149.85 | 428.481 | 1.51571 | 0.0005 | 0.00424432 |
| AT2G19470 | ckl5, casein kinase I-like 5 | 5.96176 | 17.0669 | 1.51739 | 0.00205 | 0.012221 |
| AT3G54760 | dentin sialophosphoprotein-related | 7.93217 | 22.7242 | 1.51844 | 0.00115 | 0.00787231 |
| AT2G31610 | Ribosomal protein S3 family protein | 81.5254 | 233.666 | 1.51913 | 0.00055 | 0.00457655 |
| AT2G30860 | ATGSTF7, ATGSTF9, GLUTTR, GSTF9, glutathione S-transferase PHI 9 | 58.1687 | 166.785 | 1.51968 | 0.0006 | 0.00487984 |
| AT3G01770 | ATBET10, BET10, bromodomain and extraterminal domain protein 10 | 4.32034 | 12.3982 | 1.52091 | 0.0018 | 0.0110912 |
| AT1G60850 | AAC42, ATRPAC42, DNA-directed RNA polymerase family protein | 4.44371 | 12.7581 | 1.52158 | 0.00625 | 0.0280351 |
| AT4G18240 | ATSS4, SS4, SSIV, starch synthase 4 | 3.11395 | 8.94212 | 1.52187 | 0.0023 | 0.0132526 |
| AT3G22180 | DHHC-type zinc finger family protein | 1.40587 | 4.03932 | 1.52265 | 0.0077 | 0.0326253 |
| AT1G54890 | Late embryogenesis abundant (LEA) protein-related | 9.44591 | 27.1481 | 1.52309 | 0.00295 | 0.0159014 |
| AT2G04390 | Ribosomal S17 family protein | 19.271 | 55.3886 | 1.52316 | 0.00905 | 0.0366741 |
| AT4G27380 | unknown protein | 5.17705 | 14.8867 | 1.52382 | 0.0117 | 0.0442695 |
| AT4G13750 | NOV, Histidine kinase-, DNA gyrase B-, and HSP90-like ATPase family protein | 0.716129 | 2.06036 | 1.52461 | 0.00315 | 0.0166977 |
| AT1G75670 | DNA-directed RNA polymerases | 3.8554 | 11.0924 | 1.52462 | 0.01125 | 0.0429112 |
| AT1G51745 | Tudor/PWWP/MBT superfamily protein | 2.55673 | 7.35881 | 1.52517 | 0.00455 | 0.0220583 |
| AT5G53770 | Nucleotidyltransferase family protein | 3.85369 | 11.0947 | 1.52556 | 0.0041 | 0.0203755 |
| AT2G27110 | FRS3, FAR1-related sequence 3 | 2.68816 | 7.74158 | 1.52601 | 0.00495 | 0.0235479 |
| AT1G32700 | PLATZ transcription factor family protein | 36.8889 | 106.25 | 1.52621 | 0.00065 | 0.00516972 |
| AT4G33865 | Ribosomal protein S14p/S29e family protein | 108.764 | 313.296 | 1.52633 | 0.00245 | 0.0139065 |
| AT4G36130 | Ribosomal protein L2 family | 45.1305 | 130.075 | 1.52717 | 0.0003 | 0.00292382 |
| AT4G25740 | RNA binding Plectin/S10 domain-containing protein | 23.4433 | 67.5948 | 1.52774 | 0.00225 | 0.0130442 |
| AT3G23990 | HSP60, HSP60-3B, heat shock protein 60 | 36.3393 | 104.925 | 1.52975 | 0.0004 | 0.00359419 |
| AT5G19920 | Transducin/WD40 repeat-like superfamily protein | 1.82759 | 5.28004 | 1.5306 | 0.0069 | 0.0300655 |
| AT2G38770 | EMB2765, P-loop containing nucleoside triphosphate hydrolases superfamily protein | 8.15917 | 23.5788 | 1.53099 | 0.0003 | 0.00292382 |
| AT5G50330 | Protein kinase superfamily protein | 4.84476 | 14.0014 | 1.53108 | 0.00215 | 0.0126571 |
| AT4G16720 | Ribosomal protein L23/L15e family protein | 81.481 | 235.805 | 1.53306 | 0.00005 | 0.00070028 |
| AT5G36950 | DegP10, DegP protease 10 | 2.28828 | 6.62582 | 1.53383 | 0.00875 | 0.0357584 |
| AT3G49410 | Transcription factor IIIC, subunit 5 | 1.49585 | 4.33179 | 1.53399 | 0.0087 | 0.0356202 |
| AT2G42910 | Phosphoribosyltransferase family protein | 9.63691 | 27.9103 | 1.53415 | 0.0015 | 0.00967937 |
| AT2G42570 | TBL39, TRICHOME BIREFRINGENCE-LIKE 39 | 40.3608 | 116.921 | 1.5345 | 0.0008 | 0.00603091 |
| AT4G22970 | AESP, ESP, ESP, RSW4, homolog of separase | 0.554673 | 1.60729 | 1.53492 | 0.0066 | 0.0290976 |
| AT4G24940 | AT-SAE1-1, ATSAE1A, SAE1A, SUMO-activating enzyme 1A | 7.98072 | 23.1305 | 1.53521 | 0.00275 | 0.0151029 |
| AT3G03950 | ECT1, evolutionarily conserved C-terminal region 1 | 6.14331 | 17.8056 | 1.53524 | 0.00235 | 0.0134663 |
| AT5G04510 | ATPDK1, PDK1, 3'-phosphoinositide-dependent protein kinase 1 | 5.55641 | 16.1133 | 1.53603 | 0.00275 | 0.0151029 |
| AT5G16390 | BCCP, BCCP-1, BCCP1, CAC1, CAC1-A, CAC1A, chloroplastic acetylcoenzyme A carboxylase 1 | 11.4652 | 33.2857 | 1.53764 | 0.00285 | 0.0154761 |
| AT1G20220 | Alba DNA/RNA-binding protein | 14.6404 | 42.5307 | 1.53855 | 0.001 | 0.00711095 |
| AT5G02610 | Ribosomal L29 family protein | 33.9452 | 98.6777 | 1.53952 | 0.00145 | 0.00940576 |
| AT5G60960 | Pentatricopeptide repeat (PPR) superfamily protein | 10.3729 | 30.1731 | 1.54045 | 0.00125 | 0.00840292 |
| AT2G28380 | DRB2, dsRNA-binding protein 2 | 4.06136 | 11.8221 | 1.54146 | 0.0037 | 0.0188077 |
| AT4G18465 | RNA helicase family protein | 4.56564 | 13.2931 | 1.54178 | 0.00145 | 0.00940576 |
| AT5G13520 | peptidase M1 family protein | 5.02999 | 14.651 | 1.54237 | 0.0018 | 0.0110912 |
| AT4G11450 | Protein of unknown function (DUF3527) | 1.20731 | 3.51854 | 1.54318 | 0.00605 | 0.0273236 |
| AT5G12940 | Leucine-rich repeat (LRR) family protein | 6.68961 | 19.4961 | 1.54319 | 0.00665 | 0.0292594 |
| AT5G06865 | other RNA | 49.6787 | 144.851 | 1.54387 | 0.0023 | 0.0132526 |
| AT3G11940 | AML1, ATRPS5A, RPS5A, ribosomal protein 5A | 105.121 | 306.907 | 1.54575 | 0.0003 | 0.00292382 |
| AT3G51090 | Protein of unknown function (DUF1640) | 4.52186 | 13.202 | 1.54576 | 0.007 | 0.0303609 |
| AT5G59590 | UGT76E2, UDP-glucosyl transferase 76E2 | 1.23723 | 3.61626 | 1.54738 | 0.0135 | 0.0489749 |
| AT3G61590 | HS, HWS, Galactose oxidase/kelch repeat superfamily protein | 2.83227 | 8.27848 | 1.54741 | 0.0067 | 0.0294336 |
| AT1G55130 | AtTMN6, TMN6, Endomembrane protein 70 protein family | 2.74851 | 8.03588 | 1.5478 | 0.00635 | 0.0283169 |
| AT1G26260 | CIB5, cryptochrome-interacting basic-helix-loop-helix 5 | 2.02873 | 5.9359 | 1.54889 | 0.01235 | 0.0460168 |
| AT5G18170 | GDH1, glutamate dehydrogenase 1 | 5.06556 | 14.8227 | 1.54902 | 0.0033 | 0.0173075 |
| AT1G78540 | ATSHB, SHB, STATLB, SH2 domain protein B | 2.59849 | 7.60993 | 1.55021 | 0.0037 | 0.0188077 |
| AT3G14980 | Acyl-CoA N-acyltransferase with RING/FYVE/PHD-type zinc finger protein | 1.49425 | 4.37638 | 1.55031 | 0.00615 | 0.0276932 |
| AT5G19090 | Heavy metal transport/detoxification superfamily protein | 2.59138 | 7.59178 | 1.55072 | 0.00525 | 0.0246552 |
| AT1G72370 | AP40, P40, RP40, RPSAA, 40s ribosomal protein SA | 108.382 | 317.522 | 1.55073 | 0.0002 | 0.00214445 |
| AT1G11860 | Glycine cleavage T-protein family | 19.5886 | 57.3918 | 1.55083 | 0.0011 | 0.00759303 |
| AT1G20580 | Small nuclear ribonucleoprotein family protein | 20.2933 | 59.4659 | 1.55106 | 0.0022 | 0.0128446 |
| AT4G18020 | APRR2, PRR2, CheY-like two-component responsive regulator family protein | 1.67418 | 4.90699 | 1.55138 | 0.01105 | 0.0423687 |
| AT3G18160 | PEX3-1, peroxin 3-1 | 2.52458 | 7.40422 | 1.5523 | 0.00755 | 0.0321272 |
| AT5G35940 | Mannose-binding lectin superfamily protein | 49.3543 | 144.916 | 1.55397 | 0.0003 | 0.00292382 |
| AT1G04270 | RPS15, cytosolic ribosomal protein S15 | 104.621 | 307.284 | 1.5544 | 0.00045 | 0.003919 |
| AT1G29880 | glycyl-tRNA synthetase / glycine--tRNA ligase | 10.3317 | 30.4127 | 1.5576 | 0.0005 | 0.00424432 |
| AT5G12930 | unknown protein | 1.94467 | 5.72578 | 1.55795 | 0.01255 | 0.0465083 |
| AT2G14740 | ATVSR3, BP80-2;2, VSR2;2, VSR3, VSR3, vaculolar sorting receptor 3 | 7.8508 | 23.1174 | 1.55807 | 0.00115 | 0.00787231 |
| AT3G13222 | GIP1, GBF-interacting protein 1 | 3.72791 | 10.9802 | 1.55847 | 0.0021 | 0.0124516 |
| AT5G61170 | Ribosomal protein S19e family protein | 67.3293 | 198.384 | 1.55899 | 0.0008 | 0.00603091 |
| AT4G14320 | Zinc-binding ribosomal protein family protein | 86.7677 | 255.661 | 1.559 | 0.00035 | 0.00327416 |
| AT1G61350 | ARM repeat superfamily protein | 2.54746 | 7.50785 | 1.55934 | 0.0028 | 0.0152969 |
| AT2G18900 | Transducin/WD40 repeat-like superfamily protein | 4.06887 | 12.0077 | 1.56126 | 0.0013 | 0.00867426 |
| AT5G15200 | Ribosomal protein S4 | 151.746 | 448.284 | 1.56275 | 0.00035 | 0.00327416 |
| AT3G20050 | ATTCP-1, TCP-1, T-complex protein 1 alpha subunit | 28.2907 | 83.6201 | 1.56352 | 0.0001 | 0.00123775 |
| AT1G75710 | C2H2-like zinc finger protein | 7.17571 | 21.2198 | 1.56422 | 0.0033 | 0.0173075 |
| AT1G22460 | O-fucosyltransferase family protein | 0.919708 | 2.72158 | 1.5652 | 0.01285 | 0.0472926 |
| AT1G70070 | EMB25, ISE2, PDE317, DEAD/DEAH box helicase, putative | 1.24655 | 3.68916 | 1.56535 | 0.0066 | 0.0290976 |
| AT1G72250 | Di-glucose binding protein with Kinesin motor domain | 0.927281 | 2.74435 | 1.56539 | 0.0071 | 0.0306604 |
| AT3G44320 | AtNIT3, NIT3, nitrilase 3 | 15.3103 | 45.3185 | 1.5656 | 0.00065 | 0.00516972 |
| AT4G02020 | EZA1, SDG10, SWN, SET domain-containing protein | 2.08911 | 6.18442 | 1.56575 | 0.00595 | 0.0269704 |
| AT1G49480 | RTV1, related to vernalization1 1 | 6.69453 | 19.8486 | 1.56799 | 0.00095 | 0.00685384 |
| AT2G01440 | DEAD/DEAH box RNA helicase family protein | 1.37175 | 4.06797 | 1.56829 | 0.0073 | 0.0313803 |
| AT5G09270 | unknown protein | 6.26951 | 18.5964 | 1.5686 | 0.01205 | 0.0451624 |
| AT3G16430 | JAL31, jacalin-related lectin 31 | 11.4996 | 34.1336 | 1.5696 | 0.00145 | 0.00940576 |
| AT1G17220 | FUG1, Translation initiation factor 2, small GTP-binding protein | 2.05838 | 6.11489 | 1.57081 | 0.00315 | 0.0166977 |
| AT1G80870 | Protein kinase superfamily protein | 1.50345 | 4.47015 | 1.57205 | 0.00545 | 0.025354 |
| AT3G09630 | Ribosomal protein L4/L1 family | 65.7655 | 195.693 | 1.57319 | 0.00005 | 0.00070028 |
| AT4G31600 | UDP-N-acetylglucosamine (UAA) transporter family | 1.93379 | 5.75559 | 1.57353 | 0.012 | 0.0450518 |
| AT5G14620 | DMT7, DRM2, domains rearranged methyltransferase 2 | 1.95551 | 5.82627 | 1.57503 | 0.0068 | 0.029741 |
| AT4G31820 | ENP, MAB4, NPY1, Phototropic-responsive NPH3 family protein | 6.16432 | 18.3662 | 1.57504 | 0.0015 | 0.00967937 |
| AT1G29470 | S-adenosyl-L-methionine-dependent methyltransferases superfamily protein | 42.5349 | 126.805 | 1.5759 | 0.00035 | 0.00327416 |
| AT3G01690 | alpha/beta-Hydrolases superfamily protein | 1.46945 | 4.38163 | 1.5762 | 0.01345 | 0.0488831 |
| AT4G36420 | Ribosomal protein L12 family protein | 9.49062 | 28.3357 | 1.57805 | 0.00335 | 0.0175049 |
| AT5G11480 | P-loop containing nucleoside triphosphate hydrolases superfamily protein | 1.54618 | 4.61887 | 1.57883 | 0.0133 | 0.048507 |
| AT1G14810 | semialdehyde dehydrogenase family protein | 14.5878 | 43.5799 | 1.5789 | 0.0007 | 0.00547072 |
| AT3G12550 | XH/XS domain-containing protein | 1.55789 | 4.65518 | 1.57924 | 0.00605 | 0.0273236 |
| AT5G63810 | BGAL10, beta-galactosidase 10 | 4.2397 | 12.6711 | 1.57951 | 0.0022 | 0.0128446 |
| AT3G13040 | myb-like HTH transcriptional regulator family protein | 3.8865 | 11.6167 | 1.57965 | 0.00295 | 0.0159014 |
| AT5G28640 | AN3, ATGIF1, GIF, GIF1, SSXT family protein | 2.99228 | 8.9458 | 1.57997 | 0.0114 | 0.0433581 |
| AT1G53800 | unknown protein | 1.58958 | 4.75347 | 1.58034 | 0.00885 | 0.0361074 |
| AT5G02010 | ATROPGEF7, ROPGEF7, RHO guanyl-nucleotide exchange factor 7 | 2.87201 | 8.59225 | 1.58097 | 0.0025 | 0.0140894 |
| AT2G45460 | SMAD/FHA domain-containing protein | 0.893202 | 2.67343 | 1.58163 | 0.01085 | 0.0417717 |
| AT5G05130 | DNA/RNA helicase protein | 0.776078 | 2.32434 | 1.58255 | 0.0129 | 0.0474149 |
| AT3G48930 | EMB1080, Nucleic acid-binding, OB-fold-like protein | 94.2605 | 282.545 | 1.58376 | 0.0002 | 0.00214445 |
| AT3G54720 | AMP1, COP2, HPT, MFO1, PT, Peptidase M28 family protein | 0.900296 | 2.70017 | 1.58458 | 0.0134 | 0.0487461 |
| AT2G33570 | Domain of unknown function (DUF23) | 4.01466 | 12.046 | 1.58521 | 0.0024 | 0.0136815 |
| AT1G27390 | TOM20-2, translocase outer membrane 20-2 | 12.916 | 38.762 | 1.58549 | 0.00115 | 0.00787231 |
| AT1G47490 | ATRBP47C, RBP47C, RNA-binding protein 47C | 3.85717 | 11.5792 | 1.58592 | 0.0051 | 0.0241339 |
| AT1G33140 | PGY2, Ribosomal protein L6 family | 9.08839 | 27.2992 | 1.58676 | 0.00315 | 0.0166977 |
| AT1G52980 | GTP-binding family protein | 8.0364 | 24.1479 | 1.58727 | 0.0024 | 0.0136815 |
| AT5G46430 | Ribosomal protein L32e | 81.081 | 243.717 | 1.58777 | 0.0004 | 0.00359419 |
| AT1G55900 | emb1860, TIM50, Haloacid dehalogenase-like hydrolase (HAD) superfamily protein | 5.54038 | 16.6537 | 1.58779 | 0.00065 | 0.00516972 |
| AT1G76680 | ATOPR1, OPR1, 12-oxophytodienoate reductase 1 | 192.188 | 578.059 | 1.5887 | 0.00125 | 0.00840292 |
| AT5G06460 | ATUBA2, UBA 2, ubiquitin activating enzyme 2 | 2.7654 | 8.32317 | 1.58964 | 0.00305 | 0.0163293 |
| AT5G05980 | ATDFB, DFB, DHFS-FPGS homolog B | 5.90056 | 17.7619 | 1.58986 | 0.00855 | 0.0351514 |
| AT2G34190 | Xanthine/uracil permease family protein | 1.40815 | 4.24137 | 1.59073 | 0.0112 | 0.0427699 |
| AT2G17730 | NIP2, NEP-interacting protein 2 | 1.48612 | 4.47707 | 1.591 | 0.01335 | 0.0486446 |
| AT1G20370 | Pseudouridine synthase family protein | 2.04626 | 6.1728 | 1.59293 | 0.0061 | 0.0274993 |
| AT5G46550 | DNA-binding bromodomain-containing protein | 3.61937 | 10.93 | 1.59448 | 0.0042 | 0.0207631 |
| AT3G23300 | S-adenosyl-L-methionine-dependent methyltransferases superfamily protein | 8.66649 | 26.1796 | 1.59492 | 0.0005 | 0.00424432 |
| AT1G01690 | ATPRD3, PRD3, putative recombination initiation defects 3 | 1.11883 | 3.38041 | 1.59521 | 0.01115 | 0.0426612 |
| AT5G44780 | unknown protein | 0.968891 | 2.9286 | 1.59581 | 0.0134 | 0.0487461 |
| AT2G38940 | ATPT2, PHT1;4, phosphate transporter 1;4 | 4.05908 | 12.275 | 1.5965 | 0.00245 | 0.0139065 |
| AT1G47500 | ATRBP47C', RBP47C', RNA-binding protein 47C' | 1.55217 | 4.69396 | 1.59652 | 0.012 | 0.0450518 |
| AT3G07770 | AtHsp90-6, AtHsp90.6, Hsp89.1, HEAT SHOCK PROTEIN 89.1 | 10.3436 | 31.302 | 1.59752 | 0.0016 | 0.0101494 |
| AT1G11750 | CLPP6, NCLPP1, NCLPP6, CLP protease proteolytic subunit 6 | 6.17701 | 18.698 | 1.5979 | 0.0045 | 0.0218802 |
| AT4G35335 | Nucleotide-sugar transporter family protein | 6.69301 | 20.2726 | 1.5988 | 0.00225 | 0.0130442 |
| AT5G13360 | Auxin-responsive GH3 family protein | 1.86755 | 5.65754 | 1.59903 | 0.0046 | 0.0222191 |
| AT3G13882 | Ribosomal protein L34 | 12.8366 | 38.8936 | 1.59926 | 0.0022 | 0.0128446 |
| AT5G28850 | Calcium-binding EF-hand family protein | 2.09864 | 6.36089 | 1.59977 | 0.01065 | 0.0412906 |
| AT3G46920 | Protein kinase superfamily protein with octicosapeptide/Phox/Bem1p domain | 1.65222 | 5.0125 | 1.60113 | 0.0027 | 0.0149318 |
| AT5G55820 | CONTAINS InterPro DOMAIN/s: Inner centromere protein, ARK-binding region (InterPro:IPR005635) | 1.45349 | 4.41067 | 1.60148 | 0.00395 | 0.0198341 |
| AT3G05030 | ATNHX2, NHX2, sodium hydrogen exchanger 2 | 5.13053 | 15.5769 | 1.60222 | 0.0016 | 0.0101494 |
| AT4G31700 | RPS6, RPS6A, ribosomal protein S6 | 93.9881 | 285.444 | 1.60266 | 0.00015 | 0.00170235 |
| AT4G29730 | MSI5, NFC5, nucleosome/chromatin assembly factor group C5 | 1.18245 | 3.59139 | 1.60276 | 0.01185 | 0.0447431 |
| AT3G04940 | ATCYSD1, CYSD1, cysteine synthase D1 | 5.10637 | 15.5167 | 1.60345 | 0.0027 | 0.0149318 |
| AT1G26880 | Ribosomal protein L34e superfamily protein | 49.1634 | 149.396 | 1.60349 | 0.0003 | 0.00292382 |
| AT4G17720 | RNA-binding (RRM/RBD/RNP motifs) family protein | 27.9788 | 85.0244 | 1.60354 | 0.0005 | 0.00424432 |
| AT5G05680 | MOS7, nuclear pore complex protein-related | 2.0997 | 6.38162 | 1.60374 | 0.0025 | 0.0140894 |
| AT2G16390 | CHR35, DMS1, DRD1, SNF2 domain-containing protein / helicase domain-containing protein | 1.5238 | 4.63446 | 1.60472 | 0.0021 | 0.0124516 |
| AT2G03670 | CDC48B, cell division cycle 48B | 1.08199 | 3.29151 | 1.60506 | 0.01335 | 0.0486446 |
| AT5G43700 | ATAUX2-11, IAA4, AUX/IAA transcriptional regulator family protein | 4.35558 | 13.2775 | 1.60805 | 0.00545 | 0.025354 |
| AT2G34750 | RNA polymerase I specific transcription initiation factor RRN3 protein | 11.011 | 33.6104 | 1.60996 | 0.00065 | 0.00516972 |
| AT2G36620 | RPL24A, ribosomal protein L24 | 50.0633 | 152.889 | 1.61066 | 0.0002 | 0.00214445 |
| AT1G29990 | PFD6, prefoldin 6 | 12.2651 | 37.4931 | 1.61206 | 0.00195 | 0.0117451 |
| AT1G34270 | Exostosin family protein | 1.65705 | 5.07115 | 1.6137 | 0.01015 | 0.0398672 |
| AT3G57050 | CBL, cystathionine beta-lyase | 7.90179 | 24.1943 | 1.61442 | 0.00095 | 0.00685384 |
| AT1G62970 | Chaperone DnaJ-domain superfamily protein | 1.04973 | 3.21433 | 1.61451 | 0.00445 | 0.0217117 |
| AT5G49990 | Xanthine/uracil permease family protein | 2.23481 | 6.84386 | 1.61466 | 0.0055 | 0.0254967 |
| AT1G08470 | SSL3, strictosidine synthase-like 3 | 2.93141 | 8.9846 | 1.61586 | 0.0116 | 0.0439836 |
| AT5G16710 | DHAR3, dehydroascorbate reductase 1 | 2.99291 | 9.17826 | 1.61667 | 0.01215 | 0.0454426 |
| AT1G45170 | unknown protein | 2.2316 | 6.84366 | 1.61669 | 0.0108 | 0.041652 |
| AT1G05410 | Protein of unknown function (DUF1423) | 2.45637 | 7.53344 | 1.61678 | 0.00695 | 0.0302036 |
| AT5G42630 | ATS, KAN4, Homeodomain-like superfamily protein | 3.0934 | 9.48822 | 1.61695 | 0.0117 | 0.0442695 |
| AT5G48760 | Ribosomal protein L13 family protein | 17.7102 | 54.3328 | 1.61724 | 0.0008 | 0.00603091 |
| AT5G25460 | Protein of unknown function, DUF642 | 17.7753 | 54.5325 | 1.61724 | 0.00185 | 0.0113253 |
| AT3G01380 | transferases;sulfuric ester hydrolases;catalytics;transferases | 2.54986 | 7.82272 | 1.61725 | 0.00145 | 0.00940576 |
| AT2G38650 | GAUT7, LGT7, galacturonosyltransferase 7 | 8.59218 | 26.3704 | 1.61782 | 0.00125 | 0.00840292 |
| AT5G28060 | Ribosomal protein S24e family protein | 194.707 | 597.791 | 1.61834 | 0.0001 | 0.00123775 |
| AT5G47190 | Ribosomal protein L19 family protein | 2.88428 | 8.85962 | 1.61904 | 0.00905 | 0.0366741 |
| AT2G20635 | ATP binding;protein kinases;protein serine/threonine kinases | 1.24895 | 3.84005 | 1.6204 | 0.0092 | 0.0370545 |
| AT3G06980 | DEA(D/H)-box RNA helicase family protein | 0.985662 | 3.03161 | 1.62092 | 0.01095 | 0.0420749 |
| AT2G36130 | Cyclophilin-like peptidyl-prolyl cis-trans isomerase family protein | 9.35365 | 28.807 | 1.62282 | 0.0022 | 0.0128446 |
| AT1G70670 | Caleosin-related family protein | 3.1013 | 9.55207 | 1.62294 | 0.00535 | 0.025018 |
| AT1G26740 | Ribosomal L32p protein family | 5.94432 | 18.3118 | 1.62319 | 0.0075 | 0.0319763 |
| AT2G31060 | elongation factor family protein | 8.02585 | 24.726 | 1.6233 | 0.00055 | 0.00457655 |
| AT2G35840 | Sucrose-6F-phosphate phosphohydrolase family protein | 8.8402 | 27.249 | 1.62405 | 0.00095 | 0.00685384 |
| AT2G44040 | Dihydrodipicolinate reductase, bacterial/plant | 2.65479 | 8.18476 | 1.62434 | 0.00445 | 0.0217117 |
| AT1G63180 | UGE3, UDP-D-glucose/UDP-D-galactose 4-epimerase 3 | 7.6266 | 23.5249 | 1.62507 | 0.0022 | 0.0128446 |
| AT1G32380 | PRS2, phosphoribosyl pyrophosphate (PRPP) synthase 2 | 2.54997 | 7.87475 | 1.62675 | 0.00515 | 0.024295 |
| AT1G77320 | MEI1, transcription coactivators | 1.48524 | 4.58703 | 1.62687 | 0.00185 | 0.0113253 |
| AT5G11200 | DEAD/DEAH box RNA helicase family protein | 6.6205 | 20.4477 | 1.62693 | 0.0012 | 0.00815259 |
| AT1G20410 | Pseudouridine synthase family protein | 2.28541 | 7.05875 | 1.62696 | 0.0046 | 0.0222191 |
| AT2G30590 | WRKY21, WRKY DNA-binding protein 21 | 5.19815 | 16.0556 | 1.62701 | 0.0024 | 0.0136815 |
| AT1G44446 | ATCAO, CAO, CH1, Pheophorbide a oxygenase family protein with Rieske [2Fe-2S] domain | 1.42646 | 4.40836 | 1.6278 | 0.0063 | 0.0281701 |
| AT5G08720 | CONTAINS InterPro DOMAIN/s: Streptomyces cyclase/dehydrase (InterPro:IPR005031) | 0.84022 | 2.59755 | 1.62831 | 0.01205 | 0.0451624 |
| AT3G13560 | O-Glycosyl hydrolases family 17 protein | 2.27834 | 7.0502 | 1.62968 | 0.0043 | 0.0211046 |
| AT1G59600 | ZCW7, ZCW7 | 3.99703 | 12.3771 | 1.63067 | 0.00535 | 0.025018 |
| AT4G28940 | Phosphorylase superfamily protein | 32.3272 | 100.127 | 1.63102 | 0.0002 | 0.00214445 |
| AT3G13230 | RNA-binding KH domain-containing protein | 9.56661 | 29.6628 | 1.63257 | 0.00195 | 0.0117451 |
| AT4G37410 | CYP81F4, cytochrome P450, family 81, subfamily F, polypeptide 4 | 10.9341 | 33.907 | 1.63275 | 0.0015 | 0.00967937 |
| AT1G06950 | ATTIC110, TIC110, translocon at the inner envelope membrane of chloroplasts 110 | 3.96556 | 12.3033 | 1.63345 | 0.0009 | 0.0065867 |
| AT5G21970 | Ubiquitin carboxyl-terminal hydrolase family protein | 1.586 | 4.92142 | 1.63368 | 0.01095 | 0.0420749 |
| AT5G11540 | D-arabinono-1,4-lactone oxidase family protein | 1.19958 | 3.72352 | 1.63414 | 0.0118 | 0.0445713 |
| AT1G03070 | Bax inhibitor-1 family protein | 26.9696 | 83.7187 | 1.63422 | 0.00285 | 0.0154761 |
| AT4G14680 | APS3, Pseudouridine synthase/archaeosine transglycosylase-like family protein | 11.781 | 36.5862 | 1.63484 | 0.0007 | 0.00547072 |
| AT5G66540 | function unknown | 6.59445 | 20.4849 | 1.63524 | 0.00065 | 0.00516972 |
| AT5G49640 | unknown protein | 6.21544 | 19.3124 | 1.6356 | 0.01145 | 0.0435148 |
| AT3G12270 | ATPRMT3, PRMT3, protein arginine methyltransferase 3 | 4.24457 | 13.1919 | 1.63596 | 0.0017 | 0.0106071 |
| AT3G17590 | BSH, CHE1, transcription regulatory protein SNF5, putative (BSH) | 6.21493 | 19.3568 | 1.63903 | 0.0097 | 0.0385431 |
| AT2G33430 | DAL, DAL1, differentiation and greening-like 1 | 3.36882 | 10.4993 | 1.63997 | 0.0084 | 0.0346931 |
| AT2G44180 | MAP2A, methionine aminopeptidase 2A | 11.7417 | 36.6173 | 1.64089 | 0.00045 | 0.003919 |
| AT5G26110 | Protein kinase superfamily protein | 3.59187 | 11.2078 | 1.6417 | 0.00625 | 0.0280351 |
| AT2G41560 | ACA4, autoinhibited Ca(2+)-ATPase, isoform 4 | 3.14885 | 9.82629 | 1.64182 | 0.0011 | 0.00759303 |
| AT5G07340 | Calreticulin family protein | 6.88872 | 21.5 | 1.64203 | 0.0022 | 0.0128446 |
| AT3G56430 | unknown protein | 2.40644 | 7.51147 | 1.64219 | 0.00425 | 0.0209475 |
| AT3G54220 | SCR, SGR1, GRAS family transcription factor | 2.62111 | 8.19536 | 1.64463 | 0.0022 | 0.0128446 |
| AT3G10160 | ATDFC, DFC, DHFS-FPGS homolog C | 1.6278 | 5.09021 | 1.6448 | 0.0042 | 0.0207631 |
| AT3G52050 | 5'-3' exonuclease family protein | 1.9967 | 6.24604 | 1.64533 | 0.0106 | 0.0411532 |
| AT3G54740 | Protein of unknown function, DUF593 | 12.1417 | 37.9831 | 1.64539 | 0.0006 | 0.00487984 |
| AT5G10910 | mraW methylase family protein | 1.60996 | 5.0392 | 1.64617 | 0.0097 | 0.0385431 |
| AT2G47115 | unknown protein | 28.7087 | 89.9077 | 1.64696 | 0.0002 | 0.00214445 |
| AT4G34960 | Cyclophilin-like peptidyl-prolyl cis-trans isomerase family protein | 4.6733 | 14.6382 | 1.64723 | 0.0027 | 0.0149318 |
| AT1G65470 | FAS1, NFB2, chromatin assembly factor-1 (FASCIATA1) (FAS1) | 1.19932 | 3.75951 | 1.64833 | 0.0068 | 0.029741 |
| AT5G14430 | S-adenosyl-L-methionine-dependent methyltransferases superfamily protein | 6.55618 | 20.552 | 1.64835 | 0.00515 | 0.024295 |
| AT1G15520 | ABCG40, ATABCG40, ATPDR12, PDR12, pleiotropic drug resistance 12 | 17.9469 | 56.2606 | 1.64839 | 0.0001 | 0.00123775 |
| AT5G04940 | SUVH1, SU(VAR)3-9 homolog 1 | 2.36124 | 7.41192 | 1.6503 | 0.0025 | 0.0140894 |
| AT3G17820 | ATGSKB6, GLN1.3, GLN1;3, glutamine synthetase 1.3 | 47.6736 | 149.648 | 1.65031 | 0.0003 | 0.00292382 |
| AT4G14360 | S-adenosyl-L-methionine-dependent methyltransferases superfamily protein | 9.23933 | 29.011 | 1.65074 | 0.0008 | 0.00603091 |
| AT2G25350 | Phox (PX) domain-containing protein | 1.35263 | 4.24968 | 1.65159 | 0.01065 | 0.0412906 |
| AT5G26680 | 5'-3' exonuclease family protein | 3.26875 | 10.2718 | 1.65188 | 0.0063 | 0.0281701 |
| AT3G17970 | atToc64-III, TOC64-III, translocon at the outer membrane of chloroplasts 64-III | 2.60307 | 8.18159 | 1.65217 | 0.0048 | 0.0229834 |
| AT1G02690 | IMPA-6, importin alpha isoform 6 | 5.40101 | 16.9841 | 1.65289 | 0.0006 | 0.00487984 |
| AT5G39050 | HXXXD-type acyl-transferase family protein | 15.4103 | 48.4594 | 1.65289 | 0.0007 | 0.00547072 |
| AT1G22880 | ATCEL5, ATGH9B4, CEL5, cellulase 5 | 1.54823 | 4.87196 | 1.65388 | 0.00865 | 0.0354741 |
| AT5G06550 | CONTAINS InterPro DOMAIN/s: Transcription factor jumonji/aspartyl beta-hydroxylase (InterPro:IPR003347) | 2.30411 | 7.25239 | 1.65425 | 0.00405 | 0.0202184 |
| AT1G67690 | Zincin-like metalloproteases family protein | 1.45198 | 4.5706 | 1.65436 | 0.003 | 0.0161227 |
| AT3G07110 | Ribosomal protein L13 family protein | 40.8247 | 128.53 | 1.65459 | 0.00045 | 0.003919 |
| AT1G20380 | Prolyl oligopeptidase family protein | 1.83098 | 5.76749 | 1.65533 | 0.00435 | 0.0212815 |
| AT3G48730 | GSA2, glutamate-1-semialdehyde 2,1-aminomutase 2 | 2.55708 | 8.05858 | 1.65603 | 0.0055 | 0.0254967 |
| AT4G09320 | NDPK1, Nucleoside diphosphate kinase family protein | 81.6957 | 257.591 | 1.65675 | 0.00035 | 0.00327416 |
| AT4G39280 | phenylalanyl-tRNA synthetase, putative / phenylalanine--tRNA ligase, putative | 6.69381 | 21.1227 | 1.65789 | 0.001 | 0.00711095 |
| AT3G01370 | ATCFM2, CFM2, CRM family member 2 | 2.45179 | 7.73949 | 1.6584 | 0.002 | 0.0119879 |
| AT1G74650 | ATMYB31, ATY13, MYB31, myb domain protein 31 | 0.944865 | 2.9837 | 1.65892 | 0.01115 | 0.0426612 |
| AT3G21465 | unknown protein | 2.75423 | 8.70053 | 1.65946 | 0.0047 | 0.0226084 |
| AT5G60530 | late embryogenesis abundant protein-related / LEA protein-related | 97.8294 | 309.051 | 1.6595 | 0.0007 | 0.00547072 |
| AT3G17330 | ECT6, evolutionarily conserved C-terminal region 6 | 4.59519 | 14.5271 | 1.66055 | 0.0013 | 0.00867426 |
| AT3G12965 | other RNA | 37.2624 | 117.817 | 1.66075 | 0.01375 | 0.0496726 |
| AT3G61240 | DEA(D/H)-box RNA helicase family protein | 9.63024 | 30.4491 | 1.66075 | 0.0002 | 0.00214445 |
| AT2G44810 | DAD1, alpha/beta-Hydrolases superfamily protein | 1.62437 | 5.14068 | 1.66208 | 0.00785 | 0.0330699 |
| AT3G47370 | Ribosomal protein S10p/S20e family protein | 42.7813 | 135.434 | 1.66254 | 0.00065 | 0.00516972 |
| AT1G54630 | ACP3, acyl carrier protein 3 | 32.1228 | 101.714 | 1.66285 | 0.0006 | 0.00487984 |
| AT3G11630 | Thioredoxin superfamily protein | 16.426 | 52.0349 | 1.6635 | 0.00095 | 0.00685384 |
| AT3G10440 | Shugoshin C terminus | 1.26407 | 4.00721 | 1.66452 | 0.011 | 0.0422342 |
| AT5G02450 | Ribosomal protein L36e family protein | 109.926 | 348.769 | 1.66574 | 0.0002 | 0.00214445 |
| AT5G46740 | UBP21, ubiquitin-specific protease 21 | 1.14584 | 3.63574 | 1.66585 | 0.0093 | 0.0372979 |
| AT5G60590 | DHBP synthase RibB-like alpha/beta domain | 1.50485 | 4.78112 | 1.66773 | 0.0124 | 0.046125 |
| AT5G07090 | Ribosomal protein S4 (RPS4A) family protein | 87.3458 | 278.028 | 1.67042 | 0.00015 | 0.00170235 |
| AT4G00820 | iqd17, IQ-domain 17 | 5.09225 | 16.2115 | 1.67065 | 0.0011 | 0.00759303 |
| AT4G27490 | 3'-5'-exoribonuclease family protein | 3.94414 | 12.5592 | 1.67096 | 0.00315 | 0.0166977 |
| AT1G43170 | ARP1, emb2207, RP1, RPL3A, ribosomal protein 1 | 133.498 | 425.174 | 1.67123 | 0.00025 | 0.00253767 |
| AT1G22660 | Polynucleotide adenylyltransferase family protein | 1.47929 | 4.71359 | 1.67192 | 0.00405 | 0.0202184 |
| AT2G45860 | unknown protein | 13.5456 | 43.1658 | 1.67206 | 0.01325 | 0.0484049 |
| AT2G33370 | Ribosomal protein L14p/L23e family protein | 54.3103 | 173.074 | 1.67209 | 0.0005 | 0.00424432 |
| AT5G57940 | ATCNGC5, CNGC5, cyclic nucleotide gated channel 5 | 2.07246 | 6.60612 | 1.67246 | 0.0019 | 0.0115387 |
| AT4G13050 | Acyl-ACP thioesterase | 6.43423 | 20.5107 | 1.67254 | 0.0007 | 0.00547072 |
| AT2G28000 | CH-CPN60A, CPN60A, SLP, chaperonin-60alpha | 16.4675 | 52.5604 | 1.67436 | 0.00045 | 0.003919 |
| AT4G23180 | CRK10, RLK4, cysteine-rich RLK (RECEPTOR-like protein kinase) 10 | 1.6768 | 5.35241 | 1.67448 | 0.00325 | 0.0171315 |
| AT2G37270 | ATRPS5B, RPS5B, ribosomal protein 5B | 50.8714 | 162.403 | 1.67465 | 0.00005 | 0.00070028 |
| AT5G41480 | ATDFA, DFA, EMB9, GLA1, Folylpolyglutamate synthetase family protein | 1.76668 | 5.64125 | 1.67497 | 0.0031 | 0.0165078 |
| AT5G65000 | Nucleotide-sugar transporter family protein | 2.74508 | 8.76715 | 1.67526 | 0.0032 | 0.0169175 |
| AT3G22790 | Kinase interacting (KIP1-like) family protein | 2.80941 | 8.97512 | 1.67566 | 0.00105 | 0.00735817 |
| AT1G77590 | LACS9, long chain acyl-CoA synthetase 9 | 4.62 | 14.7636 | 1.67608 | 0.00075 | 0.00575475 |
| AT3G18030 | ATHAL3, ATHAL3A, HAL3, HAL3A, HAL3-like protein A | 2.45803 | 7.85486 | 1.67608 | 0.0088 | 0.035933 |
| AT4G27090 | Ribosomal protein L14 | 244.956 | 783.115 | 1.6767 | 0.00025 | 0.00253767 |
| AT4G27900 | CCT motif family protein | 1.72286 | 5.5093 | 1.67707 | 0.01365 | 0.0494285 |
| AT3G54560 | HTA11, histone H2A 11 | 9.85564 | 31.5296 | 1.67768 | 0.0034 | 0.01771 |
| AT1G18850 | unknown protein | 3.51707 | 11.2544 | 1.67804 | 0.0041 | 0.0203755 |
| AT5G08590 | ASK2, ASK2, SNRK2-1, SNRK2.1, SRK2G, SNF1-related protein kinase 2.1 | 27.8075 | 89.0262 | 1.67875 | 0.00035 | 0.00327416 |
| AT2G33170 | Leucine-rich repeat receptor-like protein kinase family protein | 1.79914 | 5.76026 | 1.67882 | 0.00335 | 0.0175049 |
| AT1G48920 | ATNUC-L1, NUC-L1, PARL1, nucleolin like 1 | 51.5798 | 165.15 | 1.6789 | 0.00005 | 0.00070028 |
| AT4G26500 | ATSUFE, CPSUFE, EMB1374, SUFE1, chloroplast sulfur E | 2.05843 | 6.59116 | 1.67899 | 0.00815 | 0.0339652 |
| AT5G06680 | ATGCP3, ATSPC98, GCP3, SPC98, spindle pole body component 98 | 0.767696 | 2.45827 | 1.67904 | 0.00925 | 0.0371953 |
| AT1G10180 | BEST Arabidopsis thaliana protein match is: exocyst complex component 84B (TAIR:AT5G49830.1) | 2.61857 | 8.38548 | 1.67911 | 0.00155 | 0.009918 |
| AT1G77610 | EamA-like transporter family protein | 12.7652 | 40.9093 | 1.68022 | 0.0041 | 0.0203755 |
| AT5G64670 | Ribosomal protein L18e/L15 superfamily protein | 7.21182 | 23.1142 | 1.68034 | 0.0016 | 0.0101494 |
| AT5G14880 | Potassium transporter family protein | 0.698247 | 2.24113 | 1.68242 | 0.0081 | 0.0338066 |
| AT4G39900 | unknown protein | 4.9952 | 16.035 | 1.68261 | 0.0035 | 0.0180741 |
| AT2G37190 | Ribosomal protein L11 family protein | 78.9364 | 253.414 | 1.68273 | 0.0006 | 0.00487984 |
| AT2G29190 | APUM2, PUM2, pumilio 2 | 1.61627 | 5.19713 | 1.68505 | 0.00215 | 0.0126571 |
| AT4G19610 | nucleotide binding;nucleic acid binding;RNA binding | 2.71594 | 8.73389 | 1.68518 | 0.00175 | 0.0108609 |
| AT4G16650 | O-fucosyltransferase family protein | 4.79944 | 15.4362 | 1.68538 | 0.0008 | 0.00603091 |
| AT4G31200 | SWAP (Suppressor-of-White-APricot)/surp RNA-binding domain-containing protein | 1.422 | 4.57513 | 1.68589 | 0.0043 | 0.0211046 |
| AT5G23550 | Got1/Sft2-like vescicle transport protein family | 3.86348 | 12.4326 | 1.68616 | 0.00295 | 0.0159014 |
| AT3G53460 | CP29, chloroplast RNA-binding protein 29 | 5.3491 | 17.2191 | 1.68664 | 0.0018 | 0.0110912 |
| AT4G29430 | rps15ae, ribosomal protein S15A E | 6.03301 | 19.4229 | 1.68681 | 0.01065 | 0.0412906 |
| AT5G26180 | S-adenosyl-L-methionine-dependent methyltransferases superfamily protein | 1.38252 | 4.45384 | 1.68775 | 0.01025 | 0.0401565 |
| AT3G01450 | ARM repeat superfamily protein | 1.86144 | 5.99784 | 1.68803 | 0.0065 | 0.0287784 |
| AT1G76350 | Plant regulator RWP-RK family protein | 3.54859 | 11.4362 | 1.68829 | 0.007 | 0.0303609 |
| AT5G14610 | DEAD box RNA helicase family protein | 1.10095 | 3.54812 | 1.6883 | 0.0039 | 0.0196279 |
| AT5G52230 | MBD13, methyl-CPG-binding domain protein 13 | 0.68343 | 2.20615 | 1.69067 | 0.0099 | 0.0391493 |
| AT3G16700 | Fumarylacetoacetate (FAA) hydrolase family | 3.49106 | 11.2753 | 1.69143 | 0.0112 | 0.0427699 |
| AT1G76550 | Phosphofructokinase family protein | 10.7005 | 34.5947 | 1.69288 | 0.00045 | 0.003919 |
| AT4G13200 | unknown protein | 4.07208 | 13.1681 | 1.69321 | 0.0064 | 0.0284694 |
| AT5G40530 | S-adenosyl-L-methionine-dependent methyltransferases superfamily protein | 5.56029 | 17.9816 | 1.69329 | 0.00265 | 0.0147335 |
| AT5G10110 | unknown protein | 3.22467 | 10.4325 | 1.69386 | 0.00345 | 0.0179093 |
| AT5G08650 | Small GTP-binding protein | 0.95065 | 3.07622 | 1.69417 | 0.00795 | 0.0333705 |
| AT4G00490 | BAM2, BMY9, beta-amylase 2 | 5.539 | 17.9456 | 1.69593 | 0.00275 | 0.0151029 |
| AT3G60530 | GATA4, GATA transcription factor 4 | 6.3891 | 20.7078 | 1.69649 | 0.00645 | 0.0286275 |
| AT4G30920 | Cytosol aminopeptidase family protein | 2.94062 | 9.53172 | 1.69661 | 0.00135 | 0.0089118 |
| AT5G42320 | Zn-dependent exopeptidases superfamily protein | 4.58733 | 14.8739 | 1.69706 | 0.0035 | 0.0180741 |
| AT4G20980 | Eukaryotic translation initiation factor 3 subunit 7 (eIF-3) | 8.74119 | 28.3496 | 1.69743 | 0.00055 | 0.00457655 |
| AT5G37530 | NAD(P)-binding Rossmann-fold superfamily protein | 0.854398 | 2.77189 | 1.69789 | 0.0105 | 0.0408771 |
| AT4G28320 | Glycosyl hydrolase superfamily protein | 2.40516 | 7.80795 | 1.69881 | 0.00485 | 0.0231612 |
| AT5G39960 | GTP binding;GTP binding | 1.53752 | 4.99345 | 1.69943 | 0.00335 | 0.0175049 |
| AT3G43250 | Family of unknown function (DUF572) | 14.0589 | 45.6781 | 1.70002 | 0.00175 | 0.0108609 |
| AT3G16180 | Major facilitator superfamily protein | 5.70065 | 18.5303 | 1.70069 | 0.0007 | 0.00547072 |
| AT1G23890 | NHL domain-containing protein | 1.97214 | 6.41318 | 1.70128 | 0.00585 | 0.0267005 |
| AT4G25240 | SKS1, SKU5 similar 1 | 0.671093 | 2.18285 | 1.70163 | 0.01085 | 0.0417717 |
| AT1G72040 | P-loop containing nucleoside triphosphate hydrolases superfamily protein | 4.0048 | 13.0271 | 1.70172 | 0.0012 | 0.00815259 |
| AT2G23300 | Leucine-rich repeat protein kinase family protein | 1.4247 | 4.63527 | 1.702 | 0.0028 | 0.0152969 |
| AT1G80940 | unknown protein | 4.16084 | 13.547 | 1.70303 | 0.00335 | 0.0175049 |
| AT2G43460 | Ribosomal L38e protein family | 144.367 | 470.249 | 1.70368 | 0.00015 | 0.00170235 |
| AT3G57220 | Glycosyl transferase family 4 protein | 3.91399 | 12.7574 | 1.70463 | 0.00705 | 0.0305176 |
| AT1G27070 | 5'-AMP-activated protein kinase-related | 2.32387 | 7.57627 | 1.70496 | 0.0033 | 0.0173075 |
| AT2G17270 | PHT3;3, phosphate transporter 3;3 | 1.13599 | 3.70426 | 1.70524 | 0.00805 | 0.0336689 |
| AT3G22060 | Receptor-like protein kinase-related family protein | 12.9982 | 42.3939 | 1.70554 | 0.0015 | 0.00967937 |
| AT5G45920 | SGNH hydrolase-type esterase superfamily protein | 2.81732 | 9.18937 | 1.70564 | 0.0088 | 0.035933 |
| AT2G24990 | Serine/threonine-protein kinase Rio1 | 1.33089 | 4.34239 | 1.7061 | 0.00885 | 0.0361074 |
| AT1G17180 | ATGSTU25, GSTU25, glutathione S-transferase TAU 25 | 424.129 | 1383.85 | 1.70611 | 0.00075 | 0.00575475 |
| AT3G49670 | BAM2, Leucine-rich receptor-like protein kinase family protein | 1.28935 | 4.20977 | 1.70709 | 0.0033 | 0.0173075 |
| AT4G00620 | Amino acid dehydrogenase family protein | 1.81578 | 5.93219 | 1.70797 | 0.0101 | 0.039726 |
| AT2G44020 | Mitochondrial transcription termination factor family protein | 1.72616 | 5.6398 | 1.70808 | 0.0034 | 0.01771 |
| AT1G36310 | S-adenosyl-L-methionine-dependent methyltransferases superfamily protein | 5.11782 | 16.7218 | 1.70813 | 0.0016 | 0.0101494 |
| AT5G61060 | ATHDA5, HDA05, HDA5, histone deacetylase 5 | 1.93435 | 6.32347 | 1.70887 | 0.00145 | 0.00940576 |
[truncated: 207,315 more chars]
