# Supplementary material for: Re-analysis of RNA-seq transcriptome data reveals new aspects of gene activity in Arabidopsis root hairs
Source: Front Plant Sci. 2015 Jun 8;6:421. doi: 10.3389/fpls.2015.00421 (PMC4458573; doi:10.3389/fpls.2015.00421)
Supplement: Supplementary file 11 [file Table6.DOC]

**Table S6** List of the 2813 up-regulated genes in root hairs (RH) compared to non-root hair tissues (NRH).

| AGI | Annotation | | RH(RPKM) | NRH(RPKM) | | Fold_change(log2) |
| --- | --- | --- | --- | --- | --- | --- |
| AT2G24980 | | Proline-rich extensin-like family protein | 282.55 | 0.145177 | | -10.927 |
| AT5G06630 | | proline-rich extensin-like family protein | 350.094 | 0.263724 | | -10.375 |
| AT1G12560 | | ATEXP7, expansin A7 | 390.921 | 0.365196 | | -10.064 |
| AT4G40090 | | AGP3, arabinogalactan protein 3 | 1002.28 | 1.07251 | | -9.8681 |
| AT3G62680 | | ATPRP3, PRP3, proline-rich protein 3 | 419.939 | 0.453138 | | -9.856 |
| AT5G04960 | | Plant invertase/pectin methylesterase inhibitor | 179.825 | 0.201765 | | -9.7997 |
| AT3G09925 | | Pollen Ole e 1 allergen and extensin family protein | 685.787 | 0.79408 | | -9.7543 |
| AT4G25820 | | ATXTH14 | 758.459 | 0.933845 | | -9.6657 |
| AT5G06640 | | Proline-rich extensin-like family protein | 300.574 | 0.375472 | | -9.6448 |
| AT3G54590 | | ATHRGP1, HRGP1, hydroxyproline-rich glycoprotein | 882.994 | 1.10396 | | -9.6436 |
| AT5G67400 | | RHS19, root hair specific 19 | 456.332 | 0.570824 | | -9.6428 |
| AT4G13390 | | Proline-rich extensin-like family protein | 346.903 | 0.436798 | | -9.6334 |
| AT4G02270 | | RHS13, root hair specific 13 | 828.721 | 1.12794 | | -9.5211 |
| AT4G00680 | | ADF8, actin depolymerizing factor 8 | 479.401 | 0.676228 | | -9.4695 |
| AT1G62980 | | ATEXP18, expansin A18 | 208.435 | 0.345746 | | -9.2357 |
| AT5G57530 | | AtXTH12 | 125.269 | 0.209592 | | -9.2232 |
| AT5G35190 | | proline-rich extensin-like family protein | 467.479 | 0.808864 | | -9.1748 |
| AT2G29620 | | unknown protein | 35.8692 | 0.067347 | | -9.0569 |
| AT1G30870 | | Peroxidase superfamily protein | 413.75 | 0.811178 | | -8.9945 |
| AT1G12040 | | LRX1, leucine-rich repeat/extensin 1 | 184.771 | 0.367391 | | -8.9742 |
| AT5G05500 | | Pollen Ole e 1 allergen and extensin family protein | 467.413 | 0.954079 | | -8.9364 |
| AT5G11440 | | CID5, IPD1, CTC-interacting domain 5 | 141.179 | 0.300056 | | -8.8781 |
| AT1G48930 | | AtGH9C1, GH9C1, glycosyl hydrolase 9C1 | 212.097 | 0.450867 | | -8.8778 |
| AT1G54970 | | ATPRP1, PRP1, RHS7, proline-rich protein 1 | 197.916 | 0.420925 | | -8.8771 |
| AT2G41970 | | Protein kinase superfamily protein | 232.38 | 0.551853 | | -8.718 |
| AT2G47540 | | Pollen Ole e 1 allergen and extensin family protein | 213.419 | 0.513428 | | -8.6993 |
| AT5G22410 | | RHS18, root hair specific 18 | 106.322 | 0.292415 | | -8.5062 |
| AT2G47360 | | unknown protein | 62.0735 | 0.184463 | | -8.3945 |
| AT2G30670 | | NAD(P)-binding Rossmann-fold superfamily protein | 112.11 | 0.339629 | | -8.3667 |
| AT3G10710 | | RHS12, root hair specific 12 | 57.5381 | 0.189001 | | -8.25 |
| AT2G45890 | | ATROPGEF4, RHS11 | 101.066 | 0.368687 | | -8.0987 |
| AT5G49270 | | COBL9 | 80.4778 | 0.313345 | | -8.0047 |
| AT5G22555 | | unknown protein | 196.529 | 0.860094 | | -7.836 |
| AT5G40860 | | unknown protein | 136.684 | 0.628081 | | -7.7657 |
| AT3G54580 | | Proline-rich extensin-like family protein | 1903.3 | 8.85952 | | -7.7471 |
| AT4G09990 | | Protein of unknown function (DUF579) | 189.187 | 0.903108 | | -7.7107 |
| AT1G08090 | | ACH1, ATNRT2.1, nitrate transporter 2:1 | 52.9332 | 0.266549 | | -7.6336 |
| AT3G60330 | | AHA7, HA7, H(+)-ATPase 7 | 310.855 | 1.56648 | | -7.6326 |
| AT2G33460 | | RIC1, ROP-interactive CRIB motif-containing protein 1 | 58.361 | 0.294145 | | -7.6323 |
| AT4G26010 | | Peroxidase superfamily protein | 461.842 | 2.38604 | | -7.5966 |
| AT3G07070 | | Protein kinase superfamily protein | 39.4694 | 0.207821 | | -7.5693 |
| AT2G46860 | | AtPPa3, PPa3, pyrophosphorylase 3 | 111.429 | 0.593574 | | -7.5525 |
| AT3G47040 | | Glycosyl hydrolase family protein | 30.0456 | 0.168941 | | -7.4745 |
| AT4G01110 | | unknown protein | 62.5961 | 0.387963 | | -7.334 |
| AT1G08990 | | PGSIP5, plant glycogenin-like starch initiation protein 5 | 62.0058 | 0.388489 | | -7.3184 |
| AT5G51270 | | U-box domain-containing protein kinase family protein | 31.5351 | 0.198535 | | -7.3114 |
| AT5G58010 | | LRL3, LJRHL1-like 3 | 273.512 | 1.80494 | | -7.2435 |
| AT3G49960 | | Peroxidase superfamily protein | 159.373 | 1.06339 | | -7.2276 |
| AT4G34580 | | COW1 | 218.104 | 1.47609 | | -7.2071 |
| AT3G51350 | | Eukaryotic aspartyl protease family protein | 38.8901 | 0.274518 | | -7.1464 |
| AT4G38390 | | RHS17, root hair specific 17 | 36.6331 | 0.260206 | | -7.1374 |
| AT1G70460 | | RHS10, root hair specific 10 | 88.1011 | 0.641957 | | -7.1005 |
| AT5G62310 | | IRE | 64.0072 | 0.516494 | | -6.9533 |
| AT4G29180 | | RHS16, root hair specific 16 | 55.3506 | 0.448309 | | -6.948 |
| AT5G17820 | | Peroxidase superfamily protein | 1305.01 | 10.998 | | -6.8907 |
| AT1G27740 | | RSL4, root hair defective 6-like 4 | 254.49 | 2.16234 | | -6.8789 |
| AT1G09170 | | P-loop nucleoside triphosphate hydrolases superfamily protein with CH (Calponin Homology) domain | 24.029 | 0.205787 | | -6.8675 |
| AT4G30320 | | CAP (Cysteine-rich secretory proteins, Antigen 5, and Pathogenesis-related 1 protein) superfamily protein | 89.776 | 0.843779 | | -6.7333 |
| AT5G49870 | | Mannose-binding lectin superfamily protein | 31.8792 | 0.309214 | | -6.6879 |
| AT1G51860 | | Leucine-rich repeat protein kinase family protein | 8.1714 | 0.0812391 | | -6.6523 |
| AT5G65160 | | tetratricopeptide repeat (TPR)-containing protein | 74.2567 | 0.744421 | | -6.6403 |
| AT4G02830 | | unknown protein | 34.9084 | 0.352575 | | -6.6295 |
| AT5G01280 | | BEST Arabidopsis thaliana protein match is: proline-rich family protein (TAIR:AT3G09000.1) | 52.2871 | 0.534523 | | -6.6121 |
| AT5G61550 | | U-box domain-containing protein kinase family protein | 35.9846 | 0.372456 | | -6.5942 |
| AT4G25220 | | RHS15, root hair specific 15 | 24.1657 | 0.254417 | | -6.5696 |
| AT2G17890 | | CPK16, calcium-dependent protein kinase 16 | 8.41945 | 0.0946467 | | -6.475 |
| AT2G45750 | | S-adenosyl-L-methionine-dependent methyltransferases superfamily protein | 142.026 | 1.61829 | | -6.4555 |
| AT4G25090 | | Riboflavin synthase-like superfamily protein | 62.3366 | 0.714573 | | -6.4469 |
| AT1G07795 | | unknown protein | 47.4163 | 0.554259 | | -6.4187 |
| AT4G34380 | | Transducin/WD40 repeat-like superfamily protein | 8.24351 | 0.0984524 | | -6.3877 |
| AT5G25810 | | tny, Integrase-type DNA-binding superfamily protein | 21.4022 | 0.257057 | | -6.3795 |
| AT2G37820 | | Cysteine/Histidine-rich C1 domain family protein | 17.8879 | 0.221005 | | -6.3388 |
| AT1G34760 | | GF14 OMICRON, GRF11, RHS5 | 50.7197 | 0.636707 | | -6.3158 |
| AT4G25940 | | ENTH/ANTH/VHS superfamily protein | 19.5987 | 0.246851 | | -6.311 |
| AT1G12550 | | D-isomer specific 2-hydroxyacid dehydrogenase family protein | 81.9584 | 1.03544 | | -6.3066 |
| AT5G21080 | | Uncharacterized protein | 30.2492 | 0.410728 | | -6.2026 |
| AT5G65090 | | BST1, DER4, MRH3, DNAse I-like superfamily protein | 41.273 | 0.593343 | | -6.1202 |
| AT4G25110 | | AtMC2, MC2, metacaspase 2 | 25.4734 | 0.366441 | | -6.1193 |
| AT3G18450 | | PLAC8 family protein | 26.2681 | 0.378257 | | -6.1178 |
| AT1G01750 | | ADF11, actin depolymerizing factor 11 | 723.49 | 10.7564 | | -6.0717 |
| AT3G21340 | | Leucine-rich repeat protein kinase family protein | 83.7404 | 1.26439 | | -6.0494 |
| AT2G30660 | | ATP-dependent caseinolytic (Clp) protease/crotonase family protein | 24.6728 | 0.377473 | | -6.0304 |
| AT1G10385 | | Vps51/Vps67 family (components of vesicular transport) protein | 8.71123 | 0.133365 | | -6.0294 |
| AT4G14780 | | Protein kinase superfamily protein | 11.7601 | 0.181057 | | -6.0213 |
| AT3G54870 | | ARK1, CAE1, MRH2, Armadillo/beta-catenin repeat family protein / kinesin motor family protein | 48.081 | 0.745398 | | -6.0113 |
| AT3G47050 | | Glycosyl hydrolase family protein | 10.9459 | 0.183671 | | -5.8971 |
| AT2G38500 | | 2-oxoglutarate (2OG) and Fe(II)-dependent oxygenase superfamily protein | 91.0314 | 1.52791 | | -5.8967 |
| AT3G07900 | | O-fucosyltransferase family protein | 32.1397 | 0.546227 | | -5.8787 |
| AT5G15600 | | SP1L4, SPIRAL1-like4 | 148.905 | 2.58613 | | -5.8475 |
| AT1G53680 | | ATGSTU28, GSTU28, glutathione S-transferase TAU 28 | 304.142 | 5.34615 | | -5.8301 |
| AT5G42785 | | unknown protein | 80.0565 | 1.42305 | | -5.814 |
| AT2G20030 | | RING/U-box superfamily protein | 8.41905 | 0.150287 | | -5.8079 |
| AT2G03360 | | Glycosyltransferase family 61 protein | 6.24031 | 0.112152 | | -5.7981 |
| AT5G12050 | | unknown protein | 184.136 | 3.34175 | | -5.784 |
| AT4G22217 | | Arabidopsis defensin-like protein | 349.124 | 6.59131 | | -5.727 |
| AT4G21200 | | ATGA2OX8, GA2OX8, gibberellin 2-oxidase 8 | 11.7754 | 0.22266 | | -5.7248 |
| AT4G08450 | | Disease resistance protein (TIR-NBS-LRR class) family | 7.34767 | 0.139961 | | -5.7142 |
| AT1G18420 | | Aluminium activated malate transporter family protein | 55.8597 | 1.08416 | | -5.6872 |
| AT1G04280 | | P-loop containing nucleoside triphosphate hydrolases superfamily protein | 192.645 | 3.74287 | | -5.6857 |
| AT2G17590 | | Cysteine/Histidine-rich C1 domain family protein | 4.31448 | 0.0844839 | | -5.6744 |
| AT1G29230 | | ATCIPK18, ATWL1, CIPK18, SnRK3.20, WL1, CBL-interacting protein kinase 18 | 21.9501 | 0.435261 | | -5.6562 |
| AT5G14330 | | unknown protein | 469.906 | 9.38705 | | -5.6456 |
| AT1G73860 | | P-loop containing nucleoside triphosphate hydrolases superfamily protein | 70.0785 | 1.40914 | | -5.6361 |
| AT4G30670 | | Putative membrane lipoprotein | 235.479 | 4.89032 | | -5.5895 |
| AT2G05160 | | CCCH-type zinc fingerfamily protein with RNA-binding domain | 29.1287 | 0.614505 | | -5.5669 |
| AT4G25160 | | U-box domain-containing protein kinase family protein | 19.6735 | 0.418077 | | -5.5563 |
| AT1G70170 | | MMP, matrix metalloproteinase | 44.6145 | 0.961701 | | -5.5358 |
| AT1G70720 | | Plant invertase/pectin methylesterase inhibitor superfamily protein | 15.9509 | 0.345402 | | -5.5292 |
| AT4G37390 | | AUR3, BRU6, GH3-2, GH3.2, YDK1, Auxin-responsive GH3 family protein | 172.489 | 3.74332 | | -5.526 |
| AT4G29800 | | PLA IVD, PLP8, PATATIN-like protein 8 | 28.7895 | 0.638612 | | -5.4945 |
| AT4G18640 | | MRH1, Leucine-rich repeat protein kinase family protein | 85.9666 | 1.95314 | | -5.4599 |
| AT1G55290 | | 2-oxoglutarate (2OG) and Fe(II)-dependent oxygenase superfamily protein | 21.3654 | 0.487674 | | -5.4532 |
| AT3G50130 | | Plant protein of unknown function (DUF247) | 10.1628 | 0.237054 | | -5.4219 |
| AT1G16440 | | RSH3, root hair specific 3 | 22.044 | 0.524572 | | -5.3931 |
| AT5G07080 | | HXXXD-type acyl-transferase family protein | 106.986 | 2.56867 | | -5.3803 |
| AT2G37670 | | Transducin/WD40 repeat-like superfamily protein | 40.673 | 0.994725 | | -5.3536 |
| AT3G51570 | | Disease resistance protein (TIR-NBS-LRR class) family | 1.95741 | 0.0481138 | | -5.3464 |
| AT5G65100 | | Ethylene insensitive 3 family protein | 26.583 | 0.65571 | | -5.3413 |
| AT4G13310 | | CYP71A20, cytochrome P450, family 71, subfamily A, polypeptide 20 | 6.84932 | 0.169303 | | -5.3383 |
| AT2G34910 | | BEST Arabidopsis thaliana protein match is: root hair specific 4 (TAIR:AT1G30850.1) | 587.714 | 14.9774 | | -5.2943 |
| AT5G21120 | | EIL2, ETHYLENE-INSENSITIVE3-like 2 | 4.37142 | 0.115572 | | -5.2413 |
| AT2G02250 | | AtPP2-B2, PP2-B2, phloem protein 2-B2 | 24.4822 | 0.648693 | | -5.2381 |
| AT5G46360 | | ATKCO3, KCO3, Ca2+ activated outward rectifying K+ channel 3 | 7.82314 | 0.209611 | | -5.222 |
| AT1G48640 | | Transmembrane amino acid transporter family protein | 42.8959 | 1.15054 | | -5.2205 |
| AT4G21745 | | PAK-box/P21-Rho-binding family protein | 7.52484 | 0.204376 | | -5.2024 |
| AT5G61350 | | Protein kinase superfamily protein | 30.4309 | 0.832928 | | -5.1912 |
| AT5G17390 | | Adenine nucleotide alpha hydrolases-like superfamily protein | 59.2781 | 1.62984 | | -5.1847 |
| AT5G07780 | | Actin-binding FH2 (formin homology 2) family protein | 5.2055 | 0.144088 | | -5.175 |
| AT5G45840 | | Leucine-rich repeat protein kinase family protein | 30.2517 | 0.839383 | | -5.1716 |
| AT5G23270 | | ATSTP11, STP11, sugar transporter 11 | 3.49755 | 0.0984643 | | -5.1506 |
| AT2G15990 | | transposable element gene | 34.2799 | 0.978216 | | -5.1311 |
| AT1G29020 | | Calcium-binding EF-hand family protein | 52.5806 | 1.50385 | | -5.1278 |
| AT4G13440 | | Calcium-binding EF-hand family protein | 106.024 | 3.03328 | | -5.1274 |
| AT2G19050 | | GDSL-like Lipase/Acylhydrolase superfamily protein | 9.63236 | 0.278971 | | -5.1097 |
| AT3G01420 | | ALPHA-DOX1, DIOX1, DOX1, PADOX-1, Peroxidase superfamily protein | 12.4787 | 0.361786 | | -5.1082 |
| AT3G45530 | | Cysteine/Histidine-rich C1 domain family protein | 3.21827 | 0.0937341 | | -5.1016 |
| AT4G16920 | | Disease resistance protein (TIR-NBS-LRR class) family | 2.87594 | 0.0840651 | | -5.0964 |
| AT3G07490 | | AGD11, ARF-GAP domain 11 | 38.8904 | 1.13937 | | -5.0931 |
| AT1G58320 | | PLAC8 family protein | 32.1595 | 0.951443 | | -5.079 |
| AT3G51540 | | unknown protein | 140.796 | 4.18419 | | -5.0725 |
| AT3G23190 | | HR-like lesion-inducing protein-related | 366.307 | 10.9146 | | -5.0687 |
| AT1G48745 | | unknown protein | 34.1412 | 1.02216 | | -5.0618 |
| AT2G25240 | | Serine protease inhibitor (SERPIN) family protein | 52.9154 | 1.59392 | | -5.053 |
| AT1G21360 | | GLTP2, glycolipid transfer protein 2 | 43.6204 | 1.34333 | | -5.0211 |
| AT5G24310 | | ABIL3, ABL interactor-like protein 3 | 101.958 | 3.18457 | | -5.0007 |
| AT1G23720 | | Proline-rich extensin-like family protein | 1454.68 | 45.5663 | | -4.9966 |
| AT1G79250 | | AGC1.7, AGC kinase 1.7 | 3.7035 | 0.116275 | | -4.9933 |
| AT1G63930 | | ROH1, from the Czech 'roh' meaning 'corner' | 43.3653 | 1.36232 | | -4.9924 |
| AT3G13782 | | NAP1;4, NFA04, NFA4, nucleosome assembly protein1;4 | 209.37 | 6.5882 | | -4.99 |
| AT2G02300 | | AtPP2-B5, PP2-B5, phloem protein 2-B5 | 4.93553 | 0.157395 | | -4.9708 |
| AT1G66570 | | ATSUC7, SUC7, sucrose-proton symporter 7 | 31.2731 | 1.0048 | | -4.9599 |
| AT3G05155 | | Major facilitator superfamily protein | 62.3995 | 2.00907 | | -4.9569 |
| AT1G19230 | | Riboflavin synthase-like superfamily protein | 51.3866 | 1.65536 | | -4.9562 |
| AT4G22666 | | Bifunctional inhibitor/lipid-transfer protein/seed storage 2S albumin superfamily protein | 270.642 | 8.73029 | | -4.9542 |
| AT1G57560 | | AtMYB50, MYB50, myb domain protein 50 | 72.101 | 2.33254 | | -4.9501 |
| AT3G21180 | | ACA9, ATACA9, autoinhibited Ca(2+)-ATPase 9 | 115.613 | 3.74594 | | -4.9478 |
| AT3G48940 | | Remorin family protein | 49.2343 | 1.64026 | | -4.9077 |
| AT1G04700 | | PB1 domain-containing protein tyrosine kinase | 49.9354 | 1.67292 | | -4.8996 |
| AT1G05020 | | ENTH/ANTH/VHS superfamily protein | 4.18853 | 0.140718 | | -4.8956 |
| AT3G17600 | | IAA31, indole-3-acetic acid inducible 31 | 12.1628 | 0.409842 | | -4.8913 |
| AT1G65060 | | 4CL3, 4-coumarate:CoA ligase 3 | 4.04638 | 0.136674 | | -4.8878 |
| AT5G62280 | | Protein of unknown function (DUF1442) | 105.288 | 3.58599 | | -4.8758 |
| AT3G43960 | | Cysteine proteinases superfamily protein | 200.497 | 6.84545 | | -4.8723 |
| AT3G05800 | | AIF1, AtBS1(activation-tagged BRI1 suppressor 1)-interacting factor 1 | 37.6626 | 1.29728 | | -4.8596 |
| AT4G19680 | | ATIRT2, IRT2, iron regulated transporter 2 | 109.85 | 3.84865 | | -4.835 |
| AT5G13930 | | ATCHS, CHS, TT4, Chalcone and stilbene synthase family protein | 134.966 | 4.77061 | | -4.8223 |
| AT5G44480 | | DUR, NAD(P)-binding Rossmann-fold superfamily protein | 74.7474 | 2.64666 | | -4.8198 |
| AT3G28550 | | Proline-rich extensin-like family protein | 1748.86 | 62.0652 | | -4.8165 |
| AT5G63600 | | ATFLS5, FLS5, flavonol synthase 5 | 82.4453 | 2.92904 | | -4.8149 |
| AT1G75620 | | glyoxal oxidase-related protein | 2.79705 | 0.100012 | | -4.8057 |
| AT1G61795 | | PAK-box/P21-Rho-binding family protein | 28.435 | 1.01884 | | -4.8027 |
| AT2G19060 | | SGNH hydrolase-type esterase superfamily protein | 28.6291 | 1.04724 | | -4.7728 |
| AT1G73165 | | CLE1, CLAVATA3/ESR-RELATED 1 | 922.191 | 34.1179 | | -4.7565 |
| AT4G29905 | | unknown protein | 42.2351 | 1.56744 | | -4.752 |
| AT4G24580 | | REN1, Rho GTPase activation protein (RhoGAP) with PH domain | 70.9671 | 2.63631 | | -4.7506 |
| AT3G16390 | | NSP3, nitrile specifier protein 3 | 353.889 | 13.2526 | | -4.739 |
| AT4G09500 | | UDP-Glycosyltransferase superfamily protein | 5.72847 | 0.215031 | | -4.7355 |
| AT2G20520 | | FLA6, FASCICLIN-like arabinogalactan 6 | 49.4497 | 1.87766 | | -4.719 |
| AT4G32950 | | Protein phosphatase 2C family protein | 241.781 | 9.18321 | | -4.7186 |
| AT4G16600 | | Nucleotide-diphospho-sugar transferases superfamily protein | 9.0648 | 0.345115 | | -4.7151 |
| AT5G25880 | | ATNADP-ME3, NADP-ME3, NADP-malic enzyme 3 | 57.118 | 2.17689 | | -4.7136 |
| AT4G27290 | | S-locus lectin protein kinase family protein | 39.0958 | 1.50421 | | -4.6999 |
| AT5G07450 | | CYCP4;3, cyclin p4;3 | 64.7111 | 2.49592 | | -4.6964 |
| AT2G11270 | | citrate synthase-related | 260.422 | 10.0694 | | -4.6928 |
| AT4G26260 | | MIOX4, myo-inositol oxygenase 4 | 5.49002 | 0.213983 | | -4.6813 |
| AT4G07960 | | ATCSLC12, CSLC12, CSLC12, Cellulose-synthase-like C12 | 87.0289 | 3.41639 | | -4.671 |
| AT1G27140 | | ATGSTU14, GST13, GSTU14, glutathione S-transferase tau 14 | 20.9489 | 0.825657 | | -4.6652 |
| AT4G40010 | | SNRK2-7, SNRK2.7, SRK2F, SNF1-related protein kinase 2.7 | 32.4091 | 1.28034 | | -4.6618 |
| AT4G18430 | | AtRABA1e, RABA1e, RAB GTPase homolog A1E | 111.568 | 4.4472 | | -4.6489 |
| AT5G01250 | | alpha 1,4-glycosyltransferase family protein | 5.6873 | 0.227479 | | -4.6439 |
| AT3G61550 | | RING/U-box superfamily protein | 7.37236 | 0.295112 | | -4.6428 |
| AT4G30460 | | glycine-rich protein | 49.893 | 2.00971 | | -4.6338 |
| AT1G66470 | | RHD6, ROOT HAIR DEFECTIVE6 | 161.46 | 6.58115 | | -4.6167 |
| AT5G11070 | | unknown protein | 294.83 | 12.2172 | | -4.5929 |
| AT4G37070 | | AtPLAIVA, PLA IVA, PLP1, Acyl transferase/acyl hydrolase/lysophospholipase superfamily protein | 64.281 | 2.6652 | | -4.5921 |
| AT5G05840 | | Protein of unknown function (DUF620) | 8.3297 | 0.34828 | | -4.58 |
| AT3G59830 | | Integrin-linked protein kinase family | 21.766 | 0.914138 | | -4.5735 |
| AT2G29750 | | UGT71C1, UDP-glucosyl transferase 71C1 | 96.4608 | 4.05632 | | -4.5717 |
| AT1G33090 | | MATE efflux family protein | 20.4393 | 0.862959 | | -4.5659 |
| AT5G61260 | | Plant calmodulin-binding protein-related | 30.5146 | 1.31106 | | -4.5407 |
| AT2G24840 | | AGL61, DIA, AGAMOUS-like 61 | 62.0978 | 2.70141 | | -4.5228 |
| AT4G20110 | | BP80-3;1, VSR3;1, VSR7, VACUOLAR SORTING RECEPTOR 7 | 35.0237 | 1.52904 | | -4.5176 |
| AT1G44020 | | Cysteine/Histidine-rich C1 domain family protein | 1.82474 | 0.0802579 | | -4.5069 |
| AT1G62320 | | ERD (early-responsive to dehydration stress) family protein | 53.8165 | 2.38029 | | -4.4988 |
| AT4G22600 | | unknown protein | 7.47183 | 0.331342 | | -4.4951 |
| AT1G74830 | | Protein of unknown function, DUF593 | 41.5366 | 1.84494 | | -4.4927 |
| AT4G10010 | | Protein kinase superfamily protein | 2.0297 | 0.090408 | | -4.4887 |
| AT5G53830 | | VQ motif-containing protein | 9.82475 | 0.442394 | | -4.473 |
| AT3G09240 | | Protein kinase protein with tetratricopeptide repeat domain | 11.5432 | 0.51995 | | -4.4725 |
| AT3G26500 | | PIRL2, plant intracellular ras group-related LRR 2 | 38.0725 | 1.7151 | | -4.4724 |
| AT5G06800 | | myb-like HTH transcriptional regulator family protein | 104.978 | 4.74244 | | -4.4683 |
| AT1G65610 | | ATGH9A2, KOR2, Six-hairpin glycosidases superfamily protein | 165.887 | 7.60339 | | -4.4474 |
| AT4G14390 | | Ankyrin repeat family protein | 10.4832 | 0.480931 | | -4.4461 |
| AT4G03330 | | ATSYP123, SYP123, syntaxin of plants 123 | 32.7223 | 1.51902 | | -4.4291 |
| AT1G64930 | | CYP89A7, cytochrome P450, family 87, subfamily A, polypeptide 7 | 5.88106 | 0.273518 | | -4.4264 |
| AT2G45220 | | Plant invertase/pectin methylesterase inhibitor superfamily | 184.446 | 8.61363 | | -4.4204 |
| AT2G34655 | | unknown protein | 7.72599 | 0.363566 | | -4.4094 |
| AT2G21880 | | ATRAB7A, ATRABG2, RAB7A, RAB GTPase homolog 7A | 84.6027 | 3.98521 | | -4.408 |
| AT5G09440 | | EXL4, EXORDIUM like 4 | 89.7397 | 4.259 | | -4.3972 |
| AT1G16420 | | ATMC8, MC8, metacaspase 8 | 5.31635 | 0.252483 | | -4.3962 |
| AT2G01540 | | Calcium-dependent lipid-binding (CaLB domain) family protein | 474.404 | 22.5354 | | -4.3959 |
| AT3G06370 | | ATNHX4, NHX4, sodium hydrogen exchanger 4 | 19.9797 | 0.952007 | | -4.3914 |
| AT5G48320 | | Cysteine/Histidine-rich C1 domain family protein | 1.90417 | 0.0914807 | | -4.3796 |
| AT1G64380 | | Integrase-type DNA-binding superfamily protein | 11.4781 | 0.554446 | | -4.3717 |
| AT3G01760 | | Transmembrane amino acid transporter family protein | 9.59626 | 0.465696 | | -4.365 |
| AT2G29740 | | UGT71C2, UDP-glucosyl transferase 71C2 | 23.2408 | 1.13071 | | -4.3614 |
| AT3G04900 | | Heavy metal transport/detoxification superfamily protein | 10.6306 | 0.518696 | | -4.3572 |
| AT4G25433 | | peptidoglycan-binding LysM domain-containing protein | 10.3498 | 0.508522 | | -4.3472 |
| AT2G34500 | | CYP710A1, cytochrome P450, family 710, subfamily A, polypeptide 1 | 76.233 | 3.75831 | | -4.3423 |
| AT4G26330 | | ATSBT3.18, UNE17, Subtilisin-like serine endopeptidase family protein | 2.04589 | 0.101208 | | -4.3373 |
| AT1G45140 | | transposable element gene | 3.4818 | 0.173154 | | -4.3297 |
| AT2G28440 | | proline-rich family protein | 28.0882 | 1.40113 | | -4.3253 |
| AT2G34810 | | FAD-binding Berberine family protein | 12.8607 | 0.647047 | | -4.313 |
| AT4G33020 | | ATZIP9, ZIP9, ZIP metal ion transporter family | 10.8718 | 0.54834 | | -4.3094 |
| AT4G11390 | | Cysteine/Histidine-rich C1 domain family protein | 5.36556 | 0.271666 | | -4.3038 |
| AT1G24320 | | Six-hairpin glycosidases superfamily protein | 40.1752 | 2.04551 | | -4.2958 |
| AT2G34180 | | ATWL2, CIPK13, SnRK3.7, WL2, CBL-interacting protein kinase 13 | 65.395 | 3.33008 | | -4.2956 |
| AT5G41280 | | Receptor-like protein kinase-related family protein | 69.5715 | 3.55533 | | -4.2904 |
| AT1G49310 | | unknown protein | 48.4549 | 2.48173 | | -4.2872 |
| AT4G19690 | | ATIRT1, IRT1, iron-regulated transporter 1 | 3.32618 | 0.171025 | | -4.2816 |
| AT1G30050 | | unknown protein | 4.81614 | 0.250021 | | -4.2678 |
| AT1G72200 | | RING/U-box superfamily protein | 26.4992 | 1.38354 | | -4.2595 |
| AT5G13150 | | ATEXO70C1, EXO70C1, exocyst subunit exo70 family protein C1 | 52.697 | 2.7557 | | -4.2572 |
| AT1G03106 | | unknown protein | 16.4945 | 0.865869 | | -4.2517 |
| AT3G61560 | | Reticulon family protein | 226.411 | 11.9469 | | -4.2442 |
| AT5G21950 | | alpha/beta-Hydrolases superfamily protein | 10.4381 | 0.552717 | | -4.2392 |
| AT5G06990 | | Protein of unknown function, DUF617 | 38.9156 | 2.06317 | | -4.2374 |
| AT5G07150 | | Leucine-rich repeat protein kinase family protein | 13.2811 | 0.705037 | | -4.2355 |
| AT1G59850 | | ARM repeat superfamily protein | 9.36163 | 0.500439 | | -4.2255 |
| AT4G17340 | | DELTA-TIP2, TIP2;2, tonoplast intrinsic protein 2;2 | 81.8083 | 4.46908 | | -4.1942 |
| AT2G16000 | | transposable element gene | 1.50571 | 0.082748 | | -4.1856 |
| AT1G55390 | | Cysteine/Histidine-rich C1 domain family protein | 1.2962 | 0.0723664 | | -4.1628 |
| AT4G11230 | | Riboflavin synthase-like superfamily protein | 24.4326 | 1.37182 | | -4.1547 |
| AT5G01200 | | Duplicated homeodomain-like superfamily protein | 10.0362 | 0.566426 | | -4.1472 |
| AT1G18940 | | Nodulin-like / Major Facilitator Superfamily protein | 15.9634 | 0.904401 | | -4.1417 |
| AT2G38790 | | unknown protein | 89.2405 | 5.07359 | | -4.1366 |
| AT2G28460 | | Cysteine/Histidine-rich C1 domain family protein | 21.3637 | 1.21562 | | -4.1354 |
| AT1G08320 | | bZIP21, TGA9, bZIP transcription factor family protein | 11.6862 | 0.675343 | | -4.113 |
| AT2G21020 | | pseudogene, major intrinsic protein (MIP) family, contains Pfam profile: MIP PF00230 | 2.48189 | 0.143677 | | -4.1105 |
| AT3G18170 | | Glycosyltransferase family 61 protein | 27.7438 | 1.61814 | | -4.0998 |
| AT1G67105 | | other RNA | 6.45057 | 0.377261 | | -4.0958 |
| AT5G62720 | | Integral membrane HPP family protein | 17.815 | 1.04459 | | -4.0921 |
| AT1G53990 | | GLIP3, GDSL-motif lipase 3 | 5.16993 | 0.303195 | | -4.0918 |
| AT3G51330 | | Eukaryotic aspartyl protease family protein | 119.334 | 7.01409 | | -4.0886 |
| AT3G60540 | | Preprotein translocase Sec, Sec61-beta subunit protein | 35.6917 | 2.10156 | | -4.0861 |
| AT1G68620 | | alpha/beta-Hydrolases superfamily protein | 10.5542 | 0.621648 | | -4.0856 |
| AT2G26400 | | ARD, ARD3, ATARD3, acireductone dioxygenase 3 | 38.9796 | 2.29614 | | -4.0854 |
| AT4G38620 | | ATMYB4, MYB4, myb domain protein 4 | 15.3817 | 0.906201 | | -4.0852 |
| AT2G17830 | | F-box and associated interaction domains-containing protein | 59.5139 | 3.51293 | | -4.0825 |
| AT3G49700 | | ACS9, AtACS9, ETO3, 1-aminocyclopropane-1-carboxylate synthase 9 | 5.33736 | 0.315157 | | -4.082 |
| AT1G01780 | | GATA type zinc finger transcription factor family protein | 72.1454 | 4.27052 | | -4.0784 |
| AT3G16210 | | F-box family protein | 9.59477 | 0.569167 | | -4.0753 |
| AT5G04390 | | C2H2-type zinc finger family protein | 2.804 | 0.166354 | | -4.0752 |
| AT4G30270 | | MERI-5, MERI5B, SEN4, XTH24, xyloglucan endotransglucosylase/hydrolase 24 | 22.165 | 1.31669 | | -4.0733 |
| AT1G58037 | | Cysteine/Histidine-rich C1 domain family protein | 2.63675 | 0.157259 | | -4.0675 |
| AT2G02620 | | Cysteine/Histidine-rich C1 domain family protein | 11.374 | 0.682936 | | -4.0579 |
| AT5G01215 | | other RNA | 19.4062 | 1.17744 | | -4.0428 |
| AT2G35730 | | Heavy metal transport/detoxification superfamily protein | 64.8507 | 3.94136 | | -4.0404 |
| AT2G28110 | | FRA8, IRX7, Exostosin family protein | 4.15433 | 0.253887 | | -4.0324 |
| AT1G74080 | | ATMYB122, MYB122, myb domain protein 122 | 4.99956 | 0.306451 | | -4.0281 |
| AT1G61840 | | Cysteine/Histidine-rich C1 domain family protein | 4.18771 | 0.256998 | | -4.0263 |
| AT2G04680 | | Cysteine/Histidine-rich C1 domain family protein | 8.95639 | 0.551561 | | -4.0213 |
| AT5G44900 | | Toll-Interleukin-Resistance (TIR) domain family protein | 4.17558 | 0.257429 | | -4.0197 |
| AT1G63245 | | CLE14, CLAVATA3/ESR-RELATED 14 | 1507.52 | 93.2893 | | -4.0143 |
| AT4G21215 | | unknown protein | 16.8602 | 1.05 | | -4.0052 |
| AT4G35200 | | Arabidopsis protein of unknown function (DUF241) | 29.5242 | 1.8513 | | -3.9953 |
| AT5G12880 | | proline-rich family protein | 160.477 | 10.1072 | | -3.9889 |
| AT2G15980 | | Tetratricopeptide repeat (TPR)-like superfamily protein | 39.4528 | 2.4858 | | -3.9883 |
| AT4G13860 | | RNA-binding (RRM/RBD/RNP motifs) family protein | 59.7131 | 3.76483 | | -3.9874 |
| AT4G22070 | | ATWRKY31 | 19.1717 | 1.20887 | | -3.9873 |
| AT5G13990 | | ATEXO70C2, EXO70C2, exocyst subunit exo70 family protein C2 | 114.103 | 7.30302 | | -3.9657 |
| AT4G22214 | | Defensin-like (DEFL) family protein | 927.985 | 59.3972 | | -3.9656 |
| AT2G34960 | | CAT5, cationic amino acid transporter 5 | 4.33775 | 0.278032 | | -3.9636 |
| AT3G53820 | | C2H2 and C2HC zinc fingers superfamily protein | 231.311 | 14.896 | | -3.9568 |
| AT4G38830 | | CRK26, cysteine-rich RLK (RECEPTOR-like protein kinase) 26 | 14.1427 | 0.913051 | | -3.9532 |
| AT3G14850 | | TBL41, TRICHOME BIREFRINGENCE-LIKE 41 | 43.7417 | 2.83187 | | -3.9492 |
| AT5G44130 | | FLA13, FASCICLIN-like arabinogalactan protein 13 precursor | 12.2916 | 0.797854 | | -3.9454 |
| AT5G40730 | | AGP24, ATAGP24, arabinogalactan protein 24 | 2027.53 | 131.904 | | -3.9422 |
| AT5G56160 | | Sec14p-like phosphatidylinositol transfer family protein | 14.8964 | 0.969393 | | -3.9417 |
| AT2G23620 | | ATMES1, MES1, methyl esterase 1 | 19.9987 | 1.30218 | | -3.9409 |
| AT3G27510 | | Cysteine/Histidine-rich C1 domain family protein | 2.11736 | 0.138215 | | -3.9373 |
| AT4G00460 | | ATROPGEF3, ROPGEF3, RHO guanyl-nucleotide exchange factor 3 | 116.672 | 7.66071 | | -3.9288 |
| AT4G25070 | | unknown protein | 77.9769 | 5.12659 | | -3.927 |
| AT4G35660 | | Arabidopsis protein of unknown function (DUF241) | 3.58664 | 0.23592 | | -3.9263 |
| AT1G76210 | | Arabidopsis protein of unknown function (DUF241) | 36.7597 | 2.41801 | | -3.9262 |
| AT4G16350 | | CBL6, SCABP2, calcineurin B-like protein 6 | 34.8977 | 2.29663 | | -3.9255 |
| AT1G18410 | | P-loop containing nucleoside triphosphate hydrolases superfamily protein | 2.52524 | 0.166804 | | -3.9202 |
| AT4G31250 | | Leucine-rich repeat protein kinase family protein | 39.6763 | 2.62096 | | -3.9201 |
| AT5G06090 | | ATGPAT7, GPAT7, glycerol-3-phosphate acyltransferase 7 | 7.34847 | 0.4861 | | -3.9181 |
| AT1G05320 | | function unknown | 69.93 | 4.65597 | | -3.9088 |
| AT5G45220 | | Disease resistance protein (TIR-NBS-LRR class) family | 5.07928 | 0.338321 | | -3.9082 |
| AT1G33800 | | Protein of unknown function (DUF579) | 216.503 | 14.4211 | | -3.9081 |
| AT1G63450 | | RHS8, root hair specific 8 | 20.4915 | 1.37791 | | -3.8945 |
| AT2G31860 | | pseudogene, poly (ADP-ribose) glycohydrolase, putative, contains Pfam domain, PF05028: poly (ADP-ribose) glycohydrolase (PARG) | 2.91927 | 0.19662 | | -3.8921 |
| AT4G23550 | | ATWRKY29 | 24.711 | 1.67022 | | -3.887 |
| AT1G11160 | | Transducin/WD40 repeat-like superfamily protein | 17.1499 | 1.1623 | | -3.8832 |
| AT5G01610 | | Protein of unknown function, DUF538 | 180.271 | 12.2245 | | -3.8823 |
| AT3G56400 | | ATWRKY70 | 16.8807 | 1.14721 | | -3.8792 |
| AT1G34040 | | Pyridoxal phosphate (PLP)-dependent transferases superfamily protein | 2.69736 | 0.183857 | | -3.8749 |
| AT1G30900 | | BP80-3;3, VSR3;3, VSR6, VACUOLAR SORTING RECEPTOR 6 | 175.769 | 11.9986 | | -3.8727 |
| AT3G62100 | | IAA30, indole-3-acetic acid inducible 30 | 139.472 | 9.59808 | | -3.8611 |
| AT1G61750 | | Receptor-like protein kinase-related family protein | 9.87134 | 0.680327 | | -3.859 |
| AT3G55120 | | A11, CFI, TT5, Chalcone-flavanone isomerase family protein | 12.97 | 0.896791 | | -3.8543 |
| AT3G09020 | | alpha 1,4-glycosyltransferase family protein | 18.227 | 1.26062 | | -3.8539 |
| AT1G14960 | | Polyketide cyclase/dehydrase and lipid transport superfamily protein | 122.489 | 8.48455 | | -3.8517 |
| AT1G54740 | | Protein of unknown function (DUF3049) | 14.8321 | 1.03078 | | -3.8469 |
| AT1G28600 | | GDSL-like Lipase/Acylhydrolase superfamily protein | 4.93177 | 0.342918 | | -3.8462 |
| AT2G25810 | | TIP4;1, tonoplast intrinsic protein 4;1 | 197.839 | 13.7724 | | -3.8445 |
| AT5G67620 | | unknown protein | 116.588 | 8.13089 | | -3.8419 |
| AT5G10410 | | ENTH/ANTH/VHS superfamily protein | 91.9798 | 6.41601 | | -3.8416 |
| AT3G03000 | | EF hand calcium-binding protein family | 37.5296 | 2.62963 | | -3.8351 |
| AT1G64210 | | Leucine-rich repeat protein kinase family protein | 2.32369 | 0.16328 | | -3.831 |
| AT3G05920 | | Heavy metal transport/detoxification superfamily protein | 169.788 | 11.9305 | | -3.831 |
| AT1G56550 | | RXGT1, RhamnoGalacturonan speci&#64257;c Xylosyltransferase 1 | 70.9225 | 4.9953 | | -3.8276 |
| AT3G04010 | | O-Glycosyl hydrolases family 17 protein | 428.945 | 30.225 | | -3.827 |
| AT4G23070 | | ATRBL7, RBL7, RHOMBOID-like protein 7 | 7.2282 | 0.511619 | | -3.8205 |
| AT5G53370 | | ATPMEPCRF, PMEPCRF, pectin methylesterase PCR fragment F | 29.1557 | 2.06983 | | -3.8162 |
| AT3G04070 | | anac047, NAC047, NAC domain containing protein 47 | 65.2786 | 4.64433 | | -3.8131 |
| AT1G29395 | | COR413-TM1, COR413IM1, COR414-TM1, COLD REGULATED 314 INNER MEMBRANE 1 | 5.63284 | 0.402593 | | -3.8065 |
| AT4G20730 | | transposable element gene | 5.51128 | 0.397336 | | -3.794 |
| AT3G07195 | | RPM1-interacting protein 4 (RIN4) family protein | 38.2359 | 2.76776 | | -3.7881 |
| AT3G23175 | | HR-like lesion-inducing protein-related | 297.022 | 21.5645 | | -3.7838 |
| AT2G20670 | | Protein of unknown function (DUF506) | 198.928 | 14.4747 | | -3.7806 |
| AT2G26420 | | PIP5K3, 1-phosphatidylinositol-4-phosphate 5-kinase 3 | 8.57448 | 0.625113 | | -3.7779 |
| AT5G65800 | | ACS5, ATACS5, CIN5, ETO2, ACC synthase 5 | 17.145 | 1.25001 | | -3.7778 |
| AT2G20620 | | Protein of unknown function (DUF626) | 8.3447 | 0.609416 | | -3.7754 |
| AT1G72890 | | Disease resistance protein (TIR-NBS class) | 8.79457 | 0.64264 | | -3.7745 |
| AT4G10770 | | ATOPT7, OPT7, oligopeptide transporter 7 | 30.6851 | 2.25224 | | -3.7681 |
| AT5G44610 | | MAP18, PCAP2, microtubule-associated protein 18 | 327.958 | 24.1866 | | -3.7612 |
| AT5G41680 | | Protein kinase superfamily protein | 35.4244 | 2.6138 | | -3.7605 |
| AT1G02380 | | unknown protein | 26.3932 | 1.94938 | | -3.7591 |
| AT4G22758 | | unknown protein | 64.7652 | 4.80327 | | -3.7531 |
| AT5G54240 | | Protein of unknown function (DUF1223) | 8.12545 | 0.604209 | | -3.7493 |
| AT2G38640 | | Protein of unknown function (DUF567) | 22.8906 | 1.70323 | | -3.7484 |
| AT2G46750 | | D-arabinono-1,4-lactone oxidase family protein | 6.14732 | 0.458944 | | -3.7436 |
| AT1G78230 | | Outer arm dynein light chain 1 protein | 18.663 | 1.39465 | | -3.7422 |
| AT1G61230 | | Mannose-binding lectin superfamily protein | 2.43767 | 0.182278 | | -3.7413 |
| AT4G09770 | | TRAF-like family protein | 7.66564 | 0.574639 | | -3.7377 |
| AT2G11290 | | transposable element gene | 5.49121 | 0.413844 | | -3.73 |
| AT2G17660 | | RPM1-interacting protein 4 (RIN4) family protein | 761.31 | 57.4593 | | -3.7279 |
| AT5G60770 | | ATNRT2.4, NRT2.4, nitrate transporter 2.4 | 23.3093 | 1.76064 | | -3.7267 |
| AT5G67450 | | AZF1, ZF1, zinc-finger protein 1 | 14.8959 | 1.13073 | | -3.7196 |
| AT5G57070 | | hydroxyproline-rich glycoprotein family protein | 49.8687 | 3.78625 | | -3.7193 |
| AT4G19530 | | disease resistance protein (TIR-NBS-LRR class) family | 2.24198 | 0.170742 | | -3.7149 |
| AT2G38490 | | CIPK22, SnRK3.19, CBL-interacting protein kinase 22 | 12.0333 | 0.92151 | | -3.7069 |
| AT1G32950 | | Subtilase family protein | 10.2627 | 0.788284 | | -3.7026 |
| AT5G09430 | | alpha/beta-Hydrolases superfamily protein | 25.8681 | 1.99078 | | -3.6998 |
| AT3G10470 | | C2H2-type zinc finger family protein | 2.1944 | 0.16977 | | -3.6922 |
| AT3G15540 | | IAA19, MSG2, indole-3-acetic acid inducible 19 | 288.02 | 22.3099 | | -3.6904 |
| AT5G43150 | | unknown protein | 17.2338 | 1.34832 | | -3.676 |
| AT4G37220 | | Cold acclimation protein WCOR413 family | 18.8171 | 1.47407 | | -3.6742 |
| AT2G15370 | | ATFUT5, FUT5, fucosyltransferase 5 | 5.57187 | 0.438409 | | -3.6678 |
| AT5G55950 | | Nucleotide/sugar transporter family protein | 4.15352 | 0.328867 | | -3.6588 |
| AT2G28960 | | Leucine-rich repeat protein kinase family protein | 14.0629 | 1.1159 | | -3.6556 |
| AT5G40260 | | Nodulin MtN3 family protein | 5.84408 | 0.464072 | | -3.6546 |
| AT1G56010 | | anac021, ANAC022, NAC1, NAC domain containing protein 1 | 41.566 | 3.30191 | | -3.654 |
| AT3G11385 | | Cysteine/Histidine-rich C1 domain family protein | 4.47628 | 0.355941 | | -3.6526 |
| AT3G54040 | | PAR1 protein | 259.244 | 20.6245 | | -3.6519 |
| AT4G22620 | | SAUR-like auxin-responsive protein family | 45.3796 | 3.64854 | | -3.6367 |
| AT1G53340 | | Cysteine/Histidine-rich C1 domain family protein | 14.9653 | 1.20473 | | -3.6348 |
| AT1G15040 | | Class I glutamine amidotransferase-like superfamily protein | 340.555 | 27.5151 | | -3.6296 |
| AT1G05810 | | ARA, ARA-1, ATRAB11D, ATRABA5E, RABA5E, RAB GTPase homolog A5E | 153.349 | 12.397 | | -3.6288 |
| AT5G06839 | | bZIP65, TGA10, bZIP transcription factor family protein | 18.3423 | 1.49624 | | -3.6158 |
| AT1G49450 | | Transducin/WD40 repeat-like superfamily protein | 13.1922 | 1.0766 | | -3.6151 |
| AT3G55150 | | ATEXO70H1, EXO70H1, exocyst subunit exo70 family protein H1 | 21.5778 | 1.76753 | | -3.6097 |
| AT5G07770 | | Actin-binding FH2 protein | 16.0326 | 1.31705 | | -3.6056 |
| AT2G18450 | | SDH1-2, succinate dehydrogenase 1-2 | 110.514 | 9.11401 | | -3.6 |
| AT5G22570 | | ATWRKY38 | 42.3492 | 3.49925 | | -3.5972 |
| AT1G53830 | | ATPME2, PME2, pectin methylesterase 2 | 221.151 | 18.3097 | | -3.5944 |
| AT5G46040 | | Major facilitator superfamily protein | 25.1274 | 2.08116 | | -3.5938 |
| AT1G61860 | | Protein kinase superfamily protein | 22.6968 | 1.88542 | | -3.5895 |
| AT1G19250 | | FMO1, flavin-dependent monooxygenase 1 | 6.93717 | 0.577603 | | -3.5862 |
| AT2G30930 | | unknown protein | 360.634 | 30.0296 | | -3.5861 |
| AT4G30640 | | RNI-like superfamily protein | 8.28116 | 0.69283 | | -3.5793 |
| AT3G25790 | | myb-like transcription factor family protein | 9.44608 | 0.793658 | | -3.5731 |
| AT1G19190 | | alpha/beta-Hydrolases superfamily protein | 17.2723 | 1.46088 | | -3.5636 |
| AT1G27030 | | unknown protein | 36.2116 | 3.07547 | | -3.5576 |
| AT1G78100 | | F-box family protein | 786.804 | 66.8369 | | -3.5573 |
| AT1G61275 | | U12, U12; snRNA | 713.21 | 60.8873 | | -3.5501 |
| AT2G34940 | | BP80-3;2, VSR3;2, VSR5, VACUOLAR SORTING RECEPTOR 5 | 52.7697 | 4.5124 | | -3.5477 |
| AT1G64400 | | LACS3, AMP-dependent synthetase and ligase family protein | 10.3705 | 0.887508 | | -3.5466 |
| AT2G16760 | | Calcium-dependent phosphotriesterase superfamily protein | 17.5319 | 1.50405 | | -3.5431 |
| AT1G64480 | | CBL8, calcineurin B-like protein 8 | 25.1419 | 2.17402 | | -3.5317 |
| AT5G22920 | | CHY-type/CTCHY-type/RING-type Zinc finger protein | 153.729 | 13.2964 | | -3.5313 |
| AT1G73680 | | ALPHA DOX2, alpha dioxygenase | 9.59924 | 0.835131 | | -3.5229 |
| AT1G76230 | | unknown protein | 10.3168 | 0.897649 | | -3.5227 |
| AT3G27070 | | TOM20-1, translocase outer membrane 20-1 | 6.35289 | 0.554656 | | -3.5178 |
| AT1G13930 | | Involved in response to salt stress. Knockout mutants are hypersensitive to salt stress. | 40.6054 | 3.54977 | | -3.5159 |
| AT5G47450 | | ATTIP2;3, DELTA-TIP3, TIP2;3, tonoplast intrinsic protein 2;3 | 632.373 | 55.342 | | -3.5143 |
| AT4G38880 | | ASE3, ATASE3, GLN phosphoribosyl pyrophosphate amidotransferase 3 | 5.21862 | 0.458217 | | -3.5096 |
| AT1G03550 | | Secretory carrier membrane protein (SCAMP) family protein | 69.3496 | 6.09104 | | -3.5091 |
| AT1G73330 | | ATDR4, DR4, drought-repressed 4 | 4.71834 | 0.416565 | | -3.5017 |
| AT5G18150 | | Methyltransferase-related protein | 684.619 | 60.5441 | | -3.4992 |
| AT3G56000 | | ATCSLA14, CSLA14, cellulose synthase like A14 | 57.0746 | 5.07221 | | -3.4922 |
| AT1G50890 | | ARM repeat superfamily protein | 47.4969 | 4.22426 | | -3.4911 |
| AT5G24140 | | SQP2, squalene monooxygenase 2 | 108.444 | 9.65024 | | -3.4902 |
| AT2G35890 | | CPK25, calcium-dependent protein kinase 25 | 17.1009 | 1.53235 | | -3.4803 |
| AT2G27360 | | GDSL-like Lipase/Acylhydrolase superfamily protein | 14.7443 | 1.32258 | | -3.4787 |
| AT4G27480 | | Core-2/I-branching beta-1,6-N-acetylglucosaminyltransferase family protein | 25.4177 | 2.28265 | | -3.4771 |
| AT1G60110 | | Mannose-binding lectin superfamily protein | 1.22031 | 0.109971 | | -3.4721 |
| AT1G68600 | | Aluminium activated malate transporter family protein | 1.70899 | 0.154394 | | -3.4685 |
| AT1G02340 | | FBI1, HFR1, REP1, RSF1, basic helix-loop-helix (bHLH) DNA-binding superfamily protein | 9.82746 | 0.889122 | | -3.4664 |
| AT3G03290 | | Adenine nucleotide alpha hydrolases-like superfamily protein | 12.098 | 1.09724 | | -3.4628 |
| AT4G11470 | | CRK31, cysteine-rich RLK (RECEPTOR-like protein kinase) 31 | 3.33979 | 0.307411 | | -3.4415 |
| AT1G09540 | | ATMYB61, MYB61, myb domain protein 61 | 26.1597 | 2.41019 | | -3.4401 |
| AT3G12500 | | ATHCHIB, B-CHI, CHI-B, HCHIB, PR-3, PR3, basic chitinase | 54.134 | 5.00602 | | -3.4348 |
| AT2G31350 | | GLX2-5, glyoxalase 2-5 | 163.768 | 15.1616 | | -3.4332 |
| AT5G37490 | | ARM repeat superfamily protein | 1.96624 | 0.182152 | | -3.4322 |
| AT5G27100 | | ATGLR2.1, GLR2.1, glutamate receptor 2.1 | 7.90356 | 0.735548 | | -3.4256 |
| AT2G18980 | | Peroxidase superfamily protein | 77.9982 | 7.26961 | | -3.4235 |
| AT3G54363 | | unknown protein | 124.475 | 11.6312 | | -3.4198 |
| AT5G27350 | | SFP1, Major facilitator superfamily protein | 3.59609 | 0.336705 | | -3.4169 |
| AT1G04470 | | Protein of unknown function (DUF810) | 1.46973 | 0.137931 | | -3.4135 |
| AT3G48020 | | unknown protein | 15.2227 | 1.43213 | | -3.41 |
| AT3G56930 | | DHHC-type zinc finger family protein | 146.087 | 13.7746 | | -3.4067 |
| AT2G39900 | | GATA type zinc finger transcription factor family protein | 311.315 | 29.5 | | -3.3996 |
| AT4G14860 | | atofp11, OFP11, ovate family protein 11 | 13.7763 | 1.30665 | | -3.3982 |
| AT3G07940 | | Calcium-dependent ARF-type GTPase activating protein family | 48.1482 | 4.57929 | | -3.3943 |
| AT3G21560 | | UGT84A2, UDP-Glycosyltransferase superfamily protein | 7.41631 | 0.706689 | | -3.3916 |
| AT1G15405 | | other RNA | 177.029 | 16.8793 | | -3.3907 |
| AT3G61390 | | RING/U-box superfamily protein | 28.8102 | 2.77069 | | -3.3783 |
| AT5G41290 | | Receptor-like protein kinase-related family protein | 22.7611 | 2.18897 | | -3.3783 |
| AT3G18560 | | unknown protein | 25.2787 | 2.44045 | | -3.3727 |
| AT5G24170 | | Got1/Sft2-like vescicle transport protein family | 68.4319 | 6.62262 | | -3.3692 |
| AT5G48290 | | Heavy metal transport/detoxification superfamily protein | 3.77746 | 0.366212 | | -3.3667 |
| AT3G12040 | | DNA-3-methyladenine glycosylase (MAG) | 30.0115 | 2.9131 | | -3.3649 |
| AT4G27260 | | GH3.5, WES1, Auxin-responsive GH3 family protein | 168.659 | 16.4149 | | -3.361 |
| AT2G46300 | | Late embryogenesis abundant (LEA) hydroxyproline-rich glycoprotein family | 2.80355 | 0.273016 | | -3.3602 |
| AT5G15180 | | Peroxidase superfamily protein | 64.0184 | 6.26012 | | -3.3542 |
| AT1G54530 | | Calcium-binding EF hand family protein | 34.0035 | 3.32935 | | -3.3524 |
| AT3G44510 | | alpha/beta-Hydrolases superfamily protein | 11.2669 | 1.11307 | | -3.3395 |
| AT2G33310 | | IAA13, auxin-induced protein 13 | 82.2792 | 8.13975 | | -3.3375 |
| AT2G15390 | | atfut4, FUT4, fucosyltransferase 4 | 13.1441 | 1.30192 | | -3.3357 |
| AT3G09520 | | ATEXO70H4, EXO70H4, exocyst subunit exo70 family protein H4 | 3.12196 | 0.309394 | | -3.3349 |
| AT2G02700 | | Cysteine/Histidine-rich C1 domain family protein | 12.837 | 1.27248 | | -3.3346 |
| AT5G38340 | | Disease resistance protein (TIR-NBS-LRR class) family | 2.67217 | 0.265224 | | -3.3327 |
| AT5G55020 | | ATMYB120, MYB120, myb domain protein 120 | 2.53083 | 0.252435 | | -3.3256 |
| AT4G02390 | | APP, ATPARP1, PARP1, PP, poly(ADP-ribose) polymerase | 37.8809 | 3.78638 | | -3.3226 |
| AT1G01180 | | S-adenosyl-L-methionine-dependent methyltransferases superfamily protein | 22.5398 | 2.25418 | | -3.3218 |
| AT5G55050 | | GDSL-like Lipase/Acylhydrolase superfamily protein | 65.3686 | 6.53833 | | -3.3216 |
| AT4G39070 | | B-box zinc finger family protein | 24.0744 | 2.40985 | | -3.3205 |
| AT2G22905 | | Expressed protein | 4.44945 | 0.446063 | | -3.3183 |
| AT5G06820 | | SRF2, STRUBBELIG-receptor family 2 | 12.4835 | 1.25235 | | -3.3173 |
| AT2G23170 | | GH3.3, Auxin-responsive GH3 family protein | 25.0764 | 2.52091 | | -3.3143 |
| AT1G65850 | | Disease resistance protein (TIR-NBS-LRR class) family | 1.6799 | 0.169224 | | -3.3114 |
| AT5G40010 | | AATP1, AAA-ATPase 1 | 6.15818 | 0.624946 | | -3.3007 |
| AT1G22530 | | PATL2, PATELLIN 2 | 186.164 | 19.0348 | | -3.2899 |
| AT1G72125 | | Major facilitator superfamily protein | 22.674 | 2.32216 | | -3.2875 |
| AT2G37440 | | DNAse I-like superfamily protein | 49.5012 | 5.07101 | | -3.2871 |
| AT1G67330 | | Protein of unknown function (DUF579) | 97.9835 | 10.0397 | | -3.2868 |
| AT3G27884 | | other RNA | 9.49056 | 0.973835 | | -3.2847 |
| AT1G67785 | | unknown protein | 3258.96 | 334.996 | | -3.2822 |
| AT4G27350 | | Protein of unknown function (DUF1223) | 244.81 | 25.1782 | | -3.2814 |
| AT1G72860 | | Disease resistance protein (TIR-NBS-LRR class) family | 12.9882 | 1.33751 | | -3.2796 |
| AT4G25080 | | CHLM, magnesium-protoporphyrin IX methyltransferase | 26.4964 | 2.73635 | | -3.2755 |
| AT4G12520 | | Bifunctional inhibitor/lipid-transfer protein/seed storage 2S albumin superfamily protein | 37.1342 | 3.83656 | | -3.2749 |
| AT1G26970 | | Protein kinase superfamily protein | 16.2133 | 1.67576 | | -3.2743 |
| AT5G53250 | | AGP22, ATAGP22, arabinogalactan protein 22 | 220.897 | 22.9246 | | -3.2684 |
| AT3G45060 | | ATNRT2.6, NRT2.6, high affinity nitrate transporter 2.6 | 573.969 | 59.6096 | | -3.2674 |
| AT2G22460 | | Protein of unknown function, DUF617 | 2.03965 | 0.211913 | | -3.2668 |
| AT3G11390 | | Cysteine/Histidine-rich C1 domain family protein | 3.76711 | 0.391929 | | -3.2648 |
| AT1G13100 | | CYP71B29, cytochrome P450, family 71, subfamily B, polypeptide 29 | 1.91746 | 0.199706 | | -3.2632 |
| AT5G24410 | | PGL4, 6-phosphogluconolactonase 4 | 15.2605 | 1.59477 | | -3.2584 |
| AT3G21710 | | unknown protein | 190.137 | 19.9234 | | -3.2545 |
| AT1G72870 | | Disease resistance protein (TIR-NBS class) | 15.5963 | 1.63679 | | -3.2523 |
| AT5G43350 | | ATPT1, PHT1;1, phosphate transporter 1;1 | 34.3803 | 3.62091 | | -3.2472 |
| AT1G15640 | | unknown protein | 30.39 | 3.20276 | | -3.2462 |
| AT3G12502 | | other RNA | 55.0065 | 5.80044 | | -3.2454 |
| AT5G40510 | | Sucrase/ferredoxin-like family protein | 242.636 | 25.6875 | | -3.2397 |
| AT5G64100 | | Peroxidase superfamily protein | 727.186 | 77.0111 | | -3.2392 |
| AT2G32160 | | S-adenosyl-L-methionine-dependent methyltransferases superfamily protein | 7.50187 | 0.794936 | | -3.2383 |
| AT1G78260 | | RNA-binding (RRM/RBD/RNP motifs) family protein | 44.6488 | 4.73431 | | -3.2374 |
| AT1G22220 | | F-box family protein | 12.9136 | 1.37217 | | -3.2344 |
| AT3G05170 | | Phosphoglycerate mutase family protein | 90.1163 | 9.58352 | | -3.2332 |
| AT5G45580 | | Homeodomain-like superfamily protein | 134.741 | 14.3338 | | -3.2327 |
| AT2G36830 | | GAMMA-TIP, GAMMA-TIP1, TIP1;1, gamma tonoplast intrinsic protein | 974.405 | 103.689 | | -3.2323 |
| AT1G66910 | | Protein kinase superfamily protein | 12.069 | 1.28736 | | -3.2288 |
| AT2G03370 | | Glycosyltransferase family 61 protein | 2.03019 | 0.218015 | | -3.2191 |
| AT1G14550 | | Peroxidase superfamily protein | 27.0548 | 2.90707 | | -3.2183 |
| AT1G26360 | | ATMES13, MES13, methyl esterase 13 | 5.82446 | 0.627281 | | -3.2149 |
| AT2G44230 | | Plant protein of unknown function (DUF946) | 24.0215 | 2.58786 | | -3.2145 |
| AT2G28710 | | C2H2-type zinc finger family protein | 14.2587 | 1.53816 | | -3.2126 |
| AT5G26660 | | ATMYB86, MYB86, myb domain protein 86 | 54.9783 | 5.93874 | | -3.2106 |
| AT5G58940 | | CRCK1, calmodulin-binding receptor-like cytoplasmic kinase 1 | 38.1678 | 4.1385 | | -3.2052 |
| AT5G01050 | | Laccase/Diphenol oxidase family protein | 25.7688 | 2.79978 | | -3.2022 |
| AT3G11370 | | Cysteine/Histidine-rich C1 domain family protein | 1.49717 | 0.163125 | | -3.1982 |
| AT1G52750 | | alpha/beta-Hydrolases superfamily protein | 130.458 | 14.2276 | | -3.1968 |
| AT5G06230 | | TBL9, TRICHOME BIREFRINGENCE-LIKE 9 | 34.4147 | 3.75889 | | -3.1946 |
| AT3G22830 | | AT-HSFA6B, HSFA6B, heat shock transcription factor A6B | 338.883 | 37.2627 | | -3.185 |
| AT5G65500 | | U-box domain-containing protein kinase family protein | 1.41618 | 0.155872 | | -3.1836 |
| AT4G21780 | | unknown protein | 8.10851 | 0.894419 | | -3.1804 |
| AT2G27660 | | Cysteine/Histidine-rich C1 domain family protein | 41.893 | 4.62137 | | -3.1803 |
| AT5G52020 | | Integrase-type DNA-binding superfamily protein | 91.8093 | 10.1282 | | -3.1803 |
| AT5G04310 | | Pectin lyase-like superfamily protein | 4.23116 | 0.470243 | | -3.1696 |
| AT2G44290 | | Bifunctional inhibitor/lipid-transfer protein/seed storage 2S albumin superfamily protein | 20.5867 | 2.2888 | | -3.1691 |
| AT5G24105 | | AGP41, arabinogalactan protein 41 | 44.2348 | 4.92272 | | -3.1677 |
| AT4G37950 | | Rhamnogalacturonate lyase family protein | 1.35937 | 0.152123 | | -3.1596 |
| AT3G09330 | | Transmembrane amino acid transporter family protein | 5.91769 | 0.6641 | | -3.1556 |
| AT4G31380 | | FLP1, FPF1-like protein 1 | 6.08319 | 0.683178 | | -3.1545 |
| AT4G21250 | | Sulfite exporter TauE/SafE family protein | 2.56826 | 0.28897 | | -3.1518 |
| AT1G01730 | | unknown protein | 159.28 | 17.9865 | | -3.1466 |
| AT3G54950 | | PLA IIIA, PLP7, patatin-like protein 6 | 48.5347 | 5.48234 | | -3.1462 |
| AT1G71530 | | Protein kinase superfamily protein | 83.882 | 9.47798 | | -3.1457 |
| AT1G51680 | | 4CL.1, 4CL1, AT4CL1, 4-coumarate:CoA ligase 1 | 38.278 | 4.34022 | | -3.1407 |
| AT5G54490 | | PBP1, pinoid-binding protein 1 | 287.933 | 32.7306 | | -3.137 |
| AT3G21750 | | UGT71B1, UDP-glucosyl transferase 71B1 | 25.327 | 2.88012 | | -3.1365 |
| AT1G52240 | | ATROPGEF11, PIRF1, ROPGEF11, RHO guanyl-nucleotide exchange factor 11 | 222.34 | 25.3168 | | -3.1346 |
| AT5G10770 | | Eukaryotic aspartyl protease family protein | 10.952 | 1.24741 | | -3.1342 |
| AT1G66725 | | MIR163, MIR163; miRNA | 5.15305 | 0.589336 | | -3.1283 |
| AT4G00940 | | Dof-type zinc finger DNA-binding family protein | 3.70093 | 0.423566 | | -3.1272 |
| AT4G20460 | | NAD(P)-binding Rossmann-fold superfamily protein | 74.9464 | 8.57923 | | -3.1269 |
| AT3G29240 | | Protein of unknown function (DUF179) | 30.8266 | 3.53292 | | -3.1252 |
| AT3G05490 | | RALFL22, ralf-like 22 | 471.929 | 54.1202 | | -3.1243 |
| AT1G69790 | | Protein kinase superfamily protein | 10.5225 | 1.20867 | | -3.122 |
| AT4G01140 | | Protein of unknown function (DUF1191) | 24.1913 | 2.78544 | | -3.1185 |
| AT1G05630 | | 5PTASE13, AT5PTASE13, Endonuclease/exonuclease/phosphatase family protein | 24.5091 | 2.82692 | | -3.116 |
| AT4G39675 | | unknown protein | 148.814 | 17.1982 | | -3.1132 |
| AT3G51860 | | ATCAX3, ATHCX1, CAX1-LIKE, CAX3, cation exchanger 3 | 6.31005 | 0.730419 | | -3.1109 |
| AT1G03850 | | Glutaredoxin family protein | 448.238 | 51.9894 | | -3.108 |
| AT2G02690 | | Cysteine/Histidine-rich C1 domain family protein | 2.57251 | 0.298491 | | -3.1074 |
| AT3G62990 | | unknown protein | 8.04326 | 0.935722 | | -3.1036 |
| AT1G13950 | | ATELF5A-1, EIF-5A, EIF5A, ELF5A-1, eukaryotic elongation factor 5A-1 | 107.303 | 12.4879 | | -3.1031 |
| AT5G19040 | | ATIPT5, IPT5, isopentenyltransferase 5 | 22.6776 | 2.63966 | | -3.1028 |
| AT1G55365 | | unknown protein | 14.1245 | 1.65217 | | -3.0958 |
| AT1G27420 | | Galactose oxidase/kelch repeat superfamily protein | 29.9869 | 3.50773 | | -3.0957 |
| AT2G14210 | | AGL44, ANR1, AGAMOUS-like 44 | 25.7774 | 3.01874 | | -3.0941 |
| AT3G01850 | | Aldolase-type TIM barrel family protein | 14.7237 | 1.72598 | | -3.0927 |
| AT5G55780 | | Cysteine/Histidine-rich C1 domain family protein | 1.85406 | 0.217527 | | -3.0914 |
| AT1G15580 | | ATAUX2-27, AUX2-27, IAA5, indole-3-acetic acid inducible 5 | 86.7121 | 10.1769 | | -3.0909 |
| AT3G26520 | | GAMMA-TIP2, SITIP, TIP1;2, TIP2, tonoplast intrinsic protein 2 | 1343.92 | 157.822 | | -3.0901 |
| AT1G52155 | | unknown protein | 24.9453 | 2.95257 | | -3.0787 |
| AT1G69900 | | Actin cross-linking protein | 26.4183 | 3.13205 | | -3.0764 |
| AT4G01350 | | Cysteine/Histidine-rich C1 domain family protein | 13.2072 | 1.56638 | | -3.0758 |
| AT3G09710 | | IQD1, IQ-domain 1 | 47.9049 | 5.68657 | | -3.0745 |
| AT1G10020 | | Protein of unknown function (DUF1005) | 19.4171 | 2.30678 | | -3.0734 |
| AT5G49900 | | Beta-glucosidase, GBA2 type family protein | 67.753 | 8.07035 | | -3.0696 |
| AT5G11920 | | AtcwINV6, cwINV6, 6-&1-fructan exohydrolase | 49.1181 | 5.8747 | | -3.0637 |
| AT5G01550 | | LECRKA4.2, lectin receptor kinase a4.1 | 1.39997 | 0.167486 | | -3.0633 |
| AT1G18773 | | function unknown | 2.89381 | 0.347053 | | -3.0597 |
| AT3G59340 | | Eukaryotic protein of unknown function (DUF914) | 12.9469 | 1.55281 | | -3.0597 |
| AT4G01480 | | AtPPa5, PPa5, pyrophosphorylase 5 | 1274.5 | 153.007 | | -3.0583 |
| AT1G80440 | | Galactose oxidase/kelch repeat superfamily protein | 125.421 | 15.0842 | | -3.0557 |
| AT3G06990 | | Cysteine/Histidine-rich C1 domain family protein | 6.72998 | 0.811217 | | -3.0524 |
| AT5G45116 | | transposable element gene | 7.23456 | 0.873391 | | -3.0502 |
| AT5G45180 | | Flavin-binding monooxygenase family protein | 1.30539 | 0.157622 | | -3.0499 |
| AT2G24320 | | alpha/beta-Hydrolases superfamily protein | 9.39367 | 1.13674 | | -3.0468 |
| AT1G22500 | | RING/U-box superfamily protein | 44.0959 | 5.34257 | | -3.045 |
| AT5G11110 | | ATSPS2F, KNS2, SPS1, SPS2F, sucrose phosphate synthase 2F | 412.702 | 50.0113 | | -3.0448 |
| AT2G43820 | | ATSAGT1, GT, SAGT1, SGT1, UGT74F2, UDP-glucosyltransferase 74F2 | 633.66 | 77.0631 | | -3.0396 |
| AT3G58810 | | ATMTP3, ATMTPA2, MTP3, MTPA2, metal tolerance protein A2 | 10.4308 | 1.2688 | | -3.0393 |
| AT3G28310 | | Protein of unknown function (DUF677) | 449.724 | 54.7267 | | -3.0387 |
| AT1G72430 | | SAUR-like auxin-responsive protein family | 29.7269 | 3.62723 | | -3.0348 |
| AT1G13510 | | Protein of unknown function (DUF1262) | 6.14524 | 0.750605 | | -3.0333 |
| AT4G31020 | | alpha/beta-Hydrolases superfamily protein | 11.5297 | 1.41059 | | -3.031 |
| AT2G47650 | | UXS4, UDP-xylose synthase 4 | 751.056 | 92.2069 | | -3.026 |
| AT3G14470 | | NB-ARC domain-containing disease resistance protein | 18.4803 | 2.26929 | | -3.0257 |
| AT1G12950 | | RSH2, root hair specific 2 | 383.267 | 47.3697 | | -3.0163 |
| AT1G13300 | | HRS1, myb-like transcription factor family protein | 24.5871 | 3.04804 | | -3.012 |
| AT3G12700 | | Eukaryotic aspartyl protease family protein | 75.0427 | 9.33747 | | -3.0066 |
| AT4G35060 | | Heavy metal transport/detoxification superfamily protein | 30.5191 | 3.80277 | | -3.0046 |
| AT5G16900 | | Leucine-rich repeat protein kinase family protein | 24.4234 | 3.05334 | | -2.9998 |
| AT2G32270 | | ZIP3, zinc transporter 3 precursor | 165.322 | 20.7386 | | -2.9949 |
| AT3G07880 | | SCN1, Immunoglobulin E-set superfamily protein | 469.668 | 58.949 | | -2.9941 |
| AT2G20880 | | Integrase-type DNA-binding superfamily protein | 165.331 | 20.7599 | | -2.9935 |
| AT4G04410 | | transposable element gene | 1.00098 | 0.125942 | | -2.9906 |
| AT3G53150 | | UGT73D1, UDP-glucosyl transferase 73D1 | 47.678 | 6.00347 | | -2.9895 |
| AT5G44260 | | Zinc finger C-x8-C-x5-C-x3-H type family protein | 5.50629 | 0.696468 | | -2.983 |
| AT2G38060 | | PHT4;2, phosphate transporter 4;2 | 5.49993 | 0.696335 | | -2.9816 |
| AT2G04170 | | TRAF-like family protein | 168.974 | 21.5401 | | -2.9717 |
| AT1G74940 | | Protein of unknown function (DUF581) | 61.2174 | 7.83036 | | -2.9668 |
| AT1G66860 | | Class I glutamine amidotransferase-like superfamily protein | 14.4694 | 1.85397 | | -2.9643 |
| AT3G45710 | | Major facilitator superfamily protein | 3.20043 | 0.410486 | | -2.9629 |
| AT2G48080 | | oxidoreductase, 2OG-Fe(II) oxygenase family protein | 32.817 | 4.20925 | | -2.9628 |
| AT2G44670 | | Protein of unknown function (DUF581) | 441.449 | 56.6394 | | -2.9624 |
| AT1G01120 | | KCS1, 3-ketoacyl-CoA synthase 1 | 82.8902 | 10.6765 | | -2.9568 |
| AT1G62850 | | Class I peptide chain release factor | 110.321 | 14.2257 | | -2.9551 |
| AT5G56870 | | BGAL4, beta-galactosidase 4 | 59.9873 | 7.7497 | | -2.9524 |
| AT5G18670 | | BAM9, BMY3, beta-amylase 3 | 274.994 | 35.5265 | | -2.9524 |
| AT3G27270 | | TRAM, LAG1 and CLN8 (TLC) lipid-sensing domain containing protein | 3.32571 | 0.430144 | | -2.9508 |
| AT5G08640 | | ATFLS1, FLS, FLS1, flavonol synthase 1 | 32.2254 | 4.1713 | | -2.9496 |
| AT4G18610 | | LSH9, Protein of unknown function (DUF640) | 34.7173 | 4.49472 | | -2.9494 |
| AT2G30395 | | ATOFP17, OFP17, ovate family protein 17 | 42.9151 | 5.56788 | | -2.9463 |
| AT3G01720 | | unknown protein | 115.849 | 15.0466 | | -2.9447 |
| AT3G60550 | | CYCP3;2, cyclin p3;2 | 107.833 | 14.0374 | | -2.9415 |
| AT3G07000 | | Cysteine/Histidine-rich C1 domain family protein | 15.1634 | 1.97575 | | -2.9401 |
| AT3G19030 | | unknown protein | 235.993 | 30.9055 | | -2.9328 |
| AT5G09610 | | APUM21, PUM21, pumilio 21 | 4.95062 | 0.648558 | | -2.9323 |
| AT4G31450 | | RING/U-box superfamily protein | 95.7907 | 12.5648 | | -2.9305 |
| AT1G22570 | | Major facilitator superfamily protein | 33.3471 | 4.37621 | | -2.9298 |
| AT4G03490 | | Ankyrin repeat family protein | 7.47103 | 0.985009 | | -2.9231 |
| AT1G16510 | | SAUR-like auxin-responsive protein family | 29.0176 | 3.83719 | | -2.9188 |
| AT5G54020 | | Cysteine/Histidine-rich C1 domain family protein | 2.89791 | 0.383735 | | -2.9168 |
| AT5G20820 | | SAUR-like auxin-responsive protein family | 56.7986 | 7.52724 | | -2.9157 |
| AT2G33280 | | Major facilitator superfamily protein | 1.6743 | 0.222025 | | -2.9148 |
| AT5G04238 | | unknown protein | 229.223 | 30.4009 | | -2.9146 |
| AT5G17810 | | WOX12, WUSCHEL related homeobox 12 | 9.42876 | 1.25121 | | -2.9137 |
| AT5G12000 | | Protein kinase protein with adenine nucleotide alpha hydrolases-like domain | 4.96377 | 0.659195 | | -2.9127 |
| AT2G26290 | | ARSK1, root-specific kinase 1 | 119.218 | 15.8924 | | -2.9072 |
| AT2G39360 | | Protein kinase superfamily protein | 26.5529 | 3.54205 | | -2.9062 |
| AT2G19130 | | S-locus lectin protein kinase family protein | 2.53099 | 0.338059 | | -2.9044 |
| AT5G42460 | | F-box and associated interaction domains-containing protein | 10.5154 | 1.40553 | | -2.9033 |
| AT1G75360 | | unknown protein | 2.17262 | 0.290653 | | -2.9021 |
| AT4G06744 | | Leucine-rich repeat (LRR) family protein | 5.34157 | 0.714639 | | -2.902 |
| AT5G42830 | | HXXXD-type acyl-transferase family protein | 22.7574 | 3.04995 | | -2.8995 |
| AT4G30140 | | CDEF1, GDSL-like Lipase/Acylhydrolase superfamily protein | 23.5062 | 3.15126 | | -2.899 |
| AT5G26010 | | Protein phosphatase 2C family protein | 60.2823 | 8.08689 | | -2.8981 |
| AT4G04760 | | Major facilitator superfamily protein | 5.37359 | 0.722476 | | -2.8949 |
| AT5G14760 | | AO, L-aspartate oxidase | 11.158 | 1.50731 | | -2.888 |
| AT3G62850 | | zinc finger protein-related | 1.97074 | 0.266734 | | -2.8853 |
| AT2G32510 | | MAPKKK17, mitogen-activated protein kinase kinase kinase 17 | 67.5074 | 9.13729 | | -2.8852 |
| AT3G56240 | | CCH, copper chaperone | 224.175 | 30.4103 | | -2.882 |
| AT1G04110 | | SDD1, Subtilase family protein | 2.07955 | 0.28225 | | -2.8812 |
| AT4G05330 | | AGD13, ARF-GAP domain 13 | 38.9895 | 5.29924 | | -2.8792 |
| AT5G42840 | | Cysteine/Histidine-rich C1 domain family protein | 4.03071 | 0.552239 | | -2.8677 |
| AT2G04130 | | transposable element gene | 4.2392 | 0.581482 | | -2.866 |
| AT1G10550 | | XET, XTH33, xyloglucan:xyloglucosyl transferase 33 | 2.14146 | 0.293809 | | -2.8657 |
| AT2G24260 | | LRL1, LJRHL1-like 1 | 64.1733 | 8.81101 | | -2.8646 |
| AT1G30860 | | RING/U-box superfamily protein | 6.42225 | 0.883213 | | -2.8623 |
| AT5G16540 | | ZFN3, zinc finger nuclease 3 | 14.8324 | 2.04106 | | -2.8614 |
| AT5G04470 | | SIM, cyclin-dependent protein kinase inhibitors | 40.0035 | 5.50779 | | -2.8606 |
| AT1G44130 | | Eukaryotic aspartyl protease family protein | 4.01786 | 0.554995 | | -2.8559 |
| AT1G47480 | | alpha/beta-Hydrolases superfamily protein | 50.2463 | 6.95046 | | -2.8538 |
| AT3G25240 | | Protein of unknown function (DUF506) | 3.97439 | 0.550249 | | -2.8526 |
| AT4G13615 | | Uncharacterised protein family SERF | 772.4 | 107.044 | | -2.8512 |
| AT1G44090 | | ATGA20OX5, GA20OX5, gibberellin 20-oxidase 5 | 90.5283 | 12.5487 | | -2.8508 |
| AT4G33330 | | GUX2, PGSIP3, plant glycogenin-like starch initiation protein 3 | 1.16072 | 0.161244 | | -2.8477 |
| AT5G54130 | | Calcium-binding endonuclease/exonuclease/phosphatase family | 16.7033 | 2.32207 | | -2.8467 |
| AT1G07430 | | HAI2, highly ABA-induced PP2C gene 2 | 2.35077 | 0.326942 | | -2.846 |
| AT5G67520 | | APK4, adenosine-5'-phosphosulfate (APS) kinase 4 | 107.667 | 14.9742 | | -2.846 |
| AT3G01520 | | Adenine nucleotide alpha hydrolases-like superfamily protein | 191.549 | 26.7047 | | -2.8425 |
| AT5G45113 | | mitochondrial transcription termination factor-related / mTERF-related | 5.88293 | 0.820934 | | -2.8412 |
| AT2G42350 | | RING/U-box superfamily protein | 59.7442 | 8.36996 | | -2.8355 |
| AT3G22540 | | Protein of unknown function (DUF1677) | 27.0551 | 3.79169 | | -2.835 |
| AT5G60800 | | Heavy metal transport/detoxification superfamily protein | 97.0321 | 13.6142 | | -2.8334 |
| AT3G49520 | | F-box and associated interaction domains-containing protein | 3.02165 | 0.424061 | | -2.833 |
| AT1G22330 | | RNA-binding (RRM/RBD/RNP motifs) family protein | 15.1725 | 2.13332 | | -2.8303 |
| AT5G35580 | | Protein kinase superfamily protein | 22.5848 | 3.17853 | | -2.8289 |
| AT5G45500 | | RNI-like superfamily protein | 110.658 | 15.6216 | | -2.8245 |
| AT1G23090 | | AST91, SULTR3;3, sulfate transporter 91 | 10.368 | 1.46506 | | -2.8231 |
| AT5G38940 | | RmlC-like cupins superfamily protein | 46.6574 | 6.59934 | | -2.8217 |
| AT3G25930 | | Adenine nucleotide alpha hydrolases-like superfamily protein | 54.343 | 7.70837 | | -2.8176 |
| AT5G24030 | | SLAH3, SLAC1 homologue 3 | 59.1096 | 8.40522 | | -2.814 |
| AT1G18980 | | RmlC-like cupins superfamily protein | 27.905 | 3.97892 | | -2.8101 |
| AT3G49860 | | ARLA1B, ATARLA1B, ADP-ribosylation factor-like A1B | 18.318 | 2.61222 | | -2.8099 |
| AT1G70810 | | Calcium-dependent lipid-binding (CaLB domain) family protein | 54.8635 | 7.8275 | | -2.8092 |
| AT5G01100 | | O-fucosyltransferase family protein | 174.355 | 24.9128 | | -2.8071 |
| AT5G39760 | | AtHB23, HB23, homeobox protein 23 | 3.36572 | 0.481387 | | -2.8057 |
| AT5G55960 | | unknown protein | 75.9385 | 10.8671 | | -2.8049 |
| AT5G54510 | | DFL1, GH3.6, Auxin-responsive GH3 family protein | 101.655 | 14.5543 | | -2.8042 |
| AT2G35940 | | BLH1, EDA29, BEL1-like homeodomain 1 | 3.12393 | 0.448125 | | -2.8014 |
| AT4G22460 | | Bifunctional inhibitor/lipid-transfer protein/seed storage 2S albumin superfamily protein | 162.841 | 23.3611 | | -2.8013 |
| AT2G02680 | | Cysteine/Histidine-rich C1 domain family protein | 6.66328 | 0.956055 | | -2.8011 |
| AT1G10200 | | WLIM1, GATA type zinc finger transcription factor family protein | 120.914 | 17.3714 | | -2.7992 |
| AT1G30190 | | unknown protein | 15.7828 | 2.27015 | | -2.7975 |
| AT1G63580 | | Receptor-like protein kinase-related family protein | 37.164 | 5.35363 | | -2.7953 |
| AT1G02400 | | ATGA2OX4, ATGA2OX6, DTA1, GA2OX6, gibberellin 2-oxidase 6 | 92.8745 | 13.5141 | | -2.7808 |
| AT4G22980 | | function unknown | 1.68263 | 0.245126 | | -2.7791 |
| AT2G38320 | | TBL34, TRICHOME BIREFRINGENCE-LIKE 34 | 13.0348 | 1.89896 | | -2.7791 |
| AT1G28330 | | DRM1, DYL1, dormancy-associated protein-like 1 | 348.14 | 50.7583 | | -2.778 |
| AT1G70820 | | phosphoglucomutase, putative / glucose phosphomutase, putative | 1.24047 | 0.180959 | | -2.7772 |
| AT5G43620 | | Pre-mRNA cleavage complex II | 128.958 | 18.8136 | | -2.7771 |
| AT1G72800 | | RNA-binding (RRM/RBD/RNP motifs) family protein | 52.1081 | 7.61144 | | -2.7753 |
| AT1G22540 | | Major facilitator superfamily protein | 14.7275 | 2.15645 | | -2.7718 |
| AT3G29635 | | HXXXD-type acyl-transferase family protein | 13.4612 | 1.97546 | | -2.7685 |
| AT5G48770 | | Disease resistance protein (TIR-NBS-LRR class) family | 2.49246 | 0.366208 | | -2.7668 |
| AT2G25510 | | unknown protein | 32.3447 | 4.78721 | | -2.7563 |
| AT5G66040 | | STR16, sulfurtransferase protein 16 | 39.9519 | 5.92621 | | -2.7531 |
| AT4G14610 | | pseudogene, disease resistance protein (CC-NBS-LRR class) | 11.7875 | 1.74873 | | -2.7529 |
| AT5G24040 | | Protein of unknown function (DUF295) | 8.94661 | 1.32902 | | -2.751 |
| AT2G44450 | | BGLU15, beta glucosidase 15 | 288.944 | 42.9701 | | -2.7494 |
| AT1G02660 | | alpha/beta-Hydrolases superfamily protein | 58.9969 | 8.77953 | | -2.7484 |
| AT4G00700 | | C2 calcium/lipid-binding plant phosphoribosyltransferase family protein | 9.02293 | 1.34296 | | -2.7482 |
| AT2G44380 | | Cysteine/Histidine-rich C1 domain family protein | 18.7913 | 2.79727 | | -2.748 |
| AT5G41315 | | GL3, GL3, MYC6.2, basic helix-loop-helix (bHLH) DNA-binding superfamily protein | 9.02493 | 1.34671 | | -2.7445 |
| AT1G05330 | | unknown protein | 15.4411 | 2.30497 | | -2.744 |
| AT1G66170 | | MMD1, RING/FYVE/PHD zinc finger superfamily protein | 1.89059 | 0.283249 | | -2.7387 |
| AT3G53600 | | C2H2-type zinc finger family protein | 819.368 | 122.918 | | -2.7368 |
| AT5G07220 | | ATBAG3, BAG3, BCL-2-associated athanogene 3 | 263.913 | 39.6005 | | -2.7365 |
| AT3G22250 | | UDP-Glycosyltransferase superfamily protein | 25.7965 | 3.87234 | | -2.7359 |
| AT2G02630 | | Cysteine/Histidine-rich C1 domain family protein | 1.84969 | 0.277782 | | -2.7353 |
| AT4G23870 | | unknown protein | 6.33461 | 0.952756 | | -2.7331 |
| AT4G03500 | | Ankyrin repeat family protein | 16.2964 | 2.4529 | | -2.732 |
| AT3G62450 | | unknown protein | 221.769 | 33.3836 | | -2.7319 |
| AT1G01740 | | Protein kinase protein with tetratricopeptide repeat domain | 13.6531 | 2.05532 | | -2.7318 |
| AT4G18630 | | Protein of unknown function (DUF688) | 9.49518 | 1.4296 | | -2.7316 |
| AT1G23020 | | ATFRO3, FRO3, ferric reduction oxidase 3 | 21.1704 | 3.19104 | | -2.73 |
| AT1G23750 | | Nucleic acid-binding, OB-fold-like protein | 161.203 | 24.3002 | | -2.7298 |
| AT1G48670 | | auxin-responsive GH3 family protein | 1.29426 | 0.195272 | | -2.7286 |
| AT3G13650 | | Disease resistance-responsive (dirigent-like protein) family protein | 137.574 | 20.828 | | -2.7236 |
| AT4G06746 | | DEAR5, RAP2.9, related to AP2 9 | 144.094 | 21.8161 | | -2.7235 |
| AT4G01820 | | MDR3, PGP3, P-glycoprotein 3 | 3.15113 | 0.477195 | | -2.7232 |
| AT5G62100 | | ATBAG2, BAG2, BCL-2-associated athanogene 2 | 2.66142 | 0.4036 | | -2.7212 |
| AT1G56700 | | Peptidase C15, pyroglutamyl peptidase I-like | 135.873 | 20.6475 | | -2.7182 |
| AT1G18310 | | glycosyl hydrolase family 81 protein | 1.07641 | 0.163659 | | -2.7175 |
| AT2G21430 | | Papain family cysteine protease | 4.37994 | 0.666511 | | -2.7162 |
| AT3G20410 | | CPK9, calmodulin-domain protein kinase 9 | 213.485 | 32.5019 | | -2.7155 |
| AT3G03520 | | NPC3, non-specific phospholipase C3 | 109.385 | 16.6552 | | -2.7154 |
| AT3G41762 | | unknown protein | 31.5833 | 4.81253 | | -2.7143 |
| AT5G23220 | | NIC3, nicotinamidase 3 | 203.627 | 31.0288 | | -2.7143 |
| AT5G60060 | | Protein of unknown function (DUF295) | 11.0925 | 1.69081 | | -2.7138 |
| AT5G45520 | | Leucine-rich repeat (LRR) family protein | 1.26185 | 0.19259 | | -2.7119 |
| AT3G23600 | | alpha/beta-Hydrolases superfamily protein | 655.149 | 100.14 | | -2.7098 |
| AT5G01830 | | ARM repeat superfamily protein | 85.2762 | 13.036 | | -2.7096 |
| AT4G03440 | | Ankyrin repeat family protein | 9.42261 | 1.44462 | | -2.7054 |
| AT1G76080 | | ATCDSP32, CDSP32, chloroplastic drought-induced stress protein of 32 kD | 9.66811 | 1.48445 | | -2.7033 |
| AT2G26480 | | UGT76D1, UDP-glucosyl transferase 76D1 | 54.487 | 8.38316 | | -2.7004 |
| AT4G16563 | | Eukaryotic aspartyl protease family protein | 7.0778 | 1.08938 | | -2.6998 |
| AT1G56660 | | unknown protein | 519.348 | 80.1407 | | -2.6961 |
| AT4G04700 | | CPK27, calcium-dependent protein kinase 27 | 16.0592 | 2.47984 | | -2.6951 |
| AT5G41300 | | Receptor-like protein kinase-related family protein | 11.2169 | 1.7369 | | -2.6911 |
| AT2G02960 | | RING/FYVE/PHD zinc finger superfamily protein | 272.174 | 42.1596 | | -2.6906 |
| AT3G26470 | | Powdery mildew resistance protein, RPW8 domain | 6.09926 | 0.945453 | | -2.6896 |
| AT1G79270 | | ECT8, evolutionarily conserved C-terminal region 8 | 122.041 | 18.9737 | | -2.6853 |
| AT5G27930 | | Protein phosphatase 2C family protein | 96.7972 | 15.0631 | | -2.684 |
| AT5G52900 | | unknown protein | 3.78403 | 0.589383 | | -2.6827 |
| AT4G10500 | | 2-oxoglutarate (2OG) and Fe(II)-dependent oxygenase superfamily protein | 16.0491 | 2.50061 | | -2.6821 |
| AT5G19240 | | Glycoprotein membrane precursor GPI-anchored | 61.5293 | 9.59602 | | -2.6808 |
| AT4G37010 | | CEN2, centrin 2 | 122.131 | 19.1228 | | -2.6751 |
| AT2G46940 | | unknown protein | 24.4941 | 3.83807 | | -2.674 |
| AT5G48657 | | defense protein-related | 107.606 | 16.8773 | | -2.6726 |
| AT2G05910 | | Protein of unknown function (DUF567) | 6.75198 | 1.05939 | | -2.6721 |
| AT2G23340 | | DEAR3, DREB and EAR motif protein 3 | 58.0188 | 9.1049 | | -2.6718 |
| AT3G16690 | | Nodulin MtN3 family protein | 119.137 | 18.8178 | | -2.6625 |
| AT3G23800 | | SBP3, selenium-binding protein 3 | 29.2415 | 4.6188 | | -2.6624 |
| AT1G35670 | | ATCDPK2, ATCPK11, CDPK2, CPK11, calcium-dependent protein kinase 2 | 322.302 | 51.0252 | | -2.6591 |
| AT3G04630 | | WDL1, WVD2-like 1 | 90.8727 | 14.4064 | | -2.6571 |
| AT4G12410 | | SAUR-like auxin-responsive protein family | 8.42949 | 1.33892 | | -2.6544 |
| AT1G75840 | | ARAC5, ATGP3, ATROP4, ROP4, RAC-like GTP binding protein 5 | 85.6157 | 13.6596 | | -2.648 |
| AT4G27410 | | ANAC072, RD26, NAC (No Apical Meristem) domain transcriptional regulator superfamily protein | 201.019 | 32.0744 | | -2.6478 |
| AT2G44790 | | UCC2, uclacyanin 2 | 1170.14 | 186.929 | | -2.6461 |
| AT3G52740 | | unknown protein | 75.4654 | 12.0904 | | -2.642 |
| AT1G64530 | | Plant regulator RWP-RK family protein | 77.2329 | 12.3854 | | -2.6406 |
| AT3G14370 | | WAG2, Protein kinase superfamily protein | 55.5131 | 8.93205 | | -2.6358 |
| AT5G65930 | | KCBP, PKCBP, ZWI, kinesin-like calmodulin-binding protein (ZWICHEL) | 88.8466 | 14.3017 | | -2.6351 |
| AT5G12340 | | unknown protein | 223.717 | 36.0318 | | -2.6343 |
| AT2G17845 | | NAD(P)-binding Rossmann-fold superfamily protein | 14.4517 | 2.33067 | | -2.6324 |
| AT2G01430 | | ATHB-17, ATHB17, HB17, homeobox-leucine zipper protein 17 | 4.00225 | 0.64577 | | -2.6317 |
| AT2G33830 | | Dormancy/auxin associated family protein | 144.465 | 23.3838 | | -2.6271 |
| AT4G34750 | | SAUR-like auxin-responsive protein family | 16.9547 | 2.74517 | | -2.6267 |
| AT3G61270 | | Arabidopsis thaliana protein of unknown function (DUF821) | 27.2833 | 4.41893 | | -2.6263 |
| AT5G57740 | | XBAT32, XB3 ortholog 2 in Arabidopsis thaliana | 46.6273 | 7.55527 | | -2.6256 |
| AT1G60130 | | Mannose-binding lectin superfamily protein | 4.26065 | 0.690922 | | -2.6245 |
| AT4G14465 | | AHL20, AT-hook motif nuclear-localized protein 20 | 17.7409 | 2.88216 | | -2.6219 |
| AT4G28150 | | Protein of unknown function (DUF789) | 5.67665 | 0.922664 | | -2.6212 |
| AT3G59060 | | PIF5, PIL6, phytochrome interacting factor 3-like 6 | 1.19621 | 0.19472 | | -2.619 |
| AT4G18940 | | RNA ligase/cyclic nucleotide phosphodiesterase family protein | 21.8767 | 3.56177 | | -2.6187 |
| AT5G20260 | | Exostosin family protein | 20.7634 | 3.38258 | | -2.6179 |
| AT4G30980 | | LRL2, LJRHL1-like 2 | 18.4855 | 3.01509 | | -2.6161 |
| AT2G34830 | | AtWRKY35, MEE24, WRKY35, WRKY DNA-binding protein 35 | 28.5706 | 4.66111 | | -2.6158 |
| AT5G14120 | | Major facilitator superfamily protein | 80.9475 | 13.2065 | | -2.6157 |
| AT1G18860 | | ATWRKY61, WRKY61, WRKY DNA-binding protein 61 | 71.9443 | 11.7393 | | -2.6155 |
| AT3G62780 | | Calcium-dependent lipid-binding (CaLB domain) family protein | 4.07743 | 0.665696 | | -2.6147 |
| AT4G19950 | | unknown protein | 2.22853 | 0.364077 | | -2.6138 |
| AT3G16470 | | JR1, Mannose-binding lectin superfamily protein | 14.2405 | 2.32649 | | -2.6138 |
| AT3G61260 | | Remorin family protein | 418.234 | 68.3455 | | -2.6134 |
| AT1G66160 | | ATCMPG1, CMPG1, CYS, MET, PRO, and GLY protein 1 | 169.823 | 27.7535 | | -2.6133 |
| AT4G17100 | | CONTAINS InterPro DOMAIN/s: Endoribonuclease XendoU (InterPro:IPR018998) | 252.679 | 41.4201 | | -2.6089 |
| AT5G18680 | | AtTLP11, TLP11, tubby like protein 11 | 79.2469 | 12.9936 | | -2.6086 |
| AT2G37290 | | Ypt/Rab-GAP domain of gyp1p superfamily protein | 13.7737 | 2.26582 | | -2.6038 |
| AT5G66580 | | unknown protein | 83.2222 | 13.6914 | | -2.6037 |
| AT5G47250 | | LRR and NB-ARC domains-containing disease resistance protein | 10.4161 | 1.71412 | | -2.6033 |
| AT5G60660 | | PIP2;4, PIP2F, plasma membrane intrinsic protein 2;4 | 165.483 | 27.2862 | | -2.6004 |
| AT5G44770 | | Cysteine/Histidine-rich C1 domain family protein | 3.52784 | 0.58218 | | -2.5993 |
| AT3G52460 | | hydroxyproline-rich glycoprotein family protein | 35.3533 | 5.83853 | | -2.5982 |
| AT5G01840 | | ATOFP1, OFP1, ovate family protein 1 | 6.58961 | 1.08844 | | -2.5979 |
| AT3G19390 | | Granulin repeat cysteine protease family protein | 1148.84 | 190.392 | | -2.5931 |
| AT2G37750 | | unknown protein | 194.203 | 32.2008 | | -2.5924 |
| AT3G19920 | | unknown protein | 7.31043 | 1.21619 | | -2.5876 |
| AT4G14580 | | CIPK4, SnRK3.3, CBL-interacting protein kinase 4 | 1.64706 | 0.274195 | | -2.5866 |
| AT4G29210 | | GGT3, GGT4, gamma-glutamyl transpeptidase 4 | 41.2656 | 6.87892 | | -2.5847 |
| AT2G25260 | | unknown protein | 70.7576 | 11.7957 | | -2.5846 |
| AT1G22830 | | Tetratricopeptide repeat (TPR)-like superfamily protein | 46.7237 | 7.80635 | | -2.5814 |
| AT5G41830 | | RNI-like superfamily protein | 3.40181 | 0.568359 | | -2.5814 |
| AT2G39518 | | Uncharacterised protein family (UPF0497) | 84.8301 | 14.1797 | | -2.5808 |
| AT5G47980 | | HXXXD-type acyl-transferase family protein | 23.9769 | 4.01052 | | -2.5798 |
| AT3G15518 | | unknown protein | 52.0407 | 8.70883 | | -2.5791 |
| AT5G51160 | | Ankyrin repeat family protein | 28.485 | 4.77672 | | -2.5761 |
| AT4G33050 | | EDA39, calmodulin-binding family protein | 7.54189 | 1.26527 | | -2.5755 |
| AT2G29730 | | UGT71D1, UDP-glucosyl transferase 71D1 | 14.7789 | 2.48924 | | -2.5698 |
| AT5G19220 | | ADG2, APL1, ADP glucose pyrophosphorylase large subunit 1 | 20.5738 | 3.47471 | | -2.5658 |
| AT4G12690 | | Plant protein of unknown function (DUF868) | 7.12832 | 1.2062 | | -2.5631 |
| AT5G01640 | | PRA1.B5, prenylated RAB acceptor 1.B5 | 41.2527 | 6.98327 | | -2.5625 |
| AT4G29550 | | Protein of unknown function (DUF626) | 4.15822 | 0.704024 | | -2.5623 |
| AT4G11300 | | Protein of unknown function (DUF793) | 6.62397 | 1.12289 | | -2.5605 |
| AT1G75410 | | BLH3, BEL1-like homeodomain 3 | 16.2989 | 2.76465 | | -2.5596 |
| AT5G13500 | | unknown protein | 217.879 | 36.9633 | | -2.5594 |
| AT2G38090 | | Duplicated homeodomain-like superfamily protein | 14.1479 | 2.40107 | | -2.5588 |
| AT5G36260 | | Eukaryotic aspartyl protease family protein | 15.322 | 2.60715 | | -2.5551 |
| AT3G47820 | | PUB39, PLANT U-BOX 39 | 10.8078 | 1.83904 | | -2.5551 |
| AT1G63530 | | BEST Arabidopsis thaliana protein match is: hydroxyproline-rich glycoprotein family protein (TAIR:AT1G63540.1) | 19.735 | 3.35862 | | -2.5548 |
| AT5G13320 | | GDG1, GH3.12, PBS3, WIN3, Auxin-responsive GH3 family protein | 1.20584 | 0.205598 | | -2.5521 |
| AT1G63540 | | hydroxyproline-rich glycoprotein family protein | 4.22751 | 0.720945 | | -2.5519 |
| AT3G11773 | | Thioredoxin superfamily protein | 11.4881 | 1.9608 | | -2.5506 |
| AT1G61560 | | ATMLO6, MLO6, Seven transmembrane MLO family protein | 111.56 | 19.044 | | -2.5504 |
| AT5G62470 | | ATMYB96, MYB96, MYBCOV1, myb domain protein 96 | 2.05094 | 0.3503 | | -2.5496 |
| AT5G57190 | | PSD2, phosphatidylserine decarboxylase 2 | 14.9324 | 2.55125 | | -2.5492 |
| AT5G02400 | | PLL2, pol-like 2 | 4.77503 | 0.816618 | | -2.5478 |
| AT5G64510 | | unknown protein | 86.0094 | 14.7539 | | -2.5434 |
| AT4G36120 | | Plant protein of unknown function (DUF869) | 13.811 | 2.36961 | | -2.5431 |
| AT4G13420 | | ATHAK5, HAK5, high affinity K+ transporter 5 | 9.83467 | 1.68897 | | -2.5417 |
| AT1G62975 | | basic helix-loop-helix (bHLH) DNA-binding superfamily protein | 36.3364 | 6.26109 | | -2.5369 |
| AT2G04430 | | atnudt5, NUDT5, nudix hydrolase homolog 5 | 32.2966 | 5.58723 | | -2.5312 |
| AT5G38030 | | MATE efflux family protein | 1.72729 | 0.299381 | | -2.5285 |
| AT1G72900 | | Toll-Interleukin-Resistance (TIR) domain-containing protein | 26.361 | 4.57568 | | -2.5264 |
| AT3G09280 | | unknown protein | 260.201 | 45.2117 | | -2.5249 |
| AT3G20460 | | Major facilitator superfamily protein | 31.5123 | 5.47693 | | -2.5245 |
| AT5G15950 | | Adenosylmethionine decarboxylase family protein | 195.241 | 34.0288 | | -2.5204 |
| AT2G37000 | | TCP family transcription factor | 3.17257 | 0.553102 | | -2.52 |
| AT5G45000 | | Disease resistance protein (TIR-NBS-LRR class) family | 16.0892 | 2.80833 | | -2.5183 |
| AT5G21170 | | AKINBETA1, 5'-AMP-activated protein kinase beta-2 subunit protein | 104.002 | 18.1796 | | -2.5162 |
| AT5G04820 | | ATOFP13, OFP13, ovate family protein 13 | 22.7576 | 3.98028 | | -2.5154 |
| AT1G79910 | | Regulator of Vps4 activity in the MVB pathway protein | 29.603 | 5.17834 | | -2.5152 |
| AT5G46520 | | Disease resistance protein (TIR-NBS-LRR class) family | 1.16053 | 0.203355 | | -2.5127 |
| AT3G13700 | | RNA-binding (RRM/RBD/RNP motifs) family protein | 21.7998 | 3.82168 | | -2.512 |
| AT5G13548 | | Pseudogene of AT3G12600; ATNUDT16 (Arabidopsis thaliana Nudix hydrolase homolog 16); hydrolase | 219.121 | 38.4399 | | -2.5111 |
| AT3G07870 | | F-box and associated interaction domains-containing protein | 56.2917 | 9.87512 | | -2.5111 |
| AT4G19960 | | ATKUP9, HAK9, KT9, KUP9, K+ uptake permease 9 | 15.3728 | 2.69787 | | -2.5105 |
| AT5G43780 | | APS4, Pseudouridine synthase/archaeosine transglycosylase-like family protein | 148.717 | 26.1097 | | -2.5099 |
| AT5G22400 | | Rho GTPase activating protein with PAK-box/P21-Rho-binding domain | 42.9908 | 7.55046 | | -2.5094 |
| AT1G08340 | | Rho GTPase activating protein with PAK-box/P21-Rho-binding domain | 106.051 | 18.6353 | | -2.5087 |
| AT1G14540 | | Peroxidase superfamily protein | 35.4839 | 6.23932 | | -2.5077 |
| AT1G19200 | | Protein of unknown function (DUF581) | 87.7559 | 15.4307 | | -2.5077 |
| AT4G29220 | | PFK1, phosphofructokinase 1 | 59.1846 | 10.4152 | | -2.5065 |
| AT3G54990 | | SMZ, Integrase-type DNA-binding superfamily protein | 48.1095 | 8.4719 | | -2.5056 |
| AT2G17130 | | IDH-II, IDH2, isocitrate dehydrogenase subunit 2 | 211.379 | 37.2411 | | -2.5049 |
| AT5G02630 | | Lung seven transmembrane receptor family protein | 2.62401 | 0.462396 | | -2.5046 |
| AT5G46060 | | Protein of unknown function, DUF599 | 16.0353 | 2.83077 | | -2.502 |
| AT2G30040 | | MAPKKK14, mitogen-activated protein kinase kinase kinase 14 | 125.014 | 22.104 | | -2.4997 |
| AT2G20950 | | Arabidopsis phospholipase-like protein (PEARLI 4) family | 6.00665 | 1.06215 | | -2.4996 |
| AT5G36940 | | CAT3, cationic amino acid transporter 3 | 26.4842 | 4.69556 | | -2.4958 |
| AT1G79320 | | AtMC6, MC6, metacaspase 6 | 29.4424 | 5.22689 | | -2.4939 |
| AT3G20520 | | SVL3, SHV3-like 3 | 11.8833 | 2.10967 | | -2.4939 |
| AT3G13965 | | pseudogene, hypothetical protein | 19.7231 | 3.50236 | | -2.4935 |
| AT1G14170 | | RNA-binding KH domain-containing protein | 181.446 | 32.3096 | | -2.4895 |
| AT3G18060 | | transducin family protein / WD-40 repeat family protein | 143.205 | 25.5061 | | -2.4892 |
| AT5G24530 | | DMR6, 2-oxoglutarate (2OG) and Fe(II)-dependent oxygenase superfamily protein | 81.4499 | 14.5107 | | -2.4888 |
| AT2G19760 | | PFN1, PRF1, profilin 1 | 609.899 | 108.766 | | -2.4873 |
| AT4G05150 | | Octicosapeptide/Phox/Bem1p family protein | 228.29 | 40.7379 | | -2.4864 |
| AT5G47230 | | ATERF-5, ATERF5, ERF5, ethylene responsive element binding factor 5 | 136.589 | 24.3925 | | -2.4853 |
| AT3G18200 | | nodulin MtN21 /EamA-like transporter family protein | 80.5179 | 14.3929 | | -2.484 |
| AT5G44020 | | HAD superfamily, subfamily IIIB acid phosphatase | 1990.44 | 355.83 | | -2.4838 |
| AT2G45210 | | SAUR-like auxin-responsive protein family | 3.50308 | 0.626337 | | -2.4836 |
| AT4G00955 | | function unknown | 5.71203 | 1.02502 | | -2.4784 |
| AT3G21230 | | 4CL5, 4-coumarate:CoA ligase 5 | 37.0357 | 6.6544 | | -2.4765 |
| AT2G26570 | | Plant protein of unknown function (DUF827) | 46.5513 | 8.36438 | | -2.4765 |
| AT3G60980 | | Tetratricopeptide repeat (TPR)-like superfamily protein | 66.5243 | 11.9634 | | -2.4753 |
| AT3G20830 | | AGC (cAMP-dependent, cGMP-dependent and protein kinase C) kinase family protein | 11.4152 | 2.05503 | | -2.4737 |
| AT5G06300 | | Putative lysine decarboxylase family protein | 683.151 | 123.097 | | -2.4724 |
| AT4G26850 | | VTC2 | 32.0837 | 5.78333 | | -2.4719 |
| AT2G17880 | | Chaperone DnaJ-domain superfamily protein | 45.2704 | 8.16076 | | -2.4718 |
| AT2G27505 | | FBD-like domain family protein | 7.27167 | 1.31178 | | -2.4708 |
| AT4G35783 | | DVL17, RTFL6, ROTUNDIFOLIA like 6 | 22.4939 | 4.06662 | | -2.4676 |
| AT1G04445 | | C2H2-like zinc finger protein | 16.4878 | 2.98234 | | -2.4669 |
| AT1G23560 | | Domain of unknown function (DUF220) | 5.0527 | 0.914674 | | -2.4657 |
| AT4G10370 | | Cysteine/Histidine-rich C1 domain family protein | 17.9892 | 3.26055 | | -2.464 |
| AT5G05380 | | PRA1.B3, prenylated RAB acceptor 1.B3 | 27.8001 | 5.04465 | | -2.4623 |
| AT5G25250 | | SPFH/Band 7/PHB domain-containing membrane-associated protein family | 15.721 | 2.85528 | | -2.461 |
| AT1G17340 | | Phosphoinositide phosphatase family protein | 191.977 | 34.9336 | | -2.4583 |
| AT5G05400 | | LRR and NB-ARC domains-containing disease resistance protein | 6.73759 | 1.22751 | | -2.4565 |
| AT3G44900 | | ATCHX4, CHX4, cation/H+ exchanger 4 | 5.17185 | 0.942873 | | -2.4556 |
| AT4G17215 | | Pollen Ole e 1 allergen and extensin family protein | 22.3471 | 4.07523 | | -2.4551 |
| AT4G36880 | | CP1, cysteine proteinase1 | 192.723 | 35.1567 | | -2.4547 |
| AT3G02150 | | PTF1, TCP13, TFPD, plastid transcription factor 1 | 17.4453 | 3.18378 | | -2.454 |
| AT1G28370 | | ATERF11, ERF11, ERF domain protein 11 | 23.8071 | 4.35025 | | -2.4522 |
| AT3G27300 | | G6PD5, glucose-6-phosphate dehydrogenase 5 | 141.963 | 25.9497 | | -2.4517 |
| AT3G04880 | | DRT102, DNA-damage-repair/toleration protein (DRT102) | 79.6404 | 14.5778 | | -2.4497 |
| AT1G23030 | | ARM repeat superfamily protein | 51.4383 | 9.41957 | | -2.4491 |
| AT5G51780 | | basic helix-loop-helix (bHLH) DNA-binding superfamily protein | 27.1065 | 4.97286 | | -2.4465 |
| AT1G75960 | | AMP-dependent synthetase and ligase family protein | 19.483 | 3.57696 | | -2.4454 |
| AT5G02640 | | unknown protein | 3.99626 | 0.734672 | | -2.4435 |
| AT2G24330 | | Protein of unknown function (DUF2296) | 47.4154 | 8.73636 | | -2.4403 |
| AT1G18290 | | unknown protein | 13.7301 | 2.53331 | | -2.4382 |
| AT2G11280 | | pseudogene, hypothetical protein | 8.30551 | 1.53259 | | -2.4381 |
| AT4G02410 | | Concanavalin A-like lectin protein kinase family protein | 1.97336 | 0.36432 | | -2.4374 |
| AT3G49360 | | PGL2, 6-phosphogluconolactonase 2 | 4.68973 | 0.866203 | | -2.4367 |
| AT4G24310 | | Protein of unknown function (DUF679) | 67.2739 | 12.4374 | | -2.4354 |
| AT4G28030 | | Acyl-CoA N-acyltransferases (NAT) superfamily protein | 10.3257 | 1.91132 | | -2.4336 |
| AT5G17490 | | RGL3, RGA-like protein 3 | 12.5023 | 2.31459 | | -2.4334 |
| AT3G51470 | | Protein phosphatase 2C family protein | 10.1581 | 1.88337 | | -2.4312 |
| AT5G20885 | | RING/U-box superfamily protein | 99.3007 | 18.4252 | | -2.4301 |
| AT5G15630 | | COBL4, IRX6, COBRA-like extracellular glycosyl-phosphatidyl inositol-anchored protein family | 2.94826 | 0.547178 | | -2.4298 |
| AT4G40070 | | RING/U-box superfamily protein | 16.7292 | 3.12417 | | -2.4208 |
| AT1G12710 | | AtPP2-A12, PP2-A12, phloem protein 2-A12 | 51.9472 | 9.70666 | | -2.42 |
| AT1G77450 | | anac032, NAC032, NAC domain containing protein 32 | 205.205 | 38.3499 | | -2.4198 |
| AT2G30840 | | 2-oxoglutarate (2OG) and Fe(II)-dependent oxygenase superfamily protein | 2.45811 | 0.460706 | | -2.4156 |
| AT1G01680 | | ATPUB54, PUB54, plant U-box 54 | 14.0603 | 2.63551 | | -2.4155 |
| AT4G26690 | | GPDL2, MRH5, SHV3, PLC-like phosphodiesterase family protein | 215.115 | 40.482 | | -2.4098 |
| AT2G26440 | | Plant invertase/pectin methylesterase inhibitor superfamily | 4.43266 | 0.834946 | | -2.4084 |
| AT3G07570 | | Cytochrome b561/ferric reductase transmembrane with DOMON related domain | 73.5843 | 13.8761 | | -2.4068 |
| AT2G03980 | | GDSL-like Lipase/Acylhydrolase superfamily protein | 8.484 | 1.60043 | | -2.4063 |
| AT2G38080 | | ATLMCO4, IRX12, LAC4, LMCO4, Laccase/Diphenol oxidase family protein | 3.72969 | 0.703765 | | -2.4059 |
| AT1G62660 | | Glycosyl hydrolases family 32 protein | 426.585 | 80.577 | | -2.4044 |
| AT4G01120 | | ATBZIP54, GBF2, G-box binding factor 2 | 52.3565 | 9.89213 | | -2.404 |
| AT1G62440 | | LRX2, leucine-rich repeat/extensin 2 | 90.8058 | 17.1865 | | -2.4015 |
| AT4G04695 | | CPK31, calcium-dependent protein kinase 31 | 10.0239 | 1.89791 | | -2.401 |
| AT1G58270 | | ZW9, TRAF-like family protein | 251.666 | 47.6801 | | -2.4001 |
| AT4G33360 | | FLDH, NAD(P)-binding Rossmann-fold superfamily protein | 304.888 | 57.7994 | | -2.3992 |
| AT5G08240 | | unknown protein | 68.5301 | 13.01 | | -2.3971 |
| AT1G15680 | | F-box family protein | 1.77711 | 0.337676 | | -2.3958 |
| AT2G39570 | | ACT domain-containing protein | 208.337 | 39.6954 | | -2.3919 |
| AT5G43180 | | Protein of unknown function, DUF599 | 9.97131 | 1.90042 | | -2.3915 |
| AT3G46400 | | Leucine-rich repeat protein kinase family protein | 3.5756 | 0.681735 | | -2.3909 |
| AT3G47540 | | Chitinase family protein | 3.33021 | 0.634983 | | -2.3908 |
| AT4G36032 | | Potential natural antisense gene, locus overlaps with AT4G36030 | 32.5494 | 6.21787 | | -2.3881 |
| AT1G25460 | | NAD(P)-binding Rossmann-fold superfamily protein | 3.82986 | 0.731831 | | -2.3877 |
| AT1G68440 | | unknown protein | 821.724 | 157.151 | | -2.3865 |
| AT1G30640 | | Protein kinase family protein | 80.037 | 15.3104 | | -2.3862 |
| AT1G58360 | | AAP1, NAT2, amino acid permease 1 | 42.3092 | 8.09489 | | -2.3859 |
| AT2G22170 | | Lipase/lipooxygenase, PLAT/LH2 family protein | 444.232 | 85.2754 | | -2.3811 |
| AT4G15990 | | unknown protein | 79.088 | 15.1879 | | -2.3805 |
| AT1G72790 | | hydroxyproline-rich glycoprotein family protein | 72.7564 | 13.9791 | | -2.3798 |
| AT2G40420 | | Transmembrane amino acid transporter family protein | 40.1045 | 7.70715 | | -2.3795 |
| AT2G27080 | | Late embryogenesis abundant (LEA) hydroxyproline-rich glycoprotein family | 149.092 | 28.6707 | | -2.3786 |
| AT5G25890 | | IAA28, IAR2, indole-3-acetic acid inducible 28 | 174.637 | 33.5893 | | -2.3783 |
| AT4G26050 | | PIRL8, plant intracellular ras group-related LRR 8 | 13.5315 | 2.60432 | | -2.3774 |
| AT5G65990 | | Transmembrane amino acid transporter family protein | 74.4234 | 14.3419 | | -2.3755 |
| AT5G41710 | | transposable element gene | 8.58781 | 1.65892 | | -2.3721 |
| AT5G23750 | | Remorin family protein | 55.5246 | 10.7293 | | -2.3716 |
| AT5G17460 | | unknown protein | 280.23 | 54.1676 | | -2.3711 |
| AT3G28920 | | AtHB34, HB34, homeobox protein 34 | 9.49959 | 1.83695 | | -2.3706 |
| AT4G08040 | | ACS11, 1-aminocyclopropane-1-carboxylate synthase 11 | 5.301 | 1.02531 | | -2.3702 |
| AT1G80230 | | Rubredoxin-like superfamily protein | 240.118 | 46.4778 | | -2.3691 |
| AT4G15093 | | catalytic LigB subunit of aromatic ring-opening dioxygenase family | 131.876 | 25.5678 | | -2.3668 |
| AT3G15630 | | unknown protein | 848.819 | 164.601 | | -2.3665 |
| AT2G39110 | | Protein kinase superfamily protein | 285.64 | 55.4133 | | -2.3659 |
| AT4G33470 | | ATHDA14, hda14, histone deacetylase 14 | 5.09225 | 0.989774 | | -2.3631 |
| AT5G63595 | | ATFLS4, FLS4, flavonol synthase 4 | 4.64365 | 0.903461 | | -2.3617 |
| AT3G53370 | | S1FA-like DNA-binding protein | 40.7762 | 7.93863 | | -2.3608 |
| AT5G62865 | | unknown protein | 77.5281 | 15.0966 | | -2.3605 |
| AT4G13620 | | Integrase-type DNA-binding superfamily protein | 9.85908 | 1.91991 | | -2.3604 |
| AT1G21100 | | O-methyltransferase family protein | 6.22541 | 1.21403 | | -2.3584 |
| AT5G16910 | | ATCSLD2, CSLD2, cellulose-synthase like D2 | 315.453 | 61.5323 | | -2.358 |
| AT3G23090 | | TPX2 (targeting protein for Xklp2) protein family | 89.0892 | 17.3789 | | -2.3579 |
| AT1G30650 | | AR411, ATWRKY14, WRKY14, WRKY DNA-binding protein 14 | 47.0529 | 9.182 | | -2.3574 |
| AT1G30370 | | alpha/beta-Hydrolases superfamily protein | 2.57269 | 0.503002 | | -2.3546 |
| AT2G28200 | | C2H2-type zinc finger family protein | 19.3203 | 3.78208 | | -2.3529 |
| AT4G28640 | | IAA11, indole-3-acetic acid inducible 11 | 54.5393 | 10.6777 | | -2.3527 |
| AT5G44820 | | Nucleotide-diphospho-sugar transferase family protein | 44.0843 | 8.63482 | | -2.352 |
| AT3G24420 | | alpha/beta-Hydrolases superfamily protein | 15.8116 | 3.09828 | | -2.3514 |
| AT3G23810 | | ATSAHH2, SAHH2, S-adenosyl-l-homocysteine (SAH) hydrolase 2 | 38.717 | 7.60556 | | -2.3478 |
| AT3G05820 | | At-A/N-InvH, INVH, invertase H | 17.3463 | 3.40996 | | -2.3468 |
| AT4G25570 | | ACYB-2, Cytochrome b561/ferric reductase transmembrane protein family | 540.611 | 106.292 | | -2.3466 |
| AT5G27420 | | ATL31, CNI1, carbon/nitrogen insensitive 1 | 37.3679 | 7.35327 | | -2.3453 |
| AT3G13310 | | Chaperone DnaJ-domain superfamily protein | 901.908 | 177.623 | | -2.3442 |
| AT4G40020 | | Myosin heavy chain-related protein | 2.03292 | 0.400854 | | -2.3424 |
| AT1G02610 | | RING/FYVE/PHD zinc finger superfamily protein | 62.8885 | 12.4142 | | -2.3408 |
| AT1G51790 | | Leucine-rich repeat protein kinase family protein | 4.46373 | 0.881674 | | -2.3399 |
| AT4G01720 | | AtWRKY47, WRKY47, WRKY family transcription factor | 22.8129 | 4.51053 | | -2.3385 |
| AT3G13380 | | BRL3, BRI1-like 3 | 9.26606 | 1.83324 | | -2.3376 |
| AT3G15020 | | mMDH2, Lactate/malate dehydrogenase family protein | 125.129 | 24.775 | | -2.3365 |
| AT1G13110 | | CYP71B7, cytochrome P450, family 71 subfamily B, polypeptide 7 | 11.3656 | 2.25497 | | -2.3335 |
| AT5G03670 | | unknown protein | 32.2972 | 6.40875 | | -2.3333 |
| AT5G24165 | | unknown protein | 37.6647 | 7.48493 | | -2.3312 |
| AT3G29250 | | NAD(P)-binding Rossmann-fold superfamily protein | 104.045 | 20.6767 | | -2.3311 |
| AT3G14680 | | CYP72A14, cytochrome P450, family 72, subfamily A, polypeptide 14 | 2.79947 | 0.556603 | | -2.3304 |
| AT2G31020 | | ORP1A, OSBP(oxysterol binding protein)-related protein 1A | 71.1677 | 14.1562 | | -2.3298 |
| AT1G72300 | | Leucine-rich receptor-like protein kinase family protein | 8.64289 | 1.7199 | | -2.3292 |
| AT4G17905 | | ATL4H, RING/U-box superfamily protein | 11.0973 | 2.20958 | | -2.3284 |
| AT3G13810 | | AtIDD11, IDD11, indeterminate(ID)-domain 11 | 1.23961 | 0.24684 | | -2.3282 |
| AT2G18876 | | Afadin/alpha-actinin-binding protein | 46.7595 | 9.31223 | | -2.3281 |
| AT5G58730 | | pfkB-like carbohydrate kinase family protein | 190.387 | 38.1269 | | -2.3201 |
| AT5G60780 | | ATNRT2.3, NRT2.3, nitrate transporter 2.3 | 2.98772 | 0.598393 | | -2.3199 |
| AT4G37790 | | HAT22, Homeobox-leucine zipper protein family | 100.781 | 20.199 | | -2.3189 |
| AT5G52120 | | AtPP2-A14, PP2-A14, phloem protein 2-A14 | 5.31586 | 1.06545 | | -2.3188 |
| AT5G62920 | | ARR6, response regulator 6 | 130.832 | 26.2483 | | -2.3174 |
| AT1G64890 | | Major facilitator superfamily protein | 15.8609 | 3.18315 | | -2.317 |
| AT1G06840 | | Leucine-rich repeat protein kinase family protein | 69.5418 | 13.9755 | | -2.315 |
| AT1G35730 | | APUM9, PUM9, pumilio 9 | 1.99754 | 0.401597 | | -2.3144 |
| AT5G21050 | | unknown protein | 6.81651 | 1.37136 | | -2.3134 |
| AT5G59920 | | ULI3, Cysteine/Histidine-rich C1 domain family protein | 1.0775 | 0.216832 | | -2.313 |
| AT1G19210 | | Integrase-type DNA-binding superfamily protein | 11.4724 | 2.3099 | | -2.3123 |
| AT4G19220 | | Tetratricopeptide repeat (TPR)-like superfamily protein | 12.6786 | 2.56004 | | -2.3082 |
| AT1G11380 | | PLAC8 family protein | 45.9811 | 9.28869 | | -2.3075 |
| AT4G35985 | | Senescence/dehydration-associated protein-related | 137.36 | 27.7708 | | -2.3063 |
| AT1G78310 | | VQ motif-containing protein | 54.5883 | 11.0456 | | -2.3051 |
| AT1G64590 | | NAD(P)-binding Rossmann-fold superfamily protein | 6.00034 | 1.2144 | | -2.3048 |
| AT5G51060 | | ATRBOHC, RBOHC, RHD2, NADPH/respiratory burst oxidase protein D | 217.368 | 44.0457 | | -2.3031 |
| AT4G22760 | | Tetratricopeptide repeat (TPR)-like superfamily protein | 2.83829 | 0.575261 | | -2.3027 |
| AT5G58350 | | WNK4, ZIK2, with no lysine (K) kinase 4 | 26.97 | 5.48419 | | -2.298 |
| AT3G46690 | | UDP-Glycosyltransferase superfamily protein | 9.29009 | 1.89046 | | -2.297 |
| AT1G52342 | | unknown protein | 14.306 | 2.91217 | | -2.2965 |
| AT2G44010 | | unknown protein | 17.3172 | 3.52548 | | -2.2963 |
| AT3G09000 | | proline-rich family protein | 53.1497 | 10.8236 | | -2.2959 |
| AT2G04500 | | Cysteine/Histidine-rich C1 domain family protein | 7.30778 | 1.49049 | | -2.2937 |
| AT3G05990 | | Leucine-rich repeat (LRR) family protein | 136.921 | 27.9731 | | -2.2912 |
| AT1G16170 | | unknown protein | 82.7914 | 16.9436 | | -2.2887 |
| AT5G52300 | | LTI65, RD29B, CAP160 protein | 16.1364 | 3.30409 | | -2.288 |
| AT1G07000 | | ATEXO70B2, EXO70B2, exocyst subunit exo70 family protein B2 | 64.7368 | 13.2685 | | -2.2866 |
| AT1G12610 | | DDF1, Integrase-type DNA-binding superfamily protein | 55.0001 | 11.2748 | | -2.2863 |
| AT5G26740 | | Protein of unknown function (DUF300) | 132.408 | 27.153 | | -2.2858 |
| AT4G02170 | | unknown protein | 10.2176 | 2.09727 | | -2.2845 |
| AT5G22250 | | Polynucleotidyl transferase, ribonuclease H-like superfamily protein | 10.521 | 2.16107 | | -2.2835 |
| AT3G43430 | | RING/U-box superfamily protein | 123.129 | 25.2947 | | -2.2833 |
| AT3G13100 | | ATMRP7, MRP7, MRP7, multidrug resistance-associated protein 7 | 24.521 | 5.04793 | | -2.2803 |
| AT5G59540 | | 2-oxoglutarate (2OG) and Fe(II)-dependent oxygenase superfamily protein | 7.57002 | 1.55911 | | -2.2796 |
| AT1G30360 | | ERD4, Early-responsive to dehydration stress protein (ERD4) | 115.329 | 23.805 | | -2.2764 |
| AT3G46080 | | C2H2-type zinc finger family protein | 41.2372 | 8.51227 | | -2.2763 |
| AT3G01190 | | Peroxidase superfamily protein | 273.852 | 56.5778 | | -2.2751 |
| AT1G49030 | | PLAC8 family protein | 22.1289 | 4.57406 | | -2.2744 |
| AT5G60460 | | Preprotein translocase Sec, Sec61-beta subunit protein | 69.7808 | 14.4345 | | -2.2733 |
| AT3G46090 | | ZAT7, C2H2 and C2HC zinc fingers superfamily protein | 9.00391 | 1.86306 | | -2.2729 |
| AT4G21440 | | ATM4, ATMYB102, MYB102, MYB102, MYB-like 102 | 25.9516 | 5.37407 | | -2.2717 |
| AT5G55970 | | RING/U-box superfamily protein | 47.0115 | 9.74342 | | -2.2705 |
| AT5G05370 | | Cytochrome b-c1 complex, subunit 8 protein | 484.234 | 100.365 | | -2.2705 |
| AT1G76070 | | unknown protein | 159.589 | 33.0842 | | -2.2702 |
| AT5G19025 | | Ribosomal protein L34e superfamily protein | 103.039 | 21.3631 | | -2.27 |
| AT3G13760 | | Cysteine/Histidine-rich C1 domain family protein | 1.67958 | 0.348245 | | -2.2699 |
| AT5G55550 | | RNA-binding (RRM/RBD/RNP motifs) family protein | 34.1876 | 7.0897 | | -2.2697 |
| AT4G20860 | | FAD-binding Berberine family protein | 223.47 | 46.4183 | | -2.2673 |
| AT5G13200 | | GRAM domain family protein | 507.346 | 105.427 | | -2.2667 |
| AT1G22110 | | structural constituent of ribosome | 13.2423 | 2.75462 | | -2.2652 |
| AT4G37620 | | transposable element gene | 149.344 | 31.0778 | | -2.2647 |
| AT1G34750 | | Protein phosphatase 2C family protein | 62.4903 | 13.0327 | | -2.2615 |
| AT3G56090 | | ATFER3, FER3, ferritin 3 | 537.297 | 112.255 | | -2.2589 |
| AT1G31540 | | Disease resistance protein (TIR-NBS-LRR class) family | 13.9931 | 2.92638 | | -2.2575 |
| AT5G05340 | | Peroxidase superfamily protein | 13.2543 | 2.78239 | | -2.2521 |
| AT5G06570 | | alpha/beta-Hydrolases superfamily protein | 36.0666 | 7.58522 | | -2.2494 |
| AT5G52030 | | TraB family protein | 10.6033 | 2.23232 | | -2.2479 |
| AT3G15440 | | BEST Arabidopsis thaliana protein match is: RING/U-box superfamily protein (TAIR:AT3G15740.1) | 48.957 | 10.3117 | | -2.2472 |
| AT2G17650 | | AMP-dependent synthetase and ligase family protein | 4.55052 | 0.959102 | | -2.2463 |
| AT1G53330 | | Pentatricopeptide repeat (PPR) superfamily protein | 3.22577 | 0.679999 | | -2.246 |
| AT5G65600 | | Concanavalin A-like lectin protein kinase family protein | 1.56894 | 0.330751 | | -2.246 |
| AT2G27820 | | ADT3, PD1, prephenate dehydratase 1 | 111.43 | 23.5146 | | -2.2445 |
| AT5G37840 | | BEST Arabidopsis thaliana protein match is: plastid movement impaired 2 (TAIR:AT1G66480.1) | 4.52025 | 0.956007 | | -2.2413 |
| AT4G18010 | | 5PTASE2, AT5PTASE2, IP5PII, myo-inositol polyphosphate 5-phosphatase 2 | 17.3211 | 3.66368 | | -2.2412 |
| AT4G37340 | | CYP81D3, cytochrome P450, family 81, subfamily D, polypeptide 3 | 3.40692 | 0.721171 | | -2.2401 |
| AT5G35700 | | FIM2, fimbrin-like protein 2 | 104.208 | 22.0692 | | -2.2394 |
| AT3G14067 | | Subtilase family protein | 179.394 | 38.104 | | -2.2351 |
| AT3G27810 | | ATMYB21, ATMYB3, MYB21, myb domain protein 21 | 2.57032 | 0.546322 | | -2.2341 |
| AT1G10090 | | Early-responsive to dehydration stress protein (ERD4) | 39.1357 | 8.32378 | | -2.2332 |
| AT2G36210 | | SAUR-like auxin-responsive protein family | 12.9229 | 2.74903 | | -2.2329 |
| AT5G46870 | | RNA-binding (RRM/RBD/RNP motifs) family protein | 43.2856 | 9.2109 | | -2.2325 |
| AT1G15010 | | unknown protein | 758.503 | 161.428 | | -2.2323 |
| AT5G59170 | | Proline-rich extensin-like family protein | 2.61203 | 0.555923 | | -2.2322 |
| AT3G27650 | | LBD25, LOB domain-containing protein 25 | 4.11443 | 0.87623 | | -2.2313 |
| AT5G27150 | | AT-NHX1, ATNHX, ATNHX1, NHX1, Na+/H+ exchanger 1 | 179.064 | 38.1637 | | -2.2302 |
| AT2G12400 | | unknown protein | 231.808 | 49.4136 | | -2.23 |
| AT4G33666 | | unknown protein | 62.615 | 13.3773 | | -2.2267 |
| AT5G49760 | | Leucine-rich repeat protein kinase family protein | 50.3669 | 10.7752 | | -2.2248 |
| AT2G41730 | | unknown protein | 33.5927 | 7.18733 | | -2.2246 |
| AT4G19200 | | proline-rich family protein | 1242.91 | 266.046 | | -2.224 |
| AT1G12110 | | ATNRT1, B-1, CHL1, CHL1-1, NRT1, NRT1.1, nitrate transporter 1.1 | 95.1444 | 20.3747 | | -2.2233 |
| AT5G62210 | | Embryo-specific protein 3, (ATS3) | 4.0361 | 0.864379 | | -2.2232 |
| AT5G54160 | | ATOMT1, OMT1, O-methyltransferase 1 | 275.654 | 59.0608 | | -2.2226 |
| AT1G56540 | | Disease resistance protein (TIR-NBS-LRR class) family | 6.75452 | 1.44745 | | -2.2223 |
| AT2G42060 | | Cysteine/Histidine-rich C1 domain family protein | 5.72991 | 1.22976 | | -2.2201 |
| AT3G52400 | | ATSYP122, SYP122, syntaxin of plants 122 | 174.986 | 37.5873 | | -2.2189 |
| AT3G06300 | | AT-P4H-2, P4H isoform 2 | 269.199 | 57.8294 | | -2.2188 |
| AT1G67470 | | Protein kinase superfamily protein | 6.39511 | 1.37422 | | -2.2184 |
| AT2G36220 | | unknown protein | 316.413 | 68.0069 | | -2.2181 |
| AT1G61550 | | S-locus lectin protein kinase family protein | 6.01644 | 1.29779 | | -2.2129 |
| AT1G32170 | | XTH30, XTR4, xyloglucan endotransglucosylase/hydrolase 30 | 82.7574 | 17.8588 | | -2.2123 |
| AT4G20840 | | FAD-binding Berberine family protein | 86.0855 | 18.5794 | | -2.2121 |
| AT5G49850 | | Mannose-binding lectin superfamily protein | 2.38981 | 0.515843 | | -2.2119 |
| AT1G25400 | | unknown protein | 406.952 | 87.8463 | | -2.2118 |
| AT1G03950 | | VPS2.3, vacuolar protein sorting-associated protein 2.3 | 34.155 | 7.38174 | | -2.2101 |
| AT2G40330 | | PYL6, RCAR9, PYR1-like 6 | 5.41132 | 1.16989 | | -2.2096 |
| AT4G27270 | | Quinone reductase family protein | 66.3732 | 14.355 | | -2.2091 |
| AT1G32928 | | unknown protein | 182.732 | 39.5439 | | -2.2082 |
| AT5G03570 | | ATIREG2, FPN2, IREG2, iron regulated 2 | 3.00962 | 0.651364 | | -2.208 |
| AT1G20310 | | unknown protein | 82.778 | 17.9261 | | -2.2072 |
| AT5G21280 | | hydroxyproline-rich glycoprotein family protein | 91.1919 | 19.7542 | | -2.2068 |
| AT5G59150 | | ATRAB-A2D, ATRABA2D, RABA2D, RAB GTPase homolog A2D | 68.0582 | 14.7476 | | -2.2063 |
| AT5G46350 | | ATWRKY8, WRKY8, WRKY DNA-binding protein 8 | 38.8387 | 8.41764 | | -2.206 |
| AT1G78070 | | Transducin/WD40 repeat-like superfamily protein | 255.566 | 55.4027 | | -2.2057 |
| AT2G22790 | | unknown protein | 26.6624 | 5.7819 | | -2.2052 |
| AT4G13130 | | Cysteine/Histidine-rich C1 domain family protein | 8.21983 | 1.78453 | | -2.2036 |
| AT1G54500 | | Rubredoxin-like superfamily protein | 7.98526 | 1.73585 | | -2.2017 |
| AT1G49500 | | unknown protein | 8.178 | 1.77846 | | -2.2011 |
| AT1G19660 | | Wound-responsive family protein | 12.8064 | 2.78656 | | -2.2003 |
| AT1G50090 | | D-aminoacid aminotransferase-like PLP-dependent enzymes superfamily protein | 17.5034 | 3.8121 | | -2.199 |
| AT1G49860 | | ATGSTF14, GSTF14, glutathione S-transferase (class phi) 14 | 17.8026 | 3.87911 | | -2.1983 |
| AT3G59130 | | Cysteine/Histidine-rich C1 domain family protein | 8.86294 | 1.93403 | | -2.1962 |
| AT4G26970 | | ACO2, aconitase 2 | 501.061 | 109.825 | | -2.1898 |
| AT4G28630 | | ATATM1, ATM1, ABC transporter of the mitochondrion 1 | 8.19267 | 1.79713 | | -2.1886 |
| AT3G14350 | | SRF7, STRUBBELIG-receptor family 7 | 76.0462 | 16.6905 | | -2.1878 |
| AT5G49890 | | ATCLC-C, CLC-C, chloride channel C | 109.347 | 24.0093 | | -2.1872 |
| AT1G74930 | | ORA47, Integrase-type DNA-binding superfamily protein | 57.6123 | 12.6532 | | -2.1869 |
| AT5G15740 | | O-fucosyltransferase family protein | 59.7072 | 13.121 | | -2.186 |
| AT3G50830 | | ATCOR413-PM2, COR413-PM2, cold-regulated 413-plasma membrane 2 | 116.875 | 25.7024 | | -2.185 |
| AT2G31210 | | basic helix-loop-helix (bHLH) DNA-binding superfamily protein | 5.95861 | 1.31186 | | -2.1834 |
| AT1G74780 | | Nodulin-like / Major Facilitator Superfamily protein | 18.0087 | 3.96505 | | -2.1833 |
| AT4G18910 | | ATNLM2, NIP1;2, NLM2, NOD26-like intrinsic protein 1;2 | 9.94938 | 2.19134 | | -2.1828 |
| AT4G14368 | | Regulator of chromosome condensation (RCC1) family protein | 13.4937 | 2.97325 | | -2.1822 |
| AT2G47400 | | CP12, CP12-1, CP12 domain-containing protein 1 | 12.4594 | 2.74819 | | -2.1807 |
| AT3G45070 | | P-loop containing nucleoside triphosphate hydrolases superfamily protein | 11.9938 | 2.64868 | | -2.179 |
| AT3G26510 | | Octicosapeptide/Phox/Bem1p family protein | 26.4751 | 5.85085 | | -2.1779 |
| AT3G16510 | | Calcium-dependent lipid-binding (CaLB domain) family protein | 75.4123 | 16.69 | | -2.1758 |
| AT5G01720 | | RNI-like superfamily protein | 79.5281 | 17.6356 | | -2.173 |
| AT1G02310 | | MAN1, Glycosyl hydrolase superfamily protein | 42.4173 | 9.40736 | | -2.1728 |
| AT2G15960 | | unknown protein | 395.141 | 87.6931 | | -2.1718 |
| AT2G40110 | | Yippee family putative zinc-binding protein | 71.5415 | 15.8843 | | -2.1712 |
| AT4G13395 | | DVL10, RTFL12, ROTUNDIFOLIA like 12 | 366.356 | 81.3887 | | -2.1704 |
| AT2G42430 | | ASL18, LBD16, lateral organ boundaries-domain 16 | 13.3239 | 2.96005 | | -2.1703 |
| AT3G04530 | | ATPPCK2, PEPCK2, PPCK2, phosphoenolpyruvate carboxylase kinase 2 | 34.1546 | 7.5911 | | -2.1697 |
| AT3G25600 | | Calcium-binding EF-hand family protein | 149.32 | 33.1992 | | -2.1692 |
| AT4G28890 | | RING/U-box superfamily protein | 4.41765 | 0.982761 | | -2.1684 |
| AT3G51920 | | ATCML9, CAM9, CML9, calmodulin 9 | 152.785 | 34.0138 | | -2.1673 |
| AT1G01140 | | CIPK9, PKS6, SnRK3.12, CBL-interacting protein kinase 9 | 130.675 | 29.1015 | | -2.1668 |
| AT1G53920 | | GLIP5, GDSL-motif lipase 5 | 192.956 | 43.0331 | | -2.1648 |
| AT4G15400 | | HXXXD-type acyl-transferase family protein | 24.1973 | 5.41439 | | -2.16 |
| AT4G37990 | | ATCAD8, CAD-B2, ELI3, ELI3-2, elicitor-activated gene 3-2 | 46.579 | 10.4301 | | -2.1589 |
| AT4G37470 | | alpha/beta-Hydrolases superfamily protein | 9.12597 | 2.04398 | | -2.1586 |
| AT5G58160 | | actin binding | 34.973 | 7.83548 | | -2.1582 |
| AT4G18130 | | PHYE, phytochrome E | 40.1283 | 9.00403 | | -2.156 |
| AT5G42500 | | Disease resistance-responsive (dirigent-like protein) family protein | 6.0099 | 1.35197 | | -2.1523 |
| AT5G38850 | | Disease resistance protein (TIR-NBS-LRR class) | 12.3823 | 2.78548 | | -2.1523 |
| AT5G22540 | | Plant protein of unknown function (DUF247) | 2.63577 | 0.593463 | | -2.151 |
| AT3G19000 | | 2-oxoglutarate (2OG) and Fe(II)-dependent oxygenase superfamily protein | 3.47281 | 0.782038 | | -2.1508 |
| AT1G18150 | | ATMPK8, Protein kinase superfamily protein | 134.08 | 30.2339 | | -2.1489 |
| AT2G17070 | | Arabidopsis protein of unknown function (DUF241) | 10.9019 | 2.45931 | | -2.1483 |
| AT3G09540 | | Pectin lyase-like superfamily protein | 3.65896 | 0.825736 | | -2.1477 |
| AT4G32770 | | ATSDX1, VTE1, tocopherol cyclase, chloroplast / vitamin E deficient 1 (VTE1) / sucrose export defective 1 (SXD1) | 5.17269 | 1.16746 | | -2.1475 |
| AT4G12450 | | unknown protein | 26.7767 | 6.04734 | | -2.1466 |
| AT1G73510 | | unknown protein | 16.6562 | 3.76284 | | -2.1462 |
| AT1G23480 | | ATCSLA03, ATCSLA3, CSLA03, CSLA03, CSLA3, cellulose synthase-like A3 | 77.2241 | 17.4475 | | -2.146 |
| AT1G51850 | | Leucine-rich repeat protein kinase family protein | 15.024 | 3.40618 | | -2.141 |
| AT1G23870 | | ATTPS9, TPS9, TPS9, trehalose-phosphatase/synthase 9 | 356.963 | 80.9865 | | -2.14 |
| AT4G03480 | | Ankyrin repeat family protein | 1.44797 | 0.328597 | | -2.1396 |
| AT2G18690 | | unknown protein | 158.694 | 36.0347 | | -2.1388 |
| AT2G15555 | | other RNA | 12.679 | 2.88111 | | -2.1378 |
| AT1G50290 | | unknown protein | 117.154 | 26.6541 | | -2.136 |
| AT2G23960 | | Class I glutamine amidotransferase-like superfamily protein | 35.0038 | 7.96444 | | -2.1359 |
| AT5G51890 | | Peroxidase superfamily protein | 11.751 | 2.68105 | | -2.1319 |
| AT4G13340 | | Leucine-rich repeat (LRR) family protein | 56.7171 | 12.9646 | | -2.1292 |
| AT5G03360 | | DC1 domain-containing protein | 4.11148 | 0.940874 | | -2.1276 |
| AT1G65985 | | Plant protein of unknown function (DUF247) | 14.7657 | 3.37933 | | -2.1274 |
| AT4G01920 | | Cysteine/Histidine-rich C1 domain family protein | 3.67449 | 0.841037 | | -2.1273 |
| AT5G01732 | | other RNA | 14.6943 | 3.36436 | | -2.1269 |
| AT3G49820 | | unknown protein | 12.3158 | 2.82297 | | -2.1252 |
| AT1G24150 | | ATFH4, FH4, formin homologue 4 | 18.0755 | 4.14474 | | -2.1247 |
| AT4G27780 | | ACBP2, acyl-CoA binding protein 2 | 25.2476 | 5.79329 | | -2.1237 |
| AT5G42290 | | transcription activator-related | 45.5262 | 10.4498 | | -2.1232 |
| AT1G02220 | | ANAC003, NAC003, NAC domain containing protein 3 | 5.91975 | 1.35926 | | -2.1227 |
| AT4G26120 | | Ankyrin repeat family protein / BTB/POZ domain-containing protein | 2.73458 | 0.627948 | | -2.1226 |
| AT5G07470 | | ATMSRA3, PMSR3, peptidemethionine sulfoxide reductase 3 | 322.06 | 74.0045 | | -2.1216 |
| AT5G43330 | | Lactate/malate dehydrogenase family protein | 45.5838 | 10.4805 | | -2.1208 |
| AT4G37640 | | ACA2, calcium ATPase 2 | 161.434 | 37.1259 | | -2.1205 |
| AT4G39730 | | Lipase/lipooxygenase, PLAT/LH2 family protein | 479.997 | 110.412 | | -2.1201 |
| AT1G48240 | | ATNPSN12, NPSN12, novel plant snare 12 | 33.5463 | 7.72199 | | -2.1191 |
| AT5G19250 | | Glycoprotein membrane precursor GPI-anchored | 93.4861 | 21.5756 | | -2.1154 |
| AT5G56610 | | Phosphotyrosine protein phosphatases superfamily protein | 20.3032 | 4.68714 | | -2.1149 |
| AT3G49370 | | Calcium-dependent protein kinase (CDPK) family protein | 33.6561 | 7.78148 | | -2.1128 |
| AT3G01710 | | TPX2 (targeting protein for Xklp2) protein family | 9.91863 | 2.29465 | | -2.1119 |
| AT4G35380 | | SEC7-like guanine nucleotide exchange family protein | 32.7441 | 7.57528 | | -2.1119 |
| AT3G24630 | | unknown protein | 3.71161 | 0.859925 | | -2.1098 |
| AT4G02650 | | ENTH/ANTH/VHS superfamily protein | 10.8851 | 2.52248 | | -2.1094 |
| AT4G39160 | | Homeodomain-like superfamily protein | 50.7053 | 11.7527 | | -2.1091 |
| AT1G09575 | | Protein of unknown function (DUF607) | 40.3699 | 9.36591 | | -2.1078 |
| AT1G61520 | | LHCA3, photosystem I light harvesting complex gene 3 | 1.85903 | 0.431724 | | -2.1064 |
| AT3G03990 | | alpha/beta-Hydrolases superfamily protein | 357.218 | 82.981 | | -2.106 |
| AT4G00040 | | Chalcone and stilbene synthase family protein | 4.61295 | 1.07253 | | -2.1047 |
| AT1G76892 | | other RNA | 37.7102 | 8.78509 | | -2.1018 |
| AT3G14460 | | LRR and NB-ARC domains-containing disease resistance protein | 1.97703 | 0.460729 | | -2.1013 |
| AT3G21700 | | ATSGP2, SGP2, Ras-related small GTP-binding family protein | 153.294 | 35.7869 | | -2.0988 |
| AT3G10985 | | ATWI-12, SAG20, WI12, senescence associated gene 20 | 688.286 | 160.942 | | -2.0965 |
| AT3G49650 | | P-loop containing nucleoside triphosphate hydrolases superfamily protein | 11.6355 | 2.72315 | | -2.0952 |
| AT2G23770 | | protein kinase family protein / peptidoglycan-binding LysM domain-containing protein | 55.6336 | 13.0234 | | -2.0949 |
| AT1G54410 | | dehydrin family protein | 1393.94 | 327.969 | | -2.0875 |
| AT5G44030 | | CESA4, IRX5, NWS2, cellulose synthase A4 | 11.2382 | 2.64624 | | -2.0864 |
| AT5G38710 | | Methylenetetrahydrofolate reductase family protein | 134.102 | 31.5828 | | -2.0861 |
| AT3G04060 | | anac046, NAC046, NAC domain containing protein 46 | 78.1568 | 18.409 | | -2.086 |
| AT5G05270 | | Chalcone-flavanone isomerase family protein | 11.6427 | 2.74408 | | -2.085 |
| AT4G19230 | | CYP707A1, cytochrome P450, family 707, subfamily A, polypeptide 1 | 163.144 | 38.5162 | | -2.0826 |
| AT1G10370 | | ATGSTU17, ERD9, GST30, GST30B, Glutathione S-transferase family protein | 224.731 | 53.1103 | | -2.0811 |
| AT5G14310 | | AtCXE16, CXE16, carboxyesterase 16 | 66.5391 | 15.7283 | | -2.0808 |
| AT3G09580 | | FAD/NAD(P)-binding oxidoreductase family protein | 3.2806 | 0.775579 | | -2.0806 |
| AT2G23120 | | Late embryogenesis abundant protein, group 6 | 2371.51 | 560.779 | | -2.0803 |
| AT5G02420 | | unknown protein | 73.5097 | 17.3867 | | -2.08 |
| AT5G57510 | | unknown protein | 258.131 | 61.1094 | | -2.0786 |
| AT3G17410 | | Protein kinase superfamily protein | 171.128 | 40.5542 | | -2.0772 |
| AT5G45490 | | P-loop containing nucleoside triphosphate hydrolases superfamily protein | 115.1 | 27.2926 | | -2.0763 |
| AT1G10360 | | ATGSTU18, GST29, GSTU18, glutathione S-transferase TAU 18 | 6.12411 | 1.45348 | | -2.075 |
| AT4G02180 | | DC1 domain-containing protein | 1.30317 | 0.309295 | | -2.075 |
| AT1G19910 | | ATVHA-C2, AVA-2PE, AVA-P2, ATPase, F0/V0 complex, subunit C protein | 922.291 | 219.083 | | -2.0738 |
| AT5G10990 | | SAUR-like auxin-responsive protein family | 22.083 | 5.24685 | | -2.0734 |
| AT5G01480 | | Cysteine/Histidine-rich C1 domain family protein | 2.89672 | 0.688419 | | -2.0731 |
| AT5G01750 | | Protein of unknown function (DUF567) | 703.089 | 167.121 | | -2.0728 |
| AT3G49940 | | LBD38, LOB domain-containing protein 38 | 167.855 | 39.9379 | | -2.0714 |
| AT5G20250 | | DIN10, Raffinose synthase family protein | 446.084 | 106.141 | | -2.0713 |
| AT3G11900 | | ANT1, aromatic and neutral transporter 1 | 109.997 | 26.1879 | | -2.0705 |
| AT5G35460 | | unknown protein | 89.058 | 21.2174 | | -2.0695 |
| AT4G01070 | | GT72B1, UGT72B1, UDP-Glycosyltransferase superfamily protein | 290.56 | 69.3244 | | -2.0674 |
| AT5G02350 | | Cysteine/Histidine-rich C1 domain family protein | 64.5379 | 15.4004 | | -2.0672 |
| AT3G09960 | | Calcineurin-like metallo-phosphoesterase superfamily protein | 9.24363 | 2.20792 | | -2.0658 |
| AT3G09490 | | Tetratricopeptide repeat (TPR)-like superfamily protein | 15.5116 | 3.70568 | | -2.0655 |
| AT1G19397 | | unknown protein | 22.88 | 5.46617 | | -2.0655 |
| AT2G32380 | | Transmembrane protein 97, predicted | 130.904 | 31.276 | | -2.0654 |
| AT3G51180 | | Zinc finger C-x8-C-x5-C-x3-H type family protein | 3.51032 | 0.838695 | | -2.0654 |
| AT2G39050 | | hydroxyproline-rich glycoprotein family protein | 231.753 | 55.3765 | | -2.0652 |
| AT4G14280 | | ARM repeat superfamily protein | 16.2735 | 3.89118 | | -2.0642 |
| AT4G29310 | | Protein of unknown function (DUF1005) | 23.8666 | 5.72436 | | -2.0598 |
| AT5G13080 | | ATWRKY75, WRKY75, WRKY DNA-binding protein 75 | 164.884 | 39.5503 | | -2.0597 |
| AT3G25882 | | NIMIN-2, NIM1-interacting 2 | 85.1208 | 20.4362 | | -2.0584 |
| AT1G51620 | | Protein kinase superfamily protein | 18.7737 | 4.50864 | | -2.058 |
| AT5G64890 | | PROPEP2, elicitor peptide 2 precursor | 9.03668 | 2.17201 | | -2.0568 |
| AT1G05700 | | Leucine-rich repeat transmembrane protein kinase protein | 3.93789 | 0.946686 | | -2.0565 |
| AT2G28160 | | ATBHLH029, ATBHLH29, ATFIT1, BHLH029, FIT1, FRU, FER-like regulator of iron uptake | 18.8058 | 4.52206 | | -2.0561 |
| AT1G80380 | | P-loop containing nucleoside triphosphate hydrolases superfamily protein | 422.385 | 101.617 | | -2.0554 |
| AT1G23140 | | Calcium-dependent lipid-binding (CaLB domain) family protein | 46.8932 | 11.2883 | | -2.0546 |
| AT2G20230 | | Tetraspanin family protein | 143.986 | 34.7292 | | -2.0517 |
| AT4G12090 | | Cornichon family protein | 124.764 | 30.0976 | | -2.0515 |
| AT4G18780 | | ATCESA8, CESA8, IRX1, LEW2, cellulose synthase family protein | 3.73202 | 0.900624 | | -2.051 |
| AT3G27470 | | Protein of unknown function (DUF707) | 16.3202 | 3.94036 | | -2.0503 |
| AT3G56230 | | BTB/POZ domain-containing protein | 44.4348 | 10.7348 | | -2.0494 |
| AT4G14716 | | ARD1, ATARD1, acireductone dioxygenase 1 | 29.0369 | 7.0156 | | -2.0493 |
| AT1G15100 | | RHA2A, RING-H2 finger A2A | 658.001 | 159.186 | | -2.0474 |
| AT2G46140 | | Late embryogenesis abundant protein | 747.755 | 180.962 | | -2.0469 |
| AT2G38290 | | AMT2, AMT2;1, ATAMT2, ammonium transporter 2 | 72.3125 | 17.5239 | | -2.0449 |
| AT1G60010 | | unknown protein | 54.7641 | 13.2725 | | -2.0448 |
| AT3G11600 | | unknown protein | 10.6881 | 2.5913 | | -2.0443 |
| AT4G27657 | | unknown protein | 23.5732 | 5.72081 | | -2.0429 |
| AT3G53490 | | unknown protein | 37.5854 | 9.12581 | | -2.0422 |
| AT4G15070 | | Cysteine/Histidine-rich C1 domain family protein | 3.90052 | 0.950654 | | -2.0367 |
| AT4G14640 | | CAM8, calmodulin 8 | 58.0532 | 14.1588 | | -2.0357 |
| AT3G01290 | | SPFH/Band 7/PHB domain-containing membrane-associated protein family | 354.701 | 86.6315 | | -2.0336 |
| AT1G31120 | | KUP10, K+ uptake permease 10 | 27.419 | 6.69974 | | -2.033 |
| AT3G61760 | | ADL1B, DL1B, DYNAMIN-like 1B | 34.0428 | 8.31952 | | -2.0328 |
| AT3G02620 | | Plant stearoyl-acyl-carrier-protein desaturase family protein | 3.65249 | 0.892621 | | -2.0328 |
| AT2G40765 | | unknown protein | 400.871 | 98.0433 | | -2.0317 |
| AT2G16720 | | ATMYB7, ATY49, MYB7, myb domain protein 7 | 95.0576 | 23.2494 | | -2.0316 |
| AT4G15800 | | RALFL33, ralf-like 33 | 231.158 | 56.5528 | | -2.0312 |
| AT4G26710 | | ATPase, V0 complex, subunit E | 528.377 | 129.273 | | -2.0312 |
| AT3G04730 | | IAA16, indoleacetic acid-induced protein 16 | 500.06 | 122.369 | | -2.0309 |
| AT1G20840 | | TMT1, tonoplast monosaccharide transporter1 | 114.533 | 28.0419 | | -2.0301 |
| AT1G20390 | | transposable element gene | 3.7622 | 0.92127 | | -2.0299 |
| AT1G14520 | | MIOX1, myo-inositol oxygenase 1 | 3.21246 | 0.78678 | | -2.0297 |
| AT1G63220 | | Calcium-dependent lipid-binding (CaLB domain) family protein | 408.652 | 100.17 | | -2.0284 |
| AT1G70640 | | octicosapeptide/Phox/Bem1p (PB1) domain-containing protein | 6.42372 | 1.57584 | | -2.0273 |
| AT3G05580 | | Calcineurin-like metallo-phosphoesterase superfamily protein | 195.42 | 47.9499 | | -2.027 |
| AT4G13000 | | AGC (cAMP-dependent, cGMP-dependent and protein kinase C) kinase family protein | 2.27344 | 0.557861 | | -2.0269 |
| AT1G10350 | | DNAJ heat shock family protein | 33.7586 | 8.30636 | | -2.023 |
| AT1G09070 | | (AT)SRC2, SRC2, soybean gene regulated by cold-2 | 1357.32 | 334.265 | | -2.0217 |
| AT4G14430 | | ATECI2, ECHIB, ECI2, IBR10, PEC12, indole-3-butyric acid response 10 | 65.1954 | 16.0831 | | -2.0192 |
| AT5G67290 | | FAD-dependent oxidoreductase family protein | 40.1255 | 9.92739 | | -2.015 |
| AT3G57640 | | Protein kinase superfamily protein | 2.67527 | 0.662157 | | -2.0144 |
| AT5G40270 | | HD domain-containing metal-dependent phosphohydrolase family protein | 9.01667 | 2.23347 | | -2.0133 |
| AT2G38380 | | Peroxidase superfamily protein | 2.87893 | 0.714075 | | -2.0114 |
| AT1G04160 | | ATXIB, XI-8, XI-B, XIB, myosin XI B | 50.5496 | 12.575 | | -2.0071 |
| AT1G04040 | | HAD superfamily, subfamily IIIB acid phosphatase | 686.269 | 170.873 | | -2.0059 |
| AT1G62370 | | RING/U-box superfamily protein | 70.2079 | 17.4973 | | -2.0045 |
| AT4G19170 | | CCD4, NCED4, nine-cis-epoxycarotenoid dioxygenase 4 | 1.15204 | 0.28724 | | -2.0039 |
| AT5G11090 | | serine-rich protein-related | 110.826 | 27.648 | | -2.0031 |
| AT1G75820 | | ATCLV1, CLV1, FAS3, FLO5, Leucine-rich receptor-like protein kinase family protein | 1.58151 | 0.394542 | | -2.0031 |
| AT4G01360 | | unknown protein | 66.9772 | 16.7422 | | -2.0002 |
| AT3G18780 | | ACT2, DER1, ENL2, LSR2, actin 2 | 1579.69 | 395.117 | | -1.9993 |
| AT3G51460 | | RHD4, Phosphoinositide phosphatase family protein | 210.894 | 52.7531 | | -1.9992 |
| AT5G02790 | | GSTL3, Glutathione S-transferase family protein | 3.75013 | 0.938682 | | -1.9982 |
| AT5G66280 | | GMD1, GDP-D-mannose 4,6-dehydratase 1 | 109.064 | 27.3395 | | -1.9961 |
| AT1G13245 | | DVL4, RTFL17, ROTUNDIFOLIA like 17 | 127.33 | 31.9512 | | -1.9946 |
| AT3G53950 | | glyoxal oxidase-related protein | 3.74011 | 0.939628 | | -1.9929 |
| AT3G17420 | | GPK1, glyoxysomal protein kinase 1 | 115.141 | 28.969 | | -1.9908 |
| AT5G15240 | | Transmembrane amino acid transporter family protein | 1.79825 | 0.453392 | | -1.9878 |
| AT3G22850 | | Aluminium induced protein with YGL and LRDR motifs | 288.673 | 72.8211 | | -1.987 |
| AT1G61290 | | ATSYP124, SYP124, syntaxin of plants 124 | 13.1794 | 3.3257 | | -1.9866 |
| AT2G46620 | | P-loop containing nucleoside triphosphate hydrolases superfamily protein | 37.7178 | 9.56485 | | -1.9794 |
| AT4G39080 | | VHA-A3, vacuolar proton ATPase A3 | 597.978 | 151.661 | | -1.9792 |
| AT5G25820 | | Exostosin family protein | 32.8516 | 8.34222 | | -1.9775 |
| AT4G33920 | | Protein phosphatase 2C family protein | 274.739 | 69.901 | | -1.9747 |
| AT4G34720 | | ATVHA-C1, AVA-P1, VHA-C1, ATPase, F0/V0 complex, subunit C protein | 758.112 | 192.907 | | -1.9745 |
| AT3G20860 | | ATNEK5, NEK5, NIMA-related kinase 5 | 11.7719 | 2.99603 | | -1.9742 |
| AT5G24870 | | RING/U-box superfamily protein | 70.8318 | 18.0283 | | -1.9741 |
| AT1G15490 | | alpha/beta-Hydrolases superfamily protein | 15.5963 | 3.97284 | | -1.973 |
| AT3G02140 | | AFP4, TMAC2, AFP2 (ABI five-binding protein 2) family protein | 269.651 | 68.7033 | | -1.9726 |
| AT1G65180 | | Cysteine/Histidine-rich C1 domain family protein | 9.01323 | 2.29993 | | -1.9705 |
| AT1G51800 | | Leucine-rich repeat protein kinase family protein | 19.0238 | 4.8626 | | -1.968 |
| AT1G32260 | | unknown protein | 28.7455 | 7.35315 | | -1.9669 |
| AT1G56220 | | Dormancy/auxin associated family protein | 141.148 | 36.1249 | | -1.9661 |
| AT1G74740 | | ATCPK30, CDPK1A, CPK30, calcium-dependent protein kinase 30 | 22.694 | 5.80925 | | -1.9659 |
| AT4G17550 | | Major facilitator superfamily protein | 10.2507 | 2.62519 | | -1.9652 |
| AT5G13330 | | Rap2.6L, related to AP2 6l | 150.29 | 38.518 | | -1.9642 |
| AT3G09375 | | pseudogene, putative eukaryotic translation initiation factor 4A-3 (eIF-4A-3/eIF4A-3) | 17.32 | 4.43946 | | -1.964 |
| AT3G20310 | | ATERF-7, ATERF7, ERF7, ethylene response factor 7 | 84.9658 | 21.787 | | -1.9634 |
| AT3G57040 | | ARR9, ATRR4, response regulator 9 | 57.6293 | 14.7806 | | -1.9631 |
| AT1G80180 | | unknown protein | 24.7397 | 6.35601 | | -1.9606 |
| AT3G51160 | | GMD2, MUR1, MUR_1, NAD(P)-binding Rossmann-fold superfamily protein | 194.458 | 49.9687 | | -1.9604 |
| AT4G27654 | | unknown protein | 50.0616 | 12.866 | | -1.9601 |
| AT5G11230 | | Nucleotide-sugar transporter family protein | 268.903 | 69.1341 | | -1.9596 |
| AT1G20090 | | ARAC4, ATRAC4, ATROP2, ROP2, RHO-related protein from plants 2 | 120.63 | 31.0274 | | -1.959 |
| AT1G09740 | | Adenine nucleotide alpha hydrolases-like superfamily protein | 256.845 | 66.0785 | | -1.9587 |
| AT3G05936 | | unknown protein | 15.0029 | 3.86097 | | -1.9582 |
| AT3G15670 | | Late embryogenesis abundant protein (LEA) family protein | 692.424 | 178.453 | | -1.9561 |
| AT4G34920 | | PLC-like phosphodiesterases superfamily protein | 6.40281 | 1.65538 | | -1.9515 |
| AT3G11800 | | unknown protein | 171.859 | 44.4368 | | -1.9514 |
| AT1G75220 | | Major facilitator superfamily protein | 456.642 | 118.127 | | -1.9507 |
| AT3G17440 | | ATNPSN13, NPSN13, novel plant snare 13 | 132.224 | 34.2436 | | -1.9491 |
| AT1G73805 | | Calmodulin binding protein-like | 8.69024 | 2.25135 | | -1.9486 |
| AT4G12330 | | CYP706A7, cytochrome P450, family 706, subfamily A, polypeptide 7 | 23.3883 | 6.06555 | | -1.9471 |
| AT4G17670 | | Protein of unknown function (DUF581) | 17.4655 | 4.53365 | | -1.9458 |
| AT2G41110 | | ATCAL5, CAM2, calmodulin 2 | 513.368 | 133.265 | | -1.9457 |
| AT2G24545 | | other RNA | 12.1118 | 3.14438 | | -1.9456 |
| AT3G01430 | | BEST Arabidopsis thaliana protein match is: NHL domain-containing protein (TAIR:AT5G14890.1) | 6.79385 | 1.764 | | -1.9454 |
| AT5G15420 | | unknown protein | 24.0171 | 6.23667 | | -1.9452 |
| AT1G08770 | | PRA1.E, prenylated RAB acceptor 1.E | 33.5794 | 8.72335 | | -1.9446 |
| AT1G08880 | | G-H2AX, GAMMA-H2AX, H2AXA, HTA5, Histone superfamily protein | 267.518 | 69.502 | | -1.9445 |
| AT5G65310 | | ATHB-5, ATHB5, HB5, homeobox protein 5 | 22.4398 | 5.8317 | | -1.9441 |
| AT1G74640 | | alpha/beta-Hydrolases superfamily protein | 33.3475 | 8.66905 | | -1.9436 |
| AT3G45095 | | transposable element gene | 3.31498 | 0.86187 | | -1.9435 |
| AT5G38790 | | unknown protein | 25.1907 | 6.55392 | | -1.9425 |
| AT1G72880 | | Survival protein SurE-like phosphatase/nucleotidase | 83.742 | 21.7963 | | -1.9419 |
| AT5G60020 | | ATLAC17, LAC17, laccase 17 | 3.08018 | 0.801737 | | -1.9418 |
| AT1G64820 | | MATE efflux family protein | 2.49566 | 0.650383 | | -1.9401 |
| AT5G24910 | | CYP714A1, cytochrome P450, family 714, subfamily A, polypeptide 1 | 3.46155 | 0.902335 | | -1.9397 |
| AT1G10170 | | ATNFXL1, NFXL1, NF-X-like 1 | 537.884 | 140.222 | | -1.9396 |
| AT5G15210 | | ATHB30, HB30, ZFHD3, homeobox protein 30 | 3.2252 | 0.841463 | | -1.9384 |
| AT2G35610 | | XEG113, xyloglucanase 113 | 128.058 | 33.415 | | -1.9382 |
| AT1G20030 | | Pathogenesis-related thaumatin superfamily protein | 10.5941 | 2.76791 | | -1.9364 |
| AT2G21840 | | Cysteine/Histidine-rich C1 domain family protein | 27.2894 | 7.1375 | | -1.9349 |
| AT1G32460 | | unknown protein | 223.483 | 58.4616 | | -1.9346 |
| AT5G15340 | | Pentatricopeptide repeat (PPR) superfamily protein | 4.98064 | 1.30315 | | -1.9343 |
| AT4G14520 | | DNA-directed RNA polymerase II-related | 23.1685 | 6.06817 | | -1.9328 |
| AT5G60320 | | Concanavalin A-like lectin protein kinase family protein | 3.99412 | 1.04658 | | -1.9322 |
| AT5G16370 | | AAE5, acyl activating enzyme 5 | 53.1633 | 13.9336 | | -1.9319 |
| AT3G19250 | | Protein of unknown function (DUF677) | 5.74596 | 1.50749 | | -1.9304 |
| AT4G22780 | | ACR7, ACT domain repeat 7 | 61.6287 | 16.1688 | | -1.9304 |
| AT2G41410 | | Calcium-binding EF-hand family protein | 317.713 | 83.4683 | | -1.9284 |
| AT5G01700 | | Protein phosphatase 2C family protein | 14.6044 | 3.84432 | | -1.9256 |
| AT2G37040 | | ATPAL1, PAL1, PHE ammonia lyase 1 | 132.303 | 34.8381 | | -1.9251 |
| AT3G02880 | | Leucine-rich repeat protein kinase family protein | 255.226 | 67.3298 | | -1.9225 |
| AT2G23100 | | Cysteine/Histidine-rich C1 domain family protein | 5.16973 | 1.36594 | | -1.9202 |
| AT3G27210 | | unknown protein | 41.4602 | 10.9693 | | -1.9183 |
| AT1G79340 | | AtMC4, MC4, metacaspase 4 | 462.406 | 122.438 | | -1.9171 |
| AT4G14500 | | Polyketide cyclase/dehydrase and lipid transport superfamily protein | 184.224 | 48.7814 | | -1.9171 |
| AT5G62560 | | RING/U-box superfamily protein with ARM repeat domain | 38.3476 | 10.1543 | | -1.917 |
| AT2G19160 | | Core-2/I-branching beta-1,6-N-acetylglucosaminyltransferase family protein | 80.7333 | 21.3898 | | -1.9162 |
| AT3G10010 | | DML2, demeter-like 2 | 1.23067 | 0.326241 | | -1.9154 |
| AT5G11950 | | Putative lysine decarboxylase family protein | 85.2588 | 22.6206 | | -1.9142 |
| AT3G54420 | | ATCHITIV, ATEP3, CHIV, EP3, homolog of carrot EP3-3 chitinase | 5.32299 | 1.41238 | | -1.9141 |
| AT5G43040 | | Cysteine/Histidine-rich C1 domain family protein | 6.12025 | 1.62447 | | -1.9136 |
| AT3G62260 | | Protein phosphatase 2C family protein | 216.198 | 57.4319 | | -1.9124 |
| AT4G18425 | | Protein of unknown function (DUF679) | 28.2426 | 7.51435 | | -1.9102 |
| AT2G18620 | | Terpenoid synthases superfamily protein | 4.71811 | 1.25625 | | -1.9091 |
| AT2G25530 | | AFG1-like ATPase family protein | 10.3091 | 2.74497 | | -1.9091 |
| AT4G27450 | | Aluminium induced protein with YGL and LRDR motifs | 261.962 | 69.8234 | | -1.9076 |
| AT3G16910 | | AAE7, ACN1, acyl-activating enzyme 7 | 89.2772 | 23.8106 | | -1.9067 |
| AT3G09810 | | IDH-VI, isocitrate dehydrogenase VI | 242.394 | 64.7445 | | -1.9045 |
| AT3G07480 | | 2Fe-2S ferredoxin-like superfamily protein | 421.963 | 112.714 | | -1.9045 |
| AT5G38700 | | unknown protein | 98.1841 | 26.2368 | | -1.9039 |
| AT5G67190 | | DEAR2, DREB and EAR motif protein 2 | 96.7601 | 25.8656 | | -1.9034 |
| AT1G30455 | | transcription regulators;translation initiation factors;zinc ion binding;transcription activators | 6.74576 | 1.8058 | | -1.9014 |
| AT2G47950 | | unknown protein | 278.231 | 74.4824 | | -1.9013 |
| AT2G45330 | | emb1067, RNA 2'-phosphotransferase, Tpt1 / KptA family | 59.914 | 16.066 | | -1.8989 |
| AT1G19440 | | KCS4, 3-ketoacyl-CoA synthase 4 | 52.0617 | 13.9648 | | -1.8984 |
| AT5G45630 | | Protein of unknown function, DUF584 | 145.646 | 39.0753 | | -1.8981 |
| AT4G25470 | | ATCBF2, CBF2, DREB1C, FTQ4, C-repeat/DRE binding factor 2 | 6.95951 | 1.86786 | | -1.8976 |
| AT3G49120 | | ATPCB, ATPERX34, PERX34, PRX34, PRXCB, peroxidase CB | 7.84592 | 2.10598 | | -1.8975 |
| AT3G56760 | | Protein kinase superfamily protein | 54.6139 | 14.6612 | | -1.8973 |
| AT1G49032 | | unknown protein | 37.1449 | 9.9723 | | -1.8972 |
| AT4G15500 | | UGT84A4, UDP-Glycosyltransferase superfamily protein | 5.306 | 1.42474 | | -1.8969 |
| AT1G76990 | | ACR3, ACT domain repeat 3 | 194.67 | 52.2885 | | -1.8965 |
| AT5G43400 | | Uncharacterised conserved protein UCP015417, vWA | 32.7917 | 8.81557 | | -1.8952 |
| AT5G10430 | | AGP4, ATAGP4, arabinogalactan protein 4 | 198.707 | 53.4325 | | -1.8949 |
| AT4G19045 | | Mob1/phocein family protein | 54.0514 | 14.5491 | | -1.8934 |
| AT1G70230 | | TBL27, TRICHOME BIREFRINGENCE-LIKE 27 | 8.08509 | 2.17674 | | -1.8931 |
| AT1G05640 | | Ankyrin repeat family protein | 7.46793 | 2.01089 | | -1.8929 |
| AT5G18860 | | inosine-uridine preferring nucleoside hydrolase family protein | 101.121 | 27.2386 | | -1.8924 |
| AT5G67160 | | EPS1, HXXXD-type acyl-transferase family protein | 9.35251 | 2.52002 | | -1.8919 |
| AT5G47040 | | LON2, lon protease 2 | 203.331 | 54.7876 | | -1.8919 |
| AT1G07610 | | MT1C, metallothionein 1C | 104.157 | 28.0747 | | -1.8914 |
| AT1G18300 | | atnudt4, NUDT4, nudix hydrolase homolog 4 | 42.3746 | 11.423 | | -1.8913 |
| AT5G40210 | | nodulin MtN21 /EamA-like transporter family protein | 31.3616 | 8.469 | | -1.8887 |
| AT2G34585 | | unknown protein | 36.9671 | 9.98873 | | -1.8879 |
| AT3G61280 | | Arabidopsis thaliana protein of unknown function (DUF821) | 1.91361 | 0.51783 | | -1.8857 |
| AT1G54450 | | Calcium-binding EF-hand family protein | 22.2222 | 6.01481 | | -1.8854 |
| AT3G60450 | | Phosphoglycerate mutase family protein | 643.847 | 174.274 | | -1.8854 |
| AT5G67300 | | ATMYB44, ATMYBR1, MYB44, MYBR1, myb domain protein r1 | 195.702 | 53.0075 | | -1.8844 |
| AT1G71780 | | unknown protein | 110.731 | 29.9951 | | -1.8843 |
| AT4G12070 | | unknown protein | 73.5546 | 19.9464 | | -1.8827 |
| AT5G64620 | | ATC/VIF2, C/VIF2, cell wall / vacuolar inhibitor of fructosidase 2 | 31.8113 | 8.6449 | | -1.8796 |
| AT1G05340 | | unknown protein | 838.556 | 228.011 | | -1.8788 |
| AT5G06930 | | BEST Arabidopsis thaliana protein match is: nucleolar protein gar2-related (TAIR:AT2G42320.2) | 33.9366 | 9.23135 | | -1.8782 |
| AT5G42380 | | CML37, CML39, calmodulin like 37 | 244.732 | 66.6011 | | -1.8776 |
| AT2G25150 | | HXXXD-type acyl-transferase family protein | 29.7066 | 8.08563 | | -1.8774 |
| AT4G23730 | | Galactose mutarotase-like superfamily protein | 114.307 | 31.1309 | | -1.8765 |
| AT3G01990 | | ACR6, ACT domain repeat 6 | 4.73717 | 1.29036 | | -1.8763 |
| AT2G46030 | | UBC6, ubiquitin-conjugating enzyme 6 | 178.653 | 48.6747 | | -1.8759 |
| AT3G29410 | | Terpenoid cyclases/Protein prenyltransferases superfamily protein | 86.9658 | 23.6998 | | -1.8756 |
| AT5G01760 | | ENTH/VHS/GAT family protein | 61.6202 | 16.7934 | | -1.8755 |
| AT4G37830 | | cytochrome c oxidase-related | 729.118 | 198.929 | | -1.8739 |
| AT5G47240 | | atnudt8, NUDT8, nudix hydrolase homolog 8 | 54.7061 | 14.9332 | | -1.8732 |
| AT3G10120 | | unknown protein | 5.7269 | 1.56557 | | -1.8711 |
| AT3G03341 | | unknown protein | 268.034 | 73.3189 | | -1.8702 |
| AT3G24100 | | Uncharacterised protein family SERF | 106.541 | 29.181 | | -1.8683 |
| AT3G28850 | | Glutaredoxin family protein | 15.7242 | 4.30781 | | -1.868 |
| AT5G67350 | | unknown protein | 56.6379 | 15.5192 | | -1.8677 |
| AT4G26200 | | ACS7, ATACS7, 1-amino-cyclopropane-1-carboxylate synthase 7 | 15.5717 | 4.27107 | | -1.8663 |
| AT4G30160 | | ATVLN4, VLN4, villin 4 | 94.7152 | 26.0059 | | -1.8648 |
| AT3G48590 | | ATHAP5A, HAP5A, NF-YC1, nuclear factor Y, subunit C1 | 11.9801 | 3.28945 | | -1.8647 |
| AT2G47520 | | HRE2, Integrase-type DNA-binding superfamily protein | 17.249 | 4.73635 | | -1.8647 |
| AT5G35180 | | Protein of unknown function (DUF1336) | 95.7102 | 26.293 | | -1.864 |
| AT3G13720 | | PRA1.F3, PRA8, PRA1 (Prenylated rab acceptor) family protein | 79.445 | 21.8349 | | -1.8633 |
| AT3G53420 | | PIP2, PIP2;1, PIP2A, plasma membrane intrinsic protein 2A | 598 | 164.361 | | -1.8633 |
| AT5G63130 | | Octicosapeptide/Phox/Bem1p family protein | 101.391 | 27.9117 | | -1.861 |
| AT3G29575 | | AFP3, ABI five binding protein 3 | 189.405 | 52.182 | | -1.8599 |
| AT3G54020 | | AtIPCS1, Arabidopsis Inositol phosphorylceramide synthase 1 | 105.698 | 29.158 | | -1.858 |
| AT1G77120 | | ADH, ADH1, ATADH, ATADH1, alcohol dehydrogenase 1 | 594.086 | 164.008 | | -1.8569 |
| AT1G70330 | | ENT1, ENT1,AT, equilibrative nucleotide transporter 1 | 92.4346 | 25.5205 | | -1.8568 |
| AT2G44600 | | unknown protein | 8.71181 | 2.40821 | | -1.855 |
| AT5G06760 | | LEA4-5, Late Embryogenesis Abundant 4-5 | 464.108 | 128.389 | | -1.8539 |
| AT2G33510 | | CONTAINS InterPro DOMAIN/s: WW/Rsp5/WWP (InterPro:IPR001202) | 37.9458 | 10.5024 | | -1.8532 |
| AT1G14220 | | Ribonuclease T2 family protein | 109.629 | 30.3451 | | -1.8531 |
| AT3G07360 | | ATPUB9, PUB9, plant U-box 9 | 60.0916 | 16.6531 | | -1.8514 |
| AT5G11670 | | ATNADP-ME2, NADP-ME2, NADP-malic enzyme 2 | 867.564 | 240.675 | | -1.8499 |
| AT2G15970 | | ATCOR413-PM1, cold regulated 413 plasma membrane 1 | 1992.6 | 553.066 | | -1.8491 |
| AT1G20120 | | GDSL-like Lipase/Acylhydrolase superfamily protein | 11.5468 | 3.20837 | | -1.8476 |
| AT1G19700 | | BEL10, BLH10, BEL1-like homeodomain 10 | 38.6821 | 10.7547 | | -1.8467 |
| AT1G22750 | | unknown protein | 35.1327 | 9.76815 | | -1.8467 |
| AT5G37750 | | Chaperone DnaJ-domain superfamily protein | 18.5309 | 5.15544 | | -1.8458 |
| AT4G39360 | | unknown protein | 39.434 | 10.9713 | | -1.8457 |
| AT5G45350 | | proline-rich family protein | 424.782 | 118.27 | | -1.8446 |
| AT5G58320 | | Kinase interacting (KIP1-like) family protein | 235.739 | 65.6598 | | -1.8441 |
| AT2G42490 | | Copper amine oxidase family protein | 111.936 | 31.1836 | | -1.8438 |
| AT3G02470 | | SAMDC, S-adenosylmethionine decarboxylase | 1018.14 | 283.691 | | -1.8435 |
| AT1G79870 | | D-isomer specific 2-hydroxyacid dehydrogenase family protein | 34.2182 | 9.53903 | | -1.8429 |
| AT2G36320 | | A20/AN1-like zinc finger family protein | 274.787 | 76.6466 | | -1.842 |
| AT5G59220 | | HAI1, highly ABA-induced PP2C gene 1 | 40.6677 | 11.3602 | | -1.8399 |
| AT5G28630 | | glycine-rich protein | 113.9 | 31.8575 | | -1.8381 |
| AT4G22610 | | Bifunctional inhibitor/lipid-transfer protein/seed storage 2S albumin superfamily protein | 689.309 | 192.824 | | -1.8379 |
| AT1G49240 | | ACT8, actin 8 | 1495.81 | 418.454 | | -1.8378 |
| AT5G45370 | | nodulin MtN21 /EamA-like transporter family protein | 17.9421 | 5.02035 | | -1.8375 |
| AT4G27320 | | ATPHOS34, PHOS34, Adenine nucleotide alpha hydrolases-like superfamily protein | 267.712 | 74.9122 | | -1.8374 |
| AT1G80450 | | VQ motif-containing protein | 36.2114 | 10.1338 | | -1.8373 |
| AT2G21510 | | DNAJ heat shock N-terminal domain-containing protein | 29.7385 | 8.32959 | | -1.836 |
| AT4G31470 | | CAP (Cysteine-rich secretory proteins, Antigen 5, and Pathogenesis-related 1 protein) superfamily protein | 98.0658 | 27.4756 | | -1.8356 |
| AT5G13210 | | Uncharacterised conserved protein UCP015417, vWA | 121.552 | 34.0587 | | -1.8355 |
| AT5G45472 | | Potential natural antisense gene, locus overlaps with AT5G45470 | 18.5247 | 5.19783 | | -1.8335 |
| AT5G52250 | | Transducin/WD40 repeat-like superfamily protein | 9.01629 | 2.53009 | | -1.8333 |
| AT5G56150 | | UBC30, ubiquitin-conjugating enzyme 30 | 194.655 | 54.6342 | | -1.833 |
| AT3G57230 | | AGL16, AGAMOUS-like 16 | 48.8883 | 13.727 | | -1.8325 |
| AT5G67480 | | ATBT4, BT4, BTB and TAZ domain protein 4 | 255.167 | 71.6529 | | -1.8324 |
| AT3G19240 | | Vacuolar import/degradation, Vid27-related protein | 350.111 | 98.3167 | | -1.8323 |
| AT1G10140 | | Uncharacterised conserved protein UCP031279 | 383.338 | 107.714 | | -1.8314 |
| AT5G44050 | | MATE efflux family protein | 72.4441 | 20.3635 | | -1.8309 |
| AT3G29035 | | ANAC059, ATNAC3, NAC3, NAC domain containing protein 3 | 109.127 | 30.6844 | | -1.8304 |
| AT1G51360 | | ATDABB1, DABB1, dimeric A/B barrel domainS-protein 1 | 26.7404 | 7.52311 | | -1.8296 |
| AT1G64460 | | Protein kinase superfamily protein | 403.738 | 113.635 | | -1.829 |
| AT1G67050 | | unknown protein | 12.3242 | 3.47135 | | -1.8279 |
| AT4G36820 | | Protein of unknown function (DUF607) | 122.752 | 34.5936 | | -1.8272 |
| AT4G00440 | | Protein of unknown function (DUF3741) | 12.1084 | 3.41702 | | -1.8252 |
| AT4G03110 | | AtRBP-DR1, RBP-DR1, RNA-binding protein-defense related 1 | 75.7694 | 21.3893 | | -1.8247 |
| AT1G29680 | | Protein of unknown function (DUF1264) | 10.6132 | 2.99796 | | -1.8238 |
| AT3G20510 | | Transmembrane proteins 14C | 213.264 | 60.2618 | | -1.8233 |
| AT5G15190 | | unknown protein | 115.585 | 32.6688 | | -1.823 |
| AT1G67480 | | Galactose oxidase/kelch repeat superfamily protein | 486.421 | 137.648 | | -1.8212 |
| AT3G48520 | | CYP94B3, cytochrome P450, family 94, subfamily B, polypeptide 3 | 126.147 | 35.7016 | | -1.821 |
| AT3G57330 | | ACA11, autoinhibited Ca2+-ATPase 11 | 132.976 | 37.6509 | | -1.8204 |
| AT1G79160 | | unknown protein | 110.65 | 31.3521 | | -1.8194 |
| AT5G46230 | | Protein of unknown function, DUF538 | 85.5275 | 24.2494 | | -1.8184 |
| AT2G31345 | | unknown protein | 12.3679 | 3.50687 | | -1.8184 |
| AT2G35930 | | PUB23, plant U-box 23 | 113.232 | 32.1137 | | -1.818 |
| AT3G16350 | | Homeodomain-like superfamily protein | 80.7608 | 22.9207 | | -1.817 |
| AT4G03510 | | ATRMA1, RMA1, RING membrane-anchor 1 | 361.587 | 102.716 | | -1.8157 |
| AT3G10960 | | ATAZG1, AZG1, AZA-guanine resistant1 | 166.839 | 47.4165 | | -1.815 |
| AT1G23550 | | SRO2, similar to RCD one 2 | 6.67282 | 1.89675 | | -1.8148 |
| AT3G48100 | | ARR5, ATRR2, IBC6, RR5, response regulator 5 | 159.127 | 45.2639 | | -1.8138 |
| AT3G27240 | | Cytochrome C1 family | 440.435 | 125.396 | | -1.8124 |
| AT1G24180 | | IAR4, Thiamin diphosphate-binding fold (THDP-binding) superfamily protein | 398.713 | 113.525 | | -1.8123 |
| AT3G27890 | | NQR, NADPH:quinone oxidoreductase | 151.262 | 43.0954 | | -1.8114 |
| AT5G24640 | | unknown protein | 21.3747 | 6.09081 | | -1.8112 |
| AT2G17710 | | unknown protein | 35.6985 | 10.1854 | | -1.8094 |
| AT1G60750 | | NAD(P)-linked oxidoreductase superfamily protein | 36.2223 | 10.3391 | | -1.8088 |
| AT4G22592 | | CPuORF27, conserved peptide upstream open reading frame 27 | 966.459 | 275.933 | | -1.8084 |
| AT5G13910 | | LEP, Integrase-type DNA-binding superfamily protein | 39.4181 | 11.2636 | | -1.8072 |
| AT1G15670 | | Galactose oxidase/kelch repeat superfamily protein | 429.508 | 122.931 | | -1.8048 |
| AT3G61410 | | BEST Arabidopsis thaliana protein match is: U-box domain-containing protein kinase family protein (TAIR:AT2G45910.1) | 19.9168 | 5.70126 | | -1.8046 |
| AT2G16770 | | bZIP23, Basic-leucine zipper (bZIP) transcription factor family protein | 18.3224 | 5.24778 | | -1.8038 |
| AT2G02410 | | unknown protein | 6.78504 | 1.94344 | | -1.8037 |
| AT5G46780 | | VQ motif-containing protein | 73.4034 | 21.0306 | | -1.8034 |
| AT2G41660 | | MIZ1, Protein of unknown function, DUF617 | 270.944 | 77.6576 | | -1.8028 |
| AT1G22470 | | unknown protein | 118.513 | 34.0065 | | -1.8012 |
| AT5G20540 | | ATBRXL4, BRX-LIKE4, BRXL4, BREVIS RADIX-like 4 | 9.40444 | 2.69888 | | -1.801 |
| AT2G16510 | | ATPase, F0/V0 complex, subunit C protein | 242.249 | 69.5824 | | -1.7997 |
| AT1G68940 | | Armadillo/beta-catenin-like repeat family protein | 2.78771 | 0.801094 | | -1.799 |
| AT2G22470 | | AGP2, ATAGP2, arabinogalactan protein 2 | 3220.46 | 925.556 | | -1.7989 |
| AT2G05940 | | Protein kinase superfamily protein | 66.0352 | 18.9922 | | -1.7978 |
| AT5G56950 | | NAP1;3, NFA03, NFA3, nucleosome assembly protein 1;3 | 136.201 | 39.182 | | -1.7975 |
| AT3G58710 | | ATWRKY69, WRKY69, WRKY DNA-binding protein 69 | 118.658 | 34.1444 | | -1.7971 |
| AT4G00330 | | CRCK2, calmodulin-binding receptor-like cytoplasmic kinase 2 | 25.0038 | 7.19712 | | -1.7967 |
| AT1G47128 | | RD21, RD21A, Granulin repeat cysteine protease family protein | 766.855 | 220.755 | | -1.7965 |
| AT5G64310 | | AGP1, ATAGP1, arabinogalactan protein 1 | 1739.09 | 501.379 | | -1.7944 |
| AT2G28550 | | RAP2.7, TOE1, related to AP2.7 | 29.8828 | 8.61754 | | -1.794 |
| AT5G54585 | | unknown protein | 37.1068 | 10.7015 | | -1.7939 |
| AT1G69800 | | Cystathionine beta-synthase (CBS) protein | 35.1853 | 10.1535 | | -1.793 |
| AT2G17840 | | ERD7, Senescence/dehydration-associated protein-related | 677.79 | 195.594 | | -1.793 |
| AT5G56760 | | ATSERAT1;1, SAT-52, SAT5, SERAT1;1, serine acetyltransferase 1;1 | 243.944 | 70.4109 | | -1.7927 |
| AT4G32300 | | SD2-5, S-domain-2 5 | 12.9491 | 3.74348 | | -1.7904 |
| AT3G54680 | | proteophosphoglycan-related | 159.124 | 46.0036 | | -1.7903 |
| AT4G39890 | | AtRABH1c, RABH1c, RAB GTPase homolog H1C | 95.2884 | 27.5567 | | -1.7899 |
| AT5G20650 | | COPT5, copper transporter 5 | 114.657 | 33.1603 | | -1.7898 |
| AT1G53790 | | F-box and associated interaction domains-containing protein | 4.70666 | 1.36268 | | -1.7883 |
| AT3G03170 | | unknown protein | 203.964 | 59.0553 | | -1.7882 |
| AT2G39780 | | RNS2, ribonuclease 2 | 322.217 | 93.3188 | | -1.7878 |
| AT5G05440 | | PYL5, RCAR8, Polyketide cyclase/dehydrase and lipid transport superfamily protein | 96.5699 | 27.9988 | | -1.7862 |
| AT3G61180 | | RING/U-box superfamily protein | 54.5134 | 15.8121 | | -1.7856 |
| AT4G38250 | | Transmembrane amino acid transporter family protein | 77.9488 | 22.6118 | | -1.7855 |
| AT3G16560 | | Protein phosphatase 2C family protein | 20.7902 | 6.03176 | | -1.7853 |
| AT2G23910 | | NAD(P)-binding Rossmann-fold superfamily protein | 5.40046 | 1.56798 | | -1.7842 |
| AT3G52370 | | FLA15, FASCICLIN-like arabinogalactan protein 15 precursor | 14.8346 | 4.30956 | | -1.7834 |
| AT5G17350 | | unknown protein | 62.355 | 18.116 | | -1.7832 |
| AT3G13970 | | APG12, APG12B, Ubiquitin-like superfamily protein | 22.3329 | 6.48896 | | -1.7831 |
| AT2G02080 | | AtIDD4, IDD4, indeterminate(ID)-domain 4 | 6.03437 | 1.75353 | | -1.7829 |
| AT3G24670 | | Pectin lyase-like superfamily protein | 481.013 | 139.818 | | -1.7825 |
| AT1G75810 | | unknown protein | 19.3714 | 5.63276 | | -1.782 |
| AT5G58800 | | Quinone reductase family protein | 33.652 | 9.79985 | | -1.7799 |
| AT4G02075 | | PIT1, RING/FYVE/PHD zinc finger superfamily protein | 23.8967 | 6.96019 | | -1.7796 |
| AT5G46900 | | Bifunctional inhibitor/lipid-transfer protein/seed storage 2S albumin superfamily protein | 80.0738 | 23.358 | | -1.7774 |
| AT4G32920 | | glycine-rich protein | 59.7635 | 17.4505 | | -1.776 |
| AT4G35550 | | ATWOX13, HB-4, WOX13, WUSCHEL related homeobox 13 | 61.4362 | 17.9595 | | -1.7743 |
| AT4G14370 | | Disease resistance protein (TIR-NBS-LRR class) family | 28.2606 | 8.26189 | | -1.7743 |
| AT1G27910 | | ATPUB45, PUB45, plant U-box 45 | 48.9338 | 14.3056 | | -1.7743 |
| AT2G16870 | | Disease resistance protein (TIR-NBS-LRR class) family | 7.64277 | 2.23463 | | -1.7741 |
| AT4G31875 | | unknown protein | 20.0661 | 5.86968 | | -1.7734 |
| AT1G15400 | | unknown protein | 276.42 | 80.8868 | | -1.7729 |
| AT1G51370 | | F-box/RNI-like/FBD-like domains-containing protein | 11.619 | 3.40115 | | -1.7724 |
| AT5G43830 | | Aluminium induced protein with YGL and LRDR motifs | 913.848 | 267.619 | | -1.7718 |
| AT4G37530 | | Peroxidase superfamily protein | 52.2388 | 15.299 | | -1.7717 |
| AT3G14920 | | Peptide-N4-(N-acetyl-beta-glucosaminyl)asparagine amidase A protein | 12.4159 | 3.63744 | | -1.7712 |
| AT4G18070 | | unknown protein | 150.668 | 44.1548 | | -1.7707 |
| AT3G47340 | | ASN1, AT-ASN1, DIN6, glutamine-dependent asparagine synthase 1 | 622.356 | 182.487 | | -1.7699 |
| AT2G25520 | | Drug/metabolite transporter superfamily protein | 274.687 | 80.6351 | | -1.7683 |
| AT3G57410 | | ATVLN3, VLN3, villin 3 | 124.342 | 36.5097 | | -1.768 |
| AT4G02620 | | vacuolar ATPase subunit F family protein | 700.66 | 205.858 | | -1.7671 |
| AT2G47700 | | RFI2, RING/U-box superfamily protein | 98.7352 | 29.0208 | | -1.7665 |
| AT5G16380 | | Protein of unknown function, DUF538 | 55.7806 | 16.4159 | | -1.7647 |
| AT5G19120 | | Eukaryotic aspartyl protease family protein | 273.504 | 80.5682 | | -1.7633 |
| AT2G03730 | | ACR5, ACT domain repeat 5 | 102.95 | 30.3333 | | -1.763 |
| AT5G19020 | | MEF18, mitochondrial editing factor 18 | 25.5289 | 7.52262 | | -1.7628 |
| AT1G25370 | | Protein of unknown function (DUF1639) | 16.8872 | 4.97684 | | -1.7626 |
| AT4G38920 | | ATVHA-C3, AVA-P3, VHA-C3, vacuolar-type H(+)-ATPase C3 | 572.495 | 168.82 | | -1.7618 |
| AT2G43330 | | ATINT1, INT1, inositol transporter 1 | 121.522 | 35.8465 | | -1.7613 |
| AT1G07040 | | unknown protein | 55.6657 | 16.4369 | | -1.7599 |
| AT1G18620 | | unknown protein | 21.8158 | 6.46175 | | -1.7554 |
| AT2G41000 | | Chaperone DnaJ-domain superfamily protein | 5.17211 | 1.53224 | | -1.7551 |
| AT5G06810 | | Mitochondrial transcription termination factor family protein | 9.61365 | 2.85003 | | -1.7541 |
| AT4G26910 | | Dihydrolipoamide succinyltransferase | 201.259 | 59.6843 | | -1.7536 |
| AT5G39970 | | catalytics | 32.0089 | 9.49371 | | -1.7534 |
| AT2G32150 | | Haloacid dehalogenase-like hydrolase (HAD) superfamily protein | 1407.9 | 417.731 | | -1.7529 |
| AT1G11660 | | heat shock protein 70 (Hsp 70) family protein | 84.1365 | 24.9657 | | -1.7528 |
| AT5G03555 | | permease, cytosine/purines, uracil, thiamine, allantoin family protein | 23.8641 | 7.08281 | | -1.7525 |
| AT5G34930 | | arogenate dehydrogenase | 33.8319 | 10.0518 | | -1.7509 |
| AT5G65140 | | Haloacid dehalogenase-like hydrolase (HAD) superfamily protein | 131.562 | 39.1553 | | -1.7485 |
| AT3G14440 | | ATNCED3, NCED3, SIS7, STO1, nine-cis-epoxycarotenoid dioxygenase 3 | 7.49749 | 2.23388 | | -1.7469 |
| AT5G01800 | | saposin B domain-containing protein | 275.465 | 82.1149 | | -1.7462 |
| AT1G07750 | | RmlC-like cupins superfamily protein | 317.538 | 94.6886 | | -1.7457 |
| AT1G22550 | | Major facilitator superfamily protein | 10.3585 | 3.09013 | | -1.7451 |
| AT3G06470 | | GNS1/SUR4 membrane protein family | 13.1107 | 3.91154 | | -1.7449 |
| AT1G75380 | | ATBBD1, BBD1, bifunctional nuclease in basal defense response 1 | 94.0376 | 28.071 | | -1.7442 |
| AT3G15750 | | Essential protein Yae1, N-terminal | 10.5282 | 3.14541 | | -1.7429 |
| AT2G24610 | | ATCNGC14, CNGC14, cyclic nucleotide-gated channel 14 | 61.2646 | 18.3104 | | -1.7424 |
| AT5G05090 | | Homeodomain-like superfamily protein | 4.6311 | 1.38431 | | -1.7422 |
| AT3G09010 | | Protein kinase superfamily protein | 7.15196 | 2.13839 | | -1.7418 |
| AT4G25560 | | AtMYB18, MYB18, myb domain protein 18 | 11.8331 | 3.54028 | | -1.7409 |
| AT3G29770 | | ATMES11, MES11, methyl esterase 11 | 3.83842 | 1.14905 | | -1.7401 |
| AT3G59350 | | Protein kinase superfamily protein | 469.079 | 140.433 | | -1.74 |
| AT1G03900 | | ATNAP4, NAP4, non-intrinsic ABC protein 4 | 256.122 | 76.8172 | | -1.7373 |
| AT2G31570 | | ATGPX2, GPX2, glutathione peroxidase 2 | 469.491 | 140.861 | | -1.7368 |
| AT3G27170 | | ATCLC-B, CLC-B, chloride channel B | 7.05942 | 2.11832 | | -1.7366 |
| AT3G61190 | | BAP1, BON association protein 1 | 124.091 | 37.2426 | | -1.7364 |
| AT3G56275 | | pseudogene of unknown protein | 29.6053 | 8.88538 | | -1.7364 |
| AT5G03030 | | Chaperone DnaJ-domain superfamily protein | 355.549 | 106.804 | | -1.7351 |
| AT3G62830 | | ATUXS2, AUD1, UXS2, NAD(P)-binding Rossmann-fold superfamily protein | 268.258 | 80.6796 | | -1.7333 |
| AT3G21070 | | ATNADK-1, NADK1, NAD kinase 1 | 293.348 | 88.3031 | | -1.7321 |
| AT4G12080 | | AHL1, ATAHL1, AT-hook motif nuclear-localized protein 1 | 107.577 | 32.389 | | -1.7318 |
| AT1G14860 | | atnudt18, NUDT18, nudix hydrolase homolog 18 | 201.252 | 60.6038 | | -1.7315 |
| AT1G76930 | | ATEXT1, ATEXT4, EXT1, EXT4, ORG5, extensin 4 | 221.946 | 66.8919 | | -1.7303 |
| AT1G51770 | | Core-2/I-branching beta-1,6-N-acetylglucosaminyltransferase family protein | 10.5971 | 3.19391 | | -1.7303 |
| AT1G11210 | | Protein of unknown function (DUF761) | 39.6188 | 11.9545 | | -1.7286 |
| AT5G24460 | | unknown protein | 24.1055 | 7.27495 | | -1.7284 |
| AT4G36110 | | SAUR-like auxin-responsive protein family | 13.3057 | 4.01619 | | -1.7281 |
| AT4G25170 | | Uncharacterised conserved protein (UCP012943) | 34.7135 | 10.4834 | | -1.7274 |
| AT3G07960 | | Phosphatidylinositol-4-phosphate 5-kinase family protein | 16.1538 | 4.87844 | | -1.7274 |
| AT1G12810 | | proline-rich family protein | 77.6718 | 23.5191 | | -1.7236 |
| AT2G47830 | | Cation efflux family protein | 20.8916 | 6.32791 | | -1.7231 |
| AT3G08650 | | ZIP metal ion transporter family | 93.353 | 28.3398 | | -1.7199 |
| AT1G70660 | | MMZ2, UEV1B, MMS ZWEI homologue 2 | 164.031 | 49.7965 | | -1.7199 |
| AT3G10300 | | Calcium-binding EF-hand family protein | 216.4 | 65.7395 | | -1.7189 |
| AT1G30690 | | Sec14p-like phosphatidylinositol transfer family protein | 161.157 | 48.9575 | | -1.7189 |
| AT3G55770 | | GATA type zinc finger transcription factor family protein | 243.928 | 74.1422 | | -1.7181 |
| AT5G67500 | | ATVDAC2, VDAC2, voltage dependent anion channel 2 | 375.643 | 114.277 | | -1.7168 |
| AT3G48530 | | KING1, SNF1-related protein kinase regulatory subunit gamma 1 | 350.623 | 106.701 | | -1.7164 |
| AT5G39320 | | UDP-glucose 6-dehydrogenase family protein | 496.073 | 151.022 | | -1.7158 |
| AT5G57710 | | Double Clp-N motif-containing P-loop nucleoside triphosphate hydrolases superfamily protein | 60.3405 | 18.3719 | | -1.7156 |
| AT2G28080 | | UDP-Glycosyltransferase superfamily protein | 22.7174 | 6.91992 | | -1.715 |
| AT4G17570 | | GATA26, GATA transcription factor 26 | 23.1125 | 7.05021 | | -1.7129 |
| AT3G11320 | | Nucleotide-sugar transporter family protein | 123.131 | 37.5714 | | -1.7125 |
| AT2G22560 | | Kinase interacting (KIP1-like) family protein | 57.6544 | 17.5945 | | -1.7123 |
| AT1G19940 | | AtGH9B5, GH9B5, glycosyl hydrolase 9B5 | 3.81656 | 1.16492 | | -1.712 |
| AT1G22985 | | Integrase-type DNA-binding superfamily protein | 269.88 | 82.3759 | | -1.712 |
| AT3G15740 | | RING/U-box superfamily protein | 33.6194 | 10.2635 | | -1.7118 |
| AT2G21045 | | Rhodanese/Cell cycle control phosphatase superfamily protein | 125.05 | 38.1773 | | -1.7117 |
| AT5G63160 | | BT1, BTB and TAZ domain protein 1 | 53.2257 | 16.2543 | | -1.7113 |
| AT2G46735 | | unknown protein | 12.6173 | 3.85361 | | -1.7111 |
| AT4G15610 | | Uncharacterised protein family (UPF0497) | 920.066 | 281.058 | | -1.7109 |
| AT1G74770 | | zinc ion binding | 16.8997 | 5.16404 | | -1.7104 |
| AT2G02480 | | STI, AAA-type ATPase family protein | 30.9978 | 9.47418 | | -1.7101 |
| AT4G27740 | | Yippee family putative zinc-binding protein | 20.0257 | 6.12532 | | -1.709 |
| AT1G26640 | | Amino acid kinase family protein | 68.4817 | 20.9517 | | -1.7087 |
| AT5G56170 | | LLG1, LORELEI-LIKE-GPI-ANCHORED PROTEIN 1 | 207.643 | 63.5561 | | -1.708 |
| AT2G17720 | | 2-oxoglutarate (2OG) and Fe(II)-dependent oxygenase superfamily protein | 506.32 | 155.026 | | -1.7075 |
| AT4G36515 | | unknown protein | 37.8598 | 11.6054 | | -1.7059 |
| AT4G25030 | | unknown protein | 394.861 | 121.057 | | -1.7057 |
| AT1G70590 | | F-box family protein | 144.528 | 44.3325 | | -1.7049 |
| AT2G21830 | | Cysteine/Histidine-rich C1 domain family protein | 6.39639 | 1.96239 | | -1.7047 |
| AT1G78680 | | ATGGH2, GGH2, gamma-glutamyl hydrolase 2 | 77.3343 | 23.7285 | | -1.7045 |
| AT1G62600 | | Flavin-binding monooxygenase family protein | 20.218 | 6.20364 | | -1.7045 |
| AT2G16660 | | Major facilitator superfamily protein | 478.088 | 146.703 | | -1.7044 |
| AT4G13890 | | EDA36, EDA37, SHM5, Pyridoxal phosphate (PLP)-dependent transferases superfamily protein | 18.6517 | 5.72393 | | -1.7042 |
| AT1G63800 | | UBC5, ubiquitin-conjugating enzyme 5 | 31.0328 | 9.52441 | | -1.7041 |
| AT3G63060 | | EDL3, EID1-like 3 | 41.6347 | 12.7883 | | -1.703 |
| AT5G43520 | | Cysteine/Histidine-rich C1 domain family protein | 125.817 | 38.662 | | -1.7023 |
| AT2G16920 | | PFU2, UBC23, ubiquitin-conjugating enzyme 23 | 32.144 | 9.88216 | | -1.7017 |
| AT3G20300 | | Protein of unknown function (DUF3537) | 158.605 | 48.7911 | | -1.7008 |
| AT5G62540 | | UBC3, ubiquitin-conjugating enzyme 3 | 474.591 | 146.037 | | -1.7004 |
| AT1G61740 | | Sulfite exporter TauE/SafE family protein | 88.217 | 27.1569 | | -1.6997 |
| AT5G40170 | | AtRLP54, RLP54, receptor like protein 54 | 7.53384 | 2.32045 | | -1.699 |
| AT1G78600 | | DBB3, LZF1, STH3, light-regulated zinc finger protein 1 | 92.8573 | 28.608 | | -1.6986 |
| AT4G12000 | | SNARE associated Golgi protein family | 253.892 | 78.3078 | | -1.697 |
| AT5G63450 | | CYP94B1, cytochrome P450, family 94, subfamily B, polypeptide 1 | 145.002 | 44.7236 | | -1.697 |
| AT2G02955 | | MEE12, maternal effect embryo arrest 12 | 6.71312 | 2.0716 | | -1.6962 |
| AT3G03050 | | ATCSLD3, CSLD3, KJK, cellulose synthase-like D3 | 215.2 | 66.4501 | | -1.6953 |
| AT4G39670 | | Glycolipid transfer protein (GLTP) family protein | 128.564 | 39.7069 | | -1.695 |
| AT4G01050 | | TROL, thylakoid rhodanese-like | 25.4304 | 7.85427 | | -1.695 |
| AT3G49845 | | unknown protein | 154.697 | 47.7893 | | -1.6947 |
| AT3G23170 | | unknown protein | 41.2768 | 12.7546 | | -1.6943 |
| AT1G03920 | | Protein kinase family protein | 7.26049 | 2.24393 | | -1.694 |
| AT2G20820 | | unknown protein | 683.525 | 211.331 | | -1.6935 |
| AT5G19230 | | Glycoprotein membrane precursor GPI-anchored | 986.846 | 305.293 | | -1.6926 |
| AT4G28710 | | ATXIH, XIH, Myosin family protein with Dil domain | 29.7934 | 9.22395 | | -1.6915 |
| AT3G51240 | | F3'H, F3H, TT6, flavanone 3-hydroxylase | 35.9907 | 11.1429 | | -1.6915 |
| AT1G58190 | | AtRLP9, RLP9, receptor like protein 9 | 42.1046 | 13.05 | | -1.6899 |
| AT4G16110 | | ARR2, RR2, response regulator 2 | 40.1997 | 12.4712 | | -1.6886 |
| AT4G32150 | | ATVAMP711, VAMP711, vesicle-associated membrane protein 711 | 241.923 | 75.0958 | | -1.6877 |
| AT4G23470 | | PLAC8 family protein | 223.15 | 69.2986 | | -1.6871 |
| AT5G10750 | | Protein of unknown function (DUF1336) | 23.6928 | 7.36058 | | -1.6866 |
| AT2G35800 | | mitochondrial substrate carrier family protein | 42.0854 | 13.0801 | | -1.6859 |
| AT4G28085 | | unknown protein | 69.4596 | 21.5945 | | -1.6855 |
| AT2G02710 | | PLP, PLPA, PLPB, PLPC, PAS/LOV protein B | 179.907 | 55.9555 | | -1.6849 |
| AT5G24010 | | Protein kinase superfamily protein | 1.75838 | 0.547495 | | -1.6833 |
| AT4G04450 | | AtWRKY42, WRKY42, WRKY family transcription factor | 6.54948 | 2.03994 | | -1.6829 |
| AT5G66650 | | Protein of unknown function (DUF607) | 262.465 | 81.8255 | | -1.6815 |
| AT1G68760 | | ATNUDT1, ATNUDX1, NUDX1, NUDX1, nudix hydrolase 1 | 13.8704 | 4.32462 | | -1.6814 |
| AT5G49560 | | Putative methyltransferase family protein | 12.7799 | 3.98493 | | -1.6813 |
| AT2G46170 | | Reticulon family protein | 289.426 | 90.2973 | | -1.6804 |
| AT1G28480 | | GRX480, roxy19, Thioredoxin superfamily protein | 340.939 | 106.395 | | -1.6801 |
| AT1G69530 | | AT-EXP1, ATEXP1, ATEXPA1, ATHEXP ALPHA 1.2, EXP1, EXPA1, expansin A1 | 125.073 | 39.0356 | | -1.6799 |
| AT1G07030 | | Mitochondrial substrate carrier family protein | 109.426 | 34.1605 | | -1.6796 |
| AT3G55840 | | Hs1pro-1 protein | 206.042 | 64.332 | | -1.6793 |
| AT2G14100 | | CYP705A13, cytochrome P450, family 705, subfamily A, polypeptide 13 | 25.3328 | 7.90979 | | -1.6793 |
| AT5G08770 | | unknown protein | 20.0073 | 6.24761 | | -1.6792 |
| AT4G00240 | | PLDBETA2, phospholipase D beta 2 | 13.0529 | 4.07637 | | -1.679 |
| AT2G23450 | | Protein kinase superfamily protein | 87.9509 | 27.5119 | | -1.6766 |
| AT5G14470 | | GHMP kinase family protein | 22.8497 | 7.14908 | | -1.6763 |
| AT3G63200 | | PLA IIIB, PLP9, PATATIN-like protein 9 | 20.9886 | 6.57083 | | -1.6755 |
| AT4G30170 | | Peroxidase family protein | 540.027 | 169.19 | | -1.6744 |
| AT2G20080 | | unknown protein | 62.7169 | 19.6498 | | -1.6743 |
| AT5G51990 | | CBF4, DREB1D, C-repeat-binding factor 4 | 18.3051 | 5.7372 | | -1.6738 |
| AT1G12140 | | FMO GS-OX5, flavin-monooxygenase glucosinolate S-oxygenase 5 | 20.6828 | 6.48309 | | -1.6737 |
| AT5G59550 | | zinc finger (C3HC4-type RING finger) family protein | 103.794 | 32.5617 | | -1.6725 |
| AT5G11970 | | Protein of unknown function (DUF3511) | 225.769 | 70.8363 | | -1.6723 |
| AT1G62990 | | IXR11, KNAT7, KNOTTED-like homeobox of Arabidopsis thaliana 7 | 25.6446 | 8.04854 | | -1.6719 |
| AT3G26100 | | Regulator of chromosome condensation (RCC1) family protein | 38.6651 | 12.1369 | | -1.6716 |
| AT1G76180 | | ERD14, Dehydrin family protein | 4562.78 | 1432.35 | | -1.6715 |
| AT5G52310 | | COR78, RD29A, | 618.896 | 194.762 | | -1.668 |
| AT3G01980 | | NAD(P)-binding Rossmann-fold superfamily protein | 28.8917 | 9.09412 | | -1.6677 |
| AT4G11150 | | emb2448, TUF, TUFF, VHA-E1, vacuolar ATP synthase subunit E1 | 1116.71 | 351.516 | | -1.6676 |
| AT3G07470 | | Protein of unknown function, DUF538 | 51.8664 | 16.3284 | | -1.6674 |
| AT5G38550 | | Mannose-binding lectin superfamily protein | 5.29845 | 1.66839 | | -1.6671 |
| AT1G55320 | | AAE18, acyl-activating enzyme 18 | 37.389 | 11.7766 | | -1.6667 |
| AT2G16700 | | ADF5, ATADF5, actin depolymerizing factor 5 | 19.9607 | 6.29187 | | -1.6656 |
| AT2G15760 | | Protein of unknown function (DUF1645) | 10.3456 | 3.2626 | | -1.6649 |
| AT5G62770 | | Protein of unknown function (DUF1645) | 42.4198 | 13.3982 | | -1.6627 |
| AT5G40720 | | Domain of unknown function (DUF23) | 3.65133 | 1.15367 | | -1.6622 |
| AT5G47390 | | myb-like transcription factor family protein | 55.2604 | 17.4632 | | -1.6619 |
| AT4G39090 | | RD19, RD19A, Papain family cysteine protease | 1309.12 | 413.94 | | -1.6611 |
| AT4G27470 | | ATRMA3, RMA3, RING membrane-anchor 3 | 80.1146 | 25.338 | | -1.6608 |
| AT3G11020 | | DREB2, DREB2B, DRE/CRT-binding protein 2B | 91.2854 | 28.8944 | | -1.6596 |
| AT5G62460 | | RING/FYVE/PHD zinc finger superfamily protein | 182.813 | 57.8837 | | -1.6591 |
| AT4G36988 | | CPuORF49, conserved peptide upstream open reading frame 49 | 462.778 | 146.7 | | -1.6575 |
| AT4G15290 | | ATCSLB05, ATCSLB5, CSLB05, Cellulose synthase family protein | 3.61547 | 1.14629 | | -1.6572 |
| AT1G67070 | | DIN9, PMI2, Mannose-6-phosphate isomerase, type I | 83.0979 | 26.3534 | | -1.6568 |
| AT5G49450 | | AtbZIP1, bZIP1, basic leucine-zipper 1 | 937.389 | 297.292 | | -1.6568 |
| AT1G19360 | | Nucleotide-diphospho-sugar transferase family protein | 149.934 | 47.574 | | -1.6561 |
| AT5G21940 | | unknown protein | 1111.76 | 353.043 | | -1.6549 |
| AT4G27280 | | Calcium-binding EF-hand family protein | 208.262 | 66.1948 | | -1.6536 |
| AT1G13390 | | unknown protein | 267.851 | 85.197 | | -1.6526 |
| AT1G70440 | | SRO3, similar to RCD one 3 | 23.7183 | 7.54799 | | -1.6518 |
| AT2G05260 | | alpha/beta-Hydrolases superfamily protein | 54.638 | 17.3988 | | -1.6509 |
| AT5G58560 | | Phosphatidate cytidylyltransferase family protein | 71.3247 | 22.7148 | | -1.6508 |
| AT2G39410 | | alpha/beta-Hydrolases superfamily protein | 21.486 | 6.84624 | | -1.65 |
| AT3G17020 | | Adenine nucleotide alpha hydrolases-like superfamily protein | 396.817 | 126.494 | | -1.6494 |
| AT5G47140 | | GATA27, GATA transcription factor 27 | 22.0108 | 7.02159 | | -1.6483 |
| AT5G49480 | | ATCP1, CP1, Ca2+-binding protein 1 | 684.413 | 218.337 | | -1.6483 |
| AT1G29120 | | Hydrolase-like protein family | 25.7469 | 8.2233 | | -1.6466 |
| AT2G25735 | | unknown protein | 61.8509 | 19.7673 | | -1.6457 |
| AT2G30362 | | other RNA | 351.438 | 112.469 | | -1.6438 |
| AT4G26470 | | Calcium-binding EF-hand family protein | 92.9078 | 29.7429 | | -1.6433 |
| AT1G05710 | | basic helix-loop-helix (bHLH) DNA-binding superfamily protein | 25.4665 | 8.15425 | | -1.643 |
| AT5G11770 | | NADH-ubiquinone oxidoreductase 20 kDa subunit, mitochondrial | 284.336 | 91.0663 | | -1.6426 |
| AT5G12140 | | ATCYS1, CYS1, cystatin-1 | 530.192 | 169.894 | | -1.6419 |
| AT1G15350 | | unknown protein | 130.385 | 41.801 | | -1.6412 |
| AT4G10560 | | MEE53, Cysteine/Histidine-rich C1 domain family protein | 2.06922 | 0.663673 | | -1.6406 |
| AT1G01720 | | ANAC002, | 995.076 | 319.221 | | -1.6403 |
| AT1G24170 | | GATL8, LGT9, Nucleotide-diphospho-sugar transferases superfamily protein | 87.9336 | 28.2139 | | -1.64 |
| AT3G15300 | | VQ motif-containing protein | 43.5715 | 13.9914 | | -1.6388 |
| AT3G51990 | | Protein kinase superfamily protein | 34.5659 | 11.1008 | | -1.6387 |
| AT5G15130 | | ATWRKY72, WRKY72, WRKY DNA-binding protein 72 | 35.4063 | 11.3726 | | -1.6384 |
| AT5G24100 | | Leucine-rich repeat protein kinase family protein | 7.23416 | 2.32379 | | -1.6384 |
| AT1G53580 | | ETHE1, GLX2-3, GLY3, glyoxalase II 3 | 196.427 | 63.1094 | | -1.6381 |
| AT1G68410 | | Protein phosphatase 2C family protein | 145.953 | 46.9068 | | -1.6376 |
| AT5G62020 | | AT-HSFB2A, HSFB2A, heat shock transcription factor B2A | 85.586 | 27.5297 | | -1.6364 |
| AT1G55190 | | PRA1.F2, PRA7, PRA1 (Prenylated rab acceptor) family protein | 38.2822 | 12.3188 | | -1.6358 |
| AT5G18660 | | PCB2, NAD(P)-binding Rossmann-fold superfamily protein | 3.52208 | 1.13344 | | -1.6357 |
| AT1G64470 | | Ubiquitin-like superfamily protein | 433.178 | 139.503 | | -1.6347 |
| AT1G13260 | | EDF4, RAV1, related to ABI3/VP1 1 | 105.171 | 33.873 | | -1.6345 |
| AT5G49440 | | unknown protein | 242.918 | 78.3335 | | -1.6328 |
| AT1G60190 | | ARM repeat superfamily protein | 66.8287 | 21.5536 | | -1.6325 |
| AT5G16510 | | Alpha-1,4-glucan-protein synthase family protein | 185.052 | 59.6975 | | -1.6322 |
| AT1G51070 | | bHLH115, basic helix-loop-helix (bHLH) DNA-binding superfamily protein | 115.714 | 37.3772 | | -1.6303 |
| AT1G21010 | | unknown protein | 83.9291 | 27.1343 | | -1.6291 |
| AT5G55400 | | Actin binding Calponin homology (CH) domain-containing protein | 95.8395 | 31.0082 | | -1.628 |
| AT3G57790 | | Pectin lyase-like superfamily protein | 16.0836 | 5.20771 | | -1.6269 |
| AT2G22970 | | SCPL11, serine carboxypeptidase-like 11 | 38.8072 | 12.5773 | | -1.6255 |
| AT2G45160 | | ATHAM1, HAM1, GRAS family transcription factor | 80.1101 | 25.9677 | | -1.6253 |
| AT2G38740 | | Haloacid dehalogenase-like hydrolase (HAD) superfamily protein | 110.145 | 35.7121 | | -1.6249 |
| AT3G45090 | | P-loop containing nucleoside triphosphate hydrolases superfamily protein | 38.604 | 12.5221 | | -1.6243 |
| AT2G47130 | | NAD(P)-binding Rossmann-fold superfamily protein | 45.6521 | 14.8097 | | -1.6241 |
| AT1G79450 | | ALIS5, ALA-interacting subunit 5 | 78.4252 | 25.443 | | -1.6241 |
| AT5G60680 | | Protein of unknown function, DUF584 | 740.661 | 240.743 | | -1.6213 |
| AT4G03115 | | Mitochondrial substrate carrier family protein | 5.10899 | 1.66066 | | -1.6213 |
| AT3G48400 | | Cysteine/Histidine-rich C1 domain family protein | 3.11462 | 1.01241 | | -1.6213 |
| AT4G23400 | | PIP1;5, PIP1D, plasma membrane intrinsic protein 1;5 | 71.5946 | 23.2746 | | -1.6211 |
| AT2G28120 | | Major facilitator superfamily protein | 4.19448 | 1.36367 | | -1.621 |
| AT1G27730 | | STZ, ZAT10, salt tolerance zinc finger | 121.752 | 39.6096 | | -1.62 |
| AT1G11410 | | S-locus lectin protein kinase family protein | 28.1055 | 9.1435 | | -1.62 |
| AT3G13910 | | Protein of unknown function (DUF3511) | 47.1269 | 15.3329 | | -1.6199 |
| AT3G24300 | | AMT1;3, ATAMT1;3, ammonium transporter 1;3 | 4.70156 | 1.53068 | | -1.619 |
| AT4G16680 | | P-loop containing nucleoside triphosphate hydrolases superfamily protein | 16.6191 | 5.4107 | | -1.619 |
| AT1G74450 | | Protein of unknown function (DUF793) | 98.7871 | 32.1647 | | -1.6189 |
| AT5G65200 | | ATPUB38, PUB38, plant U-box 38 | 15.0479 | 4.9046 | | -1.6174 |
| AT3G51490 | | TMT3, tonoplast monosaccharide transporter3 | 2.70395 | 0.881323 | | -1.6173 |
| AT3G54770 | | RNA-binding (RRM/RBD/RNP motifs) family protein | 43.7494 | 14.2642 | | -1.6169 |
| AT3G47833 | | unknown protein | 175.647 | 57.2911 | | -1.6163 |
| AT3G61990 | | S-adenosyl-L-methionine-dependent methyltransferases superfamily protein | 18.745 | 6.11433 | | -1.6162 |
| AT5G41110 | | unknown protein | 10.8544 | 3.54203 | | -1.6156 |
| AT4G19860 | | alpha/beta-Hydrolases superfamily protein | 151.628 | 49.5037 | | -1.6149 |
| AT4G20150 | | unknown protein | 745.936 | 243.56 | | -1.6148 |
| AT3G06050 | | ATPRXIIF, PRXIIF, peroxiredoxin IIF | 179.906 | 58.7523 | | -1.6145 |
| AT2G34350 | | Nodulin-like / Major Facilitator Superfamily protein | 128.657 | 42.0444 | | -1.6135 |
| AT1G45145 | | ATH5, ATTRX5, LIV1, TRX5, thioredoxin H-type 5 | 1928.02 | 630.427 | | -1.6127 |
| AT5G07820 | | Plant calmodulin-binding protein-related | 32.4623 | 10.6263 | | -1.6111 |
| AT4G08250 | | GRAS family transcription factor | 4.92292 | 1.61171 | | -1.6109 |
| AT1G10900 | | Phosphatidylinositol-4-phosphate 5-kinase family protein | 44.6163 | 14.609 | | -1.6107 |
| AT2G40890 | | CYP98A3, cytochrome P450, family 98, subfamily A, polypeptide 3 | 53.1148 | 17.3933 | | -1.6106 |
| AT3G47670 | | Plant invertase/pectin methylesterase inhibitor superfamily protein | 58.0724 | 19.0232 | | -1.6101 |
| AT5G01450 | | RING/U-box superfamily protein | 54.1153 | 17.7375 | | -1.6092 |
| AT3G52800 | | A20/AN1-like zinc finger family protein | 495.607 | 162.456 | | -1.6091 |
| AT1G19020 | | unknown protein | 472.598 | 155.128 | | -1.6072 |
| AT1G20440 | | AtCOR47, COR47, RD17, cold-regulated 47 | 2699.7 | 886.206 | | -1.6071 |
| AT3G13690 | | Protein kinase protein with adenine nucleotide alpha hydrolases-like domain | 19.7687 | 6.49005 | | -1.6069 |
| AT5G24290 | | Vacuolar iron transporter (VIT) family protein | 92.394 | 30.3502 | | -1.6061 |
| AT5G55180 | | O-Glycosyl hydrolases family 17 protein | 875.051 | 287.572 | | -1.6054 |
| AT1G79970 | | unknown protein | 70.4907 | 23.1748 | | -1.6049 |
| AT4G39540 | | ATSK2, SK2, shikimate kinase 2 | 53.4598 | 17.5885 | | -1.6038 |
| AT3G63080 | | ATGPX5, GPX5, MEE42, glutathione peroxidase 5 | 69.9564 | 23.021 | | -1.6035 |
| AT5G15170 | | TDP1, tyrosyl-DNA phosphodiesterase-related | 10.3452 | 3.40474 | | -1.6034 |
| AT3G62770 | | AtATG18a, Transducin/WD40 repeat-like superfamily protein | 202.579 | 66.6879 | | -1.603 |
| AT1G19220 | | ARF11, ARF19, IAA22, auxin response factor 19 | 26.3406 | 8.67235 | | -1.6028 |
| AT5G10930 | | CIPK5, SnRK3.24, CBL-interacting protein kinase 5 | 18.4612 | 6.07835 | | -1.6027 |
| AT3G15356 | | Legume lectin family protein | 262.734 | 86.5204 | | -1.6025 |
| AT3G54880 | | unknown protein | 287.057 | 94.5844 | | -1.6017 |
| AT1G03870 | | FLA9, FASCICLIN-like arabinoogalactan 9 | 165.099 | 54.4052 | | -1.6015 |
| AT4G05070 | | Wound-responsive family protein | 530.071 | 174.766 | | -1.6008 |
| AT2G42310 | | unknown protein | 337.244 | 111.192 | | -1.6007 |
| AT4G26080 | | ABI1, AtABI1, Protein phosphatase 2C family protein | 730.496 | 241.738 | | -1.5954 |
| AT3G15210 | | ATERF-4, ATERF4, ERF4, RAP2.5, ethylene responsive element binding factor 4 | 307.896 | 101.922 | | -1.595 |
| AT2G46600 | | Calcium-binding EF-hand family protein | 254.759 | 84.4076 | | -1.5937 |
| AT4G32530 | | ATPase, F0/V0 complex, subunit C protein | 266.152 | 88.1837 | | -1.5937 |
| AT5G46050 | | ATPTR3, PTR3, peptide transporter 3 | 11.2631 | 3.73381 | | -1.5929 |
| AT4G20300 | | Protein of unknown function (DUF1639) | 61.1596 | 20.2828 | | -1.5923 |
| AT5G27920 | | F-box family protein | 71.768 | 23.8018 | | -1.5923 |
| AT3G61580 | | Fatty acid/sphingolipid desaturase | 173.588 | 57.601 | | -1.5915 |
| AT4G28770 | | Tetraspanin family protein | 162.082 | 53.7877 | | -1.5914 |
| AT1G18270 | | ketose-bisphosphate aldolase class-II family protein | 57.5176 | 19.0897 | | -1.5912 |
| AT3G15115 | | unknown protein | 30.0093 | 9.96011 | | -1.5912 |
| AT5G07460 | | ATMSRA2, PMSR2, peptidemethionine sulfoxide reductase 2 | 777.38 | 258.102 | | -1.5907 |
| AT4G23710 | | VAG2, VATG2, VHA-G2, vacuolar ATP synthase subunit G2 | 608.332 | 202.057 | | -1.5901 |
| AT3G21680 | | unknown protein | 1032.79 | 343.065 | | -1.59 |
| AT5G64430 | | Octicosapeptide/Phox/Bem1p family protein | 112.044 | 37.22 | | -1.5899 |
| AT5G63510 | | GAMMA CAL1, gamma carbonic anhydrase like 1 | 167.996 | 55.8076 | | -1.5899 |
| AT3G52760 | | Integral membrane Yip1 family protein | 23.5866 | 7.83734 | | -1.5895 |
| AT5G49680 | | KIP, Golgi-body localisation protein domain ;RNA pol II promoter Fmp27 protein domain | 34.1874 | 11.3609 | | -1.5894 |
| AT5G40690 | | CONTAINS InterPro DOMAIN/s: EF-Hand 1, calcium-binding site (InterPro:IPR018247) | 92.644 | 30.8061 | | -1.5885 |
| AT5G41080 | | PLC-like phosphodiesterases superfamily protein | 349.839 | 116.471 | | -1.5867 |
| AT3G15450 | | Aluminium induced protein with YGL and LRDR motifs | 3385.68 | 1127.62 | | -1.5862 |
| AT3G11780 | | MD-2-related lipid recognition domain-containing protein / ML domain-containing protein | 170.826 | 56.9062 | | -1.5859 |
| AT5G58670 | | ATPLC, ATPLC1, PLC1, PLC1, phospholipase C1 | 28.4206 | 9.47433 | | -1.5848 |
| AT3G22910 | | ATPase E1-E2 type family protein / haloacid dehalogenase-like hydrolase family protein | 252.223 | 84.0932 | | -1.5846 |
| AT1G22410 | | Class-II DAHP synthetase family protein | 358.604 | 119.588 | | -1.5843 |
| AT3G50060 | | MYB77, myb domain protein 77 | 85.2814 | 28.4441 | | -1.5841 |
| AT5G01410 | | ATPDX1, ATPDX1.3, PDX1, PDX1.3, RSR4, Aldolase-type TIM barrel family protein | 668.851 | 223.114 | | -1.5839 |
| AT4G03520 | | ATHM2, Thioredoxin superfamily protein | 156.213 | 52.1189 | | -1.5836 |
| AT5G44060 | | unknown protein | 187.904 | 62.7145 | | -1.5831 |
| AT5G03460 | | unknown protein | 305.197 | 101.874 | | -1.583 |
| AT1G77500 | | Protein of unknown function (DUF630 and DUF632) | 50.1367 | 16.7365 | | -1.5829 |
| AT2G30230 | | unknown protein | 73.2051 | 24.444 | | -1.5825 |
| AT5G15330 | | ATSPX4, SPX4, SPX domain gene 4 | 32.2728 | 10.7847 | | -1.5813 |
| AT4G14980 | | Cysteine/Histidine-rich C1 domain family protein | 12.0079 | 4.01286 | | -1.5813 |
| AT5G04020 | | calmodulin binding | 34.8892 | 11.6601 | | -1.5812 |
| AT2G37170 | | PIP2;2, PIP2B, plasma membrane intrinsic protein 2 | 216.435 | 72.4597 | | -1.5787 |
| AT2G38800 | | Plant calmodulin-binding protein-related | 85.0766 | 28.4919 | | -1.5782 |
| AT5G59490 | | Haloacid dehalogenase-like hydrolase (HAD) superfamily protein | 496.028 | 166.133 | | -1.5781 |
| AT1G43160 | | RAP2.6, related to AP2 6 | 1349.35 | 452.746 | | -1.5755 |
| AT3G11330 | | PIRL9, plant intracellular ras group-related LRR 9 | 130.341 | 43.7582 | | -1.5747 |
| AT5G16600 | | AtMYB43, MYB43, myb domain protein 43 | 8.47145 | 2.84493 | | -1.5742 |
| AT5G65690 | | PCK2, PEPCK, phosphoenolpyruvate carboxykinase 2 | 711.095 | 238.883 | | -1.5737 |
| AT5G19200 | | NAD(P)-binding Rossmann-fold superfamily protein | 24.013 | 8.06916 | | -1.5733 |
| AT3G03280 | | unknown protein | 139.049 | 46.7287 | | -1.5732 |
| AT5G18270 | | ANAC087, Arabidopsis NAC domain containing protein 87 | 85.1067 | 28.603 | | -1.5731 |
| AT4G04840 | | ATMSRB6, MSRB6, methionine sulfoxide reductase B6 | 120.257 | 40.4225 | | -1.5729 |
| AT5G58620 | | zinc finger (CCCH-type) family protein | 30.5813 | 10.2916 | | -1.5712 |
| AT3G11410 | | AHG3, ATPP2CA, PP2CA, protein phosphatase 2CA | 114.762 | 38.6339 | | -1.5707 |
| AT4G36520 | | Chaperone DnaJ-domain superfamily protein | 35.8293 | 12.0626 | | -1.5706 |
| AT5G64400 | | CONTAINS InterPro DOMAIN/s: CHCH (InterPro:IPR010625) | 1193.7 | 401.981 | | -1.5702 |
| AT1G76900 | | AtTLP1, TLP1, tubby like protein 1 | 72.8638 | 24.5432 | | -1.5699 |
| AT3G03160 | | function unknown | 176.314 | 59.4173 | | -1.5692 |
| AT2G19450 | | ABX45, AS11, ATDGAT, DGAT1, RDS1, TAG1, membrane bound O-acyl transferase (MBOAT) family protein | 78.936 | 26.608 | | -1.5688 |
| AT3G27540 | | beta-1,4-N-acetylglucosaminyltransferase family protein | 8.59467 | 2.89814 | | -1.5683 |
| AT1G73340 | | Cytochrome P450 superfamily protein | 40.8101 | 13.7892 | | -1.5654 |
| AT4G02380 | | AtLEA5, SAG21, senescence-associated gene 21 | 1491.37 | 504.116 | | -1.5648 |
| AT5G04170 | | Calcium-binding EF-hand family protein | 163.288 | 55.2346 | | -1.5638 |
| AT1G21930 | | unknown protein | 63.0837 | 21.3822 | | -1.5609 |
| AT4G29260 | | HAD superfamily, subfamily IIIB acid phosphatase | 27.8669 | 9.45206 | | -1.5599 |
| AT1G20110 | | RING/FYVE/PHD zinc finger superfamily protein | 198.756 | 67.4318 | | -1.5595 |
| AT1G51140 | | basic helix-loop-helix (bHLH) DNA-binding superfamily protein | 48.6661 | 16.5139 | | -1.5592 |
| AT4G27652 | | unknown protein | 566.795 | 192.382 | | -1.5589 |
| AT1G11800 | | endonuclease/exonuclease/phosphatase family protein | 10.307 | 3.50259 | | -1.5571 |
| AT2G33320 | | Calcium-dependent lipid-binding (CaLB domain) family protein | 9.96399 | 3.38615 | | -1.5571 |
| AT5G11790 | | NDL2, N-MYC downregulated-like 2 | 94.9003 | 32.2797 | | -1.5558 |
| AT5G64552 | | CPuORF22, conserved peptide upstream open reading frame 22 | 4.98945 | 1.69732 | | -1.5556 |
| AT3G46640 | | LUX, PCL1, Homeodomain-like superfamily protein | 160.418 | 54.5747 | | -1.5555 |
| AT1G08190 | | ATVAM2, ATVPS41, VAM2, VPS41, ZIP2, vacuolar protein sorting 41 | 93.7789 | 31.9353 | | -1.5541 |
| AT2G43850 | | Integrin-linked protein kinase family | 8.51246 | 2.90163 | | -1.5527 |
| AT1G35580 | | CINV1, cytosolic invertase 1 | 423.926 | 144.599 | | -1.5518 |
| AT5G19550 | | AAT2, ASP2, aspartate aminotransferase 2 | 385.198 | 131.399 | | -1.5516 |
| AT2G14750 | | AKN1, APK, APK1, ATAKN1, APS kinase | 86.0834 | 29.3909 | | -1.5504 |
| AT5G22940 | | F8H, FRA8 homolog | 8.3148 | 2.8421 | | -1.5487 |
| AT5G15870 | | glycosyl hydrolase family 81 protein | 209.111 | 71.5251 | | -1.5478 |
| AT5G56550 | | ATOXS3, OXS3, oxidative stress 3 | 273.422 | 93.5739 | | -1.547 |
| AT2G02970 | | GDA1/CD39 nucleoside phosphatase family protein | 61.9371 | 21.1999 | | -1.5467 |
| AT3G28710 | | ATPase, V0/A0 complex, subunit C/D | 247.945 | 84.908 | | -1.5461 |
| AT3G56440 | | ATATG18D, ATG18D, homolog of yeast autophagy 18 (ATG18) D | 26.0424 | 8.91905 | | -1.5459 |
| AT3G54930 | | Protein phosphatase 2A regulatory B subunit family protein | 4.82926 | 1.65563 | | -1.5444 |
| AT1G19450 | | Major facilitator superfamily protein | 174.887 | 59.9677 | | -1.5442 |
| AT3G26240 | | Cysteine/Histidine-rich C1 domain family protein | 3.43686 | 1.17964 | | -1.5428 |
| AT3G44310 | | ATNIT1, NIT1, NITI, nitrilase 1 | 16.4418 | 5.64391 | | -1.5426 |
| AT4G30010 | | unknown protein | 1013.58 | 348.027 | | -1.5422 |
| AT4G37370 | | CYP81D8, cytochrome P450, family 81, subfamily D, polypeptide 8 | 10.5756 | 3.63136 | | -1.5422 |
| AT3G30390 | | Transmembrane amino acid transporter family protein | 259.037 | 89.0239 | | -1.5409 |
| AT3G53410 | | RING/U-box superfamily protein | 18.7754 | 6.45322 | | -1.5408 |
| AT2G36890 | | ATMYB38, BIT1, MYB38, RAX2, Duplicated homeodomain-like superfamily protein | 55.0995 | 18.9423 | | -1.5404 |
| AT4G34230 | | ATCAD5, CAD-5, CAD5, cinnamyl alcohol dehydrogenase 5 | 258.739 | 89.1349 | | -1.5374 |
| AT3G25655 | | IDL1, inflorescence deficient in abscission (IDA)-like 1 | 56.6149 | 19.5074 | | -1.5372 |
| AT5G12170 | | CLT3, CRT (chloroquine-resistance transporter)-like transporter 3 | 14.2251 | 4.90272 | | -1.5368 |
| AT4G13510 | | AMT1;1, ATAMT1, ATAMT1;1, ammonium transporter 1;1 | 220.847 | 76.1917 | | -1.5353 |
| AT5G01850 | | Protein kinase superfamily protein | 12.4522 | 4.30297 | | -1.533 |
| AT3G51250 | | Senescence/dehydration-associated protein-related | 31.5825 | 10.9138 | | -1.533 |
| AT1G71000 | | Chaperone DnaJ-domain superfamily protein | 277.718 | 95.9805 | | -1.5328 |
| AT1G44170 | | ALDH3H1, ALDH4, aldehyde dehydrogenase 3H1 | 64.6943 | 22.3794 | | -1.5315 |
| AT5G59613 | | unknown protein | 560.233 | 193.887 | | -1.5308 |
| AT4G21160 | | AGD12, ZAC, Calcium-dependent ARF-type GTPase activating protein family | 83.2264 | 28.8137 | | -1.5303 |
| AT4G38810 | | Calcium-binding EF-hand family protein | 447.283 | 155.03 | | -1.5287 |
| AT5G47740 | | Adenine nucleotide alpha hydrolases-like superfamily protein | 113.395 | 39.317 | | -1.5281 |
| AT2G20750 | | ATEXPB1, ATHEXP BETA 1.5, EXPB1, expansin B1 | 20.3547 | 7.06312 | | -1.527 |
| AT2G35710 | | Nucleotide-diphospho-sugar transferases superfamily protein | 33.1764 | 11.5141 | | -1.5268 |
| AT4G34410 | | RRTF1, redox responsive transcription factor 1 | 193.39 | 67.1881 | | -1.5252 |
| AT1G68820 | | Transmembrane Fragile-X-F-associated protein | 63.7703 | 22.159 | | -1.525 |
| AT4G36500 | | unknown protein | 1344.94 | 467.384 | | -1.5249 |
| AT1G49300 | | ATRAB7, ATRABG3E, RABG3E, RAB GTPase homolog G3E | 170.033 | 59.0985 | | -1.5246 |
| AT1G19025 | | DNA repair metallo-beta-lactamase family protein | 43.1803 | 15.0099 | | -1.5245 |
| AT3G11700 | | FLA18, FASCICLIN-like arabinogalactan protein 18 precursor | 107.844 | 37.5015 | | -1.5239 |
| AT1G68300 | | Adenine nucleotide alpha hydrolases-like superfamily protein | 124.142 | 43.2172 | | -1.5223 |
| AT5G46890 | | Bifunctional inhibitor/lipid-transfer protein/seed storage 2S albumin superfamily protein | 202.875 | 70.645 | | -1.5219 |
| AT4G02120 | | CTP synthase family protein | 23.1412 | 8.06052 | | -1.5215 |
| AT3G56950 | | SIP2, SIP2;1, small and basic intrinsic protein 2;1 | 61.1554 | 21.3058 | | -1.5212 |
| AT5G07920 | | ATDGK1, DGK1, diacylglycerol kinase1 | 49.2252 | 17.1494 | | -1.5212 |
| AT5G25265 | | unknown protein | 100.183 | 34.9042 | | -1.5212 |
| AT2G45670 | | calcineurin B subunit-related | 187.439 | 65.3763 | | -1.5196 |
| AT2G39950 | | unknown protein | 63.0984 | 22.0124 | | -1.5193 |
| AT2G29430 | | Family of unknown function (DUF572) | 112.114 | 39.1246 | | -1.5188 |
| AT5G22300 | | AtNIT4, NIT4, nitrilase 4 | 9.08484 | 3.17038 | | -1.5188 |
| AT1G01440 | | Protein of unknown function (DUF3133) | 18.0843 | 6.3126 | | -1.5184 |
| AT5G55060 | | unknown protein | 45.3203 | 15.8217 | | -1.5183 |
| AT4G23885 | | unknown protein | 271.443 | 94.7762 | | -1.5181 |
| AT1G66260 | | RNA-binding (RRM/RBD/RNP motifs) family protein | 413.437 | 144.388 | | -1.5177 |
| AT3G20340 | | Expression of the gene is downregulated in the presence of paraquat, an inducer of photoxidative stress. | 434.051 | 151.657 | | -1.5171 |
| AT2G39530 | | Uncharacterised protein family (UPF0497) | 97.5468 | 34.0835 | | -1.517 |
| AT4G36040 | | Chaperone DnaJ-domain superfamily protein | 1082.74 | 378.673 | | -1.5157 |
| AT3G18950 | | Transducin/WD40 repeat-like superfamily protein | 146.545 | 51.2526 | | -1.5157 |
| AT5G66400 | | ATDI8, RAB18, Dehydrin family protein | 108.849 | 38.0731 | | -1.5155 |
| AT3G17710 | | F-box and associated interaction domains-containing protein | 21.2893 | 7.45126 | | -1.5146 |
| AT1G73920 | | alpha/beta-Hydrolases superfamily protein | 327.045 | 114.536 | | -1.5137 |
| AT3G48360 | | ATBT2, BT2, BTB and TAZ domain protein 2 | 72.2546 | 25.3099 | | -1.5134 |
| AT5G49360 | | ATBXL1, BXL1, beta-xylosidase 1 | 9.19674 | 3.22218 | | -1.5131 |
| AT1G18210 | | Calcium-binding EF-hand family protein | 279.4 | 97.969 | | -1.5119 |
| AT2G16380 | | Sec14p-like phosphatidylinositol transfer family protein | 7.83655 | 2.74814 | | -1.5118 |
| AT1G03090 | | MCCA, methylcrotonyl-CoA carboxylase alpha chain, mitochondrial / 3-methylcrotonyl-CoA carboxylase 1 (MCCA) | 115.017 | 40.3469 | | -1.5113 |
| AT3G24180 | | Beta-glucosidase, GBA2 type family protein | 176.934 | 62.0748 | | -1.5111 |
| AT4G03460 | | Ankyrin repeat family protein | 6.9382 | 2.437 | | -1.5095 |
| AT5G04770 | | ATCAT6, CAT6, cationic amino acid transporter 6 | 56.1107 | 19.7316 | | -1.5078 |
| AT5G64370 | | BETA-UP, PYD3, beta-ureidopropionase | 114.793 | 40.408 | | -1.5063 |
| AT5G59960 | | unknown protein | 79.6069 | 28.0324 | | -1.5058 |
| AT2G36310 | | URH1, uridine-ribohydrolase 1 | 316.913 | 111.615 | | -1.5056 |
| AT4G35500 | | Protein kinase superfamily protein | 49.6345 | 17.482 | | -1.5055 |
| AT3G10660 | | ATCPK2, CPK2, calmodulin-domain protein kinase cdpk isoform 2 | 41.8031 | 14.7242 | | -1.5054 |
| AT2G33040 | | ATP3, gamma subunit of Mt ATP synthase | 796.22 | 280.518 | | -1.5051 |
| AT2G35980 | | ATNHL10, NHL10, YLS9, Late embryogenesis abundant (LEA) hydroxyproline-rich glycoprotein family | 107.34 | 37.8361 | | -1.5044 |
| AT4G28550 | | Ypt/Rab-GAP domain of gyp1p superfamily protein | 20.7639 | 7.32025 | | -1.5041 |
| AT1G63840 | | RING/U-box superfamily protein | 159.136 | 56.144 | | -1.5031 |
| AT5G17280 | | CONTAINS InterPro DOMAIN/s: Oxidoreductase-like, N-terminal (InterPro:IPR019180) | 87.1239 | 30.7571 | | -1.5022 |
| AT3G07350 | | Protein of unknown function (DUF506) | 83.8947 | 29.6186 | | -1.5021 |
| AT2G28430 | | unknown protein | 77.1847 | 27.268 | | -1.5011 |
| AT2G42270 | | U5 small nuclear ribonucleoprotein helicase | 72.8414 | 25.7423 | | -1.5006 |
| AT1G52760 | | LysoPL2, lysophospholipase 2 | 138.289 | 48.888 | | -1.5001 |
| AT2G16980 | | Major facilitator superfamily protein | 8.17464 | 2.89167 | | -1.4993 |
| AT1G62300 | | ATWRKY6, WRKY6, WRKY family transcription factor | 188.38 | 66.6406 | | -1.4992 |
| AT5G54300 | | Protein of unknown function (DUF761) | 102.494 | 36.2608 | | -1.4991 |
| AT5G17190 | | function unknown | 128.571 | 45.4935 | | -1.4988 |
| AT3G01650 | | RGLG1, RING domain ligase1 | 116.495 | 41.2241 | | -1.4987 |
| AT1G70490 | | ARFA1D, ATARFA1D, Ras-related small GTP-binding family protein | 594.299 | 210.374 | | -1.4982 |
| AT4G34490 | | ATCAP1, CAP 1, CAP1, cyclase associated protein 1 | 167.472 | 59.2951 | | -1.4979 |
| AT1G10050 | | glycosyl hydrolase family 10 protein / carbohydrate-binding domain-containing protein | 62.7757 | 22.2312 | | -1.4976 |
| AT2G26260 | | 3BETAHSD/D2, AT3BETAHSD/D2, 3beta-hydroxysteroid-dehydrogenase/decarboxylase isoform 2 | 26.2137 | 9.2883 | | -1.4968 |
| AT2G01680 | | Ankyrin repeat family protein | 50.8468 | 18.0227 | | -1.4963 |
| AT1G20260 | | ATPase, V1 complex, subunit B protein | 515.591 | 182.769 | | -1.4962 |
| AT5G03290 | | IDH-V, isocitrate dehydrogenase V | 301.535 | 106.941 | | -1.4955 |
| AT2G21850 | | Cysteine/Histidine-rich C1 domain family protein | 19.14 | 6.79004 | | -1.4951 |
| AT4G22910 | | CCS52A1, FZR2, FIZZY-related 2 | 21.9113 | 7.7863 | | -1.4927 |
| AT1G66500 | | Pre-mRNA cleavage complex II | 134.628 | 47.8711 | | -1.4918 |
| AT3G59710 | | NAD(P)-binding Rossmann-fold superfamily protein | 17.1201 | 6.08803 | | -1.4917 |
| AT1G67570 | | Protein of unknown function (DUF3537) | 13.0727 | 4.64875 | | -1.4916 |
| AT4G26130 | | unknown protein | 8.89271 | 3.16552 | | -1.4902 |
| AT1G54320 | | LEM3 (ligand-effect modulator 3) family protein / CDC50 family protein | 162.565 | 57.8693 | | -1.4901 |
| AT3G50280 | | HXXXD-type acyl-transferase family protein | 6.97949 | 2.48473 | | -1.49 |
| AT1G56300 | | Chaperone DnaJ-domain superfamily protein | 34.5954 | 12.3162 | | -1.49 |
| AT4G32070 | | Octicosapeptide/Phox/Bem1p (PB1) domain-containing protein / tetratricopeptide repeat (TPR)-containing protein | 189.746 | 67.5944 | | -1.4891 |
| AT3G15200 | | Tetratricopeptide repeat (TPR)-like superfamily protein | 57.6436 | 20.5358 | | -1.489 |
| AT4G09570 | | ATCPK4, CPK4, calcium-dependent protein kinase 4 | 227.361 | 81.021 | | -1.4886 |
| AT5G54470 | | B-box type zinc finger family protein | 39.1342 | 13.9481 | | -1.4884 |
| AT5G57830 | | Protein of unknown function, DUF593 | 154.744 | 55.1579 | | -1.4883 |
| AT2G02310 | | AtPP2-B6, PP2-B6, phloem protein 2-B6 | 37.3252 | 13.3183 | | -1.4867 |
| AT4G02370 | | Protein of unknown function, DUF538 | 107.765 | 38.4737 | | -1.486 |
| AT3G02460 | | Ypt/Rab-GAP domain of gyp1p superfamily protein | 21.8039 | 7.78703 | | -1.4854 |
| AT3G51770 | | ATEOL1, ETO1, tetratricopeptide repeat (TPR)-containing protein | 54.9882 | 19.6388 | | -1.4854 |
| AT5G24110 | | ATWRKY30, WRKY30, WRKY DNA-binding protein 30 | 31.0568 | 11.0955 | | -1.4849 |
| AT4G35320 | | unknown protein | 177.516 | 63.4578 | | -1.4841 |
| AT3G54130 | | Josephin family protein | 308.151 | 110.16 | | -1.484 |
| AT1G47740 | | PPPDE putative thiol peptidase family protein | 72.1497 | 25.7954 | | -1.4839 |
| AT3G24070 | | Zinc knuckle (CCHC-type) family protein | 80.1151 | 28.6435 | | -1.4839 |
| AT1G80300 | | ATNTT1, NTT1, nucleotide transporter 1 | 23.7549 | 8.4935 | | -1.4838 |
| AT5G15320 | | unknown protein | 381.752 | 136.535 | | -1.4834 |
| AT3G04080 | | APY1, ATAPY1, apyrase 1 | 44.454 | 15.9001 | | -1.4833 |
| AT5G06390 | | FLA17, FASCICLIN-like arabinogalactan protein 17 precursor | 46.8757 | 16.7675 | | -1.4832 |
| AT5G03190 | | CPUORF47, conserved peptide upstream open reading frame 47 | 42.9977 | 15.3871 | | -1.4825 |
| AT4G36380 | | ROT3, Cytochrome P450 superfamily protein | 5.77683 | 2.06879 | | -1.4815 |
| AT5G55290 | | ATPase, V0 complex, subunit E | 252.328 | 90.4295 | | -1.4804 |
| AT4G24960 | | ATHVA22D, HVA22D, HVA22 homologue D | 1190.49 | 426.809 | | -1.4799 |
| AT3G26040 | | HXXXD-type acyl-transferase family protein | 12.1016 | 4.33962 | | -1.4796 |
| AT4G29480 | | Mitochondrial ATP synthase subunit G protein | 460.914 | 165.352 | | -1.479 |
| AT3G27190 | | UKL2, uridine kinase-like 2 | 76.997 | 27.6417 | | -1.478 |
| AT3G15620 | | UVR3, DNA photolyase family protein | 10.0675 | 3.61663 | | -1.477 |
| AT3G17780 | | function unknown | 254.07 | 91.3394 | | -1.4759 |
| AT5G40390 | | SIP1, Raffinose synthase family protein | 330.234 | 118.722 | | -1.4759 |
| AT3G27100 | | function unknown | 33.5851 | 12.078 | | -1.4754 |
| AT3G06500 | | Plant neutral invertase family protein | 637.654 | 229.393 | | -1.475 |
| AT4G35790 | | ATPLDDELTA, PLDDELTA, phospholipase D delta | 154.161 | 55.5105 | | -1.4736 |
| AT5G04860 | | unknown protein | 57.9319 | 20.8617 | | -1.4735 |
| AT3G15640 | | Rubredoxin-like superfamily protein | 231.597 | 83.4077 | | -1.4734 |
| AT3G60690 | | SAUR-like auxin-responsive protein family | 75.3346 | 27.1516 | | -1.4723 |
| AT3G11850 | | Protein of unknown function, DUF593 | 14.3726 | 5.18043 | | -1.4722 |
| AT5G10650 | | RING/U-box superfamily protein | 62.7646 | 22.6341 | | -1.4715 |
| AT1G76185 | | unknown protein | 46.3039 | 16.7009 | | -1.4712 |
| AT3G60200 | | unknown protein | 45.9345 | 16.5708 | | -1.4709 |
| AT1G66600 | | ABO3, ATWRKY63, WRKY63, ABA overly sensitive mutant 3 | 27.7772 | 10.0215 | | -1.4708 |
| AT3G47980 | | Integral membrane HPP family protein | 13.7067 | 4.94974 | | -1.4695 |
| AT3G14395 | | unknown protein | 40.2402 | 14.5332 | | -1.4693 |
| AT1G30700 | | FAD-binding Berberine family protein | 432.923 | 156.433 | | -1.4686 |
| AT5G56340 | | ATCRT1, RING/U-box superfamily protein | 142.462 | 51.493 | | -1.4681 |
| AT1G58110 | | Basic-leucine zipper (bZIP) transcription factor family protein | 41.7993 | 15.1132 | | -1.4677 |
| AT5G61228 | | CPuORF15, conserved peptide upstream open reading frame 15 | 122.494 | 44.2993 | | -1.4674 |
| AT1G78900 | | VHA-A, vacuolar ATP synthase subunit A | 972.528 | 351.844 | | -1.4668 |
| AT4G20780 | | CML42, calmodulin like 42 | 127.502 | 46.141 | | -1.4664 |
| AT4G36750 | | Quinone reductase family protein | 87.8109 | 31.7811 | | -1.4662 |
| AT4G16100 | | Protein of unknown function (DUF789) | 26.1483 | 9.46691 | | -1.4658 |
| AT1G05300 | | ZIP5, zinc transporter 5 precursor | 43.5397 | 15.7696 | | -1.4652 |
| AT2G40970 | | MYBC1, Homeodomain-like superfamily protein | 48.9457 | 17.7576 | | -1.4627 |
| AT1G27200 | | Domain of unknown function (DUF23) | 46.2442 | 16.8087 | | -1.4601 |
| AT4G30260 | | Integral membrane Yip1 family protein | 139.373 | 50.6832 | | -1.4594 |
| AT1G66880 | | Protein kinase superfamily protein | 28.1356 | 10.2333 | | -1.4591 |
| AT2G44840 | | ATERF13, EREBP, ERF13, ethylene-responsive element binding factor 13 | 184.521 | 67.1459 | | -1.4584 |
| AT2G03340 | | WRKY3, WRKY DNA-binding protein 3 | 49.7524 | 18.1149 | | -1.4576 |
| AT5G19855 | | Chaperonin-like RbcX protein | 27.8964 | 10.158 | | -1.4575 |
| AT5G01820 | | ATCIPK14, ATSR1, CIPK14, SnRK3.15, SR1, serine/threonine protein kinase 1 | 383.094 | 139.568 | | -1.4567 |
| AT5G52290 | | SHOC1, shortage in chiasmata 1 | 1.7618 | 0.641892 | | -1.4566 |
| AT3G06780 | | glycine-rich protein | 105.24 | 38.3617 | | -1.4559 |
| AT3G16240 | | AQP1,delta tonoplast integral protein | 916.7 | 334.163 | | -1.4559 |
| AT1G80570 | | RNI-like superfamily protein | 10.4467 | 3.80956 | | -1.4554 |
| AT5G51570 | | SPFH/Band 7/PHB domain-containing membrane-associated protein family | 96.5853 | 35.2336 | | -1.4549 |
| AT2G27690 | | CYP94C1, cytochrome P450, family 94, subfamily C, polypeptide 1 | 60.1014 | 21.9249 | | -1.4548 |
| AT4G38900 | | Basic-leucine zipper (bZIP) transcription factor family protein | 105.813 | 38.6376 | | -1.4534 |
| AT5G19900 | | PRLI-interacting factor, putative | 288.273 | 105.305 | | -1.4529 |
| AT1G58520 | | RXW8, lipases;hydrolases, acting on ester bonds | 54.7524 | 20.0112 | | -1.4521 |
| AT2G20010 | | Protein of unknown function (DUF810) | 54.4102 | 19.893 | | -1.4516 |
| AT5G42250 | | Zinc-binding alcohol dehydrogenase family protein | 53.901 | 19.7122 | | -1.4512 |
| AT3G08580 | | AAC1, ADP/ATP carrier 1 | 1968.22 | 719.857 | | -1.4511 |
| AT1G28200 | | FIP1, FH interacting protein 1 | 218.519 | 79.9274 | | -1.451 |
| AT3G12510 | | MADS-box family protein | 1085.59 | 397.172 | | -1.4506 |
| AT5G54780 | | Ypt/Rab-GAP domain of gyp1p superfamily protein | 68.1195 | 24.939 | | -1.4497 |
| AT2G02510 | | NADH dehydrogenase (ubiquinone)s | 375.097 | 137.345 | | -1.4495 |
| AT5G64660 | | ATCMPG2, CMPG2, CYS, MET, PRO, and GLY protein 2 | 56.3152 | 20.6285 | | -1.4489 |
| AT2G32390 | | ATGLR3.5, GLR3.5, GLR6, glutamate receptor 3.5 | 24.4592 | 8.96156 | | -1.4486 |
| AT4G26890 | | MAPKKK16, mitogen-activated protein kinase kinase kinase 16 | 16.4378 | 6.02283 | | -1.4485 |
| AT1G27170 | | transmembrane receptors;ATP binding | 2.6846 | 0.983701 | | -1.4484 |
| AT4G10810 | | unknown protein | 92.0803 | 33.7553 | | -1.4478 |
| AT1G61360 | | S-locus lectin protein kinase family protein | 47.7828 | 17.5205 | | -1.4475 |
| AT3G03720 | | CAT4, cationic amino acid transporter 4 | 43.2814 | 15.8769 | | -1.4468 |
| AT2G36895 | | unknown protein | 93.6666 | 34.3901 | | -1.4455 |
| AT1G75750 | | GASA1, GAST1 protein homolog 1 | 191.877 | 70.459 | | -1.4453 |
| AT5G08060 | | unknown protein | 197.224 | 72.4427 | | -1.4449 |
| AT1G12310 | | Calcium-binding EF-hand family protein | 138.584 | 50.9035 | | -1.4449 |
| AT4G33985 | | Protein of unknown function (DUF1685) | 54.9466 | 20.2031 | | -1.4435 |
| AT5G66070 | | RING/U-box superfamily protein | 215.533 | 79.2505 | | -1.4434 |
| AT5G60120 | | TOE2, target of early activation tagged (EAT) 2 | 21.5244 | 7.9194 | | -1.4425 |
| AT3G15430 | | Regulator of chromosome condensation (RCC1) family protein | 109.051 | 40.1306 | | -1.4422 |
| AT2G32260 | | ATCCT1, CCT1, phosphorylcholine cytidylyltransferase | 81.8792 | 30.137 | | -1.442 |
| AT3G57400 | | unknown protein | 53.2336 | 19.5987 | | -1.4416 |
| AT3G57480 | | zinc finger (C2H2 type, AN1-like) family protein | 45.8663 | 16.8966 | | -1.4407 |
| AT3G56790 | | RNA splicing factor-related | 25.6723 | 9.45787 | | -1.4406 |
| AT3G55940 | | Phosphoinositide-specific phospholipase C family protein | 38.9852 | 14.3651 | | -1.4404 |
| AT1G71090 | | Auxin efflux carrier family protein | 28.5045 | 10.5134 | | -1.439 |
| AT3G07890 | | Ypt/Rab-GAP domain of gyp1p superfamily protein | 119.607 | 44.1154 | | -1.439 |
| AT4G06676 | | CONTAINS InterPro DOMAIN/s: Etoposide-induced 2.4 (InterPro:IPR009890) | 31.5605 | 11.6444 | | -1.4385 |
| AT3G16800 | | Protein phosphatase 2C family protein | 119.3 | 44.0168 | | -1.4385 |
| AT5G24470 | | APRR5, PRR5, pseudo-response regulator 5 | 35.1893 | 12.9871 | | -1.4381 |
| AT5G62630 | | HIPL2, hipl2 protein precursor | 6.78242 | 2.50346 | | -1.4379 |
| AT5G58020 | | unknown protein | 208.417 | 76.9897 | | -1.4367 |
| AT5G08730 | | ARI16, ATARI16, IBR domain-containing protein | 5.27758 | 1.9497 | | -1.4366 |
| AT5G66820 | | unknown protein | 13.5814 | 5.01974 | | -1.436 |
| AT1G72770 | | HAB1, homology to ABI1 | 99.8724 | 36.9211 | | -1.4356 |
| AT5G65207 | | unknown protein | 3333.24 | 1232.51 | | -1.4353 |
| AT3G49570 | | LSU3, response to low sulfur 3 | 190.802 | 70.5889 | | -1.4346 |
| AT5G07070 | | CIPK2, SnRK3.2, CBL-interacting protein kinase 2 | 59.874 | 22.1559 | | -1.4342 |
| AT2G43490 | | Ypt/Rab-GAP domain of gyp1p superfamily protein | 44.0976 | 16.3244 | | -1.4337 |
| AT1G61770 | | Chaperone DnaJ-domain superfamily protein | 173.696 | 64.3107 | | -1.4334 |
| AT3G48680 | | GAMMA CAL2, gamma carbonic anhydrase-like 2 | 92.8943 | 34.4114 | | -1.4327 |
| AT1G21660 | | Chaperone DnaJ-domain superfamily protein | 93.6772 | 34.708 | | -1.4324 |
| AT1G17620 | | Late embryogenesis abundant (LEA) hydroxyproline-rich glycoprotein family | 380.029 | 140.832 | | -1.4321 |
| AT3G24160 | | PMP, putative type 1 membrane protein | 464.094 | 172.006 | | -1.432 |
| AT4G31980 | | unknown protein | 8.67979 | 3.2177 | | -1.4316 |
| AT5G15650 | | ATRGP2, RGP2, reversibly glycosylated polypeptide 2 | 1568.93 | 581.897 | | -1.4309 |
| AT1G25550 | | myb-like transcription factor family protein | 159.219 | 59.0713 | | -1.4305 |
| AT3G16530 | | Legume lectin family protein | 19.1794 | 7.11834 | | -1.4299 |
| AT2G33290 | | ATSUVH2, SDG3, SUVH2, SU(VAR)3-9 homolog 2 | 7.71558 | 2.8643 | | -1.4296 |
| AT4G32060 | | calcium-binding EF hand family protein | 287.227 | 106.634 | | -1.4295 |
| AT4G08180 | | ORP1C, OSBP(oxysterol binding protein)-related protein 1C | 52.3103 | 19.4261 | | -1.4291 |
| AT4G23640 | | ATKT3, KUP4, TRH1, Potassium transporter family protein | 35.4945 | 13.1821 | | -1.429 |
| AT1G06570 | | HPD, PDS1, phytoene desaturation 1 | 152.351 | 56.6254 | | -1.4279 |
| AT4G30440 | | GAE1, UDP-D-glucuronate 4-epimerase 1 | 111.756 | 41.545 | | -1.4276 |
| AT1G24330 | | ARM repeat superfamily protein | 10.0019 | 3.71994 | | -1.4269 |
| AT2G46260 | | BTB/POZ/Kelch-associated protein | 267.275 | 99.4655 | | -1.4261 |
| AT4G00860 | | AT0ZI1, ATOZI1, Protein of unknown function (DUF1138) | 712.244 | 265.061 | | -1.4261 |
| AT4G03560 | | ATCCH1, ATTPC1, FOU2, TPC1, TPC1, two-pore channel 1 | 322.19 | 119.985 | | -1.4251 |
| AT1G23880 | | NHL domain-containing protein | 22.573 | 8.40646 | | -1.425 |
| AT1G80310 | | sulfate transmembrane transporters | 8.19945 | 3.05368 | | -1.425 |
| AT1G02170 | | AMC1, ATMC1, ATMCPB1, LOL3, MCP1B, metacaspase 1 | 83.3753 | 31.0515 | | -1.425 |
| AT5G42740 | | Sugar isomerase (SIS) family protein | 133.84 | 49.8466 | | -1.4249 |
| AT1G57680 | | function unknown | 99.9128 | 37.2119 | | -1.4249 |
| AT4G36900 | | DEAR4, RAP2.10, related to AP2 10 | 56.0284 | 20.8732 | | -1.4245 |
| AT3G62400 | | unknown protein | 408.316 | 152.137 | | -1.4243 |
| AT2G32960 | | Phosphotyrosine protein phosphatases superfamily protein | 8.96415 | 3.34035 | | -1.4242 |
| AT4G35480 | | RHA3B, RING-H2 finger A3B | 108.772 | 40.5335 | | -1.4241 |
| AT5G45410 | | unknown protein | 197.066 | 73.4435 | | -1.424 |
| AT5G42930 | | alpha/beta-Hydrolases superfamily protein | 95.6437 | 35.6642 | | -1.4232 |
| AT1G77680 | | Ribonuclease II/R family protein | 236.464 | 88.1867 | | -1.423 |
| AT2G27000 | | CYP705A8, cytochrome P450, family 705, subfamily A, polypeptide 8 | 54.0366 | 20.1544 | | -1.4228 |
| AT1G50590 | | RmlC-like cupins superfamily protein | 67.8621 | 25.3163 | | -1.4225 |
| AT3G52060 | | Core-2/I-branching beta-1,6-N-acetylglucosaminyltransferase family protein | 167.77 | 62.5968 | | -1.4223 |
| AT5G20090 | | Uncharacterised protein family (UPF0041) | 180.122 | 67.2339 | | -1.4217 |
| AT2G37280 | | ATPDR5, PDR5, pleiotropic drug resistance 5 | 10.2405 | 3.82392 | | -1.4212 |
| AT1G14660 | | ATNHX8, NHX8, NHX8, Na+/H+ exchanger 8 | 5.11901 | 1.91194 | | -1.4208 |
| AT5G07250 | | ATRBL3, RBL3, RHOMBOID-like protein 3 | 22.8688 | 8.54836 | | -1.4197 |
| AT3G12260 | | LYR family of Fe/S cluster biogenesis protein | 191.433 | 71.5732 | | -1.4194 |
| AT1G53210 | | sodium/calcium exchanger family protein / calcium-binding EF hand family protein | 393.768 | 147.235 | | -1.4192 |
| AT4G32030 | | unknown protein | 204.22 | 76.3839 | | -1.4188 |
| AT2G38410 | | ENTH/VHS/GAT family protein | 94.686 | 35.4149 | | -1.4188 |
| AT5G08170 | | ATAIH, EMB1873, porphyromonas-type peptidyl-arginine deiminase family protein | 40.8038 | 15.2623 | | -1.4187 |
| AT1G62422 | | unknown protein | 12.4774 | 4.67216 | | -1.4172 |
| AT5G65390 | | AGP7, arabinogalactan protein 7 | 69.7704 | 26.1388 | | -1.4164 |
| AT5G19070 | | SNARE associated Golgi protein family | 120.401 | 45.1077 | | -1.4164 |
| AT5G63990 | | Inositol monophosphatase family protein | 146.675 | 54.9581 | | -1.4162 |
| AT5G28150 | | Plant protein of unknown function (DUF868) | 35.4704 | 13.2912 | | -1.4161 |
| AT2G37180 | | PIP2;3, PIP2C, RD28, Aquaporin-like superfamily protein | 25.1921 | 9.43978 | | -1.4161 |
| AT5G59160 | | PPO, TOPP2, type one serine/threonine protein phosphatase 2 | 71.1482 | 26.6621 | | -1.416 |
| AT1G12640 | | MBOAT (membrane bound O-acyl transferase) family protein | 56.9024 | 21.3311 | | -1.4155 |
| AT1G74790 | | catalytics | 67.7972 | 25.4186 | | -1.4153 |
| AT1G72700 | | ATPase E1-E2 type family protein / haloacid dehalogenase-like hydrolase family protein | 79.8422 | 29.9921 | | -1.4126 |
| AT4G00355 | | unknown protein | 57.7306 | 21.6882 | | -1.4124 |
| AT5G14000 | | anac084, NAC084, NAC domain containing protein 84 | 61.1485 | 22.9818 | | -1.4118 |
| AT4G04800 | | ATMSRB3, MSRB3, methionine sulfoxide reductase B3 | 113.15 | 42.5369 | | -1.4115 |
| AT5G16550 | | unknown protein | 151.309 | 56.8968 | | -1.4111 |
| AT1G60420 | | DC1 domain-containing protein | 67.0665 | 25.233 | | -1.4103 |
| AT5G57887 | | unknown protein | 49.4774 | 18.6155 | | -1.4103 |
| AT3G19290 | | ABF4, AREB2, ABRE binding factor 4 | 72.2665 | 27.2031 | | -1.4096 |
| AT4G37610 | | BT5, BTB and TAZ domain protein 5 | 600.848 | 226.306 | | -1.4087 |
| AT5G48150 | | PAT1, GRAS family transcription factor | 35.1545 | 13.2461 | | -1.4081 |
| AT1G15710 | | prephenate dehydrogenase family protein | 25.7587 | 9.70868 | | -1.4077 |
| AT3G57785 | | unknown protein | 41.8584 | 15.7982 | | -1.4058 |
| AT2G26890 | | GRV2, KAM2, DNAJ heat shock N-terminal domain-containing protein | 32.8545 | 12.4019 | | -1.4055 |
| AT4G34700 | | LYR family of Fe/S cluster biogenesis protein | 486.693 | 183.75 | | -1.4053 |
| AT2G16430 | | ATPAP10, PAP10, purple acid phosphatase 10 | 28.9498 | 10.9355 | | -1.4045 |
| AT1G15530 | | Concanavalin A-like lectin protein kinase family protein | 33.1619 | 12.5379 | | -1.4032 |
| AT5G20990 | | B73, CHL6, CNX, CNX1, SIR4, molybdopterin biosynthesis CNX1 protein / molybdenum cofactor biosynthesis enzyme CNX1 (CNX1) | 73.17 | 27.6679 | | -1.403 |
| AT3G02360 | | 6-phosphogluconate dehydrogenase family protein | 299.923 | 113.415 | | -1.403 |
| AT5G61530 | | small G protein family protein / RhoGAP family protein | 135.558 | 51.2875 | | -1.4022 |
| AT5G64260 | | EXL2, EXORDIUM like 2 | 247.955 | 93.8201 | | -1.4021 |
| AT5G10695 | | unknown protein | 537.896 | 203.558 | | -1.4019 |
| AT1G76920 | | F-box family protein | 60.7336 | 22.9938 | | -1.4013 |
| AT3G59050 | | ATPAO3, PAO3, polyamine oxidase 3 | 185.259 | 70.166 | | -1.4007 |
| AT5G66510 | | GAMMA CA3, gamma carbonic anhydrase 3 | 254.873 | 96.5655 | | -1.4002 |
| AT1G42990 | | ATBZIP60, BZIP60, BZIP60, basic region/leucine zipper motif 60 | 57.5241 | 21.8062 | | -1.3994 |
| AT1G07890 | | APX1, ATAPX01, ATAPX1, CS1, MEE6, ascorbate peroxidase 1 | 1219.8 | 462.538 | | -1.399 |
| AT1G09940 | | HEMA2, Glutamyl-tRNA reductase family protein | 333.235 | 126.406 | | -1.3985 |
| AT4G34740 | | ASE2, ATASE2, ATPURF2, CIA1, GLN phosphoribosyl pyrophosphate amidotransferase 2 | 22.8674 | 8.68136 | | -1.3973 |
| AT1G27500 | | Tetratricopeptide repeat (TPR)-like superfamily protein | 17.9537 | 6.81706 | | -1.3971 |
| AT1G75230 | | DNA glycosylase superfamily protein | 52.0697 | 19.7808 | | -1.3964 |
| AT5G08670 | | ATP synthase alpha/beta family protein | 430.79 | 163.692 | | -1.396 |
| AT1G57990 | | ATPUP18, PUP18, purine permease 18 | 81.2862 | 30.8932 | | -1.3957 |
| AT1G15130 | | Endosomal targeting BRO1-like domain-containing protein | 91.5767 | 34.8092 | | -1.3955 |
| AT1G66900 | | alpha/beta-Hydrolases superfamily protein | 40.5715 | 15.4221 | | -1.3955 |
| AT3G55260 | | ATHEX2, HEXO1, beta-hexosaminidase 1 | 21.5374 | 8.18777 | | -1.3953 |
| AT5G13810 | | Glutaredoxin family protein | 102.324 | 38.9278 | | -1.3943 |
| AT2G45960 | | ATHH2, PIP1;2, PIP1B, TMP-A, plasma membrane intrinsic protein 1B | 1323.36 | 503.512 | | -1.3941 |
| AT1G76600 | | unknown protein | 345.153 | 131.333 | | -1.394 |
| AT2G05630 | | ATG8D, Ubiquitin-like superfamily protein | 41.9784 | 15.9814 | | -1.3933 |
| AT1G25390 | | Protein kinase superfamily protein | 25.072 | 9.54529 | | -1.3932 |
| AT5G51460 | | ATTPPA, Haloacid dehalogenase-like hydrolase (HAD) superfamily protein | 71.2541 | 27.1365 | | -1.3927 |
| AT1G51980 | | Insulinase (Peptidase family M16) protein | 362.84 | 138.268 | | -1.3919 |
| AT5G14150 | | Protein of unknown function, DUF642 | 19.6594 | 7.49199 | | -1.3918 |
| AT3G03250 | | AtUGP1, UGP, UGP1, UDP-GLUCOSE PYROPHOSPHORYLASE 1 | 268.528 | 102.339 | | -1.3917 |
| AT5G63470 | | NF-YC4, nuclear factor Y, subunit C4 | 37.9743 | 14.4741 | | -1.3916 |
| AT5G01490 | | ATCAX4, CAX4, cation exchanger 4 | 63.4543 | 24.1935 | | -1.3911 |
| AT5G21990 | | Tetratricopeptide repeat (TPR)-like superfamily protein | 294.186 | 112.192 | | -1.3908 |
| AT5G47120 | | ATBI-1, ATBI1, BI-1, BI1, BAX inhibitor 1 | 1011.32 | 385.747 | | -1.3905 |
| AT1G21380 | | Target of Myb protein 1 | 218.263 | 83.266 | | -1.3903 |
| AT4G00300 | | fringe-related protein | 133.469 | 50.9446 | | -1.3895 |
| AT1G47570 | | RING/U-box superfamily protein | 17.4023 | 6.64635 | | -1.3886 |
| AT1G04250 | | AXR3, IAA17, AUX/IAA transcriptional regulator family protein | 244.712 | 93.5005 | | -1.388 |
| AT3G60260 | | ELMO/CED-12 family protein | 83.4407 | 31.885 | | -1.3879 |
| AT1G19650 | | Sec14p-like phosphatidylinositol transfer family protein | 22.7383 | 8.68943 | | -1.3878 |
| AT1G13520 | | Protein of unknown function (DUF1262) | 9.07737 | 3.47035 | | -1.3872 |
| AT4G38140 | | RING/U-box superfamily protein | 198.819 | 76.0474 | | -1.3865 |
| AT3G47960 | | Major facilitator superfamily protein | 145.322 | 55.5909 | | -1.3863 |
| AT4G30060 | | Core-2/I-branching beta-1,6-N-acetylglucosaminyltransferase family protein | 51.1928 | 19.5875 | | -1.386 |
| AT3G56800 | | acam-3, CAM3, calmodulin 3 | 357.222 | 136.684 | | -1.386 |
| AT5G65280 | | GCL1, GCR2-like 1 | 78.007 | 29.861 | | -1.3853 |
| AT3G27380 | | SDH2-1, succinate dehydrogenase 2-1 | 171.825 | 65.8095 | | -1.3846 |
| AT5G05860 | | UGT76C2, UDP-glucosyl transferase 76C2 | 12.966 | 4.96653 | | -1.3844 |
| AT4G29190 | | Zinc finger C-x8-C-x5-C-x3-H type family protein | 176.941 | 67.8202 | | -1.3835 |
| AT1G01240 | | unknown protein | 132.524 | 50.8107 | | -1.3831 |
| AT1G49340 | | ATPI4K ALPHA, Phosphatidylinositol 3- and 4-kinase family protein | 34.4437 | 13.2183 | | -1.3817 |
| AT3G11030 | | TBL32, TRICHOME BIREFRINGENCE-LIKE 32 | 49.0593 | 18.8426 | | -1.3805 |
| AT5G02230 | | Haloacid dehalogenase-like hydrolase (HAD) superfamily protein | 981.784 | 377.193 | | -1.3801 |
| AT1G79590 | | ATSYP52, SYP52, syntaxin of plants 52 | 123.737 | 47.5749 | | -1.379 |
| AT4G29780 | | unknown protein | 237.626 | 91.4077 | | -1.3783 |
| AT4G38800 | | ATMTAN1, ATMTN1, MTAN1, MTN1, methylthioadenosine nucleosidase 1 | 222.955 | 85.7774 | | -1.3781 |
| AT2G30870 | | ATGSTF10, ATGSTF4, ERD13, GSTF10, glutathione S-transferase PHI 10 | 1336.05 | 514.049 | | -1.378 |
| AT3G57520 | | AtSIP2, SIP2, seed imbibition 2 | 213.073 | 82.0158 | | -1.3774 |
| AT3G59940 | | Galactose oxidase/kelch repeat superfamily protein | 95.7151 | 36.8476 | | -1.3772 |
| AT5G32440 | | Ubiquitin system component Cue protein | 123.319 | 47.5331 | | -1.3754 |
| AT2G41190 | | Transmembrane amino acid transporter family protein | 11.8403 | 4.56825 | | -1.374 |
| AT3G11840 | | PUB24, plant U-box 24 | 9.44953 | 3.64883 | | -1.3728 |
| AT5G03455 | | ACR2, ARATH;CDC25, CDC25, Rhodanese/Cell cycle control phosphatase superfamily protein | 32.2376 | 12.4495 | | -1.3727 |
| AT1G09930 | | ATOPT2, OPT2, oligopeptide transporter 2 | 17.1248 | 6.61336 | | -1.3726 |
| AT4G22820 | | A20/AN1-like zinc finger family protein | 301.504 | 116.647 | | -1.37 |
| AT1G74400 | | Tetratricopeptide repeat (TPR)-like superfamily protein | 16.5434 | 6.40993 | | -1.3679 |
| AT3G02910 | | AIG2-like (avirulence induced gene) family protein | 101.403 | 39.2968 | | -1.3676 |
| AT3G13410 | | unknown protein | 412.011 | 159.687 | | -1.3674 |
| AT2G22910 | | NAGS1, N-acetyl-l-glutamate synthase 1 | 29.4485 | 11.4144 | | -1.3673 |
| AT1G53560 | | Ribosomal protein L18ae family | 63.0002 | 24.436 | | -1.3664 |
| AT1G79330 | | AMC6, ATMC5, ATMCP2B, MC5, metacaspase 5 | 14.6955 | 5.70211 | | -1.3658 |
| AT5G47030 | | ATPase, F1 complex, delta/epsilon subunit | 453.372 | 175.961 | | -1.3654 |
| AT4G02520 | | ATGSTF2, ATPM24, ATPM24.1, GST2, GSTF2, glutathione S-transferase PHI 2 | 99.5182 | 38.632 | | -1.3652 |
| AT5G04160 | | Nucleotide-sugar transporter family protein | 55.5043 | 21.5547 | | -1.3646 |
| AT5G24270 | | ATSOS3, CBL4, SOS3, Calcium-binding EF-hand family protein | 77.209 | 29.9928 | | -1.3642 |
| AT1G18160 | | Protein kinase superfamily protein | 42.1107 | 16.363 | | -1.3638 |
| AT1G02900 | | ATRALF1, RALF1, RALFL1, rapid alkalinization factor 1 | 47.2155 | 18.3495 | | -1.3635 |
| AT5G64750 | | ABR1, Integrase-type DNA-binding superfamily protein | 700.954 | 272.447 | | -1.3634 |
| AT3G07040 | | RPM1, RPS3, NB-ARC domain-containing disease resistance protein | 4.56268 | 1.77353 | | -1.3633 |
| AT4G22310 | | Uncharacterised protein family (UPF0041) | 224.335 | 87.2638 | | -1.3622 |
| AT3G08780 | | unknown protein | 29.3667 | 11.425 | | -1.362 |
| AT2G18210 | | unknown protein | 283.135 | 110.168 | | -1.3618 |
| AT1G18910 | | zinc ion binding;zinc ion binding | 51.6658 | 20.1109 | | -1.3612 |
| AT1G72160 | | Sec14p-like phosphatidylinositol transfer family protein | 64.9676 | 25.294 | | -1.3609 |
| AT1G76980 | | BEST Arabidopsis thaliana protein match is: embryo defective 2170 (TAIR:AT1G21390.1) | 192.358 | 74.9205 | | -1.3604 |
| AT3G11690 | | unknown protein | 20.7242 | 8.07341 | | -1.3601 |
| AT4G19040 | | EDR2, ENHANCED DISEASE RESISTANCE 2 | 30.6645 | 11.9508 | | -1.3595 |
| AT2G01190 | | Octicosapeptide/Phox/Bem1p family protein | 31.5797 | 12.3094 | | -1.3592 |
| AT5G64920 | | CIP8, COP1-interacting protein 8 | 28.1787 | 10.9882 | | -1.3587 |
| AT3G48140 | | B12D protein | 1171.8 | 457.035 | | -1.3583 |
| AT3G44190 | | FAD/NAD(P)-binding oxidoreductase family protein | 577.31 | 225.19 | | -1.3582 |
| AT4G12010 | | Disease resistance protein (TIR-NBS-LRR class) family | 15.7912 | 6.16154 | | -1.3578 |
| AT1G22930 | | T-complex protein 11 | 239.318 | 93.3886 | | -1.3576 |
| AT1G50320 | | ATHX, ATX, THX, thioredoxin X | 25.0482 | 9.77487 | | -1.3576 |
| AT1G08940 | | Phosphoglycerate mutase family protein | 97.368 | 38.0252 | | -1.3565 |
| AT2G45820 | | Remorin family protein | 386.462 | 150.942 | | -1.3563 |
| AT3G16460 | | Mannose-binding lectin superfamily protein | 1522.3 | 595.036 | | -1.3552 |
| AT1G03220 | | Eukaryotic aspartyl protease family protein | 981.314 | 383.809 | | -1.3543 |
| AT4G35260 | | IDH-I, IDH1, isocitrate dehydrogenase 1 | 133.746 | 52.3263 | | -1.3539 |
| AT5G57660 | | ATCOL5, COL5, CONSTANS-like 5 | 178.487 | 69.8385 | | -1.3537 |
| AT1G44100 | | AAP5, amino acid permease 5 | 11.8274 | 4.63074 | | -1.3528 |
| AT2G27310 | | F-box family protein | 260.642 | 102.161 | | -1.3512 |
| AT1G25560 | | EDF1, TEM1, AP2/B3 transcription factor family protein | 250.875 | 98.3487 | | -1.351 |
| AT3G62720 | | ATXT1, XT1, XXT1, xylosyltransferase 1 | 89.3846 | 35.0464 | | -1.3508 |
| AT1G64563 | | other RNA | 4.57445 | 1.79414 | | -1.3503 |
| AT1G76030 | | ATPase, V1 complex, subunit B protein | 319.447 | 125.435 | | -1.3486 |
| AT5G43030 | | Cysteine/Histidine-rich C1 domain family protein | 9.17098 | 3.60298 | | -1.3479 |
| AT3G01040 | | GAUT13, galacturonosyltransferase 13 | 48.3259 | 18.9942 | | -1.3472 |
| AT5G13430 | | Ubiquinol-cytochrome C reductase iron-sulfur subunit | 89.1908 | 35.06 | | -1.3471 |
| AT4G32480 | | Protein of unknown function (DUF506) | 237.518 | 93.3727 | | -1.347 |
| AT4G21534 | | Diacylglycerol kinase family protein | 13.6904 | 5.38207 | | -1.3469 |
| AT1G08930 | | ERD6, Major facilitator superfamily protein | 347.06 | 136.441 | | -1.3469 |
| AT1G09270 | | IMPA-4, importin alpha isoform 4 | 133.346 | 52.5573 | | -1.3432 |
| AT5G56630 | | PFK7, phosphofructokinase 7 | 237.492 | 93.6197 | | -1.343 |
| AT1G78830 | | Curculin-like (mannose-binding) lectin family protein | 185.136 | 72.9902 | | -1.3428 |
| AT4G39030 | | EDS5, SID1, MATE efflux family protein | 92.2074 | 36.3607 | | -1.3425 |
| AT5G58750 | | NAD(P)-binding Rossmann-fold superfamily protein | 28.2834 | 11.1555 | | -1.3422 |
| AT1G18740 | | Protein of unknown function (DUF793) | 125.755 | 49.6226 | | -1.3415 |
| AT3G50260 | | ATERF#011, CEJ1, DEAR1, cooperatively regulated by ethylene and jasmonate 1 | 117.099 | 46.2149 | | -1.3413 |
| AT4G16070 | | Mono-/di-acylglycerol lipase, N-terminal;Lipase, class 3 | 25.4008 | 10.0289 | | -1.3407 |
| AT5G06740 | | Concanavalin A-like lectin protein kinase family protein | 15.8401 | 6.25601 | | -1.3403 |
| AT2G24240 | | BTB/POZ domain with WD40/YVTN repeat-like protein | 17.1747 | 6.79136 | | -1.3385 |
| AT2G43160 | | ENTH/VHS family protein | 190.965 | 75.5139 | | -1.3385 |
| AT1G76200 | | unknown protein | 684.504 | 270.815 | | -1.3378 |
| AT1G16670 | | Protein kinase superfamily protein | 104.533 | 41.418 | | -1.3356 |
| AT5G43050 | | NPQ6, Protein of unknown function (DUF565) | 62.4764 | 24.7659 | | -1.335 |
| AT5G20080 | | FAD/NAD(P)-binding oxidoreductase | 248.57 | 98.5745 | | -1.3344 |
| AT5G39570 | | function unknown | 358.786 | 142.377 | | -1.3334 |
| AT1G08650 | | ATPPCK1, PPCK1, phosphoenolpyruvate carboxylase kinase 1 | 161.444 | 64.0705 | | -1.3333 |
| AT4G03430 | | EMB2770, STA1, pre-mRNA splicing factor-related | 183.66 | 72.922 | | -1.3326 |
| AT3G17890 | | unknown protein | 31.2559 | 12.4177 | | -1.3317 |
| AT4G40060 | | ATHB-16, ATHB16, HB16, homeobox protein 16 | 504.476 | 200.452 | | -1.3315 |
| AT1G79360 | | 2-Oct, ATOCT2, OCT2, organic cation/carnitine transporter 2 | 34.2739 | 13.6342 | | -1.3299 |
| AT1G01620 | | PIP1;3, PIP1C, TMP-B, plasma membrane intrinsic protein 1C | 508.785 | 202.414 | | -1.3298 |
| AT3G10020 | | unknown protein | 509.489 | 202.744 | | -1.3294 |
| AT3G02090 | | MPPBETA, Insulinase (Peptidase family M16) protein | 424.921 | 169.176 | | -1.3287 |
| AT4G40065 | | other RNA | 12.9175 | 5.14436 | | -1.3283 |
| AT5G61560 | | U-box domain-containing protein kinase family protein | 27.0559 | 10.7775 | | -1.3279 |
| AT2G18840 | | Integral membrane Yip1 family protein | 44.031 | 17.5447 | | -1.3275 |
| AT3G57090 | | BIGYIN, FIS1A, Tetratricopeptide repeat (TPR)-like superfamily protein | 132.829 | 53.0448 | | -1.3243 |
| AT3G06060 | | NAD(P)-binding Rossmann-fold superfamily protein | 59.2922 | 23.6819 | | -1.3241 |
| AT1G30130 | | unknown protein | 120.671 | 48.2212 | | -1.3233 |
| AT4G00430 | | PIP1;4, PIP1E, TMP-C, plasma membrane intrinsic protein 1;4 | 192.525 | 76.9364 | | -1.3233 |
| AT5G66200 | | ARO2, armadillo repeat only 2 | 35.8486 | 14.3294 | | -1.3229 |
| AT3G02070 | | Cysteine proteinases superfamily protein | 64.3665 | 25.7361 | | -1.3225 |
| AT5G04840 | | bZIP protein | 53.7091 | 21.4757 | | -1.3225 |
| AT5G56210 | | WIP2, WPP domain interacting protein 2 | 29.6905 | 11.8781 | | -1.3217 |
| AT5G41750 | | Disease resistance protein (TIR-NBS-LRR class) family | 12.1785 | 4.87351 | | -1.3213 |
| AT5G13180 | | ANAC083, NAC083, VNI2, NAC domain containing protein 83 | 432.024 | 172.947 | | -1.3208 |
| AT4G11280 | | ACS6, ATACS6, 1-aminocyclopropane-1-carboxylic acid (acc) synthase 6 | 67.5176 | 27.0464 | | -1.3198 |
| AT5G17060 | | ARFB1B, ATARFB1B, ADP-ribosylation factor B1B | 99.3639 | 39.8485 | | -1.3182 |
| AT5G11000 | | Plant protein of unknown function (DUF868) | 32.8341 | 13.1742 | | -1.3175 |
| AT4G35090 | | CAT2, catalase 2 | 348.67 | 139.931 | | -1.3171 |
| AT4G35950 | | ARAC6, ATRAC6, ATROP5, RAC2, RAC6, ROP5, RAC-like 6 | 32.9335 | 13.2209 | | -1.3167 |
| AT4G14410 | | bHLH104, basic helix-loop-helix (bHLH) DNA-binding superfamily protein | 137.35 | 55.1423 | | -1.3166 |
| AT2G38360 | | PRA1.B4, prenylated RAB acceptor 1.B4 | 142.622 | 57.3274 | | -1.3149 |
| AT1G16700 | | Alpha-helical ferredoxin | 219.072 | 88.1035 | | -1.3141 |
| AT1G75950 | | ASK1, ATSKP1, SKP1, SKP1A, UIP1, S phase kinase-associated protein 1 | 732.56 | 294.693 | | -1.3137 |
| AT2G43120 | | RmlC-like cupins superfamily protein | 621.829 | 250.251 | | -1.3131 |
| AT3G62660 | | GATL7, galacturonosyltransferase-like 7 | 36.5758 | 14.7213 | | -1.313 |
| AT2G40620 | | Basic-leucine zipper (bZIP) transcription factor family protein | 141.222 | 56.8848 | | -1.3119 |
| AT3G22750 | | Protein kinase superfamily protein | 33.9134 | 13.6671 | | -1.3112 |
| AT2G43340 | | Protein of unknown function (DUF1685) | 493.845 | 199.06 | | -1.3109 |
| AT3G30775 | | AT-POX, Methylenetetrahydrofolate reductase family protein | 660.893 | 266.421 | | -1.3107 |
| AT5G58375 | | Methyltransferase-related protein | 151.981 | 61.2825 | | -1.3103 |
| AT1G45688 | | unknown protein | 140.17 | 56.5209 | | -1.3103 |
| AT5G67530 | | ATPUB49, PUB49, plant U-box 49 | 62.1712 | 25.0897 | | -1.3092 |
| AT1G35720 | | ANNAT1, ATOXY5, OXY5, annexin 1 | 1520.28 | 613.756 | | -1.3086 |
| AT3G14070 | | ATCCX3, CAX9, CCX3, cation exchanger 9 | 6.94484 | 2.80479 | | -1.3081 |
| AT1G23390 | | Kelch repeat-containing F-box family protein | 13.4122 | 5.41725 | | -1.3079 |
| AT3G20560 | | ATPDI12, ATPDIL5-3, PDI12, PDIL5-3, PDI-like 5-3 | 22.5684 | 9.12075 | | -1.3071 |
| AT4G39640 | | GGT1, gamma-glutamyl transpeptidase 1 | 49.6067 | 20.0516 | | -1.3068 |
| AT4G16450 | | unknown protein | 549.051 | 222.091 | | -1.3058 |
| AT4G16480 | | ATINT4, INT4, inositol transporter 4 | 8.91291 | 3.60611 | | -1.3055 |
| AT1G74590 | | ATGSTU10, GSTU10, glutathione S-transferase TAU 10 | 62.8311 | 25.4314 | | -1.3049 |
| AT2G41780 | | unknown protein | 31.0083 | 12.5665 | | -1.3031 |
| AT1G09950 | | RAS1, RESPONSE TO ABA AND SALT 1 | 45.6906 | 18.5278 | | -1.3022 |
| AT1G51650 | | ATP synthase epsilon chain, mitochondrial | 926.751 | 376.214 | | -1.3006 |
| AT1G29390 | | COR314-TM2, COR413IM2, cold regulated 314 thylakoid membrane 2 | 25.0068 | 10.1543 | | -1.3002 |
| AT3G05050 | | Protein kinase superfamily protein | 52.5673 | 21.3469 | | -1.3001 |
| AT4G22330 | | ATCES1, Alkaline phytoceramidase (aPHC) | 170.636 | 69.3336 | | -1.2993 |
| AT5G03540 | | ATEXO70A1, EXO70A1, exocyst subunit exo70 family protein A1 | 95.0941 | 38.6426 | | -1.2992 |
| AT1G03457 | | RNA-binding (RRM/RBD/RNP motifs) family protein | 29.8703 | 12.152 | | -1.2975 |
| AT1G44770 | | unknown protein | 74.5119 | 30.3499 | | -1.2958 |
| AT3G49350 | | Ypt/Rab-GAP domain of gyp1p superfamily protein | 39.3889 | 16.0442 | | -1.2957 |
| AT2G42520 | | P-loop containing nucleoside triphosphate hydrolases superfamily protein | 63.8609 | 26.015 | | -1.2956 |
| AT1G72020 | | unknown protein | 670.043 | 272.982 | | -1.2955 |
| AT2G40800 | | unknown protein | 78.7952 | 32.1192 | | -1.2947 |
| AT3G28320 | | Protein of unknown function (DUF677) | 215.373 | 87.9915 | | -1.2914 |
| AT5G51440 | | HSP20-like chaperones superfamily protein | 196.686 | 80.3616 | | -1.2913 |
| AT5G66250 | | kinectin-related | 67.552 | 27.6104 | | -1.2908 |
| AT1G80240 | | Protein of unknown function, DUF642 | 68.2769 | 27.916 | | -1.2903 |
| AT2G17440 | | PIRL5, plant intracellular ras group-related LRR 5 | 203.578 | 83.2521 | | -1.29 |
| AT1G21590 | | Protein kinase protein with adenine nucleotide alpha hydrolases-like domain | 21.323 | 8.723 | | -1.2895 |
| AT5G45510 | | Leucine-rich repeat (LRR) family protein | 159.03 | 65.1305 | | -1.2879 |
| AT3G16760 | | Tetratricopeptide repeat (TPR)-like superfamily protein | 51.8636 | 21.2461 | | -1.2875 |
| AT2G03480 | | QUL2, QUASIMODO2 LIKE 2 | 15.591 | 6.3875 | | -1.2874 |
| AT3G19950 | | RING/U-box superfamily protein | 82.7952 | 33.9288 | | -1.287 |
| AT1G72130 | | Major facilitator superfamily protein | 30.3404 | 12.4373 | | -1.2866 |
| AT5G04340 | | C2H2, CZF2, ZAT6, zinc finger of Arabidopsis thaliana 6 | 148.762 | 60.9907 | | -1.2863 |
| AT1G07310 | | Calcium-dependent lipid-binding (CaLB domain) family protein | 45.9341 | 18.8343 | | -1.2862 |
| AT1G80820 | | ATCCR2, CCR2, cinnamoyl coa reductase | 24.6743 | 10.1207 | | -1.2857 |
| AT3G10420 | | P-loop containing nucleoside triphosphate hydrolases superfamily protein | 41.7345 | 17.1255 | | -1.2851 |
| AT1G28240 | | Protein of unknown function (DUF616) | 48.1016 | 19.7432 | | -1.2847 |
| AT3G52730 | | ubiquinol-cytochrome C reductase UQCRX/QCR9-like family protein | 660.558 | 271.279 | | -1.2839 |
| AT5G41992 | | CPuORF58, conserved peptide upstream open reading frame 58 | 166.711 | 68.5302 | | -1.2825 |
| AT5G46250 | | RNA-binding protein | 148.519 | 61.0539 | | -1.2825 |
| AT3G07460 | | Protein of unknown function, DUF538 | 41.546 | 17.0871 | | -1.2818 |
| AT3G50950 | | ZAR1, HOPZ-ACTIVATED RESISTANCE 1 | 49.7832 | 20.4758 | | -1.2817 |
| AT5G54500 | | FQR1, flavodoxin-like quinone reductase 1 | 235.851 | 97.0811 | | -1.2806 |
| AT4G11220 | | BTI2, RTNLB2, VIRB2-interacting protein 2 | 640.308 | 263.694 | | -1.2799 |
| AT5G15820 | | RING/U-box superfamily protein | 21.5046 | 8.85723 | | -1.2797 |
| AT3G62010 | | unknown protein | 321.403 | 132.627 | | -1.277 |
| AT5G04235 | | transposable element gene | 9.9816 | 4.12157 | | -1.2761 |
| AT3G28715 | | ATPase, V0/A0 complex, subunit C/D | 163.176 | 67.3854 | | -1.2759 |
| AT4G24690 | | ubiquitin-associated (UBA)/TS-N domain-containing protein / octicosapeptide/Phox/Bemp1 (PB1) domain-containing protein | 366.753 | 151.47 | | -1.2758 |
| AT5G03380 | | Heavy metal transport/detoxification superfamily protein | 226.259 | 93.4592 | | -1.2756 |
| AT3G46830 | | ATRAB-A2C, ATRAB11A, ATRABA2C, RAB-A2C, RABA2c, RAB GTPase homolog A2C | 66.6165 | 27.5503 | | -1.2738 |
| AT5G11650 | | alpha/beta-Hydrolases superfamily protein | 262.68 | 108.641 | | -1.2737 |
| AT3G05570 | | unknown protein | 98.4789 | 40.7456 | | -1.2732 |
| AT5G64120 | | Peroxidase superfamily protein | 29.2919 | 12.122 | | -1.2729 |
| AT2G39720 | | RHC2A, RING-H2 finger C2A | 62.6372 | 25.9228 | | -1.2728 |
| AT3G63150 | | ATCBG, MIRO2, MIRO-related GTP-ase 2 | 56.7568 | 23.4936 | | -1.2725 |
| AT3G08930 | | LMBR1-like membrane protein | 114.664 | 47.4671 | | -1.2724 |
| AT3G10760 | | Homeodomain-like superfamily protein | 37.684 | 15.6015 | | -1.2723 |
| AT5G25770 | | alpha/beta-Hydrolases superfamily protein | 75.721 | 31.3532 | | -1.2721 |
| AT1G65890 | | AAE12, acyl activating enzyme 12 | 17.9297 | 7.43053 | | -1.2708 |
| AT1G02816 | | Protein of unknown function, DUF538 | 77.5073 | 32.1333 | | -1.2703 |
| AT4G33980 | | BEST Arabidopsis thaliana protein match is: cold regulated gene 27 (TAIR:AT5G42900.2) | 53.8921 | 22.3509 | | -1.2697 |
| AT5G42570 | | B-cell receptor-associated 31-like | 431.165 | 178.888 | | -1.2692 |
| AT1G11910 | | APA1, ATAPA1, aspartic proteinase A1 | 491.562 | 204.151 | | -1.2677 |
| AT1G80920 | | J8, Chaperone DnaJ-domain superfamily protein | 1780.59 | 739.613 | | -1.2675 |
| AT3G12030 | | Protein of unknown function DUF106, transmembrane | 48.3214 | 20.0925 | | -1.266 |
| AT1G10470 | | ARR4, ATRR1, IBC7, MEE7, response regulator 4 | 112.292 | 46.7094 | | -1.2655 |
| AT5G18130 | | unknown protein | 17.1829 | 7.1484 | | -1.2653 |
| AT5G37600 | | ATGLN1;1, ATGSR1, GLN1;1, GSR 1, glutamine synthase clone R1 | 496.386 | 206.551 | | -1.265 |
| AT5G46340 | | O-acetyltransferase family protein | 23.7706 | 9.895 | | -1.2644 |
| AT2G31680 | | AtRABA5d, RABA5d, RAB GTPase homolog A5D | 53.6824 | 22.3527 | | -1.264 |
| AT4G12120 | | ATSEC1B, SEC1B, Sec1/munc18-like (SM) proteins superfamily | 61.3372 | 25.5521 | | -1.2633 |
| AT2G30490 | | ATC4H, C4H, CYP73A5, REF3, cinnamate-4-hydroxylase | 442.154 | 184.208 | | -1.2632 |
| AT5G57050 | | ABI2, AtABI2, Protein phosphatase 2C family protein | 18.4448 | 7.68494 | | -1.2631 |
| AT2G01570 | | RGA, RGA1, GRAS family transcription factor family protein | 81.9937 | 34.1963 | | -1.2617 |
| AT2G44410 | | RING/U-box superfamily protein | 79.0765 | 32.9902 | | -1.2612 |
| AT1G28280 | | VQ motif-containing protein | 85.7963 | 35.8002 | | -1.261 |
| AT3G52710 | | unknown protein | 28.1665 | 11.7538 | | -1.2609 |
| AT4G16444 | | function unknown | 29.7954 | 12.4395 | | -1.2602 |
| AT5G02040 | | PRA1.A1, prenylated RAB acceptor 1.A1 | 60.4105 | 25.2234 | | -1.26 |
| AT2G17450 | | RHA3A, RING-H2 finger A3A | 270.99 | 113.16 | | -1.2599 |
| AT3G07330 | | ATCSLC06, ATCSLC6, CSLC06, CSLC6, Cellulose-synthase-like C6 | 67.8097 | 28.3219 | | -1.2596 |
| AT3G42050 | | vacuolar ATP synthase subunit H family protein | 468.134 | 195.586 | | -1.2591 |
| AT4G08690 | | Sec14p-like phosphatidylinositol transfer family protein | 36.8414 | 15.4037 | | -1.2581 |
| AT3G51550 | | FER, Malectin/receptor-like protein kinase family protein | 253.929 | 106.244 | | -1.257 |
| AT4G26750 | | hydroxyproline-rich glycoprotein family protein | 53.6248 | 22.4383 | | -1.2569 |
| AT3G25900 | | ATHMT-1, HMT-1, Homocysteine S-methyltransferase family protein | 482.327 | 201.825 | | -1.2569 |
| AT5G51290 | | Diacylglycerol kinase family protein | 20.1573 | 8.44172 | | -1.2557 |
| AT1G12840 | | ATVHA-C, DET3, vacuolar ATP synthase subunit C (VATC) / V-ATPase C subunit / vacuolar proton pump C subunit (DET3) | 449.47 | 188.256 | | -1.2555 |
| AT1G63000 | | NRS/ER, UER1, nucleotide-rhamnose synthase/epimerase-reductase | 722.014 | 302.416 | | -1.2555 |
| AT2G46505 | | SDH4, succinate dehydrogenase subunit 4 | 213.752 | 89.5518 | | -1.2551 |
| AT5G11740 | | AGP15, ATAGP15, arabinogalactan protein 15 | 1943.79 | 814.374 | | -1.2551 |
| AT5G03210 | | unknown protein | 364.618 | 152.865 | | -1.2541 |
| AT1G75590 | | SAUR-like auxin-responsive protein family | 50.1066 | 21.0077 | | -1.2541 |
| AT5G43320 | | ckl8, casein kinase I-like 8 | 85.6062 | 35.9573 | | -1.2514 |
| AT3G52450 | | PUB22, plant U-box 22 | 238.403 | 100.138 | | -1.2514 |
| AT5G24810 | | ABC1 family protein | 82.9763 | 34.8598 | | -1.2511 |
| AT3G19580 | | AZF2, ZF2, zinc-finger protein 2 | 155.351 | 65.2845 | | -1.2507 |
| AT1G78300 | | 14-3-3OMEGA, GF14 OMEGA, GRF2, general regulatory factor 2 | 243.516 | 102.429 | | -1.2494 |
| AT2G30050 | | transducin family protein / WD-40 repeat family protein | 127.581 | 53.6666 | | -1.2493 |
| AT2G30410 | | KIS, TFCA, tubulin folding cofactor A (KIESEL) | 99.6529 | 41.9471 | | -1.2483 |
| AT3G26690 | | ATNUDT13, ATNUDX13, NUDX13, nudix hydrolase homolog 13 | 260.17 | 109.635 | | -1.2467 |
| AT5G37260 | | CIR1, RVE2, Homeodomain-like superfamily protein | 121.514 | 51.2159 | | -1.2465 |
| AT5G42880 | | Plant protein of unknown function (DUF827) | 30.1737 | 12.7177 | | -1.2465 |
| AT3G02840 | | ARM repeat superfamily protein | 73.0916 | 30.8079 | | -1.2464 |
| AT5G47620 | | RNA-binding (RRM/RBD/RNP motifs) family protein | 125.943 | 53.0924 | | -1.2462 |
| AT3G11950 | | TRAF-like superfamily protein | 21.5951 | 9.10515 | | -1.246 |
| AT1G08830 | | CSD1, copper/zinc superoxide dismutase 1 | 966.124 | 407.377 | | -1.2458 |
| AT2G20760 | | Clathrin light chain protein | 243.472 | 102.675 | | -1.2457 |
| AT4G10040 | | CYTC-2, cytochrome c-2 | 281.489 | 118.794 | | -1.2446 |
| AT5G15640 | | Mitochondrial substrate carrier family protein | 93.207 | 39.3618 | | -1.2436 |
| AT1G70780 | | unknown protein | 1068.35 | 451.218 | | -1.2435 |
| AT2G47970 | | Nuclear pore localisation protein NPL4 | 65.4127 | 27.6397 | | -1.2428 |
| AT5G24260 | | prolyl oligopeptidase family protein | 168.142 | 71.0576 | | -1.2426 |
| AT3G05700 | | Drought-responsive family protein | 28.8342 | 12.189 | | -1.2422 |
| AT4G29350 | | PFN2, PRF2, PRO2, profilin 2 | 688.303 | 291.369 | | -1.2402 |
| AT1G11020 | | RING/FYVE/PHD zinc finger superfamily protein | 48.4115 | 20.5031 | | -1.2395 |
| AT3G13610 | | 2-oxoglutarate (2OG) and Fe(II)-dependent oxygenase superfamily protein | 73.298 | 31.0472 | | -1.2393 |
| AT4G35770 | | ATSEN1, DIN1, SEN1, SEN1, Rhodanese/Cell cycle control phosphatase superfamily protein | 61.4599 | 26.0504 | | -1.2383 |
| AT3G63220 | | Galactose oxidase/kelch repeat superfamily protein | 20.9563 | 8.88342 | | -1.2382 |
| AT2G38230 | | ATPDX1.1, PDX1.1, pyridoxine biosynthesis 1.1 | 53.0601 | 22.4962 | | -1.238 |
| AT5G58380 | | CIPK10, PKS2, SIP1, SNRK3.8, SOS3-interacting protein 1 | 90.1232 | 38.2209 | | -1.2375 |
| AT1G55310 | | ATSCL33, SCL33, SR33, SC35-like splicing factor 33 | 62.6006 | 26.561 | | -1.2369 |
| AT5G39510 | | ATVTI11, ATVTI1A, SGR4, VTI11, VTI1A, ZIG, ZIG1, Vesicle transport v-SNARE family protein | 192.795 | 81.8063 | | -1.2368 |
| AT1G70160 | | unknown protein | 61.2197 | 26.0033 | | -1.2353 |
| AT1G18460 | | alpha/beta-Hydrolases superfamily protein | 142.281 | 60.435 | | -1.2353 |
| AT3G58730 | | vacuolar ATP synthase subunit D (VATD) / V-ATPase D subunit / vacuolar proton pump D subunit (VATPD) | 376.431 | 159.899 | | -1.2352 |
| AT3G18830 | | ATPLT5, ATPMT5, PMT5, polyol/monosaccharide transporter 5 | 35.1791 | 14.9441 | | -1.2351 |
| AT1G29640 | | Protein of unknown function, DUF584 | 219.314 | 93.2016 | | -1.2346 |
| AT1G14780 | | MAC/Perforin domain-containing protein | 50.9473 | 21.6532 | | -1.2344 |
| AT4G38420 | | sks9, SKU5 similar 9 | 11.0293 | 4.69104 | | -1.2334 |
| AT4G11860 | | Protein of unknown function (DUF544) | 75.0775 | 31.9417 | | -1.2329 |
| AT2G33470 | | ATGLTP1, GLTP1, glycolipid transfer protein 1 | 193.806 | 82.4859 | | -1.2324 |
| AT3G12630 | | A20/AN1-like zinc finger family protein | 222.784 | 94.833 | | -1.2322 |
| AT4G21105 | | cytochrome-c oxidases;electron carriers | 917.17 | 390.419 | | -1.2322 |
| AT2G33100 | | ATCSLD1, CSLD1, CSLD1, cellulose synthase-like D1 | 9.06158 | 3.85812 | | -1.2319 |
| AT4G20260 | | ATPCAP1, PCAP1, plasma-membrane associated cation-binding protein 1 | 225.514 | 96.1149 | | -1.2304 |
| AT2G01670 | | atnudt17, NUDT17, nudix hydrolase homolog 17 | 197.421 | 84.1543 | | -1.2302 |
| AT1G14530 | | THH1, Protein of unknown function (DUF1084) | 39.2488 | 16.7323 | | -1.23 |
| AT5G22350 | | ELM1, Protein of unknown function (DUF1022) | 63.7546 | 27.18 | | -1.23 |
| AT1G24440 | | RING/U-box superfamily protein | 47.8712 | 20.4145 | | -1.2296 |
| AT1G20450 | | ERD10, LTI29, LTI45, Dehydrin family protein | 2026.31 | 864.185 | | -1.2294 |
| AT5G47570 | | unknown protein | 285.769 | 121.953 | | -1.2285 |
| AT3G49780 | | ATPSK3 (FORMER SYMBOL), ATPSK4, PSK4, phytosulfokine 4 precursor | 1411.84 | 602.512 | | -1.2285 |
| AT4G12040 | | A20/AN1-like zinc finger family protein | 499.133 | 213.044 | | -1.2283 |
| AT1G66270 | | BGLU21, Glycosyl hydrolase superfamily protein | 392.327 | 167.493 | | -1.228 |
| AT4G35100 | | PIP2;7, PIP3, PIP3A, SIMIP, plasma membrane intrinsic protein 3 | 712.54 | 304.242 | | -1.2278 |
| AT5G52990 | | SNARE-like superfamily protein | 22.5286 | 9.62474 | | -1.2269 |
| AT3G63380 | | ATPase E1-E2 type family protein / haloacid dehalogenase-like hydrolase family protein | 85.7103 | 36.6285 | | -1.2265 |
| AT4G30600 | | signal recognition particle receptor alpha subunit family protein | 342.377 | 146.458 | | -1.2251 |
| AT3G54620 | | ATBZIP25, BZIP25, BZO2H4, basic leucine zipper 25 | 185.18 | 79.2276 | | -1.2249 |
| AT1G74840 | | Homeodomain-like superfamily protein | 705.371 | 302.081 | | -1.2235 |
| AT1G50640 | | ATERF3, ERF3, ethylene responsive element binding factor 3 | 121.806 | 52.18 | | -1.223 |
| AT4G39780 | | Integrase-type DNA-binding superfamily protein | 154.394 | 66.145 | | -1.2229 |
| AT5G46420 | | 16S rRNA processing protein RimM family | 67.7567 | 29.0512 | | -1.2218 |
| AT1G58290 | | HEMA1, Glutamyl-tRNA reductase family protein | 127.17 | 54.5355 | | -1.2215 |
| AT4G13010 | | Oxidoreductase, zinc-binding dehydrogenase family protein | 76.4882 | 32.8095 | | -1.2211 |
| AT3G05210 | | ERCC1, UVR7, nucleotide repair protein, putative | 22.4295 | 9.62639 | | -1.2203 |
| AT4G15410 | | PUX5, serine/threonine protein phosphatase 2A 55 kDa regulatory subunit B prime gamma | 73.8014 | 31.6752 | | -1.2203 |
| AT2G34650 | | ABR, PID, Protein kinase superfamily protein | 28.9916 | 12.4472 | | -1.2198 |
| AT4G12020 | | ATWRKY19, MAPKKK11, MEKK4, WRKY19, protein kinase family protein | 32.5696 | 13.9837 | | -1.2198 |
| AT5G01980 | | RING/U-box superfamily protein | 29.2821 | 12.5752 | | -1.2194 |
| AT1G09932 | | Phosphoglycerate mutase family protein | 66.0425 | 28.3703 | | -1.219 |
| AT3G11580 | | AP2/B3-like transcriptional factor family protein | 94.9186 | 40.8246 | | -1.2173 |
| AT2G41010 | | ATCAMBP25, CAMBP25, calmodulin (CAM)-binding protein of 25 kDa | 99.5977 | 42.8458 | | -1.217 |
| AT4G38730 | | Protein of unknown function (DUF803) | 35.0408 | 15.0826 | | -1.2162 |
| AT2G45980 | | unknown protein | 161.908 | 69.7028 | | -1.2159 |
| AT4G13660 | | ATPRR2, PRR2, pinoresinol reductase 2 | 43.3578 | 18.6721 | | -1.2154 |
| AT3G50110 | | ATPEN3, PEN3, PTEN 3 | 22.809 | 9.82651 | | -1.2149 |
| AT3G48570 | | secE/sec61-gamma protein transport protein | 225.697 | 97.2641 | | -1.2144 |
| AT5G24620 | | Pathogenesis-related thaumatin superfamily protein | 136.189 | 58.7006 | | -1.2142 |
| AT3G23180 | | HR-like lesion-inducing protein-related | 126.77 | 54.6428 | | -1.2141 |
| AT1G77000 | | ATSKP2;2, SKP2B, RNI-like superfamily protein | 160.574 | 69.242 | | -1.2135 |
| AT2G22760 | | basic helix-loop-helix (bHLH) DNA-binding superfamily protein | 110.257 | 47.5466 | | -1.2135 |
| AT4G32600 | | RING/U-box superfamily protein | 62.3853 | 26.9047 | | -1.2134 |
| AT4G33467 | | unknown protein | 503.939 | 217.403 | | -1.2129 |
| AT4G01550 | | anac069, NAC069, NAC domain containing protein 69 | 18.2169 | 7.86108 | | -1.2125 |
| AT5G11960 | | Protein of unknown function (DUF803) | 93.4594 | 40.3331 | | -1.2124 |
| AT1G70410 | | ATBCA4, BCA4, CA4, beta carbonic anhydrase 4 | 610.017 | 263.263 | | -1.2124 |
| AT2G01260 | | Protein of unknown function (DUF789) | 28.4979 | 12.2997 | | -1.2122 |
| AT2G25610 | | ATPase, F0/V0 complex, subunit C protein | 139.097 | 60.0439 | | -1.212 |
| AT3G12400 | | ATELC, ELC, Ubiquitin-conjugating enzyme/RWD-like protein | 103.913 | 44.8979 | | -1.2107 |
| AT4G15940 | | Fumarylacetoacetate (FAA) hydrolase family | 69.6264 | 30.0887 | | -1.2104 |
| AT3G60300 | | RWD domain-containing protein | 113.674 | 49.1471 | | -1.2097 |
| AT4G16760 | | ACX1, ATACX1, acyl-CoA oxidase 1 | 494.698 | 214.022 | | -1.2088 |
| AT2G46790 | | APRR9, PRR9, TL1, pseudo-response regulator 9 | 39.5027 | 17.1016 | | -1.2078 |
| AT2G36810 | | ARM repeat superfamily protein | 23.5163 | 10.1918 | | -1.2063 |
| AT4G38950 | | ATP binding microtubule motor family protein | 34.8146 | 15.0918 | | -1.2059 |
| AT4G05050 | | UBQ11, ubiquitin 11 | 407.234 | 176.662 | | -1.2049 |
| AT4G24400 | | ATCIPK8, CIPK8, PKS11, SnRK3.13, CBL-interacting protein kinase 8 | 86.0133 | 37.3208 | | -1.2046 |
| AT3G02700 | | NC domain-containing protein-related | 80.4128 | 34.9104 | | -1.2038 |
| AT5G12010 | | unknown protein | 479.603 | 208.224 | | -1.2037 |
| AT2G17550 | | unknown protein | 39.0094 | 16.9374 | | -1.2036 |
| AT4G33905 | | Peroxisomal membrane 22 kDa (Mpv17/PMP22) family protein | 57.5654 | 25.0099 | | -1.2027 |
| AT4G00720 | | ASKTHETA, ATSK32, SK32, shaggy-like protein kinase 32 | 135.23 | 58.7615 | | -1.2025 |
| AT3G01400 | | ARM repeat superfamily protein | 21.6041 | 9.39501 | | -1.2013 |
| AT1G71950 | | Proteinase inhibitor, propeptide | 492.082 | 214.177 | | -1.2001 |
| AT3G59090 | | CONTAINS InterPro DOMAIN/s: Protein of unknown function DUF1084 (InterPro:IPR009457) | 64.0939 | 27.903 | | -1.1998 |
| AT3G62290 | | ARFA1E, ATARFA1E, ADP-ribosylation factor A1E | 1398.18 | 608.985 | | -1.1991 |
| AT3G13275 | | unknown protein | 50.544 | 22.0179 | | -1.1989 |
| AT3G21630 | | CERK1, LYSM RLK1, chitin elicitor receptor kinase 1 | 88.2421 | 38.4498 | | -1.1985 |
| AT2G18160 | | ATBZIP2, bZIP2, GBF5, basic leucine-zipper 2 | 267.082 | 116.389 | | -1.1983 |
| AT1G15470 | | Transducin/WD40 repeat-like superfamily protein | 29.1532 | 12.7097 | | -1.1977 |
| AT5G61390 | | Polynucleotidyl transferase, ribonuclease H-like superfamily protein | 55.2953 | 24.1082 | | -1.1976 |
| AT4G01900 | | GLB1, PII, GLNB1 homolog | 54.5408 | 23.7929 | | -1.1968 |
| AT3G51790 | | ATG1, TG1, transmembrane protein G1P-related 1 | 25.4184 | 11.0929 | | -1.1962 |
| AT2G20960 | | pEARLI4, Arabidopsis phospholipase-like protein (PEARLI 4) family | 88.4976 | 38.6291 | | -1.196 |
| AT4G39980 | | DHS1, 3-deoxy-D-arabino-heptulosonate 7-phosphate synthase 1 | 250.454 | 109.341 | | -1.1957 |
| AT5G65430 | | 14-3-3KAPPA, GF14 KAPPA, GRF8, general regulatory factor 8 | 345.536 | 150.871 | | -1.1955 |
| AT2G36300 | | Integral membrane Yip1 family protein | 40.7937 | 17.8175 | | -1.1951 |
| AT3G61430 | | ATPIP1, PIP1, PIP1;1, PIP1A, plasma membrane intrinsic protein 1A | 258.699 | 113.041 | | -1.1944 |
| AT4G01370 | | ATMPK4, MPK4, MAP kinase 4 | 67.5353 | 29.5181 | | -1.194 |
| AT1G02360 | | Chitinase family protein | 162.167 | 70.8857 | | -1.1939 |
| AT5G17910 | | unknown protein | 32.2005 | 14.0885 | | -1.1926 |
| AT5G26990 | | Drought-responsive family protein | 63.2206 | 27.6831 | | -1.1914 |
| AT3G13930 | | Dihydrolipoamide acetyltransferase, long form protein | 248.711 | 108.991 | | -1.1903 |
| AT2G18760 | | CHR8, chromatin remodeling 8 | 15.6256 | 6.84883 | | -1.19 |
| AT3G18410 | | Complex I subunit NDUFS6 | 443.05 | 194.289 | | -1.1893 |
| AT3G51840 | | ACX4, ATG6, ATSCX, acyl-CoA oxidase 4 | 132.709 | 58.198 | | -1.1892 |
| AT3G05320 | | O-fucosyltransferase family protein | 47.7753 | 20.9784 | | -1.1874 |
| AT1G16840 | | unknown protein | 321.579 | 141.243 | | -1.187 |
| AT3G57630 | | exostosin family protein | 40.6597 | 17.859 | | -1.187 |
| AT5G14540 | | Protein of unknown function (DUF1421) | 54.5585 | 23.9687 | | -1.1867 |
| AT3G10920 | | ATMSD1, MEE33, MSD1, manganese superoxide dismutase 1 | 353.627 | 155.362 | | -1.1866 |
| AT1G53380 | | Plant protein of unknown function (DUF641) | 204.34 | 89.7754 | | -1.1866 |
| AT5G08535 | | D111/G-patch domain-containing protein | 64.4844 | 28.3386 | | -1.1862 |
| AT3G14770 | | Nodulin MtN3 family protein | 52.9303 | 23.2679 | | -1.1858 |
| AT1G80360 | | Pyridoxal phosphate (PLP)-dependent transferases superfamily protein | 51.4259 | 22.6069 | | -1.1857 |
| AT3G11420 | | Protein of unknown function (DUF604) | 46.2253 | 20.3288 | | -1.1852 |
| AT1G55530 | | RING/U-box superfamily protein | 187.9 | 82.6831 | | -1.1843 |
| AT1G49140 | | Complex I subunit NDUFS6 | 167.433 | 73.6867 | | -1.1841 |
| AT1G10150 | | Carbohydrate-binding protein | 122.213 | 53.7906 | | -1.184 |
| AT2G45010 | | PLAC8 family protein | 78.4777 | 34.5411 | | -1.184 |
| AT1G73250 | | ATFX, GER1, GDP-4-keto-6-deoxymannose-3,5-epimerase-4-reductase 1 | 73.8429 | 32.5125 | | -1.1835 |
| AT5G18800 | | Cox19-like CHCH family protein | 233.45 | 102.954 | | -1.1811 |
| AT2G32850 | | Protein kinase superfamily protein | 48.8521 | 21.5533 | | -1.1805 |
| AT1G10650 | | SBP (S-ribonuclease binding protein) family protein | 77.4289 | 34.163 | | -1.1804 |
| AT3G17090 | | Protein phosphatase 2C family protein | 133.495 | 58.9309 | | -1.1797 |
| AT1G68580 | | agenet domain-containing protein / bromo-adjacent homology (BAH) domain-containing protein | 115.215 | 50.8815 | | -1.1791 |
| AT5G02100 | | ORP3A, UNE18, Oxysterol-binding family protein | 44.405 | 19.616 | | -1.1787 |
| AT5G34850 | | ATPAP26, PAP26, purple acid phosphatase 26 | 175.202 | 77.4234 | | -1.1782 |
| AT1G29400 | | AML5, ML5, MEI2-like protein 5 | 198.348 | 87.6633 | | -1.178 |
| AT5G19860 | | Protein of unknown function, DUF538 | 162.762 | 71.951 | | -1.1777 |
| AT5G55850 | | NOI, RPM1-interacting protein 4 (RIN4) family protein | 218.099 | 96.4481 | | -1.1772 |
| AT1G80350 | | AAA1, ATKTN1, BOT1, ERH3, FRA2, FRC2, FTR, KTN1, LUE1, P-loop containing nucleoside triphosphate hydrolases superfamily protein | 42.3392 | 18.7238 | | -1.1771 |
| AT1G73030 | | CHMP1A, VPS46.2, SNF7 family protein | 182.181 | 80.5787 | | -1.1769 |
| AT4G31080 | | Protein of unknown function (DUF2296) | 63.3132 | 28.0074 | | -1.1767 |
| AT1G17500 | | ATPase E1-E2 type family protein / haloacid dehalogenase-like hydrolase family protein | 63.4985 | 28.0971 | | -1.1763 |
| AT3G49590 | | Autophagy-related protein 13 | 63.7078 | 28.2052 | | -1.1755 |
| AT3G11720 | | Polyketide cyclase/dehydrase and lipid transport superfamily protein | 20.8497 | 9.2311 | | -1.1755 |
| AT3G56410 | | Protein of unknown function (DUF3133) | 10.2364 | 4.53247 | | -1.1753 |
| AT1G36980 | | unknown protein | 103.52 | 45.8485 | | -1.175 |
| AT5G46860 | | ATSYP22, ATVAM3, SGR3, SYP22, VAM3, Syntaxin/t-SNARE family protein | 98.6521 | 43.7071 | | -1.1745 |
| AT4G34000 | | ABF3, DPBF5, abscisic acid responsive elements-binding factor 3 | 100.457 | 44.5271 | | -1.1738 |
| AT2G30250 | | ATWRKY25, WRKY25, WRKY DNA-binding protein 25 | 107.69 | 47.772 | | -1.1727 |
| AT1G48790 | | AMSH1, associated molecule with the SH3 domain of STAM 1 | 64.8856 | 28.7935 | | -1.1722 |
| AT4G20830 | | FAD-binding Berberine family protein | 611.451 | 271.399 | | -1.1718 |
| AT5G08680 | | ATP synthase alpha/beta family protein | 97.6736 | 43.3602 | | -1.1716 |
| AT1G16180 | | Serinc-domain containing serine and sphingolipid biosynthesis protein | 237.602 | 105.555 | | -1.1706 |
| AT2G26210 | | Ankyrin repeat family protein | 57.2813 | 25.4561 | | -1.1701 |
| AT5G52060 | | ATBAG1, BAG1, BCL-2-associated athanogene 1 | 101.007 | 44.8949 | | -1.1698 |
| AT1G69640 | | SBH1, sphingoid base hydroxylase 1 | 132.665 | 58.9873 | | -1.1693 |
| AT5G63570 | | GSA1, glutamate-1-semialdehyde-2,1-aminomutase | 70.572 | 31.3799 | | -1.1693 |
| AT2G23140 | | RING/U-box superfamily protein with ARM repeat domain | 28.0964 | 12.4949 | | -1.169 |
| AT4G21790 | | ATTOM1, TOM1, tobamovirus multiplication 1 | 246.026 | 109.42 | | -1.1689 |
| AT1G52200 | | PLAC8 family protein | 41.5399 | 18.4876 | | -1.1679 |
| AT1G27290 | | unknown protein | 379.088 | 168.749 | | -1.1677 |
| AT2G30020 | | Protein phosphatase 2C family protein | 60.9359 | 27.1335 | | -1.1672 |
| AT5G65210 | | TGA1, bZIP transcription factor family protein | 153.007 | 68.1556 | | -1.1667 |
| AT4G33930 | | Cupredoxin superfamily protein | 232.831 | 103.786 | | -1.1657 |
| AT3G12150 | | unknown protein | 28.1302 | 12.5421 | | -1.1653 |
| AT3G07340 | | basic helix-loop-helix (bHLH) DNA-binding superfamily protein | 170.155 | 75.8715 | | -1.1652 |
| AT1G15110 | | phosphatidyl serine synthase family protein | 149.973 | 66.874 | | -1.1652 |
| AT1G08480 | | unknown protein | 166.663 | 74.3403 | | -1.1647 |
| AT2G35680 | | Phosphotyrosine protein phosphatases superfamily protein | 160.344 | 71.5481 | | -1.1642 |
| AT3G06850 | | BCE2, DIN3, LTA1, 2-oxoacid dehydrogenases acyltransferase family protein | 62.2713 | 27.7867 | | -1.1642 |
| AT2G21870 | | MGP1, copper ion binding;cobalt ion binding;zinc ion binding | 549.933 | 245.482 | | -1.1636 |
| AT4G01700 | | Chitinase family protein | 328.298 | 146.588 | | -1.1632 |
| AT3G19970 | | alpha/beta-Hydrolases superfamily protein | 103.689 | 46.3211 | | -1.1625 |
| AT1G60140 | | ATTPS10, TPS10, TPS10, trehalose phosphate synthase | 118.214 | 52.8562 | | -1.1613 |
| AT3G20040 | | ATHXK4, HKL2, Hexokinase | 25.0599 | 11.2058 | | -1.1611 |
| AT1G61210 | | Transducin/WD40 repeat-like superfamily protein | 29.2216 | 13.073 | | -1.1605 |
| AT4G26650 | | RNA-binding (RRM/RBD/RNP motifs) family protein | 58.2405 | 26.0839 | | -1.1589 |
| AT5G67420 | | ASL39, LBD37, LOB domain-containing protein 37 | 63.8819 | 28.6128 | | -1.1587 |
| AT1G14870 | | PCR2, PLANT CADMIUM RESISTANCE 2 | 962.761 | 431.245 | | -1.1587 |
| AT5G42010 | | Transducin/WD40 repeat-like superfamily protein | 46.0646 | 20.6466 | | -1.1578 |
| AT3G59360 | | ATUTR6, UTR6, UDP-galactose transporter 6 | 77.7428 | 34.848 | | -1.1576 |
| AT3G52300 | | ATPQ, ATP synthase D chain, mitochondrial | 984.633 | 441.361 | | -1.1576 |
| AT1G23170 | | Protein of unknown function DUF2359, transmembrane | 57.0863 | 25.5955 | | -1.1573 |
| AT1G23710 | | Protein of unknown function (DUF1645) | 195.97 | 87.8665 | | -1.1573 |
| AT2G26600 | | Glycosyl hydrolase superfamily protein | 35.2209 | 15.7922 | | -1.1572 |
| AT3G10860 | | Cytochrome b-c1 complex, subunit 8 protein | 570.387 | 255.834 | | -1.1567 |
| AT5G11850 | | Protein kinase superfamily protein | 61.4303 | 27.5569 | | -1.1565 |
| AT1G03370 | | C2 calcium/lipid-binding and GRAM domain containing protein | 98.8063 | 44.3384 | | -1.1561 |
| AT3G55720 | | Protein of unknown function (DUF620) | 475.965 | 213.649 | | -1.1556 |
| AT3G02340 | | RING/U-box superfamily protein | 52.0351 | 23.3717 | | -1.1547 |
| AT1G59590 | | ZCF37, ZCF37 | 63.6587 | 28.5954 | | -1.1546 |
| AT2G19572 | | Potential natural antisense gene, locus overlaps with AT2G19570 | 182.798 | 82.1952 | | -1.1531 |
| AT1G15120 | | Ubiquinol-cytochrome C reductase hinge protein | 646.515 | 290.731 | | -1.153 |
| AT4G00585 | | unknown protein | 267.342 | 120.274 | | -1.1524 |
| AT1G74380 | | XXT5, xyloglucan xylosyltransferase 5 | 121.985 | 54.8819 | | -1.1523 |
| AT3G63310 | | BIL4, Bax inhibitor-1 family protein | 254.081 | 114.318 | | -1.1522 |
| AT1G77130 | | GUX3, PGSIP2, plant glycogenin-like starch initiation protein 2 | 52.4954 | 23.6296 | | -1.1516 |
| AT5G05850 | | PIRL1, plant intracellular ras group-related LRR 1 | 46.5866 | 20.971 | | -1.1515 |
| AT5G65290 | | LMBR1-like membrane protein | 58.8752 | 26.5078 | | -1.1512 |
| AT5G16120 | | alpha/beta-Hydrolases superfamily protein | 66.195 | 29.8132 | | -1.1508 |
| AT5G02170 | | Transmembrane amino acid transporter family protein | 101.152 | 45.575 | | -1.1502 |
| AT3G52200 | | LTA3, Dihydrolipoamide acetyltransferase, long form protein | 148.89 | 67.1345 | | -1.1491 |
| AT5G06320 | | NHL3, NDR1/HIN1-like 3 | 659.23 | 297.317 | | -1.1488 |
| AT2G23780 | | RING/U-box superfamily protein | 71.819 | 32.401 | | -1.1483 |
| AT5G02800 | | Protein kinase superfamily protein | 33.077 | 14.9237 | | -1.1482 |
| AT4G20410 | | GAMMA-SNAP, GSNAP, gamma-soluble NSF attachment protein | 60.8366 | 27.4976 | | -1.1456 |
| AT5G63910 | | FCLY, farnesylcysteine lyase | 58.6875 | 26.5321 | | -1.1453 |
| AT3G54300 | | ATVAMP727, VAMP727, VAMP727, vesicle-associated membrane protein 727 | 130.374 | 58.9712 | | -1.1446 |
| AT5G63260 | | Zinc finger C-x8-C-x5-C-x3-H type family protein | 63.4627 | 28.706 | | -1.1446 |
| AT1G22620 | | ATSAC1, Phosphoinositide phosphatase family protein | 28.7029 | 12.9851 | | -1.1443 |
| AT5G59820 | | RHL41, ZAT12, C2H2-type zinc finger family protein | 601.598 | 272.254 | | -1.1439 |
| AT4G22212 | | Arabidopsis defensin-like protein | 138.844 | 62.8338 | | -1.1439 |
| AT1G29690 | | CAD1, MAC/Perforin domain-containing protein | 74.6177 | 33.786 | | -1.1431 |
| AT2G37250 | | ADK, ATPADK1, adenosine kinase | 180.082 | 81.5465 | | -1.143 |
| AT5G48930 | | HCT, hydroxycinnamoyl-CoA shikimate/quinate hydroxycinnamoyl transferase | 65.5914 | 29.7136 | | -1.1424 |
| AT5G39660 | | CDF2, cycling DOF factor 2 | 62.8389 | 28.4673 | | -1.1424 |
| AT5G50850 | | MAB1, Transketolase family protein | 404.942 | 183.45 | | -1.1423 |
| AT3G27020 | | YSL6, YELLOW STRIPE like 6 | 31.3183 | 14.19 | | -1.1421 |
| AT5G04920 | | EAP30/Vps36 family protein | 63.3766 | 28.7155 | | -1.1421 |
| AT4G25650 | | ACD1-LIKE, PTC52, TIC55-IV, ACD1-like | 33.9499 | 15.3832 | | -1.1421 |
| AT1G35160 | | 14-3-3PHI, GF14 PHI, GRF4, GF14 protein phi chain | 396.066 | 179.483 | | -1.1419 |
| AT5G45130 | | ATRAB-F2A, ATRAB5A, ATRABF2A, RAB-F2A, RAB5A, RABF2A, RHA1, RAB homolog 1 | 199.808 | 90.5785 | | -1.1414 |
| AT3G08610 | | unknown protein | 830.467 | 376.513 | | -1.1412 |
| AT2G15620 | | ATHNIR, NIR, NIR1, nitrite reductase 1 | 87.0021 | 39.4568 | | -1.1408 |
| AT1G54710 | | ATATG18H, ATG18H, homolog of yeast autophagy 18 (ATG18) H | 73.9124 | 33.5335 | | -1.1402 |
| AT1G56280 | | ATDI19, DI19, drought-induced 19 | 205.648 | 93.3107 | | -1.1401 |
| AT4G38940 | | Galactose oxidase/kelch repeat superfamily protein | 40.418 | 18.3447 | | -1.1396 |
| AT1G72150 | | PATL1, PATELLIN 1 | 106.601 | 48.4544 | | -1.1375 |
| AT4G38220 | | Peptidase M20/M25/M40 family protein | 70.357 | 32.0089 | | -1.1362 |
| AT2G41740 | | ATVLN2, VLN2, villin 2 | 148 | 67.3669 | | -1.1355 |
| AT2G22500 | | ATPUMP5, DIC1, UCP5, uncoupling protein 5 | 82.0692 | 37.3739 | | -1.1348 |
| AT1G61100 | | disease resistance protein (TIR class), putative | 82.9566 | 37.8302 | | -1.1328 |
| AT3G25250 | | AGC2, AGC2-1, AtOXI1, OXI1, AGC (cAMP-dependent, cGMP-dependent and protein kinase C) kinase family protein | 114.308 | 52.1866 | | -1.1312 |
| AT4G25130 | | PMSR4, peptide met sulfoxide reductase 4 | 104.405 | 47.6701 | | -1.131 |
| AT5G67590 | | FRO1, NADH-ubiquinone oxidoreductase-related | 361.413 | 165.16 | | -1.1298 |
| AT3G55950 | | ATCRR3, CCR3, CRINKLY4 related 3 | 28.3315 | 12.9513 | | -1.1293 |
| AT5G13440 | | Ubiquinol-cytochrome C reductase iron-sulfur subunit | 88.4102 | 40.4281 | | -1.1289 |
| AT4G17170 | | AT-RAB2, ATRAB-B1B, ATRAB2A, ATRABB1C, RAB-B1B, RAB2A, RABB1C, RAB GTPase homolog B1C | 170.336 | 77.9387 | | -1.128 |
| AT5G20680 | | TBL16, TRICHOME BIREFRINGENCE-LIKE 16 | 37.4288 | 17.1269 | | -1.1279 |
| AT1G08920 | | ESL1, ERD (early response to dehydration) six-like 1 | 254.934 | 116.69 | | -1.1275 |
| AT4G28350 | | Concanavalin A-like lectin protein kinase family protein | 25.872 | 11.8471 | | -1.1269 |
| AT1G05850 | | ATCTL1, CTL1, ELP, ELP1, ERH2, HOT2, POM1, Chitinase family protein | 359.024 | 164.579 | | -1.1253 |
| AT5G54810 | | ATTSB1, TRP2, TRPB, TSB1, tryptophan synthase beta-subunit 1 | 408.726 | 187.42 | | -1.1249 |
| AT4G10140 | | unknown protein | 88.7803 | 40.7406 | | -1.1238 |
| AT1G29760 | | Putative adipose-regulatory protein (Seipin) | 53.1879 | 24.4435 | | -1.1217 |
| AT5G51450 | | RIN3, RPM1 interacting protein 3 | 23.5765 | 10.8369 | | -1.1214 |
| AT5G57610 | | Protein kinase superfamily protein with octicosapeptide/Phox/Bem1p domain | 50.1205 | 23.0448 | | -1.121 |
| AT5G37480 | | unknown protein | 90.0775 | 41.4186 | | -1.1209 |
| AT5G04460 | | RING/U-box superfamily protein | 47.1495 | 21.6808 | | -1.1208 |
| AT5G05570 | | transducin family protein / WD-40 repeat family protein | 30.3129 | 13.9541 | | -1.1193 |
| AT3G56880 | | VQ motif-containing protein | 361.289 | 166.446 | | -1.1181 |
| AT3G57450 | | unknown protein | 114.079 | 52.5734 | | -1.1176 |
| AT5G42300 | | UBL5, ubiquitin-like protein 5 | 614.662 | 283.277 | | -1.1176 |
| AT1G65430 | | ARI8, ATARI8, IBR domain-containing protein | 48.0461 | 22.1443 | | -1.1175 |
| AT2G01180 | | ATLPP1, ATPAP1, LPP1, PAP1, phosphatidic acid phosphatase 1 | 40.5073 | 18.6739 | | -1.1172 |
| AT3G59920 | | ATGDI2, GDI2, RAB GDP dissociation inhibitor 2 | 271.789 | 125.298 | | -1.1171 |
| AT1G12360 | | KEU, Sec1/munc18-like (SM) proteins superfamily | 142.14 | 65.539 | | -1.1169 |
| AT4G18140 | | SSP4b, SCP1-like small phosphatase 4b | 83.5229 | 38.5121 | | -1.1169 |
| AT4G14230 | | CBS domain-containing protein with a domain of unknown function (DUF21) | 18.9351 | 8.75896 | | -1.1122 |
| AT1G18470 | | Transmembrane Fragile-X-F-associated protein | 104.712 | 48.4471 | | -1.1119 |
| AT2G46540 | | unknown protein | 291.699 | 134.979 | | -1.1117 |
| AT5G12200 | | PYD2, pyrimidine 2 | 55.3213 | 25.5991 | | -1.1117 |
| AT3G17240 | | mtLPD2, lipoamide dehydrogenase 2 | 245.422 | 113.617 | | -1.1111 |
| AT3G27960 | | Tetratricopeptide repeat (TPR)-like superfamily protein | 62.2265 | 28.8217 | | -1.1104 |
| AT3G14590 | | NTMC2T6.2, NTMC2TYPE6.2, Calcium-dependent lipid-binding (CaLB domain) family protein | 78.1298 | 36.2022 | | -1.1098 |
| AT2G25460 | | CONTAINS InterPro DOMAIN/s: C2 calcium-dependent membrane targeting (InterPro:IPR000008) | 148.263 | 68.7616 | | -1.1085 |
| AT5G37790 | | Protein kinase superfamily protein | 40.1903 | 18.6451 | | -1.1081 |
| AT5G28050 | | Cytidine/deoxycytidylate deaminase family protein | 515.752 | 239.296 | | -1.1079 |
| AT3G12620 | | Protein phosphatase 2C family protein | 70.9108 | 32.903 | | -1.1078 |
| AT1G14450 | | NADH dehydrogenase (ubiquinone)s | 180.953 | 83.9771 | | -1.1076 |
| AT5G20490 | | ATXIK, XI-17, XIK, Myosin family protein with Dil domain | 42.3812 | 19.6762 | | -1.107 |
| AT3G01810 | | function unknown | 18.2464 | 8.47447 | | -1.1064 |
| AT1G11400 | | PYM, partner of Y14-MAGO | 107.741 | 50.0477 | | -1.1062 |
| AT2G41475 | | Embryo-specific protein 3, (ATS3) | 196.753 | 91.4025 | | -1.1061 |
| AT5G59570 | | Homeodomain-like superfamily protein | 61.3321 | 28.4982 | | -1.1058 |
| AT2G05170 | | ATVPS11, VPS11, vacuolar protein sorting 11 | 24.2014 | 11.2467 | | -1.1056 |
| AT5G26600 | | Pyridoxal phosphate (PLP)-dependent transferases superfamily protein | 73.489 | 34.1652 | | -1.105 |
| AT4G17230 | | SCL13, SCARECROW-like 13 | 166.049 | 77.2147 | | -1.1047 |
| AT5G47880 | | ERF1-1, eukaryotic release factor 1-1 | 264.006 | 122.814 | | -1.1041 |
| AT5G13070 | | MSF1-like family protein | 128.529 | 59.8338 | | -1.1031 |
| AT5G67320 | | HOS15, WD-40 repeat family protein | 65.5406 | 30.5166 | | -1.1028 |
| AT4G12250 | | GAE5, UDP-D-glucuronate 4-epimerase 5 | 50.134 | 23.4041 | | -1.099 |
| AT1G32400 | | TOM2A, tobamovirus multiplication 2A | 132.682 | 61.9542 | | -1.0987 |
| AT2G20330 | | Transducin/WD40 repeat-like superfamily protein | 74.8511 | 34.9562 | | -1.0985 |
| AT3G01090 | | AKIN10, KIN10, KIN10, SNRK1.1, SNF1 kinase homolog 10 | 50.0675 | 23.3842 | | -1.0983 |
| AT5G12430 | | Heat shock protein DnaJ with tetratricopeptide repeat | 19.4435 | 9.08701 | | -1.0974 |
| AT5G42870 | | ATPAH2, PAH2, phosphatidic acid phosphohydrolase 2 | 76.6876 | 35.8462 | | -1.0972 |
| AT5G13190 | | CONTAINS InterPro DOMAIN/s: LPS-induced tumor necrosis factor alpha factor (InterPro:IPR006629) | 244.012 | 114.144 | | -1.0961 |
| AT4G28060 | | Cytochrome c oxidase, subunit Vib family protein | 400.379 | 187.316 | | -1.0959 |
| AT3G14560 | | unknown protein | 87.4144 | 40.957 | | -1.0938 |
| AT2G24170 | | Endomembrane protein 70 protein family | 52.4348 | 24.573 | | -1.0935 |
| AT1G51420 | | ATSPP1, SPP1, sucrose-phosphatase 1 | 133.461 | 62.6726 | | -1.0905 |
| AT2G25430 | | epsin N-terminal homology (ENTH) domain-containing protein / clathrin assembly protein-related | 87.0358 | 40.883 | | -1.0901 |
| AT2G18730 | | ATDGK3, DGK3, diacylglycerol kinase 3 | 51.7958 | 24.3484 | | -1.089 |
| AT1G11820 | | O-Glycosyl hydrolases family 17 protein | 25.613 | 12.0497 | | -1.0879 |
| AT5G47180 | | Plant VAMP (vesicle-associated membrane protein) family protein | 84.7183 | 39.8722 | | -1.0873 |
| AT3G61800 | | CONTAINS InterPro DOMAIN/s: Protein of unknown function DUF2043 (InterPro:IPR018610), ENTH/VHS (InterPro:IPR008942) | 57.132 | 26.8988 | | -1.0868 |
| AT4G32040 | | KNAT5, KNOTTED1-like homeobox gene 5 | 66.033 | 31.0963 | | -1.0864 |
| AT2G46340 | | SPA1, SPA (suppressor of phyA-105) protein family | 78.1265 | 36.8071 | | -1.0858 |
| AT4G39820 | | Tetratricopeptide repeat (TPR)-like superfamily protein | 55.7166 | 26.298 | | -1.0832 |
| AT4G36630 | | EMB2754, Vacuolar sorting protein 39 | 36.5201 | 17.2482 | | -1.0822 |
| AT1G13350 | | Protein kinase superfamily protein | 50.3157 | 23.8079 | | -1.0796 |
| AT5G19590 | | Protein of unknown function, DUF538 | 105.21 | 49.8041 | | -1.0789 |
| AT3G22200 | | GABA-T, HER1, POP2, Pyridoxal phosphate (PLP)-dependent transferases superfamily protein | 215.053 | 101.86 | | -1.0781 |
| AT3G05420 | | ACBP4, acyl-CoA binding protein 4 | 81.9277 | 38.8215 | | -1.0775 |
| AT2G03220 | | ATFT1, ATFUT1, FT1, MUR2, fucosyltransferase 1 | 33.5285 | 15.8898 | | -1.0773 |
| AT4G32470 | | Cytochrome bd ubiquinol oxidase, 14kDa subunit | 279.211 | 132.354 | | -1.077 |
| AT5G52840 | | NADH-ubiquinone oxidoreductase-related | 189.27 | 89.7926 | | -1.0758 |
| AT5G56750 | | NDL1, N-MYC downregulated-like 1 | 167.173 | 79.4659 | | -1.0729 |
| AT1G73260 | | ATKTI1, KTI1, kunitz trypsin inhibitor 1 | 286.717 | 136.376 | | -1.072 |
| AT1G20100 | | unknown protein | 377.277 | 179.697 | | -1.0701 |
| AT2G33220 | | GRIM-19 protein | 204.967 | 97.6293 | | -1.07 |
| AT4G29790 | | unknown protein | 25.128 | 11.9841 | | -1.0682 |
| AT1G53590 | | NTMC2T6.1, NTMC2TYPE6.1, Calcium-dependent lipid-binding (CaLB domain) family protein | 46.4609 | 22.1611 | | -1.068 |
| AT1G50360 | | ATVIIIA, VIIIA, P-loop containing nucleoside triphosphate hydrolases superfamily protein | 23.2053 | 11.073 | | -1.0674 |
| AT1G12470 | | zinc ion binding | 40.089 | 19.1493 | | -1.0659 |
| AT2G35190 | | ATNPSN11, NPSN11, NSPN11, novel plant snare 11 | 61.2385 | 29.2884 | | -1.0641 |
| AT4G24020 | | NLP7, NIN like protein 7 | 72.7891 | 34.8201 | | -1.0638 |
| AT3G10550 | | Myotubularin-like phosphatases II superfamily | 38.0118 | 18.1849 | | -1.0637 |
| AT4G28540 | | CKL6, PAPK1, casein kinase I-like 6 | 87.041 | 41.6859 | | -1.0621 |
| AT1G79230 | | ATMST1, ATRDH1, MST1, ST1, STR1, mercaptopyruvate sulfurtransferase 1 | 98.0262 | 46.9804 | | -1.0611 |
| AT3G19870 | | unknown protein | 25.9462 | 12.4481 | | -1.0596 |
| AT2G01820 | | Leucine-rich repeat protein kinase family protein | 53.323 | 25.5956 | | -1.0589 |
| AT4G33530 | | KUP5, K+ uptake permease 5 | 78.8427 | 37.8523 | | -1.0586 |
| AT3G18290 | | BTS, EMB2454, zinc finger protein-related | 87.6759 | 42.1226 | | -1.0576 |
| AT5G04740 | | ACT domain-containing protein | 229.554 | 110.421 | | -1.0558 |
| AT2G47140 | | NAD(P)-binding Rossmann-fold superfamily protein | 192.21 | 92.5895 | | -1.0538 |
| AT5G19050 | | alpha/beta-Hydrolases superfamily protein | 84.3927 | 40.6916 | | -1.0524 |
| AT2G45910 | | U-box domain-containing protein kinase family protein | 34.3303 | 16.5747 | | -1.0505 |
| AT3G47080 | | Tetratricopeptide repeat (TPR)-like superfamily protein | 135.781 | 65.5732 | | -1.0501 |
| AT1G10940 | | ASK1, SNRK2-4, SNRK2.4, SRK2A, Protein kinase superfamily protein | 109.869 | 53.0696 | | -1.0498 |
| AT2G38840 | | Guanylate-binding family protein | 53.7518 | 25.9669 | | -1.0496 |
| AT3G05545 | | RING/U-box superfamily protein | 75.4836 | 36.6007 | | -1.0443 |
| AT2G41640 | | Glycosyltransferase family 61 protein | 74.2369 | 36.01 | | -1.0437 |
| AT5G54730 | | ATATG18F, ATG18F, G18F, homolog of yeast autophagy 18 (ATG18) F | 84.3504 | 41.039 | | -1.0394 |
| AT2G16365 | | F-box family protein | 64.3346 | 31.3194 | | -1.0385 |
| AT3G21510 | | AHP1, histidine-containing phosphotransmitter 1 | 197.317 | 96.1353 | | -1.0374 |
| AT1G67310 | | Calmodulin-binding transcription activator protein with CG-1 and Ankyrin domains | 111.217 | 54.1888 | | -1.0373 |
| AT1G64230 | | UBC28, ubiquitin-conjugating enzyme 28 | 458.474 | 223.702 | | -1.0353 |
| AT4G19640 | | ARA-7, ARA7, ATRAB-F2B, ATRAB5B, ATRABF2B, RAB-F2B, RABF2B, Ras-related small GTP-binding family protein | 209.27 | 102.185 | | -1.0342 |
| AT1G29280 | | ATWRKY65, WRKY65, WRKY DNA-binding protein 65 | 177.666 | 87.5543 | | -1.0209 |
| AT5G24313 | | unknown protein | 46.1877 | 0 | #NAME? | |
| AT4G33730 | | CAP (Cysteine-rich secretory proteins, Antigen 5, and Pathogenesis-related 1 protein) superfamily protein | 29.2434 | 0 | #NAME? | |
| AT1G34540 | | CYP94D1, cytochrome P450, family 94, subfamily D, polypeptide 1 | 25.9102 | 0 | #NAME? | |
| AT4G25190 | | Family of unknown function (DUF566) | 21.5618 | 0 | #NAME? | |
| AT4G06536 | | SPla/RYanodine receptor (SPRY) domain-containing protein | 21.5337 | 0 | #NAME? | |
| AT1G60050 | | Nodulin MtN21 /EamA-like transporter family protein | 17.708 | 0 | #NAME? | |
| AT5G43175 | | basic helix-loop-helix (bHLH) DNA-binding superfamily protein | 14.6086 | 0 | #NAME? | |
| AT2G44340 | | VQ motif-containing protein | 13.3286 | 0 | #NAME? | |
| AT4G04900 | | RIC10, ROP-interactive CRIB motif-containing protein 10 | 12.4933 | 0 | #NAME? | |
| AT1G05990 | | RHS1, EF hand calcium-binding protein family | 11.0517 | 0 | #NAME? | |
| AT3G50710 | | F-box/RNI-like/FBD-like domains-containing protein | 10.9567 | 0 | #NAME? | |
| AT3G58000 | | VQ motif-containing protein | 10.8119 | 0 | #NAME? | |
| AT4G06534 | | unknown protein | 10.6746 | 0 | #NAME? | |
| AT1G24485 | | function unknown | 10.4234 | 0 | #NAME? | |
| AT2G26410 | | Iqd4, IQ-domain 4 | 9.48085 | 0 | #NAME? | |
| AT1G35330 | | RING/U-box superfamily protein | 9.22559 | 0 | #NAME? | |
| AT1G51810 | | Leucine-rich repeat protein kinase family protein | 7.33672 | 0 | #NAME? | |
| AT1G51880 | | RHS6, root hair specific 6 | 6.35543 | 0 | #NAME? | |
| AT1G26250 | | Proline-rich extensin-like family protein | 6.18218 | 0 | #NAME? | |
| AT5G61650 | | CYCP4, CYCP4;2, CYCLIN P4;2 | 6.11587 | 0 | #NAME? | |
| AT3G20557 | | unknown protein | 5.65851 | 0 | #NAME? | |
| AT1G08100 | | ACH2, ATNRT2.2, NRT2.2, NRT2;2AT, nitrate transporter 2.2 | 4.91682 | 0 | #NAME? | |
| AT5G21130 | | Late embryogenesis abundant (LEA) hydroxyproline-rich glycoprotein family | 4.04504 | 0 | #NAME? | |
| AT5G22560 | | Plant protein of unknown function (DUF247) | 3.89387 | 0 | #NAME? | |
| AT4G34930 | | PLC-like phosphodiesterases superfamily protein | 3.67788 | 0 | #NAME? | |
| AT5G54050 | | Cysteine/Histidine-rich C1 domain family protein | 3.26743 | 0 | #NAME? | |
| AT2G29000 | | Leucine-rich repeat protein kinase family protein | 3.02968 | 0 | #NAME? | |
| AT5G58360 | | ATOFP3, OFP3, ovate family protein 3 | 2.8683 | 0 | #NAME? | |
| AT1G34330 | | pseudogene, putative peroxidase | 2.80905 | 0 | #NAME? | |
| AT1G61080 | | Hydroxyproline-rich glycoprotein family protein | 2.79338 | 0 | #NAME? | |
| AT3G46340 | | Leucine-rich repeat protein kinase family protein | 2.43475 | 0 | #NAME? | |
| AT1G34520 | | MBOAT (membrane bound O-acyl transferase) family protein | 2.21347 | 0 | #NAME? | |
| AT4G12360 | | Bifunctional inhibitor/lipid-transfer protein/seed storage 2S albumin superfamily protein | 2.02718 | 0 | #NAME? | |
| AT2G14760 | | basic helix-loop-helix (bHLH) DNA-binding superfamily protein | 1.99999 | 0 | #NAME? | |
| AT5G05420 | | FKBP-like peptidyl-prolyl cis-trans isomerase family protein | 1.99553 | 0 | #NAME? | |
| AT5G54790 | | unknown protein | 1.91974 | 0 | #NAME? | |
| AT2G37740 | | ATZFP10, ZFP10, zinc-finger protein 10 | 1.88115 | 0 | #NAME? | |
| AT4G10860 | | unknown protein | 1.83738 | 0 | #NAME? | |
| AT3G18460 | | PLAC8 family protein | 1.75622 | 0 | #NAME? | |
| AT4G19800 | | Glycosyl hydrolase family protein with chitinase insertion domain | 1.70628 | 0 | #NAME? | |
| AT5G56200 | | C2H2 type zinc finger transcription factor family | 1.6753 | 0 | #NAME? | |
| AT4G19760 | | Glycosyl hydrolase family protein with chitinase insertion domain | 1.65757 | 0 | #NAME? | |
| AT3G18470 | | PLAC8 family protein | 1.63194 | 0 | #NAME? | |
| AT5G48700 | | Ubiquitin-like superfamily protein | 1.54823 | 0 | #NAME? | |
| AT3G04735 | | RALFL21, RALF-like 21 | 1.53836 | 0 | #NAME? | |
| AT4G38850 | | ATSAUR15, SAUR-AC1, SAUR15, SAUR_AC1, SAUR-like auxin-responsive protein family | 1.45983 | 0 | #NAME? | |
| AT2G29010 | | pseudogene, receptor protein kinase | 1.4021 | 0 | #NAME? | |
| AT5G41761 | | unknown protein | 1.37593 | 0 | #NAME? | |
| AT2G43220 | | Cysteine/Histidine-rich C1 domain family protein | 1.36488 | 0 | #NAME? | |
| AT1G11920 | | Pectin lyase-like superfamily protein | 1.31645 | 0 | #NAME? | |
| AT4G25930 | | Protein of unknown function (DUF295) | 1.30174 | 0 | #NAME? | |
| AT1G69230 | | SP1L2, SPIRAL1-like2 | 1.27038 | 0 | #NAME? | |
| AT3G25650 | | ASK15, SK15, SKP1-like 15 | 1.26335 | 0 | #NAME? | |
| AT3G46370 | | Leucine-rich repeat protein kinase family protein | 1.21859 | 0 | #NAME? | |
| AT4G19770 | | Glycosyl hydrolase family protein with chitinase insertion domain | 1.20959 | 0 | #NAME? | |
| AT1G33870 | | P-loop containing nucleoside triphosphate hydrolases superfamily protein | 1.18212 | 0 | #NAME? | |
| AT5G22390 | | Protein of unknown function (DUF3049) | 1.14436 | 0 | #NAME? | |
| AT2G23240 | | Plant EC metallothionein-like protein, family 15 | 1.13736 | 0 | #NAME? | |
| AT5G23903 | | unknown protein | 1.07389 | 0 | #NAME? | |
| AT3G61900 | | SAUR-like auxin-responsive protein family | 1.06586 | 0 | #NAME? | |
| AT3G46480 | | 2-oxoglutarate (2OG) and Fe(II)-dependent oxygenase superfamily protein | 1.00277 | 0 | #NAME? | |
