# Supplementary material for: Re-analysis of RNA-seq transcriptome data reveals new aspects of gene activity in Arabidopsis root hairs
Source: Front Plant Sci. 2015 Jun 8;6:421. doi: 10.3389/fpls.2015.00421 (PMC4458573; doi:10.3389/fpls.2015.00421)
Supplement: Supplementary file 12 [file Table7.DOC]

**Table S7** List of the 61 genes only expressed in root hairs (RH, RPKM>1) compared with non-root hair tissues (NRH, RPKM=0).

| AGI | Annotation | RH(RPKM) | NRH(RPKM) |
| --- | --- | --- | --- |
| AT5G24313 | unknown protein | 46.1877 | 0 |
| AT4G33730 | CAP superfamily protein | 29.2434 | 0 |
| AT1G34540 | CYP94D1 | 25.9102 | 0 |
| AT4G25190 | Family of unknown function (DUF566) | 21.5618 | 0 |
| AT4G06536 | SPla/RYanodine receptor | 21.5337 | 0 |
| AT1G60050 | Nodulin MtN21 | 17.708 | 0 |
| AT5G43175 | basic helix-loop-helix DNA-binding protein | 14.6086 | 0 |
| AT2G44340 | VQ motif-containing protein | 13.3286 | 0 |
| AT4G04900 | RIC10, | 12.4933 | 0 |
| AT1G05990 | RHS1, EF hand calcium-binding protein | 11.0517 | 0 |
| AT3G50710 | F-box/RNI-like/FBD-like protein | 10.9567 | 0 |
| AT3G58000 | VQ motif-containing protein | 10.8119 | 0 |
| AT4G06534 | unknown protein | 10.6746 | 0 |
| AT1G24485 | function unknown | 10.4234 | 0 |
| AT2G26410 | Iqd4, IQ-domain 4 | 9.48085 | 0 |
| AT1G35330 | RING/U-box superfamily protein | 9.22559 | 0 |
| AT1G51810 | Leucine-rich repeat protein kinase | 7.33672 | 0 |
| AT1G51880 | RHS6, root hair specific 6 | 6.35543 | 0 |
| AT1G26250 | Proline-rich extensin-like family protein | 6.18218 | 0 |
| AT5G61650 | CYCP4, CYCP4;2, CYCLIN P4;2 | 6.11587 | 0 |
| AT3G20557 | unknown protein | 5.65851 | 0 |
| AT1G08100 | ATNRT2.2, nitrate transporter 2.2 | 4.91682 | 0 |
| AT5G21130 | hydroxyproline-rich glycoprotein family | 4.04504 | 0 |
| AT5G22560 | Plant protein of unknown function (DUF247) | 3.89387 | 0 |
| AT4G34930 | PLC-like phosphodiesterases superfamily protein | 3.67788 | 0 |
| AT5G54050 | Cysteine/Histidine-rich C1 domain protein | 3.26743 | 0 |
| AT2G29000 | Leucine-rich repeat protein kinase protein | 3.02968 | 0 |
| AT5G58360 | ATOFP3, OFP3, ovate family protein 3 | 2.8683 | 0 |
| AT1G34330 | pseudogene, putative peroxidase | 2.80905 | 0 |
| AT1G61080 | Hydroxyproline-rich glycoprotein protein | 2.79338 | 0 |
| AT3G46340 | Leucine-rich repeat protein kinase protein | 2.43475 | 0 |
| AT1G34520 | MBOAT (membrane bound O-acyl transferase) | 2.21347 | 0 |
| AT4G12360 | Bifunctional inhibitor/lipid-transfer protein | 2.02718 | 0 |
| AT2G14760 | basic helix-loop-helix DNA-binding protein | 1.99999 | 0 |
| AT5G05420 | FKBP-like peptidyl-prolyl cis-trans isomerase | 1.99553 | 0 |
| AT5G54790 | unknown protein | 1.91974 | 0 |
| AT2G37740 | ATZFP10, ZFP10, zinc-finger protein 10 | 1.88115 | 0 |
| AT4G10860 | unknown protein | 1.83738 | 0 |
| AT3G18460 | PLAC8 family protein | 1.75622 | 0 |
| AT4G19800 | Glycosyl hydrolase | 1.70628 | 0 |
| AT5G56200 | C2H2 type zinc finger transcription factor family | 1.6753 | 0 |
| AT4G19760 | Glycosyl hydrolase | 1.65757 | 0 |
| AT3G18470 | PLAC8 family protein | 1.63194 | 0 |
| AT5G48700 | Ubiquitin-like superfamily protein | 1.54823 | 0 |
| AT3G04735 | RALFL21, RALF-like 21 | 1.53836 | 0 |
| AT4G38850 | ATSAUR15, SAUR-like auxin-responsive protein | 1.45983 | 0 |
| AT2G29010 | pseudogene, receptor protein kinase | 1.4021 | 0 |
| AT5G41761 | unknown protein | 1.37593 | 0 |
| AT2G43220 | Cysteine/Histidine-rich C1 domain family protein | 1.36488 | 0 |
| AT1G11920 | Pectin lyase-like superfamily protein | 1.31645 | 0 |
| AT4G25930 | Protein of unknown function (DUF295) | 1.30174 | 0 |
| AT1G69230 | SP1L2, SPIRAL1-like2 | 1.27038 | 0 |
| AT3G25650 | ASK15, SK15, SKP1-like 15 | 1.26335 | 0 |
| AT3G46370 | Leucine-rich repeat protein kinase | 1.21859 | 0 |
| AT4G19770 | Glycosyl hydrolase f | 1.20959 | 0 |
| AT1G33870 | nucleoside triphosphate hydrolases protein | 1.18212 | 0 |
| AT5G22390 | Protein of unknown function (DUF3049) | 1.14436 | 0 |
| AT2G23240 | Plant EC metallothionein-like protein, family 15 | 1.13736 | 0 |
| AT5G23903 | unknown protein | 1.07389 | 0 |
| AT3G61900 | SAUR-like auxin-responsive protein family | 1.06586 | 0 |
| AT3G46480 | 2-oxoglutarate (2OG) and Fe(II)-dependent oxygenase superfamily protein | 1.00277 | 0 |
