# Supplementary material for: Re-analysis of RNA-seq transcriptome data reveals new aspects of gene activity in Arabidopsis root hairs
Source: Front Plant Sci. 2015 Jun 8;6:421. doi: 10.3389/fpls.2015.00421 (PMC4458573; doi:10.3389/fpls.2015.00421)
Supplement: Supplementary file 13 [file Table8.DOC]

**Table S8** List of the 1259 overlapping genes between this study and previous study (Lan et al., 2013)

| AGI | Annotation | | RH(RPKM) | NRH(RPKM) |
| --- | --- | --- | --- | --- |
| AT2G24980 | | Proline-rich extensin-like family protein | 282.55 | 0.145177 |
| AT5G06630 | | proline-rich extensin-like family protein | 350.094 | 0.263724 |
| AT1G12560 | | ATEXP7 | 390.921 | 0.365196 |
| AT4G40090 | | AGP3, arabinogalactan protein 3 | 1002.28 | 1.07251 |
| AT3G62680 | | ATPRP3, PRP3, proline-rich protein 3 | 419.939 | 0.453138 |
| AT5G04960 | | Plant invertase/pectin methylesterase inhibitor superfamily | 179.825 | 0.201765 |
| AT3G09925 | | Pollen Ole e 1 allergen and extensin family protein | 685.787 | 0.79408 |
| AT4G25820 | | ATXTH14, XTH14, XTR9, xyloglucan endotransglucosylase/hydrolase 14 | 758.459 | 0.933845 |
| AT5G06640 | | Proline-rich extensin-like family protein | 300.574 | 0.375472 |
| AT3G54590 | | ATHRGP1, HRGP1, hydroxyproline-rich glycoprotein | 882.994 | 1.10396 |
| AT5G67400 | | RHS19, root hair specific 19 | 456.332 | 0.570824 |
| AT4G13390 | | Proline-rich extensin-like family protein | 346.903 | 0.436798 |
| AT4G02270 | | RHS13, root hair specific 13 | 828.721 | 1.12794 |
| AT4G00680 | | ADF8, actin depolymerizing factor 8 | 479.401 | 0.676228 |
| AT1G62980 | | ATEXP18, ATEXPA18, ATHEXP ALPHA 1.25, EXP18, EXPA18, expansin A18 | 208.435 | 0.345746 |
| AT5G57530 | | AtXTH12, XTH12, xyloglucan endotransglucosylase/hydrolase 12 | 125.269 | 0.209592 |
| AT5G35190 | | proline-rich extensin-like family protein | 467.479 | 0.808864 |
| AT2G29620 | | unknown protein | 35.8692 | 0.067347 |
| AT1G30870 | | Peroxidase superfamily protein | 413.75 | 0.811178 |
| AT1G12040 | | LRX1, leucine-rich repeat/extensin 1 | 184.771 | 0.367391 |
| AT5G05500 | | Pollen Ole e 1 allergen and extensin family protein | 467.413 | 0.954079 |
| AT5G11440 | | CID5, IPD1, CTC-interacting domain 5 | 141.179 | 0.300056 |
| AT1G48930 | | AtGH9C1, GH9C1, glycosyl hydrolase 9C1 | 212.097 | 0.450867 |
| AT1G54970 | | ATPRP1, PRP1, RHS7, proline-rich protein 1 | 197.916 | 0.420925 |
| AT2G41970 | | Protein kinase superfamily protein | 232.38 | 0.551853 |
| AT2G47540 | | Pollen Ole e 1 allergen and extensin family protein | 213.419 | 0.513428 |
| AT5G22410 | | RHS18, root hair specific 18 | 106.322 | 0.292415 |
| AT2G47360 | | unknown protein | 62.0735 | 0.184463 |
| AT2G30670 | | NAD(P)-binding Rossmann-fold superfamily protein | 112.11 | 0.339629 |
| AT3G10710 | | RHS12, root hair specific 12 | 57.5381 | 0.189001 |
| AT2G45890 | | ATROPGEF4, RHS11, ROPGEF4, RHO guanyl-nucleotide exchange factor 4 | 101.066 | 0.368687 |
| AT5G22555 | | unknown protein | 196.529 | 0.860094 |
| AT5G40860 | | unknown protein | 136.684 | 0.628081 |
| AT3G54580 | | Proline-rich extensin-like family protein | 1903.3 | 8.85952 |
| AT4G09990 | | Protein of unknown function (DUF579) | 189.187 | 0.903108 |
| AT1G08090 | | ACH1, ATNRT2.1, ATNRT2:1, LIN1, NRT2, NRT2.1, NRT2:1, NRT2;1AT, nitrate transporter 2:1 | 52.9332 | 0.266549 |
| AT3G60330 | | AHA7, HA7, H(+)-ATPase 7 | 310.855 | 1.56648 |
| AT2G33460 | | RIC1, ROP-interactive CRIB motif-containing protein 1 | 58.361 | 0.294145 |
| AT4G26010 | | Peroxidase superfamily protein | 461.842 | 2.38604 |
| AT2G46860 | | AtPPa3, PPa3, pyrophosphorylase 3 | 111.429 | 0.593574 |
| AT3G47040 | | Glycosyl hydrolase family protein | 30.0456 | 0.168941 |
| AT4G01110 | | unknown protein | 62.5961 | 0.387963 |
| AT1G08990 | | PGSIP5, plant glycogenin-like starch initiation protein 5 | 62.0058 | 0.388489 |
| AT5G51270 | | U-box domain-containing protein kinase family protein | 31.5351 | 0.198535 |
| AT5G58010 | | LRL3, LJRHL1-like 3 | 273.512 | 1.80494 |
| AT3G49960 | | Peroxidase superfamily protein | 159.373 | 1.06339 |
| AT4G34580 | | COW1, SRH1, Sec14p-like phosphatidylinositol transfer family protein | 218.104 | 1.47609 |
| AT3G51350 | | Eukaryotic aspartyl protease family protein | 38.8901 | 0.274518 |
| AT4G38390 | | RHS17, root hair specific 17 | 36.6331 | 0.260206 |
| AT1G70460 | | RHS10, root hair specific 10 | 88.1011 | 0.641957 |
| AT5G62310 | | IRE, AGC (cAMP-dependent, cGMP-dependent and protein kinase C) kinase family protein | 64.0072 | 0.516494 |
| AT4G29180 | | RHS16, root hair specific 16 | 55.3506 | 0.448309 |
| AT5G17820 | | Peroxidase superfamily protein | 1305.01 | 10.998 |
| AT1G27740 | | RSL4, root hair defective 6-like 4 | 254.49 | 2.16234 |
| AT4G30320 | | CAP (Cysteine-rich secretory proteins, Antigen 5, and Pathogenesis-related 1 protein) superfamily protein | 89.776 | 0.843779 |
| AT5G49870 | | Mannose-binding lectin superfamily protein | 31.8792 | 0.309214 |
| AT1G51860 | | Leucine-rich repeat protein kinase family protein | 8.1714 | 0.0812391 |
| AT5G65160 | | tetratricopeptide repeat (TPR)-containing protein | 74.2567 | 0.744421 |
| AT5G01280 | | BEST Arabidopsis thaliana protein match is: proline-rich family protein (TAIR:AT3G09000.1); Has 1807 Blast hits to 1807 proteins in 277 species: Archae - 0; Bacteria - 0; Metazoa - 736; Fungi - 347; Plants - 385; Viruses - 0; Other Eukaryotes - 339 (source: NCBI BLink). | 52.2871 | 0.534523 |
| AT5G61550 | | U-box domain-containing protein kinase family protein | 35.9846 | 0.372456 |
| AT2G45750 | | S-adenosyl-L-methionine-dependent methyltransferases superfamily protein | 142.026 | 1.61829 |
| AT4G25090 | | Riboflavin synthase-like superfamily protein | 62.3366 | 0.714573 |
| AT1G07795 | | unknown protein | 47.4163 | 0.554259 |
| AT1G34760 | | GF14 OMICRON, GRF11, RHS5, general regulatory factor 11 | 50.7197 | 0.636707 |
| AT1G12550 | | D-isomer specific 2-hydroxyacid dehydrogenase family protein | 81.9584 | 1.03544 |
| AT5G65090 | | BST1, DER4, MRH3, DNAse I-like superfamily protein | 41.273 | 0.593343 |
| AT4G25110 | | AtMC2, MC2, metacaspase 2 | 25.4734 | 0.366441 |
| AT3G18450 | | PLAC8 family protein | 26.2681 | 0.378257 |
| AT1G01750 | | ADF11, actin depolymerizing factor 11 | 723.49 | 10.7564 |
| AT3G21340 | | Leucine-rich repeat protein kinase family protein | 83.7404 | 1.26439 |
| AT3G54870 | | ARK1, CAE1, MRH2, Armadillo/beta-catenin repeat family protein / kinesin motor family protein | 48.081 | 0.745398 |
| AT2G38500 | | 2-oxoglutarate (2OG) and Fe(II)-dependent oxygenase superfamily protein | 91.0314 | 1.52791 |
| AT3G07900 | | O-fucosyltransferase family protein | 32.1397 | 0.546227 |
| AT5G15600 | | SP1L4, SPIRAL1-like4 | 148.905 | 2.58613 |
| AT1G53680 | | ATGSTU28, GSTU28, glutathione S-transferase TAU 28 | 304.142 | 5.34615 |
| AT5G42785 | | unknown protein | 80.0565 | 1.42305 |
| AT5G12050 | | unknown protein | 184.136 | 3.34175 |
| AT4G22217 | | Arabidopsis defensin-like protein | 349.124 | 6.59131 |
| AT1G18420 | | Aluminium activated malate transporter family protein | 55.8597 | 1.08416 |
| AT1G04280 | | P-loop containing nucleoside triphosphate hydrolases superfamily protein | 192.645 | 3.74287 |
| AT5G14330 | | unknown protein | 469.906 | 9.38705 |
| AT1G73860 | | P-loop containing nucleoside triphosphate hydrolases superfamily protein | 70.0785 | 1.40914 |
| AT4G30670 | | Putative membrane lipoprotein | 235.479 | 4.89032 |
| AT2G05160 | | CCCH-type zinc fingerfamily protein with RNA-binding domain | 29.1287 | 0.614505 |
| AT4G25160 | | U-box domain-containing protein kinase family protein | 19.6735 | 0.418077 |
| AT1G70170 | | MMP, matrix metalloproteinase | 44.6145 | 0.961701 |
| AT4G37390 | | AUR3, BRU6, GH3-2, GH3.2, YDK1, Auxin-responsive GH3 family protein | 172.489 | 3.74332 |
| AT4G29800 | | PLA IVD, PLP8, PATATIN-like protein 8 | 28.7895 | 0.638612 |
| AT4G18640 | | MRH1, Leucine-rich repeat protein kinase family protein | 85.9666 | 1.95314 |
| AT5G07080 | | HXXXD-type acyl-transferase family protein | 106.986 | 2.56867 |
| AT2G37670 | | Transducin/WD40 repeat-like superfamily protein | 40.673 | 0.994725 |
| AT5G65100 | | Ethylene insensitive 3 family protein | 26.583 | 0.65571 |
| AT2G34910 | | BEST Arabidopsis thaliana protein match is: root hair specific 4 (TAIR:AT1G30850.1) | 587.714 | 14.9774 |
| AT1G48640 | | Transmembrane amino acid transporter family protein | 42.8959 | 1.15054 |
| AT5G17390 | | Adenine nucleotide alpha hydrolases-like superfamily protein | 59.2781 | 1.62984 |
| AT1G29020 | | Calcium-binding EF-hand family protein | 52.5806 | 1.50385 |
| AT4G13440 | | Calcium-binding EF-hand family protein | 106.024 | 3.03328 |
| AT4G16920 | | Disease resistance protein (TIR-NBS-LRR class) family | 2.87594 | 0.0840651 |
| AT3G51540 | | unknown protein | 140.796 | 4.18419 |
| AT3G23190 | | HR-like lesion-inducing protein-related | 366.307 | 10.9146 |
| AT2G25240 | | Serine protease inhibitor (SERPIN) family protein | 52.9154 | 1.59392 |
| AT1G21360 | | GLTP2, glycolipid transfer protein 2 | 43.6204 | 1.34333 |
| AT5G24310 | | ABIL3, ABL interactor-like protein 3 | 101.958 | 3.18457 |
| AT1G23720 | | Proline-rich extensin-like family protein | 1454.68 | 45.5663 |
| AT1G63930 | | ROH1, from the Czech 'roh' meaning 'corner' | 43.3653 | 1.36232 |
| AT3G13782 | | NAP1;4, NFA04, NFA4, nucleosome assembly protein1;4 | 209.37 | 6.5882 |
| AT1G66570 | | ATSUC7, SUC7, sucrose-proton symporter 7 | 31.2731 | 1.0048 |
| AT3G05155 | | Major facilitator superfamily protein | 62.3995 | 2.00907 |
| AT1G19230 | | Riboflavin synthase-like superfamily protein | 51.3866 | 1.65536 |
| AT4G22666 | | Bifunctional inhibitor/lipid-transfer protein/seed storage 2S albumin superfamily protein | 270.642 | 8.73029 |
| AT1G57560 | | AtMYB50, MYB50, myb domain protein 50 | 72.101 | 2.33254 |
| AT3G21180 | | ACA9, ATACA9, autoinhibited Ca(2+)-ATPase 9 | 115.613 | 3.74594 |
| AT3G48940 | | Remorin family protein | 49.2343 | 1.64026 |
| AT1G04700 | | PB1 domain-containing protein tyrosine kinase | 49.9354 | 1.67292 |
| AT5G62280 | | Protein of unknown function (DUF1442) | 105.288 | 3.58599 |
| AT3G43960 | | Cysteine proteinases superfamily protein | 200.497 | 6.84545 |
| AT3G05800 | | AIF1, AtBS1(activation-tagged BRI1 suppressor 1)-interacting factor 1 | 37.6626 | 1.29728 |
| AT4G19680 | | ATIRT2, IRT2, iron regulated transporter 2 | 109.85 | 3.84865 |
| AT5G44480 | | DUR, NAD(P)-binding Rossmann-fold superfamily protein | 74.7474 | 2.64666 |
| AT3G28550 | | Proline-rich extensin-like family protein | 1748.86 | 62.0652 |
| AT5G63600 | | ATFLS5, FLS5, flavonol synthase 5 | 82.4453 | 2.92904 |
| AT2G19060 | | SGNH hydrolase-type esterase superfamily protein | 28.6291 | 1.04724 |
| AT4G24580 | | REN1, Rho GTPase activation protein (RhoGAP) with PH domain | 70.9671 | 2.63631 |
| AT3G16390 | | NSP3, nitrile specifier protein 3 | 353.889 | 13.2526 |
| AT4G32950 | | Protein phosphatase 2C family protein | 241.781 | 9.18321 |
| AT5G25880 | | ATNADP-ME3, NADP-ME3, NADP-malic enzyme 3 | 57.118 | 2.17689 |
| AT4G27290 | | S-locus lectin protein kinase family protein | 39.0958 | 1.50421 |
| AT5G07450 | | CYCP4;3, cyclin p4;3 | 64.7111 | 2.49592 |
| AT4G07960 | | ATCSLC12, CSLC12, CSLC12, Cellulose-synthase-like C12 | 87.0289 | 3.41639 |
| AT4G40010 | | SNRK2-7, SNRK2.7, SRK2F, SNF1-related protein kinase 2.7 | 32.4091 | 1.28034 |
| AT4G18430 | | AtRABA1e, RABA1e, RAB GTPase homolog A1E | 111.568 | 4.4472 |
| AT4G30460 | | glycine-rich protein | 49.893 | 2.00971 |
| AT1G66470 | | RHD6, ROOT HAIR DEFECTIVE6 | 161.46 | 6.58115 |
| AT5G11070 | | unknown protein | 294.83 | 12.2172 |
| AT4G37070 | | AtPLAIVA, PLA IVA, PLP1, Acyl transferase/acyl hydrolase/lysophospholipase superfamily protein | 64.281 | 2.6652 |
| AT2G29750 | | UGT71C1, UDP-glucosyl transferase 71C1 | 96.4608 | 4.05632 |
| AT1G33090 | | MATE efflux family protein | 20.4393 | 0.862959 |
| AT5G61260 | | Plant calmodulin-binding protein-related | 30.5146 | 1.31106 |
| AT2G24840 | | AGL61, DIA, AGAMOUS-like 61 | 62.0978 | 2.70141 |
| AT4G20110 | | BP80-3;1, VSR3;1, VSR7, VACUOLAR SORTING RECEPTOR 7 | 35.0237 | 1.52904 |
| AT1G62320 | | ERD (early-responsive to dehydration stress) family protein | 53.8165 | 2.38029 |
| AT1G74830 | | Protein of unknown function, DUF593 | 41.5366 | 1.84494 |
| AT5G06800 | | myb-like HTH transcriptional regulator family protein | 104.978 | 4.74244 |
| AT1G65610 | | ATGH9A2, KOR2, Six-hairpin glycosidases superfamily protein | 165.887 | 7.60339 |
| AT2G45220 | | Plant invertase/pectin methylesterase inhibitor superfamily | 184.446 | 8.61363 |
| AT2G21880 | | ATRAB7A, ATRABG2, RAB7A, RAB GTPase homolog 7A | 84.6027 | 3.98521 |
| AT5G09440 | | EXL4, EXORDIUM like 4 | 89.7397 | 4.259 |
| AT2G01540 | | Calcium-dependent lipid-binding (CaLB domain) family protein | 474.404 | 22.5354 |
| AT3G06370 | | ATNHX4, NHX4, sodium hydrogen exchanger 4 | 19.9797 | 0.952007 |
| AT2G29740 | | UGT71C2, UDP-glucosyl transferase 71C2 | 23.2408 | 1.13071 |
| AT2G34500 | | CYP710A1, cytochrome P450, family 710, subfamily A, polypeptide 1 | 76.233 | 3.75831 |
| AT1G24320 | | Six-hairpin glycosidases superfamily protein | 40.1752 | 2.04551 |
| AT2G34180 | | ATWL2, CIPK13, SnRK3.7, WL2, CBL-interacting protein kinase 13 | 65.395 | 3.33008 |
| AT5G41280 | | Receptor-like protein kinase-related family protein | 69.5715 | 3.55533 |
| AT5G13150 | | ATEXO70C1, EXO70C1, exocyst subunit exo70 family protein C1 | 52.697 | 2.7557 |
| AT3G61560 | | Reticulon family protein | 226.411 | 11.9469 |
| AT4G17340 | | DELTA-TIP2, TIP2;2, tonoplast intrinsic protein 2;2 | 81.8083 | 4.46908 |
| AT2G38790 | | unknown protein | 89.2405 | 5.07359 |
| AT3G51330 | | Eukaryotic aspartyl protease family protein | 119.334 | 7.01409 |
| AT2G17830 | | F-box and associated interaction domains-containing protein | 59.5139 | 3.51293 |
| AT2G35730 | | Heavy metal transport/detoxification superfamily protein | 64.8507 | 3.94136 |
| AT4G35200 | | Arabidopsis protein of unknown function (DUF241) | 29.5242 | 1.8513 |
| AT5G12880 | | proline-rich family protein | 160.477 | 10.1072 |
| AT2G15980 | | Tetratricopeptide repeat (TPR)-like superfamily protein | 39.4528 | 2.4858 |
| AT5G13990 | | ATEXO70C2, EXO70C2, exocyst subunit exo70 family protein C2 | 114.103 | 7.30302 |
| AT4G22214 | | Defensin-like (DEFL) family protein | 927.985 | 59.3972 |
| AT3G53820 | | C2H2 and C2HC zinc fingers superfamily protein | 231.311 | 14.896 |
| AT3G14850 | | TBL41, TRICHOME BIREFRINGENCE-LIKE 41 | 43.7417 | 2.83187 |
| AT5G40730 | | AGP24, ATAGP24, arabinogalactan protein 24 | 2027.53 | 131.904 |
| AT4G00460 | | ATROPGEF3, ROPGEF3, RHO guanyl-nucleotide exchange factor 3 | 116.672 | 7.66071 |
| AT4G25070 | | unknown protein | 77.9769 | 5.12659 |
| AT4G16350 | | CBL6, SCABP2, calcineurin B-like protein 6 | 34.8977 | 2.29663 |
| AT1G05320 | | FUNCTIONS IN: molecular_function unknown; INVOLVED IN: biological_process unknown; LOCATED IN: cellular_component unknown; EXPRESSED IN: fruit, egg cell; CONTAINS InterPro DOMAIN/s: Prefoldin (InterPro:IPR009053); BEST Arabidopsis thaliana protein match is: unknown protein (TAIR:AT2G32240.1); Has 267650 Blast hits to 119772 proteins in 3899 species: Archae - 3706; Bacteria - 62589; Metazoa - 106546; Fungi - 19914; Plants - 13918; Viruses - 1287; Other Eukaryotes - 59690 (source: NCBI BLink). | 69.93 | 4.65597 |
| AT1G33800 | | Protein of unknown function (DUF579) | 216.503 | 14.4211 |
| AT4G23550 | | ATWRKY29, WRKY29, WRKY family transcription factor | 24.711 | 1.67022 |
| AT5G01610 | | Protein of unknown function, DUF538 | 180.271 | 12.2245 |
| AT1G30900 | | BP80-3;3, VSR3;3, VSR6, VACUOLAR SORTING RECEPTOR 6 | 175.769 | 11.9986 |
| AT3G62100 | | IAA30, indole-3-acetic acid inducible 30 | 139.472 | 9.59808 |
| AT1G14960 | | Polyketide cyclase/dehydrase and lipid transport superfamily protein | 122.489 | 8.48455 |
| AT5G67620 | | unknown protein | 116.588 | 8.13089 |
| AT5G10410 | | ENTH/ANTH/VHS superfamily protein | 91.9798 | 6.41601 |
| AT3G05920 | | Heavy metal transport/detoxification superfamily protein | 169.788 | 11.9305 |
| AT1G56550 | | RXGT1, RhamnoGalacturonan speci&#64257;c Xylosyltransferase 1 | 70.9225 | 4.9953 |
| AT3G04010 | | O-Glycosyl hydrolases family 17 protein | 428.945 | 30.225 |
| AT3G04070 | | anac047, NAC047, NAC domain containing protein 47 | 65.2786 | 4.64433 |
| AT4G20730 | | transposable element gene | 5.51128 | 0.397336 |
| AT3G23175 | | HR-like lesion-inducing protein-related | 297.022 | 21.5645 |
| AT2G20670 | | Protein of unknown function (DUF506) | 198.928 | 14.4747 |
| AT5G44610 | | MAP18, PCAP2, microtubule-associated protein 18 | 327.958 | 24.1866 |
| AT4G22758 | | unknown protein | 64.7652 | 4.80327 |
| AT2G17660 | | RPM1-interacting protein 4 (RIN4) family protein | 761.31 | 57.4593 |
| AT5G57070 | | hydroxyproline-rich glycoprotein family protein | 49.8687 | 3.78625 |
| AT3G15540 | | IAA19, MSG2, indole-3-acetic acid inducible 19 | 288.02 | 22.3099 |
| AT1G56010 | | anac021, ANAC022, NAC1, NAC domain containing protein 1 | 41.566 | 3.30191 |
| AT3G54040 | | PAR1 protein | 259.244 | 20.6245 |
| AT1G15040 | | Class I glutamine amidotransferase-like superfamily protein | 340.555 | 27.5151 |
| AT1G05810 | | ARA, ARA-1, ATRAB11D, ATRABA5E, RABA5E, RAB GTPase homolog A5E | 153.349 | 12.397 |
| AT5G07770 | | Actin-binding FH2 protein | 16.0326 | 1.31705 |
| AT2G18450 | | SDH1-2, succinate dehydrogenase 1-2 | 110.514 | 9.11401 |
| AT5G22570 | | ATWRKY38, WRKY38, WRKY DNA-binding protein 38 | 42.3492 | 3.49925 |
| AT1G53830 | | ATPME2, PME2, pectin methylesterase 2 | 221.151 | 18.3097 |
| AT5G46040 | | Major facilitator superfamily protein | 25.1274 | 2.08116 |
| AT2G30930 | | unknown protein | 360.634 | 30.0296 |
| AT1G78100 | | F-box family protein | 786.804 | 66.8369 |
| AT2G34940 | | BP80-3;2, VSR3;2, VSR5, VACUOLAR SORTING RECEPTOR 5 | 52.7697 | 4.5124 |
| AT5G22920 | | CHY-type/CTCHY-type/RING-type Zinc finger protein | 153.729 | 13.2964 |
| AT5G47450 | | ATTIP2;3, DELTA-TIP3, TIP2;3, tonoplast intrinsic protein 2;3 | 632.373 | 55.342 |
| AT3G56000 | | ATCSLA14, CSLA14, cellulose synthase like A14 | 57.0746 | 5.07221 |
| AT5G24140 | | SQP2, squalene monooxygenase 2 | 108.444 | 9.65024 |
| AT3G12500 | | ATHCHIB, B-CHI, CHI-B, HCHIB, PR-3, PR3, basic chitinase | 54.134 | 5.00602 |
| AT2G31350 | | GLX2-5, glyoxalase 2-5 | 163.768 | 15.1616 |
| AT3G56930 | | DHHC-type zinc finger family protein | 146.087 | 13.7746 |
| AT2G39900 | | GATA type zinc finger transcription factor family protein | 311.315 | 29.5 |
| AT3G07940 | | Calcium-dependent ARF-type GTPase activating protein family | 48.1482 | 4.57929 |
| AT5G24170 | | Got1/Sft2-like vescicle transport protein family | 68.4319 | 6.62262 |
| AT4G27260 | | GH3.5, WES1, Auxin-responsive GH3 family protein | 168.659 | 16.4149 |
| AT5G15180 | | Peroxidase superfamily protein | 64.0184 | 6.26012 |
| AT4G02390 | | APP, ATPARP1, PARP1, PP, poly(ADP-ribose) polymerase | 37.8809 | 3.78638 |
| AT1G22530 | | PATL2, PATELLIN 2 | 186.164 | 19.0348 |
| AT4G27350 | | Protein of unknown function (DUF1223) | 244.81 | 25.1782 |
| AT4G12520 | | Bifunctional inhibitor/lipid-transfer protein/seed storage 2S albumin superfamily protein | 37.1342 | 3.83656 |
| AT5G53250 | | AGP22, ATAGP22, arabinogalactan protein 22 | 220.897 | 22.9246 |
| AT3G45060 | | ATNRT2.6, NRT2.6, high affinity nitrate transporter 2.6 | 573.969 | 59.6096 |
| AT3G21710 | | unknown protein | 190.137 | 19.9234 |
| AT5G43350 | | ATPT1, PHT1;1, phosphate transporter 1;1 | 34.3803 | 3.62091 |
| AT5G40510 | | Sucrase/ferredoxin-like family protein | 242.636 | 25.6875 |
| AT5G64100 | | Peroxidase superfamily protein | 727.186 | 77.0111 |
| AT3G05170 | | Phosphoglycerate mutase family protein | 90.1163 | 9.58352 |
| AT5G45580 | | Homeodomain-like superfamily protein | 134.741 | 14.3338 |
| AT2G36830 | | GAMMA-TIP, GAMMA-TIP1, TIP1;1, gamma tonoplast intrinsic protein | 974.405 | 103.689 |
| AT5G26660 | | ATMYB86, MYB86, myb domain protein 86 | 54.9783 | 5.93874 |
| AT5G01050 | | Laccase/Diphenol oxidase family protein | 25.7688 | 2.79978 |
| AT1G52750 | | alpha/beta-Hydrolases superfamily protein | 130.458 | 14.2276 |
| AT3G22830 | | AT-HSFA6B, HSFA6B, heat shock transcription factor A6B | 338.883 | 37.2627 |
| AT2G27660 | | Cysteine/Histidine-rich C1 domain family protein | 41.893 | 4.62137 |
| AT5G52020 | | Integrase-type DNA-binding superfamily protein | 91.8093 | 10.1282 |
| AT1G01730 | | unknown protein | 159.28 | 17.9865 |
| AT3G54950 | | PLA IIIA, PLP7, patatin-like protein 6 | 48.5347 | 5.48234 |
| AT1G71530 | | Protein kinase superfamily protein | 83.882 | 9.47798 |
| AT5G54490 | | PBP1, pinoid-binding protein 1 | 287.933 | 32.7306 |
| AT1G52240 | | ATROPGEF11, PIRF1, ROPGEF11, RHO guanyl-nucleotide exchange factor 11 | 222.34 | 25.3168 |
| AT3G05490 | | RALFL22, ralf-like 22 | 471.929 | 54.1202 |
| AT1G03850 | | Glutaredoxin family protein | 448.238 | 51.9894 |
| AT1G13950 | | ATELF5A-1, EIF-5A, EIF5A, ELF5A-1, eukaryotic elongation factor 5A-1 | 107.303 | 12.4879 |
| AT1G15580 | | ATAUX2-27, AUX2-27, IAA5, indole-3-acetic acid inducible 5 | 86.7121 | 10.1769 |
| AT3G26520 | | GAMMA-TIP2, SITIP, TIP1;2, TIP2, tonoplast intrinsic protein 2 | 1343.92 | 157.822 |
| AT5G49900 | | Beta-glucosidase, GBA2 type family protein | 67.753 | 8.07035 |
| AT4G01480 | | AtPPa5, PPa5, pyrophosphorylase 5 | 1274.5 | 153.007 |
| AT1G80440 | | Galactose oxidase/kelch repeat superfamily protein | 125.421 | 15.0842 |
| AT5G11110 | | ATSPS2F, KNS2, SPS1, SPS2F, sucrose phosphate synthase 2F | 412.702 | 50.0113 |
| AT2G43820 | | ATSAGT1, GT, SAGT1, SGT1, UGT74F2, UDP-glucosyltransferase 74F2 | 633.66 | 77.0631 |
| AT2G47650 | | UXS4, UDP-xylose synthase 4 | 751.056 | 92.2069 |
| AT1G12950 | | RSH2, root hair specific 2 | 383.267 | 47.3697 |
| AT3G12700 | | Eukaryotic aspartyl protease family protein | 75.0427 | 9.33747 |
| AT2G32270 | | ZIP3, zinc transporter 3 precursor | 165.322 | 20.7386 |
| AT3G07880 | | SCN1, Immunoglobulin E-set superfamily protein | 469.668 | 58.949 |
| AT2G20880 | | Integrase-type DNA-binding superfamily protein | 165.331 | 20.7599 |
| AT3G53150 | | UGT73D1, UDP-glucosyl transferase 73D1 | 47.678 | 6.00347 |
| AT2G04170 | | TRAF-like family protein | 168.974 | 21.5401 |
| AT2G44670 | | Protein of unknown function (DUF581) | 441.449 | 56.6394 |
| AT1G01120 | | KCS1, 3-ketoacyl-CoA synthase 1 | 82.8902 | 10.6765 |
| AT5G56870 | | BGAL4, beta-galactosidase 4 | 59.9873 | 7.7497 |
| AT5G18670 | | BAM9, BMY3, beta-amylase 3 | 274.994 | 35.5265 |
| AT2G30395 | | ATOFP17, OFP17, ovate family protein 17 | 42.9151 | 5.56788 |
| AT3G60550 | | CYCP3;2, cyclin p3;2 | 107.833 | 14.0374 |
| AT3G07000 | | Cysteine/Histidine-rich C1 domain family protein | 15.1634 | 1.97575 |
| AT3G19030 | | unknown protein | 235.993 | 30.9055 |
| AT4G31450 | | RING/U-box superfamily protein | 95.7907 | 12.5648 |
| AT5G20820 | | SAUR-like auxin-responsive protein family | 56.7986 | 7.52724 |
| AT2G26290 | | ARSK1, root-specific kinase 1 | 119.218 | 15.8924 |
| AT5G26010 | | Protein phosphatase 2C family protein | 60.2823 | 8.08689 |
| AT2G32510 | | MAPKKK17, mitogen-activated protein kinase kinase kinase 17 | 67.5074 | 9.13729 |
| AT3G56240 | | CCH, copper chaperone | 224.175 | 30.4103 |
| AT2G24260 | | LRL1, LJRHL1-like 1 | 64.1733 | 8.81101 |
| AT4G13615 | | Uncharacterised protein family SERF | 772.4 | 107.044 |
| AT1G44090 | | ATGA20OX5, GA20OX5, gibberellin 20-oxidase 5 | 90.5283 | 12.5487 |
| AT5G67520 | | APK4, adenosine-5'-phosphosulfate (APS) kinase 4 | 107.667 | 14.9742 |
| AT3G01520 | | Adenine nucleotide alpha hydrolases-like superfamily protein | 191.549 | 26.7047 |
| AT2G42350 | | RING/U-box superfamily protein | 59.7442 | 8.36996 |
| AT5G60800 | | Heavy metal transport/detoxification superfamily protein | 97.0321 | 13.6142 |
| AT5G45500 | | RNI-like superfamily protein | 110.658 | 15.6216 |
| AT5G38940 | | RmlC-like cupins superfamily protein | 46.6574 | 6.59934 |
| AT3G25930 | | Adenine nucleotide alpha hydrolases-like superfamily protein | 54.343 | 7.70837 |
| AT5G01100 | | O-fucosyltransferase family protein | 174.355 | 24.9128 |
| AT5G55960 | | unknown protein | 75.9385 | 10.8671 |
| AT5G54510 | | DFL1, GH3.6, Auxin-responsive GH3 family protein | 101.655 | 14.5543 |
| AT1G10200 | | WLIM1, GATA type zinc finger transcription factor family protein | 120.914 | 17.3714 |
| AT1G02400 | | ATGA2OX4, ATGA2OX6, DTA1, GA2OX6, gibberellin 2-oxidase 6 | 92.8745 | 13.5141 |
| AT1G28330 | | DRM1, DYL1, dormancy-associated protein-like 1 | 348.14 | 50.7583 |
| AT5G43620 | | Pre-mRNA cleavage complex II | 128.958 | 18.8136 |
| AT1G72800 | | RNA-binding (RRM/RBD/RNP motifs) family protein | 52.1081 | 7.61144 |
| AT5G66040 | | STR16, sulfurtransferase protein 16 | 39.9519 | 5.92621 |
| AT2G44450 | | BGLU15, beta glucosidase 15 | 288.944 | 42.9701 |
| AT2G44380 | | Cysteine/Histidine-rich C1 domain family protein | 18.7913 | 2.79727 |
| AT3G53600 | | C2H2-type zinc finger family protein | 819.368 | 122.918 |
| AT5G07220 | | ATBAG3, BAG3, BCL-2-associated athanogene 3 | 263.913 | 39.6005 |
| AT1G01740 | | Protein kinase protein with tetratricopeptide repeat domain | 13.6531 | 2.05532 |
| AT1G23750 | | Nucleic acid-binding, OB-fold-like protein | 161.203 | 24.3002 |
| AT4G06746 | | DEAR5, RAP2.9, related to AP2 9 | 144.094 | 21.8161 |
| AT1G56700 | | Peptidase C15, pyroglutamyl peptidase I-like | 135.873 | 20.6475 |
| AT3G20410 | | CPK9, calmodulin-domain protein kinase 9 | 213.485 | 32.5019 |
| AT3G03520 | | NPC3, non-specific phospholipase C3 | 109.385 | 16.6552 |
| AT5G23220 | | NIC3, nicotinamidase 3 | 203.627 | 31.0288 |
| AT3G23600 | | alpha/beta-Hydrolases superfamily protein | 655.149 | 100.14 |
| AT5G01830 | | ARM repeat superfamily protein | 85.2762 | 13.036 |
| AT1G56660 | | unknown protein | 519.348 | 80.1407 |
| AT4G04700 | | CPK27, calcium-dependent protein kinase 27 | 16.0592 | 2.47984 |
| AT2G02960 | | RING/FYVE/PHD zinc finger superfamily protein | 272.174 | 42.1596 |
| AT1G79270 | | ECT8, evolutionarily conserved C-terminal region 8 | 122.041 | 18.9737 |
| AT5G27930 | | Protein phosphatase 2C family protein | 96.7972 | 15.0631 |
| AT4G37010 | | CEN2, centrin 2 | 122.131 | 19.1228 |
| AT3G16690 | | Nodulin MtN3 family protein | 119.137 | 18.8178 |
| AT1G35670 | | ATCDPK2, ATCPK11, CDPK2, CPK11, calcium-dependent protein kinase 2 | 322.302 | 51.0252 |
| AT3G04630 | | WDL1, WVD2-like 1 | 90.8727 | 14.4064 |
| AT4G27410 | | ANAC072, RD26, NAC (No Apical Meristem) domain transcriptional regulator superfamily protein | 201.019 | 32.0744 |
| AT2G44790 | | UCC2, uclacyanin 2 | 1170.14 | 186.929 |
| AT1G64530 | | Plant regulator RWP-RK family protein | 77.2329 | 12.3854 |
| AT5G65930 | | KCBP, PKCBP, ZWI, kinesin-like calmodulin-binding protein (ZWICHEL) | 88.8466 | 14.3017 |
| AT5G12340 | | unknown protein | 223.717 | 36.0318 |
| AT2G33830 | | Dormancy/auxin associated family protein | 144.465 | 23.3838 |
| AT5G14120 | | Major facilitator superfamily protein | 80.9475 | 13.2065 |
| AT1G18860 | | ATWRKY61, WRKY61, WRKY DNA-binding protein 61 | 71.9443 | 11.7393 |
| AT3G61260 | | Remorin family protein | 418.234 | 68.3455 |
| AT1G66160 | | ATCMPG1, CMPG1, CYS, MET, PRO, and GLY protein 1 | 169.823 | 27.7535 |
| AT4G17100 | | CONTAINS InterPro DOMAIN/s: Endoribonuclease XendoU (InterPro:IPR018998); Has 943 Blast hits to 770 proteins in 162 species: Archae - 0; Bacteria - 61; Metazoa - 472; Fungi - 40; Plants - 78; Viruses - 35; Other Eukaryotes - 257 (source: NCBI BLink). | 252.679 | 41.4201 |
| AT5G18680 | | AtTLP11, TLP11, tubby like protein 11 | 79.2469 | 12.9936 |
| AT5G66580 | | unknown protein | 83.2222 | 13.6914 |
| AT3G19390 | | Granulin repeat cysteine protease family protein | 1148.84 | 190.392 |
| AT2G39518 | | Uncharacterised protein family (UPF0497) | 84.8301 | 14.1797 |
| AT5G13500 | | unknown protein | 217.879 | 36.9633 |
| AT1G61560 | | ATMLO6, MLO6, Seven transmembrane MLO family protein | 111.56 | 19.044 |
| AT5G15950 | | Adenosylmethionine decarboxylase family protein | 195.241 | 34.0288 |
| AT5G21170 | | AKINBETA1, 5'-AMP-activated protein kinase beta-2 subunit protein | 104.002 | 18.1796 |
| AT1G79910 | | Regulator of Vps4 activity in the MVB pathway protein | 29.603 | 5.17834 |
| AT5G43780 | | APS4, Pseudouridine synthase/archaeosine transglycosylase-like family protein | 148.717 | 26.1097 |
| AT1G19200 | | Protein of unknown function (DUF581) | 87.7559 | 15.4307 |
| AT4G29220 | | PFK1, phosphofructokinase 1 | 59.1846 | 10.4152 |
| AT2G17130 | | IDH-II, IDH2, isocitrate dehydrogenase subunit 2 | 211.379 | 37.2411 |
| AT2G30040 | | MAPKKK14, mitogen-activated protein kinase kinase kinase 14 | 125.014 | 22.104 |
| AT1G14170 | | RNA-binding KH domain-containing protein | 181.446 | 32.3096 |
| AT3G18060 | | transducin family protein / WD-40 repeat family protein | 143.205 | 25.5061 |
| AT5G24530 | | DMR6, 2-oxoglutarate (2OG) and Fe(II)-dependent oxygenase superfamily protein | 81.4499 | 14.5107 |
| AT2G19760 | | PFN1, PRF1, profilin 1 | 609.899 | 108.766 |
| AT4G05150 | | Octicosapeptide/Phox/Bem1p family protein | 228.29 | 40.7379 |
| AT5G47230 | | ATERF-5, ATERF5, ERF5, ethylene responsive element binding factor 5 | 136.589 | 24.3925 |
| AT5G44020 | | HAD superfamily, subfamily IIIB acid phosphatase | 1990.44 | 355.83 |
| AT2G26570 | | Plant protein of unknown function (DUF827) | 46.5513 | 8.36438 |
| AT3G60980 | | Tetratricopeptide repeat (TPR)-like superfamily protein | 66.5243 | 11.9634 |
| AT5G06300 | | Putative lysine decarboxylase family protein | 683.151 | 123.097 |
| AT1G17340 | | Phosphoinositide phosphatase family protein | 191.977 | 34.9336 |
| AT4G36880 | | CP1, cysteine proteinase1 | 192.723 | 35.1567 |
| AT3G27300 | | G6PD5, glucose-6-phosphate dehydrogenase 5 | 141.963 | 25.9497 |
| AT3G04880 | | DRT102, DNA-damage-repair/toleration protein (DRT102) | 79.6404 | 14.5778 |
| AT4G26690 | | GPDL2, MRH5, SHV3, PLC-like phosphodiesterase family protein | 215.115 | 40.482 |
| AT1G62660 | | Glycosyl hydrolases family 32 protein | 426.585 | 80.577 |
| AT1G62440 | | LRX2, leucine-rich repeat/extensin 2 | 90.8058 | 17.1865 |
| AT1G58270 | | ZW9, TRAF-like family protein | 251.666 | 47.6801 |
| AT4G33360 | | FLDH, NAD(P)-binding Rossmann-fold superfamily protein | 304.888 | 57.7994 |
| AT2G39570 | | ACT domain-containing protein | 208.337 | 39.6954 |
| AT1G68440 | | unknown protein | 821.724 | 157.151 |
| AT1G30640 | | Protein kinase family protein | 80.037 | 15.3104 |
| AT2G22170 | | Lipase/lipooxygenase, PLAT/LH2 family protein | 444.232 | 85.2754 |
| AT4G15990 | | unknown protein | 79.088 | 15.1879 |
| AT1G72790 | | hydroxyproline-rich glycoprotein family protein | 72.7564 | 13.9791 |
| AT5G25890 | | IAA28, IAR2, indole-3-acetic acid inducible 28 | 174.637 | 33.5893 |
| AT5G17460 | | unknown protein | 280.23 | 54.1676 |
| AT1G80230 | | Rubredoxin-like superfamily protein | 240.118 | 46.4778 |
| AT4G15093 | | catalytic LigB subunit of aromatic ring-opening dioxygenase family | 131.876 | 25.5678 |
| AT3G15630 | | unknown protein | 848.819 | 164.601 |
| AT2G39110 | | Protein kinase superfamily protein | 285.64 | 55.4133 |
| AT5G16910 | | ATCSLD2, CSLD2, cellulose-synthase like D2 | 315.453 | 61.5323 |
| AT4G25570 | | ACYB-2, Cytochrome b561/ferric reductase transmembrane protein family | 540.611 | 106.292 |
| AT3G13310 | | Chaperone DnaJ-domain superfamily protein | 901.908 | 177.623 |
| AT3G15020 | | mMDH2, Lactate/malate dehydrogenase family protein | 125.129 | 24.775 |
| AT2G31020 | | ORP1A, OSBP(oxysterol binding protein)-related protein 1A | 71.1677 | 14.1562 |
| AT5G58730 | | pfkB-like carbohydrate kinase family protein | 190.387 | 38.1269 |
| AT4G37790 | | HAT22, Homeobox-leucine zipper protein family | 100.781 | 20.199 |
| AT5G62920 | | ARR6, response regulator 6 | 130.832 | 26.2483 |
| AT1G06840 | | Leucine-rich repeat protein kinase family protein | 69.5418 | 13.9755 |
| AT4G35985 | | Senescence/dehydration-associated protein-related | 137.36 | 27.7708 |
| AT5G51060 | | ATRBOHC, RBOHC, RHD2, NADPH/respiratory burst oxidase protein D | 217.368 | 44.0457 |
| AT3G05990 | | Leucine-rich repeat (LRR) family protein | 136.921 | 27.9731 |
| AT5G26740 | | Protein of unknown function (DUF300) | 132.408 | 27.153 |
| AT5G05370 | | Cytochrome b-c1 complex, subunit 8 protein | 484.234 | 100.365 |
| AT1G76070 | | unknown protein | 159.589 | 33.0842 |
| AT5G19025 | | Ribosomal protein L34e superfamily protein | 103.039 | 21.3631 |
| AT4G20860 | | FAD-binding Berberine family protein | 223.47 | 46.4183 |
| AT5G13200 | | GRAM domain family protein | 507.346 | 105.427 |
| AT3G56090 | | ATFER3, FER3, ferritin 3 | 537.297 | 112.255 |
| AT2G27820 | | ADT3, PD1, prephenate dehydratase 1 | 111.43 | 23.5146 |
| AT5G35700 | | FIM2, fimbrin-like protein 2 | 104.208 | 22.0692 |
| AT1G15010 | | unknown protein | 758.503 | 161.428 |
| AT5G27150 | | AT-NHX1, ATNHX, ATNHX1, NHX1, Na+/H+ exchanger 1 | 179.064 | 38.1637 |
| AT2G12400 | | unknown protein | 231.808 | 49.4136 |
| AT5G49760 | | Leucine-rich repeat protein kinase family protein | 50.3669 | 10.7752 |
| AT4G19200 | | proline-rich family protein | 1242.91 | 266.046 |
| AT1G12110 | | ATNRT1, B-1, CHL1, CHL1-1, NRT1, NRT1.1, nitrate transporter 1.1 | 95.1444 | 20.3747 |
| AT5G54160 | | ATOMT1, OMT1, O-methyltransferase 1 | 275.654 | 59.0608 |
| AT3G52400 | | ATSYP122, SYP122, syntaxin of plants 122 | 174.986 | 37.5873 |
| AT3G06300 | | AT-P4H-2, P4H isoform 2 | 269.199 | 57.8294 |
| AT2G36220 | | unknown protein | 316.413 | 68.0069 |
| AT4G20840 | | FAD-binding Berberine family protein | 86.0855 | 18.5794 |
| AT1G25400 | | unknown protein | 406.952 | 87.8463 |
| AT1G20310 | | unknown protein | 82.778 | 17.9261 |
| AT5G21280 | | hydroxyproline-rich glycoprotein family protein | 91.1919 | 19.7542 |
| AT1G78070 | | Transducin/WD40 repeat-like superfamily protein | 255.566 | 55.4027 |
| AT4G26970 | | ACO2, aconitase 2 | 501.061 | 109.825 |
| AT5G49890 | | ATCLC-C, CLC-C, chloride channel C | 109.347 | 24.0093 |
| AT5G01720 | | RNI-like superfamily protein | 79.5281 | 17.6356 |
| AT3G25600 | | Calcium-binding EF-hand family protein | 149.32 | 33.1992 |
| AT1G01140 | | CIPK9, PKS6, SnRK3.12, CBL-interacting protein kinase 9 | 130.675 | 29.1015 |
| AT1G53920 | | GLIP5, GDSL-motif lipase 5 | 192.956 | 43.0331 |
| AT1G18150 | | ATMPK8, Protein kinase superfamily protein | 134.08 | 30.2339 |
| AT1G23480 | | ATCSLA03, ATCSLA3, CSLA03, CSLA03, CSLA3, cellulose synthase-like A3 | 77.2241 | 17.4475 |
| AT1G23870 | | ATTPS9, TPS9, TPS9, trehalose-phosphatase/synthase 9 | 356.963 | 80.9865 |
| AT2G18690 | | unknown protein | 158.694 | 36.0347 |
| AT4G13340 | | Leucine-rich repeat (LRR) family protein | 56.7171 | 12.9646 |
| AT5G07470 | | ATMSRA3, PMSR3, peptidemethionine sulfoxide reductase 3 | 322.06 | 74.0045 |
| AT4G37640 | | ACA2, calcium ATPase 2 | 161.434 | 37.1259 |
| AT4G39730 | | Lipase/lipooxygenase, PLAT/LH2 family protein | 479.997 | 110.412 |
| AT3G03990 | | alpha/beta-Hydrolases superfamily protein | 357.218 | 82.981 |
| AT3G21700 | | ATSGP2, SGP2, Ras-related small GTP-binding family protein | 153.294 | 35.7869 |
| AT3G10985 | | ATWI-12, SAG20, WI12, senescence associated gene 20 | 688.286 | 160.942 |
| AT1G54410 | | dehydrin family protein | 1393.94 | 327.969 |
| AT5G38710 | | Methylenetetrahydrofolate reductase family protein | 134.102 | 31.5828 |
| AT4G19230 | | CYP707A1, cytochrome P450, family 707, subfamily A, polypeptide 1 | 163.144 | 38.5162 |
| AT1G10370 | | ATGSTU17, ERD9, GST30, GST30B, Glutathione S-transferase family protein | 224.731 | 53.1103 |
| AT2G23120 | | Late embryogenesis abundant protein, group 6 | 2371.51 | 560.779 |
| AT3G17410 | | Protein kinase superfamily protein | 171.128 | 40.5542 |
| AT1G19910 | | ATVHA-C2, AVA-2PE, AVA-P2, ATPase, F0/V0 complex, subunit C protein | 922.291 | 219.083 |
| AT5G01750 | | Protein of unknown function (DUF567) | 703.089 | 167.121 |
| AT3G49940 | | LBD38, LOB domain-containing protein 38 | 167.855 | 39.9379 |
| AT5G20250 | | DIN10, Raffinose synthase family protein | 446.084 | 106.141 |
| AT4G01070 | | GT72B1, UGT72B1, UDP-Glycosyltransferase superfamily protein | 290.56 | 69.3244 |
| AT5G02350 | | Cysteine/Histidine-rich C1 domain family protein | 64.5379 | 15.4004 |
| AT1G80380 | | P-loop containing nucleoside triphosphate hydrolases superfamily protein | 422.385 | 101.617 |
| AT2G20230 | | Tetraspanin family protein | 143.986 | 34.7292 |
| AT4G12090 | | Cornichon family protein | 124.764 | 30.0976 |
| AT4G14716 | | ARD1, ATARD1, acireductone dioxygenase 1 | 29.0369 | 7.0156 |
| AT1G15100 | | RHA2A, RING-H2 finger A2A | 658.001 | 159.186 |
| AT2G46140 | | Late embryogenesis abundant protein | 747.755 | 180.962 |
| AT3G01290 | | SPFH/Band 7/PHB domain-containing membrane-associated protein family | 354.701 | 86.6315 |
| AT4G26710 | | ATPase, V0 complex, subunit E | 528.377 | 129.273 |
| AT3G04730 | | IAA16, indoleacetic acid-induced protein 16 | 500.06 | 122.369 |
| AT1G63220 | | Calcium-dependent lipid-binding (CaLB domain) family protein | 408.652 | 100.17 |
| AT3G05580 | | Calcineurin-like metallo-phosphoesterase superfamily protein | 195.42 | 47.9499 |
| AT1G09070 | | (AT)SRC2, SRC2, soybean gene regulated by cold-2 | 1357.32 | 334.265 |
| AT1G04040 | | HAD superfamily, subfamily IIIB acid phosphatase | 686.269 | 170.873 |
| AT3G18780 | | ACT2, DER1, ENL2, LSR2, actin 2 | 1579.69 | 395.117 |
| AT3G51460 | | RHD4, Phosphoinositide phosphatase family protein | 210.894 | 52.7531 |
| AT3G17420 | | GPK1, glyoxysomal protein kinase 1 | 115.141 | 28.969 |
| AT3G22850 | | Aluminium induced protein with YGL and LRDR motifs | 288.673 | 72.8211 |
| AT4G39080 | | VHA-A3, vacuolar proton ATPase A3 | 597.978 | 151.661 |
| AT4G33920 | | Protein phosphatase 2C family protein | 274.739 | 69.901 |
| AT4G34720 | | ATVHA-C1, AVA-P1, VHA-C1, ATPase, F0/V0 complex, subunit C protein | 758.112 | 192.907 |
| AT3G02140 | | AFP4, TMAC2, AFP2 (ABI five-binding protein 2) family protein | 269.651 | 68.7033 |
| AT1G56220 | | Dormancy/auxin associated family protein | 141.148 | 36.1249 |
| AT5G13330 | | Rap2.6L, related to AP2 6l | 150.29 | 38.518 |
| AT3G20310 | | ATERF-7, ATERF7, ERF7, ethylene response factor 7 | 84.9658 | 21.787 |
| AT5G11230 | | Nucleotide-sugar transporter family protein | 268.903 | 69.1341 |
| AT1G09740 | | Adenine nucleotide alpha hydrolases-like superfamily protein | 256.845 | 66.0785 |
| AT3G15670 | | Late embryogenesis abundant protein (LEA) family protein | 692.424 | 178.453 |
| AT1G75220 | | Major facilitator superfamily protein | 456.642 | 118.127 |
| AT3G17440 | | ATNPSN13, NPSN13, novel plant snare 13 | 132.224 | 34.2436 |
| AT2G41110 | | ATCAL5, CAM2, calmodulin 2 | 513.368 | 133.265 |
| AT1G10170 | | ATNFXL1, NFXL1, NF-X-like 1 | 537.884 | 140.222 |
| AT5G16370 | | AAE5, acyl activating enzyme 5 | 53.1633 | 13.9336 |
| AT2G41410 | | Calcium-binding EF-hand family protein | 317.713 | 83.4683 |
| AT3G02880 | | Leucine-rich repeat protein kinase family protein | 255.226 | 67.3298 |
| AT1G79340 | | AtMC4, MC4, metacaspase 4 | 462.406 | 122.438 |
| AT4G14500 | | Polyketide cyclase/dehydrase and lipid transport superfamily protein | 184.224 | 48.7814 |
| AT3G62260 | | Protein phosphatase 2C family protein | 216.198 | 57.4319 |
| AT4G27450 | | Aluminium induced protein with YGL and LRDR motifs | 261.962 | 69.8234 |
| AT3G16910 | | AAE7, ACN1, acyl-activating enzyme 7 | 89.2772 | 23.8106 |
| AT3G09810 | | IDH-VI, isocitrate dehydrogenase VI | 242.394 | 64.7445 |
| AT3G07480 | | 2Fe-2S ferredoxin-like superfamily protein | 421.963 | 112.714 |
| AT1G76990 | | ACR3, ACT domain repeat 3 | 194.67 | 52.2885 |
| AT5G18860 | | inosine-uridine preferring nucleoside hydrolase family protein | 101.121 | 27.2386 |
| AT5G47040 | | LON2, lon protease 2 | 203.331 | 54.7876 |
| AT3G60450 | | Phosphoglycerate mutase family protein | 643.847 | 174.274 |
| AT5G67300 | | ATMYB44, ATMYBR1, MYB44, MYBR1, myb domain protein r1 | 195.702 | 53.0075 |
| AT4G12070 | | unknown protein | 73.5546 | 19.9464 |
| AT1G05340 | | unknown protein | 838.556 | 228.011 |
| AT5G42380 | | CML37, CML39, calmodulin like 37 | 244.732 | 66.6011 |
| AT4G23730 | | Galactose mutarotase-like superfamily protein | 114.307 | 31.1309 |
| AT2G46030 | | UBC6, ubiquitin-conjugating enzyme 6 | 178.653 | 48.6747 |
| AT4G37830 | | cytochrome c oxidase-related | 729.118 | 198.929 |
| AT3G53420 | | PIP2, PIP2;1, PIP2A, plasma membrane intrinsic protein 2A | 598 | 164.361 |
| AT1G77120 | | ADH, ADH1, ATADH, ATADH1, alcohol dehydrogenase 1 | 594.086 | 164.008 |
| AT5G06760 | | LEA4-5, Late Embryogenesis Abundant 4-5 | 464.108 | 128.389 |
| AT5G11670 | | ATNADP-ME2, NADP-ME2, NADP-malic enzyme 2 | 867.564 | 240.675 |
| AT2G15970 | | ATCOR413-PM1, cold regulated 413 plasma membrane 1 | 1992.6 | 553.066 |
| AT5G45350 | | proline-rich family protein | 424.782 | 118.27 |
| AT5G58320 | | Kinase interacting (KIP1-like) family protein | 235.739 | 65.6598 |
| AT3G02470 | | SAMDC, S-adenosylmethionine decarboxylase | 1018.14 | 283.691 |
| AT2G36320 | | A20/AN1-like zinc finger family protein | 274.787 | 76.6466 |
| AT4G22610 | | Bifunctional inhibitor/lipid-transfer protein/seed storage 2S albumin superfamily protein | 689.309 | 192.824 |
| AT1G49240 | | ACT8, actin 8 | 1495.81 | 418.454 |
| AT4G27320 | | ATPHOS34, PHOS34, Adenine nucleotide alpha hydrolases-like superfamily protein | 267.712 | 74.9122 |
| AT5G13210 | | Uncharacterised conserved protein UCP015417, vWA | 121.552 | 34.0587 |
| AT5G56150 | | UBC30, ubiquitin-conjugating enzyme 30 | 194.655 | 54.6342 |
| AT5G67480 | | ATBT4, BT4, BTB and TAZ domain protein 4 | 255.167 | 71.6529 |
| AT3G19240 | | Vacuolar import/degradation, Vid27-related protein | 350.111 | 98.3167 |
| AT1G64460 | | Protein kinase superfamily protein | 403.738 | 113.635 |
| AT1G67480 | | Galactose oxidase/kelch repeat superfamily protein | 486.421 | 137.648 |
| AT3G48520 | | CYP94B3, cytochrome P450, family 94, subfamily B, polypeptide 3 | 126.147 | 35.7016 |
| AT3G57330 | | ACA11, autoinhibited Ca2+-ATPase 11 | 132.976 | 37.6509 |
| AT4G03510 | | ATRMA1, RMA1, RING membrane-anchor 1 | 361.587 | 102.716 |
| AT3G10960 | | ATAZG1, AZG1, AZA-guanine resistant1 | 166.839 | 47.4165 |
| AT3G48100 | | ARR5, ATRR2, IBC6, RR5, response regulator 5 | 159.127 | 45.2639 |
| AT3G27240 | | Cytochrome C1 family | 440.435 | 125.396 |
| AT1G24180 | | IAR4, Thiamin diphosphate-binding fold (THDP-binding) superfamily protein | 398.713 | 113.525 |
| AT3G27890 | | NQR, NADPH:quinone oxidoreductase | 151.262 | 43.0954 |
| AT4G22592 | | CPuORF27, conserved peptide upstream open reading frame 27 | 966.459 | 275.933 |
| AT1G15670 | | Galactose oxidase/kelch repeat superfamily protein | 429.508 | 122.931 |
| AT2G41660 | | MIZ1, Protein of unknown function, DUF617 | 270.944 | 77.6576 |
| AT3G58710 | | ATWRKY69, WRKY69, WRKY DNA-binding protein 69 | 118.658 | 34.1444 |
| AT1G47128 | | RD21, RD21A, Granulin repeat cysteine protease family protein | 766.855 | 220.755 |
| AT5G64310 | | AGP1, ATAGP1, arabinogalactan protein 1 | 1739.09 | 501.379 |
| AT2G17840 | | ERD7, Senescence/dehydration-associated protein-related | 677.79 | 195.594 |
| AT5G56760 | | ATSERAT1;1, SAT-52, SAT5, SERAT1;1, serine acetyltransferase 1;1 | 243.944 | 70.4109 |
| AT3G54680 | | proteophosphoglycan-related | 159.124 | 46.0036 |
| AT4G39890 | | AtRABH1c, RABH1c, RAB GTPase homolog H1C | 95.2884 | 27.5567 |
| AT3G03170 | | unknown protein | 203.964 | 59.0553 |
| AT2G39780 | | RNS2, ribonuclease 2 | 322.217 | 93.3188 |
| AT5G05440 | | PYL5, RCAR8, Polyketide cyclase/dehydrase and lipid transport superfamily protein | 96.5699 | 27.9988 |
| AT5G46900 | | Bifunctional inhibitor/lipid-transfer protein/seed storage 2S albumin superfamily protein | 80.0738 | 23.358 |
| AT5G43830 | | Aluminium induced protein with YGL and LRDR motifs | 913.848 | 267.619 |
| AT4G37530 | | Peroxidase superfamily protein | 52.2388 | 15.299 |
| AT4G18070 | | unknown protein | 150.668 | 44.1548 |
| AT3G47340 | | ASN1, AT-ASN1, DIN6, glutamine-dependent asparagine synthase 1 | 622.356 | 182.487 |
| AT3G57410 | | ATVLN3, VLN3, villin 3 | 124.342 | 36.5097 |
| AT4G02620 | | vacuolar ATPase subunit F family protein | 700.66 | 205.858 |
| AT5G19120 | | Eukaryotic aspartyl protease family protein | 273.504 | 80.5682 |
| AT4G38920 | | ATVHA-C3, AVA-P3, VHA-C3, vacuolar-type H(+)-ATPase C3 | 572.495 | 168.82 |
| AT2G43330 | | ATINT1, INT1, inositol transporter 1 | 121.522 | 35.8465 |
| AT4G26910 | | Dihydrolipoamide succinyltransferase | 201.259 | 59.6843 |
| AT2G32150 | | Haloacid dehalogenase-like hydrolase (HAD) superfamily protein | 1407.9 | 417.731 |
| AT5G01800 | | saposin B domain-containing protein | 275.465 | 82.1149 |
| AT1G07750 | | RmlC-like cupins superfamily protein | 317.538 | 94.6886 |
| AT3G59350 | | Protein kinase superfamily protein | 469.079 | 140.433 |
| AT2G31570 | | ATGPX2, GPX2, glutathione peroxidase 2 | 469.491 | 140.861 |
| AT3G21070 | | ATNADK-1, NADK1, NAD kinase 1 | 293.348 | 88.3031 |
| AT4G12080 | | AHL1, ATAHL1, AT-hook motif nuclear-localized protein 1 | 107.577 | 32.389 |
| AT1G14860 | | atnudt18, NUDT18, nudix hydrolase homolog 18 | 201.252 | 60.6038 |
| AT1G76930 | | ATEXT1, ATEXT4, EXT1, EXT4, ORG5, extensin 4 | 221.946 | 66.8919 |
| AT3G10300 | | Calcium-binding EF-hand family protein | 216.4 | 65.7395 |
| AT1G30690 | | Sec14p-like phosphatidylinositol transfer family protein | 161.157 | 48.9575 |
| AT1G22985 | | Integrase-type DNA-binding superfamily protein | 269.88 | 82.3759 |
| AT4G15610 | | Uncharacterised protein family (UPF0497) | 920.066 | 281.058 |
| AT2G17720 | | 2-oxoglutarate (2OG) and Fe(II)-dependent oxygenase superfamily protein | 506.32 | 155.026 |
| AT1G70590 | | F-box family protein | 144.528 | 44.3325 |
| AT2G16660 | | Major facilitator superfamily protein | 478.088 | 146.703 |
| AT4G12000 | | SNARE associated Golgi protein family | 253.892 | 78.3078 |
| AT2G20820 | | unknown protein | 683.525 | 211.331 |
| AT5G19230 | | Glycoprotein membrane precursor GPI-anchored | 986.846 | 305.293 |
| AT4G32150 | | ATVAMP711, VAMP711, vesicle-associated membrane protein 711 | 241.923 | 75.0958 |
| AT4G23470 | | PLAC8 family protein | 223.15 | 69.2986 |
| AT3G55840 | | Hs1pro-1 protein | 206.042 | 64.332 |
| AT5G59550 | | zinc finger (C3HC4-type RING finger) family protein | 103.794 | 32.5617 |
| AT1G76180 | | ERD14, Dehydrin family protein | 4562.78 | 1432.35 |
| AT5G49450 | | AtbZIP1, bZIP1, basic leucine-zipper 1 | 937.389 | 297.292 |
| AT5G21940 | | unknown protein | 1111.76 | 353.043 |
| AT1G13390 | | unknown protein | 267.851 | 85.197 |
| AT5G12140 | | ATCYS1, CYS1, cystatin-1 | 530.192 | 169.894 |
| AT1G45145 | | ATH5, ATTRX5, LIV1, TRX5, thioredoxin H-type 5 | 1928.02 | 630.427 |
| AT1G19020 | | unknown protein | 472.598 | 155.128 |
| AT1G20440 | | AtCOR47, COR47, RD17, cold-regulated 47 | 2699.7 | 886.206 |
| AT3G15356 | | Legume lectin family protein | 262.734 | 86.5204 |
| AT4G26080 | | ABI1, AtABI1, Protein phosphatase 2C family protein | 730.496 | 241.738 |
| AT5G07460 | | ATMSRA2, PMSR2, peptidemethionine sulfoxide reductase 2 | 777.38 | 258.102 |
| AT5G41080 | | PLC-like phosphodiesterases superfamily protein | 349.839 | 116.471 |
| AT3G11780 | | MD-2-related lipid recognition domain-containing protein / ML domain-containing protein | 170.826 | 56.9062 |
| AT5G01410 | | ATPDX1, ATPDX1.3, PDX1, PDX1.3, RSR4, Aldolase-type TIM barrel family protein | 668.851 | 223.114 |
| AT5G59490 | | Haloacid dehalogenase-like hydrolase (HAD) superfamily protein | 496.028 | 166.133 |
| AT5G65690 | | PCK2, PEPCK, phosphoenolpyruvate carboxykinase 2 | 711.095 | 238.883 |
| AT5G04170 | | Calcium-binding EF-hand family protein | 163.288 | 55.2346 |
| AT1G71000 | | Chaperone DnaJ-domain superfamily protein | 277.718 | 95.9805 |
| AT1G73920 | | alpha/beta-Hydrolases superfamily protein | 327.045 | 114.536 |
| AT5G57830 | | Protein of unknown function, DUF593 | 154.744 | 55.1579 |
| AT2G27720 | | 60S acidic ribosomal protein family | 120.372 | 248.44 |
| AT4G17390 | | Ribosomal protein L23/L15e family protein | 72.0515 | 151.029 |
| AT2G29550 | | TUB7, tubulin beta-7 chain | 50.1578 | 105.504 |
| AT3G22440 | | FRIGIDA-like protein | 26.063 | 54.9103 |
| AT5G52650 | | RNA binding Plectin/S10 domain-containing protein | 85.3214 | 179.917 |
| AT3G09920 | | PIP5K9, phosphatidyl inositol monophosphate 5 kinase | 20.0086 | 42.2203 |
| AT5G66920 | | sks17, SKU5 similar 17 | 43.9372 | 93.5 |
| AT1G03230 | | Eukaryotic aspartyl protease family protein | 64.119 | 137.252 |
| AT5G64030 | | S-adenosyl-L-methionine-dependent methyltransferases superfamily protein | 16.853 | 36.0858 |
| AT1G68920 | | basic helix-loop-helix (bHLH) DNA-binding superfamily protein | 17.2288 | 37.0572 |
| AT3G57290 | | ATEIF3E-1, ATINT6, EIF3E, INT-6, INT6, TIF3E1, eukaryotic translation initiation factor 3E | 42.9714 | 92.552 |
| AT4G18280 | | glycine-rich cell wall protein-related | 84.3423 | 182.133 |
| AT5G28840 | | GME, GDP-D-mannose 3',5'-epimerase | 34.1286 | 73.8176 |
| AT2G29400 | | PP1-AT, TOPP1, type one protein phosphatase 1 | 21.8851 | 47.4557 |
| AT2G16060 | | AHB1, ARATH GLB1, ATGLB1, GLB1, HB1, NSHB1, hemoglobin 1 | 76.7655 | 166.921 |
| AT5G47700 | | 60S acidic ribosomal protein family | 56.2019 | 122.598 |
| AT1G78150 | | unknown protein | 49.4532 | 108.304 |
| AT3G09200 | | Ribosomal protein L10 family protein | 166.759 | 365.37 |
| AT3G02080 | | Ribosomal protein S19e family protein | 131.104 | 287.526 |
| AT1G41830 | | SKS6, SKS6, SKU5-similar 6 | 49.4095 | 108.554 |
| AT3G09270 | | ATGSTU8, GSTU8, glutathione S-transferase TAU 8 | 51.6758 | 113.559 |
| AT3G52140 | | tetratricopeptide repeat (TPR)-containing protein | 15.7628 | 34.686 |
| AT3G18740 | | Ribosomal protein L7Ae/L30e/S12e/Gadd45 family protein | 124.524 | 274.163 |
| AT1G15930 | | Ribosomal protein L7Ae/L30e/S12e/Gadd45 family protein | 109.082 | 240.561 |
| AT5G20160 | | Ribosomal protein L7Ae/L30e/S12e/Gadd45 family protein | 70.624 | 155.856 |
| AT1G77330 | | 2-oxoglutarate (2OG) and Fe(II)-dependent oxygenase superfamily protein | 102.362 | 226.015 |
| AT5G27700 | | Ribosomal protein S21e | 129.32 | 285.645 |
| AT4G02840 | | Small nuclear ribonucleoprotein family protein | 43.5676 | 96.2675 |
| AT5G39040 | | ALS1, ATTAP2, TAP2, transporter associated with antigen processing protein 2 | 26.3208 | 58.3968 |
| AT1G19870 | | iqd32, IQ-domain 32 | 24.0832 | 53.5322 |
| AT1G30580 | | GTP binding | 71.29 | 158.499 |
| AT5G65640 | | bHLH093, beta HLH protein 93 | 34.7423 | 77.2895 |
| AT5G42580 | | CYP705A12, cytochrome P450, family 705, subfamily A, polypeptide 12 | 102.872 | 229.455 |
| AT2G41840 | | Ribosomal protein S5 family protein | 152.097 | 339.905 |
| AT3G19100 | | Protein kinase superfamily protein | 22.7923 | 50.9387 |
| AT1G52300 | | Zinc-binding ribosomal protein family protein | 100.59 | 225.16 |
| AT5G03330 | | Cysteine proteinases superfamily protein | 19.3709 | 43.5725 |
| AT1G79650 | | RAD23, RAD23B, Rad23 UV excision repair protein family | 14.2154 | 31.9788 |
| AT3G56340 | | Ribosomal protein S26e family protein | 103.597 | 233.459 |
| AT1G57720 | | Translation elongation factor EF1B, gamma chain | 83.2165 | 188.35 |
| AT3G62250 | | UBQ5, ubiquitin 5 | 142.098 | 322.287 |
| AT1G30120 | | PDH-E1 BETA, pyruvate dehydrogenase E1 beta | 16.7172 | 37.9992 |
| AT1G30880 | | unknown protein | 43.5069 | 99.1328 |
| AT3G02720 | | Class I glutamine amidotransferase-like superfamily protein | 19.1086 | 43.8207 |
| AT4G37870 | | PCK1, PEPCK, phosphoenolpyruvate carboxykinase 1 | 131.217 | 301.599 |
| AT4G02930 | | GTP binding Elongation factor Tu family protein | 29.867 | 68.7868 |
| AT5G03530 | | ATRAB, ATRAB ALPHA, ATRAB18B, ATRABC2A, RABC2A, RAB GTPase homolog C2A | 13.8972 | 32.0075 |
| AT1G10840 | | TIF3H1, translation initiation factor 3 subunit H1 | 31.6854 | 73.0347 |
| AT3G59850 | | Pectin lyase-like superfamily protein | 53.4824 | 123.944 |
| AT4G29390 | | Ribosomal protein S30 family protein | 73.4066 | 170.429 |
| AT1G78560 | | Sodium Bile acid symporter family | 13.9284 | 32.4079 |
| AT5G57090 | | AGR, AGR1, ATPIN2, EIR1, PIN2, WAV6, Auxin efflux carrier family protein | 39.3992 | 91.7715 |
| AT5G62610 | | basic helix-loop-helix (bHLH) DNA-binding superfamily protein | 17.1947 | 40.1723 |
| AT5G65360 | | Histone superfamily protein | 42.5754 | 99.4859 |
| AT5G41520 | | RNA binding Plectin/S10 domain-containing protein | 79.7031 | 187.057 |
| AT1G64550 | | ATGCN3, GCN3, general control non-repressible 3 | 13.0446 | 30.6326 |
| AT3G08030 | | Protein of unknown function, DUF642 | 39.3277 | 92.5896 |
| AT2G27710 | | 60S acidic ribosomal protein family | 44.2225 | 104.141 |
| AT5G36250 | | Protein phosphatase 2C family protein | 17.1784 | 40.5044 |
| AT3G19820 | | CBB1, DIM, DIM1, DWF1, EVE1, cell elongation protein / DWARF1 / DIMINUTO (DIM) | 78.4693 | 186.005 |
| AT3G11710 | | ATKRS-1, lysyl-tRNA synthetase 1 | 23.2087 | 55.0875 |
| AT1G80070 | | EMB14, EMB177, EMB33, SUS2, Pre-mRNA-processing-splicing factor | 16.3458 | 38.9212 |
| AT4G39200 | | Ribosomal protein S25 family protein | 100.183 | 238.594 |
| AT4G17940 | | Tetratricopeptide repeat (TPR)-like superfamily protein | 89.8776 | 214.145 |
| AT1G01090 | | PDH-E1 ALPHA, pyruvate dehydrogenase E1 alpha | 23.1515 | 55.2546 |
| AT3G08690 | | ATUBC11, UBC11, ubiquitin-conjugating enzyme 11 | 22.1332 | 53.0109 |
| AT5G07010 | | ATST2A, ST2A, sulfotransferase 2A | 22.5978 | 54.1621 |
| AT2G29470 | | ATGSTU3, GST21, GSTU3, glutathione S-transferase tau 3 | 69.7378 | 168.027 |
| AT3G22230 | | Ribosomal L27e protein family | 71.7602 | 173.074 |
| AT3G06650 | | ACLB-1, ATP-citrate lyase B-1 | 23.5636 | 56.869 |
| AT4G18730 | | RPL16B, ribosomal protein L16B | 98.6908 | 238.892 |
| AT3G60245 | | Zinc-binding ribosomal protein family protein | 163.292 | 395.318 |
| AT5G63980 | | ALX8, ATSAL1, FRY1, HOS2, RON1, SAL1, Inositol monophosphatase family protein | 18.513 | 45.0875 |
| AT1G16190 | | RAD23A, Rad23 UV excision repair protein family | 15.669 | 38.2864 |
| AT2G37640 | | ATEXP3, ATEXPA3, ATHEXP ALPHA 1.9, EXP3, Barwin-like endoglucanases superfamily protein | 19.8782 | 48.824 |
| AT3G49910 | | Translation protein SH3-like family protein | 118.394 | 291.858 |
| AT4G00710 | | BSK3, BR-signaling kinase 3 | 17.5075 | 43.1594 |
| AT5G03850 | | Nucleic acid-binding, OB-fold-like protein | 183.608 | 453 |
| AT2G41800 | | Protein of unknown function, DUF642 | 110.379 | 273.359 |
| AT1G13560 | | AAPT1, ATAAPT1, aminoalcoholphosphotransferase 1 | 19.4787 | 48.2947 |
| AT3G09500 | | Ribosomal L29 family protein | 160.176 | 397.975 |
| AT1G18080 | | ATARCA, RACK1A, RACK1A_AT, Transducin/WD40 repeat-like superfamily protein | 74.7469 | 186.183 |
| AT3G52870 | | IQ calmodulin-binding motif family protein | 21.305 | 53.0686 |
| AT3G25530 | | ATGHBDH, GHBDH, GLYR1, GR1, glyoxylate reductase 1 | 15.7847 | 39.4516 |
| AT1G69620 | | RPL34, ribosomal protein L34 | 204.899 | 513.707 |
| AT1G26930 | | Galactose oxidase/kelch repeat superfamily protein | 15.2801 | 38.3111 |
| AT2G15830 | | unknown protein | 93.1343 | 233.621 |
| AT5G55190 | | ATRAN3, RAN3, RAN GTPase 3 | 37.3098 | 94.3228 |
| AT1G65730 | | YSL7, YELLOW STRIPE like 7 | 25.4127 | 64.5294 |
| AT3G48870 | | ATCLPC, ATHSP93-III, HSP93-III, Clp ATPase | 15.6384 | 39.7538 |
| AT5G62300 | | Ribosomal protein S10p/S20e family protein | 104.965 | 267.169 |
| AT4G16260 | | Glycosyl hydrolase superfamily protein | 24.5554 | 62.5874 |
| AT1G02780 | | emb2386, Ribosomal protein L19e family protein | 179.329 | 457.563 |
| AT1G74960 | | ATKAS2, FAB1, KAS2, fatty acid biosynthesis 1 | 13.4977 | 34.5203 |
| AT5G35530 | | Ribosomal protein S3 family protein | 68.2488 | 174.725 |
| AT1G01960 | | EDA10, SEC7-like guanine nucleotide exchange family protein | 11.7191 | 30.0203 |
| AT4G00740 | | S-adenosyl-L-methionine-dependent methyltransferases superfamily protein | 12.1252 | 31.0699 |
| AT3G53890 | | Ribosomal protein S21e | 106.962 | 274.759 |
| AT5G54390 | | AHL, ATAHL, HL, HAL2-like | 38.1391 | 98.3844 |
| AT2G33340 | | MAC3B, MOS4-associated complex 3B | 22.1636 | 57.5146 |
| AT5G23820 | | MD-2-related lipid recognition domain-containing protein | 230.346 | 597.935 |
| AT1G12000 | | Phosphofructokinase family protein | 42.2082 | 109.913 |
| AT1G75330 | | OTC, ornithine carbamoyltransferase | 14.3925 | 37.5142 |
| AT5G02260 | | ATEXP9, ATEXPA9, ATHEXP ALPHA 1.10, EXP9, EXPA9, expansin A9 | 215.734 | 562.346 |
| AT1G11580 | | ATPMEPCRA, PMEPCRA, methylesterase PCR A | 40.1105 | 104.645 |
| AT2G29420 | | ATGSTU7, GST25, GSTU7, glutathione S-transferase tau 7 | 220.431 | 575.141 |
| AT4G38740 | | ROC1, rotamase CYP 1 | 36.7551 | 95.9059 |
| AT3G48000 | | ALDH2, ALDH2A, ALDH2B4, aldehyde dehydrogenase 2B4 | 68.1123 | 178.105 |
| AT2G43870 | | Pectin lyase-like superfamily protein | 22.2064 | 58.1351 |
| AT5G19100 | | Eukaryotic aspartyl protease family protein | 68.1949 | 178.547 |
| AT5G10360 | | EMB3010, RPS6B, Ribosomal protein S6e | 86.437 | 226.543 |
| AT5G05730 | | AMT1, ASA1, JDL1, TRP5, WEI2, anthranilate synthase alpha subunit 1 | 34.1044 | 89.5548 |
| AT4G15900 | | PRL1, pleiotropic regulatory locus 1 | 14.6778 | 38.7042 |
| AT1G06090 | | Fatty acid desaturase family protein | 23.4209 | 62.0475 |
| AT1G67430 | | Ribosomal protein L22p/L17e family protein | 202.884 | 541.451 |
| AT3G45010 | | scpl48, serine carboxypeptidase-like 48 | 49.003 | 131.181 |
| AT4G26880 | | Stigma-specific Stig1 family protein | 21.056 | 56.4067 |
| AT1G33590 | | Leucine-rich repeat (LRR) family protein | 64.9448 | 174.174 |
| AT1G23290 | | RPL27A, RPL27AB, Ribosomal protein L18e/L15 superfamily protein | 104.011 | 279.255 |
| AT1G09590 | | Translation protein SH3-like family protein | 62.0026 | 166.559 |
| AT5G19340 | | unknown protein | 26.3961 | 71.0811 |
| AT4G34200 | | EDA9, D-3-phosphoglycerate dehydrogenase | 35.2852 | 95.095 |
| AT4G02230 | | Ribosomal protein L19e family protein | 32.0766 | 86.6097 |
| AT5G63800 | | BGAL6, MUM2, Glycosyl hydrolase family 35 protein | 13.8423 | 37.391 |
| AT5G23900 | | Ribosomal protein L13e family protein | 36.691 | 99.1495 |
| AT2G38040 | | CAC3, acetyl Co-enzyme a carboxylase carboxyltransferase alpha subunit | 23.8833 | 64.6057 |
| AT3G52590 | | EMB2167, ERD16, HAP4, UBQ1, ubiquitin extension protein 1 | 241.465 | 655.215 |
| AT2G30340 | | LBD13, LOB domain-containing protein 13 | 38.1545 | 103.73 |
| AT4G30080 | | ARF16, auxin response factor 16 | 11.8778 | 32.2976 |
| AT1G52690 | | Late embryogenesis abundant protein (LEA) family protein | 111.235 | 302.545 |
| AT1G56045 | | Ribosomal protein L41 family | 186.746 | 510.413 |
| AT4G38130 | | ATHD1, ATHDA19, HD1, HDA1, HDA19, RPD3A, histone deacetylase 1 | 29.8022 | 81.5549 |
| AT5G49510 | | PFD3, prefoldin 3 | 22.7683 | 62.3262 |
| AT1G73230 | | Nascent polypeptide-associated complex NAC | 63.2581 | 173.205 |
| AT1G80560 | | ATIMD2, IMD2, isopropylmalate dehydrogenase 2 | 10.6229 | 29.1191 |
| AT1G04510 | | MAC3A, MOS4-associated complex 3A | 10.4081 | 28.6156 |
| AT5G20290 | | Ribosomal protein S8e family protein | 218.415 | 600.703 |
| AT5G55660 | | DEK domain-containing chromatin associated protein | 13.122 | 36.1259 |
| AT2G21610 | | ATPE11, PE11, pectinesterase 11 | 22.6871 | 62.5818 |
| AT2G36170 | | Ubiquitin supergroup;Ribosomal protein L40e | 39.0439 | 107.766 |
| AT5G52882 | | P-loop containing nucleoside triphosphate hydrolases superfamily protein | 10.9377 | 30.242 |
| AT2G45640 | | ATSAP18, SAP18, SIN3 associated polypeptide P18 | 18.4281 | 50.9979 |
| AT1G70600 | | Ribosomal protein L18e/L15 superfamily protein | 110.991 | 307.179 |
| AT2G24600 | | Ankyrin repeat family protein | 10.09 | 27.9264 |
| AT5G62880 | | ARAC10, ATRAC10, ATROP11, RAC10, RAC-like 10 | 25.2184 | 69.8448 |
| AT4G21960 | | PRXR1, Peroxidase superfamily protein | 36.811 | 102.031 |
| AT1G11000 | | ATMLO4, MLO4, Seven transmembrane MLO family protein | 23.6523 | 65.938 |
| AT1G64980 | | Nucleotide-diphospho-sugar transferases superfamily protein | 19.6373 | 54.7721 |
| AT5G53500 | | Transducin/WD40 repeat-like superfamily protein | 16.6055 | 46.3983 |
| AT1G08780 | | AIP3, PFD4, ABI3-interacting protein 3 | 19.142 | 53.5117 |
| AT1G49410 | | TOM6, translocase of the outer mitochondrial membrane 6 | 21.7843 | 60.946 |
| AT1G07770 | | RPS15A, ribosomal protein S15A | 78.7206 | 220.625 |
| AT3G16440 | | ATMLP-300B, MEE36, MLP-300B, myrosinase-binding protein-like protein-300B | 87.2407 | 245.075 |
| AT4G00100 | | ATRPS13A, PFL2, RPS13, RPS13A, ribosomal protein S13A | 76.2889 | 214.325 |
| AT3G05590 | | RPL18, ribosomal protein L18 | 177.486 | 500.291 |
| AT5G12250 | | TUB6, beta-6 tubulin | 23.2084 | 65.4639 |
| AT5G64140 | | RPS28, ribosomal protein S28 | 101.388 | 286.412 |
| AT1G19850 | | ARF5, IAA24, MP, Transcriptional factor B3 family protein / auxin-responsive factor AUX/IAA-related | 21.7545 | 61.4566 |
| AT1G02930 | | ATGST1, ATGSTF3, ATGSTF6, ERD11, GST1, GSTF6, glutathione S-transferase 6 | 32.8692 | 92.9471 |
| AT1G02560 | | CLPP5, NCLPP1, NCLPP5, nuclear encoded CLP protease 5 | 13.2576 | 37.5611 |
| AT5G52470 | | ATFBR1, ATFIB1, FBR1, FIB1, SKIP7, fibrillarin 1 | 22.7644 | 64.4985 |
| AT1G17880 | | ATBTF3, BTF3, basic transcription factor 3 | 64.444 | 182.702 |
| AT3G13580 | | Ribosomal protein L30/L7 family protein | 24.8403 | 70.5245 |
| AT4G33250 | | ATTIF3K1, EIF3K, TIF3K1, eukaryotic translation initiation factor 3K | 37.2538 | 105.804 |
| AT4G24190 | | AtHsp90-7, AtHsp90.7, HSP90.7, SHD, Chaperone protein htpG family protein | 42.4991 | 121.498 |
| AT2G19730 | | Ribosomal L28e protein family | 149.85 | 428.481 |
| AT2G31610 | | Ribosomal protein S3 family protein | 81.5254 | 233.666 |
| AT2G30860 | | ATGSTF7, ATGSTF9, GLUTTR, GSTF9, glutathione S-transferase PHI 9 | 58.1687 | 166.785 |
| AT2G04390 | | Ribosomal S17 family protein | 19.271 | 55.3886 |
| AT1G32700 | | PLATZ transcription factor family protein | 36.8889 | 106.25 |
| AT4G33865 | | Ribosomal protein S14p/S29e family protein | 108.764 | 313.296 |
| AT4G36130 | | Ribosomal protein L2 family | 45.1305 | 130.075 |
| AT4G25740 | | RNA binding Plectin/S10 domain-containing protein | 23.4433 | 67.5948 |
| AT3G23990 | | HSP60, HSP60-3B, heat shock protein 60 | 36.3393 | 104.925 |
| AT4G16720 | | Ribosomal protein L23/L15e family protein | 81.481 | 235.805 |
| AT2G42570 | | TBL39, TRICHOME BIREFRINGENCE-LIKE 39 | 40.3608 | 116.921 |
| AT5G16390 | | BCCP, BCCP-1, BCCP1, CAC1, CAC1-A, CAC1A, chloroplastic acetylcoenzyme A carboxylase 1 | 11.4652 | 33.2857 |
| AT1G20220 | | Alba DNA/RNA-binding protein | 14.6404 | 42.5307 |
| AT5G02610 | | Ribosomal L29 family protein | 33.9452 | 98.6777 |
| AT5G06865 | | other RNA | 49.6787 | 144.851 |
| AT3G11940 | | AML1, ATRPS5A, RPS5A, ribosomal protein 5A | 105.121 | 306.907 |
| AT1G72370 | | AP40, P40, RP40, RPSAA, 40s ribosomal protein SA | 108.382 | 317.522 |
| AT1G11860 | | Glycine cleavage T-protein family | 19.5886 | 57.3918 |
| AT1G20580 | | Small nuclear ribonucleoprotein family protein | 20.2933 | 59.4659 |
| AT5G35940 | | Mannose-binding lectin superfamily protein | 49.3543 | 144.916 |
| AT1G04270 | | RPS15, cytosolic ribosomal protein S15 | 104.621 | 307.284 |
| AT1G29880 | | glycyl-tRNA synthetase / glycine--tRNA ligase | 10.3317 | 30.4127 |
| AT5G61170 | | Ribosomal protein S19e family protein | 67.3293 | 198.384 |
| AT4G14320 | | Zinc-binding ribosomal protein family protein | 86.7677 | 255.661 |
| AT5G15200 | | Ribosomal protein S4 | 151.746 | 448.284 |
| AT3G20050 | | ATTCP-1, TCP-1, T-complex protein 1 alpha subunit | 28.2907 | 83.6201 |
| AT1G75710 | | C2H2-like zinc finger protein | 7.17571 | 21.2198 |
| AT3G44320 | | AtNIT3, NIT3, nitrilase 3 | 15.3103 | 45.3185 |
| AT3G16430 | | JAL31, jacalin-related lectin 31 | 11.4996 | 34.1336 |
| AT3G09630 | | Ribosomal protein L4/L1 family | 65.7655 | 195.693 |
| AT1G29470 | | S-adenosyl-L-methionine-dependent methyltransferases superfamily protein | 42.5349 | 126.805 |
| AT4G36420 | | Ribosomal protein L12 family protein | 9.49062 | 28.3357 |
| AT1G14810 | | semialdehyde dehydrogenase family protein | 14.5878 | 43.5799 |
| AT3G48930 | | EMB1080, Nucleic acid-binding, OB-fold-like protein | 94.2605 | 282.545 |
| AT1G27390 | | TOM20-2, translocase outer membrane 20-2 | 12.916 | 38.762 |
| AT1G33140 | | PGY2, Ribosomal protein L6 family | 9.08839 | 27.2992 |
| AT5G46430 | | Ribosomal protein L32e | 81.081 | 243.717 |
| AT1G76680 | | ATOPR1, OPR1, 12-oxophytodienoate reductase 1 | 192.188 | 578.059 |
| AT3G23300 | | S-adenosyl-L-methionine-dependent methyltransferases superfamily protein | 8.66649 | 26.1796 |
| AT4G31700 | | RPS6, RPS6A, ribosomal protein S6 | 93.9881 | 285.444 |
| AT1G26880 | | Ribosomal protein L34e superfamily protein | 49.1634 | 149.396 |
| AT4G17720 | | RNA-binding (RRM/RBD/RNP motifs) family protein | 27.9788 | 85.0244 |
| AT2G36620 | | RPL24A, ribosomal protein L24 | 50.0633 | 152.889 |
| AT5G25460 | | Protein of unknown function, DUF642 | 17.7753 | 54.5325 |
| AT5G48760 | | Ribosomal protein L13 family protein | 17.7102 | 54.3328 |
| AT2G38650 | | GAUT7, LGT7, galacturonosyltransferase 7 | 8.59218 | 26.3704 |
| AT5G28060 | | Ribosomal protein S24e family protein | 194.707 | 597.791 |
| AT2G36130 | | Cyclophilin-like peptidyl-prolyl cis-trans isomerase family protein | 9.35365 | 28.807 |
| AT2G31060 | | elongation factor family protein | 8.02585 | 24.726 |
| AT5G11200 | | DEAD/DEAH box RNA helicase family protein | 6.6205 | 20.4477 |
| AT4G28940 | | Phosphorylase superfamily protein | 32.3272 | 100.127 |
| AT4G37410 | | CYP81F4, cytochrome P450, family 81, subfamily F, polypeptide 4 | 10.9341 | 33.907 |
| AT4G14680 | | APS3, Pseudouridine synthase/archaeosine transglycosylase-like family protein | 11.781 | 36.5862 |
| AT2G44180 | | MAP2A, methionine aminopeptidase 2A | 11.7417 | 36.6173 |
| AT3G54740 | | Protein of unknown function, DUF593 | 12.1417 | 37.9831 |
| AT2G47115 | | unknown protein | 28.7087 | 89.9077 |
| AT5G14430 | | S-adenosyl-L-methionine-dependent methyltransferases superfamily protein | 6.55618 | 20.552 |
| AT1G15520 | | ABCG40, ATABCG40, ATPDR12, PDR12, pleiotropic drug resistance 12 | 17.9469 | 56.2606 |
| AT3G17820 | | ATGSKB6, GLN1.3, GLN1;3, glutamine synthetase 1.3 | 47.6736 | 149.648 |
| AT4G14360 | | S-adenosyl-L-methionine-dependent methyltransferases superfamily protein | 9.23933 | 29.011 |
| AT5G39050 | | HXXXD-type acyl-transferase family protein | 15.4103 | 48.4594 |
| AT3G07110 | | Ribosomal protein L13 family protein | 40.8247 | 128.53 |
| AT4G09320 | | NDPK1, Nucleoside diphosphate kinase family protein | 81.6957 | 257.591 |
| AT5G60530 | | late embryogenesis abundant protein-related / LEA protein-related | 97.8294 | 309.051 |
| AT3G12965 | | other RNA | 37.2624 | 117.817 |
| AT3G61240 | | DEA(D/H)-box RNA helicase family protein | 9.63024 | 30.4491 |
| AT3G47370 | | Ribosomal protein S10p/S20e family protein | 42.7813 | 135.434 |
| AT1G54630 | | ACP3, acyl carrier protein 3 | 32.1228 | 101.714 |
| AT3G11630 | | Thioredoxin superfamily protein | 16.426 | 52.0349 |
| AT5G02450 | | Ribosomal protein L36e family protein | 109.926 | 348.769 |
| AT5G07090 | | Ribosomal protein S4 (RPS4A) family protein | 87.3458 | 278.028 |
| AT1G43170 | | ARP1, emb2207, RP1, RPL3A, ribosomal protein 1 | 133.498 | 425.174 |
| AT2G33370 | | Ribosomal protein L14p/L23e family protein | 54.3103 | 173.074 |
| AT2G28000 | | CH-CPN60A, CPN60A, SLP, chaperonin-60alpha | 16.4675 | 52.5604 |
| AT2G37270 | | ATRPS5B, RPS5B, ribosomal protein 5B | 50.8714 | 162.403 |
| AT4G27090 | | Ribosomal protein L14 | 244.956 | 783.115 |
| AT3G54560 | | HTA11, histone H2A 11 | 9.85564 | 31.5296 |
| AT5G08590 | | ASK2, ASK2, SNRK2-1, SNRK2.1, SRK2G, SNF1-related protein kinase 2.1 | 27.8075 | 89.0262 |
| AT1G48920 | | ATNUC-L1, NUC-L1, PARL1, nucleolin like 1 | 51.5798 | 165.15 |
| AT1G77610 | | EamA-like transporter family protein | 12.7652 | 40.9093 |
| AT2G37190 | | Ribosomal protein L11 family protein | 78.9364 | 253.414 |
| AT1G76550 | | Phosphofructokinase family protein | 10.7005 | 34.5947 |
| AT3G60530 | | GATA4, GATA transcription factor 4 | 6.3891 | 20.7078 |
| AT4G30920 | | Cytosol aminopeptidase family protein | 2.94062 | 9.53172 |
| AT2G43460 | | Ribosomal L38e protein family | 144.367 | 470.249 |
| AT3G22060 | | Receptor-like protein kinase-related family protein | 12.9982 | 42.3939 |
| AT1G17180 | | ATGSTU25, GSTU25, glutathione S-transferase TAU 25 | 424.129 | 1383.85 |
| AT5G16070 | | TCP-1/cpn60 chaperonin family protein | 14.7988 | 48.4299 |
| AT2G46280 | | TIF3I1, TRIP-1, TGF-beta receptor interacting protein 1 | 13.5518 | 44.3914 |
| AT5G11880 | | Pyridoxal-dependent decarboxylase family protein | 10.6083 | 34.7592 |
| AT1G17300 | | unknown protein | 5.14164 | 16.8863 |
| AT3G04400 | | emb2171, Ribosomal protein L14p/L23e family protein | 105.944 | 347.941 |
| AT5G47930 | | Zinc-binding ribosomal protein family protein | 111.366 | 365.982 |
| AT4G24830 | | arginosuccinate synthase family | 7.47327 | 24.6018 |
| AT3G53870 | | Ribosomal protein S3 family protein | 65.7694 | 216.63 |
| AT3G10720 | | Plant invertase/pectin methylesterase inhibitor superfamily | 65.4133 | 215.818 |
| AT4G30220 | | RUXF, small nuclear ribonucleoprotein F | 29.3357 | 96.8378 |
| AT3G47380 | | Plant invertase/pectin methylesterase inhibitor superfamily protein | 24.9944 | 82.5882 |
| AT4G27720 | | Major facilitator superfamily protein | 10.7365 | 35.4994 |
| AT2G36160 | | Ribosomal protein S11 family protein | 95.9392 | 317.692 |
| AT5G59870 | | HTA6, histone H2A 6 | 30.2748 | 100.396 |
| AT5G14640 | | ATSK13, SK13, shaggy-like kinase 13 | 22.3288 | 74.1812 |
| AT3G48460 | | GDSL-like Lipase/Acylhydrolase superfamily protein | 11.5256 | 38.3683 |
| AT1G63690 | | ATSPPL2, SPPL2, SIGNAL PEPTIDE PEPTIDASE-LIKE 2 | 6.66075 | 22.1752 |
| AT1G09760 | | U2A', U2 small nuclear ribonucleoprotein A | 11.9221 | 39.7292 |
| AT1G54690 | | G-H2AX, GAMMA-H2AX, H2AXB, HTA3, gamma histone variant H2AX | 17.0617 | 56.9813 |
| AT3G62870 | | Ribosomal protein L7Ae/L30e/S12e/Gadd45 family protein | 101.923 | 340.959 |
| AT3G59810 | | Small nuclear ribonucleoprotein family protein | 11.0548 | 37.0124 |
| AT4G23920 | | ATUGE2, UGE2, UDP-D-glucose/UDP-D-galactose 4-epimerase 2 | 16.4026 | 54.9604 |
| AT1G54580 | | ACP2, acyl carrier protein 2 | 32.5004 | 109.248 |
| AT5G54370 | | Late embryogenesis abundant (LEA) protein-related | 275.391 | 926.7 |
| AT2G39390 | | Ribosomal L29 family protein | 70.1616 | 236.577 |
| AT4G32330 | | TPX2 (targeting protein for Xklp2) protein family | 11.7314 | 39.6759 |
| AT2G03870 | | Small nuclear ribonucleoprotein family protein | 14.6384 | 49.5225 |
| AT2G40590 | | Ribosomal protein S26e family protein | 10.1072 | 34.2001 |
| AT1G74500 | | ATBS1, BS1, TMO7, activation-tagged BRI1(brassinosteroid-insensitive 1)-suppressor 1 | 32.4425 | 109.861 |
| AT5G62440 | | Protein of unknown function (DUF3223) | 11.681 | 39.5867 |
| AT3G24830 | | Ribosomal protein L13 family protein | 103.392 | 350.856 |
| AT3G53020 | | RPL24, RPL24B, STV1, Ribosomal protein L24e family protein | 115.27 | 391.953 |
| AT4G26110 | | ATNAP1;1, NAP1;1, nucleosome assembly protein1;1 | 15.3288 | 52.1288 |
| AT3G03920 | | H/ACA ribonucleoprotein complex, subunit Gar1/Naf1 protein | 24.0662 | 81.8627 |
| AT2G21660 | | ATGRP7, CCR2, GR-RBP7, GRP7, cold, circadian rhythm, and rna binding 2 | 92.1486 | 314.974 |
| AT3G52580 | | Ribosomal protein S11 family protein | 27.8444 | 95.3416 |
| AT2G34480 | | Ribosomal protein L18ae/LX family protein | 116.552 | 400.313 |
| AT1G77940 | | Ribosomal protein L7Ae/L30e/S12e/Gadd45 family protein | 173.36 | 595.726 |
| AT4G29690 | | Alkaline-phosphatase-like family protein | 22.6346 | 77.9902 |
| AT4G15000 | | Ribosomal L27e protein family | 114.484 | 395.472 |
| AT3G61110 | | ARS27A, RS27A, ribosomal protein S27 | 60.5084 | 209.111 |
| AT5G02870 | | Ribosomal protein L4/L1 family | 50.577 | 175.168 |
| AT4G36360 | | BGAL3, beta-galactosidase 3 | 11.6213 | 40.2905 |
| AT2G22770 | | NAI1, basic helix-loop-helix (bHLH) DNA-binding superfamily protein | 22.6026 | 78.5063 |
| AT4G18100 | | Ribosomal protein L32e | 153.826 | 535.466 |
| AT2G47610 | | Ribosomal protein L7Ae/L30e/S12e/Gadd45 family protein | 105.215 | 366.362 |
| AT3G49010 | | ATBBC1, BBC1, RSU2, breast basic conserved 1 | 54.9471 | 191.61 |
| AT3G56070 | | ROC2, rotamase cyclophilin 2 | 16.9181 | 59.0457 |
| AT2G17360 | | Ribosomal protein S4 (RPS4A) family protein | 76.0841 | 265.569 |
| AT1G45130 | | BGAL5, beta-galactosidase 5 | 8.36116 | 29.2758 |
| AT5G66860 | | Ribosomal protein L25/Gln-tRNA synthetase, anti-codon-binding domain | 8.3009 | 29.0717 |
| AT1G34030 | | Ribosomal protein S13/S18 family | 49.9913 | 175.238 |
| AT1G09690 | | Translation protein SH3-like family protein | 51.315 | 180.012 |
| AT3G11510 | | Ribosomal protein S11 family protein | 82.9163 | 291.149 |
| AT2G19740 | | Ribosomal protein L31e family protein | 54.7917 | 192.638 |
| AT4G35570 | | HMGB5, HMGD, NFD05, NFD5, high mobility group B5 | 31.985 | 112.637 |
| AT2G04030 | | AtHsp90.5, CR88, EMB1956, Hsp88.1, HSP90.5, Chaperone protein htpG family protein | 9.04988 | 31.9473 |
| AT1G74690 | | IQD31, IQ-domain 31 | 11.9168 | 42.0815 |
| AT5G14520 | | pescadillo-related | 12.2698 | 43.334 |
| AT1G22780 | | PFL, PFL1, RPS18A, Ribosomal protein S13/S18 family | 89.6342 | 316.587 |
| AT5G56710 | | Ribosomal protein L31e family protein | 123.972 | 438.197 |
| AT5G44710 | | CONTAINS InterPro DOMAIN/s: Ribosomal protein S27/S33, mitochondrial (InterPro:IPR013219); Has 101 Blast hits to 101 proteins in 55 species: Archae - 0; Bacteria - 0; Metazoa - 8; Fungi - 59; Plants - 26; Viruses - 0; Other Eukaryotes - 8 (source: NCBI BLink). | 7.95663 | 28.1612 |
| AT1G04430 | | S-adenosyl-L-methionine-dependent methyltransferases superfamily protein | 25.0784 | 89.0115 |
| AT3G20000 | | TOM40, translocase of the outer mitochondrial membrane 40 | 16.985 | 60.3176 |
| AT3G58610 | | ketol-acid reductoisomerase | 37.2474 | 132.293 |
| AT3G55020 | | Ypt/Rab-GAP domain of gyp1p superfamily protein | 9.77354 | 34.9414 |
| AT3G59540 | | Ribosomal L38e protein family | 122.329 | 437.689 |
| AT1G52670 | | Single hybrid motif superfamily protein | 7.37847 | 26.4032 |
| AT1G12270 | | stress-inducible protein, putative | 8.48363 | 30.4323 |
| AT2G20490 | | EDA27, NOP10, nucleolar RNA-binding Nop10p family protein | 13.3169 | 47.7876 |
| AT2G34300 | | S-adenosyl-L-methionine-dependent methyltransferases superfamily protein | 28.5249 | 102.378 |
| AT3G06035 | | Glycoprotein membrane precursor GPI-anchored | 9.90805 | 35.7227 |
| AT1G50920 | | Nucleolar GTP-binding protein | 15.1414 | 54.6685 |
| AT2G30200 | | catalytics;transferases;[acyl-carrier-protein] S-malonyltransferases;binding | 9.10679 | 32.9064 |
| AT5G06360 | | Ribosomal protein S8e family protein | 30.4496 | 110.168 |
| AT4G26230 | | Ribosomal protein L31e family protein | 42.376 | 154.126 |
| AT5G27850 | | Ribosomal protein L18e/L15 superfamily protein | 171.525 | 626.004 |
| AT1G69010 | | BIM2, BES1-interacting Myc-like protein 2 | 12.5529 | 45.8924 |
| AT5G62340 | | Plant invertase/pectin methylesterase inhibitor superfamily protein | 4.61395 | 16.8905 |
| AT1G23820 | | SPDS1, spermidine synthase 1 | 15.8605 | 58.325 |
| AT5G15230 | | GASA4, GAST1 protein homolog 4 | 225.647 | 830.231 |
| AT1G31850 | | S-adenosyl-L-methionine-dependent methyltransferases superfamily protein | 9.1168 | 33.6086 |
| AT2G37600 | | Ribosomal protein L36e family protein | 9.93532 | 36.6261 |
| AT2G40430 | | CONTAINS InterPro DOMAIN/s: P60-like (InterPro:IPR011687), Tumour suppressor protein Gltscr2 (InterPro:IPR011211); Has 709 Blast hits to 643 proteins in 201 species: Archae - 0; Bacteria - 32; Metazoa - 224; Fungi - 154; Plants - 45; Viruses - 0; Other Eukaryotes - 254 (source: NCBI BLink). | 11.2692 | 41.6321 |
| AT1G42960 | | expressed protein localized to the inner membrane of the chloroplast. | 22.9244 | 84.7081 |
| AT1G16920 | | ATRABA1B, RAB11, RABA1b, RAB GTPase homolog A1B | 17.6727 | 65.4491 |
| AT5G48240 | | unknown protein | 10.9016 | 40.4157 |
| AT1G23060 | | BEST Arabidopsis thaliana protein match is: TPX2 (targeting protein for Xklp2) protein family (TAIR:AT1G70950.1); Has 449 Blast hits to 419 proteins in 98 species: Archae - 0; Bacteria - 40; Metazoa - 139; Fungi - 21; Plants - 158; Viruses - 3; Other Eukaryotes - 88 (source: NCBI BLink). | 12.7268 | 47.5709 |
| AT5G08180 | | Ribosomal protein L7Ae/L30e/S12e/Gadd45 family protein | 21.5234 | 80.6209 |
| AT3G28900 | | Ribosomal protein L34e superfamily protein | 32.5153 | 121.802 |
| AT4G09800 | | RPS18C, S18 ribosomal protein | 80.0455 | 299.964 |
| AT4G18440 | | L-Aspartase-like family protein | 8.53801 | 32.0003 |
| AT2G44120 | | Ribosomal protein L30/L7 family protein | 46.757 | 175.861 |
| AT1G18800 | | NRP2, NAP1-related protein 2 | 11.1168 | 41.8453 |
| AT1G54610 | | Protein kinase superfamily protein | 5.16715 | 19.4604 |
| AT5G53760 | | ATMLO11, MLO11, Seven transmembrane MLO family protein | 8.16588 | 30.7844 |
| AT3G53740 | | Ribosomal protein L36e family protein | 69.3473 | 261.563 |
| AT3G58700 | | Ribosomal L5P family protein | 15.5191 | 58.609 |
| AT5G04600 | | RNA-binding (RRM/RBD/RNP motifs) family protein | 18.1238 | 68.5986 |
| AT4G11290 | | Peroxidase superfamily protein | 11.6934 | 44.2774 |
| AT1G74060 | | Ribosomal protein L6 family protein | 13.1704 | 49.8944 |
| AT3G23390 | | Zinc-binding ribosomal protein family protein | 87.2029 | 331.019 |
| AT1G49760 | | PAB8, PABP8, poly(A) binding protein 8 | 15.9053 | 60.4242 |
| AT2G01250 | | Ribosomal protein L30/L7 family protein | 100.764 | 384.206 |
| AT2G04160 | | AIR3, Subtilisin-like serine endopeptidase family protein | 25.4359 | 97.0762 |
| AT3G05560 | | Ribosomal L22e protein family | 60.4581 | 231.229 |
| AT3G02560 | | Ribosomal protein S7e family protein | 64.5742 | 247.416 |
| AT5G59030 | | COPT1, copper transporter 1 | 8.63904 | 33.1047 |
| AT3G28860 | | ABCB19, ATABCB19, ATMDR1, ATMDR11, ATPGP19, MDR1, MDR11, PGP19, ATP binding cassette subfamily B19 | 8.40245 | 32.3013 |
| AT2G21250 | | NAD(P)-linked oxidoreductase superfamily protein | 6.87316 | 26.4885 |
| AT3G03310 | | ATLCAT3, LCAT3, lecithin:cholesterol acyltransferase 3 | 10.4247 | 40.2835 |
| AT5G55110 | | Stigma-specific Stig1 family protein | 48.2223 | 186.738 |
| AT3G22960 | | PKP-ALPHA, PKP1, Pyruvate kinase family protein | 10.9819 | 42.5505 |
| AT3G60770 | | Ribosomal protein S13/S15 | 64.4118 | 250.469 |
| AT2G21580 | | Ribosomal protein S25 family protein | 37.415 | 145.525 |
| AT1G25260 | | Ribosomal protein L10 family protein | 22.3998 | 87.1278 |
| AT2G20060 | | Ribosomal protein L4/L1 family | 11.5039 | 44.7854 |
| AT2G43880 | | Pectin lyase-like superfamily protein | 15.0492 | 58.7877 |
| AT3G04840 | | Ribosomal protein S3Ae | 114.226 | 446.351 |
| AT3G61930 | | unknown protein | 23.0083 | 89.9316 |
| AT1G25275 | | unknown protein | 238.95 | 935.678 |
| AT2G21060 | | ATCSP4, ATGRP2B, GRP2B, glycine-rich protein 2B | 11.5172 | 45.3419 |
| AT1G30440 | | Phototropic-responsive NPH3 family protein | 8.34059 | 32.8416 |
| AT4G33680 | | AGD2, Pyridoxal phosphate (PLP)-dependent transferases superfamily protein | 7.78889 | 30.6789 |
| AT2G42870 | | HLH1, PAR1, phy rapidly regulated 1 | 4.68788 | 18.5274 |
| AT5G44200 | | ATCBP20, CBP20, CAP-binding protein 20 | 5.89899 | 23.3287 |
| AT4G28360 | | Ribosomal protein L22p/L17e family protein | 6.79295 | 27.0217 |
| AT5G15520 | | Ribosomal protein S19e family protein | 7.46798 | 29.7931 |
| AT3G04920 | | Ribosomal protein S24e family protein | 80.1522 | 320.615 |
| AT4G13850 | | ATGRP2, GR-RBP2, GRP2, glycine-rich RNA-binding protein 2 | 36.7638 | 147.221 |
| AT4G25260 | | Plant invertase/pectin methylesterase inhibitor superfamily protein | 27.6335 | 110.761 |
| AT1G78340 | | ATGSTU22, GSTU22, glutathione S-transferase TAU 22 | 41.7122 | 167.626 |
| AT5G08420 | | RNA-binding KH domain-containing protein | 7.87278 | 31.9127 |
| AT3G11830 | | TCP-1/cpn60 chaperonin family protein | 41.9922 | 170.551 |
| AT3G11120 | | Ribosomal protein L41 family | 96.4318 | 392.828 |
| AT2G41650 | | unknown protein | 17.2273 | 70.1913 |
| AT5G60700 | | glycosyltransferase family protein 2 | 14.6395 | 59.6668 |
| AT5G20950 | | Glycosyl hydrolase family protein | 15.3429 | 62.6194 |
| AT3G25520 | | ATL5, OLI5, PGY3, RPL5A, ribosomal protein L5 | 110.351 | 450.782 |
| AT3G16780 | | Ribosomal protein L19e family protein | 15.5842 | 63.7194 |
| AT3G15000 | | cobalt ion binding | 10.7037 | 43.9662 |
| AT5G20890 | | TCP-1/cpn60 chaperonin family protein | 26.9035 | 110.703 |
| AT2G25210 | | Ribosomal protein L39 family protein | 91.0872 | 374.855 |
| AT1G63980 | | D111/G-patch domain-containing protein | 12.1689 | 50.2259 |
| AT2G32060 | | Ribosomal protein L7Ae/L30e/S12e/Gadd45 family protein | 57.6381 | 238.001 |
| AT4G17190 | | FPS2, farnesyl diphosphate synthase 2 | 12.2276 | 50.5493 |
| AT1G04870 | | ATPRMT10, PRMT10, protein arginine methyltransferase 10 | 8.44696 | 34.9522 |
| AT3G56020 | | Ribosomal protein L41 family | 55.3891 | 229.233 |
| AT3G20160 | | Terpenoid synthases superfamily protein | 9.98425 | 41.3285 |
| AT5G10400 | | Histone superfamily protein | 4.66434 | 19.3878 |
| AT3G49080 | | Ribosomal protein S5 domain 2-like superfamily protein | 6.13118 | 25.4858 |
| AT4G15160 | | Bifunctional inhibitor/lipid-transfer protein/seed storage 2S albumin superfamily protein | 103.353 | 430.357 |
| AT5G27770 | | Ribosomal L22e protein family | 70.6174 | 294.347 |
| AT3G46040 | | RPS15AD, ribosomal protein S15A D | 23.6042 | 99.0316 |
| AT4G29410 | | Ribosomal L28e protein family | 39.6759 | 167.062 |
| AT5G01020 | | Protein kinase superfamily protein | 4.20038 | 17.6903 |
| AT3G62530 | | ARM repeat superfamily protein | 7.84634 | 33.1564 |
| AT4G10480 | | Nascent polypeptide-associated complex (NAC), alpha subunit family protein | 23.7579 | 100.414 |
| AT3G08680 | | Leucine-rich repeat protein kinase family protein | 5.96838 | 25.2811 |
| AT3G45030 | | Ribosomal protein S10p/S20e family protein | 26.6977 | 113.166 |
| AT1G18840 | | IQD30, IQ-domain 30 | 5.76536 | 24.4687 |
| AT5G58420 | | Ribosomal protein S4 (RPS4A) family protein | 53.4745 | 228.385 |
| AT1G61580 | | ARP2, RPL3B, R-protein L3 B | 8.2267 | 35.1682 |
| AT3G02530 | | TCP-1/cpn60 chaperonin family protein | 11.1522 | 47.7101 |
| AT1G20950 | | Phosphofructokinase family protein | 14.9309 | 64.265 |
| AT2G38120 | | AUX1, MAP1, PIR1, WAV5, Transmembrane amino acid transporter family protein | 15.3719 | 66.222 |
| AT4G02290 | | AtGH9B13, GH9B13, glycosyl hydrolase 9B13 | 7.13386 | 30.7548 |
| AT1G55240 | | Family of unknown function (DUF716) | 6.43615 | 27.7914 |
| AT2G40840 | | DPE2, disproportionating enzyme 2 | 4.79701 | 20.7178 |
| AT3G14600 | | Ribosomal protein L18ae/LX family protein | 45.7158 | 197.444 |
| AT5G23740 | | RPS11-BETA, ribosomal protein S11-beta | 59.0756 | 255.234 |
| AT5G62190 | | PRH75, DEAD box RNA helicase (PRH75) | 19.2704 | 83.3518 |
| AT4G02715 | | unknown protein | 6.62545 | 28.6751 |
| AT2G18740 | | Small nuclear ribonucleoprotein family protein | 21.9792 | 95.1869 |
| AT3G16080 | | Zinc-binding ribosomal protein family protein | 42.7314 | 185.198 |
| AT5G22650 | | ATHD2, ATHD2B, HD2, HD2B, HDA4, HDT02, HDT2, histone deacetylase 2B | 23.0289 | 100.062 |
| AT2G35040 | | AICARFT/IMPCHase bienzyme family protein | 7.85793 | 34.1793 |
| AT3G57610 | | ADSS, adenylosuccinate synthase | 5.86917 | 25.6269 |
| AT3G12390 | | Nascent polypeptide-associated complex (NAC), alpha subunit family protein | 15.4539 | 67.5073 |
| AT1G08360 | | Ribosomal protein L1p/L10e family | 81.3253 | 355.83 |
| AT3G56130 | | biotin/lipoyl attachment domain-containing protein | 8.02752 | 35.1884 |
| AT2G23930 | | SNRNP-G, probable small nuclear ribonucleoprotein G | 19.7257 | 86.8061 |
| AT1G57820 | | ORTH2, VIM1, Zinc finger (C3HC4-type RING finger) family protein | 3.31551 | 14.5944 |
| AT2G02130 | | LCR68, PDF2.3, low-molecular-weight cysteine-rich 68 | 22.5552 | 99.5676 |
| AT3G15480 | | Protein of unknown function (DUF1218) | 11.9612 | 52.8028 |
| AT2G20450 | | Ribosomal protein L14 | 26.4747 | 116.905 |
| AT3G18190 | | TCP-1/cpn60 chaperonin family protein | 18.2622 | 80.6411 |
| AT4G18030 | | S-adenosyl-L-methionine-dependent methyltransferases superfamily protein | 26.8751 | 119.187 |
| AT2G15490 | | UGT73B4, UDP-glycosyltransferase 73B4 | 21.433 | 95.7568 |
| AT4G34555 | | Ribosomal protein S25 family protein | 10.5184 | 47.0744 |
| AT5G33320 | | ARAPPT, CUE1, PPT, Glucose-6-phosphate/phosphate translocator-related | 12.3978 | 55.9428 |
| AT3G20940 | | CYP705A30, cytochrome P450, family 705, subfamily A, polypeptide 30 | 6.84581 | 30.9444 |
| AT2G33210 | | HSP60-2, heat shock protein 60-2 | 12.6536 | 57.3312 |
| AT1G47510 | | 5PTASE11, AT5PTASE11, inositol polyphosphate 5-phosphatase 11 | 31.2342 | 141.935 |
| AT1G74050 | | Ribosomal protein L6 family protein | 9.46952 | 43.1989 |
| AT3G54470 | | uridine 5'-monophosphate synthase / UMP synthase (PYRE-F) (UMPS) | 12.1166 | 55.2769 |
| AT4G32720 | | AtLa1, La1, La protein 1 | 13.5969 | 62.0473 |
| AT5G02960 | | Ribosomal protein S12/S23 family protein | 97.9541 | 447.113 |
| AT1G35780 | | unknown protein | 15.9296 | 72.8949 |
| AT5G09510 | | Ribosomal protein S19 family protein | 10.6007 | 48.6064 |
| AT5G16130 | | Ribosomal protein S7e family protein | 55.5382 | 255.076 |
| AT1G09620 | | ATP binding;leucine-tRNA ligases;aminoacyl-tRNA ligases;nucleotide binding;ATP binding;aminoacyl-tRNA ligases | 7.76209 | 35.7316 |
| AT3G17160 | | unknown protein | 9.49223 | 43.7647 |
| AT5G10510 | | AIL6, PLT3, AINTEGUMENTA-like 6 | 14.4633 | 66.8074 |
| AT5G10160 | | Thioesterase superfamily protein | 6.75571 | 31.2506 |
| AT1G06220 | | CLO, GFA1, MEE5, Ribosomal protein S5/Elongation factor G/III/V family protein | 3.28022 | 15.2126 |
| AT3G14990 | | Class I glutamine amidotransferase-like superfamily protein | 43.0321 | 199.57 |
| AT5G35360 | | CAC2, acetyl Co-enzyme a carboxylase biotin carboxylase subunit | 24.6107 | 114.512 |
| AT5G54970 | | unknown protein | 6.08011 | 28.3772 |
| AT2G27530 | | PGY1, Ribosomal protein L1p/L10e family | 85.4112 | 398.682 |
| AT3G10610 | | Ribosomal S17 family protein | 9.58928 | 44.7769 |
| AT5G60670 | | Ribosomal protein L11 family protein | 17.3108 | 80.9614 |
| AT1G64880 | | Ribosomal protein S5 family protein | 7.48854 | 35.0248 |
| AT1G51470 | | BGLU35, TGG5, beta glucosidase 35 | 32.6281 | 152.762 |
| AT3G45230 | | hydroxyproline-rich glycoprotein family protein | 7.06551 | 33.0954 |
| AT1G66700 | | PXMT1, S-adenosyl-L-methionine-dependent methyltransferases superfamily protein | 4.68774 | 21.9632 |
| AT1G33120 | | Ribosomal protein L6 family | 6.86193 | 32.2172 |
| AT5G46290 | | KAS I, KAS1, 3-ketoacyl-acyl carrier protein synthase I | 14.5691 | 68.4326 |
| AT3G03960 | | TCP-1/cpn60 chaperonin family protein | 18.4491 | 86.9818 |
| AT3G58660 | | Ribosomal protein L1p/L10e family | 8.09071 | 38.206 |
| AT1G05280 | | Protein of unknown function (DUF604) | 12.3842 | 58.7869 |
| AT5G22140 | | FAD/NAD(P)-binding oxidoreductase family protein | 15.9854 | 76.6152 |
| AT3G23940 | | dehydratase family | 9.32529 | 44.7999 |
| AT2G27840 | | HDA13, HDT04, HDT4, histone deacetylase-related / HD-related | 8.96018 | 43.0833 |
| AT5G22880 | | H2B, HTB2, histone B2 | 3.11422 | 14.9745 |
| AT1G29250 | | Alba DNA/RNA-binding protein | 14.3987 | 69.714 |
| AT3G18490 | | Eukaryotic aspartyl protease family protein | 3.3209 | 16.0885 |
| AT1G24160 | | unknown protein | 4.86267 | 23.5945 |
| AT2G19750 | | Ribosomal protein S30 family protein | 16.3515 | 79.4874 |
| AT4G21865 | | unknown protein | 8.14916 | 39.6767 |
| AT1G27400 | | Ribosomal protein L22p/L17e family protein | 42.2189 | 205.821 |
| AT1G05010 | | ACO4, EAT1, EFE, ethylene-forming enzyme | 18.8498 | 92.2947 |
| AT1G48630 | | RACK1B_AT, receptor for activated C kinase 1B | 21.6994 | 106.3 |
| AT1G34430 | | EMB3003, 2-oxoacid dehydrogenases acyltransferase family protein | 4.57425 | 22.4358 |
| AT2G43470 | | Protein of unknown function (DUF3755) | 5.31159 | 26.0554 |
| AT1G12780 | | ATUGE1, UGE1, UDP-D-glucose/UDP-D-galactose 4-epimerase 1 | 16.3777 | 80.5161 |
| AT2G39460 | | ATRPL23A, RPL23A, RPL23AA, ribosomal protein L23AA | 83.0108 | 408.604 |
| AT1G18540 | | Ribosomal protein L6 family protein | 73.999 | 366.877 |
| AT3G55750 | | Ribosomal protein L35Ae family protein | 32.7479 | 163.081 |
| AT4G22130 | | SRF8, STRUBBELIG-receptor family 8 | 4.83697 | 24.13 |
| AT3G22310 | | ATRH9, PMH1, putative mitochondrial RNA helicase 1 | 4.32579 | 21.601 |
| AT4G34670 | | Ribosomal protein S3Ae | 69.6244 | 348.524 |
| AT1G22400 | | ATUGT85A1, UGT85A1, UDP-Glycosyltransferase superfamily protein | 10.1149 | 50.6732 |
| AT1G20190 | | ATEXP11, ATEXPA11, ATHEXP ALPHA 1.14, EXP11, EXPA11, expansin 11 | 11.545 | 57.9073 |
| AT5G59850 | | Ribosomal protein S8 family protein | 45.1323 | 226.554 |
| AT5G50170 | | C2 calcium/lipid-binding and GRAM domain containing protein | 2.69481 | 13.5384 |
| AT1G33290 | | P-loop containing nucleoside triphosphate hydrolases superfamily protein | 5.50635 | 27.6906 |
| AT2G41280 | | ATM10, M10, late embryogenesis abundant protein (M10) / LEA protein M10 | 10.3855 | 52.4019 |
| AT1G13730 | | Nuclear transport factor 2 (NTF2) family protein with RNA binding (RRM-RBD-RNP motifs) domain | 4.65547 | 23.5099 |
| AT1G58380 | | XW6, Ribosomal protein S5 family protein | 3.50776 | 17.7392 |
| AT4G04460 | | Saposin-like aspartyl protease family protein | 6.52072 | 33.043 |
| AT3G10090 | | Nucleic acid-binding, OB-fold-like protein | 18.0779 | 92.1006 |
| AT5G56500 | | TCP-1/cpn60 chaperonin family protein | 5.84215 | 29.8172 |
| AT4G25340 | | ATFKBP53, FKBP53, FK506 BINDING PROTEIN 53 | 9.09031 | 46.45 |
| AT4G25730 | | FtsJ-like methyltransferase family protein | 4.99895 | 25.616 |
| AT3G51800 | | ATEBP1, ATG2, EBP1, metallopeptidase M24 family protein | 20.7088 | 106.78 |
| AT5G61030 | | GR-RBP3, glycine-rich RNA-binding protein 3 | 5.15351 | 26.5737 |
| AT3G18600 | | P-loop containing nucleoside triphosphate hydrolases superfamily protein | 7.10633 | 36.7029 |
| AT2G40360 | | Transducin/WD40 repeat-like superfamily protein | 8.47455 | 43.8702 |
| AT4G15530 | | PPDK, pyruvate orthophosphate dikinase | 7.2151 | 37.4071 |
| AT5G45775 | | Ribosomal L5P family protein | 35.0495 | 181.876 |
| AT5G02050 | | Mitochondrial glycoprotein family protein | 8.67697 | 45.218 |
| AT5G25754 | | RNA polymerase I-associated factor PAF67 | 0.534901 | 2.78824 |
| AT1G08580 | | unknown protein | 6.02861 | 31.5528 |
| AT5G62700 | | TUB3, tubulin beta chain 3 | 5.28788 | 27.859 |
| AT3G23620 | | Ribosomal RNA processing Brix domain protein | 8.41279 | 44.4323 |
| AT3G05020 | | ACP, ACP1, acyl carrier protein 1 | 22.9066 | 121.44 |
| AT5G15940 | | NAD(P)-binding Rossmann-fold superfamily protein | 5.46488 | 29.2617 |
| AT5G62690 | | TUB2, tubulin beta chain 2 | 4.9964 | 26.8251 |
| AT5G50810 | | TIM8, translocase inner membrane subunit 8 | 11.3084 | 60.7798 |
| AT1G24510 | | TCP-1/cpn60 chaperonin family protein | 22.8093 | 122.667 |
| AT3G27740 | | CARA, carbamoyl phosphate synthetase A | 4.95313 | 26.6987 |
| AT1G07070 | | Ribosomal protein L35Ae family protein | 9.77295 | 52.9787 |
| AT1G61900 | | unknown protein | 4.54538 | 24.6589 |
| AT4G28720 | | YUC8, Flavin-binding monooxygenase family protein | 3.82449 | 20.8033 |
| AT5G61020 | | ECT3, evolutionarily conserved C-terminal region 3 | 7.24788 | 39.4456 |
| AT1G80750 | | Ribosomal protein L30/L7 family protein | 10.5489 | 57.4875 |
| AT5G24070 | | Peroxidase superfamily protein | 11.763 | 64.6283 |
| AT1G47600 | | BGLU34, TGG4, beta glucosidase 34 | 19.7017 | 108.261 |
| AT5G14020 | | Endosomal targeting BRO1-like domain-containing protein | 13.8509 | 76.169 |
| AT2G17280 | | Phosphoglycerate mutase family protein | 25.46 | 140.086 |
| AT3G44750 | | ATHD2A, HD2A, HDA3, HDT1, histone deacetylase 3 | 13.162 | 72.838 |
| AT2G19520 | | ACG1, ATMSI4, FVE, MSI4, NFC04, NFC4, Transducin family protein / WD-40 repeat family protein | 4.52894 | 25.1055 |
| AT2G11910 | | unknown protein | 8.60622 | 47.7739 |
| AT3G22660 | | rRNA processing protein-related | 7.04476 | 39.3913 |
| AT2G45050 | | GATA2, GATA transcription factor 2 | 4.31905 | 24.2422 |
| AT3G55280 | | RPL23AB, ribosomal protein L23AB | 39.9824 | 224.466 |
| AT3G23830 | | GR-RBP4, GRP4, glycine-rich RNA-binding protein 4 | 20.7582 | 116.867 |
| AT1G19920 | | APS2, ASA1, Pseudouridine synthase/archaeosine transglycosylase-like family protein | 5.98343 | 33.7967 |
| AT5G39740 | | OLI7, RPL5B, ribosomal protein L5 B | 56.7214 | 325.196 |
| AT3G43980 | | Ribosomal protein S14p/S29e family protein | 8.29401 | 47.6252 |
| AT3G57150 | | AtCBF5, AtNAP57, CBF5, NAP57, homologue of NAP57 | 9.67676 | 56.082 |
| AT2G27430 | | ARM repeat superfamily protein | 4.2847 | 24.94 |
| AT1G36060 | | Integrase-type DNA-binding superfamily protein | 6.49627 | 37.8333 |
| AT4G30930 | | NFD1, Ribosomal protein L21 | 4.14373 | 24.1551 |
| AT1G05530 | | UGT2, UGT75B2, UDP-glucosyl transferase 75B2 | 2.76018 | 16.1032 |
| AT1G02920 | | ATGST11, ATGSTF7, ATGSTF8, GST11, GSTF7, glutathione S-transferase 7 | 12.7012 | 74.2917 |
| AT1G08560 | | ATSYP111, KN, SYP111, syntaxin of plants 111 | 4.28673 | 25.1507 |
| AT3G62220 | | Protein kinase superfamily protein | 3.42523 | 20.1722 |
| AT5G26360 | | TCP-1/cpn60 chaperonin family protein | 15.4786 | 91.2002 |
| AT5G23860 | | TUB8, tubulin beta 8 | 6.67904 | 39.7529 |
| AT3G18130 | | RACK1C_AT, receptor for activated C kinase 1C | 14.0314 | 83.6378 |
| AT5G52920 | | PKP-BETA1, PKP1, PKP2, plastidic pyruvate kinase beta subunit 1 | 11.0057 | 65.8539 |
| AT3G19430 | | late embryogenesis abundant protein-related / LEA protein-related | 15.0042 | 89.8936 |
| AT2G27970 | | CKS2, CDK-subunit 2 | 3.36519 | 20.4812 |
| AT1G62480 | | Vacuolar calcium-binding protein-related | 16.5631 | 101.061 |
| AT1G55490 | | CPN60B, LEN1, chaperonin 60 beta | 3.35968 | 20.5256 |
| AT1G44760 | | Adenine nucleotide alpha hydrolases-like superfamily protein | 4.608 | 28.2736 |
| AT4G12600 | | Ribosomal protein L7Ae/L30e/S12e/Gadd45 family protein | 27.7011 | 171.627 |
| AT5G50375 | | CPI1, cyclopropyl isomerase | 4.26788 | 26.5881 |
| AT3G05060 | | NOP56-like pre RNA processing ribonucleoprotein | 9.48303 | 59.3268 |
| AT4G10350 | | ANAC070, BRN2, NAC070, NAC domain containing protein 70 | 5.64203 | 35.5799 |
| AT1G57860 | | Translation protein SH3-like family protein | 0.807362 | 5.10607 |
| AT5G22440 | | Ribosomal protein L1p/L10e family | 25.9392 | 164.293 |
| AT1G56680 | | Chitinase family protein | 6.7644 | 43.2239 |
| AT5G23210 | | SCPL34, serine carboxypeptidase-like 34 | 8.13603 | 52.3781 |
| AT1G06520 | | ATGPAT1, GPAT1, glycerol-3-phosphate acyltransferase 1 | 3.7761 | 24.4868 |
| AT5G50175 | | unknown protein | 6.30201 | 41.0337 |
| AT5G48300 | | ADG1, APS1, ADP glucose pyrophosphorylase 1 | 3.2679 | 21.4228 |
| AT1G41880 | | Ribosomal protein L35Ae family protein | 17.4038 | 114.852 |
| AT3G16410 | | NSP4, nitrile specifier protein 4 | 5.14576 | 33.9805 |
| AT1G33280 | | ANAC015, BRN1, NAC015, NAC domain containing protein 15 | 13.3652 | 88.2689 |
| AT1G52930 | | Ribosomal RNA processing Brix domain protein | 6.85407 | 45.3142 |
| AT1G68560 | | ATXYL1, TRG1, XYL1, alpha-xylosidase 1 | 4.35551 | 28.9031 |
| AT4G03190 | | AFB1, ATGRH1, GRH1, GRR1-like protein 1 | 15.3526 | 102.702 |
| AT1G50060 | | CAP (Cysteine-rich secretory proteins, Antigen 5, and Pathogenesis-related 1 protein) superfamily protein | 37.49 | 251.173 |
| AT1G17285 | | unknown protein | 11.3118 | 75.8551 |
| AT1G05260 | | RCI3, RCI3A, Peroxidase superfamily protein | 29.7138 | 199.823 |
| AT2G05990 | | ENR1, MOD1, NAD(P)-binding Rossmann-fold superfamily protein | 8.70482 | 58.5864 |
| AT5G26850 | | Uncharacterized protein | 3.37829 | 22.9442 |
| AT2G43610 | | Chitinase family protein | 177.063 | 1205.59 |
| AT4G27400 | | Late embryogenesis abundant (LEA) protein-related | 17.2036 | 117.743 |
| AT5G62670 | | AHA11, HA11, H(+)-ATPase 11 | 3.90128 | 26.731 |
| AT4G11610 | | C2 calcium/lipid-binding plant phosphoribosyltransferase family protein | 2.75461 | 18.9229 |
| AT4G22235 | | Arabidopsis defensin-like protein | 3.31096 | 22.8351 |
| AT2G45470 | | AGP8, FLA8, FASCICLIN-like arabinogalactan protein 8 | 3.13959 | 21.7239 |
| AT3G07050 | | GTP-binding family protein | 6.96071 | 48.4259 |
| AT1G31970 | | STRS1, DEA(D/H)-box RNA helicase family protein | 5.20258 | 36.2868 |
| AT2G41290 | | SSL2, strictosidine synthase-like 2 | 3.75801 | 26.36 |
| AT3G57930 | | unknown protein | 5.67103 | 40.0047 |
| AT5G20045 | | unknown protein | 6.68029 | 47.5378 |
| AT3G22275 | | unknown protein | 5.25907 | 37.5873 |
| AT5G51520 | | Plant invertase/pectin methylesterase inhibitor superfamily protein | 27.5604 | 197.649 |
| AT1G56110 | | NOP56, homolog of nucleolar protein NOP56 | 11.861 | 86.2635 |
| AT5G50180 | | Protein kinase superfamily protein | 2.98207 | 21.7607 |
| AT5G27120 | | NOP56-like pre RNA processing ribonucleoprotein | 6.66396 | 48.9089 |
| AT3G42180 | | Exostosin family protein | 2.95273 | 21.6785 |
| AT2G34020 | | Calcium-binding EF-hand family protein | 7.5331 | 55.5858 |
| AT1G23410 | | Ribosomal protein S27a / Ubiquitin family protein | 6.05838 | 44.899 |
| AT4G26760 | | MAP65-2, microtubule-associated protein 65-2 | 3.12014 | 23.1281 |
| AT4G25630 | | ATFIB2, FIB2, fibrillarin 2 | 13.9495 | 103.693 |
| AT5G33290 | | XGD1, xylogalacturonan deficient 1 | 3.65177 | 27.2255 |
| AT1G74560 | | NRP1, NAP1-related protein 1 | 6.88408 | 51.6981 |
| AT2G22230 | | Thioesterase superfamily protein | 3.5812 | 26.9153 |
| AT2G22400 | | S-adenosyl-L-methionine-dependent methyltransferases superfamily protein | 2.69546 | 20.7839 |
| AT5G48900 | | Pectin lyase-like superfamily protein | 8.83755 | 68.2504 |
| AT1G73490 | | RNA-binding (RRM/RBD/RNP motifs) family protein | 2.06821 | 16.0398 |
| AT1G28290 | | AGP31, arabinogalactan protein 31 | 74.6466 | 581.627 |
| AT3G25860 | | LTA2, PLE2, 2-oxoacid dehydrogenases acyltransferase family protein | 4.61896 | 36.0991 |
| AT5G10130 | | Pollen Ole e 1 allergen and extensin family protein | 20.3089 | 159.269 |
| AT3G11250 | | Ribosomal protein L10 family protein | 3.92091 | 30.808 |
| AT4G39950 | | CYP79B2, cytochrome P450, family 79, subfamily B, polypeptide 2 | 8.18523 | 65.1306 |
| AT1G66430 | | pfkB-like carbohydrate kinase family protein | 1.94672 | 15.5611 |
| AT5G27330 | | Prefoldin chaperone subunit family protein | 2.28995 | 18.4874 |
| AT5G14750 | | ATMYB66, MYB66, WER, WER1, myb domain protein 66 | 1.79421 | 14.527 |
| AT5G23700 | | unknown protein | 2.48865 | 20.3112 |
| AT1G57660 | | Translation protein SH3-like family protein | 6.72496 | 54.9453 |
| AT5G58000 | | Reticulon family protein | 2.52254 | 20.9048 |
| AT1G21340 | | Dof-type zinc finger DNA-binding family protein | 7.15111 | 60.2249 |
| AT3G16950 | | LPD1, ptlpd1, lipoamide dehydrogenase 1 | 3.32487 | 28.0782 |
| AT1G31950 | | Terpenoid cyclases/Protein prenyltransferases superfamily protein | 1.73283 | 14.7211 |
| AT5G08580 | | Calcium-binding EF hand family protein | 2.14096 | 18.2887 |
| AT2G22330 | | CYP79B3, cytochrome P450, family 79, subfamily B, polypeptide 3 | 1.81983 | 15.7708 |
| AT1G76520 | | Auxin efflux carrier family protein | 5.10099 | 44.9443 |
| AT1G33750 | | Terpenoid cyclases/Protein prenyltransferases superfamily protein | 3.13003 | 28.0143 |
| AT3G04330 | | Kunitz family trypsin and protease inhibitor protein | 5.96559 | 53.8403 |
| AT1G05680 | | UGT74E2, Uridine diphosphate glycosyltransferase 74E2 | 11.7951 | 106.525 |
| AT3G59480 | | pfkB-like carbohydrate kinase family protein | 7.72429 | 70.3164 |
| AT1G52050 | | Mannose-binding lectin superfamily protein | 54.5289 | 498.896 |
| AT2G33790 | | AGP30, ATAGP30, arabinogalactan protein 30 | 14.6109 | 133.751 |
| AT3G49190 | | O-acyltransferase (WSD1-like) family protein | 3.78604 | 34.8532 |
| AT1G52070 | | Mannose-binding lectin superfamily protein | 60.3826 | 556.082 |
| AT4G33270 | | CDC20.1, Transducin family protein / WD-40 repeat family protein | 0.519208 | 4.79849 |
| AT4G10450 | | Ribosomal protein L6 family | 8.56964 | 79.7185 |
| AT5G57800 | | CER3, FLP1, WAX2, YRE, Fatty acid hydroxylase superfamily | 1.59943 | 15.3714 |
| AT4G23590 | | Tyrosine transaminase family protein | 7.80659 | 75.7888 |
| AT2G28260 | | ATCNGC15, CNGC15, cyclic nucleotide-gated channel 15 | 1.42357 | 13.8326 |
| AT3G03500 | | TatD related DNase | 11.0456 | 108.026 |
| AT3G04320 | | Kunitz family trypsin and protease inhibitor protein | 11.984 | 118.005 |
| AT1G07280 | | Tetratricopeptide repeat (TPR)-like superfamily protein | 1.98373 | 19.6592 |
| AT3G49630 | | 2-oxoglutarate (2OG) and Fe(II)-dependent oxygenase superfamily protein | 2.60532 | 26.2566 |
| AT1G70850 | | MLP34, MLP-like protein 34 | 2.27789 | 23.417 |
| AT4G23800 | | HMG (high mobility group) box protein | 2.8921 | 30.9364 |
| AT5G63650 | | SNRK2-5, SNRK2.5, SRK2H, SNF1-related protein kinase 2.5 | 8.84481 | 95.3899 |
| AT4G01240 | | S-adenosyl-L-methionine-dependent methyltransferases superfamily protein | 1.68931 | 18.3807 |
| AT2G28310 | | Protein of unknown function (DUF707) | 1.45483 | 15.8962 |
| AT3G52920 | | Family of unknown function (DUF662) | 1.86604 | 20.5092 |
| AT3G22740 | | HMT3, homocysteine S-methyltransferase 3 | 24.6335 | 271.882 |
| AT5G25757 | | RNA polymerase I-associated factor PAF67 | 0.238792 | 2.64535 |
| AT2G31940 | | unknown protein | 5.02049 | 56.4294 |
| AT2G33380 | | CLO-3, RD20, Caleosin-related family protein | 1.40697 | 16.0861 |
| AT5G13870 | | EXGT-A4, XTH5, xyloglucan endotransglucosylase/hydrolase 5 | 4.40953 | 51.4893 |
| AT1G07290 | | GONST2, golgi nucleotide sugar transporter 2 | 4.32277 | 51.0682 |
| AT5G16250 | | unknown protein | 1.88572 | 22.9668 |
| AT5G63660 | | LCR74, PDF2.5, Scorpion toxin-like knottin superfamily protein | 7.12469 | 88.4502 |
| AT1G54010 | | GDSL-like Lipase/Acylhydrolase superfamily protein | 7.09741 | 88.6631 |
| AT1G05560 | | UGT1, UGT75B1, UDP-glucosyltransferase 75B1 | 1.16393 | 14.6287 |
| AT4G15350 | | CYP705A2, cytochrome P450, family 705, subfamily A, polypeptide 2 | 2.85101 | 36.0766 |
| AT3G62280 | | GDSL-like Lipase/Acylhydrolase superfamily protein | 1.74749 | 22.3418 |
| AT4G15300 | | CYP702A2, cytochrome P450, family 702, subfamily A, polypeptide 2 | 2.1599 | 27.6756 |
| AT5G02580 | | Plant protein 1589 of unknown function | 2.02105 | 27.0358 |
| AT5G60520 | | Late embryogenesis abundant (LEA) protein-related | 1.55819 | 20.9503 |
| AT1G52060 | | Mannose-binding lectin superfamily protein | 24.8965 | 336.642 |
| AT4G25310 | | 2-oxoglutarate (2OG) and Fe(II)-dependent oxygenase superfamily protein | 9.10739 | 125.286 |
| AT5G44417 | | pseudogene, similar to CPRD2, blastp match of 42% identity and 7.0e-94 P-value to GP|13161397|dbj|BAB33033.1||AB056448 CPRD2 {Vigna unguiculata} | 0.855606 | 11.7806 |
| AT3G06460 | | GNS1/SUR4 membrane protein family | 2.44868 | 38.6862 |
| AT4G15370 | | BARS1, PEN2, baruol synthase 1 | 1.33687 | 21.4482 |
| AT2G23630 | | sks16, SKU5 similar 16 | 1.47886 | 25.049 |
| AT4G37160 | | sks15, SKU5 similar 15 | 1.12338 | 19.2911 |
| AT1G50050 | | CAP (Cysteine-rich secretory proteins, Antigen 5, and Pathogenesis-related 1 protein) superfamily protein | 2.97636 | 51.757 |
| AT5G39080 | | HXXXD-type acyl-transferase family protein | 0.899641 | 16.4572 |
| AT1G16905 | | Curculin-like (mannose-binding) lectin family protein | 5.82876 | 111.883 |
| AT4G36250 | | ALDH3F1, aldehyde dehydrogenase 3F1 | 0.779615 | 16.1883 |
| AT2G43600 | | Chitinase family protein | 0.958385 | 21.001 |
| AT4G15215 | | ATPDR13, PDR13, pleiotropic drug resistance 13 | 0.239051 | 5.76345 |
| AT3G55646 | | unknown protein | 0.332468 | 14.2699 |
| AT5G24313 | | unknown protein | 46.1877 | 0 |
