# Supplementary material for: Re-analysis of RNA-seq transcriptome data reveals new aspects of gene activity in Arabidopsis root hairs
Source: Front Plant Sci. 2015 Jun 8;6:421. doi: 10.3389/fpls.2015.00421 (PMC4458573; doi:10.3389/fpls.2015.00421)
Supplement: Supplementary file 14 [file Table9.DOC]

**Table S9** Gene Ontology enrichment was assessed using GOBU (Lin et al., 2006) in the 5409 ,4150, and 1259 differentially expressed genes between root hairs(RH) and non-root hair tissues (NRH) ,respectively, with *elim* method (P<0.001).

| GOID | GO Name | P value (elim, 5409) | P value (elim,4150) | P value (elim1259) |
| --- | --- | --- | --- | --- |
| GO:0009651 | response to salt stress | 1.07E-18 | 7.18E-04 | 7.12E-22 |
| GO:0042254 | ribosome biogenesis | 4.31E-16 | 0.854253 | 2.60E-37 |
| GO:0006468 | protein phosphorylation | 2.02E-15 | 2.86E-17 | 0.516537 |
| GO:0009793 | embryo development ending in seed dormancy | 6.11E-13 | 1.71E-13 | 0.211346 |
| GO:0009414 | response to water deprivation | 3.01E-10 | 3.51E-07 | 0.004738 |
| GO:0009409 | response to cold | 4.14E-09 | 0.016469 | 2.97E-11 |
| GO:0007018 | microtubule-based movement | 3.07E-08 | 9.78E-10 | 0.834053 |
| GO:0046686 | response to cadmium ion | 9.50E-08 | 0.766025 | 7.70E-20 |
| GO:0010200 | response to chitin | 2.44E-07 | 3.58E-07 | 0.199753 |
| GO:0006499 | N-terminal protein myristoylation | 3.17E-07 | 2.49E-05 | 0.006877 |
| GO:0009737 | response to abscisic acid stimulus | 1.45E-06 | 1.14E-05 | 0.002401 |
| GO:0006979 | response to oxidative stress | 2.61E-06 | 0.010223 | 7.00E-06 |
| GO:0006633 | fatty acid biosynthetic process | 3.63E-06 | 0.0086 | 3.02E-05 |
| GO:0009733 | response to auxin stimulus | 5.44E-06 | 0.003142 | 2.43E-04 |
| GO:0000910 | cytokinesis | 1.10E-05 | 5.12E-07 | 0.257641 |
| GO:0048768 | root hair cell tip growth | 1.59E-05 | 0.029621 | 1.61E-04 |
| GO:0000914 | phragmoplast assembly | 1.74E-05 | 3.54E-06 | 1 |
| GO:0080167 | response to karrikin | 1.83E-05 | 0.001964 | 0.0034 |
| GO:0009664 | plant-type cell wall organization | 1.91E-05 | 0.172025 | 2.50E-09 |
| GO:0010583 | response to cyclopentenone | 2.34E-05 | 4.90E-04 | 0.037279 |
| GO:0048364 | root development | 2.42E-05 | 4.12E-04 | 0.012817 |
| GO:0035556 | intracellular signal transduction | 2.45E-05 | 2.79E-07 | 0.716566 |
| GO:0006260 | DNA replication | 2.59E-05 | 3.04E-08 | 1 |
| GO:0016998 | cell wall macromolecule catabolic process | 3.92E-05 | 7.25E-04 | 0.047541 |
| GO:0006810 | transport | 3.98E-05 | 4.74E-07 | 0.186726 |
| GO:0009067 | aspartate family amino acid biosynthetic process | 5.21E-05 | 4.43E-04 | 0.076505 |
| GO:0007169 | transmembrane receptor protein tyrosine kinase signaling pathway | 5.28E-05 | 5.35E-06 | 0.722176 |
| GO:0009826 | unidimensional cell growth | 5.50E-05 | 0.002197 | 8.50E-04 |
| GO:0007020 | microtubule nucleation | 1.08E-04 | 2.87E-05 | 1 |
| GO:0051865 | protein autoubiquitination | 1.08E-04 | 2.87E-05 | 1 |
| GO:0080170 | hydrogen peroxide transmembrane transport | 1.08E-04 | 0.118219 | 4.96E-04 |
| GO:0009934 | regulation of meristem structural organization | 1.44E-04 | 1.13E-04 | 0.497199 |
| GO:0015672 | monovalent inorganic cation transport | 1.74E-04 | 0.001209 | 0.006607 |
| GO:0009755 | hormone-mediated signaling pathway | 2.02E-04 | 0.007326 | 0.007521 |
| GO:0006833 | water transport | 2.13E-04 | 0.004217 | 0.051695 |
| GO:0006268 | DNA unwinding involved in replication | 2.13E-04 | 3.72E-05 | 1 |
| GO:0007264 | small GTPase mediated signal transduction | 2.48E-04 | 0.015762 | 0.003732 |
| GO:0006306 | DNA methylation | 2.51E-04 | 1.13E-06 | 0.737438 |
| GO:0015986 | ATP synthesis coupled proton transport | 2.70E-04 | 0.005817 | 0.023748 |
| GO:0010311 | lateral root formation | 2.78E-04 | 0.038815 | 0.002487 |
| GO:0005983 | starch catabolic process | 2.78E-04 | 2.75E-04 | 0.457272 |
| GO:0034637 | cellular carbohydrate biosynthetic process | 2.94E-04 | 8.90E-04 | 0.06778 |
| GO:0008283 | cell proliferation | 3.09E-04 | 0.00111 | 0.138454 |
| GO:0048527 | lateral root development | 3.74E-04 | 0.025593 | 7.52E-05 |
| GO:0051726 | regulation of cell cycle | 4.01E-04 | 1.07E-04 | 0.940454 |
| GO:0007059 | chromosome segregation | 4.78E-04 | 0.017501 | 1 |
| GO:0048767 | root hair elongation | 4.78E-04 | 0.001199 | 0.017312 |
| GO:0009407 | toxin catabolic process | 4.98E-04 | 0.338886 | 6.27E-06 |
| GO:0016567 | protein ubiquitination | 5.29E-04 | 0.002852 | 0.107283 |
| GO:0046785 | microtubule polymerization | 5.61E-04 | 0.002833 | 0.204787 |
| GO:0006996 | organelle organization | 5.84E-04 | 6.02E-04 | 0.509669 |
| GO:0009968 | negative regulation of signal transduction | 6.46E-04 | 0.006185 | 0.057203 |
| GO:0006917 | induction of apoptosis | 6.71E-04 | 2.32E-04 | 1 |
| GO:0031507 | heterochromatin formation | 6.71E-04 | 0.006834 | 0.141663 |
| GO:0006520 | cellular amino acid metabolic process | 6.76E-04 | 0.037168 | 0.002356 |
| GO:0009693 | ethylene biosynthetic process | 7.15E-04 | 0.001292 | 0.282606 |
| GO:0009631 | cold acclimation | 7.30E-04 | 0.002459 | 0.184924 |
| GO:0033554 | cellular response to stress | 8.38E-04 | 0.046896 | 0.190593 |
| GO:0030422 | production of siRNA involved in RNA interference | 9.11E-04 | 4.56E-04 | 0.615206 |
| GO:0009749 | response to glucose stimulus | 9.11E-04 | 0.027933 | 0.013235 |
| GO:0006418 | tRNA aminoacylation for protein translation | 9.27E-04 | 6.76E-04 | 0.441771 |
| GO:0010639 | negative regulation of organelle organization | 9.36E-04 | 0.002346 | 0.290882 |
| GO:0006270 | DNA-dependent DNA replication initiation | 0.00104 | 1.96E-04 | 1 |
| GO:0000724 | double-strand break repair via homologous recombination | 0.001699 | 2.75E-04 | 1 |
| GO:0009605 | response to external stimulus | 0.074526 | 3.02E-04 | 0.557389 |
| GO:0045087 | innate immune response | 0.001724 | 3.14E-04 | 0.633027 |
| GO:0006400 | tRNA modification | 0.001941 | 3.79E-04 | 1 |
| GO:0010332 | response to gamma radiation | 0.00202 | 4.76E-04 | 1 |
| GO:0006310 | DNA recombination | 0.008279 | 5.89E-04 | 1 |
| GO:0000271 | polysaccharide biosynthetic process | 0.002384 | 5.94E-04 | 0.417673 |
| GO:0009555 | pollen development | 0.001552 | 6.93E-04 | 0.519888 |
| GO:0006865 | amino acid transport | 0.003429 | 7.16E-04 | 0.783034 |
| GO:0006915 | apoptosis | 0.014964 | 8.27E-04 | 0.977953 |
| GO:0010948 | negative regulation of cell cycle process | 0.003841 | 9.38E-04 | 1 |
| GO:0000279 | M phase | 0.032277 | 9.38E-04 | 0.832618 |
| GO:0006412 | translation | 0.00124 | 1 | 5.66E-59 |
| GO:0048765 | root hair cell differentiation | 0.003345 | 0.726658 | 2.04E-06 |
| GO:0006334 | nucleosome assembly | 0.009884 | 0.60443 | 6.21E-05 |
| GO:0080147 | root hair cell development | 0.007646 | 0.546611 | 9.64E-04 |
