# Supplementary material for: Re-analysis of RNA-seq transcriptome data reveals new aspects of gene activity in Arabidopsis root hairs
Source: Front Plant Sci. 2015 Jun 8;6:421. doi: 10.3389/fpls.2015.00421 (PMC4458573; doi:10.3389/fpls.2015.00421)
Supplement: Supplementary file 15 [file Table10.DOC]

**Table S10** Gene Ontology enrichment was assessed using GOBU (Lin et al., 2006) in the 100 most up-regulated and 61 genes only expressed in root hairs(RH), respectively, with *elim* method (P<0.01).

| GOID | GO Name | P value (elim,100) | P value (elim,61) |
| --- | --- | --- | --- |
| GO:0009664 | plant-type cell wall organization | 5.02E-13 | 0.13386 |
| GO:0048765 | root hair cell differentiation | 3.45E-07 | 1 |
| GO:0010054 | trichoblast differentiation | 1.31E-04 | 1 |
| GO:0006979 | response to oxidative stress | 1.89E-04 | 1 |
| GO:0009826 | unidimensional cell growth | 2.70E-04 | 0.31176 |
| GO:0006468 | protein phosphorylation | 4.64E-04 | 0.02684 |
| GO:0048768 | root hair cell tip growth | 8.98E-04 | 1 |
| GO:0009932 | cell tip growth | 0.002188 | 0.16789 |
| GO:0009831 | plant-type cell wall modification involved in multidimensional cell growth | 0.00272 | 1 |
| GO:0048767 | root hair elongation | 0.002932 | 1 |
| GO:0055114 | oxidation-reduction process | 0.004552 | 0.47221 |
| GO:0009828 | plant-type cell wall loosening | 0.004894 | 1 |
| GO:0000904 | cell morphogenesis involved in differentiation | 0.008199 | 0.24318 |
| GO:0031110 | regulation of microtubule polymerization or depolymerization | 0.008902 | 1 |
| GO:0035556 | intracellular signal transduction | 1 | 0.00821 |
