# Supplementary material for: Re-analysis of RNA-seq transcriptome data reveals new aspects of gene activity in Arabidopsis root hairs
Source: Front Plant Sci. 2015 Jun 8;6:421. doi: 10.3389/fpls.2015.00421 (PMC4458573; doi:10.3389/fpls.2015.00421)
Supplement: Supplementary file 17 [file Table12.DOC]

**Table S12** Distribution of RHE motif in the promoter regions of the differentially expressed genes between root hairs and non-root tissues.

| **AGI** | **Annotation** | **Matching Positions** | | **Hit pattern(5′to 3′)** | **RH(RPKM)** | **NRH(RPKM)** | **Fold_change(log2)** |
| --- | --- | --- | --- | --- | --- | --- | --- |
|  |  | **Start** | **End** |  |  |  |  |
| AT1G03850.1-1 | Glutaredoxin family protein | 578 | 562 | GACGTGAAAAACATATT | 448.24 | 51.99 | -3.11 |
| AT1G02970 | ATWEE1, WEE1, WEE1 kinase homolog | 1940 | 1924 | TACGTGAGAAGCATGGT | 0.87 | 3.51 | 2.01 |
| AT1G04520 | PDLP2, plasmodesmata-located protein 2 | 2903 | 2919 | AACTTGATCCTCACGAA | 2.31 | 9.47 | 2.03 |
| AT1G07030 | Mitochondrial substrate carrier family protein | 122 | 138 | AAAGTGTAGAGCACGAT | 109.43 | 34.16 | -1.68 |
| AT1G07040 | unknown protein | 2640 | 2656 | AAAGTGTAGAGCACGAT | 55.67 | 16.44 | -1.76 |
| AT1G08190 | ATVAM2, ATVPS41, VAM2, VPS41, ZIP2, vacuolar protein sorting 41 | 1136 | 1152 | TCTTTGGAGCTCACGTC | 93.78 | 31.94 | -1.55 |
| AT1G09690 | Translation protein SH3-like family protein | 637 | 653 | TTCTTGCTGTTCACGAA | 51.32 | 180.01 | 1.81 |
| AT1G09940 | HEMA2, Glutamyl-tRNA reductase family protein | 664 | 680 | TTCTTGTAGTGCACGAT | 333.24 | 126.41 | -1.4 |
| AT1G10550 | XET, XTH33, xyloglucan:xyloglucosyl transferase 33 | 153 | 137 | GTCGTGAAAATCATATA | 2.14 | 0.29 | -2.87 |
| AT1G11020 | RING/FYVE/PHD zinc finger superfamily protein | 1219 | 1203 | AACGTGAGAGCCAAGAA | 48.41 | 20.5 | -1.24 |
| AT1G12550 | D-isomer specific 2-hydroxyacid dehydrogenase family protein | 446 | 462 | ATAGTGATCCGCACGTA | 81.96 | 1.04 | -6.31 |
| AT1G13380 | Protein of unknown function (DUF1218) | 1891 | 1907 | TTAGTGATTTGCACGAC | 4.19 | 17.15 | 2.03 |
| AT1G14720 | ATXTH28, EXGT-A2, XTH28, XTR2, xyloglucan endotransglucosylase/hydrolase 28 | 1147 | 1131 | GACGTGATAATCATAAA | 1.42 | 5.32 | 1.91 |
| AT1G14960 | Polyketide cyclase/dehydrase and lipid transport superfamily protein | 634 | 618 | AACGTGAGGAACACTAA | 122.49 | 8.48 | -3.85 |
| AT1G18450 | ARP4, ATARP4, actin-related protein 4 | 154 | 138 | ATCGTGCCTCTCAAAAA | 11.11 | 28.57 | 1.36 |
| AT1G18460 | alpha/beta-Hydrolases superfamily protein | 2170 | 2154 | AACGTGAACACCATGGA | 142.28 | 60.44 | -1.24 |
| AT1G18470 | Transmembrane Fragile-X-F-associated protein | 831 | 815 | AACGTGAAACACATGTT | 104.71 | 48.45 | -1.11 |
| AT1G18910 | zinc ion binding;zinc ion binding | 1642 | 1658 | AAATTGCTTAGCACGAC | 51.67 | 20.11 | -1.36 |
| AT1G22060 | BEST Arabidopsis thaliana protein match is: FBD, F-box and Leucine Rich Repeat domains containing protein (TAIR:AT1G22000) | 2371 | 2355 | TTCGTGCATGCCACAAA | 1.62 | 7.38 | 2.19 |
| AT1G25400 | unknown protein | 211 | 227 | AATTTGGAAGTCACGTT | 406.95 | 87.85 | -2.21 |
| AT1G30860 | RING/U-box superfamily protein | 1518 | 1534 | AAAATGGTAAGCACGTT | 6.42 | 0.88 | -2.86 |
| AT1G30960 | GTP-binding family protein | 639 | 655 | AACTTGCCTTGCACGTA | 1.06 | 5.73 | 2.44 |
| AT1G32380 | PRS2, phosphoribosyl pyrophosphate (PRPP) synthase 2 | 1520 | 1536 | TAAATGGTTGTCACGTT | 2.55 | 7.87 | 1.63 |
| AT1G33750 | Terpenoid cyclases/Protein prenyltransferases superfamily protein | 1949 | 1965 | TTTTTGGTCAGCACGAT | 3.13 | 28.01 | 3.16 |
| AT1G34540 | CYP94D1, cytochrome P450, family 94, subfamily D, polypeptide 1 | 1007 | 1023 | AATATGAATCTCACGTT | 25.91 | 0 | #NAME? |
| AT1G48650 | DEA(D/H)-box RNA helicase family protein | 2771 | 2787 | ACCATGGCAGTCACGTT | 2.65 | 9.9 | 1.9 |
| AT1G50720 | Stigma-specific Stig1 family protein | 2065 | 2049 | TTCGTGATGTTCAAGGT | 1.9 | 12.21 | 2.69 |
| AT1G51745 | Tudor/PWWP/MBT superfamily protein | 1081 | 1097 | AACATGGACCTCACGTT | 2.56 | 7.36 | 1.53 |
| AT1G56045 | Ribosomal protein L41 family | 2572 | 2556 | TTCGTGATGTCCATAAA | 186.75 | 510.41 | 1.45 |
| AT1G59590 | ZCF37, ZCF37 | 1846 | 1862 | TTCATGGCATTCACGAA | 63.66 | 28.6 | -1.15 |
| AT1G60080 | 3'-5'-exoribonuclease family protein | 962 | 946 | TTCGTGATAATCAAATA | 2.37 | 9.95 | 2.07 |
| AT1G61100 | disease resistance protein (TIR class), putative | 1980 | 1996 | AAATTGGGATGCACGAA | 82.96 | 37.83 | -1.13 |
| AT1G62320 | ERD (early-responsive to dehydration stress) family protein | 1110 | 1094 | TTCGTGAGATCCAATGA | 53.82 | 2.38 | -4.5 |
| AT1G62422 | unknown protein | 1636 | 1652 | ACATTGAACTTCACGAA | 12.48 | 4.67 | -1.42 |
| AT1G62975 | basic helix-loop-helix (bHLH) DNA-binding superfamily protein | 792 | 808 | ATAGTGTTTCTCACGTT | 36.34 | 6.26 | -2.54 |
| AT1G63980 | D111/G-patch domain-containing protein | 28 | 44 | TATTTGCCTTGCACGAT | 12.17 | 50.23 | 2.05 |
| AT1G65900 | unknown protein | 2641 | 2625 | GACGTGAGAAACAAGGA | 0.26 | 2.73 | 3.41 |
| AT1G72880 | Survival protein SurE-like phosphatase/nucleotidase | 620 | 636 | AAAGTGGACAGCACGAA | 83.74 | 21.8 | -1.94 |
| AT1G73230 | Nascent polypeptide-associated complex NAC | 2505 | 2489 | ATCGTGATTTCCAAAGT | 63.26 | 173.21 | 1.45 |
| AT1G74770 | zinc ion binding | 2751 | 2767 | AATGTGCTTTGCACGAA | 16.9 | 5.16 | -1.71 |
| AT1G75840 | ARAC5, ATGP3, ATROP4, ROP4, RAC-like GTP binding protein 5 | 1411 | 1427 | ATATTGTAAGTCACGTT | 85.62 | 13.66 | -2.65 |
| AT1G77120 | ADH, ADH1, ATADH, ATADH1, alcohol dehydrogenase 1 | 218 | 234 | TTAGTGTACCTCACGTA | 594.09 | 164.01 | -1.86 |
| AT1G77130 | GUX3, PGSIP2, plant glycogenin-like starch initiation protein 2 | 1259 | 1243 | TACGTGAGGTACACTAA | 52.5 | 23.63 | -1.15 |
| AT1G79320 | AtMC6, MC6, metacaspase 6 | 287 | 271 | GACGTGCTAAGCATTTA | 29.44 | 5.23 | -2.49 |
| AT1G80240 | Protein of unknown function, DUF642 | 1825 | 1809 | TACGTGATTTTCAAAAA | 68.28 | 27.92 | -1.29 |
| AT2G03780 | Translin family protein | 1824 | 1808 | TACGTGCTCTTCAAGTA | 1.32 | 6.58 | 2.32 |
| AT2G05940 | Protein kinase superfamily protein | 570 | 586 | TATATGTAAATCACGTC | 66.04 | 18.99 | -1.8 |
| AT2G06010 | ORG4, OBP3-responsive gene 4 | 2753 | 2737 | AACGTGCAATACAATAT | 1.11 | 6.31 | 2.51 |
| AT2G16760 | Calcium-dependent phosphotriesterase superfamily protein | 204 | 220 | ATTGTGTCCGGCACGTT | 17.53 | 1.5 | -3.54 |
| AT2G19385 | zinc ion binding | 133 | 117 | TTCGTGAGAGACACATT | 4.74 | 19.18 | 2.02 |
| AT2G19600 | ATKEA4, KEA4, KEA4, K+ efflux antiporter 4 | 854 | 870 | TCTGTGACTCGCACGAT | 4.36 | 10.57 | 1.28 |
| AT2G23240 | Plant EC metallothionein-like protein, family 15 | 1113 | 1097 | ATCGTGAACTGCATTAT | 1.14 | 0 | #NAME? |
| AT2G23340 | DEAR3, DREB and EAR motif protein 3 | 374 | 358 | GACGTGACATCCAAAAT | 58.02 | 9.1 | -2.67 |
| AT2G26440 | Plant invertase/pectin methylesterase inhibitor superfamily | 2801 | 2817 | AACGTGGTAATCACGTC | 4.43 | 0.83 | -2.41 |
| AT2G28450 | zinc finger (CCCH-type) family protein | 2003 | 2019 | ATTTTGAAATGCACGAC | 2.67 | 10.88 | 2.03 |
| AT2G30395 | ATOFP17, OFP17, ovate family protein 17 | 2383 | 2399 | TATTTGAAACTCACGTT | 42.92 | 5.57 | -2.95 |
| AT2G31350 | GLX2-5, glyoxalase 2-5 | 1950 | 1966 | ACTATGTGGATCACGTT | 163.77 | 15.16 | -3.43 |
| AT2G33320 | Calcium-dependent lipid-binding (CaLB domain) family protein | 2495 | 2479 | AACGTGAAAAACATAGA | 9.96 | 3.39 | -1.56 |
| AT2G33430 | DAL, DAL1, differentiation and greening-like 1 | 1039 | 1023 | ATCGTGATGGACATGAA | 3.37 | 10.5 | 1.64 |
| AT2G33460 | RIC1, ROP-interactive CRIB motif-containing protein 1 | 2794 | 2810 | TCATTGTTGCTCACGAA | 58.36 | 0.29 | -7.63 |
| AT2G34260 | transducin family protein / WD-40 repeat family protein | 162 | 178 | TCCTTGTAGATCACGTA | 2.63 | 10.96 | 2.06 |
| AT2G34585 | unknown protein | 18 | 34 | TCTTTGATCAGCACGAA | 36.97 | 9.99 | -1.89 |
| AT2G37220 | RNA-binding (RRM/RBD/RNP motifs) family protein | 1842 | 1826 | TACGTGATTCACATGGT | 8.69 | 21.7 | 1.32 |
| AT2G38140 | PSRP4, plastid-specific ribosomal protein 4 | 2047 | 2031 | AACGTGCAACACAATTA | 4.72 | 16.89 | 1.84 |
| AT2G38160 | unknown protein | 2769 | 2785 | TAATTGTGTTGCACGTT | 0.43 | 4.02 | 3.22 |
| AT2G39300 | unknown protein | 2796 | 2780 | AACGTGCGCCGCAAATT | 2.43 | 6.9 | 1.51 |
| AT2G42570 | TBL39, TRICHOME BIREFRINGENCE-LIKE 39 | 1048 | 1064 | TCCGTGTTTCTCACGTT | 40.36 | 116.92 | 1.53 |
| AT2G44340 | VQ motif-containing protein | 2487 | 2503 | ATCATGATTTTCACGTT | 13.33 | 0 | #NAME? |
| AT2G45450 | ZPR1, protein binding | 2445 | 2461 | ATTATGCAAGGCACGTT | 4.3 | 15.34 | 1.83 |
| AT2G45670 | calcineurin B subunit-related | 1721 | 1737 | AAAGTGTGGAGCACGAT | 187.44 | 65.38 | -1.52 |
| AT3G06030 | ANP3, MAPKKK12, NP3, NPK1-related protein kinase 3 | 2466 | 2450 | TTCGTGATTTTCATTTA | 4.09 | 11.27 | 1.46 |
| AT3G07870 | F-box and associated interaction domains-containing protein | 1611 | 1627 | TTAGTGACCCTCACGAT | 56.29 | 9.88 | -2.51 |
| AT3G07890 | Ypt/Rab-GAP domain of gyp1p superfamily protein | 2299 | 2283 | ATCGTGAGGGTCACTAA | 119.61 | 44.12 | -1.44 |
| AT3G10440 | Shugoshin C terminus | 2909 | 2925 | ACAGTGGTGATCACGAC | 1.26 | 4.01 | 1.66 |
| AT3G10720 | Plant invertase/pectin methylesterase inhibitor superfamily | 2324 | 2308 | TACGTGCGAGGCACAAA | 65.41 | 215.82 | 1.72 |
| AT3G10985 | ATWI-12, SAG20, WI12, senescence associated gene 20 | 600 | 584 | AACGTGCGATGCACTGA | 688.29 | 160.94 | -2.1 |
| AT3G11390 | Cysteine/Histidine-rich C1 domain family protein | 2958 | 2974 | ATCATGCGTTGCACGTT | 3.77 | 0.39 | -3.26 |
| AT3G12502 | other RNA | 2307 | 2291 | TTCGTGCTTCACATAAA | 55.01 | 5.8 | -3.25 |
| AT3G13580 | Ribosomal protein L30/L7 family protein | 753 | 737 | GTCGTGAAGAGCAAAGT | 24.84 | 70.52 | 1.51 |
| AT3G13674 | unknown protein | 1116 | 1100 | TTCGTGATAGTCAAGAT | 3.57 | 13.42 | 1.91 |
| AT3G15450 | Aluminium induced protein with YGL and LRDR motifs | 2945 | 2961 | TTTGTGACTGTCACGTT | 3385.68 | 1127.62 | -1.59 |
| AT3G19920 | unknown protein | 160 | 144 | TACGTGAACATCAATGA | 7.31 | 1.22 | -2.59 |
| AT3G20150 | Kinesin motor family protein | 1171 | 1155 | GACGTGAAAGACACGGT | 1.26 | 5.75 | 2.19 |
| AT3G21340 | Leucine-rich repeat protein kinase family protein | 1641 | 1657 | TCTATGGAGTTCACGTT | 83.74 | 1.26 | -6.05 |
| AT3G22830 | AT-HSFA6B, HSFA6B, heat shock transcription factor A6B | 2329 | 2345 | TAAGTGGGAGTCACGTA | 338.88 | 37.26 | -3.18 |
| AT3G24160 | PMP, putative type 1 membrane protein | 2396 | 2412 | AATTTGAGAATCACGTA | 464.09 | 172.01 | -1.43 |
| AT3G24180 | Beta-glucosidase, GBA2 type family protein | 2849 | 2833 | TACGTGATTCTCAAATT | 176.93 | 62.07 | -1.51 |
| AT3G26744 | ATICE1, ICE1, SCRM, basic helix-loop-helix (bHLH) DNA-binding superfamily protein | 2461 | 2477 | TATATGTCGTTCACGTT | 2.62 | 10.28 | 1.97 |
| AT3G44050 | P-loop containing nucleoside triphosphate hydrolases superfamily protein | 1412 | 1396 | ATCGTGAAAGTCAAGAT | 0.53 | 6.79 | 3.69 |
| AT3G44900 | ATCHX4, CHX4, cation/H+ exchanger 4 | 2952 | 2936 | TTCGTGCTTGTCAAGTT | 5.17 | 0.94 | -2.46 |
|  |  | 2986 | 2970 | TACGTGACCGTCAAGTT | |  |  |
| AT3G45530 | Cysteine/Histidine-rich C1 domain family protein | 344 | 328 | AACGTGAAAACCAAAAA | 3.22 | 0.09 | -5.1 |
| AT3G46870 | Pentatricopeptide repeat (PPR) superfamily protein | 2765 | 2749 | AACGTGAACCGCAAGGT | 0.31 | 3.03 | 3.29 |
| AT3G53750 | ACT3, actin 3 | 188 | 204 | TTTTTGCAAATCACGTC | 5.42 | 18.57 | 1.78 |
| AT3G53760 | ATGCP4, GCP4, GAMMA-TUBULIN COMPLEX PROTEIN 4 | 25 | 9 | GACGTGATTTGCAAAAA | 1.82 | 7.17 | 1.98 |
| AT3G54010 | DEI1, PAS1, FKBP-type peptidyl-prolyl cis-trans isomerase family protein | 1735 | 1719 | ATCGTGAAAAGCAAGAA | 5.61 | 12.76 | 1.19 |
| AT3G54180 | CDC2B, CDKB1;1, cyclin-dependent kinase B1;1 | 2114 | 2098 | ATCGTGAGTATCATATT | 1.22 | 9.84 | 3.01 |
| AT3G54500 | BEST Arabidopsis thaliana protein match is: dentin sialophosphoprotein-related (TAIR:AT5G64170) | 662 | 678 | AATATGAAGAGCACGAA | 7.74 | 18.55 | 1.26 |
|  |  | 1580 | 1564 | GTCGTGCGAGCCAAGTA | |  |  |
| AT3G56000 | ATCSLA14, CSLA14, cellulose synthase like A14 | 377 | 393 | ATTTTGTAGGTCACGAA | 57.07 | 5.07 | -3.49 |
| AT3G56930 | DHHC-type zinc finger family protein | 626 | 610 | TTCGTGAGGAACAAGGA | 146.09 | 13.77 | -3.41 |
| AT3G56950 | SIP2, SIP2;1, small and basic intrinsic protein 2;1 | 1357 | 1373 | TCCTTGTTCCTCACGAA | 61.16 | 21.31 | -1.52 |
| AT3G58790 | GAUT15, galacturonosyltransferase 15 | 521 | 537 | TTCTTGTTATTCACGTT | 4.42 | 11.21 | 1.34 |
| AT3G58810 | ATMTP3, ATMTPA2, MTP3, MTPA2, metal tolerance protein A2 | 36 | 52 | TTCGTGTTCTTCACGAA | 10.43 | 1.27 | -3.04 |
| AT3G59650 | mitochondrial ribosomal protein L51/S25/CI-B8 family protein | 585 | 569 | AACGTGCTTTGCAAAAT | 6.98 | 25.92 | 1.89 |
| AT3G60690 | SAUR-like auxin-responsive protein family | 2409 | 2393 | TTCGTGCACTCCACATT | 75.33 | 27.15 | -1.47 |
| AT3G61410 | BEST Arabidopsis thaliana protein match is: U-box domain-containing protein kinase family protein (TAIR:AT2G45910) | 1471 | 1455 | AACGTGAGCATCAAAAT | 19.92 | 5.7 | -1.8 |
| AT3G61930 | unknown protein | 2679 | 2695 | ACCATGCAAAGCACGAA | 23.01 | 89.93 | 1.97 |
| AT3G62030 | ROC4, rotamase CYP 4 | 1896 | 1880 | ATCGTGAAGAACATATT | 2.42 | 10.59 | 2.13 |
| AT3G62720 | ATXT1, XT1, XXT1, xylosyltransferase 1 | 488 | 504 | TTTGTGGAATGCACGAC | 89.38 | 35.05 | -1.35 |
| AT4G00585 | unknown protein | 2526 | 2542 | TAAGTGGGCCTCACGTT | 267.34 | 120.27 | -1.15 |
| AT4G00620 | Amino acid dehydrogenase family protein | 2738 | 2722 | TACGTGAGGAACAAGAA | 1.82 | 5.93 | 1.71 |
| AT4G02230 | Ribosomal protein L19e family protein | 1842 | 1858 | TTCATGTTGATCACGAA | 32.08 | 86.61 | 1.43 |
| AT4G03100 | Rho GTPase activating protein with PAK-box/P21-Rho-binding domain | 2375 | 2359 | TTCGTGCAAAACATATA | 0.94 | 5.1 | 2.44 |
| AT4G03490 | Ankyrin repeat family protein | 950 | 966 | TTATTGTGAAGCACGTC | 7.47 | 0.99 | -2.92 |
| AT4G03510 | ATRMA1, RMA1, RING membrane-anchor 1 | 785 | 769 | TACGTGCCAGCCATAAA | 361.59 | 102.72 | -1.82 |
|  |  | 2777 | 2793 | AATTTGCTTAGCACGTA | |  |  |
| AT4G03560 | ATCCH1, ATTPC1, FOU2, TPC1, TPC1, two-pore channel 1 | 2618 | 2634 | TAAGTGTAGGTCACGTA | 322.19 | 119.99 | -1.43 |
| AT4G04695 | CPK31, calcium-dependent protein kinase 31 | 2308 | 2292 | ATCGTGAGTTACAAAAA | 10.02 | 1.9 | -2.4 |
| AT4G04760 | Major facilitator superfamily protein | 82 | 66 | TACGTGATTCACAAATA | 5.37 | 0.72 | -2.89 |
| AT4G04885 | PCFS4, PCF11P-similar protein 4 | 1634 | 1618 | ATCGTGACTCGCAAGGT | 2.48 | 6.68 | 1.43 |
| AT4G05330 | AGD13, ARF-GAP domain 13 | 1002 | 1018 | ATCTTGGCCTTCACGTT | 38.99 | 5.3 | -2.88 |
|  |  | 1230 | 1246 | ATCTTGGCTTTCACGTT | |  |  |
|  |  | 1458 | 1474 | ATCTTGGCTTTCACGTT | |  |  |
| AT4G10860 | unknown protein | 1036 | 1020 | GACGTGAGGCCCACTGA | 1.84 | 0 | #NAME? |
| AT4G14330 | P-loop containing nucleoside triphosphate hydrolases superfamily protein | 209 | 193 | AACGTGAACACCAAAAA | 0.26 | 3.51 | 3.77 |
| AT4G15360 | CYP705A3, cytochrome P450, family 705, subfamily A, polypeptide 3 | 1323 | 1339 | AAAATGTGAATCACGAT | 0.17 | 7.06 | 5.38 |
| AT4G15370 | BARS1, PEN2, baruol synthase 1 | 564 | 548 | ATCGTGATTCACATTTT | 1.34 | 21.45 | 4 |
| AT4G17180 | O-Glycosyl hydrolases family 17 protein | 2963 | 2979 | TTTATGGGATTCACGAT | 0.31 | 1.57 | 2.36 |
| AT4G19230 | CYP707A1, cytochrome P450, family 707, subfamily A, polypeptide 1 | 1376 | 1392 | TCCATGATTTTCACGTT | 163.14 | 38.52 | -2.08 |
| AT4G19960 | ATKUP9, HAK9, KT9, KUP9, K+ uptake permease 9 | 1169 | 1185 | ACCATGAAGAGCACGAA | 15.37 | 2.7 | -2.51 |
| AT4G20040 | Pectin lyase-like superfamily protein | 137 | 153 | TCCTTGGAAATCACGTT | 0.55 | 3.39 | 2.63 |
| AT4G21960 | PRXR1, Peroxidase superfamily protein | 329 | 313 | GACGTGAGAGACAATAA | 36.81 | 102.03 | 1.47 |
| AT4G23470 | PLAC8 family protein | 538 | 522 | TTCGTGCTCGACATGTA | 223.15 | 69.3 | -1.69 |
| AT4G23750 | CRF2, TMO3, cytokinin response factor 2 | 2860 | 2876 | TATATGTCTTTCACGTC | 2.37 | 8.59 | 1.86 |
| AT4G23950 | Galactose-binding protein | 1582 | 1566 | AACGTGAGGAACAAAGA | 0.33 | 1.83 | 2.48 |
| AT4G25940 | ENTH/ANTH/VHS superfamily protein | 357 | 341 | ATCGTGAACACCAAAGT | 19.6 | 0.25 | -6.31 |
| AT4G26050 | PIRL8, plant intracellular ras group-related LRR 8 | 2545 | 2529 | GTCGTGACCCACAAGAT | 13.53 | 2.6 | -2.38 |
| AT4G26110 | ATNAP1;1, NAP1;1, nucleosome assembly protein1;1 | 2692 | 2676 | TTCGTGAGTTTCAAATA | 15.33 | 52.13 | 1.77 |
| AT4G26120 | Ankyrin repeat family protein / BTB/POZ domain-containing protein | 84 | 68 | TTCGTGAGTTTCAAATA | 2.73 | 0.63 | -2.12 |
| AT4G26130 | unknown protein | 1408 | 1424 | TATTTGAAACTCACGAA | 8.89 | 3.17 | -1.49 |
| AT4G27410 | ANAC072, RD26, NAC (No Apical Meristem) domain transcriptional regulator superfamily protein | 988 | 1004 | TCTTTGATAGTCACGAT | 201.02 | 32.07 | -2.65 |
| AT4G28450 | nucleotide binding;protein binding | 2402 | 2386 | AACGTGAGAGTCACATT | 2.54 | 12.57 | 2.3 |
| AT4G29690 | Alkaline-phosphatase-like family protein | 446 | 462 | ATATTGATAATCACGTA | 22.63 | 77.99 | 1.78 |
| AT4G29740 | ATCKX4, CKX4, cytokinin oxidase 4 | 662 | 678 | TAAATGGTCTTCACGTA | 0.63 | 8.93 | 3.83 |
| AT4G32720 | AtLa1, La1, La protein 1 | 2251 | 2235 | TTCGTGCGCATCAAATT | 13.6 | 62.05 | 2.19 |
| AT4G35200 | Arabidopsis protein of unknown function (DUF241) | 1590 | 1574 | GTCGTGAATTCCAAGTT | 29.52 | 1.85 | -4 |
| AT4G36080 | phosphotransferases, alcohol group as acceptor;binding;inositol or phosphatidylinositol kinases | 96 | 80 | TTCGTGCGCAACAAGAT | 1.91 | 4.12 | 1.11 |
| AT4G36380 | ROT3, Cytochrome P450 superfamily protein | 134 | 150 | ACAATGCCATGCACGAA | 5.78 | 2.07 | -1.48 |
| AT4G36520 | Chaperone DnaJ-domain superfamily protein | 978 | 994 | TAATTGTATCTCACGAA | 35.83 | 12.06 | -1.57 |
| AT4G37070 | AtPLAIVA, PLA IVA, PLP1, Acyl transferase/acyl hydrolase/lysophospholipase superfamily protein | 213 | 229 | ATCATGATTAGCACGAA | 64.28 | 2.67 | -4.59 |
| AT4G37080 | Protein of unknown function, DUF547 | 1784 | 1768 | AACGTGAAGGCCAATAT | 0.33 | 3.63 | 3.48 |
| AT4G37950 | Rhamnogalacturonate lyase family protein | 561 | 577 | ATTATGATGATCACGAT | 1.36 | 0.15 | -3.16 |
| AT4G38390 | RHS17, root hair specific 17 | 2122 | 2106 | GACGTGAGCAGCATTAA | 36.63 | 0.26 | -7.14 |
| AT4G39675 | unknown protein | 2540 | 2556 | TTCTTGCTCAGCACGAA | 148.81 | 17.2 | -3.11 |
| AT5G01215 | other RNA | 2710 | 2726 | ACCATGTAAATCACGAA | 19.41 | 1.18 | -4.04 |
| AT5G01480 | Cysteine/Histidine-rich C1 domain family protein | 2905 | 2889 | GACGTGAGTGACAAGGA | 2.9 | 0.69 | -2.07 |
| AT5G02960 | Ribosomal protein S12/S23 family protein | 2372 | 2388 | TACGTGTACGTCACGAA | 97.95 | 447.11 | 2.19 |
| AT5G04160 | Nucleotide-sugar transporter family protein | 566 | 550 | GACGTGCAAACCATTAT | 55.5 | 21.55 | -1.36 |
| AT5G05850 | PIRL1, plant intracellular ras group-related LRR 1 | 394 | 378 | AACGTGCAATACACATA | 46.59 | 20.97 | -1.15 |
| AT5G06150 | CYC1BAT, CYCB1;2, Cyclin family protein | 19 | 3 | ATCGTGAATCCCAATTA | 0.81 | 7.43 | 3.2 |
| AT5G06800 | myb-like HTH transcriptional regulator family protein | 1068 | 1052 | AACGTGACATACACTGT | 104.98 | 4.74 | -4.47 |
| AT5G07090 | Ribosomal protein S4 (RPS4A) family protein | 1987 | 1971 | ATCGTGAAAGCCATTTA | 87.35 | 278.03 | 1.67 |
| AT5G08650 | Small GTP-binding protein | 725 | 709 | GACGTGAGAAACAAAAT | 0.95 | 3.08 | 1.69 |
| AT5G11850 | Protein kinase superfamily protein | 735 | 751 | TTTTTGGGTTTCACGTA | 61.43 | 27.56 | -1.16 |
| AT5G12050 | unknown protein | 2512 | 2528 | TAAGTGAATAGCACGAT | 184.14 | 3.34 | -5.78 |
| AT5G12250 | TUB6, beta-6 tubulin | 827 | 843 | TAAGTGAATGGCACGAT | 23.21 | 65.46 | 1.5 |
| AT5G12320 | ankyrin repeat family protein | 1090 | 1106 | TTTTTGGCCTTCACGTT | 3.36 | 15.21 | 2.18 |
| AT5G18270 | ANAC087, Arabidopsis NAC domain containing protein 87 | 168 | 152 | TACGTGAATGTCATGTA | 85.11 | 28.6 | -1.57 |
|  |  | 260 | 276 | TAAATGGTTCTCACGAA | |  |  |
| AT5G19550 | AAT2, ASP2, aspartate aminotransferase 2 | 1070 | 1086 | AACATGGACCGCACGTT | 385.2 | 131.4 | -1.55 |
| AT5G21050 | BEST Arabidopsis thaliana protein match is: unknown protein (TAIR:AT5G64090); | 648 | 632 | AACGTGAAATTCAAAAT | 6.82 | 1.37 | -2.31 |
| AT5G22555 | unknown protein | 1421 | 1405 | GTCGTGCCTTGCACGTT | 196.53 | 0.86 | -7.84 |
| AT5G23903 | unknown protein | 910 | 926 | AAAATGCTACTCACGAT | 1.07 | 0 | #NAME? |
| AT5G24470 | APRR5, PRR5, pseudo-response regulator 5 | 1399 | 1415 | ACAATGAATCTCACGTT | 35.19 | 12.99 | -1.44 |
| AT5G27920 | F-box family protein | 816 | 832 | ATCATGGATCTCACGAT | 71.77 | 23.8 | -1.59 |
| AT5G28640 | AN3, ATGIF1, GIF, GIF1, SSXT family protein | 802 | 818 | AAAGTGGTTTTCACGTT | 2.99 | 8.95 | 1.58 |
| AT5G35580 | Protein kinase superfamily protein | 1752 | 1736 | AACGTGAGGAACAATGT | 22.58 | 3.18 | -2.83 |
| AT5G37660 | PDLP7, plasmodesmata-located protein 7 | 2273 | 2257 | AACGTGCACTCCATGTT | 0.37 | 3.59 | 3.28 |
| AT5G40870 | ATUK/UPRT1, UK/UPRT1, UKL1, uridine kinase/uracil phosphoribosyltransferase 1 | 2333 | 2349 | TCTGTGGAATGCACGAT | 1.99 | 6.95 | 1.8 |
| AT5G43175 | basic helix-loop-helix (bHLH) DNA-binding superfamily protein | 650 | 666 | TTAATGAAATGCACGAT | 14.61 | 0 | #NAME? |
| AT5G43180 | Protein of unknown function, DUF599 | 446 | 430 | ATCGTGCATTTCATTAA | 9.97 | 1.9 | -2.39 |
| AT5G46510 | Disease resistance protein (TIR-NBS-LRR class) family | 211 | 227 | TTTATGTCAAGCACGAT | 0.38 | 1.51 | 2 |
| AT5G46890 | Bifunctional inhibitor/lipid-transfer protein/seed storage 2S albumin superfamily protein | 1702 | 1686 | GTCGTGATCCTCACGGT | 202.88 | 70.65 | -1.52 |
| AT5G47980 | HXXXD-type acyl-transferase family protein | 1713 | 1729 | ACTTTGGTGCTCACGTT | 23.98 | 4.01 | -2.58 |
| AT5G51290 | Diacylglycerol kinase family protein | 1929 | 1945 | ATCATGGGAAGCACGAA | 20.16 | 8.44 | -1.26 |
| AT5G51600 | ATMAP65-3, MAP65-3, PLE, Microtubule associated protein (MAP65/ASE1) family protein | 1899 | 1883 | GTCGTGATCATCAAGAT | 0.41 | 4.66 | 3.51 |
| AT5G52840 | NADH-ubiquinone oxidoreductase-related | 2392 | 2376 | TACGTGCATTCCATATA | 189.27 | 89.79 | -1.08 |
| AT5G52900 | unknown protein | 194 | 178 | TTCGTGATCTACATATT | 3.78 | 0.59 | -2.68 |
| AT5G52910 | ATIM, timeless family protein | 1781 | 1797 | TATGTGAATTTCACGAA | 0.21 | 2.61 | 3.61 |
| AT5G53770 | Nucleotidyltransferase family protein | 2167 | 2151 | TTCGTGCTTTCCAAATT | 3.85 | 11.09 | 1.53 |
| AT5G57510 | unknown protein | 1458 | 1474 | ACTTTGAACCTCACGTT | 258.13 | 61.11 | -2.08 |
| AT5G59240 | Ribosomal protein S8e family protein | 1840 | 1856 | AAAATGCTTATCACGAC | 1.17 | 8.51 | 2.87 |
| AT5G59850 | Ribosomal protein S8 family protein | 1561 | 1577 | ATATTGACTTGCACGTC | 45.13 | 226.55 | 2.33 |
|  |  | 1937 | 1953 | ACAATGTCGTTCACGTT | |  |  |
| AT5G60530 | late embryogenesis abundant protein-related / LEA protein-related | 1804 | 1788 | AACGTGAGAGTCACGGT | 97.83 | 309.05 | 1.66 |
| AT5G61330 | rRNA processing protein-related | 395 | 411 | TACTTGAGCGTCACGAT | 1.8 | 9.21 | 2.35 |
| AT5G65100 | Ethylene insensitive 3 family protein | 2693 | 2709 | TTAATGGGTTTCACGTC | 26.58 | 0.66 | -5.34 |
| AT5G65800 | ACS5, ATACS5, CIN5, ETO2, ACC synthase 5 | 1953 | 1969 | ACCATGTGGGTCACGTT | 17.15 | 1.25 | -3.78 |
| AT5G66610 | DAR7, DA1-related protein 7 | 2494 | 2478 | GTCGTGCTTGTCACAAA | 0.8 | 2.81 | 1.82 |
| AT5G66820 | unknown protein | 120 | 136 | ATAATGAATGTCACGTA | 13.58 | 5.02 | -1.44 |
