# Supplementary material for: Re-analysis of RNA-seq transcriptome data reveals new aspects of gene activity in Arabidopsis root hairs
Source: Front Plant Sci. 2015 Jun 8;6:421. doi: 10.3389/fpls.2015.00421 (PMC4458573; doi:10.3389/fpls.2015.00421)
Supplement: Supplementary file 18 [file Table13.DOC]

**Table S13** Distribution of RHE motif in the intron regions of the differentially expressed genes between root hairs and non-root tissues.

| AGI | Annotation | Matching Positions  Start End | | | Hit pattern(5′to 3′) | RH(RPKM) | | NRH(RPKM) | Fold_change(log2) |  |
| --- | --- | --- | --- | --- | --- | --- | --- | --- | --- | --- |
| AT1G03850.1-1 | Glutaredoxin family protein | 578 | 562 | GACGTGAAAAACATATT | | | 448.24 | 51.99 | -3.11 | |
| AT1G08320.1-10 | bZIP21, | 71 | 87 | AAAATGATATTCACGTT | | | 11.69 | 0.68 | -4.11 | |
| AT1G08580.1-2 | unknown protein | 268 | 284 | TTTGTGTATCTCACGAA | | | 6.03 | 31.55 | 2.39 | |
| AT1G20410.1-12 | Pseudouridine synthase family protein | 31 | 15 | ATCGTGAGTTTCACATT | | | 2.29 | 7.06 | 1.63 | |
| AT1G23020.1-7 | ATFRO3 | 59 | 43 | TTCGTGCAACACAAGTA | | | 21.17 | 3.19 | -2.73 | |
| AT1G32440.1-1 | PKp3, plastidial pyruvate kinase 3 | 53 | 69 | TTCGTGTTAGTCACGTT | | | 4.46 | 14.77 | 1.73 | |
| AT1G52240.1-5 | ATROPGEF11 | 429 | 413 | AACGTGCAGCCCACATT | | | 222.34 | 25.32 | -3.13 | |
| AT1G56050.1-10 | GTP-binding protein-related | 26 | 10 | TTCGTGATGTCCATAAA | | | 0.62 | 5.23 | 3.07 | |
| AT1G62440.1-2 | LRX2 | 499 | 483 | GACGTGCTTATCAATAT | | | 90.81 | 17.19 | -2.4 | |
| AT1G62660.1-1 | Glycosyl hydrolases family 32 protein | 316 | 300 | GTCGTGAATGGCAAATT | | | 426.59 | 80.58 | -2.4 | |
| AT1G62850.2-5 | Class I peptide chain release factor | 6 | 22 | ATATTGAATATCACGTC | | | 110.32 | 14.23 | -2.96 | |
| AT1G66530.1-10 | Arginyl-tRNA synthetase, class Ic | 21 | 37 | TCATTGTTTTTCACGTC | | | 3.99 | 13.63 | 1.77 | |
| AT1G80070.1-15 | EMB14, EMB177, EMB33, SUS2, Pre-mRNA-processing-splicing factor | 134 | 118 | TTCGTGCTTTACATGAA | | | 16.35 | 38.92 | 1.25 | |
| AT2G20290.1-19 | ATXIG, XIG, myosin-like protein XIG | 5 | 21 | TTTGTGAACGTCACGTT | | | 2.26 | 8.68 | 1.94 | |
| AT2G20330.1-2 | Transducin/WD40 repeat-like superfamily protein | 25 | 9 | TTCGTGAACAACACTAA | | | 74.85 | 34.96 | -1.1 | |
| AT2G24260.1-3 | LRL1, LJRHL1-like 1 | 140 | 124 | TTCGTGCGATCCATAAT | | | 64.17 | 8.81 | -2.86 | |
| AT2G27080.2-1 | Late embryogenesis abundant (LEA) hydroxyproline-rich glycoprotein family | 762 | 746 | AACGTGCACTCCACAAT | | | 149.09 | 28.67 | -2.38 | |
| AT2G28430.1-2 | unknown protein | 115 | 131 | ATTTTGAAATGCACGAC | | | 77.18 | 27.27 | -1.5 | |
| AT2G31350.1-1 | GLX2-5, glyoxalase 2-5 | 161 | 145 | TACGTGATGATCATTTT | | | 163.77 | 15.16 | -3.43 | |
| AT2G38320.1-2 | TBL34, TRICHOME BIREFRINGENCE-LIKE 34 | 257 | 241 | AACGTGATCTCCAATAA | | | 13.03 | 1.9 | -2.78 | |
| AT2G38840.1-2 | Guanylate-binding family protein | 88 | 104 | ACTTTGTTTCTCACGAT | | | 53.75 | 25.97 | -1.05 | |
| AT2G40890.1-2 | CYP98A3, cytochrome P450, family 98, subfamily A, polypeptide 3 | 49 | 65 | TTCTTGTTTCTCACGTT | | | 53.11 | 17.39 | -1.61 | |
| AT3G19050.1-19 | POK2, phragmoplast orienting kinesin 2 | 25 | 41 | TTCTTGTGCATCACGTA | | | 0.47 | 4.6 | 3.29 | |
| AT3G21630.1-4 | CERK1, LYSM RLK1, chitin elicitor receptor kinase 1 | 22 | 6 | TTCGTGATGATCATATA | | | 88.24 | 38.45 | -1.2 | |
| AT3G45130.1-1 | LAS1, lanosterol synthase 1 | 634 | 650 | AATGTGAATATCACGTA | | | 0.82 | 5.9 | 2.84 | |
| AT3G46830.1-2 | ATRAB-A2C, ATRAB11A, ATRABA2C, RAB-A2C, RABA2c, RAB GTPase homolog A2C | 674 | 658 | GTCGTGAAGGCCAATTA | | | 66.62 | 27.55 | -1.27 | |
| AT3G51800.1-8 | ATEBP1, ATG2, EBP1, metallopeptidase M24 family protein | 57 | 41 | GACGTGATCCACATGTT | | | 20.71 | 106.78 | 2.37 | |
| AT4G03110.1-2 | AtRBP-DR1, RBP-DR1, RNA-binding protein-defense related 1 | 240 | 256 | TATATGTTTTGCACGAA | | | 75.77 | 21.39 | -1.82 | |
| AT4G03500.1-1 | Ankyrin repeat family protein | 856 | 840 | TACGTGCTAAGCAAATT | | | 16.3 | 2.45 | -2.73 | |
| AT4G13420.1-3 | ATHAK5, HAK5, high affinity K+ transporter 5 | 368 | 384 | ATTATGTGACGCACGTC | | | 9.83 | 1.69 | -2.54 | |
| AT4G14370.1-4 | Disease resistance protein (TIR-NBS-LRR class) family | 99 | 83 | AACGTGATGATCATATA | | | 28.26 | 8.26 | -1.77 | |
| AT4G18780.1-7 | ATCESA8, CESA8, IRX1, LEW2, cellulose synthase family protein | 41 | 57 | TTCTTGTTAGTCACGTA | | | 3.73 | 0.9 | -2.05 | |
| AT4G37870.1-2 | PCK1, PEPCK, phosphoenolpyruvate carboxykinase 1 | 178 | 162 | GACGTGATATACACTAA | | | 131.22 | 301.6 | 1.2 | |
| AT5G04920.1-7 | EAP30/Vps36 family protein | 60 | 76 | TATATGATGCTCACGTT | | | 63.38 | 28.72 | -1.14 | |
| AT5G06570.1-1 | alpha/beta-Hydrolases superfamily protein | 1625 | 1609 | GTCGTGACTATCATTGT | | | 36.07 | 7.59 | -2.25 | |
| AT5G11480.1-1 | P-loop containing nucleoside triphosphate hydrolases superfamily protein | 36 | 20 | ATCGTGCTTATCATTTA | | | 1.55 | 4.62 | 1.58 | |
| AT5G20490.1-1 | ATXIK, XI-17, XIK, Myosin family protein with Dil domain | 188 | 204 | TCTTTGTACCTCACGTC | | | 42.38 | 19.68 | -1.11 | |
| AT5G27680.1-8 | RECQSIM, RECQ helicase SIM | 72 | 56 | TACGTGCTATTCAAATT | | | 0.18 | 2.15 | 3.59 | |
| AT5G41880.1-6 | POLA3, POLA4, DNA primases;DNA primases | 124 | 108 | AACGTGAATGTCATTGT | | | 0.65 | 5.22 | 3 | |
| AT5G51060.1-9 | ATRBOHC, RBOHC, RHD2, NADPH/respiratory burst oxidase protein D | 150 | 134 | ATCGTGATATCCACTTA | | | 217.37 | 44.05 | -2.3 | |
| AT5G52290.1-5 | SHOC1, shortage in chiasmata 1 | 215 | 231 | TCAGTGGTATTCACGTT | | | 1.76 | 0.64 | -1.46 | |
| AT5G65090.1-1 | BST1, DER4, MRH3, DNAse I-like superfamily protein | 75 | 59 | GACGTGAAACCCATTAA | | | 41.27 | 0.59 | -6.12 | |
| AT5G65210.5-1 | TGA1, bZIP transcription factor family protein | 78 | 94 | TACATGGTTTTCACGAC | | | 153.01 | 68.16 | -1.17 | |
