# Supplementary material for: Re-analysis of RNA-seq transcriptome data reveals new aspects of gene activity in Arabidopsis root hairs
Source: Front Plant Sci. 2015 Jun 8;6:421. doi: 10.3389/fpls.2015.00421 (PMC4458573; doi:10.3389/fpls.2015.00421)
Supplement: Supplementary file 19 [file Table14.DOC]

**Table S14** Distribution of RHE motif in the CDS regions of the differentially expressed genes between root hairs and non-root tissues.

| **Gene name** | **Annotation** | | **Matching Positions** | | | **Hit pattern(5′to 3′)** | | **RH(RPKM)** | | **NRH(RPKM)** | | **Fold_change(log2)** | |
| --- | --- | --- | --- | --- | --- | --- | --- | --- | --- | --- | --- | --- | --- |
|  |  |  | **Start End** | | |  |  |  |  |  |  |  |  |
| AT1G01960.1 | | EDA10, SEC7-like guanine nucleotide exchange family protein | | 3646 | 3662 | | TTCATGATTTTCACGAC | 11.72 | 30.02 | | 1.36 | |  |
| AT1G03830.1 | | guanylate-binding family protein | | 1237 | 1253 | | TCCTTGGAAAGCACGAT | 0.7 | 3.97 | | 2.51 | |  |
| AT1G05070.1 | | Protein of unknown function (DUF1068) | | 209 | 225 | | ATTGTGCAAAGCACGAT | 2.13 | 8.78 | | 2.04 | |  |
| AT1G15930.1 | | Ribosomal protein L7Ae/L30e/S12e/Gadd45 family protein | | 242 | 258 | | TCTGTGCTGATCACGAA | 109.08 | 240.56 | | 1.14 | |  |
| AT1G18460.1 | | alpha/beta-Hydrolases superfamily protein | | 1435 | 1451 | | AACATGTGTTTCACGTT | 142.28 | 60.44 | | -1.24 | |  |
| AT1G18470.1 | | Transmembrane Fragile-X-F-associated protein | | 466 | 482 | | TCCATGGTGTTCACGTT | 104.71 | 48.45 | | -1.11 | |  |
| AT1G22570.1 | | Major facilitator superfamily protein | | 1009 | 1025 | | TACGTGGTGAGCACGAT | 33.35 | 4.38 | | -2.93 | |  |
| AT1G26190.1 | | Phosphoribulokinase / Uridine kinase family | | 892 | 876 | | GTCGTGCCAATCATATT | 1.96 | 5.23 | | 1.41 | |  |
| AT1G29470.1 | | S-adenosyl-L-methionine-dependent methyltransferases superfamily protein | | 831 | 815 | | ATCGTGAGAGGCATTGT | 42.53 | 126.81 | | 1.58 | |  |
| AT1G30490.1 | | ATHB9, PHV, Homeobox-leucine zipper family protein / lipid-binding START domain-containing protein | | 959 | 943 | | TTCGTGAGAGCCAAAAT | 0.44 | 1.98 | | 2.18 | |  |
| AT1G48640.1 | | Transmembrane amino acid transporter family protein | | 170 | 154 | | AACGTGACTGCCATGGT | 42.9 | 1.15 | | -5.22 | |  |
| AT1G54890.1 | | Late embryogenesis abundant (LEA) protein-related | | 637 | 653 | | TCCGTGTTGGTCACGTT | 9.45 | 27.15 | | 1.52 | |  |
| AT1G59600.1 | | ZCW7, ZCW7 | | 788 | 772 | | TTCGTGAATGCCATGAA | 4 | 12.38 | | 1.63 | |  |
| AT1G61840.1 | | Cysteine/Histidine-rich C1 domain family protein | | 1466 | 1482 | | ATTATGTTGTTCACGTA | 4.19 | 0.26 | | -4.03 | |  |
| AT1G64590.1 | | NAD(P)-binding Rossmann-fold superfamily protein | | 650 | 634 | | AACGTGCTCCACACTGT | 6 | 1.21 | | -2.3 | |  |
| AT1G64790.1 | | ILA, ILITYHIA | | 1194 | 1210 | | TTCATGGGCATCACGAT | 5.41 | 18.31 | | 1.76 | |  |
| AT1G65890.1 | | AAE12, acyl activating enzyme 12 | | 1139 | 1123 | | GACGTGAGAAACAAGGA | 17.93 | 7.43 | | -1.27 | |  |
| AT1G66700.1 | | PXMT1, S-adenosyl-L-methionine-dependent methyltransferases superfamily protein | | 1058 | 1042 | | ATCGTGCTTCGCAAGAA | 4.69 | 21.96 | | 2.23 | |  |
| AT1G67070.1 | | DIN9, PMI2, Mannose-6-phosphate isomerase, type I | | 1241 | 1257 | | ATATTGAGATTCACGTA | 83.1 | 26.35 | | -1.66 | |  |
| AT1G68790.1 | | LINC3, little nuclei3 | | 1455 | 1439 | | TTCGTGAAGAACATGAA | 2.84 | 10.68 | | 1.91 | |  |
| AT1G72150.1 | | PATL1, PATELLIN 1 | | 782 | 766 | | GACGTGATCCTCACGAA | 106.6 | 48.45 | | -1.14 | |  |
| AT1G72870.1 | | Disease resistance protein (TIR-NBS class) | | 1305 | 1289 | | TTCGTGCTGTCCACTTT | 15.6 | 1.64 | | -3.25 | |  |
| AT2G04500.1 | | Cysteine/Histidine-rich C1 domain family protein | | 53 | 69 | | TCAATGGAAATCACGAT | 7.31 | 1.49 | | -2.29 | |  |
| AT2G19640.1 | | ASHR2, SDG39, ASH1-related protein 2 | | 108 | 92 | | TTCGTGCTGGACAAGTT | 2.89 | 8.06 | | 1.48 | |  |
| AT2G20950.1 | | Arabidopsis phospholipase-like protein (PEARLI 4) family | | 1238 | 1222 | | GTCGTGAAGGACATGGA | 6.01 | 1.06 | | -2.5 | |  |
| AT2G21590.1 | | APL4, Glucose-1-phosphate adenylyltransferase family protein | | 1463 | 1447 | | AACGTGATCATCATGAA | 0.34 | 2.2 | | 2.71 | |  |
| AT2G22170.1 | | Lipase/lipooxygenase, PLAT/LH2 family protein | | 361 | 377 | | TACGTGGAAGTCACGAC | 444.23 | 85.28 | | -2.38 | |  |
| AT2G23100.1 | | Cysteine/Histidine-rich C1 domain family protein | | 1328 | 1344 | | ACTTTGTTCTTCACGAA | 5.17 | 1.37 | | -1.92 | |  |
| AT2G27170.1 | | SMC3, TTN7, Structural maintenance of chromosomes (SMC) family protein | | 1155 | 1139 | | GACGTGCAACTCAATTT | 8.63 | 18.69 | | 1.12 | |  |
| AT2G33320.1 | | Calcium-dependent lipid-binding (CaLB domain) family protein | | 254 | 270 | | TCCGTGATGTTCACGTT | 9.96 | 3.39 | | -1.56 | |  |
| AT2G33680.1 | | Tetratricopeptide repeat (TPR)-like superfamily protein | | 114 | 98 | | GACGTGCAGTTCATGGA | 0.26 | 1.71 | | 2.72 | |  |
| AT2G34710.1 | | ATHB-14, ATHB14, PHB, PHB-1D, Homeobox-leucine zipper family protein / lipid-binding START domain-containing protein | | 2024 | 2008 | | TACGTGCGAAGCATAGT | 0.46 | 1.71 | | 1.9 | |  |
| AT2G38500.1 | | 2-oxoglutarate (2OG) and Fe(II)-dependent oxygenase superfamily protein | | 186 | 202 | | ATCTTGCGGCTCACGTC | 91.03 | 1.53 | | -5.9 | |  |
| AT2G42120.1 | | POLD2, DNA polymerase delta small subunit | | 826 | 842 | | ATCATGCCAGGCACGAA | 0.82 | 4.4 | | 2.42 | |  |
| AT2G42270.1 | | U5 small nuclear ribonucleoprotein helicase | | 117 | 101 | | GTCGTGATACGCATGAA | 72.84 | 25.74 | | -1.5 | |  |
| AT2G43160.1 | | ENTH/VHS family protein | | 327 | 311 | | AACGTGCATATCAAATT | 190.97 | 75.51 | | -1.34 | |  |
| AT2G44290.1 | | Bifunctional inhibitor/lipid-transfer protein/seed storage 2S albumin superfamily protein | | 514 | 530 | | TCTGTGCCAGGCACGAA | 20.59 | 2.29 | | -3.17 | |  |
| AT3G03720.1 | | CAT4, cationic amino acid transporter 4 | | 1099 | 1115 | | ATTATGATTGTCACGAT | 43.28 | 15.88 | | -1.45 | |  |
| AT3G06483.1 | | ATPDHK, PDK, pyruvate dehydrogenase kinase | | 491 | 507 | | TTATTGGGCAGCACGTT | 6.94 | 16.84 | | 1.28 | |  |
| AT3G06530.1 | | ARM repeat superfamily protein | | 567 | 551 | | TACGTGATAAACAAGTT | 1.97 | 10.78 | | 2.45 | |  |
| AT3G09240.1 | | Protein kinase protein with tetratricopeptide repeat domain | | 1022 | 1038 | | TAAGTGGTATGCACGAA | 11.54 | 0.52 | | -4.47 | |  |
| AT3G10010.1 | | DML2, demeter-like 2 | | 491 | 507 | | ATCATGAGCCTCACGAC | 1.23 | 0.33 | | -1.92 | |  |
| AT3G12670.1 | | emb2742, CTP synthase family protein | | 1232 | 1248 | | TTCTTGGTTTTCACGAT | 5.82 | 24.63 | | 2.08 | |  |
| AT3G18730.1 | | BRU1, MGO3, TSK, tetratricopeptide repeat (TPR)-containing protein | | 1950 | 1934 | | ATCGTGCCATCCAAGAT | 0.33 | 2.1 | | 2.68 | |  |
| AT3G19050.1 | | POK2, phragmoplast orienting kinesin 2 | | 2555 | 2571 | | ATTTTGAGCCGCACGAA | 0.47 | 4.6 | | 3.29 | |  |
| AT3G27510.1 | | Cysteine/Histidine-rich C1 domain family protein | | 710 | 726 | | TCCATGGAAGTCACGAT | 2.12 | 0.14 | | -3.94 | |  |
| AT3G45530.1 | | Cysteine/Histidine-rich C1 domain family protein | | 1442 | 1458 | | TCCATGGAAGTCACGAT | 3.22 | 0.09 | | -5.1 | |  |
| AT3G46970.1 | | ATPHS2, PHS2, alpha-glucan phosphorylase 2 | | 578 | 594 | | AAATTGTGAGGCACGAC | 3.6 | 9.95 | | 1.47 | |  |
| AT3G48730.1 | | GSA2, glutamate-1-semialdehyde 2,1-aminomutase 2 | | 58 | 42 | | TTCGTGCTCCGCAAAGT | 2.56 | 8.06 | | 1.66 | |  |
| AT3G49120.1 | | ATPCB, ATPERX34, PERX34, PRX34, PRXCB, peroxidase CB | | 806 | 790 | | TACGTGAATCTCAAAGA | 7.85 | 2.11 | | -1.9 | |  |
| AT3G54020.1 | | AtIPCS1, Arabidopsis Inositol phosphorylceramide synthase 1 | | 314 | 298 | | ATCGTGAAAAGCAAGAA | 105.7 | 29.16 | | -1.86 | |  |
| AT3G57410.1 | | ATVLN3, VLN3, villin 3 | | 312 | 296 | | ATCGTGAAATTCAAGGT | 124.34 | 36.51 | | -1.77 | |  |
| AT3G60770.1 | | Ribosomal protein S13/S15 | | 177 | 161 | | TTCGTGACTCTCACGGA | 64.41 | 250.47 | | 1.96 | |  |
| AT3G62990.1 | | unknown protein | | 233 | 249 | | AAAATGAAGGTCACGTT | 8.04 | 0.94 | | -3.1 | |  |
| AT3G63430.1 | | unknown protein | | 461 | 477 | | TATTTGACTTTCACGAT | 0.53 | 3.37 | | 2.68 | |  |
| AT3G63450.1 | | RNA-binding (RRM/RBD/RNP motifs) family protein | | 605 | 589 | | TTCGTGACCTTCATGTA | 0.37 | 3.38 | | 3.2 | |  |
| AT4G03460.1 | | Ankyrin repeat family protein | | 1828 | 1844 | | TTTATGGCTGGCACGTA | 6.94 | 2.44 | | -1.51 | |  |
| AT4G03500.1 | | Ankyrin repeat family protein | | 1723 | 1739 | | TTTATGGCTGGCACGTA | 16.3 | 2.45 | | -2.73 | |  |
| AT4G12120.1 | | ATSEC1B, SEC1B, Sec1/munc18-like (SM) proteins superfamily | | 969 | 953 | | TTCGTGATGCTCATATT | 61.34 | 25.55 | | -1.26 | |  |
| AT4G14390.1 | | Ankyrin repeat family protein | | 1943 | 1959 | | TCCTTGGCCCTCACGTC | 10.48 | 0.48 | | -4.45 | |  |
| AT4G19530.1 | | disease resistance protein (TIR-NBS-LRR class) family | | 3007 | 3023 | | TCAGTGACATGCACGTT | 2.24 | 0.17 | | -3.71 | |  |
| AT4G20210.1 | | Terpenoid cyclases/Protein prenyltransferases superfamily protein | | 165 | 149 | | TTCGTGATCCTCAAGAA | 0.88 | 3.75 | | 2.1 | |  |
| AT4G24780.1 | | Pectin lyase-like superfamily protein | | 524 | 508 | | TACGTGACCAACATTAT | 2.73 | 15.51 | | 2.5 | |  |
| AT4G25930.1 | | Protein of unknown function (DUF295) | | 995 | 1011 | | ACTTTGGTGTTCACGAT | 1.3 | 0 | | #NAME? | |  |
| AT4G26690.1 | | GPDL2, MRH5, SHV3, PLC-like phosphodiesterase family protein | | 521 | 537 | | TAAATGTTCAGCACGAT | 215.12 | 40.48 | | -2.41 | |  |
| AT4G27400.1 | | Late embryogenesis abundant (LEA) protein-related | | 641 | 625 | | ATCGTGACTATCAAAGA | 17.2 | 117.74 | | 2.77 | |  |
| AT4G29750.1 | | CRS1 / YhbY (CRM) domain-containing protein | | 267 | 283 | | TAAATGGTCTTCACGTA | 1.04 | 6.03 | | 2.53 | |  |
| AT4G30990.1 | | ARM repeat superfamily protein | | 1073 | 1057 | | ATCGTGAGCCGCATTGT | 1.47 | 5.2 | | 1.82 | |  |
| AT4G36250.1 | | ALDH3F1, aldehyde dehydrogenase 3F1 | | 1086 | 1070 | | TACGTGACATCCAAGAA | 0.78 | 16.19 | | 4.38 | |  |
| AT5G01490.1 | | ATCAX4, CAX4, cation exchanger 4 | | 842 | 826 | | GACGTGAGTGACAAGGA | 63.45 | 24.19 | | -1.39 | |  |
| AT5G01550.1 | | LECRKA4.2, lectin receptor kinase a4.1 | | 989 | 973 | | TTCGTGATGTACAAGAA | 1.4 | 0.17 | | -3.06 | |  |
| AT5G06670.1 | | P-loop containing nucleoside triphosphate hydrolases superfamily protein | | 1182 | 1166 | | ATCGTGCAAAGCATATT | 0.25 | 1.08 | | 2.14 | |  |
| AT5G18700.1 | | EMB3013, RUK, Protein kinase family protein with ARM repeat domain | | 3524 | 3508 | | TTCGTGAATGCCAAAGA | 1.18 | 4.73 | | 2 | |  |
| AT5G19540.1 | | unknown protein | | 311 | 295 | | ATCGTGAGTTACAAGAA | 1.22 | 4.32 | | 1.83 | |  |
| AT5G22560.1 | | Plant protein of unknown function (DUF247) | | 1174 | 1190 | | AACGTGCAAGGCACGAC | 3.89 | 0 | | #NAME? | |  |
| AT5G27000.1 | | ATK4, KATD, kinesin 4 | | 944 | 960 | | ATTTTGAACATCACGAA | 0.86 | 3.19 | | 1.89 | |  |
| AT5G27680.1 | | RECQSIM, RECQ helicase SIM | | 1241 | 1257 | | ATTTTGGTTCTCACGAT | 0.18 | 2.15 | | 3.59 | |  |
| AT5G35450.1 | | Disease resistance protein (CC-NBS-LRR class) family | | 2451 | 2435 | | TACGTGCTCTACAAATA | 0.84 | 3.04 | | 1.85 | |  |
| AT5G43520.1 | | Cysteine/Histidine-rich C1 domain family protein | | 405 | 389 | | ATCGTGAAGATCATGAA | 125.82 | 38.66 | | -1.7 | |  |
| AT5G45700.1 | | Haloacid dehalogenase-like hydrolase (HAD) superfamily protein | | 383 | 367 | | GTCGTGAATCCCAAAAT | 0.35 | 2.31 | | 2.73 | |  |
| AT5G48900.1 | | Pectin lyase-like superfamily protein | | 551 | 535 | | TTCGTGACTAACATTAT | 8.84 | 68.25 | | 2.95 | |  |
| AT5G49270.1 | | COBL9, DER9, MRH4, SHV2, COBRA-like extracellular glycosyl-phosphatidyl inositol-anchored protein family | | 1537 | 1553 | | ACAGTGAGGATCACGAT | 80.48 | 0.31 | | -8 | |  |
| AT5G49870.1 | | Mannose-binding lectin superfamily protein | | 1718 | 1702 | | TTCGTGCTCCACAAGGA | 31.88 | 0.31 | | -6.69 | |  |
| AT5G54370.1 | | Late embryogenesis abundant (LEA) protein-related | | 632 | 616 | | ATCGTGACAATCAAAGA | 275.39 | 926.7 | | 1.75 | |  |
| AT5G59570.1 | | Homeodomain-like superfamily protein | | 896 | 880 | | GACGTGAATGACAAGTA | 61.33 | 28.5 | | -1.11 | |  |
| AT5G60520.1 | | Late embryogenesis abundant (LEA) protein-related | | 701 | 685 | | AACGTGAGAGTCACGGT | 1.56 | 20.95 | | 3.75 | |  |
| AT5G60930.1 | | P-loop containing nucleoside triphosphate hydrolases superfamily protein | | 1263 | 1247 | | ATCGTGAGCTACACAAT | 3.99 | 9.06 | | 1.18 | |  |
| AT5G61460.1 | | ATRAD18, MIM, SMC6B, P-loop containing nucleoside triphosphate hydrolases superfamily protein | | 2078 | 2094 | | AACTTGAGATGCACGAT | 0.56 | 3.31 | | 2.56 | |  |
| AT5G66040.1 | | STR16, sulfurtransferase protein 16 | | 149 | 133 | | AACGTGCCTTACATGAA | 39.95 | 5.93 | | -2.75 | |  |
