# Supplementary material for: Re-analysis of RNA-seq transcriptome data reveals new aspects of gene activity in Arabidopsis root hairs
Source: Front Plant Sci. 2015 Jun 8;6:421. doi: 10.3389/fpls.2015.00421 (PMC4458573; doi:10.3389/fpls.2015.00421)
Supplement: Supplementary file 20 [file Table15.DOC]

**Table S15** List of 136 conserved root epidermal genes. Genes in bold letters are non-root hair genes and the rest are root hair genes. Genes underlined carry RHE motif.

| AGI | Annotation | | |  | | | | RH(RPKM) | NRH(RPKM) | | | Fold_change(log2) |
| --- | --- | --- | --- | --- | --- | --- | --- | --- | --- | --- | --- | --- |
| AT1G01750 | ADF11, actin depolymerizing factor 11 | | | | | | 723.49 | | | 10.76 | | -6.07 |
| AT1G03550 | Secretory carrier membrane protein (SCAMP) family protein | | | | | | 69.35 | | | 6.09 | | -3.51 |
| AT1G04700 | PB1 domain-containing protein tyrosine kinase | | | | | | 49.94 | | | 1.67 | | -4.90 |
| AT1G07795 | unknown protein | |  | | 47.42 | | | | | 0.55 | | -6.42 |
| AT1G09170 | P-loop nucleoside triphosphate hydrolases superfamily protein with CH (Calponin Homology) domain | | | | | | 24.03 | | | 0.21 | | -6.87 |
| AT1G12040 | LRX1, leucine-rich repeat/extensin 1 | | | | | | 184.77 | | | 0.37 | | -8.97 |
| AT1G12560 | ATEXP7, ATEXPA7, ATHEXP ALPHA 1.26, EXP7, EXPA7, expansin A7 | | | | | | 390.92 | | | 0.37 | | -10.06 |
| AT1G12950 | RSH2, root hair specific 2 | | | | | | 383.27 | | | 47.37 | | -3.02 |
| AT1G15040 | Class I glutamine amidotransferase-like superfamily protein | | | | | | 340.56 | | | 27.52 | | -3.63 |
| AT1G16440 | RSH3, root hair specific 3 | | | | | | 22.04 | | | 0.52 | | -5.39 |
| AT1G18940 | Nodulin-like / Major Facilitator Superfamily protein | | | | | | 15.96 | | | 0.90 | | -4.14 |
| AT1G22500 | RING/U-box superfamily protein | | | | | | 44.10 | | | 5.34 | | -3.05 |
| **AT1G25450** | CER60, KCS5, 3-ketoacyl-CoA synthase 5 | | | | | | 1.12 | | | 5.20 | | 2.21 |
| **AT1G25460** | NAD(P)-binding Rossmann-fold superfamily protein | | | | | | 3.83 | | | 0.73 | | -2.39 |
| AT1G27740 | RSL4, root hair defective 6-like 4 | | | | | | 254.49 | | | 2.16 | | -6.88 |
| AT1G30870 | Peroxidase superfamily protein | | | | | | 413.75 | | | 0.81 | | -8.99 |
| AT1G34330 | pseudogene, putative peroxidase | | | | | | 2.81 | | | 0.00 | | #NAME? |
| AT1G35330 | RING/U-box superfamily protein | | | | | | 9.23 | | | 0.00 | | #NAME? |
| AT1G35670 | ATCDPK2, ATCPK11, CDPK2, CPK11, calcium-dependent protein kinase 2 | | | | | | 322.30 | | | 51.03 | | -2.66 |
| AT1G48640 | Transmembrane amino acid transporter family protein | | | | | | 42.90 | | | 1.15 | | -5.22 |
| AT1G48930 | AtGH9C1, GH9C1, glycosyl hydrolase 9C1 | | | | | | 212.10 | | | 0.45 | | -8.88 |
| AT1G50890 | ARM repeat superfamily protein | | | | | | 47.50 | | | 4.22 | | -3.49 |
| AT1G53680 | ATGSTU28, GSTU28, glutathione S-transferase TAU 28 | | | | | | 304.14 | | | 5.35 | | -5.83 |
| AT1G54970 | ATPRP1, PRP1, RHS7, proline-rich protein 1 | | | | | | 197.92 | | | 0.42 | | -8.88 |
| AT1G62980 | ATEXP18, ATEXPA18, ATHEXP ALPHA 1.25, EXP18, EXPA18, expansin A18 | | | | | | 208.44 | | | 0.35 | | -9.24 |
| AT1G63450 | RHS8, root hair specific 8 | | | | | | 20.49 | | | 1.38 | | -3.89 |
| AT1G63930 | ROH1, from the Czech 'roh' meaning 'corner' | | | | | | 43.37 | | | 1.36 | | -4.99 |
| AT1G70460 | RHS10, root hair specific 10 | | | | | | 88.10 | | | 0.64 | | -7.10 |
| AT1G73860 | P-loop containing nucleoside triphosphate hydrolases superfamily protein | | | | | | 70.08 | | | 1.41 | | -5.64 |
| AT1G79320 | AtMC6, MC6, metacaspase 6 | | | | | | 29.44 | | | 5.23 | | -2.49 |
| AT2G01540 | Calcium-dependent lipid-binding (CaLB domain) family protein | | | | | | 474.40 | | | 22.54 | | -4.40 |
| AT2G02630 | Cysteine/Histidine-rich C1 domain family protein | | | | | | 1.85 | | | 0.28 | | -2.74 |
| AT2G04680 | Cysteine/Histidine-rich C1 domain family protein | | | | | | 8.96 | | | 0.55 | | -4.02 |
| AT2G05160 | CCCH-type zinc fingerfamily protein with RNA-binding domain | | | | | | 29.13 | | | 0.61 | | -5.57 |
| AT2G17590 | Cysteine/Histidine-rich C1 domain family protein | | | | | | 4.31 | | | 0.08 | | -5.67 |
| AT2G18450 | SDH1-2, succinate dehydrogenase 1-2 | | | | | | 110.51 | | | 9.11 | | -3.60 |
| AT2G18690 | unknown protein | | | | |  | | 158.69 | | | 36.03 | -2.14 |
| AT2G20520 | FLA6, FASCICLIN-like arabinogalactan 6 | | | | | | 49.45 | | | 1.88 | | -4.72 |
| AT2G21850 | Cysteine/Histidine-rich C1 domain family protein | | | | | | 19.14 | | | 6.79 | | -1.50 |
| AT2G24260 | LRL1, LJRHL1-like 1 | | | | | | 64.17 | | | 8.81 | | -2.86 |
| AT2G28440 | proline-rich family protein | | | | | | 28.09 | | | 1.40 | | -4.33 |
| AT2G29740 | UGT71C2, UDP-glucosyl transferase 71C2 | | | | | | 23.24 | | | 1.13 | | -4.36 |
| AT2G34910 | BEST Arabidopsis thaliana protein match is: root hair specific 4 (TAIR:AT1G30850.1) | | | | | | 587.71 | | | 14.98 | | -5.29 |
| AT2G37440 | DNAse I-like superfamily protein | | | | | | 49.50 | | | 5.07 | | -3.29 |
| AT2G37670 | Transducin/WD40 repeat-like superfamily protein | | | | | | 40.67 | | | 0.99 | | -5.35 |
| AT2G41970 | Protein kinase superfamily protein | | | | | | 232.38 | | | 0.55 | | -8.72 |
| **AT2G42060** | Cysteine/Histidine-rich C1 domain family protein | | | | | | 5.73 | | | 1.23 | | -2.22 |
| **AT2G45220** | Plant invertase/pectin methylesterase inhibitor superfamily | | | | | | 184.45 | | | 8.61 | | -4.42 |
| AT2G45750 | S-adenosyl-L-methionine-dependent methyltransferases superfamily protein | | | | | | 142.03 | | | 1.62 | | -6.46 |
| AT2G45890 | ATROPGEF4, RHS11, ROPGEF4, RHO guanyl-nucleotide exchange factor 4 | | | | | | 101.07 | | | 0.37 | | -8.10 |
| AT2G46860 | AtPPa3, PPa3, pyrophosphorylase 3 | | | | | | 111.43 | | | 0.59 | | -7.55 |
| AT2G47540 | Pollen Ole e 1 allergen and extensin family protein | | | | | | 213.42 | | | 0.51 | | -8.70 |
| AT3G03520 | NPC3, non-specific phospholipase C3 | | | | | | 109.39 | | | 16.66 | | -2.72 |
| **AT3G04940** | ATCYSD1, CYSD1, cysteine synthase D1 | | | | | | 5.11 | | | 15.52 | | 1.60 |
| AT3G07070 | Protein kinase superfamily protein | | | | | | 39.47 | | | 0.21 | | -7.57 |
| AT3G07880 | SCN1, Immunoglobulin E-set superfamily protein | | | | | | 469.67 | | | 58.95 | | -2.99 |
| AT3G10710 | RHS12, root hair specific 12 | | | | | | 57.54 | | | 0.19 | | -8.25 |
| AT3G13782 | NAP1;4, NFA04, NFA4, nucleosome assembly protein1;4 | | | | | | 209.37 | | | 6.59 | | -4.99 |
| AT3G23190 | HR-like lesion-inducing protein-related | | | | | | 366.31 | | | 10.91 | | -5.07 |
| **AT3G26744** | ATICE1, ICE1, SCRM, basic helix-loop-helix (bHLH) DNA-binding superfamily protein | | | | | | 2.62 | | | 10.28 | | 1.97 |
| **AT3G29410** | Terpenoid cyclases/Protein prenyltransferases superfamily protein | | | | | | 86.97 | | | 23.70 | | -1.88 |
| AT3G49960 | Peroxidase superfamily protein | | | | | | 159.37 | | | 1.06 | | -7.23 |
| AT3G54040 | PAR1 protein | | | | | | 259.24 | | | 20.62 | | -3.65 |
| AT3G54580 | Proline-rich extensin-like family protein | | | | | | 1903.30 | | | 8.86 | | -7.75 |
| AT3G54870 | ARK1, CAE1, MRH2, Armadillo/beta-catenin repeat family protein / kinesin motor family protein | | | | | | 48.08 | | | 0.75 | | -6.01 |
| AT3G56000 | ATCSLA14, CSLA14, cellulose synthase like A14 | | | | | | 57.07 | | | 5.07 | | -3.49 |
| AT3G60330 | AHA7, HA7, H(+)-ATPase 7 | | | | | | 310.86 | | | 1.57 | | -7.63 |
| AT3G62680 | ATPRP3, PRP3, proline-rich protein 3 | | | | | | 419.94 | | | 0.45 | | -9.86 |
| AT4G00680 | ADF8, actin depolymerizing factor 8 | | | | | | 479.40 | | | 0.68 | | -9.47 |
| AT4G02270 | RHS13, root hair specific 13 | | | | | | 828.72 | | | 1.13 | | -9.52 |
| AT4G03330 | ATSYP123, SYP123, syntaxin of plants 123 | | | | | | 32.72 | | | 1.52 | | -4.43 |
| AT4G07960 | ATCSLC12, CSLC12, CSLC12, Cellulose-synthase-like C12 | | | | | | 87.03 | | | 3.42 | | -4.67 |
| AT4G09990 | Protein of unknown function (DUF579) | | | | | | 189.19 | | | 0.90 | | -7.71 |
| AT4G13390 | Proline-rich extensin-like family protein | | | | | | 346.90 | | | 0.44 | | -9.63 |
| AT4G14610 | pseudogene, disease resistance protein (CC-NBS-LRR class) | | | | | | 11.79 | | | 1.75 | | -2.75 |
| AT4G14980 | Cysteine/Histidine-rich C1 domain family protein | | | | | | 12.01 | | | 4.01 | | -1.58 |
| AT4G16350 | CBL6, SCABP2, calcineurin B-like protein 6 | | | | | | 34.90 | | | 2.30 | | -3.93 |
| **AT4G17215** | Pollen Ole e 1 allergen and extensin family protein | | | | | | 22.35 | | | 4.08 | | -2.46 |
| AT4G18640 | MRH1, Leucine-rich repeat protein kinase family protein | | | | | | 85.97 | | | 1.95 | | -5.46 |
| AT4G19680 | ATIRT2, IRT2, iron regulated transporter 2 | | | | | | 109.85 | | | 3.85 | | -4.84 |
| AT4G24580 | REN1, Rho GTPase activation protein (RhoGAP) with PH domain | | | | | | 70.97 | | | 2.64 | | -4.75 |
| AT4G25160 | U-box domain-containing protein kinase family protein | | | | | | 19.67 | | | 0.42 | | -5.56 |
| AT4G25220 | RHS15, root hair specific 15 | | | | | | 24.17 | | | 0.25 | | -6.57 |
| AT4G25820 | ATXTH14, XTH14, XTR9, xyloglucan endotransglucosylase/hydrolase 14 | | | | | | 758.46 | | | 0.93 | | -9.67 |
| AT4G29180 | RHS16, root hair specific 16 | | | | | | 55.35 | | | 0.45 | | -6.95 |
| AT4G29800 | PLA IVD, PLP8, PATATIN-like protein 8 | | | | | | 28.79 | | | 0.64 | | -5.49 |
| AT4G31250 | Leucine-rich repeat protein kinase family protein | | | | | | 39.68 | | | 2.62 | | -3.92 |
| AT4G33730 | CAP (Cysteine-rich secretory proteins, Antigen 5, and Pathogenesis-related 1 protein) superfamily protein | | | | | | 29.24 | | | 0.00 | | #NAME? |
| AT4G34580 | COW1, SRH1, Sec14p-like phosphatidylinositol transfer family protein | | | | | | 218.10 | | | 1.48 | | -7.21 |
| AT4G40090 | AGP3, arabinogalactan protein 3 | | | | | | 1002.28 | | | 1.07 | | -9.87 |
| AT5G01610 | Protein of unknown function, DUF538 | | | | | | 180.27 | | | 12.22 | | -3.88 |
| AT5G04960 | Plant invertase/pectin methylesterase inhibitor superfamily | | | | | | 179.83 | | | 0.20 | | -9.80 |
| AT5G05400 | LRR and NB-ARC domains-containing disease resistance protein | | | | | | 6.74 | | | 1.23 | | -2.46 |
| AT5G05500 | Pollen Ole e 1 allergen and extensin family protein | | | | | | 467.41 | | | 0.95 | | -8.94 |
| AT5G06640 | Proline-rich extensin-like family protein | | | | | | 300.57 | | | 0.38 | | -9.64 |
| AT5G07080 | HXXXD-type acyl-transferase family protein | | | | | | 106.99 | | | 2.57 | | -5.38 |
| AT5G13150 | ATEXO70C1, EXO70C1, exocyst subunit exo70 family protein C1 | | | | | | 52.70 | | | 2.76 | | -4.26 |
| AT5G13990 | ATEXO70C2, EXO70C2, exocyst subunit exo70 family protein C2 | | | | | | 114.10 | | | 7.30 | | -3.97 |
| AT5G16900 | Leucine-rich repeat protein kinase family protein | | | | | | 24.42 | | | 3.05 | | -3.00 |
| AT5G22410 | RHS18, root hair specific 18 | | | | | | 106.32 | | | 0.29 | | -8.51 |
| AT5G24310 | ABIL3, ABL interactor-like protein 3 | | | | | | 101.96 | | | 3.18 | | -5.00 |
| AT5G35190 | proline-rich extensin-like family protein | | | | | | 467.48 | | | 0.81 | | -9.17 |
| AT5G40510 | Sucrase/ferredoxin-like family protein | | | | | | 242.64 | | | 25.69 | | -3.24 |
| AT5G46250 | RNA-binding protein | | | | | | 148.52 | | | 61.05 | | -1.28 |
| AT5G49270 | COBL9, DER9, MRH4, SHV2, COBRA-like extracellular glycosyl-phosphatidyl inositol-anchored protein family | | | | | | 80.48 | | | 0.31 | | -8.00 |
| AT5G57530 | AtXTH12, XTH12, xyloglucan endotransglucosylase/hydrolase 12 | | | | | | 125.27 | | | 0.21 | | -9.22 |
| AT5G58010 | LRL3, LJRHL1-like 3 | | | | | | 273.51 | | | 1.80 | | -7.24 |
| AT5G61350 | Protein kinase superfamily protein | | | | | | 30.43 | | | 0.83 | | -5.19 |
| AT5G61550 | U-box domain-containing protein kinase family protein | | | | | | 35.98 | | | 0.37 | | -6.59 |
| AT5G61650 | CYCP4, CYCP4;2, CYCLIN P4;2 | | | | | | 6.12 | | | 0.00 | | #NAME? |
| AT5G62310 | IRE, AGC (cAMP-dependent, cGMP-dependent and protein kinase C) kinase family protein | | | | | | 64.01 | | | 0.52 | | -6.95 |
| AT5G65090 | BST1, DER4, MRH3, DNAse I-like superfamily protein | | | | | | 41.27 | | | 0.59 | | -6.12 |
| AT5G67400 | RHS19, root hair specific 19 | | | | | | 456.33 | | | 0.57 | | -9.64 |
| AT1G05630 | 5PTASE13, AT5PTASE13, Endonuclease/exonuclease/phosphatase family protein | | | | | | 24.51 | | | 2.83 | | -3.12 |
| **AT1G10550** | XET, XTH33, xyloglucan:xyloglucosyl transferase 33 | | | | | | 2.14 | | | 0.29 | | -2.87 |
| AT1G22570 | Major facilitator superfamily protein | | | | | | 33.35 | | | 4.38 | | -2.93 |
| AT1G34760 | GF14 OMICRON, GRF11, RHS5, general regulatory factor 11 | | | | | | 50.72 | | | 0.64 | | -6.32 |
| AT1G65180 | Cysteine/Histidine-rich C1 domain family protein | | | | | | 9.01 | | | 2.30 | | -1.97 |
| AT1G66470 | RHD6, ROOT HAIR DEFECTIVE6 | | | | | | 161.46 | | | 6.58 | | -4.62 |
| AT1G71530 | Protein kinase superfamily protein | | | | | | 83.88 | | | 9.48 | | -3.15 |
| AT2G22560 | Kinase interacting (KIP1-like) family protein | | | | | | 57.65 | | | 17.59 | | -1.71 |
| **AT2G37260** | ATWRKY44, DSL1, TTG2, WRKY44, WRKY family transcription factor family protein | | | | | | 1.74 | | | 7.81 | | 2.16 |
| AT2G40010 | Ribosomal protein L10 family protein | | | | | | 9.41 | | | 24.70 | | 1.39 |
| AT2G48080 | oxidoreductase, 2OG-Fe(II) oxygenase family protein | | | | | | 32.82 | | | 4.21 | | -2.96 |
| AT3G05170 | Phosphoglycerate mutase family protein | | | | | | 90.12 | | | 9.58 | | -3.23 |
| **AT3G16800** | Protein phosphatase 2C family protein | | | | | | 119.30 | | | 44.02 | | -1.44 |
| **AT3G28910** | ATMYB30, MYB30, myb domain protein 30 | | | | | | 1.69 | | | 7.82 | | 2.21 |
| AT3G47340 | ASN1, AT-ASN1, DIN6, glutamine-dependent asparagine synthase 1 | | | | | | 622.36 | | | 182.49 | | -1.77 |
| **AT3G54400** | Eukaryotic aspartyl protease family protein | | | | | | 2.98 | | | 18.36 | | 2.62 |
| AT4G02390 | APP, ATPARP1, PARP1, PP, poly(ADP-ribose) polymerase | | | | | | 37.88 | | | 3.79 | | -3.32 |
| AT4G12330 | CYP706A7, cytochrome P450, family 706, subfamily A, polypeptide 7 | | | | | | 23.39 | | | 6.07 | | -1.95 |
| AT4G27290 | S-locus lectin protein kinase family protein | | | | | | 39.10 | | | 1.50 | | -4.70 |
| AT5G15950 | Adenosylmethionine decarboxylase family protein | | | | | | 195.24 | | | 34.03 | | -2.52 |
| AT5G24140 | SQP2, squalene monooxygenase 2 | | | | | | 108.44 | | | 9.65 | | -3.49 |
| AT5G40860 | unknown protein |  | | | | | 136.68 | | | 0.63 | | -7.77 |
| AT5G65160 | tetratricopeptide repeat (TPR)-containing protein | | | | | | 74.26 | | | 0.74 | | -6.64 |
